# Umbilical cord artery-derived perivascular stem cells for treatment of ovarian failure through CD146 signaling

**DOI:** 10.1038/s41392-022-01029-4

**Published:** 2022-07-13

**Authors:** Lu Xu, Yanjun Yang, Lingling Zhang, Guijun Yan, Shiyuan Li, Yifan Li, Yali Hu, Lijun Ding, Bruno Péault, Haixiang Sun

**Affiliations:** 1 Center for Reproductive Medicine and Obstetrics and Gynecology, the Affiliated Drum Tower Hospital of Nanjing University Medical School, Nanjing, 210008 China; 2 Center for Molecular Reproductive Medicine, Nanjing University, Nanjing, 210093 China; 3 Department of Obstetrics and Gynecology, the Affiliated Drum Tower Hospital of Nanjing University Medical School, Nanjing, 210008 China; 4 NHC Key Lab of Reproduction Regulation, Shanghai Institute of Planned Parenthood Research, Pharmacy School, Fudan University, Shanghai, 200032 China; 5 Clinical Center for Stem Cell Research, the Affiliated Drum Tower Hospital of Nanjing University Medical School, Nanjing, 210008 China; 6 Orthopedic Hospital Research Center and Broad Stem Cell Center, David Geffen School of Medicine, University of California, Los Angeles, CA USA; 7 State Key Laboratory of Pharmaceutical Biotechnology, Nanjing University, Nanjing, 210008 China

**Keywords:** Mesenchymal stem cells

**Dear Editor**,

Chemotherapy-induced ovarian failure significantly diminishes ovarian blood flow, ovarian size, and follicular development. Angiogenesis plays a vital role in repairing ovarian damage.^1^ Perivascular stem cells (PSCs), known as mural cells covering the vasculature, are essential for blood vessel formation and postulated as progenitors of mesenchymal stem cells (MSCs).^2,3^ We previously established umbilical cord artery-derived PSCs (UCA-PSCs) and Wharton’s jelly-derived MSCs (WJ-MSCs) and UCA-PSCs display optimal angiogenic capacity in vitro.^4^ Therefore, we explored the angiogenesis and pro-angiogenesis mechanisms of UCA-PSCs and provided them as an efficient treatment strategy for ovarian failure.

In this study, we isolated and cultured UCA-PSCs and WJ-MSCs, which morphologically resembled fibroblasts, and differentiated into adipocytes, osteoblasts, or neural-like cells after induction in vitro (Supplementary Fig. 1a, b). These cells presented a typical MSC phenotype (Supplementary Fig. 1c). In particular, more UCA-PSCs than WJ-MSCs expressed CD146 (Fig. 1a) and stably expressed CD146, Jagged1, and other perivascular cell markers, PDGF-Rβ, NG2, and α-SMA after long-term in vitro culture (Supplementary Fig. 1d), suggesting their perivascular origin and association with angiogenesis.^5^

**Fig. 1:**
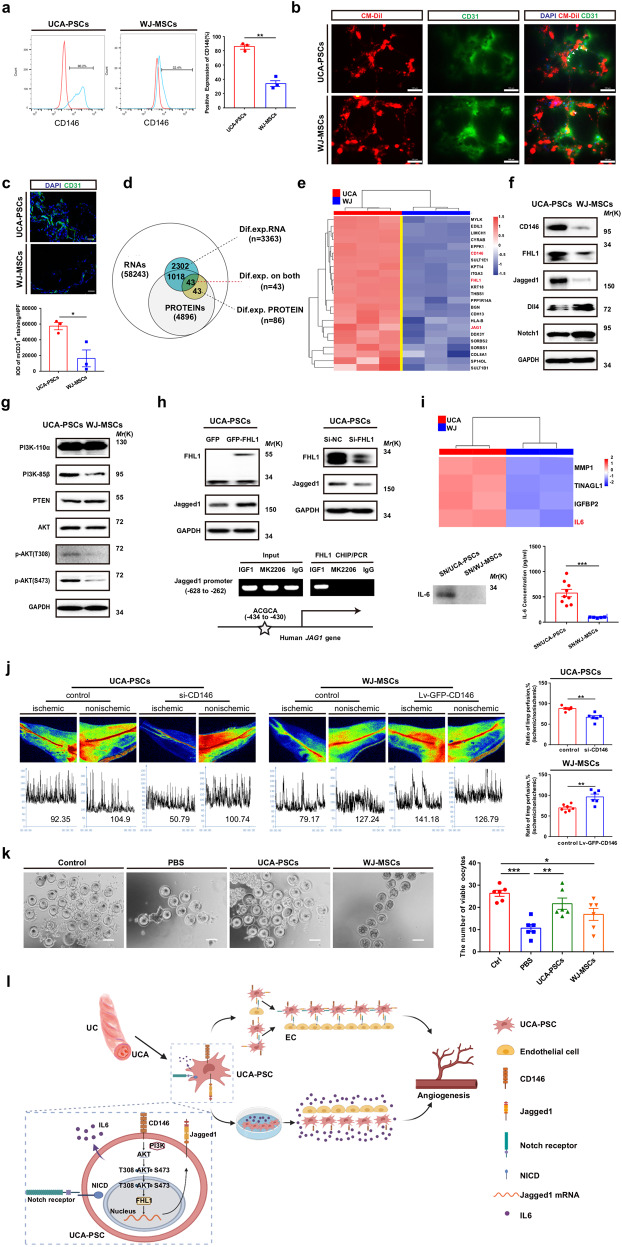
UCA-PSCs efficiently promote angiogenesis through the CD146/FHL1/Jagged1 signaling and IL6 secretion. **a** Flow cytometry analysis of UCA-PSCs and WJ-MSCs for CD146. UCA-PSCs had more CD146^+^ cells than WJ-MSCs. The data are shown as the mean ± SEM. *n* = 3 per group, **P < 0.01 (*t*-test). **b** Representative images of HUVECs (5000 cells/well) tube formation assay with the presence of CM-Dil-labeled UCA-PSCs (5000 cells/well) or WJ-MSCs (5000 cells/well) and successively stained with endothelial cell marker, CD31 (green) (*n* = 3). Scale bars, 100 μm. White arrowheads indicate UCA-PSCs or WJ- MSCs directly contacting with HUVECs. **c** Sections of cryopreserved Matrigel plugs were stained for mouse CD31 (green), and cell nuclei were visualized with DAPI (blue). Scale bars, 100 μm. The integrated optical density (IOD) values of positive staining in 3–5 randomly selected high-power fields of view were counted. The data were shown as the mean ± SEM. *n* = 3 per group. *P < 0.05 (*t*-test). **d** Venn analysis was used to assess the 43 common differentially expressed genes at the mRNA and protein levels in UCA-PSCs and WJ-MSCs. **e** Heatmaps showed that 23 angiogenesis-related genes were upregulated in UCA-PSCs. **f** Western blot analysis of the expression of CD146, FHL1, Jagged1, Dll4, and Notch1 in UCA-PSCs and WJ-MSCs. GAPDH was used as an internal loading control (*n* = 3). **g** Western blot analysis of the expression of the indicated proteins involved in the PI3K/AKT pathway in UCA-PSCs and WJ-MSCs, including PI3K-110α, PI3K-85β, PTEN, AKT, p- Akt (T308), and p-Akt (S473). GAPDH was used as an internal loading control (*n* = 3). **h** UCA-PSCs were transfected with GFP or GFP-FHL1 plasmids, Si-NC, or Si-FHL1 siRNAs at the indicated concentrations for 48 h. Protein levels of FHL1 and Jagged1 were measured by Western blot. GAPDH was used as an internal loading control (*n* = 3). ChIP-PCR amplification using primers against the human Jagged1 promoter region. Input (non-precipitated) chromatin was utilized as a positive control (*n* = 3). **i** Four angiogenetic factors differentially expressed with fold change >2.0 between the supernatant in UCA-PSCs and WJ-MSCs. IL6 released from UCA-PSCs and WJ-MSCs into the medium was measured by Western blot and ELISA. ***P < 0.001 (*t*-test). **j** The blood perfusion ratio of ischemic limbs was measured by LDPI at 4 weeks after transplantation. The color scale for perfusion range is shown, with blue indicating impeded perfusion. The blood perfusion in si-CD146 UCA-PSC-transplanted mice showed a pronounced decrease compared with that in si-NC UCA-PSC-transplanted mice 4 weeks after transplantation. Additionally, the ratio of ischemic to normal limb blood perfusion was significantly increased in Lv-GFP-CD146 WJ-MSC-transplanted mice compared with the WJ-MSC-transplanted mice. LDPI: laser doppler perfusion imaging. Quantitative analysis of hindlimb blood perfusion with the LDPI index, the ratio of ischemic to non-ischemic hindlimb blood perfusion. The data were shown as the mean ± SEM. *n* = 6 per group. **P < 0.01 (*t*-test). **k** Representative images and quantitative analysis of the number of viable oocytes in POF mice ovaries after superovulation. The data were shown as the mean ± SEM. *n* = 6 per group. Scale bars,100 μm. *P < 0.05, **P < 0.01, ***P < 0.001 (one-way ANOVA). **l** Schematic diagram showing that UCA-PSCs augment angiogenesis through cell-to-cell communication via CD146/AKT/FHL1/Jagged1 signaling as well as the IL6 paracrine activity.

We then assessed the in vitro angiogenic capacity of UCA-PSCs and WJ-MSCs by tube formation assay. After incubation for 3 h, the tubule number, tube branching point number, and tube length formed by UCA-PSCs were apparently greater than those formed by WJ-MSCs (Supplementary Fig. 1e). In addition, UCA-PSCs significantly promoted interconnected tubule formation when cocultured with HUVECs (Supplementary Fig. 1e), and more UCA-PSCs than WJ-MSCs interacted with HUVECs, as indicated by CM-Dil labeling, and formed tubular structures (Fig. 1b). In vivo Matrigel plugs formed by UCA-PSCs showed a ruddier appearance than those formed by WJ-MSCs 2 weeks after transplantation (Supplementary Fig. 1f). UCA-PSC-treated Matrigel plugs showed higher expression of mouse CD31 and vWF, and more capillaries per high-power field (HPF) than the WJ-MSC counterpart (Fig. 1c and Supplementary Fig. 1g, h).

Next, we investigated the angiogenesis-related mechanisms for UCA-PSCs. cDNA libraries based on the UCA-PSC and WJ-MSC primary single-cell colonies (~10^2^ cells) were used for sequencing (Supplementary Fig. 2a). A total of 3363 differentially expressed genes (DEGs) were identified between the UCA-PSCs and WJ-MSCs (Supplementary Fig. 2b and Supplementary Table 1). A Gene Ontology (GO) analysis showed several DEGs belonging to “cell adhesion” and “angiogenesis” (Supplementary Fig. 2c). Among the ten most-enriched molecular pathways, “PI3K-AKT signaling” and “cytokine and cytokine receptor interaction” were identified (Supplementary Fig. 2d). As detected by liquid chromatography with tandem mass spectrometry (LC-MS/MS), 86 proteins were differentially expressed between cultured UCA-PSCs and WJ-MSCs (Supplementary Fig. 2e and Supplementary Table 2), 54 of which were upregulated in UCA-PSCs (Supplementary Fig. 2f). Among the 15 most-enriched molecular pathways, “ECM-receptor interaction” and “Notch signaling” were identified (Supplementary Fig. 2g). Then, 43 DEGs were identified by Venn analysis at both the mRNA and protein levels (Fig. 1d and Supplementary Table 3). Twenty-three genes were upregulated in UCA-PSCs compared with WJ-MSCs, including melanoma cell adhesion molecule (MCAM or CD146), four-and-a-half LIM domain 1 (FHL1), and Notch ligand Jagged1 (JAG1) (Fig. 1e), which was confirmed by qRT-PCR and western blot analysis (Supplementary Fig. 2h and Fig. 1f). Additionally, UCA-PSCs expressed less PTEN, and more p-Akt (T308) and p-Akt (S473) proteins than WJ-MSCs (Fig. 1g). To further characterize UCA-PSCs in situ, gene expression was analyzed in human UCs (Supplementary Fig. 3a–e). CD146 was highly expressed in cells wrapped around the UCA endothelium (Supplementary Fig. 3b), and the Notch ligand Jagged1 was abundantly expressed in the UCAs (Supplementary Fig. 3c). However, CD146^+^ or Jagged1^+^ cells were rarely detected in WJ. CD146^+^Jagged1^+^ cells were prevalent in the UCA tunica media (Supplementary Fig. 3d). Notably, more CD146^+^α-SMA^+^ cells were observed in the UCA than in WJ (Supplementary Fig. 3e).

Knockdown of either CD146 or Jagged1 resulted in reduced tube formation by UCA-PSCs or WJ-MSCs in vitro (Supplementary Fig. 4a, b). The expression of p-Akt (T308), p-Akt (S473), FHL1, and Jagged1 was decreased after UCA-PSCs were transfected with si-CD146, while it was increased after WJ-MSCs were infected with adenovirus encoding CD146 (Ad-CD146), suggesting that CD146 expression activated PI3K/AKT signaling pathway (Supplementary Fig. 4c, d). Then, we found that the AKT activator, IGF-1, activated PI3K/AKT signaling and increased the expression of FHL1 and Jagged1, whereas the AKT inhibitor, MK2206, significantly reduced the expression of FHL1 and Jagged1 in UCA-PSCs (Supplementary Fig. 4e, f). Moreover, upregulating FHL1 expression with GFP-FHL1-expressing plasmid (GFP-FHL1) resulted in an increase in Jagged1 expression while knocking down FHL1 with si-FHL1 led to the opposite effect in UCA-PSCs (Fig. 1h). Chromatin immunoprecipitation (ChIP) assays showed that FHL1 specifically binds to the Jagged1 promoter in UCA-PSCs (Fig. 1h). Furthermore, the luciferase reporter assay showed a twofold increase in Jagged1 activity after GFP-FHL1 transfection when its promoter contained the FHL1-binding site “ACGCA”. Knocking down FHL1 resulted in an ~21% reduction in Jagged1 activity (Supplementary Fig. 4g). Besides, the impairment of tube formation caused by CD146 knockdown in vitro was attenuated by Jagged1 overexpression in UCA-PSCs (Supplementary Fig. 4h, i).

Interestingly, we also found that HUVECs formed more tubes and branching points when cultured with UCA-PSC supernatant than with WJ-MSC supernatant (Supplementary Fig. 5a). Therefore, we performed LC-MS/MS to detect the secreted proteins in UCA-PSC and WJ-MSC supernatants. Among the identified 839 proteins in the supernatants, 69 of them were differentially expressed in the two groups (Supplementary Fig. 5b), and 26 were secreted at higher levels in UCA-PSC supernatants, as indicated by heatmap analysis and hierarchical clustering (Supplementary Fig. 5c). A Kyoto Encyclopedia of Genes and Genomes (KEGG) pathway analysis showed that these secretory proteins are related to “ECM-receptor interaction” and “PI3K-AKT pathways” (Supplementary Fig. 5d). GO analysis indicated that these proteins are involved with blood vessel morphogenesis and VEGF receptor 2 binding (Supplementary Fig. 5e). Six proteins were differentially expressed in UCA-PSC and WJ-MSC supernatants with an FC >2.0 (Supplementary Table 4), and four of them were angiogenic factors, IGFBP2 (2.66-fold), TINAGL1 (2.49-fold), MMP1 (2.23-fold), and IL6 (2.10-fold) (Fig. 1i). Using ELISA analysis, the UCA-PSC supernatant exhibited an approximately fivefold increase in IL6 expression compared with the WJ-MSC counterpart (Fig. 1i). A protein–protein interaction (PPI) analysis revealed IL6 as the central mediator of angiogenesis among UCA-PSC secretory proteins (Supplementary Fig. 5f).

We then applied UCA-PSCs to promote angiogenesis in the hindlimb ischemia (HLI) mouse model (Supplementary Fig. 6a). After femoral artery ligation, the mice received UCA-PSC transplantation, WJ-MSC transplantation, or PBS as control (Supplementary Fig. 6b). Both the capillary density and capillary-to-muscle fiber ratio in ischemic gastrocnemius muscles per HPF were significantly augmented in the UCA-PSC group compared with the other groups (Supplementary Fig. 6c, d). In addition, compared with the control, the number of capillaries and percentage of capillaries per muscle fiber significantly decreased after the transplantation of si-CD146-transfected UCA-PSCs, while both indexes increased notably after the treatment with WJ-MSCs infected with GFP-CD146 lentivirus (Lv-GFP-CD146) (Supplementary Fig. 6e, f). We also found significantly lower perfusion volume in the mice after the transplantation of si-CD146-transfected UCA-PSCs and higher perfusion volume in the mice after the treatment with Lv-GFP-CD146-infected WJ-MSCs by laser Doppler perfusion imaging (LDPI) (Fig. 1j). Importantly, 4 weeks after UCA-PSC transplantation, mice showed improved stepping ability with hind limbs compared with those after WJ-MSC transplantation (Supplementary Videos 1, 2).

Finally, we investigated the therapeutic effect of UCA-PSCs in a premature ovarian failure (POF) mouse model (Supplementary Fig. 7a). After cyclophosphamide (CTX) treatment, the number of blood vessels in ovaries decreased in the PBS-treated POF mice, whereas it was restored in the UCA-PSC group (Supplementary Fig. 7b, c). Furthermore, the ovarian blood perfusion rate decreased after transplantation of si-CD146-transfected UCA-PSCs compared with that in the control group, while it increased after transplantation of Lv-GFP-CD146-infected WJ-MSCs compared with the corresponding control (Supplementary Fig. 7d, e). Mice receiving PBS treatment exhibited the absence of estrus and prolonged periods of diestrus, while those in the UCA-PSC and WJ-MSC groups showed prolonged estrus stage (Supplementary Fig. 7f, g). Additionally, the ovary index was markedly increased in the UCA-PSC group (Supplementary Fig. 7h). The numbers of primordial and primary follicles were significantly higher in mice transplanted with UCA-PSCs or WJ-MSCs than that in the PBS group (Supplementary Fig. 7i). After UCA-PSC transplantation, mice showed similar serum estradiol (E_2_) levels to normal controls (Supplementary Fig. 7j). Four weeks after cell transplantation, the number of viable oocytes in POF mice of the UCA-PSC group (21.67 ± 2.54) was significantly higher than that in the PBS group (10.67 ± 1.65) (*P* < 0.01) but similar to that in the normal control group (26.33 ± 1.36) (Fig. 1k).

In conclusion, our study found that UCA-PSCs showed greater angiogenic and proangiogenic potential than WJ-MSCs in vivo and ex vivo, which was related to cell-to-cell communication through the CD146/AKT/FHL1/Jagged1 signaling pathway and IL6 paracrine activity (Fig. 1l). Moreover, UCA-PSCs sufficiently restored blood supply and organ function in HLI and POF mouse models. Therefore, we identified UCA-PSCs as a new kind of effective “seeding” cells for revascularization in regenerative medicine.

## Supplementary information

Supplementary Figure1

Supplementary Figure2

Supplementary Figure3

Supplementary Figure4

Supplementary Figure5

Supplementary Figure6

Supplementary Figure7

Supplementary Table 1

Supplementary Table 2

Supplementary Table 3

Supplementary Table 4

Supplementary video1-UCA-PSCs

Supplementary video2-WJ-MSCs

Supplementary Materials

Raw data

## Data Availability

All data generated or analyzed during this study are included in this article. The data that support the findings of this study are also available on request from the corresponding author. RNA-seq, proteome, and secretory proteomic data during this study are included in this published article and its Supplementary Information files.

## References

[1] Ding L (2018). Transplantation of UC-MSCs on collagen scaffold activates follicles in dormant ovaries of POF patients with long history of infertility. Sci. China Life Sci..

[2] Berthiaume A-A (2018). Dynamic remodeling of pericytes in vivo maintains capillary coverage in the adult mouse brain. Cell Rep..

[3] Esteves CL (2017). Isolation and characterization of equine native MSC populations. Stem Cell Res Ther..

[4] Xu, L. et al. Different angiogenic potentials of mesenchymal stem cells derived from umbilical artery, umbilical vein, and Wharton’s jelly. *Stem Cells Int.* **2017**, 3175748 (2017). 10.1155/2017/3175748 PMC5569878 28874910

[5] Luo Y (2019). CD146-HIF-1α hypoxic reprogramming drives vascular remodeling and pulmonary arterial hypertension. Nat. Commun..

