# Supplementary figures and images for: Umbilical cord artery-derived perivascular stem cells for treatment of ovarian failure through CD146 signaling
Source: Signal Transduct Target Ther. 2022 Jul 13;7:223. doi: 10.1038/s41392-022-01029-4 (PMC9276707; doi:10.1038/s41392-022-01029-4)

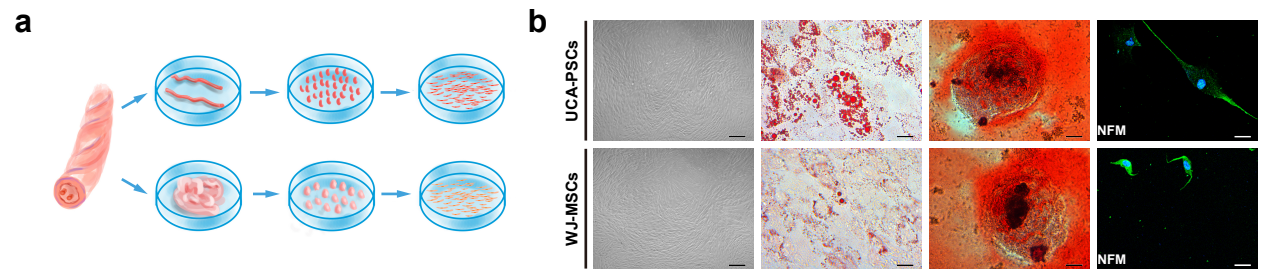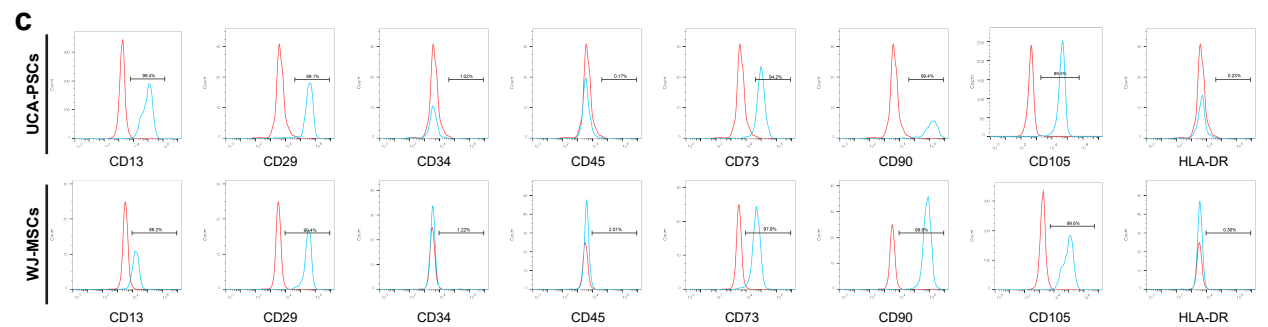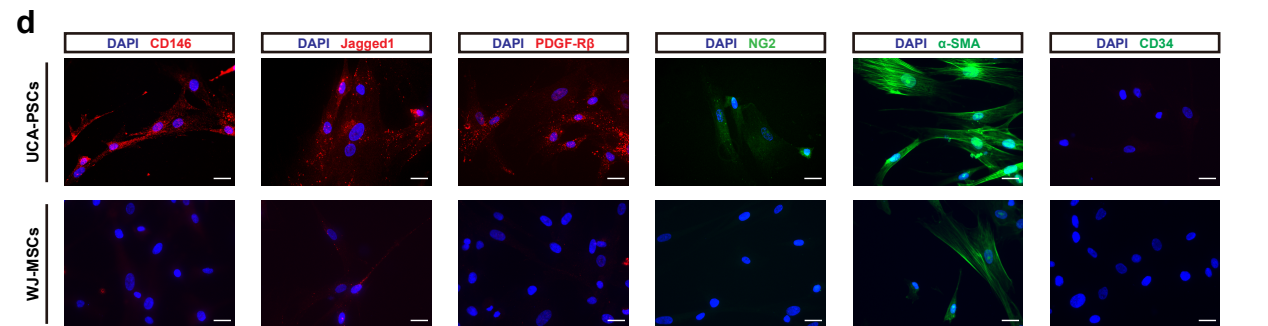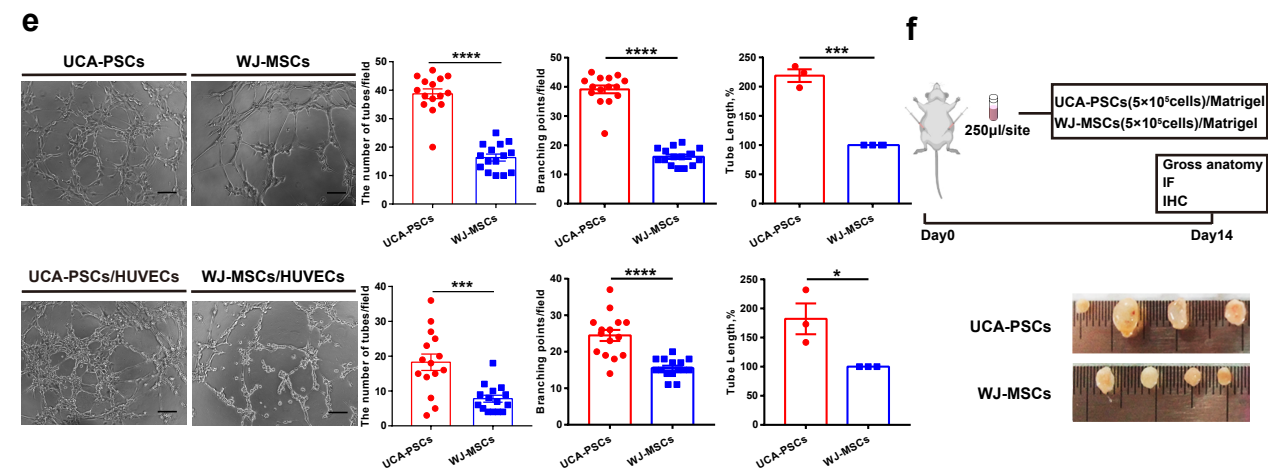

Supplement: Supplementary file 1 — Supplementary Figure1 [file 41392_2022_1029_MOESM1_ESM.pdf]

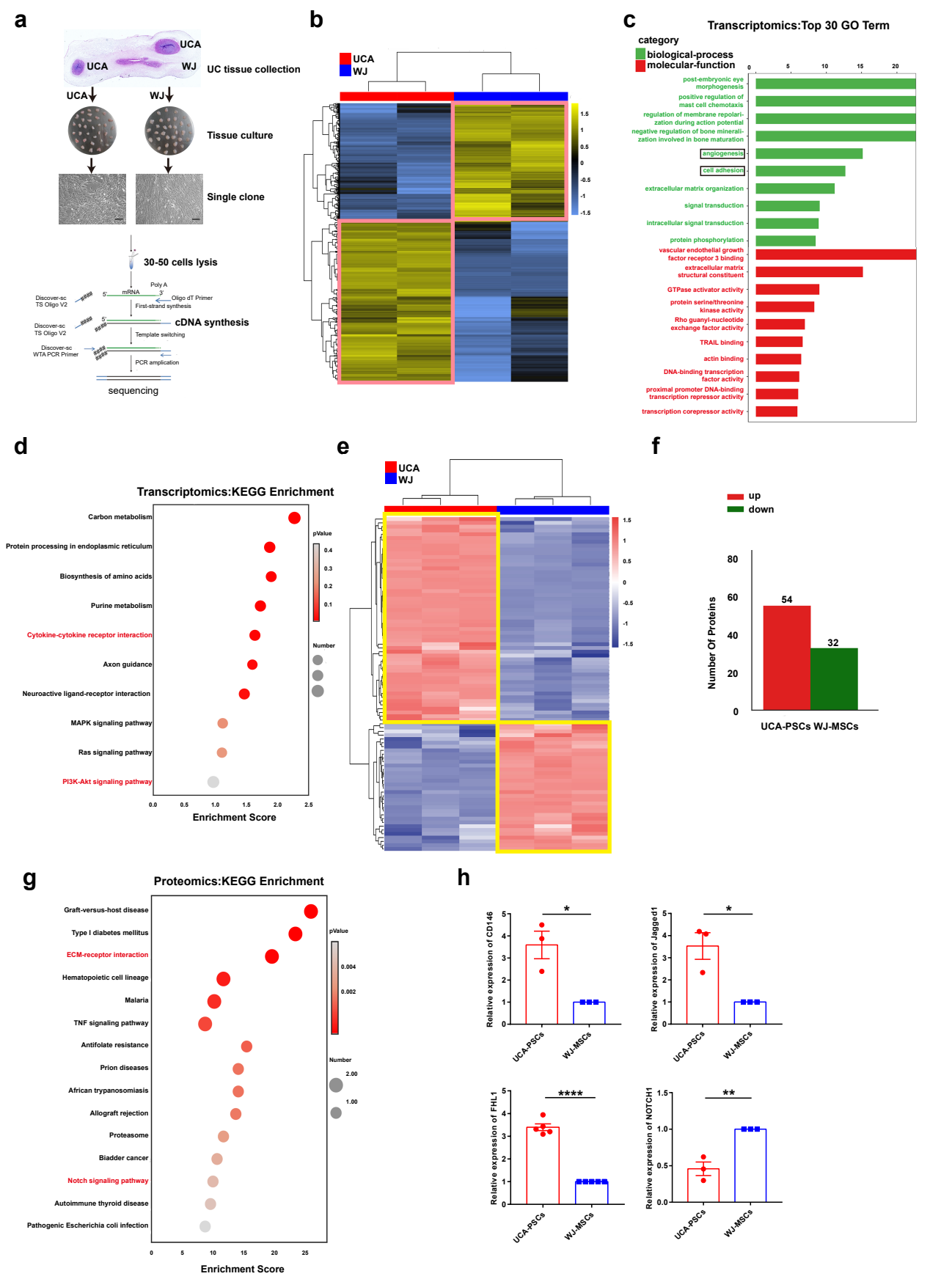

Supplement: Supplementary file 2 — Supplementary Figure2 [file 41392_2022_1029_MOESM2_ESM.pdf]

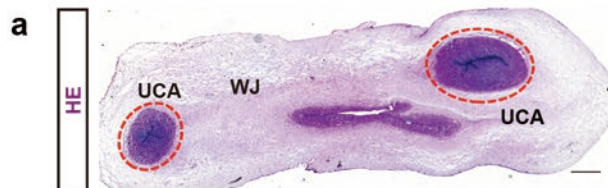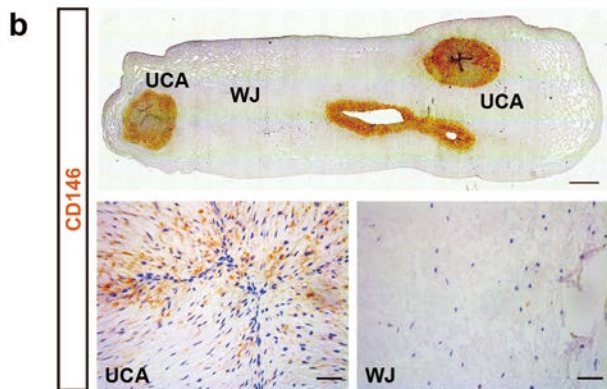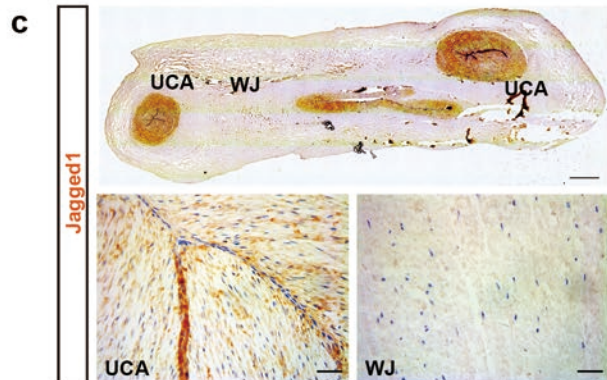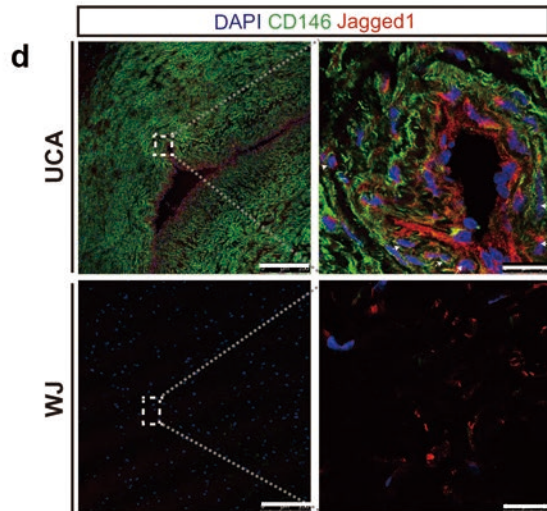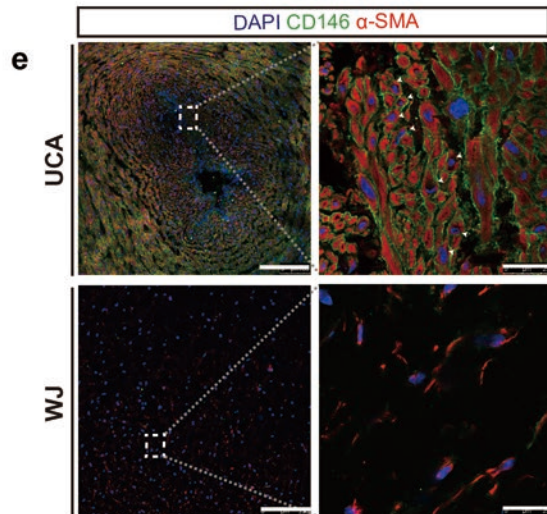

Supplement: Supplementary file 3 — Supplementary Figure3 [file 41392_2022_1029_MOESM3_ESM.pdf]

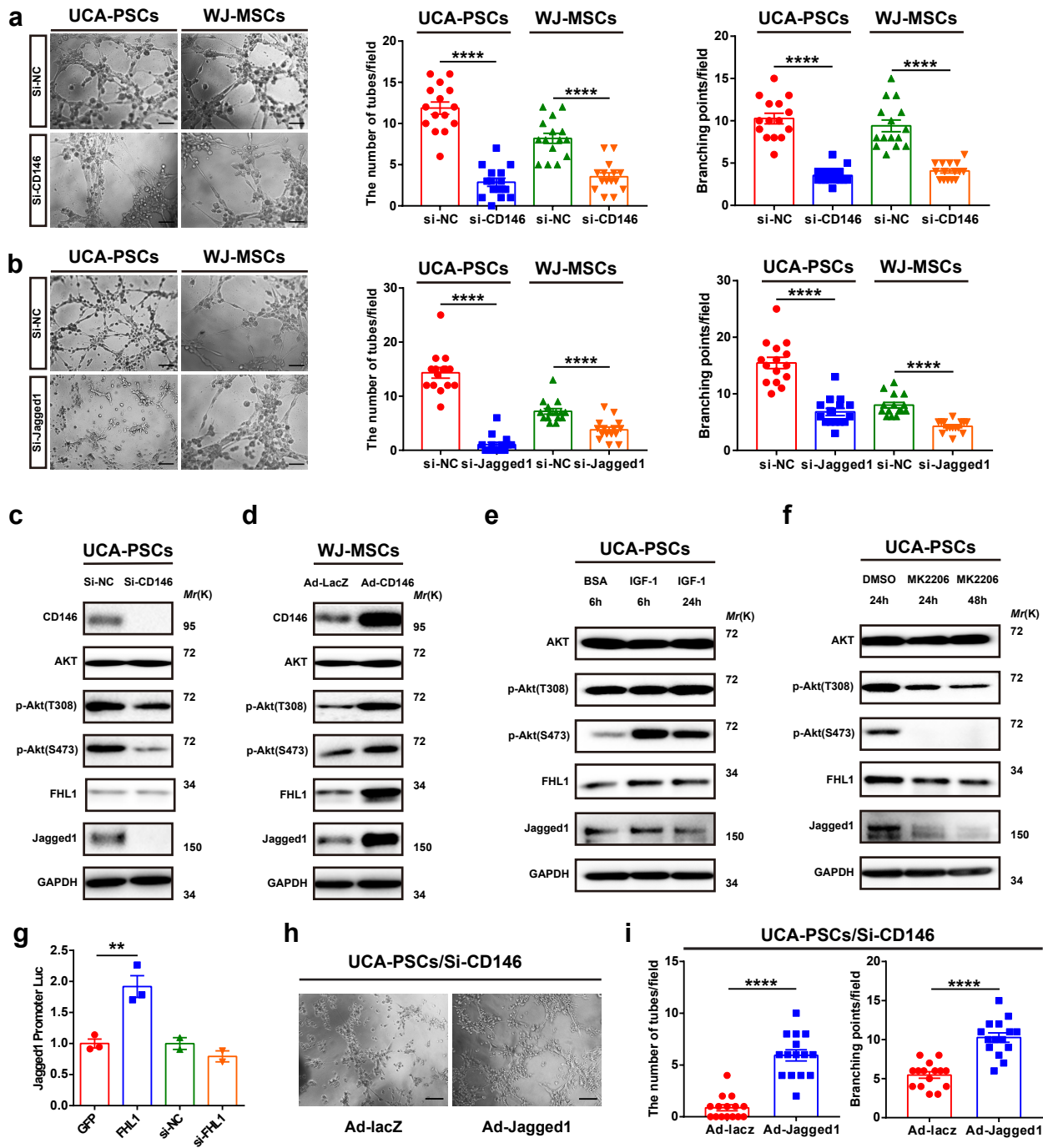

Supplement: Supplementary file 4 — Supplementary Figure4 [file 41392_2022_1029_MOESM4_ESM.pdf]

C

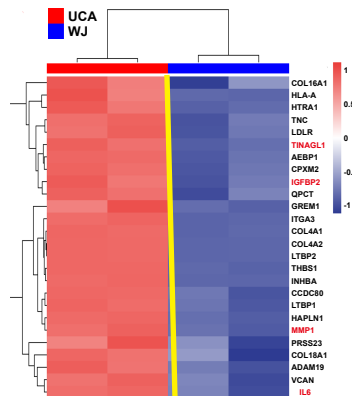

f

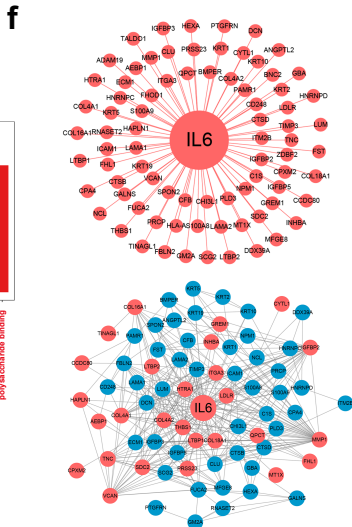

Supplement: Supplementary file 5 — Supplementary Figure5 [file 41392_2022_1029_MOESM5_ESM.pdf]

**a**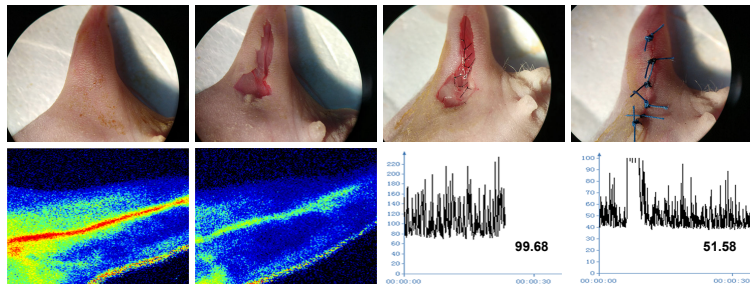**b**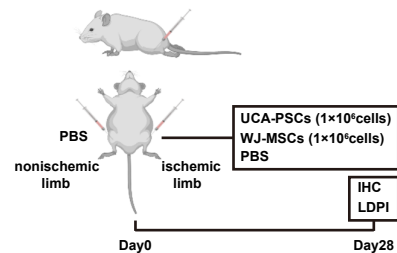**c**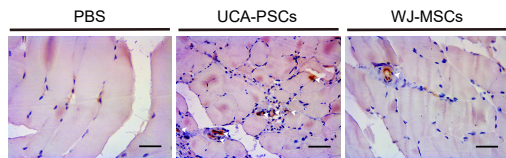**e**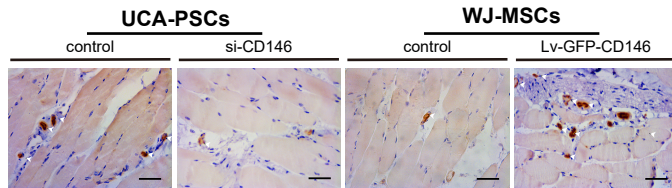**d**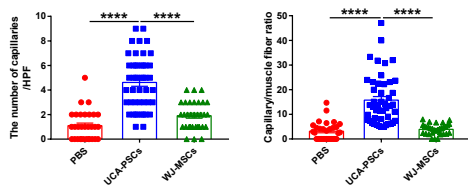**f**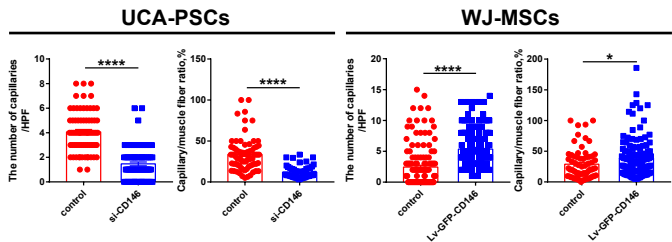

Supplement: Supplementary file 6 — Supplementary Figure6 [file 41392_2022_1029_MOESM6_ESM.pdf]

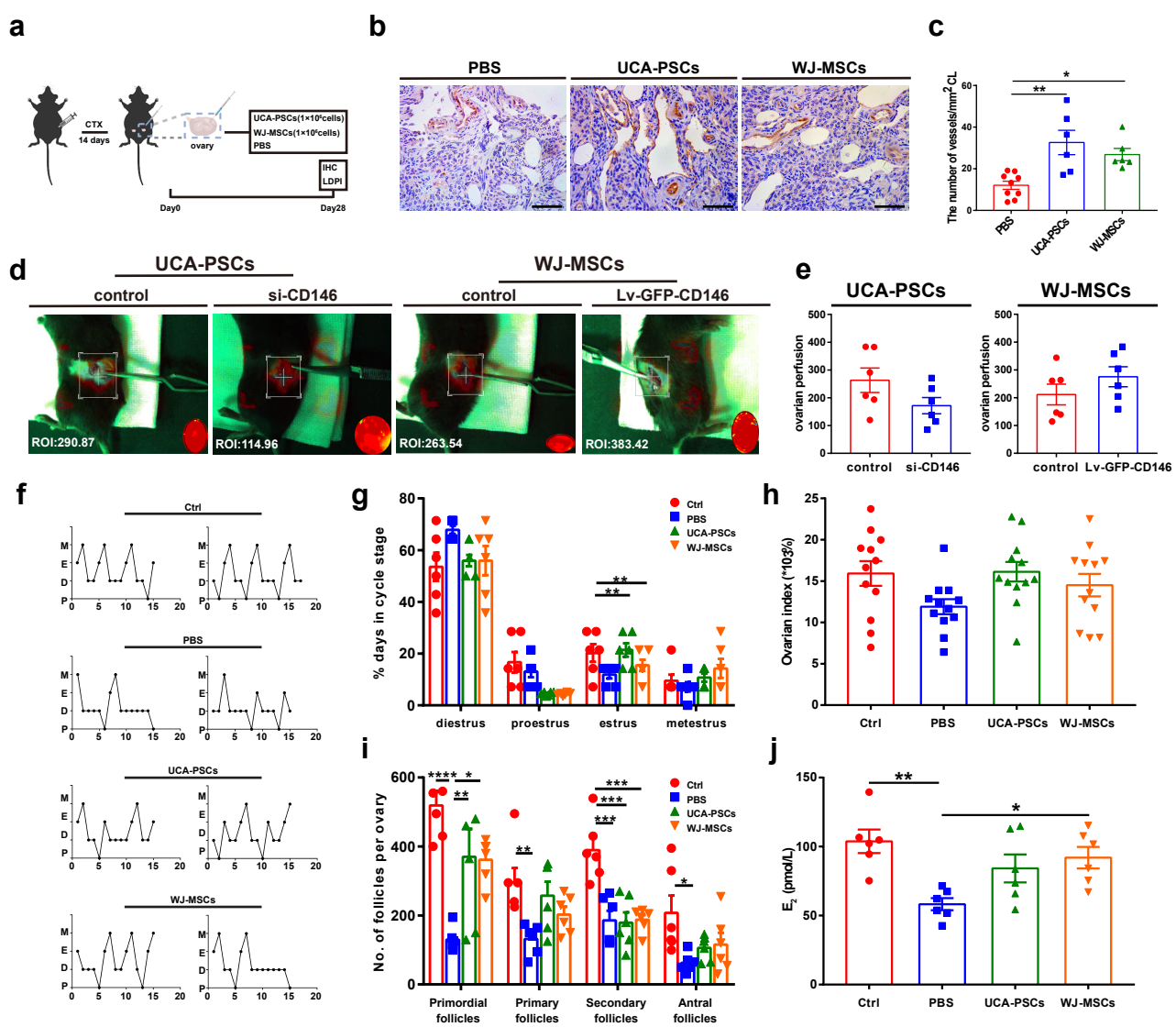

Supplement: Supplementary file 7 — Supplementary Figure7 [file 41392_2022_1029_MOESM7_ESM.pdf]
