# Supplementary material for: Umbilical cord artery-derived perivascular stem cells for treatment of ovarian failure through CD146 signaling
Source: Signal Transduct Target Ther. 2022 Jul 13;7:223. doi: 10.1038/s41392-022-01029-4 (PMC9276707; doi:10.1038/s41392-022-01029-4)
Supplement: Supplementary file 9 — Supplementary Table 2 [file 41392_2022_1029_MOESM9_ESM.pdf]

Accession	Gene Name	Descripti	Coverage	# Peptide	# PSMs	# Unique	# AAs	MW [kDa]
Q09666	AHNAK	Neuroblas	69	254	670	252	5890	628.7
Q15149	PLEC	Plectin C	56	255	593	248	4684	531.5
P21333	FLNA	Filamin-A	61	134	753	116	2647	280.6
P35579	MYH9	Myosin-9	62	135	810	112	1960	226.4
Q9Y490	TLN1	Talin-1 C	70	144	442	128	2541	269.6
O75369	FLNB	Filamin-B	57	124	404	107	2602	278
Q14315	FLNC	Filamin-C	53	128	413	110	2725	290.8
Q14204	DYNC1H1	Cytoplasm	42	172	327	172	4646	532.1
Q13813	SPTAN1	Spectrin	56	127	256	3	2472	284.4
A0A0D9SF5	SPTAN1	Spectrin	55	125	250	1	2457	282.7
P35580	MYH10	Myosin-10	48	102	279	81	1976	228.9
H3BPE1	MACF1	Microtubu	22	148	198	3	7555	856.3
Q9UPN3	MACF1	Microtubu	22	145	195	2	7388	837.8
Q01082	SPTBN1	Spectrin	47	105	205	105	2364	274.4
P12814	ACTN1	Alpha-act	64	55	344	9	892	103
P18206	VCL	Vinculin	66	74	272	74	1134	123.7
P02452	COL1A1	Collagen	63	64	216	64	1464	138.9
Q9NZM1	MYOF	Myoferlin	50	95	192	95	2061	234.6
A0A087WVQ	CLTC	Clathrin	44	63	192	63	1679	191.9
P46821	MAP1B	Microtubu	40	84	182	82	2468	270.5
P78527	PRKDC	DNA-depen	28	110	164	110	4128	468.8
H9KV75	ACTN1	Alpha-act	60	46	318	3	822	94.8
P46940	IQGAP1	Ras GTPas	43	66	180	64	1657	189.1
P02751	FN1	Fibronect	37	65	182	65	2386	262.5
O43707	ACTN4	Alpha-act	63	56	296	39	911	104.8
Q9P2E9	RRBP1	Ribosome-	67	72	173	71	1410	152.4
Q99715	COL12A1	Collagen	35	87	160	87	3063	332.9
P49327	FASN	Fatty aci	34	67	144	67	2511	273.3
P11021	HSPA5	Endoplasm	63	45	283	42	654	72.3
P14618	PKM	Pyruvate	71	36	309	16	531	57.9
P08238	HSP90AB1	Heat shoc	61	46	273	29	724	83.2
P11142	HSPA8	Heat shoc	77	46	221	20	646	70.9
A0A087WTA	COL1A2	Collagen	54	51	145	51	1364	129.1
P07814	EPRS	Bifunctic	47	62	124	61	1512	170.5
P07900	HSP90AA1	Heat shoc	57	45	232	30	732	84.6
P08670	VIM	Vimentin	70	42	578	36	466	53.6
P13639	EEF2	Elongatic	59	49	230	48	858	95.3
P27816	MAP4	Microtubu	55	59	129	1	1152	120.9
E7EVA0	MAP4	Microtubu	27	60	130	1	2297	245.3
P02545	LMNA	Prelamin-	68	47	188	46	664	74.1
P53396	ACLY	ATP-citra	51	50	153	50	1101	120.8
P34932	HSPA4	Heat shoc	66	53	113	48	840	94.3
A0A2R8Y79	ACTB	Actin, cy	68	18	899	9	309	34.1
P08133	ANXA6	Annexin A	63	44	159	16	673	75.8
E9PGZ1	CALD1	Caldesmon	60	40	168	3	536	61.7
E7EX44	CALD1	Caldesmon	61	39	170	1	557	64.1
Q07065	CKAP4	Cytoskele	66	38	170	36	602	66
Q05682	CALD1	Caldesmon	42	39	170	1	793	93.2
Q92616	GCN1	eIF-2-alp	31	71	112	71	2671	292.6
P05787	KRT8	Keratin,	71	41	239	32	483	53.7

P26038	MSN	Moesin OS	65	46	202	32	577	67.8
P14625	HSP90B1	Endoplasm	47	43	215	41	803	92.4
P12111	COL6A3	Collagen	25	69	98	55	3177	343.5
P50454	SERPINH1	Serpin H1	63	25	178	25	418	46.4
P55072	VCP	Transitic	61	41	122	41	806	89.3
P62736	ACTA2	Actin, ac	62	21	549	12	377	42
P13667	PDIA4	Protein d	57	38	133	38	645	72.9
Q9Y4L1	HYOU1	Hypoxia u	44	41	108	41	999	111.3
Q14764	MVP	Major vau	57	38	120	38	893	99.3
P04406	GAPDH	Glycerald	69	22	416	22	335	36
P13797	PLS3	Plastin-3	54	36	139	29	630	70.8
P07237	P4HB	Protein d	60	37	205	37	508	57.1
A0A024R4E	HDLBP	High dens	39	52	120	51	1268	141.4
P22314	UBA1	Ubiquitin	47	40	101	40	1058	117.8
H3BR70	PKM	Pyruvate	56	23	203	3	366	40.2
Q86UP2	KTN1	Kinectin	44	61	83	61	1357	156.2
P30101	PDIA3	Protein d	63	36	159	30	505	56.7
P33176	KIF5B	Kinesin-1	50	45	76	45	963	109.6
O00410	IPO5	Importin-	40	35	91	33	1097	123.6
P53621	COPA	Coatomer	42	50	95	50	1224	138.3
P04264	KRT1	Keratin,	50	40	121	33	644	66
P10809	HSPD1	60 kDa he	76	36	114	36	573	61
P50990	CCT8	T-complex	63	34	107	34	548	59.6
Q07954	LRP1	Prolow-de	15	66	89	66	4544	504.3
Q01813	PFKP	ATP-depen	45	34	102	28	784	85.5
P68104	EEF1A1	Elongatic	70	26	302	26	462	50.1
P06733	ENO1	Alpha-enc	61	24	169	22	434	47.1
Q7KZF4	SND1	Staphyloc	45	39	106	39	910	101.9
P42704	LRPPRC	Leucine-r	41	52	85	52	1394	157.8
P12270	TPR	Nucleoprc	26	59	76	58	2363	267.1
P38646	HSPA9	Stress-7C	48	35	105	34	679	73.6
E7EUU4	EIF4G1	Eukaryoti	32	45	86	1	1560	171.5
Q04637	EIF4G1	Eukaryoti	31	45	86	1	1599	175.4
P00558	PGK1	Phosphogl	76	29	125	25	417	44.6
P27797	CALR	Calreticu	60	26	146	26	417	48.1
E7EX90	DCTN1	Dynactin	42	43	74	43	1256	139
P07355	ANXA2	Annexin A	73	31	228	31	339	38.6
P68366	TUBA4A	Tubulin a	55	21	231	6	448	49.9
P02461	COL3A1	Collagen	33	34	99	34	1466	138.5
P07437	TUBB	Tubulin b	68	21	259	4	444	49.6
F5H5D3	TUBA1C	Tubulin a	50	22	201	7	519	57.7
P50991	CCT4	T-complex	65	30	76	29	539	57.9
Q8WUM4	PDCD6IP	Programme	52	44	77	44	868	96
O75083	WDR1	WD repeat	51	27	107	27	606	66.2
P05783	KRT18	Keratin,	71	34	124	33	430	48
P21980	TGM2	Protein-g	57	30	121	21	687	77.3
P48643	CCT5	T-complex	71	36	87	35	541	59.6
P68371	TUBB4B	Tubulin b	67	21	241	3	445	49.8
P52272	HNRNPM	Heterogen	57	38	94	38	730	77.5
P49368	CCT3	T-complex	55	30	86	30	545	60.5
Q92598	HSPH1	Heat shoc	45	35	74	30	858	96.8

P60842	EIF4A1	Eukaryoti	63	25	107	14	406	46.1
P16615	ATP2A2	Sarcoplas	35	36	77	36	1042	114.7
P78371	CCT2	T-complex	58	26	79	26	535	57.5
E7ETU9	PLOD2	Procollag	37	24	106	1	703	81.1
P35221	CTNNA1	Catenin a	48	38	69	38	906	100
A0A0G2JIWHSPA1B		Heat shoc	55	30	105	24	642	70.1
P04083	ANXA1	Annexin A	67	22	111	22	346	38.7
P15311	EZR	Ezrin OS=	58	39	133	26	586	69.4
Q15075	EEA1	Early end	37	48	56	48	1411	162.4
P49588	AARS	Alanine--	46	37	72	37	968	106.7
Q01518	CAP1	Adenylyl	63	27	107	25	475	51.9
Q14152	EIF3A	Eukaryoti	34	48	86	48	1382	166.5
Q6ZN40	TPM1	Tropomyos	65	31	146	2	326	37.4
E9PGC8	MAP1A	Microtubu	16	45	65	44	3041	331.1
P17987	TCP1	T-complex	58	24	71	24	556	60.3
P11047	LAMC1	Laminin s	27	37	67	37	1609	177.5
P53618	COPB1	Coatomer	37	28	70	28	953	107.1
Q01995	TAGLN	Transgeli	85	19	243	18	201	22.6
F8WD26	LMO7	LIM domai	31	44	60	3	1631	186.1
P12956	XRCC6	X-ray rep	54	35	77	35	609	69.8
P50395	GDI2	Rab GDP d	63	27	81	19	445	50.6
P35908	KRT2	Keratin,	48	31	66	23	639	65.4
P54652	HSPA2	Heat shoc	57	35	93	19	639	70
E5RK69	ANXA6	Annexin C	59	30	96	2	460	51.7
Q9Y678	COPG1	Coatomer	46	32	77	30	874	97.7
O00469	PLOD2	Procollag	34	24	94	1	737	84.6
P04075	ALDOA	Fructose-	63	24	172	19	364	39.4
P05023	ATP1A1	Sodium/pc	41	35	60	35	1023	112.8
P13645	KRT10	Keratin,	45	30	72	24	584	58.8
Q99832	CCT7	T-complex	58	28	71	28	543	59.3
A0A0S2Z4CTPM1		Tropomyos	64	29	136	2	284	32.7
E9PMS6	LMO7	LIM domai	38	42	56	1	1275	145.3
A7XZE4	TPM2	Beta trop	63	26	163	6	284	33
Q9P0K7	RAI14	Ankycorbi	38	39	54	39	980	110
P63010	AP2B1	AP-2 comp	35	34	72	15	937	104.5
P19367	HK1	Hexokinas	38	36	71	33	917	102.4
P06576	ATP5F1B	ATP synth	53	21	82	21	529	56.5
Q13885	TUBB2A	Tubulin b	54	18	223	2	445	49.9
J3KN16	ECPAS	Proteasom	23	39	52	39	2017	223.6
P08727	KRT19	Keratin,	75	31	81	19	400	44.1
P41252	IARS	Isoleucin	34	41	63	41	1262	144.4
Q14195	DPYSL3	Dihydropy	52	20	77	16	570	61.9
P35606	COPB2	Coatomer	41	32	69	32	906	102.4
P11586	MTHFD1	C-1-tetra	37	33	61	32	935	101.5
P07942	LAMB1	Laminin s	24	42	64	42	1786	197.9
Q96AY3	FKBP10	Peptidyl-	42	25	102	24	582	64.2
P31948	STIP1	Stress-in	62	38	80	38	543	62.6
O00159	MYO1C	Unconvent	36	35	62	35	1063	121.6
Q08211	DHX9	ATP-depen	31	37	71	37	1270	140.9
P13674	P4HA1	Prolyl 4-	54	28	80	28	534	61
Q00839	HNRNPU	Heterogen	34	28	84	2	825	90.5

A0A1W2PPS	HNRNPU	Heterogen	33	27	81	1	804	88.3
Q14008	CKAP5	Cytoskele	22	41	51	41	2032	225.4
E9PK25	CFL1	Cofilin-1	61	20	131	15	204	22.7
Q6P2Q9	PRPF8	Pre-mRNA-	21	44	58	44	2335	273.4
H0YFD6	HADHA	Trifuncti	36	27	62	27	792	86.3
Q9NYU2	UGGT1	UDP-gluco	28	37	61	35	1555	177.1
Q13509	TUBB3	Tubulin b	52	17	138	4	450	50.4
Q8IVF2	AHNAK2	Protein A	21	44	57	42	5795	616.2
Q86VP6	CAND1	Cullin-as	31	35	74	35	1230	136.3
P54136	RARS	Arginine-	46	31	66	31	660	75.3
F5H365	SEC23A	Protein t	37	24	73	22	736	82.9
Q16643	DBN1	Drebrin C	48	24	56	24	649	71.4
O43852	CALU	Calumenin	63	20	61	20	315	37.1
P60174	TPI1	Triosepho	71	17	101	17	286	30.8
P29401	TKT	Transketo	51	27	73	27	623	67.8
P11216	PYGB	Glycogen	43	35	62	29	843	96.6
Q8NF91	SYNE1	Nesprin-1	6	47	51	45	8797	1010.5
P05556	ITGB1	Integrin	32	23	87	23	798	88.4
Q10567	AP1B1	AP-1 comp	32	31	56	12	949	104.6
Q92896	GLG1	Golgi app	36	40	56	40	1179	134.5
P62258	YWHAE	14-3-3 pr	72	24	95	21	255	29.2
O60664	PLIN3	Perilipin	64	23	78	23	434	47
P35527	KRT9	Keratin,	50	27	70	25	623	62
P19338	NCL	Nucleolin	37	36	84	36	710	76.6
P02786	TFRC	Transferr	41	27	53	27	760	84.8
P27824	CANX	Calnexin	41	24	85	16	592	67.5
Q92499	DDX1	ATP-depen	47	32	64	32	740	82.4
Q14697	GANAB	Neutral a	31	28	78	5	944	106.8
A0A1W2PQ5	DDX17	Probable	47	31	71	21	731	80.4
P41250	GARS	Glycine--	45	29	71	29	739	83.1
O60506	SYNCRIP	Heterogen	43	24	57	4	623	69.6
J3KN67	TPM3	Tropomyos	55	26	123	1	285	33.2
P35998	PSMC2	26S prote	67	26	60	26	433	48.6
Q9P2J5	LARS	Leucine--	31	33	49	33	1176	134.4
Q16658	FSCN1	Fascin OS	46	23	89	23	493	54.5
P49792	RANBP2	E3 SUMO-p	18	49	58	48	3224	358
Q14789	GOLGB1	Golgin su	16	45	48	44	3259	375.8
P61158	ACTR3	Actin-rel	69	22	70	22	418	47.3
P08758	ANXA5	Annexin A	66	22	103	22	320	35.9
Q08378	GOLGA3	Golgin su	29	38	47	38	1498	167.3
Q13200	PSMD2	26S prote	34	28	58	28	908	100.1
O75643	SNRNP200	U5 small	19	36	49	36	2136	244.4
O95782	AP2A1	AP-2 comp	34	33	59	24	977	107.5
E9PLK3	NPEPPS	Aminopept	36	32	61	32	915	102.9
Q06210	GFPT1	Glutamine	46	28	52	25	699	78.8
Q02952	AKAP12	A-kinase	21	33	46	33	1782	191.4
P25705	ATP5F1A	ATP synth	56	29	70	29	553	59.7
O60701	UGDH	UDP-gluco	56	23	60	23	494	55
Q99460	PSMD1	26S prote	32	29	54	29	953	105.8
O60763	USO1	General v	37	27	45	27	962	107.8
O00571	DDX3X	ATP-depen	49	31	59	7	662	73.2

Q5TCU3	TPM2	Tropomyos	62	25	141	4	284	32.8
Q7Z6Z7	HUWE1	E3 ubiqui	11	42	52	42	4374	481.6
P67936	TPM4	Tropomyos	58	22	140	4	248	28.5
P63104	YWHAZ	14-3-3 pr	57	16	144	11	245	27.7
P54577	YARS	Tyrosine-	49	30	57	30	528	59.1
Q14247	CTTN	Src subst	49	29	56	29	550	61.5
Q02809	PLOD1	Procollag	40	25	52	24	727	83.5
P09874	PARP1	Poly [ADF	33	29	41	29	1014	113
Q5T4S7	UBR4	E3 ubiqui	8	41	47	41	5183	573.5
P26639	TARS	Threonine	42	29	67	29	723	83.4
D6REX3	SEC31A	Protein t	26	27	51	27	1251	136.1
Q96HC4	PDLIM5	PDZ and L	48	23	53	23	596	63.9
P46939	UTRN	Utrophin	14	45	46	43	3433	394.2
P18669	PGAM1	Phosphogl	58	14	75	14	254	28.8
Q14974	KPNB1	Importin	31	25	66	25	876	97.1
P48444	ARCN1	Coatomer	49	25	61	25	511	57.2
P17980	PSMC3	26S prote	55	21	52	21	439	49.2
P04843	RPN1	Dolichyl-	50	28	66	28	607	68.5
P28838	LAP3	Cytosol a	57	25	44	25	519	56.1
P48681	NES	Nestin OS	24	37	44	37	1621	177.3
Q16881	TXNRD1	Thioredox	40	23	53	22	649	70.9
P13010	XRCC5	X-ray rep	37	27	64	27	732	82.7
P07384	CAPN1	Calpain-1	36	24	52	24	714	81.8
Q3SY69	ALDH1L2	Mitochond	37	30	48	27	923	101.7
P15144	ANPEP	Aminopept	32	31	44	31	967	109.5
P37802	TAGLN2	Transgeli	79	16	93	15	199	22.4
P40926	MDH2	Malate de	62	18	92	18	338	35.5
P17655	CAPN2	Calpain-2	40	23	60	23	700	79.9
A0A087WTT	PABPC1	Polyadeny	35	21	60	15	522	58.5
Q12906	ILF3	Interleuk	28	25	57	25	894	95.3
P80723	BASP1	Brain aci	78	17	50	17	227	22.7
E9PK54	HSPA8	Heat shoc	86	14	86	1	183	19.9
J3KTA4	DDX5	Probable	50	28	66	19	614	69
H0Y4R1	IMPDH2	Inosine-5	39	18	38	16	470	51
O75534	CSDE1	Cold shoc	36	33	53	33	798	88.8
E9PKU7	GANAB	Neutral a	31	24	67	1	852	96.5
P26641	EEF1G	Elongatic	42	21	79	21	437	50.1
P35241	RDX	Radixin C	42	31	100	13	583	68.5
P30153	PPP2R1A	Serine/th	43	23	57	18	589	65.3
P20700	LMNB1	Lamin-B1	46	29	48	24	586	66.4
P63244	RACK1	Receptor	70	19	61	19	317	35.1
P46060	RANGAP1	Ran GTPas	53	24	35	24	587	63.5
A0A024R57	EHD1	EH domair	41	24	58	18	548	61.9
P23634	ATP2B4	Plasma me	27	32	46	24	1241	137.8
Q9Y696	CLIC4	Chloride	69	16	48	15	253	28.8
Q9ULV4	CORO1C	Coronin-1	36	23	67	22	474	53.2
P02768	ALB	Serum alb	52	26	56	26	609	69.3
A0A0C4DGE	CAST	Calpastat	45	28	44	1	754	81
P06748	NPM1	Nucleophc	45	13	85	13	294	32.6
P26640	VARS	Valine--t	22	27	47	27	1264	140.4
B7Z645	SYNCRIP	Synaptota	44	19	48	1	464	52

P62424	RPL7A	60S ribos	48	18	70	18	266	30
K7ELL7	PRKCSH	Glucosida	44	23	61	23	535	60.2
P27348	YWHAQ	14-3-3 pr	63	17	76	11	245	27.7
O75874	IDH1	Isocitrat	64	25	57	25	414	46.6
P36578	RPL4	60S ribos	50	23	70	23	427	47.7
P11717	IGF2R	Cation-in	15	34	40	34	2491	274.2
P27708	CAD	CAD prote	19	34	41	34	2225	242.8
A0A087WWU	TPM3	Tropomyos	65	22	102	3	227	26.4
P47895	ALDH1A3	Aldehyde	36	21	51	19	512	56.1
K7ENT6	TPM4	Tropomyos	61	22	127	2	247	28.5
P11413	G6PD	Glucose-6	47	24	58	24	515	59.2
P31939	ATIC	Bifunctic	46	23	43	23	592	64.6
Q03252	LMNB2	Lamin-B2	46	30	52	25	620	69.9
Q06830	PRDX1	Peroxired	64	13	93	10	199	22.1
G3V3H3	KLC1	Kinesin l	39	24	47	17	609	68.7
Q9UHB6	LIMA1	LIM domai	36	26	48	26	759	85.2
Q8NBS9	TXNDC5	Thioredox	42	19	63	19	432	47.6
P31150	GDI1	Rab GDP d	50	19	50	11	447	50.6
P22102	GART	Trifuncti	35	31	47	31	1010	107.7
Q92900	UPF1	Regulator	29	28	39	28	1129	124.3
Q16555	DPYSL2	Dihydropy	37	16	48	12	572	62.3
Q5SW79	CEP170	Centrosom	23	32	42	30	1584	175.2
P56192	MARS	Methionin	35	24	44	24	900	101.1
Q9Y4G6	TLN2	Talin-2 C	11	27	58	10	2542	271.4
Q9BUF5	TUBB6	Tubulin b	46	16	100	8	446	49.8
Q5JRX3	PITRM1	Presequen	32	29	41	29	1037	117.3
P07195	LDHB	L-lactate	42	17	59	16	334	36.6
Q13310	PABPC4	Polyadeny	30	22	52	3	644	70.7
P46459	NSF	Vesicle-f	33	25	50	24	744	82.5
Q16531	DDB1	DNA damag	25	27	42	27	1140	126.9
Q14192	FHL2	Four and	70	18	47	18	279	32.2
Q9Y3I0	RTCB	tRNA-spli	42	18	42	18	505	55.2
P62195	PSMC5	26S prote	55	21	51	20	406	45.6
Q15233	NONO	Non-POU d	44	21	46	20	471	54.2
O15460	P4HA2	Prolyl 4-	47	23	59	23	535	60.9
H0YNH8	UACA	Uveal aut	23	35	41	34	1401	161.2
E9PKG1	PRMT1	Protein a	56	18	41	18	325	37.7
Q15019	SEPT2	Septin-2	68	18	44	18	361	41.5
B4DY08	HNRNPC	Heterogen	51	22	71	20	288	32
P49748	ACADVL	Very long	42	23	39	23	655	70.3
P23396	RPS3	40S ribos	77	19	87	19	243	26.7
P39023	RPL3	60S ribos	42	21	67	21	403	46.1
P00338	LDHA	L-lactate	42	18	113	17	332	36.7
P21291	CSRP1	Cysteine	68	12	62	4	193	20.6
P49411	TUFM	Elongatic	51	20	43	20	452	49.5
O43390	HNRNPR	Heterogen	33	21	45	14	633	70.9
Q16851	UGP2	UTP--gluc	42	22	53	22	508	56.9
Q96AC1	FERMT2	Fermitin	39	24	56	24	680	77.8
Q9C0C2	TNKS1BP1	182 kDa t	22	30	36	30	1729	181.7
P55884	EIF3B	Eukaryoti	27	21	40	21	814	92.4
E7EVY3	CAST	Calpastat	43	24	40	1	693	74.9

B4DKY1	CARS	Cysteine-	34	23	43	22	739	84.2
O60841	EIF5B	Eukaryoti	22	28	37	28	1220	138.7
E9PDF6	MYO1B	Unconvent	27	30	44	30	1107	128.4
P06756	ITGAV	Integrin	26	26	36	26	1048	116
Q8NC51	SERBP1	Plasminog	43	23	51	23	408	44.9
O94925	GLS	Glutamina	33	18	49	17	669	73.4
D3DQV9	EIF4G2	Eukaryoti	35	31	51	31	907	102.3
Q15365	PCBP1	Poly(rC)-	53	14	44	8	356	37.5
A0A2R8Y6J	RPL5	60S ribos	44	14	64	14	238	27
F5GZS6	SLC3A2	4F2 cell-	29	14	44	14	599	64.8
O15523	DDX3Y	ATP-depen	41	27	47	3	660	73.1
P17301	ITGA2	Integrin	23	23	38	23	1181	129.2
H7BYY1	TPM1	Tropomyos	59	21	100	6	248	28.7
Q9UHB9	SRP68	Signal re	42	24	37	24	627	70.7
P11387	TOP1	DNA topoi	38	31	43	31	765	90.7
P06737	PYGL	Glycogen	39	32	43	26	847	97.1
P54886	ALDH18A1	Delta-1-p	33	25	44	25	795	87.2
Q9BSJ8	ESYT1	Extended	25	24	35	24	1104	122.8
Q93008	USP9X	Probable	14	30	36	30	2570	292.1
P36871	PGM1	Phosphogl	42	24	47	24	562	61.4
P30622	CLIP1	CAP-Gly d	24	33	37	27	1438	162.1
P15170	GSPT1	Eukaryoti	45	22	38	22	499	55.7
O43175	PHGDH	D-3-phosp	41	16	40	16	533	56.6
Q15084	PDIA6	Protein d	43	17	57	17	440	48.1
Q9Y262	EIF3L	Eukaryoti	39	20	35	20	564	66.7
P05455	SSB	Lupus La	46	25	41	25	408	46.8
Q16222	UAP1	UDP-N-ace	35	19	43	18	522	58.7
Q13263	TRIM28	Transcrip	35	24	41	24	835	88.5
P23284	PPIB	Peptidyl-	51	14	93	13	216	23.7
P13489	RNH1	Ribonucle	48	19	52	19	461	49.9
P04792	HSPB1	Heat shoc	66	13	78	13	205	22.8
P55060	CSE1L	Exportin-	26	24	42	24	971	110.3
P61981	YWHAG	14-3-3 pr	64	16	79	11	247	28.3
P12081	HARS	Histidine	50	25	44	19	509	57.4
O95373	IPO7	Importin-	23	20	38	19	1038	119.4
A0A0A0MTS	GPI	Glucose-6	36	19	50	19	573	64.8
P09211	GSTP1	Glutathic	55	10	75	10	210	23.3
P23246	SFPQ	Splicing	31	22	49	21	707	76.1
P61978	HNRNPK	Heterogen	47	19	56	3	463	50.9
P55084	HADHB	Trifuncti	33	18	39	18	474	51.3
Q8WX93	PALLD	Palladin	19	22	46	22	1383	150.5
Q9H223	EHD4	EH domain	49	22	41	15	541	61.1
Q9NR30	DDX21	Nucleolar	32	22	33	20	783	87.3
P30041	PRDX6	Peroxired	48	14	63	14	224	25
H0YNC7	TPM1	Tropomyos	65	20	93	1	223	25.6
Q16891	IMMT	MICOS com	38	27	41	27	758	83.6
O00231	PSMD11	26S prote	57	22	45	22	422	47.4
P22626	HNRNPA2B1	Heterogen	50	17	61	15	353	37.4
P20908	COL5A1	Collagen	17	26	52	20	1838	183.4
P07954	FH	Fumarate	41	17	42	17	510	54.6
Q13423	NNT	NAD(P) tr	26	24	35	24	1086	113.8

Q12797	ASPH	Aspartyl/	30	22	44	22	758	85.8
Q15029	EFTUD2	116 kDa U	29	24	39	23	972	109.4
P28331	NDUFS1	NADH-ubiq	41	21	31	21	727	79.4
P47897	QARS	Glutamine	37	24	38	23	775	87.7
P52907	CAPZA1	F-actin-c	65	12	36	10	286	32.9
M0R0P8	MYO9B	Unconvent	16	35	39	35	2157	243.2
P50995	ANXA11	Annexin A	37	18	43	18	505	54.4
A8MXP9	MATR3	Matrin-3	25	24	48	24	895	99.9
A0A0A0MTC	GSN	Gelsolin	35	21	47	16	767	84.7
Q13308	PTK7	Inactive	28	25	40	25	1070	118.3
P51659	HSD17B4	Peroxisom	40	24	34	24	736	79.6
O14980	XPO1	Exportin-	22	21	39	21	1071	123.3
P00367	GLUD1	Glutamate	34	18	38	18	558	61.4
B4DDF4	CNN2	Calponin	54	12	60	10	298	32.6
P08243	ASNS	Asparagin	42	20	41	20	561	64.3
O43776	NARS	Asparagin	32	18	44	18	548	62.9
Q9UHD8	SEPT9	Septin-9	47	23	41	23	586	65.4
Q32Q12	NME1-NME2	Nucleosid	74	17	60	2	292	32.6
P29144	TPP2	Tripeptid	22	27	36	27	1249	138.3
P07737	PFN1	Profilin-	72	11	137	11	140	15
P62937	PPIA	Peptidyl-	68	13	119	12	165	18
F8W6I7	HNRNPA1	Heterogen	50	15	67	13	307	33.1
Q14980	NUMA1	Nuclear m	15	30	34	29	2115	238.1
P37837	TALDO1	Transaldo	44	19	52	19	337	37.5
O14617	AP3D1	AP-3 comp	22	23	30	23	1153	130.1
Q92538	GBF1	Golgi-spe	16	25	35	25	1859	206.3
O94808	GFPT2	Glutamine	38	25	47	22	682	76.9
K7EKE6	LONP1	Lon prote	28	21	29	21	845	95.1
G8JLD5	DNM1L	Dynamin-1	36	22	30	22	712	79.6
P54578	USP14	Ubiquitin	33	18	29	18	494	56
F8W914	RTN4	Reticulon	54	9	35	9	345	37.1
P14868	DARS	Aspartate	53	25	39	25	501	57.1
O94855	SEC24D	Protein t	29	24	37	23	1032	112.9
O00299	CLIC1	Chloride	74	14	48	13	241	26.9
P23526	AHCY	Adenosylh	46	19	44	19	432	47.7
Q9UQ80	PA2G4	Prolifera	44	15	44	15	394	43.8
P12109	COL6A1	Collagen	23	21	39	21	1028	108.5
Q9NZN4	EHD2	EH domain	51	23	40	18	543	61.1
Q9NTK5	OLA1	Obg-like	46	17	38	17	396	44.7
P21589	NT5E	5’-nucleo	34	17	45	17	574	63.3
P24844	MYL9	Myosin re	66	10	63	3	172	19.8
Q5JPE7	NOMO2	Nodal mod	20	20	35	20	1267	139.4
O14974	PPP1R12A	Protein p	24	26	37	26	1030	115.2
B5ME19	EIF3CL	Eukaryoti	26	26	45	26	914	105.4
Q5T6W2	HNRNPK	Heterogen	50	17	55	1	379	41.8
P42224	STAT1	Signal tr	27	19	35	19	750	87.3
Q13045	FLII	Protein f	23	26	34	15	1269	144.7
Q6DD88	ATL3	Atlastin-	41	16	40	16	541	60.5
P14866	HNRNPL	Heterogen	44	19	37	18	589	64.1
Q92945	KHSRP	Far upstr	43	25	38	23	711	73.1
Q9Y3F4	STRAP	Serine-th	53	16	41	16	350	38.4

Q6WCQ1	MPRIP	Myosin ph	26	23	32	22	1025	116.5
H0Y8G5	HNRNPD	Heterogen	48	17	46	10	260	29.6
Q9Y230	RUVBL2	RuvB-like	41	18	32	18	463	51.1
Q9Y265	RUVBL1	RuvB-like	46	17	28	17	456	50.2
P09486	SPARC	SPARC OS=	46	14	48	14	303	34.6
E9PCY7	HNRNPH1	Heterogen	41	15	45	4	429	47.1
A0A2R8Y4T	TNS1	Tensin-1	17	26	35	26	1860	199.4
Q13435	SF3B2	Splicing	30	27	41	27	895	100.2
P08240	SRPRA	Signal re	32	20	33	20	638	69.8
P35555	FBN1	Fibrillin	11	28	33	24	2871	312
Q16181	SEPT7	Septin-7	44	19	45	18	437	50.6
A0A1B0GTC	ALDH7A1	Alpha-ami	41	17	29	17	536	58.1
Q14240	EIF4A2	Eukaryoti	40	16	54	5	407	46.4
P21796	VDAC1	Voltage-d	54	13	44	12	283	30.8
E9PND2	CSRP1	Cysteine	63	9	49	1	153	16.1
P00352	ALDH1A1	Retinal d	33	17	41	16	501	54.8
Q96D15	RCN3	Reticuloc	48	12	38	12	328	37.5
P09936	UCHL1	Ubiquitin	53	10	81	10	223	24.8
P30837	ALDH1B1	Aldehyde	34	16	32	14	517	57.2
A0AVT1	UBA6	Ubiquitin	24	23	32	23	1052	117.9
P07996	THBS1	Thrombosp	24	27	34	27	1170	129.3
Q02790	FKBP4	Peptidyl-	46	20	34	20	459	51.8
Q96TA1	FAM129B	Niban-lik	25	21	48	21	746	84.1
P60981	DSTN	Destrin O	66	15	50	14	165	18.5
O43491	EPB41L2	Band 4.1-	24	21	34	21	1005	112.5
P17812	CTPS1	CTP synth	31	18	38	15	591	66.6
Q96AG4	LRRC59	Leucine-r	47	15	41	15	307	34.9
Q15181	PPA1	Inorganic	67	16	40	15	289	32.6
C9JZR2	CTNND1	Catenin d	31	23	40	23	938	104.8
P58107	EPPK1	Epiplakin	23	26	34	20	5088	555.3
P23381	WARS	Tryptopha	30	15	35	15	471	53.1
Q15008	PSMD6	26S prote	45	21	37	21	389	45.5
P45974	USP5	Ubiquitin	30	19	36	19	858	95.7
Q9UGI8	TES	Testin OS	49	18	29	18	421	48
D6RER5	SEPT11	Septin-11	44	15	32	8	432	49.8
O43242	PSMD3	26S prote	39	20	32	20	534	60.9
P63241	EIF5A	Eukaryoti	71	11	49	11	154	16.8
A0A2R8YCH	CTNNB1	Catenin b	31	20	34	17	779	85.2
P15880	RPS2	40S ribos	43	13	70	13	293	31.3
E9PBS1	PAICS	Multifunc	44	19	37	19	413	45.6
A2A274	ACO2	Aconitate	27	19	37	19	805	87.8
P31946	YWHAB	14-3-3 pr	52	12	65	5	246	28.1
Q15293	RCN1	Reticuloc	56	15	35	15	331	38.9
Q13561	DCTN2	Dynactin	49	15	34	15	401	44.2
P50552	VASP	Vasodilat	47	17	31	17	380	39.8
P04181	OAT	Ornithine	34	14	29	14	439	48.5
P61247	RPS3A	40S ribos	61	20	63	20	264	29.9
O00203	AP3B1	AP-3 comp	23	25	36	24	1094	121.2
Q16543	CDC37	Hsp90 co-	35	12	39	12	378	44.4
A0A087WVM	MTHFD1L	Monofunct	24	20	30	19	913	99.2
Q32MZ4	LRRFIP1	Leucine-r	22	16	25	15	808	89.2

P61221	ABCE1	ATP-bindi	33	18	33	15	599	67.3
Q9Y570	PPME1	Protein p	50	17	31	17	386	42.3
Q99497	PARK7	Protein/n	59	12	50	12	189	19.9
Q9Y520	PRRC2C	Protein P	10	27	31	26	2896	316.7
P05997	COL5A2	Collagen	18	20	32	20	1499	144.8
P20042	EIF2S2	Eukaryoti	50	15	31	15	333	38.4
P49257	LMAN1	Protein E	33	17	52	17	510	57.5
Q9NZI8	IGF2BP1	Insulin-l	39	22	34	20	577	63.4
P09382	LGALS1	Galectin-	78	9	175	9	135	14.7
Q15046	KARS	Lysine--t	33	20	45	20	597	68
Q9Y617	PSAT1	Phosphose	51	19	33	18	370	40.4
P52306	RAP1GDS1	Rap1 GTPa	33	17	28	17	607	66.3
Q92973	TNPO1	Transport	22	17	33	13	898	102.3
I3L1L3	MYBBP1A	Myb-bindi	21	24	31	24	1252	140.2
P50570	DNM2	Dynamin-2	26	22	31	15	870	98
Q15393	SF3B3	Splicing	19	21	39	21	1217	135.5
Q9UJU6	DBNL	Drebrin-l	42	14	30	14	430	48.2
O14950	MYL12B	Myosin re	55	10	67	3	172	19.8
Q15366	PCBP2	Poly(rC)-	41	12	40	5	365	38.6
P05388	RPLP0	60S acidi	48	13	51	13	317	34.3
P20020	ATP2B1	Plasma me	19	18	23	10	1258	138.7
A0A2Q2TH7	GOLGA2	Golgin su	21	18	23	13	990	111.6
P00505	GOT2	Aspartate	38	18	31	18	430	47.5
P18124	RPL7	60S ribos	53	16	48	16	248	29.2
Q02218	OGDH	2-oxoglut	23	22	28	17	1023	115.9
H3BQZ7	HNRNPUL2-	HCG20447	27	20	32	20	746	84.6
Q96KP4	CNDP2	Cytosolic	41	16	35	16	475	52.8
P30520	ADSS	Adenylosu	36	14	25	14	456	50.1
H0Y5F5	PABPC4	Polyadeny	28	18	38	1	550	60.2
Q8N163	CCAR2	Cell cycl	23	17	29	17	923	102.8
P51991	HNRNPA3	Heterogen	25	11	35	10	378	39.6
G3V180	DPP3	Dipeptidy	32	18	30	18	757	84.3
B1AK88	CAPZB	Capping p	41	16	56	15	301	33.8
P30086	PEBP1	Phosphati	80	11	47	11	187	21
P29966	MARCKS	Myristoyl	34	7	45	7	332	31.5
A0A087X2I	PSMC6	26S prote	50	16	28	16	403	45.8
P51149	RAB7A	Ras-relat	72	13	37	13	207	23.5
P62701	RPS4X	40S ribos	55	16	42	11	263	29.6
O95340	PAPSS2	Bifunctio	31	17	43	16	614	69.5
Q8WVM8	SCFD1	Sec1 fami	30	17	24	17	642	72.3
Q5JXI8	FHL1	Four and	60	12	36	4	257	29.1
Q562R1	ACTBL2	Beta-acti	34	13	209	7	376	42
P48147	PREP	Prolyl en	37	22	30	22	710	80.6
O76094	SRP72	Signal re	35	20	28	20	671	74.6
A0A0J9YVF	PUF60	Poly(U)-b	36	16	34	16	534	57.4
P13804	ETFA	Electron	43	11	31	11	333	35.1
P62191	PSMC1	26S prote	50	21	46	18	440	49.2
Q13085	ACACA	Acetyl-Co	12	25	28	25	2346	265.4
A0A2U3TZM	CHD4	Chromodom	15	24	26	24	1902	216.7
P54920	NAPA	Alpha-sol	62	15	26	15	295	33.2
P08865	RPSA	40S ribos	41	12	39	11	295	32.8

Q9BXJ9	NAA15	N-alpha-a	29	24	34	19	866	101.2
Q15417	CNN3	Calponin-	45	12	37	10	329	36.4
P00387	CYB5R3	NADH-cytc	49	12	37	12	301	34.2
P53992	SEC24C	Protein t	26	23	29	21	1094	118.2
P10316	HLA-A	HLA class	47	13	28	5	365	41
P61011	SRP54	Signal re	41	19	32	19	504	55.7
P78417	GSTO1	Glutathic	44	16	51	16	241	27.5
Q06323	PSME1	Proteasom	58	15	36	15	249	28.7
P80303	NUCB2	Nucleobir	42	16	29	14	420	50.2
Q9NZB2	FAM120A	Constitut	22	19	28	19	1118	121.8
Q5T7C4	HMGB1	High mobi	54	11	51	8	158	18.3
O75718	CRTAP	Cartilage	39	16	31	16	401	46.5
P05141	SLC25A5	ADP/ATP t	44	15	48	5	298	32.8
Q9NSD9	FARSB	Phenylala	34	21	34	21	589	66.1
Q7Z406	MYH14	Myosin-14	5	12	60	1	1995	227.7
P48735	IDH2	Isocitrat	41	19	31	19	452	50.9
Q13740	ALCAM	CD166 ant	29	16	28	16	583	65.1
O00232	PSMD12	26S prote	36	16	30	16	456	52.9
P08237	PFKM	ATP-deper	30	20	37	17	780	85.1
P55795	HNRNPH2	Heteroger	39	15	38	5	449	49.2
P25786	PSMA1	Proteasom	51	14	34	14	263	29.5
Q08J23	NSUN2	tRNA (cyt	37	22	29	22	767	86.4
Q96QK1	VPS35	Vacuolar	27	18	26	16	796	91.6
Q9NR12	PDLIM7	PDZ and L	40	16	54	12	457	49.8
P40925	MDH1	Malate de	40	15	46	15	334	36.4
Q13492	PICALM	Phosphati	28	16	27	13	652	70.7
P49189	ALDH9A1	4-trimeth	30	14	25	14	494	53.8
P32119	PRDX2	Peroxirec	58	11	42	10	198	21.9
Q9Y2A7	NCKAP1	Nck-assoc	20	23	31	23	1128	128.7
H3BVG0	NUP93	Nuclear p	27	23	30	23	880	99.5
P12110	COL6A2	Collagen	22	19	30	19	1019	108.5
Q9NYL9	TMOD3	Tropomodu	51	17	31	16	352	39.6
P55010	EIF5	Eukaryoti	36	17	30	17	431	49.2
P41091	EIF2S3	Eukaryoti	34	13	33	13	472	51.1
Q9UBG0	MRC2	C-type ma	15	17	29	17	1479	166.6
P21399	ACO1	Cytoplasm	28	23	33	23	889	98.3
Q9Y266	NUDC	Nuclear m	47	15	30	15	331	38.2
Q9H4A4	RNPEP	Aminopept	28	16	27	16	650	72.5
O95573	ACSL3	Long-chai	29	16	23	15	720	80.4
Q9Y263	PLAA	Phospholi	28	20	25	20	795	87.1
P15924	DSP	Desmoplak	9	29	29	29	2871	331.6
O15371	EIF3D	Eukaryoti	32	12	24	12	548	63.9
Q8NE71	ABCF1	ATP-bindi	18	15	22	10	845	95.9
P62906	RPL10A	60S ribos	42	11	40	11	217	24.8
Q16270	IGFBP7	Insulin-l	41	11	36	11	282	29.1
P61160	ACTR2	Actin-rel	33	11	48	11	394	44.7
P62753	RPS6	40S ribos	39	13	38	13	249	28.7
P78347	GTF2I	General t	19	21	26	21	998	112.3
P10412	HIST1H1E	Histone H	42	14	57	4	219	21.9
Q02818	NUCB1	Nucleobir	42	19	32	17	461	53.8
P04844	RPN2	Dolichyl-	33	14	31	14	631	69.2

Q14847	LASP1	LIM and S	52	16	47	16	261	29.7
P16403	HIST1H1C	Histone H	40	13	54	4	213	21.4
P04899	GNAI2	Guanine n	46	13	28	6	355	40.4
P05091	ALDH2	Aldehyde	41	14	22	13	517	56.3
P40227	CCT6A	T-complex	33	17	40	17	531	58
O15144	ARPC2	Actin-rel	48	17	43	17	300	34.3
P14923	JUP	Junction	29	17	22	14	745	81.7
Q96A49	SYAP1	Synapse-a	47	15	21	15	352	39.9
P17858	PFKL	ATP-depen	24	17	40	12	780	85
P39019	RPS19	40S ribos	62	13	58	13	145	16.1
Q53EP0	FNDC3B	Fibronect	20	17	25	17	1204	132.8
P28074	PSMB5	Proteasom	54	13	23	13	263	28.5
P24534	EEF1B2	Elongatic	56	11	44	8	225	24.7
O15031	PLXNB2	Plexin-B2	14	25	28	25	1838	205
F8W9J4	DST	Dystonin	4	27	28	23	7461	847.4
P55265	ADAR	Double-st	19	22	26	22	1226	136
A0A0C4DG8	DDX46	Probable	23	25	30	25	1032	117.4
P98160	HSPG2	Basement	6	22	25	22	4391	468.5
P13647	KRT5	Keratin,	25	18	49	7	590	62.3
P11766	ADH5	Alcohol d	33	13	41	13	374	39.7
P09972	ALDOC	Fructose-	35	13	49	8	364	39.4
A0A1W2PNX	UNC45A	Protein u	20	21	25	20	1084	118.3
A0A0C4DGA	ECI2	Enoyl-CoA	39	11	21	11	364	40.2
Q07960	ARHGAP1	Rho GTPas	38	15	36	15	439	50.4
Q96CW1	AP2M1	AP-2 comp	47	19	34	19	435	49.6
Q8IWE2	FAM114A1	Protein N	30	15	25	15	563	60.7
J3KTL2	SRSF1	Serine/ar	52	15	47	15	253	28.3
O95817	BAG3	BAG famil	42	17	22	17	575	61.6
O75821	EIF3G	Eukaryoti	41	13	26	2	320	35.6
H0Y6I0	GOLGA4	Golgin su	11	25	27	24	2099	246.5
P50502	ST13	Hsc70-int	29	12	29	12	369	41.3
E7EQL5	DYNC1I2	Cytoplasm	47	11	22	1	305	34.9
P49915	GMPS	GMP synth	32	20	28	20	693	76.7
Q9BT78	COPS4	COP9 sign	55	17	26	17	406	46.2
Q13620	CUL4B	Cullin-4E	23	23	33	13	913	103.9
Q32P28	P3H1	Prolyl 3-	26	16	33	15	736	83.3
Q13976	PRKG1	cGMP-depe	31	21	28	21	671	76.3
Q96I24	FUBP3	Far upstr	41	19	27	17	572	61.6
Q10471	GALNT2	Polypepti	43	24	33	22	571	64.7
Q8TAQ2	SMARCC2	SWI/SNF c	16	18	22	12	1214	132.8
Q04917	YWHAH	14-3-3 pr	52	13	55	9	246	28.2
P60900	PSMA6	Proteasom	48	12	31	12	246	27.4
Q9Y2X3	NOP58	Nucleolar	34	15	21	15	529	59.5
Q13162	PRDX4	Peroxirec	49	11	40	9	271	30.5
Q8NBJ5	COLGALT1	Procollag	30	18	27	18	622	71.6
P49321	NASP	Nuclear a	26	17	21	17	788	85.2
A0A0A0MTH	ILK	Integrin-	36	17	36	17	483	54.6
H3BNC9		Uncharact	13	7	23	7	584	64.5
O75533	SF3B1	Splicing	20	21	23	21	1304	145.7
Q70UQ0	IKBIP	Inhibitor	43	17	38	17	350	39.3
P43034	PAFAH1B1	Platelet-	39	16	26	15	410	46.6

Q9HD20	ATP13A1	Manganese	18	18	21	18	1204	132.9
Q12965	MYO1E	Unconvent	16	17	22	15	1108	127
Q9H0U4	RAB1B	Ras-relat	69	13	40	5	201	22.2
P10155	TROVE2	60 kDa SS	33	17	25	17	538	60.6
Q13177	PAK2	Serine/th	29	12	20	10	524	58
P27695	APEX1	DNA-(apur	52	16	25	16	318	35.5
P15531	NME1	Nucleosid	70	11	37	1	152	17.1
P12236	SLC25A6	ADP/ATP t	41	14	46	2	298	32.8
P30533	LRPAP1	Alpha-2-m	47	20	33	20	357	41.4
P62805	HIST1H4A	Histone H	58	7	99	7	103	11.4
Q9Y6M1	IGF2BP2	Insulin-l	31	16	26	15	599	66.1
Q27J81	INF2	Inverted	15	17	21	17	1249	135.5
Q9UDY4	DNAJB4	DnaJ homo	39	12	24	11	337	37.8
P62979	RPS27A	Ubiquitin	67	12	127	4	156	18
P48059	LIMS1	LIM and s	50	14	32	8	325	37.2
Q99536	VAT1	Synaptic	32	10	34	10	393	41.9
P60228	EIF3E	Eukaryoti	39	15	27	15	445	52.2
Q15459	SF3A1	Splicing	24	16	29	16	793	88.8
H0Y7A7	CALM2	Calmoduli	39	7	86	7	187	20.7
O75116	ROCK2	Rho-assoc	16	22	25	20	1388	160.8
A0A0U1RRM	ENAH	Protein e	18	16	30	16	802	87.3
X6RLX0	ERC1	ELKS/Rab6	17	19	23	19	1120	128.4
P62820	RAB1A	Ras-relat	75	14	41	6	205	22.7
P62241	RPS8	40S ribos	52	11	37	11	208	24.2
Q9Y281	CFL2	Cofilin-2	66	14	40	9	166	18.7
Q9Y5M8	SRPRB	Signal re	48	11	20	11	271	29.7
Q92696	RABGGTA	Geranylge	34	18	24	18	567	65
E9PFF5	FXR1	Fragile X	35	14	20	12	490	55.1
Q8IVL6	P3H3	Prolyl 3-	30	18	29	18	736	81.8
Q9Y6N5	SQOR	Sulfide:q	46	18	25	18	450	49.9
Q9HB71	CACYBP	Calcyclin	57	12	21	12	228	26.2
H0Y449	YBX1	Nuclease-	32	8	36	6	374	42
P62873	GNB1	Guanine n	36	11	30	5	340	37.4
P46063	RECQL	ATP-depen	31	19	30	19	649	73.4
O95394	PGM3	Phosphoac	33	15	28	15	542	59.8
P05387	RPLP2	60S acidi	54	6	29	6	115	11.7
A0A0R4J2G	NCEH1	Arylaceta	40	16	29	16	440	49
Q8WUP2	FBLIM1	Filamin-b	44	13	26	13	373	40.6
P28066	PSMA5	Proteasom	44	8	27	8	241	26.4
P43686	PSMC4	26S prote	34	14	28	14	418	47.3
E7EQT4	ACIN1	Apoptotic	15	18	23	18	1301	147.3
Q92888	ARHGEF1	Rho guani	25	19	24	19	912	102.4
P16402	HIST1H1D	Histone H	42	13	52	5	221	22.3
Q9UNH7	SNX6	Sorting n	39	17	21	16	406	46.6
Q15942	ZYX	Zyxin OS=	28	13	38	13	572	61.2
O95747	OXSR1	Serine/th	35	16	24	12	527	58
P12235	SLC25A4	ADP/ATP t	45	14	37	4	298	33
P09960	LTA4H	Leukotrie	26	14	20	14	611	69.2
P63000	RAC1	Ras-relat	41	9	36	3	192	21.4
P25788	PSMA3	Proteasom	44	14	35	14	255	28.4
P51148	RAB5C	Ras-relat	54	8	29	6	216	23.5

Q02878	RPL6	60S ribos	36	15	54	15	288	32.7
Q7L1Q6	BZW1	Basic leu	32	17	43	17	419	48
P54727	RAD23B	UV excisi	34	14	31	12	409	43.1
Q92626	PXDN	Peroxidas	14	20	26	20	1479	165.2
K7EL20	EIF3G	Eukaryoti	44	12	24	1	262	29.3
P45880	VDAC2	Voltage-d	44	12	39	12	294	31.5
P25398	RPS12	40S ribos	55	8	32	8	132	14.5
Q9BS26	ERP44	Endoplasm	37	15	27	14	406	46.9
P20073	ANXA7	Annexin A	32	14	25	14	488	52.7
O94973	AP2A2	AP-2 comp	22	20	25	11	939	103.9
P30050	RPL12	60S ribos	69	9	44	9	165	17.8
P09525	ANXA4	Annexin A	48	14	22	14	319	35.9
P05121	SERPINE1	Plasminog	37	12	25	12	402	45
E7EX17	EIF4B	Eukaryoti	28	18	25	18	616	69.7
Q16698	DECR1	2,4-dienc	42	14	22	14	335	36
P24752	ACAT1	Acetyl-Cc	35	14	21	14	427	45.2
Q9NZU5	LMCD1	LIM and c	47	16	27	16	365	40.8
Q15042	RAB3GAP1	Rab3 GTPa	20	16	22	16	981	110.5
P02538	KRT6A	Keratin,	21	15	57	1	564	60
P22307	SCP2	Non-speci	24	14	26	14	547	59
Q96G03	PGM2	Phosphogl	29	17	23	16	612	68.2
Q9BXP5	SRRT	Serrate R	23	18	22	18	876	100.6
Q8N3C0	ASCC3	Activatin	11	21	22	21	2202	251.3
Q13464	ROCK1	Rho-assoc	15	21	24	19	1354	158.1
Q5T5C7	SARS	Serine--t	34	15	28	15	536	61.3
A0A2R8Y81	RPS14	40S ribos	41	7	23	7	150	16.1
P52789	HK2	Hexokinas	22	19	20	16	917	102.3
Q96C19	EFHD2	EF-hand d	47	12	20	12	240	26.7
P19623	SRM	Spermidin	53	10	22	10	302	33.8
Q9Y4E8	USP15	Ubiquitin	22	20	26	19	981	112.3
Q9UNF0	PACSIN2	Protein k	36	14	25	14	486	55.7
P62714	PPP2CB	Serine/th	39	10	23	2	309	35.6
P48739	PITPNB	Phosphati	65	17	21	14	271	31.5
P52209	PGD	6-phosphc	33	15	40	15	483	53.1
P34897	SHMT2	Serine hy	30	14	30	14	504	56
Q9UNM6	PSMD13	26S prote	33	13	30	13	376	42.9
A0A087X0K	TJP1	Tight jun	11	17	23	17	1676	187.7
Q9Y450	HBS1L	HBS1-like	28	16	25	16	684	75.4
P11177	PDHB	Pyruvate	31	9	24	9	359	39.2
O75131	CPNE3	Copine-3	30	15	25	14	537	60.1
P62136	PPP1CA	Serine/th	44	13	32	3	330	37.5
Q8TDX7	NEK7	Serine/th	43	14	30	14	302	34.5
P21964	COMT	Catechol	53	14	22	14	271	30
P30044	PRDX5	Peroxirec	55	8	32	8	214	22.1
Q15691	MAPRE1	Microtubu	42	11	32	9	268	30
Q9Y2Z0	SUGT1	Protein S	42	13	21	13	365	41
I3L0H8	DDX19A	ATP-depen	31	15	29	15	447	50.5
Q9Y6Y8	SEC23IP	SEC23-int	19	18	23	18	1000	111
B7Z7P8	ETF1	Eukaryoti	28	11	30	11	423	47.4
Q15424	SAFB	Scaffold	17	14	22	9	915	102.6
H9KV28	DIAPH1	Protein d	14	17	21	17	1228	136.8

O43143	DHX15	Pre-mRNA-	22	18	32	17	795	90.9
P62140	PPP1CB	Serine/th	40	12	30	3	327	37.2
P51572	BCAP31	B-cell re	37	13	28	13	246	28
Q14BN4	SLMAP	Sarcolemm	18	16	21	16	828	95.1
P83731	RPL24	60S ribos	52	13	41	13	157	17.8
P18085	ARF4	ADP-ribos	53	8	33	6	180	20.5
Q9UBS4	DNAJB11	DnaJ homo	39	14	26	14	358	40.5
A0A0U1RQK	EIF4G3	Eukaryoti	10	16	21	12	1774	195.2
P35232	PHB	Prohibiti	47	13	30	13	272	29.8
Q13409	DYNC1I2	Cytoplasm	20	11	21	1	638	71.4
Q9H3P7	ACBD3	Golgi res	31	12	16	12	528	60.6
O43399	TPD52L2	Tumor pro	54	12	34	12	206	22.2
P08779	KRT16	Keratin,	39	18	27	6	473	51.2
P52565	ARHGDIA	Rho GDP-d	37	9	36	9	204	23.2
Q01469	FABP5	Fatty aci	66	8	25	8	135	15.2
P27487	DPP4	Dipeptidy	21	17	26	17	766	88.2
H0Y2W2	ATAD3A	ATPase fa	32	19	25	7	572	64.3
O75165	DNAJC13	DnaJ homo	11	22	23	22	2243	254.3
Q12931	TRAP1	Heat shoc	27	18	26	17	704	80.1
B4DY09	ILF2	cDNA FLJ5	35	11	24	11	352	38.9
O60488	ACSL4	Long-chai	23	15	21	14	711	79.1
P13861	PRKAR2A	cAMP-depe	42	14	20	14	404	45.5
Q5QNW6	HIST2H2BF	Histone H	59	10	94	4	126	13.9
P62750	RPL23A	60S ribos	42	11	43	11	156	17.7
Q9H2M9	RAB3GAP2	Rab3 GTPa	15	20	24	20	1393	155.9
D6RBZ0	HNRNPAB	Heterogen	37	13	30	11	327	35.7
Q9H0D6	XRN2	5'-3' exo	20	17	21	17	950	108.5
P02533	KRT14	Keratin,	33	18	26	4	472	51.5
Q6PGP7	TTC37	Tetratric	14	19	22	19	1564	175.4
P55735	SEC13	Protein S	38	9	30	9	322	35.5
O00425	IGF2BP3	Insulin-l	28	15	25	13	579	63.7
P55145	MANF	Mesenceph	38	11	27	11	182	20.7
Q96AE4	FUBP1	Far upstr	27	17	29	14	644	67.5
P38606	ATP6V1A	V-type pr	29	15	23	15	617	68.3
P16401	HIST1H1B	Histone H	35	12	45	8	226	22.6
P27635	RPL10	60S ribos	39	7	37	1	214	24.6
Q14444	CAPRIN1	Caprin-1	15	12	25	12	709	78.3
P22059	OSBP	Oxysterol	24	17	21	17	807	89.4
O14744	PRMT5	Protein a	32	17	21	17	637	72.6
O14818	PSMA7	Proteasom	50	12	24	12	248	27.9
O95302	FKBP9	Peptidyl-	29	16	32	15	570	63
A0A1B0GVD	CTSD	Cathepsin	33	11	32	11	409	44.2
Q00325	SLC25A3	Phosphate	30	12	42	12	362	40.1
P38919	EIF4A3	Eukaryoti	39	14	31	11	411	46.8
P61586	RHOA	Transform	52	9	29	4	193	21.8
Q13724	MOGS	Mannosyl-	23	15	24	15	837	91.9
C9JIZ6	PSAP	Prosaposi	26	15	27	15	527	58.4
F6WLT2	DDX39B	Spliceoso	41	12	28	5	289	32.9
P08729	KRT7	Keratin,	32	16	47	12	469	51.4
Q9UNZ2	NSFL1C	NSFL1 cof	41	14	22	14	370	40.5
O76021	RSL1D1	Ribosomal	30	15	26	15	490	54.9

Q9Y5S2	CDC42BPB	Serine/th	12	21	22	17	1711	194.2
B7Z6Z4	MYL6	cDNA FLJ5	32	7	64	7	238	26.7
P30040	ERP29	Endoplasm	41	12	38	12	261	29
P22061	PCMT1	Protein-L	52	9	18	9	227	24.6
O60568	PLOD3	Procollag	22	16	30	16	738	84.7
Q14257	RCN2	Reticuloc	39	11	16	11	317	36.9
P67775	PPP2CA	Serine/th	39	10	23	2	309	35.6
P62879	GNB2	Guanine n	36	11	25	3	340	37.3
P17661	DES	Desmin OS	27	15	69	10	470	53.5
P36873	PPP1CC	Serine/th	42	12	28	2	323	37
P00492	HPRT1	Hypoxanth	65	10	17	10	218	24.6
Q12904	AIMP1	Aminoacyl	42	11	18	11	312	34.3
Q13596	SNX1	Sorting n	24	11	18	9	522	59
Q92878	RAD50	DNA repai	13	19	21	19	1312	153.8
P31930	UQCRC1	Cytochrom	35	12	21	11	480	52.6
Q9NQW7	XPNPEP1	Xaa-Pro a	23	13	24	13	623	69.9
Q9BZQ8	FAM129A	Protein N	20	16	22	16	928	103.1
Q9Y5B9	SUPT16H	FACT comp	18	17	20	17	1047	119.8
Q92734	TFG	Protein T	31	12	21	12	400	43.4
V9GYM8	ARHGEF2	Rho guani	18	18	22	18	1031	116
Q02880	TOP2B	DNA topoi	12	19	20	13	1626	183.2
O95861	BPNT1	3'(2'),5'	39	13	19	13	308	33.4
H0YDU8	PPP5C	Serine/th	31	13	19	13	485	55.2
P20340	RAB6A	Ras-relat	55	11	26	9	208	23.6
H3BRG4	UQCRC2	Cytochrom	36	12	22	12	412	44.6
Q93052	LPP	Lipoma-pr	26	11	22	11	612	65.7
A6NNK5	TP53BP1	TP53-bind	11	16	17	16	1927	208.9
Q13185	CBX3	Chromobox	55	9	19	8	183	20.8
Q8TCS8	PNPT1	Polyribon	24	18	21	18	783	85.9
Q9Y224	RTRAF	RNA trans	47	12	25	12	244	28.1
Q8N257	HIST3H2BE	Histone H	53	9	72	3	126	13.9
J3KR44	OTUB1	Ubiquitin	50	10	22	10	272	31.4
P55036	PSMD4	26S prote	29	10	20	10	377	40.7
O94826	TOMM70	Mitochond	30	19	28	19	608	67.4
O14964	HGS	Hepatocyt	23	17	27	17	777	86.1
Q9Y2W1	THRAP3	Thyroid h	16	14	22	13	955	108.6
O94905	ERLIN2	Erlin-2 C	40	12	21	9	339	37.8
Q9NZN3	EHD3	EH domain	33	16	30	7	535	60.8
P62917	RPL8	60S ribos	50	12	33	12	257	28
P10644	PRKAR1A	cAMP-depe	41	16	24	13	381	43
O43252	PAPSS1	Bifunctic	23	11	22	10	624	70.8
P00390	GSR	Glutathic	32	14	22	14	522	56.2
Q9H3S7	PTPN23	Tyrosine-	12	16	17	16	1636	178.9
Q7L576	CYFIP1	Cytoplasm	15	16	22	8	1253	145.1
P29317	EPHA2	Ephrin ty	17	15	18	14	976	108.2
P56537	EIF6	Eukaryoti	51	8	17	8	245	26.6
O00154	ACOT7	Cytosolic	32	11	22	11	380	41.8
P21266	GSTM3	Glutathic	51	11	22	9	225	26.5
O60313	OPA1	Dynamin-l	19	19	20	19	960	111.6
Q5JRA6	MIA3	Transport	10	19	22	19	1907	213.6
P46783	RPS10	40S ribos	58	12	42	11	165	18.9

P51911	CNN1	Calponin-	45	11	21	10	297	33.2
K7EIG1	CLUH	Clustered	17	19	21	2	1251	140.5
P38159	RBMX	RNA-bindi	41	18	38	18	391	42.3
Q99623	PHB2	Prohibiti	56	17	28	17	299	33.3
P43487	RANBP1	Ran-speci	51	12	25	12	201	23.3
Q9NQR4	NIT2	Omega-ami	49	13	17	13	276	30.6
P61224	RAP1B	Ras-relat	57	9	23	4	184	20.8
P23368	ME2	NAD-depen	28	15	22	15	584	65.4
Q15746	MYLK	Myosin li	10	15	23	15	1914	210.6
P62829	RPL23	60S ribos	61	8	37	8	140	14.9
Q6IBS0	TWF2	Twinfilin	52	14	29	11	349	39.5
Q9UBB4	ATXN10	Ataxin-10	33	15	24	15	475	53.5
P26368	U2AF2	Splicing	31	9	25	9	475	53.5
O14979	HNRNPDL	Heterogen	30	14	28	12	420	46.4
Q9UHG3	PCYOX1	Prenylcys	30	12	23	12	505	56.6
P38117	ETFB	Electron	53	14	19	14	255	27.8
P40222	TXLNA	Alpha-tax	32	15	19	15	546	61.9
O00567	NOP56	Nucleolar	23	12	19	12	594	66
P29692	EEF1D	Elongatio	39	11	41	8	281	31.1
A6NLN1	PTBP1	Polypyrim	23	10	24	10	527	56.5
P00491	PNP	Purine nu	44	10	19	10	289	32.1
Q5W0H4	TPT1	Translati	38	8	37	8	188	21.5
O75368	SH3BGRL	SH3 domai	90	9	22	9	114	12.8
Q6NZI2	CAVIN1	Caveolae-	28	13	39	13	390	43.5
Q16401	PSMD5	26S prote	37	15	24	15	504	56.2
O95336	PGLS	6-phospho	55	10	23	10	258	27.5
Q8NCA5	FAM98A	Protein F	24	9	21	9	519	55.4
P51858	HDGF	Hepatoma-	50	10	21	9	240	26.8
Q96CV9	OPTN	Optineuri	29	17	20	17	577	65.9
Q13283	G3BP1	Ras GTPas	36	13	26	12	466	52.1
Q16822	PCK2	Phosphoen	26	15	21	15	640	70.7
P16152	CBR1	Carbonyl	42	9	25	7	277	30.4
Q9UI42	CPA4	Carboxype	28	11	27	11	421	47.3
Q7L2H7	EIF3M	Eukaryoti	29	9	19	9	374	42.5
O94874	UFL1	E3 UFM1-p	21	16	22	16	794	89.5
P46977	STT3A	Dolichyl-	20	15	30	13	705	80.5
P05198	EIF2S1	Eukaryoti	45	14	23	14	315	36.1
P35637	FUS	RNA-bindi	16	9	27	7	526	53.4
P62280	RPS11	40S ribos	46	9	28	9	158	18.4
O76003	GLRX3	Glutaredo	32	11	24	11	335	37.4
P36957	DLST	Dihydroli	25	11	23	11	453	48.7
P52948	NUP98	Nuclear p	11	19	21	19	1817	197.5
O60361	NME2P1	Putative	66	8	34	1	137	15.5
Q9UGP8	SEC63	Transloca	23	15	21	15	760	87.9
P12004	PCNA	Prolifera	44	10	21	10	261	28.8
Q9UQE7	SMC3	Structura	15	17	21	17	1217	141.5
G3V0I5	NDUFV1	NADH dehy	42	16	20	16	457	50
Q9BZZ5	API5	Apoptosis	31	13	21	13	524	59
P30084	ECHS1	Enoyl-CoA	36	11	23	11	290	31.4
Q86W92	PPFIBP1	Liprin-be	18	16	19	16	1011	114
P13746	HLA-A	HLA class	33	8	16	2	365	40.9

Q15477	SKIV2L	Helicase	17	17	19	17	1246	137.7
Q12929	EPS8	Epidermal	18	11	14	11	822	91.8
Q08257	CRYZ	Quinone c	46	12	23	12	329	35.2
P13798	APEH	Acylaminc	18	13	22	13	732	81.2
E9PFP8	PCBP3	Poly(rC)-	22	7	27	1	361	38.2
Q9Y4K0	LOXL2	Lysyl oxi	20	15	21	14	774	86.7
Q8N6T3	ARFGAP1	ADP-ribos	34	10	19	10	406	44.6
P30419	NMT1	Glycylpep	30	14	22	11	496	56.8
Q14554	PDIA5	Protein d	27	14	20	14	519	59.6
A0A087WYN	DHX29	ATP-deper	13	16	19	16	1370	155.2
P48047	ATP5O	ATP synth	48	9	27	9	213	23.3
O75367	H2AFY	Core hist	34	9	17	8	372	39.6
P05120	SERPINB2	Plasminog	32	13	19	13	415	46.6
Q99961	SH3GL1	Endophili	41	16	26	16	368	41.5
Q9NR45	NANS	Sialic ac	45	12	26	12	359	40.3
P25789	PSMA4	Proteason	44	10	22	10	261	29.5
I3L2B0	CLUH	Clusterec	15	18	20	1	1236	138.1
Q86UE4	MTDH	Protein L	25	11	16	11	582	63.8
Q96I99	SUCLG2	Succinate	26	11	19	11	432	46.5
P17813	ENG	Endoglin	21	12	20	12	658	70.5
P61019	RAB2A	Ras-relat	39	7	21	7	212	23.5
Q8N3V7	SYNPO	Synaptopc	20	12	16	12	929	99.4
P25205	MCM3	DNA repli	25	20	23	20	808	90.9
Q96P70	IPO9	Importin-	15	14	17	14	1041	115.9
Q9UH65	SWAP70	Switch-as	28	17	22	16	585	69
O43396	TXNL1	Thioredox	44	8	15	8	289	32.2
Q04446	GBE1	1,4-alpha	20	13	21	13	702	80.4
Q9H2D6	TRIOBP	TRIO and	7	16	20	15	2365	261.2
Q9BY44	EIF2A	Eukaryoti	30	13	17	13	585	64.9
Q8IXB1	DNAJC10	DnaJ homc	18	12	19	12	793	91
O43592	XPOT	Exportin-	17	14	20	14	962	109.9
P62277	RPS13	40S ribos	64	11	44	11	151	17.2
P52888	THOP1	Thimet ol	25	15	18	15	689	78.8
A0A0D9SEN	FAP	Prolyl en	18	14	24	14	759	87.5
P68036	UBE2L3	Ubiquitir	49	6	27	6	154	17.9
B1AXG1	RPS6KA3	Non-speci	27	15	17	14	711	80.7
Q16706	MAN2A1	Alpha-mar	15	16	19	16	1144	131.1
Q6P996	PDXDC1	Pyridoxal	19	14	23	14	788	86.7
Q9UDT6	CLIP2	CAP-Gly d	17	18	21	12	1046	115.8
P16949	STMN1	Stathmin	60	11	27	9	149	17.3
C9JNG9	COL6A3	Collagen	25	15	17	1	708	77.1
O95831	AIFM1	Apoptosis	24	13	19	13	613	66.9
Q96HE7	ERO1A	ERO1-like	20	8	15	8	468	54.4
P46087	NOP2	Probable	18	12	17	11	812	89.2
P00533	EGFR	Epidermal	17	17	18	17	1210	134.2
O75390	CS	Citrate s	25	12	35	11	466	51.7
Q92621	NUP205	Nuclear p	8	14	18	14	2012	227.8
O95202	LETM1	Mitochonc	24	14	21	14	739	83.3
O75822	EIF3J	Eukaryoti	39	9	14	9	258	29
Q9Y3A5	SBDS	Ribosome	36	12	20	12	250	28.7
O43294	TGFB1I1	Transforn	40	13	16	13	461	49.8

P05534	HLA-A	HLA class	36	9	16	1	365	40.7
P29279	CTGF	Connectiv	54	15	19	15	349	38.1
O60749	SNX2	Sorting n	30	16	18	14	519	58.4
P49736	MCM2	DNA repli	19	17	20	17	904	101.8
Q12765	SCRN1	Secernin-	28	11	22	11	414	46.4
E7EU96	CSNK2A1	Casein ki	32	10	15	10	385	45.3
P31153	MAT2A	S-adenosy	32	13	22	13	395	43.6
P22087	FBL	rRNA 2'-C	37	11	19	11	321	33.8
Q16666	IFI16	Gamma-int	22	14	19	13	785	88.2
Q99714	HSD17B10	3-hydroxy	39	7	18	7	261	26.9
H0Y9Y3	SYNPO2	Synaptopc	17	14	20	14	1155	125.3
B5MDF5	RAN	GTP-bindi	35	9	31	9	233	26.2
Q9UBT2	UBA2	SUMO-acti	22	14	20	14	640	71.2
P48637	GSS	Glutathic	34	16	21	16	474	52.4
O60684	KPNA6	Importin	27	13	18	9	536	60
P50281	MMP14	Matrix me	21	12	21	12	582	65.9
O75396	SEC22B	Vesicle-t	33	7	28	7	215	24.6
O94851	MICAL2	[F-actin]	13	13	18	12	1124	126.6
P52926	HMGA2	High mobi	49	7	24	7	109	11.8
P54687	BCAT1	Branched-	30	10	25	10	386	42.9
H7BXI1	ESYT2	Extended	16	11	17	11	884	97.9
Q9BTV4	TMEM43	Transmemb	36	13	25	13	400	44.8
P98194	ATP2C1	Calcium-t	18	13	15	13	919	100.5
P84098	RPL19	60S ribos	42	10	46	10	196	23.5
E9PEX6	DLD	Dihydroli	23	10	20	10	486	51.8
Q96N67	DOCK7	Dedicator	8	14	17	13	2140	242.4
Q9UBE0	SAE1	SUMO-acti	37	12	18	12	346	38.4
P40763	STAT3	Signal tr	20	13	17	13	770	88
Q13617	CUL2	Cullin-2	22	16	20	16	745	86.9
Q8WWM7	ATXN2L	Ataxin-2-	16	15	15	14	1075	113.3
Q8WXF1	PSPC1	Paraspeck	23	10	15	10	523	58.7
Q9UPQ0	LIMCH1	LIM and c	16	15	19	15	1083	121.8
Q13126	MTAP	S-methyl-	47	9	15	9	283	31.2
P16189	HLA-A	HLA class	32	8	14	1	365	41
P61088	UBE2N	Ubiquitir	58	8	26	8	152	17.1
P20618	PSMB1	Proteasom	39	7	21	7	241	26.5
P08559	PDHA1	Pyruvate	34	13	23	13	390	43.3
Q13347	EIF3I	Eukaryoti	34	10	22	10	325	36.5
Q93009	USP7	Ubiquitir	15	15	19	15	1102	128.2
P26373	RPL13	60S ribos	33	10	37	10	211	24.2
A5YKK6	CNOT1	CCR4-NOT	8	17	18	17	2376	266.8
P33992	MCM5	DNA repli	19	11	13	11	734	82.2
P42765	ACAA2	3-ketoacy	42	11	17	11	397	41.9
P26196	DDX6	Probable	30	11	17	11	483	54.4
Q14141	SEPT6	Septin-6	30	9	19	3	434	49.7
A0A087WT4	HMOX2	Heme oxyg	40	12	19	12	370	41.6
B8ZWD1	DBI	Acyl-CoA-	57	5	23	5	97	11.1
F8WAN1	SPECC1L-A	SPECC1L-A	16	15	18	14	911	101.5
P61313	RPL15	60S ribos	50	11	36	11	204	24.1
X6RFL8	RAB14	Ras-relat	63	11	18	10	181	20.4
Q9BYT8	NLN	Neurolysi	20	13	18	13	704	80.6

Q8N1G4	LRRC47	Leucine-r	26	13	17	13	583	63.4
Q96KG9	SCYL1	N-termina	22	15	21	15	808	89.6
P41240	CSK	Tyrosine-	25	11	17	11	450	50.7
O43615	TIMM44	Mitochond	31	12	16	12	452	51.3
P52597	HNRNPF	Heterogen	31	10	23	8	415	45.6
Q14019	COTL1	Coactosin	64	10	32	10	142	15.9
Q14566	MCM6	DNA repli	18	15	17	15	821	92.8
P21281	ATP6V1B2	V-type pr	24	12	19	12	511	56.5
P41227	NAA10	N-alpha-a	49	10	14	10	235	26.4
Q6VY07	PACS1	Phosphofu	16	14	16	14	963	104.8
P61204	ARF3	ADP-ribos	39	7	32	4	181	20.6
Q9H857	NT5DC2	5'-nuclec	34	16	19	16	520	60.7
Q9HDC9	APMAP	Adipocyte	33	12	21	12	416	46.5
Q00688	FKBP3	Peptidyl-	55	12	30	12	224	25.2
Q14690	PDCD11	Protein R	8	15	18	15	1871	208.6
P55268	LAMB2	Laminin s	9	14	17	14	1798	195.9
A0A0G2JLE	GBA	Glucosylc	23	11	17	11	536	59.6
P40123	CAP2	Adenylyl	25	10	22	8	477	52.8
G5E972	TMPO	Lamina-as	36	9	13	5	414	46.3
O60264	SMARCA5	SWI/SNF-r	17	19	21	16	1052	121.8
Q9NYF8	BCLAF1	Bcl-2-ass	18	16	19	15	920	106.1
O15143	ARPC1B	Actin-rel	34	11	24	11	372	40.9
F8VW96	CSRP2	Cysteine	34	8	23	8	243	26.7
Q15404	RSU1	Ras suppr	43	10	33	10	277	31.5
A0A140T91	HLA-C	HLA class	35	9	16	2	372	41.4
Q9NSE4	IARS2	Isoleucin	18	17	18	17	1012	113.7
Q13442	PDAP1	28 kDa he	46	10	19	10	181	20.6
Q5SSJ5	HP1BP3	Heterochr	23	13	21	13	553	61.2
P08648	ITGA5	Integrin	12	11	20	11	1049	114.5
P07686	HEXB	Beta-hexc	19	12	22	11	556	63.1
Q9Y6G9	DYNC1LI1	Cytoplasm	28	11	18	11	523	56.5
Q9H0B6	KLC2	Kinesin l	25	14	19	7	622	68.9
P61163	ACTR1A	Alpha-cen	26	11	27	5	376	42.6
Q9UJZ1	STOML2	Stomatin-	29	7	15	7	356	38.5
O94906	PRPF6	Pre-mRNA-	16	15	18	15	941	106.9
P16278	GLB1	Beta-gala	13	7	14	7	677	76
F8W726	UBAP2L	Ubiquitin	15	10	15	10	1079	113.6
Q96RP9	GFM1	Elongatic	21	14	19	14	751	83.4
Q9UMX0	UBQLN1	Ubiquilin	23	8	16	5	589	62.5
Q86VS8	HOOK3	Protein H	20	14	17	14	718	83.1
Q9Y680	FKBP7	Peptidyl-	43	12	17	12	222	25.8
F6TLX2	GLOD4	Glyoxalas	27	13	22	4	502	54.7
O60271	SPAG9	C-Jun-ami	11	12	16	12	1321	146.1
Q68EM7	ARHGAP17	Rho GTPas	15	12	15	12	881	95.4
A0A087WVF	SMTN	Smootheli	17	16	20	16	971	104.8
Q14683	SMC1A	Structura	11	15	17	15	1233	143.1
Q99615	DNAJC7	DnaJ homc	29	14	17	14	494	56.4
Q5JXI2	FHL1	Four and	43	9	29	1	210	23.7
P14735	IDE	Insulin-d	15	14	19	14	1019	117.9
O43747	AP1G1	AP-1 comp	17	14	20	14	822	91.3
O43237	DYNC1LI2	Cytoplasm	24	13	15	13	492	54.1

A0A087WZKEIF3H		Eukaryoti	28	10	25	10	349	39.6
P84095	RHOG	Rho-relat	61	9	13	8	191	21.3
A0A0A0MRFAKAP9		A-kinase	4	16	17	15	3910	453.2
H0YIV4	NAP1L1	Nucleosom	24	8	20	7	385	44.7
O00116	AGPS	Alkyldihy	24	10	14	10	658	72.9
Q99733	NAP1L4	Nucleosom	29	10	24	9	375	42.8
O94776	MTA2	Metastasi	23	16	18	12	668	75
Q7Z417	NUFIP2	Nuclear f	20	12	14	12	695	76.1
Q9NTJ5	SACM1L	Phosphati	23	14	21	14	587	66.9
Q96B97	SH3KBP1	SH3 domai	23	12	16	12	665	73.1
P62070	RRAS2	Ras-relat	51	10	18	8	204	23.4
Q9UKK3	PARP4	Poly [ADF	8	13	17	13	1724	192.5
A0A2R8Y84RPS24		40S ribos	40	6	26	6	131	15.2
Q9Y6E2	BZW2	Basic leu	31	14	23	14	419	48.1
Q15785	TOMM34	Mitochond	45	12	19	11	309	34.5
Q9NUQ6	SPATS2L	SPATS2-li	24	12	16	5	558	61.7
Q6PKG0	LARP1	La-relate	14	15	17	15	1096	123.4
Q13098	GPS1	COP9 sigr	29	12	18	1	491	55.5
Q86V48	LUZP1	Leucine z	11	13	17	13	1076	120.2
Q9UQ35	SRRM2	Serine/ar	7	13	15	13	2752	299.4
O00487	PSMD14	26S prote	43	12	18	12	310	34.6
P98082	DAB2	Disabled	17	13	19	13	770	82.4
P09110	ACAA1	3-ketoacy	33	8	12	8	424	44.3
Q0ZGT2	NEXN	Nexilin C	20	14	20	14	675	80.6
K7ER00	FARSA	Phenylala	20	10	20	10	548	62.4
Q14151	SAFB2	Scaffold	12	11	16	6	953	107.4
Q5JWF2	GNAS	Guanine n	10	11	18	9	1037	111
A6NFX8	NUDT5	ADP-sugar	47	9	15	9	232	25.9
P47755	CAPZA2	F-actin-c	53	9	18	7	286	32.9
Q13616	CUL1	Cullin-1	19	15	17	15	776	89.6
Q8N766	EMC1	ER membra	14	13	19	13	993	111.7
Q12874	SF3A3	Splicing	27	12	18	12	501	58.8
Q6P2E9	EDC4	Enhancer	12	13	16	13	1401	151.6
O75947	ATP5H	ATP synth	76	11	22	11	161	18.5
H7BZJ3	PDIA3	Protein d	57	7	30	1	123	13.5
P14550	AKR1A1	Alcohol d	44	13	27	13	325	36.6
P62888	RPL30	60S ribos	57	7	22	7	115	12.8
M0R210	RPS16	40S ribos	60	10	36	10	129	14.4
Q13546	RIPK1	Receptor-	20	12	15	12	671	75.9
O75347	TBCA	Tubulin-s	59	9	21	9	108	12.8
O95479	H6PD	GDH/6PGL	19	12	16	12	791	88.8
P33316	DUT	Deoxyurid	42	8	14	8	252	26.5
J3KRX5	RPL17	60S ribos	47	9	32	8	174	20.2
P40429	RPL13A	60S ribos	43	13	50	13	203	23.6
P40261	NNMT	Nicotinam	31	8	30	8	264	29.6
O95352	ATG7	Ubiquitir	17	11	15	11	703	77.9
A0A096LPJGPS1		COP9 sigr	29	12	18	1	490	55.4
O00303	EIF3F	Eukaryoti	26	9	17	9	357	37.5
O95347	SMC2	Structura	11	14	15	14	1197	135.6
M0R3D6	RPL18A	60S ribos	44	7	26	7	141	16.7
P25325	MPST	3-mercapt	42	10	17	10	297	33.2

E7ETY2	TCOF1	Treacle p	11	16	16	16	1488	152.2
Q13619	CUL4A	Cullin-4A	19	15	19	5	759	87.6
O14579	COPE	Coatomer	37	9	16	9	308	34.5
P48449	LSS	Lanosterc	18	12	19	12	732	83.3
Q9UG63	ABCF2	ATP-bindi	18	12	20	12	623	71.2
Q14677	CLINT1	Clathrin	21	13	14	13	625	68.2
P17612	PRKACA	cAMP-depe	32	10	19	3	351	40.6
Q00169	PITPNA	Phosphati	47	13	22	10	270	31.8
Q9UJ70	NAGK	N-acetyl-	37	13	18	3	344	37.4
Q8TDZ2	MICAL1	[F-actin]	14	13	17	13	1067	117.8
O15511	ARPC5	Actin-rel	66	7	21	7	151	16.3
H3BPC4	UBE2I	SUMO-conj	79	8	20	8	70	8
Q92890	UFD1	Ubiquitin	38	10	15	10	307	34.5
Q9BWD1	ACAT2	Acetyl-Cc	34	7	9	7	397	41.3
P17174	GOT1	Aspartate	28	11	17	11	413	46.2
O00461	GOLIM4	Golgi int	17	13	18	13	696	81.8
P55809	OXCT1	Succinyl-	19	8	17	8	520	56.1
Q9H9B4	SFXN1	Siderofle	34	9	15	9	322	35.6
Q8WU90	ZC3H15	Zinc fing	32	12	16	12	426	48.6
Q13217	DNAJC3	DnaJ homc	33	16	21	16	504	57.5
P08754	GNAI3	Guanine n	32	11	16	4	354	40.5
Q96PK6	RBM14	RNA-bindi	20	13	19	13	669	69.4
P50479	PDLIM4	PDZ and L	42	10	21	10	330	35.4
Q01105	SET	Protein S	29	8	22	8	290	33.5
Q15643	TRIP11	Thyroid r	9	17	18	17	1979	227.4
O00148	DDX39A	ATP-depen	28	12	23	5	427	49.1
Q9UBQ7	GRHPR	Glyoxylat	29	7	13	7	328	35.6
P15121	AKR1B1	Aldose re	34	10	25	10	316	35.8
Q9P0L0	VAPA	Vesicle-a	43	10	26	9	249	27.9
Q92922	SMARCC1	SWI/SNF c	13	12	17	6	1105	122.8
Q9ULC4	MCTS1	Malignant	59	9	14	9	181	20.5
Q14498	RBM39	RNA-bindi	19	9	16	9	530	59.3
P49755	TMED10	Transmemb	40	8	16	8	219	25
F8W7C6	RPL10	60S ribos	46	7	34	1	163	18.6
Q96IZ0	PAWR	PRKC apop	36	7	12	7	340	36.5
P09543	CNP	2',3'-cyc	26	14	16	14	421	47.5
Q9H0A0	NAT10	RNA cytid	17	15	16	15	1025	115.7
P62987	UBA52	Ubiquitin	59	9	120	1	128	14.7
Q7Z460	CLASP1	CLIP-assc	9	12	13	9	1538	169.3
P21283	ATP6V1C1	V-type pr	40	16	17	16	382	43.9
P52788	SMS	Spermine	32	12	18	12	366	41.2
P35659	DEK	Protein D	22	8	14	8	375	42.6
A0A0A0MRM	MYO6	Unconvent	11	14	16	14	1253	144.9
O75489	NDUFS3	NADH dehy	47	11	19	11	264	30.2
A0A0U1RR3	hCG_20395	Histone H	49	7	36	1	169	18.5
P32455	GBP1	Guanylate	22	13	16	11	592	67.9
O14558	HSPB6	Heat shoc	53	5	28	5	160	17.1
P55263	ADK	Adenosine	34	10	16	10	362	40.5
Q7Z2W4	ZC3HAV1	Zinc fing	17	14	18	14	902	101.4
P29992	GNA11	Guanine n	28	10	15	5	359	42.1
Q9UKY7	CDV3	Protein C	53	9	13	9	258	27.3

P83436	COG7	Conservec	19	13	15	13	770	86.3
Q6XZF7	DNMBP	Dynamin-b	9	12	14	12	1577	177.2
O95433	AHSA1	Activator	36	11	18	11	338	38.3
O95793	STAU1	Double-st	12	8	13	2	577	63.1
P52292	KPNA2	Importin	19	9	12	9	529	57.8
P42892	ECE1	Endotheli	18	14	19	14	770	87.1
P33993	MCM7	DNA repli	23	15	18	15	719	81.3
P35237	SERPINB6	Serpin B6	36	11	16	11	376	42.6
P32322	PYCR1	Pyrroline	33	8	17	7	319	33.3
Q07666	KHDRBS1	KH domair	23	8	18	8	443	48.2
O95816	BAG2	BAG famil	41	12	24	11	211	23.8
P50148	GNAQ	Guanine n	30	10	15	5	359	42.1
P08621	SNRNP70	U1 small	25	13	22	13	437	51.5
H7C286	NAGK	N-acetyl-	61	11	15	1	196	22
Q01433	AMPD2	AMP deami	17	15	17	15	879	100.6
Q14166	TTLL12	Tubulin--	17	9	16	9	644	74.4
Q10713	PMPCA	Mitochonc	23	10	14	10	525	58.2
Q00577	PURA	Transcrip	31	9	15	9	322	34.9
O15355	PPM1G	Protein p	23	13	19	13	546	59.2
P00441	SOD1	Superoxic	44	8	33	8	154	15.9
P19525	EIF2AK2	Interferc	19	10	15	10	551	62.1
Q9UEW8	STK39	STE20/SPS	23	12	13	8	545	59.4
Q9BUQ8	DDX23	Probable	15	12	14	12	820	95.5
Q9UNE7	STUB1	E3 ubiqui	40	13	16	12	303	34.8
Q9Y223	GNE	Bifunctic	19	13	15	13	722	79.2
Q9BUJ2	HNRNPUL1	Heteroger	19	12	13	12	856	95.7
D6RB85	CANX	Calnexin	48	9	29	1	144	16
P31040	SDHA	Succinate	20	11	20	11	664	72.6
Q53GQ0	HSD17B12	Very-long	26	7	20	7	312	34.3
Q9UH99	SUN2	SUN domai	24	12	17	11	717	80.3
P39656	DDOST	Dolichyl-	24	11	25	11	456	50.8
P48556	PSMD8	26S prote	43	13	19	13	350	39.6
Q14165	MLEC	Malectin	29	8	16	8	292	32.2
P13796	LCP1	Plastin-2	12	8	19	1	627	70.2
P33991	MCM4	DNA repli	16	15	19	15	863	96.5
Q8TAT6	NPLOC4	Nuclear p	26	15	20	15	608	68.1
O96019	ACTL6A	Actin-lik	20	9	14	9	429	47.4
Q86XP3	DDX42	ATP-deper	19	13	16	13	938	102.9
P59998	ARPC4	Actin-rel	69	10	25	10	168	19.7
Q5T9A4	ATAD3B	ATPase fa	22	16	19	4	648	72.5
Q5GLZ8	HERC4	Probable	12	12	14	12	1057	118.5
Q9BR76	CORO1B	Coronin-1	21	12	22	12	489	54.2
O43865	AHCYL1	S-adenosy	24	14	17	6	530	58.9
P84085	ARF5	ADP-ribos	45	7	26	4	180	20.5
Q9UNF1	MAGED2	Melanoma-	18	12	22	11	606	64.9
Q99471	PFDN5	Prefoldir	54	7	19	7	154	17.3
P09104	ENO2	Gamma-enc	26	8	31	6	434	47.2
H0YGR4	REXO2	Oligoribc	34	7	21	7	192	22.1
P17252	PRKCA	Protein k	24	14	20	13	672	76.7
Q9UIJ7	AK3	GTP:AMP p	44	10	13	10	227	25.6
O00170	AIP	AH recept	37	10	14	10	330	37.6

P36542	ATP5F1C	ATP synth	23	7	17	7	298	33
P18031	PTPN1	Tyrosine-	29	12	16	12	435	49.9
Q92688	ANP32B	Acidic le	27	8	18	4	251	28.8
P43304	GPD2	Glycerol-	18	13	14	13	727	80.8
Q99747	NAPG	Gamma-sol	35	10	13	10	312	34.7
E9PBG7	CAMK2D	Calcium/c	26	11	16	9	512	57.7
Q9UBQ5	EIF3K	Eukaryoti	44	7	13	7	218	25
P62266	RPS23	40S ribos	29	5	21	5	143	15.8
P15374	UCHL3	Ubiquitir	41	7	12	7	230	26.2
K9J957	PSME3	Proteasom	39	10	14	10	231	26.9
P12107	COL11A1	Collagen	9	13	17	9	1806	181
O75976	CPD	Carboxype	10	13	15	13	1380	152.8
O00151	PDLIM1	PDZ and L	39	10	13	10	329	36
Q13443	ADAM9	Disintegr	21	14	14	14	819	90.5
E7EW49	CLASP2	CLIP-assc	9	12	13	9	1514	165.6
P42566	EPS15	Epidermal	18	14	15	14	896	98.6
Q13618	CUL3	Cullin-3	18	14	15	14	768	88.9
P04632	CAPNS1	Calpain s	35	7	22	7	268	28.3
P11388	TOP2A	DNA topoi	9	14	16	8	1531	174.3
O75955	FLOT1	Flotillir	28	10	13	10	427	47.3
P54289	CACNA2D1	Voltage-d	14	13	13	13	1103	124.5
Q15121	PEA15	Astrocyti	58	6	15	6	130	15
A0A2R8Y6Y	SUCLA2	Succinate	30	13	15	13	484	52.6
Q8N0X7	SPART	Spartin C	21	14	18	14	666	72.8
G3V1R5	NRDC	Nardilysi	10	12	14	12	1087	125
E7EQ69	NAA50	N-alpha-a	55	9	15	9	168	19.3
Q9HCC0	MCCC2	Methylcrc	18	8	12	8	563	61.3
Q96AQ6	PBXIP1	Pre-B-cel	19	11	15	11	731	80.6
Q15020	SART3	Squamous	11	12	14	12	963	109.9
A0A0A0MSE	EXOC7	Exocyst c	19	12	13	12	693	78.8
P31942	HNRNPH3	Heterogen	25	7	17	6	346	36.9
Q5JR08	RHOC	Rho-relat	34	6	17	1	188	21.5
P61201	COPS2	COP9 sigr	30	12	19	12	443	51.6
Q9UHV9	PFDN2	Prefoldir	60	10	18	10	154	16.6
Q96JJ7	TMX3	Protein d	22	9	16	9	454	51.8
Q13425	SNTB2	Beta-2-sy	26	12	14	11	540	57.9
P17096	HMGA1	High mobi	56	7	24	7	107	11.7
Q5JTV8	TOR1AIP1	Torsin-1A	22	13	16	12	583	66.2
P04040	CAT	Catalase	22	10	13	10	527	59.7
Q9Y4P3	TBL2	Transduci	25	13	16	13	447	49.8
Q99426	TBCB	Tubulin-f	45	9	23	9	244	27.3
O60832	DKC1	H/ACA rib	23	14	18	14	514	57.6
Q9H6R4	NOL6	Nucleolar	14	13	14	13	1146	127.5
Q92804	TAF15	TATA-binc	29	11	18	9	592	61.8
Q7Z4I7	LIMS2	LIM and s	20	7	18	2	341	38.9
P25685	DNAJB1	DnaJ homc	35	12	22	11	340	38
Q14258	TRIM25	E3 ubiqui	21	10	12	10	630	70.9
Q6NUK1	SLC25A24	Calcium-b	24	13	21	13	477	53.3
O95486	SEC24A	Protein t	11	10	14	9	1093	119.7
O75475	PSIP1	PC4 and S	22	11	20	10	530	60.1
P08473	MME	Neprilysi	16	12	14	12	750	85.5

Q969V3	NCLN	Nicalin C	16	8	13	8	563	62.9
H0YD13	CD44	CD44 anti	35	7	30	7	206	22.7
Q09028	RBBP4	Histone-b	24	10	19	3	425	47.6
Q15056	EIF4H	Eukaryoti	47	10	21	10	248	27.4
O75436	VPS26A	Vacuolar	34	9	18	8	327	38.1
Q8N392	ARHGAP18	Rho GTPas	21	14	15	14	663	74.9
Q99459	CDC5L	Cell divi	19	10	11	10	802	92.2
P43490	NAMPT	Nicotinam	20	9	17	9	491	55.5
H0YM70	PSME2	Proteasom	35	7	16	7	228	26
H7C0E5	ZPR1	Zinc fing	29	11	16	11	386	42.6
O60256	PRPSAP2	Phosphori	39	12	14	10	369	40.9
Q9UNS2	COPS3	COP9 sign	25	8	14	8	423	47.8
Q53FA7	TP53I3	Quinone c	36	11	23	11	332	35.5
D3YTB1	RPL32	60S ribos	46	7	24	7	133	15.6
G3V3E8	NPC2	NPC intra	37	6	16	6	174	19.2
Q9H845	ACAD9	Acyl-CoA	17	10	15	10	621	68.7
P48163	ME1	NADP-depe	24	13	19	13	572	64.1
Q08AF3	SLFN5	Schlafen	15	14	16	13	891	101
Q13011	ECH1	Delta(3,5	33	10	15	10	328	35.8
P10768	ESD	S-formylg	27	8	15	8	282	31.4
E9PFN5	GSTK1	Glutathic	44	7	15	7	190	21.7
P10515	DLAT	Dihydroli	22	12	16	12	647	69
F5GZ78	PXN	Paxillin	18	11	15	11	589	64.2
P31749	AKT1	RAC-alpha	28	12	14	8	480	55.7
Q9H8Y8	GORASP2	Golgi rea	25	10	15	9	452	47.1
Q9NP72	RAB18	Ras-relat	51	8	14	8	206	23
E7EM64	COPS6	COP9 sign	35	10	18	10	326	36
P28370	SMARCA1	Probable	15	15	15	12	1054	122.5
Q06124	PTPN11	Tyrosine-	25	15	18	15	597	68.4
D6RB59	EXOC3	Exocyst c	22	11	14	11	462	53.2
I1E4Y6	GIGYF2	GRB10-int	12	13	15	13	1321	152.4
P40121	CAPG	Macrophag	21	6	11	6	348	38.5
A0A087WY5	VTA1	Chromosom	29	8	13	8	280	31.1
A6NHL2	TUBAL3	Tubulin a	10	5	40	1	446	49.9
K7ELC7	RPL27	60S ribos	45	8	30	7	144	16.5
P61081	UBE2M	NEDD8-con	54	10	15	10	183	20.9
P04179	SOD2	Superoxid	33	8	21	8	222	24.7
P20936	RASA1	Ras GTPas	11	10	12	10	1047	116.3
Q9UHD1	CHORDC1	Cysteine	36	9	13	9	332	37.5
P50914	RPL14	60S ribos	31	7	24	7	215	23.4
P61020	RAB5B	Ras-relat	38	6	11	4	215	23.7
Q92905	COPS5	COP9 sign	31	8	11	8	334	37.6
P53007	SLC25A1	Tricarbox	24	8	15	8	311	34
A0A024RCR	BAG6	HLA-B ass	12	9	12	9	1126	118.6
F5GYQ1	ATP6V0D1	V-type pr	27	10	17	10	392	44.6
O14737	PDCD5	Programme	59	8	17	8	125	14.3
P61604	HSPE1	10 kDa he	64	9	29	5	102	10.9
Q9Y295	DRG1	Developme	28	8	12	8	367	40.5
P20339	RAB5A	Ras-relat	49	7	13	5	215	23.6
Q5JSH3	WDR44	WD repeat	15	13	13	13	913	101.3
P42166	TMPO	Lamina-as	17	7	11	3	694	75.4

P20290	BTF3	Transcrip	57	9	19	5	206	22.2
E7EWW0	VPS35L	VPS35 end	10	11	13	11	1052	118.5
O15061	SYNM	Synemin C	8	14	14	14	1565	172.7
P13073	COX4I1	Cytochrom	40	8	24	8	169	19.6
Q9Y371	SH3GLB1	Endophili	32	10	14	9	365	40.8
Q9HD45	TM9SF3	Transmemb	13	8	14	8	589	67.8
O75828	CBR3	Carbonyl	32	8	15	6	277	30.8
O75746	SLC25A12	Calcium-b	18	10	12	9	678	74.7
Q15631	TSN	Translin	37	8	15	8	228	26.2
Q9NRV9	HEBP1	Heme-bind	59	9	12	9	189	21.1
A0A1B0GUA	KIF1BP	KIF1-bind	22	12	17	12	646	74.7
O00139	KIF2A	Kinesin-l	16	11	13	11	706	79.9
E9PDE8	HSPA4L	Heat shoc	10	8	13	3	813	91.9
Q14254	FLOT2	Flotillin	29	12	16	12	428	47
O75351	VPS4B	Vacuolar	25	10	14	6	444	49.3
P47813	EIF1AX	Eukaryoti	38	8	18	1	144	16.5
Q15907	RAB11B	Ras-relat	44	9	21	9	218	24.5
Q92520	FAM3C	Protein F	33	6	11	6	227	24.7
O00161	SNAP23	Synaptoso	41	7	10	7	211	23.3
Q96A65	EXOC4	Exocyst c	15	14	15	14	974	110.4
P10301	RRAS	Ras-relat	40	7	12	5	218	23.5
Q96JB5	CDK5RAP3	CDK5 regu	21	10	12	10	506	56.9
Q04695	KRT17	Keratin,	17	11	19	1	432	48.1
P63151	PPP2R2A	Serine/th	23	8	13	6	447	51.7
Q9NVP1	DDX18	ATP-depen	17	11	13	11	670	75.4
Q96K76	USP47	Ubiquitin	11	12	12	12	1375	157.2
Q9HC35	EML4	Echinoder	13	11	13	11	981	108.8
P04424	ASL	Argininos	27	12	15	12	464	51.6
Q16630	CPSF6	Cleavage	22	10	18	10	551	59.2
Q96A33	CCDC47	Coiled-co	25	9	12	9	483	55.8
Q9Y4W6	AFG3L2	AFG3-like	18	17	18	16	797	88.5
J3KS54	FLII	Protein f	21	12	15	1	700	78
P23921	RRM1	Ribonucle	15	11	16	11	792	90
O60443	GSDME	Gasdermin	22	8	9	8	496	54.5
Q8WXX5	DNAJC9	DnaJ homo	28	7	10	7	260	29.9
Q9Y305	ACOT9	Acyl-coen	23	10	16	10	439	49.9
O14776	TCERG1	Transcrip	13	13	14	13	1098	123.8
Q9H5N1	RABEP2	Rab GTPas	21	12	13	12	569	63.5
O43488	AKR7A2	Aflatoxin	26	8	12	8	359	39.6
B5MCF9	PES1	Pescadill	21	13	15	13	571	66
Q9BQS8	FYCO1	FYVE and	9	14	15	14	1478	166.9
Q8TEX9	IPO4	Importin-	13	12	17	12	1081	118.6
Q8WUM0	NUP133	Nuclear p	8	9	11	9	1156	128.9
Q9UI15	TAGLN3	Transgeli	32	6	22	4	199	22.5
P11498	PC	Pyruvate	11	10	12	10	1178	129.6
Q5T123	SH3BGRL3	SH3 domai	51	6	23	6	88	9.4
P27361	MAPK3	Mitogen-a	26	9	18	5	379	43.1
P52943	CRIP2	Cysteine-	39	5	11	5	208	22.5
Q14318	FKBP8	Peptidyl-	22	8	11	8	412	44.5
P60953	CDC42	Cell divi	42	7	23	6	191	21.2
Q14232	EIF2B1	Translati	30	10	15	10	305	33.7

Q5QPL9	RALY	RNA-bindi	43	11	13	11	237	24.7
A0A0A0MS5	PRKACB	cAMP-depe	27	9	17	2	357	41.3
E9PC52	RBBP7	Histone-b	22	9	17	2	416	46.9
Q9ULT8	HECTD1	E3 ubiqui	5	13	13	13	2610	289.2
Q01780	EXOSC10	Exosome c	14	11	12	11	885	100.8
Q9HAV0	GNB4	Guanine n	31	9	15	4	340	37.5
P35442	THBS2	Thrombosp	9	9	12	9	1172	129.9
P28482	MAPK1	Mitogen-a	28	10	17	6	360	41.4
P54819	AK2	Adenylate	32	7	14	7	239	26.5
P31937	HIBADH	3-hydroxy	23	5	8	5	336	35.3
Q86UU1	PHLDB1	Pleckstri	9	11	12	6	1377	151.1
Q92769	HDAC2	Histone d	20	8	11	5	488	55.3
Q70E73	RAPH1	Ras-assoc	11	12	14	12	1250	135.2
O15357	INPPL1	Phosphati	10	12	13	12	1258	138.5
Q16777	HIST2H2AC	Histone H	63	7	35	2	129	14
Q9UJS0	SLC25A13	Calcium-b	16	9	11	8	675	74.1
P36405	ARL3	ADP-ribos	48	8	16	8	182	20.4
B4DLN1		cDNA FLJ6	18	7	14	2	442	48.1
Q9NUQ9	FAM49B	Protein F	35	9	11	9	324	36.7
P61086	UBE2K	Ubiquitin	45	7	20	7	200	22.4
Q01970	PLCB3	1-phospha	8	9	10	9	1234	138.7
O75439	PMPCB	Mitochond	22	11	13	10	489	54.3
O00273	DFFA	DNA fragm	31	9	14	9	331	36.5
Q13242	SRSF9	Serine/ar	37	9	15	9	221	25.5
A0A024R4M	RPS9	40S ribos	40	10	25	10	194	22.6
P46779	RPL28	60S ribos	49	9	32	9	137	15.7
Q9UDY2	TJP2	Tight jun	12	14	14	14	1190	133.9
K7EQJ5	RPS15	40S ribos	28	5	25	5	141	16.6
P28161	GSTM2	Glutathio	52	12	16	7	218	25.7
I3L3Q4	GLOD4	Glyoxalas	44	10	16	1	227	25.5
P32969	RPL9	60S ribos	29	6	20	6	192	21.9
P07858	CTSB	Cathepsin	24	7	20	7	339	37.8
A6NDG6	PGP	Glycerol-	29	7	10	7	321	34
A0A1W2PQR	SCARB2	Lysosome	14	6	14	6	449	51
A2RRP1	NBAS	Neuroblas	6	13	14	13	2371	268.4
Q9UK76	JPT1	Jupiter m	42	6	16	6	154	16
O60493	SNX3	Sorting n	47	8	14	6	162	18.8
Q15582	TGFBI	Transform	20	12	16	12	683	74.6
Q86V21	AACS	Acetoacet	18	12	17	12	672	75.1
B0QYK0	EWSR1	RNA-bindi	11	6	13	6	618	64.9
Q86Y82	STX12	Syntaxin-	31	8	14	8	276	31.6
O14602	EIF1AY	Eukaryoti	38	8	17	1	144	16.4
Q16512	PKN1	Serine/th	12	13	13	12	942	103.9
F8VZJ2	NACA	Nascent p	36	4	19	4	136	15
O43493	TGOLN2	Trans-Gol	16	7	14	7	479	51
P07093	SERPINE2	Glia-deri	23	9	11	9	398	44
Q6PCE3	PGM2L1	Glucose 1	18	11	13	10	622	70.4
Q6DKJ4	NXN	Nucleored	26	9	11	9	435	48.4
O00505	KPNA3	Importin	17	8	19	4	521	57.8
P16989	YBX3	Y-box-bin	26	7	15	5	372	40.1
Q14137	BOP1	Ribosome	17	10	11	10	746	83.6

P28072	PSMB6	Proteasom	30	8	20	8	239	25.3
O43290	SART1	U4/U6.U5	15	9	11	9	800	90.2
O00442	RTCA	RNA 3'-te	27	7	9	7	366	39.3
O60888	CUTA	Protein C	34	5	14	5	179	19.1
P18859	ATP5J	ATP synth	45	5	12	5	108	12.6
Q13131	PRKAA1	5'-AMP-ac	24	10	12	10	559	64
P30626	SRI	Sorcin OS	46	8	18	8	198	21.7
P06703	S100A6	Protein S	66	7	52	7	90	10.2
Q16186	ADRM1	Proteasom	23	10	18	10	407	42.1
Q12907	LMAN2	Vesicular	22	8	16	8	356	40.2
O76031	CLPX	ATP-depen	17	10	11	10	633	69.2
Q9NQ88	TIGAR	Fructose-	28	7	12	7	270	30
P30043	BLVRB	Flavin re	53	8	15	8	206	22.1
Q9BRF8	CPPED1	Serine/th	37	9	14	9	314	35.5
O43684	BUB3	Mitotic c	32	9	10	9	328	37.1
P09497	CLTB	Clathrin	31	9	18	9	229	25.2
Q04721	NOTCH2	Neurogeni	5	9	10	9	2471	265.2
Q9UBI6	GNG12	Guanine n	69	5	15	5	72	8
A0A087X14	SEPT8	Septin-8	20	8	12	5	426	49.3
P39687	ANP32A	Acidic le	22	7	17	3	249	28.6
Q9H444	CHMP4B	Charged m	27	6	16	6	224	24.9
P25787	PSMA2	Proteasom	35	7	14	7	234	25.9
P30085	CMPK1	UMP-CMP k	44	7	17	7	196	22.2
P30038	ALDH4A1	Delta-1-p	20	11	12	11	563	61.7
P50579	METAP2	Methionin	24	10	13	10	478	52.9
Q99805	TM9SF2	Transmemb	12	7	16	7	663	75.7
Q9UHQ9	CYB5R1	NADH-cytc	28	9	15	9	305	34.1
Q8TD16	BICD2	Protein b	13	11	12	11	824	93.5
P53990	IST1	IST1 homc	27	10	12	10	364	39.7
Q14669	TRIP12	E3 ubiqui	6	11	12	11	1992	220.3
P62993	GRB2	Growth fa	47	10	13	10	217	25.2
Q9H7Z7	PTGES2	Prostagla	25	9	12	9	377	41.9
Q8IVM0	CCDC50	Coiled-cc	31	9	11	9	306	35.8
Q9H832	UBE2Z	Ubiquitin	26	8	11	8	354	38.2
Q9Y2T2	AP3M1	AP-3 comp	21	8	12	8	418	46.9
P53597	SUCLG1	Succinate	20	6	13	6	346	36.2
Q9H2G2	SLK	STE20-lik	9	12	13	11	1235	142.6
P26006	ITGA3	Integrin	7	7	12	7	1051	116.5
Q9BRK5	SDF4	45 kDa ca	28	9	12	9	362	41.8
Q9BZF1	OSBPL8	Oxysterol	14	11	11	11	889	101.1
P51153	RAB13	Ras-relat	38	8	16	6	203	22.8
Q66K74	MAP1S	Microtubu	11	10	13	10	1059	112.1
O15305	PMM2	Phosphoma	35	9	14	8	246	28.1
E7ESC6	XPO7	Exportin-	10	10	11	10	1088	124
P26022	PTX3	Pentraxin	27	7	10	7	381	41.9
Q8TDQ7	GNPDA2	Glucosami	39	8	10	6	276	31.1
Q8IWB7	WDFY1	WD repeat	22	8	11	8	410	46.3
Q9BZE1	MRPL37	39S ribos	23	7	10	7	423	48.1
Q9NTZ6	RBM12	RNA-bindi	12	11	13	10	932	97.3
Q12788	TBL3	Transduci	15	9	11	9	808	89
E9PFH4	TNPO3	Transport	13	10	11	10	857	96.6

P48729	CSNK1A1	Casein ki	26	8	13	3	337	38.9
P51398	DAP3	28S ribos	25	9	11	9	398	45.5
O60826	CCDC22	Coiled-cc	16	8	9	8	627	70.7
Q6NYC8	PPP1R18	Phostensi	15	8	11	8	613	67.9
Q02750	MAP2K1	Dual spec	25	8	14	3	393	43.4
P49756	RBM25	RNA-bindi	12	10	12	10	843	100.1
E9PFR3	PPP2R5D	Serine/th	22	11	14	7	594	69.1
Q9Y5S1	TRPV2	Transient	13	7	10	7	764	85.9
P63173	RPL38	60S ribos	50	4	24	4	70	8.2
Q96CT7	CCDC124	Coiled-cc	45	10	13	10	223	25.8
P10606	COX5B	Cytochrom	44	6	20	6	129	13.7
Q96T51	RUFY1	RUN and F	15	10	10	9	708	79.8
P41567	EIF1	Eukaryoti	42	6	9	6	113	12.7
P53999	SUB1	Activated	51	9	17	9	127	14.4
Q15435	PPP1R7	Protein p	29	11	12	11	360	41.5
E5RJR5	SKP1	S-phase k	29	7	14	7	163	18.7
E7ESP9	NEFM	Neurofila	11	11	32	10	877	98.3
P84157	MXRA7	Matrix-re	33	6	12	4	204	21.5
P00568	AK1	Adenylate	30	7	15	6	194	21.6
Q96C36	PYCR2	Pyrroline	24	6	11	5	320	33.6
Q96N66	MBOAT7	Lysophosp	13	6	13	6	472	52.7
A0A087WY3	YTHDF3	YTH domai	16	8	13	4	588	64.5
P52815	MRPL12	39S ribos	35	6	13	1	198	21.3
P06865	HEXA	Beta-hexo	18	9	13	9	529	60.7
Q14571	ITPR2	Inositol	6	17	17	14	2701	307.9
Q9BZE4	GTPBP4	Nucleolar	16	9	11	9	634	73.9
P62834	RAP1A	Ras-relat	53	7	16	2	184	21
Q6NUQ4	TMEM214	Transmemb	16	10	13	10	689	77.1
F8W031		Uncharact	29	7	13	6	263	29.2
Q14CX7	NAA25	N-alpha-a	10	10	13	10	972	112.2
O95865	DDAH2	N(G),N(G)	33	8	11	7	285	29.6
P05362	ICAM1	Intercell	19	8	12	8	532	57.8
P19838	NFKB1	Nuclear f	11	10	10	10	968	105.3
Q95365	HLA-B	HLA class	30	8	14	1	362	40.4
O96000	NDUFB10	NADH dehy	43	7	10	7	172	20.8
O75477	ERLIN1	Erlin-1 C	24	10	15	7	346	38.9
P30740	SERPINB1	Leukocyte	25	9	11	9	379	42.7
A8MZF9	DRG2	Developme	27	8	10	8	343	38.1
A0A087WUC	SPCS2	Signal pe	36	9	17	9	227	25.1
Q9BXS5	AP1M1	AP-1 comp	25	10	15	10	423	48.6
Q9Y277	VDAC3	Voltage-d	29	8	16	7	283	30.6
Q8IY81	FTSJ3	pre-rRNA	20	12	12	12	847	96.5
A0A1B0GTG	ADSL	Adenylosu	24	8	13	8	377	42.5
P0CG39	POTEJ	POTE anky	6	5	62	2	1038	117.3
P01111	NRAS	GTPase NR	31	5	13	1	189	21.2
P02462	COL4A1	Collagen	7	9	11	9	1669	160.5
P05386	RPLP1	60S acidi	52	2	16	2	114	11.5
Q16795	NDUFA9	NADH dehy	27	9	12	9	377	42.5
P26358	DNMT1	DNA (cyto	9	14	14	14	1616	183.1
Q9NTX5	ECHDC1	Ethylmalo	30	7	9	7	307	33.7
Q15113	PCOLCE	Procollag	22	8	10	8	449	47.9

Q8IV08	PLD3	Phospholi	15	6	16	6	490	54.7
P17931	LGALS3	Galectin-	28	7	16	7	250	26.1
Q9BQ67	GRWD1	Glutamate	21	7	10	7	446	49.4
O43765	SGTA	Small glu	27	8	14	8	313	34
Q5QNY5	PEX19	Peroxisom	40	7	10	5	235	26.1
Q9NP61	ARFGAP3	ADP-ribos	22	10	12	10	516	56.9
M0R165	EPS15L1	Epidermal	12	8	11	8	756	83.4
P31949	S100A11	Protein S	52	6	35	6	105	11.7
Q9Y5X3	SNX5	Sorting n	22	7	10	6	404	46.8
P53004	BLVRA	Biliverdi	24	8	13	8	296	33.4
P42285	MTREX	Exosome R	10	11	13	11	1042	117.7
Q5T3Q7	HEATR1	HEAT repe	5	11	13	11	2063	233.1
P53582	METAP1	Methionin	24	7	10	7	386	43.2
P62330	ARF6	ADP-ribos	50	6	11	6	175	20.1
Q9Y2H6	FNDC3A	Fibronect	11	10	10	10	1198	131.8
P01112	HRAS	GTPase HR	39	6	10	2	189	21.3
P62851	RPS25	40S ribos	36	7	21	7	125	13.7
Q9BVK6	TMED9	Transmemb	28	6	13	6	235	27.3
P49790	NUP153	Nuclear p	8	9	11	9	1475	153.8
Q9P258	RCC2	Protein R	23	12	13	12	522	56
P00966	ASS1	Argininos	20	8	14	8	412	46.5
O60869	EDF1	Endotheli	45	9	19	9	148	16.4
P13807	GYS1	Glycogen	13	8	10	8	737	83.7
P15153	RAC2	Ras-relat	34	7	16	2	192	21.4
Q96RS6	NUDCD1	NudC doma	19	12	14	12	583	66.7
J3KR97	TBCD	Tubulin-s	10	11	12	11	1230	136.5
F8VYN9	ARL1	ADP-ribos	24	4	7	4	194	21.8
A0A087WXS	ASNA1	ATPase AS	24	6	12	4	331	37.1
Q13155	AIMP2	Aminoacyl	26	9	15	9	320	35.3
Q15555	MAPRE2	Microtubu	28	8	11	6	327	37
Q9NR50	EIF2B3	Translati	26	11	12	11	452	50.2
P51610	HCFC1	Host cell	6	10	12	10	2035	208.6
Q9UP95	SLC12A4	Solute ca	10	10	13	10	1085	120.6
Q9HD26	GOPC	Golgi-ass	19	10	13	10	462	50.5
O60462	NRP2	Neuropili	12	11	12	11	931	104.8
G5E9D5	ELAC2	ElaC homo	14	9	12	9	807	90
Q13564	NAE1	NEDD8-act	20	8	9	8	534	60.2
P28300	LOX	Protein-l	24	7	9	6	417	46.9
G3V203	RPL18	60S ribos	34	5	17	5	164	18.7
F2Z2Y4	PDXK	Pyridoxal	26	7	11	7	272	30.6
O94760	DDAH1	N(G),N(G)	36	7	9	6	285	31.1
Q9Y6K9	IKBKG	NF-kappa-	21	8	10	8	419	48.2
Q8WXF7	ATL1	Atlastin-	22	10	11	10	558	63.5
Q0VDF9	HSPA14	Heat shoc	22	11	13	11	509	54.8
E7ENY0	ADD1	Alpha-add	15	8	10	8	663	73.4
Q9H2U2	PPA2	Inorganic	31	9	12	8	334	37.9
Q9HB07	C12orf10	UPF0160 p	27	9	13	9	376	42.4
O15269	SPTLC1	Serine pa	21	11	12	11	473	52.7
O75400	PRPF40A	Pre-mRNA-	10	11	13	11	957	108.7
A0A087WUQ	GPX1	Glutathio	38	6	11	6	202	21.9
A0A2R8Y85	SMARCE1	SWI/SNF-r	29	11	13	10	367	41.8

Q08209	PPP3CA	Serine/th	17	8	10	3	521	58.7
Q03135	CAV1	Caveolin-	46	7	16	7	178	20.5
Q12996	CSTF3	Cleavage	17	9	10	9	717	82.9
P61254	RPL26	60S ribos	41	9	22	2	145	17.2
Q9UJW0	DCTN4	Dynactin	23	9	14	9	460	52.3
Q6UW63	KDELC1	KDEL moti	23	11	11	10	502	58
P14324	FDPS	Farnesyl	14	6	16	6	419	48.2
Q9H7D0	DOCK5	Dedicator	7	13	13	12	1870	215.2
Q92791	P3H4	Endoplasm	25	9	13	9	437	50.3
Q9UBF2	COPG2	Coatomer	13	9	12	7	871	97.6
Q9BZK7	TBL1XR1	F-box-lik	17	7	7	7	514	55.6
Q99436	PSMB7	Proteasom	33	7	13	7	277	29.9
Q16629	SRSF7	Serine/ar	31	8	17	7	238	27.4
Q9Y3U8	RPL36	60S ribos	36	6	20	6	105	12.2
H3BN98		Uncharact	23	6	25	2	237	27.2
O15400	STX7	Syntaxin-	36	8	12	8	261	29.8
Q9Y5Z4	HEBP2	Heme-binc	41	8	12	8	205	22.9
Q9Y2G5	POFUT2	GDP-fucos	24	13	18	6	429	49.9
P26583	HMGB2	High mobi	34	10	15	8	209	24
O75694	NUP155	Nuclear p	7	8	12	8	1391	155.1
E7ENQ1	MAP4K4	Mitogen-a	10	10	11	8	1154	132.1
Q13630	TSTA3	GDP-L-fuc	31	9	14	9	321	35.9
Q02978	SLC25A11	Mitochonc	33	9	12	9	314	34
P51116	FXR2	Fragile X	15	9	10	6	673	74.2
O94804	STK10	Serine/th	13	12	12	11	968	112.1
O15212	PFDN6	Prefoldir	50	8	11	8	129	14.6
Q9HD15	SRA1	Steroid r	31	6	10	6	236	25.7
K7ERE3	KRT13	Keratin,	16	7	15	1	415	45.2
Q01085	TIAL1	Nucleolys	19	6	10	4	375	41.6
Q15642	TRIP10	Cdc42-int	19	11	12	10	601	68.3
F5H442	TSG101	Tumor sus	22	7	9	7	365	40.9
Q96CS3	FAF2	FAS-assoc	18	7	9	7	445	52.6
P62244	RPS15A	40S ribos	61	8	27	4	130	14.8
Q9NZ08	ERAP1	Endoplasm	11	10	11	10	941	107.2
Q9UN86	G3BP2	Ras GTPas	21	10	13	9	482	54.1
O95299	NDUFA10	NADH dehy	17	7	10	7	355	40.7
P62995	TRA2B	Transform	27	6	10	6	288	33.6
O43395	PRPF3	U4/U6 sma	19	12	12	12	683	77.5
O43854	EDIL3	EGF-like	22	9	11	9	480	53.7
Q14914	PTGR1	Prostagla	29	9	11	9	329	35.8
P51452	DUSP3	Dual spec	31	4	9	4	185	20.5
Q9Y3C6	PPIL1	Peptidyl-	42	7	9	7	166	18.2
Q9ULH1	ASAP1	Arf-GAP w	8	8	9	8	1129	125.4
Q9NYU1	UGGT2	UDP-glucc	7	11	12	9	1516	174.6
Q8N556	AFAP1	Actin fil	14	9	11	9	730	80.7
E9PSI1		Transmemb	14	9	9	3	815	92.3
P46778	RPL21	60S ribos	36	8	23	8	160	18.6
Q14C86	GAPVD1	GTPase-ac	7	9	10	9	1478	164.9
Q04323	UBXN1	UBX domai	28	6	9	6	297	33.3
Q9BYD6	MRPL1	39S ribos	19	7	10	7	325	36.9
P12955	PEPD	Xaa-Pro d	17	8	11	8	493	54.5

P30048	PRDX3	Thioredox	25	4	14	4	256	27.7
P84090	ERH	Enhancer	71	6	17	6	104	12.3
Q8IWJ2	GCC2	GRIP and	7	11	11	11	1684	195.8
O60884	DNAJA2	DnaJ homc	24	10	14	10	412	45.7
Q8TBC4	UBA3	NEDD8-act	27	9	9	9	463	51.8
A0A1P0AYU	SFXN3	Sideroflε	28	7	8	7	325	36
P63096	GNAI1	Guanine n	22	8	12	1	354	40.3
Q9UN37	VPS4A	Vacuolar	22	10	14	6	437	48.9
A0A1W2PPZ	TCEA1	Transcrip	30	9	14	9	301	33.9
A0A0U1RQL	GSN	Gelsolin	28	6	13	1	232	26.3
Q7Z4H8	KDELC2	KDEL moti	25	10	10	10	507	58.5
P53367	ARFIP1	Arfaptin-	23	11	12	11	373	41.7
P46776	RPL27A	60S ribos	35	6	17	6	148	16.6
Q9UHN6	TMEM2	Cell surf	8	10	10	10	1383	154.3
Q8TCJ2	STT3B	Dolichyl-	11	11	16	9	826	93.6
Q9NZL9	MAT2B	Methionir	24	8	11	8	334	37.5
P08572	COL4A2	Collagen	6	8	9	8	1712	167.4
C9JYJ6	FILIP1L	Filamin A	12	10	10	10	837	96.2
Q96IJ6	GMPPA	Mannose-1	21	8	11	8	420	46.3
A0A0A6YYA	TMED7-TIC	Protein T	32	7	12	7	188	21.2
Q9HCE1	MOV10	Putative	13	11	11	11	1003	113.6
Q13637	RAB32	Ras-relat	30	6	11	5	225	25
P43121	MCAM	Cell surf	14	8	11	8	646	71.6
P31689	DNAJA1	DnaJ homc	21	8	11	8	397	44.8
P11172	UMPS	Uridine 5	20	11	14	11	480	52.2
P61326	MAGOH	Protein m	49	7	14	7	146	17.2
Q5T0I0	GSN	Gelsolin	22	6	14	1	260	28.9
Q9NVJ2	ARL8B	ADP-ribos	36	6	10	3	186	21.5
M0R0R2	RPS5	40S ribos	27	7	19	7	225	25.3
Q9NTJ3	SMC4	Structura	8	11	11	11	1288	147.1
O00622	CYR61	Protein C	27	10	10	10	381	42
I3L0A0	TMEM189-U	HCG204478	20	7	36	3	370	42.2
S4R3N1	HSPE1-MOE	HSPE1-MOE	35	9	19	5	261	29.7
P63167	DYNLL1	Dynein li	45	3	14	2	89	10.4
M0R026	ILVBL	Acetolact	20	8	9	8	525	56.7
I3L4X2	ABCC1	Multidrug	8	12	13	12	1440	160.4
Q86TU7	SETD3	Histone-l	15	8	10	8	594	67.2
Q15043	SLC39A14	Zinc tran	11	5	10	5	492	54.2
O95881	TXNDC12	Thioredox	35	6	12	6	172	19.2
Q15437	SEC23B	Protein t	8	4	9	2	767	86.4
A0A0A0MSI	EXOC5	Exocyst c	14	10	12	9	711	82.1
Q07021	C1QBP	Complemer	19	4	8	4	282	31.3
P46734	MAP2K3	Dual spec	29	9	11	9	347	39.3
Q13547	HDAC1	Histone d	20	8	10	5	482	55.1
Q96EY7	PTCD3	Pentatric	15	8	10	8	689	78.5
E9PGT6	COPS8	COP9 sigr	36	4	10	4	173	19.3
Q9H6S3	EPS8L2	Epidermal	14	8	10	8	715	80.6
Q9P016	THYN1	Thymocyte	42	10	12	10	225	25.7
Q8NC56	LEMD2	LEM domai	17	8	9	8	503	56.9
O94919	ENDOD1	Endonucle	22	7	8	7	500	55
Q6VEQ5	WASH2P	WAS prote	17	7	9	7	465	50.3

P51532	SMARCA4	Transcrip	6	10	11	5	1647	184.5
E9PB61	ALYREF	THO compl	27	6	9	6	264	27.5
C9J0J7	PFN2	Profilin	60	5	13	3	91	9.8
P06132	UROD	Uroporphy	24	5	9	5	367	40.8
P10253	GAA	Lysosomal	11	7	10	7	952	105.3
P62316	SNRPD2	Small nuc	62	7	22	7	118	13.5
E9PNQ8	THY1	Thy-1 mem	24	3	16	3	165	18.2
Q9H2J4	PDCL3	Phosducin	28	6	11	6	239	27.6
F6UJY9	HYI	Putative	44	6	10	6	213	23.1
Q969H8	MYDGF	Myeloid-d	27	5	16	5	173	18.8
Q96CG8	CTHRC1	Collagen	23	5	16	5	243	26.2
P28070	PSMB4	Proteasom	29	5	13	5	264	29.2
Q09161	NCBP1	Nuclear c	10	8	9	8	790	91.8
Q9NW13	RBM28	RNA-bindi	15	11	12	11	759	85.7
A0A0A6YYJMACF1		Microtubu	5	9	11	1	1668	177.2
P26885	FKBP2	Peptidyl-	49	6	14	6	142	15.6
O15067	PFAS	Phosphori	8	11	11	11	1338	144.6
F5GWT4	WNK1	Serine/th	5	8	9	8	2134	225.4
P10319	HLA-B	HLA class	25	6	9	1	362	40.3
P36543	ATP6V1E1	V-type pr	32	8	13	8	226	26.1
Q9UIQ6	LNPEP	Leucyl-cy	10	9	9	9	1025	117.3
Q15717	ELAVL1	ELAV-like	21	6	11	6	326	36.1
Q13610	PWP1	Periodic	19	8	9	8	501	55.8
Q13190	STX5	Syntaxin-	24	8	9	8	355	39.6
O95292	VAPB	Vesicle-a	26	7	14	6	243	27.2
Q9UMS4	PRPF19	Pre-mRNA-	21	8	13	8	504	55.1
Q9Y512	SAMM50	Sorting a	21	9	11	9	469	51.9
O75179	ANKRD17	Ankyrin r	4	8	9	4	2603	274.1
Q92747	ARPC1A	Actin-rel	22	8	13	8	370	41.5
O00629	KPNA4	Importin	17	8	18	4	521	57.9
Q5JVZ5	ELMO2	Engulfmen	14	8	10	8	718	82.3
Q15738	NSDHL	Sterol-4-	24	9	10	9	373	41.9
O15498	YKT6	Synaptobr	40	9	14	9	198	22.4
O14787	TNPO2	Transport	9	8	12	4	897	101.3
O00267	SUPT5H	Transcrip	9	9	11	9	1087	120.9
Q9UHD9	UBQLN2	Ubiquilin	14	6	11	4	624	65.7
Q9Y673	ALG5	Dolichyl-	26	6	10	6	324	36.9
P60866	RPS20	40S ribos	36	6	18	6	119	13.4
P69905	HBA1	Hemoglobi	63	6	17	6	142	15.2
Q08945	SSRP1	FACT comp	14	8	12	8	709	81
Q3LXA3	TKFC	Triokinas	24	9	10	9	575	58.9
P35080	PFN2	Profilin-	31	4	10	2	140	15
G3V3A4	SNW1	SNW domai	19	9	9	9	571	65.4
Q14696	MESD	LRP chape	32	8	16	8	234	26.1
Q9BQ61	TRIR	Telomeras	34	5	10	5	176	18.4
P47985	UQCRFS1	Cytochrom	30	8	12	8	274	29.6
Q9BVP2	GNL3	Guanine n	21	8	11	8	549	62
Q9Y5X1	SNX9	Sorting n	15	8	11	8	595	66.6
P56945	BCAR1	Breast ca	11	9	10	9	870	93.3
Q9Y5V3	MAGED1	Melanoma-	12	9	12	8	778	86.1
P50452	SERPINB8	Serpin B8	25	9	13	8	374	42.7

F8W6C2	SPATS2L	SPATS2-li	35	8	9	1	265	29.9
Q9UNX3	RPL26L1	60S ribos	34	8	17	1	145	17.2
P35573	AGL	Glycogen	6	10	10	10	1532	174.7
P62633	CNBP	Cellular	41	6	12	6	177	19.5
P05106	ITGB3	Integrin	12	8	9	8	788	87
O60502	MGEA5	Protein O	12	10	12	10	916	102.8
P19022	CDH2	Cadherin-	12	6	9	6	906	99.7
Q15819	UBE2V2	Ubiquitin	51	7	33	3	145	16.4
A0A2R8Y5F	HARS2	Probable	17	9	12	3	512	57.5
Q15637	SF1	Splicing	11	6	9	6	639	68.3
P62318	SNRPD3	Small nuc	48	4	12	4	126	13.9
P46976	GYG1	Glycogeni	18	6	8	6	350	39.4
E9PQ57	RAE1	mRNA expo	23	9	10	9	437	47.8
Q5T4U5	ACADM	Acyl-Coen	23	9	11	9	454	50.2
Q0IIM8	TBC1D8B	TBC1 doma	9	9	10	9	1120	128.6
Q9UKD2	MRTO4	mRNA turn	36	8	11	8	239	27.5
Q9H4A6	GOLPH3	Golgi pho	33	9	11	9	298	33.8
P07205	PGK2	Phosphogl	14	6	20	2	417	44.8
F8VXU5	VPS29	Vacuolar	31	7	14	7	214	24
Q96BM9	ARL8A	ADP-ribos	39	5	8	2	186	21.4
A0A024R44	DNPEP	Aspartyl	18	8	10	8	471	52
P46109	CRKL	Crk-like	28	8	13	8	303	33.8
Q9NZD2	GLTP	Glycolipi	27	6	9	6	209	23.8
P16104	H2AFX	Histone H	48	7	26	3	143	15.1
P50453	SERPINB9	Serpin B9	29	9	11	8	376	42.4
Q4J6C6	PREPL	Prolyl en	13	9	9	9	727	83.9
H7BXH2	PPP6R3	Serine/th	10	7	10	7	827	92.4
J3KNQ4	PARVA	Alpha-par	16	7	15	6	412	46.6
Q5T0F9	CC2D1B	Coiled-co	15	11	11	11	858	94.2
H0Y8B3	AHCYL2	Adenosylh	18	9	12	1	518	58.1
Q5SWX3	CAMK2G	Calcium/c	16	8	9	6	516	57.8
P35354	PTGS2	Prostagla	20	10	12	10	604	69
Q12792	TWF1	Twinfilin	20	7	13	4	350	40.3
Q15363	TMED2	Transmemb	28	6	21	6	201	22.7
Q9NPQ8	RIC8A	Synembryn	20	8	10	7	531	59.7
Q9UIG0	BAZ1B	Tyrosine-	7	9	9	9	1483	170.8
Q9Y608	LRRFIP2	Leucine-r	8	6	7	5	721	82.1
P35240	NF2	Merlin OS	16	8	9	8	595	69.6
D6RBQ9	HNRNPD	Heterogen	41	7	24	1	155	15.6
C9JFR7	CYCS	Cytochrom	41	5	14	5	101	11.3
P29373	CRABP2	Cellular	56	6	11	6	138	15.7
Q9Y3B7	MRPL11	39S ribos	24	4	8	4	192	20.7
Q5RKV6	EXOSC6	Exosome c	36	8	10	8	272	28.2
P58335	ANTXR2	Anthrax t	18	8	15	7	489	53.6
O15145	ARPC3	Actin-rel	23	5	14	5	178	20.5
Q12841	FSTL1	Follistat	24	8	14	8	308	35
Q9Y383	LUC7L2	Putative	19	7	10	5	392	46.5
P30154	PPP2R1B	Serine/th	13	7	11	2	601	66.2
P15586	GNS	N-acetylg	16	7	11	7	552	62
Q08380	LGALS3BP	Galectin-	15	7	11	7	585	65.3
O95487	SEC24B	Protein t	7	7	11	6	1268	137.3

Q99614	TTC1	Tetratric	21	7	7	7	292	33.5
Q9UK41	VPS28	Vacuolar	41	8	11	8	221	25.4
Q9Y6B6	SAR1B	GTP-bindi	29	6	13	3	198	22.4
O75569	PRKRA	Interferc	23	7	9	7	313	34.4
Q9BQE5	APOL2	Apolipopr	28	10	10	10	337	37.1
C9J5C3	PDCD10	Programme	28	5	7	5	202	23.6
F5H5V4	PSMD9	26S prote	42	6	12	6	153	16.9
Q9NX46	ADPRHL2	Poly(ADP-	21	7	9	7	363	38.9
Q71UI9	H2AFV	Histone H	31	4	23	2	128	13.5
M0QXD6	GTF2F1	General t	23	8	11	8	433	48.6
Q9Y5K5	UCHL5	Ubiquitin	26	7	9	7	329	37.6
O15260	SURF4	Surfeit l	21	5	17	5	269	30.4
Q96KP1	EXOC2	Exocyst c	12	11	11	11	924	104
Q16740	CLPP	ATP-depen	39	8	10	8	277	30.2
Q9Y2W2	WBP11	WW domain	13	7	10	7	641	70
Q8NI27	THOC2	THO compl	7	11	11	11	1593	182.7
P61006	RAB8A	Ras-relat	32	7	13	3	207	23.7
Q96Q11	TRNT1	CCA tRNA	23	9	9	9	434	50.1
Q96DI7	SNRNP40	U5 small	27	7	7	7	357	39.3
Q9H488	POFUT1	GDP-fucos	20	7	12	7	388	43.9
Q5VT25	CDC42BPA	Serine/th	5	9	9	5	1732	197.2
A0A087X0R	SNX12	Sorting n	38	8	14	6	172	19.8
O15270	SPTLC2	Serine pa	14	7	11	7	562	62.9
M0QXM4	SLC1A5	Amino aci	17	5	11	5	365	39.4
O94903	PLPBP	Pyridoxal	25	7	10	7	275	30.3
O14976	GAK	Cyclin-G-	7	8	9	8	1311	143.1
Q9BTT0	ANP32E	Acidic le	18	4	7	4	268	30.7
P19784	CSNK2A2	Casein ki	21	7	10	7	350	41.2
H7BXY3	DHX30	Putative	8	9	9	9	1166	130.5
Q9BVJ6	UTP14A	U3 small	13	9	11	9	771	87.9
P29353	SHC1	SHC-trans	16	7	9	7	583	62.8
Q9BVG4	PBDC1	Protein P	33	7	9	7	233	26
O43293	DAPK3	Death-ass	16	6	8	6	454	52.5
P28288	ABCD3	ATP-bindi	12	8	12	8	659	75.4
A0A286YFF	MON2	Protein M	5	8	9	8	1718	190.4
A0A1W2PPT	POLR2B	DNA-direc	8	9	10	9	1099	125.1
Q5SQP8	CTBP2	C-termina	17	9	13	5	513	56.1
A6PVN5	PTPA	Serine/th	28	9	14	9	329	37.4
Q13363	CTBP1	C-termina	19	8	13	4	440	47.5
P43897	TSFM	Elongatic	20	6	10	6	325	35.4
Q9HAV7	GRPEL1	GrpE prot	28	6	11	6	217	24.3
A0A096LNZ	ISG15	Ubiquitin	48	6	9	6	143	15.6
Q15386	UBE3C	Ubiquitin	8	9	9	9	1083	123.8
Q9Y6D6	ARFGEF1	Brefeldin	5	8	9	3	1849	208.6
O15294	OGT	UDP-N-ace	11	9	9	9	1046	116.9
O60504	SORBS3	Vinexin O	15	9	10	9	671	75.3
P56199	ITGA1	Integrin	8	10	11	10	1179	130.8
Q12769	NUP160	Nuclear p	6	8	8	8	1436	162
Q9H307	PNN	Pinin OS=	12	10	10	10	717	81.6
H7C3P7	RALA	Ras-relat	29	4	9	3	164	18.4
A0A0A0MQX	MBNL1	Musclebli	19	6	12	1	400	43

Q5F2F8	PPP3CB	Serine/th	16	7	9	2	496	56
P42766	RPL35	60S ribos	46	7	20	7	123	14.5
O00264	PGRMC1	Membrane-	25	8	13	7	195	21.7
P21810	BGN	Biglycan	20	6	8	5	368	41.6
Q08623	PUDP	Pseudouri	26	5	8	5	228	25.2
O75695	RP2	Protein X	18	8	12	8	350	39.6
P24539	ATP5F1	ATP synth	26	8	11	8	256	28.9
Q9NW15	ANO10	Anoctamin	9	6	10	6	660	76.3
P61513	RPL37A	60S ribos	59	5	15	5	92	10.3
Q9BZG1	RAB34	Ras-relat	25	6	8	6	259	29
P07099	EPHX1	Epoxide h	20	9	12	9	455	52.9
Q96DZ1	ERLEC1	Endoplasm	21	11	13	11	483	54.8
P52294	KPNA1	Importin	17	9	12	5	538	60.2
Q9H9J2	MRPL44	39S ribos	24	7	9	7	332	37.5
Q9NR31	SAR1A	GTP-bindi	29	7	16	4	198	22.4
Q52LJ0	FAM98B	Protein F	22	6	7	6	330	37.2
Q9P2R3	ANKFY1	Rabankyri	7	7	8	7	1169	128.3
A0A140TA6	KRT34	Keratin,	18	8	9	8	436	49.4
O00186	STXBP3	Syntaxin-	11	7	11	7	592	67.7
Q8TD19	NEK9	Serine/th	8	7	8	7	979	107.1
P29590	PML	Protein P	12	10	11	10	882	97.5
P35658	NUP214	Nuclear p	5	9	10	9	2090	213.5
Q7Z4V5	HDGFL2	Hepatoma-	10	7	13	6	671	74.3
P61764	STXBP1	Syntaxin-	15	7	9	7	594	67.5
Q00653	NFKB2	Nuclear f	11	10	10	10	900	96.7
A0A2R8Y5Q	TBCE	Tubulin-s	17	9	9	9	549	61.8
Q9Y4C2	TCAF1	TRPM8 cha	8	7	9	7	921	102.1
Q01968	OCRL	Inositol	11	9	9	9	901	104.1
P49207	RPL34	60S ribos	42	6	30	6	117	13.3
Q96EP5	DAZAP1	DAZ-assoc	20	6	9	6	407	43.4
Q5T6V5	C9orf64	Queuosine	19	6	10	6	341	39
Q13243	SRSF5	Serine/ar	22	6	12	5	272	31.2
Q7L5N7	LPCAT2	Lysophosp	13	8	10	8	544	60.2
P06493	CDK1	Cyclin-de	33	8	9	6	297	34.1
D6RBW1	EIF4E	Eukaryoti	24	6	12	6	245	28.5
G3V4T7	FRMD6	FERM doma	16	7	8	7	545	62.8
E9PHV5	SSFA2	Sperm-spe	8	9	10	9	1237	136.1
Q53H82	LACTB2	Endoribon	31	7	7	7	288	32.8
O75962	TRIO	Triple fu	3	9	10	9	3097	346.7
A0A2R8Y85	CUX1	Homeobox	6	9	10	2	1460	159.3
A0A2R8Y5A	ATXN2	Ataxin-2	8	9	10	8	1166	125.9
P46013	MKI67	Prolifera	4	8	8	8	3256	358.5
P35625	TIMP3	Metallopr	32	7	17	7	211	24.1
O14562	UBFD1	Ubiquitin	21	6	11	6	309	33.4
P48723	HSPA13	Heat shoc	17	8	10	8	471	51.9
Q14699	RFTN1	Raftlin O	18	10	10	10	578	63.1
Q15185	PTGES3	Prostagla	39	6	18	6	160	18.7
P60903	S100A10	Protein S	28	4	22	4	97	11.2
P62854	RPS26	40S ribos	37	4	14	4	115	13
Q9Y3D6	FIS1	Mitochond	29	5	10	5	152	16.9
Q96S52	PIGS	GPI trans	14	7	9	7	555	61.6

Q9H3N1	TMX1	Thioredox	22	7	12	7	280	31.8
O95630	STAMBP	STAM-bind	20	8	9	8	424	48
O75934	BCAS2	Pre-mRNA-	34	7	8	7	225	26.1
Q14353	GAMT	Guanidinc	31	5	7	5	236	26.3
P58546	MTPN	Myotrophi	57	5	9	5	118	12.9
Q92783	STAM	Signal tr	17	8	9	8	540	59.1
O94875	SORBS2	Sorbin an	9	7	9	7	1100	124
J3QRU1	YES1	Tyrosine-	15	7	9	5	548	61.3
P16435	POR	NADPH--cy	12	7	11	7	677	76.6
P82094	TMF1	TATA elem	9	8	8	8	1093	122.8
Q15836	VAMP3	Vesicle-a	40	3	9	1	100	11.3
Q9Y2L1	DIS3	Exosome c	10	9	10	9	958	108.9
Q13867	BLMH	Bleomycin	16	6	8	6	455	52.5
Q99584	S100A13	Protein S	48	5	14	5	98	11.5
Q969S3	ZNF622	Zinc fing	21	9	10	9	477	54.2
A0A087WVIPDLIM2		PDZ and L	21	6	10	6	325	34.7
P61758	VBP1	Prefoldin	37	7	14	7	197	22.6
H0YL72	IDH3A	Isocitrat	17	6	10	6	331	35.8
P27105	STOM	Erythrocy	28	7	8	7	288	31.7
O95218	ZRANB2	Zinc fing	20	6	10	6	330	37.4
P68871	HBB	Hemoglobi	38	5	15	5	147	16
A0A087WYRSRP19		Signal re	44	4	7	4	120	13.6
Q9NTI5	PDS5B	Sister ch	6	8	9	7	1447	164.6
Q9Y2V7	COG6	Conserved	14	9	10	9	657	73.2
P09012	SNRPA	U1 small	33	7	9	6	282	31.3
Q9UBU9	NXF1	Nuclear R	10	6	8	6	619	70.1
E9PR44	CRYAB	Alpha-cry	30	5	16	5	174	20
P54725	RAD23A	UV excisi	19	7	12	5	363	39.6
Q99848	EBNA1BP2	Probable	23	8	11	8	306	34.8
Q05209	PTPN12	Tyrosine-	11	8	8	8	780	88.1
Q8N684	CPSF7	Cleavage	19	8	11	8	471	52
Q6UWE0	LRSAM1	E3 ubiqui	14	9	9	9	723	83.5
P46108	CRK	Adapter m	25	6	8	6	304	33.8
Q9BWS9	CHID1	Chitinase	16	5	7	5	393	44.9
P47914	RPL29	60S ribos	24	5	18	5	159	17.7
Q8TC12	RDH11	Retinol d	23	7	8	7	318	35.4
P42025	ACTR1B	Beta-cent	22	8	10	2	376	42.3
P21912	SDHB	Succinate	28	7	12	7	280	31.6
Q96M27	PRRC1	Protein P	14	6	11	6	445	46.7
P60763	RAC3	Ras-relat	27	5	16	1	192	21.4
O43818	RRP9	U3 small	20	9	10	9	475	51.8
Q96EM0	L3HYPDH	Trans-3-h	27	7	8	7	354	38.1
P22090	RPS4Y1	40S ribos	21	7	12	2	263	29.4
P26440	IVD	Isovalery	19	8	9	8	423	46.3
Q96D46	NMD3	60S ribos	17	8	9	8	503	57.6
Q9UBM7	DHCR7	7-dehydrc	14	6	11	6	475	54.5
Q15036	SNX17	Sorting n	14	7	8	7	470	52.9
K7ELP0	TPM4	Tropomyos	57	6	17	1	69	8
Q8IWU6	SULF1	Extracell	10	9	11	9	871	101
Q8TBA6	GOLGA5	Golgin su	13	9	9	9	731	83
P48507	GCLM	Glutamate	27	6	7	6	274	30.7

P23919	DTYMK	Thymidyla	27	6	10	6	212	23.8
Q16204	CCDC6	Coiled-cc	19	8	9	8	474	53.3
P18077	RPL35A	60S ribos	52	7	19	7	110	12.5
P29218	IMPA1	Inositol	25	7	8	7	277	30.2
Q96C86	DCPS	m7GpppX d	30	8	10	8	337	38.6
C9J381	IMPDH1	Inosine-5	15	7	7	5	513	55.2
P50402	EMD	Emerin OS	23	7	10	7	254	29
P11234	RALB	Ras-relat	25	4	7	3	206	23.4
Q9BRX2	PELO	Protein p	21	7	10	7	385	43.3
O75663	TIPRL	TIP41-lik	28	8	14	8	272	31.4
J3QRU4	VAMP2	Vesicle-a	35	3	7	1	113	12.2
O14745	SLC9A3R1	Na(+)/H(+	27	8	10	8	358	38.8
O60925	PFDN1	Prefoldir	42	7	12	7	122	14.2
O95361	TRIM16	Tripartit	20	9	10	9	564	63.9
P51570	GALK1	Galactoki	19	6	10	6	392	42.2
P36551	CPOX	Oxygen-de	17	6	9	6	454	50.1
Q92820	GGH	Gamma-glu	18	7	9	7	318	35.9
Q04837	SSBP1	Single-st	44	5	7	5	148	17.2
P49720	PSMB3	Proteasom	35	5	10	5	205	22.9
Q5TFE4	NT5DC1	5'-nuclec	18	8	10	8	455	51.8
P33240	CSTF2	Cleavage	16	8	10	3	577	60.9
Q15369	ELOC	Elongin-C	47	4	7	4	112	12.5
P24666	ACP1	Low molec	53	5	9	5	158	18
H7C4T5	MBNL1	Musclebli	24	6	11	1	329	35.8
Q96CN7	ISOC1	Isochoris	28	6	6	6	298	32.2
O75935	DCTN3	Dynactin	32	8	12	8	186	21.1
Q9Y2U8	LEMD3	Inner nuc	8	7	9	7	911	99.9
P82933	MRPS9	28S ribos	22	7	7	7	396	45.8
P49888	SULT1E1	Estrogen	27	8	11	8	294	35.1
D6RA82	ANXA3	Annexin (	23	6	7	6	284	32.1
O43633	CHMP2A	Charged m	34	8	11	8	222	25.1
J3KNI1	COG4	Conservec	11	7	7	7	789	89.4
F5H479	LAMTOR1	Ragulator	49	4	7	4	99	11.1
Q9NRR5	UBQLN4	Ubiquilir	13	6	8	5	601	63.8
P43307	SSR1	Transloco	21	4	11	4	286	32.2
O96008	TOMM40	Mitochond	22	6	11	6	361	37.9
A0A024R21	HDGFRP3	Hepatoma-	24	5	11	4	203	22.6
B1ALA9	PRPS1	Ribose-ph	29	8	11	5	285	31.4
F8W9X7	CCDC93	Coiled-cc	16	9	9	9	630	73
Q5JTH9	RRP12	RRP12-lik	8	10	10	10	1297	143.6
P04080	CSTB	Cystatin-	77	6	12	6	98	11.1
P32321	DCTD	Deoxycyti	45	6	6	6	178	20
B4DLR8	NQO1	NAD(P)H d	33	6	9	6	202	22.8
Q99707	MTR	Methionir	6	7	8	7	1265	140.4
P98175	RBM10	RNA-bindi	9	8	8	8	930	103.5
Q8NB90	SPATA5	Spermatog	9	6	11	5	893	97.8
P46926	GNPDA1	Glucosami	24	6	8	4	289	32.6
Q6P587	FAHD1	Acylpyruv	38	6	10	6	224	24.8
E9PL57	NEDD8-MDF	NEDD8-MDF	28	5	22	5	170	19.5
A0A1W2PNW	STAT6	Signal tr	10	8	9	8	679	76
P36507	MAP2K2	Dual spec	17	7	11	2	400	44.4

P54619	PRKAG1	5'-AMP-ac	23	7	8	7	331	37.6
Q8IUE6	HIST2H2AE	Histone H	47	5	18	2	130	14
O43896	KIF1C	Kinesin-l	8	8	8	5	1103	122.9
Q8N3D4	EHBP1L1	EH domain	6	8	8	7	1523	161.8
P50416	CPT1A	Carnitine	13	9	10	9	773	88.3
O14908	GIPC1	PDZ domai	18	6	9	6	333	36
G5E9L0	ARFGAP2	ADP-ribos	18	9	10	9	493	53.4
Q08752	PPID	Peptidyl-	20	8	9	7	370	40.7
Q9NWV4	C1orf123	UPF0587 p	41	6	7	6	160	18
Q2NL82	TSR1	Pre-rRNA-	14	8	9	8	804	91.8
Q5VVC8	RPL11	60S ribos	27	5	18	5	167	19
P45973	CBX5	Chromobox	30	5	7	5	191	22.2
Q9NUQ8	ABCF3	ATP-bindi	10	8	11	8	709	79.7
Q96DB5	RMDN1	Regulator	22	6	8	6	314	35.8
Q9Y6W5	WASF2	Wiskott-A	16	8	8	8	498	54.3
O14656	TOR1A	Torsin-1A	18	5	8	5	332	37.8
A0A182DWF	TXNRD2	Thioredox	10	4	8	3	494	53.5
Q9BRA2	TXNDC17	Thioredox	51	5	11	5	123	13.9
Q96DG6	CMBL	Carboxyme	27	7	10	7	245	28
Q9UHY7	ENOPH1	Enolase-p	27	5	9	5	261	28.9
Q14694	USP10	Ubiquitin	8	5	7	5	798	87.1
H0YAN8	ARHGEF10	Rho guani	6	5	8	5	988	109.6
Q9BPW8	NIPSNAP1	Protein N	21	5	10	4	284	33.3
P39748	FEN1	Flap endo	22	7	8	7	380	42.6
Q969X5	ERGIC1	Endoplasm	18	5	11	5	290	32.6
F8WE88	MYO5A	Unconvent	6	10	10	10	1855	215.2
Q8WUH6	TMEM263	Transmemb	49	4	7	4	116	11.7
Q9H4G4	GLIPR2	Golgi-ass	38	5	7	5	154	17.2
P05026	ATP1B1	Sodium/po	25	7	10	7	303	35
O75326	SEMA7A	Semaphori	11	5	7	5	666	74.8
Q8IYI6	EXOC8	Exocyst c	12	9	9	9	725	81.7
Q709C8	VPS13C	Vacuolar	2	7	7	7	3753	422.1
Q8N183	NDUFAF2	NADH dehy	33	6	10	6	169	19.8
O43813	LANCL1	LanC-like	23	8	8	8	399	45.3
P19971	TYMP	Thymidine	18	6	8	6	482	49.9
Q8IX12	CCAR1	Cell divi	7	8	9	8	1150	132.7
Q99598	TSNAX	Translin-	23	6	9	6	290	33.1
Q8NBJ7	SUMF2	Inactive	21	5	8	5	301	33.8
Q92614	MYO18A	Unconvent	5	10	10	10	2054	233
Q9BZV1	UBXN6	UBX domai	18	5	6	5	441	49.7
Q13501	SQSTM1	Sequestos	15	4	8	4	440	47.7
Q68CQ7	GLT8D1	Glycosylt	23	8	9	8	371	41.9
Q9Y4W2	LAS1L	Ribosomal	11	7	8	7	734	83
P49585	PCYT1A	Choline-p	21	8	15	8	367	41.7
Q9Y3T9	NOC2L	Nucleolar	8	6	9	6	749	84.9
Q93034	CUL5	Cullin-5	12	9	10	9	780	90.9
Q13907	IDI1	Isopenten	23	4	6	4	227	26.3
Q8IZ07	ANKRD13A	Ankyrin r	15	6	6	6	590	67.6
Q86Y56	DNAAF5	Dynein as	13	9	9	9	855	93.5
Q9NS69	TOMM22	Mitochond	34	4	6	4	142	15.5
P35613	BSG	Basigin (	14	6	17	6	385	42.2

Q9C0C9	UBE2O	(E3-indep	7	7	7	7	1292	141.2
D6RBR1	CAST	Calpastat	50	6	10	1	150	15.5
B0QZ18	CPNE1	Copine-1	10	6	12	6	542	59.7
Q8TED1	GPX8	Probable	31	7	11	7	209	23.9
Q9UHY1	NRBP1	Nuclear r	14	6	7	6	535	59.8
Q15645	TRIP13	Pachytene	20	8	9	8	432	48.5
O95155	UBE4B	Ubiquitin	7	8	8	8	1302	146.1
Q6UVK1	CSPG4	Chondroit	4	6	6	6	2322	250.4
Q8TCT9	HM13	Minor his	16	6	15	6	377	41.5
P01023	A2M	Alpha-2-m	4	6	15	5	1474	163.2
Q9NZL4	HSPBP1	Hsp70-bin	19	7	9	7	362	39.4
Q9UKV8	AGO2	Protein a	12	9	9	9	859	97.1
A0A2Q3DPC	ARMC9	LisH doma	14	9	10	9	665	75.5
O60507	TPST1	Protein-t	20	6	7	6	370	42.2
P09496	CLTA	Clathrin	22	8	18	8	248	27.1
P37108	SRP14	Signal re	54	6	16	6	136	14.6
Q9UJ41	RABGEF1	Rab5 GDP/	11	6	6	6	708	79.3
A1X283	SH3PXD2B	SH3 and P	9	8	9	6	911	101.5
P20645	M6PR	Cation-de	16	5	8	5	277	31
Q13247	SRSF6	Serine/ar	24	10	14	6	344	39.6
H0Y368	DPM1	Dolichol-	16	4	9	4	295	33.3
A0A0D9SEM	SRSF4	Serine/ar	20	8	13	4	378	43.6
P48307	TFPI2	Tissue fa	35	6	7	6	235	26.9
P67870	CSNK2B	Casein ki	26	4	9	4	215	24.9
Q14126	DSG2	Desmoglei	8	6	8	6	1118	122.2
Q14320	FAM50A	Protein F	24	7	9	7	339	40.2
Q8N668	COMMD1	COMM doma	23	4	6	4	190	21.2
Q9BWF3	RBM4	RNA-bindi	23	8	10	8	364	40.3
E9PDP5	ANKHD1	Ankyrin r	5	6	6	2	1565	166.6
Q9UKG1	APPL1	DCC-inter	12	7	7	7	709	79.6
P23497	SP100	Nuclear a	7	7	19	4	879	100.4
A0A2U3TZL	CD59	CD59 glyc	27	4	17	4	120	13.3
A0A0B4J2D	GATD3B	Glutamine	23	6	9	6	268	28.1
Q08431	MFGE8	Lactadher	19	7	8	7	387	43.1
Q96EK6	GNPNAT1	Glucosami	32	4	9	4	184	20.7
O00566	MPHOSPH1C	U3 small	11	9	10	9	681	78.8
Q9NRF8	CTPS2	CTP synth	11	6	8	3	586	65.6
A0A087X0W	RELA	Transcrip	17	6	8	6	448	49.5
Q96J02	ITCH	E3 ubiqui	10	7	7	7	903	102.7
O75306	NDUFS2	NADH dehy	22	7	9	7	463	52.5
Q2M2I8	AAK1	AP2-assoc	11	8	8	7	961	103.8
A0A087WXE	SCAMP1	Secretory	13	4	6	4	312	35
E9PNM1	FDFT1	Squalene	20	6	6	6	410	47.3
F6Y5H0	RBMS1	RNA-bindi	14	5	7	1	386	41.9
P20674	COX5A	Cytochrom	37	5	10	5	150	16.8
Q9P035	HACD3	Very-long	22	7	11	7	362	43.1
O95834	EML2	Echinoder	9	6	7	6	649	70.6
P54709	ATP1B3	Sodium/po	21	7	11	7	279	31.5
Q9UKM7	MAN1B1	Endoplasm	12	7	9	7	699	79.5
P14927	UQCRB	Cytochrom	50	6	8	6	111	13.5
P13716	ALAD	Delta-ami	20	5	6	5	330	36.3

Q9ULD0	OGDHL	2-oxoglut	6	6	8	1	1010	114.4
P0DN79	CBSL	Cystathic	18	8	8	8	551	60.5
J3KQN4	RPL36A	60S ribos	24	6	20	2	142	16.4
Q8TEQ6	GEMIN5	Gem-assoc	6	9	10	9	1508	168.5
Q8NEZ2	VPS37A	Vacuolar	16	5	5	5	397	44.3
Q9HCJ6	VAT1L	Synaptic	20	7	8	7	419	45.9
Q13895	BYSL	Bystin OS	20	8	9	8	437	49.6
P61923	COPZ1	Coatomer	19	3	9	3	177	20.2
P01116	KRAS	GTPase KR	28	4	9	1	189	21.6
A0A0A0MTC	RNF213	E3 ubiqui	2	9	9	8	5256	596.1
B7ZC38	SH3GLB2	Endophili	19	8	8	7	400	44.3
Q96KC8	DNAJC1	DnaJ homc	12	6	7	6	554	63.8
P10599	TXN	Thioredox	40	5	18	5	105	11.7
Q63ZY3	KANK2	KN motif	9	7	7	7	851	91.1
Q13948	CUX1	Protein C	12	8	9	1	678	77.4
Q9Y5K8	ATP6V1D	V-type pr	30	6	9	6	247	28.2
A0A1B0GTL	RAB11FIP5	Rab11 fam	6	7	8	7	1324	138.3
Q86XL3	ANKLE2	Ankyrin r	9	7	8	7	938	104.1
P31751	AKT2	RAC-beta	18	8	8	4	481	55.7
Q16799	RTN1	Reticulon	13	7	9	7	776	83.6
Q10472	GALNT1	Polypepti	13	7	13	7	559	64.2
Q96QR8	PURB	Transcrip	21	8	9	8	312	33.2
O75688	PPM1B	Protein p	14	6	7	5	479	52.6
P61026	RAB10	Ras-relat	30	8	16	6	200	22.5
P04222	HLA-C	HLA class	22	6	8	1	366	40.8
H0YNE9	RAB8B	Ras-relat	31	6	11	2	188	21.9
P62269	RPS18	40S ribos	33	6	18	6	152	17.7
O95140	MFN2	Mitofusin	11	7	7	6	757	86.3
Q8TAE8	GADD45GIP	Growth ar	40	7	8	7	222	25.4
Q9Y2H1	STK38L	Serine/th	11	5	6	4	464	54
E7ERK9	EIF2B4	Translati	19	7	7	7	544	59.7
A0A087WVF	PDE4DIP	Myomegali	3	8	9	8	2240	253.9
Q9H6T3	RPAP3	RNA polym	12	7	8	7	665	75.7
Q9ULZ3	PYCARD	Apoptosis	26	4	5	4	195	21.6
Q9Y6E0	STK24	Serine/th	17	6	6	3	443	49.3
E7EPT4	NDUFV2	NADH dehy	31	8	10	8	252	27.9
A0A0A0MRM	NOLC1	Nucleolar	12	10	12	10	708	74.6
Q9BRP8	PYM1	Partner c	43	5	7	5	204	22.6
K7EMQ3	DNM2	Dynamin-2	23	8	10	1	289	33.7
P49959	MRE11	Double-st	8	6	8	6	708	80.5
P48426	PIP4K2A	Phosphati	18	8	9	4	406	46.2
Q9P2B4	CTTNBP2NL	CTTNBP2 N	10	7	9	7	639	70.1
Q9H269	VPS16	Vacuolar	7	5	6	5	839	94.6
C9JRZ6	CHCHD3	MICOS com	30	8	10	8	232	26.7
O75691	UTP20	Small sub	3	9	9	9	2785	318.2
H0YJ75	PPP2R5C	Serine/th	12	6	8	3	553	64
Q14644	RASA3	Ras GTPas	11	7	7	7	834	95.6
Q8NCC3	PLA2G15	Group XV	16	5	6	5	412	46.6
Q9NZZ3	CHMP5	Charged m	32	5	12	5	219	24.6
P57740	NUP107	Nuclear p	7	5	7	5	925	106.3
O95864	FADS2	Fatty aci	14	6	10	5	444	52.2

A0A0G2JPF	SCRIB	Protein s	4	8	9	8	1655	177.6
O14561	NDUFAB1	Acyl carr	19	4	11	4	156	17.4
A0A0A0MT6	FKBP15	Peptidylp	7	7	7	7	1244	136.2
Q9NQ48	LZTFL1	Leucine z	19	5	6	5	299	34.6
P54802	NAGLU	Alpha-N-a	14	7	8	7	743	82.2
P62899	RPL31	60S ribos	42	6	16	6	125	14.5
Q15654	TRIP6	Thyroid r	16	6	10	6	476	50.3
F5GZ97	WASHC3	WASH comp	32	5	7	5	193	21
P52594	AGFG1	Arf-GAP d	14	7	7	7	562	58.2
B4E3T4	RBPMS	HCG204342	37	6	6	6	224	25
O75964	ATP5L	ATP synth	39	4	12	4	103	11.4
P17677	GAP43	Neuromodu	40	5	5	5	238	24.8
Q9NSC5	HOMER3	Homer prc	24	7	7	7	361	39.8
E9PNP3	AAMDC	Mth938 dc	47	7	8	7	141	15.6
P46937	YAP1	Transcrip	19	6	7	6	504	54.4
Q96C90	PPP1R14B	Protein p	31	3	6	2	147	15.9
Q9NS86	LANCL2	LanC-like	11	4	6	4	450	50.8
O75340	PDCD6	Programme	28	5	8	5	191	21.9
E7EVX8	PRPF31	U4/U6 sma	15	7	9	7	493	54.7
P11166	SLC2A1	Solute ca	7	4	7	4	492	54
P82650	MRPS22	28S ribos	22	7	7	7	360	41.3
Q02539	HIST1H1A	Histone H	24	5	16	2	215	21.8
P09601	HMOX1	Heme oxyg	23	5	6	5	288	32.8
Q9HAV4	XPO5	Exportin-	7	8	9	8	1204	136.2
P51665	PSMD7	26S prote	16	6	9	6	324	37
H3BLV9	SRPK1	SRSF prot	9	6	7	5	671	76
Q6N069	NAA16	N-alpha-a	6	6	9	1	864	101.4
Q6YP21	KYAT3	Kynurenir	17	7	8	7	454	51.4
Q9GZR7	DDX24	ATP-deper	7	5	5	5	859	96.3
Q04760	GLO1	Lactoylgl	28	6	10	6	184	20.8
D3DTX6	PPP1R9B	Neurabin-	8	7	8	7	817	89.3
O60784	TOM1	Target of	10	4	5	4	492	53.8
Q15397	PUM3	Pumilio h	11	7	9	7	648	73.5
Q99538	LGMN	Legumain	10	4	9	4	433	49.4
P41223	BUD31	Protein E	47	8	8	8	144	17
Q9BV57	ADI1	1,2-dihyd	51	8	8	8	179	21.5
Q8NFQ8	TOR1AIP2	Torsin-1A	17	7	8	6	470	51.2
Q00796	SORD	Sorbitol	20	7	9	7	357	38.3
P84103	SRSF3	Serine/ar	28	6	15	5	164	19.3
Q9BYJ9	YTHDF1	YTH domai	12	7	9	2	559	60.8
Q9HAU4	SMURF2	E3 ubiqui	12	8	8	8	748	86.1
Q8WTT2	NOC3L	Nucleolar	9	9	9	9	800	92.5
Q01650	SLC7A5	Large neu	8	4	6	4	507	55
B4DR61	SEC61A1	cDNA FLJ5	12	6	15	6	482	52.9
A0A0C4DFM	TM9SF4	Transmemb	12	6	9	6	625	72.5
P18754	RCC1	Regulator	18	5	6	5	421	44.9
Q9NUY8	TBC1D23	TBC1 doma	14	7	7	7	699	78.3
A0A0A0MTL	RIPOR1	Rho famil	6	8	8	8	1238	134
Q92572	AP3S1	AP-3 comp	38	6	7	6	193	21.7
Q99575	POP1	Ribonucle	7	7	8	7	1024	114.6
Q03154	ACY1	Aminoacyl	21	6	9	6	408	45.9

Q6GMV3	PTRHD1	Putative	31	5	8	5	140	15.8
Q9BYD2	MRPL9	39S ribos	23	6	9	6	267	30.2
Q96T76	MMS19	MMS19 nuc	9	7	7	7	1030	113.2
F1T0I1	SEC16A	Protein t	4	9	9	9	2334	249.3
Q03701	CEBPZ	CCAAT/enh	8	8	9	8	1054	120.9
Q8IZ52	CHPF	Chondroit	9	6	8	6	775	85.4
H7C2T5	POFUT2	GDP-fucos	26	8	13	1	265	30.4
Q9BZH6	WDR11	WD repeat	7	8	10	8	1224	136.6
B4E0K5	MAPK14	Mitogen-a	25	6	6	6	283	32.3
Q9GZS3	WDR61	WD repeat	25	5	7	5	305	33.6
Q9BYX2	TBC1D2	TBC1 doma	7	5	6	5	928	105.3
Q86X55	CARM1	Histone-a	9	6	8	6	608	65.8
F6U1T9	PPP3R1	Calcineur	33	4	5	4	160	18.2
P27694	RPA1	Replicati	13	7	7	7	616	68.1
A0A087WYFPDLIM3		PDZ and L	21	4	7	4	276	30.1
H0YEH1	PICALM	Phosphati	19	4	5	1	308	32.2
B8ZZA8	GLS	Glutamina	25	4	9	3	169	18.6
F5H5I6	GRSF1	G-rich se	16	6	8	6	424	47.9
P49458	SRP9	Signal re	50	5	11	5	86	10.1
Q9NUJ1	ABHD10	Mycophenc	22	5	6	5	306	33.9
O76024	WFS1	Wolframir	9	8	9	8	890	100.2
Q9Y4X5	ARIH1	E3 ubiqui	11	6	9	6	557	64.1
Q9H8H0	NOL11	Nucleolar	10	7	9	7	719	81.1
Q06203	PPAT	Amidophos	14	6	6	6	517	57.4
Q6FI81	CIAPIN1	Anamorsir	19	7	11	7	312	33.6
P62081	RPS7	40S ribos	26	6	15	2	194	22.1
Q96K17	BTF3L4	Transcrip	37	6	9	4	158	17.3
E9PKP7	UBTF	Nucleolar	11	8	8	8	745	87.4
Q99653	CHP1	Calcineur	31	6	7	6	195	22.4
Q99470	SDF2	Stromal c	25	5	9	5	211	23
Q9Y3I1	FBXO7	F-box onl	13	6	7	6	522	58.5
Q13228	SELENBP1	Methaneth	12	5	7	5	472	52.4
P51648	ALDH3A2	Fatty ald	18	7	7	7	485	54.8
Q9UNX4	WDR3	WD repeat	6	6	6	6	943	106
Q9H0S4	DDX47	Probable	15	6	7	6	455	50.6
Q9H9A6	LRRC40	Leucine-r	12	7	8	7	602	68.2
Q3KQU3	MAP7D1	MAP7 doma	9	7	10	7	841	92.8
Q9Y6D5	ARFGEF2	Brefeldir	5	9	9	4	1785	201.9
O43353	RIPK2	Receptor-	11	5	6	4	540	61.2
O00462	MANBA	Beta-manr	7	6	7	6	879	100.8
O00743	PPP6C	Serine/th	21	7	10	7	305	35.1
Q8NF37	LPCAT1	Lysophosp	9	5	7	5	534	59.1
P49916	LIG3	DNA ligas	8	8	8	8	1009	112.8
Q9NSK0	KLC4	Kinesin l	8	5	7	1	619	68.6
O15254	ACOX3	Peroxisom	6	3	6	3	700	77.6
Q96GQ7	DDX27	Probable	9	9	9	9	796	89.8
Q16537	PPP2R5E	Serine/th	11	6	9	6	467	54.7
Q9Y243	AKT3	RAC-gamma	18	8	8	3	479	55.7
Q9UBR2	CTSZ	Cathepsir	16	6	9	6	303	33.8
Q15382	RHEB	GTP-bindi	28	6	7	6	184	20.5
P01584	IL1B	Interleuk	21	5	8	5	269	30.7

P49841	GSK3B	Glycogen	14	4	6	2	420	46.7
Q9BVL2	NUP58	Nucleopor	12	6	8	6	599	60.9
P35270	SPR	Sepiapter	30	6	7	6	261	28
Q8IZ83	ALDH16A1	Aldehyde	8	5	6	5	802	85.1
H0Y990	ABCE1	ATP-bindi	30	4	10	1	147	16.2
Q9UI12	ATP6V1H	V-type pr	15	6	6	6	483	55.8
P35813	PPM1A	Protein p	22	7	7	6	382	42.4
Q05086	UBE3A	Ubiquitin	9	6	6	6	875	100.6
P09488	GSTM1	Glutathic	26	7	10	1	218	25.7
Q9UMS0	NFU1	NFU1 iron	26	5	7	5	254	28.4
P48634	PRRC2A	Protein F	3	7	7	6	2157	228.7
Q9BRJ6	C7orf50	Uncharact	42	6	7	6	194	22.1
A6NHR9	SMCHD1	Structura	4	8	9	7	2005	226.2
F8VU90	FKBP11	Peptidylp	26	4	10	4	182	19.8
O60231	DHX16	Pre-mRNA-	8	8	9	7	1041	119.2
Q86TI2	DPP9	Dipeptidy	7	6	8	6	863	98.2
P51531	SMARCA2	Probable	5	7	7	2	1590	181.2
O00193	SMAP	Small aci	30	4	8	4	183	20.3
O75794	CDC123	Cell divi	19	8	9	8	336	39.1
Q7L0Y3	TRMT10C	tRNA meth	21	7	8	7	403	47.3
P39060	COL18A1	Collagen	4	6	6	6	1754	178.1
O95163	ELP1	Elongator	6	6	6	6	1332	150.2
H3BMM9	RNPS1	RNA-bindi	22	4	6	4	284	31.7
P07741	APRT	Adenine p	32	5	12	5	180	19.6
P41208	CETN2	Centrin-2	27	4	6	4	172	19.7
Q9P0V9	SEPT10	Septin-10	12	4	6	3	454	52.6
Q9NZJ4	SACS	Sacsin OS	2	8	8	8	4579	520.8
P51571	SSR4	Transloco	25	4	8	4	173	19
Q9NV70	EXOC1	Exocyst c	9	9	9	9	894	101.9
Q15208	STK38	Serine/th	12	5	6	4	465	54.2
Q9NRY4	ARHGAP35	Rho GTPas	7	9	9	9	1499	170.4
Q2TAY7	SMU1	WD40 repe	13	7	8	7	513	57.5
Q9NRX4	PHPT1	14 kDa ph	48	6	8	6	125	13.8
Q9NXF1	TEX10	Testis-ex	8	6	6	6	929	105.6
Q14558	PRPSAP1	Phosphori	15	5	7	3	356	39.4
Q7KZ85	SUPT6H	Transcrip	5	7	7	7	1726	198.9
Q9Y3Z3	SAMHD1	Deoxynucl	11	6	7	6	626	72.2
A0A0U1RRK	MICU1	Calcium u	15	7	10	7	480	54.9
P61970	NUTF2	Nuclear t	35	4	11	4	127	14.5
Q9Y320	TMX2	Thioredox	22	5	7	5	296	34
O95833	CLIC3	Chloride	25	5	7	5	236	26.6
Q15067	ACOX1	Peroxisom	12	7	8	7	660	74.4
Q9H173	SIL1	Nucleotid	14	5	7	5	461	52.1
J3KQ48	PTRH2	Peptidyl-	43	5	7	5	180	19.3
E7EQI7	WASHC5	WASH comp	6	6	6	6	1011	117
Q8N5K1	CISD2	CDGSH irc	45	5	10	5	135	15.3
Q9NVH1	DNAJC11	DnaJ homc	12	7	7	7	559	63.2
A0A087X0K	CAB39	Calcium-b	19	7	8	5	339	39.4
P53634	CTSC	Dipeptidy	13	4	5	4	463	51.8
P20337	RAB3B	Ras-relat	26	5	7	2	219	24.7
P49750	YLPM1	YLP motif	3	6	7	6	2146	241.5

H0Y742	SUN1	SUN domai	10	7	7	6	710	79.5
Q969Q0	RPL36AL	60S ribos	32	6	17	2	106	12.5
Q8IXI1	RHOT2	Mitochond	10	4	5	2	618	68.1
Q8IZL8	PELP1	Proline-,	8	6	6	6	1130	119.6
O15042	U2SURP	U2 snRNP-	9	9	9	9	1029	118.2
Q9NQ29	LUC7L	Putative	12	4	8	2	371	43.7
O15056	SYNJ2	Synaptoja	4	5	5	5	1496	165.4
Q9BRK3	MXRA8	Matrix re	13	6	6	6	442	49.1
Q00059	TFAM	Transcrip	26	6	6	6	246	29.1
P49006	MARCKSL1	MARCKS-re	19	3	10	3	195	19.5
M0QXF9	BCAT2	Branched-	17	6	8	6	445	49.9
O95716	RAB3D	Ras-relat	24	5	6	2	219	24.3
P19174	PLCG1	1-phospha	7	9	11	9	1290	148.4
J3KRC4	NT5C	5'(3')-de	26	5	7	5	182	20.4
Q9BXK5	BCL2L13	Bcl-2-lik	16	5	5	5	485	52.7
Q16774	GUK1	Guanylate	27	5	5	5	197	21.7
Q96JG6	VPS50	Syndetin	6	6	7	6	964	111.1
P13984	GTF2F2	General t	28	6	6	6	249	28.4
Q99447	PCYT2	Ethanolam	17	7	8	7	389	43.8
P36915	GNL1	Guanine n	13	8	8	8	607	68.6
O43447	PPIH	Peptidyl-	33	7	11	6	177	19.2
J3KS05	CBX1	Chromobox	20	3	6	2	173	20
O00754	MAN2B1	Lysosomal	7	7	8	7	1011	113.7
Q12959	DLG1	Disks lar	7	6	7	6	904	100.4
P09619	PDGFRB	Platelet-	6	7	8	5	1106	123.9
Q15286	RAB35	Ras-relat	23	6	10	4	201	23
A0A087WZL	PHLDB1	Pleckstri	9	6	7	1	641	73.1
Q15102	PAFAH1B3	Platelet-	25	6	8	6	231	25.7
Q8WUW1	BRK1	Protein E	52	4	9	4	75	8.7
P50583	NUDT2	Bis(5'-nu	39	5	7	5	147	16.8
P23743	DGKA	Diacylgly	10	6	8	6	735	82.6
Q9Y3P9	RABGAP1	Rab GTPas	6	7	7	6	1069	121.7
P09417	QDPR	Dihydropt	23	5	6	5	244	25.8
Q9H4L5	OSBPL3	Oxysterol	9	6	8	6	887	101.2
Q8IZP0	ABI1	Abl inter	11	5	7	3	508	55
B5MC98	PREB	Prolactin	22	4	4	4	359	38.9
Q9H089	LSG1	Large sub	10	6	6	6	658	75.2
P22033	MUT	Methylmal	12	8	8	8	750	83.1
Q07812	BAX	Apoptosis	20	3	6	3	192	21.2
P55769	SNU13	NHP2-like	34	4	10	4	128	14.2
Q9UDR5	AASS	Alpha-ami	7	7	7	7	926	102.1
Q6P1N0	CC2D1A	Coiled-cc	7	6	6	6	951	104
P60510	PPP4C	Serine/th	11	3	5	2	307	35.1
P61225	RAP2B	Ras-relat	31	6	8	4	183	20.5
Q8WYA6	CTNNBL1	Beta-cate	12	7	7	7	563	65.1
Q969G5	CAVIN3	Caveolae-	20	4	8	4	261	27.7
Q8TB61	SLC35B2	Adenosine	15	6	10	6	432	47.5
Q9NYH9	UTP6	U3 small	9	6	7	6	597	70.1
Q9H1I8	ASCC2	Activatir	11	6	6	6	757	86.3
P11182	DBT	Lipoamide	10	5	6	5	482	53.5
E7EPM6	ACSL1	Long-chai	12	8	8	8	664	74.2

Q7Z3B4	NUP54	Nucleopor	15	7	7	7	507	55.4
Q9UEY8	ADD3	Gamma-add	9	6	7	6	706	79.1
P24928	POLR2A	DNA-direc	3	5	6	5	1970	217
E9PMR6	ARHGEF12	Rho guani	5	5	6	5	1441	161.8
Q9H993	ARMT1	Protein-g	12	5	7	5	441	51.1
P02787	TF	Serotrans	4	2	7	2	698	77
P49593	PPM1F	Protein p	14	5	7	5	454	49.8
Q8NCW5	NAXE	NAD(P)H-h	19	4	5	4	288	31.7
Q92797	SYMPK	Symplekin	7	7	9	7	1274	141.1
F5H345	HMBS	Porphobil	21	7	7	7	330	35.7
A0A2R8Y62	RPS7	40S ribos	25	5	15	1	169	19.4
Q6P4E1	CASC4	Protein C	15	6	7	6	433	48.8
P14678	SNRPB	Small nuc	18	4	9	4	240	24.6
Q9UKX7	NUP50	Nuclear p	14	5	6	5	468	50.1
Q96AX1	VPS33A	Vacuolar	11	7	8	7	596	67.6
A0A087X26	TARDBP	TAR DNA-b	26	5	8	5	301	34.2
Q8TBX8	PIP4K2C	Phosphati	13	7	8	6	421	47.3
Q96S97	MYADM	Myeloid-a	16	3	5	3	322	35.3
Q9Y639	NPTN	Neuroplas	12	4	6	4	398	44.4
H0YC42		Uncharact	18	4	5	2	278	31.2
Q9UMX5	NENF	Neudesin	26	4	5	4	172	18.8
Q9NZJ9	NUDT4	Diphospho	32	4	5	3	180	20.3
Q5TEC6	HIST2H3PS	Histone H	27	5	19	3	136	15.4
P83111	LACTB	Serine be	13	6	7	6	547	60.7
Q9Y3L5	RAP2C	Ras-relat	36	6	8	3	183	20.7
S4R3Q9	OXA1L	Mitochond	10	4	6	4	435	48.5
J3KMZ9	LDLR	Low-densi	7	6	7	6	945	104.6
Q8NBL1	POGLUT1	Protein O	14	6	7	6	392	46.2
Q14376	GALE	UDP-gluco	13	4	8	4	348	38.3
Q9UKI2	CDC42EP3	Cdc42 eff	26	5	8	5	254	27.7
Q8NBY1	STK26	Serine/th	17	6	7	3	392	43.8
A8MYT4	PIK3C3	Phosphati	6	4	6	4	824	94.3
A0A2R8Y56	RELCH	RAB11-bin	8	8	8	8	1216	134.5
P21127	CDK11B	Cyclin-de	8	6	7	6	795	92.6
P49902	NT5C2	Cytosolic	10	6	7	6	561	64.9
Q9Y5P6	GMPPB	Mannose-1	14	4	7	4	360	39.8
P42126	ECI1	Enoyl-CoA	13	4	7	4	302	32.8
Q15370	ELOB	Elongin-B	36	6	9	6	118	13.1
Q5TDH0	DDI2	Protein D	13	4	5	4	399	44.5
Q16718	NDUFA5	NADH dehy	43	4	6	4	116	13.5
Q9H270	VPS11	Vacuolar	9	8	8	8	941	107.8
Q8IYB3	SRRM1	Serine/ar	5	4	6	4	904	102.3
Q5SW96	LDLRAP1	Low densi	18	5	6	5	308	33.9
C9JQ41	CCDC58	Coiled-co	46	5	5	5	130	15.3
Q9HC07	TMEM165	Transmemb	13	2	4	2	324	34.9
Q9BQ39	DDX50	ATP-depen	7	6	9	4	737	82.5
O43665	RGS10	Regulator	35	5	7	5	173	20.2
H0YEH2	PUM1	Pumilio h	5	6	7	6	1125	120.2
P14854	COX6B1	Cytochrom	52	4	10	4	86	10.2
Q8TF42	UBASH3B	Ubiquitin	7	5	6	5	649	72.6
Q13523	PRPF4B	Serine/th	6	6	6	6	1007	116.9

E9PG40	APP	Amyloid-b	8	6	7	6	714	80.8
Q9HB40	SCPEP1	Retinoid-	13	5	6	5	452	50.8
O95721	SNAP29	Synaptosc	25	5	6	5	258	29
E7ESD2	WASHC2A	WASH comp	5	5	5	5	1279	140.1
Q13123	IK	Protein F	11	6	7	6	557	65.6
A0A087X1G	SELENOF	Selenopro	20	3	6	3	164	17.9
Q9P0I2	EMC3	ER membra	21	4	6	4	261	29.9
Q16647	PTGIS	Prostacyc	12	4	5	4	500	57.1
Q99567	NUP88	Nuclear p	11	7	8	7	741	83.5
Q7L9L4	MOB1B	MOB kinas	20	4	8	4	216	25.1
Q6UXH1	CRELD2	Cysteine-	15	5	7	5	353	38.2
P13612	ITGA4	Integrin	6	6	7	6	1032	114.8
Q86SQ0	PHLDB2	Pleckstri	5	6	6	6	1253	142.1
Q5T6F2	UBAP2	Ubiquitin	7	6	7	6	1119	117
Q9NQS1	AVEN	Cell deat	25	7	8	7	362	38.5
P02795	MT2A	Metalloth	67	4	8	2	61	6
Q9H0A8	COMMD4	COMM doma	34	6	6	6	199	21.8
Q13151	HNRNPA0	Heterogen	24	6	11	5	305	30.8
Q00765	REEP5	Receptor	15	4	7	4	189	21.5
Q9UHL4	DPP7	Dipeptidy	11	5	8	5	492	54.3
Q9NX62	IMPAD1	Inositol	14	7	9	7	359	38.7
Q9BTE6	AARSD1	Alanyl-tR	22	6	7	6	412	45.5
B4DTF2	ANXA8L1	Annexin C	22	6	6	6	270	30.5
A0A2R8YG4	ARHGEF7	Rho guani	10	7	7	7	862	97.1
Q9NX55	HYPK	Huntingti	31	3	5	3	129	14.7
Q5VW36	FOCAD	Focadhesi	3	5	5	5	1801	199.9
Q9P253	VPS18	Vacuolar	6	5	5	5	973	110.1
O43172	PRPF4	U4/U6 sma	15	6	6	6	522	58.4
P18583	SON	Protein S	3	7	7	7	2426	263.7
A0A2R8YFH	MSH2	DNA misma	8	6	6	6	918	102.8
P61960	UFM1	Ubiquitin	68	4	9	4	85	9.1
Q9Y4P1	ATG4B	Cysteine	13	3	5	3	393	44.3
O60524	NEMF	Nuclear e	6	7	8	7	1076	122.9
Q96EQ0	SGTB	Small glu	11	3	6	3	304	33.4
Q96H20	SNF8	Vacuolar-	23	5	5	5	258	28.8
P00167	CYB5A	Cytochrom	27	3	5	3	134	15.3
Q92879	CELF1	CUGBP Ela	13	6	13	6	486	52
Q9BV38	WDR18	WD repeat	15	6	6	6	432	47.4
O14672	ADAM10	Disintegr	10	6	6	6	748	84.1
P10619	CTSA	Lysosomal	10	5	10	5	480	54.4
P82673	MRPS35	28S ribos	23	4	5	4	323	36.8
F8VZQ9	SARNP	SAP domai	21	5	7	5	213	24.1
P42345	MTOR	Serine/th	3	8	8	8	2549	288.7
O43583	DENR	Density-r	17	4	7	4	198	22.1
Q6ZMI0	PPP1R21	Protein p	8	4	5	4	780	88.3
P29536	LMOD1	Leiomodin	12	9	9	9	600	67
F8VTV8	CDK4	Cyclin-de	29	6	6	5	201	22
Q96RT1	ERBIN	Erbin OS=	5	7	7	7	1412	158.2
Q9Y2R9	MRPS7	28S ribos	24	5	7	5	242	28.1
Q96F86	EDC3	Enhancer	13	7	7	7	508	56
Q13526	PIN1	Peptidyl-	31	4	8	4	163	18.2

A0A1W2PNF	PIGT	GPI trans	7	4	6	4	580	65.9
E9PC15	AGK	Acylglyce	10	4	6	4	394	43.8
A6NMQ3	ENSA	Alpha-end	42	5	8	4	140	15.6
Q16762	TST	Thiosulfa	16	3	4	3	297	33.4
P17948	FLT1	Vascular	4	5	7	4	1338	150.7
P49773	HINT1	Histidine	40	5	9	5	126	13.8
Q96F85	CNRIP1	CB1 canna	42	4	5	4	164	18.6
K7EJE1	NDUFA13	NADH dehy	26	4	6	4	150	17.1
A0A087X21	CIP2A	Protein C	8	7	7	7	906	102.2
Q99622	C12orf57	Protein C	44	4	5	4	126	13.2
Q9NZ32	ACTR10	Actin-rel	13	5	6	5	417	46.3
Q9Y2D4	EXOC6B	Exocyst c	8	7	8	7	811	94.1
P07738	BPGM	Bisphosph	29	5	5	5	259	30
P09001	MRPL3	39S ribos	13	6	6	6	348	38.6
Q9H6Z4	RANBP3	Ran-bindi	10	5	5	5	567	60.2
P11279	LAMP1	Lysosome-	12	5	9	5	417	44.9
P23786	CPT2	Carnitine	10	6	6	6	658	73.7
O43148	RNMT	mRNA cap	10	5	7	5	476	54.8
P82675	MRPS5	28S ribos	12	6	7	6	430	48
Q15061	WDR43	WD repeat	9	6	7	6	677	74.8
P40818	USP8	Ubiquitin	6	6	6	6	1118	127.4
Q9H0L4	CSTF2T	Cleavage	12	6	7	1	616	64.4
O75223	GGCT	Gamma-glu	30	5	5	5	188	21
Q9BW27	NUP85	Nuclear p	11	6	6	6	656	75
Q9Y3C1	NOP16	Nucleolar	35	6	6	6	178	21.2
Q6NXE6	ARMC6	Armadillo	13	6	7	6	501	54.1
Q9UNW1	MINPP1	Multiple	11	5	8	5	487	55
Q6ZR64	MXRA7	HBV PreS1	15	3	4	1	213	23.4
Q9H7C4	SYNC	Syncoilin	9	3	4	3	482	55.3
Q15334	LLGL1	Lethal(2)	8	7	7	7	1064	115.3
P05114	HMGN1	Non-histo	28	4	6	4	100	10.7
Q9Y394	DHRS7	Dehydroge	12	4	4	4	339	38.3
A0A0J9YXF	PON2	Paraoxona	17	5	7	5	375	41.5
E7ESY4	MTA1	Metastasi	8	6	7	2	703	79.3
Q86XZ4	SPATS2	Spermatog	18	8	8	8	545	59.5
O95182	NDUFA7	NADH dehy	56	6	6	6	113	12.5
Q9NUP9	LIN7C	Protein l	19	4	5	2	197	21.8
P35754	GLRX	Glutaredo	31	3	9	3	106	11.8
O00483	NDUFA4	Cytochrom	56	5	12	5	81	9.4
Q12981	BNIP1	Vesicle t	29	6	6	6	228	26.1
Q9UBW8	COPS7A	COP9 sign	19	6	6	6	275	30.3
Q9H0E2	TOLLIP	Toll-inte	23	6	7	6	274	30.3
P62857	RPS28	40S ribos	46	3	15	3	69	7.8
P82909	MRPS36	28S ribos	42	3	4	3	103	11.5
H0Y7W6	FNBP1	Formin-bi	13	8	8	8	568	65.3
G5E9Z2	CLPTM1L	Cisplatin	13	4	5	4	369	43.3
Q9BX68	HINT2	Histidine	35	4	5	4	163	17.2
Q9HB90	RRAGC	Ras-relat	12	4	5	4	399	44.2
Q8N129	CNPY4	Protein c	13	4	5	4	248	28.3
Q6ZW31	SYDE1	Rho GTPas	9	6	6	6	735	79.7
Q3ZCQ8	TIMM50	Mitochond	15	4	5	4	353	39.6

Q9UPY8	MAPRE3	Microtubu	15	5	10	3	281	32
O75608	LYPLA1	Acyl-prot	20	5	10	5	230	24.7
B1AKR6	DYNLRB1	Dynein li	33	3	4	3	148	16.2
P13747	HLA-E	HLA class	14	4	6	2	358	40.1
P42677	RPS27	40S ribos	38	3	12	1	84	9.5
Q9H8M7	MINDY3	Ubiquitir	9	4	6	4	445	49.7
Q9H1E3	NUCKS1	Nuclear u	21	4	9	4	243	27.3
P81605	DCD	Dermcidir	25	4	7	4	110	11.3
O75886	STAM2	Signal tr	11	6	7	6	525	58.1
Q8NBF2	NHLRC2	NHL repea	8	6	6	6	726	79.4
Q9NY12	GAR1	H/ACA rib	25	5	7	5	217	22.3
Q86TX2	ACOT1	Acyl-coer	14	5	6	5	421	46.2
O15347	HMGB3	High mobi	22	5	7	4	200	23
O75323	NIPSNAP2	Protein N	22	6	9	5	286	33.7
J3KP15	SRSF2	Serine/ar	30	4	12	4	133	15.4
Q9BPX3	NCAPG	Condensir	5	5	6	5	1015	114.3
Q5QJ74	TBCEL	Tubulin-s	13	4	5	4	424	48.2
Q8TC07	TBC1D15	TBC1 doma	7	5	7	5	691	79.4
Q9NWB6	ARGLU1	Arginine	22	8	8	8	273	33.2
A8MQB8	FMR1	Synaptic	12	7	7	5	582	65.8
Q92552	MRPS27	28S ribos	14	6	7	6	414	47.6
O00330	PDHX	Pyruvate	9	5	6	5	501	54.1
Q7LBR1	CHMP1B	Charged m	30	8	9	8	199	22.1
Q9H2W6	MRPL46	39S ribos	21	5	6	5	279	31.7
Q9BSJ2	TUBGCP2	Gamma-tub	5	4	5	4	902	102.5
Q8WZA9	IRGQ	Immunity-	12	4	5	4	623	62.7
O75506	HSBP1	Heat shoc	57	4	6	4	76	8.5
Q9UP83	COG5	Conserved	9	7	7	7	839	92.7
P78537	BLOC1S1	Biogenesi	37	5	8	5	153	17.3
P04114	APOB	Apolipopr	1	7	7	6	4563	515.3
O75891	ALDH1L1	Cytosolic	4	4	7	1	902	98.8
Q8NCN5	PDPR	Pyruvate	7	5	6	5	879	99.3
P11908	PRPS2	Ribose-ph	19	6	8	3	318	34.7
Q9H1B7	IRF2BPL	Interferc	8	6	6	4	796	82.6
Q15021	NCAPD2	Condensir	5	8	8	8	1401	157.1
P31146	CORO1A	Coronin-1	11	5	9	4	461	51
Q9Y5X2	SNX8	Sorting n	10	4	5	4	465	52.5
Q9Y5A9	YTHDF2	YTH domai	9	5	7	2	579	62.3
F8W8I6	TIA1	Nucleolys	12	4	5	2	385	42.8
Q16513	PKN2	Serine/th	8	7	7	6	984	112
Q14139	UBE4A	Ubiquitir	5	4	6	4	1066	122.5
O95232	LUC7L3	Luc7-like	13	5	6	5	432	51.4
Q9BQA1	WDR77	Methylosc	18	5	7	5	342	36.7
Q6ZVM7	TOM1L2	TOM1-like	12	6	6	6	507	55.5
Q7Z2Z2	EFL1	Elongatic	5	6	8	5	1120	125.4
D6RGX2	UFSP2	Ufm1-spec	21	5	5	5	387	44.1
Q9H0U3	MAGT1	Magnesium	15	5	8	4	335	38
P42574	CASP3	Caspase-3	19	4	4	4	277	31.6
Q6EMK4	VASN	Vasorin C	7	4	5	4	673	71.7
Q9BTY7	HGH1	Protein H	18	7	7	7	390	42.1
Q8WZA0	LZIC	Protein L	33	6	6	6	190	21.5

X6R4W8	ZNF207	BUB3-inte	7	3	7	3	497	52.6
Q96HS1	PGAM5	Serine/th	20	4	5	4	289	32
P78356	PIP4K2B	Phosphati	11	6	7	2	416	47.3
F8WAN9	GMPR2	GMP reduc	19	6	6	6	349	38.2
Q9UGR2	ZC3H7B	Zinc fing	7	7	7	7	993	111.5
Q86WR0	CCDC25	Coiled-cc	31	7	8	7	208	24.5
D6W5Y5	CIRBP	Cold indu	11	3	5	3	297	31.9
O60831	PRAF2	PRA1 fami	26	4	5	4	178	19.2
Q16775	HAGH	Hydroxyac	18	6	7	6	308	33.8
Q14197	MRPL58	Peptidyl-	29	6	8	6	206	23.6
Q9BYD3	MRPL4	39S ribos	25	6	6	6	311	34.9
Q9BV86	NTMT1	N-termina	25	4	4	4	223	25.4
O75381	PEX14	Peroxisom	12	4	6	4	377	41.2
P45984	MAPK9	Mitogen-a	8	3	5	3	424	48.1
Q6UXV4	APOOL	MICOS com	17	5	5	5	268	29.1
Q7Z7K6	CENPV	Centromer	17	4	5	4	275	29.9
Q9NZV1	CRIM1	Cysteine-	7	5	5	5	1036	113.7
A0A0A0MSAPVR		Poliovirt	10	4	6	4	392	42.9
Q15276	RABEP1	Rab GTPas	6	6	6	6	862	99.2
J3KMZ8	DPF2	Zinc fing	15	5	6	5	405	45.8
Q29RF7	PDS5A	Sister ch	7	8	8	7	1337	150.7
P08579	SNRPB2	U2 small	20	5	7	4	225	25.5
H0Y5K5	ERGIC3	Endoplasm	14	6	7	6	397	44.6
H7BY82	COL5A1	Collagen	17	3	9	1	210	23.6
Q6NUQ1	RINT1	RAD50-int	7	6	7	6	792	90.6
Q9Y5S9	RBM8A	RNA-bindi	21	4	10	4	174	19.9
Q05655	PRKCD	Protein k	11	7	7	6	676	77.5
O15118	NPC1	NPC intra	5	5	5	5	1278	142.1
A0A067XG5ATP11C		Phospholi	5	5	5	5	1113	127.6
X6R700	CHTOP	Chromatir	19	3	4	3	223	23.6
A0A1B0GVFFTO		Alpha-ket	10	5	6	5	559	64.1
P84022	SMAD3	Mothers a	8	4	7	3	425	48.1
Q9NWT6	HIF1AN	Hypoxia-i	14	5	6	5	349	40.3
Q10469	MGAT2	Alpha-1,6	13	4	4	4	447	51.5
C9JME2	FARP1	FERM, ARH	6	7	8	7	1076	122
Q8NFH4	NUP37	Nucleopor	16	5	7	5	326	36.7
Q9BW83	IFT27	Intraflag	30	5	7	5	186	20.5
Q15434	RBMS2	RNA-bindi	12	4	6	2	407	43.9
P53801	PTTG1IP	Pituitary	18	3	10	3	180	20.3
E7EPV7	SNCA	Alpha-syr	38	3	5	3	115	11.8
Q9Y3B4	SF3B6	Splicing	34	4	8	4	125	14.6
O43837	IDH3B	Isocitrat	14	6	6	6	385	42.2
A0A0D9SGDTATDN1		Putative	19	4	5	4	243	27.6
Q86YP4	GATAD2A	Transcrip	8	4	5	3	633	68
Q15050	RRS1	Ribosome	14	4	5	4	365	41.2
Q9Y315	DERA	Deoxyribc	16	4	4	4	318	35.2
A0A0A0MT6FDXR		NADPH:adr	12	6	8	6	534	58.2
Q7Z5L9	IRF2BP2	Interferc	11	5	5	3	587	61
Q9Y2D5	AKAP2	A-kinase	6	6	7	6	859	94.6
G3V4P8	GMFB	Glia matu	25	4	8	3	150	17.5
Q99523	SORT1	Sortilin	5	4	6	4	831	92

Q9H1Y0	ATG5	Autophagy	17	4	5	4	275	32.4
R4GMQ1	KDM1A	Lysine-sp	5	4	5	4	858	93.5
Q13426	XRCC4	DNA repai	17	6	6	6	336	38.3
Q9Y3D9	MRPS23	28S ribos	30	6	8	6	190	21.8
K7ERP4	GPX4	Glutathic	35	5	6	5	155	17.6
O95168	NDUFB4	NADH dehy	31	3	6	3	129	15.2
K7EM24	ATP6V0A1	V-type pr	38	4	4	4	130	15.4
P62166	NCS1	Neuronal	28	5	7	5	190	21.9
C9JZY6	UBE2H	Ubiquitir	34	4	6	4	123	13.8
A0A087X2D	MRPL45	39S ribos	21	5	6	5	306	35.3
Q96FN4	CPNE2	Copine-2	7	3	7	2	548	61.2
P15090	FABP4	Fatty aci	25	3	5	3	132	14.7
K7EMH1	RPL22	60S ribos	27	2	7	2	89	10.4
H0Y488	ARID1A	AT-rich i	3	5	5	5	1901	205.7
Q01581	HMGCS1	Hydroxyme	11	5	6	5	520	57.3
Q9NXG2	THUMPD1	THUMP dom	14	6	6	6	353	39.3
F6T1Q0	PDE12	2',5'-phc	17	7	8	7	472	52.1
A0A0C4DFL	CYP51A1	Lanosterc	9	4	7	4	509	57.2
Q13325	IFIT5	Interferc	13	5	5	5	482	55.8
C9JG97	AAMP	Angio-ass	12	4	4	4	415	44.7
J3KRJ9	COPZ2	Coatomer	33	4	7	4	141	16.1
Q86SX6	GLRX5	Glutaredc	25	3	5	3	157	16.6
Q5W0V3	FAM160B1	Protein F	7	6	7	6	765	86.5
O75844	ZMPSTE24	CAAX prer	11	5	6	5	475	54.8
Q9UFN0	NIPSNAP3A	Protein N	19	4	5	4	247	28.4
Q9P265	DIP2B	Disco-int	4	5	5	5	1576	171.4
B1AJY5	PSMD10	26S prote	24	4	5	4	185	20.2
Q9H3H3	C11orf68	UPF0696 p	14	3	5	3	251	27.3
O43678	NDUFA2	NADH dehy	48	5	7	5	99	10.9
D6REB4	PAIP1	Polyadeny	9	4	5	4	351	39.9
Q9NT62	ATG3	Ubiquitir	20	7	9	7	314	35.8
D6RAN1	PDLIM7	PDZ and L	46	5	9	1	90	9.5
Q96FQ6	S100A16	Protein S	33	3	7	3	103	11.8
Q14997	PSME4	Proteasom	4	6	6	6	1843	211.2
Q9NR28	DIABLO	Diablo hc	18	5	7	5	239	27.1
P35249	RFC4	Replicati	15	5	5	5	363	39.7
P49770	EIF2B2	Translati	12	3	5	3	351	39
Q96GK7	FAHD2A	Fumarylac	14	4	6	4	314	34.6
Q13107	USP4	Ubiquitir	5	6	7	5	963	108.5
Q8IY17	PNPLA6	Neuropath	4	6	6	6	1366	149.9
Q6ZXV5	TMTC3	Transmemb	8	7	8	7	915	103.9
P49840	GSK3A	Glycogen	12	5	6	3	483	50.9
Q96PU8	QKI	Protein q	13	4	5	4	341	37.6
O75494	SRSF10	Serine/ar	16	6	6	6	262	31.3
Q15018	ABRAXAS2	BRISC com	8	3	4	3	415	46.9
Q92882	OSTF1	Osteoclas	34	5	5	5	214	23.8
Q7RTV0	PHF5A	PHD finge	48	5	5	5	110	12.4
Q6NVY1	HIBCH	3-hydroxy	18	8	9	8	386	43.5
Q9NVZ3	NECAP2	Adaptin e	23	6	6	6	263	28.3
O95169	NDUFB8	NADH dehy	33	5	7	5	186	21.8
Q9H3K2	GHITM	Growth hc	9	3	5	3	345	37.2

Q9P2W9	STX18	Syntaxin-	19	5	7	5	335	38.7
P16930	FAH	Fumarylac	11	5	8	5	419	46.3
Q8WVV9	HNRNPLL	Heterogen	12	5	7	4	542	60
Q1JUQ5	FKBP1A	Peptidylp	15	1	9	1	92	10.1
H0YME5	EIF2AK4	eIF-2-alp	5	6	6	6	1427	161.1
Q96EE3	SEH1L	Nucleopor	14	4	4	4	360	39.6
Q5JVF3	PCID2	PCI domai	13	6	6	6	399	46
Q92522	H1FX	Histone H	21	4	4	4	213	22.5
Q9NR09	BIRC6	Baculovir	2	6	6	6	4857	529.9
Q8NFW8	CMAS	N-acylneu	15	7	7	7	434	48.3
Q15269	PWP2	Periodic	6	5	5	5	919	102.4
Q99543	DNAJC2	DnaJ homo	13	7	7	7	621	72
Q15599	SLC9A3R2	Na(+)/H(+	17	5	6	5	337	37.4
Q3MHD2	LSM12	Protein L	14	3	9	3	195	21.7
O95219	SNX4	Sorting n	12	5	5	5	450	51.9
H0YNG3	SEC11A	Signal pe	26	5	10	5	163	18.6
Q96QD8	SLC38A2	Sodium-co	13	4	4	4	506	56
C9JBI3	PSPH	Phosphose	17	3	4	3	187	20.7
Q9Y508	RNF114	E3 ubiqui	21	4	6	4	228	25.7
O00115	DNASE2	Deoxyribo	12	4	6	4	360	39.6
Q96I25	RBM17	Splicing	11	5	5	5	401	44.9
Q9BRR6	ADPGK	ADP-deper	7	3	4	3	497	54.1
M0R2N5	TECR	Very-long	15	6	10	6	346	39.9
Q9H3Z4	DNAJC5	DnaJ homo	19	4	6	4	198	22.1
Q8IYM9	TRIM22	E3 ubiqui	10	5	5	5	498	56.9
Q5SWX8	ODR4	Protein o	14	5	5	5	454	51.1
Q02252	ALDH6A1	Methylmal	10	5	6	5	535	57.8
Q15796	SMAD2	Mothers a	7	3	6	2	467	52.3
Q9HD33	MRPL47	39S ribos	20	5	6	5	250	29.4
P52630	STAT2	Signal tr	5	4	5	4	851	97.9
Q92541	RTF1	RNA polym	8	5	6	5	710	80.3
Q9NWH9	SLTM	SAFB-like	6	6	6	6	1034	117.1
Q13636	RAB31	Ras-relat	25	4	5	3	194	21.6
Q7Z4G1	COMMD6	COMM doma	49	4	6	4	85	9.6
Q96IU4	ABHD14B	Protein A	30	5	8	5	210	22.3
E7EQY4	MTA3	Metastasi	11	7	8	1	514	58.7
P26447	S100A4	Protein S	26	3	6	3	101	11.7
Q9Y4K4	MAP4K5	Mitogen-a	6	5	5	5	846	95
Q9P015	MRPL15	39S ribos	18	4	4	4	296	33.4
Q9H2U1	DHX36	ATP-deper	5	6	7	5	1008	114.7
Q9NX40	OCIAD1	OCIA doma	18	4	7	4	245	27.6
E9PQY2	PFDN4	Prefoldir	28	3	6	3	136	15.6
Q8N1B4	VPS52	Vacuolar	9	5	5	5	723	82.2
Q7RTP6	MICAL3	[F-actin]	2	5	6	4	2002	224.2
Q9NPJ3	ACOT13	Acyl-coer	16	2	4	2	140	15
P48060	GLIPR1	Glioma pa	13	2	5	2	266	30.3
H0YJ50	VRK1	Serine/th	16	4	5	4	232	26.7
C9JA28	SSR3	Transloco	16	4	9	4	174	20.1
Q9BZQ6	EDEM3	ER degrad	5	4	4	4	932	104.6
Q9UNQ2	DIMT1	Probable	16	5	5	5	313	35.2
Q9BU89	DOHH	Deoxyhypu	15	3	4	3	302	32.9

P01857	IGHG1	Immunoglc	9	2	4	2	330	36.1
P32456	GBP2	Guanylate	8	5	5	3	591	67.2
O95470	SGPL1	Sphingosi	10	7	8	7	568	63.5
Q92508	PIEZO1	Piezo-typ	2	5	5	5	2521	286.6
A2A2V1	PRNP	Major pri	12	3	8	3	249	27.3
O96005	CLPTM1	Cleft lip	7	4	6	4	669	76
Q7Z434	MAVS	Mitochond	8	4	8	4	540	56.5
Q9UEU0	VTI1B	Vesicle t	22	5	5	5	232	26.7
E9PRJ8	CD81	Tetraspan	10	1	3	1	209	22.5
Q5STZ8	ABCF1	ATP-bindi	15	6	7	2	339	38.3
Q9H7D7	WDR26	WD repeat	7	5	5	5	661	72.1
Q9NRL3	STRN4	Striatin-	6	4	4	3	753	80.5
Q96LJ7	DHRS1	Dehydroge	20	5	5	5	313	33.9
A0A2R8YE1VPS45		Vacuolar	9	6	7	6	534	61.1
P01033	TIMP1	Metallopr	19	4	5	4	207	23.2
Q5T0D9	TPRG1L	Tumor prc	18	4	5	4	272	30.2
Q5T8P6	RBM26	RNA-bindi	6	6	6	6	1007	113.5
Q9UI30	TRMT112	Multifunc	33	3	5	3	125	14.2
H3BMV3	JPT2	Jupiter m	32	4	6	4	190	20.7
Q7L5D6	GET4	Golgi to	17	5	5	5	327	36.5
C9JYQ9	RPL22L1	60S ribos	26	3	5	3	121	14.5
Q96SL4	GPX7	Glutathic	29	5	5	5	187	21
Q9UI09	NDUFA12	NADH dehy	39	4	4	4	145	17.1
Q9H553	ALG2	Alpha-1,3	6	2	3	2	416	47.1
Q92615	LARP4B	La-relate	10	6	6	6	738	80.5
G0XQ39	STIM1	STIM1L OS	5	4	5	4	791	88.6
Q9Y3C8	UFC1	Ubiquitin	24	4	5	4	167	19.4
Q9NVG8	TBC1D13	TBC1 doma	12	5	5	5	400	46.5
P50851	LRBA	Lipopolys	2	6	6	6	2863	318.9
Q8TDN6	BRIX1	Ribosome	14	5	5	5	353	41.4
Q96TC7	RMDN3	Regulator	15	6	6	6	470	52.1
Q6P3W7	SCYL2	SCY1-like	6	5	5	5	929	103.6
Q00534	CDK6	Cyclin-de	19	5	5	4	326	36.9
Q86YR5	GPSM1	G-protein	8	5	6	5	675	74.5
Q6P1J9	CDC73	Parafibrc	13	6	6	6	531	60.5
Q96FJ2	DYNLL2	Dynein li	45	3	6	2	89	10.3
Q96HD1	CRELD1	Cysteine-	11	4	5	4	420	45.4
P26572	MGAT1	Alpha-1,3	10	4	4	4	445	50.8
Q7L7X3	TAOK1	Serine/th	5	5	7	4	1001	116
O75911	DHRS3	Short-cha	16	4	4	4	302	33.5
O14828	SCAMP3	Secretory	14	3	4	3	347	38.3
Q8WWX9	SELENOM	Selenoprc	26	3	6	3	145	16.2
P17050	NAGA	Alpha-N-a	10	3	4	3	411	46.5
Q14573	ITPR3	Inositol	3	8	8	5	2671	303.9
Q2TAA2	IAH1	Isoamyl a	24	5	9	5	248	27.6
P78357	CNTNAP1	Contactin	4	6	6	6	1384	156.2
K7ENV7	ISOC2	Isochoris	37	3	4	3	174	18.9
V9HW50	ADH1B	Alcohol d	12	2	4	2	375	39.8
Q5R372	RABGAP1L	Rab GTPas	7	5	5	4	815	92.5
Q6YN16	HSDL2	Hydroxyst	11	5	6	5	418	45.4
P41214	EIF2D	Eukaryoti	9	4	4	4	584	64.7

K7EPR5	PRKAR1A	cAMP-depe	93	4	6	1	45	5.1
O75348	ATP6V1G1	V-type pr	28	4	6	4	118	13.7
Q9NUM4	TMEM106B	Transmemb	16	4	6	4	274	31.1
H0YB34	RIDA	2-iminobu	27	3	4	3	148	16
Q86VR2	RETREG3	Reticulop	8	4	6	4	466	51.4
P21926	CD9	CD9 antig	13	3	6	3	228	25.4
Q9BZL4	PPP1R12C	Protein p	7	5	5	5	782	84.8
Q96FZ7	CHMP6	Charged m	24	5	6	5	201	23.5
O00160	MYO1F	Unconvent	3	3	4	1	1098	124.8
O15228	GNPAT	Dihydroxy	8	5	5	5	680	77.1
Q9UJY5	GGA1	ADP-ribos	6	5	6	5	639	70.3
Q9UKS6	PACSIN3	Protein k	15	5	5	5	424	48.5
Q9UL25	RAB21	Ras-relat	18	4	6	4	225	24.3
Q9ULC3	RAB23	Ras-relat	19	4	5	4	237	26.6
O60547	GMDS	GDP-manno	15	6	6	6	372	41.9
H0YB16	PTK2	Focal adh	6	4	4	4	724	81.4
Q14344	GNA13	Guanine n	9	4	7	2	377	44
Q2M389	WASHC4	WASH comp	4	5	6	5	1173	136.3
A0A087WT2	DCAF13	DDB1- and	6	3	4	3	597	67.5
Q13563	PKD2	Polycysti	5	5	7	5	968	109.6
A3KMH1	VWA8	von Wille	3	5	5	5	1905	214.7
Q92665	MRPS31	28S ribos	13	5	5	5	395	45.3
Q6PD62	CTR9	RNA polym	5	6	6	6	1173	133.4
O43264	ZW10	Centromer	6	4	5	4	779	88.8
Q10588	BST1	ADP-ribos	12	4	7	4	318	35.7
P56385	ATP5ME	ATP synth	32	2	4	2	69	7.9
Q13505	MTX1	Metaxin-1	8	3	5	3	466	51.4
Q9BTE1	DCTN5	Dynactin	19	4	6	4	182	20.1
E7EQB8	IDH3G	Isocitrat	15	4	4	4	340	37
P37235	HPCAL1	Hippocalc	33	5	6	5	193	22.3
Q9NY27	PPP4R2	Serine/th	12	5	5	5	417	46.9
O43181	NDUFS4	NADH dehy	19	3	4	3	175	20.1
P04732	MT1E	Metalloth	34	3	6	1	61	6
Q9P287	BCCIP	BRCA2 and	18	4	4	4	314	36
Q9H814	PHAX	Phosphory	9	3	5	3	394	44.4
Q9HBH5	RDH14	Retinol d	15	5	5	5	336	36.8
A0A075B79	GRIPAP1	GRIP1-ass	8	5	5	5	810	92.7
Q9NWM8	FKBP14	Peptidyl-	25	5	6	5	211	24.2
Q92805	GOLGA1	Golgin su	8	5	5	5	767	88.1
C9JPE1	SLC25A20	Mitochond	27	5	5	5	228	25.1
Q5TD07	NQO2	Ribosyldi	29	4	5	4	193	21.5
Q96JB2	COG3	Conserved	7	5	5	5	828	94
E5RGX5	STMN2	Stathmin	15	3	6	1	168	19.6
P09234	SNRPC	U1 small	13	2	4	2	159	17.4
Q8TF05	PPP4R1	Serine/th	4	4	4	4	950	106.9
Q9UNP9	PPIE	Peptidyl-	16	5	6	4	301	33.4
P78563	ADARB1	Double-st	8	4	4	4	741	80.7
P19474	TRIM21	E3 ubiqui	11	5	5	5	475	54.1
Q8ND24	RNF214	RING fing	8	5	5	5	703	77.6
Q9UJ68	MSRA	Mitochond	24	5	5	5	235	26.1
O14530	TXNDC9	Thioredox	12	3	5	3	226	26.5

O75382	TRIM3	Tripartit	6	5	5	5	744	80.8
Q8IVD9	NUDCD3	NudC doma	17	5	6	5	361	40.8
Q4G0F5	VPS26B	Vacuolar	17	5	6	4	336	39.1
A0A0D9SGEPHF6		PHD finge	14	5	5	5	366	41.3
A0A0J9YWWUTP4		U3 small	7	3	4	3	511	57.3
Q13136	PPFIA1	Liprin-al	6	7	7	7	1202	135.7
P07305	H1F0	Histone H	15	3	5	3	194	20.9
A0A0C4DGQDHRS7B		Dehydroge	15	4	4	4	310	33.5
Q9BVC6	TMEM109	Transmemb	9	3	5	3	243	26.2
Q8IWA5	SLC44A2	Choline t	5	4	6	4	706	80.1
E7ER68	FAM91A1	Protein F	6	4	4	4	789	88.9
O60343	TBC1D4	TBC1 doma	4	5	5	5	1298	146.5
Q9NX58	LYAR	Cell grow	11	4	5	4	379	43.6
Q9HA77	CARS2	Probable	11	5	5	5	564	62.2
P35556	FBN2	Fibrillin	2	6	6	2	2912	314.6
Q9H9Q2	COPS7B	COP9 sign	10	2	4	2	264	29.6
Q5QPQ0	LYPLA2	Acyl-prot	31	4	6	4	164	17.6
Q9NWU2	GID8	Glucose-i	25	4	4	4	228	26.7
Q5SRE5	NUP188	Nucleopor	3	6	6	6	1749	195.9
O14936	CASK	Periphera	6	6	6	6	926	105.1
F2Z2E2	IQGAP3	Ras GTPas	3	4	12	2	1588	179.4
C9JIJ9	RBMS3	RNA-bindi	8	4	5	1	436	47.7
Q12974	PTP4A2	Protein t	26	4	4	2	167	19.1
O60828	PQBP1	Polygluta	21	5	6	5	265	30.5
Q9BYN8	MRPS26	28S ribos	27	5	5	5	205	24.2
P41743	PRKCI	Protein k	9	5	5	5	596	68.2
P21359	NF1	Neurofibr	2	6	6	6	2839	319.2
P51003	PAPOLA	Poly(A) p	5	4	5	4	745	82.8
P56182	RRP1	Ribosomal	10	4	4	4	461	52.8
O14907	TAX1BP3	Tax1-binc	25	3	7	3	124	13.7
A0A087X1FACAP2		Arf-GAP w	8	6	6	6	777	88
E9PQP6	FNTA	Protein f	17	4	4	4	249	30.1
Q9GZY8	MFF	Mitochonc	16	3	3	3	342	38.4
O43809	NUDT21	Cleavage	23	5	6	5	227	26.2
Q86VP1	TAX1BP1	Tax1-binc	7	7	8	7	789	90.8
J3KS22	DCXR	L-xylulos	17	4	4	4	223	23.8
O00422	SAP18	Histone d	29	5	9	5	153	17.6
Q13595	TRA2A	Transforn	17	5	5	5	282	32.7
P10620	MGST1	Microsoma	9	2	4	2	155	17.6
Q9NYB9	ABI2	Abl inter	9	4	4	2	513	55.6
A8MTY9	VPS26C	VPS26 enc	12	2	3	2	249	27.9
D4Q8H0	pk	Mitogen-a	11	4	4	4	455	51.5
Q9BYG3	NIFK	MKI67 FHA	17	6	9	6	293	34.2
Q9NX20	MRPL16	39S ribos	18	3	4	3	251	28.4
Q9HA64	FN3KRP	Ketosamin	13	5	5	5	309	34.4
O95205	MBNL2	Musclebli	16	5	9	2	255	28.1
Q8WW12	PCNP	PEST prot	18	3	4	3	178	18.9
Q92692	NECTIN2	Nectin-2	9	6	8	6	538	57.7
Q96L92	SNX27	Sorting n	7	4	4	4	541	61.2
A0A096LP2AAK1		AP2-assoc	12	4	4	3	511	54.4
Q93063	EXT2	Exostosin	7	5	6	5	718	82.2

H0YMJ0	MORF4L1	Mortality	15	3	4	3	245	28.2
H0YGX7	ARHGDIB	Rho GDP-d	29	4	4	4	195	22.4
Q9H4A5	GOLPH3L	Golgi pho	15	4	6	4	285	32.7
P25445	FAS	Tumor nec	14	4	4	4	335	37.7
A0A1B0GUE	ASAH1	Acid cera	20	6	6	6	330	37.4
Q9NX14	NDUFB11	NADH dehy	27	3	4	3	153	17.3
H7BYT1	CSNK1D	Casein ki	12	5	7	4	427	49
F8WC86	LIMS1	LIM and s	26	3	6	2	117	13.3
J3KNF4	CCS	Copper ch	20	6	6	6	255	27.1
P52298	NCBP2	Nuclear c	28	5	5	5	156	18
O75970	MPDZ	Multiple	3	5	5	5	2070	221.5
Q8IWV7	UBR1	E3 ubiqui	3	4	5	4	1749	200.1
O76071	CIAO1	Probable	17	4	4	4	339	37.8
J3KPS0	DNAJB12	DnaJ (Hsp	11	4	4	4	409	45.5
Q15126	PMVK	Phosphome	23	4	4	4	192	22
Q9Y3E7	CHMP3	Charged m	18	5	6	5	222	25.1
B4E321	OS9	Protein O	15	5	5	5	406	46.3
Q6SZW1	SARM1	Sterile a	5	3	4	3	724	79.3
P13995	MTHFD2	Bifunctio	17	5	7	5	350	37.9
P13611	VCAN	Versican	1	5	5	5	3396	372.6
Q8IYB5	SMAP1	Stromal m	8	4	6	4	467	50.4
Q6IA86	ELP2	Elongator	6	4	5	4	826	92.4
P35914	HMGCL	Hydroxyme	14	4	4	4	325	34.3
Q15428	SF3A2	Splicing	7	3	4	3	464	49.2
Q96CX2	KCTD12	BTB/POZ d	20	6	6	5	325	35.7
Q8WU10	PYROXD1	Pyridine	8	3	5	3	500	55.8
Q86WN1	FCHSD1	F-BAR and	6	5	5	5	690	76.9
F8VXI9	GIT2	ARF GTPas	8	5	5	3	708	78.8
P09669	COX6C	Cytochrom	36	5	5	5	75	8.8
Q6PJT7	ZC3H14	Zinc fing	7	5	5	5	736	82.8
G3V599	CTAGE5	Endoplasm	3	4	5	4	1339	151.6
P61956	SUMO2	Small ubi	23	2	6	1	95	10.9
P04818	TYMS	Thymidyla	9	2	3	2	313	35.7
O00400	SLC33A1	Acetyl-co	8	4	6	4	549	60.9
M0R3D4	RABAC1	PRA1 fami	25	3	4	3	151	17
P63220	RPS21	40S ribos	48	4	11	4	83	9.1
O75915	ARL6IP5	PRA1 fami	20	4	5	4	188	21.6
Q9H792	PEAK1	Inactive	3	4	4	4	1746	193
Q15814	TBCC	Tubulin-s	15	5	6	4	346	39.2
Q9Y6A4	CFAP20	Cilia- an	17	3	5	3	193	22.8
Q9NZW5	MPP6	MAGUK p55	9	5	5	4	540	61.1
Q9Y6I3	EPN1	Epsin-1 O	8	5	6	5	576	60.3
O60711	LPXN	Leupaxin	14	4	4	4	386	43.3
C9J1V9	EEF1E1-BL	EEF1E1-BL	27	4	5	4	151	17
Q6BCY4	CYB5R2	NADH-cyto	20	5	6	5	276	31.4
Q6ZRP7	QSOX2	Sulfhydry	9	6	7	6	698	77.5
Q9NW64	RBM22	Pre-mRNA-	11	4	4	4	420	46.9
Q7Z4Q2	HEATR3	HEAT repe	8	4	4	4	680	74.5
P17568	NDUFB7	NADH dehy	26	4	7	4	137	16.4
Q5JWE9	GNAS	Guanine n	15	3	4	1	193	21.9
G3V2S9	SLIRP	SRA stem-	19	2	3	2	124	13.9

O43823	AKAP8	A-kinase	7	3	3	3	692	76.1
P61457	PCBD1	Pterin-4-	30	4	5	3	104	12
O60783	MRPS14	28S ribos	19	2	3	2	128	15.1
Q9H0R4	HDHD2	Haloacid	20	4	5	4	259	28.5
Q96TA2	YME1L1	ATP-depen	6	5	7	5	773	86.4
Q5T760	SRSF11	Serine/ar	8	3	4	3	389	42.3
Q9BQE4	SELENOS	Selenopro	21	3	4	3	189	21.2
O14773	TPP1	Tripeptid	8	4	7	4	563	61.2
Q7L775	EPM2AIP1	EPM2A-int	9	5	5	5	607	70.3
Q3YEC7	RABL6	Rab-like	9	6	6	6	729	79.5
Q9GZL7	WDR12	Ribosome	18	6	6	6	423	47.7
Q9NV31	IMP3	U3 small	15	2	5	2	184	21.8
P43155	CRAT	Carnitine	9	6	6	6	626	70.8
A0A286YFL	PPT1	Palmitoyl	9	4	4	4	323	35.7
G3V2U7	ACYP1	Acylphosp	18	2	3	2	129	14.1
Q8WVY7	UBLCP1	Ubiquitin	14	5	6	5	318	36.8
Q9Y3A6	TMED5	Transmemb	10	3	5	3	229	26
Q68DW7	STAG1	Cohesin s	5	5	5	1	998	114.9
Q7Z7H8	MRPL10	39S ribos	17	3	5	3	261	29.3
Q9BX67	JAM3	Junctiona	11	2	3	2	310	35
Q9BY77	POLDIP3	Polymeras	11	4	5	4	421	46.1
P23508	MCC	Colorecta	6	5	5	5	829	93
Q9ULJ7	ANKRD50	Ankyrin r	3	4	4	4	1429	155.8
Q13405	MRPL49	39S ribos	27	4	5	4	166	19.2
P53701	HCCS	Cytochrom	20	5	5	5	268	30.6
Q8N4A0	GALNT4	Polypepti	7	4	4	3	578	66.6
Q99570	PIK3R4	Phosphoin	4	4	4	4	1358	153
Q9NPF4	OSGEP	Probable	12	4	5	4	335	36.4
Q96GA7	SDSL	Serine de	11	3	4	3	329	34.7
Q9NWU5	MRPL22	39S ribos	21	5	8	5	206	23.6
H3BR29	C16orf58	RUS1 fami	10	4	5	4	423	46.3
Q9NRY5	FAM114A2	Protein F	8	4	4	4	505	55.4
Q9Y2R5	MRPS17	28S ribos	45	4	5	4	130	14.5
Q03113	GNA12	Guanine n	8	3	6	1	381	44.3
Q9NVM9	INTS13	Integrato	7	5	5	5	706	80.2
P45985	MAP2K4	Dual spec	15	5	5	5	399	44.3
Q53S08	RAB6D	Ras-relat	12	2	6	1	254	28.2
B7Z2L0	BCAP29	cDNA FLJ5	21	6	6	6	147	17.4
Q5VW32	BROX	BRO1 doma	10	3	4	3	411	46.4
Q9H0W8	SMG9	Protein S	13	5	5	5	520	57.6
Q9NZT2	OGFR	Opioid gr	7	5	5	5	677	73.3
Q15629	TRAM1	Transloca	6	2	3	2	374	43
Q8N4C8	MINK1	Misshapen	3	3	4	1	1332	149.7
P60468	SEC61B	Protein t	30	3	7	3	96	10
P62072	TIMM10	Mitochond	31	3	4	3	90	10.3
Q9HBH1	PDF	Peptide d	23	3	3	3	243	27
P07711	CTSL	Cathepsin	10	3	4	3	333	37.5
O14657	TOR1B	Torsin-1B	12	4	4	4	336	38
Q9P2I0	CPSF2	Cleavage	4	2	4	2	782	88.4
A0A075B6F	NOSIP	Nitric ox	12	3	5	3	304	33.4
Q8IZH2	XRN1	5’-3’ exo	2	3	4	3	1706	194

Q92643	PIGK	GPI-ancho	10	3	3	3	395	45.2
Q71RC2	LARP4	La-relate	5	3	4	3	724	80.5
Q13643	FHL3	Four and	11	3	5	3	280	31.2
H0Y9X1	TMA16	Translati	19	4	4	4	242	27.7
Q9C035	TRIM5	Tripartit	10	6	6	6	493	56.3
I3L4C3	SPAG7	Sperm-ass	22	4	4	4	194	22.1
Q5RI15	COX20	Cytochrom	23	3	5	3	118	13.3
P32189	GK	Glycerol	8	5	5	4	559	61.2
Q5TEJ7	RPA2	Replicati	23	3	3	3	179	19.4
H0Y8C3	MTCH1	Mitochond	11	3	3	3	394	43.1
Q12802	AKAP13	A-kinase	2	4	4	4	2813	307.4
Q96T23	RSF1	Remodelin	3	5	5	5	1441	163.7
H3BMD8	ARPP19	cAMP-regu	37	3	5	2	131	14.5
Q9H3K6	BOLA2	BolA-like	59	4	5	4	86	10.1
Q5BKZ1	ZNF326	DBIRD com	9	4	4	4	582	65.6
Q9UKF6	CPSF3	Cleavage	8	5	5	5	684	77.4
P52735	VAV2	Guanine n	7	6	6	6	878	101.2
Q9P0M6	H2AFY2	Core hist	11	4	5	3	372	40
Q01081	U2AF1	Splicing	16	4	4	4	240	27.9
Q9Y237	PIN4	Peptidyl-	33	2	3	2	131	13.8
P68402	PAFAH1B2	Platelet-	17	4	8	4	229	25.6
Q13823	GNL2	Nucleolar	6	5	5	5	731	83.6
Q86XA9	HEATR5A	HEAT repe	3	5	5	5	2040	221.9
Q9GZX9	TWSG1	Twisted g	17	3	5	3	223	25
O43166	SIPA1L1	Signal-in	2	5	5	5	1804	199.9
O00762	UBE2C	Ubiquitin	31	4	4	4	179	19.6
Q99442	SEC62	Transloca	9	4	6	4	399	45.8
Q6IQ22	RAB12	Ras-relat	21	6	6	5	244	27.2
Q9NQ50	MRPL40	39S ribos	15	2	3	2	206	24.5
P52701	MSH6	DNA misma	4	5	5	5	1360	152.7
P62312	LSM6	U6 snRNA-	34	3	6	3	80	9.1
C9JLV4	APAF1	Apoptotic	3	4	4	4	1163	132.4
Q8N3U4	STAG2	Cohesin s	3	5	5	1	1231	141.2
H3BQQ2	ZNF598	E3 ubiqui	4	3	3	3	849	93.2
P42771	CDKN2A	Cyclin-de	26	3	3	2	156	16.5
Q8N9N7	LRRC57	Leucine-r	12	3	4	3	239	26.7
R4GMU7	RPL7L1	60S ribos	25	5	6	5	198	23
Q9Y2E5	MAN2B2	Epididymi	5	4	5	4	1009	113.9
Q9UPU7	TBC1D2B	TBC1 doma	5	5	5	5	963	109.8
Q16831	UPP1	Uridine p	12	4	4	4	310	33.9
Q9BVM2	DPCD	Protein D	18	4	4	4	203	23.2
Q9Y4P8	WIPI2	WD repeat	10	3	3	3	454	49.4
Q8IUI8	CRLF3	Cytokine	8	3	3	3	442	49.7
P30049	ATP5F1D	ATP synth	14	2	6	2	168	17.5
Q7LG56	RRM2B	Ribonucle	11	4	4	3	351	40.7
A0A2R8Y4M	CLIC5	Chloride	12	3	6	2	200	22.3
O15427	SLC16A3	Monocarbo	6	3	11	3	465	49.4
Q9Y5J7	TIMM9	Mitochond	29	2	5	2	89	10.4
O75937	DNAJC8	DnaJ homo	15	4	4	4	253	29.8
Q08AM6	VAC14	Protein V	7	3	3	3	782	87.9
C9JAB9	NCK1	Cytoplasm	17	3	5	3	174	20.1

O43310	CTIF	CBP80/20-	6	4	4	4	598	67.5
Q00535	CDK5	Cyclin-de	18	4	7	3	292	33.3
C9JWU9	MEST	Mesoderm-	18	3	5	3	213	24.3
Q96C01	FAM136A	Protein F	24	4	5	4	138	15.6
P61009	SPCS3	Signal pe	16	3	4	3	180	20.3
P09455	RBP1	Retinol-b	30	4	4	4	135	15.8
Q86VM9	ZC3H18	Zinc fing	4	4	4	4	953	106.3
P83876	TXNL4A	Thioredox	24	3	5	3	142	16.8
O43251	RBFOX2	RNA bindi	12	3	4	3	390	41.3
Q13445	TMED1	Transmemb	13	3	4	3	227	25.2
Q53H96	PYCR3	Pyrroline	18	3	3	3	274	28.6
Q9Y3D7	PAM16	Mitochond	44	5	7	5	125	13.8
Q15006	EMC2	ER membra	14	3	4	3	297	34.8
Q16836	HADH	Hydroxyac	17	6	8	6	314	34.3
E9PHA2	NCAPH	Condensin	5	4	6	4	730	81.5
P52895	AKR1C2	Aldo-keto	19	4	4	4	323	36.7
P61927	RPL37	60S ribos	38	5	30	5	97	11.1
A0A0A0MT3	LIPA	Lysosomal	10	2	5	2	283	32.5
P30405	PPIF	Peptidyl-	18	5	11	3	207	22
P14406	COX7A2	Cytochrom	28	2	5	2	83	9.4
P15848	ARSB	Arylsulfa	8	4	4	4	533	59.6
P07948	LYN	Tyrosine-	8	4	4	2	512	58.5
O14920	IKBKB	Inhibitor	6	4	4	3	756	86.5
Q9NW68	BSDC1	BSD domai	10	4	4	4	430	47.1
Q8N0U8	VKORC1L1	Vitamin K	15	3	7	3	176	19.8
Q6P9B6	TLDC1	TLD domai	11	4	7	4	456	51
Q96A35	MRPL24	39S ribos	21	4	4	4	216	24.9
Q86SE5	RALYL	RNA-bindi	9	3	4	1	291	32.3
E7EVC7	ATG16L1	Autophagy	7	5	5	5	624	70
Q9Y547	HSPB11	Intraflag	25	2	3	2	144	16.3
O15439	ABCC4	Multidrug	3	4	4	4	1325	149.4
Q92504	SLC39A7	Zinc tran	4	3	6	3	469	50.1
Q96KR1	ZFR	Zinc fing	5	4	4	4	1074	116.9
Q9NQY0	BIN3	Bridging	15	4	4	4	253	29.6
Q9Y399	MRPS2	28S ribos	11	4	5	4	296	33.2
E9PNK6	TPD52L1	Tumor pro	22	3	4	3	166	18.6
P37198	NUP62	Nuclear p	7	3	3	3	522	53.2
D6RIY7	TUSC3	Tumor sup	11	4	5	3	314	35.7
A0A0A0MSH	CHD1L	Chromodom	8	6	6	6	797	89.9
Q9UNN5	FAF1	FAS-assoc	6	3	4	3	650	73.9
Q9H939	PSTPIP2	Proline-s	13	5	5	5	334	38.8
Q68DU8	KCTD16	BTB/POZ d	11	5	6	4	428	49.1
E7EP22	RNF14	RBR-type	21	3	4	3	152	17.5
Q86Y39	NDUFA11	NADH dehy	35	3	3	3	141	14.8
Q9H078	CLPB	Caseinoly	6	4	4	3	707	78.7
Q9HD42	CHMP1A	Charged m	20	5	6	5	196	21.7
A8MU27	SUMO3	Small ubi	14	2	6	1	147	16.9
A0A1W2PQV	GCSH	Glycine c	30	2	4	2	100	11.1
Q92575	UBXN4	UBX domai	11	5	5	5	508	56.7
O95478	NSA2	Ribosome	14	4	4	4	260	30
Q9HAB8	PPCS	Phosphopa	13	5	5	5	311	34

P23458	JAK1	Tyrosine-	4	5	5	5	1154	133.2
Q00587	CDC42EP1	Cdc42 eff	11	3	3	3	391	40.3
A2IDC6	MRPL28	39S ribos	12	4	4	4	240	28.5
A0A0A0MS7	MYD88	Myeloid d	13	3	3	3	309	34.6
Q9Y3D0	FAM96B	Mitotic s	26	2	2	2	163	17.7
P82912	MRPS11	28S ribos	22	3	4	3	194	20.6
P63172	DYNLT1	Dynein li	23	2	3	2	113	12.4
O60447	EVI5	Ecotropic	5	4	4	3	810	92.9
D6RCB9	NHP2	H/ACA rib	21	2	7	2	135	15.2
A0A1W2PP1	VPS35	Vacuolar	65	3	4	1	46	5.4
Q9UMY1	NOL7	Nucleolar	11	3	4	3	257	29.4
Q5VZE5	NAA35	N-alpha-a	5	4	4	4	725	83.6
Q96DA6	DNAJC19	Mitochond	34	4	4	4	116	12.5
Q9BR61	ACBD6	Acyl-CoA-	9	2	3	2	282	31.1
Q96S59	RANBP9	Ran-bindi	4	2	3	2	729	77.8
D6RDG3	BTF3	Transcrip	36	3	8	1	109	11.8
P62314	SNRPD1	Small nuc	28	2	5	2	119	13.3
E7EMN6	PPP1R2	Protein p	21	3	3	3	170	19.2
P30530	AXL	Tyrosine-	5	3	3	3	894	98.3
Q9Y5K6	CD2AP	CD2-assoc	6	4	5	4	639	71.4
A0A0C4DFX	NELFA	Negative	6	3	3	3	539	58.5
Q9H446	RWDD1	RWD domai	11	3	4	3	243	27.9
K4DIA7	CD151	Tetraspan	17	5	12	5	230	25.6
O43402	EMC8	ER membra	17	3	3	3	210	23.8
Q9Y2Y0	ARL2BP	ADP-ribos	24	3	3	3	163	18.8
Q5TCZ1	SH3PXD2A	SH3 and P	3	4	4	2	1133	125.2
Q16864	ATP6V1F	V-type pr	24	3	5	3	119	13.4
A0A087X0N	SLC4A1AP	Kanadapti	6	3	3	3	742	82.8
C9JJ19	MRPS34	28S ribos	17	4	4	4	225	26.3
Q15847	ADIRF	Adipogene	29	2	3	2	76	7.9
P42785	PRCP	Lysosomal	6	3	4	3	496	55.8
F8W7U0	ITSN1	Intersect	3	4	5	4	1149	129.9
Q96EY5	MVB12A	Multivesi	16	3	4	3	273	28.8
Q86VN1	VPS36	Vacuolar	11	4	5	4	386	43.8
Q9HBL8	NMRAL1	NmrA-like	11	3	3	3	299	33.3
Q15650	TRIP4	Activatin	8	4	4	4	581	66.1
Q13951	CBFB	Core-bind	20	3	3	3	182	21.5
Q9P2E5	CHPF2	Chondroit	5	4	5	4	772	85.9
O75175	CNOT3	CCR4-NOT	5	4	4	4	753	81.8
Q07092	COL16A1	Collagen	3	3	3	3	1604	157.7
B8ZZS0	BET1L	BET1-like	18	2	3	2	152	17.1
Q8WYP5	AHCTF1	Protein E	2	4	4	4	2266	252.3
Q16595	FXN	Frataxin,	11	2	3	2	210	23.1
Q8IWX8	CHERP	Calcium h	5	5	5	5	916	103.6
O95298	NDUFC2	NADH dehy	17	2	5	2	119	14.2
A0A0A0MRJ	NAV1	Neuron na	2	4	4	4	1830	197.3
Q7Z7N9	TMEM179B	Transmemb	11	2	3	2	219	23.5
P78346	RPP30	Ribonucle	16	4	4	4	268	29.3
P49427	CDC34	Ubiquitin	15	3	4	3	236	26.7
P06400	RB1	Retinobla	4	5	5	5	928	106.1
Q96T60	PNKP	Bifunctic	8	4	4	4	521	57

Q9HAU5	UPF2	Regulator	4	5	6	5	1272	147.7
Q6NUM9	RETSAT	All-trans	4	3	4	3	610	66.8
I3L4C2	BAIAP2	Brain-spe	8	5	5	5	553	61.3
Q9Y2H0	DLGAP4	Disks lar	4	5	5	5	992	107.9
Q13451	FKBP5	Peptidyl-	11	4	4	4	457	51.2
Q16563	SYPL1	Synaptoph	10	2	5	2	259	28.5
A0A0A0MQF	RTF2	Replicati	11	3	3	3	336	37.5
Q8WTS6	SETD7	Histone-l	10	3	3	3	366	40.7
Q8ND04	SMG8	Protein S	4	3	3	3	991	109.6
E5RGN3	ATOX1	Copper tr	29	3	6	3	59	6.3
Q15678	PTPN14	Tyrosine-	4	5	6	5	1187	135.2
Q92542	NCSTN	Nicastrin	6	5	7	5	709	78.4
M0QXB5	ETHE1	Persulfid	11	3	3	3	260	28.4
P29466	CASP1	Caspase-1	9	4	4	2	404	45.1
Q6UXN9	WDR82	WD repeat	19	5	5	5	313	35.1
Q7Z7H5	TMED4	Transmemb	8	2	3	2	227	25.9
Q15904	ATP6AP1	V-type pr	8	3	3	3	470	52
Q4R9M9	KIF1B	Kinesin f	2	4	4	1	1809	203.5
Q9HCN8	SDF2L1	Stromal c	26	3	3	3	221	23.6
F5H5N1	NDUFS7	NADH dehy	13	2	3	2	182	19.8
Q9Y676	MRPS18B	28S ribos	16	3	3	3	258	29.4
O15160	POLR1C	DNA-direc	13	3	4	3	346	39.2
C9JG87	MRPL39	39S ribos	14	4	4	4	297	34
O00506	STK25	Serine/th	11	4	4	2	426	48.1
Q9Y316	MEMO1	Protein M	12	3	4	3	297	33.7
P31350	RRM2	Ribonucle	14	5	5	4	389	44.8
P84243	H3F3A	Histone H	24	4	18	2	136	15.3
Q8ND56	LSM14A	Protein L	8	3	3	3	463	50.5
P07919	UQCRH	Cytochrom	44	4	4	4	91	10.7
O15111	CHUK	Inhibitor	6	6	6	5	745	84.6
O95674	CDS2	Phosphati	9	3	3	3	445	51.4
Q13601	KRR1	KRR1 smal	10	4	5	4	381	43.6
P78318	IGBP1	Immunoglo	10	3	3	3	339	39.2
O95295	SNAPIN	SNARE-ass	32	4	4	4	136	14.9
Q53GS9	USP39	U4/U6.U5	6	3	3	3	565	65.3
P22830	FECH	Ferrochel	11	5	5	5	423	47.8
O95810	CAVIN2	Caveolae-	10	3	3	3	425	47.1
Q16352	INA	Alpha-int	4	2	23	1	499	55.4
O43660	PLRG1	Pleiotrop	8	5	5	5	514	57.2
Q9Y697	NFS1	Cysteine	13	4	4	4	457	50.2
Q9BZX2	UCK2	Uridine-c	18	4	4	4	261	29.3
P49757	NUMB	Protein n	6	4	5	3	651	70.8
P32929	CTH	Cystathio	6	2	3	2	405	44.5
Q9UEE9	CFDP1	Craniofac	17	4	4	4	299	33.6
P29972	AQP1	Aquaporin	10	2	3	2	269	28.5
O60678	PRMT3	Protein a	9	4	4	4	531	59.9
Q9HAT2	SIAE	Sialate O	7	4	4	4	523	58.3
Q9UBB6	NCDN	Neurochon	5	4	5	4	729	78.8
Q9UID3	VPS51	Vacuolar	8	4	4	4	782	86
Q96SU4	OSBPL9	Oxysterol	4	3	4	3	736	83.1
Q7L8L6	FASTKD5	FAST kina	5	4	4	4	764	86.5

K7EIN2	NUDT16L1	Tudor-int	25	4	4	3	198	21.9
K7EIU8	SMAD4	Mothers a	9	5	5	5	456	50
P51580	TPMT	Thiopurin	18	5	7	5	245	28.2
P49754	VPS41	Vacuolar	4	4	4	4	854	98.5
O43920	NDUFS5	NADH dehy	35	3	3	3	106	12.5
Q8IYB7	DIS3L2	DIS3-like	4	4	5	4	885	99.2
J3KQS6	BABAM1	BRISC and	16	3	3	3	254	28.1
Q96GG9	DCUN1D1	DCN1-like	15	4	4	4	259	30.1
Q9NQG5	RPRD1B	Regulatic	10	2	3	2	326	36.9
P42696	RBM34	RNA-bindi	7	2	2	2	430	48.5
Q8WUA2	PPIL4	Peptidyl-	8	3	3	3	492	57.2
Q92600	CNOT9	CCR4-NOT	16	4	4	4	299	33.6
Q8NEU8	APPL2	DCC-inter	6	3	3	3	664	74.4
E9PPN1	TSEN15	tRNA-spli	17	1	2	1	134	14.6
O43314	PPIP5K2	Inositol	4	5	5	5	1243	140.3
A0A0C4DG3	SFTPA1	Pulmonary	9	1	3	1	158	16
F5H7R9	PTMS	Parathymc	30	2	8	2	57	6.3
Q6RFH5	WDR74	WD repeat	10	3	3	3	385	42.4
P82921	MRPS21	28S ribos	30	2	3	2	87	10.7
Q06265	EXOSC9	Exosome c	9	5	5	5	439	48.9
Q9BXW7	HDHD5	Haloacid	8	2	2	2	423	46.3
Q8IUR0	TRAPPC5	Trafficki	26	5	5	5	188	20.8
Q5TDF0	NTPCR	Cancer-re	15	3	5	3	228	25.1
Q8IX01	SUGP2	SURP and	6	4	4	4	1082	120.1
F8W7Q4	FAM162A	Protein F	27	4	4	4	144	16.5
Q14534	SQLE	Squalene	8	3	3	3	574	63.9
H3BUN4	NOL3	Nucleolar	18	2	4	2	206	22.4
A0A0A0MR5	FADS1	Fatty aci	10	5	6	4	501	57.8
O60216	RAD21	Double-st	5	3	3	3	631	71.6
Q96S66	CLCC1	Chloride	8	4	5	4	551	62
P78316	NOP14	Nucleolar	5	4	4	4	857	97.6
Q13336	SLC14A1	Urea tran	9	4	4	4	389	42.5
E9PLP0	CARS	Cysteine-	20	2	3	1	128	14.3
A0A087X29	WDR6	WD repeat	4	3	3	3	1151	124.9
Q9BZE9	ASPSCR1	Tether cc	7	3	3	3	553	60.1
Q9GZS1	POLR1E	DNA-direc	8	4	4	2	481	53.9
Q8NEJ9	NGDN	Neuroguic	10	3	3	3	315	35.9
P15954	COX7C	Cytochron	29	2	4	2	63	7.2
Q96GX9	APIP	Methylthi	13	2	2	2	242	27.1
Q8WU79	SMAP2	Stromal m	8	4	4	4	429	46.8
G5EA36	CDC27	Cell divi	4	3	3	3	823	91.7
P01834	IGKC	Immunoglc	13	1	6	1	107	11.8
E9PF49	NDUFB9	NADH dehy	17	3	4	3	221	26.6
P00403	MT-CO2	Cytochron	23	4	5	4	227	25.5
Q7Z7M9	GALNT5	Polypepti	3	3	3	2	940	106.2
Q9BY32	ITPA	Inosine t	24	3	4	3	194	21.4
Q712K3	UBE2R2	Ubiquitin	18	4	4	4	238	27.1
Q92543	SNX19	Sorting n	4	3	3	3	992	108.5
Q9UJX3	ANAPC7	Anaphase-	7	4	4	4	599	66.8
P07585	DCN	Decorin C	11	4	4	3	359	39.7
E9PHM2	LARS2	Probable	5	5	5	5	860	96.9

O75503	CLN5	Ceroid-li	10	4	4	4	358	41.5
Q8MH48	HLA-G	HLA class	7	2	4	1	338	38.2
O75431	MTX2	Metaxin-2	8	2	3	2	263	29.7
Q12800	TFCP2	Alpha-glc	8	3	3	2	502	57.2
G3V1D1	FTH1	Ferritin	39	3	3	3	113	12.9
Q13144	EIF2B5	Translati	3	2	2	2	721	80.3
Q9UKX5	ITGA11	Integrin	4	6	6	6	1188	133.4
E9PNY1	ZFPL1	Zinc fing	23	4	4	4	207	22.7
E9PKV8	TTC9C	Tetratric	21	3	4	3	140	16.3
Q9UHI6	DDX20	Probable	5	3	3	3	824	92.2
Q8N2K0	ABHD12	Monoacylg	10	3	4	3	398	45.1
Q96DV4	MRPL38	39S ribos	10	3	5	3	380	44.6
F5H1F6	VPS37B	Vacuolar	19	3	3	3	184	20.6
Q9Y217	MTMR6	Myotubula	8	4	4	4	621	71.9
Q9Y4F5	CEP170B	Centrosom	2	3	4	1	1589	171.6
H0Y6Y8	MRPL43	39S ribos	11	2	3	2	169	18.8
O15121	DEGS1	Sphingoli	11	3	4	3	323	37.8
P62745	RHOB	Rho-relat	15	2	3	1	196	22.1
Q9Y3A2	UTP11	Probable	14	4	4	4	253	30.4
Q9UQN3	CHMP2B	Charged m	15	4	4	4	213	23.9
O15173	PGRMC2	Membrane-	11	3	5	2	223	23.8
A0A1B0GWC	DPY19L1	Probable	6	3	3	3	748	84.5
P50750	CDK9	Cyclin-de	9	4	5	3	372	42.8
J3KNF8	CYB5B	Cytochrom	27	3	6	3	150	16.7
Q96IK1	BOD1	Biorienta	18	3	3	3	185	19.2
Q04771	ACVR1	Activin r	5	2	3	2	509	57.1
P53602	MVD	Diphosphc	6	2	3	2	400	43.4
B4DHE8	MSI2	cDNA FLJ5	8	2	3	2	324	34.8
O94915	FRYL	Protein f	1	4	4	4	3013	339.4
P27449	ATP6V0C	V-type pr	12	1	3	1	155	15.7
H7C5K4	CCDC80	Coiled-cc	8	2	3	2	261	30.5
Q9GZT9	EGLN1	Egl nine	7	3	3	3	426	46
Q9Y385	UBE2J1	Ubiquitir	6	2	3	2	318	35.2
Q8NB37	GATD1	Glutamine	14	3	3	3	220	23.3
P19387	POLR2C	DNA-direc	12	3	3	3	275	31.4
Q8WTW3	COG1	Conservec	3	4	4	4	980	108.9
O96011	PEX11B	Peroxisom	12	3	4	3	259	28.4
Q96RF0	SNX18	Sorting n	7	3	3	3	628	68.9
O00291	HIP1	Huntingti	4	6	6	5	1037	116.1
Q8NEW0	SLC30A7	Zinc trar	7	2	4	2	376	41.6
Q9H0U6	MRPL18	39S ribos	18	3	3	3	180	20.6
Q9UK45	LSM7	U6 snRNA-	48	4	6	4	103	11.6
Q99519	NEU1	Sialidase	10	4	4	4	415	45.4
P59768	GNG2	Guanine n	39	3	4	3	71	7.8
Q96RD7	PANX1	Pannexin-	8	2	2	2	426	48
J3QQZ9	PNPO	Pyridoxir	15	3	3	3	238	27.3
Q9Y6C9	MTCH2	Mitochonc	14	4	4	4	303	33.3
Q96MX6	WDR92	WD repeat	9	3	3	3	357	39.7
Q96P16	RPRD1A	Regulatic	6	2	3	2	312	35.7
P49406	MRPL19	39S ribos	14	3	4	3	292	33.5
O00401	WASL	Neural Wi	8	4	4	4	505	54.8

Q7Z3T8	ZFYVE16	Zinc fing	3	4	4	4	1539	168.8
U3KQG5	CD200	OX-2 memb	13	2	3	2	202	22.5
Q8NBX0	SCCPDH	Saccharop	8	3	5	3	429	47.1
Q13488	TCIRG1	V-type pr	4	3	3	3	830	92.9
Q9UPN7	PPP6R1	Serine/th	5	3	3	3	881	96.7
P23258	TUBG1	Tubulin g	7	3	4	3	451	51.1
Q9BXR0	QTRT1	Queuine t	7	3	3	3	403	44
P08962	CD63	CD63 anti	10	3	8	3	238	25.6
P53365	ARFIP2	Arfaptin-	10	3	3	3	341	37.8
Q9Y3L3	SH3BP1	SH3 domai	4	3	3	3	701	75.7
Q5JSZ5	PRRC2B	Protein F	2	4	4	4	2229	242.8
Q96T88	UHRF1	E3 ubiqui	5	3	3	3	793	89.8
Q8NBQ5	HSD17B11	Estradiol	11	3	3	3	300	32.9
Q86U86	PBRM1	Protein p	2	3	3	3	1689	192.8
F5GXX5	DAD1	Dolichyl-	26	2	3	2	85	9.5
O14734	ACOT8	Acyl-coen	13	3	3	3	319	35.9
Q7L099	RUFY3	Protein R	8	4	4	3	469	52.9
P01137	TGFB1	Transform	8	3	3	3	390	44.3
Q14653	IRF3	Interfero	8	3	4	3	427	47.2
Q96FV9	THOC1	THO compl	4	3	4	3	657	75.6
Q8ND76	CCNY	Cyclin-Y	9	3	4	3	341	39.3
Q15007	WTAP	Pre-mRNA-	9	3	3	3	396	44.2
Q16540	MRPL23	39S ribos	10	1	3	1	153	17.8
J3QRU8	GIT1	ARF GTPas	6	5	6	3	694	76.8
Q9BT09	CNPY3	Protein c	11	4	4	4	278	30.7
Q9HCE0	EPG5	Ectopic F	2	3	3	3	2579	292.3
Q9Y2S7	POLDIP2	Polymeras	7	3	5	3	368	42
P09914	IFIT1	Interfero	7	3	3	3	478	55.3
Q8WUF5	PPP1R13L	RelA-asso	4	3	3	3	828	89
Q86SF2	GALNT7	N-acetylg	5	3	4	3	657	75.3
K7EK35	STAT5A	Signal tr	4	3	3	3	763	87.3
Q8NEZ5	FBXO22	F-box onl	7	3	4	3	403	44.5
Q13043	STK4	Serine/th	7	3	3	3	487	55.6
A0A1W2PNF	IER3IP1	Immediate	29	1	2	1	69	7.6
O15231	ZNF185	Zinc fing	6	4	4	4	689	73.5
Q5J8M3	EMC4	ER membra	21	3	3	3	183	20.1
Q8N9N2	ASCC1	Activatin	7	3	3	3	400	45.5
Q9HCD5	NCOA5	Nuclear r	8	4	4	4	579	65.5
H7C3J3	THUMPD3	THUMP dom	10	3	3	3	269	30.3
P15529	CD46	Membrane	8	3	5	3	392	43.7
V5IRT4	UQCC2	Ubiquinol	10	1	2	1	126	14.8
A0A0B4J2C		Uncharact	6	4	5	2	849	94.6
P49137	MAPKAPK2	MAP kinas	8	4	4	4	400	45.5
Q9BU23	LMF2	Lipase ma	6	3	3	3	707	79.6
Q9Y6C2	EMILIN1	EMILIN-1	5	4	4	4	1016	106.6
E9PJH7	SLC25A22	Mitochond	10	3	3	3	313	33.3
Q12899	TRIM26	Tripartit	5	3	3	3	539	62.1
Q9NZM5	NOP53	Ribosome	8	3	3	3	478	54.4
A0A1B0GU5	PCCA	Propionyl	5	3	3	3	615	67.3
P49184	DNASE1L1	Deoxyribo	11	3	3	3	302	33.9
Q9BYM8	RBCK1	RanBP-typ	7	3	3	3	510	57.5

A0A1B0GTE	ATP6AP2	Renin rec	10	3	3	3	294	33
Q69YN2	CWF19L1	CWF19-lik	7	3	3	3	538	60.6
O95456	PSMG1	Proteasom	16	4	4	4	288	32.8
Q14728	MFSD10	Major fac	5	2	3	2	455	48.3
Q9UNK0	STX8	Syntaxin-	17	3	3	3	236	26.9
Q53T59	HS1BP3	HCLS1-bin	4	1	2	1	392	42.8
Q12979	ABR	Active br	3	2	4	2	859	97.5
A0A140TA8	C19orf70	MICOS com	33	3	4	3	140	15.4
Q13671	RIN1	Ras and R	8	3	3	3	783	84
P30825	SLC7A1	High affi	7	3	4	3	629	67.6
E7EWE1	UBA5	Ubiquitin	9	3	4	3	347	38.7
Q9UII2	ATP5IF1	ATPase in	15	3	6	3	106	12.2
Q8IV38	ANKMY2	Ankyrin r	8	4	4	4	441	49.3
Q9BTU6	PI4K2A	Phosphati	7	4	4	4	479	54
E9PHT6	PANK4	Pantothen	5	3	3	3	737	81.7
C9JE79	CALD1	Caldesmon	43	2	6	1	72	9
C9JCC6	DRAP1	Dr1-assoc	14	3	3	3	212	23.2
Q9Y2P8	RCL1	RNA 3'-te	10	4	4	4	373	40.8
Q9H330	TMEM245	Transmemb	4	4	5	4	879	97.3
Q96HY6	DDRGK1	DDRGK dom	8	2	3	2	314	35.6
G3V325	ATP5MF-PT	ATP5MF-PT	3	2	3	2	749	84.1
O95777	LSM8	U6 snRNA-	35	2	2	2	96	10.4
Q96QG7	MTMR9	Myotubula	7	3	3	3	549	63.4
Q9H6S0	YTHDC2	3'-5' RNA	3	5	5	4	1430	160.1
O00767	SCD	Acyl-CoA	4	1	2	1	359	41.5
A0A0A0MTE	WDR36	WD repeat	4	3	3	3	895	99.3
Q969E2	SCAMP4	Secretory	8	2	5	2	229	25.7
O00214	LGALS8	Galectin-	9	3	3	3	317	35.8
O00178	GTPBP1	GTP-bindi	5	3	3	3	669	72.4
O15258	RER1	Protein R	13	3	6	3	196	22.9
V9GZ56	LSM4	U6 snRNA-	10	3	5	3	238	25.7
Q9NRX5	SERINC1	Serine in	6	2	3	2	453	50.5
Q6P158	DHX57	Putative	2	3	3	2	1386	155.5
J3KQ18	DDT	D-dopachr	14	2	7	2	132	14.2
E1CEI4	GCLC	Glutamate	7	4	4	4	599	68.6
M0QZ21	AP2S1	AP comple	14	2	5	2	122	14.5
Q3KQV9	UAP1L1	UDP-N-ace	5	3	7	2	507	57
Q8NHP8	PLBD2	Putative	5	4	5	4	589	65.4
Q9NVV4	MTPAP	Poly(A) R	5	4	4	4	582	66.1
Q9GZP4	PITHD1	PITH doma	23	4	4	4	211	24.2
Q8N5C1	CALHM5	Calcium h	11	3	3	3	309	35.1
Q9H6U8	ALG9	Alpha-1,2	6	3	3	3	611	69.8
Q8NFH5	NUP35	Nucleopor	10	3	3	3	326	34.8
A0A087WZT	SAMD4B	Protein S	7	4	4	4	663	72.4
O95989	NUDT3	Diphospho	16	3	3	3	172	19.5
Q9H6F5	CCDC86	Coiled-co	11	4	5	4	360	40.2
Q8IWZ8	SUGP1	SURP and	6	4	4	4	645	72.4
P04150	NR3C1	Glucocort	4	3	3	3	777	85.6
Q14999	CUL7	Cullin-7	2	2	2	2	1698	191
Q6NZY4	ZCCHC8	Zinc fing	5	3	3	3	707	78.5
J3QLH3	SAP30BP	SAP30-bin	15	3	3	3	248	27.7

P11441	UBL4A	Ubiquitin	16	2	3	2	157	17.8
O14910	LIN7A	Protein l	12	3	3	1	233	26
Q9NUL5	RYDEN	Repressor	10	3	3	3	291	33.1
Q9H773	DCTPP1	dCTP pyro	11	2	3	2	170	18.7
A0A0C4DGH	NUMBL	Numb-like	3	2	3	1	568	60.8
O43617	TRAPPC3	Trafficki	19	4	5	4	180	20.3
Q9NPH2	ISYNA1	Inositol-	6	4	5	4	558	61
P29084	GTF2E2	Transcrip	10	3	3	3	291	33
F8WCP5	THOC5	THO compl	8	3	4	3	343	39.7
O14735	CDIPT	CDP-diacy	13	3	4	3	213	23.5
Q13795	ARFRP1	ADP-ribos	15	3	3	3	201	22.6
P51809	VAMP7	Vesicle-a	21	4	4	4	220	24.9
P78362	SRPK2	SRSF prot	5	3	3	2	688	77.5
Q15388	TOMM20	Mitochond	19	3	4	3	145	16.3
Q9Y5J1	UTP18	U3 small	7	2	2	2	556	62
F6QUN3	ERI3	ERI1 exor	24	3	4	3	176	19.7
Q9NPA8	ENY2	Transcrip	33	4	4	4	101	11.5
Q8NHP6	MOSPD2	Motile sp	7	4	4	4	518	59.7
Q9UJF2	RASAL2	Ras GTPas	2	2	3	2	1139	128.5
E9PBY7	ZC3H11A	Zinc fing	8	4	4	4	515	57.4
A0A087WXM	BCAM	Basal cel	6	3	3	3	588	63.7
Q9BW91	NUDT9	ADP-ribos	8	3	4	3	350	39.1
Q9NRX1	PNO1	RNA-bindi	15	3	3	3	252	27.9
Q5VIR6	VPS53	Vacuolar	6	5	5	5	699	79.6
Q9NRP0	OSTC	Oligosacc	13	2	6	2	149	16.8
E7EW05	SDAD1	Protein S	4	3	3	3	650	75.4
P24941	CDK2	Cyclin-de	11	3	4	1	298	33.9
Q9BTZ2	DHRS4	Dehydroge	12	4	4	4	278	29.5
Q4G0N4	NADK2	NAD kinas	7	3	3	3	442	49.4
Q9BS40	LXN	Latexin C	9	2	4	2	222	25.7
Q9GZV5	WWTR1	WW domain	10	5	5	5	400	44.1
O00499	BIN1	Myc box-d	9	3	3	3	593	64.7
P50135	HNMT	Histamine	15	3	3	3	292	33.3
Q96KB5	PBK	Lymphokin	8	3	4	3	322	36.1
Q9NXS2	QPCTL	Glutaminy	8	3	3	3	382	42.9
P55290	CDH13	Cadherin-	7	5	6	5	713	78.2
Q8N3F8	MICALL1	MICAL-lik	3	3	4	3	863	93.4
A8MUM1	EIPR1	EARP-inte	7	3	3	3	414	46.3
O43719	HTATSF1	HIV Tat-s	5	4	4	4	755	85.8
O60232	SSSCA1	Sjoegren	16	3	3	3	199	21.5
Q14767	LTBP2	Latent-tr	2	3	3	3	1821	194.9
D6RCP9	DCK	Deoxycyti	13	3	3	3	199	22.9
P08476	INHBA	Inhibin b	6	3	3	3	426	47.4
J3QR68	HP	Haptoglob	3	1	2	1	404	45
O43815	STRN	Striatin	4	4	4	3	780	86.1
A0A0A0MRQ	PTPN9	Tyrosine-	6	3	3	3	583	66.9
Q5VTR2	RNF20	E3 ubiqui	4	5	6	5	975	113.6
Q9H000	MKRN2	Probable	7	3	3	3	416	46.9
O75063	FAM20B	Glycosami	8	2	2	2	409	46.4
P98179	RBM3	RNA-bindi	25	3	4	3	157	17.2
Q9UNN8	PROCR	Endotheli	5	1	2	1	238	26.7

P38435	GGCX	Vitamin K	5	3	3	3	758	87.5
P50613	CDK7	Cyclin-de	9	3	3	3	346	39
O95400	CD2BP2	CD2 antig	12	3	4	3	341	37.6
I3L2L5	MCRIP1	Mapk-regu	24	2	3	2	92	10.5
O60331	PIP5K1C	Phosphati	5	3	3	2	668	73.2
P17302	GJA1	Gap junct	4	1	3	1	382	43
K7ERY2	YIF1B	Protein Y	16	2	2	2	231	25.1
P63165	SUMO1	Small ubi	39	5	5	5	101	11.6
F8WCT7	SLC35F6	Solute ca	27	1	2	1	51	5.7
A0A2R8Y4D	ARHGEF17	Rho guani	3	3	3	3	1044	114.1
Q6P3X3	TTC27	Tetratric	4	4	4	4	843	96.6
A0A0A0MQX	MYO10	Unconvent	1	3	6	2	2069	238.4
A8K727	PLEKHA2	Pleckstri	7	3	3	3	425	47.2
Q9Y3D8	AK6	Adenylate	15	3	3	3	172	20
P51970	NDUFA8	NADH dehy	20	3	4	3	172	20.1
P56377	AP1S2	AP-1 comp	18	3	3	3	157	18.6
F5GXS0	C4B	Complemen	1	3	4	3	1698	187.6
Q13033	STRN3	Striatin-	4	3	3	2	797	87.2
Q7Z4N8	P4HA3	Prolyl 4-	6	3	3	3	544	61.1
P48200	IREB2	Iron-resp	3	3	3	3	963	105
E7EWX8	MGLL	Monoglyce	9	2	3	2	277	30.7
O75157	TSC22D2	TSC22 dom	3	3	4	1	780	79.2
D6RAA6	TMEM33	Transmemb	12	3	5	3	222	25.2
P41226	UBA7	Ubiquitin	4	3	3	3	1012	111.6
Q66K14	TBC1D9B	TBC1 doma	2	2	3	2	1250	140.4
Q9UIC8	LCMT1	Leucine c	7	2	3	2	334	38.4
E9PQW1	CARD16	Caspase r	27	3	3	1	95	10.3
O15530	PDPK1	3-phospho	7	3	3	3	556	63.1
Q9UBB9	TFIP11	Tuftelin-	5	3	3	3	837	96.8
Q9NWZ3	IRAK4	Interleuk	6	3	3	3	460	51.5
Q7LGA3	HS2ST1	Heparan s	9	3	5	3	356	41.9
Q9GZM5	YIPF3	Protein Y	5	2	3	2	350	38.2
H3BQI7	HSDL1	Inactive	12	1	2	1	108	12.1
P51151	RAB9A	Ras-relat	12	2	2	2	201	22.8
Q8IXM3	MRPL41	39S ribos	19	2	3	2	137	15.4
Q9BV19	C1orf50	Uncharact	16	2	2	2	199	21.9
B7WPL0	RIC8B	Synembryn	4	2	3	1	560	63.5
J9JIE6	TMCO1	Calcium l	10	3	4	3	239	27.1
Q7Z7F7	MRPL55	39S ribos	24	3	3	3	128	15.1
P33897	ABCD1	ATP-bindi	4	3	3	2	745	82.9
Q9UJX2	CDC23	Cell divi	6	3	3	3	597	68.8
Q15274	QPRT	Nicotinat	12	4	5	4	297	30.8
Q9H6X2	ANTXR1	Anthrax t	6	3	3	2	564	62.7
O95980	RECK	Reversion	4	4	4	4	971	106.4
C9JST7	YIF1A	Protein Y	6	1	2	1	199	21.7
O95425	SVIL	Supervill	2	3	3	3	2214	247.6
Q13232	NME3	Nucleosid	15	3	4	3	169	19
Q4TT34	NME4	Nucleosid	15	2	2	2	195	21.5
A0A2R8YDQ		Uncharact	5	3	3	3	599	68.7
Q9NXH9	TRMT1	tRNA (gua	5	2	2	2	659	72.2
Q9NPA0	EMC7	ER membra	13	3	4	3	242	26.5

Q9H013	ADAM19	Disintegr	4	3	3	3	955	104.9
Q08722	CD47	Leukocyte	9	3	4	3	323	35.2
A0A0X1KG7	NELFB	Negative	4	3	3	3	628	70
Q5VZM2	RRAGB	Ras-relat	7	3	4	3	374	43.2
Q5T1J5	CHCHD2P9	Putative	9	1	2	1	151	15.5
P49356	FNTB	Protein f	6	2	2	2	437	48.7
C9JLU1	POLR2H	DNA-direc	11	2	3	2	148	16.9
Q96LW7	CARD19	Caspase r	11	3	4	3	228	25.6
Q86WB0	ZC3HC1	Nuclear-i	7	3	3	3	502	55.2
Q8TDB4	MGARP	Protein M	11	3	3	3	240	25.4
P82932	MRPS6	28S ribos	22	3	3	3	125	14.2
A0A0D9SG7	UBAP1	Ubiquitin	8	4	4	4	528	57.8
E9PBC1	EPN2	Epsin-2 C	4	2	3	2	484	53
Q6RW13	AGTRAP	Type-1 an	14	1	2	1	159	17.4
Q12846	STX4	Syntaxin-	12	3	3	3	297	34.2
Q8NBI6	XXYLT1	Xyloside	10	4	4	4	393	43.8
Q13257	MAD2L1	Mitotic s	16	4	4	4	205	23.5
O95166	GABARAP	Gamma-ami	15	2	3	1	117	13.9
Q9UJX4	ANAPC5	Anaphase-	4	2	2	2	755	85
Q96BH1	RNF25	E3 ubiqui	5	2	3	2	459	51.2
P02649	APOE	Apolipopr	9	3	4	3	317	36.1
P47712	PLA2G4A	Cytosolic	5	4	4	4	749	85.2
A8MX75	ERCC2	General t	3	2	3	2	706	80.5
O60551	NMT2	Glycylpep	7	4	4	1	498	56.9
Q86YS6	RAB43	Ras-relat	17	3	3	2	212	23.3
X6R2S6	SPCS1	Signal pe	10	2	4	2	169	18.3
P35244	RPA3	Replicati	24	2	3	2	121	13.6
Q96BP3	PPWD1	Peptidylp	6	4	4	4	646	73.5
Q9Y2Z4	YARS2	Tyrosine-	12	4	4	4	477	53.2
O15213	WDR46	WD repeat	4	2	2	2	610	68
O95248	SBF1	Myotubula	1	3	3	3	1868	208.3
Q9H4L7	SMARCAD1	SWI/SNF-r	2	2	2	2	1026	117.3
Q8WXI9	GATAD2B	Transcrip	3	2	3	1	593	65.2
Q92925	SMARCD2	SWI/SNF-r	6	2	2	2	531	58.9
Q8TD55	PLEKHO2	Pleckstri	6	2	2	2	490	53.3
Q9BV79	MECR	Enoyl-[ac	6	2	3	2	373	40.4
J3QL71	SCRN2	Secernin-	6	2	2	2	433	47.5
Q5EBL4	RILPL1	RILP-like	9	3	3	3	403	47.1
Q8WVQ1	CANT1	Soluble c	8	3	3	3	401	44.8
S4R347	FNBP1L	Formin-bi	7	4	4	3	609	70.5
Q03405	PLAUR	Urokinase	9	3	4	3	335	37
P08651	NFIC	Nuclear f	5	2	2	2	508	55.6
A0A2R8YGH	AP1S1	AP comple	18	3	3	3	157	18.6
Q9BQ70	TCF25	Transcrip	6	3	3	3	676	76.6
Q9H1E5	TMX4	Thioredox	7	2	2	2	349	38.9
H0Y8X6	NEDD4	E3 ubiqui	4	3	3	3	910	104.7
Q8IWA4	MFN1	Mitofusin	4	3	3	2	741	84.1
H0YN65	CHST14	Carbohydr	10	4	4	4	351	39.9
P17405	SMPD1	Sphingomy	4	3	3	3	629	69.7
Q13042	CDC16	Cell divi	5	3	3	3	620	71.6
A0A087WYV	SYNJ2BP-C	SYNJ2BP-C	22	3	4	3	182	20.5

Q03519	TAP2	Antigen p	5	4	4	4	686	75.6
Q9GZQ8	MAP1LC3B	Microtubu	22	3	4	3	125	14.7
O94829	IPO13	Importin-	5	3	3	3	963	108.1
O95671	ASMTL	N-acetyls	5	4	4	4	621	68.8
A0A0A0MQV	FGF2	Fibroblas	10	3	3	3	288	30.7
Q8TEA8	DTD1	D-aminoac	11	2	2	2	209	23.4
O60306	AQR	RNA helic	3	4	4	4	1485	171.2
Q5SZE1	CERS2	Ceramide	11	3	3	3	304	36.4
B5MD46	TBC1D10A	TBC1 doma	6	3	3	3	420	47.9
P51784	USP11	Ubiquitin	3	3	3	3	963	109.7
O96007	MOCS2	Molybdopt	14	2	2	2	188	20.9
P62837	UBE2D2	Ubiquitin	12	2	7	2	147	16.7
Q66LE6	PPP2R2D	Serine/th	10	3	3	1	453	52
A3KFL1	EXOSC2	Exosome c	17	3	4	3	200	22.1
H7C2N1	PTMA	Prothymos	9	1	2	1	148	15.8
Q9BY67	CADM1	Cell adhe	6	3	3	3	442	48.5
P86790	CCZ1B	Vacuolar	7	3	3	3	482	55.8
Q99418	CYTH2	Cytohesin	6	2	2	2	400	46.5
Q9NQH7	XPNPEP3	Xaa-Pro a	6	2	2	2	507	57
Q9HCS7	XAB2	Pre-mRNA-	6	4	4	4	855	99.9
A0A087WZX	NDUFB6	NADH dehy	26	2	4	2	97	11.7
Q96CP2	FLYWCH2	FLYWCH fa	22	2	2	2	140	14.6
Q9BT22	ALG1	Chitobios	8	3	3	3	464	52.5
Q9BV20	MRI1	Methylthi	8	3	3	3	369	39.1
O43676	NDUFB3	NADH dehy	18	2	6	2	98	11.4
H3BSE1	TBC1D10B	TBC1 doma	14	3	3	3	184	21.1
M0R1E3	NAT14	N-acetylt	17	2	2	2	173	17.8
P08253	MMP2	72 kDa ty	6	3	3	3	660	73.8
Q13017	ARHGAP5	Rho GTPas	2	4	4	4	1502	172.4
C9JEL3	EIF4E2	Eukaryoti	11	2	3	2	213	24.6
Q8IXI2	RHOT1	Mitochond	3	2	3	1	618	70.7
Q92979	EMG1	Ribosomal	11	2	2	2	244	26.7
G8JLK1	USE1	Vesicle t	10	3	4	3	256	29.1
Q9BZJ0	CRNKL1	Crooked n	2	2	2	2	848	100.4
H3BTP8	FAM192A	Protein F	24	2	2	2	130	16
Q8NFP7	NUDT10	Diphospho	18	2	2	1	164	18.5
B1APM4	SOAT1	Sterol O-	12	2	2	2	260	29.9
Q9GZP9	DERL2	Derlin-2	17	2	2	2	239	27.5
Q9NZD8	SPG21	Maspardin	12	3	3	3	308	34.9
Q6PD74	AAGAB	Alpha- an	9	3	3	3	315	34.6
A0A096LNH	DOCK1	Dedicator	2	3	3	2	1886	217.6
A0A0B4J1V	PPAN-P2RY	HCG203999	3	3	4	3	794	87.9
Q8N5N7	MRPL50	39S ribos	15	2	2	2	158	18.3
Q5T160	RARS2	Probable	5	3	3	3	578	65.5
O95149	SNUPN	Snurporti	9	4	4	3	360	41.1
F8WCT1	ARL6IP4	ADP-ribos	5	1	2	1	229	25.6
Q13641	TPBG	Trophobla	6	3	3	3	420	46
P34949	MPI	Mannose-6	12	3	3	3	423	46.6
A0A0J9YX6	DNAJB6	DnaJ homo	7	3	3	2	334	36.6
Q9BWH2	FUNDC2	FUN14 dom	13	2	2	2	189	20.7
Q8IWA0	WDR75	WD repeat	4	3	3	3	830	94.4

P51884	LUM	Lumican C	9	3	3	3	338	38.4
P16455	MGMT	Methylate	18	3	4	3	207	21.6
Q53FP2	TMEM35A	Transmemb	14	2	3	2	167	18.4
K7ERC8	KDSR	3-ketodih	18	4	4	4	298	32.7
Q8N3X1	FNBP4	Formin-bi	3	3	3	3	1017	110.2
P56381	ATP5F1E	ATP synth	59	4	6	4	51	5.8
Q9H0V9	LMAN2L	VIP36-lik	10	3	3	3	348	39.7
O95197	RTN3	Reticulon	2	2	3	2	1032	112.5
A0A2R8Y6C	CLPB	Caseinoly	8	2	2	1	382	43.3
Q9Y487	ATP6V0A2	V-type pr	5	4	4	4	856	98
E7EX70	POLR1E	DNA-direc	23	3	3	1	141	15.9
Q9Y3B9	RRP15	RRP15-lik	10	3	4	3	282	31.5
F5H225	EOGT	EGF domai	13	3	3	3	206	24.1
Q92990	GLMN	Glomulin	4	3	3	3	594	68.2
O00391	QSOX1	Sulfhydry	5	3	3	3	747	82.5
O00443	PIK3C2A	Phosphati	1	1	2	1	1686	190.6
P00374	DHFR	Dihydrofo	14	2	2	2	187	21.4
Q9GZT6	CCDC90B	Coiled-co	7	2	2	2	254	29.5
Q96C23	GALM	Aldose 1-	14	4	4	4	342	37.7
Q5R3I4	TTC38	Tetratric	9	4	4	4	469	52.8
Q5TH30	NDRG3	NDRG fami	5	2	2	2	388	42.8
Q8NBJ4	GOLM1	Golgi mem	6	4	5	4	401	45.3
Q4G0J3	LARP7	La-relate	5	4	4	4	582	66.9
G8JLB3	PUS1	tRNA pseu	8	3	3	3	384	42.9
Q14684	RRP1B	Ribosomal	5	3	3	3	758	84.4
P36404	ARL2	ADP-ribos	11	2	4	2	184	20.9
Q92871	PMM1	Phosphoma	15	4	4	3	262	29.7
Q13049	TRIM32	E3 ubiqui	3	2	2	2	653	71.9
Q9UBK9	UXT	Protein U	20	3	3	3	157	18.2
B8ZZ77	PPIL3	Peptidyl-	18	3	3	3	157	17.7
P05412	JUN	Transcrip	13	4	4	4	331	35.7
A6PW57	PIP5K1A	Phosphati	5	3	3	2	550	61.2
Q14739	LBR	Lamin-B r	6	4	4	4	615	70.7
P53985	SLC16A1	Monocarbo	4	3	3	3	500	53.9
Q15750	TAB1	TGF-beta-	7	3	3	3	504	54.6
H7C0B3	TMEM87B	Transmemb	15	3	3	3	184	21.4
Q9NP77	SSU72	RNA polym	12	2	2	2	194	22.6
Q495W5	FUT11	Alpha-(1,	7	3	3	3	492	55.8
B9ZVT1	RBM12B	RNA-bindi	4	3	3	3	881	102.6
F8W1S1	KRT74	Keratin,	5	3	10	1	543	59.4
P85037	FOXK1	Forkhead	4	4	5	4	733	75.4
A0A024R6I	SERPINA1	Alpha-1-a	5	2	2	2	418	46.7
O14925	TIMM23	Mitochond	17	3	3	3	209	21.9
A0A1B0GUA	KIF13A	Kinesin-l	1	2	2	2	1845	207.1
O60513	B4GALT4	Beta-1,4-	13	4	4	4	344	40
Q5T653	MRPL2	39S ribos	7	2	2	2	305	33.3
Q14112	NID2	Nidogen-2	2	3	3	3	1375	151.2
Q9BSH4	TACO1	Translati	10	2	2	2	297	32.5
P49459	UBE2A	Ubiquitin	18	2	2	2	152	17.3
Q9BUT1	BDH2	3-hydroxy	12	3	3	3	245	26.7
Q8IYB8	SUPV3L1	ATP-depen	2	1	2	1	786	87.9

O14929	HAT1	Histone a	8	3	4	3	419	49.5
O75607	NPM3	Nucleopla	26	4	4	4	178	19.3
A0A0B4J23	IGLL5	Immunoglc	7	1	2	1	215	23.1
Q5VWZ2	LYPLAL1	Lysophosp	10	3	4	3	237	26.3
Q96F24	NRBF2	Nuclear r	9	3	3	3	287	32.4
O75531	BANF1	Barrier-t	36	2	3	2	89	10.1
O95235	KIF20A	Kinesin-l	3	2	2	2	890	100.2
Q8TED0	UTP15	U3 small	6	3	3	3	518	58.4
P53384	NUBP1	Cytosolic	8	2	3	2	320	34.5
Q5VXN0	RPF2	Ribosome	12	3	3	3	214	24.6
Q9UHD2	TBK1	Serine/th	3	3	3	1	729	83.6
Q96S44	TP53RK	EKC/KEOPS	8	2	3	2	253	28.1
Q9H0C8	ILKAP	Integrin-	6	3	3	3	392	42.9
Q9NPD3	EXOSC4	Exosome c	8	2	3	2	245	26.4
Q6ZSJ8	C1orf122	Uncharact	23	2	2	2	110	11.5
P35250	RFC2	Replicati	7	3	3	3	354	39.1
Q9UJ83	HACL1	2-hydroxy	6	3	4	3	578	63.7
P15289	ARSA	Arylsulfa	11	4	4	3	507	53.6
Q8NB16	MLKL	Mixed lin	9	2	2	2	471	54.4
Q9Y6D9	MAD1L1	Mitotic s	4	4	4	4	718	83
M0QWZ7	SARS2	Serine--t	8	3	3	3	518	58.1
Q8WUI4	HDAC7	Histone d	3	3	3	3	952	102.9
Q8IYB1	MB21D2	Protein M	4	2	2	2	491	55.8
Q9UIW2	PLXNA1	Plexin-A1	1	3	3	3	1896	210.9
Q9NVX2	NLE1	Notchless	5	2	2	2	485	53.3
E9PEP6	NRP1	Neuropili	4	2	2	2	906	101.2
E9PRK2	NARS2	Probable	8	2	2	2	241	27.2
P53609	PGGT1B	Geranylge	8	3	3	3	377	42.3
Q96J01	THOC3	THO compl	9	3	3	3	351	38.7
Q96DE0	NUDT16	U8 snoRNA	16	3	3	2	195	21.3
Q9BSC4	NOL10	Nucleolar	5	3	3	3	688	80.3
O14730	RIOK3	Serine/th	4	2	3	2	519	59.1
K7EJB0	NFIX	Nuclear f	15	2	2	2	183	21.2
M0QZD9	BRD4	Bromodoma	3	2	2	2	572	63.7
O60234	GMFG	Glia matu	13	2	2	1	142	16.8
Q7Z3B1	NEGR1	Neuronal	4	1	2	1	354	38.7
P17900	GM2A	Gangliosi	12	3	3	3	193	20.8
Q9BSL1	UBAC1	Ubiquitin	5	2	3	2	405	45.3
A0A0U1RQX	CBL	E3 ubiqui	3	3	3	3	882	96.8
P06280	GLA	Alpha-gal	8	2	2	2	429	48.7
Q9NWY4	HPF1	Histone P	6	2	2	2	346	39.4
P19634	SLC9A1	Sodium/hy	4	2	2	2	815	90.7
A6NIH7	UNC119B	Protein u	12	2	2	2	251	28.1
Q9C037	TRIM4	E3 ubiqui	5	2	2	2	500	57.4
D6RHX2	ELMOD2	ELMO doma	24	2	2	2	96	11.2
P78536	ADAM17	Disintegr	3	3	3	3	824	93
Q92968	PEX13	Peroxisom	5	2	2	2	403	44.1
O43752	STX6	Syntaxin-	9	2	2	2	255	29.2
O95749	GGPS1	Geranylge	10	3	3	3	300	34.8
J3QL56	SCO1	Protein S	15	3	3	3	270	30.2
Q8NC54	KCT2	Keratinoc	9	2	2	2	265	29.2

P55011	SLC12A2	Solute ca	3	3	3	3	1212	131.4
O15484	CAPN5	Calpain-5	5	2	3	2	640	73.1
Q96D71	REPS1	RalBP1-as	3	3	3	3	796	86.6
Q7L8J4	SH3BP5L	SH3 domai	7	3	3	3	393	43.5
O75792	RNASEH2A	Ribonucle	8	2	2	2	299	33.4
Q9H3Q1	CDC42EP4	Cdc42 eff	9	3	3	3	356	38
A0A1B0GW9	IWS1	Protein I	15	4	4	4	207	24
B5MC51	LIMK2	LIM domai	4	2	3	1	629	71.1
Q9UL26	RAB22A	Ras-relat	16	3	4	2	194	21.8
H0YAX3	MRPL13	39S ribos	51	2	2	2	47	5.8
P62308	SNRPG	Small nuc	25	2	5	2	76	8.5
P32856	STX2	Syntaxin-	8	2	2	2	288	33.3
Q5TC84	OGFRL1	Opioid gr	6	3	3	3	451	51.2
Q9BT73	PSMG3	Proteasom	23	2	3	2	122	13.1
Q6UW02	CYP20A1	Cytochrom	5	2	2	2	462	52.4
O95183	VAMP5	Vesicle-a	11	1	2	1	116	12.8
Q9C0E8	LNPK	Endoplasm	4	2	4	2	428	47.7
F5GXE4	ATE1	Arginyl-t	6	2	3	2	511	58.1
Q9NS00	C1GALT1	Glycoprot	7	2	2	2	363	42.2
Q8N999	C12orf29	Uncharact	6	2	2	2	325	37.5
E7EN73	KIAA0319L	Dyslexia-	3	3	3	3	1026	113.2
A0A2R8YEK	COL4A3BP	Collagen	5	3	3	3	567	64.3
Q14410	GK2	Glycerol	3	2	3	1	553	60.6
P52756	RBM5	RNA-bindi	3	2	2	2	815	92.1
Q6PML9	SLC30A9	Zinc tran	5	3	3	3	568	63.5
Q96II8	LRCH3	Leucine-r	3	2	3	2	777	86
Q9NSI2	FAM207A	Protein F	15	2	2	2	230	25.4
G3V4T2	PABPN1	Polyadeny	10	3	3	3	178	20.2
Q9UJ72	ANXA10	Annexin A	8	2	2	2	324	37.3
B2WTI3	JMJD6	Bifunctic	4	1	2	1	335	39.2
Q99757	TXN2	Thioredox	13	2	3	2	166	18.4
Q56VL3	OCIAD2	OCIA doma	27	4	4	4	154	16.9
A0A0C4DGS	NDUFA6	NADH dehy	19	3	4	3	128	15.1
A0A024RAC	ELOA	Elongin-A	4	3	3	3	772	87.2
Q9UBL3	ASH2L	Set1/Ash2	4	2	2	2	628	68.7
A0A0A0MTC	STAU2	Double-st	7	3	3	3	504	55.4
Q93096	PTP4A1	Protein t	18	3	3	1	173	19.8
Q9H9T3	ELP3	Elongator	6	3	3	3	547	62.2
H0YLF3	B2M	Beta-2-mi	14	1	3	1	71	8.5
Q9H061	TMEM126A	Transmemb	11	2	2	2	195	21.5
O14613	CDC42EP2	Cdc42 eff	13	2	2	2	210	22.5
Q8WUJ3	CEMIP	Cell migr	2	3	3	3	1361	152.9
Q86X83	COMMD2	COMM doma	12	3	3	3	199	22.7
A0A2R8YDT	GLUL	Glutamine	5	3	3	3	507	57.1
Q8WVJ2	NUDCD2	NudC doma	18	2	2	2	157	17.7
P06702	S100A9	Protein S	18	2	3	2	114	13.2
E5RHP7	CA1	Carbonic	5	1	3	1	251	27.7
Q99735	MGST2	Microsoma	10	1	2	1	147	16.6
Q8TAD7	OCC1	Overexpre	21	1	1	1	63	6.4
O14662	STX16	Syntaxin-	10	3	4	3	325	37
O00488	ZNF593	Zinc fing	17	2	2	2	134	15.2

Q9Y4C8	RBM19	Probable	2	2	2	2	960	107.3
Q9BXW6	OSBPL1A	Oxysterol	3	2	2	2	950	108.4
Q8TBM8	DNAJB14	DnaJ homo	6	2	2	2	379	42.5
Q9UNH6	SNX7	Sorting n	9	4	4	4	387	45.3
Q8IZV5	RDH10	Retinol d	9	3	3	3	341	38.1
F8W038	C17orf49	Chromosom	13	2	4	2	157	16.3
Q93100	PHKB	Phosphory	2	3	3	3	1093	124.8
Q9NUJ3	TCP11L1	T-complex	5	2	2	2	509	57
Q92947	GCDH	Glutaryl-	6	3	3	3	438	48.1
Q6PI48	DARS2	Aspartate	4	3	3	3	645	73.5
J3KSG2	SPECC1	Cytospin-	5	2	2	2	534	60.1
E5RJY0	PIK3R1	Phosphati	8	1	2	1	137	16.3
Q9NSQ0	RRP7BP	Putative	22	2	2	2	103	12.6
H3BUU9	CDH11	Cadherin-	4	2	3	2	670	73.7
O43427	FIBP	Acidic fi	5	2	2	2	364	41.9
Q5T440	IBA57	Putative	13	2	2	2	356	38.1
E9PDN5	DMD	Dystrophi	1	2	2	1	3681	425.8
Q13614	MTMR2	Myotubula	4	3	3	3	643	73.3
P57772	EEFSEC	Selenocys	4	2	2	2	596	65.3
O95071	UBR5	E3 ubiqui	1	3	3	3	2799	309.2
P00846	MT-ATP6	ATP synth	4	1	2	1	226	24.8
E9PIT5	SSH3	Protein p	6	1	2	1	219	24.7
Q969Q5	RAB24	Ras-relat	13	2	2	2	203	23.1
P08574	CYC1	Cytochrom	7	2	3	2	325	35.4
Q96MW1	CCDC43	Coiled-co	11	3	3	3	224	25.2
Q96B36	AKT1S1	Proline-r	5	1	2	1	256	27.4
A0A2R8Y4M	SPG7	Paraplegi	5	2	2	2	383	42.8
Q9Y2C4	EXOG	Nuclease	9	3	3	3	368	41.1
H3BVH7	RAB27A	Ras-relat	14	2	2	2	146	16.7
B1AKC9	EPHB2	Ephrin ty	2	2	2	1	946	105.5
A0A087X1U	MECP2	Methyl-Cp	6	2	2	2	324	34.9
Q96GD0	PDXP	Pyridoxal	8	2	2	2	296	31.7
Q9BTE3	MCMBP	Mini-chro	4	3	3	3	642	72.9
A0A087WTU	TEX264	Testis-ex	10	2	2	2	239	26
I3L2C7	GEMIN4	Gem-assoc	2	2	2	2	1047	118.7
Q8WUH1	CHURC1	Protein C	15	2	3	2	139	16.1
P30536	TSPO	Transloca	9	2	4	2	169	18.8
Q3ZCW2	LGALSL	Galectin-	17	2	2	2	172	19
Q9Y333	LSM2	U6 snRNA-	27	2	2	2	95	10.8
Q9BRG1	VPS25	Vacuolar	10	2	3	2	176	20.7
O75380	NDUFS6	NADH dehy	12	1	1	1	124	13.7
Q96CP6	GRAMD1A	GRAM doma	2	2	2	2	724	80.6
P82664	MRPS10	28S ribos	9	1	2	1	201	23
Q9H2K8	TAOK3	Serine/th	3	3	4	2	898	105.3
Q9NQT5	EXOSC3	Exosome c	8	2	2	2	275	29.6
O43709	BUD23	Probable	11	2	2	2	281	31.9
P51668	UBE2D1	Ubiquitin	7	1	2	1	147	16.6
O94806	PRKD3	Serine/th	2	2	2	2	890	100.4
P0CG30	GSTT2B	Glutathio	13	2	3	2	244	27.5
Q86SR1	GALNT10	Polypepti	4	3	4	3	603	68.9
Q96QC0	PPP1R10	Serine/th	3	4	4	4	940	99

C9J1S9	BCS1L	Mitochond	15	2	2	2	150	17.1
Q5QJE6	DNTTIP2	Deoxynucl	3	2	2	2	756	84.4
Q96EY8	MMAB	Cob(I)yri	13	2	3	2	250	27.4
Q96QZ7	MAGI1	Membrane-	2	2	2	1	1491	164.5
H0YNI7	TLE3	Transduci	6	2	2	2	353	38.3
A0A087WZ1	RAVER1	Ribonucle	4	2	2	2	739	77.8
Q9UJA5	TRMT6	tRNA (ade	6	3	3	3	497	55.8
Q9NXG6	P4HTM	Transmemb	3	1	2	1	502	56.6
Q9GZQ3	COMMD5	COMM doma	10	2	2	2	224	24.7
O95273	CCNDBP1	Cyclin-D1	4	1	2	1	360	40.2
A0A087WUC	ERGIC2	Endoplasm	7	3	4	3	386	43.4
E7ESK6	SDC2	Syndecan	15	2	2	2	165	18.4
Q9Y2R4	DDX52	Probable	4	2	2	2	599	67.5
Q9ULX3	NOB1	RNA-bindi	6	3	3	3	412	46.6
A1A4S6	ARHGAP10	Rho GTPas	3	2	2	2	786	89.3
Q9UH62	ARMCX3	Armadillo	8	3	3	3	379	42.5
A0A0G2JNK	TSEN34	tRNA-spli	10	2	3	2	252	27.3
Q8NBU5	ATAD1	ATPase fa	6	2	2	2	361	40.7
P26374	CHML	Rab prote	3	2	2	1	656	74
P28799	GRN	Granulins	5	3	3	3	593	63.5
Q9NUG6	PDRG1	p53 and D	10	1	2	1	133	15.5
Q96GA3	LTV1	Protein L	6	2	2	2	475	54.8
Q9UPU5	USP24	Ubiquitin	1	2	2	2	2620	294.2
Q68E01	INTS3	Integrato	2	2	2	2	1043	118
P49721	PSMB2	Proteasom	12	3	5	3	201	22.8
Q8WU76	SCFD2	Sec1 fami	4	2	2	2	684	75.1
K4DI92	RWDD4	RWD domai	10	2	2	2	187	21.1
P82914	MRPS15	28S ribos	7	2	2	2	257	29.8
Q96L35	EPHB4	EPH recep	3	2	2	1	935	102.5
Q9UNZ5	C19orf53	Leydig ce	17	2	2	2	99	10.6
P78381	SLC35A2	UDP-galac	3	1	2	1	396	41.3
Q9GZT4	SRR	Serine ra	9	2	2	2	340	36.5
P24386	CHM	Rab prote	2	2	2	1	653	73.4
Q92629	SGCD	Delta-sar	8	2	2	2	289	32.1
Q9HBL7	PLGRKT	Plasminog	18	3	3	3	147	17.2
P0DPB6	POLR1D	DNA-direc	16	2	2	2	133	15.2
Q9Y5J6	TIMM10B	Mitochond	23	2	2	2	103	11.6
Q9Y5A7	NUB1	NEDD8 ult	3	2	2	2	615	70.5
P14174	MIF	Macrophag	14	2	5	2	115	12.5
Q9Y6M7	SLC4A7	Sodium bi	2	2	3	2	1214	136
E9PC69	MARK2	Non-speci	2	2	2	2	778	86.6
Q13158	FADD	FAS-assoc	10	2	2	2	208	23.3
J3KNN5	DDX41	Probable	4	3	3	3	640	71.6
C9JRD2	DNAJB2	DnaJ homo	11	2	2	1	228	25.4
Q13541	EIF4EBP1	Eukaryoti	22	3	3	3	118	12.6
A0A0B4J28	TRAPPC2L	Trafficki	21	3	4	3	109	12.7
P11274	BCR	Breakpoin	2	3	3	3	1271	142.7
Q9UIV1	CNOT7	CCR4-NOT	8	2	2	2	285	32.7
P61964	WDR5	WD repeat	8	2	2	2	334	36.6
Q96EL3	MRPL53	39S ribos	21	2	2	2	112	12.1
Q86U44	METTL3	N6-adenos	4	2	2	2	580	64.4

Q9HBM1	SPC25	Kinetochc	10	2	3	2	224	26.1
A0A087WTCCBWD1		COBW doma	15	2	2	2	160	17.4
P10398	ARAF	Serine/th	4	2	2	2	606	67.5
Q92997	DVL3	Segment p	3	2	2	2	716	78
P16591	FER	Tyrosine-	2	2	2	2	822	94.6
H7C5T8	MAGI1	Membrane-	3	2	2	1	1014	110.7
Q9BRT3	MIEN1	Migratior	16	2	3	2	115	12.4
Q9BUK6	MSTO1	Protein m	7	3	3	3	570	61.8
Q9UPN9	TRIM33	E3 ubiqui	2	2	2	2	1127	122.5
Q9BRT6	LLPH	Protein L	19	2	2	2	129	15.2
Q9BXB4	OSBPL11	Oxysterol	3	2	3	2	747	83.6
P24468	NR2F2	COUP trar	6	3	3	2	414	45.5
P10746	UROS	Uroporphy	13	2	2	2	265	28.6
Q9Y5Q0	FADS3	Fatty aci	8	3	3	3	445	51.1
O15066	KIF3B	Kinesin-l	2	2	2	2	747	85.1
Q9Y2Q9	MRPS28	28S ribos	10	2	2	1	187	20.8
Q86TB9	PATL1	Protein F	3	2	2	2	770	86.8
A0A2R8Y7ECCDC88A		Girdin OS	1	2	2	2	1604	185.4
Q9H5Q4	TFB2M	Dimethyla	4	2	2	2	396	45.3
P57081	WDR4	tRNA (gua	5	2	2	2	412	45.5
J3KT75	MPDU1	Mannose-F	6	1	3	1	154	16.5
Q13057	COASY	Bifunctic	3	2	2	2	564	62.3
Q9Y2V2	CARHSP1	Calcium-r	11	1	2	1	147	15.9
Q96J84	KIRREL1	Kin of IR	5	2	2	2	757	83.5
Q7KYR7	BTN2A1	Butyrophi	4	2	3	2	527	59.6
O94822	LTN1	E3 ubiqui	2	4	4	4	1766	200.4
Q9BRP4	PAAF1	Proteasom	7	3	3	3	392	42.2
Q5T2E6	ARMH3	Armadillc	3	3	3	3	689	78.7
Q96HW7	INTS4	Integratc	3	2	2	2	963	108.1
Q5VSL9	STRIP1	Striatin-	2	1	2	1	837	95.5
Q8N335	GPD1L	Glycerol-	6	2	2	2	351	38.4
H7BXJ4	FCHO2	F-BAR dom	7	3	3	3	378	43.4
J3QSU6	TNC	Tenascin	1	2	2	2	2019	220.7
Q7Z6J0	SH3RF1	E3 ubiqui	2	2	2	2	888	93.1
F8W733	BABAM2	BRISC anc	5	1	2	1	284	32
P42858	HTT	Huntingti	1	2	2	2	3142	347.4
P49914	MTHFS	5-formylt	13	3	3	3	203	23.2
G3V583	FAM177A1	Protein F	19	3	3	3	140	16.3
Q9HBR0	SLC38A10	Putative	2	2	2	2	1119	119.7
Q7Z7L1	SLFN11	Schlafen	2	2	2	1	901	102.8
Q9P2B2	PTGFRN	Prostagla	3	2	2	2	879	98.5
E5RK00	DCTN6	Dynactin	11	2	2	2	170	18.4
Q12972	PPP1R8	Nuclear i	7	2	2	2	351	38.5
Q06546	GABPA	GA-bindir	5	2	2	2	454	51.3
Q12824	SMARCB1	SWI/SNF-r	9	2	2	2	385	44.1
Q4KMQ2	ANO6	Anoctamir	3	3	4	3	910	106.1
Q92609	TBC1D5	TBC1 doma	3	2	2	2	795	88.9
Q10570	CPSF1	Cleavage	1	2	2	2	1443	160.8
P11169	SLC2A3	Solute ca	2	1	2	1	496	53.9
Q5QPA5	MRPS18A	39S ribos	8	2	2	2	263	29.6
G3V5Z3	PPP4R3A	Serine/th	4	3	3	3	706	82

Q9NZR1	TMOD2	Tropomodu	7	3	3	2	351	39.6
Q86UA1	PRPF39	Pre-mRNA-	5	3	3	3	669	78.4
Q969S9	GFM2	Ribosome-	3	2	2	2	779	86.5
O43674	NDUFB5	NADH dehy	17	2	2	2	189	21.7
P62861	FAU	40S ribos	17	1	4	1	59	6.6
P31273	HOXC8	Homeobox	9	2	2	2	242	27.7
F5H5N2	ISCU	Iron-sulf	16	3	3	3	154	16.6
Q4G148	GXYLT1	Glucoside	4	2	2	2	440	50.5
Q9HBI1	PARVB	Beta-parv	6	2	2	1	364	41.7
Q6P1M0	SLC27A4	Long-chai	4	2	2	2	643	72
Q9Y5J5	PHLDA3	Pleckstri	14	2	2	2	127	13.9
Q9NR19	ACSS2	Acetyl-cc	3	1	2	1	701	78.5
A6NML8	DIAPH2	Diaphanou	3	3	3	3	1096	124.8
H0YAJ5	APBB2	Amyloid-b	3	2	2	2	728	80
O14949	UQCRQ	Cytochrom	22	2	2	2	82	9.9
Q9BRS8	LARP6	La-relate	4	2	2	2	491	54.7
Q969E8	TSR2	Pre-rRNA-	11	2	2	2	191	20.9
Q9UBV2	SEL1L	Protein s	2	2	2	2	794	88.7
P40938	RFC3	Replicati	5	2	2	2	356	40.5
Q8N241	HSPB7	Heat shoc	9	2	2	2	245	27.4
Q8IU81	IRF2BP1	Interferc	3	2	2	2	584	61.6
Q9H7B4	SMYD3	Histone-l	5	2	2	2	428	49.1
B3KNS4	ERVK3-1	Endogenou	26	2	2	2	109	11.6
B7ZAX5	GALK2	cDNA, FLJ	5	2	2	2	434	47.6
K7ELH8	DPY19L3	Probable	3	2	2	2	688	79.5
Q96EK9	KTI12	Protein K	9	2	2	2	354	38.6
Q96GC5	MRPL48	39S ribos	9	2	2	2	212	23.9
Q14012	CAMK1	Calcium/c	7	2	2	2	370	41.3
Q8IVL5	P3H2	Prolyl 3-	3	2	5	1	708	80.9
O14684	PTGES	Prostagla	12	2	2	2	152	17.1
Q9H7E9	C8orf33	UPF0488 p	8	2	2	2	229	25
Q7Z392	TRAPPC11	Trafficki	2	2	2	2	1133	128.8
Q14746	COG2	Conserved	4	2	2	2	738	83.2
Q8WUH2	TGFBRAP1	Transform	2	2	2	2	860	97.1
H0YL10	CD276	CD276 ant	11	2	2	2	199	21.4
P01034	CST3	Cystatin-	11	1	1	1	146	15.8
K7ENR6	PSMG2	Proteason	6	2	3	2	240	26.7
Q9NUQ3	TXLNG	Gamma-tax	5	2	2	2	528	60.5
E5RFR7	TPD52	Tumor prc	20	2	2	1	111	12.4
M0QXD5	PIH1D1	PIH1 doma	11	2	2	2	236	26.4
Q8TD30	GPT2	Alanine a	4	2	2	2	523	57.9
Q9H425	C1orf198	Uncharact	6	2	2	2	327	36.3
D6RBR7	ZNF330	Zinc fing	12	3	4	3	229	25.8
Q14149	MORC3	MORC fami	2	2	2	2	939	107
O75817	POP7	Ribonucle	18	2	2	2	140	15.6
Q9P291	ARMCX1	Armadillc	5	2	2	2	453	49.2
Q9NPL8	TIMMDC1	Complex I	9	3	3	3	285	32.2
Q9UFG5	C19orf25	UPF0449 p	22	2	2	2	118	12.9
Q9BQB6	VKORC1	Vitamin K	13	3	4	3	163	18.2
Q06481	APLP2	Amyloid-l	1	1	2	1	763	86.9
A0A087WVC		Uncharact	19	2	2	2	110	12.7

Q5EBL8	PDZD11	PDZ domai	17	1	1	1	140	16.1
E7EQ64	PRSS1	Trypsin-1	11	2	7	2	261	28.1
Q6DN90	IQSEC1	IQ motif	2	2	2	2	963	108.2
P05109	S100A8	Protein S	19	2	2	2	93	10.8
O96013	PAK4	Serine/th	3	2	2	2	591	64
Q16637	SMN1	Survival	7	2	2	2	294	31.8
P28676	GCA	Grancalci	14	3	3	3	217	24
E9PE15	CLCN3	Chloride	3	2	2	2	725	80.5
Q9NRZ7	AGPAT3	1-acyl-sn	5	2	3	2	376	43.4
C9J6A7	RPE	Ribulose-	11	2	2	2	159	17.4
F8WCZ3	CCDC115	Coiled-cc	11	2	2	2	193	21.1
Q9UBV8	PEF1	Peflin OS	8	2	2	2	284	30.4
O94888	UBXN7	UBX domai	5	3	4	3	489	54.8
A0A2R8YDF	TSC2	Tuberin (	2	2	2	2	1781	197.7
O95396	MOCS3	Adenylylt	6	2	2	2	460	49.6
E9PPY3	RRP8	Ribosomal	7	2	2	2	306	34.2
Q8NBP0	TTC13	Tetratric	4	2	2	2	860	96.8
Q7Z6B7	SRGAP1	SLIT-ROBO	1	2	2	2	1085	124.2
Q9UK39	NOCT	Nocturnin	4	1	1	1	431	48.2
Q8N122	RPTOR	Regulator	2	2	2	2	1335	148.9
Q9NX08	COMMD8	COMM doma	11	2	2	2	183	21.1
Q8TCE6	FAM45A	Protein F	7	2	2	2	357	40.5
Q5SY16	NOL9	Polynucle	3	2	3	2	702	79.3
Q9NRN7	AASDHPPT	L-aminoad	6	2	2	2	309	35.8
O75909	CCNK	Cyclin-K	4	2	2	2	580	64.2
Q8N5G0	SMIM20	Small int	33	2	2	2	67	7.7
A0A096LP6	CD99	CD99 anti	9	2	2	2	184	18.8
Q9H7M9	VSIR	V-type im	5	2	2	2	311	33.9
Q6UWP7	LCLAT1	Lysocardi	7	3	3	3	414	48.9
Q9P2C4	TMEM181	Transmemb	3	1	1	1	612	69.3
Q86UE8	TLK2	Serine/th	2	2	2	1	772	87.6
B9ZVN9	POLR1A	DNA-direc	2	2	2	2	1659	187.7
P04217	A1BG	Alpha-1B-	4	1	1	1	495	54.2
P19256	CD58	Lymphocyt	10	2	2	2	250	28.1
Q6UVY6	MOXD1	DBH-like	4	2	2	2	613	69.6
R4GN18	CD46	Membrane	27	2	2	2	78	8.5
Q9ULP9	TBC1D24	TBC1 doma	4	2	3	2	559	62.9
Q9C0I1	MTMR12	Myotubula	3	2	2	2	747	86.1
H0Y555	PSMF1	Proteasom	20	2	3	2	107	11.6
Q8TCC3	MRPL30	39S ribos	15	3	3	3	161	18.5
Q9BPX5	ARPC5L	Actin-rel	14	3	4	3	153	16.9
Q86VX2	COMMD7	COMM doma	10	2	2	2	200	22.5
A0A0B4J25	LYZ	Lysozyme	15	2	2	2	137	15.3
O43318	MAP3K7	Mitogen-a	4	2	2	2	606	67.2
Q8NC60	NOA1	Nitric ox	4	2	3	2	698	78.4
Q9Y613	FHOD1	FH1/FH2 d	2	3	3	3	1164	126.5
O00217	NDUFS8	NADH dehy	9	2	2	2	210	23.7
Q5JTJ3	COA6	Cytochrom	21	2	2	2	125	14.1
P02792	FTL	Ferritin	18	3	3	3	175	20
Q5JSL0	HMGN5	High mobi	16	2	2	2	148	16.5
Q6ZUT6	CCDC9B	Coiled-cc	3	2	2	2	534	57.3

Q9BTY2	FUCA2	Plasma al	4	2	2	2	467	54
A0A2U3TZV	PLCH1	1-phospha	0	1	10	1	1685	188.6
G3V150	B3GAT3	Galactosy	5	2	2	2	319	34.9
A8MQ02	AFDN	Afadin OS	1	2	2	2	1781	201.7
Q5TAW7	CAB39L	Calcium-b	9	3	3	1	280	32.4
Q7Z2X4	PID1	PTB-conta	8	1	1	1	250	28.3
Q15172	PPP2R5A	Serine/th	6	2	2	2	486	56.2
Q96BZ9	TBC1D20	TBC1 doma	6	2	2	2	403	45.8
A0A0S2Z4R	TAP1	Antigen p	10	2	2	2	323	34.6
Q9NY61	AATF	Protein A	4	2	2	2	560	63.1
Q5VWJ9	SNX30	Sorting n	4	2	2	2	437	49.6
Q96L58	B3GALT6	Beta-1,3-	4	1	1	1	329	37.1
P62341	SELENOT	Thioredox	10	2	2	2	195	22.3
Q8NI22	MCFD2	Multiple	12	1	2	1	146	16.4
A6XND1	IGFBP3	Insulin-l	7	2	2	2	263	29
F6SYF8	DKK3	Dickkopf-	10	2	2	2	364	39.9
Q9GZT8	NIF3L1	NIF3-like	9	3	3	3	377	41.9
Q9NY93	DDX56	Probable	3	2	2	2	547	61.6
P78524	ST5	Suppressi	2	2	2	2	1137	126.4
Q9NXE4	SMPD4	Sphingomy	2	2	2	2	866	97.7
Q9UQ03	CORO2B	Coronin-2	5	2	2	2	480	54.9
Q00403	GTF2B	Transcrip	8	3	3	3	316	34.8
P80217	IFI35	Interferc	7	2	2	2	286	31.5
O14646	CHD1	Chromodom	1	3	3	3	1710	196.6
Q9UL54	TAOK2	Serine/th	1	2	3	1	1235	138.2
Q13424	SNTA1	Alpha-1-s	4	2	2	1	505	53.9
O75884	RBBP9	Putative	16	2	2	2	186	21
F8WF16	PPHLN1	Periphili	6	2	2	2	303	34.8
Q96IP4	TENT5A	Terminal	5	2	2	2	442	49.6
Q5T1B5	INPP5A	Type I in	4	2	3	2	387	44.9
Q9UBI1	COMMD3	COMM doma	12	2	3	2	195	22.1
P34059	GALNS	N-acetylg	3	2	3	1	522	58
Q96CN4	EVI5L	EVI5-like	3	2	2	1	794	91.3
Q9H974	QTRT2	Queuine t	5	1	1	1	415	46.7
Q96H79	ZC3HAV1L	Zinc fing	7	2	2	2	300	32.9
O75815	BCAR3	Breast ca	3	3	4	3	825	92.5
O00479	HMGN4	High mobi	19	2	2	2	90	9.5
O95628	CNOT4	CCR4-NOT	3	2	2	2	575	63.5
Q6ICB0	DESI1	Desumoyla	10	2	2	2	168	18.3
Q96DX4	RSPRY1	RING fing	5	2	2	2	576	64.1
P57723	PCBP4	Poly(rC)-	5	2	4	1	403	41.5
Q86U38	NOP9	Nucleolar	3	2	2	2	636	69.4
J3KRR1	C17orf75	Protein N	10	2	2	2	275	30.6
Q9UET6	FTSJ1	Putative	8	2	2	2	329	36.1
Q96AJ9	VTI1A	Vesicle t	7	2	2	2	217	25.2
O15397	IPO8	Importin-	2	2	2	1	1037	119.9
A0A088AWN	DOCK9	Dedicator	1	2	2	1	2081	237.8
Q14118	DAG1	Dystrogly	3	1	1	1	895	97.4
Q96EY1	DNAJA3	DnaJ homc	5	2	2	2	480	52.5
Q6P6C2	ALKBH5	RNA demet	7	2	2	2	394	44.2
Q9UBV7	B4GALT7	Beta-1,4-	8	3	3	3	327	37.4

E9PIQ7	HAX1	HCLS1-ass	13	2	2	2	151	17.2
P40937	RFC5	Replicati	5	2	2	2	340	38.5
Q9P0P0	RNF181	E3 ubiqui	10	2	2	2	153	17.9
Q16656	NRF1	Nuclear r	4	2	2	2	503	53.5
Q8IXM6	NRM	Nurim OS=	7	2	2	2	262	29.4
Q9BX40	LSM14B	Protein L	8	2	2	2	385	42
J3QRZ6	MIF4GD	MIF4G dom	15	2	2	2	204	23.3
Q96QU8	XPO6	Exportin-	2	2	2	2	1125	128.8
Q9NPJ6	MED4	Mediator	8	2	2	2	270	29.7
O95786	DDX58	Probable	2	2	2	2	925	106.5
Q9NYV4	CDK12	Cyclin-de	2	3	3	2	1490	164.1
Q9P013	CWC15	Spliceosc	8	2	2	2	229	26.6
Q9UKU7	ACAD8	Isobutyry	5	2	2	2	415	45
Q9Y3B2	EXOSC1	Exosome c	12	2	2	2	195	21.4
Q15154	PCM1	Pericentr	1	3	3	2	2024	228.4
H0YJM2	UBR7	Putative	8	2	2	2	174	20.5
O14879	IFIT3	Interferc	5	2	2	2	490	56
H3BQV3	COG8	Conservec	3	2	2	2	534	60.4
Q32P41	TRMT5	tRNA (gua	4	2	2	2	509	58.2
Q7Z674	FEZ2	Fascicula	11	2	2	2	182	20.9
O75146	HIP1R	Huntingti	2	3	3	2	1068	119.3
Q9UJY1	HSPB8	Heat shoc	12	2	2	2	196	21.6
P56589	PEX3	Peroxisom	5	2	2	2	373	42.1
O96033	MOCS2	Molybdopt	25	2	2	2	88	9.7
Q8WVC0	LEO1	RNA polym	2	1	1	1	666	75.4
Q8TDQ4	TMEM222	Transmemb	11	1	1	1	175	19.8
E9PG73	PPIG	Peptidyl-	2	2	2	2	739	87
Q9P2P6	STARD9	StAR-rela	0	2	2	2	4700	516
P42356	PI4KA	Phosphati	1	2	2	2	2102	236.7
Q9HAN9	NMNAT1	Nicotinam	7	2	2	2	279	31.9
Q16134	ETFDH	Electron	5	2	2	2	617	68.5
P21953	BCKDHB	2-oxoisov	6	2	2	2	392	43.1
Q70JA7	CHSY3	Chondroit	3	3	3	3	882	100.2
Q92995	USP13	Ubiquitin	2	2	2	2	863	97.3
Q92597	NDRG1	Protein N	8	2	2	2	394	42.8
Q9NWX6	THG1L	Probable	6	2	2	2	298	34.8
E7EM50	PIGG	GPI ethan	5	2	2	2	463	51.2
Q8TCD1	C18orf32	UPF0729 p	13	1	2	1	76	8.7
Q9Y294	ASF1A	Histone c	13	2	2	2	204	23
Q99496	RNF2	E3 ubiqui	6	2	2	1	336	37.6
L0R819	ASDURF	ASNSD1 up	19	2	2	2	96	11.2
Q9UMZ2	SYNRG	Synergin	1	2	2	2	1314	140.6
H7C224	IRAK1	Interleuk	5	2	2	2	392	42.2
Q6P1L8	MRPL14	39S ribos	11	2	2	2	145	15.9
Q8WY22	BRI3BP	BRI3-bind	9	2	2	1	251	27.8
Q08426	EHHADH	Peroxisom	4	2	2	2	723	79.4
O43184	ADAM12	Disintegr	3	3	4	3	909	99.5
F5GYT8	MCCC1	Methylcrc	4	2	2	2	575	64
Q8N511	TMEM199	Transmemb	10	2	2	2	208	23.1
Q9NRG9	AAAS	Aladin OS	3	2	2	2	546	59.5
K7EQH1	C18orf25	Uncharact	15	1	1	1	146	15.5

Q86X76	NIT1	Deaminate	6	2	2	2	327	35.9
Q9UQ13	SHOC2	Leucine-r	3	2	2	2	582	64.8
Q8N3P4	VPS8	Vacuolar	2	2	2	2	1428	161.7
P45877	PPIC	Peptidyl-	9	3	7	2	212	22.7
Q9P2Q2	FRMD4A	FERM doma	1	2	2	2	1039	115.4
Q8NE86	MCU	Calcium u	5	2	2	2	351	39.8
Q9BVM4	GGACT	Gamma-glu	14	2	2	2	153	17.3
Q8IWR0	ZC3H7A	Zinc fing	2	2	2	2	971	110.5
Q8N5I4	DHRSX	Dehydroge	5	1	1	1	330	36.4
Q15427	SF3B4	Splicing	3	1	1	1	424	44.4
Q4LDG9	DNAL1	Dynein li	11	2	2	2	190	21.5
Q02040	AKAP17A	A-kinase	4	3	3	3	695	80.7
Q6XQN6	NAPRT	Nicotinat	4	2	2	2	538	57.5
E5RH51	SMIM12	Small int	26	2	2	2	74	8.7
P51397	DAP	Death-ass	16	2	3	2	102	11.2
Q9UHR4	BAIAP2L1	Brain-spe	4	2	2	2	511	56.8
P52657	GTF2A2	Transcrip	15	2	2	2	109	12.4
O15226	NKRF	NF-kappa-	3	2	2	2	690	77.6
Q92733	PRCC	Proline-r	4	2	2	2	491	52.4
G3V4K3	VIPAS39	Spermatog	3	2	2	2	519	59.7
A5PLN9	TRAPPC13	Trafficki	4	2	2	2	417	46.5
Q9NP92	MRPS30	39S ribos	10	3	4	3	439	50.3
H7BY55	CD55	Complemen	3	2	2	2	550	58.9
B1APR7	EYA3	Eyes abse	3	1	1	1	416	45.5
Q8WXH0	SYNE2	Nesprin-2	0	3	3	1	6885	795.9
H3BPK1	KATNB1	Katanin p	8	2	2	2	210	22.9
P01024	C3	Complemen	1	1	1	1	1663	187
A0A0A0MSY	TK2	Thymidine	6	2	2	2	289	33.3
A0A087WU8	TNFAIP3	Tumor nec	3	3	3	3	749	84.9
A0A087WZK	DHPS	Deoxyhypu	6	2	2	2	370	41.1
Q9Y2Q5	LAMTOR2	Ragulator	8	1	2	1	125	13.5
P12074	COX6A1	Cytochrom	27	1	2	1	109	12.1
O14880	MGST3	Microsoma	5	1	2	1	152	16.5
Q9NVM6	DNAJC17	DnaJ homo	5	2	2	2	304	34.7
O75084	FZD7	Frizzled-	3	2	3	2	574	63.6
P25490	YY1	Transcrip	4	2	2	2	414	44.7
Q969T9	WBP2	WW domain	7	2	2	2	261	28.1
P15291	B4GALT1	Beta-1,4-	4	2	2	2	398	43.9
Q14289	PTK2B	Protein-t	2	2	2	2	1009	115.8
A0A0U1RQI	SORBS1	Sorbin an	3	1	1	1	507	58
A0A2R8Y5F	KSR1	Kinase su	2	2	2	2	928	103
Q14692	BMS1	Ribosome	1	2	2	2	1282	145.7
Q9UKN8	GTF3C4	General t	2	2	2	2	822	91.9
O43292	GPAA1	Glycosylp	3	2	2	2	621	67.6
H0Y3M3	EML3	Echinoder	3	2	2	2	911	97.5
Q96BW9	TAMM41	Phosphati	4	1	1	1	452	51
P35251	RFC1	Replicati	1	1	1	1	1148	128.2
E9PI99	NFYC	Nuclear t	18	3	3	3	141	16.2
Q99808	SLC29A1	Equilibra	4	2	2	2	456	50.2
Q06587	RING1	E3 ubiqui	5	2	2	1	406	42.4
Q96I51	RCC1L	RCC1-like	5	1	1	1	464	50

Q8NHG7	SVIP	Small VCF	14	1	1	1	77	8.4
Q5R3B4	MPC2	Mitochonc	11	1	1	1	105	11.7
Q96JP5	ZFP91	E3 ubiqui	4	1	1	1	570	63.4
Q68DK2	ZFYVE26	Zinc fing	1	2	3	1	2539	284.4
Q5JTZ9	AARS2	Alanine--	2	2	2	2	985	107.3
O14682	ENC1	Ectoderm-	3	2	2	2	589	66.1
H9KVA9	BRCC3	Lys-63-sp	3	1	1	1	317	36.2
Q86UV5	USP48	Ubiquitin	2	3	3	3	1035	119
Q96P47	AGAP3	Arf-GAP w	4	2	2	2	875	95
O43301	HSPA12A	Heat shoc	3	2	2	2	675	74.9
H0YAG5	MRPS18C	28S ribos	16	1	1	1	113	12.5
A0A096LNX	SCARF2	Scavenger	3	2	2	2	871	92.3
P62328	TMSB4X	Thymosin	32	2	4	2	44	5.1
A2IDA3	MPG	DNA-3-met	5	1	1	1	251	27.3
Q99797	MIPEP	Mitochonc	3	2	2	2	713	80.6
Q9ULF5	SLC39A10	Zinc tran	2	2	2	2	831	94.1
Q9Y6K8	AK5	Adenylate	3	2	5	1	562	63.3
G3V1J9	TMED3	Transmemb	8	1	1	1	146	16.7
Q9BXP2	SLC12A9	Solute ca	2	1	1	1	914	96
O60508	CDC40	Pre-mRNA-	3	2	2	2	579	65.5
Q9BQ51	PDCD1LG2	Programme	4	1	1	1	273	30.9
H7BXY6	TSPAN14	Tetraspan	9	2	2	2	213	23.9
Q9NWZ8	GEMIN8	Gem-assoc	8	2	2	2	242	28.6
Q5T5Y3	CAMSAP1	Calmoduli	1	3	3	2	1602	177.9
Q96SK3	ZNF607	Zinc fing	4	2	2	2	696	80.5
Q9BQQ3	GORASP1	Golgi rea	4	2	2	1	440	46.5
Q8IVH4	MMAA	Methylmal	4	2	2	2	418	46.5
Q8WWH5	TRUB1	Probable	5	2	2	2	349	37.2
Q7Z6U0	TBPL1	TATA box-	14	1	1	1	91	10.2
O95639	CPSF4	Cleavage	4	1	1	1	269	30.2
Q9C0H2	TTYH3	Protein t	3	2	2	2	523	57.5
Q96EC8	YIPF6	Protein Y	5	1	1	1	236	26.2
E5RGA1	PLAT	Tissue-ty	7	1	1	1	178	20.1
Q96AA3	RFT1	Protein R	4	2	2	2	541	60.3
P55899	FCGRT	IgG recep	4	2	2	2	365	39.7
Q9NTJ4	MAN2C1	Alpha-man	2	2	2	2	1040	115.8
O94923	GLCE	D-glucurc	4	3	3	3	617	70.1
G3V5T0	GSTZ1	Maleylace	8	2	2	2	202	22.6
J3QQK6	MBP	Myelin ba	9	2	2	2	253	27.7
Q9Y221	NIP7	60S ribos	9	2	2	2	180	20.4
H0YDK7	RAB30	Ras-relat	11	2	2	1	167	19.2
C9JWV9	TMBIM1	Protein l	8	1	1	1	171	19
A0A0C4DG9	KLHL13	Kelch-lik	3	2	2	2	658	74.6
Q99717	SMAD5	Mothers a	4	2	2	2	465	52.2
K7EM09	TMEM205	Transmemb	11	1	1	1	120	13.4
B3KRD8	SEC14L2	SEC14-lik	5	2	2	2	329	37.4
Q9ULI3	HEG1	Protein H	2	2	2	2	1381	147.4
P52179	MYOM1	Myomesin-	1	1	3	1	1685	187.5
P46100	ATRX	Transcrip	1	2	3	1	2492	282.4
Q16566	CAMK4	Calcium/c	4	2	2	1	473	51.9
H7C5U3	MFSD1	Major fac	19	1	1	1	64	6.8

Q15118	PDK1	[Pyruvate	5	2	2	2	436	49.2
Q9Y312	AAR2	Protein A	5	1	2	1	384	43.4
A0A0C4DFF	NGLY1	Peptide-N	4	2	2	2	633	72
Q6PI78	TMEM65	Transmemb	8	2	2	2	240	25.5
F5H303	NOC4L	Nucleolar	8	2	2	2	270	31.1
Q7L4I2	RSRC2	Arginine/	5	1	1	1	434	50.5
H0YFW5	MSRB3	Methionin	9	2	2	2	174	19.5
R4GMU8	LAMTOR5	Regulator	25	1	1	1	79	8.2
Q9BVL4	SELENOO	Selenopro	2	1	1	1	669	73.4
Q9Y657	SPIN1	Spindlin-	6	1	1	1	262	29.6
G3V2M2	ZFYVE21	Zinc fing	22	1	2	1	93	10.5
Q5T4B2	CERCAM	Inactive	4	2	2	2	595	67.5
Q9UFW8	CGGBP1	CGG tripl	5	1	1	1	167	18.8
H3BRM1	ZFYVE19	Abscissic	5	2	2	1	307	33.2
A0A087WYY	ATP13A3	Cation-tr	2	1	1	1	701	77.3
Q8IYS2	KIAA2013	Uncharact	2	2	2	2	634	69.1
F2Z328	DYNLT3	Dynein li	8	1	1	1	122	13.4
Q5T0V2	SLIT1	Slit homo	2	1	3	1	409	45
Q8WVT3	TRAPPC12	Trafficki	2	1	1	1	735	79.3
Q9H977	WDR54	WD repeat	4	1	1	1	334	35.9
Q8IV48	ERI1	3'-5' exo	5	2	2	2	349	40
Q01658	DR1	Protein D	9	2	2	2	176	19.4
Q9NXH8	TOR4A	Torsin-4A	5	2	2	2	423	46.9
Q86YZ3	HRNR	Hornerin	2	2	2	2	2850	282.2
B7Z7D2	ACP2	cDNA FLJ5	5	2	2	2	391	44.5
F5GZ28	LIG1	DNA ligas	2	2	3	1	851	93.9
C9JBX7	WDR45	WD repeat	5	1	1	1	213	23.5
A0A2R8YFQ	CRYL1	Lambda-cr	8	2	2	2	229	25.3
Q9Y6M5	SLC30A1	Zinc tran	4	2	3	2	507	55.3
P12277	CKB	Creatine	4	2	2	2	381	42.6
O75940	SMNDC1	Survival	4	1	1	1	238	26.7
A0A087X09	WDR13	WD repeat	10	2	2	2	187	20.6
Q96A00	PPP1R14A	Protein p	12	2	2	2	147	16.7
H7C024	GPC1	Glypican-	5	1	1	1	294	32.9
C9J236	NDUFAF7	Protein a	9	1	1	1	230	25.4
Q9BUH6	PAXX	Protein P	7	1	1	1	204	21.6
Q96JH7	VCPIP1	Deubiquit	1	1	1	1	1222	134.2
C9JKF1	SAMD9	Sterile a	2	2	6	1	1283	148.7
D6RCB6	RIOX2	Ribosomal	13	1	1	1	112	12.4
A0A2R8Y4J	RMND1	Required	3	1	1	1	444	50.5
E9PIN5	TP53I11	Tumor pro	8	1	1	1	123	13.3
Q5JPH6	EARS2	Probable	3	2	2	2	523	58.7
O14777	NDC80	Kinetocho	2	2	2	2	642	73.9
H0YCT3	POGZ	Pogo tran	6	1	1	1	316	35.4
Q7Z2K6	ERMP1	Endoplasm	1	1	1	1	904	100.2
E9PI02	HSF1	Heat shoc	7	1	1	1	138	16.1
E7EUH7	PUS7	Pseudouri	4	2	2	2	448	50.5
Q8N4H5	TOMM5	Mitochond	43	3	4	3	51	6
Q9HBM6	TAF9B	Transcrip	4	1	1	1	251	27.6
K7ELY2	STX10	Syntaxin-	5	1	1	1	201	22.5
Q9NXU5	ARL15	ADP-ribos	9	2	2	2	204	22.9

Q5JXX2	MORF4L2	Mortality	13	2	2	2	177	19.8
P22413	ENPP1	Ectonucle	3	1	1	1	925	104.9
Q9H9A5	CNOT10	CCR4-NOT	2	2	2	2	744	82.3
P61024	CKS1B	Cyclin-de	20	1	1	1	79	9.7
P98170	XIAP	E3 ubiqui	3	1	1	1	497	56.6
A0A087WYU	METTL26	Methyltra	5	1	1	1	184	20.4
Q8IWB9	TEX2	Testis-ex	2	2	2	2	1127	125.2
Q9Y2U5	MAP3K2	Mitogen-a	2	1	1	1	619	69.7
F8VQD9	ATG101	Autophagy	13	2	2	2	190	21.9
J3KNN7	BRAP	BRCA1-ass	2	1	1	1	562	64.2
O95801	TTC4	Tetratric	5	2	2	2	387	44.7
P36639	NUDT1	7,8-dihyd	7	1	1	1	197	22.5
Q969M3	YIPF5	Protein Y	5	1	1	1	257	28
Q9P003	CNIH4	Protein c	14	1	1	1	139	16.1
J3QQW2	SS18	Protein S	13	1	1	1	94	10.6
O43741	PRKAB2	5'-AMP-ac	7	1	1	1	272	30.3
Q9H788	SH2D4A	SH2 domai	4	2	2	2	454	52.7
Q99595	TIMM17A	Mitochond	13	1	1	1	171	18
Q09328	MGAT5	Alpha-1,6	1	1	1	1	741	84.5
O95633	FSTL3	Follistat	7	1	1	1	263	27.6
I3L0T6	MRM3	rRNA meth	11	1	1	1	110	12.6
A0A0U1RRL	SPATA20	Spermatog	1	1	1	1	786	88
Q969Z0	TBRG4	FAST kina	2	2	3	2	631	70.7
Q8NHZ8	CDC26	Anaphase-	13	1	2	1	85	9.8
H0YI10	CERS5	Ceramide	5	2	2	2	259	30.5
P63272	SUPT4H1	Transcrip	8	1	1	1	117	13.2
Q9UF94	KLHDC4	Kelch dom	9	2	2	2	258	29.2
Q9H501	ESF1	ESF1 homo	1	1	1	1	851	98.7
E7EPJ2	ITSN2	Intersect	3	1	1	1	609	68.9
Q15742	NAB2	NGFI-A-bi	2	1	1	1	525	56.6
O14531	DPYSL4	Dihydropy	3	1	3	1	572	61.8
Q9Y679	AUP1	Ancient u	4	1	1	1	476	53
H7BXQ8	ARMC10	Armadillo	6	1	1	1	193	20.9
A1L188	NDUFAF8	NADH dehy	18	1	1	1	74	7.8
Q9BU61	NDUFAF3	NADH dehy	6	1	1	1	184	20.3
P61812	TGFB2	Transform	6	2	2	2	414	47.7
Q9BXJ0	C1QTNF5	Complemen	6	1	1	1	243	25.3
Q9ULE0	WWC3	Protein W	2	2	2	1	1092	122.6
Q08554	DSC1	Desmocoll	2	1	1	1	894	99.9
Q16832	DDR2	Discoidin	2	2	2	2	855	96.7
E9PEC4	CCM2	Cerebral	6	2	2	2	271	29.8
P22670	RFX1	MHC class	1	1	2	1	979	104.7
A8MXF6	RABL2B	Rab-like	7	1	1	1	165	18.5
Q8TF68	ZNF384	Zinc fing	2	1	1	1	577	63.2
P09661	SNRPA1	U2 small	8	2	2	2	255	28.4
H0YCP5	RNASEH2C	Ribonucle	12	1	1	1	129	13.9
H3BU43	SIRPB1	Signal-re	19	1	1	1	77	8.2
O95104	SCAF4	Splicing	1	2	2	2	1147	125.8
Q9BVS4	RIOK2	Serine/th	3	2	2	2	552	63.2
Q9NP84	TNFRSF12A	Tumor nec	8	1	1	1	129	13.9
Q9Y5T5	USP16	Ubiquitin	3	2	2	2	823	93.5

H0YME2	ZFAND6	AN1-type	16	1	1	1	159	17
Q9BRQ0	PYGO2	Pygopus h	4	1	1	1	406	41.2
Q8NG68	TTL	Tubulin--	3	1	1	1	377	43.2
P56378	MP68	6.8 kDa m	16	1	4	1	58	6.7
Q96EV2	RBM33	RNA-bindi	2	1	1	1	1170	129.9
C9J3D7	CROT	Peroxisom	3	1	1	1	574	65.8
O94992	HEXIM1	Protein H	4	2	2	2	359	40.6
Q8N2E6	TOR2A	Prosalusi	4	1	1	1	242	26.2
O15379	HDAC3	Histone d	6	2	2	2	428	48.8
Q9UKL0	RCOR1	REST core	2	1	1	1	485	53.3
F5H2Z7	CCDC92	Coiled-cc	11	1	1	1	96	11
A6NMN0	PHKA1	Phosphory	1	1	1	1	1240	139
Q9BRX5	GINS3	DNA repli	6	1	1	1	216	24.5
Q9P266	JCAD	Junctiona	1	2	2	2	1359	148.3
Q96HR3	MED30	Mediator	7	1	1	1	178	20.3
H7C3I5	CRYZL1	Quinone c	5	1	1	1	241	26.3
Q8N4V1	MMGT1	Membrane	7	1	1	1	131	14.7
P18440	NAT1	Arylamine	3	1	1	1	290	33.9
Q7L2J0	MEPCE	7SK snRNA	2	1	1	1	689	74.3
O43150	ASAP2	Arf-GAP w	2	2	2	2	1006	111.6
H0Y7V5	CCDC186	Coiled-cc	3	1	1	1	436	50.9
P27658	COL8A1	Collagen	1	1	1	1	744	73.3
O60870	KIN	DNA/RNA-b	3	1	1	1	393	45.3
A0A087WVZ	POLR2E	DNA-direc	9	2	2	2	184	21.4
P78345	RPP38	Ribonucle	4	1	1	1	283	31.8
O95772	STARD3NL	STARD3 N-	4	1	1	1	234	26.6
B4DUA7	MED29	Intersex-	5	1	1	1	221	23.5
C9J2Q2	ATPAF2	ATP synth	6	1	1	1	205	23.1
Q9ULK4	MED23	Mediator	1	1	1	1	1368	156.4
X6RLT1	NELFCD	Negative	3	2	2	2	593	66.5
F8WBT0	ARAP1	Arf-GAP w	5	1	1	1	307	34.9
Q96NT1	NAP1L5	Nucleosom	5	1	1	1	182	19.6
H0YJ92	MNAT1	CDK-activ	5	1	1	1	169	19.4
A0A0A0MTQ	KIAA0391	Mitochond	5	1	1	1	185	21.7
J3KRP9	TANC2	Protein T	1	1	1	1	1254	138.9
Q9NSY2	STARD5	StAR-rela	4	1	1	1	213	23.8
E7EV41	SLC8A1	Sodium/ca	2	2	2	2	664	74.4
Q6GMV2	SMYD5	SET and M	3	1	1	1	418	47.3
Q96FX2	DPH3	DPH3 homo	26	1	1	1	82	9.2
Q9NQW6	ANLN	Anillin C	1	1	1	1	1124	124.1
Q5F1R6	DNAJC21	DnaJ homo	4	2	2	2	531	62
Q6UWJ1	TMCO3	Transmemb	1	1	1	1	677	75.5
Q96NC0	ZMAT2	Zinc fing	8	2	2	2	199	23.6
O95999	BCL10	B-cell ly	4	1	1	1	233	26.2
Q96AY4	TTC28	Tetratric	0	1	1	1	2481	270.7
P60520	GABARAPL2	Gamma-ami	13	2	2	1	117	13.7
A0A087WZG	ARHGEF18	Rho guani	1	2	2	1	1122	126.1
A0A087WTT	TMEM230	Transmemb	9	1	1	1	127	13.6
X6R717	DHX32	Putative	4	1	1	1	367	41.5
Q96IW7	SEC22A	Vesicle-t	4	1	1	1	307	34.9
F8VV52	CNOT2	CCR4-NOT	4	2	2	2	531	58.6

H7BYP4	SP140L	Nuclear b	3	2	2	1	520	60.5
A0A087WYW	HIKESHI	Protein H	7	1	1	1	131	14.4
Q8N5W9	RFLNB	Refilin-E	6	1	1	1	214	22.9
Q53TN4	CYBRD1	Cytochrom	3	1	1	1	286	31.6
Q96CW5	TUBGCP3	Gamma-tub	2	2	2	2	907	103.5
Q9NXD2	MTMR10	Myotubula	2	1	1	1	777	88.2
Q5TIH2	SFT2D2	Vesicle t	9	1	1	1	108	11.7
O14763	TNFRSF10E	Tumor nec	2	1	1	1	440	47.8
Q9UKZ1	CNOT11	CCR4-NOT	2	1	1	1	510	55.2
Q96G74	OTUD5	OTU domai	3	1	1	1	571	60.6
Q9UJJ9	GNPTG	N-acetylg	3	1	1	1	305	34
P49903	SEPHS1	Selenide,	3	1	2	1	392	42.9
Q9NWT1	PAK1IP1	p21-activ	4	2	2	2	392	43.9
F5GWI4	ADA	Adenosine	3	1	1	1	339	38.3
P42336	PIK3CA	Phosphati	1	2	2	2	1068	124.2
Q53SF7	COBLL1	Cordon-bl	1	1	1	1	1204	131.7
F8WDM6	MED15	Mediator	19	1	1	1	54	6.1
P08047	SP1	Transcrip	2	2	2	2	785	80.6
Q9BXJ8	TMEM120A	Transmemb	3	1	1	1	343	40.6
P62875	POLR2L	DNA-direc	13	1	1	1	67	7.6
Q5HYK7	SH3D19	SH3 domai	1	1	1	1	790	86.5
P17706	PTPN2	Tyrosine-	4	2	2	2	415	48.4
P24385	CCND1	G1/S-spec	6	2	2	2	295	33.7
A0A0A0MTE	BTAF1	TATA-binc	1	1	1	1	1849	206.8
Q96B45	BORCS7	BLOC-1-re	16	1	1	1	105	11.6
A0A087WXD	SLC9A7	Sodium/hy	1	1	1	1	726	80.2
U3KQ69	MTG1	Mitochonc	3	1	1	1	293	32.6
P18887	XRCC1	DNA repai	3	2	2	2	633	69.4
Q5C9Z4	NOM1	Nucleolar	1	1	1	1	860	96.2
Q96BN8	OTULIN	Ubiquitir	5	2	2	2	352	40.2
K7ENP1	TRMT11	tRNA (gua	4	1	1	1	230	26.4
Q9BYC9	MRPL20	39S ribos	13	2	2	2	149	17.4
Q8WXA9	SREK1	Splicing	2	1	1	1	508	59.3
Q9H0N5	PCBD2	Pterin-4-	10	2	3	1	130	14.4
Q9BVJ7	DUSP23	Dual spec	7	1	1	1	150	16.6
O94817	ATG12	Ubiquitir	6	1	1	1	140	15.1
Q9Y4R8	TELO2	Telomere	2	2	2	2	837	91.7
Q8N108	MIER1	Mesoderm	4	1	1	1	512	57.9
P98155	VLDLR	Very low-	1	1	1	1	873	96
K7EQ17	NOL4	Nucleolar	2	1	1	1	558	61.3
A0A1W2PNW	KCNMA1	Calcium-a	1	1	1	1	1230	137
Q7Z4H3	HDDC2	HD domain	5	1	1	1	204	23.4
Q14331	FRG1	Protein F	5	1	1	1	258	29.2
O95905	ECD	Protein e	2	1	1	1	644	72.7
Q8NBN3	TMEM87A	Transmemb	3	2	2	2	555	63.4
P82663	MRPS25	28S ribos	10	1	1	1	173	20.1
Q8IW45	NAXD	ATP-deper	4	1	1	1	347	36.6
P04066	FUCA1	Tissue al	2	1	2	1	466	53.7
A0A087WVZ	ZMYND8	Protein k	1	2	2	2	1136	125.7
Q96GM8	TOE1	Target of	3	1	1	1	510	56.5
Q8TD43	TRPM4	Transient	1	1	1	1	1214	134.2

Q49B96	COX19	Cytochrom	11	1	1	1	90	10.4
A0A1W2PQ7	GOSR2	Golgi SNA	15	2	2	2	164	19.4
Q8IUR7	ARMC8	Armadillo	1	1	1	1	673	75.5
E5RFJ9	SARAF	Store-ope	12	1	1	1	147	16.1
Q8IX04	UEVLD	Ubiquitin	2	1	1	1	471	52.2
J3KPT4	TRABD	TraB doma	2	1	1	1	379	42.7
C9JE98	NCOR2	Nuclear r	1	1	1	1	2458	267.9
K7EPS8	C19orf12	Protein C	9	1	1	1	106	11
Q9Y6D0	SELENOK	Selenopro	18	2	2	2	94	10.6
Q96BI3	APH1A	Gamma-sec	5	1	1	1	265	29
Q04941	PLP2	Proteolip	9	1	2	1	152	16.7
I3L213	PHKB	Phosphory	8	1	1	1	210	23.5
Q8N9T8	KRI1	Protein K	3	2	2	2	703	82.5
P49069	CAMLG	Calcium s	3	1	1	1	296	32.9
Q96MG7	NSMCE3	Non-struc	6	2	2	2	304	34.3
C9JVK8	AZI2	5-azacyti	5	1	1	1	215	25
Q9Y625	GPC6	Glypican-	3	1	1	1	555	62.7
C9JBY7	MRPS33	28S ribos	9	1	1	1	96	11.4
C9JQZ0	SERF2	Small EDR	13	1	1	1	71	7.7
E5RK75	ZFAND1	AN1-type	10	1	1	1	97	10.9
O95297	MPZL1	Myelin pr	4	1	1	1	269	29.1
Q9P1F3	ABRACL	Costars f	16	1	1	1	81	9.1
Q8IY37	DHX37	Probable	1	1	1	1	1157	129.5
Q9NRG4	SMYD2	N-lysine	5	2	2	2	433	49.7
C9JWF7	SRPK2	SRSF prot	6	2	2	2	231	25.8
Q9H5V9	CXorf56	UPF0428 p	6	1	1	1	222	25.6
P48651	PTDSS1	Phosphati	4	2	2	2	473	55.5
P58004	SESN2	Sestrin-2	3	1	1	1	480	54.5
Q8IY33	MICALL2	MICAL-lik	2	1	1	1	904	97.4
Q9UHW5	GPN3	GPN-loop	7	2	2	2	284	32.7
Q6P161	MRPL54	39S ribos	7	1	1	1	138	15.8
Q9H2P9	DPH5	Diphthine	6	2	2	2	285	31.6
B0QY95	MIEF1	Mitochond	2	1	1	1	478	53.3
Q9H1A4	ANAPC1	Anaphase-	1	2	2	2	1944	216.4
J3KQ43	ALS2	Alsin (Fr	8	1	1	1	106	11.2
A6NHK2	SNRPE	Small nuc	23	1	1	1	52	5.9
F8VRX4	DDX54	ATP-depen	10	2	2	2	208	22.9
Q15628	TRADD	Tumor nec	6	1	1	1	312	34.2
Q14146	URB2	Unhealthy	1	1	1	1	1524	170.4
O95857	TSPAN13	Tetraspan	7	2	2	1	204	22.1
P58557	YBEY	Endoribon	7	1	1	1	167	19.3
P52435	POLR2J	DNA-direc	13	1	1	1	117	13.3
A0A024QZ3	NSRP1	Coiled-co	2	1	1	1	504	60.4
M0R176	MRPL51	39S ribos	34	1	1	1	32	4
P63218	GNG5	Guanine n	13	1	2	1	68	7.3
Q8NHV4	NEDD1	Protein N	1	1	1	1	660	71.9
F8VVY2	RNF41	E3 ubiqui	7	1	1	1	130	15
H3BNT2	COQ9	Ubiquinon	4	2	2	2	303	33.9
Q9BV81	EMC6	ER membra	9	1	1	1	110	12
Q8N5A5	ZGPAT	Zinc fing	2	1	1	1	531	57.3
Q8N653	LZTR1	Leucine-z	2	1	1	1	840	94.7

Q13287	NMI	N-myc-int	3	1	1	1	307	35
Q8N1Q1	CA13	Carbonic	3	1	1	1	262	29.4
J3QT51	CRBN	Protein c	4	1	1	1	228	26.2
C9JS61	ANKZF1	Ankyrin r	10	1	1	1	115	13.5
A0A087WUTMYEF2		Myelin ex	3	2	2	2	547	58.6
P58397	ADAMTS12	A disinte	1	2	2	2	1594	177.6
Q14249	ENDOG	Endonucle	3	1	1	1	297	32.6
O60942	RNGTT	mRNA-capp	1	1	1	1	597	68.5
A0A096LP2PTRH1		Probable	6	1	1	1	172	18.5
Q0VDG4	SCRN3	Secernin-	3	2	2	2	424	48.5
Q8TBQ9	TMEM167A	Protein k	13	1	1	1	72	8.1
Q9BQC6	MRPL57	Ribosomal	13	1	1	1	102	12.3
Q9NX47	MARCH5	E3 ubiqui	5	1	1	1	278	31.2
O95379	TNFAIP8	Tumor nec	7	2	3	2	198	23
Q86UL3	GPAT4	Glycerol-	2	1	1	1	456	52
Q9NYP7	ELOVL5	Elongatic	3	1	1	1	299	35.3
C9JUN5	CCDC12	Coiled-cc	11	1	1	1	89	10.5
E9PL46	FBXO3	F-box onl	5	2	3	2	129	15.2
Q9BQ48	MRPL34	39S ribos	11	1	1	1	92	10.2
P51178	PLCD1	1-phospha	2	2	2	2	756	85.6
Q86WC4	OSTM1	Osteopetr	3	1	1	1	334	37.2
Q8N584	TTC39C	Tetratric	2	1	1	1	583	65.8
E9PNG8	RNF170	E3 ubiqui	20	1	1	1	45	4.8
Q13322	GRB10	Growth fa	2	1	1	1	594	67.2
Q01831	XPC	DNA repai	2	2	2	2	940	105.9
D6RD44	TCEAL4	Transcrip	16	1	1	1	129	14.3
E7EW18	POLB	DNA polym	3	1	1	1	280	31.3
O43934	MFSD11	UNC93-lik	2	1	1	1	449	49.2
Q8TDB6	DTX3L	E3 ubiqui	1	1	1	1	740	83.5
P10915	HAPLN1	Hyalurona	3	1	1	1	354	40.1
P27144	AK4	Adenylate	4	1	1	1	223	25.3
E9PCT3	CAV2	Caveolin	16	2	2	2	113	12.9
G3V3R7	ATXN3	Ataxin-3	4	1	1	1	329	37.7
O75600	GCAT	2-amino-3	4	1	1	1	419	45.3
Q9H3L0	MMADHC	Methylmal	4	1	1	1	296	32.9
Q12923	PTPN13	Tyrosine-	0	1	1	1	2485	276.7
Q9Y2S6	TMA7	Translati	14	1	1	1	64	7.1
H0YG25	ETV6	Transcrip	9	1	1	1	88	10.3
H0YF29	C8orf82	UPF0598 p	7	1	1	1	261	28.6
A0A140T99PSMB8		Proteasom	5	1	1	1	252	27.8
Q8TF74	WIPF2	WAS/WASL-	2	1	1	1	440	46.3
Q8IW41	MAPKAPK5	MAP kinas	2	1	1	1	473	54.2
G3V4M9	ITPK1	Inositol	3	1	1	1	295	31.7
Q9BSY9	DESI2	Deubiquit	12	2	2	2	194	21.4
Q9NRY2	INIP	SOSS comp	13	1	1	1	104	11.4
A0A0U1RQYCSNK1G1		Casein ki	2	1	1	1	459	52.6
Q7L5Y9	MAEA	Macrophag	2	1	1	1	396	45.3
R4GNH2	FBXO2	F-box onl	13	1	1	1	120	12.8
Q9H490	PIGU	Phosphati	2	1	1	1	435	50
Q92604	LPGAT1	Acyl-CoA:	2	1	1	1	370	43.1
Q9BYC5	FUT8	Alpha-(1,	1	1	1	1	575	66.5

E9PGM9	RBM6	RNA-bindi	1	1	1	1	991	113.9
G5E994	GPR107	G protein	2	1	1	1	571	63.9
E9PHH9	POLR3C	DNA-direc	2	1	1	1	411	46.2
Q8TAF3	WDR48	WD repeat	1	1	1	1	677	76.2
Q8WUX9	CHMP7	Charged m	2	1	1	1	453	50.9
Q96HH9	GRAMD2B	GRAM doma	4	2	2	2	432	47.8
P05204	HMGN2	Non-histo	9	1	3	1	90	9.4
E5RI96	PDP1	[Pyruvate	6	1	1	1	147	16.8
O75592	MYCBP2	E3 ubiqui	0	2	2	2	4678	513.3
Q9NUN5	LMBRD1	Probable	3	1	1	1	540	61.3
P60604	UBE2G2	Ubiquitin	9	1	1	1	165	18.6
Q86UY6	NAA40	N-alpha-a	5	1	2	1	237	27.2
A2ACR1	PSMB9	Proteasom	5	1	1	1	196	20.9
Q14457	BECN1	Beclin-1	2	1	1	1	450	51.9
Q96SZ5	ADO	2-aminoet	3	1	1	1	270	29.7
F8VPW2	ZDHHC17	Palmitoyl	7	1	1	1	126	14.4
Q8WZA1	POMGNT1	Protein O	2	1	1	1	660	75.2
Q8N201	INTS1	Integrato	1	2	2	2	2190	244.1
Q8NB46	ANKRD52	Serine/th	1	1	1	1	1076	115
A0A0G2JHL	BRD2	Bromodoma	2	1	1	1	613	67.2
Q03393	PTS	6-pyruvoy	6	1	1	1	145	16.4
Q9BVT8	TMUB1	Transmemb	5	1	1	1	246	26.2
Q96B26	EXOSC8	Exosome c	4	1	1	1	276	30
Q9Y5B8	NME7	Nucleosid	3	1	1	1	376	42.5
Q9P032	NDUFAF4	NADH dehy	9	2	2	2	175	20.3
Q9BPX7	C7orf25	UPF0415 p	3	1	1	1	421	46.4
Q96CU9	FOXRED1	FAD-depen	4	2	2	2	486	53.8
A0A087WYM	SIRT2	NAD-depen	4	2	2	2	389	43.3
F8WF50	RABL3	Rab-like	14	2	2	2	129	14
F8VXD5	ORMDL2	ORM1-like	9	1	1	1	119	13.6
Q9UPY3	DICER1	Endoribon	1	1	1	1	1922	218.5
Q8NAV1	PRPF38A	Pre-mRNA-	3	1	1	1	312	37.5
O43598	DNPH1	2'-deoxyn	10	1	1	1	174	19.1
C9JJG2	POLR1B	DNA-direc	9	1	1	1	114	12.7
H0YEP3	NPEPL1	Probable	6	1	1	1	180	19.4
Q9ULW3	ABT1	Activator	3	1	1	1	272	31.1
Q9Y619	SLC25A15	Mitochond	3	1	1	1	301	32.7
F5H0Y3	MLF2	Myeloid l	4	1	1	1	230	24.7
F8WDV0	IPO11	Importin-	1	1	1	1	863	99.6
A0A087X0W	OTUD6B	Deubiquit	4	2	2	1	323	37.3
J3QRG5	CYBC1	Cytochrom	5	1	1	1	148	16.7
I3L2J0	CIC	Protein c	0	1	1	1	2514	257.6
Q9NVN8	GNL3L	Guanine n	1	1	1	1	582	65.5
Q96T58	SPEN	Msx2-inte	0	1	1	1	3664	402
Q9UBN7	HDAC6	Histone d	1	1	1	1	1215	131.3
H3BRK1	PARN	Poly(A)-s	3	1	1	1	261	29.4
A0A1B0GUM	CDKL5	Cyclin-de	1	1	1	1	881	98.8
E9PEY4	DTNB	Dystrobre	2	1	1	1	590	67
Q9BXX3	ANKRD30A	Ankyrin r	1	2	2	1	1397	158.7
A0A087X2C	AGRN	Agrin OS=	1	1	1	1	1930	202.2
Q5VTL8	PRPF38B	Pre-mRNA-	2	1	1	1	546	64.4

G3V5E8	GALC	Galactoce	4	1	1	1	211	23.5
C9JWL3	UBP1	Upstream-	9	1	1	1	148	16.8
Q9UBP9	GULP1	PTB domai	3	1	1	1	304	34.5
P19447	ERCC3	General t	1	1	1	1	782	89.2
P36959	GMPR	GMP reduc	3	1	1	1	345	37.4
Q8IWT6	LRRC8A	Volume-re	1	1	1	1	810	94.1
Q5W111	SPRYD7	SPRY doma	6	1	1	1	196	21.7
K7ELL0	GLYR1	Putative	15	1	1	1	52	5.9
Q8IXQ5	KLHL7	Kelch-lik	2	1	1	1	586	65.9
Q9UER7	DAXX	Death dom	1	1	1	1	740	81.3
K7ENL9	RMC1	Regulator	2	1	1	1	609	69.3
Q96B54	ZNF428	Zinc fing	7	1	1	1	188	20.5
Q96S15	WDR24	GATOR com	1	1	1	1	920	101.9
Q1RLN5	ARHGAP12	ARHGAP12	1	1	1	1	799	90.8
E7EPI0	IBTK	Inhibitor	1	2	2	2	1338	148.9
D6R9T3	HMCES	Embryonic	3	1	1	1	282	32.1
P49795	RGS19	Regulator	5	1	1	1	217	24.6
G3XAN8	TIMM8B	Mitochond	8	1	1	1	98	11.1
Q9NRK6	ABCB10	ATP-bindi	2	2	2	2	738	79.1
B8ZZC8	METTL5	Methyltra	3	1	1	1	244	27.8
O43734	TRAF3IP2	Adapter p	3	2	3	2	574	64.6
Q147X3	NAA30	N-alpha-a	3	1	1	1	362	39.3
E9PF16	ACSF2	Acyl-CoA	2	1	1	1	572	63.6
P62273	RPS29	40S ribos	27	2	4	2	56	6.7
C9JCU6	MAP4K2	Mitogen-a	4	1	1	1	307	34.7
Q9ULG6	CCPG1	Cell cycl	2	1	1	1	757	87.3
Q9NVH6	TMLHE	Trimethyl	2	1	1	1	421	49.5
Q6B0J5	HFE	HFE prote	6	1	1	1	345	39.7
P48553	TRAPPC10	Trafficki	1	1	1	1	1259	142.1
F8VSI7	TMBIM6	Bax inhib	6	1	1	1	138	15.1
Q5JPI3	C3orf38	Uncharact	4	1	1	1	329	37.5
H7C5U2	ITGB5	Integrin	2	1	1	1	401	43.7
J3KNE1	CDKN2AIP	CDKN2A-in	8	1	1	1	126	14
A0A087WUCMINOS1		MICOS com	13	1	1	1	62	7
Q9H1K6	TLNRD1	Talin rod	2	1	1	1	362	37.7
Q96Q05	TRAPPC9	Trafficki	1	1	1	1	1148	128.4
Q9C005	DPY30	Protein d	11	1	1	1	99	11.2
K7EIX4	TIMP2	Metallopr	7	1	1	1	109	12.2
Q9NRA2	SLC17A5	Sialin OS	2	1	1	1	495	54.6
Q9NQ92	COPRS	Coordinat	7	1	1	1	184	20.1
Q9BZM5	ULBP2	UL16-bind	4	1	1	1	246	27.4
Q9H2H9	SLC38A1	Sodium-co	2	1	1	1	487	54
Q9NUI1	DECR2	Peroxisom	4	1	1	1	292	30.8
O95229	ZWINT	ZW10 inte	4	1	1	1	277	31.3
F8W681	DPAGT1	UDP-N-ace	14	1	1	1	57	6.4
Q9UL42	PNMA2	Paraneopl	4	2	2	1	364	41.5
Q9BSR8	YIPF4	Protein Y	4	1	2	1	244	27.1
O14967	CLGN	Calmegin	1	1	1	1	610	70
H3BSB3	MPHOSPH6	M-phase p	8	1	1	1	131	15.5
Q9Y2K7	KDM2A	Lysine-sp	1	1	1	1	1162	132.7
O95684	FGFR1OP	FGFR1 onc	2	1	1	1	399	43

Q96BJ3	AIDA	Axin inte	2	1	1	1	306	35
Q9Y3D3	MRPS16	28S ribos	6	1	1	1	137	15.3
P10109	FDX1	Adrenodox	5	1	1	1	184	19.4
P61962	DCAF7	DDB1- and	4	1	1	1	342	38.9
P10588	NR2F6	Nuclear r	4	2	2	1	404	43
A0A0A0MTJ	SPOCK3	Sparc/ost	3	1	1	1	344	39.4
Q8N4Q1	CHCHD4	Mitochond	6	1	1	1	142	16
M0R2A0	EMC10	ER membra	2	1	1	1	371	39
F5H5A3	MAP4K3	Mitogen-a	1	1	1	1	810	91.9
Q9NUD5	ZCCHC3	Zinc fing	2	1	1	1	404	43.6
X6RM59	NT5C3A	5'-nuclec	3	1	1	1	331	37.4
R4GN70	ANKS1B	Ankyrin r	55	1	1	1	20	2.3
Q13888	GTF2H2	General t	2	1	1	1	395	44.4
Q8N3Y1	FBXW8	F-box/WD	2	1	1	1	598	67.4
S4R3I5	NDUFA3	NADH dehy	27	1	2	1	41	4.6
Q9BTT4	MED10	Mediator	7	1	1	1	135	15.7
Q13772	NCOA4	Nuclear r	1	1	1	1	614	69.7
Q14790	CASP8	Caspase-8	3	1	1	1	479	55.4
Q15022	SUZ12	Polycomb	1	1	1	1	739	83
Q9BVG9	PTDSS2	Phosphati	2	1	1	1	487	56.2
Q86YS7	C2CD5	C2 domain	1	1	1	1	1000	110.4
P29083	GTF2E1	General t	3	1	1	1	439	49.4
Q9BTX1	NDC1	Nucleopor	1	1	1	1	674	76.3
P50747	HLCS	Biotin--p	1	1	1	1	726	80.7
Q6ZTI6	RFLNA	Refilin-A	4	1	1	1	216	23.6
Q9H1C3	GLT8D2	Glycosylt	3	1	1	1	349	40
P05230	FGF1	Fibroblas	8	1	1	1	155	17.4
U3KQI2	MEIS2	Homeobox	5	1	1	1	235	26
A0A087WWF	RBM15	RNA-bindi	1	1	1	1	933	102.1
H0Y3V5	THADA	Thyroid a	1	1	1	1	1193	133.9
F8VRQ4	SMARCD1	SWI/SNF-r	4	1	1	1	313	36.8
Q14156	EFR3A	Protein E	1	1	1	1	821	92.9
Q07617	SPAG1	Sperm-ass	2	2	2	1	926	103.6
P20585	MSH3	DNA misma	1	1	1	1	1137	127.3
D6RG18	CCNH	Cyclin-H	3	1	1	1	255	29.5
Q96PX6	CCDC85A	Coiled-cc	2	1	1	1	553	59.9
E7EVJ3	NDST1	Bifunctic	1	1	1	1	825	94.3
Q86V87	FAM160B2	Protein F	1	1	1	1	743	82.3
Q99943	AGPAT1	1-acyl-sn	3	1	1	1	283	31.7
Q9H6E4	CCDC134	Coiled-cc	3	1	1	1	229	26.5
Q14393	GAS6	Growth ar	2	1	1	1	721	79.6
Q99959	PKP2	Plakophil	1	1	1	1	881	97.4
C9JFZ1	SYNJ1	Synaptoja	1	1	1	1	1350	149.2
O43156	TTI1	TELO2-int	1	1	1	1	1089	122
Q96CB9	NSUN4	5-methylc	3	1	1	1	384	43.1
P33947	KDELR2	ER lumen	4	1	1	1	212	24.4
Q96G28	CFAP36	Cilia- an	3	1	1	1	342	39.4
Q6YHK3	CD109	CD109 ant	1	1	1	1	1445	161.6
Q96B96	TMEM159	Promethin	5	1	2	1	161	17.5
Q9HAC8	UBTD1	Ubiquitin	6	2	2	2	227	25.9
A0A1B0GUU	IGHM	Immunoglc	2	1	1	1	474	51.9

K7EQF2	PLCD3	1-phospha	4	1	1	1	195	23
A0A0U1RRC	DOCK7	Dedicator	7	2	2	1	248	27
A0A0A0MRV	FAM213B	Prostamid	5	1	1	1	192	20.7
Q86UP3	ZFHX4	Zinc fing	0	2	2	2	3567	393.5
O75683	SURF6	Surfeit l	5	2	2	2	361	41.4
Q8TAC2	JOSD2	Josephin-	6	1	1	1	188	20.7
E9PS63	RABGAP1L	Rab GTPas	5	1	1	1	190	22.5
O14494	PLPP1	Phospholi	3	1	1	1	284	32.1
Q7L3B6	CDC37L1	Hsp90 co-	3	1	1	1	337	38.8
P08590	MYL3	Myosin li	8	1	2	1	195	21.9
A0A0A0MSZ	CSTF1	Cleavage	4	1	1	1	345	38.4
A0A0D9SGH	SLC9A6	Sodium/hy	1	1	1	1	679	75.8
Q5MNZ6	WDR45B	WD repeat	4	2	2	2	344	38.1
Q9NR48	ASH1L	Histone-l	0	1	3	1	2969	332.6
O94885	SASH1	SAM and S	1	1	1	1	1247	136.6
C9JVH1	VOPP1	Vesicular	5	1	1	1	170	19.1
P78504	JAG1	Protein j	1	1	1	1	1218	133.7
Q99541	PLIN2	Perilipin	2	1	1	1	437	48
F6RY50	SIPA1	Signal-in	1	1	1	1	940	101.8
A0A096LP1	ALG13	Putative	4	1	1	1	162	17.8
F8WDR3	AP4M1	AP-4 comp	11	1	1	1	64	6.8
A0A2R8Y6L	DYRK1A	Dual-spec	1	1	1	1	605	68.4
Q9BX59	TAPBPL	Tapasin-r	2	1	1	1	468	50.2
Q6ZWT7	MBOAT2	Lysophosp	2	1	1	1	520	59.5
O60220	TIMM8A	Mitochond	11	1	1	1	97	11
Q9P2X0	DPM3	Dolichol-	13	1	1	1	92	10.1
Q9P0U1	TOMM7	Mitochond	44	2	2	2	55	6.2
A0A0G2JH3	TAPBP	Tapasin C	2	1	1	1	466	49.5
Q8N573	OXR1	Oxidation	1	1	1	1	874	97.9
Q9Y6V7	DDX49	Probable	2	1	1	1	483	54.2
Q68D91	MBLAC2	Metallo-b	4	1	1	1	279	31.4
Q9P1Y5	CAMSAP3	Calmoduli	1	2	2	1	1249	134.7
Q9NQZ2	UTP3	Something	2	1	1	1	479	54.5
Q70CQ2	USP34	Ubiquitin	0	1	1	1	3546	404
Q9H1C7	CYSTM1	Cysteine-	10	1	1	1	97	10.6
O60285	NUAK1	NUAK fami	1	1	1	1	661	74.3
Q96I36	COX14	Cytochrom	12	1	1	1	57	6.6
Q9HAU0	PLEKHA5	Pleckstri	1	1	1	1	1116	127.4
O95251	KAT7	Histone a	1	1	1	1	611	70.6
Q9Y5Y2	NUBP2	Cytosolic	4	1	1	1	271	28.8
C9JWG4	SLC38A5	Sodium-co	3	1	1	1	272	29.3
Q7Z7F0	KHDC4	KH homolo	2	1	1	1	614	64.8
Q7RTN0	RTN2	Reticulon	2	1	1	1	461	50.4
A0A087WXF	ING1	Inhibitor	6	1	1	1	111	12.6
Q9ULR0	ISY1	Pre-mRNA-	3	1	1	1	285	33
Q969J3	BORCS5	BLOC-1-re	6	1	1	1	196	22.2
F8WDB4	LRWD1	Leucine-r	5	1	1	1	154	16.7
O43704	SULT1B1	Sulfotran	4	1	1	1	296	34.9
H0YAA3	WDR41	WD repeat	4	1	1	1	394	44.5
Q8WZ19	KCTD13	BTB/POZ d	2	1	1	1	329	36.3
E9PKE9	RNF121	RING fing	4	1	1	1	167	19.5

P62877	RBX1	E3 ubiqui	7	1	2	1	108	12.3
C9J0E4	CSTA	Cystatin-	11	1	1	1	63	7.1
Q04844	CHRNE	Acetylcho	1	1	1	1	493	54.7
Q9BWW4	SSBP3	Single-st	3	1	1	1	388	40.4
Q9NVT9	ARMC1	Armadillo	3	1	1	1	282	31.3
B5MDL5	MAPK12	Mitogen-a	3	1	1	1	277	31.8
Q9Y6Y0	IVNS1ABP	Influenza	2	1	1	1	642	71.7
Q96EL2	MRPS24	28S ribos	5	1	1	1	167	19
Q6XYQ8	SYT10	Synaptota	2	1	1	1	523	59.1
Q8WWC4	MAIP1	m-AAA pro	3	1	1	1	291	32.5
B8ZZW5	AVL9	Late secr	1	1	1	1	630	69.8
J3QL63	TVP23B	Golgi app	5	1	1	1	141	16.3
Q17RB0	RTL8B	Retrotran	6	1	1	1	113	13.2
Q14966	ZNF638	Zinc fing	1	1	1	1	1978	220.5
Q13356	PPIL2	RING-type	2	1	1	1	520	58.8
Q8IY63	AMOTL1	Angiomoti	1	1	1	1	956	106.5
Q9Y3M8	STARD13	StAR-rela	1	1	1	1	1113	124.9
Q96NL6	SCLT1	Sodium ch	1	1	1	1	688	80.9
Q92935	EXTL1	Exostosin	1	1	1	1	676	74.6
B1ALY0	PALM2-AKA	PALM2-AKA	2	1	1	1	433	47.6
A0A0C4DGW	C5orf51	UPF0600 p	5	1	1	1	152	17
Q5QPE8	MGME1	Mitochond	3	1	1	1	264	30
Q68DQ2	CRYBG3	Very larg	0	1	1	1	2970	330.4
F8W8Y1	NTM	Neurotrim	5	1	1	1	179	19.7
Q96DY7	MTBP	Mdm2-bind	1	1	1	1	904	102.1
X1WI34	MARC2	Mitochond	5	1	1	1	172	19.8
E9PNL8	DGKZ	Diacylgly	1	1	1	1	707	78.9
Q9NV96	TMEM30A	Cell cycl	3	1	1	1	361	40.7
A0A087WYF	DTNBP1	Dysbindin	3	1	1	1	316	35.5
L0R6Q1	SLC35A4	SLC35A4 u	7	1	1	1	103	11.1
P15407	FOSL1	Fos-relat	3	1	1	1	271	29.4
Q96HQ2	CDKN2AIPN	CDKN2AIP	6	1	1	1	116	13.2
O60563	CCNT1	Cyclin-T1	1	1	1	1	726	80.6
P16220	CREB1	Cyclic AM	2	1	1	1	341	36.7
O43715	TRIAP1	TP53-regu	11	1	1	1	76	8.8
Q5VTE6	ANGEL2	Protein a	2	1	1	1	544	62.3
O43861	ATP9B	Probable	1	1	1	1	1147	129.2
A0A0S2Z43	NEFL	Neurofila	2	1	3	1	284	32.6
C9J719	EBP	3-beta-hy	7	1	1	1	145	16.6
C9J2P0	UBE2E1	Ubiquitin	5	1	1	1	147	16.2
A0A087WVS	MOB2	MOB kinas	3	1	1	1	234	26.6
P13498	CYBA	Cytochrom	5	1	1	1	195	21
E9PHT3	GRAMD1C	GRAM doma	2	1	2	1	495	56.5
Q96AT1	KIAA1143	Uncharact	5	1	1	1	154	17.5
Q8TEA7	TBCK	TBC domai	1	1	1	1	893	100.6
P23229	ITGA6	Integrin	1	1	1	1	1130	126.5
P49662	CASP4	Caspase-4	2	1	1	1	377	43.2
Q3LIE7	DHCR24	Delta(24)	2	1	1	1	427	49.4
U3KQ50	TARS2	Threonine	7	1	1	1	143	15.4
Q5JUR7	TEX30	Testis-ex	5	1	1	1	227	25.6
O14548	COX7A2L	Cytochrom	8	1	1	1	114	12.6

H0YCA5	SPATA5L1	Spermatog	3	1	1	1	258	28.6
B5MD58	SREBF1	Sterol re	1	1	1	1	893	95.8
G3V5K2	NEMP1	Nuclear e	8	1	1	1	110	12
A2ABF8	EHMT2	Histone-l	1	1	1	1	1233	135.3
Q7Z2T5	TRMT1L	TRMT1-lik	1	1	1	1	733	81.7
O15120	AGPAT2	1-acyl-sn	3	1	1	1	278	30.9
G3V1J0	WDR55	WD repeat	4	1	1	1	222	24.5
M0QZR8	POLD1	DNA polym	1	1	1	1	1009	111.6
Q9Y4D1	DAAM1	Dishevele	1	1	1	1	1078	123.4
C9JD84	LTBP1	Latent-tr	1	1	1	1	1340	146.9
Q02487	DSC2	Desmocoll	1	1	1	1	901	99.9
G5E9F5	MPV17	MpV17 tra	7	1	1	1	113	13
Q9Y5T4	DNAJC15	DnaJ homo	6	1	1	1	150	16.4
P05496	ATP5MC1	ATP synth	5	1	3	1	136	14.3
H9KV31	NCAM2	Neural ce	1	1	1	1	819	91.1
C9JPV1	SLC6A6	Transport	8	1	1	1	109	12.1
G3V2H7	TRAPPC6B	Trafficki	8	1	1	1	102	11.7
Q2KHT3	CLEC16A	Protein C	1	1	2	1	1053	117.6
Q8IYK4	COLGALT2	Procollag	1	1	1	1	626	72.9
P42685	FRK	Tyrosine-	2	1	1	1	505	58.2
Q9H2K0	MTIF3	Translati	3	1	1	1	278	31.7
H0YAB2	PLXND1	Plexin-D1	3	1	1	1	380	43.6
H0Y9C8	FAT1	Protocadh	4	1	1	1	285	31.4
P57764	GSDMD	Gasdermin	1	1	1	1	484	52.8
Q6PJF5	RHBDF2	Inactive	1	1	2	1	856	96.6
A4FU01	MTMR11	Myotubula	1	1	2	1	709	79.5
O95503	CBX6	Chromobox	2	1	1	1	412	43.9
Q13206	DDX10	Probable	1	1	1	1	875	100.8
X6RK58	LRRC20	Leucine-r	6	1	1	1	174	19.5
K7ELQ4		Uncharact	2	1	1	1	463	49.9
P53803	POLR2K	DNA-direc	12	1	1	1	58	7
F8VRE5	YAF2	YY1-assoc	10	1	1	1	72	8.1
A0A0U1RQJATRIP		ATR-inter	2	1	2	1	604	65.2
Q53F19	NCBP3	Nuclear c	2	1	1	1	620	70.5
H7C3Y7	NBEAL2	Neurobeac	1	1	1	1	1093	121.6
K7EL74	VMP1	Vacuole m	9	1	1	1	75	9
H3BQG1	CLK3	Dual-spec	2	1	1	1	306	36.4
G5EA09	SDCBP	Syndecan	2	1	1	1	318	34.8
Q9H0C5	BTBD1	BTB/POZ d	2	1	1	1	482	52.7
F5H450	FZD10	Frizzled-	2	1	1	1	454	48.7
Q5BJD5	TMEM41B	Transmemb	3	1	1	1	291	32.5
C9J5X1	IGF1R	Tyrosine-	1	1	1	1	1366	154.7
Q96B70	LENG9	Leukocyte	2	1	1	1	501	53.1
D6REA0	GATB	Glutamyl-	1	1	1	1	516	57.6
F5GWH5	TMEM258	Transmemb	16	1	1	1	49	5.7
P50461	CSRP3	Cysteine	4	1	2	1	194	21
Q9BWJ5	SF3B5	Splicing	13	1	1	1	86	10.1
P17152	TMEM11	Transmemb	4	1	1	1	192	21.5
Q9BW60	ELOVL1	Elongatio	5	1	1	1	279	32.6
Q9HCJ1	ANKH	Progressi	2	1	1	1	492	54.2
O60934	NBN	Nibrin OS	1	1	1	1	754	84.9

Q9H0J9	PARP12	Poly [ADF	1	1	1	1	701	79
Q8N697	SLC15A4	Solute ca	1	1	1	1	577	62
Q9Y2X9	ZNF281	Zinc fing	1	1	1	1	895	96.9
Q86X02	CDR2L	Cerebella	2	1	1	1	465	53
O60637	TSPAN3	Tetraspar	3	1	1	1	253	28
E9PKQ5	CCDC90B	Coiled-cc	3	1	1	1	208	24.3
Q9BZA7	PCDH11X	Protocadh	1	1	1	1	1347	147.5
Q6Y1H2	HACD2	Very-long	4	1	1	1	254	28.4
P50151	GNG10	Guanine r	10	1	1	1	68	7.2
Q9H5V8	CDCP1	CUB domai	1	1	1	1	836	92.9
O15344	MID1	E3 ubiqui	1	1	1	1	667	75.2
A0A2R8Y4I	SPAST	Spastin C	1	1	1	1	583	63.5
Q8NEC6	MTMR1	MTMR1 prc	2	1	1	1	363	39.8
Q9UK59	DBR1	Lariat de	1	1	1	1	544	61.5
A0A087WW6	ABCB7	ATP-bindi	1	1	1	1	713	78.2
Q69YN4	VIRMA	Protein v	0	1	1	1	1812	201.9
O43657	TSPAN6	Tetraspar	3	1	1	1	245	27.5
A0A0G2JJD	ABHD16A	HLA-B ass	1	1	1	1	601	67.5
Q9H147	DNTTIP1	Deoxynucl	2	1	1	1	329	37
P28347	TEAD1	Transcrip	2	1	1	1	426	47.9
Q9BRV8	SIKE1	Suppressc	3	1	1	1	207	23.7
Q9H8H2	DDX31	Probable	1	1	1	1	851	94
H0YEU7	CREB3L1	Cyclic AM	7	1	1	1	123	13.9
A0A2R8Y5Q	SLC11A2	Natural r	3	1	1	1	331	36.4
Q9P000	COMMD9	COMM doma	4	1	1	1	198	21.8
A0A087X0T	NTAN1	Protein N	3	1	1	1	205	23.4
H0YCP6	TMEM63B	CSC1-like	2	1	1	1	520	60.5
P00451	F8	Coagulati	0	1	1	1	2351	266.8
Q8TE73	DNAH5	Dynein he	0	1	1	1	4624	528.7
C9JE50	UXS1	UDP-glucu	4	1	1	1	170	18.8
Q9BTC0	DIDO1	Death-ind	0	1	1	1	2240	243.7
C9JTA8	C2orf76	UPF0538 p	8	1	1	1	85	9.6
Q9Y244	POMP	Proteasom	5	1	1	1	141	15.8
H0YLH9	IL16	Pro-inter	1	1	4	1	611	64.6
O60239	SH3BP5	SH3 domai	2	1	1	1	455	50.4
E9PEE2	NCK2	Cytoplasm	6	1	1	1	110	12.6
A0A2R8YEX	OSBPL2	Oxysterol	2	1	1	1	413	47.9
A0A087WT9	LAMTOR4	Ragulator	10	1	1	1	72	7.9
Q7Z422	SZRD1	SUZ domai	5	1	1	1	152	17
Q6V1X1	DPP8	Dipeptidy	1	1	1	1	898	103.3
Q06190	PPP2R3A	Serine/th	1	1	1	1	1150	130.2
Q5T5C0	STXBP5	Syntaxin-	1	1	1	1	1151	127.5
I3L2S8	NDE1	Nuclear d	6	1	1	1	127	15.2
Q9BXT8	RNF17	RING fing	0	1	1	1	1623	184.5
A0A0J9YWW	APOO	MICOS com	8	1	1	1	90	10
H0YCN4	DCUN1D5	DCN1-like	4	1	2	1	195	22.9
P55789	GFER	FAD-linke	6	1	1	1	205	23.4
B1AL33	UHRF2	E3 ubiqui	7	1	1	1	136	15.3
Q9NXF7	DCAF16	DDB1- and	4	1	1	1	216	24.2
Q96SK2	TMEM209	Transmemb	1	1	1	1	561	62.9
Q9H1K0	RBSN	Rabenosyr	1	1	1	1	784	88.8

Q8TCU6	PREX1	Phosphati	0	1	1	1	1659	186.1
Q9BZL1	UBL5	Ubiquitin	8	1	1	1	73	8.5
Q5BJF2	TMEM97	Sigma int	5	1	1	1	176	20.8
O75030	MITF	Microphth	1	1	1	1	526	58.8
Q9BW61	DDA1	DET1- and	8	1	1	1	102	11.8
Q8WVX9	FAR1	Fatty acy	2	1	2	1	515	59.3
Q8NI51	CTCFL	Transcrip	1	1	1	1	663	75.7
Q9UJX6	ANAPC2	Anaphase-	1	1	1	1	822	93.8
P0C860	MSL3P1	Putative	1	1	1	1	447	51
K7ELS8	SYNGR2	Synaptogy	6	1	1	1	135	14.7
Q9Y2J4	AMOTL2	Angiomoti	1	1	1	1	779	85.7
Q5JR91	KIF2C	Kinesin-l	2	1	1	1	336	37.9
O43663	PRC1	Protein r	1	1	1	1	620	71.6
Q9BZI7	UPF3B	Regulator	2	1	1	1	483	57.7
Q9H7P9	PLEKHG2	Pleckstri	1	1	1	1	1386	147.9
A2A2F0	RALGAPB	Ral GTPas	1	1	1	1	1323	147.3
Q15648	MED1	Mediator	1	1	1	1	1581	168.4
J3QS80	C19orf47	Uncharact	6	1	1	1	168	17.7
I3L130	URI1	Unconvent	16	1	1	1	50	5.5
O75164	KDM4A	Lysine-sp	1	1	1	1	1064	120.6
Q9UJA3	MCM8	DNA helic	1	1	2	1	840	93.6
Q86V42	FAM124A	Protein F	1	1	1	1	546	60.1
Q6ZWJ1	STXBP4	Syntaxin-	1	1	1	1	553	61.6
Q9ULD2	MTUS1	Microtubu	1	1	1	1	1270	141.3
G3V515	ZFP36L1	mRNA deca	4	1	1	1	189	20.4
K7EK57	WIPI1	WD repeat	2	1	1	1	369	41.1
Q9P2K3	RCOR3	REST core	2	1	1	1	495	55.5
H3BNK2	LYRM1	LYR motif	7	1	1	1	107	12.4
Q3KRB8	ARHGAP11E	Rho GTPas	2	1	1	1	267	30.2
E9PGH5	CYP2U1	Cytochrom	2	1	1	1	335	38.7
O95926	SYF2	Pre-mRNA-	2	1	1	1	243	28.7
E9PR71	UVRAG	UV radiat	1	1	1	1	598	67
H7BYP1	TRPM3	Transient	1	1	1	1	1556	177.7
K7EIY6	RNF126	E3 ubiqui	3	1	1	1	283	30.8
K7EPC4	KAT2A	Histone a	9	1	2	1	70	8.4
Q9UHA4	LAMTOR3	Ragulator	6	1	1	1	124	13.6
E9PCW1	GOSR1	Golgi SNA	3	1	1	1	248	28.3
O75818	RPP40	Ribonucle	2	1	1	1	363	41.8
A0A2R8Y4Z	CCDC9	Coiled-cc	1	1	1	1	587	65.3
Q8N2G8	GHDC	GH3 domai	2	1	1	1	530	57.5
O95239	KIF4A	Chromosom	1	1	1	1	1232	139.8
J3KNF5	CEP290	Centrosom	0	1	1	1	2481	290.4
Q9P0B6	CCDC167	Coiled-cc	6	1	1	1	97	11.5
O95210	STBD1	Starch-bi	2	1	1	1	358	39
Q96IG2	FBXL20	F-box/LRR	2	1	1	1	436	48.4
Q01974	ROR2	Tyrosine-	1	1	1	1	943	104.7
Q6AWC2	WWC2	Protein W	1	1	1	1	1192	133.8
H0YC48	HACE1	E3 ubiqui	4	1	1	1	175	19.9
A0A087WYI	INTS11	Integrato	1	1	1	1	502	56.3
Q8IUA7	ABCA9	ATP-bindi	0	1	1	1	1624	184.2
Q8WZ42	TTN	Titin OS=	0	1	2	1	34350	3813.7

E9PFH2	KDM5D	Lysine-sp	0	1	1	1	1476	166.8
P24311	COX7B	Cytochrom	9	1	1	1	80	9.2
Q96NW7	LRRC7	Leucine-r	0	1	1	1	1537	172.5
P18615	NELFE	Negative	3	1	1	1	380	43.2
Q8NA19	L3MBTL4	Lethal(3)	1	1	1	1	623	71.1
O94887	FARP2	FERM, ARH	1	1	1	1	1054	119.8
Q8WWI5	SLC44A1	Choline t	1	1	1	1	657	73.3
O95644	NFATC1	Nuclear f	1	1	1	1	943	101.2
Q9UKK6	NXT1	NTF2-rela	5	1	1	1	140	15.8
F8VWZ8	ARHGAP29	Rho GTPas	1	1	1	1	1035	117.4
Q14520	HABP2	Hyalurona	2	1	1	1	560	62.6
A0A087X0C	GLS2	Glutamina	2	1	1	1	337	37.1
Q15835	GRK1	Rhodopsin	2	1	1	1	563	63.5
P01008	SERPINC1	Antithrom	2	1	1	1	464	52.6
Q5T2S9	ARMC4	Armadillo	2	1	1	1	389	44.3
Q8IWV8	UBR2	E3 ubiqui	0	1	1	1	1755	200.4
Q12933	TRAF2	TNF recep	2	1	1	1	501	55.8
Q9Y227	ENTPD4	Ectonucle	1	1	1	1	616	70.2
A6NC98	CCDC88B	Coiled-co	1	1	1	1	1476	164.7
Q96FI4	NEIL1	Endonucle	2	1	1	1	390	43.7
Q8TD26	CHD6	Chromodom	0	1	1	1	2715	305.2
Q7Z7E8	UBE2Q1	Ubiquitin	1	1	1	1	422	46.1
E9PKV2	MRPL17	39S ribos	6	1	1	1	142	16.4
Q3MIP1	ITPRIPL2	Inositol	2	1	1	1	535	58.4
Q9BZ68	FRMD8P1	Putative	2	1	1	1	369	41.1
P46019	PHKA2	Phosphory	1	1	1	1	1235	138.3
Q9C0D5	TANC1	Protein T	0	1	1	1	1861	202.1
O15235	MRPS12	28S ribos	6	1	1	1	138	15.2
A0A0A0MSC	SLIT3	Slit homo	1	1	1	1	1393	153.7
H7BZN3	RIF1	Telomere-	1	1	1	1	845	93.6
Q9UBQ6	EXTL2	Exostosin	2	1	1	1	330	37.4
Q8N2G6	ZCCHC24	Zinc fing	4	1	1	1	241	26.9
Q9BXY5	CAPS2	Calcyphos	1	1	1	1	557	63.8
H0YBS1	INTS8	Integrato	1	1	1	1	800	91.2
P51854	TKTL1	Transketo	1	1	1	1	596	65.3
H0YBT8	TACC1	Transform	2	1	1	1	454	50.5
Q9BYN0	SRXN1	Sulfiredo	5	1	1	1	137	14.3
Q86YF9	DZIP1	Zinc fing	1	1	1	1	867	98.6
H0YMT3	WDR73	WD repeat	3	1	1	1	178	20.1
O60673	REV3L	DNA polym	0	1	1	1	3130	352.6
M0R228	KXD1	KxDL moti	5	1	1	1	131	15
Q9P2G4	MAP10	Microtubu	1	1	1	1	905	100.3
Q6ZW61	BBS12	Bardet-Bi	1	1	1	1	710	79
O00635	TRIM38	E3 ubiqui	1	1	1	1	465	53.4
Q16654	PDK4	[Pyruvate	1	1	1	1	411	46.4
K7ERQ2	FAM210A	Protein F	4	1	1	1	178	20
P38936	CDKN1A	Cyclin-de	4	1	1	1	164	18.1
Q8N1G2	CMTR1	Cap-speci	1	1	1	1	835	95.3
A6NF31	OFD1	Oral-faci	1	1	1	1	872	100.9
Q6TFL3	CCDC171	Coiled-co	1	1	2	1	1326	152.7
P55199	ELL	RNA polym	1	1	1	1	621	68.2

Q02410	APBA1	Amyloid-b	1	1	2	1	837	92.8

calc. pI	Score Seq	Abundance	Abundance	Abundance	Abundance	Abundance	Abundances (Groupe
6.15	2040.52	90.7	92.4	91	104.7	103.3	103.8
5.96	1836.84	90.9	90.5	89.6	112.4	112	112
6.06	2706	97.7	98.1	97.4	93.8	93	93.1
5.6	2625.4	101.3	102.1	101.5	90.5	90.1	89.7
6.07	1495.51	102	102.5	101.5	93.8	93.7	93.6
5.73	1261.9	96.4	96.9	96.2	102.6	102.9	102.7
5.97	1330.66	92.8	93.6	93.3	104.9	104.4	104.7
6.4	922.24	95.5	95.2	95.3	97.7	97.3	98
5.35	777.91	101.5	103.5	101.7	106.4	105.6	105.1
5.34	762.21	96.4	94.1	109.1	104.5	111.1	109.9
5.54	857.63	101.5	100.9	100.2	89.2	89.4	89.1
5.39	601.03	97.9	96.3	96.3	95.6	96.4	97.1
5.39	593.09	98.2	97.5	106.4	98.2	105.5	100.9
5.57	645.15	101	101.3	102.2	106.2	105.6	105.8
5.41	1110.66	95.9	96.5	97	92.7	92.3	93.2
5.66	843.7	100.5	101.5	100.5	91.5	90.5	91.1
5.8	753.37	92.7	91.8	92.6	119.9	120.6	121.1
6.18	564.45	99.6	100	98.4	93.5	93	93.8
5.69	664.25	96.4	97.5	96.6	99.3	98.9	97.6
4.81	562.95	99.2	100.3	99.9	111.7	112.3	111.2
7.12	457.43	100.5	101.8	100.8	106	107.4	106.8
5.69	998.92	102	100.9	101.6	88.2	83.2	84
6.48	562.2	99.3	99.7	100.7	95.1	94.9	95.9
5.71	593.87	106.8	107.6	106.1	87.9	88.4	87.9
5.44	911.8	100.8	101.4	100.9	91.4	91.1	92.1
8.6	556.35	108.9	110.1	109	118.7	116.8	117.1
5.53	478.19	71.7	70.1	69.8	98.5	98.7	98.5
6.44	447.88	96.2	96.8	96	102.2	102.5	103.2
5.16	963.45	100.7	101.9	100.9	100	99.6	99.9
7.84	1025.25	100.9	101.2	101.3	96.8	96.5	96.9
5.03	785.47	101.7	102.4	102.6	104.1	103.3	103.7
5.52	692.49	99.9	100.7	101.1	102	102.2	102.3
9.01	474.45	90.9	91	90	118.5	118	119.5
7.33	388.27	99.9	98.7	99.7	99.3	99	100.4
5.02	663.78	99.1	99.2	99.8	102.9	102	101.4
5.12	1700.09	102.4	101.9	106.9	98.3	105.1	100.5
6.83	622.13	96.6	97.9	96.6	105.9	105	104.8
5.43	376.29	117.3	86.8	101.1	137.9	138.1	139.3
6.23	372.1	101.5	101.6	102.2	116.1	116.3	116
7.02	619.15	108.5	109.5	108.5	96.7	96.8	96.6
7.33	497.24	93.1	92.4	91.7	105.3	105.1	105.4
5.19	364.47	98.2	98.3	98.5	99.7	100.2	101.1
5.17	3029.45	96.4	98.4	100.7	92	97.9	94.6
5.6	463.35	99	99.6	98.1	91.8	91.9	90.5
6.11	574.02	89.1	91.9	90.5	86.6	87.3	85.4
7.02	580.84	103.6	96.1	91.1	88.9	109	105.4
5.92	576.4	101.5	100.9	100.7	97.2	95.8	96.2
5.66	580.7	94.3	100.2	95	77.7	79.5	75.6
7.47	311.28	102.3	100.8	99.5	99.9	98.1	100
5.59	665.25	102.1	102.8	102.7	73.6	73.6	74.1

6.4	539.52	105.5	106.7	106.6	96.5	96.4	95.1
4.84	656.67	105.8	106.9	105.1	93.2	94.1	93.3
6.68	295.45	114	113.6	113.1	102.3	101.7	103.5
8.69	588.65	101.6	101.4	105.2	93.9	97.1	94.8
5.26	364.14	97.5	97.9	97.8	99.2	99.7	98.8
5.39	1684.6	96.1	97.4	102.6	88.5	97.9	92
5.07	408.05	104.3	105.6	104.6	97.5	96.4	96.1
5.22	320.72	104.2	104.2	103	96.7	95.8	95.9
5.48	356.81	98.2	99.7	97.7	102.5	100.5	102.4
8.46	1270.02	102.9	103.3	107	91.3	97.2	94
5.6	435.62	99.6	100.4	100.4	94.5	93.7	93.8
4.87	607.95	100.5	101.6	102.1	93.6	94	93.1
6.87	339.37	99.6	100.3	99.3	101.4	100.7	100.5
5.76	325.11	96	96.5	95.6	100.6	100.4	99.2
8.03	669.63	107.4	105.2	101.8	86.9	90	86.9
5.64	258.95	102.9	102.6	103.4	97.7	98.1	97.9
6.35	492.27	105.7	105.3	104.4	94.3	94.7	95.2
6.51	233.29	98.5	99.4	99.3	98	96.4	97.8
4.94	259.52	99.8	99.7	98.9	99.4	98.4	97.6
7.66	291.12	99.9	99.4	99.2	99.2	99.6	99.4
8.12	358.14	129.8	131.4	130.4	81.5	79.5	80.8
5.87	349.5	97.8	99.1	99.2	106.3	106.2	105.9
5.6	333.38	100.4	99.7	99.4	103.7	101.9	102.6
5.39	244.39	99	97.1	98.1	96.6	95.8	96.4
7.55	314.77	108	104.2	104.2	94.5	95	92.4
9.01	1043.15	99.1	102.1	100.5	98.9	100	98.5
7.39	497.84	98.3	98.9	100.3	100.3	102.9	100.6
7.17	310.44	99.3	98.7	95.4	98.8	96.7	96.5
6.13	236.22	98.6	96.4	95.9	104.9	105	104.8
5.02	215.04	105.4	104.2	105.5	105.3	105.4	106.3
6.16	316.63	94.5	94.7	95.2	106.1	105.3	105.9
5.31	238.78	104.1	98.2	102	90.6	91.9	93.7
5.33	238.26	100.1	101.1	99.9	102.8	101.5	102.6
8.1	402.02	98.6	98.8	96	98.2	97.8	97.2
4.44	517.21	101.4	104.3	104.3	97.7	98.7	97.1
5.67	223.09	99.5	97.8	99.2	97	96.2	96.9
7.75	698.07	93.1	93.3	94.3	98.5	102	99.6
5.06	672.79	97.6	98.9	99.5	99.4	98.9	100.3
6.61	305.5	97.3	96.5	97.5	110.8	112.4	113.7
4.89	780.04	95.9	96.7	96.4	101.1	100.3	101.8
5.07	677.98	95.7	98	98	100.4	103.4	101.6
7.83	267.22	98.9	99.7	100.5	102.3	100.3	102.9
6.52	238.44	101.2	102.2	101.4	95	95.6	94.2
6.65	383.58	101.3	104.7	104.9	91	92.9	92
5.45	393.54	108.3	108.6	106	63.5	63	64.6
5.22	324.77	111.4	111.1	110.5	66.6	66.1	65.8
5.66	270.38	97.7	98.4	98.7	103	102.7	104.5
4.89	710.34	98.1	96.2	97.8	102.6	102	104.5
8.7	274.79	102.6	103.3	104.1	104.9	104.5	105.2
6.49	257.74	99.4	98.4	97.7	102.1	102.2	104.2
5.39	224.59	96.7	97.5	97.1	107	106.7	109.6

5.48	326.16	97.2	100.1	98	107.3	106.5	105.6
5.34	239.52	98.3	97.1	97.2	101.6	101.9	101.1
6.46	259.17	99.4	99.2	100.4	102.4	102.4	102.1
6.65	346.92	84.8	91.7	85.6	105.4	103	105.6
6.29	216.55	96.8	97.1	97	98.6	97.4	99.2
5.66	346.73	92.4	92.1	91.3	124.5	123.5	123.4
7.02	395.08	94.9	96.3	96.1	102	100.4	100.1
6.27	341.63	101.9	103.8	102.1	98.8	95.6	97.9
5.68	185.02	98.2	99.5	102.5	101.9	105.1	102.8
5.53	201.2	100	100.8	100.1	96.4	93.9	93.2
8.06	298.41	101.8	104.1	103.3	92.5	92.8	91.2
6.79	211.79	99.1	98.8	97.7	105.6	105.1	106.4
4.72	423.85	98.1	100.1	98.9	92.9	92.3	91.6
4.93	176.58	92.9	93.4	92.9	108.9	108	109.2
6.11	222.33	100.5	98.9	98.6	102.1	102.4	105.3
5.12	203.95	104.5	101	100.5	102.4	101.3	102.7
6.05	215.75	97.8	98.8	100.1	100.7	100.4	99.1
8.84	751.36	98	99.3	100.8	78.5	81.6	80
7.18	174.58	93.5	92.2	93.3	90	87.8	89.1
6.64	234.67	102.2	104	102.1	108.4	107.3	106.6
6.47	246	99	98.4	98.3	98.6	98.2	99.8
8	198.57	111.4	112.7	111.4	90.1	91.7	91.7
5.74	274.59	92	93.4	93.2	80.7	79.6	81.1
6.05	263.41	98.7	95.6	96	94.8	89.7	84.8
5.47	224.15	98.3	99.3	98.7	98.4	99.8	99.7
6.71	292.31	86.9	87.4	85.6	105.2	105.3	104.8
8.09	575.72	99.6	99.4	97.5	93.5	91.8	91.4
5.49	183.56	102.7	102.5	100.8	99.2	98.3	99.2
5.21	221.86	109.7	111.3	110.6	88.2	89.5	88.8
7.65	222.86	99.7	100.8	101	101.8	101.5	100.5
4.74	398.46	96.9	98.7	97.2	84.4	86	84.8
7.3	168.03	109.9	124	97.6	68.1	83.3	83.6
4.69	485.33	100	101	99	92.6	92	91.3
6.21	177.91	99.1	98.4	99	96.7	95.5	97.3
5.38	229.89	97.2	97.3	97.2	98.3	97.4	98.3
6.8	214.08	104.3	102	102.3	97	95.9	97.4
5.4	265.07	100.1	98.5	100.6	102.4	102	102.1
4.89	660.26	91.4	84.9	93.5	107	91.3	111.3
8.75	136.1	101.5	98.6	101.7	96.1	94.3	97
5.14	247.87	81.2	77.9	76.7	115.9	117.3	116.8
6.15	181.08	97.7	99.7	98.5	94.6	97.3	94.7
6.49	240.3	103.2	103.5	102.6	94.8	94.4	95.6
5.27	217.99	100.7	100.7	100.3	100.2	101.3	99.6
7.3	186.63	100.9	99.5	98.5	102.3	101.6	102
4.94	183.4	107.9	104.9	106.3	95.3	93.5	95.4
5.62	298.64	101.8	104.5	101.3	99.3	98.9	98.5
6.8	218.09	99.3	102.2	101.5	105.6	102.8	103.4
9.41	180.84	98.1	95.6	96.5	95.7	96.5	98.6
6.84	209.91	102	99.6	102.2	108.6	106.1	105.9
6.01	228.31	101.2	103.3	101.4	95.2	94.4	94.1
6	263.37	102.4	104.2	104	113.2	113.1	112.9

5.78	263.12	97.9	102.8	97.4	137.8	125.6	128.8
7.8	147.9	99.4	101	100.2	105.7	104.4	105.4
8.34	412.54	94.3	95.9	96.1	100.5	100.2	99.1
8.84	150.19	103.8	103.5	103.2	107.5	106.8	107.6
9.1	180.2	96.1	95.5	95	97.8	99.9	98.3
5.63	160.24	101.4	100.6	101.6	98	97.5	98.2
4.93	400.1	103.2	103.1	101.5	97.8	98.2	102.8
5.36	151.4	90.4	90.4	89.8	116.7	117.8	118
5.78	215.14	101	99.9	100.7	97	96	97.3
6.68	196.89	101.2	99.9	99.5	98.4	97.2	97.1
7.46	178.37	99.1	98.6	99.3	98	97.6	97.2
4.45	202.45	94.9	93.2	97	113.5	110.7	112.6
4.64	211.95	96	96.8	97.2	111.1	110.3	109.6
5.92	305.63	104.1	104.8	103.4	97.8	98.2	96.7
7.66	213.96	101.3	101.1	100.8	100.1	100.6	100.2
6.86	186.91	103.3	102.7	103.5	86.8	86	87.8
5.53	117.9	99	98.7	99.6	95.5	97	97.4
5.39	286.77	101.8	102.9	103.5	98.9	98.8	99.6
5.06	169.7	94.5	95.6	94.1	98.3	96.9	98.5
6.9	163.53	103.4	103.7	103.9	98.9	97.9	98
4.74	244.87	99.7	100.9	101.6	100.3	100.3	101
5.44	272.3	96.7	97.9	95.8	95	95.4	96.3
5.24	210.61	136.7	140.5	141.3	83.2	80.3	81.9
4.7	223.95	102.7	103.2	103.2	117.4	115.6	116.2
6.61	163.36	96.8	97.6	98.5	110.7	111.1	110.2
4.6	283.46	99	101	98.8	103.1	102.7	101.8
7.23	164.66	98.5	96.9	97.8	99.7	98.3	98.4
6.14	203.64	103.4	103.4	102	97.6	97	97.7
8.37	204.34	102.9	105.5	105.6	105.6	105	105.2
7.03	189.21	96.1	95.5	94.6	93.8	93.2	92.4
8.59	184.19	101	102.5	99.8	103.5	105.3	104.6
4.77	332.78	100.8	103.2	101	104.1	103.3	102.1
5.95	194.55	100.9	101	100.2	100.2	97.3	99.2
7.3	139.4	100.3	98.2	100.8	97.4	95.5	98.2
7.24	289.5	92.9	93.2	93.7	107.9	107.1	107.5
6.2	144.85	100.6	100	102.7	105.3	103.7	105.1
5	134.53	99.6	99.7	99.2	100.4	100	102.3
5.88	240.25	97.1	99.5	97.7	97.6	96	97
5.05	265.5	97.9	98.1	98.4	99.8	98.2	98.1
5.44	132.55	102.4	100.1	102.2	100.5	96.9	100.8
5.2	170.67	100.4	100.5	98.4	104.8	103.3	104.4
6.06	135.17	102.4	102.7	102.7	106.1	105.1	106.3
7.03	179.5	97.8	98.4	97.1	97.6	95.3	94.5
5.6	158.18	102.3	100.8	99.6	94.7	92.8	94.7
7.11	149.48	99.3	99.2	96.7	96.6	96	96.2
4.41	145.36	134.3	132.3	136.1	91.7	92.4	90.5
9.13	195.67	101.8	99.1	99.6	102.2	103.2	102.4
7.12	200.65	100.8	103.5	103.2	97.9	96.5	97.6
5.39	160.1	99.7	96.4	97.4	101.7	101.4	102.2
4.91	155.01	99.2	96.6	98.5	95.9	97.9	97.5
7.18	181.32	100.4	99	97.5	105.7	106.3	105.6

4.68	407.93	99.8	94.5	94	92.7	94.9	91
5.22	140.45	99.4	98	97.4	101.3	100.4	102.6
4.69	413.4	105.4	108.8	107	98.5	98.2	97.6
4.79	396.09	99.8	101.2	98.8	101.2	101	99.3
7.05	172.43	94.9	96.8	96.2	98.9	98	98.5
5.4	167.21	90.9	91.3	89.4	104.4	104.5	104.7
6.95	131.07	101.3	100	99.9	99.5	99.7	99.4
8.88	133.11	103.2	103.4	101.9	110.7	111	111.7
6.04	128.86	99.8	98.1	96.5	99.4	98.6	97.8
6.67	188.83	100.5	99.7	99.9	99.9	100	99.9
6.98	164.28	100.4	100.2	97.5	97.4	95.9	97
8.21	170.13	106.2	107.5	107.1	91.3	90.2	88.3
5.33	121.58	98.6	97.5	99	96.1	96	94
7.18	254.42	97.1	97.2	98.7	99.2	98.5	98.7
4.78	203.02	98	97.9	97.9	99.6	102.3	100.8
6.21	165.67	99.4	98.1	98.3	100.6	101.6	99.6
5.24	153.13	102.1	102.9	101.5	98.3	99.3	99.9
6.38	187.28	101.6	101.7	102.1	100.5	103.3	101.5
7.93	140.77	103.3	99.8	99.8	98	100.2	96.6
4.36	120.75	124.2	125.6	121.6	119.5	118.5	118.7
7.39	183.82	92.3	92.1	89.7	80.6	80.5	82.5
5.81	204.39	101.9	103.1	104.9	107.1	106.8	106.2
5.67	160.88	100.4	98.6	100.6	96.9	96.2	96.2
6.52	146.29	102.9	102.6	102.6	88.8	88.7	88.2
5.48	134.9	113.8	114.9	117.4	92.1	91.2	89.8
8.25	262.51	101	102	101.3	94.4	97.2	95.5
8.68	258.17	98.7	99	98.3	96.5	95.3	97.6
4.98	180.61	97.3	98.4	98.5	102.8	103.6	102.3
9.26	187.6	100.5	100.4	103.8	102.2	106.4	103.3
8.76	158.93	102.3	102.5	103.2	106.2	109.4	106.4
4.63	187.39	87.3	89.8	88.1	125.5	125.6	124.4
7.33	285.96	124	80.2	102.7	94.5	87.3	102.2
8.85	185.18	101.9	101.8	104.5	109.3	107.1	107.2
8.18	150.81	103.3	105.1	104.9	103	100.6	101.7
6.25	131.65	97.5	95.9	94.9	105.8	105.3	106.9
5.94	170.09	99	103	117.4	108.1	78.4	103.4
6.67	229.56	98.7	99.3	99.9	102.5	101.7	100.2
6.37	254.11	100.6	102.5	102.3	104.7	101.3	102.5
5.11	154.75	99.8	99.3	100	99	96.1	97.1
5.16	154.89	105.7	108.2	107.9	111.1	108.5	108
7.69	194.83	98.6	101.8	99.9	106	102.7	103.9
4.68	119.84	98.3	94.4	96.7	107.6	107.9	107.4
6.71	154.28	99.8	100.5	101.9	94.6	94.7	93
6.6	131.08	95.4	93.5	92.5	93.1	92.3	92.9
5.59	165.91	97.8	98.7	98.3	86.8	86	86.3
7.08	177.28	98.4	98	99.9	98	100.7	98
6.28	159.93	113.4	112.9	117.1	105	104.7	106
5.1	123.29	104.4	106.5	106.3	126.2	127.2	123.9
4.78	252.57	100.7	100.5	100	109.8	109.5	108.3
7.59	127.1	101	97.9	98.1	100.5	101.5	100.6
9.16	158.76	68.8	104.9	97.8	85.7	93.2	183.9

10.61	214.53	99	99.9	99.6	104.8	104.2	105.3
4.41	175.92	104.3	104.5	103.3	97.7	95.4	95.6
4.78	232.13	99.8	102.4	100.4	104.6	104.2	102
7.01	153.51	92	92.6	90.6	94.6	94.9	96.7
11.06	212.79	99.2	100	101.4	104	102.8	103.4
5.94	122.38	90.7	89.2	91.4	108.9	110.1	112.1
6.46	115.06	103.1	99.1	98.2	105.6	106.6	107.5
4.78	296.44	102.6	105	103.2	103.4	98.5	100.3
7.25	148.38	96.2	96.3	94.8	108.7	107.8	109.1
4.63	352.18	158.2	156	146.1	58.2	54.3	60.8
6.84	152.81	102.8	99.2	99.1	96.5	96.1	96.3
6.71	133.08	97.9	101.1	99.3	103.4	100.7	100.9
5.59	153.03	105.8	105.1	106.5	107.9	104.9	105.8
8.13	290.96	97.8	98.4	97.6	98.5	100	100.2
5.96	144.16	98.8	97.7	97.4	97.8	97.1	99.4
6.84	144.67	93	91.5	91.3	100.9	99.8	100.5
5.97	228.12	102.6	102.8	102.4	97.2	97.4	96.4
5.14	153.7	98.6	97.5	98.4	100.8	100.6	101.6
6.7	119.72	97.4	98.5	99.6	102.5	104.1	104
6.61	129.01	100.5	97.9	101.2	103.2	100.9	103.1
6.38	156.41	97.1	98.8	98.1	104.7	104.6	104.8
7.11	118.36	100.5	99.5	99.7	111.3	110.3	111.4
6.16	119.91	100.3	98.8	97.9	100.6	99.6	101.1
5.57	164.29	99.8	96.7	99.8	99.6	102.8	101.3
4.88	272.88	94.8	98.3	96.3	101.2	100.6	101.8
6.92	124.83	104.8	103.5	101.6	101.5	101.1	103.5
6.05	189.8	97.6	100.7	100.2	102.4	101.4	101.1
9.26	148.2	102.1	103.1	103.6	104.9	105.3	104
6.95	128.8	95.2	93.9	92.4	97.9	94.4	95.3
5.26	126.17	101.3	100.8	101.3	103.6	102.3	103.9
7.55	152.96	98.2	101.1	100.2	90	88.4	89.7
7.23	140.25	97.1	97.4	97.6	104.8	103.9	103.3
7.55	142.11	104.4	101.6	101.3	100.1	99.7	100.2
8.95	139.18	104.4	105.6	104.7	108.3	107.4	104.7
5.71	175.28	100.2	103.3	100.7	97.5	94.2	95.6
6.93	116.76	104.1	104.8	104.5	101.4	102.5	104
6.15	120.23	96.9	99.3	98.8	108.1	107.6	107.2
6.6	155.98	97.1	98.3	97.6	98.4	98.1	99.9
8.72	172.46	104.5	107.1	106.3	111	110.3	107.8
8.75	116.01	105	105.5	108.1	98.9	98.1	97.6
9.66	253.97	102.4	100.9	101.5	105.2	103.8	104.3
10.18	160.08	103.4	101.9	99.7	103.9	105.6	101
8.27	328.51	104.2	107.7	105.7	99.5	98.7	96
8.57	246.09	92.3	95.8	93.8	77.2	79.7	77.1
7.61	131.97	106.4	100.4	100.8	102.3	107.7	102.7
8.13	147.84	103.8	103.7	103.9	106.4	105.6	104.2
8.15	158.89	90.7	91.2	91.2	97.7	96.5	97.4
6.7	147.84	101.6	100.3	99.2	92.7	91.7	91.4
4.86	87.82	96.9	98.4	99.2	109.9	114.1	115.6
5	118.7	97.6	99.5	98.6	105.2	105.3	104.9
5.07	114.87	99.2	102.1	98.3	108.4	104.2	124.5

7.05	130.65	96.7	98.3	97.1	99.5	96.7	98.9
5.49	125.13	98.3	98.7	98.4	104.8	106.4	105.9
9.31	123.81	91.5	91.8	90.9	96.9	95.4	93.6
5.68	105.61	104.9	106.4	105.6	95.9	93.3	92.9
8.65	153.15	99.6	102.2	103.9	108.2	110.9	107
7.77	149.56	86.1	88.5	88	94.5	94.8	93.9
7.14	129.08	100.4	99.9	101.4	104.3	105.9	105.8
7.09	145.97	101.1	99.8	99.4	105.3	104.4	103.5
9.13	185.14	102.8	102.8	102.7	102.8	103	101.6
5.1	136.19	96.2	96.9	96.5	88.9	88	88.4
7.55	143.71	119.4	126.4	131.3	69.5	78.3	79.1
5.31	117.76	101.7	99.6	99.2	104.6	105.6	103.1
4.82	284.93	98.5	100.5	100	91.9	91.7	89.8
8.56	107.65	101.1	103.4	101.6	98.1	98	96.2
9.31	105.67	101.6	103.7	103.5	110.5	107.7	107.7
7.17	119.67	92.1	92.2	91.4	96.3	95.7	93.9
7.12	124.8	100.7	100.7	99.8	96.7	96.5	95.6
5.83	106.5	100.8	101.1	101.2	97.3	97.3	100.4
5.8	101.8	96.1	96.5	98.6	95.9	96.1	96.4
6.76	131.05	91.4	91.4	90.4	93.5	92.6	91.5
5.36	114.22	101.4	101.8	102.5	99.9	96.4	100.8
5.62	123.45	95.8	98.3	96.5	104	104.1	104.2
6.71	134.4	98.7	100.3	102.4	95.9	94.3	94.9
5.08	187.44	103.2	103.1	103.2	94.3	96.1	96.4
6.34	106.66	99.1	98.4	97.7	105	107.1	106.9
7.12	120.46	101.5	102.7	101	107.2	106.8	106.3
6.33	149.03	88.7	91	90.4	104.5	105.3	104.2
5.77	129.14	104.4	104	102.9	108	110.1	107
9.41	269.76	102.1	105.9	102.2	97	96.3	96.5
4.82	167.98	96.3	96.6	95.5	99	97.5	98.1
6.4	263.27	97.1	99.3	97.7	90.9	90.5	89.7
5.77	119.71	98.7	97.1	99.5	108.8	107.5	108.2
4.89	192.3	100.2	96.9	96.9	97.1	99	97.6
5.88	135.56	98.1	98.5	97.3	104.9	103	102.4
4.82	118.32	99.7	96.1	97.2	104.3	102.8	106
9.04	151.21	102.6	100	100.4	96.5	97	95.1
5.64	258.92	96.6	99.2	99.8	93.3	93.8	92.8
9.44	152.74	105.6	105.6	105.5	109.3	112	110.8
5.54	168.73	103.1	104.2	101.8	106.4	106.9	106.7
9.41	120.1	97.6	97.4	97.9	106.4	105.3	104.4
7.09	140.42	110.6	110.9	110.7	80.3	80.5	81.8
6.76	110.99	121.2	102.1	99.4	94.2	105.1	91.5
9.28	116.01	98	98.4	99	116.3	117.8	118.5
6.38	192.64	101.7	105	102.3	88	87.1	87.6
4.64	272.11	100.3	102.7	109.9	82	86.2	81.9
6.48	129.14	99.7	97.8	97.6	105.3	99.7	103.1
6.48	111.63	101.1	101.5	98.9	100.7	101.5	100.9
8.95	184.64	102.5	102.6	103.4	108.9	108.5	107
5.06	139.17	93.6	93.4	93.7	108	107.4	106.4
8.76	122.64	105.7	103.5	102.1	95.6	94.2	94.5
8.09	109.38	100.8	100.9	99.8	96.6	95.4	97.9

5.01	106.06	99	98.6	99.7	99.2	98.6	99.1
5	115.79	103.8	102	104.3	105.3	106.3	106.7
6.23	96.99	103	97.4	99.3	103	103.1	103.7
7.15	100.94	100	99.3	99.3	100.5	99	99.2
5.69	137.06	98.4	100	97.5	98.9	99.1	100
8.78	91.56	103.7	99.7	102.1	100.3	97.6	104.4
7.65	141.73	97.5	99.5	98.1	100.2	99.2	99.3
6.04	109.1	104.4	103.1	105.1	105.3	107.2	108.3
5.83	138.83	99.7	100.3	100.4	82.9	83.3	82.7
7.09	104.21	95.4	92.8	91.8	101.4	101.2	102.1
8.84	110.09	99.1	102.1	99.5	96.6	93.1	93.4
6.06	88.24	98.3	98.1	99.6	100.1	104.7	101.1
7.8	125.06	99.4	100.3	100.9	97.8	98.5	100
6.98	199.16	92.8	94.4	92.1	91.6	90.8	91.5
6.86	109.32	93.6	94.2	94.8	98.7	97.7	96.7
6.25	148.05	97.8	97.5	95.9	95.9	95.8	95.2
8.97	138.04	101.8	100.8	101.5	110.5	109.9	108.8
8.48	172.32	98.6	99.1	101	97.8	98.5	98.3
6.32	88.68	100.8	101.2	102.1	101	97.8	100.8
8.27	345.28	102	102.9	102.3	94.2	93.6	93.1
7.81	277.83	104.5	104.3	106.5	92.5	95	93.8
9.13	169.77	106.9	106.3	106.8	107.5	110.3	105.1
5.78	102.53	103.4	102.6	103.6	109.8	111.9	108.8
6.81	148.63	97.9	98.8	98.1	102	100	101.6
8.48	98.54	97.8	98.8	97.2	104.2	103.6	104.4
5.73	88.61	96.6	101.2	95.8	98.1	100.2	100.9
7.37	135.16	97.3	101	100.3	117.8	116.2	117.7
6.27	88.45	100.6	97.3	101.8	95.4	102.1	99
7.08	87.66	98.7	101.4	98.5	97.4	94.8	96.8
5.3	95.79	99.1	97.8	99.6	92.8	95.3	95.5
4.77	149.42	93.5	93.3	92.1	106.9	105.5	104.2
6.55	108.07	99.5	97	97.6	100.3	103.8	101.8
7.25	105.42	100.8	103.5	99.4	98.7	96.7	96
5.17	179.15	99.8	99.9	100.8	96.8	97.9	96.8
6.34	134.74	98.7	102.9	99.3	96.1	95.8	96.1
6.55	157.28	98.2	98.5	99.1	107.9	107.2	108.4
5.43	104.95	97.8	98.3	98.7	113.7	112.2	111.4
6.46	115.28	100.5	99.4	98.9	95.2	96.4	94.8
7.81	112.3	99.6	100.5	101.3	99.9	98.7	98.9
7.03	149.63	114	118.2	115.6	78.2	78.5	79.1
4.92	198.62	103.8	96.2	103.3	89	87.8	87.4
5.76	92.38	100.1	97.9	97.7	101.5	101.3	101.1
5.4	104.82	99.9	99.2	97.9	100.1	102.8	100.3
5.64	125.21	99.4	99.3	99.1	105.4	106.1	105
5.59	158.3	95.7	103.7	110	109	105.2	116.3
6.05	93.56	97	96.8	97	95.2	91.9	93.3
6.05	87.93	100	101.8	100.5	98.4	97.4	99.4
5.66	113.01	98.2	98.7	95.3	99.7	96.4	99.1
8.22	121.34	103.7	99.6	101.9	108	109.1	108
7.3	105.82	104.8	97.6	99.1	108.1	114.3	109.3
5.12	108.51	96.9	98.5	99.5	101.1	100	100.6

6.21	95.54	104.5	103.3	101.3	97.3	99.3	99.4
9.16	129.09	105.3	106.4	105.9	105.9	105	103.1
5.64	103.2	97.9	99.8	99	108.6	107.6	107.6
6.42	92.81	100.9	101	100.4	107	105.4	106
4.84	143.45	110	111.5	110.4	89.8	89.4	90.2
6.34	122.06	100.3	101.8	108.4	107.1	104.6	107.7
8.34	110.36	102.7	103.5	103.2	92.3	93.7	94
5.67	110.91	102.9	104.2	102.7	110.2	108.6	112.2
8.95	91.13	100.6	97.9	99.6	102.1	102.2	100.9
4.93	95.93	99.2	101	101.8	92.3	93.1	94.6
8.63	123.45	98.1	97.3	97.3	99.3	100.4	101
8.13	93.95	99	96.8	97.7	100.7	98.3	98.8
5.48	150.2	95.7	109.1	106.4	94.4	95	96.6
8.54	153.77	103	100.4	99.1	103.6	105.6	103.9
8.9	205.45	81.8	73.4	95.4	87	88.3	100.6
6.73	113.98	92.5	90.7	91.8	96.8	96.6	98.5
4.89	132.36	98	96.1	97.1	97.8	96.7	100.5
5.48	282.29	92.9	93.7	93.6	89.3	91	90.9
6.8	108.64	98.9	99.5	98.9	76.8	76.2	76.7
6.14	99.22	96.8	98.5	96.5	99.3	98.2	99
4.94	95.28	107.5	107	105.7	66.9	66.1	67.1
5.43	116.88	97	96.2	98.4	108.3	103.6	105.1
6.19	138.11	105.4	104	104.1	99.4	99.4	97.6
7.85	130.96	97.1	96.1	96.4	91.3	93.1	92.1
5.44	86.93	102.5	102.6	105	107.7	102.7	104.8
6.46	119.51	103.4	102.3	103.7	78.5	81.3	80.5
9.57	123.78	102	107.3	103.2	104.7	102.7	103.2
5.86	116.37	94.8	96.8	94.8	103.7	102.3	103.1
6.87	88.7	101	95.9	95.8	102.3	103.7	101.8
5.62	87.14	116.4	116.1	119.8	68.2	68.6	72.3
6.23	103.19	95.8	97.1	96	93.7	92.6	91.9
5.62	89.55	99.1	101.8	101.8	97.1	97.2	96.9
5.03	105.79	100.9	99.2	99.3	95.7	95.7	100.1
7.68	89.55	95.9	98.6	97.8	97.1	97.9	96.6
6.68	94.27	95.6	95.3	96.5	101.7	98.9	101.2
8.44	96.99	98.8	100.1	97.4	105.2	103.8	101.7
5.24	138.91	103.8	104.8	103	100.4	98.3	96.3
5.92	108.74	92.7	92.7	94.5	102.7	100.5	102.5
10.24	196.91	100.9	102.4	103	103.6	101.6	103
6.68	106.07	98.7	95.9	95.4	110	109.2	107.8
7.37	97.82	99.7	97.6	100.5	96.2	95.6	97.9
4.83	162.9	97.6	97.7	96.1	95.8	98.7	98.9
5	105.01	94.8	96	95.5	99.3	99.1	100.2
5.21	109.85	103	102.8	101.9	96	92.1	94.6
8.94	109.59	100.5	101.5	101.5	90.3	89.8	89.7
7.03	99.96	97	97.2	97.1	105	102.4	102.3
9.73	161.44	102.9	103.8	103	103.9	100.2	101.8
6.04	87.02	97.4	98.6	97.6	102	97.9	98.8
5.25	114.05	96.1	95.8	94.2	106.5	102.5	103.6
7.27	83.77	106.6	100.6	103.2	98.9	104.9	100.6
4.65	79.01	105.7	104.6	105.8	111.2	109.4	109.7

8.34	91.41	98.3	99.4	100.2	103.4	101	103.1
5.97	97.05	70.4	71.5	69.4	89.5	85.2	86.2
6.79	167.92	99.6	102.7	100.7	98.1	97.5	97.3
9.13	71.61	100.2	100.1	100.4	115.4	114.6	114.1
6.46	89.49	94.6	93.1	93.6	106.7	102.7	107.5
5.8	107.91	97.1	97.2	95.8	109	107.7	106.6
6.77	163.02	101.9	104.7	103.1	94.6	97.4	96.8
9.2	94.21	97.8	94.3	97.8	106.3	104.7	103.3
5.5	513.6	101.5	102.7	102.4	96.3	97.2	95.3
6.35	115.11	101.4	101.6	102.8	103.1	101.2	102.1
7.66	100.16	95	96.1	94.3	93.8	91.6	91.5
5.31	89.48	95.7	92.8	94.4	97.8	98.9	94.8
4.98	85.13	95.5	94.4	95.1	101.9	100.8	101.7
9.26	80.41	100.4	99	99	109.7	110.9	110.9
7.44	90.06	104.5	99.6	99.5	97.6	98.5	98
5.26	85.97	103.3	100.9	102.7	106.6	105.5	106.8
5.05	85.35	95.9	90.6	92.1	106.2	105.4	103.7
4.84	200.56	101	103.1	101.6	92.3	92.4	92.8
6.79	129.41	97.7	100	100.2	108	108	104.8
5.97	168.36	101.1	103.4	102.1	102.5	102.1	101.1
6.04	76.56	95.7	95.2	98.4	86	85.1	88.4
5	70.07	100	101.3	100	102.8	99.8	100.7
9.01	98.78	96.6	97	96.9	103.5	105.2	103.5
10.65	149.12	101.1	104.5	101.4	104.7	104.5	103.8
6.86	76.98	96.5	100.1	100.1	97.6	96.2	97.2
4.93	96.03	104.9	106	105.1	102.5	103.9	103.4
5.97	89.51	94.9	93.8	94.8	99.1	99.7	98.5
6.55	75.56	92.5	96.1	95.4	106.6	111	109.5
9.41	101	117.8	84	100.9	114	106	130.2
5.22	76.35	105.3	103.1	101.7	104.6	105.9	108.5
9.01	109.01	103.6	103	105.2	106.3	105	107.4
5.03	97.3	97.7	95.9	98.2	96.2	93.8	92.6
6.43	167.2	99	100.4	98.5	98.5	97.1	97.9
7.53	137.57	98.3	96.5	99.6	96.8	98.3	96.9
4.45	169.48	99.8	104.6	100.3	105.1	102.3	102.5
7.78	89.62	101.4	104.3	101.2	97.4	96.8	96.8
6.7	129.38	97.2	95.1	97.2	107.2	110.1	107.2
10.15	120.45	102.1	101.9	101.7	104.3	101.7	101.1
8.03	142.5	92.1	95	92.1	95.7	95.6	95.5
6.27	73.26	102	97.1	96.7	97.2	94.7	96
8.27	108.96	108.1	109.7	108.6	50.5	48.7	51.2
5.59	625.09	132.8	87.3	115.2	80.4	84.9	70
5.86	76.51	102.2	99.1	98.3	100.4	100	102.5
9.26	85.74	99.2	100.1	98.1	99.4	100.3	102.3
5.35	106.06	102.6	104.1	100.4	110	106.9	106.7
8.38	96.21	102.1	100.2	99.3	97.3	97.1	102.5
6.21	134.71	99.6	102.2	101.4	99.4	99.2	100.1
6.37	76.95	96.4	96.7	98.2	102	99.9	101.4
6	63.46	104.9	102.3	108.3	107.7	109.4	111.3
5.36	89.75	95.6	96.7	94.7	103.4	99.1	100.2
4.87	129.78	100.6	100.9	101.7	103.5	103.6	104.7

7.42	92.66	99.5	96.8	98.6	100.8	101.3	103.8
6.05	128.02	93.2	94.4	93.1	95.4	93.7	95.3
7.59	118.84	97.8	99.1	97.8	102.8	99.5	101.8
7.06	72.92	98.8	97.4	100.4	99.5	98.3	100.3
6.9	91.64	114	113.7	111.8	75.3	75.5	78.2
8.75	84.36	99.3	102.7	103	98.7	97.4	99.6
6.6	142.19	94.9	98.1	96.6	106	103.4	103.8
6.02	91.39	108.4	105.6	110	92.5	92.6	93.2
5.12	82.61	103.2	102.5	101.6	92	93.3	93.7
8.88	76.2	99.2	98.3	96.7	100.1	99.5	100.4
9.7	135.95	108.1	112.7	110.6	111.5	108.5	105.5
5.73	93.6	99.9	100.4	98.9	100.6	99.6	100.2
9.69	141.37	100.9	101.4	99.7	102.3	101.2	100.5
6.84	80.71	99	99	99.4	96.9	98.5	97.9
5.6	203.77	90	100.4	90	78.3	89.8	99.1
8.69	86.75	103.5	102.2	103.6	100.1	98.7	97.3
6.25	77.81	117.5	122.7	122.9	78.6	78.9	77.8
7.65	79.93	100.8	100	98.9	101.8	101.4	104.5
7.99	88.29	98.4	99	99.8	100.3	99.4	100.7
6.3	89.12	102.5	101.6	106	102.8	106	104.9
6.61	106.36	104.1	101.2	103.3	100.8	101.5	101.3
6.77	75.47	99.7	98.9	99.4	105.2	103.7	103
5.49	85.01	97.3	97.5	96.4	100.8	99.4	100.2
8.41	121.5	98.2	97.3	98.1	94.6	94.9	94.3
7.36	140.59	97.1	98.5	97.3	93.7	95.2	94
7.9	80.36	97.6	94.7	96.1	100.3	100.5	99.2
5.87	83.41	91.9	93.1	90.7	90.7	91.4	89.1
5.97	155.44	98.4	100.4	99.7	96	95.4	94.9
6.62	79.74	100.6	98.2	99.3	98.8	97.7	97.7
5.73	85.09	101.3	99	102	105	107.4	105.4
6.21	97.98	101.3	100.4	99.8	111.8	111.2	111.4
5.19	93.19	99.7	102.1	102.6	95.3	98	99.9
5.58	79.06	98.8	97.7	97.4	100.9	99.8	100.2
8.4	106.43	98.6	98.7	99.7	102.7	100.9	103.2
5.83	86.31	103.1	95.4	95.7	98.1	100.4	99.9
6.68	81.44	96.7	95.6	93.7	106.9	106	106.1
5.38	88.48	101	99.8	101.8	111	108.2	108.2
5.74	85.27	96.4	99	97.5	97.2	99.5	103
8.38	67.62	97.7	97.7	97.8	102.5	101.8	106.4
6.37	67.22	95.5	99.3	100.1	96.9	95.8	97.3
6.81	71.66	116.9	112.8	113.6	95.2	96.5	98.5
6.05	87.33	98.6	96.8	97.6	103.9	105.6	105.7
6.8	67.83	99.1	99.5	99.7	112.3	108.9	115.9
9.94	120.43	100.7	102.5	102.3	104	103	102.1
7.9	108.97	104.1	104.1	102.4	95.4	96	94.8
6.74	144.26	101.3	103.3	99.9	94.1	92	93.7
10.84	103.44	102.1	101.1	101.5	102.4	102.8	103.4
6.39	79.66	101	99.7	100	107.7	107.1	109.4
11.03	172.28	112.3	114.2	113.9	100.8	100	99.3
5.25	84.61	99.4	99	100.8	107.3	107.4	107.6
5.69	86.52	100.5	97.4	101.1	102	104.6	105.9

7.05	112.57	93.5	90.3	93.6	103.4	101.6	102.5
10.93	172.8	108.3	112.6	105.5	137.3	133.2	126.3
5.54	96.69	99.9	100.4	100	100.4	100.7	100.9
7.05	75.89	101.6	104.1	106.8	95.9	93.2	95.5
6.68	104.94	103.4	100.9	99.6	100.6	99.8	99.4
7.36	97.83	98.9	99.7	97.9	96.5	95.2	94.8
6.14	70.6	103.5	100.9	101.8	96.4	95.3	94.5
4.53	67.59	95.5	95	95.6	106.9	107.5	107.3
7.5	115.35	98.3	100.5	103.8	97.3	104.1	99
10.32	165.35	104.8	106.1	101.6	102.4	99.7	99.6
5.95	84.56	100.3	98.2	100.7	103.9	99.6	101.6
6.92	80.19	99.9	100.2	99.8	99.8	100.1	99.8
4.67	154.35	100.7	100.9	98.6	103.7	103.4	100.8
6.24	75.83	97.4	96.2	99.4	96.2	94.1	95.2
5.25	62.81	100.4	96.9	99.6	104.2	101.3	107.2
8.65	71.06	102.9	101.7	102.1	103.8	102.2	103.1
9.29	82.37	105.3	101.5	103.1	103.5	103	104.7
6.51	61	101.9	96.2	114.3	94.6	102.1	102.7
7.74	100.58	120.2	120.9	116	84.6	79.3	81.6
7.49	135.05	99.4	100.6	97.9	92.6	93.7	92.7
6.87	167.37	101.4	102.5	101.9	91.4	92.2	92.9
6.71	62.19	100.6	100.1	98.2	103.4	102.3	102.5
8.76	76.96	98.6	105.1	104.2	83.9	82.9	84.7
6.29	92.39	102.8	102.4	99.2	94.2	94.7	95.6
9.54	90.86	96.8	94.4	96.7	101.9	100.5	99.9
4.68	58.06	98.9	100.9	101.7	94.1	94.1	94.7
10.08	139.23	104.4	103.7	104.2	109.4	108.2	109.5
6.95	74.15	99.8	101.3	99.8	97.7	98.5	97
6.13	82.78	99.6	100.1	102.6	103.7	108.2	105.6
5.39	70.46	100.7	98.5	98.3	96	98.7	100.7
5.27	92.23	95.9	100	96	104.9	108.3	103.9
5.03	81.66	87	84.4	102	107.4	104.8	152.1
6.87	68.43	104.3	97.3	101.3	108.1	109.9	107.1
5.83	76.04	102.6	100.7	101.7	97.1	98.6	96.3
7.37	74.95	97.6	95.4	99.9	110.9	111.7	110.9
5.14	102.34	99.2	97.1	98.9	100.8	104.2	101.7
6.04	69.3	114.2	111.7	113.2	85.8	83.3	84.1
8.38	77.46	100.9	100.3	99.2	106.9	104.1	106.3
8.35	77.59	98	97	96.7	104.2	104.3	102.7
5.69	66.76	104.9	103.3	101.4	108.1	108.9	111.2
4.84	136.77	101.9	102.7	102.6	101.6	99.7	100.9
6.76	85.06	102	100.7	102.5	97	98.7	97.4
8.92	62.22	101.8	100	102	109.1	108.1	106.4
6.29	138.92	102.6	104.3	102.8	96.8	97.8	97
7.31	73	98.3	99.5	97.5	104.8	101.5	103.9
4.3	64.46	105.5	101.5	103.5	119.4	119.9	121
7.97	106.56	104.7	104.5	103.9	93.1	90.5	91.6
8.78	71.43	99.8	99.4	101.4	106.1	106.8	107.5
7.09	61.12	99.3	103.5	100	106.9	107.4	109.2
9.17	96.1	99.9	97.6	96.8	104.5	103.3	102.9
7.37	77.96	99.7	101.8	99.9	97.5	97.9	97.5

8.13	64.28	96.3	96	95.6	99.6	96.4	97.4
8.92	62.46	99.9	99.7	101.9	97.2	94.2	95.7
5.73	113.6	97.5	98.5	97.9	103.1	104.5	100.7
8.03	67.31	101.2	100.4	100.9	94.7	98.8	96.7
5.96	70.95	96.7	95.2	93.7	103.7	101.3	105.5
8.12	62.82	103.1	99.7	100	113.5	111.9	109.5
6.19	124.3	101.8	106.1	98.2	102.6	103.6	94.4
9.74	123.72	99.5	96.8	99.4	103.3	100.6	104.7
8.78	73.08	105.8	105.9	101.2	96.5	96.1	98.5
11.36	290.23	111	112.3	113.1	106.8	110	107.4
8.46	79.06	101.6	97.4	99.1	101.9	101	100.8
5.38	66.09	101.1	99.7	100.4	96.5	95.2	100.2
8.5	77.54	100.4	101.1	100.7	101.1	102.4	101.9
9.64	318.71	107.4	105.4	106.1	94.5	95.8	95.9
8.05	100.19	106.7	103.9	104.2	94.3	94.7	94.7
6.29	98.65	74.3	78.9	72.2	118.8	117.5	117.2
6.04	77.48	98.1	99.4	96.2	107.5	108.5	107.5
5.22	83.08	102	103.6	104	111	111.3	109.3
4.36	312.51	98.1	101.5	97.2	98.6	99	98.2
6.02	63.47	100.7	99.9	102.6	98.5	104	101.1
7.77	82.21	96.5	94.2	94.7	97.5	95.4	97.3
5.97	63.54	106	102	104.4	98.4	97.4	96
6.21	122.24	98.5	98.3	98.7	100	99.1	101.6
10.32	102.77	100.9	100.7	101.1	105.4	105.4	103.2
7.88	114.79	97.2	95.6	90.8	98.3	95.2	94.4
9.04	66.21	95.3	97	95.2	111	110.5	109
5.67	59.97	100.7	98	101.1	93.3	92.1	93.8
7.49	57.98	102.6	102.4	103.8	103.2	101.7	102.3
6.32	84.36	110	107.8	105.3	96.9	94.8	95.9
9.11	74.69	93.5	93.5	96.7	91.9	91.6	95.6
8.25	81.9	100.1	99.4	100	108.4	109.4	105.4
10.43	125.76	97.4	109.2	103.9	107.3	110.2	109.5
6	95.14	102.1	102.3	100.7	100.5	100.9	102.4
7.88	81.6	105.6	103.6	106.1	102.5	102.5	99.6
6.25	78.06	99.9	101.5	99.2	94.8	94.3	93.4
4.54	119.13	102.2	102.6	100.5	102.2	101.8	102.4
7.93	72.42	100.6	109.4	110.2	95.5	94.7	94.6
6.02	86.01	95.4	92.6	90.7	92.5	93.9	95.2
4.79	103.98	99.3	100.7	99.7	100.6	98	96.6
5.21	77.9	100.9	100.7	102.8	97.8	98.3	101.3
6.73	74.75	105.3	107.5	111	114.4	113	113.9
5.66	64.12	105.3	101.8	105.4	96.2	96.1	98.6
11.02	168.58	98	105.6	106.8	136	140.9	148.2
6.16	67.28	100.8	100.5	100.1	94.5	91.5	95.5
6.67	125.2	97.6	97.2	97.8	109.1	107.3	105.6
6.43	68.57	102.2	98.9	104.3	92.3	94.2	92.9
9.76	106.53	95.2	94.6	92.9	85.5	85.4	84.3
6.18	64.17	95.3	98.2	99.7	91.3	89.4	91.3
8.5	109.94	99.6	97.9	97.8	97.4	97	96.9
5.33	86.89	102.6	104.7	102.6	98.2	95.4	94.6
8.41	90.29	97.2	97.7	98.9	99	102.2	102.1

10.58	139.25	97.4	99.7	100.1	109.7	111	111
5.92	113.98	100.7	100.7	98.9	104.3	103.5	104.5
4.84	79.73	106	101.5	102.4	99.9	103.9	102
7.17	74.89	97.5	98.7	95.8	97.6	96.7	97.9
5.41	82.28	100.5	94.5	91.3	116.7	111	114.9
7.56	126.78	99.3	98	97.7	103.4	103.4	102.1
7.21	114.97	100.5	104.6	103	104	101.6	101.8
5.26	78.74	99	100	97.1	100.2	96.1	97.6
5.68	76.74	101.2	99.4	98.7	93.7	92.6	94.2
6.96	71.22	98.7	98.7	100.2	97.9	100.2	106
9.42	137.79	102.7	100.1	102.9	104.7	101.4	105.2
6.13	71.14	97.3	98.4	99.3	93.2	92.1	92.3
7.2	71.25	97.2	96.4	94.6	100.5	99.4	98.6
5.67	74.54	105.7	104	104.8	125	120.9	122.6
9.28	64.19	95.1	97.8	96.4	99.7	101	99.8
8.85	71.62	98.8	98.6	99.1	102.4	99.8	100.3
7.93	78.42	98.5	97.4	95.1	90.2	93	92.6
5.55	53.31	97.8	95	95.8	99.5	98.7	99.7
8	127.73	115.6	118.1	126.5	88.6	80.4	86.9
6.89	61.75	99.8	102.3	97.1	95.9	96.3	97.5
6.73	61.69	101	102.4	102.2	95.9	97.2	94.8
5.96	64.84	107.3	101.1	101.1	112.9	110.6	113.7
7.09	59.01	99.9	96.8	99	100.7	98.8	101.4
5.9	65.43	98.1	101.5	103.2	98.2	102.3	100.5
7.06	69.13	95	96.6	95.3	103	104	103.7
10.05	92.38	104	104.6	101.2	102	102	101
6.05	56.14	106.7	106.7	111	92.7	93.7	95.6
5.2	61.89	102	101.7	100.5	105.4	104.1	105.3
5.49	70.33	96.1	96.4	97.5	100.4	101.7	100.6
5.22	69.7	92.4	95.4	95.6	102.2	100.5	104.5
5.2	81.59	99.6	97.6	100.1	103.4	100.6	104.2
5.43	75.02	97.8	99	97.8	99.1	101.4	102.2
6.87	67.5	102.5	98.8	101.2	100.3	101.9	99.2
7.23	102.09	96.9	97.2	96.8	92.8	92.4	92.4
8.53	73.51	102.9	101.3	103.3	104.5	103.8	101.7
5.81	89.04	100.5	99.2	100.6	99.8	99.8	101.1
6.7	53.43	101.2	99.8	107.6	99.2	101.6	102.3
6.61	59.82	101.3	97.7	96.4	101.5	101.8	103
6.65	72.26	97.6	98.6	99.3	97.6	99.4	101
5.85	77.06	93.2	93.6	93.5	101.2	101.1	99.9
6.33	99.22	102.4	99.7	102.9	97.5	97.8	97.9
8.25	79.89	95.6	100	99	96	95.9	97.1
5.47	63.04	98.6	98.8	97.1	96.9	95.8	94.9
8.7	85.45	102	101	99	90.1	91	91.7
5.14	103.07	96.8	94.9	95.5	107.1	103.2	106.9
5.16	71.38	97	95.7	94.1	101.9	104.7	103.7
6.38	75.42	99.2	97	96.5	98.8	100.2	100.4
5.54	62.73	98.4	98.5	99.3	97.5	97.5	103.1
5.57	89.59	97.2	97	95.7	101.1	104.5	103.8
5.47	71.83	103.9	103.2	105.6	115.1	109.8	112.2
5.24	53.39	99.2	103.9	102.6	102.2	101.3	101.2

7.46	88.04	104.8	101.4	101.8	107.9	109.5	109
6.19	93.85	98.2	102.2	96.2	95.9	94.8	95.3
8.44	81	99.3	98.5	97.9	101.2	101.5	99.8
5.47	64.39	98.7	101.4	98.5	77.3	79.2	78.5
11.25	110.03	100.1	101.2	99.6	107.4	106.1	103.8
7.14	97.83	94.4	96.6	95.2	101.9	102	100.3
6.18	80.6	100.1	100.2	98.4	100.8	101.5	101.8
5.62	58.29	100.6	96.3	98	98.5	102.3	102.6
5.76	99.85	103.9	102.4	100	108.7	107.8	106.9
5.2	74.09	96.2	99.7	95.2	105	103.9	101.7
5.06	53.13	95.9	99.9	100.2	99.1	102.2	100.5
5.36	114.11	96.9	99.9	99.8	100	98	100
5.05	71.84	129.9	129.8	126.1	73.8	69	69.8
5.11	112.49	99.2	103	100	101.2	101.3	101.1
7.01	96.62	95.9	94.2	100	112.3	112.8	110.8
6.04	69.07	102.9	103.2	104.3	84.3	83.9	86.2
9.44	79.55	100.2	102.6	99	107	107.6	106.3
6.74	43.28	102.3	98.4	94.7	95.7	97.6	98.6
8.21	70.19	106	105.3	102.3	102.5	103	103.1
4.94	67.37	103.2	106.6	106.2	107	104.4	102.8
8.38	57.79	98.9	97.2	95.2	106.5	106.1	108.3
5.07	63.94	100.4	103.2	100.8	100.9	101	97.9
10.32	217.53	111.2	111	113.2	106	111.3	105.9
10.45	111.77	101.6	103.7	102.1	106.3	104.1	103.3
5.62	61.08	99.7	99.2	95.9	98.9	96.1	95.9
6.95	77.17	106.7	106.9	105.7	109	108.5	107.9
7.47	64.62	102.6	100.9	103	104.7	106.1	106
5.16	76.86	128.1	132.1	128.7	78.7	77.3	81
7.53	64.55	98.7	97.1	97.8	97.9	98	97.6
5.48	87.31	98.5	98.2	101.1	101.8	97.3	104.3
8.87	71.78	103.8	94.7	100.9	112.4	114.4	114.4
8.69	95.8	111.4	110.1	111.4	95.3	96.9	95.5
7.61	89.12	102.8	104.8	101.7	111.9	109.9	110.2
5.52	68.33	94.7	97.4	96.6	94.4	92.6	95.4
10.92	146.65	113.2	113.7	113	99.8	99.4	98.2
10.08	98.44	99.7	102.3	99.1	103.6	105.3	105.1
5.25	74.35	100.3	99.9	99.8	107.8	106.7	107
7.3	61.74	98.6	98.9	99.6	96	95.1	95.1
6.29	61.09	103.4	98.6	96.7	101.8	104	100
8.46	80.22	101.1	103	103.6	99.5	99.1	99.4
5.08	99.56	86.5	86.7	86.6	104.6	106.2	104
6.54	89.77	99.3	96	95	88.9	90.6	90.2
9.38	93.35	94.7	93.9	95.8	102.3	100.3	104.4
6.73	82.87	102.7	101.2	106.2	113	103.2	113.7
6.1	93.54	102.9	103.4	102.6	98.1	98.4	96.8
8.9	68.64	103	97.2	98.9	98.1	99.1	98.6
5.17	66.75	99	101.4	103.5	95.4	94.8	92.2
5.83	69.95	105.2	104.6	104.5	106.7	104.1	100.3
5.48	131.84	85	84.3	82.5	131.4	128.9	127
5.1	64.21	101.9	98.1	97.5	104.3	103.3	103.4
10.13	72.4	97	97.7	97.5	114	114.9	113.8

6.37	57.54	99.2	97.6	97.6	96.7	93.1	93.9
5.08	161.93	103.1	101.7	102.1	90.2	89.5	90
7.31	111.23	101.8	102.4	101.9	99.2	99.5	97.2
7.21	66.51	99.6	99.4	98.4	96.8	96.1	95.8
6.05	82.08	102.5	98.4	100.4	100.3	100.2	101.8
4.4	56.07	98.8	97.3	96.9	110.6	109.3	109.7
5.54	74.28	108.7	96.6	103.3	103.7	103.9	100.6
6	80.75	105.4	94.9	104	98.9	103.6	102.8
5.27	212.53	74.3	66	68.7	91.1	85.5	95
6.54	85.96	106.7	96.9	100.3	107.9	96.1	95.2
6.68	54.66	102.2	102.3	99.2	107.2	106.2	102.6
8.43	66.35	99.7	100.3	99.4	101	100.4	100
5.15	56.39	105.4	102.9	99.8	95.8	97.6	93.9
6.89	49.15	104.1	101.2	102	103.1	105.4	108.6
6.37	73.77	102.6	101.4	102.4	106.3	105.2	106.2
5.67	69.32	99.1	99.7	98.7	96.6	98.7	97.7
4.78	58.61	100.7	97.7	105.1	87.3	93	88.2
5.66	54.35	106.5	103.8	102.5	111.3	110.9	109.6
5.1	66.3	102.9	98.5	97.8	105.9	103.7	105.6
7.37	65.36	94	92.4	95.3	103.5	106.6	106.6
8	55.5	105.9	101.8	103	105.5	104.3	105.1
5.69	48.41	97.2	96.5	97.8	95.5	95.8	97.1
6.2	60.59	104.6	102.6	102.6	100.6	101.6	98.9
5.54	61.67	103.7	99.7	101.4	95.3	98.2	96.7
9	92.58	100.3	104.3	102.3	108.6	104.9	107.1
7.37	67.19	107.8	106.4	107.2	96.1	98	97.5
4.67	51.87	97.8	97.2	99.6	111.4	107.3	113
5.33	62.33	105.5	104.2	107.8	115.5	113.7	112.2
7.77	56.46	98.1	99.5	99.7	106.6	104.9	106.5
6.65	63.72	98.8	98.5	100.8	98.9	99.5	99
10.32	161.83	108.3	113.7	110.2	107.2	106.7	103.3
5	73.5	97.1	96.7	99.3	101.3	99.6	100.3
4.79	73.68	100.8	102	100.4	101.3	100.8	100.9
7.12	77.37	98.7	98.8	97.6	102.6	101.2	102.5
6.16	73.03	100.3	100.8	99.3	98.9	100.2	101.2
10.15	57.65	101.5	103.9	104.5	120.8	122.7	122.6
5.62	61.42	97.9	98	96.7	105.2	105.3	109.6
6.57	67.36	107.4	102.4	102	92.3	96.9	106.6
11.03	96.25	101.4	101.7	101.1	107.9	101.9	102.6
5.35	67.3	97.1	93.6	96.9	101.1	101.5	102.5
6.86	81.22	96	97.1	95.8	102.9	110.5	105
8.5	64.9	102.4	102.8	103	96.3	97	98.5
6.92	50.23	102.8	97.8	100.8	98.3	100.5	98.2
6.9	60.19	103.7	96.6	97.6	97.4	95.6	97.6
6.23	52.25	93.6	96.4	94.1	97.2	96.2	94.5
4.68	53.23	99	99.1	105	103	103.1	105.9
8.54	60.52	102.2	99.6	99.5	100.8	100.9	101.3
5.54	57.59	94.9	91.5	93.8	104.2	101.1	105.8
7.87	43.84	100.1	101.2	97.2	102.3	98.3	99.5
4.84	55.43	99.1	100.7	101.3	99.8	105.5	103.9
10.15	99.5	101	101.9	102.1	105.4	103.3	105.8

9.07	71.29	103.2	100.9	102.2	67.6	66.1	67.4
6.4	54.15	102.8	103.1	100.7	104.9	105.6	104.4
10.05	89.43	104.1	102.5	105.5	118.2	116.8	117.5
9.83	84.37	101.8	102.2	103.3	106	105.6	103.3
5.29	75.94	99.1	98.5	103.1	106.8	106.9	104.2
7.21	55.32	94.9	97.7	95.9	95.3	97.2	98
5.78	69.74	101.1	101.1	102.3	95.2	94.3	97.2
7.61	63.5	99.3	99.5	98.3	107.6	107.1	105.6
6.15	66.8	114.2	110.7	114.9	72.8	76.2	77.3
10.51	123.15	100.3	99.2	98.6	106.2	107.3	106.6
6.84	77.56	102.4	102.6	99.8	98.1	97.2	97.7
5.25	57.14	93.3	93.9	91.6	103.2	104.6	103.7
9.09	67.16	100.1	98.3	98.6	108	106.6	108.5
9.57	76.79	102.5	105.3	106.2	106.4	109.6	106.6
6.18	70.39	97.6	96	94.4	103.8	102	102.3
8.1	51.56	100.1	99.7	99.6	97.2	96.2	96.2
6.52	57.24	99.3	98	99.2	105.4	103.9	106
9.19	62.51	101.4	103.2	102	103.4	105.5	107.4
5.01	131.34	101.6	101.3	99.8	104	102.2	100.7
9.38	83.35	101.3	102.2	103.9	104.1	105.7	106.6
6.95	71.84	100	98.1	100.8	102.6	101.5	102.1
5.49	121.03	97.3	100.8	98.3	97.1	97.5	95.9
5.25	63.33	101.5	105.4	102.3	89.4	94.8	92
5.6	115.12	95.8	96.8	95.5	96.4	94.5	96
5.48	53.46	101.7	98.5	96	100.8	99.3	98
6.05	75.38	96.7	97.4	99.6	95.2	92.2	97.8
9.03	74.26	98	99.9	99.7	99.1	98.2	95.8
4.73	55.53	104.5	102.2	102.2	108	111.6	109.4
5.17	51.11	98.8	100.6	98	100.8	98	100.5
5.52	65.41	99	98.4	100.5	108.4	110.2	112.8
7.62	50.42	95.7	92.2	92.5	92.6	90.2	93.2
8.32	84.5	98.6	101.2	99.5	96.1	93.3	95.5
6.7	70.23	64.6	62.9	61.2	81.9	82.2	82.3
5.63	65.82	99	94.4	94.9	110.3	111.1	108.2
6.79	63.01	102.3	99.8	99.7	101.1	101	99.9
8.07	67.59	105	97.9	99.8	98.9	104.8	100.2
5.08	67.19	100.4	97.9	97.5	102.4	103.8	105.4
9.36	90.32	101.5	101	102.7	108.7	111.2	109.3
10.3	97.03	99.7	104	99.2	103.2	104.5	102.2
5.39	62.57	103.1	100.8	98.7	100.9	101.8	98.3
8.95	72.78	102.4	102.8	103.9	104.3	104.3	100.1
6.4	45.82	99.8	100.5	100.4	110.5	105.9	107.5
8.57	95.54	99	102	109.5	84.6	93.7	95.6
5.31	60.89	97.6	97.1	98.1	102.4	99.1	100.1
4.69	69.57	105.5	105.8	106.2	105.3	105.7	105.9
7.18	53.73	108.6	104.4	110.7	102.6	102.9	106.3
8.21	47.33	103.7	102.8	99.9	100.2	100.3	100.5
7.34	58.72	105.9	102.5	106.2	103.7	102.1	104
8.07	72.46	94.6	99.3	94.7	98.1	97.4	96.7
5.55	43.9	114.3	107	110.8	90.7	91.3	90.3
6.15	53.59	108.3	99.3	107.8	91.3	90.6	82.8

6.06	52.44	104	97.6	98.3	99	97	99.9
7.5	49.61	108	108.3	106.2	94.4	92	97.5
8.44	58.69	102.5	97.1	99.2	83.4	84.9	88.8
5.48	55.17	103.6	105.8	104	100.1	97.3	96.5
8.06	86.38	93.9	103.8	99.9	99	110.2	99.1
6.38	62.29	93.1	94	93	106.6	108.5	106.2
5.66	61.02	96.2	94.4	96	108.9	108.5	108.2
7.8	55.45	95.8	96	92.5	114.4	109.6	112.7
7.91	48.36	103.4	102	101.5	91.8	89.7	93
8.15	40.8	95.3	94.5	93.2	107.1	105.3	108.9
9.96	83.37	101.4	101.8	102	99.5	97.2	99
9.79	58.82	103.6	103.4	110	99.9	106.9	106.7
5.63	63.68	93.5	95.3	92.3	88.4	83.8	87.2
5.43	66	98	100	102.4	102.9	101.2	100.3
6.74	65.02	103.4	103.8	100.9	91.8	88.7	88.7
7.72	66.6	100.8	102.9	99.6	97.8	97.5	95.1
6.04	52.3	102.5	105.3	99.9	140.2	123.4	135.4
9.32	57.55	99	98	99.5	120.5	122	119.7
6.39	51.53	98.8	96.6	97.4	101.8	104.3	105.8
6.61	50.46	105.1	106.8	107.4	93.3	95	96.6
6.54	68.88	101	100.5	97.6	103	101.3	102.7
8.72	51.14	99.7	95.5	93.7	94.7	91.4	96.8
5.77	56.52	104.1	106	108	110.5	116.3	114.2
4.81	48.26	101.5	97.2	101.4	104.8	105.5	104.3
5.87	63.45	121.7	121.9	121.5	85	84.7	85.4
4.96	52.61	96.6	92.9	94	107.1	102.2	106.4
6.32	50.46	106.7	107.1	107.1	98.3	94.1	95.6
8.48	58.44	101.6	96.8	97.4	102.7	103.9	102
8.87	50.21	100.9	100.1	103.6	98.9	103	102.4
7.18	41.49	104.2	105.5	104.5	95.1	92.7	92.8
5.39	44.58	111.5	96.3	98.6	91	96.6	95.1
10.54	105.87	104.5	103.6	103.4	101.7	102.9	102.1
6.05	56.15	98.3	100.2	99.1	104.2	103.8	104.1
6.55	64.2	105.6	103.7	105.6	86.9	89.5	90.6
8.51	84.46	95.3	102.1	99.1	99.7	98.7	99.8
8.28	38.92	97.1	101	101.5	100.8	101.6	97.6
7.58	49.12	92.2	90.9	90.7	103.7	106.1	109.4
5.38	52.46	96.5	96	98.7	99.3	105.7	102.9
6.73	56.01	108	104.8	105.4	95.7	96.5	102.3
5.97	75.74	102.3	105.6	104.6	111.6	116	112.7
5.43	54.15	123.2	98.1	107.7	91.4	90.4	92.8
8.95	53.19	105.2	101.8	100.7	104.3	100.7	104.5
5.68	55	97.4	101.3	102.3	106.5	107.6	107
9.23	43.36	102.3	101.5	109.5	109.2	104.9	110
6.68	51.91	101.4	101	98.8	108.9	109.7	107.9
8.32	89.96	103.4	102.2	100.9	102.8	100.3	101.5
6.19	49.91	108.1	101.1	102.1	106.5	104	103.7
6.7	61.84	97.5	96.3	99.1	109.4	104.1	105.4
4.83	48.02	95.5	98.3	96.2	102.9	105.2	103.1
8.75	47.63	102	102	99.1	102.4	100.6	99.2
7.03	53.21	100.7	99.1	99.9	95.1	92.7	93.9

6.34	53.14	131.2	123.5	126.5	92.2	95.5	92.5
8	63.14	103	95.8	97.9	88.6	89	90.2
5.12	50.06	95.6	97.5	100	94.5	95.5	98.3
5.52	42.41	106.5	103.6	104.3	111.8	114.5	114.4
4.75	62.93	82.4	81.2	82.4	106.6	110.7	105.5
7.94	59.11	99.6	102.5	100.4	108.4	106.7	105.8
6.48	70.09	106.9	103.2	102.1	102	100.6	99.6
10.18	60.06	101.5	103.9	104.6	109.4	110.2	110.4
9.28	41.73	106	101.2	105.6	95.3	95.5	98
7.78	65.88	96.7	99.3	98.3	103.5	100.8	107.4
8.56	67.64	86.4	85.6	84.7	72.6	72.9	71.6
7.01	93.74	99.4	98.9	100.3	104.4	104.9	104.9
5.29	59.43	104.3	106.2	104.4	108.9	108.8	108.4
5.92	60.44	100.4	100.1	98.3	97.3	96.1	97.8
4.98	57.24	96.4	94.2	95.8	101	103.9	105
7.77	58.07	113.2	113.8	109.2	91.9	92.2	93.9
6.92	100.61	100.5	100.4	101.6	92	92.9	91.9
8.65	40.21	106.1	103.6	108	94.8	93.1	94.5
10.62	86.3	96.6	101.8	100.3	118.7	117.3	116.8
5.3	64.02	94.7	97.6	96.6	100	98.5	99.2
8.57	46.55	111.5	103.9	106.3	96.9	98.4	97.6
8.13	67.42	99.9	98.8	100.6	104.7	103.9	103.5
6.74	46.38	89.8	88.2	90.4	105.8	104.6	101.4
11.47	81.83	98.7	101.5	97.7	105.6	104.4	103.1
7.96	58.02	97.7	94.3	97.1	106.9	108.1	105.6
6.8	44.76	103	94.8	101.1	103.6	101.9	102.5
5.3	54.87	103.4	100.9	103.5	108.5	109.3	105.9
6.3	51.57	95.1	97.9	100.5	93.8	96.6	98.7
6.92	50.05	100	100.4	97.4	98.5	98.7	97.2
8.59	50.08	97.8	100.3	97.7	116.1	113.3	113.9
6.67	45.86	102	99.8	103.9	108.1	103.8	105.9
6.47	42.74	124.9	125.8	125.1	62	60.7	59.7
7.18	54.43	95.8	103.2	102	104.2	100.8	106.1
6.47	48.25	151.3	148.5	142.5	92.7	114.5	121.7
6.57	59.55	102.1	102.9	99.7	100.2	100.2	98.7
8.13	67.54	100.1	98.1	100.4	102	98.8	100.4
8.06	66.4	97.5	96.7	97.1	102	105.2	104.2
5.64	70.56	99.6	98	98.3	106.6	102.9	106.9
5.55	41.48	102.3	101.4	99.9	103.1	101	101.5
11.65	116.54	98.9	99.8	97.3	107.2	105.8	105.9
7.11	45.27	101.4	102.1	103.1	103.9	98.9	103.8
8.37	43.11	108.2	99.9	103.6	115.6	108.6	118.6
8.09	57.1	102	102.7	101.3	100.3	100.7	102.4
8.66	53.97	102.4	101.5	103.9	107	105.7	106.8
6.67	51.52	105.2	107.8	103.4	89.6	92.9	95
5.44	53.7	98.8	98.6	96.3	103.6	101	100.5
8.32	75.44	98.1	100.7	100.2	96.4	97.2	100.1
5.36	42.1	96.1	98.6	99.6	95	96.2	102.1
11.62	64.6	98.9	96.7	96.6	105.8	109.7	106.5
6.33	55.57	101.8	103.2	103	100.9	100.3	96.7
6.64	40.08	95.1	96.4	95.7	102.8	103.4	102.6

8.28	49.73	99.1	96.9	99.2	103.6	101.4	99.1
6.3	52.49	102.8	94.2	100.7	99.8	97	100.9
7.06	42.83	100.7	98.5	96.9	102.9	100	105.3
8.32	49.47	101.6	100.6	101.4	95.9	101.2	98.7
5.58	64.86	113	103.8	102.1	104.7	111.9	103.1
5.67	91.53	88.6	91.5	87.8	97.3	99.9	96.9
5.41	47.12	106.1	101.9	109	111.6	111.1	114.7
5.81	50.22	99.5	96.3	96.4	96.2	95	100.7
5.64	50.24	98.7	100.1	100.9	114.6	116.8	116.1
7.74	40.3	95.8	98.7	96.8	93.3	97.6	97.3
7.43	82.83	100.8	99.1	99.5	99.8	97.7	99.2
6.77	42.66	107.3	97.6	101.9	104.1	107.2	105
6.16	73.21	103.8	102.2	105.3	96.3	93.9	96
9.28	62.38	97.3	100.5	98.9	104.6	101	101.9
8.87	51.39	97.4	102	100.4	108.9	106.3	110
6.52	40.96	96.3	98.5	103.7	106.2	105.2	107.6
7.42	49.11	96.2	95.2	99.5	95.2	91.1	95.5
6.37	63.84	106	101.1	101.7	87.6	80.5	84.6
9.03	44.65	107	106.1	104.4	110.7	112.3	112
8.09	46.84	102.6	99.6	101	109.5	108.1	111
9.98	51.39	98.7	98.9	99.7	111.8	112.7	112.3
8.35	76.31	97.6	97	97.9	95.4	93.6	94.7
8.48	76.95	88.3	89	86.7	98.2	97.9	98
8.65	73.17	105.9	102.2	98.7	91.9	91.6	91.8
6.39	50.52	124.2	115.1	123.2	82.6	84.1	83.4
7.2	45	93.9	95.3	99.3	106.4	110	108.4
8.87	62.67	98.6	101.6	100.7	112.6	111.3	113.8
9.67	43.1	104.9	103.8	101.7	97.9	99.9	98.4
5.77	56.7	107.9	108.3	108.5	96.2	93.9	98.5
6.76	68.23	97.6	98.1	99.1	101.1	101.2	97.1
6.42	51.66	94.7	94.6	96	105.3	104.6	104.3
7.15	54.14	103.8	94.2	101.5	97.5	96.5	100.7
6.64	82.01	99.9	101.1	101.4	98.6	100.4	97.2
7.39	55.27	102.6	103.2	102	102.5	104	107.9
8.25	45.53	102.4	100.8	102.7	98.4	99.3	101.4
6.57	39.74	93.6	92.2	94.1	89.1	92.2	89.8
6.68	51.32	104.7	104.8	103.2	105.9	111.3	105.1
7.01	50.44	97.5	97.9	99	101.9	109.1	106
5.11	57.56	102.8	103.2	99.9	101.6	99.8	104.2
5.17	46.21	99.9	102.2	102.3	97.8	98	98.3
6.32	42.65	97.9	100.2	91.9	113	109.7	113.7
8.7	53.48	101.2	101.7	101	104.5	101.3	101.7
5.15	47.11	99.5	98	101.9	105.2	97.6	105.2
7.62	48.29	101.9	101.5	101.2	103.1	99	100
8.73	48.17	96.9	93.8	93.6	96	90.5	98.3
7.64	48.74	107.8	105.3	108.9	103.3	103.9	109.3
6.96	46.35	100	98.8	99.3	110	105.5	109.2
8.59	74.67	94.2	88.9	94.4	51.2	53.9	51.3
6.61	48.81	105.2	103.7	97.9	97.3	90.9	94.2
6.8	55.04	101.7	99.7	97.4	99.2	102.1	98.3
6.38	53.66	94.9	96.3	96.5	100.9	99.6	99.2

6.39	70.62	99.6	100.1	100	109.2	108.9	109.2
8.12	48.19	93.9	100.1	100.5	95.9	99.7	96.3
4.98	43.08	95.4	94.2	98	100.4	102.7	106.1
4.49	62.86	97.5	100.5	103.9	99.9	100.5	102.4
7.34	42.58	97	97.1	97.7	104.2	100.7	103
4.69	70.62	105.5	99.6	98.9	101.4	99.3	100.5
9.66	45.14	110	104.7	108	105	106.3	108.1
8.7	35.6	99.6	99	99.8	108.3	108.6	107.3
7.12	46.16	99	102.8	99.5	95.4	96.2	94.4
6.62	48	100.2	100.8	102.2	114.2	108.7	111.5
6.01	56.47	97.4	97.1	100.3	94.9	97	99
5.66	41.36	105.1	101.5	99.2	99.5	96.9	101.3
10.9	58	103.5	99.8	98.9	111.2	108.6	113.9
6.68	70.44	102.7	94.6	96.6	106.7	103.9	104.6
8.98	57.17	108.3	98.8	95.7	107.4	110.3	108.1
9.64	46.95	91	90	93.9	117	117.9	117.6
8.82	52.45	95.8	96.6	96.2	107.2	108	106.9
6.74	63.13	114.8	96.4	104	99.5	110.5	111.1
8.5	44.58	97.7	95.3	96	106	100.8	102.1
12.06	43.54	108.5	100.7	103.9	117.8	117.7	118.2
6.52	59.02	98.1	104.3	100.8	100.3	97.7	95.5
5.53	53.01	77.3	78.6	77.9	110.7	115.6	113.1
8.44	44.14	93.4	90.3	91.2	100.7	102.3	103.2
5.33	58.9	96	92.3	94.4	93.2	92.4	94.2
7.84	57.85	102.7	96.4	102	100.7	99.7	104.1
6.16	46.74	103.5	99.9	101.7	99	105.4	116.5
5.03	42.2	102.2	101.5	100.7	92	89.9	96.2
5.19	46.43	100.3	100.6	100.9	101.7	100.6	103
5.85	71.06	98.1	99.8	99	96.6	92.6	97.9
8	43.88	102.5	99.4	100.6	98.5	97.1	96.9
7.66	56.42	97	97	101.5	103.2	102.1	101.3
5.38	47.57	102.3	104.7	100.8	106.9	104	105.8
5.86	44.43	105.2	99.3	100.2	100.9	97.9	102.1
5.3	62.57	100.4	99.7	100.3	102.4	100.5	102.8
7.3	93.38	108.7	109.4	108.3	148.4	156.7	145.4
6.79	65.27	104.2	103.7	102.1	85.3	86.5	88.8
9.63	74.45	102.1	107.3	102.9	127.5	129.6	124.7
10.23	87.09	103	103.5	100.5	104.5	105.2	105
6.33	46.46	100.5	93.5	91.1	96.1	95.5	99.5
5.29	75.15	93.1	94.5	93	117.6	117.4	117.4
7.3	46.22	93.1	91	88	103.1	107.1	106.1
9.36	48.51	109	108.1	108.5	113.9	113.7	117.8
10.1	81.94	98.8	97.9	98.3	108.3	109.1	110.5
10.93	113.08	101.8	100.7	100	104.2	104.3	105.8
5.74	94.33	102	103.7	102.8	82.7	82.9	81.1
6.24	41.76	100.1	95.4	97.1	98.7	97	99.4
6.74	62.17	100.5	98.6	100.5	103.7	98.9	103.8
5.45	57.61	96.4	97	99.8	105.3	107.9	109
8.43	43.59	104.6	103.5	104.3	108	105.4	110
10.77	85.67	100.9	99.2	97.7	103.9	104.5	103.5
6.6	42.11	107.3	100.3	100.9	96.9	99	98.8

8.85	48.03	106	96.4	104.5	127.1	119.7	127.1
8.13	48.14	103.6	100.8	98.7	101.5	97.9	98.4
5.12	49.82	99.1	99.5	97.2	100	98.5	103.2
6.61	47.85	95.6	94.9	95.2	98	99.5	100.7
7.37	51.94	97.2	97.2	96.5	108.8	111.7	108.9
6.42	45.05	95.4	98.6	100.2	103.4	102.8	102.4
8.79	53.04	98.2	103.9	103.7	93.8	92.7	94.7
6.55	61.83	98.8	94.1	95	100.8	102.7	101.3
6.24	52.82	103.2	104.4	101.9	99.3	100.9	106.1
6.4	49.25	104	108	112	91.8	89.4	88.8
5.67	69.4	100.4	102.1	99.5	96.5	95.5	93.9
9.72	52.52	102.3	107	102.5	104	102.8	103
6.7	46.65	101.9	101	99.1	96.6	98.7	95.5
6.92	43.4	91.5	92.4	91.3	94.5	90.4	92.4
7.01	49.86	97.9	96	94	92.6	92.4	93.6
4.77	43.75	108.4	101.1	102.7	111.5	103.2	109.5
7.46	49.85	99.8	100.9	102.8	99.2	99.7	103.4
9.07	41.01	103.9	96.6	110.6	97.7	105.3	103.2
5.31	45.15	101.5	97.9	97.8	114.1	111.8	114.2
6.15	51.99	109.7	98	96.2	101	103.6	103.5
5.69	43.03	115.7	106.2	109.3	98	100.9	104.6
9.67	44.4	104.8	102.4	102.3	110.9	106.2	108.1
7.91	77.48	90.5	92	89.6	107.1	104.7	106.1
4.32	70.2	110.6	107.7	107.9	100	101.8	101
5.26	36.38	101.2	101.9	99.8	99.6	102	102.2
5.68	55.03	101.6	102.8	104.5	108.3	112.2	106.7
7.39	42.99	99.4	98	98.6	105.3	102.9	101.4
6.98	74.76	90.5	93.4	92.9	100	99.4	100.1
8.62	75.85	100.5	101.7	103.4	104.5	103.8	104.2
5.76	43.7	102.9	101.2	102.8	118.6	123.6	127.9
8.82	48.75	101	101.2	99.7	98.8	100.4	97.9
10.1	48.49	105.9	102.9	105.4	105.5	108.1	106.9
7.44	57	102.1	106.2	100.8	95.3	96.2	96.5
9.95	76.13	89.8	96.1	99.4	113.1	116.9	107.2
5.41	40.9	98.2	103.6	105.3	86.4	85.7	85
9.07	50.12	101.3	100.3	103.9	97.2	97	97.2
8.27	44.51	100.7	100.5	100.8	110.8	109.9	112.2
9.83	283.69	94.6	97.3	113.9	112.1	131.3	136.8
9.03	39.74	100.5	101.4	93.1	100.5	100.1	105.1
7.46	37.21	95.1	95.1	94.1	100.3	97.9	97.9
5.02	46.5	101.6	102	99.6	97.7	97.8	99.9
8.56	43.25	110.4	105.7	111.7	109.8	109.6	111.1
8.56	38.3	98.5	98.6	98.3	94.4	93.6	96.7
7.5	37.9	98.9	106.4	100.5	104.2	100.5	104.5
11.52	96.87	115.6	115.7	116.9	104.5	111.9	101.2
6.32	53.32	88	87	87.3	89.8	90.1	91.5
6.4	84.23	96.5	100.4	102.4	92	91.4	98
6.7	42.56	101.3	101.8	102.6	100.9	98.6	100.3
8.4	36.87	102.1	98.8	101.6	107.4	111.6	108.9
5.69	46.54	102.5	103.4	99	96.7	98.3	98
6.4	45.26	105.7	102	106.6	132.2	130.2	138.7

5.47	37.69	102.3	98.1	100.8	99.7	98.2	103
5.39	37.21	99.9	100.2	98.3	99.3	99.5	105
5.53	56.24	97.1	99.2	97.9	102.5	106.3	104.3
9.44	40	100.5	99.9	101.8	104.2	103	104.4
5.4	42.59	94.5	94.1	97.1	117.2	118.7	121.9
5.88	45.57	129.7	129.9	132.2	84.8	91.3	87.8
6.46	41.7	108.2	108.3	105.5	112.4	113.4	114.6
5.27	51.05	92.5	94	98.7	148.3	144.2	144.1
7.61	57.92	97.8	97.9	100.5	103	101	102.4
8.66	56.69	106.9	106.4	105.2	105.8	103.5	105.6
6.7	71.74	95.4	95.4	93.5	110	108.7	107.1
5.68	45.01	105.5	100.2	104.9	87.2	88.2	91.6
9.94	48.45	102.9	104.2	104.6	107	108.7	105.9
7.94	46.66	89.3	108.5	97	105.8	103.8	117.3
6.93	42.84	90.2	94	93.4	101.2	100.9	100.4
5.53	41.33	99.9	94.2	98.9	108.6	112.6	113.8
6.92	45.67	100.8	99.1	97.8	106.2	104.9	103.6
6.44	40.96	101.4	103.9	103.9	96.7	93.3	95.2
4.36	51.09	108	104.6	104.6	109.9	108.7	107.3
6.13	79.67	100.4	102.3	99.9	93.7	92.3	94.1
8.4	40.62	101.5	105.1	102.5	96	96.5	97.7
6.29	41.18	96	91.6	94.7	96.6	94.4	100.8
9.55	41.96	101.9	103.3	101.4	106.5	108.6	104.9
5.87	45.82	95	93.3	92.2	117.7	115.2	114.4
6.8	38.99	98.1	98.4	98.5	100.7	100.9	100.2
6.92	46.13	101	99.7	99.8	106.7	108.8	107.6
4.74	98.41	94.7	97.9	98.5	114	113.6	103.5
7.39	49.97	97.9	102.4	100.1	100.3	97.5	103.1
9.32	55.44	95.7	96	97	97.6	96.3	98
6.73	43.08	104.8	106.3	105.6	98.3	100.2	100
6.55	55.36	103.7	105.1	104.1	98.9	96.3	98.5
9.7	46.7	106.7	99.1	100.1	96.7	99.4	100.1
5.41	51.05	100.4	103.4	102.9	100.6	99	98.6
5.43	59.02	114.7	114.2	89.8	114	90.9	77.8
6.74	45.58	107.9	105.4	106	114.6	113.5	116
6.38	48.19	99.2	96.7	100.6	97.2	95.9	98.7
5.6	41.48	102.4	102.4	104.8	105	105.9	106.4
7.02	39.11	99.3	103.1	107.1	101.5	105.5	104.3
8.43	74.91	103.1	100.5	98.2	94.7	94.1	92.2
9.2	54.62	107.4	101.1	98.7	114.2	113.8	111.1
6.19	40.1	90.3	98.2	95.6	97.9	94.6	94.1
5.88	48.95	97.7	97.5	97.3	95.9	98.3	97.4
6.89	42.98	95.9	96.2	95.6	105.5	104.6	104.9
6.79	68.58	104.9	104.7	108.6	93.6	95	96.8
9.32	63.42	100.5	101.3	101.4	104.6	103.7	103.5
6.33	52.91	98.7	101.1	100	99.1	95.7	97.2
5.03	79.46	98	104.8	107	109.7	109.7	107.8
8.24	56.34	105.1	101.1	98.2	92.1	89.9	94
7.05	46.01	103.5	104.4	100.1	95.2	92.1	91.9
9.16	44.82	99.3	95.6	96.6	99.5	98.6	99.2
6.29	36.97	104.5	98.2	99.5	98.8	95.1	100.7

9.22	56.78	99.1	101.5	99.7	101.1	103	99.3
6.27	50.16	94.5	97.6	94.1	102.4	101.6	100.3
4.06	58.63	108.2	108.9	107.6	104	104.3	102.7
7.69	39.68	102.2	102.1	103.9	98.8	96.7	98.1
5.41	38.93	101.8	101.3	98.9	98	97.8	96.3
7.27	51.14	101.2	98.5	101	97.6	99.7	98.4
4.93	43.96	97.6	101.8	101.4	102.7	101.1	106.5
10.49	73.22	100.1	103.5	101.4	103.3	102.1	101
4.92	40.91	96.1	96	95	93.3	92.5	93.5
5.94	47.91	88.3	87.2	110.2	108.4	140.2	116
5.17	42.37	83.5	78.6	80.9	90.8	91.1	93
6.05	38.9	97.9	98.5	100.8	103.6	101	104.1
7.02	36.05	114	112.1	119	101.5	101.8	104.5
7.52	38.37	109	100.4	99.3	101.3	98.9	98.2
8.25	37.75	97.8	101	99.7	103.2	98.9	98.1
4.64	39.01	102.1	102.4	105.4	93.9	93.4	100.4
8.48	37.93	100.4	100.8	101.5	96.6	100.5	96.2
5.2	61.42	100.3	103.9	100.4	100.1	96.2	96.6
8.72	32.06	98.7	102	102.6	131.6	131.9	127.5
7.49	38.09	98.2	95.3	95.6	101.6	105.1	110.1
5.27	30.75	118.9	106.2	108.5	84.4	84.3	88.2
5.02	50.37	101.3	98.8	100.9	89.4	87.9	88.1
7.49	41.3	117.9	95.4	94.9	96.3	108.5	96.3
5.91	44.36	98.3	98.7	95.2	97.8	99.1	100.9
4.91	37.87	97	96.5	95.1	102.1	101.9	102.2
8.81	40.51	101.9	103.5	98.4	107.9	106	104.5
7.68	36.55	103.9	96.9	100.6	101.5	94	104.9
5.33	33.16	98.1	100.4	98.3	98.9	92.7	92.4
5.57	34.74	106.6	108.9	103.4	105.8	101.8	106.9
6.55	34.68	102.2	97.6	98.4	99.5	96.1	99.7
6.87	57.4	105	105.8	105.5	120.2	106.3	108.2
6.58	68.58	99.2	102.7	99.7	97.6	96	93.5
5.53	32.99	99.7	100.8	100.8	104.4	98	100.3
6.58	48.71	100.5	103.8	101.7	100.8	98.8	99
4.91	36.83	101.5	99.3	101.4	100.6	98.9	101.6
8.82	42.45	102.4	98.5	102.3	93.3	92.6	94
10.32	86.57	110.7	111.8	111.4	117.8	118.7	117
8.18	40.72	100.7	99.8	100.6	109.5	108.1	105.7
7.39	33.05	95.7	97.2	94.6	104.4	106.5	108.6
9.44	43.9	99.8	97.9	97.7	100.1	101.2	104
5.15	74.47	102	99.9	97.9	106.3	106.3	106.2
9.42	46.85	111.9	103.2	106	103.8	106.8	103.7
7.64	36.7	95.7	94.2	94.8	107.7	127.2	111.1
8.02	45.86	100.8	104.2	104.8	104.8	108.9	106.7
8.05	53.49	90.5	98	97.7	85.2	89.1	98.3
8.63	34.36	94.3	102.2	97.6	101.6	105.9	101.9
8.09	44.49	102	99.8	99.3	94.3	93.9	96.3
6.33	49.68	103.1	100.1	100.1	97	96.3	97.6
7.66	31.49	98.6	97	101.8	101.9	103.3	98.6
9.13	45.74	108.8	106.5	105.9	113.7	108.5	107.5
5.73	37.14	76.4	71.5	72.6	99.9	96.3	99.1

6.89	47.12	101.1	100.2	98.9	100.9	99.2	102.5
8.19	84.98	96.9	96.9	98.6	120.3	122.2	121.5
4.89	54.1	105.8	105.3	105.1	111.9	106.8	105.8
7.23	57.04	95.6	100.5	96.8	103.7	103.5	104.4
6.57	44.52	100.5	99.7	103	94.3	95.6	97
6.44	37.43	109.1	109.8	114.4	83.1	86.7	85.3
8.18	39.71	103.1	100.5	100.7	111.9	107.1	110.7
7.15	41.17	98.9	98.8	98.5	101.4	97.4	102.4
5.92	49.92	105.8	111.6	108.6	86.8	86.9	87
4.73	46.94	99.4	99.6	101.2	98.8	96.8	100.4
7.44	33.43	97.8	98.8	93.5	97.4	97.3	95.8
6.65	50.38	99.7	103.3	99.8	100.7	103.8	101.1
7.17	59.94	95.7	95.1	91.2	96.5	97.4	96.8
11.44	51.46	96.9	99.7	100.4	110.2	110.8	106.5
8.44	47.64	97.5	97	98.9	99.6	96.8	100.8
7.96	37.11	96.2	100	99.8	106.1	101.6	102.4
6.13	42.47	92.7	92.5	94.1	89.9	87.8	87.8
8.22	40.06	101.2	101.2	99.7	96.3	97.7	95.4
8	47.8	98.9	97.8	97.3	98.9	99.3	100.6
7.02	52.87	100.5	98.7	100.3	111.5	110.6	110.4
8.73	39.26	98.2	100.1	99.5	95	95.4	96.4
7.84	45.84	105.1	103.2	100	99.1	98	107.8
6.35	39.66	96.3	93.5	95.7	105.1	104.6	106.8
6.07	41.01	96.1	97.2	100.6	99.4	106.6	100.5
4.82	43.8	96.7	102.3	100	95.9	94.9	111.2
5.24	47.29	97.8	99.6	97.2	101.2	103.1	101.7
5.73	47.98	96.5	100.6	96.7	104.9	100.7	101.6
8.09	38.74	103.3	102.6	101.9	98.1	96.1	103.7
7.3	45.26	99.1	98.9	96.1	99.5	99.5	96
7.75	37.74	96.1	97.2	99.1	95.7	94.7	97.7
5.6	33.67	101.5	96.3	97.7	113.3	105.8	112.4
6.19	37.75	87.6	85.7	89.4	115.1	116.5	114
6.44	43.4	97.1	96	94.1	104.4	103.2	103.3
6.05	93.01	109.1	96.6	88.6	81.4	87.4	79.6
10.58	80.21	104.9	104.5	105	103.6	101.6	101.1
7.69	46.38	98.9	100.4	101.5	108	104.8	106.9
8.25	58.69	103.7	104.1	103	78.1	77.1	79.2
6.54	36.71	103	96.8	102.6	100.7	96	99.9
7.87	42.07	97.8	97.5	100.6	106.9	107	104.7
10.93	64.54	104.5	102.7	101.2	99.1	96.7	96.7
8.13	36.8	102	93.8	99.5	97.7	97.8	95.4
6.54	35.27	100.2	100.9	102.9	98.3	100.3	99.2
9.89	38.89	95.4	91.5	93.5	101.9	99.2	100.3
5.6	37.23	101.1	102	99.7	107.2	104.9	110
5.14	43.97	94.4	92	92.5	102.1	103.4	106.4
6.04	39.48	102	99.9	102.1	102	99.3	101.3
8.92	70.98	102	104.9	104.7	109.6	103.5	107.4
8.9	36.8	101.6	94.1	101.8	104.7	106	103.4
8.15	42.82	103.1	97.1	100.5	99.7	90.9	98.4
5.45	33.99	99.1	99.4	99.8	97.5	99.7	100.7
7.66	34.55	105.8	96.6	100	105.9	101.5	110

9.38	41.14	99.5	100.9	99.3	107.7	110.9	105.6
7.84	34.99	98.4	94.8	95.6	95.6	95.7	98.7
5.16	35.33	110.3	109	110.2	92.5	86.1	89
9.51	55.36	104.1	99.2	100	104.9	106.8	106.8
6.04	41.36	97.2	98.5	100.2	91.8	94.7	93.1
7.21	44.04	96.7	93.1	96.3	103	102.9	101.8
6.18	50.72	110.1	115.1	117.5	91.9	89.4	96.8
8.38	30.15	104.9	111.6	93.2	96.6	102	108.2
6.44	45.03	95.2	100.2	101.6	101.7	99.8	99.5
5.8	34.46	91.7	89.1	89.5	97.2	99	100.7
5.76	48.45	100.5	101	95.4	105.2	96.8	104.2
6.68	39.11	101.8	102	100.8	96	98.8	98.2
6.09	42.67	103.1	86.4	92.1	98.4	105.6	109.5
5.25	42.03	99.3	95.1	97	97.5	93.2	97.4
7.23	33.19	107.1	97.6	99.6	103.5	96.5	99.5
5.24	48.85	101.2	103.4	98.3	105.7	106.1	104.3
5.94	64.38	99.4	98.5	95.8	99.2	102.1	100.4
8.29	33.75	100.2	104.7	107.4	97.6	89.2	94.1
5.01	33.12	99.3	91.6	94.1	95.7	97.6	99.3
6.49	34	94.7	96.8	98.7	99.3	98.5	101
6.93	39.62	102.2	98.1	103.7	86.7	88.9	95.6
4.75	36.79	104.5	103	99.1	98.5	96.6	101.1
5.02	60.36	111.7	105.6	104.6	97.5	100.9	102.6
6.2	40.52	97.8	96.3	99.7	97.5	97.5	103.5
9.5	33.98	99.4	103.9	106	110.8	111.8	110
5.08	28.3	101.6	98.2	99.1	99	100.9	103.4
6.4	34.51	100.9	102	102.6	100	99.9	98.8
6.48	36.52	97.5	96.9	96.3	100.1	97.1	98.7
7.15	43.07	101	100.4	102.7	106.1	102.6	105.4
4.87	40.7	96.4	96.4	106.3	100.4	107.1	100.7
8.66	35.75	100	100.8	98.7	99.9	99.4	103.7
6.32	41.24	103.4	88.2	94.7	84.8	116.5	87.1
7.15	33.65	93.6	96.7	96.9	118.7	121.5	121.7
5.17	29.33	91.8	82	89.4	81.6	80.8	80.2
5.73	28.74	108.1	107.2	104.7	109.6	108.1	106.4
8.6	28.97	99.5	97.1	102.6	101.3	102.2	102
8.65	35.51	106.1	107.8	107.2	105.1	104.8	107.9
4.78	29.18	101.6	103.9	97.5	97.1	101.8	96.7
7.17	42.69	96.7	94.4	98.9	98	96.1	98
7.5	41.69	100.4	100.8	98.3	115.5	112.1	113.4
4.92	31.22	106.4	94.2	93.7	96.7	95.7	99.1
4.96	27.72	95.6	96.9	98.1	105.3	112.9	113.7
5.1	32.64	104.7	96.1	99.2	108.4	108.5	109.4
7.33	62.91	129.7	129	127.9	86.6	82.1	88
6.84	35.2	100.4	90.7	103.1	107.2	106.3	114.3
9.36	71.66	110.3	105.6	108.4	103.9	106.7	104.9
6.74	54.21	94.9	94.2	100.1	100.9	98.6	101.4
8.72	44.52	130.9	133.1	128.6	85.1	82.1	80.5
4.84	34.59	101.2	103.2	100.4	104.2	101.9	107.5
6.55	68.75	94.7	97.5	97.7	100.6	97	97.9
7.33	47.38	99.1	99.6	100	99.5	96.9	98.1

10.49	48.31	103.2	105.8	106.3	103.7	105.9	104.4
8.56	45.9	95	96.6	90.5	98.7	92.3	89.5
5.07	51.68	102.9	95.7	98.9	105.6	115.2	114.5
5.35	35.47	100	98.7	101.6	104.7	103.4	104.9
8.46	33.21	101	95.6	105.5	107.4	115.7	112.6
6	41.51	101.9	95.3	103.1	100.3	104.3	105.5
4.83	30.14	109.6	110.9	108.1	75.4	76.2	86
6.98	44.5	98.5	97.8	97	101.2	98.6	97.7
7.81	47.37	103.7	104.7	100.8	102.2	97.7	97.9
8.13	30.23	100.8	106.5	100.9	90.6	87.6	90.5
8.63	30.76	103.3	100.1	98.1	105.2	99.6	100.1
5.91	43.62	102	101.8	105	109.8	108.6	109.9
8.85	32.8	102.2	101.3	98	94.5	95.3	93.1
6.54	30.29	97	89.9	97.6	100.2	104.5	104.7
10.9	82.08	116.2	111.7	112.7	106.5	105.2	105.3
8.62	32.98	97	96.5	103.2	107.4	106.9	103.7
7.24	36.34	100.8	102.9	98.4	102.1	95.4	100.9
9.52	44.45	96.3	103.7	99.8	107.1	105.5	107.9
6.06	28.71	94.5	94.3	94	95.7	100.9	106.1
5.44	54.48	102.8	100.4	100	100	97.8	100.3
5.9	31.5	101.5	103	104.2	103.5	96.6	99.2
6.83	35.72	103	101.1	97.5	106.1	102.9	101.6
4.79	42.45	99.2	97.5	97.7	101.4	101	100.9
8.65	44.65	103.7	101.9	104.6	105.6	109.1	105.4
10.65	55	100	99.8	101.5	105.5	104	104
12.02	90.86	100.9	100.3	100.7	105.2	105.2	106.6
7.4	32.65	109.6	105.3	107.8	106.4	95.6	103.4
10.46	64.75	102.2	107	104.8	98.6	100.5	97.6
6.37	37.24	89.2	91	92.2	99.6	101.8	101
7.01	40.5	102.2	100.7	100.3	99.5	94.1	97.6
9.95	58.17	99.5	99.1	102.1	105.3	103.7	103.1
6.3	72.06	94.4	94.4	93.1	108.1	104.5	105
6.14	36.19	94.2	93.4	98.6	100.1	97.9	107.1
5.16	40	97.1	96.8	94.2	106.5	111.2	111.8
5.96	25.34	94.5	94.1	99.9	97.3	99.3	101.6
5.6	42.63	103.2	98.9	101	136.5	142	144.2
8.66	44.18	97.2	92.6	93.5	98.6	96.6	98.1
7.71	31.56	99.6	91.5	92.9	110.1	104.9	110.7
6.24	36.42	89.2	94.5	92.5	96.7	97.1	100.6
9.38	43.03	101.9	104.7	104.7	106	100.8	102.5
5.59	46.99	93.9	94.4	94.3	95.6	97.4	96.5
5.24	47.12	103.4	103.6	101.1	102.2	101	101.8
6.37	24.49	99.1	96.5	93.5	97.6	95.8	101.8
4.91	80.7	100.8	102.1	103	107.5	106.1	108.5
5.73	32.15	101.3	98.2	100.6	135.2	136.5	135.6
9.29	30.62	102.2	101.9	104.9	95.5	98.1	97.7
7.15	36.34	102.7	102	103.1	91.3	87.7	90
4.97	33.61	102.6	99.2	98	100.7	101.8	99.9
4.94	49.2	98.7	98	97	108.4	99.4	107.5
9.77	46.25	109.5	109.1	110.3	98.8	99.3	98.5
6.19	26.6	107.5	102.5	102.6	106.3	105.9	109.6

4.92	39.66	100.9	99.4	101.8	97.7	100	102.8
6.13	28.96	104.4	106.3	102.9	106.8	106.9	113.1
7.85	29.71	94.2	98.4	93.7	97.4	98	102.2
5.5	43.93	94.9	92.2	98.1	99.6	102.5	104
9.52	47.9	99.3	102.1	104.4	100.4	99.4	100.6
8.12	33.37	100.1	101.3	101	93.1	95.1	93
5.59	45.66	102.6	100.2	100.6	96.7	98.8	95.4
5.48	114.46	104.1	103.2	112.5	95.6	104.9	95.1
5.07	33.52	97.4	103.7	101	102.4	104.6	104.1
6.95	43.37	102.5	102.2	103.3	95.6	93.5	94.1
7.58	29.59	101	100	96.5	109.2	107	109.7
7.69	35.62	95.9	96.6	94.8	93.7	98.1	93.9
7.65	38.81	92.8	100.7	92.4	95.1	92.4	98.2
6.2	38.4	103.7	102.8	102.7	90.9	91.7	93.2
6.84	34.52	107.3	101.5	102.5	109.7	111.1	108.6
4.64	55.02	97.2	96.8	96	99.1	98.6	98.9
5.14	25.76	97.8	93.8	98.1	106.8	100	100.6
8.97	47.91	103.2	101.3	102	95.8	94.8	98.9
6.2	36.87	99.7	104.7	106.2	103.1	99.3	100.8
4.09	55.65	109.8	107.9	113.2	99.6	99.2	97.8
4.82	52.67	101.8	99.3	99	98.2	103.5	102.1
7.43	53.35	109.4	95.9	102.6	95.8	93.9	95.8
5.57	52.98	95.1	95.6	91.4	91.4	88.9	89.5
8.07	33.65	104.3	104	106.1	93.5	97.5	92.2
5.82	39.77	106.7	99	103.1	99.4	100.9	104
7.44	46.28	101.3	96	97.7	102.5	105.5	102.5
9.38	32.35	97	94.7	94.3	94.1	90.2	94
5.44	37.31	103.8	103	99.9	109.9	108.3	107.3
5.35	38.08	102.2	101.4	101.1	96.6	100.2	101
8.48	28.67	103.6	96.6	100.3	106.8	103.7	103.5
6.32	34.38	98.8	96.1	96	104.2	105.6	104.4
9.16	32.11	106	101	95.1	100.3	93.2	97.5
6.65	38.74	118.4	122.7	124.4	101.8	97.3	95.5
5.62	36.49	99.8	96.3	95.9	100.2	98.5	99.3
6.93	31.92	97.4	96.9	97.5	95.3	95	94.8
8.79	35.85	97.8	98.7	96.2	102.4	100.4	101.3
5.15	31.89	107.1	105	103.7	100.1	97.9	95.9
6.77	37.55	116.6	113.9	114.3	73.4	71.3	73.5
4.86	39.78	103	100	97.3	109.5	102.9	108.4
6.96	34.43	97.9	94.3	96.2	98.6	99.2	98.5
9.19	47.57	100	102.5	97.8	97.6	100.4	102.9
7.3	35.71	96.4	103.8	102.3	102.6	107.6	103.5
6.77	36.95	100.3	103.7	102.6	97.8	90.2	95
6.48	26.3	97.4	99.5	107.4	103.6	99.7	102.5
5.01	30.32	97.7	93.3	101.6	88.8	95.2	100.1
6.95	31.46	94	94.9	91.7	100.3	100.7	101.6
7.33	28.33	93.6	101.9	101.3	102.5	104.1	103.7
8.59	33.48	107	103.2	100.7	101.9	102.7	106.5
8.63	25.78	104.7	106.3	104.6	106.4	103.9	101.3
6.9	29.5	96.1	105.4	107.7	106.8	111	110.8
5.66	24.28	102.6	103.2	105.6	106.6	104.3	104.1

9.57	26.59	98.4	101.1	100	104.1	102.1	104.2
8.88	25.73	90.2	101.8	93	103.9	113.5	108.7
6.74	28.93	100.5	92.8	102.7	99.1	100.9	107.5
5.4	38.05	101.7	100.9	102.8	97.3	98.8	94.8
6.62	45.58	97.6	102.2	102.2	99.4	97.4	98.7
6.32	39.97	102.7	100.5	100.6	109.9	103.4	105.3
8.13	31.48	105.6	100.1	101.7	100.9	96.3	101.9
5.83	30.25	101	94.7	97.9	103.4	104.4	102
10.1	69.52	96.5	99.9	99.2	103.5	107.6	107
9.54	41.18	102.9	100.9	103	122.9	124.5	120.4
8.81	52.07	99.9	100.8	100.4	109	107.7	106.9
5.74	32.71	98.1	99.2	103.9	96.2	97.1	96.5
7.44	41.27	93.9	97.3	101.7	104.8	102.9	102.9
9.6	53.48	100.3	101.1	102.4	112.9	112.6	110.7
4.91	35.58	100	96.8	98.3	98.2	97.6	97.6
4.7	45.74	100.3	99.4	96.1	106.8	104.5	104.7
4.86	92.61	59.8	56.8	57.3	168.3	166.9	167.7
4.26	44.97	93.9	96.3	96.8	97.7	98	97.6
8.63	38.65	97.1	97.7	94.9	95.3	94.1	96.6
7.77	39.09	99.4	96.1	100.5	100.8	104.1	101.2
8.97	44.56	96	97.9	100.4	108.9	111.5	110.4
8.24	33.46	104.8	101.2	105.5	100.8	107.1	105.2
8.87	40.34	119	116.2	107.5	105.6	82.6	89.2
5.16	31.59	95.5	92.8	92.9	91.5	92.2	91.8
6.43	32.94	109.8	93.3	91.8	90.2	93.2	104.5
9.5	31.69	95.9	99.2	95.9	111.4	113.5	115.5
6.67	43.62	100.7	95.7	103.9	99.2	94.2	85.4
9.14	36.13	101	105	104	102.4	101.6	102.3
7.01	41.22	102.4	98.7	99.9	97.5	101.7	100.8
6.64	24.02	93.3	101.2	103.2	101.7	103.1	100.9
6.01	36.32	103.3	101.7	107.1	94.1	95.7	93.2
7.99	25.85	73.8	69.5	69.6	91.4	91.2	93.4
5.4	34.68	104.1	100.2	101	97.5	93.3	97.3
6.3	41.17	134.8	122.1	119.6	61.4	77	73.3
8.48	33.77	99.9	103.5	102.5	98.5	100.9	102.5
7.87	45.59	104.1	96.7	104.4	107.2	104.5	108.3
6.28	33.69	90.3	89.6	88.7	102.6	102.2	105.8
8.91	27.04	96.3	103.7	97.8	101.1	100.7	101.8
8.47	33.08	97.7	100.4	100.2	99.4	99.1	103.3
7.3	34.41	100.1	99.3	99	101.7	97.9	101.3
8.66	40.84	102.9	101.3	105.6	103.2	104.4	101
8.4	28.79	103.1	98.4	98.3	110.8	108.9	108.4
8.09	28.29	98.6	99.6	94.1	96.1	99.1	95.3
5.97	140.03	93.7	89.8	108.4	82.3	118.8	97.8
5.17	41.74	96.8	102	100.1	90.1	91.2	93.4
8.28	30.89	104.6	102.1	110.6	95.5	102.2	95.1
4.32	72.44	98.1	101.1	97.4	104.3	101.6	100.8
9.8	28.34	98.3	93.5	101.5	105.4	104.3	105.8
7.75	31.29	103.6	104	107.3	115.5	114.3	116.1
8.21	28.73	92.7	94.7	92.7	102.7	102.4	104.4
7.43	28.82	92.3	90.7	92	114.2	119.2	124.3

6.47	51.82	94.3	94.5	93.3	101	96.5	97.7
8.56	45.01	102.7	99.6	97.7	102	100.5	101.5
4.92	35.04	96.6	96.2	104.9	106.6	107.6	109.4
4.87	36.59	102.3	102.8	98	103.6	104.5	104.8
4.44	36.12	97.2	93.1	102	104.8	98.4	99.9
7.36	35.02	94.3	91.3	96.7	106.5	103.9	99.8
5.11	24.83	100.9	99	104.3	99.4	98.7	103.4
7.12	95.06	102.6	100.8	99.8	94.8	95.4	94.3
6.76	32.04	99.3	95.1	97.1	101.1	102.7	104.8
6.44	38.19	95.5	94.1	94.8	97.7	100.3	100.5
6.52	31.51	104	102.2	100.8	108.8	106.4	105.8
6.6	25.27	105.1	100.2	99.6	109.5	107.4	111.7
7.17	36.87	106.5	106.5	107.1	96.4	96.3	90.9
8.95	25.33	94	93.7	99.8	102.5	100.2	103.5
6.71	28.98	97.5	97.3	95.7	104.9	103.6	107.2
5.31	31.31	100.3	98.4	95.4	110.1	99.6	103.1
10.11	56.1	101.7	106.9	103.8	110.3	108	106.2
8.02	50.27	101.1	104	101.9	100.2	96.4	95.2
8.73	34.13	97.1	97.5	94.6	114.8	118	114.7
8.78	37.46	103.4	101.1	105.4	107.7	110.2	110.8
8.02	36.67	78.4	73.8	77.6	96.2	101.1	95.6
9.95	40.86	98.1	98.6	99.8	110.5	110.5	109.1
6.18	25.86	97.2	89.2	92.8	115.2	112.5	113
7.61	41.95	85.1	89.3	87.2	79.8	95.1	96.6
5.11	41.94	97.8	99.5	99.7	100.1	99.5	98
6.34	29.63	98.8	96.2	98.8	100.1	100.6	101.9
6.77	26.16	102	93	91.5	99.4	102.9	101.3
5.14	28.61	102.8	96.1	99.5	96.2	105.7	100.7
8.22	42.39	99.8	97.6	104.9	100.3	105.7	97
5.57	31.69	75.8	81.4	81.8	121.9	125.1	129.8
6.47	31.02	97	98.8	100.3	102.4	105.4	102
7.46	27.84	107	105.9	104	110.3	102.7	107.7
6.44	34.6	105	98	102.7	94.9	95.4	93.6
5.92	37.55	92.2	94.3	94.7	96.9	100.7	100.4
5.17	28.85	108.7	104.4	104.8	90.8	91.5	92.3
8	24.02	105.2	99.8	99	100.2	115.3	106.8
5.4	29.15	102.8	99.8	105.6	96.3	99.9	98.6
8.09	31.75	94.4	97.5	94.5	106.2	117.2	102.7
11.59	59.47	97.1	99.4	99.6	105.6	104.8	107.4
6.65	41.21	97.9	98.1	97.6	96.1	93.8	97.5
5.81	32.8	106.7	107.2	110.5	100.1	94.5	97.1
5.71	27.88	97.3	98.7	100.9	100.8	103.7	106.3
6.18	24.27	101.4	91.1	91.6	94.6	94.6	94.8
5.59	30.61	107	99.7	98.8	101.1	99.9	99.7
6.55	28.85	103.6	97.2	101.9	106.2	112.1	111.4
7.39	35.21	100.3	99	103.5	98	101.4	102.2
6.67	31.16	104.6	102.5	107.3	100.7	99.5	104
6.01	26.92	102.6	98.8	98.3	99.5	99.3	109.8
7.56	36.31	102.9	101.5	106.7	105	105.3	107.9
6.55	41.11	95.6	98.1	99.4	102.5	101.2	102.4
5.83	38.98	105.3	107.2	107.5	102.8	102.1	106.1

5.86	29.19	99	96.2	97.9	100.7	97	98
6.02	42.49	104	101.4	102.4	108.6	107.6	108.8
8.12	21.15	100.4	100.9	102.1	101.7	102.1	110.5
10.55	47.52	104.8	104.5	105	107.1	107.9	106.8
7.34	22.57	99.1	101.5	96.8	99	98.3	102.6
7.71	33.41	111.3	114.7	108.7	88.2	85.1	87.2
6.15	46.76	97.9	99.8	94.5	97.9	95.4	96.5
7.96	27.59	100.3	95.1	94.6	100.5	101.9	105
4.77	34.1	106	103.1	104.6	96.8	96.9	96
5.81	28.78	103.6	106.1	104.2	99.8	99.1	100.5
5.55	30.71	106.2	102.8	103.6	104.2	102.8	106.4
7.68	35.02	100.6	100.8	100.3	104.3	99.9	100.3
11.82	46.49	101.9	102.9	103.6	112.2	107.3	108.6
11.59	49.43	100.4	101	100.4	114.8	118.3	116.3
9.55	64.49	103.2	93	99	103.7	100.3	112.5
5.55	31.42	95.6	99.9	99.1	100	96.7	97.4
4.63	32.49	101.4	97.7	99	96.6	97.5	97
6.6	38.21	99	99.3	96.4	100.5	98.1	100.5
7.81	31.38	111.1	114.1	113	107	105	102.9
6.16	29.13	102.7	103	98.7	110.4	104	106.4
7.53	27.54	110.8	106.9	106.2	99.1	96.7	95.8
6.6	35.2	102.4	100.9	98.3	90.8	89.1	94.8
9.91	35.14	104.1	101.7	104.6	98.2	98.5	100.2
6.23	26.23	97.1	100.8	95.3	94.5	103.3	129.3
6.95	28.48	99.4	94.3	94.3	100	102.3	99.7
8.88	36.71	102.3	103.6	101.7	103.6	102.2	103.2
7.03	31.21	96.1	98.6	94.9	101.9	97	100.6
4.81	45.79	78.6	76.4	77.3	126.1	119.2	124.9
7.74	28.32	95	98.8	98.7	113.5	107.7	107.3
5.73	35.65	100.4	101.2	100.5	102	101.8	103.4
5.3	33.07	98.5	97.2	101.8	98.5	98.2	100.8
5.62	27.41	98.1	92.6	87.2	111.1	107.4	107.8
10.13	66.84	100.5	97.6	101	105.5	104.5	107.4
6.46	21.97	102.9	99.9	100.6	97.7	88.9	93.4
5.55	34.79	99.1	99.3	98	103.5	104.1	101.3
8.48	30.44	96.3	96.2	97.7	108.8	102.4	106.2
11.25	28.26	104.4	109.3	103.9	109.4	104.9	99.6
9.5	27.34	102.5	102.1	102	104.3	103.1	104
7.28	34.99	117.3	115.6	118.4	75.8	74.7	73.8
8.29	38.32	101.1	102.5	99.7	102.8	102.6	101.2
7.8	24.32	97.5	91.1	95.5	100.8	105.8	100.7
7.99	27.37	99.6	102.3	106.1	99.3	96.8	96.8
7.31	28.03	95.5	94.3	96.1	99.5	100.8	106.8
6.89	25.4	98.8	104.7	100	91.7	96.1	92.6
8.68	31.5	111.6	105.3	104.9	101.3	97.4	101.4
6.23	29.47	99.7	107.1	97.2	101.2	100.5	101.7
10.49	66.28	101.8	104.5	101.5	104.3	102.8	102.6
5.22	25.8	102.8	99.6	96	99.2	94	94.3
5.25	31.7	99.6	98.8	102.9	101.2	102.7	106.1
8.78	28.32	107.9	100.5	104.1	104.5	102.1	104.3
6	38.22	96.8	96.6	95.5	99.4	98.4	101.5

7.78	53.92	96.4	98.5	97.7	105.1	107.8	103.6
5.92	45.57	106.5	106.5	104.2	104.6	101	102.4
5.14	23.04	98.4	95.4	97.4	97.3	98.6	96.8
6.48	25.42	95.3	94.4	98.5	104.4	104.5	103.8
5.45	28.29	101.7	99.7	100.7	102.7	103.4	100.8
9.09	24.26	101.8	100.9	108	96.3	96.8	118.8
5.97	30.73	108.4	89.3	88.3	98.1	127.4	110.5
7.8	33.33	102.1	102.2	101	102.9	99	98.9
8.38	29.76	106.4	102.5	100.5	95.1	102.5	99
7.08	38.52	74.7	87.3	106.8	117.6	91.1	94.9
8.24	25.56	103.3	104	101	91.5	86.3	86.2
6.7	33.02	101.5	101.1	99.9	97.6	97.1	96.3
11	44.64	99.2	95.1	98.2	105.7	106.8	105
8.15	27.73	89.2	90.1	89.9	106	106.2	106.3
8.91	40.53	101.8	100.7	98	99.2	103.7	102.8
7.36	24.52	102.6	101.3	102.2	105.1	104.1	102.9
8.66	25.92	106.2	103	104.2	88.6	93.7	94.8
6.28	30.63	90.4	87.3	86	87.8	93.5	91.2
7.21	29.14	103.8	96.4	103.8	97	98.9	99.6
6.2	39.42	102.7	103.2	98.6	99.7	97.9	98
8.82	29.6	102.4	101.1	100.1	99.8	96.1	93.1
6.54	35.32	95.5	87.2	86	98.1	98.5	99.9
5.76	32.79	127.1	126.6	129.4	69.3	71.9	71.7
7.08	27.54	97.7	100.9	101	109.6	110.2	113
7.24	28.52	100.1	100.4	97.5	103.5	102	103.1
6.11	36.96	109.5	105.3	111	102.7	102.7	102.8
7.9	38.97	103.4	99.8	110.4	71.1	80.9	87.3
8.43	29.23	93.6	98.6	97.8	104.9	106.7	103.7
9.76	49.29	104.7	102.2	99.1	105.4	104	103.7
6.79	31.39	101.9	103.3	103.8	104.3	106.9	106.8
8.21	26.56	94.6	95.1	94.1	109.6	105.5	105.7
6.71	67.21	98	97.7	99.6	95.7	98.2	96.8
6.16	44.16	104.4	102.6	104	106.6	103.3	104.8
7.4	48.71	99.6	101.2	98	101.7	102.2	99.7
7.88	25.82	96.4	95.5	90.3	98.2	100.7	96.7
6.74	30.38	96.9	93	95	101.1	101.6	100.6
5.96	20.87	94.6	99.7	103.4	97.5	101.6	102
5.33	28.87	98.1	100.2	97.9	98.6	97.4	100.6
5.4	32.35	99.2	100.4	100.2	106.2	97.9	99.2
6.89	26.19	106.3	101.1	98.3	103	98.2	99.5
6.87	28.35	98.8	100.5	96.4	96	93.8	91.8
4.84	36.11	95	101.1	99.4	114.1	113.5	113.7
7.43	28.99	96	91.7	92.5	100.4	101.8	99.4
5.48	33.9	105	99.6	102.8	110.7	105.9	105.4
6.42	26.88	105.7	100.1	97.6	108.2	103.5	104.2
5.53	30.78	100.3	101.9	98.2	101.1	106.6	103.3
6.84	29.83	83.7	83.5	82.3	100.2	95.1	98.9
9.25	28.37	111.9	111.4	107.7	99	99	98.1
9	27.52	101.2	99.6	102	96.1	119.9	99
5.71	21.05	124.1	106.5	112	80.9	78.9	86.8
5.71	25.34	104.5	97.9	97.8	97.2	98.7	95.9

7.88	24.1	99.3	97.7	100.6	116.1	107.1	116.8
11.05	23.47	100.2	102.4	112.3	106.3	106.3	112.2
9.17	37.2	93.6	100.6	98.8	98.3	95.7	100
6.14	30.11	99.6	95.6	98.7	114	111.8	109.7
6	31.18	92	88.9	93.4	98.3	105.3	104
9.91	84.14	103.8	106.2	103.2	104.9	105.1	103.2
9.16	57.33	95.7	98	96.7	90.5	91.9	92.6
4.84	27.73	98.8	97.3	97.4	101.5	105.4	106.5
4.94	41.12	102.4	95.1	91.7	100	98.6	106
6.68	38.73	100	101.2	99.3	102.5	102.7	102.9
7.99	42.76	102.9	104	98.1	105.5	100.8	104.1
5.97	48.23	102.7	96.7	98.5	100.8	101.1	106.7
6.43	30.1	105.6	98.2	101.7	113.9	102	108.9
9.22	27.27	107.7	94.5	109.9	109.4	116.6	109.9
4.4	28.69	87.8	112.4	98.9	105.7	113.8	98.9
9.13	36.02	102.9	102.7	96.8	96.7	96.3	93.4
5.76	30.11	100.1	98.7	100	112.1	108.8	110.3
6.44	21.11	95.8	102.3	94.5	97.8	100.2	99.5
6.3	27.08	97.2	110.5	92.3	70.5	65.1	78.7
8	28.61	99.4	94.8	90.6	101.4	103.5	104
5.73	28.24	109.6	98.5	97.1	94	103.3	98.1
9.17	34.19	101.6	99.5	105.1	99.3	100.5	103
4.77	26.37	99.7	96.8	94.3	110.9	110.9	110.3
9.16	30.71	101.6	104.5	105.1	96.1	97.6	100.7
7.3	42.9	93.4	91.2	90.8	105.4	104.2	104
6.61	33.71	107.3	102	102.3	102.5	104.6	104.8
6.9	31.86	100.6	96.7	100.1	103.8	104.9	105.5
6.52	27.16	101.7	99.6	111.8	103.3	104.8	101.2
8.18	35.25	95.4	96.4	99.1	100.7	102.4	100.9
4.96	48.54	94.9	93.5	94.8	102.6	100.4	100.4
5.9	26.58	99.8	96.1	95.2	100	101.1	99.8
8.06	26.46	96.6	98	94.7	103.3	104.4	103.7
6.92	38.1	98.3	96.6	97.5	101.3	97.7	102.1
5.01	29.53	95.8	95	98.5	97.1	100.7	103.1
5.06	24.91	97.9	100.9	100	101.4	106.3	104.1
5.22	33.44	103.3	100.1	97.8	109.4	101.6	108
9.28	26.42	94.6	99.3	95	106.7	102.7	103.2
9.94	55.54	101.3	100.9	103.2	102.9	103.6	106.9
8.68	49.26	84.1	82.7	85.1	111.9	115.1	114.2
6.87	26.73	104.9	103.2	101.3	107.4	107.5	109.4
7.49	19.93	104.4	105.9	100	94.8	89.4	96.8
6.99	30.4	96.2	103.4	93.8	92.2	85.8	87.4
9.67	27.91	102.1	102.3	98.6	110.1	110.1	109.8
7.78	46	97.9	98.7	96.2	105.1	104.7	103.2
9.44	30.09	109.3	102.4	107.2	106.8	107.3	110.2
8.32	37.35	105	105.9	102.9	110.7	109.8	109.1
9.16	26.5	105.2	96.7	102	103.6	112.4	106.4
5.58	25.46	101.9	103.5	98.9	101.1	104.9	99.7
5.67	24.35	101.5	98.1	94.9	110	107.6	109.7
5.83	30.64	90.4	87.7	110.9	102.2	133.7	114.1
5.57	36.95	98.5	94.5	97.8	89.5	88.4	89.3

9.04	27.05	102.4	96	98.4	114.3	115.3	108.4
10.55	40.28	97.6	103.2	101.7	133.4	132.8	127.4
6.76	26.93	97.3	98.8	99.3	90	90.2	93
7.71	34.32	107.8	102.1	104.4	126.9	129.3	120.6
5.24	24.86	100.8	102.3	102.8	85.2	93.2	94.8
4.91	25.85	99.7	95.2	99.4	102.6	102.1	109.4
4.81	21.32	88.7	95	98.6	102	118.1	117.3
8.09	60.98	101.6	107.2	95.2	95.2	89.6	99.5
8.02	29.51	113	103.6	103.9	98.7	103.2	97.6
8.98	29.87	101.4	100.8	101.6	108.1	107.3	108.4
10.32	46.2	104.1	96.6	103.5	104.7	104.7	108.6
5.53	30.92	105.4	109.3	108.7	102.9	99.2	100.7
8.07	25.99	100.8	96.8	95.5	107	104.9	104.8
7.8	28.72	102.6	95.7	98.2	94.2	91.7	95.9
5.95	22.46	104.8	103.6	105	93.4	88.4	96.1
8.29	33.31	102.6	101.8	104.4	107.8	108.7	111.2
6.44	22.69	103.9	104.9	99	105.2	104.4	102.3
8.54	49.61	95.2	99.2	100.4	98.7	98.4	101.3
8.18	42.55	102.2	96.8	98.9	101.1	98.4	98.3
7.77	27.01	102.1	96.1	99.9	101.3	97.1	92.6
7.42	25.68	101.6	97.4	97.7	96.5	96.4	97.3
6.74	25.29	102	100.8	96	97.1	98.5	110.9
7.39	31.59	98.2	95.8	97.5	108	99.1	106.3
10.74	63.59	110.6	104.8	119.3	101.3	116.9	107.8
5.86	27.63	89.6	88.9	89.2	95.2	94.1	91.2
6.38	26.02	92.8	92.6	90.1	99.8	100.6	102.4
4.63	27.47	101.2	97.1	100.9	106.5	103.3	104.8
8.4	48.28	105.9	104.5	105.7	90.9	92.3	91.3
5.26	24.2	99.4	96.5	98.8	98.2	96.1	106.5
8.05	28.42	87	77.7	124.4	89.8	109.7	95.2
7.3	30.6	92.8	93.1	96.5	100.5	102.1	101.8
7.39	28.37	105.6	102.6	106	125	129.5	122.1
6.96	36.22	100.2	99.7	99.1	97.3	96.7	94.7
5.17	48.03	99.2	102	99.4	102.7	106.6	107.5
5.33	23.31	99.6	96.2	98.7	101.2	106.3	105.1
8.48	19.39	100.1	103	103.5	109.2	106.1	109.2
6.95	26.96	100.2	106.3	96.4	104.8	102.5	105.1
6.47	19.7	97.1	92.5	97.3	102.9	105.6	110.1
4.91	48.29	112	110.2	124.7	94.1	88.7	91.3
9.66	36.9	96.5	96.6	97	104.8	108.2	108.2
5.4	42.57	94.4	96.8	93.5	114.9	113	114.6
9.91	24.83	108	108.6	100.1	111.6	105.3	102.9
6.28	23.13	103.8	114.3	100.5	103.3	108.6	107.5
7.46	37.33	108.2	106.5	106.5	91.4	96.3	95.5
8.59	34.02	95.6	97.6	94.5	99.4	96.6	98.1
5.52	37.42	94.1	94	93.8	103.2	101.3	105.4
10.01	26.06	104.1	103.8	102.2	106.4	105.9	103.5
4.94	29.09	106.8	88.7	101.8	102.5	111.4	104.8
8.31	27.28	96	99.1	99	96.3	97.4	94.4
5.27	29.82	99.1	98.4	92.6	88.2	88.6	89.6
6.67	19.24	98.6	97	100.6	101.4	100.1	101.5

4.84	26.98	97.1	99.5	100	100.5	105.4	105.2
5.54	25.95	102.6	101.5	100	98	97.7	98.4
6.11	36.52	101.3	99.4	92.4	98.9	98.4	102.5
8.41	24.38	96.8	99.8	98.7	100	99	96
6.74	27.26	104.6	102.8	105.3	82.6	81.4	85.9
7.39	27.11	103.7	104.4	100.6	96.5	94.9	95.8
6.11	36.09	101.4	100.5	94.8	104.5	102.8	105.9
5.07	26.12	96.9	97.9	100.4	89.6	97.8	100
10.58	56.17	113.3	110	108	111.4	110.3	110.6
7.44	19.46	93.5	97.2	94.6	116.3	110.4	109
5.33	21.74	100.8	100.7	102.7	100.3	97.7	97.6
7.78	33.64	94.4	95.8	97.2	101.2	103.5	102.6
6.9	26.33	97.6	95.6	95.9	104.6	92.9	101.9
8.09	22.88	101.2	96.9	93.4	104.2	105.1	104.3
8.38	24.31	112.4	110.5	110.7	108.4	111.2	112.9
8.44	23.9	105.4	102.5	100.4	108.5	105.1	104
9.07	35.05	97.2	96.7	102.5	101.3	103.4	98.3
8.1	28.25	103	99.4	100.1	99.1	100.7	98.9
8.1	20.7	103.1	99.2	101.8	107.1	108.3	106.9
8.53	34.19	95.4	97.3	99.5	102.3	100.8	100.6
6.58	26.05	92.2	90	90.9	97.9	89.7	97.9
7.78	37.59	100.2	96.1	102.5	96.3	101.4	100.9
7.78	31.04	95.7	95.4	101.8	104.2	106.9	103.1
5.66	33.38	89	95.6	89.2	119	114.9	121.8
7.5	23.59	99.3	99.9	104.4	93.1	91.4	94.1
5.73	21.16	99.1	96.6	98.6	101.9	98.4	99.2
3.85	22.81	102.6	113.3	111.3	105	94.1	98.8
8.56	26.15	99	101.3	94.6	100.5	99.4	101
8.56	24.72	105.9	97.6	102.3	100.5	102.4	107.5
7.87	18.26	102.3	100.7	101.8	109.6	107.4	108.8
6.44	19.45	96.8	93.5	97.5	105	105.4	104
4.79	26.38	105.9	100.5	104	103.6	101.2	103
6.89	21.29	93.7	97.8	100	95.6	94.4	96.9
9.36	26.65	95.1	94.8	94.8	97.7	97.6	98.7
6.1	20.9	99.6	99.2	95.2	98.8	97.7	100.4
8.29	24.14	101	98.8	104.8	102.2	113.6	109.6
6.96	31.27	105.3	102.5	104.9	104	104.3	103.8
6.29	29.41	108.6	102.3	98.8	98.8	95.2	96.5
6.77	36.26	100.1	99.3	101.2	106.8	102.3	104
8.38	23.31	99.4	103.8	97.2	102.7	109.6	99.6
8.12	24.21	93.8	98.4	95.7	110.4	105.7	106.2
6.29	24.26	107	105.5	111.4	86.1	79.7	81.4
6.71	24.94	96.3	98.1	98.9	101.1	97.5	100.9
5.85	20.95	100.9	102.7	105.4	91.5	95.2	96.7
6.7	24.97	112.9	100.1	103.9	101.7	99	100.2
9.45	25.75	105.2	104.6	107.2	95.3	96.9	96.1
6.29	25.3	114.4	111.7	114	90.5	81.5	80.6
5.5	21.78	97.9	105	101.3	103.5	104.9	108.4
7.14	29.98	105.2	105.5	104.7	102.9	101.7	108.4
4.7	23.13	108.6	106	112.1	107.3	102.5	97.7
8.82	27.13	102.2	93.8	104.7	100.6	96.6	100.8

5.71	23.35	95.4	91.2	92.2	104.9	105	104.4
11.05	49.99	106.9	98.1	101	105.1	110.6	104.8
4.7	34.44	94.3	92.9	94.5	107.1	108.4	106.8
7.52	26.79	130.3	128.6	124.5	77.5	73.1	74.7
5.31	20.49	96.7	86.9	95.9	93.4	92.2	96
5.12	26.74	99.5	98.8	98.4	95	96	96.2
9.36	33.7	102.3	100	103.2	99.1	100.7	101.4
7.4	23.65	94.2	96.1	95.8	94	95.6	96.4
10.43	42.86	102.8	101	99.9	97.4	96.7	98.7
7.88	27.99	99	102.8	100.5	102.9	102	99.2
7.25	21.64	96.1	91.6	95.6	101	97.5	98.8
6.28	33.01	97.9	95.2	98.8	97.3	100	101.8
5.01	31.38	94.9	99	97.7	97.4	96.1	101.7
8.4	26.95	101.8	100.4	95.1	106.7	106.3	102.9
6.68	41.51	97	98.4	92.7	102.8	99.8	99.8
6.29	25.21	99.4	94	99.8	102	99.9	106.8
6.1	25.79	101.2	98.9	99.6	102.4	96.9	97.9
5.06	25.15	111.1	107.9	109.3	61.5	59	62.8
7.8	23.57	95.3	94.8	97	96.2	97.1	98.8
5.74	23.43	101.3	103	97.2	97.5	97.4	96.4
6.21	24.43	98.2	95.4	101.8	101.9	97	103.8
7.47	24.37	105	101.4	102.6	103	102.3	107
7.49	31.6	101.7	100.3	104.1	109.2	110	110.9
6.96	22.73	93.2	87.8	88	103.9	106.2	105.6
6.25	25.11	100	100.3	100.4	98.6	98.4	98.3
6.24	19.99	103.8	97.6	96	103.5	107.3	102.4
6.54	23.54	87.9	83.3	89	107.9	111.8	106.9
6.55	24.14	102.6	95.1	100.2	94.2	90.1	95.1
11.47	66.31	102.6	100.9	97.4	105.3	107.8	102.8
8.56	25.51	99.9	106.5	107.2	109.3	102.6	106.6
5.88	23.21	90	89.8	93.6	106.7	104.2	112.9
11.59	28.59	101.9	98.1	104.5	111.7	106	107.9
6.55	29	110.2	106	107.3	88.6	89	92.2
8.4	22.53	94.7	102.1	97.8	121.8	120.7	119.2
8.12	36.26	102.3	100	98.3	105.6	104.3	102.1
7.31	21.38	96.2	99.5	100.1	102.8	99.4	111.6
5.2	21.76	105.7	99.3	98.2	98.1	97.2	100.7
6.8	24.62	95.3	97.3	97.2	93.6	93.7	96.5
6.37	20.71	94.2	96.2	97	107.4	99	111.5
5.74	18.92	101	99.7	99.1	103.7	105.5	108.8
9.13	26.05	98.9	97.8	99.8	112.5	108.7	107.8
9.45	21.81	111.2	109.7	111.9	120.4	118.8	117.6
8.72	36.73	63.1	60.2	58.7	112	110.2	113
5.77	26.54	100.3	103.1	101.7	99.4	99.3	100.7
5.76	29.46	101.5	106	102	100.1	101.1	104.7
5.67	22.8	100.8	96.9	97.9	99.5	95.4	97.9
4.54	52.39	102.3	101.1	99.6	110.6	107	109.1
7.37	66.43	107.8	110.8	107.4	96.5	98.2	96.2
11	38.99	100.2	101.1	102.6	105	112.1	106.3
8.79	25.61	103.1	102	106.7	101	96.7	97
6.49	19.72	99.5	93.2	100.9	101.8	97.9	100.6

4.98	34.1	98.5	101.3	100.6	105.8	107.3	108
6.29	24.53	103.2	100	96.9	99.3	96.9	101.9
5.66	24.26	100.5	96.5	103.9	106.3	107.4	108.7
6.14	25.26	95.1	90.2	94	99.6	104.7	101.9
5.52	29.77	98	102.9	99.2	101.2	97.6	96.8
4.82	21.76	94.9	101.6	91	117.1	118.6	113.8
8.31	22.73	111.1	115.4	119.1	61.7	70	63.5
6.57	25.03	105.4	103	98.2	93.6	93.2	92.6
5.58	29.44	98.6	100.5	96.5	101.7	104.5	110.9
4.92	19.59	104.3	98.2	100.6	107.3	100.7	109.2
8.79	33.11	98.3	96.9	94	104.7	101	101.5
7.14	22.4	104.5	99.4	100.5	104.7	102.4	105.5
6.27	22.99	102.5	106.2	104.5	95.7	97.3	97.7
6.16	44.92	105.9	106.2	105.3	89.4	88.8	89.2
6.15	21.42	90.1	96.1	93.4	107.1	100.5	104.2
8.47	38.95	98.9	95.3	93.2	103	102.8	99.3
7.11	33.55	99.4	100.7	100.1	98	101.8	99.8
6.44	26.8	97.1	101.1	98.9	101.2	98.4	100.2
7.88	23.58	103.8	100.5	103.1	84.1	90.8	91.9
10.01	29.44	100.4	100.9	107.2	111.9	110.9	109.6
7.28	50.3	68	67.3	69.6	131	125.4	124
10.23	19.04	102.2	93.9	102.5	103.8	115.5	109.3
8.47	27.95	105.7	108	105.9	107.2	109.2	108.1
5.76	19	104.7	98	104.9	98.2	95.5	97.1
9.83	29.12	102.5	96.4	104	109.6	108.8	110.1
8.51	22.12	109.5	102.3	107.2	98.6	101.7	104.3
7.03	29.74	129.7	132.7	128.3	50.8	50.8	50.2
4.58	36.43	104.9	99.3	98.9	106.6	104.7	103.8
10.1	22.69	102.9	102	100.1	108	110	105.6
5.62	23.53	102.9	104.5	99.1	100.1	97.8	100.6
8	22.9	105.2	109.4	104.1	106	99.4	97.1
5.94	18.27	98.6	97.8	101.6	93.9	101.7	113.1
5.55	20.65	106.6	103.1	97.6	98.1	105.3	102.9
8.63	20.34	105.3	99.5	100.5	101.5	101.6	110.5
11.66	47.99	98.1	100.4	103.3	126.2	124.3	123
8.82	20.01	104.5	106.4	106.7	95.5	94.7	91.1
6.4	30.43	102.9	93.9	88.7	105	88.7	108.3
8.76	30.85	98.6	106.4	104	99.7	93.1	93.9
5.83	36.6	102.7	102.5	101.6	98.7	97.1	98.1
8.15	48.1	88.7	127.4	109.5	82.9	76.3	108.3
7.85	26	106	106.1	104.2	112.4	110.3	112
6.68	24.57	96.7	96.4	97.6	101.9	99.7	97.6
10.24	23.94	114.2	110.8	98.9	88.5	95.7	97.3
8.19	26.99	99.2	99.2	101.4	96.9	94.2	96.2
7.14	18.44	101.4	96.6	98	104.2	106.6	103.3
8.7	32.62	90.6	95.3	95.5	92.8	96.8	97.6
7.46	23.06	96.1	95.3	98.7	102.1	105.3	101.7
5.01	43.23	140.9	145.7	138.8	71	63.9	69.4
9.09	33.85	97.8	90.2	93.6	87	87.5	87.3
5.83	20.13	105.1	98.3	102.1	101	100.6	104.6
6.02	24.18	96.5	96.9	95.5	96.2	95.6	100.6

8.27	32.55	103.5	101.5	105.4	109.2	101.2	105.7
7.34	25.85	105.5	98.7	103.1	95.8	97.3	97.6
11.06	51.95	102.2	99.6	104.9	99.7	109.4	103.8
5.26	22.06	100	102	100.9	94.8	94	95.3
6.38	13.37	101	101.9	111.9	105.7	107.9	105.4
6.76	24.93	100.6	97.8	96.4	101.9	100.2	101.3
5.5	29.34	97.3	96.8	95.6	117.2	115.3	114.5
6.62	22.56	99	108.1	99.6	101	100.5	99.4
6.34	25.53	94.1	90.7	95.8	102.7	104	110
5.91	32.43	93.2	97.5	92.4	100.6	99.5	100.6
6.7	22.52	95.2	89.1	97.3	95.1	102	101.8
5.77	24.18	85.5	83.2	86	101.4	101.6	100.6
6.81	33.09	103.8	100.4	99.4	99.7	99.6	97.1
5.49	21.87	102.7	106.8	109.3	99.4	99.4	98.9
6.46	25.2	106.4	99.2	94.1	94.7	96.3	100
8.25	18.77	97.7	93.4	97.4	101.8	106.5	113
7.11	26.9	97.6	102.1	94.5	92.5	93.1	93.6
9.6	26.01	97	98.5	99.5	107.2	101.3	104.3
6.55	33.23	99.6	106.6	101	96.8	99.8	100
6.35	18.14	95.4	97.2	93.8	101.2	95.1	93.5
6.83	22.22	111.6	103.2	101.5	104.8	105	108.1
4.78	27.93	103.8	100.8	95.8	103.1	102	103.2
6.74	26.67	96.9	95.5	94.5	103.8	99.6	101.2
8.66	27.71	100.2	97.4	97.6	89.7	97.9	116.5
7.39	23.02	101.9	108.4	108.5	97.2	101.7	100.3
5.47	28.66	102.6	100	100.7	97.5	97.9	99.1
7.55	18.29	106.5	106.6	105.5	100.9	99.6	98.9
9.51	22.97	102.3	100.2	101.4	105.5	105.2	102.6
6.62	28.78	150.9	152.2	152.1	62.2	63.5	64.1
5.94	21.18	85	82.6	77.9	87	89.3	87.5
5.97	37.29	102.9	103.5	103.2	99.2	99.6	99
5.21	19.93	94.8	98.2	98.1	97.8	96.9	102.4
5.07	20.31	96.7	87.7	112.5	94.9	115.2	101.1
5.22	24.39	93.1	94.3	99.9	103.7	130.5	109.3
4.49	33.5	105.1	103.2	101.4	98.5	102.4	98.6
7.25	22.32	100.5	95.3	101.8	109.5	105.9	108.7
7.99	19.9	112.7	115.1	103.1	111.8	109.7	108.3
6.86	35.29	89.1	92.3	90.4	99.2	97.6	97.6
7.99	23.27	100.4	101.2	101.5	95	95.4	97.4
8.75	25.48	100.7	95.8	100.2	108.9	119.4	111.1
7.56	39.14	98.4	103.2	102.8	91.4	93.5	95.1
7.56	23.37	95.1	98.4	96.4	97.7	102.5	98.4
8.5	29.73	69.1	67.5	65.7	88.6	90.1	86.9
5.58	22.22	98.1	99.7	97.6	99	102.9	95.9
5.97	22.01	104.2	102.3	100.3	109.2	111.8	108.6
5.66	31.93	103.7	100.8	111.2	95.3	110.5	102.3
6.92	26.72	90.1	95.1	99.6	98.3	96.1	99.1
7.39	22.68	100.2	99.3	100.9	100	100.6	96.4
7.43	41.93	100.4	93.4	94.6	101.1	105	101.5
7.68	18.22	97.2	100	96.4	95.1	94.1	99.3
6.55	32.01	97.6	98.6	91.3	103.5	100.4	104.5

6.92	25.85	103.8	105.1	102.8	93.4	92.2	97.1
10.89	43.85	117.1	114.9	118.8	106.1	99.8	103.7
6.9	19.6	100	93.4	98.4	100	96.4	104.8
4.83	19.82	106.7	104.4	103.7	95.7	94.6	97.6
8.65	17.54	106.3	117.8	111.4	100	98.5	100.8
6.28	30.1	94.7	94.1	95	98.8	99	101.4
8.5	24.61	101.1	101.4	100.6	96.9	98.1	102.9
7.21	26.2	99.4	99.9	96.9	100.8	112.8	104.3
5.01	23.88	102.4	106.2	103.6	104.9	101.6	101.3
7.42	16.38	100.8	96.7	103	112.3	106.2	101.3
9.8	54.17	102.9	102.4	101.9	102.6	102.8	100
5.86	23.29	111.2	112.4	107.3	115.7	115.9	115.1
6.34	27.59	102.5	101.9	98.3	103.3	98.3	99
8.5	18.39	89.7	95	95.8	105.7	106.8	109.9
5.53	22.49	104	98.5	99.9	98.5	100.7	100.6
6.99	20.08	94	94.6	97.5	100.4	100.2	106.3
6.52	28.99	97.7	94.5	98.7	93.1	92.4	101.1
5.52	35.24	101.8	102.2	101.1	99.7	96	97.1
7.18	26.13	94.2	96	92.7	99.3	103.5	98.7
4.78	23.08	104.3	102.8	94.7	103.7	98.3	104.1
5.31	21.63	94.7	98.4	98.4	108.9	109.6	109.1
8	13.98	103.9	101.1	107.4	99.3	111.3	103.9
9.31	34.03	96	94.1	97.6	111	94.7	113.1
8.62	27.27	109.5	105.9	106.7	105.4	104.6	103.5
7.06	31.51	97.7	99.6	100.3	97.8	95.9	96.7
8.51	22.46	107	96.9	95.8	96.8	95.8	100.3
9.32	25.21	98.2	97.1	103	96	94.8	90.5
9.41	27.7	110.6	111.3	112.8	88.4	91.4	93.9
8.53	18.46	108.9	112.6	110	89.2	94.5	92.8
7.64	16.32	74.2	74.4	75.2	105.3	98.4	102.4
5.49	19.97	97.9	96.9	97.5	95.9	92.6	99.2
6.83	19.83	108.6	96.2	102.5	93.4	106.7	95.8
8.97	24.3	98.9	105.8	101.2	101.6	105.6	107.4
7.75	24.81	100.6	100.1	100.2	98.8	96.9	96.4
5.53	20.35	80.6	78.7	85.7	126.7	122.1	121.1
5.76	19.1	97.9	102.7	103.5	110.6	103.9	109
6.55	22.85	99.6	96.7	97.2	103.5	101.3	104.3
8	21.29	104.3	104.5	103.4	96.9	103.4	96.4
6.3	19.81	96.7	95.3	94.7	100.3	98.5	96.2
6.89	23.46	102.5	102.6	93.9	98.5	99.5	97.6
5.22	28.05	98.2	88.6	88.9	105.7	106.5	111.4
9.35	24.06	103.4	104.9	107.7	97.7	98	100.1
4.73	21.03	101.9	93.4	99.3	106.4	107.3	106
7.25	26.61	102.3	100.6	98	104.7	100.9	105.4
5.62	25	98	100.6	98.8	105.2	103	102.4
7.94	21.34	100.3	97.4	100.9	91.4	92.7	98.1
6.34	21.29	103.3	99.4	101.4	94.3	89.3	89.9
5.02	20.73	91.6	87.8	95.1	113	121.8	112.1
6.42	19.83	108.1	98.6	96.3	102.1	108.7	100.6
4.34	23.26	106.2	102.6	98.6	105.5	103.4	107.2
5.66	47.01	94.7	93.5	96.4	105.3	103.7	104.2

5.12	20.95	98.8	95.2	98.5	101.8	99.1	106.6
9.7	24.66	106.3	83.9	121.6	160.8	176.9	153.8
6.04	33.63	105.3	106.2	106.1	104.2	103.4	107.7
9.35	30.38	100.6	100.3	98.1	91	97.2	93.8
5.08	28.29	95.3	93.2	92.2	103.1	103.6	105.9
6.09	20.97	102.8	104	112.4	106.5	111.5	105.1
6.55	18.65	96.1	98.7	101.6	102.1	95.9	100.6
5.47	19.61	105.1	114.2	115.9	109.3	114.9	112.5
6.43	39.65	93.4	95.2	95.6	103	105	105.6
6.46	39.71	148.2	146.1	146.7	42.9	43.7	43.9
5.21	22.76	103.2	94.1	95	109.8	104.2	105.4
9.19	18.8	100.1	98.4	106.2	94.8	97.5	101.6
5.55	18.92	97.5	95.3	97.9	89.8	85.9	91.9
9.09	20.02	98.3	103.8	105.2	102.5	96.5	96
4.51	51.59	98.7	99.8	100.1	104.6	102.7	105.5
10.04	47.05	105.2	105.1	107.8	104.8	105.5	103.6
6.81	21.77	94.5	93.2	101.2	102.6	93.7	107.1
8.69	27.67	100	101.9	100.9	104.4	103.9	101.1
5.83	25.95	97.1	104.6	102.7	93.2	94.5	96
11.43	30.04	105.8	104.4	104.9	106.5	109.4	105.6
9.14	25.29	98.8	97.2	96.5	99.6	102.2	101.6
11.08	31.34	111.7	106	102.4	112.9	108.8	103.2
8.53	22.6	123.7	129.6	123.9	94.5	94.3	93.4
5.55	30.51	102.8	102.1	99	102.3	105.2	106.5
5.24	18.46	100.7	89.7	91.6	107.9	104.5	107.9
6.83	23.66	105.1	113.1	108.9	109.1	107.3	104.3
6.2	21.55	98.4	91.3	106.8	99.2	95.4	100.2
7.08	25.9	106.7	101.1	100.8	110.1	114.1	110.6
5.01	19.9	110.8	100.6	127.1	110.7	107.2	111.6
5.41	18.61	107.5	104.5	116	93.2	97.8	94.6
8.22	43.17	111.1	109.9	106.2	90.2	96	94.7
6.25	58.36	111.2	116.3	113.9	94.4	92.8	92.3
8.27	26.45	102	106.9	102.3	97.1	96.7	96.6
8.15	16.93	104.9	95	103.3	84.3	80.6	88.8
7.99	31.7	101.8	98.9	104.2	100.2	96.1	94.7
4.86	25.21	99.5	99.4	99.6	117.2	114.6	111.8
6.9	26.56	106.1	95.1	104.5	97.7	91.1	104.1
7.4	22.15	95.7	92.1	100.8	99.9	101.6	100.9
6.3	18.7	100.7	103.5	96.3	104.6	99.4	108.7
7.55	20.71	94.8	93.2	111.3	105.5	98.9	102.1
6.6	18.61	98.7	101	107.4	108.9	103.3	103.3
7.96	20.12	94.9	96.1	96.8	102.2	102.8	105.9
6.54	17.62	94.1	88.6	94.3	92.8	99.9	88.6
8.62	22.64	108.2	101.7	98.7	101.6	103.7	98
6.79	24.44	98.9	95.4	105.2	105.3	104.8	103.2
8.94	29.28	94.4	96.4	96.5	108.7	105.7	102.2
6.32	19.54	97.1	97.6	96.2	97.1	89.4	90.1
8.35	34.34	107.3	100	104.4	96.3	101.4	97.6
7.72	21.88	102	100.9	100.7	101.4	100.9	100.5
8.78	27.28	109.5	107.5	108.2	106.6	104.9	106.1
6.79	18.82	97.1	96.1	95.5	94.7	89.9	92.8

6.65	24.33	86.7	105	107.2	90.3	97.7	99.2
6.65	21.08	90	85.3	85.6	103.6	101.6	97.6
10.43	44.08	99.3	98	100.1	104.2	100.9	104.7
6.62	23.37	95	99.7	97.9	107.4	109.7	111.2
5.57	20.49	98.5	94.4	91.2	103.2	90.9	106.2
5.06	18.03	90.8	88.6	89.6	104.5	109	109.2
8.12	23.26	102.5	100.7	99.8	106	114.6	113
4.81	30.26	96.7	101.9	104.9	106.7	102.6	97
6.77	27.91	96.3	105.7	102.4	104.2	100.3	95.7
6.42	12.41	101	97.4	96.3	106.3	109.2	99.9
5.82	25.29	104	99.7	95.9	93.9	99.9	94.7
8.63	20.6	93.4	100.1	99.8	96.5	99.1	98.2
4.92	49.75	96.7	95.6	95.9	102.3	102.7	100.8
5.63	21.46	104.7	105.2	104	103	96.3	98.5
5.44	16.35	93.2	96.3	89.3	106.9	108.5	112.2
9.36	21.46	97.3	96.5	95.5	94.2	91.3	96.4
5.21	22.64	94.2	93.9	95.1	100.2	104.9	103
7.09	19.87	89.6	99.7	96	97.9	101.4	101.4
6.37	22.77	92.9	95.3	94.8	100.2	105.6	109.3
4.69	22.99	69.9	62.6	63.6	117.5	134.9	127.7
7.72	24.59	106.9	106.5	111.7	92.5	91.6	88.6
5.43	21.83	98.2	100.7	100.2	97.2	94.9	100.3
5.05	21.59	99.4	102.1	93.8	99.3	96.1	96.4
8.38	39.64	102.2	100.8	98.2	99.7	99.2	100.2
6.39	23.13	132.5	124	117.8	84.9	82.7	82.9
8.48	30.05	109.1	114.9	79.4	119.8	80	119.6
10.99	49.48	104	104	103.4	102.6	102	100.2
6.98	21.76	104.1	101.7	99.8	99.5	105.5	102.5
10.02	19.05	101.6	103.2	108	101.2	106.9	117.3
6.81	20.77	111.3	111	112.1	82.6	83.5	80.5
9.42	16.87	100.1	102.6	108.9	103.8	95.8	97.1
5.39	23.01	100.6	91.1	96.7	98.8	99.2	99
6.84	22.03	101	96.4	96.4	98.6	104	105.4
6.34	19.16	101.2	102.6	99.4	97.4	97.4	98.7
5.69	18.88	101.5	97.2	94.9	99.4	101	103.5
8	19.4	97.7	99.2	104.6	104.3	110	104.3
9.47	24.48	105.8	110	115.5	124.4	117.5	121.1
9.45	22.16	109.4	103	110.3	100.1	101.6	104.3
9.16	29.09	111.7	125.9	90.9	103.9	102.4	107.5
5.9	15.58	101.9	99.7	100.1	121.5	123.1	112.7
6.99	17.44	95.7	103.7	96.3	92.4	87.8	86.5
8.06	18.59	102.2	97.9	101.5	103.3	106.1	107.1
6.77	20.95	95.4	103.6	95.2	99.5	95.4	103
7.75	25.64	98.9	98.5	103.2	101.2	98.6	98.4
7.39	17.9	99.8	96.9	101.4	106.9	104.1	117
6.74	21.5	93.9	103.8	99.2	108.4	102	103.1
7.15	17.84	103.2	95.5	95.3	102.8	109.8	106.2
6.73	19.06	90.8	91.6	95.6	101.7	107.7	104.9
4.83	36.32	97.6	96.8	100.4	98.2	99.8	99.5
5.43	19.87	96.2	101.2	108.5	106.3	106.7	105.1
8.82	25.33	109.9	103.6	105.9	96.4	95.2	91.5

5.1	24.15	99.5	98.8	103.4	100.2	102.4	103
4.93	23.31	94.6	101.1	97.1	108.3	109.6	109.8
5.21	21.48	98	100	93.7	102.3	104.2	93.7
5.36	16.59	105	85.1	103.7	110.7	86.5	112.4
6.65	18.95	99.3	99	102.9	85.6	92.5	88.2
10.54	41.65	100.5	101	105.9	104.1	104.9	105.4
7.37	26.04	94.7	101.4	102	100.3	103	105.3
4.46	22.42	98.8	100.9	99.4	101.7	96	99.5
8.63	21.21	95	94	99.8	120.3	112.1	114.2
9.23	23.07	101.9	107	103.4	102.5	96.9	97.4
9.64	35.26	100.5	99.9	101.1	98.2	99.2	103.6
4.72	20.7	115.2	125.3	130.8	109.2	106.4	114.4
5.45	19.6	103.2	101	104.2	97.7	95.2	103.1
9.06	23.98	96.3	98.6	92.8	80	78.3	77
5.17	20.11	94.3	92.4	96.5	104.2	116.6	118.9
4.86	17.98	103.5	97.9	106	103.5	101.8	100.9
7.43	20.91	93.7	101	95.9	100.2	104.3	108.5
5.4	22.25	101.4	99.7	100.4	98.7	95.6	96
5.78	27.69	101.5	99.3	104.2	106.3	108.3	108.1
8.72	20.46	95.8	94.4	95.6	102.2	104.6	99.9
7.9	18.89	99.7	100.4	101.7	104.4	107.1	108.3
10.99	47.96	96.9	101.5	94.5	152.9	171.4	158.5
8.25	17.12	95.4	92.7	95.3	116.4	107.6	111.1
5.8	18.8	96.6	105.5	101.4	106.3	103.7	105.2
6.77	18.7	103.6	107.2	100.2	96.1	100.4	97.4
6.28	22.07	101.1	100.6	99.4	116.9	113.3	115.4
7.87	23.94	95.4	97	85.4	104.3	98.6	91.1
8.19	22.33	97.3	94.1	99.6	100.5	103	99.8
9.06	19.2	97.8	100.7	94.8	114.4	112.6	111.4
5.31	35.68	92.4	93.4	92.6	105	101.6	102.5
4.97	18.48	111	96.8	98.6	102.9	104.1	102
4.7	19.69	80.5	79.1	79	108.4	100.8	106.2
9.64	20.01	100.6	100.3	97.8	109.5	109.9	114.6
6.55	29.48	103.6	98.9	96.7	83.8	79.4	85
8.82	24.9	102.2	105.5	105	112	110.1	109.2
5.68	20.97	100.8	102.3	98.8	96.6	91.5	89.5
4.96	19.46	90.8	93	96.7	105.6	104.1	106.3
7.97	23.2	94	90.8	94.1	103.4	108.6	106.4
11.65	37.35	103.9	102.6	105	108.3	105	106.5
8.79	25.73	105.3	106.2	91.9	105.4	97.3	104.3
7.96	20.05	96.7	94.5	89	102.4	97.8	101
9.17	18.26	97.4	96.6	103.5	108.4	104.8	106.1
7.72	23.24	92.4	95.6	90.5	89.9	89.3	92.4
8.24	35.32	100.3	99.5	101.8	104.2	104	106.2
6.65	25.7	100.6	100.7	97.3	97.6	99.8	104.8
7.52	19.55	108.7	108.2	115.6	105	109.5	104.8
5.41	19.44	100.5	100.4	95.1	100.3	95.3	96.9
6.43	15.4	100.5	102.7	99.1	108.1	102	116.9
5.39	21.54	99.3	95.9	96.4	104	101.6	104.4
9.22	14.69	101.5	96	98.8	113.3	101.9	99.1
6.18	10.23	93.1	88.2	94.4	103.7	88.4	102.6

9.1	31.04	101.9	102.1	104.4	94.4	89.2	91.6
10.08	21.68	98	103.4	103.8	98.3	100.9	97.7
6.35	11.47	103.7	100	97.8	100.1	103.3	106
5.85	20.04	94.6	99.5	97.6	99.3	106	99.6
5.94	18.21	95.5	101.8	102.3	111.3	113.7	109.7
6.93	13.12	107.5	90.6	104.8	97.4	92.9	103.4
8.31	22.83	95.5	112.8	108.5	102.5	100.3	109.9
6.92	23.67	98.4	98.6	99.3	108.3	100.3	104.2
4.92	20.41	102.6	90.7	95.6	104.8	106.2	111.7
5.47	25.58	107.8	97.1	103.2	96.5	94.4	97.6
6.58	16.46	98	102.7	97.7	97.9	96.1	98.3
6.73	22.7	101.2	102.2	98.5	105.2	104.9	104
4.92	19.74	97.3	100.4	97.8	104.3	102.2	102.3
7.21	17.86	97.9	100.9	101.9	105.4	103.1	105.5
7.72	24.99	109.3	112.7	112.8	81.7	77.6	78.9
8.31	20.18	94.4	99.3	99.4	105.9	97.9	101.1
6.86	35.92	87.5	82.8	84.6	106.4	103.5	107.2
5.64	25.72	99.6	100.4	100.1	101.9	97.1	100.8
7.97	25.28	105.3	105.2	106.3	109.3	105.1	108
8.57	18.28	100.9	91.2	95.8	105.9	111.5	106.2
8.05	24.65	86.1	86.9	81.5	90	87.4	86.8
5.08	17.05	100.3	97.9	97.7	98.1	97.2	102.2
6.07	16.05	94.6	101.6	104.6	108.9	112.3	110.6
6.76	16.93	97.7	90.8	97.4	106.2	105.3	107.6
5.62	29.12	98.8	97.4	97.8	103.7	99.7	102
10.1	43.04	104.2	104.2	103.6	103.2	102.5	100.7
6.35	26.21	99.2	105.6	96.8	97.6	100.7	93.2
9.89	17.45	101.7	107.1	105.8	113.1	111.2	108.5
5.1	15.9	95.8	97.3	100.4	101.3	98.3	100.6
7.33	13.37	95.4	101.8	94.9	104.3	100.9	102.5
6.55	16.8	95.7	99.2	98	101.6	102.9	103.8
6.37	17.49	98.9	93.5	99.7	101.5	103.4	110
7.88	15.76	112.8	109	108	87.9	88.8	86.7
6.64	19.28	99.4	98.5	101.2	112.5	107.2	112.8
9.1	17.41	102.6	100.5	105.5	103	104.6	110.8
6.43	20.26	100.1	101	101.7	109.9	99.3	107.6
10.11	11.47	103.6	110	106.6	112.4	116.7	119.3
6.33	13.19	103.7	98.7	97.3	96.5	107.4	95.9
7.09	17.28	114.7	102.7	104.8	99	94.4	96
5.52	18.69	98.6	98.4	94	79.3	78	81
5.69	27.24	99.2	98.6	102.4	98.9	98.9	101.6
6.02	14.2	112.8	111.4	116.6	109.8	113.4	109.8
9.01	18.99	106.7	98.6	96.8	109.1	108.5	102.5
6.18	16.71	101.5	92.6	91.9	85.8	101.9	101.5
7.25	15.86	104	91.7	96.7	89.5	85.1	95.9
9.28	17.06	100.3	99.7	106.5	105.6	112.7	113.3
6.95	22.23	97.4	98.4	103.6	93.5	96.6	100.8
6.02	22.26	101.4	101.6	105.4	96	96.3	91
7.11	24.26	106.1	107.6	105.6	86.2	87.7	89.6
5.92	21.99	100.5	98.6	100.9	96.1	93.3	96.3
4.83	22.24	109.2	103.1	101.6	59.4	65.9	60.7

8.78	22.96	94.2	108.5	90.3	90.7	96.8	98.3
9.33	18.33	104	104.6	100	100.7	103.1	107.9
8.05	17.63	96.4	94	94.2	97.3	95.3	98.4
6.79	15.78	101.2	99.4	98.4	104.7	112.7	102.3
8.81	30.46	150	98.1	118.4	104.5	92.8	120.6
6.48	17.84	94.5	98	98.2	94.5	102.8	94.9
5.36	19.48	100.8	97.8	100.1	100	98.4	101
5.22	19.5	92.7	95.8	105.6	100.7	101.2	96.6
6.7	23.27	104.8	124.2	103.7	106.7	102.7	119.9
5.07	23.02	96.3	97.3	99.3	109.2	103.3	102.9
9.45	14.97	105.6	98.6	105.4	109.8	109.8	110.6
9.64	16.54	107.5	106.3	113.1	107	129	114.7
7.3	12.3	101.3	104.1	103.5	107.6	106.7	115.3
6.99	31.45	100.8	106.1	107.6	91.6	94.4	96.8
6.8	17.37	93.5	100.6	107.7	110.9	110.5	109.8
6.46	26.92	99.4	97.6	99.5	102.4	105.5	104.3
7.2	14.61	99.6	83.7	124.5	101.1	97.3	133.3
4.72	27.21	107.3	98.4	100.9	105.7	106.2	105.3
4.81	23.7	100.6	100.6	103	100.3	97.6	107.2
9.36	23.28	105.4	98.7	96.1	102.7	103.5	106.2
6.01	18.68	121.8	115.9	125.9	92	99.9	98.3
5.94	12.28	134.6	91.3	87.8	94.8	123.3	95.1
11.62	26.07	102.6	111.2	100.3	99.6	103.6	105.6
6.02	31.49	99.4	98.5	98.2	94.7	94.2	96.7
5	12.58	103.1	102	100.5	107.3	99	102.2
6.8	15.28	104.3	107.6	102.3	103.6	100.8	95.7
7.05	14.89	116	101.6	114.7	88.2	98.8	93.2
6.15	25.82	107.6	95.4	101.2	101.3	102.5	106.8
6.61	15.34	103.7	95.5	97.1	94.6	93.5	97.1
7.15	16.24	88	92.1	88.6	109.9	102	106
6.64	15.73	100.4	94	99.4	100.7	100.3	106.8
7.18	17.31	104.6	104.5	105.4	106.5	107.9	102.8
6.07	23.82	98	97	96.6	101.2	100.7	99.2
9.36	19.32	99	92.6	103.5	102	108.5	104
7.2	12.96	92.5	98.9	95	98.1	121.3	116.8
4.91	19.11	104.3	93.5	108.6	101.8	116.8	99.8
7.14	15.5	110.6	108.4	117.9	80.4	79.5	83.6
8.27	22.81	103.2	94.6	90.7	95.1	102.1	100.7
5.38	26.72	95.6	98.7	102.6	98	97.4	97.8
8.69	15.28	100.1	91.2	97.8	108	100	104.1
6.43	13.73	86.9	83.9	81	103.9	102.4	99.6
8.16	19.86	101	95.6	94.8	105.7	94.5	102.4
5.36	16.79	92.6	97.7	96.4	106.5	99	102.7
8.73	17.87	97.4	91.1	101.4	102.5	105.1	98.6
8.27	16.54	101	100.5	97.2	104.1	94.4	95.2
9.61	34.6	115.7	98.9	93.2	97.6	103.2	103.2
8.4	15	92.8	96.9	95.7	113.3	107.1	114.6
7.11	21.55	98.4	88.6	106.4	96.4	115.2	103.2
6.99	16.27	110.3	119.3	114.9	102.8	107.1	101.5
5.02	22.97	95	102	94.7	105.4	102.3	103.2
6.55	14.23	99.5	105.8	101.5	99.6	100.8	112.8

7.09	19.25	105.3	102.2	104.3	97.3	107.2	97.6
10.65	37.49	111.2	102.3	95.5	100.6	106.8	97.1
5.86	17.6	108	88.7	97.1	92.2	103.6	98.2
4.34	17.48	96.8	99.4	94	107.9	113.2	105.6
8.47	18.63	106.4	102.5	103.5	99	104.7	108
9.92	20.82	103.4	102.7	102.2	112.6	109.6	108.9
7.34	17.11	99.3	107.1	106.3	114	105.9	103.7
7.23	17.81	86.8	89.1	89.9	116	119.7	119.2
9.72	18.25	102	104.6	108.4	104.9	101	107.7
4.67	34.36	102	101.2	104.2	127.8	126.1	123.4
7.46	22.7	97.5	99	96.2	105.1	102	104
4.93	19.79	86.8	95.8	90.8	113.7	108.2	111.7
6.05	24.09	96.1	95.9	98	101.2	102.1	105.8
5.82	15.09	98.5	100.2	102.1	98.1	105	103
4.44	15.13	100.5	106	101.1	97.3	98	92.8
6.55	19.27	100.6	97.6	96.3	94.6	96.9	95.5
6.2	17.42	100.3	98.1	96.7	98.6	97.7	98.1
9.23	20.75	100.6	100	103.3	106.8	100.4	104.3
6.92	16.27	93.1	89.6	89	98	104.8	113.8
5.8	20.37	104.5	100.5	102.3	97.1	99.1	93.2
8.07	18.96	108	106.8	107.5	97.7	100.2	102
5.02	18.31	111	107.5	107.5	118.5	103.6	114.9
7.28	20.67	95.6	95.4	93.8	93.7	97.9	97.7
5.76	14.69	101.7	89.3	98.9	96.2	92.3	93.5
4.98	19.36	106.4	94.4	95.8	110.9	101.6	104
8.29	30.57	102.9	97.6	101.5	102	96	105.6
8.57	15.65	108	129.9	82	99.6	102.3	86.7
6.84	19.81	104.7	99.8	98.1	102.8	100.1	104.2
5.45	19.59	99.4	99.5	102.9	88.9	100.7	99.7
5.35	21.8	101.6	102	108	101.7	96.6	99.6
6.73	21.86	99.8	97.3	105.5	84.6	89.1	86.6
5.25	15.79	106.8	105.9	100.5	96.7	97	97.5
7.37	15.68	95.2	100.2	98.1	101.1	104.1	97.3
6.87	10.28	94.4	99.4	86.5	99.1	99.6	96.6
7.06	14.8	90.9	101.9	97.8	96.9	104.3	97.6
7.31	17.68	104.1	89.5	109.7	109.4	79.8	117.6
6.38	17.52	99.1	90.8	98.4	105.6	103.4	104
6.93	14.58	99.1	105.1	107.5	96.1	96.3	103.5
5.22	16.71	99.8	100.5	99.6	100.3	103.2	97.8
8.46	27.56	101.6	105.7	102.5	103.7	104.7	100.5
6.64	18.12	106.8	102.4	100.1	99.8	93.1	94.1
8.09	17.65	102.6	94.9	102.9	97.4	93.2	105.8
5.06	19.12	83.9	87.2	87.4	96.4	92.2	171
4.81	22	106.8	101.8	104.3	96.3	96.8	95.6
5.05	19.39	101.7	95.6	102.1	105.6	110.9	110.8
6.43	22.8	92.5	95.4	97.4	99.5	97.9	105
9.16	21.61	99.8	97	93.9	113.6	111.9	110.1
7.28	18.88	100.9	106.7	103.2	111.4	103.5	106
5.16	18.8	95.5	100.6	97.8	103.3	97	98.9
8.51	16.9	100.6	98.9	97.7	93.5	98.4	97.6
7.33	16.07	95.1	95.7	93.1	101.5	106.1	102.6

7.02	16.62	101.8	103.4	102.5	102.4	100.4	107.2
6.32	19.02	111.1	100.3	103.2	104.9	117.6	110.5
7.37	17.41	99.9	104.2	104	98.2	105.1	100.2
5.66	15.87	103.9	99.4	106.8	99.3	96.2	94
5.76	15.86	94.9	100.2	101.3	96.7	96.7	97.3
7.12	20.91	80	74.9	74.8	120.7	118.1	120.4
5.1	18.85	103.7	97.7	102.3	101.1	98.5	98.7
7.66	18.01	93.6	96.9	93.1	98	101.2	100
6.13	11.8	96.2	99.4	99.5	110.6	106.2	110.8
6.96	20.03	99.5	97.3	98.3	99.2	103	107.7
10.1	46.01	91.9	82.1	86.2	95.2	91.4	97.7
5.82	13.24	102.7	97.7	110.6	102.2	102.4	103.9
11.19	22.27	106.5	95.1	101	105.2	105.9	107.5
7.06	15.94	105.7	102.4	102	102.1	108.9	117.4
6.96	18.86	95.1	90.3	98.7	98.9	93.5	101.9
6.44	23.08	100.2	103.3	100.8	105.6	110.6	107.2
6.84	20.02	88.3	92.2	92.4	94.8	95.3	94.8
8.15	18.39	98.4	92.3	87.3	109	110.8	116.9
7.99	19.73	99.9	98.5	96	95.1	91.6	90.6
6.19	14.61	114.9	123.9	112.3	74.1	67.7	76.4
5.69	17.41	104.9	102.4	104.2	109.7	107.1	107.9
6.35	17.47	107.5	106.4	105.3	97.9	93.5	96.5
11.27	55.37	106.2	114.6	116.7	104.8	102.2	101.4
8.53	14.4	92.5	91.2	93.4	83.1	82.6	85.2
4.94	19.44	100.8	92	97	98.2	99.4	101.4
9.45	16.1	100.2	104.9	102.3	108.5	102.7	104.8
5.27	17.37	100.6	93.3	92.6	98.9	104.9	104.7
8.72	14.84	107	108.4	105.9	97.1	100.3	99.4
6.73	24.23	99.5	100.6	94.2	101.1	104.9	101.3
5.78	13.6	96.5	90.3	92.3	114.1	133.2	110.3
5.41	23.1	99.2	97.8	94.5	100.7	102.2	102.9
6.64	14.64	97.4	99.5	108.4	107.3	94.3	103.5
5.44	11.97	96.8	100	100.6	95.7	96.2	92.6
5.54	19.6	102.3	99.8	103.9	105.4	110.1	106.5
6.14	18.6	91.8	90.9	87.8	86.9	84.7	91.5
6.61	23.18	104.9	98	106.7	101.9	101.6	102.1
8.54	22.18	94	95.7	97	100.4	97.2	98.4
4.88	22.87	99.9	98.8	96.5	105.7	99	101.3
5.05	14.26	100.8	105.6	103.9	105.3	100.6	99.9
5.99	19.51	100.9	98.5	99.5	101.6	104.2	107.2
7.05	22.44	102.1	95.1	101.8	96.1	93.7	95
11.84	20.88	101.6	98.4	105	117.3	121	121.7
6.7	15.8	96.3	91.4	107.5	96.6	91.9	98.3
8.88	18.84	106	102.4	103.6	105	108.3	104.8
7.02	19.28	89.1	97.6	100.1	106.5	106.5	106.2
9.17	25.76	102	104	104.8	111.9	104.3	113
5.49	16.74	109.2	101.1	105.3	106.4	93.8	104.5
6.8	17.56	94.7	102.2	98.4	103.2	100.5	108.9
7.05	29.05	102.4	99.3	102.3	112.4	106.1	106.6
6.93	18.17	95.7	95.5	99.6	99.5	108.4	106.2
10.26	17.18	105.1	105.2	103.4	104	105.2	103.1

4.82	16.55	95.6	104.2	101.6	95.9	91.8	88.6
5.81	17.55	92.7	94.9	90	101.6	101.9	106
5.81	14.21	101.2	93.5	95.1	103.2	98.7	98.3
4.73	16.74	91	97.3	100.2	105.5	107.9	105.3
6.64	12.68	104.8	104.6	104.5	109.8	106.6	105
5.03	17.23	102.7	99.1	102.4	102.8	100.4	101.9
6.81	16.89	113.3	89.5	96.8	122.1	102.3	118.4
7.31	15.36	121.2	125.9	104.2	85.1	83.5	81.1
5.69	21.29	107.2	102.8	100.5	105.7	104.1	104.7
6.73	24.13	97	98	99.6	104.9	100.1	94.7
4.59	26.73	93.5	90.8	100.3	104.1	104.5	106.7
6.48	17.78	107.1	97.9	97.8	87.6	93.9	87.4
7.43	17.68	103.4	102.4	99.5	94.5	97.6	94.2
7.34	9.67	102.4	98.2	99.5	111.1	106.5	106.8
4.98	14.33	101.5	95.7	95.9	92.6	103.5	91.4
7.83	28.06	86.9	84.5	87	92	92.6	91
7.31	16.95	95.1	99.9	99.6	108	93.2	98.3
9.29	24.05	106.7	102.2	99.8	110	112.1	114.1
8.1	25.24	94.7	96.3	91.6	100	98.1	98.2
6.32	15.97	91.8	95.2	94.9	104	104.4	102.5
6.86	20.67	97	96	103.2	103.9	103.8	106.4
6.42	18.14	97.3	96	97	105	98.9	106.2
6.8	15.98	93.9	94.2	90.8	102.9	97.3	95.5
6.73	15.34	96.9	97.1	93.7	114.3	109.4	107.3
4.93	20.43	97.9	97	95.8	115.6	105.5	110.9
6.62	15.35	101.9	95.9	100.9	87.2	86.5	94.8
6.07	15.7	102.2	94.4	99.5	101.4	102.6	106.3
7.42	17.67	103.4	109.3	101.6	112.2	109.4	113.2
5.64	17.29	103.9	99.6	104.2	113.3	104.9	105.3
6.18	15.53	105.2	102.6	106.2	101.7	106	111.1
9.31	34.98	92.5	93	99.6	100.2	101.3	106.3
5.07	14.72	98.2	99.8	105	94.1	98.5	100.7
6.35	14.97	97.8	99	100.7	102.5	99.4	105.5
4.92	10.15	90.1	83.6	81.2	103.2	106.1	105.9
6.65	16.93	94.9	103.4	95	98.3	103.1	102.2
4.96	15.2	96.7	100.2	85.9	106	109.3	100.8
8.46	25.34	107	102.8	106.5	104.3	100	104.5
6.7	15.24	101	95.8	97.5	103.8	102.1	102.8
7.77	15.06	99.4	105	103.3	98.4	95.2	100.5
6.61	26.78	94.7	94.9	98.7	96.4	94.7	93.6
8.24	13.15	94.2	102.2	93.2	97.3	110.4	109.2
5.3	17.12	104.9	101.7	104.6	100	104.9	106.5
7.17	13.7	98.8	100.6	99.5	99.7	99.4	102.4
5.3	22.66	101.3	103.5	99	98.3	98.3	101
6.84	13.94	102.3	103.9	97.5	96.1	102.9	105.8
9.33	18.55	95.9	96.3	93.7	82.4	79.7	83.8
8.47	15.27	92.3	98.4	89.4	109.8	108.4	110.1
5.5	14.97	94.1	95.4	97.1	105.6	102.8	93.7
9.99	19.8	100.3	99	95.3	105	103	102.7
7.11	14.78	95	98.1	97.9	101.8	104	102.4
8.82	29.04	94.2	98.3	100.2	100.7	105.6	102.9

8.65	21.02	100.7	101.1	99.9	105	99.3	102.2
6.34	18.91	106.4	96.7	104.8	101.5	102.5	100.7
8.24	25.38	102.4	100.6	100.4	102.4	100.1	101
7.25	14.93	114	100.1	91.7	89.7	90.1	90.2
8.38	16.56	112.7	99.9	99.7	83.5	86.1	89.9
6.95	20.43	105.5	102.2	103.3	90.2	89.1	87.1
7.94	15.92	101.8	102.1	96.9	98.1	92.7	95.1
10.3	17.93	100.5	98.3	104	103.8	101.6	99
6.23	17.05	106	96.5	100.8	111.7	109.3	115.4
5.14	10.77	100	101.3	113.4	98.1	91.9	88.3
7.37	15	100.1	100.1	105.1	93.6	100.4	94.8
6.46	16.74	96.1	92.6	99.5	96.6	98.2	99.1
6.54	16.98	99.6	93.6	85.2	96.6	87.1	95.7
9.48	15.46	116.2	102.5	99.8	99.3	95.4	107.4
4.78	14.98	97.9	98.2	98.9	104.4	108.2	103.4
8.75	23.07	99.4	103.9	99.8	99.6	102	96.9
8.18	17.46	101.7	94.5	99.7	96.1	92.4	91.5
6.61	11.86	100.7	104.6	99.5	116.3	112.6	108.6
9.92	20.72	105.3	101.7	96.7	104.7	104.9	108.7
5.57	18.12	97.9	100.5	106.8	108.8	113.1	114.8
8.51	14.51	96.3	99.3	94.8	101.9	105.5	102.9
7.25	15.42	107.3	128.8	120.7	127.4	128	130.3
5.14	16.32	100.9	98.2	99.4	99.1	94.8	102.4
5.55	16.86	106.9	97.9	106.1	105.1	105.4	106.3
9.94	15.21	97.9	101.6	102.2	110.5	107.5	112.4
6.24	17.14	107.5	92.9	87.3	101.3	108.5	108.1
7.81	25.72	94	99.8	95.5	101.1	99.2	99.9
6.52	15.48	89.9	109.5	96.9	105.7	89.6	88.2
4.61	14.2	83.4	88.8	79.5	104.8	99.4	96.3
6.29	13.68	100.8	96.3	97	106.3	116.3	115.5
9.6	16.29	103.3	99.7	96.5	137.5	141.2	135.8
8.32	16.17	98.6	94.4	91.1	94.4	94.4	93.4
5.72	16.08	92.4	96.9	94.6	106.5	105.5	106.6
9.11	16.75	106.1	94.5	89.5	99.1	114.3	113
8.9	13.94	108.1	106.1	105.7	96.2	98.8	97.1
10.18	16.18	102.2	98.8	98.1	113	117.7	116.3
8.43	16.53	99.5	89.4	92.1	96.8	96.6	101.2
8.09	24.51	83.1	86.2	81.6	107.4	102.8	106.1
9.38	32.3	99.3	100.3	101.1	106.8	106.5	106.3
8.95	15.02	97.6	94	94.7	98.2	98.8	99.6
8.22	17.5	101.9	97.8	100.9	98.2	101.8	96.4
5.97	14.83	94.1	93.6	92.1	104.9	98.9	106
10.7	42.41	100.5	94.4	97.4	107.1	108	110.4
9.99	15.87	92.2	96.7	96.8	107.5	106.9	104
5.97	14.72	102.7	96.3	100	96.9	95.6	101.4
8.79	16.83	94.5	100.4	93.4	110.7	113.4	106.7
9.16	9.2	110	110.8	103.9	107	99.3	110.3
5.1	14.48	103.8	99.3	101.6	97.9	94.4	95
4.64	11.68	92.4	96.8	89.9	114.7	116.1	112.8
8.43	13.21	93.5	90	90.3	104.9	98.6	109.8
8.37	18.84	100.4	103.5	103	105.9	105	107.7

5.54	25.59	94.3	96.2	91.7	103.3	102.9	103.6
6.77	26.68	102.8	102.2	102.1	97.6	95.1	95.3
7.02	14.12	101.3	94.7	94.9	102.5	100	104.3
5.95	18.79	88.3	98.1	88.7	101.4	92.3	100.8
9.45	36.85	102.5	101.4	97.2	107.3	107	107.5
4.77	14.45	95.3	94.6	93.5	102.2	91.6	96.2
5.08	25	111.8	105.3	101.7	129.5	132	133.5
6.54	18.17	113.9	110.4	110.2	77.7	73.1	74.6
5.07	12.38	99.3	102.3	99.9	107.4	107.7	100
5.55	15.79	99.6	97.3	100.2	102.6	104.7	98.4
10.92	18.67	103.8	101	100	105.9	106.8	108.8
7.34	20.99	102	98.3	97	91.9	92.5	94
8.37	15.52	102.2	114.7	112.8	107.9	104.7	107
9.36	21.75	103.9	100.7	100.1	100.9	97	96.8
10.95	44.07	109.5	103.6	101.8	102.6	104.6	106.7
5.59	13.37	102.2	94.9	98.4	109.8	112.7	109.3
5.38	13.4	102.3	102.4	98	92.5	92.5	92
5.67	17.8	98.3	97.9	99.4	101.1	99.6	106.5
10.35	18.33	105.8	103.6	108.5	108.3	105.5	105.1
7.62	16.53	102.2	108.5	104.4	99.7	95.5	102.2
6.18	14.62	100.1	109.8	101.2	101.2	106.5	101.9
8.66	18.4	105	97.3	97.3	100.7	100.9	103.7
8.1	23.41	99.9	99.2	101.7	102	101.3	102.7
7.05	14.69	98.8	102	97	104.4	102.1	105.8
6.84	10.57	108.2	104.6	96.7	102.7	102.6	102.9
4.88	18.51	98.4	94.2	101.1	99.4	101.6	95.1
4.36	19.22	95.3	92.1	90.1	101.9	97.6	109.9
6.6	13.85	107.6	107.1	105.8	91.1	88.4	91.1
9.33	11.19	107.9	101.7	98.9	90.3	102.2	100.3
7.05	18.44	95.4	94.2	93.2	101	107.4	108
5.94	24.86	104.2	110.7	107.8	84.8	91.9	100.4
6.35	8	99.8	99.5	114.8	102.3	95.1	100.7
6.61	23.67	88.1	83.6	96.2	112.2	115	115.3
8.24	16.15	103.1	102.8	104	104	109.3	106.7
6.61	7.53	104.6	96.6	102.2	113.2	110.6	112.4
6.68	15.21	99.6	108.1	94.7	103.9	107.1	95.5
7.39	11.64	85.6	90.4	99.8	110.6	107.1	110.9
8.79	20.06	107.4	113	103.8	109.1	97.8	109.7
7.74	17.96	97.5	101.5	106	107.6	105.5	126.9
6.3	14.42	96.9	99.4	96.1	99.9	97.4	100.3
5.24	14.85	93.2	95.3	95	104.2	105.7	103.6
9.79	15.3	105.9	99.5	102	108.2	107.9	111.9
5.17	14.5	95	89.6	107.6	93.8	117.2	104.7
4.79	13.54	101.1	99.4	102.3	96.3	95.5	101.6
5.91	12.98	104.4	99.3	100	105.7	104.7	88.5
8.43	9.48	100.3	103.9	98.7	99.3	96	105.9
9.63	12.8	97.7	109.7	101.7	97.3	104	97.4
6.54	12.38	101.3	102.2	103.1	88.3	92.8	87.3
7.39	15.47	94.5	85.1	95.3	116.3	114.9	120.3
4.81	14.25	108.3	91.5	102.5	100.3	98.5	101.3
4.94	15.29	100.1	100.1	99.7	97.8	106.1	108.3

8.47	16.04	100.8	103.3	98.4	99.6	105.7	111.1
8.68	16.76	100.2	99.6	107	109.2	106.4	100.2
7.33	14.86	98	96.7	94.9	95.1	98.7	104.9
8.47	12.26	103.1	99.9	97.8	87.1	87	92.6
7.17	15.25	105	95.6	96.5	105.7	105.5	105.9
6.8	17.35	97.3	97.3	96.3	109.9	106.1	110.2
9.61	15.2	99	106.8	104.7	105.2	102.8	107.1
9.19	14.26	98.9	97.4	94.6	98.8	100.1	98.8
8.12	18.42	106.5	99.2	100.3	92	95.8	91.7
10.07	16.59	102.9	95.1	102.2	102.9	104.6	107.4
9.72	13.17	95.9	96	91.2	103.5	88	122.9
5.52	13.82	94.5	98.3	102	105.9	96.3	100.2
4.94	11.78	99.9	100.8	101.1	100.5	97.6	98.3
5.63	12.27	98.3	98.5	101.1	95.6	103.7	98.2
9.52	16.35	100.7	92.7	102.5	91.4	96.5	98.8
9.73	12.43	103	99.3	90.3	118.4	111.7	110.2
5.21	15.44	95.8	97	91.2	92.2	91.3	95.3
6.83	20.93	101.7	96.8	98.7	96.5	99.6	94.1
5.01	14.61	93.5	93.1	100.5	105.2	115.2	101.7
6.98	11.94	99.9	96.4	100.9	121.1	106.6	129.9
7.91	11.38	105.3	106.7	102.5	114.3	107.9	105.2
9.72	18.23	106.1	106.4	105.3	105.4	109.2	109.4
6.47	19.02	101.4	95.9	108.5	97.4	99.3	103.5
5.16	26.19	95.4	102.1	95	99	94.8	99.8
5.45	10.65	102.1	84.4	95.1	107.1	112.2	106.9
5.72	25.02	104.3	107.8	100.8	109	105.2	106.8
7.75	15.09	85.3	95	95.9	98.1	103.2	102.4
5.36	11.46	104.3	92.6	99.9	99.9	92.2	95.9
6.62	10.22	100.2	99.1	95.2	100.2	93.6	94.8
12.26	14.77	95.8	107.2	103.8	100.4	99.2	101.3
5.38	17.34	97.4	94.4	96.4	105.1	107.3	113
7.15	12.19	105.7	102	103.6	96.9	96.7	102
5.57	15.86	94	98.6	92.5	108.3	108.4	111.7
8.76	13.49	101.1	94.8	106.4	98.2	105.1	112.6
8.48	20.33	85.4	94.4	83.8	99.3	98.9	97.5
5.92	19.09	104	104.1	104	102.8	107.2	101.7
5.41	15.69	106.5	98	97.7	101.3	103.6	98.5
9.07	19.42	90.6	96.8	107.9	100	106.4	105.6
8.79	30.41	95.1	104.6	97.4	109.9	110.1	107.8
9.23	15.81	97.6	99.4	103.2	105.4	96.1	99.2
9.38	17.56	103	102.1	105.1	104.3	109.6	110.9
8.46	15.88	96.1	94.2	100	108	107.1	108.9
7.39	13.6	107.8	102.4	97.8	98	98.2	107.4
9.94	10.88	111.1	105.6	99.3	118.5	118.3	111.4
10.7	12.47	100.1	109.9	91.1	116.9	108.6	110.6
8.94	10.92	97.8	96.9	96.9	103.9	100.8	102.5
7.87	11.33	97.4	97.1	91.4	97.9	98.5	100.7
8.69	12.09	105.6	100.5	99.7	116.3	97.7	120.7
5.11	15.19	97.2	95.6	92.8	110.6	106	105.2
5.31	25.64	94.8	98.4	94.1	100.2	92.7	97.1
5.74	18.76	96.4	86.5	94.1	82.1	86.9	93.3

5.77	10.24	93.4	94.1	102.4	93.2	109.7	100.3
6.51	10.35	107.8	100.2	101.1	104.3	108.8	105.2
4.98	15.77	99.4	102.9	98.6	100.4	101.6	105.1
8.9	19.8	104	96.3	103.6	102.7	109	106.8
7.71	16.52	97	91.3	100.3	107.7	112.7	101.8
9.85	22.21	104.4	103.1	101.5	100.8	103.8	103.6
4.78	14.18	91.6	95.6	98.4	96	97.6	101.2
4.83	16.36	102.9	95.8	102.5	113.2	107.2	106.4
7.43	18.83	97.4	97.8	92.4	98.3	96.8	95.1
9.03	12.78	105.3	100.8	100.3	100.1	113.7	110.1
6.07	11.96	105.7	101	99.1	107.2	99.3	103.5
7.14	19.94	116.9	117.4	118.2	70.6	73.5	71.8
9.66	26.9	104.6	101.1	108.4	95.3	107	99.4
6.73	12.57	103.5	98.7	107.7	116.8	97.6	108.5
5.41	16.39	101.3	100.1	98	91.6	94	96.6
7.88	16.46	106.6	102.2	103	102	101.7	104.4
7.84	16.45	104	103.2	93.3	99.7	98	104.9
8.53	14.01	92.7	89.2	94.9	107.3	108.6	101.7
7.4	15.9	95.8	92.6	97.4	102.7	97.6	95.6
4.61	14.15	104.7	97.3	99.9	110	102.4	103.3
5.1	26.42	94	95.6	94.5	97.9	90.3	96.6
6.79	13.25	103.1	96.9	109.6	100.2	104.5	103.7
5.29	16.47	99.3	98.9	104	101	98	104.3
7.49	20.78	101.4	101.6	99.3	100.2	95.3	100.1
9.16	15.11	93.9	92.3	95	97.7	98.8	105
8.09	10.53	106.8	100.5	100.7	95.2	88.5	94.7
5.58	18.02	103.4	105.2	99.4	99.9	102.8	99.2
5.45	9.88	101.3	95.2	98.7	100.9	100.7	107.9
9.57	17.38	107.4	101.5	97	93.9	99.4	102.2
4.86	15.56	92.9	100.2	102.4	100.9	101.7	101
4.74	23.8	97.7	92.8	97.6	102.2	100.2	107.9
8.92	16.26	105.8	106.3	103.4	82.5	99.7	89.1
6.79	16.8	116.5	101.3	104.8	87.8	88.1	94.5
6.9	11.06	111.2	97.2	97.8	102.1	96.2	102.6
5.9	15.34	98.5	98.3	96.2	100.1	100.2	107.6
8.02	13.56	106.4	104.5	100.5	109	94.3	112.4
6.16	15.3	101.4	102.4	102.2	100.1	96.2	102.1
8.24	18.35	94.9	99.2	97.9	96.6	99.5	97.9
5.71	18.02	106.7	96.2	94.8	104.2	98.6	101.6
7.81	13.37	105.2	98.7	98.8	93.8	98.9	102.4
8.87	18.81	99.7	97.9	99.5	102	106.6	110.5
8.75	17.46	101.8	101	102.3	98.4	98.9	101
8.56	16.63	107.4	95.9	99	107.1	110	116.5
11.27	18.24	101	105.5	108.9	108	107	116.8
6.21	12.61	97.7	97.8	100.9	101.7	100.6	93.9
5.68	11.33	91.2	88.6	107.1	106.4	99.2	99.5
8.41	15.1	107.3	98.5	104.9	105.4	104.2	107.8
8.19	13.75	100.5	96.1	102	98.2	99.9	100
8.38	14.98	97.2	97.5	91.7	100	96.5	96.6
6.8	8.57	104.8	102.2	100.7	102.7	107	112.3
9.94	16.2	101.2	105.1	103.6	98.6	101.8	99.3

5.49	15.49	97.5	102.2	102.9	105	95.6	100.4
6.95	13.13	82.4	77.5	78.2	116.8	109.8	117.8
7.72	16.64	107.7	100.7	103.5	107.5	102.9	100.4
5.9	32.03	101.1	104.8	101	99.5	94	96.1
6.54	8.05	101	97.5	101	99.4	96	100.4
8.09	11.72	102.6	100.7	98.2	116.8	102.7	111.3
8.53	13.18	104.5	104.5	103.7	97.6	104.8	103.1
10.76	13.64	109.4	95.4	130.8	104.9	131.8	106.4
6.05	9.96	103.8	96.7	95	92.5	97.4	100.3
7.93	13.58	107.1	107.6	103	92.2	93.5	90.9
6.15	13.51	106.9	98.2	104.6	112	108.1	108.9
8.7	13.35	103.6	95.9	100.5	100.2	104.3	104.1
7.93	16.73	121.9	113.8	108.7	94.9	100.3	100.9
7.74	26.46	95.3	97.3	98.5	106.9	107.5	109.5
5.99	13.22	97	93.1	108.1	98.9	100.3	96.5
9.55	21.29	99.8	98.5	101	102.8	98.3	97.8
8	16.89	95.7	85.6	90.4	102.9	110.1	109.7
6.93	12.13	92.9	87.5	97.1	100	96.8	95.2
7.25	16.77	93.6	90.2	100.7	105.6	113.1	115
8.05	17.06	104.7	98.5	98.9	96.7	94.4	96.8
5.97	13.63	101.6	100.6	97.5	103.7	116.5	104.4
6.2	13.76	94.1	88.7	94.8	103.9	105.9	108.9
9.61	11.66	108	103.5	94.1	104.7	102.5	111.8
5.07	16.71	95.3	91.6	101.8	97.1	106.7	106.4
7.72	12.16	101	95	101	94.5	97.6	91.7
5.92	11.73	106.1	98.6	97.2	108.6	95.4	96.3
8.5	12.87	97	91.7	88.4	103.2	103.4	106
6.58	12.9	109.2	108.9	98.3	97.4	88.4	97.8
10.37	9.41	101.8	95.4	104	111.4	107.9	116.3
5.49	12.75	101.8	98.2	100.7	97.3	97.2	103.6
8.15	12.62	100.9	111	105.6	109.9	101.1	106.6
7.87	15.11	103.5	98.5	104.7	112.7	113.9	116.2
7.06	15	112	103.9	105.1	100.8	91.9	99.7
6	12.76	93.5	102.9	101.1	101.9	95	102.6
6.4	24.1	96.2	94.4	94.7	92.6	93.8	98.5
8.21	14.97	99.6	100.4	98.7	127.8	113.2	103.2
6.11	16.07	63.8	65.1	63.4	135.7	136.4	131.3
7.83	14.78	107.4	97.2	97.9	101	101.3	97.2
10.01	14.43	99.4	108.2	103.6	103.2	109.5	104.1
7.68	14.94	105.7	99.9	90.3	101	105.3	107.5
7.49	22.12	92	90.9	93.2	106.7	112.8	112.5
4.58	18.05	103.9	97.2	94.9	96.1	101.7	100.6
5.99	11.5	106.8	98.4	99.2	101.7	98.6	106.8
5.55	10.14	99.9	92.5	107.2	92.1	92	110.2
9.14	12.55	109.5	98.9	98	97	95.9	95.1
8.48	13.78	93	102	98.2	103.4	103.3	99.4
7.14	13.43	109.2	107.5	98.5	108.5	119.1	111.4
9.44	33.82	98.9	102.2	105.7	107.3	101.8	108.5
4.93	13.07	99.9	103.2	104.3	97.9	99.1	106.5
9.99	12.75	102.1	96.9	102.5	111.3	108.6	113.9
4.83	8.94	97.8	90.6	97.7	111.2	97	96

8.19	14.21	101.5	107	111.3	84.5	89.2	94.4
5.71	14.29	87.2	85.4	84.1	100.4	100.2	105.8
9.16	17.72	103.5	101.5	100.9	94.6	94.7	96.7
7.47	12.64	105.1	97.8	101.3	92.8	96.4	97.1
9	18.3	96.3	88.4	86.7	110.6	108.6	105
6.3	17.53	98.9	88	83.7	105.8	106.8	105.4
5.52	19.39	96.6	94.4	88.8	108.3	103.5	105.7
9.04	14.63	96.8	96	95.1	105.5	104.7	110.8
6.15	16.23	100	95.2	99.9	86.1	95.5	95.2
5.87	14.14	118	74.7	111.6	148.4	120.7	131.5
6.16	14.63	98.8	92.9	92.9	104	100.2	101.2
5.4	14.19	104.5	102.6	97.5	106	104.8	106.7
7.83	13.85	92.7	97.4	99.7	96.1	98	102.6
8.25	14.38	108.9	96.6	96.6	95.5	96.5	96
8.1	15.72	102.3	100.5	97.2	105.8	103.2	103.1
7.34	10.03	94.4	98.6	103	105.2	99.2	103.7
9.16	12.75	101.3	114.8	107.4	97.3	103.1	109.7
5.26	14.36	97.9	97.6	107.4	96.6	99.8	103.3
8.13	15.36	113.6	110.2	107.1	127.7	127.4	134.6
5.41	13.87	102.7	94.3	104.8	95.5	102.9	102.9
9.38	14.44	110.4	106.1	105.1	106.6	104.8	109.6
8.27	16.59	108	107.3	102.9	101.3	105	101.7
9.63	12.92	101.8	104	99	101.2	106.6	102.6
7.05	10.45	101.3	82.9	100.3	90	109.9	98.5
6.92	12.52	107.1	105.9	103.7	105.5	100.6	102.9
6.64	8.03	100.9	84.8	99.2	91.4	105.2	92.9
7.4	16.15	107.4	98.8	102.2	95	95.1	96.7
5.24	16.04	94.6	96.1	95.2	104.2	98.7	105.2
5.6	11.93	100.3	100.7	104.5	99.9	96.8	96.4
9.92	11.81	97.9	95.9	100.6	109.7	109.7	112.5
5.1	11.68	102.6	94.8	100.8	98.7	101.5	99.3
8.22	12.76	96.9	94.1	95.7	96.7	99.5	99.9
6.46	12.13	95.9	99.7	100.2	98.5	100.6	91.4
6.54	11.06	97.7	89.2	86.9	103.7	115.5	109
9.61	10.03	103.2	103.2	108.6	103.3	100.1	103.8
7.37	20.91	102.6	102	83	100.7	99.5	99.2
4.87	10.88	96.3	96.8	93.9	97.3	93.7	91.7
9.16	14.87	95.2	90.4	93.3	106.9	115.5	114
7.65	12.59	102.8	95.2	90.5	98.3	101.1	98
8.84	13.27	76	89.1	69.2	103.9	97.3	104.2
7.64	12.81	99.9	91.9	97.5	96.5	96.4	104.5
5.54	15.2	94.2	103.9	110.1	98.6	97.2	94.6
5.19	13.1	101.6	103	98.5	104	101.4	93.5
6.48	11.97	94.5	96.9	93.8	109.5	101.2	101
5.3	21.84	104.6	94.6	107.5	91.7	92.1	91.2
7.05	11.99	113	110.6	111.8	90.2	89.8	95
7.75	10.14	91.3	106.5	96.8	93.6	97.7	104.6
8.19	15.84	54.5	54.5	62.9	135.7	140	138.2
5.31	11.35	97.8	95.7	94.4	92.5	91.1	94.8
7.99	16.27	100.6	97.6	99.6	107.2	112.8	108.3
7.65	13.11	98.4	100.4	101.9	97.9	96.2	111.9

5.91	17.31	101.9	71.5	69.2	122.2	104.4	92
8.79	18.16	98.2	94.5	94.8	106.5	101.5	98.7
6.99	17.73	97.2	94.4	99	103.4	102.7	106.8
7.39	9.46	95.6	94.5	93	111.6	111.5	118
4.97	16.58	95.9	98.7	104.8	98.3	93.3	91.2
7.15	18.36	97.4	99.9	99.3	98.9	100.7	100.9
5.57	11.82	96.4	95.7	96.6	101.7	99.1	96.9
5.31	17.81	102.6	95	98.6	100.3	101.7	100.6
9.11	14.57	103.9	109.6	93.1	97.5	93.9	86.8
6.57	13.05	99.2	103.9	103.7	100.1	94	95.9
5.29	15.57	96.7	95	99	93.8	106.5	101.5
6.18	13.94	98.2	90.9	90.1	99	102.5	114
7.94	14.43	95.8	93.6	101.1	102.6	104.2	96.3
6.6	15.84	106.9	102.3	99.1	97.7	97.4	95.3
7.31	16.17	107.1	102.2	97.9	83.5	93.1	92.1
6.13	14.25	107.4	109.8	103.5	96.6	98.3	98.4
8	20.61	96.1	97	118	101.6	101	76.9
7.44	15.13	96.3	96.3	97.5	105.3	101.9	107.1
9.29	13.5	104.2	96.8	95.3	105.7	111.6	110.6
5.69	17.88	102.2	99.5	95.2	89.4	87.5	91.1
7.4	11.95	108.8	108.2	108.8	97.4	101.8	98.9
9.29	12	108.7	97.2	98.4	103.6	108.1	105.4
6.77	13.75	105.7	99.6	104.2	102.7	102.5	103.2
6.27	15.54	108.8	94.8	94	101.4	105.1	99.3
7.8	16.86	107.9	108.7	109.6	82.1	76.4	78.3
9.35	13.08	101.9	102.4	99.5	114.5	112.3	108.4
9.79	13.07	103.9	95.1	98	105.4	105.4	105.8
8.02	13.18	90.7	96.5	96.7	98.8	97.2	105.8
8.65	13.54	104.7	101.6	97.6	98.7	98.9	97.9
5.35	15.35	92	100.6	94.4	110.5	108.7	107.5
4.54	12.29	105.2	100.7	102.3	106.3	105.6	113.9
10.3	14.5	99.4	103.4	104	105	107.3	108.9
7.96	20.05	105.2	74.9	69.6	101.3	103.2	105.9
4.61	11.63	102.6	99.1	100.9	110	115.6	110.8
5.4	11.57	110.7	98.1	104.1	108	110.5	109
8.79	12.28	98.9	98.2	105	99	97.2	105.5
5.17	9.6	96.2	97.8	104.8	93.6	98	91
6.07	16.49	101.9	100.1	99.3	105.1	97.7	101.4
5.27	11.57	99.4	100.4	100.9	97.5	104.9	98.1
9.41	11.6	98.3	94.1	93.3	99.4	99	98.5
6.52	15.68	108.4	103.2	106.5	97.9	95.7	105.7
5.57	8.26	96.8	90.4	102.8	93.8	100.1	97.6
7.28	16.78	100.6	109	107	116	116.7	116.4
9.67	13.82	120.1	92.3	94.6	100.5	122.5	102.1
4.77	11.71	102.4	105.8	101.1	96.7	100.3	105.9
5.6	15.9	95.1	87.5	93.5	99.4	102.3	101.5
9.01	12.34	89.7	86.1	87.8	103.5	110.4	113.5
6.38	8.62	108	114.4	96.9	102.3	94.9	85.2
6.95	9.55	99.6	100.9	105.6	97	103.9	105.9
8.09	10.55	137.2	94.6	88.7	98.3	124.9	90
5.88	11.62	97.4	99.4	97.9	105.4	103.9	106.5

7.83	13.37	103.1	107.8	110.2	86.7	95.5	101.2
5.25	13.29	97.3	98.5	101.9	106	110.8	108.6
7.36	15.78	92.7	105	102.9	105.6	90.2	105
8.68	10.62	99.5	106.8	102.4	104	117.6	117
9.16	11.6	107.4	98.1	97	110.6	110.2	117.3
6.29	13.14	102.4	97	98.9	100.7	97.4	102.1
10.84	15.36	112.3	109.9	111.4	91.9	90.4	85.6
9.29	12.57	100.6	94.6	95.4	92.4	101.7	96.2
10.48	17.83	102.7	100.6	95.9	109.6	106.2	106.2
8.57	19.36	94.1	99.5	99	96.3	95.1	92.3
6.76	10.87	95	93.3	99	105.8	101.1	95.9
7.01	11.45	101	97	109.6	81.6	110	90.2
9.54	9.7	90.1	103.6	97.7	103	103	112.1
8.34	10.67	97.5	90.3	105.3	106.8	120.6	110.8
4.86	16.45	96.8	96.3	95.1	96	96.5	97.8
6.15	14.34	89	99.7	90	110.1	102.9	106.1
7.42	16.58	97.5	101.8	103.4	99.2	100.7	91.9
4.97	12.94	104.4	93.6	103.7	104	103.4	105.8
6.73	14.22	119.4	104.3	103.7	105	104.8	95.3
6.43	8.19	93.6	89.9	85.1	100.4	101.5	106.4
7.66	23.09	94.7	113.1	105.8	119.3	112	122.5
8.03	13.61	118.9	92.5	97	87.5	102.1	109.6
8.37	13.22	102.7	98.2	98.5	96.3	99.8	95.9
6.33	14.95	95.8	98.6	104.8	109.5	107.1	114.6
10.39	13.04	104.3	102.6	101	111	108.2	102.8
5.85	8.19	87.8	85.6	95.5	95.6	100.2	97.4
7.39	6.61	106.1	101.3	91.3	71.4	97	97.9
7.37	7.93	93.8	106.6	105.3	104.1	103.9	114.7
9.33	12.09	98.7	94.5	104.9	98.9	113.1	111.1
8.48	18.58	103	96.1	97.4	98.1	93.6	99.1
6.9	11.86	94.6	93.7	89.8	95.5	100.4	112.6
6	14.65	110.4	106.6	104.4	91.8	94.9	88.3
8.95	13.5	107.1	97.3	93.8	107	106.5	110.4
8.82	14.24	101.2	102.2	107.5	106	106.8	102.8
5.43	18.36	92.2	84	94.1	98.3	98.3	99
8.21	13.02	94.7	99.4	108	103.4	104.5	105.7
9.35	15.48	101	106.2	104.6	107	112.1	108.2
11.27	14.94	106	105.2	99	106.4	112.9	106.6
9.39	16.37	73.7	79.8	66.8	132.1	127.1	126
6.16	13.32	96.2	94.6	96	103.3	109.7	103.1
7.91	10.29	104.3	96.3	94.2	114.9	114	107.4
5.21	12.54	101.5	93.3	94.1	89.5	83.2	87.6
9.88	10.94	94.9	92.6	94.6	117.9	112.2	116.2
10.13	11.59	94.4	96.7	102.6	103.4	108.1	102
7.33	11.67	103.9	98.8	92.4	101.8	99.6	103.7
8.85	13.81	94.6	95.3	101.2	89.1	102.5	85.6
7.49	11.74	116.1	109.3	113.6	115.4	113.9	112
4.82	21.66	94.3	94.6	93.8	105	104.9	106.3
6.49	12.5	103.7	92.4	105.4	101.3	100.8	101.6
5.12	13.12	103.3	91.5	93.3	97.4	101.6	110.6
6.55	9.69	90.5	89.7	99	95	104	101.4

9.26	12.86	100.9	113	103.4	101.7	105.9	108.9
5.2	13.01	121.5	121.4	117.9	73.8	79.5	77.3
5.83	9.93	96.1	100.6	100.4	104.7	105.8	107.2
7.94	9.35	118.8	114.7	96	82.6	76.7	81.6
6.79	13.42	99	93	92.1	89.5	85.7	90.9
5.22	13.31	97.5	94.5	99.9	117.3	108.4	117.4
9.94	8.97	100.6	101.4	98.9	103.7	102	99.7
5.86	17.14	111.8	108.8	111.2	94.7	86.3	88.6
5.9	16.21	98.7	94.3	98.1	95.8	98.2	102.1
8.21	15.98	107.7	100.7	102.2	107.6	110.2	114.1
5.06	11.61	110	94	97.5	97.8	110.6	105.4
6.01	9.02	95.8	98.6	98.8	104.2	105.4	107.8
4.97	9.93	98.7	98.8	89.1	117.5	107.7	104.3
9.36	9.34	102	97.3	101.1	106.2	102.2	108.1
5.73	12.9	102.5	103.5	103.6	95.8	100.5	97.3
5.12	15.96	99.6	99.3	103.6	101	101.9	101.7
4.84	11.01	112.7	84.9	101.1	108.3	101.6	101.2
6.55	13.11	93.2	97	87.1	101.1	106.6	103
8.73	13.09	103.3	94.5	101.5	103.5	100.9	97.1
4.51	12.32	106.6	99.6	101.3	99.4	101.8	104.5
8.75	15.22	96.4	103.7	93.4	93	99.7	100.7
5.96	14.4	95.6	98.6	104.9	97.3	105.9	105.6
8.54	12.66	111.6	99.2	102.1	92	91.5	99.9
9.64	12.57	103.4	106.6	105.6	103.6	106.2	108.6
5.64	8.77	116.8	107	115.4	96.6	90	94.5
5.85	10.83	106.9	90.1	95	104.4	100.7	107
5.34	13.44	99.6	97.1	92.5	93.2	104.4	100.8
7.46	11.01	99.1	100	107.6	95.7	91.9	104.8
10.39	14.37	103.1	108.2	104.9	100.1	103.2	103.8
7.31	10.73	103.2	99.7	104.8	111.5	112.8	117.7
4.7	12.66	98	99	98.8	95.8	107.9	109.2
5.5	23.14	109.3	96.1	114.1	113.4	122.8	109.1
7.01	10.25	100.9	101.7	101	130.6	127	111.4
7.33	14.17	97.8	101.1	105	101.7	91.7	105.3
7.24	13.57	100	97.8	104.1	94.6	100.9	104.4
8.5	26.98	112.2	98.4	109.5	96.8	105.8	98.2
9.77	16.85	99.7	97.6	101.9	95	94.7	93.7
6.89	9.24	91.2	83.5	91.3	114.7	107.9	108
5.71	13.12	98.3	99.9	100.5	100.6	111.3	115.7
9.76	14.64	100.2	91.3	99.1	99.4	102.6	106.6
6.18	11.08	101	99.1	92.5	89.2	95.9	89
4.83	15.98	96.6	102.2	102.6	103.3	100.7	107.8
6.01	8.03	109.9	107.7	112.3	102.5	108.1	120.3
7.55	16.38	103.7	99.4	97.6	95.7	100.2	95.5
8.5	11.52	92.6	84.2	75.5	106.5	102.1	109
7.72	11.43	97.2	101.4	103.6	101.4	103.1	103.5
8.54	10.71	108.8	102.6	104.4	100	106.5	118.5
5.11	11.31	102.6	101.6	99.9	104	100.7	99.3
8.92	16.58	101.5	104.9	96.9	107.6	106.7	107.6
8.5	11.99	94.1	91.7	109.3	93.4	74	87.3
11.09	11.76	100.3	97.8	101.1	105.9	103.2	105.2

5.15	9.41	112.6	105.9	99.1	101.6	99	112.1
6.8	17.44	103	96.7	102.3	90.3	84.6	85.6
11.41	6.54	110.7	99.9	102.1	105.2	105.2	106.9
6.24	6.29	103.1	109.7	80.9	100.5	96.2	93.5
8.76	18.09	100.2	95.5	98.1	110.2	114.6	113.9
11.65	12.54	105.8	102.6	103.1	104.1	110.9	105.8
9.7	9.88	97.9	101.7	108.7	111.3	95.1	112.5
6.48	18.07	88	92.8	94.3	102.7	94.6	95.9
6.11	12.69	101.3	97.8	96.6	97.8	95.7	102.7
5.22	11.22	106.9	96.1	99.3	101.9	99.3	101.2
5.9	13.51	110.2	99.4	98.6	110	111.5	114.2
9.5	9.89	113.3	101.5	83.5	106.1	112.6	111.1
8.44	10.24	95.3	90.9	98.6	102.1	98.1	104.5
8.44	13.11	103.9	104.3	94.4	102.4	94	98.6
9.7	9.61	107.7	97.9	110.6	104	94.9	97.1
6.46	13.68	99.6	94.1	102.5	93.2	95.6	100.9
4.84	13.35	110.1	100.6	107.4	96.2	96	95
5.44	11.65	135.1	71.2	87.4	72.5	98.9	98
9.58	10.47	100.4	95.8	99.6	106.5	106.4	104.8
7.59	12.66	94.7	111.4	98	100.3	99.8	103.4
9.99	11.8	118.4	103.5	103.3	106	103	108.8
5.52	8.5	101.1	103.9	113.5	104.6	113.5	110.9
6.6	8.94	112.3	105.7	104.6	101.3	94.3	108.6
9.45	10.46	98.7	114	101.9	101.7	100.7	98.1
6.68	13.12	107.1	101.5	106.1	96.1	99	104.7
7.61	12.08	105.4	107.9	94.1	95.2	99.4	103.4
7.17	8.75	96.2	101.7	112.1	93	98.3	106.4
6.35	10.93	91.5	91	102.3	114.8	104.8	96
6.89	9.66	92.2	95.9	91.6	102.7	101	99.3
9.94	12.66	102.3	99.8	99.3	103.7	105.7	111.6
6.73	15.24	97.6	99.1	106	95.7	94.6	103.8
4.88	12.15	105.7	100.2	98.1	103.6	97.9	97.6
9.85	10.02	119.9	95.2	115.9	107.7	88.9	92.3
9.83	16.33	117.7	77.1	88.1	115.9	59.9	101.9
6.7	10.65	95.4	91.9	106.9	103.1	111.8	103.2
8.07	8.45	104.2	99	94.4	101.7	99	90.3
6.74	16.77	91.1	94	98.6	110.4	108.2	94.1
9.52	16.27	100.4	98.8	98.7	102.5	104.4	106.4
7.65	12.27	99.5	102.6	97.9	98	91.7	99.2
7.01	10.71	101.2	102.8	108.1	102.6	109.7	113.2
4.84	13.8	98.8	102	100.3	99.8	105	113.8
9.63	12.9	89.7	95.2	95.5	97	97.8	100.5
7.85	7.33	104.8	66.9	110.6	100.6	97	96.9
11.56	21.9	93.3	91.6	93.5	122	123.5	123.3
6.29	14.55	108.7	106.6	105	110.9	109.4	112.5
9.16	11.49	99.8	97.7	94.1	108.7	99.1	115.8
5.45	11.98	99	93.9	93	105.7	102.7	105.9
8.54	12.99	86.6	92.6	100.4	106.4	106.3	102.8
5.11	8.19	102.6	103.6	107.3	110.9	103.8	93.7
8.72	11.91	111.2	104.4	88.7	97.4	140.9	111.5
7.21	11.67	101.6	104.9	104.7	96	102.1	98.6

6.16	11.19	98.9	105	100.2	100.9	98.7	100.9
6.61	12.54	104.2	97	94.2	102	101.7	107.7
6.2	15.81	103.7	105.1	97.5	105.6	106.4	105.7
9.57	11.87	109.5	105.7	101.3	109.1	111.9	108.8
6.05	8.67	95.1	99.5	100.8	100.6	93.1	97
7.93	12.4	116.1	111.9	108.7	103.7	100.5	99.3
8.76	10.92	101.6	94.6	105.1	104.3	103.3	97.2
6.54	10.25	88.4	89.9	89	107.9	105.7	119.1
6.07	10.76	106.7	100.4	104.9	105	104.5	101.8
9.16	9.97	111.2	88.1	99.9	117.7	105.2	129.7
5.24	8.51	107.3	102.3	94.4	104.3	89.5	98.6
5.01	10.67	106.7	95.2	100.1	114.9	116.3	116.8
8.31	19.88	100.5	110.5	107.2	92.1	102.4	97.1
6.52	16.65	98.2	99	98.4	102.4	102.6	104.6
5.15	12.68	99.8	101.4	97	109.6	104.3	108.3
5.6	13.17	98.9	94.9	97.3	102.8	106.9	106.3
7.08	11.56	95.5	97.5	98.4	105.5	102.7	106.7
9.69	11.91	105	113.2	105.7	94.6	91.4	100.5
8.81	14.96	101.7	106.7	101.4	106.7	111.1	106.1
9.77	9.09	93.6	105.5	95.6	105.4	107.1	102.4
5.92	11.71	100.1	102.2	99	99.1	98.2	96.4
9.25	10.09	101.6	97.2	99.3	113.9	112	121.5
6.58	10.99	92.6	99.9	102.8	95.6	91.3	97.6
5.34	17.4	77.8	77.6	79	125.2	125.6	123.1
8.19	12.93	93.1	95.3	102	107.4	112.9	111.4
7.37	9.24	95.3	90.3	96.3	128.2	128.1	120.2
7.12	14.55	98.4	98.1	94.7	100.2	99	102.7
8.41	10.72	99.2	95.7	101.3	102.1	97.1	96.8
9.63	10.31	110.8	108.4	107.4	106.1	105.7	103.8
6.9	9.49	100.1	89.8	98.4	102.7	106.1	114.5
9.58	16.7	104.7	104.4	102.9	101.4	103.9	100.4
6.51	13.63	95.8	90.8	102.2	96.6	93.8	97.5
5.43	10.97	101.9	106.4	114.8	104.9	99.3	103.6
8.53	9.82	97.8	102.3	106.5	109.8	105.8	111.1
5.81	10.81	90.6	108.6	105	96.8	92.4	100.5
8.43	8.53	103.3	103.6	108.5	105.9	89.8	94.4
10.64	10.58	103.3	100.1	98.9	113.1	111.3	103.5
7.24	10.33	96.9	93.4	95.4	98.4	102.6	109.9
6.16	8.68	101.5	94.4	101.5	95.3	99.7	97.1
7.88	11.21	86.1	86.7	83	118.2	116.9	120.9
9.03	8.77	98	95.9	95.2	100.7	98.1	108.2
6.46	11.72	99.3	98.5	99.4	95.9	98.2	89.4
5.14	9.7	106.4	101.8	93.7	86.8	102.1	95.7
5.49	21.29	102.4	105	100.7	104.5	107	105.2
4.97	10.42	105.2	104	95.4	103.7	105.8	98.4
5.68	12.39	99.5	102.3	108.1	96.7	92.9	99.9
7.96	15.46	92.8	91.8	93.9	114.6	109.2	108.7
7.21	17.55	103.8	96.7	101.2	104.1	107.1	101.1
9.06	15.06	103.2	105.4	104	108.6	106.5	105.3
6.13	11.64	90.8	96.4	103.6	101.5	108.6	108.1
7.43	13.6	101.2	107.9	99.1	91.3	96.1	101.3

6.54	9.62	102.9	102.3	96.3	96.2	93.1	103
7.66	5.3	106.2	110.3	97.9	96.2	109.1	83.8
9.8	18.23	78.9	93.4	86.9	106.9	110.2	102.8
7.61	14.98	109.6	101	107.9	93.3	105.3	104.4
8.62	10.79	95	88.4	90.5	114	105.8	103.9
5.11	10.88	55.9	53.2	60.1	171.4	166.1	164.5
8.32	12.49	105	108.5	102.9	106.7	103.1	101.8
5.85	6.49	102.7	101	92.6	111.1	108.5	109.7
7.27	15.03	109.3	96.3	97.5	109.3	105.4	113.7
4.48	11.12	102.5	93.3	101.5	108.6	102.1	100.5
7.72	9.28	95.9	103.4	100.1	90.7	93.7	96.6
9.7	11.37	108.9	103.1	98.9	103.8	100	100.1
6.57	12.31	99.3	95	96.1	97.5	100.5	110.3
8.85	17.69	102.4	101.9	96.8	102.8	100.3	104.5
5.06	15.57	100.1	101.5	103.6	100	103.9	105.1
7.49	11.97	92.7	95	94.3	112.6	107.6	112.7
11.74	35.5	95.8	99.6	96.3	117.3	111.6	114.4
7.84	11.88	83.4	85.6	83.2	87.6	85.2	86.1
9.38	28.23	90.9	95	90.4	98.7	98.2	94.7
9.76	17.98	98.3	105.5	102.6	105.4	106.7	105.9
8.21	11.19	99.8	101.4	96.4	86.2	84.9	92.1
7.11	11.89	107	93.7	94.9	85.4	80.5	107.5
5.78	10.44	118.7	106.2	91.6	112.3	79.7	91.8
4.49	10.83	96.8	93.6	94.7	100.9	93.4	104.3
9.13	10.49	96.8	97.9	98.6	103.5	101.5	101.7
6.24	17.35	97.3	94.5	96.5	99.8	95.7	96.7
9.29	13.65	104.3	96.2	94.5	110.8	109.2	107.7
7.93	9.21	97.2	110.3	124.1	95.5	95.9	100.1
6.62	9.99	91.4	98.1	103.3	103.4	98.9	102.4
5.03	10.01	94.7	93.8	105.7	97.4	114.1	108.9
8.19	9.97	90.8	92.8	90.9	112	110.4	118
6.87	10.69	102.2	97.6	102.6	97.6	100.2	100.9
9.04	13.2	102.4	93.7	100.7	109.4	112.7	111
7.47	8.18	99.9	103.2	100.4	99.2	100.5	102.9
9.26	15.03	105.1	93.1	99.2	96.2	96.8	100.5
5.83	13.95	84.5	77.5	79.7	89.3	89.2	89.5
5.31	11.65	108.2	83.5	104.8	97.5	111.6	114.8
10.15	11.88	101.1	93.5	97.5	103.5	111.9	110.5
7.02	9.57	96.7	98.4	99.8	103.6	106.6	102
4.88	14.01	119.2	102.8	100.3	103.7	105.4	101.5
8.48	11.61	101.8	105.1	103.7	100.1	99	104.1
8.09	6.96	77.9	88.8	93.4	112	108.8	110.1
5.02	11.41	95.3	98.6	94.4	98.8	96.7	101.9
8.72	8.65	94.5	95.4	103.1	111.7	118.8	102
9.01	5.62	98.8	104.3	109.4	107.3	98.8	111
8.06	17.95	100.6	100.9	100.7	106.2	102.9	103.9
9.67	22.34	101	105.2	101.9	107.5	105.2	98.9
4.25	15.37	97.4	102.9	100.2	104.4	103.5	105.9
6.38	9.48	101.4	96	95.3	103.8	101	108.8
10.27	10.76	102.8	102.4	97.2	110.2	115.5	108.2
6.71	13.6	101.6	99.7	103.1	102.5	97	99.2

7.55	9.12	99.5	94.3	89	98.4	103.7	102.2
7.15	10.11	103.1	83.8	91.3	104.6	119	120.5
7.94	11.52	99.4	99.9	98.1	107.6	107.2	108.3
6.16	10.73	93.2	103.5	98.7	104.1	97.4	101.3
5.19	10.14	104	101.7	99.7	95.3	107.9	102
10.81	10.8	99.6	106.1	100.6	102.2	104.4	111.7
5.08	7.47	103.3	88.4	92.8	110.2	112.4	109.4
6.1	11.11	103.4	89.8	94.9	99.4	104.2	102.9
9.38	19.94	101.5	97.2	101.4	103.3	104.1	100.8
5.11	12.72	101.4	98.9	80.4	116.9	101.7	84
9.67	8.91	98.9	97.8	104.4	106.4	105.1	108
7.05	7.89	99.8	98.6	96.5	109	104.6	96.1
10.1	8.82	129.3	99.1	96.4	100.1	102.8	91
5.11	8.77	103.5	107.5	89.7	98.2	98.1	101.2
6.79	10.6	95.7	87.5	94.9	104.4	111.8	109.1
5.9	9.98	101.7	107	106.8	123.2	122.5	116
11.56	14.07	106.9	95.2	107.1	95.5	92.8	95.6
4.89	10.65	100.5	104.9	101.1	112.6	106.9	107
5.39	10.53	102.2	100.6	110.3	101.5	103.6	103.9
6.4	13.14	96.4	97.9	102.7	99.9	98.9	100.4
9.26	9.23	104.6	107.2	112.6	112.5	99.1	99.4
4.2	6.85	97.7	98	99.1	101.2	100.7	102.5
7.24	23.01	106.6	102	105.4	97.3	91.3	95
6.4	10.35	100.2	90.5	100.8	103.2	104.3	104.5
4.35	9.73	99	88.2	95.7	104.4	98.9	105.8
8.9	11.66	91.4	92.7	91.9	101.2	109.9	99.6
5.52	13.07	95.3	100.8	106.3	95.3	102.1	99.5
5.1	11.08	97	104.1	96.7	107.7	116.9	111.6
9.89	12.44	108.6	97.8	102.2	102.9	102.4	108.1
5.31	10.95	111.3	104.2	120	100.2	103.5	101.8
7.21	12.41	94.9	91.1	92.6	108.8	108.9	107.2
7.71	15.49	91	94.4	86.8	91.3	99	100.2
8.91	7.89	97	99.3	102.1	115.4	104.6	104
7.2	10.26	91.9	99.4	108.1	103.1	94.7	94.2
7.52	9.13	95.7	93.6	100.5	103.9	96.5	116.1
7.85	11.37	104.8	93.5	104.9	102.8	98.7	112.6
6.6	10.07	105.4	109.7	107.9	94.4	103.2	91.9
7.83	12.12	107.6	94.4	88.9	82.1	98.6	116.9
6.2	11.18	108.1	99.8	102.2	103.6	103.8	109.4
7.84	9.83	123.5	120.3	122.9	76.6	70.9	72
8.88	10.11	98.9	97.7	106.8	99.3	93.1	110.6
6.6	7.01	111.9	108.4	98.1	122.7	111.6	121.4
8.69	9.53	105.5	97.7	98.1	97.3	113.2	98.3
9.04	13.05	102.4	107.8	107	103.2	103.3	102.1
8.98	16.73	100.3	98.5	94.9	103.4	102.7	115.3
8.22	8.21	100.9	95.5	95.3	118.6	107.1	113.3
7.85	9.18	99	102.2	95.2	103.7	111.3	113.9
8.91	8.17	107.4	94.8	106.8	106.5	109.2	104.2
4.54	12.01	94.1	94.2	97.5	102.7	97.3	103.2
7.94	10.7	107.5	103.8	103.5	106.2	103.5	102.6
8.46	10.5	100.6	110.9	98.7	110.2	110.7	106

5.69	6.8	107.8	99.9	103.5	103.4	102.8	103.6
8.28	11.23	104.3	99.2	107.5	96.7	100.1	95
8.94	11.59	98.1	98.7	107.3	102.5	100.6	101.3
7.08	7.77	98.7	99.1	109.8	105	115.8	108.2
5.9	11.55	101.5	96.2	96.4	110.7	108	114.1
8.43	11.42	102.8	98.5	104	102.1	100.3	101.8
8.44	7.69	107.6	99.3	100.1	116.5	112.8	124.2
4.63	9.91	105.9	101.2	95.3	103.9	97.1	94.9
7.68	8.01	104.7	94.4	110.1	101.8	94.9	104.8
7.85	14.06	98.9	104.8	98.2	101.9	103.1	101
8.31	10	99.1	88.4	98.6	103.7	106.1	113.2
5.99	12.28	99.5	95.1	96.1	106	103.7	103.7
6.52	11.42	89.1	91.7	85.5	94.3	97.4	93.8
5.91	10.1	91	91.6	90.8	103.8	104.3	107
7.69	10.43	99.7	103.7	99.3	97.7	105.1	108.4
8.28	11.05	105.8	102.4	102.1	102.2	98.8	93
6.14	9.69	94.2	95.7	98.3	102.1	100.9	104.8
5.55	10.56	105	88.2	104.1	109.3	92.2	92
7.03	8.78	100.2	101.1	103.9	106.3	97.2	104.2
9.5	9.28	92.4	91	100.1	106.4	100.7	104.2
9.38	10.54	104.3	101.4	100.3	106.3	107.6	105.4
5.5	7.27	98.3	95.1	105.5	112.5	111.9	109.2
8.15	10.78	101.7	102.2	101.8	102.8	105.2	101.4
6.74	10.36	95.7	103.4	115.4	92.2	107.3	98.6
7.14	12.16	103.6	93.8	96.6	93.3	100.4	103.3
5.38	9.78	110.4	109.4	105.6	110.4	100.4	99.9
11.27	50.19	112	117.1	112.7	108.9	108.8	107.4
9.52	10.18	98.4	101.5	98.3	101.3	100.4	106.3
4.44	10.33	104	102.5	99.1	133	124.1	114.2
6.73	9.85	100.2	98.2	99.3	94.7	90.7	101.4
7.09	9.32	101.8	100.6	78.4	106.2	107	109.9
9.77	8.32	101.1	102.4	110.6	108.8	110.5	107.2
5.38	9.84	101.4	95.9	95.2	98.3	100.4	101.9
9.31	8.58	105.1	102.7	103	101	96.5	90.5
8.91	10.87	102.1	102.4	100.2	113.2	104.3	113.2
8.73	8.35	85.6	92	94.7	109.8	103.9	92.7
5.21	9.47	102.6	93	102.2	98.6	97.1	98.9
5.4	71.41	83.4	99	102.3	97.8	93.9	87.5
9.17	10.45	99.7	84	105.3	117.4	116.4	122.3
8.31	9.49	105.2	102.1	100.3	98.8	100.3	107.2
6.7	9.53	104.2	111	99.6	112.2	106.2	106.4
8.51	11.59	98.7	96.6	96.3	96.6	100.7	99.1
6.7	10.7	84.2	85	89.6	104.4	99.6	106.3
4.81	10.55	105.7	104.6	103.8	112.3	110.3	110.5
7.42	10.73	80.5	80.9	72.8	84.2	80.3	84.3
5.35	8.54	104.9	99.8	106.8	104.6	104.2	103.4
7.33	6.62	100.4	94	103.4	94.2	81.7	97.7
5.48	7.69	100.8	93.7	89.8	99.1	101	94.9
6.47	5.43	111	101.2	95.7	93.2	94.4	107.5
6.18	12.26	104.3	100.1	100.8	98.1	99.9	94.6
8.13	5.14	110.6	108	80.7	125.8	104.6	123.7

8.92	7.77	102.3	93	94.2	105.7	117.8	113.9
6.79	13.27	101.9	96.3	100.4	94.6	96.9	104.6
6.23	14.05	105.3	90.7	110.2	89.6	98.9	91.9
5.85	8.21	99.8	99.2	98.3	98.2	107	102
9.14	9.18	102.2	90.1	94.3	102.5	112.2	101.7
6.1	8.3	97.2	96.8	101.7	94.4	103.5	103.8
4.54	9.4	104.1	95	100.6	113.5	115.6	110.7
5.34	6.74	101.9	99.5	95.9	99.8	99.6	107.6
5.97	8.09	95.9	100.5	113.3	107.2	105.6	102.7
10.11	8.03	103.9	99.5	105.2	113.3	110.5	109.5
5.92	10.27	96.8	100.5	112.1	105.8	116.2	126.7
8.03	8.35	94.4	91.5	107.7	100	113.9	103.7
4.94	10.49	93.6	83.6	92.8	99.5	98.4	104.4
4.58	7.89	104.7	89.4	99.8	110.1	107.4	108.1
8.22	9.64	94.5	90	96.7	99.5	103.2	99.1
4.93	13.23	49.6	50.4	53.1	150.1	144.6	142.1
8.12	24.76	96.3	100.1	97.7	123.1	123.4	121.2
8.32	8.08	109.7	97.6	99.5	104.7	100.7	110.6
9.92	6.83	101.1	106.7	108.8	105.3	101.3	109.7
5.29	14	110.6	96.2	105.6	108.5	102.5	114.4
8.13	8.92	96.5	90.6	98.8	108.1	112.2	105.3
9.66	6.23	100.9	106.3	100.2	96	102.7	102.2
9.42	13.43	94.6	98.5	102.2	104.6	108	105.1
7.28	7.37	124.9	80.8	94.3	119.2	89.4	108
9.77	9.78	101.1	99.2	96.6	113	102.5	104.8
8.63	7.73	110.1	102.8	102.1	84.2	81.1	91.5
4.2	9.96	89.9	97.5	106.7	112	103.5	101.8
9.48	10.12	125.2	106.9	93.6	105.8	95.6	99.8
4.65	9.5	112.7	104.7	118.3	108.1	112.7	113.4
5.55	4.82	102.1	96.8	90.4	101.5	93.3	108.2
7.58	6.41	103.5	97.3	106.4	122.5	119.4	130.3
7.15	11.01	157.3	160.2	167.1	59.9	53.5	66.7
9.13	9.71	101.4	94.2	100.5	106.4	101.4	105.7
6.92	9.18	115.8	97.7	94	88.4	115.9	100.9
6.64	8.54	109.4	82.3	104.7	81	98.2	95.8
8.56	10.34	108.1	102.3	100.5	112.9	114.8	112.1
9.57	8.95	104.8	109.2	100.5	94.2	107.5	109
10.27	9.47	113.4	97.2	96.9	101.2	94.7	105.5
7.12	8	101.4	114.7	106.9	97.5	98.4	101.4
8.87	9.22	109.2	110.6	110.7	99.9	98.5	99.6
7.12	6.74	88.7	104.4	101.1	115.9	89.8	116.4
6.52	23.15	98.9	104.4	99.8	100.1	93.2	91.6
8.87	13.79	96.8	101.4	99.9	103.7	100.8	104.4
4.82	12.63	97.4	107.6	102.7	104.7	104.6	109.4
9.47	8.41	83	72	71.6	101.3	107.5	104.1
5.66	12.02	100.1	102.5	100.1	94.2	103.4	97.6
4.42	9.77	100.2	101.3	95.9	98.7	106	101
5.03	6.9	96.1	88.8	103.9	97.2	91.1	90.2
5.64	8.21	95.7	90.4	111.3	100.3	112.4	107.6
8.54	6.34	100.4	92.3	97.7	102.3	102.2	109.9
7.56	8.97	95.7	96.4	97.1	101.4	96.3	102.7

7.4	7.14	91.2	86.8	84.1	101.5	91.5	91.5
5.69	13.17	103.9	95.7	128.6	103.2	101	107.5
6.29	11.12	107.6	99.7	95.7	103.2	92.1	98.6
5.8	6.35	98.3	104.2	102	96.2	97.2	104.4
5.3	7.55	98.9	104.7	102.7	86	92.3	98.4
5.08	7.5	102.6	96.7	102.1	108.7	107.4	111.3
6.7	9.87	85.7	95.8	96	88.4	87.3	92.1
6.49	6.74	106.9	104.2	98.6	101.1	96	98.6
8.43	5.46	97.3	94.7	97	106.2	96.2	103.3
6.95	8.64	102	99.7	98.3	94.5	102	109.2
8.65	4.93	98.5	87	100.4	90	99.7	109.9
7.53	4.1	94.6	103.9	112.1	97	109.6	91.6
6.19	9.92	98	102.6	100.7	100	97.5	99.4
7.66	5.01	89.9	102.9	99.2	104.9	105	112.8
6.84	10.3	95.6	90.5	123.7	106.8	115.8	130.3
8.51	8.89	103.7	104.3	108.4	106.1	104.5	99.2
7.46	8	109.8	82.7	97.8	96.3	89.1	93.2
5.24	9.27	84.7	93	98.7	106.7	107.3	99.8
10.15	9.23	100.8	99.6	101.2	105.6	110.8	109.2
8.76	11.29	96.5	92.4	96.3	110.5	112	112.5
4.88	14.05	91	95.5	90.1	100.7	97.1	104
9.25	6.89	97.5	97.1	94.7	99.3	102.7	116.9
8.79	5.95	92.4	86.6	89.4	91.5	77.8	243.8
4.97	6.59	98.2	101.8	91.1	99.9	99.6	100.5
6.33	4.99	100	109.9	113.9	93.4	96.7	92.9
7.33	7.87	95.1	89.7	93.1	108.4	98.7	108.7
7.23	6.28	99.6	110.4	100.6	85.8	96.6	100.8
8.88	5.42	84.6	88.2	93.8	105.8	119.5	103
5.58	8.28	100.9	100.3	106.9	103.9	94	98.4
8.44	7.22	88.2	88.1	91	117.3	110.8	113.9
7.24	9.87	112.9	100.2	106.3	66	63.2	60.8
8.53	8.94	105.5	87.6	100.3	91.5	101.9	100.8
6.74	8.39	88.6	87.7	93.6	99.1	104.9	103.4
6.61	9.45	100	95.1	87.7	90.2	89.2	128.2
4.92	5.73	94.2	94.4	94	71.5	111.6	119.8
7.31	10.15	102.6	103.5	97	91.5	98.6	96.3
9.85	7.53	96.4	86	98.7	96	94.1	108.9
5.68	5.17	128.3	120.1	104.2	101.4	97.8	90.7
5.3	10.92	103.9	101.4	105.5	100.9	97.6	101
6.95	10.12	89.6	90.8	99.1	103.6	102.1	106.8
9.54	6.95	110.7	99.5	103.2	102.2	103.3	102.7
5.27	11.53	103.2	100.8	102.2	103.8	108.8	108
5.88	6.14	105.3	103.2	103.1	97.5	98.1	100
7.99	9.25	89.6	88.2	92.7	122.9	126.7	128.2
6.01	9.12	96.1	106.3	98.2	106.5	114.9	93
7.43	9.96	100.9	98.1	93.5	99.2	101.7	94.6
7.97	11.78	94.8	94	93.7	105.9	106.9	108.4
8.09	7.47	102.1	105.1	111.3	97.7	90.8	94.3
7.55	7.98	115.2	105.7	99.8	110	100.5	91.4
9.5	12.28	102.8	102.4	101.7	110.6	108	106.8
7.93	9.08	101.2	105.1	103.5	98	99	97.4

4.82	6.37	99.2	95.8	102.8	101.1	99.8	102.8
8.02	10.18	102.8	95	98.3	105.4	105.7	100.8
9.14	9.16	109.6	105.8	98	112.7	92.6	94.9
7.12	8.91	93.9	103.4	110.7	94.4	99.5	104.1
4.55	10.33	99.1	95.7	96	108.1	109.1	109.6
6.14	12.65	108	104.3	95.8	103.5	102.3	106.2
7.23	9.93	96.8	98.7	100.9	111.3	100.3	113.4
7.81	12.44	98.6	99.1	94.5	108	109.9	106.7
6.04	10.1	105.2	102	105.6	84.3	108.2	101.8
6.77	9.59	105.8	98.7	103.1	94.1	98.1	95.4
8.34	6.81	101	94.6	97.6	108.5	118.5	121
7.56	8.27	95.9	85.2	95.4	114	139.8	142.1
9.07	6.59	98.2	88.2	105.6	97.2	111	104.4
6.89	7.93	115	109.2	98.6	103.9	102.8	115.3
7.18	10.27	100.4	98.7	98.7	102.4	107.6	105.5
7.56	7.75	83.6	105.3	100.9	97.2	103.3	98.4
5.49	6.95	87.4	97.5	91	107.4	98.7	116.1
8.53	9.14	101.8	99.5	101.7	102.1	104	106.6
5.34	9.03	105.1	102.4	105.4	95.4	90.2	95.8
4.98	11.18	98.8	104.6	97	106.5	96.3	102
7.2	7.9	98.4	98.7	99.5	94.7	98.6	104.8
5.19	8.57	106.3	102.8	111.2	103.1	101.6	104.8
9.69	10.96	106.7	96.5	115.1	94.8	102.1	113
6.51	11.79	102.7	97.5	109	91.3	95.7	100.5
5.49	8.49	100.8	97.5	94.8	101.9	103.4	106.8
6.43	7.51	96.6	100.7	87.2	115.3	100.7	109.2
8.63	8.56	100.7	99.9	105.2	106.1	102.2	99.8
7.2	8.39	93.5	91	79.7	82	78.5	79.8
6.81	9.77	100.1	104.1	100.8	89.1	88	95.9
7.11	11.98	99.9	107.9	105.5	94.3	98.1	90.3
6.55	7.97	90.7	95.3	101.2	107.1	104	99.2
7.03	10.34	101.1	97.3	105.1	97.4	95.9	91.3
5.07	8.43	102.8	88.4	96.9	118	103.3	119.7
9.72	9.23	89.1	90.8	104.7	110.1	116	127.5
7.01	5.52	112.9	121	119.9	90.2	92.9	97.2
8.62	5.96	105	102.3	98.4	104.7	98.5	103.4
5.54	8.87	95.6	96.4	100.9	100.3	103.5	103.1
9.6	10.4	109	100.8	105.9	96.2	99.8	114.4
6.73	9.91	100	99	97.1	102.9	103	101.7
6.74	8.97	101.8	98.4	108	97.2	97.2	93.8
7.37	7.44	114.3	100.9	101.4	98.3	98.2	100.3
4.96	13.1	105.4	104.7	100.2	80.5	95.9	87.2
8.68	10.89	97.5	97.1	98.2	101.4	101.5	100.8
10.1	7.54	95.6	98.2	92.7	105.5	103.4	105.3
5.17	8.76	85.9	92.6	86.3	72	82.7	255.5
9.67	8.82	102.2	104	99	94.3	95.5	103
5.03	8.68	107.1	95.8	92.5	101.4	93.2	112.7
10.32	8.3	103.6	96.3	106.6	109.3	112.2	111.6
6.8	7.42	102.8	94.6	107.2	94.5	112.7	100.3
5.74	7.82	104.7	100.6	101.7	108.7	100.9	107.7
5.67	6.95	102.9	93.3	108.8	89.1	96.9	87.9

6.07	9.16	91.4	90.9	92.8	109.9	110.2	117.3
7.24	7.84	107.8	103.1	102.1	112.6	112.1	105.6
7.17	7.49	96.5	99.4	89	106	94.5	104
9.6	7.33	103.2	101.1	104	107.2	102.9	99.2
4.98	8.22	101.8	95	87.5	100.9	120	110.1
5.01	8.24	95.5	94.4	85.8	125.5	92.1	129.8
6.55	9.02	107.6	104	72.2	110	114.2	107
9.03	12.23	97.3	104.6	100.8	94.8	98.8	99.6
8.02	9.07	109.5	90.2	114	106.9	107.9	106.8
5.43	5.01	105.8	102.7	89.1	124	135.8	129
5.47	11.86	103.2	94.1	100.9	94.5	96.7	97.3
9.35	13.42	101.7	104	103.4	98.9	93.4	97.5
6.25	5.17	99.1	99.6	102.2	95.5	95.4	94.1
8.29	10.12	92.1	103	98.7	90.7	91.7	87.5
6.23	8.04	100.2	101.9	87.6	106.5	91.3	96.3
5.38	18.12	89.3	88.9	85.2	89.6	88.8	81.9
5.27	10.46	103.8	106.2	106.6	99.9	107.3	101
9.26	7	112.5	94.3	102.6	106.7	103.2	105.2
8.91	7.87	101.1	99.9	99.3	87.8	91.9	104.6
5.12	11.44	104.3	102	101	92.2	94.6	102.8
8.72	6.6	105.4	100.3	94.3	104	108.6	95.9
4.48	7.58	105.8	98.2	106.5	119	103.1	99.9
6.39	8.62	105.6	98.6	106.2	103.7	91.5	102.3
8.4	4.63	109.2	94.5	106.4	97.7	95	93.6
9	7.76	110.4	114.4	110.9	92.2	93.8	96.3
7.36	8.09	98.1	99.3	103.5	117.1	107.6	115.5
8.82	12.09	97.9	96.6	93.4	102.2	108.9	102.4
8.25	8.79	105.1	95.8	96.6	102.8	103.5	96.1
8.34	6.12	98.6	99.9	104.7	99	103	100.7
9.54	16.69	96.3	98.4	100.1	98.7	101.4	106.5
10.15	12.47	99	100.6	102.7	103.5	106.5	99.5
5.85	9.63	94.2	100.1	97.4	114	114.1	100.8
7.71	9.23	97.4	97	104.1	108.6	102.4	94.3
7.3	12.23	107.3	92.9	97.3	89.5	89.6	96.9
5.94	8.02	92.8	99.9	77.3	97	82.3	96.1
5	5.83	98.9	79.7	99.6	106.1	105.7	109.9
6.32	16.55	91.3	79.3	104.4	92.7	104.3	99
6.8	14.87	95.5	95.1	94.1	93.6	96.2	103.7
9.04	8.33	89.3	98.6	95.3	122.1	97.8	121.8
5.74	8.45	100.9	91	100	99	102.8	98.6
8.13	8.01	98.4	102.1	94.1	104.8	94	94.3
8.68	8.68	91.4	100.7	105.4	118.1	81.8	137.4
9.09	9.04	101.2	103.3	99.3	115.5	113.4	111.4
7.31	6.76	95.4	96.2	95.9	103.3	98.7	110.2
6.34	9.86	97.6	100.5	99.9	104.4	98	105.9
10.33	11.39	106.1	104.2	108.5	111.1	115	110.2
7.61	6.3	95.1	100.1	111.4	111.6	103.9	105.3
6.38	8.25	96.7	91.6	94.7	111.7	105.1	116.6
5.87	7.13	100.2	92.2	91.3	100.3	103.3	102.6
4.87	8.06	100.6	90.5	118.3	127.8	109	107.9
4.63	3.56	102.8	121.5	104.1	100.8	112.8	101.9

8.66	5.5	106.9	99.6	106.4	98.2	101.8	95.2
8.72	8.78	112.2	102.7	110.1	97.1	99	102.5
7.25	8.82	114.2	109.4	97.1	91.6	95.6	84.6
5.03	6.02	93.7	109.7	89.1	117.4	103.9	108.7
8.88	6.5	108.4	96.5	97.4	106.7	94.4	94.6
4.96	10.76	96.5	100.3	95.9	99.7	102.1	103.3
5.76	10.54	115.5	104.6	110.9	88.1	86.8	92.4
9.66	8.62	108.5	99	101.7	101.5	102	110.2
5.77	10.75	98.4	101.2	100.2	109.4	110.7	115.8
8.03	12	102.2	98.1	92.9	102.2	98.4	100.8
7.56	5.48	112.5	99.7	104.4	99.9	88.5	97.3
8.6	6.59	93.1	95.3	89.8	105.8	99.1	102.5
4.97	5.54	66.8	83.5	97.4	125.2	120.9	117.4
8.6	7.19	99	97.7	93.8	112.5	107.4	111.9
8.76	7.43	95.1	102.7	107.4	104.4	115.7	111.2
5.24	4.4	93.4	112.5	83.9	103	114.1	92.7
9.33	6.32	102.3	101.1	104.2	96.2	102.4	110.7
5.96	4.93	103.7	103.8	102.7	104.7	95.9	100.8
8.24	7.96	94.2	95.3	93.9	95.6	103.6	92.3
8.63	5.3	96.1	92.1	92.9	113.6	126.1	115.3
6.1	6.32	90.7	90.7	91.8	111.5	111	114.4
8.22	5.78	92.2	109.8	108	99.5	84.8	99.2
9.73	6.43	106.3	100.5	109	106.8	111.3	113.3
6.02	14.55	95.7	98.5	96.2	102	98.7	100.8
9.13	13.02	102.5	110.7	99.7	100.3	99.8	96.7
9.17	9.55	133.8	92.7	91.4	98.8	128.7	102.4
8.68	9.56	92.3	112.8	112.9	117	110.9	108.4
8.56	11.98	95.9	92.8	92	110.4	111.5	112
8.18	9.14	91.5	93.4	109.8	85.6	86.4	86.9
5.78	7.93	113.3	109.2	109.2	105.9	93.8	99.8
5.82	9.24	112.8	108.9	103.2	96.2	93.8	95.2
5.06	4.32	108.3	115.6	97.1	87.7	118.7	98.1
5.34	10.11	108.3	103	97.3	70.7	77.4	74.1
5.12	7.1	109.4	99.2	103.7	113.6	115.1	110.5
9.82	9.35	92	88.7	95.5	98.3	101.2	98.7
4.98	7.39	133.4	125.8	123	76.3	75	76.2
7.25	7.54	107	113.1	105.5	89.3	100.4	100
5.07	7.45	112.9	100.1	94.1	95.3	95	102.5
4.4	7.48	104.8	110	95.6	101.7	112.6	106.8
5.24	8.96	102.3	89.2	103.9	112.7	103.9	107.6
5.19	7.91	114.1	107.3	117.3	94.4	86.6	99.5
5.12	5.23	113.4	94.2	103.1	107.1	120.2	109.4
8.03	8.61	122.1	111.3	117.1	97.4	90.8	95.7
6.58	6.33	65.1	86	71.3	120.4	107.6	125.6
5.27	8.3	94.5	106	99.5	98.6	105.4	98.6
8.15	6.37	103.7	106.1	92.8	112.8	103.5	116.1
5.94	12.01	100.3	107.1	105.1	110.1	103.9	105.8
7.61	8.19	108.4	94.6	98.7	100.4	102.5	106.9
6.87	7.01	96.4	107.7	98.9	105.4	101.6	102.8
8.91	10.88	112.1	86.7	105	93.9	100.5	104.9
7.18	8.65	85.3	83	79.5	119.9	114.4	116.4

8.02	4.75	101	104.4	94.1	104.6	97.4	123.3
8.47	8.07	106.4	107.8	100.9	109.1	113	102
4.61	6.93	100.7	95.9	96.6	105.2	105.2	104.3
9.41	10.09	100.2	99.9	105.4	114.5	108.3	112.6
5.29	7.65	100.3	98.3	104	93.8	91.2	101
8.76	8.24	102.6	115.4	109.1	91.8	87.2	90.9
6.32	7.26	96.8	100.3	106.5	95.1	98.9	99.2
5.52	9.21	102.1	92.9	103.3	105.6	106	93.1
7.34	7.45	94.7	88.7	84.4	115.8	113.5	112.8
5.39	6.86	101.4	101.2	109.9	96.6	96.3	92.4
5.59	9.89	107.1	99.5	97.7	99.9	92.6	104.8
6.14	12.24	72.6	151	114.4	116.5	111.8	90.7
8.66	7.86	113.8	99.8	95.5	94.9	84	105.8
4.58	8.23	91.5	98.6	98.7	111.8	106.3	108.5
7.65	9.29	100.4	97.8	104.4	106.5	99.2	101.1
5.47	7.94	106.1	104.5	105.4	103.8	100.4	99.8
7.33	11.16	132.7	131.2	126.7	61.2	62.9	63.1
5.36	9	101.5	100.5	102.1	95.9	101.2	103.9
6.49	3.05	110.4	98.8	110.2	99.9	77.4	95
7.05	8.01	107.8	114.4	109.3	104.7	101.2	105.6
8.54	5.47	109.1	92.1	107.8	110.4	114.1	106.9
5.02	10.23	99.2	98.2	101.4	103.6	100.7	106.6
9.57	16.3	97.8	92.2	105.4	100.9	107.9	105.5
6.04	7.54	124.8	98.6	123.6	82.2	85	84.2
5.25	4.78	113.3	93.3	110.9	106.4	86.4	100.3
6.04	5.51	99.6	105.3	93.9	92.7	92.4	105.4
4.48	7.38	69.3	81.8	84	108.4	113.2	119
7.36	2.93	91.5	94.4	111.6	92.3	116.6	107.8
5.67	3.42	95.9	115.8	122.7	117.7	103.3	104.7
5.41	4.7	102.4	111.1	89.8	92.9	101.5	99.6
8.69	11.2	110.1	109.9	101.2	99.6	105.5	99.1
5.76	4.5	98.5	104.6	91	98.7	113.1	107.1
8.63	6.15	99.1	104.2	88.5	110.4	92.5	105.2
5.47	6.96	101.6	97.3	103.2	100.5	101.8	100.3
9.57	7.17	101.9	102.4	101.4	101.6	103.8	115.1
5.88	7.08	93.8	104.5	91.5	115.7	61.7	118.8
5.41	5.13	107.4	78.8	122.1	85.6	95.4	98.4
10.26	10.11	108.9	88	99.7	99.9	102.7	105.6
11.15	8.99	91.6	110.4	113.1	108	96.7	110.2
8.95	8.77	90.2	90.5	120.1	101.4	97.2	103
7.02	7.67	100.1	96.9	95	110.2	108.3	107.7
6.21	7.28	79.3	74.3	80.7	109.7	107.1	105.3
7.61	5.88	96.8	111.6	94.3	85.4	85.1	76.5
6.74	6.71	98.9	99.2	91.1	89.3	84.5	88.7
8.16	7.06	97.9	95.5	97.8	98.4	103.3	111.6
6.98	7.77	116.1	105.8	111	95.3	99.9	94.9
7.84	7.09	93.7	98.5	103.4	93.1	105.5	97.3
10.56	5.84	109.2	113.8	105.9	91.8	95.4	90.4
7.01	4.22	98.9	92.2	109.8	107.2	97.4	102.7
7.64	7.16	117.9	91.7	107.7	108	107.5	96
9.25	12.91	115.7	99.4	97	105.9	102.3	111.1

8.35	7.07	103.8	97.9	113.1	89.1	92.6	72.5
7.21	13.32	103.8	99.7	100.2	93.8	95.4	97.5
6.04	7.92	108	102.9	107.2	105.8	106.7	106
6.21	9.49	98.5	98.6	95.1	97.8	106	101
9.89	7.28	104.2	92.2	100.9	100.3	105.5	107.1
5.82	7.41	102.3	99.7	100.5	94.7	86.6	88.4
4.68	8.35	103.1	99.7	109.2	107.3	105	104.1
9.2	7.8	96.3	98.7	101.2	103.5	103.1	105.9
5.62	6.68	108.6	97	95.9	118.8	105.8	100
4.44	8.99	90.8	87	79.1	100.2	99.2	94.5
9.26	7.34	103.5	99.5	97	113.1	105.9	105.4
5.57	5.1	94.8	89.9	93.6	105.7	95.9	111.8
7.52	5.86	107.8	102	99.5	96.1	101.8	92.8
6.14	6.51	78.7	91.6	105.5	115.1	156.7	95
6.28	8.03	97.6	100.4	107.3	101.5	101.1	100.1
8.13	6.31	115.5	106	94.5	91.6	91.7	103.9
5.08	10.48	95.7	100	104.9	121.7	117.5	117.5
8.79	8.74	94.2	100.6	91.6	109.5	108.6	115.8
6.87	8.09	92.7	79.5	100.6	115.8	102.4	110.8
6.54	5.12	93.2	82.1	97.3	119.3	110.1	127.6
5.73	7.11	93.1	87.6	95	101.6	101.6	108.6
5.38	5.24	105.5	110.5	115.2	80	83.5	82.4
8.02	8.16	101	91	101.7	99.8	94.6	98.9
7.58	8.86	119.1	92.4	100.1	133.3	129.8	92.4
5.64	6.31	96.5	92.5	98.4	102.3	106.4	103.9
8.72	16.5	94.6	106.7	103.3	99.7	91.2	103.6
5.08	6.02	106.8	101	103.1	109.7	102.8	103.5
7.15	6.96	107.3	105.6	106.9	100.9	100.8	96.7
8.98	4.49	96	98.5	87.6	107	108.7	105.6
9.67	5.97	94.2	110.9	95.3	133.2	133.2	123.2
6.9	7.16	101.2	96.6	92.4	105.5	103.2	93.3
5.55	5.91	108.3	106.9	106.6	104.7	110	106.1
9.7	5.45	106.8	128.2	85.1	88.9	119.8	88.1
9.64	6.85	96.9	117.5	100.5	106.8	106.7	103.5
5.43	6.93	103.6	90.2	89.8	83.3	91.2	79.5
8.76	2.8	99.8	94.6	94.9	112.9	106	104.7
5.68	7.17	100.4	111.2	106.5	87	86.2	86.4
5.21	4.36	99.7	103.2	85.6	77.9	89.2	103.7
6.09	6.89	96.2	88.6	98	108.7	110.3	100.6
6.64	7.11	91	96.7	102.2	114	115.3	116.8
6.65	9.4	90	90	94.4	124.2	123.2	122.3
8.38	7.85	105.8	108.4	105.5	115.6	121.4	116
5.73	8.11	102	96.4	93.9	107.6	100.6	103
6.35	7.76	103.6	97.1	85.9	105.9	107.1	108.5
4.37	7.13	106	96.4	103.1	100.3	101.1	103.4
6.29	5.52	91.6	105.2	97.3	99.6	100.9	87.6
6.33	7.66	87.9	99.4	120.8	101.3	98.5	134.6
9.31	8.21	98.4	103.6	109.3	97.7	101.9	108.9
7.28	8.55	86.1	95.1	96.3	96.6	91.1	103.7
5.85	6.01	101.9	97.1	94.4	89.8	117.1	99.4
6.1	5.28	104.2	99.8	123.9	92.4	104	94.1

8.02	6.34	110.6	100.5	107.6	102.2	93.5	91.9
8.94	10.27	96.3	100.9	107.3	98.9	97.4	97.6
5.3	6.7	90.3	94.8	102.3	119.9	113.5	105.3
6.07	5.33	104.8	89.4	94.1	98.2	96.9	106.1
11.18	8.06	88.8	86	88.2	111.2	111.1	106.7
8.24	7.93	99.8	98.2	96.7	104.8	103.6	102.9
6.37	8.06	107.6	97.2	93.8	100.4	95.9	120.7
9.25	6.57	102	101.1	99.8	93.9	104.4	92.5
9.11	10.11	93	99.4	91.6	105.8	107.8	103.2
5.45	5.53	108.5	93.2	90.1	107.8	108.5	105.3
5.44	8.58	92	98.2	101.2	97	98.2	103.1
7.83	18.99	99.8	98.3	96.3	99.1	97.4	93.1
6.39	4.61	87.3	73	100.1	76.6	105.4	94.4
8.02	4.97	106.7	102.1	102.9	101.8	110.3	111.7
4.23	7.64	105.9	121.6	114.3	134.3	138.4	131.6
5.07	6.83	73.8	74.1	71.6	115.6	121.4	118
6.48	4.73	75.8	105.2	89.8	113.4	116.7	108
5.54	6.86	101.8	106.6	107.9	93.7	90.7	99.8
6.83	6.94	100.6	102.5	114.4	71.3	80.9	85.5
6.23	5.99	89.3	95.6	101.4	114.1	102.6	118.8
9.48	6	132.3	87.1	102	97.5	144.2	99.9
8.46	6.75	98.7	110.1	108.7	95.8	95	88.4
7.23	9.5	90.1	115.1	109.6	100.2	106	107.5
6.3	6.27	110.2	92.7	82.6	115.6	100.8	106.2
9.2	18.2	97.3	98	102.7	102.4	101.5	106.7
8.81	6.58	98.4	107.4	91.2	105.9	94.6	95.1
11.96	7.72	88.3	102.3	104.7	99.3	98.4	106.7
5.47	4.31	105.5	96.4	102.3	114	108.9	114.5
6.62	4.75	92.3	94.9	94.9	92.9	93.3	98.5
7.53	6.13	104.7	90.5	109.8	103.9	106.1	96.3
6.27	5.4	86.3	106.3	93.8	110.4	99.5	124.3
9.17	6.66	93.2	114.8	97.6	121	115.9	108.5
9.22	9.53	101.8	96	98.8	104.9	100.2	103.2
8	6.17	109.3	110.3	93.8	108.1	105.6	105.9
5.03	5.74	108.9	104.7	99.7	100.2	113.2	123.6
5.68	7.72	79.2	115.1	107	114.4	113.9	119.3
6.44	7	90.1	105.5	98.4	107.5	111.2	117.8
7.28	6.86	102.9	112.8	94.9	103	88.8	99.8
6.28	5.73	103.8	90.2	93.6	108.8	117	118.6
4.64	7.68	104.2	103	95	101	101.2	99.1
7.71	7.22	100.9	93.2	113.2	99.5	97.7	96.9
9.51	7.88	106.8	101.1	102.4	113.4	105.5	112.4
7.88	7.6	95.2	107.2	99.8	103.9	114.9	107.5
8.21	6.07	98.1	85	95	101.8	107.8	122.8
6.64	9.64	103	96.5	95	108.9	104.8	102.9
10.95	6.26	100.9	100.7	101.9	105.7	104.2	116.3
6.83	8.2	104.9	88.6	87.9	116	115.1	103.6
5.95	5.8	80.8	94.5	95.1	94.3	109.1	98.7
7.18	6.71	99.1	96.5	99.2	109.6	99.7	99.4
9.73	8.7	93.3	98.3	103	112.8	105.3	108
5.96	8.35	94	105.5	104.1	106	107.3	107.7

6.61	7.63	81.1	87	92.2	109.2	109	112.4
8.1	7.2	90.6	93.2	89.5	114.4	102.8	114.6
10.08	6.33	130.9	114.8	122.4	90.8	96.2	100.4
7.96	4.87	91.3	92.8	99.9	101.7	108	103.8
4.74	6.85	103.3	104.1	109.3	104.4	118.3	123.8
9.92	14.44	104.7	101.6	104.8	96.1	98.5	95.3
8.38	8.8	103.9	102.1	98.8	96.7	99	104.4
4.96	8.86	101.9	92.3	96.5	93.6	95.7	100.9
6.96	6.89	112.8	92.1	96.8	111.4	84.1	77.7
6.61	4.66	111	100.8	115.9	100.4	90.8	107.2
9.39	8.69	84.2	101.5	90	125.6	121.2	114.7
5.52	9.14	101.6	106	101.5	103.7	120.7	99.3
7.18	7.94	102.2	99.2	101.2	88.2	96	99.4
5.33	8.66	94.6	99.6	99.5	100.2	99.8	99.6
8.92	9.29	105.9	109	88.1	100	91.4	95
8.02	5.44	109.9	90.3	98.6	93.7	83.6	104.3
7.42	6.57	99.7	98.7	103.6	128.2	125.2	124
7.55	7.11	91.6	98.6	104.9	104.2	99.3	105.9
6.65	5.2	87.9	84.6	100.4	103.2	88.2	99.1
5.99	6.85	102	99.1	102.7	86.5	86	105.5
5.33	7.74	103.2	84.8	101.5	91.8	98.3	98.4
4.97	7.91	98.6	99.2	95.4	115.2	116.4	119.4
9.55	10.18	107.4	104.4	105	108	112.8	106.3
8.47	6.64	98.8	94.2	97.2	111.2	106.1	119
9.76	7.6	95	113.9	94	113.2	104.8	102.9
6.34	7.22	101.8	94.7	98.7	89.2	105.4	120
5.74	7.4	78.7	78.9	79.4	106.5	103.4	99.3
6.98	6.43	112.6	74.4	89.4	95.3	123.3	114.1
7.59	2.89	94.1	91.9	106.4	103.1	91.1	95.8
7.02	8.97	104.4	109.7	106.7	96.4	90	97.6
8.76	7.83	103.1	107.2	112.3	103.7	114.2	103.1
8.34	8.51	83.7	78.9	94	111.8	109.4	134
9.36	11.56	115.2	112	116.8	99.9	101.3	100
8.66	5.62	93.5	99.6	100.7	91.9	105.3	96.2
5.52	3.84	82.3	91.7	113.5	104.3	101.1	111.3
6.6	8.61	97.3	94.9	99.4	100.8	97.5	102.7
5.33	6.28	106.6	101.7	105.7	87.5	113.3	113.5
5.94	5.57	112.9	92.5	105.1	92.8	96.2	102.7
7.53	7.78	97.2	99.4	100.3	111.1	113.6	105.3
7.44	20.18	119.8	119	136.7	80.1	76	85.9
9.32	10.35	105.1	110	104.3	111.8	106.4	113.4
5.59	7.96	73.8	77	74.4	125.4	119.8	116.9
8.6	6.84	105.1	89.4	102.2	110.1	90.4	135.9
5.72	6.25	100.3	95.8	109.5	88.7	108	102.8
9.07	2.85	104.3	97.2	100.8	97.6	108.3	94.8
11.3	6.59	106.6	91.8	103.4	103.6	104	108.3
5.29	7.33	114.6	117	117.1	88	93.5	100
8.13	7.95	99.8	106.1	100.5	102.5	103.6	110
5.15	6.91	86.6	100.5	109.8	101.5	108.4	102.6
7.65	7.82	94.9	84.6	110.4	87.6	110.3	92.8
7.99	6.74	95	98.2	95.1	109.3	112	112.1

5.69	11.22	97.3	101.9	104.4	106.9	111	112.2
4.63	5.94	113.1	103.1	90.6	115.5	111.1	102.8
8.84	5.99	99.1	98.5	105.7	93.4	67.3	108.5
7.84	11.39	97.2	92.8	106.5	89.5	103.5	96.1
5.87	8.34	99.6	91.4	95.8	98.9	100.9	97.4
6.09	9.79	104.3	106.2	108.7	103.7	104	99.9
6.92	5.05	92.3	104	91.6	109.5	112.4	125.4
9.11	7.91	106.2	102	97.2	103.2	106.8	108
5.33	5.32	97.3	94.4	109.4	95.1	122.4	101.2
9.55	8.8	94.8	98.9	98.9	114.4	110.7	110.4
6.79	7.79	89.3	102.6	99.2	95.9	97.5	99.4
9.54	9.3	100.5	99.7	103.2	101	104.1	108.3
7.09	7.7	96.8	103.3	108.7	108.6	102.9	107.4
6.52	3.49	112.7	107.6	94.6	90.9	118.8	87.8
6.73	6.2	104.4	104.5	96.9	99.6	103.6	100.6
6.44	8.07	112.9	100.7	106.6	109.4	113.3	114.9
7.36	5.15	93.7	101.2	105.4	97.9	91.4	91.2
6.07	4.11	99.6	95.4	94.9	94.7	100.2	89.4
8.82	6.67	105.4	111.8	91.4	90.4	96.1	99.1
5.92	8.51	111.6	101.5	100.5	111.3	117	113.9
8.28	5.4	114.8	106.5	87.2	96.8	106	97.8
7.58	6.93	100.1	104.9	92.9	98.4	100.2	131
7.03	5.78	97	100	97.5	98	95.8	97.6
6.92	7.25	95.8	92.7	91.3	102.6	98.3	114.6
7.34	5.46	104	96.8	85.5	103.3	112.7	119.2
6	3.54	86.8	124.5	112.9	83.6	90.4	103.5
9.03	6.21	104.3	103.1	90.7	111.3	109.9	99.2
6.83	7.1	97	93.5	101.8	87.4	100.8	101.2
6.09	5.51	90.1	96	103.9	101.2	107.4	103.6
6.89	6.25	103.7	101.7	104.9	89.5	105	111.3
8.46	3.08	96.5	105	107.9	98.4	100.7	103.4
5.76	7.32	108.3	86.8	99.7	100.3	102.9	98.3
9.22	5.55	102.7	104.6	106.2	106.4	104.1	109.9
8.87	6.53	103.6	95.4	103	100	109.1	107.3
5.26	6.96	87.6	102.3	89	99.6	95.5	104.3
6.21	6.24	79.8	104.4	102	90.9	98	94.1
5.31	7.9	93.4	99.9	96.4	104.4	106.7	108.1
4.92	1.9	120.6	101.1	71.3	119.5	112.5	107.9
6.71	4.71	98.8	103.2	99.7	110.6	97.3	102.3
5.6	6.31	91.5	122.3	82.5	102.4	96.7	125.9
6.8	4.19	110	110.1	111.1	102	106.3	98.8
7.21	6.47	97.7	86.5	89.5	103.6	99.3	100.3
5.68	7.22	92	100.9	97.4	103.3	98.4	101.7
8.1	5.33	101	92.5	98.6	99.4	102.7	99.8
8.78	6.42	92.6	97.3	97.7	97.8	102.3	132
5.76	4.64	104.8	101.6	99.3	99.7	103.2	104.5
8.05	6.11	105.9	83.2	96	102.2	99.1	114.6
4.93	5.67	91.4	94.9	105.1	97	100.7	90.9
6.14	6.88	98.9	93.4	100.1	99.4	95.2	100.9
9.57	5.9	111.8	103.6	97.9	96	91.7	95.3
5.08	5.12	101.5	97.4	92.5	104.7	101.2	108.8

6.4	5.49	97.1	100.9	98.3	97.9	109	114.6
7.64	3.14	118.6	92	87.7	91.3	99.6	115.4
5.69	3.09	106.1	93.8	114.9	105.7	98.2	100.5
5.77	7.48	108.7	102.4	109.4	94.2	101.3	102
5.25	6.37	97.4	105.6	110.1	110	104.7	112
5.19	4.29	119.5	117.4	120.2	90.8	86.2	89.4
10.35	6.76	103.3	108.7	102	103.7	95.9	102.3
7.15	1.81	74.9	111.6	93.7	118.8	99	96.9
8.15	7.07	98	88.6	102	107.5	89.8	114.6
4.65	7.59	87.8	99	105.7	114.5	101.6	112.1
8.88	9.59	98.7	112.2	112.2	97.2	119.4	99.1
6.28	5.4	82.1	109.2	104.9	98.6	120.3	109.1
6.13	5.8	81.1	81.9	84.2	94	93.6	88.1
7.88	5.18	88.1	92	118.6	97.8	90.7	103.4
6.21	7.5	106.9	108.5	101	106.7	101.8	102.4
7.85	6.17	80.4	110.3	105.2	113.1	97.5	99.6
5.11	8.62	99.5	89.9	94	111.1	101.4	110.5
7.36	3.2	100.3	124	111.2	103.1	109.2	103.4
6.64	6.7	101.9	97.7	101.4	96.4	101.9	100.9
7.06	6.2	102.6	93.5	99.1	110.8	103.6	98
5.99	5.57	113.2	92.6	96.1	96.6	92.4	96.5
5.34	5.85	114.8	95.5	86.8	105.8	95.9	113.8
5.77	4.6	76.7	74.9	82.7	108.5	143.2	124.9
6.28	5.79	102.4	105.4	91.7	106.7	102.9	106
8.32	4.34	104.1	99.3	107.4	108.6	97.6	95.3
6.71	4.18	98.7	91.8	100.4	99.2	136	112.1
11.08	5.5	107.8	91.5	113.8	112.6	101	105.1
9.48	8.9	111.4	106.9	107.8	123.8	122.5	113.9
5.33	6.33	76	70.1	54.1	138.7	136.4	138.3
9.54	6.35	91.2	95.1	108.7	100.1	98.2	103.8
8.29	11.87	95.9	98.8	96.3	107.6	114.4	111.6
9.03	2.42	103.5	108.8	123.1	87.2	96.4	80.2
9.98	6.56	99.2	112.5	111.4	101.5	111.6	100.5
9.57	7.57	95.7	95.3	99.5	101.2	105.6	106
5.69	7.47	110.4	91.2	101.3	106.4	100.8	98.6
9.38	4.62	104.9	99.3	105	84.8	104.6	102.9
8.97	6.44	88.9	90.6	84.9	84.2	71.4	88.9
8.88	2.76	103.9	106	98.4	110	107.6	106.6
5.15	7.77	120.5	118.2	110.6	85	78.3	87.6
9.26	7.86	96.6	107	107.6	96.1	98.8	101.3
5.12	6.61	103.1	101.7	97.3	108.2	91.1	97.1
7.85	4.17	82	79.2	76.6	97.1	98.4	97.6
6.73	8.09	96.4	99.1	108.4	102.5	95.5	94.7
8.37	8.21	100.7	100.1	94.2	107.3	103	103.9
5.07	6.76	103.6	104.3	109.2	115.8	122.6	120.1
6.13	9.94	85.4	89.5	86.8	65	58	56.2
6.6	5.21	72.6	48.7	69.9	140.9	117.9	101
9.55	6.29	101.4	101.3	113.2	100.5	77.3	101.6
6.57	5.41	90.7	91	82.8	120.3	124.6	120
6.11	6.31	90.2	107.6	111.6	89.6	91.4	99
9.82	4.76	106.2	91.3	106.2	105	102.1	106.8

6.54	4.2	118.6	78.2	112	108.8	100.4	122.4
6.38	4.86	92.5	91.9	99.9	91	95.5	95.6
8.59	5.71	103.8	97.1	96.4	110.6	97.8	109.5
5.11	7.54	103.2	100.7	94.3	97	99.2	102.8
7.4	3.18	71.9	68.1	66.1	80.8	83.4	101.3
8.76	7.37	109	110.4	103.4	103	98.5	106.9
6.95	6.8	114.8	89.9	102.6	97.7	97.3	93.4
5.59	4.54	94.2	81.4	108.8	95.5	106.1	133.5
8.06	7.39	107.7	106.7	90.1	98.9	98.1	101.8
8.02	7.36	102.4	105.7	95.8	114	96.6	108.5
6.58	6.53	105.6	99	87.8	100.4	110.8	102.1
6.95	6.35	96.8	101.9	98.4	103.1	104.5	108.5
9.73	7.08	91.4	105	100.9	113.7	102.7	120.9
4.7	8.28	94.9	84.2	88.7	109.4	116	115.7
6.48	6.41	102.4	110.2	98.5	105	107.5	104.4
9.83	4.76	102.2	63.5	114	105.5	94.2	131.7
5.92	6.37	110.6	105.4	103	84.8	86.6	97.5
7.4	4.22	107	105.9	98.9	97.8	102.9	106.9
8.35	6.73	109.2	106.4	110.7	104	95.9	94.2
5.85	5.77	106.1	97.9	104.8	109.9	105.7	111.9
10.1	6.83	102	101.9	91.2	114.2	102.8	112
5.6	3.72	110.5	117.2	99.9	80.5	76.8	85
6.23	6.74	107.6	101.7	100.9	104.2	97.7	95.3
9	7.79	96.5	102.5	103.8	107.6	120.5	117
4.92	6.09	95.7	89.7	90.5	106.1	104.7	108.4
4.75	6.46	87	82.3	81.4	117.8	118.6	136.1
9.04	5.24	108.7	93.4	96.3	94.6	100.6	112.8
8.27	5.19	99.6	92.2	101.9	91.3	89.8	96.4
5.59	5.96	95.7	97.5	101.4	99	101.1	97.1
5.43	6.8	119.1	116.8	114	97.5	99.7	101.5
10.07	3.99	102.5	106.8	96.4	114.7	92	98.7
6.55	5.88	98.4	102.2	105.3	102.4	106.3	98
5.87	7.96	101.1	106.4	109.8	107.2	112.8	108.3
4.65	4.76	90.7	90.3	97.3	128.1	119.6	113.4
6.19	6.65	97.6	100.2	102	107.8	104.6	110.9
5.54	7.67	93.7	90.1	93.3	119	115.7	116.4
9.36	8.73	98.8	93.6	97.2	91.8	89.5	93.2
5.35	6.68	97	95.8	98.3	97.9	92.2	89.5
6.52	7.88	96.3	98.2	109.7	106	104	104.8
6.34	6.51	99.5	98.6	101.7	103.2	101.9	107.4
8.28	5.6	104.1	96.6	102.6	139.6	147.6	147.2
6.74	4.98	101.6	93.6	92.3	101	104.3	97.8
8	5.56	93.9	111.8	75.5	90.3	116.3	115.1
7.3	7.65	98.9	95.4	100.8	92	85.9	104.5
8.1	5.37	114.8	113.9	95.3	108	121.3	102.4
8.73	5.84	76.4	110.1	77.8	112.7	114.9	107.1
7.42	6.81	103.2	91.8	90.6	89.5	110.9	98.5
6.87	6.62	119.6	91.6	87.6	87.7	133.6	100.7
6.4	8.21	90.3	105.8	99.6	94.7	88.9	98.2
8.59	8.9	95.9	104	106.8	94	94.4	92.8
9.17	6.81	103	103.3	107.6	109.9	111.9	106.9

5.99	5.02	114.3	109.2	96	92.3	86.5	101.6
6.16	5.47	94	100.2	108.3	105.9	104.2	116.8
8.6	5.48	100.7	105.9	103.7	100.5	97.5	99.6
7.58	5.63	101.2	85.5	109.4	90.7	104.2	102.9
6.99	5.39	111.2	86.5	102.4	97.3	111.1	108.2
8.84	5.33	105.7	109.3	98.4	99.7	110.6	97.8
7.55	4.5	98.6	101.4	103.1	103.5	112.7	119.3
6.06	5.91	83.1	94.3	120.6	115.6	68.5	115.2
7.02	5.83	109.5	99.6	91.7	100.5	86.3	94.3
4.82	5.6	91.6	104.3	83.6	101.7	95.1	104.7
5.85	12.1	102.7	100.7	97.6	96.6	100.5	98.5
6.05	4.14	115.8	121.9	113.5	106.7	96.2	96.3
9.67	5.12	96.5	88.1	112	98	103.3	100.5
7.18	7.35	97	95.8	103.5	105.6	102.5	108
7.18	5.31	103.3	84.8	91.2	101.6	94.5	121.6
8.37	3.9	94.2	96.8	93.5	115.7	106.4	111.7
9.51	8.48	106.3	106.6	98.4	91.1	100.4	93.8
6.9	5.53	103.7	95.3	102.1	88.8	101.1	106.1
4.93	4.85	103.4	105.2	96.1	95.8	103.2	96
6.83	7.49	100.4	93.3	95.9	103.2	95.2	88.4
6.06	4.87	100.3	106.5	108.6	96.8	94.3	99
4.91	6.57	105.6	107.3	89.2	109.6	110.3	99.5
6.14	6.71	102.8	89.6	99.6	110.8	106.1	108.5
5.8	5.81	100.5	106.7	115.6	97.8	100.5	103.2
7.02	15.72	116.2	95.7	100.2	95.8	107.5	95.9
6.68	4.81	107.2	115.1	65.9	91.5	87.8	70.7
5.19	7.03	103.2	110.4	94.6	110.1	102.7	101.4
10.48	5.59	105.6	99.9	92	92.5	93.3	100.8
7.03	5.88	77.4	115.4	107.5	123.6	103.1	94.2
11.55	5.97	118.1	109.9	114.4	108.4	116.4	115.3
9.96	4.99	105	108.6	105.9	111.6	92.4	106.3
6.54	3.27	96.2	92.6	115.4	104	87.7	114.4
4.75	5.48	112.2	94.9	88.9	87.8	91.4	110.5
9.11	5.73	96.1	104.4	88.8	107.1	88.2	99.2
9.58	5.17	116.4	96.2	107.1	100.8	105.4	97
5.8	3.96	107.4	99.4	100.6	95.9	104.9	110.2
7.43	6.11	90.6	101.7	95.3	102.8	109.2	105.8
5.96	5.32	96.9	88.6	102.6	96.9	98.3	105.3
7.88	11.64	100.1	95.6	104.4	96.2	95.2	100.2
6.71	4.32	90.2	96.2	100.4	122.2	120.6	122.4
9.64	5.97	94.3	97.9	94	106.3	101.1	95.6
5.69	6.31	120.6	70.7	99.4	91.5	98.1	91.9
7.46	6.13	115.8	99.6	97.6	96	98.2	112.1
5.73	3.94	118.3	99.7	115.2	75.9	92.7	120.1
5.48	8.41	96	97.5	100.5	127.5	126.2	121.1
6.28	9.07	98.2	98.3	96.6	103.8	99.1	106.9
7.03	6.55	95.3	93.8	87.3	150.4	96	116.9
4.84	6.07	89.7	109.6	104.5	94.8	102.1	98.6
8.27	5.85	128.7	85.1	101.9	87.3	101.7	106.6
8.76	5.42	104.5	103.2	110.4	106	104.4	99
6.42	2.57	98.3	94.9	90.5	106.1	99.8	114.7

8	4.26	97	101.1	105.9	121.6	132.3	111.6
4.59	4.92	104.7	95.4	91.2	104	98.3	106.3
9.01	6.28	91.5	94.3	99.5	98.4	98.2	110.9
6.65	5.03	109.1	116.2	101.8	109	96.6	105.2
7.14	5.58	103.3	105.7	96.5	89.3	86.2	85.9
5.64	6.03	87.3	83.6	94.3	94.5	121.9	108.3
4.37	9.44	102.5	91.3	100.9	95.7	98.7	92.7
6.11	3.87	112	102.5	91.7	98.9	102.7	101.1
6.67	3.18	97.4	102.3	107.5	101.2	98.2	113.2
10.37	7.38	87.5	97.5	90.8	103.4	103.5	118
7.06	5.62	96.6	96	93.5	112.1	109.2	116.6
8.28	5.7	107.5	88.3	106.8	110.3	98	95.4
5.48	3.51	86.5	93.4	105.1	99.3	65.5	78.2
7.77	4.99	82.1	108.8	98.9	106.4	83	91.7
7.69	5.11	97.9	93.8	98.1	103.5	92.1	104.9
9.1	6.12	85.3	75.2	144.9	90.3	186.5	98.7
6.67	4.87	96.8	100.9	100.9	113.2	99.6	94.7
5.86	4.81	89.7	121.3	119.6	103.5	111	102.3
9.19	5.59	101.7	98.9	97.3	101.9	103.4	106.5
7.11	5.77	104.8	106.5	104.9	104.3	111.1	104.2
8.18	4.96	108.7	109.7	109.9	120	114.7	89
6.99	5.23	101.1	108.9	97.7	102.6	90.6	97.6
8.21	2.59	89.9	101.9	76.8	110.1	116.4	104.9
5.73	2.2	117.7	98.9	109.4	81	82.7	85.5
6.48	6.82	109.9	93	101.6	107.8	95.4	97.6
6.25	3.97	93.5	96.6	99.9	114	100.5	99.3
6.32	6.78	100.8	105.1	100.6	102.9	101.2	107.9
6.6	6.73	97.4	100.3	107.5	99.6	93.6	98.7
6.44	5.94	109	88.9	113.1	100.2	88.3	101.6
6.29	5.66	98.3	97.9	103.2	93.7	97.7	110.4
7.02	5.04	98.7	93.4	99	82.9	91.2	96.8
8.73	4.73	102.3	104.1	89.7	94.8	95.3	101
4.93	5.43	88.7	92.2	90.8	117.1	107.8	100
8.54	2.59	107.1	87.9	78	104.4	103.6	110.5
4.89	4.81	90.9	106.3	97.4	89.4	84	108.5
6.2	5.92	105.4	94.2	99.6	87.8	84.4	99.3
7.88	4.11	105.7	94.9	94.1	99.1	97.8	99.2
4.7	5.58	99.7	88.9	86.5	102	106.1	94.3
5.73	4.48	76.2	91.9	100.6	148.9	108.9	114.8
7.77	4.72	126.6	113.2	127.7	97.6	94	62.3
6.61	3.59	95.7	84.8	91.2	105	111.4	99.1
5.03	6.11	105.8	91.8	100.3	118.2	113.9	117.2
7.37	4.15	94	99.5	106.8	109.2	125.5	117
4.97	5.86	95.1	86.5	116.1	119.7	104.3	84.3
6.23	4.61	100.7	88.9	109.8	89	88.7	119.7
7.77	9.28	97.9	102.4	105.4	90.1	86.1	94.8
6.54	5.74	100.4	92.3	110.6	105.6	104.5	104.6
6.4	5.08	104.1	89.3	89.2	111.1	112.8	111.6
7.2	2.86	95.7	99.5	105.4	84.3	97.9	96
10.7	5.53	100.8	101.7	98.8	118.5	113.3	117.6
4.89	6.51	105.4	110	100.3	100.4	97.2	105.4

5.27	8.33	87.6	95.3	91	107.9	105.2	109.1
5.4	2.16	135.3	100.5	93.7	92.5	114.9	111.1
6.51	6.76	93.1	94.3	97.5	93.9	107.3	104.4
9.63	5.64	97.5	99.1	99.1	107.5	106.7	107.2
12.15	12.34	91.6	94.3	97.1	107.6	106	106.3
7.05	4.87	86.7	78	87.2	120.6	128	127.8
9.57	6.16	108.6	93.3	93.6	100.8	94.7	99.1
8.65	5.72	90.5	88.9	100.2	86.4	94.7	97.7
6.73	3.53	109.3	114.6	104.1	104.4	117.7	101.8
8.47	6	97.5	104	96.1	97.9	96.6	104.4
9.67	6.37	107.4	100	106.1	88.8	98.1	87.9
6.46	3.57	157.5	71.2	83.5	79.9	81.8	96.2
6.43	4.04	102.5	101.4	99.4	99.2	91.4	101.8
6.18	4.1	103.8	89.2	99.6	93.3	99.6	104.9
10.08	7.55	108.7	102.5	94.5	108.9	105.9	103.7
8.25	5.23	103.7	106.3	103.5	113	92.1	96.1
4.39	4.89	102.8	102.4	96.8	107.8	94.9	96.1
5.39	6.03	136	89.4	87.8	91.3	87.1	93
8.34	4.48	104.2	97.7	101.1	116.9	116.9	93.3
8.22	5.38	104.6	92.2	98.2	74.5	79.1	84.3
8.18	4.23	97.1	89.3	110.1	110.7	104.6	116
7.25	5.14	95.5	111.1	88.6	98.4	96	100.6
7.78	5.05	113.9	105.8	106.4	114.9	112.9	104.7
6.19	5.35	110.4	113.4	94	80.4	95.6	100.7
8.72	5.68	105.8	100.9	98.1	101	90.8	92.9
6.95	3.52	109	114	97.6	102.6	103.1	85.8
8.98	5.65	106.7	95.3	107.7	110.5	99.7	106.1
5.29	5.23	98.7	97.9	109.3	102.7	104.2	103.8
5.71	13.59	86.9	74.4	102.7	100.9	95.5	98.7
9.5	5.58	104.8	77.5	92.9	118.4	122	127.9
9.95	6.11	103	95.5	99.5	111.2	97.9	112
7.14	5.31	90.9	104.2	108.9	88.6	109	93.4
6.62	3.92	98.2	101.2	100.8	94.8	87.7	104.2
6.55	5.39	109.7	95.3	111.8	102.3	81.3	89.2
4.75	5.73	96.5	101.9	100.4	99.4	124	106.4
8.75	4.81	103.8	99	112.6	86.1	90.7	89.5
6.19	8.69	94	96.9	98.9	103.4	98.4	103.6
7.52	5.07	110.6	99.4	102.8	101.6	107.1	99.8
4.68	4.7	110.7	103.9	117.2	65.8	78.4	70.2
4.73	4.59	94	94.5	95.7	94.4	98.1	100.8
7.71	5.08	97.8	96.2	84.1	99.1	101.7	95.8
5.72	6.07	89.7	94.3	85.3	62	65	63.3
8.29	4.2	100.1	98.1	96.6	108.8	102.3	95.6
5.6	4.97	120.3	97.3	93.5	104.6	90.9	109.1
8.94	3.02	107.5	94.6	100.9	94	108.5	105
9.22	2.76	98.2	96	74.5	104.3	99.6	103.6
8.5	2.07	125.3	105	115.7	89.9	105.9	97.6
5.07	6.23	102.8	97.1	90.9	101.5	105.1	114.6
9.36	6.17	119.8	98.9	105.2	91.6	88.3	89
4.79	2.94	108.7	105.2	101.3	89.1	79.8	77.1
6.02	5.77	93	103.8	99.4	100.3	102.4	108.9

7.21	3.77	126.2	87.8	80.2	121.4	102	98
7.25	2.22	101.7	104.5	100.1	98.2	95.1	90.2
6.93	2.38	94.1	88.8	103.1	100.7	98.3	103.7
7.03	4.69	95.3	85.1	81.6	44.7	53.2	49.9
9.73	5.71	101.7	102.7	96.4	101.7	99.8	104.8
6.55	4.53	100.3	98.6	98.9	107.6	108.5	108
5.21	7.09	100.5	101.1	109	88.5	85.5	86.4
6.83	5.04	106.9	92.6	100.1	98.2	100.9	108
8.72	4.98	101.9	100.3	104.9	94.1	95.3	97.5
5.82	5.48	84.3	107.4	103.2	101.6	101.5	104.8
5.48	3.31	89	83.7	93.3	102.9	114.1	118.7
6.54	3.04	106.4	97.5	102.9	102.6	97.2	96.8
5.16	4.47	98.3	110.1	97	109.3	115.8	103.8
7.21	3.01	86.2	82	95.6	102.1	106.2	107.9
6.21	4.65	81.6	100.5	100.3	93	94.1	95.3
9.76	5.12	102.5	109.8	94.6	114.4	112.6	113.3
7.01	5.79	101.4	83.1	111.3	59	98.7	93.8
6.83	3.05	91.2	100.5	104.2	119.9	115	119.2
7.23	3.64	83.6	92.3	106.8	101.7	110.9	107.3
6.89	4.47	111.9	110.3	104.2	91.8	99.3	90.8
5.43	6.54	97.7	90.9	104.1	98.4	97.1	101.8
6.61	5.99	96.8	96.9	93.8	96	92.5	96.2
9.13	2.3	113.8	113.5	111.7	111.3	105.1	103.5
6.8	5.47	90.6	97.3	95.4	112.8	110.4	105.4
8.41	4.79	101.8	104.8	108.2	116.3	105.7	106.9
9.86	3.25	117.4	112.8	89.1	99.8	101.4	100.8
4.75	6.04	96.9	95.2	102.2	128.8	123.8	121.2
6.95	5.62	78.5	74.1	62.5	97.3	82.4	89.5
8.62	5.81	107.9	101.5	103.1	99.1	89.1	96.3
8.92	4.93	95.1	98.4	114.9	98.4	98.3	104.6
8.41	5.78	99.6	102.9	98.8	100.1	99.5	109.8
7.39	2.27	103.3	100.2	103.7	123.1	116	101
5.86	4.27	114.6	84.5	126.1	106.3	80.3	87.4
6.76	3.3	100.5	86.5	92.6	100.6	81.2	102.8
6.43	5.88	109.7	115	109.6	77	83.8	95.8
9.07	5.6	110.8	106.3	98.3	139.2	127.1	123.1
7.36	4.87	100	100.8	105.5	98	82.7	98.8
6.62	4.98	93.4	105.5	95.2	102.3	96.1	94.2
10.45	7.93	94.2	94.3	98	100.7	99.6	104.5
9.99	6.75	110.3	98.1	112.1	96.8	108.3	108
6.6	8.06	99.7	104.8	101.8	102.4	100.2	104.5
5.92	3.04	105.8	95.3	100.6	111.3	97.9	97
8.69	2.6	166.8	85.1	108.1	103.4	87	81
7.11	2.66	95.1	100.3	86	110.4	100.8	102.6
8.66	2.02	81.3	108.9	96.6	126.6	96.1	119.5
6.39	2.67	102.8	105.3	93.3	108	118.3	117.6
6.34	6.35	96.7	95	99.5	111.5	112.5	114.4
8.25	6.35	106.5	99.7	108.7	97.7	104.3	102.8
5.78	5.14	75.1	106.1	96.9	98.9	91.2	103.9
4.72	3.3	111.4	110.9	100.4	111.8	84.2	106.7
9.13	4.94	101.7	104.4	113.8	78.6	77.7	74.8

6.25	4.76	94.5	104	97.7	91.4	94.2	92.4
7.84	27.16	108.2	109.2	103.5	87.4	87.7	86.7
7.85	5.16	92.6	100.3	95.4	97.7	106.1	106.4
6.51	5.27	103.4	112.6	118.4	85.8	92.5	95.8
7.49	4.26	95.4	108.7	82.2	109.6	116.9	104.3
7.01	4.42	81.6	104.4	100.4	97.8	87.6	94.7
6.71	4.28	126.2	115.1	101.6	79.1	112	95.6
6.86	2.86	99.6	111.5	101.8	87.2	100.3	98.7
7.77	5.54	105	108.4	106.9	102.2	93.3	92.7
4.94	4.71	93.6	123.9	112.3	114.1	104.2	111.4
5.35	5.21	100.3	95.8	93	89.4	87.9	100.9
9.66	3.4	97	100.8	96.8	89.2	97.2	103.9
8.6	5.54	107.4	112.2	116.3	91.2	98.8	95.9
4.63	9.45	98.8	103.7	104.2	107.1	116.4	109.9
8.46	3.84	89.7	85.5	78.7	101.9	109.3	98.7
4.7	5.47	108.1	102.7	100.2	100.1	106.7	122.4
6.65	3.48	99.4	103.7	100.4	101.5	101.4	107.1
9.26	5.04	98.5	102.9	105.8	106.4	112.5	116.7
9.25	4.65	101.6	95.2	95	85.8	89.6	93.6
8.27	5.38	100.7	87.9	105.7	107	105.8	99.1
8.27	2.7	103.6	104.4	110.7	93.5	90.5	85.4
8.35	6.13	106.4	109.9	103.5	105.9	104	107.6
6.09	4.43	102.7	101.2	107.2	94.1	83	84.6
7.14	4.14	108.2	106.4	110.6	106.9	98.9	100.5
7.27	8	94.1	103.4	77.9	103.4	93	140.6
6.8	5.33	97.3	102.9	110.5	76.4	104.4	85.1
6.2	2.18	99.2	102.3	108.6	86.6	100.4	100.4
8.02	2.78	107	98.1	96.7	116.2	101	109.2
5.17	5.86	84.7	100.4	90.8	116	106.2	120.8
7.06	5.19	93.8	110.9	96	78.3	86	91.8
5.99	1.71	103.9	91.7	110.6	111.2	94.4	93.3
6.74	4.59	96	104	94.4	107.1	82.1	105.6
5.34	4.98	100.9	104.4	66.8	121.4	128.2	108.7
6.81	4.32	104.7	82.6	106.4	64.1	111.9	88.8
8.13	3.93	88.9	112.1	109.2	115.4	99.5	105.8
7.96	2.01	94.5	87.7	85.3	118.6	111.8	114.1
10.48	5.67	99.9	113.7	109.9	159.4	150.1	159.8
7.03	5.55	101.2	99.6	101.4	101.1	106.2	99.5
4.94	5.47	89	83.8	93.6	109.2	103.1	113
5.57	3.15	110.6	107.3	103.5	102.8	92.5	91
8.16	9.15	95.1	93.8	96.1	143.2	144.1	95.7
7.28	4.38	105.3	98.9	112.1	102.5	91.6	115.1
4.84	4.6	86.7	128.2	71.5	102.7	89.7	99.5
5.69	2.31	105	113.9	114.5	111.7	116.1	91.6
6.4	5.96	96.4	98.9	96.6	106.5	98.8	103.4
5.16	3.42	84.4	96.4	96.5	94.3	96.3	111.3
7.4	2.75	95.5	117.5	102.2	75.3	102.1	101.8
8.56	3.89	109.9	94	87.5	84.1	107.6	114.5
9.26	5.21	101.3	115.2	92.1	95	96.1	112.6
9.09	5.18	102	92.8	103.8	110.7	96.9	96.9
8.98	2.6	107.5	89.5	89.7	102	97.5	111.3

5.41	4.76	101.4	93.4	105.1	99.6	100.3	95.1
7.2	2.72	96.8	90	104.5	102.6	105.9	128.1
5.06	5.15	93.7	100.6	99.6	102.1	104.1	112.4
5.05	5.42	93.1	97.5	100.1	110.4	111.4	117
8.63	5.36	104.1	109.5	110.2	97.7	107	101.3
9.69	2.78	112	112.6	81.7	108.3	83.7	100.8
5.17	2.18	105.9	97.5	115	73.8	90.4	81.1
6.35	5.04	94.6	89.5	101.9	99.7	114	122.8
5.1	4.96	112.5	91.8	110.4	91.4	86.4	122.6
6.4	4.68	105.5	86	93.6	79.4	91.4	89.8
9.44	2.05	106.4	95.9	96.5	142.4	123	126.4
5.71	4.76	105	107.5	105.7	109	119.2	114.7
7.85	2.94	104.2	97.9	103.2	88.1	101.5	98
8.24	5.87	90.5	100.7	106.6	111	110.9	116.4
5.02	6.36	105.7	113.5	106.6	116.9	104.2	114.3
6.52	2.82	92.7	126	117.9	97.7	122.9	97.1
5.2	3.4	101.6	94.6	88.1	76.2	89.2	95.8
5.81	4.68	120.4	98.8	105.4	95.5	86.1	85
8.62	4.97	103.4	113.9	100.4	99	119.8	110.3
5.26	4.45	100.8	94.3	91.4	94.4	98.9	82.6
6.67	4.09	99.6	94.9	98.7	100	87.9	99
5.12	2.58	92.6	97.2	102.3	86.5	100.3	93.4
8.15	2.69	90.9	87.4	93.7	97.4	103.4	113.8
4.72	4.27	96.7	100.6	111.3	96.2	89.4	107.9
4.51	3.45	111.4	86.2	91.9	98.8	119.3	107.3
7.96	3.86	111.5	90.4	118.3	86.4	92.8	90
10.3	4.34	113.6	108.4	105.1	114.2	118.6	131.8
6.32	5	94.8	92.1	102.2	105.7	105.2	107
7.06	5.1	93.7	101.3	86.3	93.2	95.8	102.2
8.87	5.57	95.7	105.7	107	106	105.1	103.4
7.55	1.99	97.9	82	91.3	92.8	97.4	88.6
6.29	5.53	90.7	104.4	91.6	102.6	109.4	101.2
8.75	4.78	102.1	109.7	107.6	101.6	104.3	99.7
5.53	6.13	86.3	98.3	89	93.8	110.4	112.3
5.82	2.24	84.2	77.3	79.8	105.7	116	103.9
8	5.62	100.9	104.7	94.9	103.4	95.2	105.4
7.62	4.99	91.1	88.2	88.1	116.5	108.9	106.5
9.13	2.06	78.9	104.3	105.2	112.7	102	101.2
4.41	3.42	115.6	110.8	115.8	107.4	103.1	98.4
6.84	3.55	123.8	105.7	125.1	90.9	89.8	110.7
8.88	3.08	103.7	92.5	103.7	109.8	107.8	103.4
5.03	2.4	94.2	101.6	101.3	107.5	110.1	114.9
6.61	4.9	95	92.5	104.2	93.3	86.9	110.9
10.24	5.5	102.1	98.3	101.3	108.5	106.6	122.9
9.44	3.38	65.1	91.9	114.7	101.9	130.3	169.6
9.14	2.32	96.7	78.7	85.8	102.9	101.4	92.9
8.4	2.19	108.9	107.1	110.5	98.3	77.9	103.1
6.9	5.04	109.5	114	90.2	95.4	86	98.5
9.01	5.27	90	114.2	81.2	105.1	89.2	120.8
7.5	5.53	106.3	111.3	104.9	109.4	113.5	105.2
4.44	4.27	79.3	108	99.8	111.7	108.2	110.4

7.74	2.43	100.8	99.5	94.7	99.2	99	97.3
8.46	4.79	97	82.2	119	102.2	114.4	104.3
5.64	2.67	77.9	104.8	113.8	97.6	101.1	84.5
8.4	6.59	103.8	111	109.1	89.8	87.5	98
8.87	5.72	120.7	111.7	121	80.3	86.4	97.8
8.65	2.67	92.7	87.2	92.7	99.1	107.4	110.1
6.87	5.08	109.7	103.6	104.4	102.7	97.1	97.4
7.3	5.27	103.7	94.1	99.6	101.4	102.6	97.9
9	4.8	97.4	99.7	97.7	86.2	115	117.8
8.56	4.65	94.6	89.4	102.9	114.1	112.1	114.8
6.1	5.05	99.5	93	92.8	108.2	100.7	113.7
9.73	3.86	98.5	120.2	88.8	136.6	178.3	169.6
5.68	3.89	99.6	86.6	109.8	91.4	103.2	105.6
6.54	4.89	101.1	106.4	114.7	98.4	111.6	109.2
9.32	7.48	97.6	105.6	96.6	121.3	111	123.6
8.68	4.19	97.1	86.4	104.5	94.2	101.1	119.1
6.62	5.3	97.3	113.9	103.5	106.6	106	112
8.79	4.23	103.8	102.1	103.2	110.4	109.4	108.6
5.1	5.08	114.8	94.2	96.9	109.2	93.7	118.3
8.1	4.55	120.2	109.5	97.1	90.5	97.8	90.4
5.6	4.82	102.1	101.5	101.8	101.3	91.2	102.3
7.97	5.04	109.9	102.2	102.7	108.5	95.5	105.4
8.78	6.17	73.1	79.5	78.5	97.4	89	91.9
5.85	4.13	89.9	91.2	101.1	110.6	117	112.5
5.36	5.12	102.7	107.6	97.7	96.8	89.7	102.6
8.12	5.06	105.9	107	98	89.6	94.4	100.8
6.4	4.72	85.2	88.1	96.7	111.4	117.8	110.1
9.32	4.28	95.8	95.8	94.6	103.9	94.9	102.1
8.12	4.24	110.6	110.9	114.6	94	90	97.6
5.47	3.2	112.7	119.7	105.2	105.3	105.7	100
5.4	2.75	91.2	93	90.7	89.1	111.2	107.2
9.32	8.53	95.6	63.2	104.8	96.8	102.3	133.4
9.38	2.7	96	97.7	102.8	102.4	94.6	94.2
8.53	4.7	108.3	106.1	108.2	107.2	105.7	108.3
7.81	7	93.8	95.6	100.2	102.4	98.4	110.8
6.25	4.5	116.1	102.8	107.8	99.1	105.1	114.1
5.91	6.23	96.2	89.7	98.4	91.6	96.9	98.7
8.65	4.58	106.2	101.1	93.9	110	113.3	104.8
6.25	5.5	95.6	86	98.1	83.7	80.1	85.2
6.99	4.03	144.2	126.8	120.8	59.5	57.7	47
8.79	2.58	92.7	87.7	97.5	103.5	113.8	151.9
6.44	5.52	104.3	99.9	103.5	104.6	107.9	105.9
6.65	2.26	114.1	101	94.7	118.3	123.2	101.9
8.06	3.69	115.6	98.9	105.6	96.9	92.8	90
9.03	2.08	105.4	133.2	89.2	99.7	103.6	77.7
7.94	4.38	98.6	90.8	118.5	89.3	114.8	93.8
9.36	3.36	115.8	83.9	102.7	119.6	120.3	97.8
7.27	1.69	99.3	108.5	99.9	112.3	102	108
8.29	4.9	98.2	116.4	107.3	111.8	99.8	108.3
5.62	5.26	95.6	103.6	97.5	111.2	125.5	103.2
8.4	4.06	89.9	85.9	101.6	107.1	113.5	110.4

8.91	3.6	118.9	107	109	98.9	100.7	111.7
10.2	4.63	104.8	111.5	106.4	113.4	108.4	94.1
7.36	3.4	110.2	101.4	129.4	104.4	102.6	120.5
6.39	7.56	115.7	50.4	88.8	178.4	51.8	196.4
6.27	2.44	104.1	89.3	88.6	102.5	103.4	110.5
6.84	3.76	85.6	73.6	83.8	117.8	100.4	119.9
6	3.81	108.8	99.3	93.8	90.4	102.6	97
6.05	4.31	97.8	102.5	99.2	108.1	108.9	103.8
7.97	1.83	93.2	77	102.7	109.9	82.9	105
6.77	5.14	119.5	120.5	111.1	87.7	114.4	115.1
9.77	3.49	91	88.3	83.5	121.2	112.7	123.8
8.32	2.5	83.7	119.2	74	91	98.7	97.7
5.06	10.11	83.8	88.2	88.4	169.6	171.8	171.8
9.03	3.74	114.7	104.4	99	110.5	109.7	115
7.05	4.19	124.4	125.8	94.4	118.1	99.8	95.7
6.76	1.99	79.8	91.6	98.6	92.6	100.7	113.3
5.07	11.81	98	124.1	91	116.6	72.8	95.8
5	4	107	96.9	107.2	97.1	94.7	97.1
8.07	4.65	93.8	101.7	109.1	97.6	85.4	88.6
7.06	4.65	99.8	102.4	91.3	105	109.8	103.5
8.03	2.95	65.8	92.9	102.6	81.5	108.8	105.9
7.77	2.78	100.2	87.4	86.5	110.9	102.3	108.5
6.8	1.96	104.1	92	101.3	91.4	99.7	111.7
6.73	3.91	121.7	95.2	107.6	98.6	92.9	107
8.6	4.98	101.1	99	105.1	112.3	109.8	117.9
4.5	2.07	92.7	100.1	95.1	97.6	99.2	94.5
9.29	3.93	94.4	95.2	97.2	105.7	106.2	94.7
8.25	2.35	94.6	87.5	78.9	103.1	105.8	93.8
9.91	2.99	99.1	107.2	117	109	107.4	88.1
8.31	3.58	107.1	105.5	100.8	100.8	96.6	116
5.39	5.38	93.3	96.3	95.8	107	109.4	109.7
5.64	3.91	94.4	107.2	103.3	96.3	99.5	98.2
8.19	3.57	95.3	98.1	84.7	77.6	71.8	78.9
8.85	2.17	110.8	124	97.1	96.3	101	94.9
6.54	2.26	116.4	124.9	105.8	70.3	88.6	94.1
6.57	2.49	91.3	93.1	104.9	96.6	94	93.1
8.97	3.95	94.1	101	96.2	99.6	105.4	110.6
7.69	5.53	129.9	88.2	103.5	82.9	130.9	85.8
9.36	2.19	97.3	86.7	93	88.5	77.5	96.1
8.51	6.1	102.6	98.7	100.2	106.8	111.9	110.3
4.92	4.74	116.1	102.4	89.6	102.2	123.2	71.2
8.41	3.42	91.8	93.5	99.4	121.1	115.2	107.8
6.83	1.82	104.2	105.9	97	106.7	116.8	107.2
7.71	3.98	101.9	103.6	97.6	104.7	103.5	101.1
8.1	3.15	89.8	83.7	83.8	116.5	102.5	119.2
7.84	2.49	120.4	111	116.6	102.2	91.3	89.2
6.18	2.88	90.8	94.3	101	119.2	111.4	98.3
6.93	4.79	104.4	108.3	100.5	105.3	94.4	103.1
6.58	7.86	88.2	98.2	116.1	99.3	113.8	125
5.82	4.88	101.8	79.9	101.7	108.9	132.7	167.2
4.83	4.04	95.2	92.7	89.5	120.4	114.3	111.4

8.81	2.05	100	118.5	102.6	83.6	96.1	99.9
5.96	7.69	95.7	92.9	97	97.2	108	100.3
8.12	2.46	94.9	106.2	117.7	92.2	91.5	94.4
8.6	4.48	94.4	97.2	98.4	107.8	97.4	110.6
9.55	2.74	104.1	95.1	100.7	105.7	113.5	116.2
11.33	3.37	109.7	115.6	88.9	117.9	112.4	86
8.03	4.1	97.1	92.4	132	82.6	95.1	81.6
5.68	3.9	98	89.9	127.8	107.7	108	96.6
5.97	3.23	98.5	95.1	115.3	82.3	89.5	98
6.96	3.01	121.4	97.6	137	112.4	97	113.9
10.77	7.42	112.1	107.4	93.5	101.9	91.2	102.2
6.06	1.78	87.8	85.3	100	97.6	97.2	109.3
8.95	3.41	138.1	99.2	119.7	97.3	105.4	98.6
7.88	4.7	103.1	104.5	101.3	97.2	92.7	106.8
5.66	3.41	101.1	99.1	80.5	89.1	87.2	128.8
8.19	6.11	97	99.4	96.7	93.1	97.7	100.6
6.49	2.91	112.1	88.6	97.9	89.9	95.8	95.8
8.31	3.84	95.2	110.9	102.3	96.6	93.9	97.9
4.91	3.12	101.9	101.2	97.5	80.6	92.4	103.3
6.2	2.48	106.3	136.6	74.4	96.4	101	121.8
6.7	3.64	100.4	104.1	114	105.8	105.5	108.3
4.75	6.2	104.5	105.5	104.3	113.1	104.3	105.7
9.94	4.17	95.5	93.5	96.7	125.4	122.8	125.7
10.04	4.09	137.2	129.8	167.8	84.7	57.1	78.8
6.68	4.53	107.1	97.2	103	104	94.3	102.6
6.01	6.92	103.9	108.7	113.4	110.5	106.8	118.1
6.98	3.34	92.1	98.3	95.8	99.8	105.6	95.9
7.31	3.93	107.8	86.1	103.7	89.9	111	109
6.48	3.38	93.1	101.6	91.2	108.1	103.6	93.5
5.59	2.53	81.5	89	88.7	93.7	98.1	103
7.24	3.89	113.6	103.5	100.4	107.9	106.8	103.5
9.31	2.53	93.7	85.9	98.9	107.4	114.1	94.8
9.38	4.92	121.4	122.8	125.3	36.4	28.8	39.7
7.4	2.87	121.4	98.9	105	95.7	88	99.1
5.31	4.15	115.4	117.8	86.8	82.3	115.9	107.2
5.48	2.93	110.2	90	105.8	105.5	97.4	108.7
7.2	3.22	91.6	100.4	103.1	107.3	97.7	84.6
7.14	6.01	133.9	113.1	150	72.5	67.8	68.2
9.07	3.44	102.2	79	99.9	100.2	101.5	108.6
9.2	3.8	100.4	95	96.8	105.4	92.6	107.4
6.52	3.26	112	106.6	95.8	95.8	86.6	85.4
8.76	3.78	96.2	93.6	105	92.3	106.1	119.2
5.6	4.62	98.9	96.5	106	114.6	117.1	133.3
9.57	3.46	107.5	83.4	89	119	94.4	104.1
7.52	3.16	101.7	97.2	99.4	106.2	100.1	96.1
9.58	3.46	104.3	102.2	106.5	104.3	107.3	108.2
5.81	2.55	96.7	101.2	112.4	117.9	93.2	105.9
9.7	4.95	105.5	103.7	104.4	105.8	102.7	101.2
9.55	3.57	111.6	96.7	100.7	104	113.8	105.8
4.82	2.8	101.1	120.6	120.7	100.6	89.1	77.5
5.63	2.3	102.2	105	94.2	84.8	93.3	104.2

10.02	3.87	107.2	104.9	90.6	104.4	109.6	109.8
7.14	3.6	126.3	108.2	109.9	71.5	126.9	78.9
7.78	3.02	105.4	105.8	101.8	103.9	100.4	104.3
8.94	4.12	106.8	110	102.8	127	110.1	124.4
6.65	3.06	102.9	102.6	97.8	118.1	105.9	116.1
6.77	3.8	106.2	111.7	103.5	93	93	92.9
6.01	3.47	100.1	76.1	90.5	109.1	89.9	104.3
8	2.97	125.5	95.5	101.8	86.3	99	89.8
5.66	4.27	102.4	99.2	97.3	93.4	93.2	103.3
6.01	3.36	92.5	100.7	98.3	108.7	102	103.7
5.6	3.95	108	93.4	95.1	103.6	98.2	107.3
5.27	2.67	101.4	100.4	99.5	104.4	91.1	89.3
4.36	3.87	89.5	87.8	92.5	127.1	132.3	113.6
6.65	3.78	131.7	121.2	88.8	90.6	65.8	95.7
8.02	3.5	80.2	108.2	97.8	112.3	122.2	108.1
6.46	2.84	105.7	102.1	101.3	96.1	89.4	93.4
8.06	2.01	124.8	86.3	96.2	96.6	120.9	93.2
7.87	3.28	108.3	114.4	82.2	127.9	115.2	94.4
8.12	2.73	83.7	92.7	70.4	127.3	156.2	139.8
6.77	2.97	108.1	96	93.2	81.6	91.5	95.7
10.37	3.77	103	105.7	97.1	123	136.2	126.5
7.27	3.1	111.9	110.1	101.8	89.2	88.4	97.7
7.42	8.24	101	102.8	103.7	108.3	104.3	104.7
6.81	5.49	99.9	102.6	104.6	118.3	113.5	110.8
8.65	4.82	96.8	95.2	102.5	99.2	101.2	102.9
8.06	3.63	108.3	106.4	102.8	99.5	98.5	98.9
9.72	2.1	104	87.9	101.3	97.9	88.9	108.2
5.11	3.15	104.1	113.7	90.5	115.5	113.4	117.5
9.35	3.71	115.3	94.6	76.7	77.7	98.4	81.8
6.96	2.72	101.5	90.1	123.6	102.1	107.8	93.3
7.09	5.03	101.9	102.7	109.4	95.2	94.1	99
8.09	3.36	108.8	117.5	106.3	89.3	104.2	106.1
7.84	3.41	94.9	102.8	94	94.5	97.4	98.7
9.32	2.87	91.8	90.5	82.8	119.1	101	93
8.22	3.91	103.7	94.6	87.7	107.2	114.4	102.6
8.53	3.41	113.5	103.4	105.9	99.1	107.4	102.3
6.54	3.55	97.4	87.2	111.7	93.2	96.4	114.1
6.37	4.45	118.3	146.7	103.9	95.9	54.4	85.9
5.43	3.61	122.7	105.6	97.2	78.8	76.5	103
5.36	4.66	94.9	106.8	104.4	85.5	82.1	96.5
6.99	3.27	98.5	92.1	105.3	99.2	81.1	93.9
6.29	2.28	118	98.4	104.6	99	87.3	99.2
4.55	4.24	94.8	85.9	79	104.6	103.4	99.1
8.95	2.95	93.6	102.1	87.2	110.2	117.4	108
8.62	2.05	111.3	97.9	111.7	105.9	104.1	107.8
5.12	3.88	103.2	104.5	104.7	117	113.4	98.9
8.78	4.14	91.2	86.8	88.9	116.2	104.5	124.8
9.55	4.66	108.3	102.4	100.6	102.6	101.9	103.5
5.94	1.74	101.8	104.4	115	114.1	99	114.7
8.95	3.02	98.1	107.3	98.5	116.5	106.3	101.1
6.93	2.99	89.1	96.8	119.9	95.1	114.3	111.3

5.94	3.65	90	118.6	88.3	148.1	114.6	98.3
7.28	2.04	138.7	103.2	62.5	101.7	145.5	125.4
6.74	2.99	111.3	105.1	80.8	99.2	97.4	111.8
10.08	4.25	93.1	108.2	114.8	133.1	114.6	101.7
6.93	3.17	94.1	112.1	98.1	107.7	100.2	103.6
7.28	3.87	92.3	100.9	108.9	100.8	109.4	101.8
4.89	4.76	96.7	106.2	107.3	109.3	103.8	111.8
10.14	3.46	84	88.4	81.1	121	127.3	124.9
5.16	2.46	119.1	83.6	83.4	102.9	90.4	106.9
7.03	3.01	122.3	98.5	89.9	119.3	121.9	130
6.52	2.6	85.1	99.5	88	86.6	79	98.7
6.18	2.38	117.6	87.2	79.3	79.8	107.3	95.5
5.34	3.94	102.8	109.8	119.3	103	107.4	108
7.09	4.78	83.1	67.8	82.7	112.8	136.2	120.1
8.27	3	93	110.2	100.3	105.6	104.7	93.9
5.96	3.23	99.8	95.9	94.3	102.8	94.6	96.6
9.16	3.23	110.7	96.7	93.5	92.1	112.7	96
6.54	3.64	101.7	100.6	102.7	73.2	74.8	82.6
9.57	3.46	97.4	136.4	120.5	131.2	127.7	131.7
6.68	5.65	117.5	102.4	105.9	95	90.7	85.1
7.47	3.15	107	113.4	86.5	102.5	116	104.9
9.61	2.61	101	110.9	96.8	61.9	53.4	40.8
8.95	3.06	108.8	102.2	107	145.1	143.2	130.7
7.44	2.91	96.1	95.2	98.8	103.2	100.9	116.7
9.92	3.63	131.4	91.3	94.7	106.1	114.4	94.5
4.81	3.24	98.6	92.5	95	111.3	108.2	100.8
8.16	3.98	103.6	95.4	119.9	106	115.9	113.7
8.27	3.29	97.8	110.2	96.9	100.3	98.5	106.5
7.4	3	112	111.3	99.2	92.9	102.8	99.2
5.07	2.31	113.8	117.3	93.5	135.7	105	84.5
6.13	3.4	93.2	91.9	101.1	99	91.7	104.8
4.21	3.37	99.3	100.7	96	94.4	94.1	99.5
5.58	2.85	95.6	106.6	115.1	84.5	122.6	106.5
9.45	3.03	98.3	80.5	126.2	119.6	112.6	110.1
7.66	2.97	89.8	105.9	106.4	98.3	113.4	73.1
6.67	2.48	91.8	112.4	98.8	72.9	60	68.5
5.07	4.38	98.2	84.6	87.3	113.5	112.1	113
5.05	2.95	84.8	69.9	112.3	86.2	121.5	109.2
4.09	3.9	101.5	72.1	88.1	123.8	106	110.1
8.07	2.67	116.2	117.7	126	134.1	144.1	130.7
5.47	2.73	110.9	89.6	100.3	110.1	102.6	115.2
6.9	3.27	107.6	113.1	110.2	100.8	92.7	79.2
9.01	2.16	110.7	103.6	102.7	102.3	97.5	110.1
5.74	2.81	91.9	100.9	93.4	89	113.8	120
6.89	3.11	118.4	94.6	85.7	95.3	79.9	93.4
8.1	4.74	100.7	101.8	100.9	108.6	104.6	103
8.41	5.86	122.2	91.1	95.9	116.3	91.1	113.6
9.94	3.09	101.3	93.6	101.6	105.5	114.6	108.3
4.97	2.39	84.9	109	80.8	94.2	92.6	108.1
8.24	3.64	105.7	99.4	99.3	88.9	102.2	103.9
7.37	2.96	107	98.1	108.4	106.8	99.2	100.1

8.46	2.56	113.3	139.9	134.2	81.1	84.6	82.7
9.19	3.72	95	102.6	99.5	104.5	102.7	97.6
5.44	2.62	79.8	86.5	70.7	118.8	114.7	128.1
8.76	3.64	103.9	81.8	91.9	133.2	129.1	136.1
8.12	1.9	97.8	111.4	83.5	138.3	125.5	134.3
8.53	3.59	104.1	96.3	101.4	101.4	88.7	84.8
8.12	3.21	87.9	88.3	90.1	101.8	105.3	128.5
5.55	3.9	97.3	104.6	103.7	104.4	83.9	105.3
6.4	2.2	92.7	114.9	92.4	124.4	114.5	110.2
6.54	3.27	128.9	100.7	119.4	90.8	87.6	119
6.95	3.32	89.5	103.2	99.9	98	88.2	107.8
5.97	2.15	112.1	94.8	94.4	104.9	94.7	112.7
8.91	4.85	111.1	107.6	101.3	102.8	99.1	103.1
5.85	3.81	86.8	79.3	82.1	96.1	99.6	97.8
7.23	4.06	95.2	95.5	113.2	102.3	108.3	102.3
6.67	3.31	67.8	81	91.7	92.3	104	129.1
9.7	3.47	111.6	109.5	98.7	107.8	99.4	96.5
7.34	2.43	116.1	101.9	97.4	98.7	120.4	114.8
8.98	2.86	80.6	86.4	105.5	90.5	109.6	101.9
7.77	3.71	93.8	98.5	102.4	109.8	102.7	105.6
8.32	3.08	92.2	94.9	91.6	101.5	112.3	108.8
8.29	2.3	102	99.3	94.7	100.8	107.7	115.3
5.02	4.83	86.6	76.9	93.6	118.8	118.4	117.4
6.52	2.69	108.2	100.5	81.5	108.6	102.6	112.8
6.79	3.44	101.3	106.2	102.1	99.3	94.6	114.8
6.42	3.36	104.9	102.5	74.1	107.4	93.1	91.1
9.11	2.33	100.2	86.9	110.8	116.3	96.6	92.6
6.39	1.9	88	106.5	118.9	101.4	124	105
8.1	3.34	94	99.5	107.7	104.8	114.8	119.1
5.47	2.21	95.8	108.3	108.6	79.2	81	92.9
7.21	3.45	109.7	94.7	103.2	82.9	98.8	108.5
10.86	1.74	91.5	84.8	110.8	80.3	81.1	119
10.39	3.74	105.1	99.4	103.7	103	112.4	109
9.11	8.27	94.2	95.1	99.5	110.4	99.2	97.8
8.21	2.79	96.3	98.3	101	98.5	121	113
5.1	3.32	94.2	99.7	107.5	95.9	99.8	94.5
5.76	1.61	97.1	100.6	93.9	102.2	96	105.9
4.42	3.23	115.1	117.9	98.3	112.9	96.6	86.1
4.79	2.99	83.8	87	100.5	104.2	103.5	111.8
5.21	2.69	101.7	115.2	79.3	104.4	119.7	105
6.92	2.58	92.8	59.3	81.5	98.4	120.7	128.7
5.49	2.95	76.1	87.6	81.5	120.2	104.5	121.6
9.01	3.09	105.8	85.8	116.7	111.1	115.5	123
4.87	2.84	106.9	83.4	103.1	118.4	112.5	104.1
6.74	5.55	139.5	100.8	91.7	99	98.7	97.7
8.82	3.6	80.2	107.8	98.2	107.9	88.5	103.5
8.06	2.56	100.7	92.1	91.4	89.2	102.4	90.7
6.84	4.37	109.1	92.2	105.3	95.7	80.6	84.7
6.8	1.84	104.8	91.6	103.5	92.5	101.8	129.3
7.18	2.49	112	79.5	108	120.5	127.4	115
8.15	2.61	97.6	113.9	100	103.9	102.5	80.8

8.72	3.67	105.1	104.1	104	107	99.6	105.4
9.36	2.18	92.4	109	95.1	90.2	105.9	92.4
6.73	2.46	123.4	114.9	118.9	78.5	72.4	87.3
5.49	3.01	123.9	57.5	93.1	106	99.8	120.1
7.09	3.07	110.8	96.6	98.6	104.2	94.2	103.2
8	3.96	97.7	82	98.9	120	120.4	111.3
7.83	2.7	117.4	136.8	101.3	120	85.1	119.7
8.21	2.63	94.9	117.1	95.8	89.2	109.4	95.1
10.86	4.5	103.1	78.7	68.7	83.7	132.5	157
7.9	2.06	125.7	105.5	71.4	106.4	93	109.7
7.24	5.25	115.1	85.4	94.5	106.2	96	126.6
9.41	2.79	95.8	110.5	114.1	91.6	95.3	80.5
5.14	1.61	112.3	122.9	101.8	106.2	98.8	116.4
8.05	2.04	107.6	109.6	100.8	117.3	112	90.8
9.28	2.21	112.4	89.1	95.8	115.2	106	123.4
5.6	2.81	84.1	97.8	116.4	107.7	106	101.4
5.41	3.22	89.2	102.6	91.2	100.2	94.7	109.5
10.17	3.8	99.6	96	109.6	104.3	105.9	100.4
10.14	3.33	108.6	110.8	111.7	136.4	118.7	135.1
10.05	2.98	105.8	87.2	103.8	94.1	102	106.8
8.72	2.77	111.9	105.6	89.5	108.5	157.3	108.1
6.29	2.54	80.2	81.3	96.4	95.5	103.1	107.8
8.1	3.23	108	101.4	112.5	100.8	105.1	104.9
6.71	4.88	96.5	101.8	106.2	92.2	107.9	107
8.59	2.35	91.2	89.7	108.2	106.7	120.8	118.5
8.73	2.94	92.3	92	86.6	103.5	115.3	127.8
8.43	2.19	91.4	83.5	85.2	106.4	113.1	114.5
5.9	2.44	82.8	99.9	100.4	114.2	103.6	97.8
9.57	2.56	95	104.4	121.3	84.5	121.6	102.5
4.5	4.02	103.1	98.3	84.2	112.8	108.3	103.4
9.6	2.81	116.7	86.9	101.9	112.3	98.4	91.7
5.31	1.99	122.5	94	98.8	103.3	122	103.5
9.16	2.87	95.4	95	113.2	105	99.1	108.1
6.3	1.91	102.1	93.2	103.2	94.8	93.7	109.3
6.77	2.13	99.2	87.3	103.9	86	100.2	101.5
5.95	3.01	96.3	118.1	118.2	101.4	105	116
9.83	2.24	115.8	95.6	85.4	116.8	104.3	120.9
6.27	2.93	69.7	101.9	123.2	104	88.8	117.5
7.31	2.67	108.2	105.2	127.2	112	98.9	100.8
7.93	2.45	101.6	95.5	106.1	99.7	104.6	91.8
7.55	1.93	93	85.5	90.1	100.8	121.8	99.8
5.86	4.05	96.5	104.1	106.8	100.4	104.3	103.3
8.94	2.52	98.3	103.3	98.3	123.5	103.8	136.5
11	2.2	63.5	100.9	97.6	110.8	126.9	87.8
9.85	6.72	115.8	105.9	102	99.5	106	95.1
7.97	3.6	98.4	101.5	95.5	112.6	106.5	107.6
6.77	3.15	89.4	92.1	95.7	101.2	100.6	103.4
5.5	4.39	247.4	83.7	86.3	91.1	83.3	85.2
10.07	2.58	97.3	106.9	102	99.9	99.4	115.3
5.43	2.87	98.5	111.2	120.5	102	90.9	111.9
6.57	2.59	88.6	70.8	92.3	105.1	102.7	67.3

5.34	3.31	89	96.9	91.6	106	89	103.5
6.96	2.93	79.8	96.2	84.8	92.6	98.3	92.9
5.07	2.38	93.7	90.7	84	106.8	91.2	122.1
5.82	3.59	105.2	99	113.5	104.1	96.9	108.1
9.22	1.65	138.6	102.2	138.7	67.6	76.8	81.2
7.87	2.53	105.9	97.3	93.3	91	92.9	85.8
9.5	2.65	108.7	105.8	94.6	100.5	94.2	95.3
8.13	3.11	93.7	97	100.5	118.7	102.5	100.4
9.98	2.87	104.9	127.4	88.6	105.4	98.6	120.8
5.55	1.99	120.4	94.3	89.9	102.3	81.2	117.1
8.95	3.29	89.5	90.3	95.4	113.2	130.7	115.5
11.44	4.02	104.8	99.9	99.9	107.1	103.1	105.7
8.7	3.19	94.4	92.3	101.6	108.9	107.4	114.4
7.93	7.31	103.3	96.6	99.6	76.5	81.4	79.8
9.19	2.46	87.5	94.4	103.8	106.3	92.8	101.3
9.42	2.58	87.3	94.4	93.2	103.9	101.6	102.2
8.37	2.43	101.7	108.2	103.4	108	105.2	112.3
8.32	6.89	99.4	105.3	102.5	99	96.3	95.9
12.25	2.74	98.6	94.2	99.2	125.6	133.4	117.4
6.7	4.05	83.9	100.4	106.4	97.3	107.7	107.4
6.01	2.4	97.4	97.7	90.9	116.3	107.2	107.7
6.99	2.49	93.6	97.7	74	106.5	95.8	97.5
4.17	3.42	104.4	100.8	97.9	108.2	87.1	93.4
7.87	2.29	120.3	91.1	90.9	120.2	100.7	117.6
8.9	2.22	109.4	103.1	104.8	116.2	106.7	108.7
4.74	2.62	78.7	114	132.3	119.9	123.5	109.3
9.16	2.46	98.6	105.5	108.5	77.9	105.6	105.3
5.68	2.47	104.4	95.8	101.5	104.9	100.2	102.7
8.06	2.23	105.6	95.6	110.7	84.7	75.6	96.1
7.42	2.82	133.1	144.6	126.2	105.8	104.5	79.1
8.4	2.82	107.3	102	109.7	103.6	95.8	92.8
5.06	2.37	98.6	92.5	102.7	91.7	102.6	98.8
4.82	1.85	117.7	87.5	90.3	100.5	95.5	120.5
8.05	2.74	114.3	150.2	104.3	100.1	81	77.8
5.29	2.21	118.1	74.5	94.3	102.8	85.9	116.2
6.42	3.19	98.2	103.1	106.1	102	100.6	111
9.99	3.01	103.6	109.7	110	106.5	114	109
5.82	3.13	102.5	120.5	110.1	97	99.5	89.6
9.72	2.28	82.2	108.9	96.6	92.6	109.9	74.3
7.8	2.67	120.2	102.9	100	105.4	106.5	94.4
10.93	2.45	100.6	89	103.8	91.8	86.7	106.3
7.78	2.97	94.5	109.2	108.8	91.3	86.9	86.4
5.74	2.47	108.6	89.5	94.6	102.3	94.4	86.7
4.92	2.09	100.4	99.5	96.2	95.4	97.8	95.7
9.25	2.9	85	87.8	126.6	95.4	128.8	115.7
9.01	2.82	100.6	91.8	93.6	107.1	104.3	97.8
8.69	3.19	100.2	88.8	92.1	100.1	106.6	108
4.02	1.8	101.4	78.7	57.5	133.7	100.1	108.3
7.72	2.89	116.3	94.8	96.6	91.1	79.8	98.6
8.92	2.91	99	99.4	109	95	94.6	100.1
7.66	3.21	116.6	110.5	120.1	103.1	79.5	93.4

6.34	2.39	116.1	87.2	92.9	108.1	133.4	102.4
7.59	2.5	90.7	54.2	139.9	87.5	79.2	91.7
7.77	2.8	107.5	86.1	106.9	108.2	95.9	102.5
7.03	2.61	103.4	94	93.2	99.5	97.8	99.3
5.35	2.12	112.6	102.8	89.3	109.8	110.1	104.3
7.9	4.1	97.1	87.3	97.6	94.1	89.4	92.6
9.99	7.27	112.8	104.9	106	155.6	141.3	140.7
8.9	2.87	93.3	87	93.7	112.6	107.7	117.6
7.02	2.24	93.9	91	84.9	101.1	99.5	96.2
7.77	2.2	99.3	90.4	89.6	134.8	93.3	87.1
4.7	2.84	101.5	86.7	89.3	129.4	93.5	104.7
7.39	2.32	112.3	119	103.4	114.5	105.6	95.6
4.89	2.08	147.1	123.7	137.8	83.7	71.2	82.9
4.89	2.87	100.5	94.6	88.1	99.5	101.2	111.4
6.04	2.37	107.3	125.3	80.7	108	89.6	97.9
6.79	2.6	101.9	97.7	90.4	92.7	84.4	98.5
6.83	3.51	100.2	102.1	98.1	115.2	99.7	93.6
6.13	1.76	116.2	88.6	95	97.3	105.7	96
6.48	2.7	95.9	97.7	99	106.3	101.1	124.2
9.09	3.04	128.7	98.6	93.6	83.3	112.1	105.3
6.68	3.17	92.6	84.5	90.7	104.9	103.2	99.2
5.72	3.56	82.5	107.4	97.2	104.6	106.3	116.9
5.3	2.92	96.9	108.2	109.2	102.8	117.8	97
6.47	2.56	110.4	90.6	93	110.1	97.1	121.9
8.82	2.79	107.7	97.1	102.3	97.7	115.6	108.7
6.42	1.97	102.4	95.5	94.8	110.1	86.1	90.4
7.78	1.91	93.6	97.6	105.1	93.9	122.5	124.7
5.12	1.61	98.1	106.8	114.4	86.7	87.4	104.9
7.39	2.53	92.8	100.5	93.3	102.4	95.8	105.3
9.52	3.2	100.6	95.4	90.2	113.9	114.7	110.7
5.68	2.08	90.4	50.5	104.5	113.6	106.2	121.7
9.96	2.51	118.4	77.8	129.2	89.8	96.9	106.4
5.05	2.9	99.5	96.1	108.2	97.8	100.5	104.8
8.88	2.06	93.1	98.1	103.5	92.6	128.3	120.4
8.18	3.08	97.8	97	86.1	103.4	90.2	94.3
9.88	2.88	100.2	91.8	103.9	109.3	111.4	111.9
9.13	2.7	108.2	110.5	101.6	95.3	94.9	105.4
10.29	2.88	99.8	100.5	98.1	118.4	121.1	114.2
5.58	2.52	82	87.9	101.1	107.4	110.6	113.6
6.74	4.56	102.1	109.3	97	101.3	101.6	102.1
9.54	2.87	93.9	97.7	92.8	120	111.5	114.7
8.1	2.28	102.1	111.2	94.3	121.3	73.8	95.8
8.44	3.25	104.9	88.9	95.6	104.5	107.3	111.7
7.64	2.37	111.9	113.3	110.7	126.8	109.4	123.8
5.3	2.79	98.5	92.1	96.2	101.2	102.6	100.4
5.36	2.9	95.6	93.8	108.4	103.7	98.9	111.9
9.41	2.56	101.6	82.3	80	101.5	107.5	120.6
8.15	2.03	117	92.7	73.3	86.9	99.3	115.3
6.48	4.35	96.8	92	109.4	97.2	98.5	100.5
6.37	2.2	72.2	83.2	76.1	122.2	114.5	119
10.54	2.21	92.2	96	123.1	106.6	99.7	91.6

6.19	3.11	153.1	81.8	77.5	94.4	128.4	96.2
5.06	3.63	98.6	106.5	104.6	90.5	110.5	87.4
7.9	3.21	111.1	122.4	123.9	83.7	82.4	86
7.23	2.96	110.4	99.8	109.1	100.3	99.6	104.8
7.06	2.03	136.4	124.6	126	75.7	50.6	82.5
7.94	2.5	82.8	96	104.4	116.3	106.6	98.5
6.7	3.5	87.1	98.3	97.1	102.8	91.8	110.2
10.1	2.59	117	93.2	103.4	108.4	111	103.2
6.48	1.9	113.5	102.2	97.2	89.3	115.6	85.9
4.87	2.54	102	106.7	106.5	108.3	116	116.7
8.47	1.72	104.1	84.2	94.5	95.5	101.5	103.1
4.17	2.59	82.9	112.5	106.3	123.1	102.4	120.1
6.58	2.09	106	86.6	106.1	89.7	106.9	116
8.51	2.34	122.7	93.7	95	89.9	94.8	92.7
7.65	2.35	96.5	73	88.9	101.6	111.9	122.8
8.05	2.13	84.5	76.6	83.7	96.2	116.2	99.5
5.62	2.91	72.7	117.9	94.7	97.7	99.3	97.5
8.1	3.35	103.4	98.5	107.9	111	112.3	111.6
9.85	1.81	95.3	113.8	90.8	94.8	93.3	97
8.22	2.82	100.5	100.2	102.1	107.1	100.2	103.3
6.76	3.86	98.5	105.2	111.9	110.6	110.3	103.6
5.52	2.27	83.8	102	106.9	100.4	116.4	120.9
7.42	2.24	89.7	80.5	80.2	102.9	110.5	107
10.13	9.57	112.9	120.2	112.7	88.6	89.9	87.5
8.5	2.23	100	100.2	98.7	99.9	100.2	100.1
5.95	2.94	104.9	93.5	96.8	97.8	94.2	89.6
7.72	2.1	88.5	86.4	100.6	105.5	103.1	102.3
6.19	2.05	100.6	96	112.5	109.6	69	92.3
6.04	2.24	112.7	98.8	98.6	103.8	100.8	111.9
9.33	3.47	96.3	99.9	97.8	112.1	106.1	108.8
6.47	2.01	89.9	87.8	126.4	87.8	124.6	119
5.17	2.77	116.4	99.8	119.4	93.6	93.8	82.1
6.79	2.55	107	123.7	107.4	91.9	97.1	90.8
7.28	3.04	100.6	96.2	102.1	96.4	93.2	92
8.15	2.49	123.7	102.2	87.8	96.5	84.9	89.8
6.62	3.12	112	110.6	94.6	91.3	103.4	101.8
4.88	2.49	111.9	97.8	104.5	111.6	81.9	90.5
7.62	2.84	108.8	95.7	112.6	88.2	95.4	94.4
8.27	2.91	107.6	89.8	98.5	93.1	95.6	106.3
4.18	2.5	161.7	88.8	98.8	100.5	102	87.3
7.3	2.15	67.2	105.5	109.6	92.2	105.1	96
7.02	3.23	89.6	95.7	95.7	113.2	108.8	103.4
9.22	2.03	99.2	88.4	91	76.3	65.2	76.3
5.15	2.42	111.8	103.7	117.4	108.1	91.4	113.7
9.32	3.05	103.7	98.3	101.9	97.5	97	101.3
4.86	3.91	127.4	94.5	85.9	93.5	77.6	90.5
4.65	4.77	146.1	91.6	101.5	94.3	92.9	92.6
4.69	3.11	122.1	118.9	107.7	89.6	97.3	92.5
4.88	2.83	106.5	97.2	115.4	102.2	100	96.3
7.58	2.23	104.1	100.4	101.4	125.7	110.6	130.2
4.81	1.81	86.7	84.9	71.1	135	123.3	143.9

6.55	3.03	92	101.4	114.9	84.3	87.8	86.4
9.5	3.43	94.4	94.8	121.1	103.7	101.8	103.2
5.83	2.23	118.4	83.2	111.7	106.9	99.1	116
5.52	2.81	102.4	97.6	125.9	80	121.4	98.7
7.78	1.9	97.3	96.3	93.2	103.5	142.3	91.5
5.02	2.7	103.9	97.8	111.8	87.3	98.7	96.1
4.31	2.19	97	122.3	98.5	105.1	94.4	112.9
6.9	3.52	107.7	98.1	97.7	95.5	95	102.5
8.12	2.49	87.1	100	82.4	116.9	101.5	101.8
8.53	2.13	107.9	103.8	111.6	84.8	96.9	120.2
6.87	2.15	103.4	63.3	125.7	100.1	95.4	87.2
8.22	1.86	93.8	98.9	93.1	109.1	121.2	100.4
6.76	1.75	134.1	98	89.4	99	83.1	98.5
5.67	2.4	87.6	86.2	107.8	120.6	94	100.3
5.83	4.05	94.4	108.8	103.3	107.8	112.4	110.4
6.19	2.03	101.9	88.3	104	106.5	112.8	110.8
6.01	1.87	101.5	92.9	106.6	100.6	87	93.6
5.1	2.36	121.1	89.1	100.9	102.1	97.3	101.9
8.81	2.1	88.2	103.4	116.2	93.3	111.5	91.7
6.25	2.15	101.9	84.1	100.1	110.9	121.1	124.1
5.69	2.18	88.5	90.2	114.2	100	103.6	87.7
4.82	1.71	87.4	85.6	78	124	96.9	104.5
9.09	2.56	106.3	102.1	104.1	119	105.4	96.8
5.62	2.15	102.4	108.5	98.6	94.1	76.3	90.2
8.48	1.91	86.3	65.9	111.8	104.1	129.8	117.1
7.06	3.59	99.5	101.3	98.1	99.8	118	112.5
7.02	2.16	61.7	94.8	92.7	111.9	120.9	95.3
6.32	2.7	93	102.1	98	111.1	122.8	123
9.94	1.95	107	97.7	95.5	105.2	95	109.5
5.91	2.26	85.4	105.3	123.4	95.4	105	109.3
6.62	1.66	84.1	106.4	88.8	103.9	88.8	117.1
6.7	2.3	121.3	100.1	102.1	75.2	85.7	124.3
6.86	4.32	104.1	99.5	99.8	98.2	96.9	105.1
8.02	1.82	105	93.8	71.2	112.8	126.7	125.2
8.19	2.88	98.7	98.6	102.9	106.1	108.1	105.1
8.79	2.34	90.3	98.7	102.4	93.4	108.1	113
7.88	3.02	96.3	83.6	105.6	92.2	95.5	100.2
5.47	2.43	100.8	102.1	95.5	94.5	104.6	100.1
9.38	2.2	72.6	65.9	82.3	92.1	103	249.4
8.85	2.9	102.8	108.5	105.7	115.5	106.1	102.8
6.21	2.55	98.8	98.8	108.7	122.6	85.2	93.8
9.33	2.93	85.3	82.8	79.9	87.7	99.6	102.6
8.25	1.94	99.2	86.9	114.4	110.8	68.3	94.4
5.97	2.52	95.7	97.4	111.7	96.5	112.3	100.7
8.18	2.17	100.2	94.8	110.2	109.8	98.8	110.6
8.72	3	89.1	88.7	92.2	127	124.9	118.1
4.97	2.77	116.9	112.2	89.2	91.8	84.8	113.2
5.85	2.6	107.1	103.6	99.5	124.4	116.2	116.3
5.06	4.33	110.6	93.5	88.1	95.3	88.2	96.8
7.83	4.75	100.7	105.3	98.2	99.9	94.7	89
6.15	2.54	119.8	110.5	114.3	89.9	85	72.2

10.64	2.21	81.5	90.2	98.7	76	85	87.3
7.02	2.08	108.7	94.9	77.9	95	118.3	130.7
6.21	2.96	105.2	97.8	104.4	113.3	109.9	94.4
6.37	2.02	121	109.7	120.2	139.8	147.4	129.2
10.64	1.73	112.2	104	100.4	99	104.8	113.8
7.42	1.95	65.6	103.3	89.9	105.7	118	116
5.25	2.28	109.7	109.6	105.8	116.8	102.5	107.6
7.97	2.95	95.3	96.7	109.8	88.9	94.4	93.4
5.34	1.8	90.6	90.4	91.2	113.3	107.4	84.1
5.1	2.44	111.1	101	118.1	88.6	97.4	87.8
5.97	2.47	96.5	106.5	105.6	104.2	90.9	102.1
5.9	2.62	103.9	90.4	112.2	98.9	93.2	105.2
7.59	4.14	100.6	96.4	105.6	97.8	103.8	100.2
9.39	2.32	98.2	100.4	95.4	95.5	97.4	97
6.09	2.7	102.2	95.4	90.8	107.9	105.4	96.7
8.27	2.49	95.4	90.2	86.3	119	89.4	91.9
6.06	2.33	146	124.3	164.2	52.5	61.1	66.3
6.8	2.73	84.8	82	75	139.7	135	147.8
6.48	1.69	114.3	101.2	99.1	107.5	97.1	114.4
6.52	2.72	102.3	105.4	86.6	92.4	97.7	96.9
5.19	2.25	98.8	109.2	104.8	100.6	101.5	92.7
8.95	2.27	102.1	94.7	103.4	108.6	98	113.1
5.26	1.93	93.7	83	92.3	86.2	63.9	104.3
8.03	2.25	109.6	84.5	94.9	98.7	83.4	97.2
5.16	1.96	102.6	97.9	110.7	120.2	110.9	95.5
5.94	2.65	108	107.6	87.6	99.2	68.8	98.1
10.29	2.25	123.4	97.8	101.5	90.5	89.8	97
6.92	2.92	108	110.6	106.5	94.5	94.1	96.5
5.47	1.98	86.7	84.2	101.3	110.7	74.4	118.6
9.06	2.82	108	96	116.5	108.8	99.8	100.3
6.92	2.06	92.6	90.4	108.4	99.7	99.7	95.8
8.35	3.95	108.9	108.2	106.2	117	113.4	112.6
5.62	3	94.9	93.2	97.3	109.6	120.5	124.9
5.82	2.37	114.7	97.8	99.2	109.2	104.7	111.3
4.32	1.97	141.6	87.9	83.7	76.3	105.8	87.9
8.82	2.14	79.9	99.9	92	102.4	83.9	98.1
9.55	2.49	106.9	98.8	84.3	105.1	109.7	106.2
7.53	1.64	89.2	101.3	102	127.2	154.6	102
8.85	2.36	114.4	103.6	103.8	93.5	93.9	102.8
5.83	2.04	132.5	97.5	68	99.2	106.9	98.7
6.51	2.25	106.6	94.9	91.2	121.4	99.2	120.6
8.73	1.78	106.7	115.8	105.7	111.9	80.8	125.3
4.94	2.24	87.7	131.9	113	91.1	70.5	74.9
4.83	2.41	115.7	99.3	100.3	104	88.6	117
5.17	2.41	101.1	109.2	105.7	117.3	113.8	102.9
6.35	2.24	105	91.9	118.4	108.4	90.9	82.3
5.59	2.65	102.2	102.2	120.1	105.3	111.2	112.5
7.06	1.94	131	143.6	110.1	74.7	84.5	82.1
5.48	2.64	101.1	97.1	93.7	93.6	92.7	109
7.23	3.09	97.4	106.1	110.2	112.4	102.9	101.2
8.21	1.66	105.4	96.5	100.1	109.3	101.3	98.2

6.96	5.58	101.1	104	102.5	103.4	91.4	106.7
7.24	2.78	118.8	100.5	118.7	83.3	93.2	86.4
5.26	2.65	93.4	93.7	98.9	104.3	111.6	107.6
6.9	1.79	107.5	68.3	97.9	104.1	132.8	104.9
5.74	1.95	97.5	85.2	87.7	104.8	115.2	106.6
4.97	1.74	90.8	86.7	78.6	100.6	101	114.8
5.53	2.24	88.6	90.9	101.4	95.3	104.8	105.1
9.38	2.26	89.7	103.1	98.4	104.3	109.4	114
7.64	3.44	106.4	101.6	104.7	88.8	98.7	96.8
9.17	2.35	91.9	98.8	87	108.9	100.6	94
6.04	2.4	91.1	82.8	95.3	96.9	116.7	99
9.42	2.36	105	98.7	105.4	92	101.5	102.4
5.27	2.95	91.1	94.4	90.6	113.9	112.1	106.9
6.38	2.12	104.4	102.8	115.5	112	102.8	110.8
8.78	2.67	111.1	90.4	91.1	116.8	116.5	123.7
7.11	2.04	100.8	116.9	117.2	114.8	103.8	102
7.02	2.69	100.2	99.4	95.8	98.4	102	103.8
6.07	2.24	76.2	86.3	128.7	119.4	132.9	104.4
8.19	1.81	87.9	101.4	93.9	95.2	118.7	106
4.89	2.96	107.6	112.8	104.1	88	95.9	100.5
4.56	2.47	96.3	101.4	99.3	95.1	100.8	98.5
6.86	2.26	83.2	114.5	102.5	109.5	117.2	107.4
5.2	2.07	101.4	105.8	99.1	103.2	95.4	105.5
7.78	1.98	138.7	101.4	113.5	111.4	105.3	107.9
7.77	2.51	101.9	101.4	98.1	97.3	85.8	94.9
7.66	1.74	110.1	108.9	91.8	83.3	87.9	91.9
6.83	1.82	90.7	110.4	102.6	104.1	92.4	85.4
8.59	1.86	92.4	85.4	86.2	105.5	109.9	104.3
4.46	2.04	124.5	85.7	88.5	87.4	101.1	84.5
8.1	2.36	108.7	117.3	92.7	109.1	107.8	95.7
8.02	2.63	108.9	112.2	111.1	93.4	107.6	109.9
5	2.27	97.8	107.8	106.6	105.3	115.1	109.2
8.78	1.77	107.9	109.4	99	123.9	104.1	90.1
5.57	3.15	114.2	110	99.9	101.8	104.8	98.3
5.48	2.6	107.2	123.9	120.6	98.5	90.3	113
7.81	1.65	70	121.6	87	98.8	105.4	94.9
7.61	1.94	100.5	103.7	95.4	84.7	96.3	110.3
5.54	3.76	95.2	106.2	66.7	142.7	105.2	117.6
7.01	2.36	110.4	105.6	102.8	105.2	97.1	99
8.56	2.01	90.3	98.7	101.9	101.5	113.6	112
7.12	2.25	95.1	117	95.1	86.8	91.6	86.3
9.54	2.09	90.6	70.7	91.6	111.2	109.1	114.1
8.38	4.09	103.2	98.4	105.5	104.5	112.1	109
6.11	2.62	108.3	103.5	112.8	104	114.7	90.5
6.58	1.8	83.5	65.9	107.7	93.1	110.5	110.3
6.61	2.62	105.3	86.2	110	96	100.4	107.9
6	2.33	98.7	78.4	97.3	107.6	105.5	92.3
7.55	2.28	99.2	91.2	97.5	83	107.3	105.1
5.07	2.51	93.6	92.9	118.4	110.9	105.2	106.6
8.66	2.13	112.9	86.9	53.9	128.3	103.5	94.5
9.42	3.18	92.9	98.7	109.3	107.6	103.9	115.5

5.43	1.95	93.5	89.8	88	106.1	90.5	122.2
9.25	2.05	102.2	99.8	89.5	96.1	105.4	98.1
7.87	2.02	108.6	81.6	98.1	106.3	130.8	83.6
6.16	2.27	119.2	93.8	105.8	109.2	111.6	112.2
7.88	1.98	124.8	107	104.1	124.2	113.6	99.3
9.01	2.02	88.5	93.9	85.4	119.1	101.4	110.3
5.39	1.74	78.6	115.6	128	100.8	92.2	112.9
7.68	2.2	106.3	103.8	106.2	117.5	114.4	118.5
7.23	1.68	109	89.5	108.7	79.2	87.7	87.2
4.91	1.87	129.9	101.3	94.6	88.1	100.1	91.4
5.34	2.11	84.8	102.6	105.3	101.9	95.2	94.6
9.51	2.59	104.9	97.2	100.7	106	84.6	89.1
10.08	2.2	111	99.2	102.7	107	117.7	106.6
9.74	7.56	100.2	102.3	103	100	108.8	103.8
5.6	1.79	100.7	114.9	93.1	92.7	106.4	83.8
8.43	2.03	121.6	90.9	100.1	77.1	82.1	80.5
7.24	1.83	77.1	87.8	106.5	99.8	101.6	87.4
5.86	4.71	97.3	93.1	96.2	106	109.5	109
6.2	3.03	103.3	100.7	96.1	108.2	106.9	100.1
6.67	2.93	97	103.7	97.6	93.3	105.7	99.5
9.69	2.11	130.4	90.4	91.3	78	90.9	104.3
6.8	1.76	104.5	103.1	108.2	100	106.1	98.3
4.67	2.14	105.8	94.3	85.1	100.1	97.8	112.3
5.08	1.93	101.6	106.6	128.8	91.1	83.6	102
8.82	1.64	101.3	99.5	97.1	95.1	92.9	94.1
7.03	4.82	103	105.3	95.9	102.8	95.9	97.9
10.01	1.89	85.2	137.1	105.1	96	114.8	87.3
8.63	2.09	109.9	82.1	113.1	108.9	94.2	115.7
6.55	1.83	93.8	108.1	84.1	89.6	111.2	95.4
7.52	2.09	118.2	128.4	107.4	95.2	96.8	82
9.06	2.53	103.7	110.5	98.2	116.5	114.7	118
9.6	1.73	130	99.3	88.6	87.9	111.2	97
6.32	4.17	107.5	105.4	110.6	90.7	92.3	103.7
5.73	1.61	108.5	95.1	90.4	142.8	103.8	109.8
6.28	1.97	108	95.8	101.5	96.1	88.2	98.5
8.54	2.15	89.7	93.4	89	113.6	108.1	107.1
10.18	2.64	104.2	104.1	108.8	97.7	99.7	106.2
8.51	2.84	102.4	97.7	99.8	95.4	97.3	101.7
6.1	1.96	94.8	83.5	101	107.4	95.2	101
11.84	2.41	112.3	102.8	116	96.9	101.6	88.7
9.58	2.33	99.7	93.1	96.2	104.8	94.9	110.7
5.83	1.82	96.9	122	101.1	80.9	117.1	116.4
7.84	2.39	102.1	86.8	89.5	88.5	95	84.8
9.17	2.47	105.7	98.9	104	101	104.7	99.3
4.6	2.22	108.6	93.1	106.3	113	109.5	89.5
8.54	5.48	91.6	89.8	90.8	90.6	87.5	90.6
6.35	2.39	99.1	104.1	107.2	103.9	94.8	89.2
7.36	2.18	107.7	107.1	101.1	101.7	95.2	123.9
9.6	1.62	88.6	90.7	92.7	121.4	115.7	120.1
7.88	1.96	95	97.8	92.5	94.8	70	109.7
6.9	1.98	88.7	98.8	86.6	108	110	98.6

8.51	1.74	111.1	101.3	117.2	96.1	98.1	99.7
9	1.97	92.5	104.9	99.8	105.9	103.4	117.5
8.48	2.13	121.4	109.6	106.8	95.2	105.1	101.3
6.01	2.66	101.1	101.2	121.8	98.2	98.2	111.8
5.81	2.43	103.2	107	91.4	92.2	103.6	99.4
9.73	3.16	95.7	103.7	96.6	95.9	101.5	95.1
5.21	2.35	94	101.1	99.5	99	100.1	107.6
9.55	1.86	127.9	78.3	82.3	129.2	118.2	160.8
7.85	2.08	106.9	113.9	123.4	104.9	101.7	103.4
7.96	3.47	93.6	88.2	89	101.8	105.7	117.5
6.8	1.88	92	111.3	100.7	102.6	99.6	86.3
9.72	1.67	86	114.6	117.9	86.7	89.1	105.9
7.64	2.53	95.9	90.4	92.3	120.1	121.1	116.9
5.47	1.95	93.4	102	112.7	99.6	108.5	98.9
9.13	1.78	111.9	96.1	116.6	95.2	89.2	115.8
5.01	2.25	91.2	97.6	97.3	106.8	114.5	113.6
8.1	2.3	102.2	105.6	103.2	104.8	113.9	103.6
8.84	1.77	96.2	86.9	99.7	75.5	122.2	76.5
8.97	1.73	88.6	91.4	105.5	115.7	107.7	103.3
8.15	2.79	95.9	103.2	96.6	104.5	110.3	112.1
5.21	1.84	95.3	96.5	86.7	97.1	91.5	92.3
9.99	1.85	106.7	89.1	82.2	120.9	109.8	112.2
9.35	1.66	89.9	93.3	101.2	112.8	107.3	104.6
7.75	2.14	104.8	92.3	91.5	99.8	97.5	88.6
5.88	2.32	113.7	82.1	108.4	92.9	105.2	97.2
5.85	2.14	90.7	116.5	106	95.2	84.8	87.5
8.76	1.7	83.3	77.7	127.5	77.6	75.1	118.6
7.36	3.05	89.6	91.3	90.2	116.6	116.9	113
6.1	2.79	107	106.6	106.6	102.3	96.4	95.3
9.33	2.15	104.6	107.2	90.8	89.7	101.3	109.7
7.88	1.65	119.1	94.7	83.2	98.4	108.2	85.1
6.28	1.7	36	106.3	119	131.8	79.9	113.8
5.11	2.36	129.3	97	99	109	79.6	111.5
6.6	11.15	105.8	103.8	105.1	93.3	88.1	87.4
4.97	2.84	104.6	93.6	97.2	85.6	86.6	84.9
9.52	2.75	105.5	99.1	97.7	101	98.4	95.7
6.8	2.18	92.4	100.7	93.3	97.5	113.8	100.1
8.25	2.19	103.3	102.4	92.4	106	103.4	101.1
8.95	2.44	99.5	83.4	96.6	120.1	118.3	115
5.8	2.03	108.4	107.2	109.2	82	82.9	105.6
5.21	2.02	101.8	98.1	105.6	108.9	111.2	97.6
7.28	2.09	109	94.2	92	103.7	90.7	87.7
4.54	2.22	101	94.1	93.5	103.2	122.1	127.4
5.4	2.73	106	101.5	113.5	109.9	106.2	110.9
9.85	2.08	97.5	117.9	86.6	90.3	102.5	96.7
7.72	5.73	114.1	102.9	104.3	99.6	97.5	91.4
7.62	2.16	99.8	95.6	109.7	102.9	99.8	116.8
8.92	1.61	96.9	100.7	117.2	98.6	93.4	96
6.04	1.85	96.3	92.6	92	109.2	93.8	127.8
8.63	1.91	110.1	75.7	82.6	118.5	104.9	119
5.5	1.74	93.2	100.5	98.4	105.3	98.8	93.3

6.44	2.17	102.7	102.3	105.2	97.5	96.3	98.4
8.44	2.48	79	85.9	65.9	114.7	124.1	119.5
9.38	1.88	80.5	89.4	113.7	99.8	71.7	95.8
6.33	1.84	114.1	106.4	101.3	85.3	94.9	86.2
8.68	2.74	110.6	91	100.8	99.8	95.4	108.5
9.17	4.1	99.4	107.4	110.2	96.2	103.3	107.8
8.24	2.07	108.1	103.4	108.2	94.7	98.5	102.6
5.22	1.82	102.9	102.4	97.7	110.9	100.1	101.8
8.37	2.63	100.3	99.8	91.7	81.8	84.7	99.7
8.09	1.75	116.4	100.8	97.1	98.6	84.7	95.3
7.3	2.06	107.5	102.6	106.7	93	94	97.7
9.07	1.92	95.4	101.1	92.9	120.7	98.6	124
6.68	2.35	94.1	106.3	106.7	105.4	107.2	119.6
9.48	1.68	112.1	98	113.9	104.8	113.9	98.4
5.86	2.22	102.7	107.4	109.6	93.2	88	114.2
6.76	2.07	121.5	84.7	108.5	89.2	78.9	105.4
8.73	1.87	79.2	86.9	120.1	91.8	112.3	122.1
9.88	1.67	103.1	90.7	101.9	100.9	109.6	124.7
4.88	1.69	103.2	104.8	92.6	98.3	92.5	89
5.85	2.81	99.5	90.1	92.8	106.1	94.6	97
7.75	5.34	102.9	101.1	100	86.1	85.2	81.8
6.6	1.9	124.5	71.1	107	88	83.9	107.4
5.2	1.81	85.4	99	99.9	88.4	99	122.5
7.5	1.82	102.8	94.4	100.3	99.6	102.9	100
11.15	1.79	106.7	108	95.9	99.4	119	119.1
7.42	2.51	87.9	96	94.3	98.8	98.1	101.8
8.27	1.82	96.3	108.4	87.4	97.2	99.8	106.9
9.52	1.93	108.9	97.6	98.5	95.9	96.3	99.1
9.14	2.52	96.2	88.7	104.1	98.7	94.7	111.8
5.55	1.67	99.4	88.5	77.3	92.2	101.3	81.6
8.4	2	117.1	93.7	104.7	92.3	105.8	108
7.65	2.35	104.2	99.3	95.6	99.5	89.4	90
6.87	1.79	118.2	85.4	88.4	115.8	88.1	102
5	2.35	97.5	99.3	103.4	106.8	104.6	105.1
7.61	4.32	114.4	118.3	114.3	75	71.5	69.9
7.34	2.54	97.3	88.5	89.8	104.2	100.4	110.3
9.32	1.83	92.3	102.2	84.9	113.5	107.9	93
6.67	1.8	96.9	94.7	112	85	120	112.9
5.29	2.62	91	87.2	110.3	110.4	111.8	110
7.88	2.07	94.8	96	95.1	97.7	99.7	93.2
6.27	1.85	93.4	101	104.8	99.2	102.3	128.6
5.95	2.53	106	108.5	96.6	92.2	94.1	86.9
9.5	2.16	103.8	106.3	119.2	107.9	99.2	98.2
5.73	1.67	101.8	84.3	92.7	98.1	92.3	106.2
7.49	2.09	94.2	103.7	98.4	89	88.1	99.3
6.55	1.89	105.4	96	103.9	96.8	82.7	98.2
5.53	2.07	99	94.3	97.7	112.4	114.7	109
6.51	2.13	89	92.9	114.2	101.2	103.3	96.9
8.9	2.19	110.2	104.1	86.4	115.8	111.2	115.7
6.93	1.88	92.3	86.6	112.3	127.7	94.6	87.8
6.35	4.47	91.6	97.3	90.6	107.4	107.4	96.4

5.81	1.86	75.5	96.4	112.1	90.3	116.1	104
10.27	2.1	104.7	96.2	105.6	108.5	124	116.4
6.81	2.02	128.7	106.8	100.1	118.8	108.3	122
9.33	2.02	132	113.7	94	130.4	85.2	121.8
7.43	2.14	105	90.8	97.1	101.1	97.7	89.2
8.79	2.37	112.9	93.5	104.7	106.3	104.2	89.7
8.6	2.81	102.8	102.5	105.8	90.9	85.1	85.5
6.99	2.56	100.6	98.8	105.3	105.2	101.8	116
5.03	2.53	99.4	110.1	105.9	103.5	91.2	111.3
6.64	1.71	103.2	99.7	122.4	76.4	84.9	98.3
6.54	2.59	103.5	109.6	94	93.7	80.5	84.7
6	2.45	85.5	82.4	87.5	84.1	78.3	85.2
5.87	1.63	101.4	101	101.7	94.7	96.2	90.3
6.71	1.99	112.6	85.6	100.4	92.8	78.6	102.9
9.03	2.26	85.4	92.3	90.7	115.4	102.4	105.6
6.24	2.31	81.9	103.6	98	97.1	98.3	94.9
7.53	1.67	100.7	112.9	96.4	120.4	97.9	134.7
8.29	1.69	95	106.2	88.8	98.7	98	110.5
5.11	1.82	95	85.6	126.3	99.7	139.3	85
9.92	1.8	110.9	98.5	95.6	103.5	92.1	107.8
6.27	2.44	88.8	99.3	95.4	106.6	100.5	93.4
5.1	1.93	90.2	92.2	90.9	101.5	109.9	102.2
10.48	2.02	98.6	110.5	103.5	104.1	102.8	117.4
9.42	1.67	86.8	102.3	103.1	98.8	98.5	99.8
5.82	1.76	94.4	100.2	126	70.3	109	102.1
6.44	2.45	96.5	95.9	97.3	104.8	98.2	92
8.32	2.01	99.9	102.6	92.5	98	99.2	101.9
10.29	2.31	105.1	95.4	92	109.5	122	121.3
7.39	1.69	74.1	90.5	101.8	73.6	82.9	74.8
8.21	2.44	91.7	96.6	90.4	118.8	116.6	112.5
8.95	2.13	99.7	97.6	96.4	93.7	103.1	99.2
8.7	2.82	98.8	94.6	100.3	106.6	102.7	110.4
8.66	2.43	103.6	98.9	104.7	110.2	108.3	101.5
6.54	1.7	104.9	99.3	97.6	113.8	112.3	115.7
5.86	2.1	113.4	89.4	81.8	94.7	121.5	99.1
4.96	1.62	121.6	104.6	113.4	94.8	94.4	117.2
8.19	1.94	90.9	82	101.6	123.4	118	113.4
6.15	2.03	109.8	102.3	100	100.6	99.3	112.1
6.54	1.82	96.9	103.3	94.3	110	108.4	110.7
8.47	2.28	99.6	96	102.1	110.1	98.8	98.2
5.83	1.67	89.1	78.9	108.7	118.3	97.5	74
7.17	2.33	99.1	92.4	92.5	98.2	92.2	97.2
6.2	1.89	101	91.9	94.2	125.1	125.3	122.8
7.02	2.45	96.7	90.6	102	94	83.8	81.8
6.65	2.36	112.1	112.6	99.3	93.7	92.5	87.3
9.99	2.03	107.7	106.7	104.7	105.3	83.9	89.5
8.37	1.94	87.6	105.4	96.8	111.8	116.5	102.8
7.05	1.61	91.4	77.4	100.4	122.3	114.8	105.5
5.96	2.51	95.7	97.4	104.6	102.9	108.4	96.8
6.81	4.26	104.9	92.9	92.7	104	92	110.7
9.33	2.14	91.2	106.1	103.4	100.1	96.8	98.5

4.93	5.54	91.3	98	94.6	97.2	99.1	97.7

ed)：F1，131

Accessior	Gene Name	Descripti	Coverage	# Peptide	# PSMs	# Unique	# AAs	MW [kDa]
Q09666	AHNAK	Neuroblas	69	254	670	252	5890	628.7
Q15149	PLEC	Plectin C	56	255	593	248	4684	531.5
P21333	FLNA	Filamin-A	61	134	753	116	2647	280.6
P35579	MYH9	Myosin-9	62	135	810	112	1960	226.4
Q9Y490	TLN1	Talin-1 C	70	144	442	128	2541	269.6
O75369	FLNB	Filamin-B	57	124	404	107	2602	278
Q14315	FLNC	Filamin-C	53	128	413	110	2725	290.8
Q14204	DYNC1H1	Cytoplasm	42	172	327	172	4646	532.1
Q13813	SPTAN1	Spectrin	56	127	256	3	2472	284.4
A0A0D9SF5	SPTAN1	Spectrin	55	125	250	1	2457	282.7
P35580	MYH10	Myosin-10	48	102	279	81	1976	228.9
H3BPE1	MACF1	Microtubu	22	148	198	3	7555	856.3
Q9UPN3	MACF1	Microtubu	22	145	195	2	7388	837.8
Q01082	SPTBN1	Spectrin	47	105	205	105	2364	274.4
P12814	ACTN1	Alpha-act	64	55	344	9	892	103
P18206	VCL	Vinculin	66	74	272	74	1134	123.7
P02452	COL1A1	Collagen	63	64	216	64	1464	138.9
Q9NZM1	MYOF	Myoferlin	50	95	192	95	2061	234.6
A0A087WVQ	CLTC	Clathrin	44	63	192	63	1679	191.9
P46821	MAP1B	Microtubu	40	84	182	82	2468	270.5
P78527	PRKDC	DNA-depen	28	110	164	110	4128	468.8
H9KV75	ACTN1	Alpha-act	60	46	318	3	822	94.8
P46940	IQGAP1	Ras GTPas	43	66	180	64	1657	189.1
P02751	FN1	Fibronect	37	65	182	65	2386	262.5
O43707	ACTN4	Alpha-act	63	56	296	39	911	104.8
Q9P2E9	RRBP1	Ribosome-	67	72	173	71	1410	152.4
Q99715	COL12A1	Collagen	35	87	160	87	3063	332.9
P49327	FASN	Fatty aci	34	67	144	67	2511	273.3
P11021	HSPA5	Endoplasm	63	45	283	42	654	72.3
P14618	PKM	Pyruvate	71	36	309	16	531	57.9
P08238	HSP90AB1	Heat shoc	61	46	273	29	724	83.2
P11142	HSPA8	Heat shoc	77	46	221	20	646	70.9
A0A087WTA	COL1A2	Collagen	54	51	145	51	1364	129.1
P07814	EPRS	Bifunctic	47	62	124	61	1512	170.5
P07900	HSP90AA1	Heat shoc	57	45	232	30	732	84.6
P08670	VIM	Vimentin	70	42	578	36	466	53.6
P13639	EEF2	Elongatic	59	49	230	48	858	95.3
P27816	MAP4	Microtubu	55	59	129	1	1152	120.9
E7EVA0	MAP4	Microtubu	27	60	130	1	2297	245.3
P02545	LMNA	Prelamin-	68	47	188	46	664	74.1
P53396	ACLY	ATP-citra	51	50	153	50	1101	120.8
P34932	HSPA4	Heat shoc	66	53	113	48	840	94.3
A0A2R8Y79	ACTB	Actin, cy	68	18	899	9	309	34.1
P08133	ANXA6	Annexin A	63	44	159	16	673	75.8
E9PGZ1	CALD1	Caldesmon	60	40	168	3	536	61.7
E7EX44	CALD1	Caldesmon	61	39	170	1	557	64.1
Q07065	CKAP4	Cytoskele	66	38	170	36	602	66
Q05682	CALD1	Caldesmon	42	39	170	1	793	93.2
Q92616	GCN1	eIF-2-alp	31	71	112	71	2671	292.6
P05787	KRT8	Keratin,	71	41	239	32	483	53.7

P26038	MSN	Moesin OS	65	46	202	32	577	67.8
P14625	HSP90B1	Endoplasm	47	43	215	41	803	92.4
P12111	COL6A3	Collagen	25	69	98	55	3177	343.5
P50454	SERPINH1	Serpin H1	63	25	178	25	418	46.4
P55072	VCP	Transitic	61	41	122	41	806	89.3
P62736	ACTA2	Actin, ac	62	21	549	12	377	42
P13667	PDIA4	Protein d	57	38	133	38	645	72.9
Q9Y4L1	HYOU1	Hypoxia u	44	41	108	41	999	111.3
Q14764	MVP	Major vau	57	38	120	38	893	99.3
P04406	GAPDH	Glycerald	69	22	416	22	335	36
P13797	PLS3	Plastin-3	54	36	139	29	630	70.8
P07237	P4HB	Protein d	60	37	205	37	508	57.1
A0A024R4E	HDLBP	High dens	39	52	120	51	1268	141.4
P22314	UBA1	Ubiquitir	47	40	101	40	1058	117.8
H3BR70	PKM	Pyruvate	56	23	203	3	366	40.2
Q86UP2	KTN1	Kinectin	44	61	83	61	1357	156.2
P30101	PDIA3	Protein d	63	36	159	30	505	56.7
P33176	KIF5B	Kinesin-1	50	45	76	45	963	109.6
O00410	IPO5	Importin-	40	35	91	33	1097	123.6
P53621	COPA	Coatomer	42	50	95	50	1224	138.3
P04264	KRT1	Keratin,	50	40	121	33	644	66
P10809	HSPD1	60 kDa he	76	36	114	36	573	61
P50990	CCT8	T-complex	63	34	107	34	548	59.6
Q07954	LRP1	Prolow-de	15	66	89	66	4544	504.3
Q01813	PFKP	ATP-deper	45	34	102	28	784	85.5
P68104	EEF1A1	Elongatic	70	26	302	26	462	50.1
P06733	ENO1	Alpha-enc	61	24	169	22	434	47.1
Q7KZF4	SND1	Staphyloc	45	39	106	39	910	101.9
P42704	LRPPRC	Leucine-r	41	52	85	52	1394	157.8
P12270	TPR	Nucleoprc	26	59	76	58	2363	267.1
P38646	HSPA9	Stress-7C	48	35	105	34	679	73.6
E7EUU4	EIF4G1	Eukaryoti	32	45	86	1	1560	171.5
Q04637	EIF4G1	Eukaryoti	31	45	86	1	1599	175.4
P00558	PGK1	Phosphogl	76	29	125	25	417	44.6
P27797	CALR	Calreticu	60	26	146	26	417	48.1
E7EX90	DCTN1	Dynactin	42	43	74	43	1256	139
P07355	ANXA2	Annexin A	73	31	228	31	339	38.6
P68366	TUBA4A	Tubulin a	55	21	231	6	448	49.9
P02461	COL3A1	Collagen	33	34	99	34	1466	138.5
P07437	TUBB	Tubulin b	68	21	259	4	444	49.6
F5H5D3	TUBA1C	Tubulin a	50	22	201	7	519	57.7
P50991	CCT4	T-complex	65	30	76	29	539	57.9
Q8WUM4	PDCD6IP	Programme	52	44	77	44	868	96
O75083	WDR1	WD repeat	51	27	107	27	606	66.2
P05783	KRT18	Keratin,	71	34	124	33	430	48
P21980	TGM2	Protein-g	57	30	121	21	687	77.3
P48643	CCT5	T-complex	71	36	87	35	541	59.6
P68371	TUBB4B	Tubulin b	67	21	241	3	445	49.8
P52272	HNRNPM	Heterogen	57	38	94	38	730	77.5
P49368	CCT3	T-complex	55	30	86	30	545	60.5
Q92598	HSPH1	Heat shoc	45	35	74	30	858	96.8

P60842	EIF4A1	Eukaryoti	63	25	107	14	406	46.1
P16615	ATP2A2	Sarcoplas	35	36	77	36	1042	114.7
P78371	CCT2	T-complex	58	26	79	26	535	57.5
E7ETU9	PLOD2	Procollag	37	24	106	1	703	81.1
P35221	CTNNA1	Catenin a	48	38	69	38	906	100
A0A0G2JIW	HSPA1B	Heat shoc	55	30	105	24	642	70.1
P04083	ANXA1	Annexin A	67	22	111	22	346	38.7
P15311	EZR	Ezrin OS=	58	39	133	26	586	69.4
Q15075	EEA1	Early enc	37	48	56	48	1411	162.4
P49588	AARS	Alanine--	46	37	72	37	968	106.7
Q01518	CAP1	Adenylyl	63	27	107	25	475	51.9
Q14152	EIF3A	Eukaryoti	34	48	86	48	1382	166.5
Q6ZN40	TPM1	Tropomyos	65	31	146	2	326	37.4
E9PGC8	MAP1A	Microtubu	16	45	65	44	3041	331.1
P17987	TCP1	T-complex	58	24	71	24	556	60.3
P11047	LAMC1	Laminin s	27	37	67	37	1609	177.5
P53618	COPB1	Coatomer	37	28	70	28	953	107.1
Q01995	TAGLN	Transgeli	85	19	243	18	201	22.6
F8WD26	LMO7	LIM domai	31	44	60	3	1631	186.1
P12956	XRCC6	X-ray rep	54	35	77	35	609	69.8
P50395	GDI2	Rab GDP d	63	27	81	19	445	50.6
P35908	KRT2	Keratin,	48	31	66	23	639	65.4
P54652	HSPA2	Heat shoc	57	35	93	19	639	70
E5RK69	ANXA6	Annexin C	59	30	96	2	460	51.7
Q9Y678	COPG1	Coatomer	46	32	77	30	874	97.7
O00469	PLOD2	Procollag	34	24	94	1	737	84.6
P04075	ALDOA	Fructose-	63	24	172	19	364	39.4
P05023	ATP1A1	Sodium/pc	41	35	60	35	1023	112.8
P13645	KRT10	Keratin,	45	30	72	24	584	58.8
Q99832	CCT7	T-complex	58	28	71	28	543	59.3
A0A0S2Z4C	TPM1	Tropomyos	64	29	136	2	284	32.7
E9PMS6	LMO7	LIM domai	38	42	56	1	1275	145.3
A7XZE4	TPM2	Beta trop	63	26	163	6	284	33
Q9P0K7	RAI14	Ankycorbi	38	39	54	39	980	110
P63010	AP2B1	AP-2 comp	35	34	72	15	937	104.5
P19367	HK1	Hexokinas	38	36	71	33	917	102.4
P06576	ATP5F1B	ATP synth	53	21	82	21	529	56.5
Q13885	TUBB2A	Tubulin b	54	18	223	2	445	49.9
J3KN16	ECPAS	Proteasom	23	39	52	39	2017	223.6
P08727	KRT19	Keratin,	75	31	81	19	400	44.1
P41252	IARS	Isoleucin	34	41	63	41	1262	144.4
Q14195	DPYSL3	Dihydropy	52	20	77	16	570	61.9
P35606	COPB2	Coatomer	41	32	69	32	906	102.4
P11586	MTHFD1	C-1-tetra	37	33	61	32	935	101.5
P07942	LAMB1	Laminin s	24	42	64	42	1786	197.9
Q96AY3	FKBP10	Peptidyl-	42	25	102	24	582	64.2
P31948	STIP1	Stress-in	62	38	80	38	543	62.6
O00159	MYO1C	Unconvent	36	35	62	35	1063	121.6
Q08211	DHX9	ATP-deper	31	37	71	37	1270	140.9
P13674	P4HA1	Prolyl 4-	54	28	80	28	534	61
Q00839	HNRNPU	Heterogen	34	28	84	2	825	90.5

A0A1W2PPS	HNRNPU	Heterogen	33	27	81	1	804	88.3
Q14008	CKAP5	Cytoskele	22	41	51	41	2032	225.4
E9PK25	CFL1	Cofilin-1	61	20	131	15	204	22.7
Q6P2Q9	PRPF8	Pre-mRNA-	21	44	58	44	2335	273.4
H0YFD6	HADHA	Trifuncti	36	27	62	27	792	86.3
Q9NYU2	UGGT1	UDP-gluco	28	37	61	35	1555	177.1
Q13509	TUBB3	Tubulin b	52	17	138	4	450	50.4
Q8IVF2	AHNAK2	Protein A	21	44	57	42	5795	616.2
Q86VP6	CAND1	Cullin-as	31	35	74	35	1230	136.3
P54136	RARS	Arginine-	46	31	66	31	660	75.3
F5H365	SEC23A	Protein t	37	24	73	22	736	82.9
Q16643	DBN1	Drebrin C	48	24	56	24	649	71.4
O43852	CALU	Calumenin	63	20	61	20	315	37.1
P60174	TPI1	Triosepho	71	17	101	17	286	30.8
P29401	TKT	Transketo	51	27	73	27	623	67.8
P11216	PYGB	Glycogen	43	35	62	29	843	96.6
Q8NF91	SYNE1	Nesprin-1	6	47	51	45	8797	1010.5
P05556	ITGB1	Integrin	32	23	87	23	798	88.4
Q10567	AP1B1	AP-1 comp	32	31	56	12	949	104.6
Q92896	GLG1	Golgi app	36	40	56	40	1179	134.5
P62258	YWHAE	14-3-3 pr	72	24	95	21	255	29.2
O60664	PLIN3	Perilipin	64	23	78	23	434	47
P35527	KRT9	Keratin,	50	27	70	25	623	62
P19338	NCL	Nucleolin	37	36	84	36	710	76.6
P02786	TFRC	Transferr	41	27	53	27	760	84.8
P27824	CANX	Calnexin	41	24	85	16	592	67.5
Q92499	DDX1	ATP-depen	47	32	64	32	740	82.4
Q14697	GANAB	Neutral a	31	28	78	5	944	106.8
A0A1W2PQ5	DDX17	Probable	47	31	71	21	731	80.4
P41250	GARS	Glycine--	45	29	71	29	739	83.1
O60506	SYNCRIP	Heterogen	43	24	57	4	623	69.6
J3KN67	TPM3	Tropomyos	55	26	123	1	285	33.2
P35998	PSMC2	26S prote	67	26	60	26	433	48.6
Q9P2J5	LARS	Leucine--	31	33	49	33	1176	134.4
Q16658	FSCN1	Fascin OS	46	23	89	23	493	54.5
P49792	RANBP2	E3 SUMO-p	18	49	58	48	3224	358
Q14789	GOLGB1	Golgin su	16	45	48	44	3259	375.8
P61158	ACTR3	Actin-rel	69	22	70	22	418	47.3
P08758	ANXA5	Annexin A	66	22	103	22	320	35.9
Q08378	GOLGA3	Golgin su	29	38	47	38	1498	167.3
Q13200	PSMD2	26S prote	34	28	58	28	908	100.1
O75643	SNRNP200	U5 small	19	36	49	36	2136	244.4
O95782	AP2A1	AP-2 comp	34	33	59	24	977	107.5
E9PLK3	NPEPPS	Aminopept	36	32	61	32	915	102.9
Q06210	GFPT1	Glutamine	46	28	52	25	699	78.8
Q02952	AKAP12	A-kinase	21	33	46	33	1782	191.4
P25705	ATP5F1A	ATP synth	56	29	70	29	553	59.7
O60701	UGDH	UDP-gluco	56	23	60	23	494	55
Q99460	PSMD1	26S prote	32	29	54	29	953	105.8
O60763	USO1	General v	37	27	45	27	962	107.8
O00571	DDX3X	ATP-depen	49	31	59	7	662	73.2

Q5TCU3	TPM2	Tropomyos	62	25	141	4	284	32.8
Q7Z6Z7	HUWE1	E3 ubiqui	11	42	52	42	4374	481.6
P67936	TPM4	Tropomyos	58	22	140	4	248	28.5
P63104	YWHAZ	14-3-3 pr	57	16	144	11	245	27.7
P54577	YARS	Tyrosine-	49	30	57	30	528	59.1
Q14247	CTTN	Src subst	49	29	56	29	550	61.5
Q02809	PLOD1	Procollag	40	25	52	24	727	83.5
P09874	PARP1	Poly [ADF	33	29	41	29	1014	113
Q5T4S7	UBR4	E3 ubiqui	8	41	47	41	5183	573.5
P26639	TARS	Threonine	42	29	67	29	723	83.4
D6REX3	SEC31A	Protein t	26	27	51	27	1251	136.1
Q96HC4	PDLIM5	PDZ and L	48	23	53	23	596	63.9
P46939	UTRN	Utrophin	14	45	46	43	3433	394.2
P18669	PGAM1	Phosphogl	58	14	75	14	254	28.8
Q14974	KPNB1	Importin	31	25	66	25	876	97.1
P48444	ARCN1	Coatomer	49	25	61	25	511	57.2
P17980	PSMC3	26S prote	55	21	52	21	439	49.2
P04843	RPN1	Dolichyl-	50	28	66	28	607	68.5
P28838	LAP3	Cytosol a	57	25	44	25	519	56.1
P48681	NES	Nestin OS	24	37	44	37	1621	177.3
Q16881	TXNRD1	Thioredox	40	23	53	22	649	70.9
P13010	XRCC5	X-ray rep	37	27	64	27	732	82.7
P07384	CAPN1	Calpain-1	36	24	52	24	714	81.8
Q3SY69	ALDH1L2	Mitochond	37	30	48	27	923	101.7
P15144	ANPEP	Aminopept	32	31	44	31	967	109.5
P37802	TAGLN2	Transgeli	79	16	93	15	199	22.4
P40926	MDH2	Malate de	62	18	92	18	338	35.5
P17655	CAPN2	Calpain-2	40	23	60	23	700	79.9
A0A087WTTPABPC1		Polyadeny	35	21	60	15	522	58.5
Q12906	ILF3	Interleuk	28	25	57	25	894	95.3
P80723	BASP1	Brain aci	78	17	50	17	227	22.7
E9PK54	HSPA8	Heat shoc	86	14	86	1	183	19.9
J3KTA4	DDX5	Probable	50	28	66	19	614	69
H0Y4R1	IMPDH2	Inosine-5	39	18	38	16	470	51
O75534	CSDE1	Cold shoc	36	33	53	33	798	88.8
E9PKU7	GANAB	Neutral a	31	24	67	1	852	96.5
P26641	EEF1G	Elongatic	42	21	79	21	437	50.1
P35241	RDX	Radixin C	42	31	100	13	583	68.5
P30153	PPP2R1A	Serine/th	43	23	57	18	589	65.3
P20700	LMNB1	Lamin-B1	46	29	48	24	586	66.4
P63244	RACK1	Receptor	70	19	61	19	317	35.1
P46060	RANGAP1	Ran GTPas	53	24	35	24	587	63.5
A0A024R57EHD1		EH domain	41	24	58	18	548	61.9
P23634	ATP2B4	Plasma me	27	32	46	24	1241	137.8
Q9Y696	CLIC4	Chloride	69	16	48	15	253	28.8
Q9ULV4	CORO1C	Coronin-1	36	23	67	22	474	53.2
P02768	ALB	Serum alb	52	26	56	26	609	69.3
A0A0C4DGECAST		Calpastat	45	28	44	1	754	81
P06748	NPM1	Nucleophc	45	13	85	13	294	32.6
P26640	VARS	Valine--t	22	27	47	27	1264	140.4
B7Z645	SYNCRIP	Synaptota	44	19	48	1	464	52

P62424	RPL7A	60S ribos	48	18	70	18	266	30
K7ELL7	PRKCSH	Glucosida	44	23	61	23	535	60.2
P27348	YWHAQ	14-3-3 pr	63	17	76	11	245	27.7
O75874	IDH1	Isocitrat	64	25	57	25	414	46.6
P36578	RPL4	60S ribos	50	23	70	23	427	47.7
P11717	IGF2R	Cation-in	15	34	40	34	2491	274.2
P27708	CAD	CAD prote	19	34	41	34	2225	242.8
A0A087WWU	TPM3	Tropomyos	65	22	102	3	227	26.4
P47895	ALDH1A3	Aldehyde	36	21	51	19	512	56.1
K7ENT6	TPM4	Tropomyos	61	22	127	2	247	28.5
P11413	G6PD	Glucose-6	47	24	58	24	515	59.2
P31939	ATIC	Bifunctic	46	23	43	23	592	64.6
Q03252	LMNB2	Lamin-B2	46	30	52	25	620	69.9
Q06830	PRDX1	Peroxirec	64	13	93	10	199	22.1
G3V3H3	KLC1	Kinesin l	39	24	47	17	609	68.7
Q9UHB6	LIMA1	LIM domai	36	26	48	26	759	85.2
Q8NBS9	TXNDC5	Thioredox	42	19	63	19	432	47.6
P31150	GDI1	Rab GDP d	50	19	50	11	447	50.6
P22102	GART	Trifuncti	35	31	47	31	1010	107.7
Q92900	UPF1	Regulator	29	28	39	28	1129	124.3
Q16555	DPYSL2	Dihydropy	37	16	48	12	572	62.3
Q5SW79	CEP170	Centrosom	23	32	42	30	1584	175.2
P56192	MARS	Methionir	35	24	44	24	900	101.1
Q9Y4G6	TLN2	Talin-2 C	11	27	58	10	2542	271.4
Q9BUF5	TUBB6	Tubulin b	46	16	100	8	446	49.8
Q5JRX3	PITRM1	Presequer	32	29	41	29	1037	117.3
P07195	LDHB	L-lactate	42	17	59	16	334	36.6
Q13310	PABPC4	Polyadeny	30	22	52	3	644	70.7
P46459	NSF	Vesicle-f	33	25	50	24	744	82.5
Q16531	DDB1	DNA damag	25	27	42	27	1140	126.9
Q14192	FHL2	Four and	70	18	47	18	279	32.2
Q9Y3I0	RTCB	tRNA-spli	42	18	42	18	505	55.2
P62195	PSMC5	26S prote	55	21	51	20	406	45.6
Q15233	NONO	Non-POU d	44	21	46	20	471	54.2
O15460	P4HA2	Prolyl 4-	47	23	59	23	535	60.9
H0YNH8	UACA	Uveal aut	23	35	41	34	1401	161.2
E9PKG1	PRMT1	Protein a	56	18	41	18	325	37.7
Q15019	SEPT2	Septin-2	68	18	44	18	361	41.5
B4DY08	HNRNPC	Heterogen	51	22	71	20	288	32
P49748	ACADVL	Very long	42	23	39	23	655	70.3
P23396	RPS3	40S ribos	77	19	87	19	243	26.7
P39023	RPL3	60S ribos	42	21	67	21	403	46.1
P00338	LDHA	L-lactate	42	18	113	17	332	36.7
P21291	CSRP1	Cysteine	68	12	62	4	193	20.6
P49411	TUFM	Elongatic	51	20	43	20	452	49.5
O43390	HNRNPR	Heteroger	33	21	45	14	633	70.9
Q16851	UGP2	UTP--gluc	42	22	53	22	508	56.9
Q96AC1	FERMT2	Fermitin	39	24	56	24	680	77.8
Q9C0C2	TNKS1BP1	182 kDa t	22	30	36	30	1729	181.7
P55884	EIF3B	Eukaryoti	27	21	40	21	814	92.4
E7EVY3	CAST	Calpastat	43	24	40	1	693	74.9

B4DKY1	CARS	Cysteine-	34	23	43	22	739	84.2
O60841	EIF5B	Eukaryoti	22	28	37	28	1220	138.7
E9PDF6	MYO1B	Unconvent	27	30	44	30	1107	128.4
P06756	ITGAV	Integrin	26	26	36	26	1048	116
Q8NC51	SERBP1	Plasminog	43	23	51	23	408	44.9
O94925	GLS	Glutamina	33	18	49	17	669	73.4
D3DQV9	EIF4G2	Eukaryoti	35	31	51	31	907	102.3
Q15365	PCBP1	Poly(rC)-	53	14	44	8	356	37.5
A0A2R8Y6J	RPL5	60S ribos	44	14	64	14	238	27
F5GZS6	SLC3A2	4F2 cell-	29	14	44	14	599	64.8
O15523	DDX3Y	ATP-depen	41	27	47	3	660	73.1
P17301	ITGA2	Integrin	23	23	38	23	1181	129.2
H7BYY1	TPM1	Tropomyos	59	21	100	6	248	28.7
Q9UHB9	SRP68	Signal re	42	24	37	24	627	70.7
P11387	TOP1	DNA topoi	38	31	43	31	765	90.7
P06737	PYGL	Glycogen	39	32	43	26	847	97.1
P54886	ALDH18A1	Delta-1-p	33	25	44	25	795	87.2
Q9BSJ8	ESYT1	Extended	25	24	35	24	1104	122.8
Q93008	USP9X	Probable	14	30	36	30	2570	292.1
P36871	PGM1	Phosphogl	42	24	47	24	562	61.4
P30622	CLIP1	CAP-Gly d	24	33	37	27	1438	162.1
P15170	GSPT1	Eukaryoti	45	22	38	22	499	55.7
O43175	PHGDH	D-3-phosp	41	16	40	16	533	56.6
Q15084	PDIA6	Protein d	43	17	57	17	440	48.1
Q9Y262	EIF3L	Eukaryoti	39	20	35	20	564	66.7
P05455	SSB	Lupus La	46	25	41	25	408	46.8
Q16222	UAP1	UDP-N-ace	35	19	43	18	522	58.7
Q13263	TRIM28	Transcrip	35	24	41	24	835	88.5
P23284	PPIB	Peptidyl-	51	14	93	13	216	23.7
P13489	RNH1	Ribonucle	48	19	52	19	461	49.9
P04792	HSPB1	Heat shoc	66	13	78	13	205	22.8
P55060	CSE1L	Exportin-	26	24	42	24	971	110.3
P61981	YWHAG	14-3-3 pr	64	16	79	11	247	28.3
P12081	HARS	Histidine	50	25	44	19	509	57.4
O95373	IPO7	Importin-	23	20	38	19	1038	119.4
A0A0A0MTS	GPI	Glucose-6	36	19	50	19	573	64.8
P09211	GSTP1	Glutathio	55	10	75	10	210	23.3
P23246	SFPQ	Splicing	31	22	49	21	707	76.1
P61978	HNRNPK	Heterogen	47	19	56	3	463	50.9
P55084	HADHB	Trifuncti	33	18	39	18	474	51.3
Q8WX93	PALLD	Palladin	19	22	46	22	1383	150.5
Q9H223	EHD4	EH domain	49	22	41	15	541	61.1
Q9NR30	DDX21	Nucleolar	32	22	33	20	783	87.3
P30041	PRDX6	Peroxired	48	14	63	14	224	25
H0YNC7	TPM1	Tropomyos	65	20	93	1	223	25.6
Q16891	IMMT	MICOS com	38	27	41	27	758	83.6
O00231	PSMD11	26S prote	57	22	45	22	422	47.4
P22626	HNRNPA2B1	Heterogen	50	17	61	15	353	37.4
P20908	COL5A1	Collagen	17	26	52	20	1838	183.4
P07954	FH	Fumarate	41	17	42	17	510	54.6
Q13423	NNT	NAD(P) tr	26	24	35	24	1086	113.8

Q12797	ASPH	Aspartyl/	30	22	44	22	758	85.8
Q15029	EFTUD2	116 kDa U	29	24	39	23	972	109.4
P28331	NDUFS1	NADH-ubiq	41	21	31	21	727	79.4
P47897	QARS	Glutamine	37	24	38	23	775	87.7
P52907	CAPZA1	F-actin-c	65	12	36	10	286	32.9
M0R0P8	MYO9B	Unconvent	16	35	39	35	2157	243.2
P50995	ANXA11	Annexin A	37	18	43	18	505	54.4
A8MXP9	MATR3	Matrin-3	25	24	48	24	895	99.9
A0A0A0MTC	GSN	Gelsolin	35	21	47	16	767	84.7
Q13308	PTK7	Inactive	28	25	40	25	1070	118.3
P51659	HSD17B4	Peroxisom	40	24	34	24	736	79.6
O14980	XPO1	Exportin-	22	21	39	21	1071	123.3
P00367	GLUD1	Glutamate	34	18	38	18	558	61.4
B4DDF4	CNN2	Calponin	54	12	60	10	298	32.6
P08243	ASNS	Asparagin	42	20	41	20	561	64.3
O43776	NARS	Asparagin	32	18	44	18	548	62.9
Q9UHD8	SEPT9	Septin-9	47	23	41	23	586	65.4
Q32Q12	NME1-NME2	Nucleosid	74	17	60	2	292	32.6
P29144	TPP2	Tripeptid	22	27	36	27	1249	138.3
P07737	PFN1	Profilin-	72	11	137	11	140	15
P62937	PPIA	Peptidyl-	68	13	119	12	165	18
F8W6I7	HNRNPA1	Heterogen	50	15	67	13	307	33.1
Q14980	NUMA1	Nuclear m	15	30	34	29	2115	238.1
P37837	TALDO1	Transaldo	44	19	52	19	337	37.5
O14617	AP3D1	AP-3 comp	22	23	30	23	1153	130.1
Q92538	GBF1	Golgi-spe	16	25	35	25	1859	206.3
O94808	GFPT2	Glutamine	38	25	47	22	682	76.9
K7EKE6	LONP1	Lon prote	28	21	29	21	845	95.1
G8JLD5	DNM1L	Dynamin-1	36	22	30	22	712	79.6
P54578	USP14	Ubiquitin	33	18	29	18	494	56
F8W914	RTN4	Reticulon	54	9	35	9	345	37.1
P14868	DARS	Aspartate	53	25	39	25	501	57.1
O94855	SEC24D	Protein t	29	24	37	23	1032	112.9
O00299	CLIC1	Chloride	74	14	48	13	241	26.9
P23526	AHCY	Adenosylh	46	19	44	19	432	47.7
Q9UQ80	PA2G4	Prolifera	44	15	44	15	394	43.8
P12109	COL6A1	Collagen	23	21	39	21	1028	108.5
Q9NZN4	EHD2	EH domain	51	23	40	18	543	61.1
Q9NTK5	OLA1	Obg-like	46	17	38	17	396	44.7
P21589	NT5E	5'-nucleo	34	17	45	17	574	63.3
P24844	MYL9	Myosin re	66	10	63	3	172	19.8
Q5JPE7	NOMO2	Nodal mod	20	20	35	20	1267	139.4
O14974	PPP1R12A	Protein p	24	26	37	26	1030	115.2
B5ME19	EIF3CL	Eukaryoti	26	26	45	26	914	105.4
Q5T6W2	HNRNPK	Heterogen	50	17	55	1	379	41.8
P42224	STAT1	Signal tr	27	19	35	19	750	87.3
Q13045	FLII	Protein f	23	26	34	15	1269	144.7
Q6DD88	ATL3	Atlastin-	41	16	40	16	541	60.5
P14866	HNRNPL	Heterogen	44	19	37	18	589	64.1
Q92945	KHSRP	Far upstr	43	25	38	23	711	73.1
Q9Y3F4	STRAP	Serine-th	53	16	41	16	350	38.4

Q6WCQ1	MPRIP	Myosin ph	26	23	32	22	1025	116.5
H0Y8G5	HNRNPD	Heterogen	48	17	46	10	260	29.6
Q9Y230	RUVBL2	RuvB-like	41	18	32	18	463	51.1
Q9Y265	RUVBL1	RuvB-like	46	17	28	17	456	50.2
P09486	SPARC	SPARC OS=	46	14	48	14	303	34.6
E9PCY7	HNRNPH1	Heterogen	41	15	45	4	429	47.1
A0A2R8Y4I	TNS1	Tensin-1	17	26	35	26	1860	199.4
Q13435	SF3B2	Splicing	30	27	41	27	895	100.2
P08240	SRPRA	Signal re	32	20	33	20	638	69.8
P35555	FBN1	Fibrillin	11	28	33	24	2871	312
Q16181	SEPT7	Septin-7	44	19	45	18	437	50.6
A0A1B0GTG	ALDH7A1	Alpha-ami	41	17	29	17	536	58.1
Q14240	EIF4A2	Eukaryoti	40	16	54	5	407	46.4
P21796	VDAC1	Voltage-d	54	13	44	12	283	30.8
E9PND2	CSRP1	Cysteine	63	9	49	1	153	16.1
P00352	ALDH1A1	Retinal d	33	17	41	16	501	54.8
Q96D15	RCN3	Reticuloc	48	12	38	12	328	37.5
P09936	UCHL1	Ubiquitin	53	10	81	10	223	24.8
P30837	ALDH1B1	Aldehyde	34	16	32	14	517	57.2
A0AVT1	UBA6	Ubiquitin	24	23	32	23	1052	117.9
P07996	THBS1	Thrombosp	24	27	34	27	1170	129.3
Q02790	FKBP4	Peptidyl-	46	20	34	20	459	51.8
Q96TA1	FAM129B	Niban-lik	25	21	48	21	746	84.1
P60981	DSTN	Destrin C	66	15	50	14	165	18.5
O43491	EPB41L2	Band 4.1-	24	21	34	21	1005	112.5
P17812	CTPS1	CTP synth	31	18	38	15	591	66.6
Q96AG4	LRRC59	Leucine-r	47	15	41	15	307	34.9
Q15181	PPA1	Inorganic	67	16	40	15	289	32.6
C9JZR2	CTNND1	Catenin d	31	23	40	23	938	104.8
P58107	EPPK1	Epiplakin	23	26	34	20	5088	555.3
P23381	WARS	Tryptopha	30	15	35	15	471	53.1
Q15008	PSMD6	26S prote	45	21	37	21	389	45.5
P45974	USP5	Ubiquitin	30	19	36	19	858	95.7
Q9UGI8	TES	Testin OS	49	18	29	18	421	48
D6RER5	SEPT11	Septin-11	44	15	32	8	432	49.8
O43242	PSMD3	26S prote	39	20	32	20	534	60.9
P63241	EIF5A	Eukaryoti	71	11	49	11	154	16.8
A0A2R8YCH	CTNNB1	Catenin b	31	20	34	17	779	85.2
P15880	RPS2	40S ribos	43	13	70	13	293	31.3
E9PBS1	PAICS	Multifunc	44	19	37	19	413	45.6
A2A274	ACO2	Aconitate	27	19	37	19	805	87.8
P31946	YWHAB	14-3-3 pr	52	12	65	5	246	28.1
Q15293	RCN1	Reticuloc	56	15	35	15	331	38.9
Q13561	DCTN2	Dynactin	49	15	34	15	401	44.2
P50552	VASP	Vasodilat	47	17	31	17	380	39.8
P04181	OAT	Ornithine	34	14	29	14	439	48.5
P61247	RPS3A	40S ribos	61	20	63	20	264	29.9
O00203	AP3B1	AP-3 comp	23	25	36	24	1094	121.2
Q16543	CDC37	Hsp90 co-	35	12	39	12	378	44.4
A0A087WVM	MTHFD1L	Monofunct	24	20	30	19	913	99.2
Q32MZ4	LRRFIP1	Leucine-r	22	16	25	15	808	89.2

P61221	ABCE1	ATP-bindi	33	18	33	15	599	67.3
Q9Y570	PPME1	Protein p	50	17	31	17	386	42.3
Q99497	PARK7	Protein/n	59	12	50	12	189	19.9
Q9Y520	PRRC2C	Protein P	10	27	31	26	2896	316.7
P05997	COL5A2	Collagen	18	20	32	20	1499	144.8
P20042	EIF2S2	Eukaryoti	50	15	31	15	333	38.4
P49257	LMAN1	Protein E	33	17	52	17	510	57.5
Q9NZI8	IGF2BP1	Insulin-l	39	22	34	20	577	63.4
P09382	LGALS1	Galectin-	78	9	175	9	135	14.7
Q15046	KARS	Lysine--t	33	20	45	20	597	68
Q9Y617	PSAT1	Phosphose	51	19	33	18	370	40.4
P52306	RAP1GDS1	Rap1 GTPa	33	17	28	17	607	66.3
Q92973	TNPO1	Transport	22	17	33	13	898	102.3
I3L1L3	MYBBP1A	Myb-bindi	21	24	31	24	1252	140.2
P50570	DNM2	Dynamin-2	26	22	31	15	870	98
Q15393	SF3B3	Splicing	19	21	39	21	1217	135.5
Q9UJU6	DBNL	Drebrin-l	42	14	30	14	430	48.2
O14950	MYL12B	Myosin re	55	10	67	3	172	19.8
Q15366	PCBP2	Poly(rC)-	41	12	40	5	365	38.6
P05388	RPLP0	60S acidi	48	13	51	13	317	34.3
P20020	ATP2B1	Plasma me	19	18	23	10	1258	138.7
A0A2Q2TH7	GOLGA2	Golgin su	21	18	23	13	990	111.6
P00505	GOT2	Aspartate	38	18	31	18	430	47.5
P18124	RPL7	60S ribos	53	16	48	16	248	29.2
Q02218	OGDH	2-oxoglut	23	22	28	17	1023	115.9
H3BQZ7	HNRNPUL2-	HCG20447S	27	20	32	20	746	84.6
Q96KP4	CNDP2	Cytosolic	41	16	35	16	475	52.8
P30520	ADSS	Adenylosu	36	14	25	14	456	50.1
H0Y5F5	PABPC4	Polyadeny	28	18	38	1	550	60.2
Q8N163	CCAR2	Cell cycl	23	17	29	17	923	102.8
P51991	HNRNPA3	Heterogen	25	11	35	10	378	39.6
G3V180	DPP3	Dipeptidy	32	18	30	18	757	84.3
B1AK88	CAPZB	Capping p	41	16	56	15	301	33.8
P30086	PEBP1	Phosphati	80	11	47	11	187	21
P29966	MARCKS	Myristoyl	34	7	45	7	332	31.5
A0A087X2I	PSMC6	26S prote	50	16	28	16	403	45.8
P51149	RAB7A	Ras-relat	72	13	37	13	207	23.5
P62701	RPS4X	40S ribos	55	16	42	11	263	29.6
O95340	PAPSS2	Bifunctic	31	17	43	16	614	69.5
Q8WVM8	SCFD1	Sec1 fami	30	17	24	17	642	72.3
Q5JXI8	FHL1	Four and	60	12	36	4	257	29.1
Q562R1	ACTBL2	Beta-acti	34	13	209	7	376	42
P48147	PREP	Prolyl en	37	22	30	22	710	80.6
O76094	SRP72	Signal re	35	20	28	20	671	74.6
A0A0J9YVF	PUF60	Poly(U)-b	36	16	34	16	534	57.4
P13804	ETFA	Electron	43	11	31	11	333	35.1
P62191	PSMC1	26S prote	50	21	46	18	440	49.2
Q13085	ACACA	Acetyl-Cc	12	25	28	25	2346	265.4
A0A2U3TZN	CHD4	Chromodom	15	24	26	24	1902	216.7
P54920	NAPA	Alpha-sol	62	15	26	15	295	33.2
P08865	RPSA	40S ribos	41	12	39	11	295	32.8

Q9BXJ9	NAA15	N-alpha-a	29	24	34	19	866	101.2
Q15417	CNN3	Calponin-	45	12	37	10	329	36.4
P00387	CYB5R3	NADH-cytc	49	12	37	12	301	34.2
P53992	SEC24C	Protein t	26	23	29	21	1094	118.2
P10316	HLA-A	HLA class	47	13	28	5	365	41
P61011	SRP54	Signal re	41	19	32	19	504	55.7
P78417	GSTO1	Glutathic	44	16	51	16	241	27.5
Q06323	PSME1	Proteasom	58	15	36	15	249	28.7
P80303	NUCB2	Nucleobir	42	16	29	14	420	50.2
Q9NZB2	FAM120A	Constitut	22	19	28	19	1118	121.8
Q5T7C4	HMGB1	High mobi	54	11	51	8	158	18.3
O75718	CRTAP	Cartilage	39	16	31	16	401	46.5
P05141	SLC25A5	ADP/ATP t	44	15	48	5	298	32.8
Q9NSD9	FARSB	Phenylala	34	21	34	21	589	66.1
Q7Z406	MYH14	Myosin-14	5	12	60	1	1995	227.7
P48735	IDH2	Isocitrat	41	19	31	19	452	50.9
Q13740	ALCAM	CD166 ant	29	16	28	16	583	65.1
O00232	PSMD12	26S prote	36	16	30	16	456	52.9
P08237	PFKM	ATP-deper	30	20	37	17	780	85.1
P55795	HNRNPH2	Heterogen	39	15	38	5	449	49.2
P25786	PSMA1	Proteasom	51	14	34	14	263	29.5
Q08J23	NSUN2	tRNA (cyt	37	22	29	22	767	86.4
Q96QK1	VPS35	Vacuolar	27	18	26	16	796	91.6
Q9NR12	PDLIM7	PDZ and L	40	16	54	12	457	49.8
P40925	MDH1	Malate de	40	15	46	15	334	36.4
Q13492	PICALM	Phosphati	28	16	27	13	652	70.7
P49189	ALDH9A1	4-trimeth	30	14	25	14	494	53.8
P32119	PRDX2	Peroxirec	58	11	42	10	198	21.9
Q9Y2A7	NCKAP1	Nck-assoc	20	23	31	23	1128	128.7
H3BVG0	NUP93	Nuclear p	27	23	30	23	880	99.5
P12110	COL6A2	Collagen	22	19	30	19	1019	108.5
Q9NYL9	TMOD3	Tropomodu	51	17	31	16	352	39.6
P55010	EIF5	Eukaryoti	36	17	30	17	431	49.2
P41091	EIF2S3	Eukaryoti	34	13	33	13	472	51.1
Q9UBG0	MRC2	C-type ma	15	17	29	17	1479	166.6
P21399	ACO1	Cytoplasm	28	23	33	23	889	98.3
Q9Y266	NUDC	Nuclear m	47	15	30	15	331	38.2
Q9H4A4	RNPEP	Aminopept	28	16	27	16	650	72.5
O95573	ACSL3	Long-chai	29	16	23	15	720	80.4
Q9Y263	PLAA	Phospholi	28	20	25	20	795	87.1
P15924	DSP	Desmoplak	9	29	29	29	2871	331.6
O15371	EIF3D	Eukaryoti	32	12	24	12	548	63.9
Q8NE71	ABCF1	ATP-bindi	18	15	22	10	845	95.9
P62906	RPL10A	60S ribos	42	11	40	11	217	24.8
Q16270	IGFBP7	Insulin-l	41	11	36	11	282	29.1
P61160	ACTR2	Actin-rel	33	11	48	11	394	44.7
P62753	RPS6	40S ribos	39	13	38	13	249	28.7
P78347	GTF2I	General t	19	21	26	21	998	112.3
P10412	HIST1H1E	Histone H	42	14	57	4	219	21.9
Q02818	NUCB1	Nucleobir	42	19	32	17	461	53.8
P04844	RPN2	Dolichyl-	33	14	31	14	631	69.2

Q14847	LASP1	LIM and S	52	16	47	16	261	29.7
P16403	HIST1H1C	Histone H	40	13	54	4	213	21.4
P04899	GNAI2	Guanine n	46	13	28	6	355	40.4
P05091	ALDH2	Aldehyde	41	14	22	13	517	56.3
P40227	CCT6A	T-complex	33	17	40	17	531	58
O15144	ARPC2	Actin-rel	48	17	43	17	300	34.3
P14923	JUP	Junction	29	17	22	14	745	81.7
Q96A49	SYAP1	Synapse-a	47	15	21	15	352	39.9
P17858	PFKL	ATP-depen	24	17	40	12	780	85
P39019	RPS19	40S ribos	62	13	58	13	145	16.1
Q53EP0	FNDC3B	Fibronect	20	17	25	17	1204	132.8
P28074	PSMB5	Proteasom	54	13	23	13	263	28.5
P24534	EEF1B2	Elongatic	56	11	44	8	225	24.7
O15031	PLXNB2	Plexin-B2	14	25	28	25	1838	205
F8W9J4	DST	Dystonin	4	27	28	23	7461	847.4
P55265	ADAR	Double-st	19	22	26	22	1226	136
A0A0C4DG8	DDX46	Probable	23	25	30	25	1032	117.4
P98160	HSPG2	Basement	6	22	25	22	4391	468.5
P13647	KRT5	Keratin,	25	18	49	7	590	62.3
P11766	ADH5	Alcohol d	33	13	41	13	374	39.7
P09972	ALDOC	Fructose-	35	13	49	8	364	39.4
A0A1W2PNX	UNC45A	Protein u	20	21	25	20	1084	118.3
A0A0C4DGA	ECI2	Enoyl-CoA	39	11	21	11	364	40.2
Q07960	ARHGAP1	Rho GTPas	38	15	36	15	439	50.4
Q96CW1	AP2M1	AP-2 comp	47	19	34	19	435	49.6
Q8IWE2	FAM114A1	Protein N	30	15	25	15	563	60.7
J3KTL2	SRSF1	Serine/ar	52	15	47	15	253	28.3
O95817	BAG3	BAG famil	42	17	22	17	575	61.6
O75821	EIF3G	Eukaryoti	41	13	26	2	320	35.6
H0Y6I0	GOLGA4	Golgin su	11	25	27	24	2099	246.5
P50502	ST13	Hsc70-int	29	12	29	12	369	41.3
E7EQL5	DYNC1I2	Cytoplasm	47	11	22	1	305	34.9
P49915	GMPS	GMP synth	32	20	28	20	693	76.7
Q9BT78	COPS4	COP9 sign	55	17	26	17	406	46.2
Q13620	CUL4B	Cullin-4B	23	23	33	13	913	103.9
Q32P28	P3H1	Prolyl 3-	26	16	33	15	736	83.3
Q13976	PRKG1	cGMP-depe	31	21	28	21	671	76.3
Q96I24	FUBP3	Far upstr	41	19	27	17	572	61.6
Q10471	GALNT2	Polypepti	43	24	33	22	571	64.7
Q8TAQ2	SMARCC2	SWI/SNF c	16	18	22	12	1214	132.8
Q04917	YWHAH	14-3-3 pr	52	13	55	9	246	28.2
P60900	PSMA6	Proteasom	48	12	31	12	246	27.4
Q9Y2X3	NOP58	Nucleolar	34	15	21	15	529	59.5
Q13162	PRDX4	Peroxired	49	11	40	9	271	30.5
Q8NBJ5	COLGALT1	Procollag	30	18	27	18	622	71.6
P49321	NASP	Nuclear a	26	17	21	17	788	85.2
A0A0A0MTH	ILK	Integrin-	36	17	36	17	483	54.6
H3BNC9		Uncharact	13	7	23	7	584	64.5
O75533	SF3B1	Splicing	20	21	23	21	1304	145.7
Q70UQ0	IKBIP	Inhibitor	43	17	38	17	350	39.3
P43034	PAFAH1B1	Platelet-	39	16	26	15	410	46.6

Q9HD20	ATP13A1	Manganese	18	18	21	18	1204	132.9
Q12965	MYO1E	Unconvent	16	17	22	15	1108	127
Q9H0U4	RAB1B	Ras-relat	69	13	40	5	201	22.2
P10155	TROVE2	60 kDa SS	33	17	25	17	538	60.6
Q13177	PAK2	Serine/th	29	12	20	10	524	58
P27695	APEX1	DNA-(apur	52	16	25	16	318	35.5
P15531	NME1	Nucleosic	70	11	37	1	152	17.1
P12236	SLC25A6	ADP/ATP t	41	14	46	2	298	32.8
P30533	LRPAP1	Alpha-2-m	47	20	33	20	357	41.4
P62805	HIST1H4A	Histone H	58	7	99	7	103	11.4
Q9Y6M1	IGF2BP2	Insulin-l	31	16	26	15	599	66.1
Q27J81	INF2	Inverted	15	17	21	17	1249	135.5
Q9UDY4	DNAJB4	DnaJ homo	39	12	24	11	337	37.8
P62979	RPS27A	Ubiquitin	67	12	127	4	156	18
P48059	LIMS1	LIM and s	50	14	32	8	325	37.2
Q99536	VAT1	Synaptic	32	10	34	10	393	41.9
P60228	EIF3E	Eukaryoti	39	15	27	15	445	52.2
Q15459	SF3A1	Splicing	24	16	29	16	793	88.8
H0Y7A7	CALM2	Calmoduli	39	7	86	7	187	20.7
O75116	ROCK2	Rho-assoc	16	22	25	20	1388	160.8
A0A0U1RRM	ENAH	Protein e	18	16	30	16	802	87.3
X6RLX0	ERC1	ELKS/Rab6	17	19	23	19	1120	128.4
P62820	RAB1A	Ras-relat	75	14	41	6	205	22.7
P62241	RPS8	40S ribos	52	11	37	11	208	24.2
Q9Y281	CFL2	Cofilin-2	66	14	40	9	166	18.7
Q9Y5M8	SRPRB	Signal re	48	11	20	11	271	29.7
Q92696	RABGGTA	Geranylge	34	18	24	18	567	65
E9PFF5	FXR1	Fragile X	35	14	20	12	490	55.1
Q8IVL6	P3H3	Prolyl 3-	30	18	29	18	736	81.8
Q9Y6N5	SQOR	Sulfide:q	46	18	25	18	450	49.9
Q9HB71	CACYBP	Calcyclin	57	12	21	12	228	26.2
H0Y449	YBX1	Nuclease-	32	8	36	6	374	42
P62873	GNB1	Guanine n	36	11	30	5	340	37.4
P46063	RECQL	ATP-depen	31	19	30	19	649	73.4
O95394	PGM3	Phosphoac	33	15	28	15	542	59.8
P05387	RPLP2	60S acidi	54	6	29	6	115	11.7
A0A0R4J2G	NCEH1	Arylaceta	40	16	29	16	440	49
Q8WUP2	FBLIM1	Filamin-b	44	13	26	13	373	40.6
P28066	PSMA5	Proteasom	44	8	27	8	241	26.4
P43686	PSMC4	26S prote	34	14	28	14	418	47.3
E7EQT4	ACIN1	Apoptotic	15	18	23	18	1301	147.3
Q92888	ARHGEF1	Rho guani	25	19	24	19	912	102.4
P16402	HIST1H1D	Histone H	42	13	52	5	221	22.3
Q9UNH7	SNX6	Sorting n	39	17	21	16	406	46.6
Q15942	ZYX	Zyxin OS=	28	13	38	13	572	61.2
O95747	OXSR1	Serine/th	35	16	24	12	527	58
P12235	SLC25A4	ADP/ATP t	45	14	37	4	298	33
P09960	LTA4H	Leukotrie	26	14	20	14	611	69.2
P63000	RAC1	Ras-relat	41	9	36	3	192	21.4
P25788	PSMA3	Proteasom	44	14	35	14	255	28.4
P51148	RAB5C	Ras-relat	54	8	29	6	216	23.5

Q02878	RPL6	60S ribos	36	15	54	15	288	32.7
Q7L1Q6	BZW1	Basic leu	32	17	43	17	419	48
P54727	RAD23B	UV excisi	34	14	31	12	409	43.1
Q92626	PXDN	Peroxidas	14	20	26	20	1479	165.2
K7EL20	EIF3G	Eukaryoti	44	12	24	1	262	29.3
P45880	VDAC2	Voltage-d	44	12	39	12	294	31.5
P25398	RPS12	40S ribos	55	8	32	8	132	14.5
Q9BS26	ERP44	Endoplasm	37	15	27	14	406	46.9
P20073	ANXA7	Annexin A	32	14	25	14	488	52.7
O94973	AP2A2	AP-2 comp	22	20	25	11	939	103.9
P30050	RPL12	60S ribos	69	9	44	9	165	17.8
P09525	ANXA4	Annexin A	48	14	22	14	319	35.9
P05121	SERPINE1	Plasminog	37	12	25	12	402	45
E7EX17	EIF4B	Eukaryoti	28	18	25	18	616	69.7
Q16698	DECR1	2,4-dienc	42	14	22	14	335	36
P24752	ACAT1	Acetyl-Cc	35	14	21	14	427	45.2
Q9NZU5	LMCD1	LIM and c	47	16	27	16	365	40.8
Q15042	RAB3GAP1	Rab3 GTPa	20	16	22	16	981	110.5
P02538	KRT6A	Keratin,	21	15	57	1	564	60
P22307	SCP2	Non-speci	24	14	26	14	547	59
Q96G03	PGM2	Phosphogl	29	17	23	16	612	68.2
Q9BXP5	SRRT	Serrate R	23	18	22	18	876	100.6
Q8N3C0	ASCC3	Activatir	11	21	22	21	2202	251.3
Q13464	ROCK1	Rho-assoc	15	21	24	19	1354	158.1
Q5T5C7	SARS	Serine--t	34	15	28	15	536	61.3
A0A2R8Y81	RPS14	40S ribos	41	7	23	7	150	16.1
P52789	HK2	Hexokinas	22	19	20	16	917	102.3
Q96C19	EFHD2	EF-hand d	47	12	20	12	240	26.7
P19623	SRM	Spermidir	53	10	22	10	302	33.8
Q9Y4E8	USP15	Ubiquitir	22	20	26	19	981	112.3
Q9UNF0	PACSIN2	Protein k	36	14	25	14	486	55.7
P62714	PPP2CB	Serine/th	39	10	23	2	309	35.6
P48739	PITPNB	Phosphati	65	17	21	14	271	31.5
P52209	PGD	6-phosphc	33	15	40	15	483	53.1
P34897	SHMT2	Serine hy	30	14	30	14	504	56
Q9UNM6	PSMD13	26S prote	33	13	30	13	376	42.9
A0A087X0K	TJP1	Tight jur	11	17	23	17	1676	187.7
Q9Y450	HBS1L	HBS1-like	28	16	25	16	684	75.4
P11177	PDHB	Pyruvate	31	9	24	9	359	39.2
O75131	CPNE3	Copine-3	30	15	25	14	537	60.1
P62136	PPP1CA	Serine/th	44	13	32	3	330	37.5
Q8TDX7	NEK7	Serine/th	43	14	30	14	302	34.5
P21964	COMT	Catechol	53	14	22	14	271	30
P30044	PRDX5	Peroxirec	55	8	32	8	214	22.1
Q15691	MAPRE1	Microtubu	42	11	32	9	268	30
Q9Y2Z0	SUGT1	Protein S	42	13	21	13	365	41
I3L0H8	DDX19A	ATP-deper	31	15	29	15	447	50.5
Q9Y6Y8	SEC23IP	SEC23-int	19	18	23	18	1000	111
B7Z7P8	ETF1	Eukaryoti	28	11	30	11	423	47.4
Q15424	SAFB	Scaffold	17	14	22	9	915	102.6
H9KV28	DIAPH1	Protein d	14	17	21	17	1228	136.8

O43143	DHX15	Pre-mRNA-	22	18	32	17	795	90.9
P62140	PPP1CB	Serine/th	40	12	30	3	327	37.2
P51572	BCAP31	B-cell re	37	13	28	13	246	28
Q14BN4	SLMAP	Sarcolemm	18	16	21	16	828	95.1
P83731	RPL24	60S ribos	52	13	41	13	157	17.8
P18085	ARF4	ADP-ribos	53	8	33	6	180	20.5
Q9UBS4	DNAJB11	DnaJ homo	39	14	26	14	358	40.5
A0A0U1RQK	EIF4G3	Eukaryoti	10	16	21	12	1774	195.2
P35232	PHB	Prohibiti	47	13	30	13	272	29.8
Q13409	DYNC1I2	Cytoplasm	20	11	21	1	638	71.4
Q9H3P7	ACBD3	Golgi res	31	12	16	12	528	60.6
O43399	TPD52L2	Tumor pro	54	12	34	12	206	22.2
P08779	KRT16	Keratin,	39	18	27	6	473	51.2
P52565	ARHGDIA	Rho GDP-d	37	9	36	9	204	23.2
Q01469	FABP5	Fatty aci	66	8	25	8	135	15.2
P27487	DPP4	Dipeptidy	21	17	26	17	766	88.2
H0Y2W2	ATAD3A	ATPase fa	32	19	25	7	572	64.3
O75165	DNAJC13	DnaJ homo	11	22	23	22	2243	254.3
Q12931	TRAP1	Heat shoc	27	18	26	17	704	80.1
B4DY09	ILF2	cDNA FLJ5	35	11	24	11	352	38.9
O60488	ACSL4	Long-chai	23	15	21	14	711	79.1
P13861	PRKAR2A	cAMP-depe	42	14	20	14	404	45.5
Q5QNW6	HIST2H2BF	Histone H	59	10	94	4	126	13.9
P62750	RPL23A	60S ribos	42	11	43	11	156	17.7
Q9H2M9	RAB3GAP2	Rab3 GTPa	15	20	24	20	1393	155.9
D6RBZ0	HNRNPAB	Heterogen	37	13	30	11	327	35.7
Q9H0D6	XRN2	5'-3' exo	20	17	21	17	950	108.5
P02533	KRT14	Keratin,	33	18	26	4	472	51.5
Q6PGP7	TTC37	Tetratric	14	19	22	19	1564	175.4
P55735	SEC13	Protein S	38	9	30	9	322	35.5
O00425	IGF2BP3	Insulin-l	28	15	25	13	579	63.7
P55145	MANF	Mesenceph	38	11	27	11	182	20.7
Q96AE4	FUBP1	Far upstr	27	17	29	14	644	67.5
P38606	ATP6V1A	V-type pr	29	15	23	15	617	68.3
P16401	HIST1H1B	Histone H	35	12	45	8	226	22.6
P27635	RPL10	60S ribos	39	7	37	1	214	24.6
Q14444	CAPRIN1	Caprin-1	15	12	25	12	709	78.3
P22059	OSBP	Oxysterol	24	17	21	17	807	89.4
O14744	PRMT5	Protein a	32	17	21	17	637	72.6
O14818	PSMA7	Proteasom	50	12	24	12	248	27.9
O95302	FKBP9	Peptidyl-	29	16	32	15	570	63
A0A1B0GVD	CTSD	Cathepsin	33	11	32	11	409	44.2
Q00325	SLC25A3	Phosphate	30	12	42	12	362	40.1
P38919	EIF4A3	Eukaryoti	39	14	31	11	411	46.8
P61586	RHOA	Transform	52	9	29	4	193	21.8
Q13724	MOGS	Mannosyl-	23	15	24	15	837	91.9
C9JIZ6	PSAP	Prosaposi	26	15	27	15	527	58.4
F6WLT2	DDX39B	Spliceoso	41	12	28	5	289	32.9
P08729	KRT7	Keratin,	32	16	47	12	469	51.4
Q9UNZ2	NSFL1C	NSFL1 cof	41	14	22	14	370	40.5
O76021	RSL1D1	Ribosomal	30	15	26	15	490	54.9

Q9Y5S2	CDC42BPB	Serine/th	12	21	22	17	1711	194.2
B7Z6Z4	MYL6	cDNA FLJ5	32	7	64	7	238	26.7
P30040	ERP29	Endoplasm	41	12	38	12	261	29
P22061	PCMT1	Protein-L	52	9	18	9	227	24.6
O60568	PLOD3	Procollag	22	16	30	16	738	84.7
Q14257	RCN2	Reticuloc	39	11	16	11	317	36.9
P67775	PPP2CA	Serine/th	39	10	23	2	309	35.6
P62879	GNB2	Guanine n	36	11	25	3	340	37.3
P17661	DES	Desmin OS	27	15	69	10	470	53.5
P36873	PPP1CC	Serine/th	42	12	28	2	323	37
P00492	HPRT1	Hypoxanth	65	10	17	10	218	24.6
Q12904	AIMP1	Aminoacyl	42	11	18	11	312	34.3
Q13596	SNX1	Sorting n	24	11	18	9	522	59
Q92878	RAD50	DNA repai	13	19	21	19	1312	153.8
P31930	UQCRC1	Cytochrom	35	12	21	11	480	52.6
Q9NQW7	XPNPEP1	Xaa-Pro a	23	13	24	13	623	69.9
Q9BZQ8	FAM129A	Protein N	20	16	22	16	928	103.1
Q9Y5B9	SUPT16H	FACT comp	18	17	20	17	1047	119.8
Q92734	TFG	Protein T	31	12	21	12	400	43.4
V9GYM8	ARHGEF2	Rho guani	18	18	22	18	1031	116
Q02880	TOP2B	DNA topoi	12	19	20	13	1626	183.2
O95861	BPNT1	3'(2'),5'	39	13	19	13	308	33.4
H0YDU8	PPP5C	Serine/th	31	13	19	13	485	55.2
P20340	RAB6A	Ras-relat	55	11	26	9	208	23.6
H3BRG4	UQCRC2	Cytochrom	36	12	22	12	412	44.6
Q93052	LPP	Lipoma-pr	26	11	22	11	612	65.7
A6NNK5	TP53BP1	TP53-bind	11	16	17	16	1927	208.9
Q13185	CBX3	Chromobox	55	9	19	8	183	20.8
Q8TCS8	PNPT1	Polyribon	24	18	21	18	783	85.9
Q9Y224	RTRAF	RNA trans	47	12	25	12	244	28.1
Q8N257	HIST3H2BE	Histone H	53	9	72	3	126	13.9
J3KR44	OTUB1	Ubiquitin	50	10	22	10	272	31.4
P55036	PSMD4	26S prote	29	10	20	10	377	40.7
O94826	TOMM70	Mitochond	30	19	28	19	608	67.4
O14964	HGS	Hepatocyt	23	17	27	17	777	86.1
Q9Y2W1	THRAP3	Thyroid h	16	14	22	13	955	108.6
O94905	ERLIN2	Erlin-2 C	40	12	21	9	339	37.8
Q9NZN3	EHD3	EH domain	33	16	30	7	535	60.8
P62917	RPL8	60S ribos	50	12	33	12	257	28
P10644	PRKAR1A	cAMP-depe	41	16	24	13	381	43
O43252	PAPSS1	Bifunctic	23	11	22	10	624	70.8
P00390	GSR	Glutathic	32	14	22	14	522	56.2
Q9H3S7	PTPN23	Tyrosine-	12	16	17	16	1636	178.9
Q7L576	CYFIP1	Cytoplasm	15	16	22	8	1253	145.1
P29317	EPHA2	Ephrin ty	17	15	18	14	976	108.2
P56537	EIF6	Eukaryoti	51	8	17	8	245	26.6
O00154	ACOT7	Cytosolic	32	11	22	11	380	41.8
P21266	GSTM3	Glutathic	51	11	22	9	225	26.5
O60313	OPA1	Dynamin-l	19	19	20	19	960	111.6
Q5JRA6	MIA3	Transport	10	19	22	19	1907	213.6
P46783	RPS10	40S ribos	58	12	42	11	165	18.9

P51911	CNN1	Calponin-	45	11	21	10	297	33.2
K7EIG1	CLUH	Clusterec	17	19	21	2	1251	140.5
P38159	RBMX	RNA-bindi	41	18	38	18	391	42.3
Q99623	PHB2	Prohibiti	56	17	28	17	299	33.3
P43487	RANBP1	Ran-speci	51	12	25	12	201	23.3
Q9NQR4	NIT2	Omega-ami	49	13	17	13	276	30.6
P61224	RAP1B	Ras-relat	57	9	23	4	184	20.8
P23368	ME2	NAD-deper	28	15	22	15	584	65.4
Q15746	MYLK	Myosin li	10	15	23	15	1914	210.6
P62829	RPL23	60S ribos	61	8	37	8	140	14.9
Q6IBS0	TWF2	Twinfilir	52	14	29	11	349	39.5
Q9UBB4	ATXN10	Ataxin-1C	33	15	24	15	475	53.5
P26368	U2AF2	Splicing	31	9	25	9	475	53.5
O14979	HNRNPDL	Heteroger	30	14	28	12	420	46.4
Q9UHG3	PCYOX1	Prenylcys	30	12	23	12	505	56.6
P38117	ETFB	Electron	53	14	19	14	255	27.8
P40222	TXLNA	Alpha-tax	32	15	19	15	546	61.9
O00567	NOP56	Nucleolar	23	12	19	12	594	66
P29692	EEF1D	Elongatic	39	11	41	8	281	31.1
A6NLN1	PTBP1	Polypyrim	23	10	24	10	527	56.5
P00491	PNP	Purine nu	44	10	19	10	289	32.1
Q5W0H4	TPT1	Translati	38	8	37	8	188	21.5
O75368	SH3BGRL	SH3 domai	90	9	22	9	114	12.8
Q6NZI2	CAVIN1	Caveolae-	28	13	39	13	390	43.5
Q16401	PSMD5	26S prote	37	15	24	15	504	56.2
O95336	PGLS	6-phosphc	55	10	23	10	258	27.5
Q8NCA5	FAM98A	Protein F	24	9	21	9	519	55.4
P51858	HDGF	Hepatoma-	50	10	21	9	240	26.8
Q96CV9	OPTN	Optineuri	29	17	20	17	577	65.9
Q13283	G3BP1	Ras GTPas	36	13	26	12	466	52.1
Q16822	PCK2	Phosphoer	26	15	21	15	640	70.7
P16152	CBR1	Carbonyl	42	9	25	7	277	30.4
Q9UI42	CPA4	Carboxype	28	11	27	11	421	47.3
Q7L2H7	EIF3M	Eukaryoti	29	9	19	9	374	42.5
O94874	UFL1	E3 UFM1-p	21	16	22	16	794	89.5
P46977	STT3A	Dolichyl-	20	15	30	13	705	80.5
P05198	EIF2S1	Eukaryoti	45	14	23	14	315	36.1
P35637	FUS	RNA-bindi	16	9	27	7	526	53.4
P62280	RPS11	40S ribos	46	9	28	9	158	18.4
O76003	GLRX3	Glutaredc	32	11	24	11	335	37.4
P36957	DLST	Dihydroli	25	11	23	11	453	48.7
P52948	NUP98	Nuclear p	11	19	21	19	1817	197.5
O60361	NME2P1	Putative	66	8	34	1	137	15.5
Q9UGP8	SEC63	Transloca	23	15	21	15	760	87.9
P12004	PCNA	Prolifera	44	10	21	10	261	28.8
Q9UQE7	SMC3	Structura	15	17	21	17	1217	141.5
G3V0I5	NDUFV1	NADH dehy	42	16	20	16	457	50
Q9BZZ5	API5	Apoptosis	31	13	21	13	524	59
P30084	ECHS1	Enoyl-CoA	36	11	23	11	290	31.4
Q86W92	PPFIBP1	Liprin-be	18	16	19	16	1011	114
P13746	HLA-A	HLA class	33	8	16	2	365	40.9

Q15477	SKIV2L	Helicase	17	17	19	17	1246	137.7
Q12929	EPS8	Epidermal	18	11	14	11	822	91.8
Q08257	CRYZ	Quinone c	46	12	23	12	329	35.2
P13798	APEH	Acylaminc	18	13	22	13	732	81.2
E9PFP8	PCBP3	Poly(rC)-	22	7	27	1	361	38.2
Q9Y4K0	LOXL2	Lysyl oxi	20	15	21	14	774	86.7
Q8N6T3	ARFGAP1	ADP-ribos	34	10	19	10	406	44.6
P30419	NMT1	Glycylpep	30	14	22	11	496	56.8
Q14554	PDIA5	Protein d	27	14	20	14	519	59.6
A0A087WYN	DHX29	ATP-depen	13	16	19	16	1370	155.2
P48047	ATP5O	ATP synth	48	9	27	9	213	23.3
O75367	H2AFY	Core hist	34	9	17	8	372	39.6
P05120	SERPINB2	Plasminog	32	13	19	13	415	46.6
Q99961	SH3GL1	Endophili	41	16	26	16	368	41.5
Q9NR45	NANS	Sialic ac	45	12	26	12	359	40.3
P25789	PSMA4	Proteasom	44	10	22	10	261	29.5
I3L2B0	CLUH	Clustered	15	18	20	1	1236	138.1
Q86UE4	MTDH	Protein L	25	11	16	11	582	63.8
Q96I99	SUCLG2	Succinate	26	11	19	11	432	46.5
P17813	ENG	Endoglin	21	12	20	12	658	70.5
P61019	RAB2A	Ras-relat	39	7	21	7	212	23.5
Q8N3V7	SYNPO	Synaptopc	20	12	16	12	929	99.4
P25205	MCM3	DNA repli	25	20	23	20	808	90.9
Q96P70	IPO9	Importin-	15	14	17	14	1041	115.9
Q9UH65	SWAP70	Switch-as	28	17	22	16	585	69
O43396	TXNL1	Thioredox	44	8	15	8	289	32.2
Q04446	GBE1	1,4-alpha	20	13	21	13	702	80.4
Q9H2D6	TRIOBP	TRIO and	7	16	20	15	2365	261.2
Q9BY44	EIF2A	Eukaryoti	30	13	17	13	585	64.9
Q8IXB1	DNAJC10	DnaJ homc	18	12	19	12	793	91
O43592	XPOT	Exportin-	17	14	20	14	962	109.9
P62277	RPS13	40S ribos	64	11	44	11	151	17.2
P52888	THOP1	Thimet ol	25	15	18	15	689	78.8
A0A0D9SEN	FAP	Prolyl en	18	14	24	14	759	87.5
P68036	UBE2L3	Ubiquitir	49	6	27	6	154	17.9
B1AXG1	RPS6KA3	Non-speci	27	15	17	14	711	80.7
Q16706	MAN2A1	Alpha-mar	15	16	19	16	1144	131.1
Q6P996	PDXDC1	Pyridoxal	19	14	23	14	788	86.7
Q9UDT6	CLIP2	CAP-Gly d	17	18	21	12	1046	115.8
P16949	STMN1	Stathmin	60	11	27	9	149	17.3
C9JNG9	COL6A3	Collagen	25	15	17	1	708	77.1
O95831	AIFM1	Apoptosis	24	13	19	13	613	66.9
Q96HE7	ERO1A	ERO1-like	20	8	15	8	468	54.4
P46087	NOP2	Probable	18	12	17	11	812	89.2
P00533	EGFR	Epidermal	17	17	18	17	1210	134.2
O75390	CS	Citrate s	25	12	35	11	466	51.7
Q92621	NUP205	Nuclear p	8	14	18	14	2012	227.8
O95202	LETM1	Mitochonc	24	14	21	14	739	83.3
O75822	EIF3J	Eukaryoti	39	9	14	9	258	29
Q9Y3A5	SBDS	Ribosome	36	12	20	12	250	28.7
O43294	TGFB1I1	Transforn	40	13	16	13	461	49.8

P05534	HLA-A	HLA class	36	9	16	1	365	40.7
P29279	CTGF	Connectiv	54	15	19	15	349	38.1
O60749	SNX2	Sorting n	30	16	18	14	519	58.4
P49736	MCM2	DNA repli	19	17	20	17	904	101.8
Q12765	SCRN1	Secernin-	28	11	22	11	414	46.4
E7EU96	CSNK2A1	Casein ki	32	10	15	10	385	45.3
P31153	MAT2A	S-adenosy	32	13	22	13	395	43.6
P22087	FBL	rRNA 2'-C	37	11	19	11	321	33.8
Q16666	IFI16	Gamma-int	22	14	19	13	785	88.2
Q99714	HSD17B10	3-hydroxy	39	7	18	7	261	26.9
H0Y9Y3	SYNPO2	Synaptopc	17	14	20	14	1155	125.3
B5MDF5	RAN	GTP-bindi	35	9	31	9	233	26.2
Q9UBT2	UBA2	SUMO-acti	22	14	20	14	640	71.2
P48637	GSS	Glutathic	34	16	21	16	474	52.4
O60684	KPNA6	Importin	27	13	18	9	536	60
P50281	MMP14	Matrix me	21	12	21	12	582	65.9
O75396	SEC22B	Vesicle-t	33	7	28	7	215	24.6
O94851	MICAL2	[F-actin]	13	13	18	12	1124	126.6
P52926	HMGA2	High mobi	49	7	24	7	109	11.8
P54687	BCAT1	Branched-	30	10	25	10	386	42.9
H7BXI1	ESYT2	Extended	16	11	17	11	884	97.9
Q9BTV4	TMEM43	Transmemb	36	13	25	13	400	44.8
P98194	ATP2C1	Calcium-t	18	13	15	13	919	100.5
P84098	RPL19	60S ribos	42	10	46	10	196	23.5
E9PEX6	DLD	Dihydroli	23	10	20	10	486	51.8
Q96N67	DOCK7	Dedicator	8	14	17	13	2140	242.4
Q9UBE0	SAE1	SUMO-acti	37	12	18	12	346	38.4
P40763	STAT3	Signal tr	20	13	17	13	770	88
Q13617	CUL2	Cullin-2	22	16	20	16	745	86.9
Q8WWM7	ATXN2L	Ataxin-2-	16	15	15	14	1075	113.3
Q8WXF1	PSPC1	Paraspeck	23	10	15	10	523	58.7
Q9UPQ0	LIMCH1	LIM and c	16	15	19	15	1083	121.8
Q13126	MTAP	S-methyl-	47	9	15	9	283	31.2
P16189	HLA-A	HLA class	32	8	14	1	365	41
P61088	UBE2N	Ubiquitir	58	8	26	8	152	17.1
P20618	PSMB1	Proteasom	39	7	21	7	241	26.5
P08559	PDHA1	Pyruvate	34	13	23	13	390	43.3
Q13347	EIF3I	Eukaryoti	34	10	22	10	325	36.5
Q93009	USP7	Ubiquitir	15	15	19	15	1102	128.2
P26373	RPL13	60S ribos	33	10	37	10	211	24.2
A5YKK6	CNOT1	CCR4-NOT	8	17	18	17	2376	266.8
P33992	MCM5	DNA repli	19	11	13	11	734	82.2
P42765	ACAA2	3-ketoacy	42	11	17	11	397	41.9
P26196	DDX6	Probable	30	11	17	11	483	54.4
Q14141	SEPT6	Septin-6	30	9	19	3	434	49.7
A0A087WT4	HMOX2	Heme oxyg	40	12	19	12	370	41.6
B8ZWD1	DBI	Acyl-CoA-	57	5	23	5	97	11.1
F8WAN1	SPECC1L-A	SPECC1L-A	16	15	18	14	911	101.5
P61313	RPL15	60S ribos	50	11	36	11	204	24.1
X6RFL8	RAB14	Ras-relat	63	11	18	10	181	20.4
Q9BYT8	NLN	Neurolysi	20	13	18	13	704	80.6

Q8N1G4	LRRC47	Leucine-r	26	13	17	13	583	63.4
Q96KG9	SCYL1	N-termina	22	15	21	15	808	89.6
P41240	CSK	Tyrosine-	25	11	17	11	450	50.7
O43615	TIMM44	Mitochond	31	12	16	12	452	51.3
P52597	HNRNPF	Heterogen	31	10	23	8	415	45.6
Q14019	COTL1	Coactosin	64	10	32	10	142	15.9
Q14566	MCM6	DNA repli	18	15	17	15	821	92.8
P21281	ATP6V1B2	V-type pr	24	12	19	12	511	56.5
P41227	NAA10	N-alpha-a	49	10	14	10	235	26.4
Q6VY07	PACS1	Phosphofu	16	14	16	14	963	104.8
P61204	ARF3	ADP-ribos	39	7	32	4	181	20.6
Q9H857	NT5DC2	5'-nuclec	34	16	19	16	520	60.7
Q9HDC9	APMAP	Adipocyte	33	12	21	12	416	46.5
Q00688	FKBP3	Peptidyl-	55	12	30	12	224	25.2
Q14690	PDCD11	Protein R	8	15	18	15	1871	208.6
P55268	LAMB2	Laminin s	9	14	17	14	1798	195.9
A0A0G2JLE	GBA	Glucosylc	23	11	17	11	536	59.6
P40123	CAP2	Adenylyl	25	10	22	8	477	52.8
G5E972	TMPO	Lamina-as	36	9	13	5	414	46.3
O60264	SMARCA5	SWI/SNF-r	17	19	21	16	1052	121.8
Q9NYF8	BCLAF1	Bcl-2-ass	18	16	19	15	920	106.1
O15143	ARPC1B	Actin-rel	34	11	24	11	372	40.9
F8VW96	CSRP2	Cysteine	34	8	23	8	243	26.7
Q15404	RSU1	Ras suppr	43	10	33	10	277	31.5
A0A140T91	HLA-C	HLA class	35	9	16	2	372	41.4
Q9NSE4	IARS2	Isoleucin	18	17	18	17	1012	113.7
Q13442	PDAP1	28 kDa he	46	10	19	10	181	20.6
Q5SSJ5	HP1BP3	Heterochr	23	13	21	13	553	61.2
P08648	ITGA5	Integrin	12	11	20	11	1049	114.5
P07686	HEXB	Beta-hexc	19	12	22	11	556	63.1
Q9Y6G9	DYNC1LI1	Cytoplasm	28	11	18	11	523	56.5
Q9H0B6	KLC2	Kinesin l	25	14	19	7	622	68.9
P61163	ACTR1A	Alpha-cen	26	11	27	5	376	42.6
Q9UJZ1	STOML2	Stomatin-	29	7	15	7	356	38.5
O94906	PRPF6	Pre-mRNA-	16	15	18	15	941	106.9
P16278	GLB1	Beta-gala	13	7	14	7	677	76
F8W726	UBAP2L	Ubiquitin	15	10	15	10	1079	113.6
Q96RP9	GFM1	Elongatic	21	14	19	14	751	83.4
Q9UMX0	UBQLN1	Ubiquilin	23	8	16	5	589	62.5
Q86VS8	HOOK3	Protein H	20	14	17	14	718	83.1
Q9Y680	FKBP7	Peptidyl-	43	12	17	12	222	25.8
F6TLX2	GLOD4	Glyoxalas	27	13	22	4	502	54.7
O60271	SPAG9	C-Jun-ami	11	12	16	12	1321	146.1
Q68EM7	ARHGAP17	Rho GTPas	15	12	15	12	881	95.4
A0A087WVF	SMTN	Smootheli	17	16	20	16	971	104.8
Q14683	SMC1A	Structura	11	15	17	15	1233	143.1
Q99615	DNAJC7	DnaJ homc	29	14	17	14	494	56.4
Q5JXI2	FHL1	Four and	43	9	29	1	210	23.7
P14735	IDE	Insulin-d	15	14	19	14	1019	117.9
O43747	AP1G1	AP-1 comp	17	14	20	14	822	91.3
O43237	DYNC1LI2	Cytoplasm	24	13	15	13	492	54.1

A0A087WZK	EIF3H	Eukaryoti	28	10	25	10	349	39.6
P84095	RHOG	Rho-relat	61	9	13	8	191	21.3
A0A0A0MRF	AKAP9	A-kinase	4	16	17	15	3910	453.2
H0YIV4	NAP1L1	Nucleosom	24	8	20	7	385	44.7
O00116	AGPS	Alkyldihy	24	10	14	10	658	72.9
Q99733	NAP1L4	Nucleosom	29	10	24	9	375	42.8
O94776	MTA2	Metastasi	23	16	18	12	668	75
Q7Z417	NUFIP2	Nuclear f	20	12	14	12	695	76.1
Q9NTJ5	SACM1L	Phosphati	23	14	21	14	587	66.9
Q96B97	SH3KBP1	SH3 domai	23	12	16	12	665	73.1
P62070	RRAS2	Ras-relat	51	10	18	8	204	23.4
Q9UKK3	PARP4	Poly [ADF	8	13	17	13	1724	192.5
A0A2R8Y84	RPS24	40S ribos	40	6	26	6	131	15.2
Q9Y6E2	BZW2	Basic leu	31	14	23	14	419	48.1
Q15785	TOMM34	Mitochond	45	12	19	11	309	34.5
Q9NUQ6	SPATS2L	SPATS2-li	24	12	16	5	558	61.7
Q6PKG0	LARP1	La-relate	14	15	17	15	1096	123.4
Q13098	GPS1	COP9 sigr	29	12	18	1	491	55.5
Q86V48	LUZP1	Leucine z	11	13	17	13	1076	120.2
Q9UQ35	SRRM2	Serine/ar	7	13	15	13	2752	299.4
O00487	PSMD14	26S prote	43	12	18	12	310	34.6
P98082	DAB2	Disabled	17	13	19	13	770	82.4
P09110	ACAA1	3-ketoacy	33	8	12	8	424	44.3
Q0ZGT2	NEXN	Nexilin C	20	14	20	14	675	80.6
K7ER00	FARSA	Phenylala	20	10	20	10	548	62.4
Q14151	SAFB2	Scaffold	12	11	16	6	953	107.4
Q5JWF2	GNAS	Guanine n	10	11	18	9	1037	111
A6NFX8	NUDT5	ADP-sugar	47	9	15	9	232	25.9
P47755	CAPZA2	F-actin-c	53	9	18	7	286	32.9
Q13616	CUL1	Cullin-1	19	15	17	15	776	89.6
Q8N766	EMC1	ER membra	14	13	19	13	993	111.7
Q12874	SF3A3	Splicing	27	12	18	12	501	58.8
Q6P2E9	EDC4	Enhancer	12	13	16	13	1401	151.6
O75947	ATP5H	ATP synth	76	11	22	11	161	18.5
H7BZJ3	PDIA3	Protein d	57	7	30	1	123	13.5
P14550	AKR1A1	Alcohol d	44	13	27	13	325	36.6
P62888	RPL30	60S ribos	57	7	22	7	115	12.8
M0R210	RPS16	40S ribos	60	10	36	10	129	14.4
Q13546	RIPK1	Receptor-	20	12	15	12	671	75.9
O75347	TBCA	Tubulin-s	59	9	21	9	108	12.8
O95479	H6PD	GDH/6PGL	19	12	16	12	791	88.8
P33316	DUT	Deoxyurid	42	8	14	8	252	26.5
J3KRX5	RPL17	60S ribos	47	9	32	8	174	20.2
P40429	RPL13A	60S ribos	43	13	50	13	203	23.6
P40261	NNMT	Nicotinam	31	8	30	8	264	29.6
O95352	ATG7	Ubiquitir	17	11	15	11	703	77.9
A0A096LPJ	GPS1	COP9 sigr	29	12	18	1	490	55.4
O00303	EIF3F	Eukaryoti	26	9	17	9	357	37.5
O95347	SMC2	Structura	11	14	15	14	1197	135.6
M0R3D6	RPL18A	60S ribos	44	7	26	7	141	16.7
P25325	MPST	3-mercapt	42	10	17	10	297	33.2

E7ETY2	TCOF1	Treacle p	11	16	16	16	1488	152.2
Q13619	CUL4A	Cullin-4A	19	15	19	5	759	87.6
O14579	COPE	Coatomer	37	9	16	9	308	34.5
P48449	LSS	Lanosterc	18	12	19	12	732	83.3
Q9UG63	ABCF2	ATP-bindi	18	12	20	12	623	71.2
Q14677	CLINT1	Clathrin	21	13	14	13	625	68.2
P17612	PRKACA	cAMP-depe	32	10	19	3	351	40.6
Q00169	PITPNA	Phosphati	47	13	22	10	270	31.8
Q9UJ70	NAGK	N-acetyl-	37	13	18	3	344	37.4
Q8TDZ2	MICAL1	[F-actin]	14	13	17	13	1067	117.8
O15511	ARPC5	Actin-rel	66	7	21	7	151	16.3
H3BPC4	UBE2I	SUMO-conj	79	8	20	8	70	8
Q92890	UFD1	Ubiquitir	38	10	15	10	307	34.5
Q9BWD1	ACAT2	Acetyl-Cc	34	7	9	7	397	41.3
P17174	GOT1	Aspartate	28	11	17	11	413	46.2
O00461	GOLIM4	Golgi int	17	13	18	13	696	81.8
P55809	OXCT1	Succinyl-	19	8	17	8	520	56.1
Q9H9B4	SFXN1	Sideroflе	34	9	15	9	322	35.6
Q8WU90	ZC3H15	Zinc fing	32	12	16	12	426	48.6
Q13217	DNAJC3	DnaJ homc	33	16	21	16	504	57.5
P08754	GNAI3	Guanine n	32	11	16	4	354	40.5
Q96PK6	RBM14	RNA-bindi	20	13	19	13	669	69.4
P50479	PDLIM4	PDZ and L	42	10	21	10	330	35.4
Q01105	SET	Protein S	29	8	22	8	290	33.5
Q15643	TRIP11	Thyroid r	9	17	18	17	1979	227.4
O00148	DDX39A	ATP-deper	28	12	23	5	427	49.1
Q9UBQ7	GRHPR	Glyoxylat	29	7	13	7	328	35.6
P15121	AKR1B1	Aldose re	34	10	25	10	316	35.8
Q9P0L0	VAPA	Vesicle-a	43	10	26	9	249	27.9
Q92922	SMARCC1	SWI/SNF c	13	12	17	6	1105	122.8
Q9ULC4	MCTS1	Malignant	59	9	14	9	181	20.5
Q14498	RBM39	RNA-bindi	19	9	16	9	530	59.3
P49755	TMED10	Transmemb	40	8	16	8	219	25
F8W7C6	RPL10	60S ribos	46	7	34	1	163	18.6
Q96IZ0	PAWR	PRKC apop	36	7	12	7	340	36.5
P09543	CNP	2',3'-cyc	26	14	16	14	421	47.5
Q9H0A0	NAT10	RNA cytid	17	15	16	15	1025	115.7
P62987	UBA52	Ubiquitir	59	9	120	1	128	14.7
Q7Z460	CLASP1	CLIP-assc	9	12	13	9	1538	169.3
P21283	ATP6V1C1	V-type pr	40	16	17	16	382	43.9
P52788	SMS	Spermine	32	12	18	12	366	41.2
P35659	DEK	Protein D	22	8	14	8	375	42.6
A0A0A0MRM	MYO6	Unconvent	11	14	16	14	1253	144.9
O75489	NDUFS3	NADH dehy	47	11	19	11	264	30.2
A0A0U1RR3	hCG_20395	Histone H	49	7	36	1	169	18.5
P32455	GBP1	Guanylate	22	13	16	11	592	67.9
O14558	HSPB6	Heat shoc	53	5	28	5	160	17.1
P55263	ADK	Adenosine	34	10	16	10	362	40.5
Q7Z2W4	ZC3HAV1	Zinc fing	17	14	18	14	902	101.4
P29992	GNA11	Guanine n	28	10	15	5	359	42.1
Q9UKY7	CDV3	Protein C	53	9	13	9	258	27.3

P83436	COG7	Conservec	19	13	15	13	770	86.3
Q6XZF7	DNMBP	Dynamin-b	9	12	14	12	1577	177.2
O95433	AHSA1	Activator	36	11	18	11	338	38.3
O95793	STAU1	Double-st	12	8	13	2	577	63.1
P52292	KPNA2	Importin	19	9	12	9	529	57.8
P42892	ECE1	Endotheli	18	14	19	14	770	87.1
P33993	MCM7	DNA repli	23	15	18	15	719	81.3
P35237	SERPINB6	Serpin B6	36	11	16	11	376	42.6
P32322	PYCR1	Pyrroline	33	8	17	7	319	33.3
Q07666	KHDRBS1	KH domair	23	8	18	8	443	48.2
O95816	BAG2	BAG famil	41	12	24	11	211	23.8
P50148	GNAQ	Guanine n	30	10	15	5	359	42.1
P08621	SNRNP70	U1 small	25	13	22	13	437	51.5
H7C286	NAGK	N-acetyl-	61	11	15	1	196	22
Q01433	AMPD2	AMP deami	17	15	17	15	879	100.6
Q14166	TTLL12	Tubulin--	17	9	16	9	644	74.4
Q10713	PMPCA	Mitochonc	23	10	14	10	525	58.2
Q00577	PURA	Transcrip	31	9	15	9	322	34.9
O15355	PPM1G	Protein p	23	13	19	13	546	59.2
P00441	SOD1	Superoxic	44	8	33	8	154	15.9
P19525	EIF2AK2	Interferc	19	10	15	10	551	62.1
Q9UEW8	STK39	STE20/SPS	23	12	13	8	545	59.4
Q9BUQ8	DDX23	Probable	15	12	14	12	820	95.5
Q9UNE7	STUB1	E3 ubiqui	40	13	16	12	303	34.8
Q9Y223	GNE	Bifunctic	19	13	15	13	722	79.2
Q9BUJ2	HNRNPUL1	Heterogen	19	12	13	12	856	95.7
D6RB85	CANX	Calnexin	48	9	29	1	144	16
P31040	SDHA	Succinate	20	11	20	11	664	72.6
Q53GQ0	HSD17B12	Very-long	26	7	20	7	312	34.3
Q9UH99	SUN2	SUN domai	24	12	17	11	717	80.3
P39656	DDOST	Dolichyl-	24	11	25	11	456	50.8
P48556	PSMD8	26S prote	43	13	19	13	350	39.6
Q14165	MLEC	Malectin	29	8	16	8	292	32.2
P13796	LCP1	Plastin-2	12	8	19	1	627	70.2
P33991	MCM4	DNA repli	16	15	19	15	863	96.5
Q8TAT6	NPLOC4	Nuclear p	26	15	20	15	608	68.1
O96019	ACTL6A	Actin-lik	20	9	14	9	429	47.4
Q86XP3	DDX42	ATP-deper	19	13	16	13	938	102.9
P59998	ARPC4	Actin-rel	69	10	25	10	168	19.7
Q5T9A4	ATAD3B	ATPase fa	22	16	19	4	648	72.5
Q5GLZ8	HERC4	Probable	12	12	14	12	1057	118.5
Q9BR76	CORO1B	Coronin-1	21	12	22	12	489	54.2
O43865	AHCYL1	S-adenosy	24	14	17	6	530	58.9
P84085	ARF5	ADP-ribos	45	7	26	4	180	20.5
Q9UNF1	MAGED2	Melanoma-	18	12	22	11	606	64.9
Q99471	PFDN5	Prefoldir	54	7	19	7	154	17.3
P09104	ENO2	Gamma-enc	26	8	31	6	434	47.2
H0YGR4	REXO2	Oligoribc	34	7	21	7	192	22.1
P17252	PRKCA	Protein k	24	14	20	13	672	76.7
Q9UIJ7	AK3	GTP:AMP p	44	10	13	10	227	25.6
O00170	AIP	AH recept	37	10	14	10	330	37.6

P36542	ATP5F1C	ATP synth	23	7	17	7	298	33
P18031	PTPN1	Tyrosine-	29	12	16	12	435	49.9
Q92688	ANP32B	Acidic le	27	8	18	4	251	28.8
P43304	GPD2	Glycerol-	18	13	14	13	727	80.8
Q99747	NAPG	Gamma-sol	35	10	13	10	312	34.7
E9PBG7	CAMK2D	Calcium/c	26	11	16	9	512	57.7
Q9UBQ5	EIF3K	Eukaryoti	44	7	13	7	218	25
P62266	RPS23	40S ribos	29	5	21	5	143	15.8
P15374	UCHL3	Ubiquitin	41	7	12	7	230	26.2
K9J957	PSME3	Proteasom	39	10	14	10	231	26.9
P12107	COL11A1	Collagen	9	13	17	9	1806	181
O75976	CPD	Carboxype	10	13	15	13	1380	152.8
O00151	PDLIM1	PDZ and L	39	10	13	10	329	36
Q13443	ADAM9	Disintegr	21	14	14	14	819	90.5
E7EW49	CLASP2	CLIP-asso	9	12	13	9	1514	165.6
P42566	EPS15	Epidermal	18	14	15	14	896	98.6
Q13618	CUL3	Cullin-3	18	14	15	14	768	88.9
P04632	CAPNS1	Calpain s	35	7	22	7	268	28.3
P11388	TOP2A	DNA topoi	9	14	16	8	1531	174.3
O75955	FLOT1	Flotillin	28	10	13	10	427	47.3
P54289	CACNA2D1	Voltage-d	14	13	13	13	1103	124.5
Q15121	PEA15	Astrocyti	58	6	15	6	130	15
A0A2R8Y6Y	SUCLA2	Succinate	30	13	15	13	484	52.6
Q8N0X7	SPART	Spartin C	21	14	18	14	666	72.8
G3V1R5	NRDC	Nardilysi	10	12	14	12	1087	125
E7EQ69	NAA50	N-alpha-a	55	9	15	9	168	19.3
Q9HCC0	MCCC2	Methylcro	18	8	12	8	563	61.3
Q96AQ6	PBXIP1	Pre-B-cel	19	11	15	11	731	80.6
Q15020	SART3	Squamous	11	12	14	12	963	109.9
A0A0A0MSE	EXOC7	Exocyst c	19	12	13	12	693	78.8
P31942	HNRNPH3	Heterogen	25	7	17	6	346	36.9
Q5JR08	RHOC	Rho-relat	34	6	17	1	188	21.5
P61201	COPS2	COP9 sign	30	12	19	12	443	51.6
Q9UHV9	PFDN2	Prefoldin	60	10	18	10	154	16.6
Q96JJ7	TMX3	Protein d	22	9	16	9	454	51.8
Q13425	SNTB2	Beta-2-sy	26	12	14	11	540	57.9
P17096	HMGA1	High mobi	56	7	24	7	107	11.7
Q5JTV8	TOR1AIP1	Torsin-1A	22	13	16	12	583	66.2
P04040	CAT	Catalase	22	10	13	10	527	59.7
Q9Y4P3	TBL2	Transduci	25	13	16	13	447	49.8
Q99426	TBCB	Tubulin-f	45	9	23	9	244	27.3
O60832	DKC1	H/ACA rib	23	14	18	14	514	57.6
Q9H6R4	NOL6	Nucleolar	14	13	14	13	1146	127.5
Q92804	TAF15	TATA-bind	29	11	18	9	592	61.8
Q7Z4I7	LIMS2	LIM and s	20	7	18	2	341	38.9
P25685	DNAJB1	DnaJ homo	35	12	22	11	340	38
Q14258	TRIM25	E3 ubiqui	21	10	12	10	630	70.9
Q6NUK1	SLC25A24	Calcium-b	24	13	21	13	477	53.3
O95486	SEC24A	Protein t	11	10	14	9	1093	119.7
O75475	PSIP1	PC4 and S	22	11	20	10	530	60.1
P08473	MME	Neprilysi	16	12	14	12	750	85.5

Q969V3	NCLN	Nicalin C	16	8	13	8	563	62.9
H0YD13	CD44	CD44 anti	35	7	30	7	206	22.7
Q09028	RBBP4	Histone-b	24	10	19	3	425	47.6
Q15056	EIF4H	Eukaryoti	47	10	21	10	248	27.4
O75436	VPS26A	Vacuolar	34	9	18	8	327	38.1
Q8N392	ARHGAP18	Rho GTPas	21	14	15	14	663	74.9
Q99459	CDC5L	Cell divi	19	10	11	10	802	92.2
P43490	NAMPT	Nicotinam	20	9	17	9	491	55.5
H0YM70	PSME2	Proteasom	35	7	16	7	228	26
H7C0E5	ZPR1	Zinc fing	29	11	16	11	386	42.6
O60256	PRPSAP2	Phosphori	39	12	14	10	369	40.9
Q9UNS2	COPS3	COP9 sign	25	8	14	8	423	47.8
Q53FA7	TP53I3	Quinone c	36	11	23	11	332	35.5
D3YTB1	RPL32	60S ribos	46	7	24	7	133	15.6
G3V3E8	NPC2	NPC intra	37	6	16	6	174	19.2
Q9H845	ACAD9	Acyl-CoA	17	10	15	10	621	68.7
P48163	ME1	NADP-depe	24	13	19	13	572	64.1
Q08AF3	SLFN5	Schlafen	15	14	16	13	891	101
Q13011	ECH1	Delta(3,5	33	10	15	10	328	35.8
P10768	ESD	S-formylg	27	8	15	8	282	31.4
E9PFN5	GSTK1	Glutathio	44	7	15	7	190	21.7
P10515	DLAT	Dihydroli	22	12	16	12	647	69
F5GZ78	PXN	Paxillin	18	11	15	11	589	64.2
P31749	AKT1	RAC-alpha	28	12	14	8	480	55.7
Q9H8Y8	GORASP2	Golgi rea	25	10	15	9	452	47.1
Q9NP72	RAB18	Ras-relat	51	8	14	8	206	23
E7EM64	COPS6	COP9 sign	35	10	18	10	326	36
P28370	SMARCA1	Probable	15	15	15	12	1054	122.5
Q06124	PTPN11	Tyrosine-	25	15	18	15	597	68.4
D6RB59	EXOC3	Exocyst c	22	11	14	11	462	53.2
I1E4Y6	GIGYF2	GRB10-int	12	13	15	13	1321	152.4
P40121	CAPG	Macrophag	21	6	11	6	348	38.5
A0A087WY5	VTA1	Chromosom	29	8	13	8	280	31.1
A6NHL2	TUBAL3	Tubulin a	10	5	40	1	446	49.9
K7ELC7	RPL27	60S ribos	45	8	30	7	144	16.5
P61081	UBE2M	NEDD8-con	54	10	15	10	183	20.9
P04179	SOD2	Superoxid	33	8	21	8	222	24.7
P20936	RASA1	Ras GTPas	11	10	12	10	1047	116.3
Q9UHD1	CHORDC1	Cysteine	36	9	13	9	332	37.5
P50914	RPL14	60S ribos	31	7	24	7	215	23.4
P61020	RAB5B	Ras-relat	38	6	11	4	215	23.7
Q92905	COPS5	COP9 sign	31	8	11	8	334	37.6
P53007	SLC25A1	Tricarbox	24	8	15	8	311	34
A0A024RCR	BAG6	HLA-B ass	12	9	12	9	1126	118.6
F5GYQ1	ATP6V0D1	V-type pr	27	10	17	10	392	44.6
O14737	PDCD5	Programme	59	8	17	8	125	14.3
P61604	HSPE1	10 kDa he	64	9	29	5	102	10.9
Q9Y295	DRG1	Developme	28	8	12	8	367	40.5
P20339	RAB5A	Ras-relat	49	7	13	5	215	23.6
Q5JSH3	WDR44	WD repeat	15	13	13	13	913	101.3
P42166	TMPO	Lamina-as	17	7	11	3	694	75.4

P20290	BTF3	Transcrip	57	9	19	5	206	22.2
E7EWW0	VPS35L	VPS35 end	10	11	13	11	1052	118.5
O15061	SYNM	Synemin C	8	14	14	14	1565	172.7
P13073	COX4I1	Cytochrom	40	8	24	8	169	19.6
Q9Y371	SH3GLB1	Endophili	32	10	14	9	365	40.8
Q9HD45	TM9SF3	Transmemb	13	8	14	8	589	67.8
O75828	CBR3	Carbonyl	32	8	15	6	277	30.8
O75746	SLC25A12	Calcium-b	18	10	12	9	678	74.7
Q15631	TSN	Translin	37	8	15	8	228	26.2
Q9NRV9	HEBP1	Heme-bind	59	9	12	9	189	21.1
A0A1B0GUA	KIF1BP	KIF1-bind	22	12	17	12	646	74.7
O00139	KIF2A	Kinesin-l	16	11	13	11	706	79.9
E9PDE8	HSPA4L	Heat shoc	10	8	13	3	813	91.9
Q14254	FLOT2	Flotillin	29	12	16	12	428	47
O75351	VPS4B	Vacuolar	25	10	14	6	444	49.3
P47813	EIF1AX	Eukaryoti	38	8	18	1	144	16.5
Q15907	RAB11B	Ras-relat	44	9	21	9	218	24.5
Q92520	FAM3C	Protein F	33	6	11	6	227	24.7
O00161	SNAP23	Synaptoso	41	7	10	7	211	23.3
Q96A65	EXOC4	Exocyst c	15	14	15	14	974	110.4
P10301	RRAS	Ras-relat	40	7	12	5	218	23.5
Q96JB5	CDK5RAP3	CDK5 regu	21	10	12	10	506	56.9
Q04695	KRT17	Keratin,	17	11	19	1	432	48.1
P63151	PPP2R2A	Serine/th	23	8	13	6	447	51.7
Q9NVP1	DDX18	ATP-depen	17	11	13	11	670	75.4
Q96K76	USP47	Ubiquitin	11	12	12	12	1375	157.2
Q9HC35	EML4	Echinoder	13	11	13	11	981	108.8
P04424	ASL	Argininos	27	12	15	12	464	51.6
Q16630	CPSF6	Cleavage	22	10	18	10	551	59.2
Q96A33	CCDC47	Coiled-co	25	9	12	9	483	55.8
Q9Y4W6	AFG3L2	AFG3-like	18	17	18	16	797	88.5
J3KS54	FLII	Protein f	21	12	15	1	700	78
P23921	RRM1	Ribonucle	15	11	16	11	792	90
O60443	GSDME	Gasdermin	22	8	9	8	496	54.5
Q8WXX5	DNAJC9	DnaJ homo	28	7	10	7	260	29.9
Q9Y305	ACOT9	Acyl-coen	23	10	16	10	439	49.9
O14776	TCERG1	Transcrip	13	13	14	13	1098	123.8
Q9H5N1	RABEP2	Rab GTPas	21	12	13	12	569	63.5
O43488	AKR7A2	Aflatoxin	26	8	12	8	359	39.6
B5MCF9	PES1	Pescadill	21	13	15	13	571	66
Q9BQS8	FYCO1	FYVE and	9	14	15	14	1478	166.9
Q8TEX9	IPO4	Importin-	13	12	17	12	1081	118.6
Q8WUM0	NUP133	Nuclear p	8	9	11	9	1156	128.9
Q9UI15	TAGLN3	Transgeli	32	6	22	4	199	22.5
P11498	PC	Pyruvate	11	10	12	10	1178	129.6
Q5T123	SH3BGRL3	SH3 domai	51	6	23	6	88	9.4
P27361	MAPK3	Mitogen-a	26	9	18	5	379	43.1
P52943	CRIP2	Cysteine-	39	5	11	5	208	22.5
Q14318	FKBP8	Peptidyl-	22	8	11	8	412	44.5
P60953	CDC42	Cell divi	42	7	23	6	191	21.2
Q14232	EIF2B1	Translati	30	10	15	10	305	33.7

Q5QPL9	RALY	RNA-bindi	43	11	13	11	237	24.7
A0A0A0MS5	PRKACB	cAMP-depe	27	9	17	2	357	41.3
E9PC52	RBBP7	Histone-b	22	9	17	2	416	46.9
Q9ULT8	HECTD1	E3 ubiqui	5	13	13	13	2610	289.2
Q01780	EXOSC10	Exosome c	14	11	12	11	885	100.8
Q9HAV0	GNB4	Guanine n	31	9	15	4	340	37.5
P35442	THBS2	Thrombosp	9	9	12	9	1172	129.9
P28482	MAPK1	Mitogen-a	28	10	17	6	360	41.4
P54819	AK2	Adenylate	32	7	14	7	239	26.5
P31937	HIBADH	3-hydroxy	23	5	8	5	336	35.3
Q86UU1	PHLDB1	Pleckstri	9	11	12	6	1377	151.1
Q92769	HDAC2	Histone d	20	8	11	5	488	55.3
Q70E73	RAPH1	Ras-assoc	11	12	14	12	1250	135.2
O15357	INPPL1	Phosphati	10	12	13	12	1258	138.5
Q16777	HIST2H2AC	Histone H	63	7	35	2	129	14
Q9UJS0	SLC25A13	Calcium-b	16	9	11	8	675	74.1
P36405	ARL3	ADP-ribos	48	8	16	8	182	20.4
B4DLN1		cDNA FLJ6	18	7	14	2	442	48.1
Q9NUQ9	FAM49B	Protein F	35	9	11	9	324	36.7
P61086	UBE2K	Ubiquitin	45	7	20	7	200	22.4
Q01970	PLCB3	1-phospha	8	9	10	9	1234	138.7
O75439	PMPCB	Mitochond	22	11	13	10	489	54.3
O00273	DFFA	DNA fragm	31	9	14	9	331	36.5
Q13242	SRSF9	Serine/ar	37	9	15	9	221	25.5
A0A024R4M	RPS9	40S ribos	40	10	25	10	194	22.6
P46779	RPL28	60S ribos	49	9	32	9	137	15.7
Q9UDY2	TJP2	Tight jun	12	14	14	14	1190	133.9
K7EQJ5	RPS15	40S ribos	28	5	25	5	141	16.6
P28161	GSTM2	Glutathic	52	12	16	7	218	25.7
I3L3Q4	GLOD4	Glyoxalas	44	10	16	1	227	25.5
P32969	RPL9	60S ribos	29	6	20	6	192	21.9
P07858	CTSB	Cathepsin	24	7	20	7	339	37.8
A6NDG6	PGP	Glycerol-	29	7	10	7	321	34
A0A1W2PQF	SCARB2	Lysosome	14	6	14	6	449	51
A2RRP1	NBAS	Neuroblas	6	13	14	13	2371	268.4
Q9UK76	JPT1	Jupiter m	42	6	16	6	154	16
O60493	SNX3	Sorting n	47	8	14	6	162	18.8
Q15582	TGFBI	Transform	20	12	16	12	683	74.6
Q86V21	AACS	Acetoacet	18	12	17	12	672	75.1
B0QYK0	EWSR1	RNA-bindi	11	6	13	6	618	64.9
Q86Y82	STX12	Syntaxin-	31	8	14	8	276	31.6
O14602	EIF1AY	Eukaryoti	38	8	17	1	144	16.4
Q16512	PKN1	Serine/th	12	13	13	12	942	103.9
F8VZJ2	NACA	Nascent p	36	4	19	4	136	15
O43493	TGOLN2	Trans-Gol	16	7	14	7	479	51
P07093	SERPINE2	Glia-deri	23	9	11	9	398	44
Q6PCE3	PGM2L1	Glucose 1	18	11	13	10	622	70.4
Q6DKJ4	NXN	Nucleored	26	9	11	9	435	48.4
O00505	KPNA3	Importin	17	8	19	4	521	57.8
P16989	YBX3	Y-box-bin	26	7	15	5	372	40.1
Q14137	BOP1	Ribosome	17	10	11	10	746	83.6

P28072	PSMB6	Proteasom	30	8	20	8	239	25.3
O43290	SART1	U4/U6.U5	15	9	11	9	800	90.2
O00442	RTCA	RNA 3'-te	27	7	9	7	366	39.3
O60888	CUTA	Protein C	34	5	14	5	179	19.1
P18859	ATP5J	ATP synth	45	5	12	5	108	12.6
Q13131	PRKAA1	5'-AMP-ac	24	10	12	10	559	64
P30626	SRI	Sorcin OS	46	8	18	8	198	21.7
P06703	S100A6	Protein S	66	7	52	7	90	10.2
Q16186	ADRM1	Proteasom	23	10	18	10	407	42.1
Q12907	LMAN2	Vesicular	22	8	16	8	356	40.2
O76031	CLPX	ATP-depen	17	10	11	10	633	69.2
Q9NQ88	TIGAR	Fructose-	28	7	12	7	270	30
P30043	BLVRB	Flavin re	53	8	15	8	206	22.1
Q9BRF8	CPPED1	Serine/th	37	9	14	9	314	35.5
O43684	BUB3	Mitotic c	32	9	10	9	328	37.1
P09497	CLTB	Clathrin	31	9	18	9	229	25.2
Q04721	NOTCH2	Neurogeni	5	9	10	9	2471	265.2
Q9UBI6	GNG12	Guanine n	69	5	15	5	72	8
A0A087X14	SEPT8	Septin-8	20	8	12	5	426	49.3
P39687	ANP32A	Acidic le	22	7	17	3	249	28.6
Q9H444	CHMP4B	Charged m	27	6	16	6	224	24.9
P25787	PSMA2	Proteasom	35	7	14	7	234	25.9
P30085	CMPK1	UMP-CMP k	44	7	17	7	196	22.2
P30038	ALDH4A1	Delta-1-p	20	11	12	11	563	61.7
P50579	METAP2	Methionin	24	10	13	10	478	52.9
Q99805	TM9SF2	Transmemb	12	7	16	7	663	75.7
Q9UHQ9	CYB5R1	NADH-cytc	28	9	15	9	305	34.1
Q8TD16	BICD2	Protein b	13	11	12	11	824	93.5
P53990	IST1	IST1 homc	27	10	12	10	364	39.7
Q14669	TRIP12	E3 ubiqui	6	11	12	11	1992	220.3
P62993	GRB2	Growth fa	47	10	13	10	217	25.2
Q9H7Z7	PTGES2	Prostagla	25	9	12	9	377	41.9
Q8IVM0	CCDC50	Coiled-cc	31	9	11	9	306	35.8
Q9H832	UBE2Z	Ubiquitin	26	8	11	8	354	38.2
Q9Y2T2	AP3M1	AP-3 comp	21	8	12	8	418	46.9
P53597	SUCLG1	Succinate	20	6	13	6	346	36.2
Q9H2G2	SLK	STE20-lik	9	12	13	11	1235	142.6
P26006	ITGA3	Integrin	7	7	12	7	1051	116.5
Q9BRK5	SDF4	45 kDa ca	28	9	12	9	362	41.8
Q9BZF1	OSBPL8	Oxysterol	14	11	11	11	889	101.1
P51153	RAB13	Ras-relat	38	8	16	6	203	22.8
Q66K74	MAP1S	Microtubu	11	10	13	10	1059	112.1
O15305	PMM2	Phosphoma	35	9	14	8	246	28.1
E7ESC6	XPO7	Exportin-	10	10	11	10	1088	124
P26022	PTX3	Pentraxin	27	7	10	7	381	41.9
Q8TDQ7	GNPDA2	Glucosami	39	8	10	6	276	31.1
Q8IWB7	WDFY1	WD repeat	22	8	11	8	410	46.3
Q9BZE1	MRPL37	39S ribos	23	7	10	7	423	48.1
Q9NTZ6	RBM12	RNA-bindi	12	11	13	10	932	97.3
Q12788	TBL3	Transduci	15	9	11	9	808	89
E9PFH4	TNPO3	Transport	13	10	11	10	857	96.6

P48729	CSNK1A1	Casein ki	26	8	13	3	337	38.9
P51398	DAP3	28S ribos	25	9	11	9	398	45.5
O60826	CCDC22	Coiled-cc	16	8	9	8	627	70.7
Q6NYC8	PPP1R18	Phostensi	15	8	11	8	613	67.9
Q02750	MAP2K1	Dual spec	25	8	14	3	393	43.4
P49756	RBM25	RNA-bindi	12	10	12	10	843	100.1
E9PFR3	PPP2R5D	Serine/th	22	11	14	7	594	69.1
Q9Y5S1	TRPV2	Transient	13	7	10	7	764	85.9
P63173	RPL38	60S ribos	50	4	24	4	70	8.2
Q96CT7	CCDC124	Coiled-cc	45	10	13	10	223	25.8
P10606	COX5B	Cytochrom	44	6	20	6	129	13.7
Q96T51	RUFY1	RUN and F	15	10	10	9	708	79.8
P41567	EIF1	Eukaryoti	42	6	9	6	113	12.7
P53999	SUB1	Activated	51	9	17	9	127	14.4
Q15435	PPP1R7	Protein p	29	11	12	11	360	41.5
E5RJR5	SKP1	S-phase k	29	7	14	7	163	18.7
E7ESP9	NEFM	Neurofila	11	11	32	10	877	98.3
P84157	MXRA7	Matrix-re	33	6	12	4	204	21.5
P00568	AK1	Adenylate	30	7	15	6	194	21.6
Q96C36	PYCR2	Pyrroline	24	6	11	5	320	33.6
Q96N66	MBOAT7	Lysophosp	13	6	13	6	472	52.7
A0A087WY3	YTHDF3	YTH domai	16	8	13	4	588	64.5
P52815	MRPL12	39S ribos	35	6	13	1	198	21.3
P06865	HEXA	Beta-hexo	18	9	13	9	529	60.7
Q14571	ITPR2	Inositol	6	17	17	14	2701	307.9
Q9BZE4	GTPBP4	Nucleolar	16	9	11	9	634	73.9
P62834	RAP1A	Ras-relat	53	7	16	2	184	21
Q6NUQ4	TMEM214	Transmemb	16	10	13	10	689	77.1
F8W031		Uncharact	29	7	13	6	263	29.2
Q14CX7	NAA25	N-alpha-a	10	10	13	10	972	112.2
O95865	DDAH2	N(G),N(G)	33	8	11	7	285	29.6
P05362	ICAM1	Intercell	19	8	12	8	532	57.8
P19838	NFKB1	Nuclear f	11	10	10	10	968	105.3
Q95365	HLA-B	HLA class	30	8	14	1	362	40.4
O96000	NDUFB10	NADH dehy	43	7	10	7	172	20.8
O75477	ERLIN1	Erlin-1 C	24	10	15	7	346	38.9
P30740	SERPINB1	Leukocyte	25	9	11	9	379	42.7
A8MZF9	DRG2	Developme	27	8	10	8	343	38.1
A0A087WUC	SPCS2	Signal pe	36	9	17	9	227	25.1
Q9BXS5	AP1M1	AP-1 comp	25	10	15	10	423	48.6
Q9Y277	VDAC3	Voltage-d	29	8	16	7	283	30.6
Q8IY81	FTSJ3	pre-rRNA	20	12	12	12	847	96.5
A0A1B0GTG	ADSL	Adenylosu	24	8	13	8	377	42.5
P0CG39	POTEJ	POTE anky	6	5	62	2	1038	117.3
P01111	NRAS	GTPase NR	31	5	13	1	189	21.2
P02462	COL4A1	Collagen	7	9	11	9	1669	160.5
P05386	RPLP1	60S acidi	52	2	16	2	114	11.5
Q16795	NDUFA9	NADH dehy	27	9	12	9	377	42.5
P26358	DNMT1	DNA (cyto	9	14	14	14	1616	183.1
Q9NTX5	ECHDC1	Ethylmalo	30	7	9	7	307	33.7
Q15113	PCOLCE	Procollag	22	8	10	8	449	47.9

Q8IV08	PLD3	Phospholi	15	6	16	6	490	54.7
P17931	LGALS3	Galectin-	28	7	16	7	250	26.1
Q9BQ67	GRWD1	Glutamate	21	7	10	7	446	49.4
O43765	SGTA	Small glu	27	8	14	8	313	34
Q5QNY5	PEX19	Peroxisom	40	7	10	5	235	26.1
Q9NP61	ARFGAP3	ADP-ribos	22	10	12	10	516	56.9
M0R165	EPS15L1	Epidermal	12	8	11	8	756	83.4
P31949	S100A11	Protein S	52	6	35	6	105	11.7
Q9Y5X3	SNX5	Sorting n	22	7	10	6	404	46.8
P53004	BLVRA	Biliverdi	24	8	13	8	296	33.4
P42285	MTREX	Exosome R	10	11	13	11	1042	117.7
Q5T3Q7	HEATR1	HEAT repe	5	11	13	11	2063	233.1
P53582	METAP1	Methionin	24	7	10	7	386	43.2
P62330	ARF6	ADP-ribos	50	6	11	6	175	20.1
Q9Y2H6	FNDC3A	Fibronect	11	10	10	10	1198	131.8
P01112	HRAS	GTPase HR	39	6	10	2	189	21.3
P62851	RPS25	40S ribos	36	7	21	7	125	13.7
Q9BVK6	TMED9	Transmemb	28	6	13	6	235	27.3
P49790	NUP153	Nuclear p	8	9	11	9	1475	153.8
Q9P258	RCC2	Protein R	23	12	13	12	522	56
P00966	ASS1	Argininos	20	8	14	8	412	46.5
O60869	EDF1	Endotheli	45	9	19	9	148	16.4
P13807	GYS1	Glycogen	13	8	10	8	737	83.7
P15153	RAC2	Ras-relat	34	7	16	2	192	21.4
Q96RS6	NUDCD1	NudC doma	19	12	14	12	583	66.7
J3KR97	TBCD	Tubulin-s	10	11	12	11	1230	136.5
F8VYN9	ARL1	ADP-ribos	24	4	7	4	194	21.8
A0A087WXS	ASNA1	ATPase AS	24	6	12	4	331	37.1
Q13155	AIMP2	Aminoacyl	26	9	15	9	320	35.3
Q15555	MAPRE2	Microtubu	28	8	11	6	327	37
Q9NR50	EIF2B3	Translati	26	11	12	11	452	50.2
P51610	HCFC1	Host cell	6	10	12	10	2035	208.6
Q9UP95	SLC12A4	Solute ca	10	10	13	10	1085	120.6
Q9HD26	GOPC	Golgi-ass	19	10	13	10	462	50.5
O60462	NRP2	Neuropili	12	11	12	11	931	104.8
G5E9D5	ELAC2	ElaC homo	14	9	12	9	807	90
Q13564	NAE1	NEDD8-act	20	8	9	8	534	60.2
P28300	LOX	Protein-l	24	7	9	6	417	46.9
G3V203	RPL18	60S ribos	34	5	17	5	164	18.7
F2Z2Y4	PDXK	Pyridoxal	26	7	11	7	272	30.6
O94760	DDAH1	N(G),N(G)	36	7	9	6	285	31.1
Q9Y6K9	IKBKG	NF-kappa-	21	8	10	8	419	48.2
Q8WXF7	ATL1	Atlastin-	22	10	11	10	558	63.5
Q0VDF9	HSPA14	Heat shoc	22	11	13	11	509	54.8
E7ENY0	ADD1	Alpha-add	15	8	10	8	663	73.4
Q9H2U2	PPA2	Inorganic	31	9	12	8	334	37.9
Q9HB07	C12orf10	UPF0160 p	27	9	13	9	376	42.4
O15269	SPTLC1	Serine pa	21	11	12	11	473	52.7
O75400	PRPF40A	Pre-mRNA-	10	11	13	11	957	108.7
A0A087WUQ	GPX1	Glutathic	38	6	11	6	202	21.9
A0A2R8Y85	SMARCE1	SWI/SNF-r	29	11	13	10	367	41.8

Q08209	PPP3CA	Serine/th	17	8	10	3	521	58.7
Q03135	CAV1	Caveolin-	46	7	16	7	178	20.5
Q12996	CSTF3	Cleavage	17	9	10	9	717	82.9
P61254	RPL26	60S ribos	41	9	22	2	145	17.2
Q9UJW0	DCTN4	Dynactin	23	9	14	9	460	52.3
Q6UW63	KDELC1	KDEL moti	23	11	11	10	502	58
P14324	FDPS	Farnesyl	14	6	16	6	419	48.2
Q9H7D0	DOCK5	Dedicator	7	13	13	12	1870	215.2
Q92791	P3H4	Endoplasm	25	9	13	9	437	50.3
Q9UBF2	COPG2	Coatomer	13	9	12	7	871	97.6
Q9BZK7	TBL1XR1	F-box-lik	17	7	7	7	514	55.6
Q99436	PSMB7	Proteasom	33	7	13	7	277	29.9
Q16629	SRSF7	Serine/ar	31	8	17	7	238	27.4
Q9Y3U8	RPL36	60S ribos	36	6	20	6	105	12.2
H3BN98		Uncharact	23	6	25	2	237	27.2
O15400	STX7	Syntaxin-	36	8	12	8	261	29.8
Q9Y5Z4	HEBP2	Heme-binc	41	8	12	8	205	22.9
Q9Y2G5	POFUT2	GDP-fucos	24	13	18	6	429	49.9
P26583	HMGB2	High mobi	34	10	15	8	209	24
O75694	NUP155	Nuclear p	7	8	12	8	1391	155.1
E7ENQ1	MAP4K4	Mitogen-a	10	10	11	8	1154	132.1
Q13630	TSTA3	GDP-L-fuc	31	9	14	9	321	35.9
Q02978	SLC25A11	Mitochonc	33	9	12	9	314	34
P51116	FXR2	Fragile X	15	9	10	6	673	74.2
O94804	STK10	Serine/th	13	12	12	11	968	112.1
O15212	PFDN6	Prefoldir	50	8	11	8	129	14.6
Q9HD15	SRA1	Steroid r	31	6	10	6	236	25.7
K7ERE3	KRT13	Keratin,	16	7	15	1	415	45.2
Q01085	TIAL1	Nucleolys	19	6	10	4	375	41.6
Q15642	TRIP10	Cdc42-int	19	11	12	10	601	68.3
F5H442	TSG101	Tumor sus	22	7	9	7	365	40.9
Q96CS3	FAF2	FAS-assoc	18	7	9	7	445	52.6
P62244	RPS15A	40S ribos	61	8	27	4	130	14.8
Q9NZ08	ERAP1	Endoplasm	11	10	11	10	941	107.2
Q9UN86	G3BP2	Ras GTPas	21	10	13	9	482	54.1
O95299	NDUFA10	NADH dehy	17	7	10	7	355	40.7
P62995	TRA2B	Transform	27	6	10	6	288	33.6
O43395	PRPF3	U4/U6 sma	19	12	12	12	683	77.5
O43854	EDIL3	EGF-like	22	9	11	9	480	53.7
Q14914	PTGR1	Prostagla	29	9	11	9	329	35.8
P51452	DUSP3	Dual spec	31	4	9	4	185	20.5
Q9Y3C6	PPIL1	Peptidyl-	42	7	9	7	166	18.2
Q9ULH1	ASAP1	Arf-GAP w	8	8	9	8	1129	125.4
Q9NYU1	UGGT2	UDP-gluco	7	11	12	9	1516	174.6
Q8N556	AFAP1	Actin fil	14	9	11	9	730	80.7
E9PSI1		Transmemb	14	9	9	3	815	92.3
P46778	RPL21	60S ribos	36	8	23	8	160	18.6
Q14C86	GAPVD1	GTPase-ac	7	9	10	9	1478	164.9
Q04323	UBXN1	UBX domai	28	6	9	6	297	33.3
Q9BYD6	MRPL1	39S ribos	19	7	10	7	325	36.9
P12955	PEPD	Xaa-Pro d	17	8	11	8	493	54.5

P30048	PRDX3	Thioredox	25	4	14	4	256	27.7
P84090	ERH	Enhancer	71	6	17	6	104	12.3
Q8IWJ2	GCC2	GRIP and	7	11	11	11	1684	195.8
O60884	DNAJA2	DnaJ homc	24	10	14	10	412	45.7
Q8TBC4	UBA3	NEDD8-act	27	9	9	9	463	51.8
A0A1P0AYU	SFXN3	Sideroflε	28	7	8	7	325	36
P63096	GNAI1	Guanine n	22	8	12	1	354	40.3
Q9UN37	VPS4A	Vacuolar	22	10	14	6	437	48.9
A0A1W2PPZ	TCEA1	Transcrip	30	9	14	9	301	33.9
A0A0U1RQL	GSN	Gelsolin	28	6	13	1	232	26.3
Q7Z4H8	KDELC2	KDEL moti	25	10	10	10	507	58.5
P53367	ARFIP1	Arfaptin-	23	11	12	11	373	41.7
P46776	RPL27A	60S ribos	35	6	17	6	148	16.6
Q9UHN6	TMEM2	Cell surf	8	10	10	10	1383	154.3
Q8TCJ2	STT3B	Dolichyl-	11	11	16	9	826	93.6
Q9NZL9	MAT2B	Methionin	24	8	11	8	334	37.5
P08572	COL4A2	Collagen	6	8	9	8	1712	167.4
C9JYJ6	FILIP1L	Filamin A	12	10	10	10	837	96.2
Q96IJ6	GMPPA	Mannose-1	21	8	11	8	420	46.3
A0A0A6YYA	TMED7-TIC	Protein T	32	7	12	7	188	21.2
Q9HCE1	MOV10	Putative	13	11	11	11	1003	113.6
Q13637	RAB32	Ras-relat	30	6	11	5	225	25
P43121	MCAM	Cell surf	14	8	11	8	646	71.6
P31689	DNAJA1	DnaJ homc	21	8	11	8	397	44.8
P11172	UMPS	Uridine 5	20	11	14	11	480	52.2
P61326	MAGOH	Protein m	49	7	14	7	146	17.2
Q5T0I0	GSN	Gelsolin	22	6	14	1	260	28.9
Q9NVJ2	ARL8B	ADP-ribos	36	6	10	3	186	21.5
M0R0R2	RPS5	40S ribos	27	7	19	7	225	25.3
Q9NTJ3	SMC4	Structura	8	11	11	11	1288	147.1
O00622	CYR61	Protein C	27	10	10	10	381	42
I3L0A0	TMEM189-U	HCG204478	20	7	36	3	370	42.2
S4R3N1	HSPE1-MOB	HSPE1-MOB	35	9	19	5	261	29.7
P63167	DYNLL1	Dynein li	45	3	14	2	89	10.4
M0R026	ILVBL	Acetolact	20	8	9	8	525	56.7
I3L4X2	ABCC1	Multidrug	8	12	13	12	1440	160.4
Q86TU7	SETD3	Histone-l	15	8	10	8	594	67.2
Q15043	SLC39A14	Zinc tran	11	5	10	5	492	54.2
O95881	TXNDC12	Thioredox	35	6	12	6	172	19.2
Q15437	SEC23B	Protein t	8	4	9	2	767	86.4
A0A0A0MSI	EXOC5	Exocyst c	14	10	12	9	711	82.1
Q07021	C1QBP	Complemen	19	4	8	4	282	31.3
P46734	MAP2K3	Dual spec	29	9	11	9	347	39.3
Q13547	HDAC1	Histone d	20	8	10	5	482	55.1
Q96EY7	PTCD3	Pentatric	15	8	10	8	689	78.5
E9PGT6	COPS8	COP9 sign	36	4	10	4	173	19.3
Q9H6S3	EPS8L2	Epidermal	14	8	10	8	715	80.6
Q9P016	THYN1	Thymocyte	42	10	12	10	225	25.7
Q8NC56	LEMD2	LEM domai	17	8	9	8	503	56.9
O94919	ENDOD1	Endonucle	22	7	8	7	500	55
Q6VEQ5	WASH2P	WAS prote	17	7	9	7	465	50.3

P51532	SMARCA4	Transcrip	6	10	11	5	1647	184.5
E9PB61	ALYREF	THO compl	27	6	9	6	264	27.5
C9J0J7	PFN2	Profilin	60	5	13	3	91	9.8
P06132	UROD	Uroporphy	24	5	9	5	367	40.8
P10253	GAA	Lysosomal	11	7	10	7	952	105.3
P62316	SNRPD2	Small nuc	62	7	22	7	118	13.5
E9PNQ8	THY1	Thy-1 mem	24	3	16	3	165	18.2
Q9H2J4	PDCL3	Phosducin	28	6	11	6	239	27.6
F6UJY9	HYI	Putative	44	6	10	6	213	23.1
Q969H8	MYDGF	Myeloid-d	27	5	16	5	173	18.8
Q96CG8	CTHRC1	Collagen	23	5	16	5	243	26.2
P28070	PSMB4	Proteasom	29	5	13	5	264	29.2
Q09161	NCBP1	Nuclear c	10	8	9	8	790	91.8
Q9NW13	RBM28	RNA-bindi	15	11	12	11	759	85.7
A0A0A6YYJMACF1		Microtubu	5	9	11	1	1668	177.2
P26885	FKBP2	Peptidyl-	49	6	14	6	142	15.6
O15067	PFAS	Phosphori	8	11	11	11	1338	144.6
F5GWT4	WNK1	Serine/th	5	8	9	8	2134	225.4
P10319	HLA-B	HLA class	25	6	9	1	362	40.3
P36543	ATP6V1E1	V-type pr	32	8	13	8	226	26.1
Q9UIQ6	LNPEP	Leucyl-cy	10	9	9	9	1025	117.3
Q15717	ELAVL1	ELAV-like	21	6	11	6	326	36.1
Q13610	PWP1	Periodic	19	8	9	8	501	55.8
Q13190	STX5	Syntaxin-	24	8	9	8	355	39.6
O95292	VAPB	Vesicle-a	26	7	14	6	243	27.2
Q9UMS4	PRPF19	Pre-mRNA-	21	8	13	8	504	55.1
Q9Y512	SAMM50	Sorting a	21	9	11	9	469	51.9
O75179	ANKRD17	Ankyrin r	4	8	9	4	2603	274.1
Q92747	ARPC1A	Actin-rel	22	8	13	8	370	41.5
O00629	KPNA4	Importin	17	8	18	4	521	57.9
Q5JVZ5	ELMO2	Engulfmen	14	8	10	8	718	82.3
Q15738	NSDHL	Sterol-4-	24	9	10	9	373	41.9
O15498	YKT6	Synaptobr	40	9	14	9	198	22.4
O14787	TNPO2	Transport	9	8	12	4	897	101.3
O00267	SUPT5H	Transcrip	9	9	11	9	1087	120.9
Q9UHD9	UBQLN2	Ubiquilin	14	6	11	4	624	65.7
Q9Y673	ALG5	Dolichyl-	26	6	10	6	324	36.9
P60866	RPS20	40S ribos	36	6	18	6	119	13.4
P69905	HBA1	Hemoglobi	63	6	17	6	142	15.2
Q08945	SSRP1	FACT comp	14	8	12	8	709	81
Q3LXA3	TKFC	Triokinas	24	9	10	9	575	58.9
P35080	PFN2	Profilin-	31	4	10	2	140	15
G3V3A4	SNW1	SNW domai	19	9	9	9	571	65.4
Q14696	MESD	LRP chape	32	8	16	8	234	26.1
Q9BQ61	TRIR	Telomeras	34	5	10	5	176	18.4
P47985	UQCRFS1	Cytochrom	30	8	12	8	274	29.6
Q9BVP2	GNL3	Guanine n	21	8	11	8	549	62
Q9Y5X1	SNX9	Sorting n	15	8	11	8	595	66.6
P56945	BCAR1	Breast ca	11	9	10	9	870	93.3
Q9Y5V3	MAGED1	Melanoma-	12	9	12	8	778	86.1
P50452	SERPINB8	Serpin B8	25	9	13	8	374	42.7

F8W6C2	SPATS2L	SPATS2-li	35	8	9	1	265	29.9
Q9UNX3	RPL26L1	60S ribos	34	8	17	1	145	17.2
P35573	AGL	Glycogen	6	10	10	10	1532	174.7
P62633	CNBP	Cellular	41	6	12	6	177	19.5
P05106	ITGB3	Integrin	12	8	9	8	788	87
O60502	MGEA5	Protein C	12	10	12	10	916	102.8
P19022	CDH2	Cadherin-	12	6	9	6	906	99.7
Q15819	UBE2V2	Ubiquitin	51	7	33	3	145	16.4
A0A2R8Y5F	HARS2	Probable	17	9	12	3	512	57.5
Q15637	SF1	Splicing	11	6	9	6	639	68.3
P62318	SNRPD3	Small nuc	48	4	12	4	126	13.9
P46976	GYG1	Glycogeni	18	6	8	6	350	39.4
E9PQ57	RAE1	mRNA expo	23	9	10	9	437	47.8
Q5T4U5	ACADM	Acyl-Coen	23	9	11	9	454	50.2
Q0IIM8	TBC1D8B	TBC1 doma	9	9	10	9	1120	128.6
Q9UKD2	MRTO4	mRNA turn	36	8	11	8	239	27.5
Q9H4A6	GOLPH3	Golgi pho	33	9	11	9	298	33.8
P07205	PGK2	Phosphogl	14	6	20	2	417	44.8
F8VXU5	VPS29	Vacuolar	31	7	14	7	214	24
Q96BM9	ARL8A	ADP-ribos	39	5	8	2	186	21.4
A0A024R44	DNPEP	Aspartyl	18	8	10	8	471	52
P46109	CRKL	Crk-like	28	8	13	8	303	33.8
Q9NZD2	GLTP	Glycolipi	27	6	9	6	209	23.8
P16104	H2AFX	Histone H	48	7	26	3	143	15.1
P50453	SERPINB9	Serpin B9	29	9	11	8	376	42.4
Q4J6C6	PREPL	Prolyl en	13	9	9	9	727	83.9
H7BXH2	PPP6R3	Serine/th	10	7	10	7	827	92.4
J3KNQ4	PARVA	Alpha-par	16	7	15	6	412	46.6
Q5T0F9	CC2D1B	Coiled-cc	15	11	11	11	858	94.2
H0Y8B3	AHCYL2	Adenosylh	18	9	12	1	518	58.1
Q5SWX3	CAMK2G	Calcium/c	16	8	9	6	516	57.8
P35354	PTGS2	Prostagla	20	10	12	10	604	69
Q12792	TWF1	Twinfilin	20	7	13	4	350	40.3
Q15363	TMED2	Transmemb	28	6	21	6	201	22.7
Q9NPQ8	RIC8A	Synembryn	20	8	10	7	531	59.7
Q9UIG0	BAZ1B	Tyrosine-	7	9	9	9	1483	170.8
Q9Y608	LRRFIP2	Leucine-r	8	6	7	5	721	82.1
P35240	NF2	Merlin OS	16	8	9	8	595	69.6
D6RBQ9	HNRNPD	Heterogen	41	7	24	1	155	15.6
C9JFR7	CYCS	Cytochrom	41	5	14	5	101	11.3
P29373	CRABP2	Cellular	56	6	11	6	138	15.7
Q9Y3B7	MRPL11	39S ribos	24	4	8	4	192	20.7
Q5RKV6	EXOSC6	Exosome c	36	8	10	8	272	28.2
P58335	ANTXR2	Anthrax t	18	8	15	7	489	53.6
O15145	ARPC3	Actin-rel	23	5	14	5	178	20.5
Q12841	FSTL1	Follistat	24	8	14	8	308	35
Q9Y383	LUC7L2	Putative	19	7	10	5	392	46.5
P30154	PPP2R1B	Serine/th	13	7	11	2	601	66.2
P15586	GNS	N-acetylg	16	7	11	7	552	62
Q08380	LGALS3BP	Galectin-	15	7	11	7	585	65.3
O95487	SEC24B	Protein t	7	7	11	6	1268	137.3

Q99614	TTC1	Tetratric	21	7	7	7	292	33.5
Q9UK41	VPS28	Vacuolar	41	8	11	8	221	25.4
Q9Y6B6	SAR1B	GTP-bindi	29	6	13	3	198	22.4
O75569	PRKRA	Interferc	23	7	9	7	313	34.4
Q9BQE5	APOL2	Apolipopr	28	10	10	10	337	37.1
C9J5C3	PDCD10	Programme	28	5	7	5	202	23.6
F5H5V4	PSMD9	26S prote	42	6	12	6	153	16.9
Q9NX46	ADPRHL2	Poly(ADP-	21	7	9	7	363	38.9
Q71UI9	H2AFV	Histone H	31	4	23	2	128	13.5
M0QXD6	GTF2F1	General t	23	8	11	8	433	48.6
Q9Y5K5	UCHL5	Ubiquitin	26	7	9	7	329	37.6
O15260	SURF4	Surfeit l	21	5	17	5	269	30.4
Q96KP1	EXOC2	Exocyst c	12	11	11	11	924	104
Q16740	CLPP	ATP-deper	39	8	10	8	277	30.2
Q9Y2W2	WBP11	WW domain	13	7	10	7	641	70
Q8NI27	THOC2	THO compl	7	11	11	11	1593	182.7
P61006	RAB8A	Ras-relat	32	7	13	3	207	23.7
Q96Q11	TRNT1	CCA tRNA	23	9	9	9	434	50.1
Q96DI7	SNRNP40	U5 small	27	7	7	7	357	39.3
Q9H488	POFUT1	GDP-fucos	20	7	12	7	388	43.9
Q5VT25	CDC42BPA	Serine/th	5	9	9	5	1732	197.2
A0A087X0R	SNX12	Sorting n	38	8	14	6	172	19.8
O15270	SPTLC2	Serine pa	14	7	11	7	562	62.9
M0QXM4	SLC1A5	Amino aci	17	5	11	5	365	39.4
O94903	PLPBP	Pyridoxal	25	7	10	7	275	30.3
O14976	GAK	Cyclin-G-	7	8	9	8	1311	143.1
Q9BTT0	ANP32E	Acidic le	18	4	7	4	268	30.7
P19784	CSNK2A2	Casein ki	21	7	10	7	350	41.2
H7BXY3	DHX30	Putative	8	9	9	9	1166	130.5
Q9BVJ6	UTP14A	U3 small	13	9	11	9	771	87.9
P29353	SHC1	SHC-trans	16	7	9	7	583	62.8
Q9BVG4	PBDC1	Protein P	33	7	9	7	233	26
O43293	DAPK3	Death-ass	16	6	8	6	454	52.5
P28288	ABCD3	ATP-bindi	12	8	12	8	659	75.4
A0A286YFF	MON2	Protein M	5	8	9	8	1718	190.4
A0A1W2PPI	POLR2B	DNA-direc	8	9	10	9	1099	125.1
Q5SQP8	CTBP2	C-termina	17	9	13	5	513	56.1
A6PVN5	PTPA	Serine/th	28	9	14	9	329	37.4
Q13363	CTBP1	C-termina	19	8	13	4	440	47.5
P43897	TSFM	Elongatic	20	6	10	6	325	35.4
Q9HAV7	GRPEL1	GrpE prot	28	6	11	6	217	24.3
A0A096LNZ	ISG15	Ubiquitin	48	6	9	6	143	15.6
Q15386	UBE3C	Ubiquitin	8	9	9	9	1083	123.8
Q9Y6D6	ARFGEF1	Brefeldin	5	8	9	3	1849	208.6
O15294	OGT	UDP-N-ace	11	9	9	9	1046	116.9
O60504	SORBS3	Vinexin O	15	9	10	9	671	75.3
P56199	ITGA1	Integrin	8	10	11	10	1179	130.8
Q12769	NUP160	Nuclear p	6	8	8	8	1436	162
Q9H307	PNN	Pinin OS=	12	10	10	10	717	81.6
H7C3P7	RALA	Ras-relat	29	4	9	3	164	18.4
A0A0A0MQX	MBNL1	Musclebli	19	6	12	1	400	43

Q5F2F8	PPP3CB	Serine/th	16	7	9	2	496	56
P42766	RPL35	60S ribos	46	7	20	7	123	14.5
O00264	PGRMC1	Membrane-	25	8	13	7	195	21.7
P21810	BGN	Biglycan	20	6	8	5	368	41.6
Q08623	PUDP	Pseudouri	26	5	8	5	228	25.2
O75695	RP2	Protein X	18	8	12	8	350	39.6
P24539	ATP5F1	ATP synth	26	8	11	8	256	28.9
Q9NW15	ANO10	Anoctamin	9	6	10	6	660	76.3
P61513	RPL37A	60S ribos	59	5	15	5	92	10.3
Q9BZG1	RAB34	Ras-relat	25	6	8	6	259	29
P07099	EPHX1	Epoxide h	20	9	12	9	455	52.9
Q96DZ1	ERLEC1	Endoplasm	21	11	13	11	483	54.8
P52294	KPNA1	Importin	17	9	12	5	538	60.2
Q9H9J2	MRPL44	39S ribos	24	7	9	7	332	37.5
Q9NR31	SAR1A	GTP-bindi	29	7	16	4	198	22.4
Q52LJ0	FAM98B	Protein F	22	6	7	6	330	37.2
Q9P2R3	ANKFY1	Rabankyri	7	7	8	7	1169	128.3
A0A140TA6	KRT34	Keratin,	18	8	9	8	436	49.4
O00186	STXBP3	Syntaxin-	11	7	11	7	592	67.7
Q8TD19	NEK9	Serine/th	8	7	8	7	979	107.1
P29590	PML	Protein F	12	10	11	10	882	97.5
P35658	NUP214	Nuclear p	5	9	10	9	2090	213.5
Q7Z4V5	HDGFL2	Hepatoma-	10	7	13	6	671	74.3
P61764	STXBP1	Syntaxin-	15	7	9	7	594	67.5
Q00653	NFKB2	Nuclear f	11	10	10	10	900	96.7
A0A2R8Y5Q	TBCE	Tubulin-s	17	9	9	9	549	61.8
Q9Y4C2	TCAF1	TRPM8 cha	8	7	9	7	921	102.1
Q01968	OCRL	Inositol	11	9	9	9	901	104.1
P49207	RPL34	60S ribos	42	6	30	6	117	13.3
Q96EP5	DAZAP1	DAZ-assoc	20	6	9	6	407	43.4
Q5T6V5	C9orf64	Queuosine	19	6	10	6	341	39
Q13243	SRSF5	Serine/ar	22	6	12	5	272	31.2
Q7L5N7	LPCAT2	Lysophosp	13	8	10	8	544	60.2
P06493	CDK1	Cyclin-de	33	8	9	6	297	34.1
D6RBW1	EIF4E	Eukaryoti	24	6	12	6	245	28.5
G3V4T7	FRMD6	FERM doma	16	7	8	7	545	62.8
E9PHV5	SSFA2	Sperm-spe	8	9	10	9	1237	136.1
Q53H82	LACTB2	Endoribon	31	7	7	7	288	32.8
O75962	TRIO	Triple fu	3	9	10	9	3097	346.7
A0A2R8Y85	CUX1	Homeobox	6	9	10	2	1460	159.3
A0A2R8Y5A	ATXN2	Ataxin-2	8	9	10	8	1166	125.9
P46013	MKI67	Prolifera	4	8	8	8	3256	358.5
P35625	TIMP3	Metallopr	32	7	17	7	211	24.1
O14562	UBFD1	Ubiquitin	21	6	11	6	309	33.4
P48723	HSPA13	Heat shoc	17	8	10	8	471	51.9
Q14699	RFTN1	Raftlin C	18	10	10	10	578	63.1
Q15185	PTGES3	Prostagla	39	6	18	6	160	18.7
P60903	S100A10	Protein S	28	4	22	4	97	11.2
P62854	RPS26	40S ribos	37	4	14	4	115	13
Q9Y3D6	FIS1	Mitochond	29	5	10	5	152	16.9
Q96S52	PIGS	GPI trans	14	7	9	7	555	61.6

Q9H3N1	TMX1	Thioredox	22	7	12	7	280	31.8
O95630	STAMBP	STAM-binc	20	8	9	8	424	48
O75934	BCAS2	Pre-mRNA-	34	7	8	7	225	26.1
Q14353	GAMT	Guanidinc	31	5	7	5	236	26.3
P58546	MTPN	Myotrophi	57	5	9	5	118	12.9
Q92783	STAM	Signal tr	17	8	9	8	540	59.1
O94875	SORBS2	Sorbin ar	9	7	9	7	1100	124
J3QRU1	YES1	Tyrosine-	15	7	9	5	548	61.3
P16435	POR	NADPH--cy	12	7	11	7	677	76.6
P82094	TMF1	TATA elem	9	8	8	8	1093	122.8
Q15836	VAMP3	Vesicle-a	40	3	9	1	100	11.3
Q9Y2L1	DIS3	Exosome c	10	9	10	9	958	108.9
Q13867	BLMH	Bleomycir	16	6	8	6	455	52.5
Q99584	S100A13	Protein S	48	5	14	5	98	11.5
Q969S3	ZNF622	Zinc fing	21	9	10	9	477	54.2
A0A087WVIPDLIM2		PDZ and L	21	6	10	6	325	34.7
P61758	VBP1	Prefoldir	37	7	14	7	197	22.6
H0YL72	IDH3A	Isocitrat	17	6	10	6	331	35.8
P27105	STOM	Erythrocy	28	7	8	7	288	31.7
O95218	ZRANB2	Zinc fing	20	6	10	6	330	37.4
P68871	HBB	Hemoglobi	38	5	15	5	147	16
A0A087WYRSRP19		Signal re	44	4	7	4	120	13.6
Q9NTI5	PDS5B	Sister ch	6	8	9	7	1447	164.6
Q9Y2V7	COG6	Conservec	14	9	10	9	657	73.2
P09012	SNRPA	U1 small	33	7	9	6	282	31.3
Q9UBU9	NXF1	Nuclear R	10	6	8	6	619	70.1
E9PR44	CRYAB	Alpha-cry	30	5	16	5	174	20
P54725	RAD23A	UV excisi	19	7	12	5	363	39.6
Q99848	EBNA1BP2	Probable	23	8	11	8	306	34.8
Q05209	PTPN12	Tyrosine-	11	8	8	8	780	88.1
Q8N684	CPSF7	Cleavage	19	8	11	8	471	52
Q6UWE0	LRSAM1	E3 ubiqui	14	9	9	9	723	83.5
P46108	CRK	Adapter m	25	6	8	6	304	33.8
Q9BWS9	CHID1	Chitinase	16	5	7	5	393	44.9
P47914	RPL29	60S ribos	24	5	18	5	159	17.7
Q8TC12	RDH11	Retinol d	23	7	8	7	318	35.4
P42025	ACTR1B	Beta-cent	22	8	10	2	376	42.3
P21912	SDHB	Succinate	28	7	12	7	280	31.6
Q96M27	PRRC1	Protein F	14	6	11	6	445	46.7
P60763	RAC3	Ras-relat	27	5	16	1	192	21.4
O43818	RRP9	U3 small	20	9	10	9	475	51.8
Q96EM0	L3HYPDH	Trans-3-h	27	7	8	7	354	38.1
P22090	RPS4Y1	40S ribos	21	7	12	2	263	29.4
P26440	IVD	Isovalery	19	8	9	8	423	46.3
Q96D46	NMD3	60S ribos	17	8	9	8	503	57.6
Q9UBM7	DHCR7	7-dehydrc	14	6	11	6	475	54.5
Q15036	SNX17	Sorting r	14	7	8	7	470	52.9
K7ELP0	TPM4	Tropomyos	57	6	17	1	69	8
Q8IWU6	SULF1	Extracell	10	9	11	9	871	101
Q8TBA6	GOLGA5	Golgin su	13	9	9	9	731	83
P48507	GCLM	Glutamate	27	6	7	6	274	30.7

P23919	DTYMK	Thymidyla	27	6	10	6	212	23.8
Q16204	CCDC6	Coiled-cc	19	8	9	8	474	53.3
P18077	RPL35A	60S ribos	52	7	19	7	110	12.5
P29218	IMPA1	Inositol	25	7	8	7	277	30.2
Q96C86	DCPS	m7GpppX d	30	8	10	8	337	38.6
C9J381	IMPDH1	Inosine-5	15	7	7	5	513	55.2
P50402	EMD	Emerin OS	23	7	10	7	254	29
P11234	RALB	Ras-relat	25	4	7	3	206	23.4
Q9BRX2	PELO	Protein p	21	7	10	7	385	43.3
O75663	TIPRL	TIP41-lik	28	8	14	8	272	31.4
J3QRU4	VAMP2	Vesicle-a	35	3	7	1	113	12.2
O14745	SLC9A3R1	Na(+)/H(+	27	8	10	8	358	38.8
O60925	PFDN1	Prefoldir	42	7	12	7	122	14.2
O95361	TRIM16	Tripartit	20	9	10	9	564	63.9
P51570	GALK1	Galactoki	19	6	10	6	392	42.2
P36551	CPOX	Oxygen-de	17	6	9	6	454	50.1
Q92820	GGH	Gamma-glu	18	7	9	7	318	35.9
Q04837	SSBP1	Single-st	44	5	7	5	148	17.2
P49720	PSMB3	Proteasom	35	5	10	5	205	22.9
Q5TFE4	NT5DC1	5'-nuclec	18	8	10	8	455	51.8
P33240	CSTF2	Cleavage	16	8	10	3	577	60.9
Q15369	ELOC	Elongin-C	47	4	7	4	112	12.5
P24666	ACP1	Low molec	53	5	9	5	158	18
H7C4T5	MBNL1	Musclebli	24	6	11	1	329	35.8
Q96CN7	ISOC1	Isochoris	28	6	6	6	298	32.2
O75935	DCTN3	Dynactin	32	8	12	8	186	21.1
Q9Y2U8	LEMD3	Inner nuc	8	7	9	7	911	99.9
P82933	MRPS9	28S ribos	22	7	7	7	396	45.8
P49888	SULT1E1	Estrogen	27	8	11	8	294	35.1
D6RA82	ANXA3	Annexin C	23	6	7	6	284	32.1
O43633	CHMP2A	Charged m	34	8	11	8	222	25.1
J3KNI1	COG4	Conservec	11	7	7	7	789	89.4
F5H479	LAMTOR1	Ragulator	49	4	7	4	99	11.1
Q9NRR5	UBQLN4	Ubiquilir	13	6	8	5	601	63.8
P43307	SSR1	Transloco	21	4	11	4	286	32.2
O96008	TOMM40	Mitochonc	22	6	11	6	361	37.9
A0A024R21	HDGFRP3	Hepatoma-	24	5	11	4	203	22.6
B1ALA9	PRPS1	Ribose-ph	29	8	11	5	285	31.4
F8W9X7	CCDC93	Coiled-cc	16	9	9	9	630	73
Q5JTH9	RRP12	RRP12-lik	8	10	10	10	1297	143.6
P04080	CSTB	Cystatin-	77	6	12	6	98	11.1
P32321	DCTD	Deoxycyti	45	6	6	6	178	20
B4DLR8	NQO1	NAD(P)H d	33	6	9	6	202	22.8
Q99707	MTR	Methionir	6	7	8	7	1265	140.4
P98175	RBM10	RNA-bindi	9	8	8	8	930	103.5
Q8NB90	SPATA5	Spermatog	9	6	11	5	893	97.8
P46926	GNPDA1	Glucosami	24	6	8	4	289	32.6
Q6P587	FAHD1	Acylpyruv	38	6	10	6	224	24.8
E9PL57	NEDD8-MDF	NEDD8-MDF	28	5	22	5	170	19.5
A0A1W2PNW	STAT6	Signal tr	10	8	9	8	679	76
P36507	MAP2K2	Dual spec	17	7	11	2	400	44.4

P54619	PRKAG1	5'-AMP-ac	23	7	8	7	331	37.6
Q8IUE6	HIST2H2AE	Histone H	47	5	18	2	130	14
O43896	KIF1C	Kinesin-l	8	8	8	5	1103	122.9
Q8N3D4	EHBP1L1	EH domain	6	8	8	7	1523	161.8
P50416	CPT1A	Carnitine	13	9	10	9	773	88.3
O14908	GIPC1	PDZ domai	18	6	9	6	333	36
G5E9L0	ARFGAP2	ADP-ribos	18	9	10	9	493	53.4
Q08752	PPID	Peptidyl-	20	8	9	7	370	40.7
Q9NWV4	C1orf123	UPF0587 p	41	6	7	6	160	18
Q2NL82	TSR1	Pre-rRNA-	14	8	9	8	804	91.8
Q5VVC8	RPL11	60S ribos	27	5	18	5	167	19
P45973	CBX5	Chromobox	30	5	7	5	191	22.2
Q9NUQ8	ABCF3	ATP-bindi	10	8	11	8	709	79.7
Q96DB5	RMDN1	Regulator	22	6	8	6	314	35.8
Q9Y6W5	WASF2	Wiskott-A	16	8	8	8	498	54.3
O14656	TOR1A	Torsin-1A	18	5	8	5	332	37.8
A0A182DWF	TXNRD2	Thioredox	10	4	8	3	494	53.5
Q9BRA2	TXNDC17	Thioredox	51	5	11	5	123	13.9
Q96DG6	CMBL	Carboxyme	27	7	10	7	245	28
Q9UHY7	ENOPH1	Enolase-p	27	5	9	5	261	28.9
Q14694	USP10	Ubiquitin	8	5	7	5	798	87.1
H0YAN8	ARHGEF10	Rho guani	6	5	8	5	988	109.6
Q9BPW8	NIPSNAP1	Protein N	21	5	10	4	284	33.3
P39748	FEN1	Flap endo	22	7	8	7	380	42.6
Q969X5	ERGIC1	Endoplasm	18	5	11	5	290	32.6
F8WE88	MYO5A	Unconvent	6	10	10	10	1855	215.2
Q8WUH6	TMEM263	Transmemb	49	4	7	4	116	11.7
Q9H4G4	GLIPR2	Golgi-ass	38	5	7	5	154	17.2
P05026	ATP1B1	Sodium/po	25	7	10	7	303	35
O75326	SEMA7A	Semaphori	11	5	7	5	666	74.8
Q8IYI6	EXOC8	Exocyst c	12	9	9	9	725	81.7
Q709C8	VPS13C	Vacuolar	2	7	7	7	3753	422.1
Q8N183	NDUFAF2	NADH dehy	33	6	10	6	169	19.8
O43813	LANCL1	LanC-like	23	8	8	8	399	45.3
P19971	TYMP	Thymidine	18	6	8	6	482	49.9
Q8IX12	CCAR1	Cell divi	7	8	9	8	1150	132.7
Q99598	TSNAX	Translin-	23	6	9	6	290	33.1
Q8NBJ7	SUMF2	Inactive	21	5	8	5	301	33.8
Q92614	MYO18A	Unconvent	5	10	10	10	2054	233
Q9BZV1	UBXN6	UBX domai	18	5	6	5	441	49.7
Q13501	SQSTM1	Sequestos	15	4	8	4	440	47.7
Q68CQ7	GLT8D1	Glycosylt	23	8	9	8	371	41.9
Q9Y4W2	LAS1L	Ribosomal	11	7	8	7	734	83
P49585	PCYT1A	Choline-p	21	8	15	8	367	41.7
Q9Y3T9	NOC2L	Nucleolar	8	6	9	6	749	84.9
Q93034	CUL5	Cullin-5	12	9	10	9	780	90.9
Q13907	IDI1	Isopenten	23	4	6	4	227	26.3
Q8IZ07	ANKRD13A	Ankyrin r	15	6	6	6	590	67.6
Q86Y56	DNAAF5	Dynein as	13	9	9	9	855	93.5
Q9NS69	TOMM22	Mitochond	34	4	6	4	142	15.5
P35613	BSG	Basigin C	14	6	17	6	385	42.2

Q9C0C9	UBE2O	(E3-indep	7	7	7	7	1292	141.2
D6RBR1	CAST	Calpastat	50	6	10	1	150	15.5
B0QZ18	CPNE1	Copine-1	10	6	12	6	542	59.7
Q8TED1	GPX8	Probable	31	7	11	7	209	23.9
Q9UHY1	NRBP1	Nuclear r	14	6	7	6	535	59.8
Q15645	TRIP13	Pachytene	20	8	9	8	432	48.5
O95155	UBE4B	Ubiquitin	7	8	8	8	1302	146.1
Q6UVK1	CSPG4	Chondroit	4	6	6	6	2322	250.4
Q8TCT9	HM13	Minor his	16	6	15	6	377	41.5
P01023	A2M	Alpha-2-m	4	6	15	5	1474	163.2
Q9NZL4	HSPBP1	Hsp70-bin	19	7	9	7	362	39.4
Q9UKV8	AGO2	Protein a	12	9	9	9	859	97.1
A0A2Q3DPC	ARMC9	LisH doma	14	9	10	9	665	75.5
O60507	TPST1	Protein-t	20	6	7	6	370	42.2
P09496	CLTA	Clathrin	22	8	18	8	248	27.1
P37108	SRP14	Signal re	54	6	16	6	136	14.6
Q9UJ41	RABGEF1	Rab5 GDP/	11	6	6	6	708	79.3
A1X283	SH3PXD2B	SH3 and F	9	8	9	6	911	101.5
P20645	M6PR	Cation-de	16	5	8	5	277	31
Q13247	SRSF6	Serine/ar	24	10	14	6	344	39.6
H0Y368	DPM1	Dolichol-	16	4	9	4	295	33.3
A0A0D9SEM	SRSF4	Serine/ar	20	8	13	4	378	43.6
P48307	TFPI2	Tissue fa	35	6	7	6	235	26.9
P67870	CSNK2B	Casein ki	26	4	9	4	215	24.9
Q14126	DSG2	Desmoglei	8	6	8	6	1118	122.2
Q14320	FAM50A	Protein F	24	7	9	7	339	40.2
Q8N668	COMMD1	COMM doma	23	4	6	4	190	21.2
Q9BWF3	RBM4	RNA-bindi	23	8	10	8	364	40.3
E9PDP5	ANKHD1	Ankyrin r	5	6	6	2	1565	166.6
Q9UKG1	APPL1	DCC-inter	12	7	7	7	709	79.6
P23497	SP100	Nuclear a	7	7	19	4	879	100.4
A0A2U3TZL	CD59	CD59 glyc	27	4	17	4	120	13.3
A0A0B4J2D	GATD3B	Glutamine	23	6	9	6	268	28.1
Q08431	MFGE8	Lactadher	19	7	8	7	387	43.1
Q96EK6	GNPNAT1	Glucosami	32	4	9	4	184	20.7
O00566	MPHOSPH1C	U3 small	11	9	10	9	681	78.8
Q9NRF8	CTPS2	CTP synth	11	6	8	3	586	65.6
A0A087X0W	RELA	Transcrip	17	6	8	6	448	49.5
Q96J02	ITCH	E3 ubiqui	10	7	7	7	903	102.7
O75306	NDUFS2	NADH dehy	22	7	9	7	463	52.5
Q2M2I8	AAK1	AP2-assoc	11	8	8	7	961	103.8
A0A087WXE	SCAMP1	Secretory	13	4	6	4	312	35
E9PNM1	FDFT1	Squalene	20	6	6	6	410	47.3
F6Y5H0	RBMS1	RNA-bindi	14	5	7	1	386	41.9
P20674	COX5A	Cytochrom	37	5	10	5	150	16.8
Q9P035	HACD3	Very-long	22	7	11	7	362	43.1
O95834	EML2	Echinoder	9	6	7	6	649	70.6
P54709	ATP1B3	Sodium/po	21	7	11	7	279	31.5
Q9UKM7	MAN1B1	Endoplasm	12	7	9	7	699	79.5
P14927	UQCRB	Cytochrom	50	6	8	6	111	13.5
P13716	ALAD	Delta-ami	20	5	6	5	330	36.3

Q9ULD0	OGDHL	2-oxoglut	6	6	8	1	1010	114.4
P0DN79	CBSL	Cystathic	18	8	8	8	551	60.5
J3KQN4	RPL36A	60S ribos	24	6	20	2	142	16.4
Q8TEQ6	GEMIN5	Gem-assoc	6	9	10	9	1508	168.5
Q8NEZ2	VPS37A	Vacuolar	16	5	5	5	397	44.3
Q9HCJ6	VAT1L	Synaptic	20	7	8	7	419	45.9
Q13895	BYSL	Bystin OS	20	8	9	8	437	49.6
P61923	COPZ1	Coatomer	19	3	9	3	177	20.2
P01116	KRAS	GTPase KR	28	4	9	1	189	21.6
A0A0A0MTC	RNF213	E3 ubiqui	2	9	9	8	5256	596.1
B7ZC38	SH3GLB2	Endophili	19	8	8	7	400	44.3
Q96KC8	DNAJC1	DnaJ homc	12	6	7	6	554	63.8
P10599	TXN	Thioredox	40	5	18	5	105	11.7
Q63ZY3	KANK2	KN motif	9	7	7	7	851	91.1
Q13948	CUX1	Protein C	12	8	9	1	678	77.4
Q9Y5K8	ATP6V1D	V-type pr	30	6	9	6	247	28.2
A0A1B0GTL	RAB11FIP5	Rab11 fam	6	7	8	7	1324	138.3
Q86XL3	ANKLE2	Ankyrin r	9	7	8	7	938	104.1
P31751	AKT2	RAC-beta	18	8	8	4	481	55.7
Q16799	RTN1	Reticulon	13	7	9	7	776	83.6
Q10472	GALNT1	Polypepti	13	7	13	7	559	64.2
Q96QR8	PURB	Transcrip	21	8	9	8	312	33.2
O75688	PPM1B	Protein p	14	6	7	5	479	52.6
P61026	RAB10	Ras-relat	30	8	16	6	200	22.5
P04222	HLA-C	HLA class	22	6	8	1	366	40.8
H0YNE9	RAB8B	Ras-relat	31	6	11	2	188	21.9
P62269	RPS18	40S ribos	33	6	18	6	152	17.7
O95140	MFN2	Mitofusin	11	7	7	6	757	86.3
Q8TAE8	GADD45GIP	Growth ar	40	7	8	7	222	25.4
Q9Y2H1	STK38L	Serine/th	11	5	6	4	464	54
E7ERK9	EIF2B4	Translati	19	7	7	7	544	59.7
A0A087WVF	PDE4DIP	Myomegali	3	8	9	8	2240	253.9
Q9H6T3	RPAP3	RNA polym	12	7	8	7	665	75.7
Q9ULZ3	PYCARD	Apoptosis	26	4	5	4	195	21.6
Q9Y6E0	STK24	Serine/th	17	6	6	3	443	49.3
E7EPT4	NDUFV2	NADH dehy	31	8	10	8	252	27.9
A0A0A0MRM	NOLC1	Nucleolar	12	10	12	10	708	74.6
Q9BRP8	PYM1	Partner c	43	5	7	5	204	22.6
K7EMQ3	DNM2	Dynamin-2	23	8	10	1	289	33.7
P49959	MRE11	Double-st	8	6	8	6	708	80.5
P48426	PIP4K2A	Phosphati	18	8	9	4	406	46.2
Q9P2B4	CTTNBP2NL	CTTNBP2 N	10	7	9	7	639	70.1
Q9H269	VPS16	Vacuolar	7	5	6	5	839	94.6
C9JRZ6	CHCHD3	MICOS com	30	8	10	8	232	26.7
O75691	UTP20	Small sub	3	9	9	9	2785	318.2
H0YJ75	PPP2R5C	Serine/th	12	6	8	3	553	64
Q14644	RASA3	Ras GTPas	11	7	7	7	834	95.6
Q8NCC3	PLA2G15	Group XV	16	5	6	5	412	46.6
Q9NZZ3	CHMP5	Charged m	32	5	12	5	219	24.6
P57740	NUP107	Nuclear p	7	5	7	5	925	106.3
O95864	FADS2	Fatty aci	14	6	10	5	444	52.2

A0A0G2JPF	SCRIB	Protein s	4	8	9	8	1655	177.6
O14561	NDUFAB1	Acyl carr	19	4	11	4	156	17.4
A0A0A0MT6	FKBP15	Peptidylp	7	7	7	7	1244	136.2
Q9NQ48	LZTFL1	Leucine z	19	5	6	5	299	34.6
P54802	NAGLU	Alpha-N-a	14	7	8	7	743	82.2
P62899	RPL31	60S ribos	42	6	16	6	125	14.5
Q15654	TRIP6	Thyroid r	16	6	10	6	476	50.3
F5GZ97	WASHC3	WASH comp	32	5	7	5	193	21
P52594	AGFG1	Arf-GAP d	14	7	7	7	562	58.2
B4E3T4	RBPMS	HCG204342	37	6	6	6	224	25
O75964	ATP5L	ATP synth	39	4	12	4	103	11.4
P17677	GAP43	Neuromodu	40	5	5	5	238	24.8
Q9NSC5	HOMER3	Homer pro	24	7	7	7	361	39.8
E9PNP3	AAMDC	Mth938 do	47	7	8	7	141	15.6
P46937	YAP1	Transcrip	19	6	7	6	504	54.4
Q96C90	PPP1R14B	Protein p	31	3	6	2	147	15.9
Q9NS86	LANCL2	LanC-like	11	4	6	4	450	50.8
O75340	PDCD6	Programme	28	5	8	5	191	21.9
E7EVX8	PRPF31	U4/U6 sma	15	7	9	7	493	54.7
P11166	SLC2A1	Solute ca	7	4	7	4	492	54
P82650	MRPS22	28S ribos	22	7	7	7	360	41.3
Q02539	HIST1H1A	Histone H	24	5	16	2	215	21.8
P09601	HMOX1	Heme oxyg	23	5	6	5	288	32.8
Q9HAV4	XPO5	Exportin-	7	8	9	8	1204	136.2
P51665	PSMD7	26S prote	16	6	9	6	324	37
H3BLV9	SRPK1	SRSF prot	9	6	7	5	671	76
Q6N069	NAA16	N-alpha-a	6	6	9	1	864	101.4
Q6YP21	KYAT3	Kynurenin	17	7	8	7	454	51.4
Q9GZR7	DDX24	ATP-depen	7	5	5	5	859	96.3
Q04760	GLO1	Lactoylgl	28	6	10	6	184	20.8
D3DTX6	PPP1R9B	Neurabin-	8	7	8	7	817	89.3
O60784	TOM1	Target of	10	4	5	4	492	53.8
Q15397	PUM3	Pumilio h	11	7	9	7	648	73.5
Q99538	LGMN	Legumain	10	4	9	4	433	49.4
P41223	BUD31	Protein B	47	8	8	8	144	17
Q9BV57	ADI1	1,2-dihyd	51	8	8	8	179	21.5
Q8NFQ8	TOR1AIP2	Torsin-1A	17	7	8	6	470	51.2
Q00796	SORD	Sorbitol	20	7	9	7	357	38.3
P84103	SRSF3	Serine/ar	28	6	15	5	164	19.3
Q9BYJ9	YTHDF1	YTH domai	12	7	9	2	559	60.8
Q9HAU4	SMURF2	E3 ubiqui	12	8	8	8	748	86.1
Q8WTT2	NOC3L	Nucleolar	9	9	9	9	800	92.5
Q01650	SLC7A5	Large neu	8	4	6	4	507	55
B4DR61	SEC61A1	cDNA FLJ5	12	6	15	6	482	52.9
A0A0C4DFM	TM9SF4	Transmemb	12	6	9	6	625	72.5
P18754	RCC1	Regulator	18	5	6	5	421	44.9
Q9NUY8	TBC1D23	TBC1 doma	14	7	7	7	699	78.3
A0A0A0MTL	RIPOR1	Rho famil	6	8	8	8	1238	134
Q92572	AP3S1	AP-3 comp	38	6	7	6	193	21.7
Q99575	POP1	Ribonucle	7	7	8	7	1024	114.6
Q03154	ACY1	Aminoacyl	21	6	9	6	408	45.9

Q6GMV3	PTRHD1	Putative	31	5	8	5	140	15.8
Q9BYD2	MRPL9	39S ribos	23	6	9	6	267	30.2
Q96T76	MMS19	MMS19 nuc	9	7	7	7	1030	113.2
F1T0I1	SEC16A	Protein t	4	9	9	9	2334	249.3
Q03701	CEBPZ	CCAAT/enh	8	8	9	8	1054	120.9
Q8IZ52	CHPF	Chondroit	9	6	8	6	775	85.4
H7C2T5	POFUT2	GDP-fucos	26	8	13	1	265	30.4
Q9BZH6	WDR11	WD repeat	7	8	10	8	1224	136.6
B4E0K5	MAPK14	Mitogen-a	25	6	6	6	283	32.3
Q9GZS3	WDR61	WD repeat	25	5	7	5	305	33.6
Q9BYX2	TBC1D2	TBC1 doma	7	5	6	5	928	105.3
Q86X55	CARM1	Histone-a	9	6	8	6	608	65.8
F6U1T9	PPP3R1	Calcineur	33	4	5	4	160	18.2
P27694	RPA1	Replicati	13	7	7	7	616	68.1
A0A087WYF	PDLIM3	PDZ and L	21	4	7	4	276	30.1
H0YEH1	PICALM	Phosphati	19	4	5	1	308	32.2
B8ZZA8	GLS	Glutamina	25	4	9	3	169	18.6
F5H5I6	GRSF1	G-rich se	16	6	8	6	424	47.9
P49458	SRP9	Signal re	50	5	11	5	86	10.1
Q9NUJ1	ABHD10	Mycopheno	22	5	6	5	306	33.9
O76024	WFS1	Wolframin	9	8	9	8	890	100.2
Q9Y4X5	ARIH1	E3 ubiqui	11	6	9	6	557	64.1
Q9H8H0	NOL11	Nucleolar	10	7	9	7	719	81.1
Q06203	PPAT	Amidophos	14	6	6	6	517	57.4
Q6FI81	CIAPIN1	Anamorsin	19	7	11	7	312	33.6
P62081	RPS7	40S ribos	26	6	15	2	194	22.1
Q96K17	BTF3L4	Transcrip	37	6	9	4	158	17.3
E9PKP7	UBTF	Nucleolar	11	8	8	8	745	87.4
Q99653	CHP1	Calcineur	31	6	7	6	195	22.4
Q99470	SDF2	Stromal c	25	5	9	5	211	23
Q9Y3I1	FBXO7	F-box onl	13	6	7	6	522	58.5
Q13228	SELENBP1	Methaneth	12	5	7	5	472	52.4
P51648	ALDH3A2	Fatty ald	18	7	7	7	485	54.8
Q9UNX4	WDR3	WD repeat	6	6	6	6	943	106
Q9H0S4	DDX47	Probable	15	6	7	6	455	50.6
Q9H9A6	LRRC40	Leucine-r	12	7	8	7	602	68.2
Q3KQU3	MAP7D1	MAP7 doma	9	7	10	7	841	92.8
Q9Y6D5	ARFGEF2	Brefeldin	5	9	9	4	1785	201.9
O43353	RIPK2	Receptor-	11	5	6	4	540	61.2
O00462	MANBA	Beta-mann	7	6	7	6	879	100.8
O00743	PPP6C	Serine/th	21	7	10	7	305	35.1
Q8NF37	LPCAT1	Lysophosp	9	5	7	5	534	59.1
P49916	LIG3	DNA ligas	8	8	8	8	1009	112.8
Q9NSK0	KLC4	Kinesin l	8	5	7	1	619	68.6
O15254	ACOX3	Peroxisom	6	3	6	3	700	77.6
Q96GQ7	DDX27	Probable	9	9	9	9	796	89.8
Q16537	PPP2R5E	Serine/th	11	6	9	6	467	54.7
Q9Y243	AKT3	RAC-gamma	18	8	8	3	479	55.7
Q9UBR2	CTSZ	Cathepsin	16	6	9	6	303	33.8
Q15382	RHEB	GTP-bindi	28	6	7	6	184	20.5
P01584	IL1B	Interleuk	21	5	8	5	269	30.7

P49841	GSK3B	Glycogen	14	4	6	2	420	46.7
Q9BVL2	NUP58	Nucleopor	12	6	8	6	599	60.9
P35270	SPR	Sepiapter	30	6	7	6	261	28
Q8IZ83	ALDH16A1	Aldehyde	8	5	6	5	802	85.1
H0Y990	ABCE1	ATP-bindi	30	4	10	1	147	16.2
Q9UI12	ATP6V1H	V-type pr	15	6	6	6	483	55.8
P35813	PPM1A	Protein p	22	7	7	6	382	42.4
Q05086	UBE3A	Ubiquitir	9	6	6	6	875	100.6
P09488	GSTM1	Glutathic	26	7	10	1	218	25.7
Q9UMS0	NFU1	NFU1 iror	26	5	7	5	254	28.4
P48634	PRRC2A	Protein F	3	7	7	6	2157	228.7
Q9BRJ6	C7orf50	Uncharact	42	6	7	6	194	22.1
A6NHR9	SMCHD1	Structura	4	8	9	7	2005	226.2
F8VU90	FKBP11	Peptidylp	26	4	10	4	182	19.8
O60231	DHX16	Pre-mRNA-	8	8	9	7	1041	119.2
Q86TI2	DPP9	Dipeptidy	7	6	8	6	863	98.2
P51531	SMARCA2	Probable	5	7	7	2	1590	181.2
O00193	SMAP	Small aci	30	4	8	4	183	20.3
O75794	CDC123	Cell divi	19	8	9	8	336	39.1
Q7L0Y3	TRMT10C	tRNA meth	21	7	8	7	403	47.3
P39060	COL18A1	Collagen	4	6	6	6	1754	178.1
O95163	ELP1	Elongator	6	6	6	6	1332	150.2
H3BMM9	RNPS1	RNA-bindi	22	4	6	4	284	31.7
P07741	APRT	Adenine p	32	5	12	5	180	19.6
P41208	CETN2	Centrin-2	27	4	6	4	172	19.7
Q9P0V9	SEPT10	Septin-1C	12	4	6	3	454	52.6
Q9NZJ4	SACS	Sacsin OS	2	8	8	8	4579	520.8
P51571	SSR4	Transloco	25	4	8	4	173	19
Q9NV70	EXOC1	Exocyst c	9	9	9	9	894	101.9
Q15208	STK38	Serine/th	12	5	6	4	465	54.2
Q9NRY4	ARHGAP35	Rho GTPas	7	9	9	9	1499	170.4
Q2TAY7	SMU1	WD40 repe	13	7	8	7	513	57.5
Q9NRX4	PHPT1	14 kDa ph	48	6	8	6	125	13.8
Q9NXF1	TEX10	Testis-ex	8	6	6	6	929	105.6
Q14558	PRPSAP1	Phosphori	15	5	7	3	356	39.4
Q7KZ85	SUPT6H	Transcrip	5	7	7	7	1726	198.9
Q9Y3Z3	SAMHD1	Deoxynucl	11	6	7	6	626	72.2
A0A0U1RRK	MICU1	Calcium u	15	7	10	7	480	54.9
P61970	NUTF2	Nuclear t	35	4	11	4	127	14.5
Q9Y320	TMX2	Thioredox	22	5	7	5	296	34
O95833	CLIC3	Chloride	25	5	7	5	236	26.6
Q15067	ACOX1	Peroxisom	12	7	8	7	660	74.4
Q9H173	SIL1	Nucleotic	14	5	7	5	461	52.1
J3KQ48	PTRH2	Peptidyl-	43	5	7	5	180	19.3
E7EQI7	WASHC5	WASH comp	6	6	6	6	1011	117
Q8N5K1	CISD2	CDGSH irc	45	5	10	5	135	15.3
Q9NVH1	DNAJC11	DnaJ homc	12	7	7	7	559	63.2
A0A087X0K	CAB39	Calcium-b	19	7	8	5	339	39.4
P53634	CTSC	Dipeptidy	13	4	5	4	463	51.8
P20337	RAB3B	Ras-relat	26	5	7	2	219	24.7
P49750	YLPM1	YLP motif	3	6	7	6	2146	241.5

H0Y742	SUN1	SUN domai	10	7	7	6	710	79.5
Q969Q0	RPL36AL	60S ribos	32	6	17	2	106	12.5
Q8IXI1	RHOT2	Mitochond	10	4	5	2	618	68.1
Q8IZL8	PELP1	Proline-,	8	6	6	6	1130	119.6
O15042	U2SURP	U2 snRNP-	9	9	9	9	1029	118.2
Q9NQ29	LUC7L	Putative	12	4	8	2	371	43.7
O15056	SYNJ2	Synaptoja	4	5	5	5	1496	165.4
Q9BRK3	MXRA8	Matrix re	13	6	6	6	442	49.1
Q00059	TFAM	Transcrip	26	6	6	6	246	29.1
P49006	MARCKSL1	MARCKS-re	19	3	10	3	195	19.5
M0QXF9	BCAT2	Branched-	17	6	8	6	445	49.9
O95716	RAB3D	Ras-relat	24	5	6	2	219	24.3
P19174	PLCG1	1-phospha	7	9	11	9	1290	148.4
J3KRC4	NT5C	5’(3’)-de	26	5	7	5	182	20.4
Q9BXK5	BCL2L13	Bcl-2-lik	16	5	5	5	485	52.7
Q16774	GUK1	Guanylate	27	5	5	5	197	21.7
Q96JG6	VPS50	Syndetin	6	6	7	6	964	111.1
P13984	GTF2F2	General t	28	6	6	6	249	28.4
Q99447	PCYT2	Ethanolam	17	7	8	7	389	43.8
P36915	GNL1	Guanine n	13	8	8	8	607	68.6
O43447	PPIH	Peptidyl-	33	7	11	6	177	19.2
J3KS05	CBX1	Chromobox	20	3	6	2	173	20
O00754	MAN2B1	Lysosomal	7	7	8	7	1011	113.7
Q12959	DLG1	Disks lar	7	6	7	6	904	100.4
P09619	PDGFRB	Platelet-	6	7	8	5	1106	123.9
Q15286	RAB35	Ras-relat	23	6	10	4	201	23
A0A087WZLPHLDB1		Pleckstri	9	6	7	1	641	73.1
Q15102	PAFAH1B3	Platelet-	25	6	8	6	231	25.7
Q8WUW1	BRK1	Protein B	52	4	9	4	75	8.7
P50583	NUDT2	Bis(5’-nu	39	5	7	5	147	16.8
P23743	DGKA	Diacylgly	10	6	8	6	735	82.6
Q9Y3P9	RABGAP1	Rab GTPas	6	7	7	6	1069	121.7
P09417	QDPR	Dihydropt	23	5	6	5	244	25.8
Q9H4L5	OSBPL3	Oxysterol	9	6	8	6	887	101.2
Q8IZP0	ABI1	Abl inter	11	5	7	3	508	55
B5MC98	PREB	Prolactin	22	4	4	4	359	38.9
Q9H089	LSG1	Large sub	10	6	6	6	658	75.2
P22033	MUT	Methylmal	12	8	8	8	750	83.1
Q07812	BAX	Apoptosis	20	3	6	3	192	21.2
P55769	SNU13	NHP2-like	34	4	10	4	128	14.2
Q9UDR5	AASS	Alpha-ami	7	7	7	7	926	102.1
Q6P1N0	CC2D1A	Coiled-cc	7	6	6	6	951	104
P60510	PPP4C	Serine/th	11	3	5	2	307	35.1
P61225	RAP2B	Ras-relat	31	6	8	4	183	20.5
Q8WYA6	CTNNBL1	Beta-cate	12	7	7	7	563	65.1
Q969G5	CAVIN3	Caveolae-	20	4	8	4	261	27.7
Q8TB61	SLC35B2	Adenosine	15	6	10	6	432	47.5
Q9NYH9	UTP6	U3 small	9	6	7	6	597	70.1
Q9H1I8	ASCC2	Activatir	11	6	6	6	757	86.3
P11182	DBT	Lipoamide	10	5	6	5	482	53.5
E7EPM6	ACSL1	Long-chai	12	8	8	8	664	74.2

Q7Z3B4	NUP54	Nucleopor	15	7	7	7	507	55.4
Q9UEY8	ADD3	Gamma-add	9	6	7	6	706	79.1
P24928	POLR2A	DNA-direc	3	5	6	5	1970	217
E9PMR6	ARHGEF12	Rho guani	5	5	6	5	1441	161.8
Q9H993	ARMT1	Protein-g	12	5	7	5	441	51.1
P02787	TF	Serotrans	4	2	7	2	698	77
P49593	PPM1F	Protein p	14	5	7	5	454	49.8
Q8NCW5	NAXE	NAD(P)H-h	19	4	5	4	288	31.7
Q92797	SYMPK	Symplekin	7	7	9	7	1274	141.1
F5H345	HMBS	Porphobil	21	7	7	7	330	35.7
A0A2R8Y62	RPS7	40S ribos	25	5	15	1	169	19.4
Q6P4E1	CASC4	Protein C	15	6	7	6	433	48.8
P14678	SNRPB	Small nuc	18	4	9	4	240	24.6
Q9UKX7	NUP50	Nuclear p	14	5	6	5	468	50.1
Q96AX1	VPS33A	Vacuolar	11	7	8	7	596	67.6
A0A087X26	TARDBP	TAR DNA-b	26	5	8	5	301	34.2
Q8TBX8	PIP4K2C	Phosphati	13	7	8	6	421	47.3
Q96S97	MYADM	Myeloid-a	16	3	5	3	322	35.3
Q9Y639	NPTN	Neuroplas	12	4	6	4	398	44.4
H0YC42		Uncharact	18	4	5	2	278	31.2
Q9UMX5	NENF	Neudesin	26	4	5	4	172	18.8
Q9NZJ9	NUDT4	Diphospho	32	4	5	3	180	20.3
Q5TEC6	HIST2H3PS	Histone H	27	5	19	3	136	15.4
P83111	LACTB	Serine be	13	6	7	6	547	60.7
Q9Y3L5	RAP2C	Ras-relat	36	6	8	3	183	20.7
S4R3Q9	OXA1L	Mitochond	10	4	6	4	435	48.5
J3KMZ9	LDLR	Low-densi	7	6	7	6	945	104.6
Q8NBL1	POGLUT1	Protein O	14	6	7	6	392	46.2
Q14376	GALE	UDP-gluco	13	4	8	4	348	38.3
Q9UKI2	CDC42EP3	Cdc42 eff	26	5	8	5	254	27.7
Q8NBY1	STK26	Serine/th	17	6	7	3	392	43.8
A8MYT4	PIK3C3	Phosphati	6	4	6	4	824	94.3
A0A2R8Y56	RELCH	RAB11-bin	8	8	8	8	1216	134.5
P21127	CDK11B	Cyclin-de	8	6	7	6	795	92.6
P49902	NT5C2	Cytosolic	10	6	7	6	561	64.9
Q9Y5P6	GMPPB	Mannose-1	14	4	7	4	360	39.8
P42126	ECI1	Enoyl-CoA	13	4	7	4	302	32.8
Q15370	ELOB	Elongin-B	36	6	9	6	118	13.1
Q5TDH0	DDI2	Protein D	13	4	5	4	399	44.5
Q16718	NDUFA5	NADH dehy	43	4	6	4	116	13.5
Q9H270	VPS11	Vacuolar	9	8	8	8	941	107.8
Q8IYB3	SRRM1	Serine/ar	5	4	6	4	904	102.3
Q5SW96	LDLRAP1	Low densi	18	5	6	5	308	33.9
C9JQ41	CCDC58	Coiled-co	46	5	5	5	130	15.3
Q9HC07	TMEM165	Transmemb	13	2	4	2	324	34.9
Q9BQ39	DDX50	ATP-depen	7	6	9	4	737	82.5
O43665	RGS10	Regulator	35	5	7	5	173	20.2
H0YEH2	PUM1	Pumilio h	5	6	7	6	1125	120.2
P14854	COX6B1	Cytochrom	52	4	10	4	86	10.2
Q8TF42	UBASH3B	Ubiquitin	7	5	6	5	649	72.6
Q13523	PRPF4B	Serine/th	6	6	6	6	1007	116.9

E9PG40	APP	Amyloid-b	8	6	7	6	714	80.8
Q9HB40	SCPEP1	Retinoid-	13	5	6	5	452	50.8
O95721	SNAP29	Synaptosc	25	5	6	5	258	29
E7ESD2	WASHC2A	WASH comp	5	5	5	5	1279	140.1
Q13123	IK	Protein R	11	6	7	6	557	65.6
A0A087X1G	SELENOF	Selenopro	20	3	6	3	164	17.9
Q9P0I2	EMC3	ER membra	21	4	6	4	261	29.9
Q16647	PTGIS	Prostacyc	12	4	5	4	500	57.1
Q99567	NUP88	Nuclear p	11	7	8	7	741	83.5
Q7L9L4	MOB1B	MOB kinas	20	4	8	4	216	25.1
Q6UXH1	CRELD2	Cysteine-	15	5	7	5	353	38.2
P13612	ITGA4	Integrin	6	6	7	6	1032	114.8
Q86SQ0	PHLDB2	Pleckstri	5	6	6	6	1253	142.1
Q5T6F2	UBAP2	Ubiquitin	7	6	7	6	1119	117
Q9NQS1	AVEN	Cell deat	25	7	8	7	362	38.5
P02795	MT2A	Metalloth	67	4	8	2	61	6
Q9H0A8	COMMD4	COMM doma	34	6	6	6	199	21.8
Q13151	HNRNPA0	Heterogen	24	6	11	5	305	30.8
Q00765	REEP5	Receptor	15	4	7	4	189	21.5
Q9UHL4	DPP7	Dipeptidy	11	5	8	5	492	54.3
Q9NX62	IMPAD1	Inositol	14	7	9	7	359	38.7
Q9BTE6	AARSD1	Alanyl-tR	22	6	7	6	412	45.5
B4DTF2	ANXA8L1	Annexin C	22	6	6	6	270	30.5
A0A2R8YG4	ARHGEF7	Rho guani	10	7	7	7	862	97.1
Q9NX55	HYPK	Huntingti	31	3	5	3	129	14.7
Q5VW36	FOCAD	Focadhesi	3	5	5	5	1801	199.9
Q9P253	VPS18	Vacuolar	6	5	5	5	973	110.1
O43172	PRPF4	U4/U6 sma	15	6	6	6	522	58.4
P18583	SON	Protein S	3	7	7	7	2426	263.7
A0A2R8YFH	MSH2	DNA misma	8	6	6	6	918	102.8
P61960	UFM1	Ubiquitin	68	4	9	4	85	9.1
Q9Y4P1	ATG4B	Cysteine	13	3	5	3	393	44.3
O60524	NEMF	Nuclear e	6	7	8	7	1076	122.9
Q96EQ0	SGTB	Small glu	11	3	6	3	304	33.4
Q96H20	SNF8	Vacuolar-	23	5	5	5	258	28.8
P00167	CYB5A	Cytochrom	27	3	5	3	134	15.3
Q92879	CELF1	CUGBP Ela	13	6	13	6	486	52
Q9BV38	WDR18	WD repeat	15	6	6	6	432	47.4
O14672	ADAM10	Disintegr	10	6	6	6	748	84.1
P10619	CTSA	Lysosomal	10	5	10	5	480	54.4
P82673	MRPS35	28S ribos	23	4	5	4	323	36.8
F8VZQ9	SARNP	SAP domai	21	5	7	5	213	24.1
P42345	MTOR	Serine/th	3	8	8	8	2549	288.7
O43583	DENR	Density-r	17	4	7	4	198	22.1
Q6ZMI0	PPP1R21	Protein p	8	4	5	4	780	88.3
P29536	LMOD1	Leiomodin	12	9	9	9	600	67
F8VTV8	CDK4	Cyclin-de	29	6	6	5	201	22
Q96RT1	ERBIN	Erbin OS=	5	7	7	7	1412	158.2
Q9Y2R9	MRPS7	28S ribos	24	5	7	5	242	28.1
Q96F86	EDC3	Enhancer	13	7	7	7	508	56
Q13526	PIN1	Peptidyl-	31	4	8	4	163	18.2

A0A1W2PNFPIGT		GPI trans	7	4	6	4	580	65.9
E9PC15	AGK	Acylglyce	10	4	6	4	394	43.8
A6NMQ3	ENSA	Alpha-enc	42	5	8	4	140	15.6
Q16762	TST	Thiosulfa	16	3	4	3	297	33.4
P17948	FLT1	Vascular	4	5	7	4	1338	150.7
P49773	HINT1	Histidine	40	5	9	5	126	13.8
Q96F85	CNRIP1	CB1 canna	42	4	5	4	164	18.6
K7EJE1	NDUFA13	NADH dehy	26	4	6	4	150	17.1
A0A087X21CIP2A		Protein C	8	7	7	7	906	102.2
Q99622	C12orf57	Protein C	44	4	5	4	126	13.2
Q9NZ32	ACTR10	Actin-rel	13	5	6	5	417	46.3
Q9Y2D4	EXOC6B	Exocyst c	8	7	8	7	811	94.1
P07738	BPGM	Bisphosph	29	5	5	5	259	30
P09001	MRPL3	39S ribos	13	6	6	6	348	38.6
Q9H6Z4	RANBP3	Ran-bindi	10	5	5	5	567	60.2
P11279	LAMP1	Lysosome-	12	5	9	5	417	44.9
P23786	CPT2	Carnitine	10	6	6	6	658	73.7
O43148	RNMT	mRNA cap	10	5	7	5	476	54.8
P82675	MRPS5	28S ribos	12	6	7	6	430	48
Q15061	WDR43	WD repeat	9	6	7	6	677	74.8
P40818	USP8	Ubiquitin	6	6	6	6	1118	127.4
Q9H0L4	CSTF2T	Cleavage	12	6	7	1	616	64.4
O75223	GGCT	Gamma-glu	30	5	5	5	188	21
Q9BW27	NUP85	Nuclear p	11	6	6	6	656	75
Q9Y3C1	NOP16	Nucleolar	35	6	6	6	178	21.2
Q6NXE6	ARMC6	Armadillo	13	6	7	6	501	54.1
Q9UNW1	MINPP1	Multiple	11	5	8	5	487	55
Q6ZR64	MXRA7	HBV PreS1	15	3	4	1	213	23.4
Q9H7C4	SYNC	Syncoilin	9	3	4	3	482	55.3
Q15334	LLGL1	Lethal(2)	8	7	7	7	1064	115.3
P05114	HMGN1	Non-histo	28	4	6	4	100	10.7
Q9Y394	DHRS7	Dehydroge	12	4	4	4	339	38.3
A0A0J9YXFPON2		Paraoxona	17	5	7	5	375	41.5
E7ESY4	MTA1	Metastasi	8	6	7	2	703	79.3
Q86XZ4	SPATS2	Spermatog	18	8	8	8	545	59.5
O95182	NDUFA7	NADH dehy	56	6	6	6	113	12.5
Q9NUP9	LIN7C	Protein l	19	4	5	2	197	21.8
P35754	GLRX	Glutaredo	31	3	9	3	106	11.8
O00483	NDUFA4	Cytochrom	56	5	12	5	81	9.4
Q12981	BNIP1	Vesicle t	29	6	6	6	228	26.1
Q9UBW8	COPS7A	COP9 sign	19	6	6	6	275	30.3
Q9H0E2	TOLLIP	Toll-inte	23	6	7	6	274	30.3
P62857	RPS28	40S ribos	46	3	15	3	69	7.8
P82909	MRPS36	28S ribos	42	3	4	3	103	11.5
H0Y7W6	FNBP1	Formin-bi	13	8	8	8	568	65.3
G5E9Z2	CLPTM1L	Cisplatin	13	4	5	4	369	43.3
Q9BX68	HINT2	Histidine	35	4	5	4	163	17.2
Q9HB90	RRAGC	Ras-relat	12	4	5	4	399	44.2
Q8N129	CNPY4	Protein c	13	4	5	4	248	28.3
Q6ZW31	SYDE1	Rho GTPas	9	6	6	6	735	79.7
Q3ZCQ8	TIMM50	Mitochond	15	4	5	4	353	39.6

Q9UPY8	MAPRE3	Microtubu	15	5	10	3	281	32
O75608	LYPLA1	Acyl-prot	20	5	10	5	230	24.7
B1AKR6	DYNLRB1	Dynein li	33	3	4	3	148	16.2
P13747	HLA-E	HLA class	14	4	6	2	358	40.1
P42677	RPS27	40S ribos	38	3	12	1	84	9.5
Q9H8M7	MINDY3	Ubiquitin	9	4	6	4	445	49.7
Q9H1E3	NUCKS1	Nuclear u	21	4	9	4	243	27.3
P81605	DCD	Dermcidin	25	4	7	4	110	11.3
O75886	STAM2	Signal tr	11	6	7	6	525	58.1
Q8NBF2	NHLRC2	NHL repea	8	6	6	6	726	79.4
Q9NY12	GAR1	H/ACA rib	25	5	7	5	217	22.3
Q86TX2	ACOT1	Acyl-coen	14	5	6	5	421	46.2
O15347	HMGB3	High mobi	22	5	7	4	200	23
O75323	NIPSNAP2	Protein N	22	6	9	5	286	33.7
J3KP15	SRSF2	Serine/ar	30	4	12	4	133	15.4
Q9BPX3	NCAPG	Condensin	5	5	6	5	1015	114.3
Q5QJ74	TBCEL	Tubulin-s	13	4	5	4	424	48.2
Q8TC07	TBC1D15	TBC1 doma	7	5	7	5	691	79.4
Q9NWB6	ARGLU1	Arginine	22	8	8	8	273	33.2
A8MQB8	FMR1	Synaptic	12	7	7	5	582	65.8
Q92552	MRPS27	28S ribos	14	6	7	6	414	47.6
O00330	PDHX	Pyruvate	9	5	6	5	501	54.1
Q7LBR1	CHMP1B	Charged m	30	8	9	8	199	22.1
Q9H2W6	MRPL46	39S ribos	21	5	6	5	279	31.7
Q9BSJ2	TUBGCP2	Gamma-tub	5	4	5	4	902	102.5
Q8WZA9	IRGQ	Immunity-	12	4	5	4	623	62.7
O75506	HSBP1	Heat shoc	57	4	6	4	76	8.5
Q9UP83	COG5	Conserved	9	7	7	7	839	92.7
P78537	BLOC1S1	Biogenesi	37	5	8	5	153	17.3
P04114	APOB	Apolipopr	1	7	7	6	4563	515.3
O75891	ALDH1L1	Cytosolic	4	4	7	1	902	98.8
Q8NCN5	PDPR	Pyruvate	7	5	6	5	879	99.3
P11908	PRPS2	Ribose-ph	19	6	8	3	318	34.7
Q9H1B7	IRF2BPL	Interfero	8	6	6	4	796	82.6
Q15021	NCAPD2	Condensin	5	8	8	8	1401	157.1
P31146	CORO1A	Coronin-1	11	5	9	4	461	51
Q9Y5X2	SNX8	Sorting n	10	4	5	4	465	52.5
Q9Y5A9	YTHDF2	YTH domai	9	5	7	2	579	62.3
F8W8I6	TIA1	Nucleolys	12	4	5	2	385	42.8
Q16513	PKN2	Serine/th	8	7	7	6	984	112
Q14139	UBE4A	Ubiquitin	5	4	6	4	1066	122.5
O95232	LUC7L3	Luc7-like	13	5	6	5	432	51.4
Q9BQA1	WDR77	Methylosc	18	5	7	5	342	36.7
Q6ZVM7	TOM1L2	TOM1-like	12	6	6	6	507	55.5
Q7Z2Z2	EFL1	Elongatio	5	6	8	5	1120	125.4
D6RGX2	UFSP2	Ufm1-spec	21	5	5	5	387	44.1
Q9H0U3	MAGT1	Magnesium	15	5	8	4	335	38
P42574	CASP3	Caspase-3	19	4	4	4	277	31.6
Q6EMK4	VASN	Vasorin C	7	4	5	4	673	71.7
Q9BTY7	HGH1	Protein H	18	7	7	7	390	42.1
Q8WZA0	LZIC	Protein L	33	6	6	6	190	21.5

X6R4W8	ZNF207	BUB3-inte	7	3	7	3	497	52.6
Q96HS1	PGAM5	Serine/th	20	4	5	4	289	32
P78356	PIP4K2B	Phosphati	11	6	7	2	416	47.3
F8WAN9	GMPR2	GMP reduc	19	6	6	6	349	38.2
Q9UGR2	ZC3H7B	Zinc fing	7	7	7	7	993	111.5
Q86WR0	CCDC25	Coiled-cc	31	7	8	7	208	24.5
D6W5Y5	CIRBP	Cold indu	11	3	5	3	297	31.9
O60831	PRAF2	PRA1 fami	26	4	5	4	178	19.2
Q16775	HAGH	Hydroxyac	18	6	7	6	308	33.8
Q14197	MRPL58	Peptidyl-	29	6	8	6	206	23.6
Q9BYD3	MRPL4	39S ribos	25	6	6	6	311	34.9
Q9BV86	NTMT1	N-termina	25	4	4	4	223	25.4
O75381	PEX14	Peroxisom	12	4	6	4	377	41.2
P45984	MAPK9	Mitogen-a	8	3	5	3	424	48.1
Q6UXV4	APOOL	MICOS com	17	5	5	5	268	29.1
Q7Z7K6	CENPV	Centromer	17	4	5	4	275	29.9
Q9NZV1	CRIM1	Cysteine-	7	5	5	5	1036	113.7
A0A0A0MSA	PVR	Polioviru	10	4	6	4	392	42.9
Q15276	RABEP1	Rab GTPas	6	6	6	6	862	99.2
J3KMZ8	DPF2	Zinc fing	15	5	6	5	405	45.8
Q29RF7	PDS5A	Sister ch	7	8	8	7	1337	150.7
P08579	SNRPB2	U2 small	20	5	7	4	225	25.5
H0Y5K5	ERGIC3	Endoplasm	14	6	7	6	397	44.6
H7BY82	COL5A1	Collagen	17	3	9	1	210	23.6
Q6NUQ1	RINT1	RAD50-int	7	6	7	6	792	90.6
Q9Y5S9	RBM8A	RNA-bindi	21	4	10	4	174	19.9
Q05655	PRKCD	Protein k	11	7	7	6	676	77.5
O15118	NPC1	NPC intra	5	5	5	5	1278	142.1
A0A067XG5	ATP11C	Phospholi	5	5	5	5	1113	127.6
X6R700	CHTOP	Chromatin	19	3	4	3	223	23.6
A0A1B0GVH	FTO	Alpha-ket	10	5	6	5	559	64.1
P84022	SMAD3	Mothers a	8	4	7	3	425	48.1
Q9NWT6	HIF1AN	Hypoxia-i	14	5	6	5	349	40.3
Q10469	MGAT2	Alpha-1,6	13	4	4	4	447	51.5
C9JME2	FARP1	FERM, ARH	6	7	8	7	1076	122
Q8NFH4	NUP37	Nucleopor	16	5	7	5	326	36.7
Q9BW83	IFT27	Intraflag	30	5	7	5	186	20.5
Q15434	RBMS2	RNA-bindi	12	4	6	2	407	43.9
P53801	PTTG1IP	Pituitary	18	3	10	3	180	20.3
E7EPV7	SNCA	Alpha-syn	38	3	5	3	115	11.8
Q9Y3B4	SF3B6	Splicing	34	4	8	4	125	14.6
O43837	IDH3B	Isocitrat	14	6	6	6	385	42.2
A0A0D9SGD	TATDN1	Putative	19	4	5	4	243	27.6
Q86YP4	GATAD2A	Transcrip	8	4	5	3	633	68
Q15050	RRS1	Ribosome	14	4	5	4	365	41.2
Q9Y315	DERA	Deoxyribo	16	4	4	4	318	35.2
A0A0A0MT6	FDXR	NADPH:adr	12	6	8	6	534	58.2
Q7Z5L9	IRF2BP2	Interfero	11	5	5	3	587	61
Q9Y2D5	AKAP2	A-kinase	6	6	7	6	859	94.6
G3V4P8	GMFB	Glia matu	25	4	8	3	150	17.5
Q99523	SORT1	Sortilin	5	4	6	4	831	92

Q9H1Y0	ATG5	Autophagy	17	4	5	4	275	32.4
R4GMQ1	KDM1A	Lysine-sp	5	4	5	4	858	93.5
Q13426	XRCC4	DNA repai	17	6	6	6	336	38.3
Q9Y3D9	MRPS23	28S ribos	30	6	8	6	190	21.8
K7ERP4	GPX4	Glutathic	35	5	6	5	155	17.6
O95168	NDUFB4	NADH dehy	31	3	6	3	129	15.2
K7EM24	ATP6V0A1	V-type pr	38	4	4	4	130	15.4
P62166	NCS1	Neuronal	28	5	7	5	190	21.9
C9JZY6	UBE2H	Ubiquitir	34	4	6	4	123	13.8
A0A087X2DMRPL45		39S ribos	21	5	6	5	306	35.3
Q96FN4	CPNE2	Copine-2	7	3	7	2	548	61.2
P15090	FABP4	Fatty aci	25	3	5	3	132	14.7
K7EMH1	RPL22	60S ribos	27	2	7	2	89	10.4
H0Y488	ARID1A	AT-rich i	3	5	5	5	1901	205.7
Q01581	HMGCS1	Hydroxyme	11	5	6	5	520	57.3
Q9NXG2	THUMPD1	THUMP dom	14	6	6	6	353	39.3
F6T1Q0	PDE12	2',5'-phc	17	7	8	7	472	52.1
A0A0C4DFLCYP51A1		Lanosterc	9	4	7	4	509	57.2
Q13325	IFIT5	Interferc	13	5	5	5	482	55.8
C9JG97	AAMP	Angio-ass	12	4	4	4	415	44.7
J3KRJ9	COPZ2	Coatomer	33	4	7	4	141	16.1
Q86SX6	GLRX5	Glutaredc	25	3	5	3	157	16.6
Q5W0V3	FAM160B1	Protein F	7	6	7	6	765	86.5
O75844	ZMPSTE24	CAAX prer	11	5	6	5	475	54.8
Q9UFN0	NIPSNAP3AProtein N		19	4	5	4	247	28.4
Q9P265	DIP2B	Disco-int	4	5	5	5	1576	171.4
B1AJY5	PSMD10	26S prote	24	4	5	4	185	20.2
Q9H3H3	C11orf68	UPF0696 p	14	3	5	3	251	27.3
O43678	NDUFA2	NADH dehy	48	5	7	5	99	10.9
D6REB4	PAIP1	Polyadeny	9	4	5	4	351	39.9
Q9NT62	ATG3	Ubiquitir	20	7	9	7	314	35.8
D6RAN1	PDLIM7	PDZ and L	46	5	9	1	90	9.5
Q96FQ6	S100A16	Protein S	33	3	7	3	103	11.8
Q14997	PSME4	Proteasom	4	6	6	6	1843	211.2
Q9NR28	DIABLO	Diablo hc	18	5	7	5	239	27.1
P35249	RFC4	Replicati	15	5	5	5	363	39.7
P49770	EIF2B2	Translati	12	3	5	3	351	39
Q96GK7	FAHD2A	Fumarylac	14	4	6	4	314	34.6
Q13107	USP4	Ubiquitir	5	6	7	5	963	108.5
Q8IY17	PNPLA6	Neuropath	4	6	6	6	1366	149.9
Q6ZXV5	TMTC3	Transmemt	8	7	8	7	915	103.9
P49840	GSK3A	Glycogen	12	5	6	3	483	50.9
Q96PU8	QKI	Protein q	13	4	5	4	341	37.6
O75494	SRSF10	Serine/ar	16	6	6	6	262	31.3
Q15018	ABRAXAS2	BRISC com	8	3	4	3	415	46.9
Q92882	OSTF1	Osteoclas	34	5	5	5	214	23.8
Q7RTV0	PHF5A	PHD finge	48	5	5	5	110	12.4
Q6NVY1	HIBCH	3-hydroxy	18	8	9	8	386	43.5
Q9NVZ3	NECAP2	Adaptin e	23	6	6	6	263	28.3
O95169	NDUFB8	NADH dehy	33	5	7	5	186	21.8
Q9H3K2	GHITM	Growth hc	9	3	5	3	345	37.2

Q9P2W9	STX18	Syntaxin-	19	5	7	5	335	38.7
P16930	FAH	Fumarylac	11	5	8	5	419	46.3
Q8WVV9	HNRNPLL	Heterogen	12	5	7	4	542	60
Q1JUQ5	FKBP1A	Peptidylp	15	1	9	1	92	10.1
H0YME5	EIF2AK4	eIF-2-alp	5	6	6	6	1427	161.1
Q96EE3	SEH1L	Nucleopor	14	4	4	4	360	39.6
Q5JVF3	PCID2	PCI domai	13	6	6	6	399	46
Q92522	H1FX	Histone H	21	4	4	4	213	22.5
Q9NR09	BIRC6	Baculovir	2	6	6	6	4857	529.9
Q8NFW8	CMAS	N-acylneu	15	7	7	7	434	48.3
Q15269	PWP2	Periodic	6	5	5	5	919	102.4
Q99543	DNAJC2	DnaJ homo	13	7	7	7	621	72
Q15599	SLC9A3R2	Na(+)/H(+	17	5	6	5	337	37.4
Q3MHD2	LSM12	Protein L	14	3	9	3	195	21.7
O95219	SNX4	Sorting n	12	5	5	5	450	51.9
H0YNG3	SEC11A	Signal pe	26	5	10	5	163	18.6
Q96QD8	SLC38A2	Sodium-co	13	4	4	4	506	56
C9JBI3	PSPH	Phosphose	17	3	4	3	187	20.7
Q9Y508	RNF114	E3 ubiqui	21	4	6	4	228	25.7
O00115	DNASE2	Deoxyribo	12	4	6	4	360	39.6
Q96I25	RBM17	Splicing	11	5	5	5	401	44.9
Q9BRR6	ADPGK	ADP-depen	7	3	4	3	497	54.1
M0R2N5	TECR	Very-long	15	6	10	6	346	39.9
Q9H3Z4	DNAJC5	DnaJ homo	19	4	6	4	198	22.1
Q8IYM9	TRIM22	E3 ubiqui	10	5	5	5	498	56.9
Q5SWX8	ODR4	Protein o	14	5	5	5	454	51.1
Q02252	ALDH6A1	Methylmal	10	5	6	5	535	57.8
Q15796	SMAD2	Mothers a	7	3	6	2	467	52.3
Q9HD33	MRPL47	39S ribos	20	5	6	5	250	29.4
P52630	STAT2	Signal tr	5	4	5	4	851	97.9
Q92541	RTF1	RNA polym	8	5	6	5	710	80.3
Q9NWH9	SLTM	SAFB-like	6	6	6	6	1034	117.1
Q13636	RAB31	Ras-relat	25	4	5	3	194	21.6
Q7Z4G1	COMMD6	COMM doma	49	4	6	4	85	9.6
Q96IU4	ABHD14B	Protein A	30	5	8	5	210	22.3
E7EQY4	MTA3	Metastasi	11	7	8	1	514	58.7
P26447	S100A4	Protein S	26	3	6	3	101	11.7
Q9Y4K4	MAP4K5	Mitogen-a	6	5	5	5	846	95
Q9P015	MRPL15	39S ribos	18	4	4	4	296	33.4
Q9H2U1	DHX36	ATP-depen	5	6	7	5	1008	114.7
Q9NX40	OCIAD1	OCIA doma	18	4	7	4	245	27.6
E9PQY2	PFDN4	Prefoldin	28	3	6	3	136	15.6
Q8N1B4	VPS52	Vacuolar	9	5	5	5	723	82.2
Q7RTP6	MICAL3	[F-actin]	2	5	6	4	2002	224.2
Q9NPJ3	ACOT13	Acyl-coen	16	2	4	2	140	15
P48060	GLIPR1	Glioma pa	13	2	5	2	266	30.3
H0YJ50	VRK1	Serine/th	16	4	5	4	232	26.7
C9JA28	SSR3	Transloco	16	4	9	4	174	20.1
Q9BZQ6	EDEM3	ER degrad	5	4	4	4	932	104.6
Q9UNQ2	DIMT1	Probable	16	5	5	5	313	35.2
Q9BU89	DOHH	Deoxyhypu	15	3	4	3	302	32.9

P01857	IGHG1	Immunoglc	9	2	4	2	330	36.1
P32456	GBP2	Guanylate	8	5	5	3	591	67.2
O95470	SGPL1	Sphingosi	10	7	8	7	568	63.5
Q92508	PIEZO1	Piezo-typ	2	5	5	5	2521	286.6
A2A2V1	PRNP	Major pri	12	3	8	3	249	27.3
O96005	CLPTM1	Cleft lip	7	4	6	4	669	76
Q7Z434	MAVS	Mitochond	8	4	8	4	540	56.5
Q9UEU0	VTI1B	Vesicle t	22	5	5	5	232	26.7
E9PRJ8	CD81	Tetraspan	10	1	3	1	209	22.5
Q5STZ8	ABCF1	ATP-bindi	15	6	7	2	339	38.3
Q9H7D7	WDR26	WD repeat	7	5	5	5	661	72.1
Q9NRL3	STRN4	Striatin-	6	4	4	3	753	80.5
Q96LJ7	DHRS1	Dehydroge	20	5	5	5	313	33.9
A0A2R8YE1	VPS45	Vacuolar	9	6	7	6	534	61.1
P01033	TIMP1	Metallopr	19	4	5	4	207	23.2
Q5T0D9	TPRG1L	Tumor prc	18	4	5	4	272	30.2
Q5T8P6	RBM26	RNA-bindi	6	6	6	6	1007	113.5
Q9UI30	TRMT112	Multifunc	33	3	5	3	125	14.2
H3BMV3	JPT2	Jupiter m	32	4	6	4	190	20.7
Q7L5D6	GET4	Golgi to	17	5	5	5	327	36.5
C9JYQ9	RPL22L1	60S ribos	26	3	5	3	121	14.5
Q96SL4	GPX7	Glutathic	29	5	5	5	187	21
Q9UI09	NDUFA12	NADH dehy	39	4	4	4	145	17.1
Q9H553	ALG2	Alpha-1,3	6	2	3	2	416	47.1
Q92615	LARP4B	La-relate	10	6	6	6	738	80.5
G0XQ39	STIM1	STIM1L OS	5	4	5	4	791	88.6
Q9Y3C8	UFC1	Ubiquitin	24	4	5	4	167	19.4
Q9NVG8	TBC1D13	TBC1 doma	12	5	5	5	400	46.5
P50851	LRBA	Lipopolys	2	6	6	6	2863	318.9
Q8TDN6	BRIX1	Ribosome	14	5	5	5	353	41.4
Q96TC7	RMDN3	Regulator	15	6	6	6	470	52.1
Q6P3W7	SCYL2	SCY1-like	6	5	5	5	929	103.6
Q00534	CDK6	Cyclin-de	19	5	5	4	326	36.9
Q86YR5	GPSM1	G-protein	8	5	6	5	675	74.5
Q6P1J9	CDC73	Parafibrc	13	6	6	6	531	60.5
Q96FJ2	DYNLL2	Dynein li	45	3	6	2	89	10.3
Q96HD1	CRELD1	Cysteine-	11	4	5	4	420	45.4
P26572	MGAT1	Alpha-1,3	10	4	4	4	445	50.8
Q7L7X3	TAOK1	Serine/th	5	5	7	4	1001	116
O75911	DHRS3	Short-cha	16	4	4	4	302	33.5
O14828	SCAMP3	Secretory	14	3	4	3	347	38.3
Q8WWX9	SELENOM	Selenoprc	26	3	6	3	145	16.2
P17050	NAGA	Alpha-N-a	10	3	4	3	411	46.5
Q14573	ITPR3	Inositol	3	8	8	5	2671	303.9
Q2TAA2	IAH1	Isoamyl a	24	5	9	5	248	27.6
P78357	CNTNAP1	Contactin	4	6	6	6	1384	156.2
K7ENV7	ISOC2	Isochoris	37	3	4	3	174	18.9
V9HW50	ADH1B	Alcohol d	12	2	4	2	375	39.8
Q5R372	RABGAP1L	Rab GTPas	7	5	5	4	815	92.5
Q6YN16	HSDL2	Hydroxyst	11	5	6	5	418	45.4
P41214	EIF2D	Eukaryoti	9	4	4	4	584	64.7

K7EPR5	PRKAR1A	cAMP-depe	93	4	6	1	45	5.1
O75348	ATP6V1G1	V-type pr	28	4	6	4	118	13.7
Q9NUM4	TMEM106B	Transmemb	16	4	6	4	274	31.1
H0YB34	RIDA	2-iminobu	27	3	4	3	148	16
Q86VR2	RETREG3	Reticulop	8	4	6	4	466	51.4
P21926	CD9	CD9 antig	13	3	6	3	228	25.4
Q9BZL4	PPP1R12C	Protein p	7	5	5	5	782	84.8
Q96FZ7	CHMP6	Charged m	24	5	6	5	201	23.5
O00160	MYO1F	Unconvent	3	3	4	1	1098	124.8
O15228	GNPAT	Dihydroxy	8	5	5	5	680	77.1
Q9UJY5	GGA1	ADP-ribos	6	5	6	5	639	70.3
Q9UKS6	PACSIN3	Protein k	15	5	5	5	424	48.5
Q9UL25	RAB21	Ras-relat	18	4	6	4	225	24.3
Q9ULC3	RAB23	Ras-relat	19	4	5	4	237	26.6
O60547	GMDS	GDP-manno	15	6	6	6	372	41.9
H0YB16	PTK2	Focal adh	6	4	4	4	724	81.4
Q14344	GNA13	Guanine n	9	4	7	2	377	44
Q2M389	WASHC4	WASH comp	4	5	6	5	1173	136.3
A0A087WT2	DCAF13	DDB1- and	6	3	4	3	597	67.5
Q13563	PKD2	Polycysti	5	5	7	5	968	109.6
A3KMH1	VWA8	von Wille	3	5	5	5	1905	214.7
Q92665	MRPS31	28S ribos	13	5	5	5	395	45.3
Q6PD62	CTR9	RNA polym	5	6	6	6	1173	133.4
O43264	ZW10	Centromer	6	4	5	4	779	88.8
Q10588	BST1	ADP-ribos	12	4	7	4	318	35.7
P56385	ATP5ME	ATP synth	32	2	4	2	69	7.9
Q13505	MTX1	Metaxin-1	8	3	5	3	466	51.4
Q9BTE1	DCTN5	Dynactin	19	4	6	4	182	20.1
E7EQB8	IDH3G	Isocitrat	15	4	4	4	340	37
P37235	HPCAL1	Hippocalc	33	5	6	5	193	22.3
Q9NY27	PPP4R2	Serine/th	12	5	5	5	417	46.9
O43181	NDUFS4	NADH dehy	19	3	4	3	175	20.1
P04732	MT1E	Metalloth	34	3	6	1	61	6
Q9P287	BCCIP	BRCA2 and	18	4	4	4	314	36
Q9H814	PHAX	Phosphory	9	3	5	3	394	44.4
Q9HBH5	RDH14	Retinol d	15	5	5	5	336	36.8
A0A075B79	GRIPAP1	GRIP1-ass	8	5	5	5	810	92.7
Q9NWM8	FKBP14	Peptidyl-	25	5	6	5	211	24.2
Q92805	GOLGA1	Golgin su	8	5	5	5	767	88.1
C9JPE1	SLC25A20	Mitochond	27	5	5	5	228	25.1
Q5TD07	NQO2	Ribosyldi	29	4	5	4	193	21.5
Q96JB2	COG3	Conserved	7	5	5	5	828	94
E5RGX5	STMN2	Stathmin	15	3	6	1	168	19.6
P09234	SNRPC	U1 small	13	2	4	2	159	17.4
Q8TF05	PPP4R1	Serine/th	4	4	4	4	950	106.9
Q9UNP9	PPIE	Peptidyl-	16	5	6	4	301	33.4
P78563	ADARB1	Double-st	8	4	4	4	741	80.7
P19474	TRIM21	E3 ubiqui	11	5	5	5	475	54.1
Q8ND24	RNF214	RING fing	8	5	5	5	703	77.6
Q9UJ68	MSRA	Mitochond	24	5	5	5	235	26.1
O14530	TXNDC9	Thioredox	12	3	5	3	226	26.5

O75382	TRIM3	Tripartit	6	5	5	5	744	80.8
Q8IVD9	NUDCD3	NudC doma	17	5	6	5	361	40.8
Q4G0F5	VPS26B	Vacuolar	17	5	6	4	336	39.1
A0A0D9SGEPHF6		PHD finge	14	5	5	5	366	41.3
A0A0J9YWWUTP4		U3 small	7	3	4	3	511	57.3
Q13136	PPFIA1	Liprin-al	6	7	7	7	1202	135.7
P07305	H1F0	Histone H	15	3	5	3	194	20.9
A0A0C4DGQDHRS7B		Dehydroge	15	4	4	4	310	33.5
Q9BVC6	TMEM109	Transmemb	9	3	5	3	243	26.2
Q8IWA5	SLC44A2	Choline t	5	4	6	4	706	80.1
E7ER68	FAM91A1	Protein F	6	4	4	4	789	88.9
O60343	TBC1D4	TBC1 doma	4	5	5	5	1298	146.5
Q9NX58	LYAR	Cell grow	11	4	5	4	379	43.6
Q9HA77	CARS2	Probable	11	5	5	5	564	62.2
P35556	FBN2	Fibrillin	2	6	6	2	2912	314.6
Q9H9Q2	COPS7B	COP9 sign	10	2	4	2	264	29.6
Q5QPQ0	LYPLA2	Acyl-prot	31	4	6	4	164	17.6
Q9NWU2	GID8	Glucose-i	25	4	4	4	228	26.7
Q5SRE5	NUP188	Nucleopor	3	6	6	6	1749	195.9
O14936	CASK	Periphera	6	6	6	6	926	105.1
F2Z2E2	IQGAP3	Ras GTPas	3	4	12	2	1588	179.4
C9JIJ9	RBMS3	RNA-bindi	8	4	5	1	436	47.7
Q12974	PTP4A2	Protein t	26	4	4	2	167	19.1
O60828	PQBP1	Polygluta	21	5	6	5	265	30.5
Q9BYN8	MRPS26	28S ribos	27	5	5	5	205	24.2
P41743	PRKCI	Protein k	9	5	5	5	596	68.2
P21359	NF1	Neurofibr	2	6	6	6	2839	319.2
P51003	PAPOLA	Poly(A) p	5	4	5	4	745	82.8
P56182	RRP1	Ribosomal	10	4	4	4	461	52.8
O14907	TAX1BP3	Tax1-bind	25	3	7	3	124	13.7
A0A087X1FACAP2		Arf-GAP w	8	6	6	6	777	88
E9PQP6	FNTA	Protein f	17	4	4	4	249	30.1
Q9GZY8	MFF	Mitochond	16	3	3	3	342	38.4
O43809	NUDT21	Cleavage	23	5	6	5	227	26.2
Q86VP1	TAX1BP1	Tax1-bind	7	7	8	7	789	90.8
J3KS22	DCXR	L-xylulos	17	4	4	4	223	23.8
O00422	SAP18	Histone d	29	5	9	5	153	17.6
Q13595	TRA2A	Transform	17	5	5	5	282	32.7
P10620	MGST1	Microsoma	9	2	4	2	155	17.6
Q9NYB9	ABI2	Abl inter	9	4	4	2	513	55.6
A8MTY9	VPS26C	VPS26 end	12	2	3	2	249	27.9
D4Q8H0	pk	Mitogen-a	11	4	4	4	455	51.5
Q9BYG3	NIFK	MKI67 FHA	17	6	9	6	293	34.2
Q9NX20	MRPL16	39S ribos	18	3	4	3	251	28.4
Q9HA64	FN3KRP	Ketosamin	13	5	5	5	309	34.4
O95205	MBNL2	Musclebli	16	5	9	2	255	28.1
Q8WW12	PCNP	PEST prot	18	3	4	3	178	18.9
Q92692	NECTIN2	Nectin-2	9	6	8	6	538	57.7
Q96L92	SNX27	Sorting n	7	4	4	4	541	61.2
A0A096LP2AAK1		AP2-assoc	12	4	4	3	511	54.4
Q93063	EXT2	Exostosin	7	5	6	5	718	82.2

H0YMJ0	MORF4L1	Mortality	15	3	4	3	245	28.2
H0YGX7	ARHGDIB	Rho GDP-d	29	4	4	4	195	22.4
Q9H4A5	GOLPH3L	Golgi phc	15	4	6	4	285	32.7
P25445	FAS	Tumor nec	14	4	4	4	335	37.7
A0A1B0GUE	ASAH1	Acid cera	20	6	6	6	330	37.4
Q9NX14	NDUFB11	NADH dehy	27	3	4	3	153	17.3
H7BYT1	CSNK1D	Casein ki	12	5	7	4	427	49
F8WC86	LIMS1	LIM and s	26	3	6	2	117	13.3
J3KNF4	CCS	Copper ch	20	6	6	6	255	27.1
P52298	NCBP2	Nuclear c	28	5	5	5	156	18
O75970	MPDZ	Multiple	3	5	5	5	2070	221.5
Q8IWV7	UBR1	E3 ubiqui	3	4	5	4	1749	200.1
O76071	CIAO1	Probable	17	4	4	4	339	37.8
J3KPS0	DNAJB12	DnaJ (Hsp	11	4	4	4	409	45.5
Q15126	PMVK	Phosphome	23	4	4	4	192	22
Q9Y3E7	CHMP3	Charged m	18	5	6	5	222	25.1
B4E321	OS9	Protein C	15	5	5	5	406	46.3
Q6SZW1	SARM1	Sterile a	5	3	4	3	724	79.3
P13995	MTHFD2	Bifunctic	17	5	7	5	350	37.9
P13611	VCAN	Versican	1	5	5	5	3396	372.6
Q8IYB5	SMAP1	Stromal m	8	4	6	4	467	50.4
Q6IA86	ELP2	Elongator	6	4	5	4	826	92.4
P35914	HMGCL	Hydroxyme	14	4	4	4	325	34.3
Q15428	SF3A2	Splicing	7	3	4	3	464	49.2
Q96CX2	KCTD12	BTB/POZ d	20	6	6	5	325	35.7
Q8WU10	PYROXD1	Pyridine	8	3	5	3	500	55.8
Q86WN1	FCHSD1	F-BAR and	6	5	5	5	690	76.9
F8VXI9	GIT2	ARF GTPas	8	5	5	3	708	78.8
P09669	COX6C	Cytochrom	36	5	5	5	75	8.8
Q6PJT7	ZC3H14	Zinc fing	7	5	5	5	736	82.8
G3V599	CTAGE5	Endoplasm	3	4	5	4	1339	151.6
P61956	SUMO2	Small ubi	23	2	6	1	95	10.9
P04818	TYMS	Thymidyla	9	2	3	2	313	35.7
O00400	SLC33A1	Acetyl-cc	8	4	6	4	549	60.9
M0R3D4	RABAC1	PRA1 fami	25	3	4	3	151	17
P63220	RPS21	40S ribos	48	4	11	4	83	9.1
O75915	ARL6IP5	PRA1 fami	20	4	5	4	188	21.6
Q9H792	PEAK1	Inactive	3	4	4	4	1746	193
Q15814	TBCC	Tubulin-s	15	5	6	4	346	39.2
Q9Y6A4	CFAP20	Cilia- an	17	3	5	3	193	22.8
Q9NZW5	MPP6	MAGUK p55	9	5	5	4	540	61.1
Q9Y6I3	EPN1	Epsin-1 C	8	5	6	5	576	60.3
O60711	LPXN	Leupaxin	14	4	4	4	386	43.3
C9J1V9	EEF1E1-BL	EEF1E1-BL	27	4	5	4	151	17
Q6BCY4	CYB5R2	NADH-cytc	20	5	6	5	276	31.4
Q6ZRP7	QSOX2	Sulfhydry	9	6	7	6	698	77.5
Q9NW64	RBM22	Pre-mRNA-	11	4	4	4	420	46.9
Q7Z4Q2	HEATR3	HEAT repe	8	4	4	4	680	74.5
P17568	NDUFB7	NADH dehy	26	4	7	4	137	16.4
Q5JWE9	GNAS	Guanine n	15	3	4	1	193	21.9
G3V2S9	SLIRP	SRA stem-	19	2	3	2	124	13.9

O43823	AKAP8	A-kinase	7	3	3	3	692	76.1
P61457	PCBD1	Pterin-4-	30	4	5	3	104	12
O60783	MRPS14	28S ribos	19	2	3	2	128	15.1
Q9H0R4	HDHD2	Haloacid	20	4	5	4	259	28.5
Q96TA2	YME1L1	ATP-depen	6	5	7	5	773	86.4
Q5T760	SRSF11	Serine/ar	8	3	4	3	389	42.3
Q9BQE4	SELENOS	Selenopro	21	3	4	3	189	21.2
O14773	TPP1	Tripeptid	8	4	7	4	563	61.2
Q7L775	EPM2AIP1	EPM2A-int	9	5	5	5	607	70.3
Q3YEC7	RABL6	Rab-like	9	6	6	6	729	79.5
Q9GZL7	WDR12	Ribosome	18	6	6	6	423	47.7
Q9NV31	IMP3	U3 small	15	2	5	2	184	21.8
P43155	CRAT	Carnitine	9	6	6	6	626	70.8
A0A286YFL	PPT1	Palmitoyl	9	4	4	4	323	35.7
G3V2U7	ACYP1	Acylphosp	18	2	3	2	129	14.1
Q8WVY7	UBLCP1	Ubiquitin	14	5	6	5	318	36.8
Q9Y3A6	TMED5	Transmemb	10	3	5	3	229	26
Q68DW7	STAG1	Cohesin s	5	5	5	1	998	114.9
Q7Z7H8	MRPL10	39S ribos	17	3	5	3	261	29.3
Q9BX67	JAM3	Junctiona	11	2	3	2	310	35
Q9BY77	POLDIP3	Polymeras	11	4	5	4	421	46.1
P23508	MCC	Colorecta	6	5	5	5	829	93
Q9ULJ7	ANKRD50	Ankyrin r	3	4	4	4	1429	155.8
Q13405	MRPL49	39S ribos	27	4	5	4	166	19.2
P53701	HCCS	Cytochrom	20	5	5	5	268	30.6
Q8N4A0	GALNT4	Polypepti	7	4	4	3	578	66.6
Q99570	PIK3R4	Phosphoin	4	4	4	4	1358	153
Q9NPF4	OSGEP	Probable	12	4	5	4	335	36.4
Q96GA7	SDSL	Serine de	11	3	4	3	329	34.7
Q9NWU5	MRPL22	39S ribos	21	5	8	5	206	23.6
H3BR29	C16orf58	RUS1 fami	10	4	5	4	423	46.3
Q9NRY5	FAM114A2	Protein F	8	4	4	4	505	55.4
Q9Y2R5	MRPS17	28S ribos	45	4	5	4	130	14.5
Q03113	GNA12	Guanine n	8	3	6	1	381	44.3
Q9NVM9	INTS13	Integrato	7	5	5	5	706	80.2
P45985	MAP2K4	Dual spec	15	5	5	5	399	44.3
Q53S08	RAB6D	Ras-relat	12	2	6	1	254	28.2
B7Z2L0	BCAP29	cDNA FLJ5	21	6	6	6	147	17.4
Q5VW32	BROX	BRO1 doma	10	3	4	3	411	46.4
Q9H0W8	SMG9	Protein S	13	5	5	5	520	57.6
Q9NZT2	OGFR	Opioid gr	7	5	5	5	677	73.3
Q15629	TRAM1	Transloca	6	2	3	2	374	43
Q8N4C8	MINK1	Misshapen	3	3	4	1	1332	149.7
P60468	SEC61B	Protein t	30	3	7	3	96	10
P62072	TIMM10	Mitochond	31	3	4	3	90	10.3
Q9HBH1	PDF	Peptide d	23	3	3	3	243	27
P07711	CTSL	Cathepsin	10	3	4	3	333	37.5
O14657	TOR1B	Torsin-1B	12	4	4	4	336	38
Q9P2I0	CPSF2	Cleavage	4	2	4	2	782	88.4
A0A075B6F	NOSIP	Nitric ox	12	3	5	3	304	33.4
Q8IZH2	XRN1	5’-3’ exc	2	3	4	3	1706	194

Q92643	PIGK	GPI-ancho	10	3	3	3	395	45.2
Q71RC2	LARP4	La-relate	5	3	4	3	724	80.5
Q13643	FHL3	Four and	11	3	5	3	280	31.2
H0Y9X1	TMA16	Translati	19	4	4	4	242	27.7
Q9C035	TRIM5	Tripartit	10	6	6	6	493	56.3
I3L4C3	SPAG7	Sperm-ass	22	4	4	4	194	22.1
Q5RI15	COX20	Cytochrom	23	3	5	3	118	13.3
P32189	GK	Glycerol	8	5	5	4	559	61.2
Q5TEJ7	RPA2	Replicati	23	3	3	3	179	19.4
H0Y8C3	MTCH1	Mitochond	11	3	3	3	394	43.1
Q12802	AKAP13	A-kinase	2	4	4	4	2813	307.4
Q96T23	RSF1	Remodelin	3	5	5	5	1441	163.7
H3BMD8	ARPP19	cAMP-regu	37	3	5	2	131	14.5
Q9H3K6	BOLA2	BolA-like	59	4	5	4	86	10.1
Q5BKZ1	ZNF326	DBIRD com	9	4	4	4	582	65.6
Q9UKF6	CPSF3	Cleavage	8	5	5	5	684	77.4
P52735	VAV2	Guanine n	7	6	6	6	878	101.2
Q9P0M6	H2AFY2	Core hist	11	4	5	3	372	40
Q01081	U2AF1	Splicing	16	4	4	4	240	27.9
Q9Y237	PIN4	Peptidyl-	33	2	3	2	131	13.8
P68402	PAFAH1B2	Platelet-	17	4	8	4	229	25.6
Q13823	GNL2	Nucleolar	6	5	5	5	731	83.6
Q86XA9	HEATR5A	HEAT repe	3	5	5	5	2040	221.9
Q9GZX9	TWSG1	Twisted g	17	3	5	3	223	25
O43166	SIPA1L1	Signal-in	2	5	5	5	1804	199.9
O00762	UBE2C	Ubiquitin	31	4	4	4	179	19.6
Q99442	SEC62	Transloca	9	4	6	4	399	45.8
Q6IQ22	RAB12	Ras-relat	21	6	6	5	244	27.2
Q9NQ50	MRPL40	39S ribos	15	2	3	2	206	24.5
P52701	MSH6	DNA misma	4	5	5	5	1360	152.7
P62312	LSM6	U6 snRNA-	34	3	6	3	80	9.1
C9JLV4	APAF1	Apoptotic	3	4	4	4	1163	132.4
Q8N3U4	STAG2	Cohesin s	3	5	5	1	1231	141.2
H3BQQ2	ZNF598	E3 ubiqui	4	3	3	3	849	93.2
P42771	CDKN2A	Cyclin-de	26	3	3	2	156	16.5
Q8N9N7	LRRC57	Leucine-r	12	3	4	3	239	26.7
R4GMU7	RPL7L1	60S ribos	25	5	6	5	198	23
Q9Y2E5	MAN2B2	Epididymi	5	4	5	4	1009	113.9
Q9UPU7	TBC1D2B	TBC1 doma	5	5	5	5	963	109.8
Q16831	UPP1	Uridine p	12	4	4	4	310	33.9
Q9BVM2	DPCD	Protein D	18	4	4	4	203	23.2
Q9Y4P8	WIPI2	WD repeat	10	3	3	3	454	49.4
Q8IUI8	CRLF3	Cytokine	8	3	3	3	442	49.7
P30049	ATP5F1D	ATP synth	14	2	6	2	168	17.5
Q7LG56	RRM2B	Ribonucle	11	4	4	3	351	40.7
A0A2R8Y4M	CLIC5	Chloride	12	3	6	2	200	22.3
O15427	SLC16A3	Monocarbo	6	3	11	3	465	49.4
Q9Y5J7	TIMM9	Mitochond	29	2	5	2	89	10.4
O75937	DNAJC8	DnaJ homo	15	4	4	4	253	29.8
Q08AM6	VAC14	Protein V	7	3	3	3	782	87.9
C9JAB9	NCK1	Cytoplasm	17	3	5	3	174	20.1

O43310	CTIF	CBP80/20-	6	4	4	4	598	67.5
Q00535	CDK5	Cyclin-de	18	4	7	3	292	33.3
C9JWU9	MEST	Mesoderm-	18	3	5	3	213	24.3
Q96C01	FAM136A	Protein F	24	4	5	4	138	15.6
P61009	SPCS3	Signal pe	16	3	4	3	180	20.3
P09455	RBP1	Retinol-b	30	4	4	4	135	15.8
Q86VM9	ZC3H18	Zinc fing	4	4	4	4	953	106.3
P83876	TXNL4A	Thioredox	24	3	5	3	142	16.8
O43251	RBFOX2	RNA bindi	12	3	4	3	390	41.3
Q13445	TMED1	Transmemb	13	3	4	3	227	25.2
Q53H96	PYCR3	Pyrroline	18	3	3	3	274	28.6
Q9Y3D7	PAM16	Mitochond	44	5	7	5	125	13.8
Q15006	EMC2	ER membra	14	3	4	3	297	34.8
Q16836	HADH	Hydroxyac	17	6	8	6	314	34.3
E9PHA2	NCAPH	Condensin	5	4	6	4	730	81.5
P52895	AKR1C2	Aldo-keto	19	4	4	4	323	36.7
P61927	RPL37	60S ribos	38	5	30	5	97	11.1
A0A0A0MT3	LIPA	Lysosomal	10	2	5	2	283	32.5
P30405	PPIF	Peptidyl-	18	5	11	3	207	22
P14406	COX7A2	Cytochrom	28	2	5	2	83	9.4
P15848	ARSB	Arylsulfa	8	4	4	4	533	59.6
P07948	LYN	Tyrosine-	8	4	4	2	512	58.5
O14920	IKBKB	Inhibitor	6	4	4	3	756	86.5
Q9NW68	BSDC1	BSD domai	10	4	4	4	430	47.1
Q8N0U8	VKORC1L1	Vitamin K	15	3	7	3	176	19.8
Q6P9B6	TLDC1	TLD domai	11	4	7	4	456	51
Q96A35	MRPL24	39S ribos	21	4	4	4	216	24.9
Q86SE5	RALYL	RNA-bindi	9	3	4	1	291	32.3
E7EVC7	ATG16L1	Autophagy	7	5	5	5	624	70
Q9Y547	HSPB11	Intraflag	25	2	3	2	144	16.3
O15439	ABCC4	Multidrug	3	4	4	4	1325	149.4
Q92504	SLC39A7	Zinc tran	4	3	6	3	469	50.1
Q96KR1	ZFR	Zinc fing	5	4	4	4	1074	116.9
Q9NQY0	BIN3	Bridging	15	4	4	4	253	29.6
Q9Y399	MRPS2	28S ribos	11	4	5	4	296	33.2
E9PNK6	TPD52L1	Tumor pro	22	3	4	3	166	18.6
P37198	NUP62	Nuclear p	7	3	3	3	522	53.2
D6RIY7	TUSC3	Tumor sup	11	4	5	3	314	35.7
A0A0A0MSH	CHD1L	Chromodom	8	6	6	6	797	89.9
Q9UNN5	FAF1	FAS-assoc	6	3	4	3	650	73.9
Q9H939	PSTPIP2	Proline-s	13	5	5	5	334	38.8
Q68DU8	KCTD16	BTB/POZ d	11	5	6	4	428	49.1
E7EP22	RNF14	RBR-type	21	3	4	3	152	17.5
Q86Y39	NDUFA11	NADH dehy	35	3	3	3	141	14.8
Q9H078	CLPB	Caseinoly	6	4	4	3	707	78.7
Q9HD42	CHMP1A	Charged m	20	5	6	5	196	21.7
A8MU27	SUMO3	Small ubi	14	2	6	1	147	16.9
A0A1W2PQV	GCSH	Glycine c	30	2	4	2	100	11.1
Q92575	UBXN4	UBX domai	11	5	5	5	508	56.7
O95478	NSA2	Ribosome	14	4	4	4	260	30
Q9HAB8	PPCS	Phosphopa	13	5	5	5	311	34

P23458	JAK1	Tyrosine-	4	5	5	5	1154	133.2
Q00587	CDC42EP1	Cdc42 eff	11	3	3	3	391	40.3
A2IDC6	MRPL28	39S ribos	12	4	4	4	240	28.5
A0A0A0MS7	MYD88	Myeloid d	13	3	3	3	309	34.6
Q9Y3D0	FAM96B	Mitotic s	26	2	2	2	163	17.7
P82912	MRPS11	28S ribos	22	3	4	3	194	20.6
P63172	DYNLT1	Dynein li	23	2	3	2	113	12.4
O60447	EVI5	Ecotropic	5	4	4	3	810	92.9
D6RCB9	NHP2	H/ACA rib	21	2	7	2	135	15.2
A0A1W2PP1	VPS35	Vacuolar	65	3	4	1	46	5.4
Q9UMY1	NOL7	Nucleolar	11	3	4	3	257	29.4
Q5VZE5	NAA35	N-alpha-a	5	4	4	4	725	83.6
Q96DA6	DNAJC19	Mitochond	34	4	4	4	116	12.5
Q9BR61	ACBD6	Acyl-CoA-	9	2	3	2	282	31.1
Q96S59	RANBP9	Ran-bindi	4	2	3	2	729	77.8
D6RDG3	BTF3	Transcrip	36	3	8	1	109	11.8
P62314	SNRPD1	Small nuc	28	2	5	2	119	13.3
E7EMN6	PPP1R2	Protein p	21	3	3	3	170	19.2
P30530	AXL	Tyrosine-	5	3	3	3	894	98.3
Q9Y5K6	CD2AP	CD2-assoc	6	4	5	4	639	71.4
A0A0C4DFX	NELFA	Negative	6	3	3	3	539	58.5
Q9H446	RWDD1	RWD domai	11	3	4	3	243	27.9
K4DIA7	CD151	Tetraspan	17	5	12	5	230	25.6
O43402	EMC8	ER membra	17	3	3	3	210	23.8
Q9Y2Y0	ARL2BP	ADP-ribos	24	3	3	3	163	18.8
Q5TCZ1	SH3PXD2A	SH3 and P	3	4	4	2	1133	125.2
Q16864	ATP6V1F	V-type pr	24	3	5	3	119	13.4
A0A087X0M	SLC4A1AP	Kanadapti	6	3	3	3	742	82.8
C9JJ19	MRPS34	28S ribos	17	4	4	4	225	26.3
Q15847	ADIRF	Adipogene	29	2	3	2	76	7.9
P42785	PRCP	Lysosomal	6	3	4	3	496	55.8
F8W7U0	ITSN1	Intersect	3	4	5	4	1149	129.9
Q96EY5	MVB12A	Multivesi	16	3	4	3	273	28.8
Q86VN1	VPS36	Vacuolar	11	4	5	4	386	43.8
Q9HBL8	NMRAL1	NmrA-like	11	3	3	3	299	33.3
Q15650	TRIP4	Activatin	8	4	4	4	581	66.1
Q13951	CBFB	Core-bind	20	3	3	3	182	21.5
Q9P2E5	CHPF2	Chondroit	5	4	5	4	772	85.9
O75175	CNOT3	CCR4-NOT	5	4	4	4	753	81.8
Q07092	COL16A1	Collagen	3	3	3	3	1604	157.7
B8ZZS0	BET1L	BET1-like	18	2	3	2	152	17.1
Q8WYP5	AHCTF1	Protein E	2	4	4	4	2266	252.3
Q16595	FXN	Frataxin,	11	2	3	2	210	23.1
Q8IWX8	CHERP	Calcium h	5	5	5	5	916	103.6
O95298	NDUFC2	NADH dehy	17	2	5	2	119	14.2
A0A0A0MRJ	NAV1	Neuron na	2	4	4	4	1830	197.3
Q7Z7N9	TMEM179B	Transmemb	11	2	3	2	219	23.5
P78346	RPP30	Ribonucle	16	4	4	4	268	29.3
P49427	CDC34	Ubiquitin	15	3	4	3	236	26.7
P06400	RB1	Retinobla	4	5	5	5	928	106.1
Q96T60	PNKP	Bifunctic	8	4	4	4	521	57

Q9HAU5	UPF2	Regulator	4	5	6	5	1272	147.7
Q6NUM9	RETSAT	All-trans	4	3	4	3	610	66.8
I3L4C2	BAIAP2	Brain-spe	8	5	5	5	553	61.3
Q9Y2H0	DLGAP4	Disks lar	4	5	5	5	992	107.9
Q13451	FKBP5	Peptidyl-	11	4	4	4	457	51.2
Q16563	SYPL1	Synaptoph	10	2	5	2	259	28.5
A0A0A0MQF	RTF2	Replicati	11	3	3	3	336	37.5
Q8WTS6	SETD7	Histone-l	10	3	3	3	366	40.7
Q8ND04	SMG8	Protein S	4	3	3	3	991	109.6
E5RGN3	ATOX1	Copper tr	29	3	6	3	59	6.3
Q15678	PTPN14	Tyrosine-	4	5	6	5	1187	135.2
Q92542	NCSTN	Nicastrin	6	5	7	5	709	78.4
M0QXB5	ETHE1	Persulfid	11	3	3	3	260	28.4
P29466	CASP1	Caspase-1	9	4	4	2	404	45.1
Q6UXN9	WDR82	WD repeat	19	5	5	5	313	35.1
Q7Z7H5	TMED4	Transmemb	8	2	3	2	227	25.9
Q15904	ATP6AP1	V-type pr	8	3	3	3	470	52
Q4R9M9	KIF1B	Kinesin f	2	4	4	1	1809	203.5
Q9HCN8	SDF2L1	Stromal c	26	3	3	3	221	23.6
F5H5N1	NDUFS7	NADH dehy	13	2	3	2	182	19.8
Q9Y676	MRPS18B	28S ribos	16	3	3	3	258	29.4
O15160	POLR1C	DNA-direc	13	3	4	3	346	39.2
C9JG87	MRPL39	39S ribos	14	4	4	4	297	34
O00506	STK25	Serine/th	11	4	4	2	426	48.1
Q9Y316	MEMO1	Protein M	12	3	4	3	297	33.7
P31350	RRM2	Ribonucle	14	5	5	4	389	44.8
P84243	H3F3A	Histone H	24	4	18	2	136	15.3
Q8ND56	LSM14A	Protein L	8	3	3	3	463	50.5
P07919	UQCRH	Cytochrom	44	4	4	4	91	10.7
O15111	CHUK	Inhibitor	6	6	6	5	745	84.6
O95674	CDS2	Phosphati	9	3	3	3	445	51.4
Q13601	KRR1	KRR1 smal	10	4	5	4	381	43.6
P78318	IGBP1	Immunoglo	10	3	3	3	339	39.2
O95295	SNAPIN	SNARE-ass	32	4	4	4	136	14.9
Q53GS9	USP39	U4/U6.U5	6	3	3	3	565	65.3
P22830	FECH	Ferrochel	11	5	5	5	423	47.8
O95810	CAVIN2	Caveolae-	10	3	3	3	425	47.1
Q16352	INA	Alpha-int	4	2	23	1	499	55.4
O43660	PLRG1	Pleiotrop	8	5	5	5	514	57.2
Q9Y697	NFS1	Cysteine	13	4	4	4	457	50.2
Q9BZX2	UCK2	Uridine-c	18	4	4	4	261	29.3
P49757	NUMB	Protein n	6	4	5	3	651	70.8
P32929	CTH	Cystathio	6	2	3	2	405	44.5
Q9UEE9	CFDP1	Craniofac	17	4	4	4	299	33.6
P29972	AQP1	Aquaporin	10	2	3	2	269	28.5
O60678	PRMT3	Protein a	9	4	4	4	531	59.9
Q9HAT2	SIAE	Sialate O	7	4	4	4	523	58.3
Q9UBB6	NCDN	Neurochon	5	4	5	4	729	78.8
Q9UID3	VPS51	Vacuolar	8	4	4	4	782	86
Q96SU4	OSBPL9	Oxysterol	4	3	4	3	736	83.1
Q7L8L6	FASTKD5	FAST kina	5	4	4	4	764	86.5

K7EIN2	NUDT16L1	Tudor-int	25	4	4	3	198	21.9
K7EIU8	SMAD4	Mothers a	9	5	5	5	456	50
P51580	TPMT	Thiopurin	18	5	7	5	245	28.2
P49754	VPS41	Vacuolar	4	4	4	4	854	98.5
O43920	NDUFS5	NADH dehy	35	3	3	3	106	12.5
Q8IYB7	DIS3L2	DIS3-like	4	4	5	4	885	99.2
J3KQS6	BABAM1	BRISC and	16	3	3	3	254	28.1
Q96GG9	DCUN1D1	DCN1-like	15	4	4	4	259	30.1
Q9NQG5	RPRD1B	Regulatio	10	2	3	2	326	36.9
P42696	RBM34	RNA-bindi	7	2	2	2	430	48.5
Q8WUA2	PPIL4	Peptidyl-	8	3	3	3	492	57.2
Q92600	CNOT9	CCR4-NOT	16	4	4	4	299	33.6
Q8NEU8	APPL2	DCC-inter	6	3	3	3	664	74.4
E9PPN1	TSEN15	tRNA-spli	17	1	2	1	134	14.6
O43314	PPIP5K2	Inositol	4	5	5	5	1243	140.3
A0A0C4DG3	SFTPA1	Pulmonary	9	1	3	1	158	16
F5H7R9	PTMS	Parathymo	30	2	8	2	57	6.3
Q6RFH5	WDR74	WD repeat	10	3	3	3	385	42.4
P82921	MRPS21	28S ribos	30	2	3	2	87	10.7
Q06265	EXOSC9	Exosome c	9	5	5	5	439	48.9
Q9BXW7	HDHD5	Haloacid	8	2	2	2	423	46.3
Q8IUR0	TRAPPC5	Trafficki	26	5	5	5	188	20.8
Q5TDF0	NTPCR	Cancer-re	15	3	5	3	228	25.1
Q8IX01	SUGP2	SURP and	6	4	4	4	1082	120.1
F8W7Q4	FAM162A	Protein F	27	4	4	4	144	16.5
Q14534	SQLE	Squalene	8	3	3	3	574	63.9
H3BUN4	NOL3	Nucleolar	18	2	4	2	206	22.4
A0A0A0MR5	FADS1	Fatty aci	10	5	6	4	501	57.8
O60216	RAD21	Double-st	5	3	3	3	631	71.6
Q96S66	CLCC1	Chloride	8	4	5	4	551	62
P78316	NOP14	Nucleolar	5	4	4	4	857	97.6
Q13336	SLC14A1	Urea tran	9	4	4	4	389	42.5
E9PLP0	CARS	Cysteine-	20	2	3	1	128	14.3
A0A087X29	WDR6	WD repeat	4	3	3	3	1151	124.9
Q9BZE9	ASPSCR1	Tether co	7	3	3	3	553	60.1
Q9GZS1	POLR1E	DNA-direc	8	4	4	2	481	53.9
Q8NEJ9	NGDN	Neuroguid	10	3	3	3	315	35.9
P15954	COX7C	Cytochrom	29	2	4	2	63	7.2
Q96GX9	APIP	Methylthi	13	2	2	2	242	27.1
Q8WU79	SMAP2	Stromal m	8	4	4	4	429	46.8
G5EA36	CDC27	Cell divi	4	3	3	3	823	91.7
P01834	IGKC	Immunoglo	13	1	6	1	107	11.8
E9PF49	NDUFB9	NADH dehy	17	3	4	3	221	26.6
P00403	MT-CO2	Cytochrom	23	4	5	4	227	25.5
Q7Z7M9	GALNT5	Polypepti	3	3	3	2	940	106.2
Q9BY32	ITPA	Inosine t	24	3	4	3	194	21.4
Q712K3	UBE2R2	Ubiquitin	18	4	4	4	238	27.1
Q92543	SNX19	Sorting n	4	3	3	3	992	108.5
Q9UJX3	ANAPC7	Anaphase-	7	4	4	4	599	66.8
P07585	DCN	Decorin C	11	4	4	3	359	39.7
E9PHM2	LARS2	Probable	5	5	5	5	860	96.9

O75503	CLN5	Ceroid-li	10	4	4	4	358	41.5
Q8MH48	HLA-G	HLA class	7	2	4	1	338	38.2
O75431	MTX2	Metaxin-2	8	2	3	2	263	29.7
Q12800	TFCP2	Alpha-glc	8	3	3	2	502	57.2
G3V1D1	FTH1	Ferritin	39	3	3	3	113	12.9
Q13144	EIF2B5	Translati	3	2	2	2	721	80.3
Q9UKX5	ITGA11	Integrin	4	6	6	6	1188	133.4
E9PNY1	ZFPL1	Zinc fing	23	4	4	4	207	22.7
E9PKV8	TTC9C	Tetratric	21	3	4	3	140	16.3
Q9UHI6	DDX20	Probable	5	3	3	3	824	92.2
Q8N2K0	ABHD12	Monoacylg	10	3	4	3	398	45.1
Q96DV4	MRPL38	39S ribos	10	3	5	3	380	44.6
F5H1F6	VPS37B	Vacuolar	19	3	3	3	184	20.6
Q9Y217	MTMR6	Myotubula	8	4	4	4	621	71.9
Q9Y4F5	CEP170B	Centrosom	2	3	4	1	1589	171.6
H0Y6Y8	MRPL43	39S ribos	11	2	3	2	169	18.8
O15121	DEGS1	Sphingoli	11	3	4	3	323	37.8
P62745	RHOB	Rho-relat	15	2	3	1	196	22.1
Q9Y3A2	UTP11	Probable	14	4	4	4	253	30.4
Q9UQN3	CHMP2B	Charged m	15	4	4	4	213	23.9
O15173	PGRMC2	Membrane-	11	3	5	2	223	23.8
A0A1B0GWC	DPY19L1	Probable	6	3	3	3	748	84.5
P50750	CDK9	Cyclin-de	9	4	5	3	372	42.8
J3KNF8	CYB5B	Cytochrom	27	3	6	3	150	16.7
Q96IK1	BOD1	Biorienta	18	3	3	3	185	19.2
Q04771	ACVR1	Activin r	5	2	3	2	509	57.1
P53602	MVD	Diphosphc	6	2	3	2	400	43.4
B4DHE8	MSI2	cDNA FLJ5	8	2	3	2	324	34.8
O94915	FRYL	Protein f	1	4	4	4	3013	339.4
P27449	ATP6V0C	V-type pr	12	1	3	1	155	15.7
H7C5K4	CCDC80	Coiled-cc	8	2	3	2	261	30.5
Q9GZT9	EGLN1	Egl nine	7	3	3	3	426	46
Q9Y385	UBE2J1	Ubiquitir	6	2	3	2	318	35.2
Q8NB37	GATD1	Glutamine	14	3	3	3	220	23.3
P19387	POLR2C	DNA-direc	12	3	3	3	275	31.4
Q8WTW3	COG1	Conservec	3	4	4	4	980	108.9
O96011	PEX11B	Peroxisom	12	3	4	3	259	28.4
Q96RF0	SNX18	Sorting n	7	3	3	3	628	68.9
O00291	HIP1	Huntingti	4	6	6	5	1037	116.1
Q8NEW0	SLC30A7	Zinc trar	7	2	4	2	376	41.6
Q9H0U6	MRPL18	39S ribos	18	3	3	3	180	20.6
Q9UK45	LSM7	U6 snRNA-	48	4	6	4	103	11.6
Q99519	NEU1	Sialidase	10	4	4	4	415	45.4
P59768	GNG2	Guanine n	39	3	4	3	71	7.8
Q96RD7	PANX1	Pannexin-	8	2	2	2	426	48
J3QQZ9	PNPO	Pyridoxir	15	3	3	3	238	27.3
Q9Y6C9	MTCH2	Mitochonc	14	4	4	4	303	33.3
Q96MX6	WDR92	WD repeat	9	3	3	3	357	39.7
Q96P16	RPRD1A	Regulatic	6	2	3	2	312	35.7
P49406	MRPL19	39S ribos	14	3	4	3	292	33.5
O00401	WASL	Neural Wi	8	4	4	4	505	54.8

Q7Z3T8	ZFYVE16	Zinc fing	3	4	4	4	1539	168.8
U3KQG5	CD200	OX-2 memb	13	2	3	2	202	22.5
Q8NBX0	SCCPDH	Saccharop	8	3	5	3	429	47.1
Q13488	TCIRG1	V-type pr	4	3	3	3	830	92.9
Q9UPN7	PPP6R1	Serine/th	5	3	3	3	881	96.7
P23258	TUBG1	Tubulin g	7	3	4	3	451	51.1
Q9BXR0	QTRT1	Queuine t	7	3	3	3	403	44
P08962	CD63	CD63 anti	10	3	8	3	238	25.6
P53365	ARFIP2	Arfaptin-	10	3	3	3	341	37.8
Q9Y3L3	SH3BP1	SH3 domai	4	3	3	3	701	75.7
Q5JSZ5	PRRC2B	Protein F	2	4	4	4	2229	242.8
Q96T88	UHRF1	E3 ubiqui	5	3	3	3	793	89.8
Q8NBQ5	HSD17B11	Estradiol	11	3	3	3	300	32.9
Q86U86	PBRM1	Protein p	2	3	3	3	1689	192.8
F5GXX5	DAD1	Dolichyl-	26	2	3	2	85	9.5
O14734	ACOT8	Acyl-coen	13	3	3	3	319	35.9
Q7L099	RUFY3	Protein R	8	4	4	3	469	52.9
P01137	TGFB1	Transform	8	3	3	3	390	44.3
Q14653	IRF3	Interfero	8	3	4	3	427	47.2
Q96FV9	THOC1	THO compl	4	3	4	3	657	75.6
Q8ND76	CCNY	Cyclin-Y	9	3	4	3	341	39.3
Q15007	WTAP	Pre-mRNA-	9	3	3	3	396	44.2
Q16540	MRPL23	39S ribos	10	1	3	1	153	17.8
J3QRU8	GIT1	ARF GTPas	6	5	6	3	694	76.8
Q9BT09	CNPY3	Protein c	11	4	4	4	278	30.7
Q9HCE0	EPG5	Ectopic P	2	3	3	3	2579	292.3
Q9Y2S7	POLDIP2	Polymeras	7	3	5	3	368	42
P09914	IFIT1	Interfero	7	3	3	3	478	55.3
Q8WUF5	PPP1R13L	RelA-asso	4	3	3	3	828	89
Q86SF2	GALNT7	N-acetylg	5	3	4	3	657	75.3
K7EK35	STAT5A	Signal tr	4	3	3	3	763	87.3
Q8NEZ5	FBXO22	F-box onl	7	3	4	3	403	44.5
Q13043	STK4	Serine/th	7	3	3	3	487	55.6
A0A1W2PNF	IER3IP1	Immediate	29	1	2	1	69	7.6
O15231	ZNF185	Zinc fing	6	4	4	4	689	73.5
Q5J8M3	EMC4	ER membra	21	3	3	3	183	20.1
Q8N9N2	ASCC1	Activatin	7	3	3	3	400	45.5
Q9HCD5	NCOA5	Nuclear r	8	4	4	4	579	65.5
H7C3J3	THUMPD3	THUMP dom	10	3	3	3	269	30.3
P15529	CD46	Membrane	8	3	5	3	392	43.7
V5IRT4	UQCC2	Ubiquinol	10	1	2	1	126	14.8
A0A0B4J2C		Uncharact	6	4	5	2	849	94.6
P49137	MAPKAPK2	MAP kinas	8	4	4	4	400	45.5
Q9BU23	LMF2	Lipase ma	6	3	3	3	707	79.6
Q9Y6C2	EMILIN1	EMILIN-1	5	4	4	4	1016	106.6
E9PJH7	SLC25A22	Mitochond	10	3	3	3	313	33.3
Q12899	TRIM26	Tripartit	5	3	3	3	539	62.1
Q9NZM5	NOP53	Ribosome	8	3	3	3	478	54.4
A0A1B0GU5	PCCA	Propionyl	5	3	3	3	615	67.3
P49184	DNASE1L1	Deoxyribo	11	3	3	3	302	33.9
Q9BYM8	RBCK1	RanBP-typ	7	3	3	3	510	57.5

A0A1B0GTE	ATP6AP2	Renin rec	10	3	3	3	294	33
Q69YN2	CWF19L1	CWF19-lik	7	3	3	3	538	60.6
O95456	PSMG1	Proteason	16	4	4	4	288	32.8
Q14728	MFSD10	Major fac	5	2	3	2	455	48.3
Q9UNK0	STX8	Syntaxin-	17	3	3	3	236	26.9
Q53T59	HS1BP3	HCLS1-bir	4	1	2	1	392	42.8
Q12979	ABR	Active br	3	2	4	2	859	97.5
A0A140TA8	C19orf70	MICOS com	33	3	4	3	140	15.4
Q13671	RIN1	Ras and R	8	3	3	3	783	84
P30825	SLC7A1	High affi	7	3	4	3	629	67.6
E7EWE1	UBA5	Ubiquitir	9	3	4	3	347	38.7
Q9UII2	ATP5IF1	ATPase in	15	3	6	3	106	12.2
Q8IV38	ANKMY2	Ankyrin r	8	4	4	4	441	49.3
Q9BTU6	PI4K2A	Phosphati	7	4	4	4	479	54
E9PHT6	PANK4	Pantother	5	3	3	3	737	81.7
C9JE79	CALD1	Caldesmor	43	2	6	1	72	9
C9JCC6	DRAP1	Dr1-assoc	14	3	3	3	212	23.2
Q9Y2P8	RCL1	RNA 3'-te	10	4	4	4	373	40.8
Q9H330	TMEM245	Transmemb	4	4	5	4	879	97.3
Q96HY6	DDRGK1	DDRGK dom	8	2	3	2	314	35.6
G3V325	ATP5MF-PT	ATP5MF-PT	3	2	3	2	749	84.1
O95777	LSM8	U6 snRNA-	35	2	2	2	96	10.4
Q96QG7	MTMR9	Myotubula	7	3	3	3	549	63.4
Q9H6S0	YTHDC2	3'-5' RNA	3	5	5	4	1430	160.1
O00767	SCD	Acyl-CoA	4	1	2	1	359	41.5
A0A0A0MTE	WDR36	WD repeat	4	3	3	3	895	99.3
Q969E2	SCAMP4	Secretory	8	2	5	2	229	25.7
O00214	LGALS8	Galectin-	9	3	3	3	317	35.8
O00178	GTPBP1	GTP-bindi	5	3	3	3	669	72.4
O15258	RER1	Protein R	13	3	6	3	196	22.9
V9GZ56	LSM4	U6 snRNA-	10	3	5	3	238	25.7
Q9NRX5	SERINC1	Serine in	6	2	3	2	453	50.5
Q6P158	DHX57	Putative	2	3	3	2	1386	155.5
J3KQ18	DDT	D-dopachr	14	2	7	2	132	14.2
E1CEI4	GCLC	Glutamate	7	4	4	4	599	68.6
M0QZ21	AP2S1	AP comple	14	2	5	2	122	14.5
Q3KQV9	UAP1L1	UDP-N-ace	5	3	7	2	507	57
Q8NHP8	PLBD2	Putative	5	4	5	4	589	65.4
Q9NVV4	MTPAP	Poly(A) R	5	4	4	4	582	66.1
Q9GZP4	PITHD1	PITH doma	23	4	4	4	211	24.2
Q8N5C1	CALHM5	Calcium h	11	3	3	3	309	35.1
Q9H6U8	ALG9	Alpha-1,2	6	3	3	3	611	69.8
Q8NFH5	NUP35	Nucleopor	10	3	3	3	326	34.8
A0A087WZT	SAMD4B	Protein S	7	4	4	4	663	72.4
O95989	NUDT3	Diphosphc	16	3	3	3	172	19.5
Q9H6F5	CCDC86	Coiled-cc	11	4	5	4	360	40.2
Q8IWZ8	SUGP1	SURP and	6	4	4	4	645	72.4
P04150	NR3C1	Glucocort	4	3	3	3	777	85.6
Q14999	CUL7	Cullin-7	2	2	2	2	1698	191
Q6NZY4	ZCCHC8	Zinc fing	5	3	3	3	707	78.5
J3QLH3	SAP30BP	SAP30-bir	15	3	3	3	248	27.7

P11441	UBL4A	Ubiquitin	16	2	3	2	157	17.8
O14910	LIN7A	Protein l	12	3	3	1	233	26
Q9NUL5	RYDEN	Repressor	10	3	3	3	291	33.1
Q9H773	DCTPP1	dCTP pyro	11	2	3	2	170	18.7
A0A0C4DGH	NUMBL	Numb-like	3	2	3	1	568	60.8
O43617	TRAPPC3	Trafficki	19	4	5	4	180	20.3
Q9NPH2	ISYNA1	Inositol-	6	4	5	4	558	61
P29084	GTF2E2	Transcrip	10	3	3	3	291	33
F8WCP5	THOC5	THO compl	8	3	4	3	343	39.7
O14735	CDIPT	CDP-diacy	13	3	4	3	213	23.5
Q13795	ARFRP1	ADP-ribos	15	3	3	3	201	22.6
P51809	VAMP7	Vesicle-a	21	4	4	4	220	24.9
P78362	SRPK2	SRSF prot	5	3	3	2	688	77.5
Q15388	TOMM20	Mitochond	19	3	4	3	145	16.3
Q9Y5J1	UTP18	U3 small	7	2	2	2	556	62
F6QUN3	ERI3	ERI1 exor	24	3	4	3	176	19.7
Q9NPA8	ENY2	Transcrip	33	4	4	4	101	11.5
Q8NHP6	MOSPD2	Motile sp	7	4	4	4	518	59.7
Q9UJF2	RASAL2	Ras GTPas	2	2	3	2	1139	128.5
E9PBY7	ZC3H11A	Zinc fing	8	4	4	4	515	57.4
A0A087WXM	BCAM	Basal cel	6	3	3	3	588	63.7
Q9BW91	NUDT9	ADP-ribos	8	3	4	3	350	39.1
Q9NRX1	PNO1	RNA-bindi	15	3	3	3	252	27.9
Q5VIR6	VPS53	Vacuolar	6	5	5	5	699	79.6
Q9NRP0	OSTC	Oligosacc	13	2	6	2	149	16.8
E7EW05	SDAD1	Protein S	4	3	3	3	650	75.4
P24941	CDK2	Cyclin-de	11	3	4	1	298	33.9
Q9BTZ2	DHRS4	Dehydroge	12	4	4	4	278	29.5
Q4G0N4	NADK2	NAD kinas	7	3	3	3	442	49.4
Q9BS40	LXN	Latexin (	9	2	4	2	222	25.7
Q9GZV5	WWTR1	WW domain	10	5	5	5	400	44.1
O00499	BIN1	Myc box-d	9	3	3	3	593	64.7
P50135	HNMT	Histamine	15	3	3	3	292	33.3
Q96KB5	PBK	Lymphokin	8	3	4	3	322	36.1
Q9NXS2	QPCTL	Glutaminy	8	3	3	3	382	42.9
P55290	CDH13	Cadherin-	7	5	6	5	713	78.2
Q8N3F8	MICALL1	MICAL-lik	3	3	4	3	863	93.4
A8MUM1	EIPR1	EARP-inte	7	3	3	3	414	46.3
O43719	HTATSF1	HIV Tat-s	5	4	4	4	755	85.8
O60232	SSSCA1	Sjoegren	16	3	3	3	199	21.5
Q14767	LTBP2	Latent-tr	2	3	3	3	1821	194.9
D6RCP9	DCK	Deoxycyti	13	3	3	3	199	22.9
P08476	INHBA	Inhibin b	6	3	3	3	426	47.4
J3QR68	HP	Haptoglob	3	1	2	1	404	45
O43815	STRN	Striatin	4	4	4	3	780	86.1
A0A0A0MRC	PTPN9	Tyrosine-	6	3	3	3	583	66.9
Q5VTR2	RNF20	E3 ubiqui	4	5	6	5	975	113.6
Q9H000	MKRN2	Probable	7	3	3	3	416	46.9
O75063	FAM20B	Glycosami	8	2	2	2	409	46.4
P98179	RBM3	RNA-bindi	25	3	4	3	157	17.2
Q9UNN8	PROCR	Endotheli	5	1	2	1	238	26.7

P38435	GGCX	Vitamin K	5	3	3	3	758	87.5
P50613	CDK7	Cyclin-de	9	3	3	3	346	39
O95400	CD2BP2	CD2 antig	12	3	4	3	341	37.6
I3L2L5	MCRIP1	Mapk-regu	24	2	3	2	92	10.5
O60331	PIP5K1C	Phosphati	5	3	3	2	668	73.2
P17302	GJA1	Gap junct	4	1	3	1	382	43
K7ERY2	YIF1B	Protein Y	16	2	2	2	231	25.1
P63165	SUMO1	Small ubi	39	5	5	5	101	11.6
F8WCT7	SLC35F6	Solute ca	27	1	2	1	51	5.7
A0A2R8Y4D	ARHGEF17	Rho guani	3	3	3	3	1044	114.1
Q6P3X3	TTC27	Tetratric	4	4	4	4	843	96.6
A0A0A0MQX	MYO10	Unconvent	1	3	6	2	2069	238.4
A8K727	PLEKHA2	Pleckstri	7	3	3	3	425	47.2
Q9Y3D8	AK6	Adenylate	15	3	3	3	172	20
P51970	NDUFA8	NADH dehy	20	3	4	3	172	20.1
P56377	AP1S2	AP-1 comp	18	3	3	3	157	18.6
F5GXS0	C4B	Complemen	1	3	4	3	1698	187.6
Q13033	STRN3	Striatin-	4	3	3	2	797	87.2
Q7Z4N8	P4HA3	Prolyl 4-	6	3	3	3	544	61.1
P48200	IREB2	Iron-resp	3	3	3	3	963	105
E7EWX8	MGLL	Monoglyce	9	2	3	2	277	30.7
O75157	TSC22D2	TSC22 dom	3	3	4	1	780	79.2
D6RAA6	TMEM33	Transmemb	12	3	5	3	222	25.2
P41226	UBA7	Ubiquitin	4	3	3	3	1012	111.6
Q66K14	TBC1D9B	TBC1 doma	2	2	3	2	1250	140.4
Q9UIC8	LCMT1	Leucine c	7	2	3	2	334	38.4
E9PQW1	CARD16	Caspase r	27	3	3	1	95	10.3
O15530	PDPK1	3-phospho	7	3	3	3	556	63.1
Q9UBB9	TFIP11	Tuftelin-	5	3	3	3	837	96.8
Q9NWZ3	IRAK4	Interleuk	6	3	3	3	460	51.5
Q7LGA3	HS2ST1	Heparan s	9	3	5	3	356	41.9
Q9GZM5	YIPF3	Protein Y	5	2	3	2	350	38.2
H3BQI7	HSDL1	Inactive	12	1	2	1	108	12.1
P51151	RAB9A	Ras-relat	12	2	2	2	201	22.8
Q8IXM3	MRPL41	39S ribos	19	2	3	2	137	15.4
Q9BV19	C1orf50	Uncharact	16	2	2	2	199	21.9
B7WPL0	RIC8B	Synembryn	4	2	3	1	560	63.5
J9JIE6	TMCO1	Calcium l	10	3	4	3	239	27.1
Q7Z7F7	MRPL55	39S ribos	24	3	3	3	128	15.1
P33897	ABCD1	ATP-bindi	4	3	3	2	745	82.9
Q9UJX2	CDC23	Cell divi	6	3	3	3	597	68.8
Q15274	QPRT	Nicotinat	12	4	5	4	297	30.8
Q9H6X2	ANTXR1	Anthrax t	6	3	3	2	564	62.7
O95980	RECK	Reversion	4	4	4	4	971	106.4
C9JST7	YIF1A	Protein Y	6	1	2	1	199	21.7
O95425	SVIL	Supervill	2	3	3	3	2214	247.6
Q13232	NME3	Nucleosid	15	3	4	3	169	19
Q4TT34	NME4	Nucleosid	15	2	2	2	195	21.5
A0A2R8YDQ		Uncharact	5	3	3	3	599	68.7
Q9NXH9	TRMT1	tRNA (gua	5	2	2	2	659	72.2
Q9NPA0	EMC7	ER membra	13	3	4	3	242	26.5

Q9H013	ADAM19	Disintegr	4	3	3	3	955	104.9
Q08722	CD47	Leukocyte	9	3	4	3	323	35.2
A0A0X1KG7	NELFB	Negative	4	3	3	3	628	70
Q5VZM2	RRAGB	Ras-relat	7	3	4	3	374	43.2
Q5T1J5	CHCHD2P9	Putative	9	1	2	1	151	15.5
P49356	FNTB	Protein f	6	2	2	2	437	48.7
C9JLU1	POLR2H	DNA-direc	11	2	3	2	148	16.9
Q96LW7	CARD19	Caspase r	11	3	4	3	228	25.6
Q86WB0	ZC3HC1	Nuclear-i	7	3	3	3	502	55.2
Q8TDB4	MGARP	Protein M	11	3	3	3	240	25.4
P82932	MRPS6	28S ribos	22	3	3	3	125	14.2
A0A0D9SG7	UBAP1	Ubiquitin	8	4	4	4	528	57.8
E9PBC1	EPN2	Epsin-2 (	4	2	3	2	484	53
Q6RW13	AGTRAP	Type-1 an	14	1	2	1	159	17.4
Q12846	STX4	Syntaxin-	12	3	3	3	297	34.2
Q8NBI6	XXYLT1	Xyloside	10	4	4	4	393	43.8
Q13257	MAD2L1	Mitotic s	16	4	4	4	205	23.5
O95166	GABARAP	Gamma-ami	15	2	3	1	117	13.9
Q9UJX4	ANAPC5	Anaphase-	4	2	2	2	755	85
Q96BH1	RNF25	E3 ubiqui	5	2	3	2	459	51.2
P02649	APOE	Apolipopr	9	3	4	3	317	36.1
P47712	PLA2G4A	Cytosolic	5	4	4	4	749	85.2
A8MX75	ERCC2	General t	3	2	3	2	706	80.5
O60551	NMT2	Glycylpep	7	4	4	1	498	56.9
Q86YS6	RAB43	Ras-relat	17	3	3	2	212	23.3
X6R2S6	SPCS1	Signal pe	10	2	4	2	169	18.3
P35244	RPA3	Replicati	24	2	3	2	121	13.6
Q96BP3	PPWD1	Peptidylp	6	4	4	4	646	73.5
Q9Y2Z4	YARS2	Tyrosine-	12	4	4	4	477	53.2
O15213	WDR46	WD repeat	4	2	2	2	610	68
O95248	SBF1	Myotubula	1	3	3	3	1868	208.3
Q9H4L7	SMARCAD1	SWI/SNF-r	2	2	2	2	1026	117.3
Q8WXI9	GATAD2B	Transcrip	3	2	3	1	593	65.2
Q92925	SMARCD2	SWI/SNF-r	6	2	2	2	531	58.9
Q8TD55	PLEKHO2	Pleckstri	6	2	2	2	490	53.3
Q9BV79	MECR	Enoyl-[ac	6	2	3	2	373	40.4
J3QL71	SCRN2	Secernin-	6	2	2	2	433	47.5
Q5EBL4	RILPL1	RILP-like	9	3	3	3	403	47.1
Q8WVQ1	CANT1	Soluble c	8	3	3	3	401	44.8
S4R347	FNBP1L	Formin-bi	7	4	4	3	609	70.5
Q03405	PLAUR	Urokinase	9	3	4	3	335	37
P08651	NFIC	Nuclear f	5	2	2	2	508	55.6
A0A2R8YGH	AP1S1	AP comple	18	3	3	3	157	18.6
Q9BQ70	TCF25	Transcrip	6	3	3	3	676	76.6
Q9H1E5	TMX4	Thioredox	7	2	2	2	349	38.9
H0Y8X6	NEDD4	E3 ubiqui	4	3	3	3	910	104.7
Q8IWA4	MFN1	Mitofusin	4	3	3	2	741	84.1
H0YN65	CHST14	Carbohydr	10	4	4	4	351	39.9
P17405	SMPD1	Sphingomy	4	3	3	3	629	69.7
Q13042	CDC16	Cell divi	5	3	3	3	620	71.6
A0A087WYV	SYNJ2BP-C	SYNJ2BP-C	22	3	4	3	182	20.5

Q03519	TAP2	Antigen p	5	4	4	4	686	75.6
Q9GZQ8	MAP1LC3B	Microtubu	22	3	4	3	125	14.7
O94829	IPO13	Importin-	5	3	3	3	963	108.1
O95671	ASMTL	N-acetyls	5	4	4	4	621	68.8
A0A0A0MQV	FGF2	Fibroblas	10	3	3	3	288	30.7
Q8TEA8	DTD1	D-aminoac	11	2	2	2	209	23.4
O60306	AQR	RNA helic	3	4	4	4	1485	171.2
Q5SZE1	CERS2	Ceramide	11	3	3	3	304	36.4
B5MD46	TBC1D10A	TBC1 doma	6	3	3	3	420	47.9
P51784	USP11	Ubiquitin	3	3	3	3	963	109.7
O96007	MOCS2	Molybdopt	14	2	2	2	188	20.9
P62837	UBE2D2	Ubiquitin	12	2	7	2	147	16.7
Q66LE6	PPP2R2D	Serine/th	10	3	3	1	453	52
A3KFL1	EXOSC2	Exosome c	17	3	4	3	200	22.1
H7C2N1	PTMA	Prothymos	9	1	2	1	148	15.8
Q9BY67	CADM1	Cell adhe	6	3	3	3	442	48.5
P86790	CCZ1B	Vacuolar	7	3	3	3	482	55.8
Q99418	CYTH2	Cytohesin	6	2	2	2	400	46.5
Q9NQH7	XPNPEP3	Xaa-Pro a	6	2	2	2	507	57
Q9HCS7	XAB2	Pre-mRNA-	6	4	4	4	855	99.9
A0A087WZX	NDUFB6	NADH dehy	26	2	4	2	97	11.7
Q96CP2	FLYWCH2	FLYWCH fa	22	2	2	2	140	14.6
Q9BT22	ALG1	Chitobios	8	3	3	3	464	52.5
Q9BV20	MRI1	Methylthi	8	3	3	3	369	39.1
O43676	NDUFB3	NADH dehy	18	2	6	2	98	11.4
H3BSE1	TBC1D10B	TBC1 doma	14	3	3	3	184	21.1
M0R1E3	NAT14	N-acetylt	17	2	2	2	173	17.8
P08253	MMP2	72 kDa ty	6	3	3	3	660	73.8
Q13017	ARHGAP5	Rho GTPas	2	4	4	4	1502	172.4
C9JEL3	EIF4E2	Eukaryoti	11	2	3	2	213	24.6
Q8IXI2	RHOT1	Mitochond	3	2	3	1	618	70.7
Q92979	EMG1	Ribosomal	11	2	2	2	244	26.7
G8JLK1	USE1	Vesicle t	10	3	4	3	256	29.1
Q9BZJ0	CRNKL1	Crooked n	2	2	2	2	848	100.4
H3BTP8	FAM192A	Protein F	24	2	2	2	130	16
Q8NFP7	NUDT10	Diphospho	18	2	2	1	164	18.5
B1APM4	SOAT1	Sterol O-	12	2	2	2	260	29.9
Q9GZP9	DERL2	Derlin-2	17	2	2	2	239	27.5
Q9NZD8	SPG21	Maspardin	12	3	3	3	308	34.9
Q6PD74	AAGAB	Alpha- an	9	3	3	3	315	34.6
A0A096LNH	DOCK1	Dedicator	2	3	3	2	1886	217.6
A0A0B4J1V	PPAN-P2RY	HCG203999	3	3	4	3	794	87.9
Q8N5N7	MRPL50	39S ribos	15	2	2	2	158	18.3
Q5T160	RARS2	Probable	5	3	3	3	578	65.5
O95149	SNUPN	Snurporti	9	4	4	3	360	41.1
F8WCT1	ARL6IP4	ADP-ribos	5	1	2	1	229	25.6
Q13641	TPBG	Trophobla	6	3	3	3	420	46
P34949	MPI	Mannose-6	12	3	3	3	423	46.6
A0A0J9YX6	DNAJB6	DnaJ homo	7	3	3	2	334	36.6
Q9BWH2	FUNDC2	FUN14 dom	13	2	2	2	189	20.7
Q8IWA0	WDR75	WD repeat	4	3	3	3	830	94.4

P51884	LUM	Lumican C	9	3	3	3	338	38.4
P16455	MGMT	Methylate	18	3	4	3	207	21.6
Q53FP2	TMEM35A	Transmemb	14	2	3	2	167	18.4
K7ERC8	KDSR	3-ketodih	18	4	4	4	298	32.7
Q8N3X1	FNBP4	Formin-bi	3	3	3	3	1017	110.2
P56381	ATP5F1E	ATP synth	59	4	6	4	51	5.8
Q9H0V9	LMAN2L	VIP36-lik	10	3	3	3	348	39.7
O95197	RTN3	Reticulon	2	2	3	2	1032	112.5
A0A2R8Y6C	CLPB	Caseinoly	8	2	2	1	382	43.3
Q9Y487	ATP6V0A2	V-type pr	5	4	4	4	856	98
E7EX70	POLR1E	DNA-direc	23	3	3	1	141	15.9
Q9Y3B9	RRP15	RRP15-lik	10	3	4	3	282	31.5
F5H225	EOGT	EGF domai	13	3	3	3	206	24.1
Q92990	GLMN	Glomulin	4	3	3	3	594	68.2
O00391	QSOX1	Sulfhydry	5	3	3	3	747	82.5
O00443	PIK3C2A	Phosphati	1	1	2	1	1686	190.6
P00374	DHFR	Dihydrofc	14	2	2	2	187	21.4
Q9GZT6	CCDC90B	Coiled-cc	7	2	2	2	254	29.5
Q96C23	GALM	Aldose 1-	14	4	4	4	342	37.7
Q5R3I4	TTC38	Tetratric	9	4	4	4	469	52.8
Q5TH30	NDRG3	NDRG fami	5	2	2	2	388	42.8
Q8NBJ4	GOLM1	Golgi mem	6	4	5	4	401	45.3
Q4G0J3	LARP7	La-relate	5	4	4	4	582	66.9
G8JLB3	PUS1	tRNA pseu	8	3	3	3	384	42.9
Q14684	RRP1B	Ribosomal	5	3	3	3	758	84.4
P36404	ARL2	ADP-ribos	11	2	4	2	184	20.9
Q92871	PMM1	Phosphoma	15	4	4	3	262	29.7
Q13049	TRIM32	E3 ubiqui	3	2	2	2	653	71.9
Q9UBK9	UXT	Protein U	20	3	3	3	157	18.2
B8ZZ77	PPIL3	Peptidyl-	18	3	3	3	157	17.7
P05412	JUN	Transcrip	13	4	4	4	331	35.7
A6PW57	PIP5K1A	Phosphati	5	3	3	2	550	61.2
Q14739	LBR	Lamin-B r	6	4	4	4	615	70.7
P53985	SLC16A1	Monocarbc	4	3	3	3	500	53.9
Q15750	TAB1	TGF-beta-	7	3	3	3	504	54.6
H7C0B3	TMEM87B	Transmemb	15	3	3	3	184	21.4
Q9NP77	SSU72	RNA polym	12	2	2	2	194	22.6
Q495W5	FUT11	Alpha-(1,	7	3	3	3	492	55.8
B9ZVT1	RBM12B	RNA-bindi	4	3	3	3	881	102.6
F8W1S1	KRT74	Keratin,	5	3	10	1	543	59.4
P85037	FOXK1	Forkhead	4	4	5	4	733	75.4
A0A024R6I	SERPINA1	Alpha-1-a	5	2	2	2	418	46.7
O14925	TIMM23	Mitochond	17	3	3	3	209	21.9
A0A1B0GUA	KIF13A	Kinesin-l	1	2	2	2	1845	207.1
O60513	B4GALT4	Beta-1,4-	13	4	4	4	344	40
Q5T653	MRPL2	39S ribos	7	2	2	2	305	33.3
Q14112	NID2	Nidogen-2	2	3	3	3	1375	151.2
Q9BSH4	TACO1	Translati	10	2	2	2	297	32.5
P49459	UBE2A	Ubiquitin	18	2	2	2	152	17.3
Q9BUT1	BDH2	3-hydroxy	12	3	3	3	245	26.7
Q8IYB8	SUPV3L1	ATP-depen	2	1	2	1	786	87.9

O14929	HAT1	Histone a	8	3	4	3	419	49.5
O75607	NPM3	Nucleopla	26	4	4	4	178	19.3
A0A0B4J23	IGLL5	Immunoglo	7	1	2	1	215	23.1
Q5VWZ2	LYPLAL1	Lysophosp	10	3	4	3	237	26.3
Q96F24	NRBF2	Nuclear r	9	3	3	3	287	32.4
O75531	BANF1	Barrier-t	36	2	3	2	89	10.1
O95235	KIF20A	Kinesin-l	3	2	2	2	890	100.2
Q8TED0	UTP15	U3 small	6	3	3	3	518	58.4
P53384	NUBP1	Cytosolic	8	2	3	2	320	34.5
Q5VXN0	RPF2	Ribosome	12	3	3	3	214	24.6
Q9UHD2	TBK1	Serine/th	3	3	3	1	729	83.6
Q96S44	TP53RK	EKC/KEOPS	8	2	3	2	253	28.1
Q9H0C8	ILKAP	Integrin-	6	3	3	3	392	42.9
Q9NPD3	EXOSC4	Exosome c	8	2	3	2	245	26.4
Q6ZSJ8	C1orf122	Uncharact	23	2	2	2	110	11.5
P35250	RFC2	Replicati	7	3	3	3	354	39.1
Q9UJ83	HACL1	2-hydroxy	6	3	4	3	578	63.7
P15289	ARSA	Arylsulfa	11	4	4	3	507	53.6
Q8NB16	MLKL	Mixed lin	9	2	2	2	471	54.4
Q9Y6D9	MAD1L1	Mitotic s	4	4	4	4	718	83
M0QWZ7	SARS2	Serine--t	8	3	3	3	518	58.1
Q8WUI4	HDAC7	Histone d	3	3	3	3	952	102.9
Q8IYB1	MB21D2	Protein M	4	2	2	2	491	55.8
Q9UIW2	PLXNA1	Plexin-A1	1	3	3	3	1896	210.9
Q9NVX2	NLE1	Notchless	5	2	2	2	485	53.3
E9PEP6	NRP1	Neuropili	4	2	2	2	906	101.2
E9PRK2	NARS2	Probable	8	2	2	2	241	27.2
P53609	PGGT1B	Geranylge	8	3	3	3	377	42.3
Q96J01	THOC3	THO compl	9	3	3	3	351	38.7
Q96DE0	NUDT16	U8 snoRNA	16	3	3	2	195	21.3
Q9BSC4	NOL10	Nucleolar	5	3	3	3	688	80.3
O14730	RIOK3	Serine/th	4	2	3	2	519	59.1
K7EJB0	NFIX	Nuclear f	15	2	2	2	183	21.2
M0QZD9	BRD4	Bromodoma	3	2	2	2	572	63.7
O60234	GMFG	Glia matu	13	2	2	1	142	16.8
Q7Z3B1	NEGR1	Neuronal	4	1	2	1	354	38.7
P17900	GM2A	Gangliosi	12	3	3	3	193	20.8
Q9BSL1	UBAC1	Ubiquitin	5	2	3	2	405	45.3
A0A0U1RQX	CBL	E3 ubiqui	3	3	3	3	882	96.8
P06280	GLA	Alpha-gal	8	2	2	2	429	48.7
Q9NWY4	HPF1	Histone P	6	2	2	2	346	39.4
P19634	SLC9A1	Sodium/hy	4	2	2	2	815	90.7
A6NIH7	UNC119B	Protein u	12	2	2	2	251	28.1
Q9C037	TRIM4	E3 ubiqui	5	2	2	2	500	57.4
D6RHX2	ELMOD2	ELMO doma	24	2	2	2	96	11.2
P78536	ADAM17	Disintegr	3	3	3	3	824	93
Q92968	PEX13	Peroxisom	5	2	2	2	403	44.1
O43752	STX6	Syntaxin-	9	2	2	2	255	29.2
O95749	GGPS1	Geranylge	10	3	3	3	300	34.8
J3QL56	SCO1	Protein S	15	3	3	3	270	30.2
Q8NC54	KCT2	Keratinoc	9	2	2	2	265	29.2

P55011	SLC12A2	Solute ca	3	3	3	3	1212	131.4
O15484	CAPN5	Calpain-5	5	2	3	2	640	73.1
Q96D71	REPS1	RalBP1-as	3	3	3	3	796	86.6
Q7L8J4	SH3BP5L	SH3 domai	7	3	3	3	393	43.5
O75792	RNASEH2A	Ribonucle	8	2	2	2	299	33.4
Q9H3Q1	CDC42EP4	Cdc42 eff	9	3	3	3	356	38
A0A1B0GW9	IWS1	Protein I	15	4	4	4	207	24
B5MC51	LIMK2	LIM domai	4	2	3	1	629	71.1
Q9UL26	RAB22A	Ras-relat	16	3	4	2	194	21.8
H0YAX3	MRPL13	39S ribos	51	2	2	2	47	5.8
P62308	SNRPG	Small nuc	25	2	5	2	76	8.5
P32856	STX2	Syntaxin-	8	2	2	2	288	33.3
Q5TC84	OGFRL1	Opioid gr	6	3	3	3	451	51.2
Q9BT73	PSMG3	Proteasom	23	2	3	2	122	13.1
Q6UW02	CYP20A1	Cytochrom	5	2	2	2	462	52.4
O95183	VAMP5	Vesicle-a	11	1	2	1	116	12.8
Q9C0E8	LNPK	Endoplasm	4	2	4	2	428	47.7
F5GXE4	ATE1	Arginyl-t	6	2	3	2	511	58.1
Q9NS00	C1GALT1	Glycoprot	7	2	2	2	363	42.2
Q8N999	C12orf29	Uncharact	6	2	2	2	325	37.5
E7EN73	KIAA0319L	Dyslexia-	3	3	3	3	1026	113.2
A0A2R8YEK	COL4A3BP	Collagen	5	3	3	3	567	64.3
Q14410	GK2	Glycerol	3	2	3	1	553	60.6
P52756	RBM5	RNA-bindi	3	2	2	2	815	92.1
Q6PML9	SLC30A9	Zinc tran	5	3	3	3	568	63.5
Q96II8	LRCH3	Leucine-r	3	2	3	2	777	86
Q9NSI2	FAM207A	Protein F	15	2	2	2	230	25.4
G3V4T2	PABPN1	Polyadeny	10	3	3	3	178	20.2
Q9UJ72	ANXA10	Annexin A	8	2	2	2	324	37.3
B2WTI3	JMJD6	Bifunctio	4	1	2	1	335	39.2
Q99757	TXN2	Thioredox	13	2	3	2	166	18.4
Q56VL3	OCIAD2	OCIA doma	27	4	4	4	154	16.9
A0A0C4DGS	NDUFA6	NADH dehy	19	3	4	3	128	15.1
A0A024RAC	ELOA	Elongin-A	4	3	3	3	772	87.2
Q9UBL3	ASH2L	Set1/Ash2	4	2	2	2	628	68.7
A0A0A0MTC	STAU2	Double-st	7	3	3	3	504	55.4
Q93096	PTP4A1	Protein t	18	3	3	1	173	19.8
Q9H9T3	ELP3	Elongator	6	3	3	3	547	62.2
H0YLF3	B2M	Beta-2-mi	14	1	3	1	71	8.5
Q9H061	TMEM126A	Transmemb	11	2	2	2	195	21.5
O14613	CDC42EP2	Cdc42 eff	13	2	2	2	210	22.5
Q8WUJ3	CEMIP	Cell migr	2	3	3	3	1361	152.9
Q86X83	COMMD2	COMM doma	12	3	3	3	199	22.7
A0A2R8YDT	GLUL	Glutamine	5	3	3	3	507	57.1
Q8WVJ2	NUDCD2	NudC doma	18	2	2	2	157	17.7
P06702	S100A9	Protein S	18	2	3	2	114	13.2
E5RHP7	CA1	Carbonic	5	1	3	1	251	27.7
Q99735	MGST2	Microsoma	10	1	2	1	147	16.6
Q8TAD7	OCC1	Overexpre	21	1	1	1	63	6.4
O14662	STX16	Syntaxin-	10	3	4	3	325	37
O00488	ZNF593	Zinc fing	17	2	2	2	134	15.2

Q9Y4C8	RBM19	Probable	2	2	2	2	960	107.3
Q9BXW6	OSBPL1A	Oxysterol	3	2	2	2	950	108.4
Q8TBM8	DNAJB14	DnaJ homo	6	2	2	2	379	42.5
Q9UNH6	SNX7	Sorting n	9	4	4	4	387	45.3
Q8IZV5	RDH10	Retinol d	9	3	3	3	341	38.1
F8W038	C17orf49	Chromosom	13	2	4	2	157	16.3
Q93100	PHKB	Phosphory	2	3	3	3	1093	124.8
Q9NUJ3	TCP11L1	T-complex	5	2	2	2	509	57
Q92947	GCDH	Glutaryl-	6	3	3	3	438	48.1
Q6PI48	DARS2	Aspartate	4	3	3	3	645	73.5
J3KSG2	SPECC1	Cytospin-	5	2	2	2	534	60.1
E5RJY0	PIK3R1	Phosphati	8	1	2	1	137	16.3
Q9NSQ0	RRP7BP	Putative	22	2	2	2	103	12.6
H3BUU9	CDH11	Cadherin-	4	2	3	2	670	73.7
O43427	FIBP	Acidic fi	5	2	2	2	364	41.9
Q5T440	IBA57	Putative	13	2	2	2	356	38.1
E9PDN5	DMD	Dystrophi	1	2	2	1	3681	425.8
Q13614	MTMR2	Myotubula	4	3	3	3	643	73.3
P57772	EEFSEC	Selenocys	4	2	2	2	596	65.3
O95071	UBR5	E3 ubiqui	1	3	3	3	2799	309.2
P00846	MT-ATP6	ATP synth	4	1	2	1	226	24.8
E9PIT5	SSH3	Protein p	6	1	2	1	219	24.7
Q969Q5	RAB24	Ras-relat	13	2	2	2	203	23.1
P08574	CYC1	Cytochrom	7	2	3	2	325	35.4
Q96MW1	CCDC43	Coiled-co	11	3	3	3	224	25.2
Q96B36	AKT1S1	Proline-r	5	1	2	1	256	27.4
A0A2R8Y4N	SPG7	Paraplegi	5	2	2	2	383	42.8
Q9Y2C4	EXOG	Nuclease	9	3	3	3	368	41.1
H3BVH7	RAB27A	Ras-relat	14	2	2	2	146	16.7
B1AKC9	EPHB2	Ephrin ty	2	2	2	1	946	105.5
A0A087X1U	MECP2	Methyl-Cp	6	2	2	2	324	34.9
Q96GD0	PDXP	Pyridoxal	8	2	2	2	296	31.7
Q9BTE3	MCMBP	Mini-chro	4	3	3	3	642	72.9
A0A087WTU	TEX264	Testis-ex	10	2	2	2	239	26
I3L2C7	GEMIN4	Gem-assoc	2	2	2	2	1047	118.7
Q8WUH1	CHURC1	Protein C	15	2	3	2	139	16.1
P30536	TSPO	Transloca	9	2	4	2	169	18.8
Q3ZCW2	LGALSL	Galectin-	17	2	2	2	172	19
Q9Y333	LSM2	U6 snRNA-	27	2	2	2	95	10.8
Q9BRG1	VPS25	Vacuolar	10	2	3	2	176	20.7
O75380	NDUFS6	NADH dehy	12	1	1	1	124	13.7
Q96CP6	GRAMD1A	GRAM doma	2	2	2	2	724	80.6
P82664	MRPS10	28S ribos	9	1	2	1	201	23
Q9H2K8	TAOK3	Serine/th	3	3	4	2	898	105.3
Q9NQT5	EXOSC3	Exosome c	8	2	2	2	275	29.6
O43709	BUD23	Probable	11	2	2	2	281	31.9
P51668	UBE2D1	Ubiquitin	7	1	2	1	147	16.6
O94806	PRKD3	Serine/th	2	2	2	2	890	100.4
P0CG30	GSTT2B	Glutathio	13	2	3	2	244	27.5
Q86SR1	GALNT10	Polypepti	4	3	4	3	603	68.9
Q96QC0	PPP1R10	Serine/th	3	4	4	4	940	99

C9J1S9	BCS1L	Mitochond	15	2	2	2	150	17.1
Q5QJE6	DNTTIP2	Deoxynucl	3	2	2	2	756	84.4
Q96EY8	MMAB	Cob(I)yri	13	2	3	2	250	27.4
Q96QZ7	MAGI1	Membrane-	2	2	2	1	1491	164.5
H0YNI7	TLE3	Transduci	6	2	2	2	353	38.3
A0A087WZ1	RAVER1	Ribonucle	4	2	2	2	739	77.8
Q9UJA5	TRMT6	tRNA (ade	6	3	3	3	497	55.8
Q9NXG6	P4HTM	Transmemb	3	1	2	1	502	56.6
Q9GZQ3	COMMD5	COMM doma	10	2	2	2	224	24.7
O95273	CCNDBP1	Cyclin-D1	4	1	2	1	360	40.2
A0A087WUC	ERGIC2	Endoplasm	7	3	4	3	386	43.4
E7ESK6	SDC2	Syndecan	15	2	2	2	165	18.4
Q9Y2R4	DDX52	Probable	4	2	2	2	599	67.5
Q9ULX3	NOB1	RNA-bindi	6	3	3	3	412	46.6
A1A4S6	ARHGAP10	Rho GTPas	3	2	2	2	786	89.3
Q9UH62	ARMCX3	Armadillo	8	3	3	3	379	42.5
A0A0G2JNK	TSEN34	tRNA-spli	10	2	3	2	252	27.3
Q8NBU5	ATAD1	ATPase fa	6	2	2	2	361	40.7
P26374	CHML	Rab prote	3	2	2	1	656	74
P28799	GRN	Granulins	5	3	3	3	593	63.5
Q9NUG6	PDRG1	p53 and D	10	1	2	1	133	15.5
Q96GA3	LTV1	Protein L	6	2	2	2	475	54.8
Q9UPU5	USP24	Ubiquitin	1	2	2	2	2620	294.2
Q68E01	INTS3	Integrato	2	2	2	2	1043	118
P49721	PSMB2	Proteasom	12	3	5	3	201	22.8
Q8WU76	SCFD2	Sec1 fami	4	2	2	2	684	75.1
K4DI92	RWDD4	RWD domai	10	2	2	2	187	21.1
P82914	MRPS15	28S ribos	7	2	2	2	257	29.8
Q96L35	EPHB4	EPH recep	3	2	2	1	935	102.5
Q9UNZ5	C19orf53	Leydig ce	17	2	2	2	99	10.6
P78381	SLC35A2	UDP-galac	3	1	2	1	396	41.3
Q9GZT4	SRR	Serine ra	9	2	2	2	340	36.5
P24386	CHM	Rab prote	2	2	2	1	653	73.4
Q92629	SGCD	Delta-sar	8	2	2	2	289	32.1
Q9HBL7	PLGRKT	Plasminog	18	3	3	3	147	17.2
P0DPB6	POLR1D	DNA-direc	16	2	2	2	133	15.2
Q9Y5J6	TIMM10B	Mitochond	23	2	2	2	103	11.6
Q9Y5A7	NUB1	NEDD8 ult	3	2	2	2	615	70.5
P14174	MIF	Macrophag	14	2	5	2	115	12.5
Q9Y6M7	SLC4A7	Sodium bi	2	2	3	2	1214	136
E9PC69	MARK2	Non-speci	2	2	2	2	778	86.6
Q13158	FADD	FAS-assoc	10	2	2	2	208	23.3
J3KNN5	DDX41	Probable	4	3	3	3	640	71.6
C9JRD2	DNAJB2	DnaJ homo	11	2	2	1	228	25.4
Q13541	EIF4EBP1	Eukaryoti	22	3	3	3	118	12.6
A0A0B4J28	TRAPPC2L	Trafficki	21	3	4	3	109	12.7
P11274	BCR	Breakpoin	2	3	3	3	1271	142.7
Q9UIV1	CNOT7	CCR4-NOT	8	2	2	2	285	32.7
P61964	WDR5	WD repeat	8	2	2	2	334	36.6
Q96EL3	MRPL53	39S ribos	21	2	2	2	112	12.1
Q86U44	METTL3	N6-adenos	4	2	2	2	580	64.4

Q9HBM1	SPC25	Kinetocho	10	2	3	2	224	26.1
A0A087WTC	CBWD1	COBW doma	15	2	2	2	160	17.4
P10398	ARAF	Serine/th	4	2	2	2	606	67.5
Q92997	DVL3	Segment p	3	2	2	2	716	78
P16591	FER	Tyrosine-	2	2	2	2	822	94.6
H7C5T8	MAGI1	Membrane-	3	2	2	1	1014	110.7
Q9BRT3	MIEN1	Migration	16	2	3	2	115	12.4
Q9BUK6	MSTO1	Protein m	7	3	3	3	570	61.8
Q9UPN9	TRIM33	E3 ubiqui	2	2	2	2	1127	122.5
Q9BRT6	LLPH	Protein L	19	2	2	2	129	15.2
Q9BXB4	OSBPL11	Oxysterol	3	2	3	2	747	83.6
P24468	NR2F2	COUP tran	6	3	3	2	414	45.5
P10746	UROS	Uroporphy	13	2	2	2	265	28.6
Q9Y5Q0	FADS3	Fatty aci	8	3	3	3	445	51.1
O15066	KIF3B	Kinesin-l	2	2	2	2	747	85.1
Q9Y2Q9	MRPS28	28S ribos	10	2	2	1	187	20.8
Q86TB9	PATL1	Protein P	3	2	2	2	770	86.8
A0A2R8Y7E	CCDC88A	Girdin OS	1	2	2	2	1604	185.4
Q9H5Q4	TFB2M	Dimethyla	4	2	2	2	396	45.3
P57081	WDR4	tRNA (gua	5	2	2	2	412	45.5
J3KT75	MPDU1	Mannose-P	6	1	3	1	154	16.5
Q13057	COASY	Bifunctio	3	2	2	2	564	62.3
Q9Y2V2	CARHSP1	Calcium-r	11	1	2	1	147	15.9
Q96J84	KIRREL1	Kin of IR	5	2	2	2	757	83.5
Q7KYR7	BTN2A1	Butyrophi	4	2	3	2	527	59.6
O94822	LTN1	E3 ubiqui	2	4	4	4	1766	200.4
Q9BRP4	PAAF1	Proteasom	7	3	3	3	392	42.2
Q5T2E6	ARMH3	Armadillo	3	3	3	3	689	78.7
Q96HW7	INTS4	Integrato	3	2	2	2	963	108.1
Q5VSL9	STRIP1	Striatin-	2	1	2	1	837	95.5
Q8N335	GPD1L	Glycerol-	6	2	2	2	351	38.4
H7BXJ4	FCHO2	F-BAR dom	7	3	3	3	378	43.4
J3QSU6	TNC	Tenascin	1	2	2	2	2019	220.7
Q7Z6J0	SH3RF1	E3 ubiqui	2	2	2	2	888	93.1
F8W733	BABAM2	BRISC and	5	1	2	1	284	32
P42858	HTT	Huntingti	1	2	2	2	3142	347.4
P49914	MTHFS	5-formylt	13	3	3	3	203	23.2
G3V583	FAM177A1	Protein F	19	3	3	3	140	16.3
Q9HBR0	SLC38A10	Putative	2	2	2	2	1119	119.7
Q7Z7L1	SLFN11	Schlafen	2	2	2	1	901	102.8
Q9P2B2	PTGFRN	Prostagla	3	2	2	2	879	98.5
E5RK00	DCTN6	Dynactin	11	2	2	2	170	18.4
Q12972	PPP1R8	Nuclear i	7	2	2	2	351	38.5
Q06546	GABPA	GA-bindin	5	2	2	2	454	51.3
Q12824	SMARCB1	SWI/SNF-r	9	2	2	2	385	44.1
Q4KMQ2	ANO6	Anoctamin	3	3	4	3	910	106.1
Q92609	TBC1D5	TBC1 doma	3	2	2	2	795	88.9
Q10570	CPSF1	Cleavage	1	2	2	2	1443	160.8
P11169	SLC2A3	Solute ca	2	1	2	1	496	53.9
Q5QPA5	MRPS18A	39S ribos	8	2	2	2	263	29.6
G3V5Z3	PPP4R3A	Serine/th	4	3	3	3	706	82

Q9NZR1	TMOD2	Tropomodu	7	3	3	2	351	39.6
Q86UA1	PRPF39	Pre-mRNA-	5	3	3	3	669	78.4
Q969S9	GFM2	Ribosome-	3	2	2	2	779	86.5
O43674	NDUFB5	NADH dehy	17	2	2	2	189	21.7
P62861	FAU	40S ribos	17	1	4	1	59	6.6
P31273	HOXC8	Homeobox	9	2	2	2	242	27.7
F5H5N2	ISCU	Iron-sulf	16	3	3	3	154	16.6
Q4G148	GXYLT1	Glucoside	4	2	2	2	440	50.5
Q9HBI1	PARVB	Beta-parv	6	2	2	1	364	41.7
Q6P1M0	SLC27A4	Long-chai	4	2	2	2	643	72
Q9Y5J5	PHLDA3	Pleckstri	14	2	2	2	127	13.9
Q9NR19	ACSS2	Acetyl-co	3	1	2	1	701	78.5
A6NML8	DIAPH2	Diaphanou	3	3	3	3	1096	124.8
H0YAJ5	APBB2	Amyloid-b	3	2	2	2	728	80
O14949	UQCRQ	Cytochrom	22	2	2	2	82	9.9
Q9BRS8	LARP6	La-relate	4	2	2	2	491	54.7
Q969E8	TSR2	Pre-rRNA-	11	2	2	2	191	20.9
Q9UBV2	SEL1L	Protein s	2	2	2	2	794	88.7
P40938	RFC3	Replicati	5	2	2	2	356	40.5
Q8N241	HSPB7	Heat shoc	9	2	2	2	245	27.4
Q8IU81	IRF2BP1	Interferc	3	2	2	2	584	61.6
Q9H7B4	SMYD3	Histone-l	5	2	2	2	428	49.1
B3KNS4	ERVK3-1	Endogenou	26	2	2	2	109	11.6
B7ZAX5	GALK2	cDNA, FLJ	5	2	2	2	434	47.6
K7ELH8	DPY19L3	Probable	3	2	2	2	688	79.5
Q96EK9	KTI12	Protein K	9	2	2	2	354	38.6
Q96GC5	MRPL48	39S ribos	9	2	2	2	212	23.9
Q14012	CAMK1	Calcium/c	7	2	2	2	370	41.3
Q8IVL5	P3H2	Prolyl 3-	3	2	5	1	708	80.9
O14684	PTGES	Prostagla	12	2	2	2	152	17.1
Q9H7E9	C8orf33	UPF0488 p	8	2	2	2	229	25
Q7Z392	TRAPPC11	Trafficki	2	2	2	2	1133	128.8
Q14746	COG2	Conserved	4	2	2	2	738	83.2
Q8WUH2	TGFBRAP1	Transform	2	2	2	2	860	97.1
H0YL10	CD276	CD276 ant	11	2	2	2	199	21.4
P01034	CST3	Cystatin-	11	1	1	1	146	15.8
K7ENR6	PSMG2	Proteasom	6	2	3	2	240	26.7
Q9NUQ3	TXLNG	Gamma-tax	5	2	2	2	528	60.5
E5RFR7	TPD52	Tumor prc	20	2	2	1	111	12.4
M0QXD5	PIH1D1	PIH1 doma	11	2	2	2	236	26.4
Q8TD30	GPT2	Alanine a	4	2	2	2	523	57.9
Q9H425	C1orf198	Uncharact	6	2	2	2	327	36.3
D6RBR7	ZNF330	Zinc fing	12	3	4	3	229	25.8
Q14149	MORC3	MORC fami	2	2	2	2	939	107
O75817	POP7	Ribonucle	18	2	2	2	140	15.6
Q9P291	ARMCX1	Armadillc	5	2	2	2	453	49.2
Q9NPL8	TIMMDC1	Complex I	9	3	3	3	285	32.2
Q9UFG5	C19orf25	UPF0449 p	22	2	2	2	118	12.9
Q9BQB6	VKORC1	Vitamin K	13	3	4	3	163	18.2
Q06481	APLP2	Amyloid-l	1	1	2	1	763	86.9
A0A087WVC		Uncharact	19	2	2	2	110	12.7

Q5EBL8	PDZD11	PDZ domai	17	1	1	1	140	16.1
E7EQ64	PRSS1	Trypsin-1	11	2	7	2	261	28.1
Q6DN90	IQSEC1	IQ motif	2	2	2	2	963	108.2
P05109	S100A8	Protein S	19	2	2	2	93	10.8
O96013	PAK4	Serine/th	3	2	2	2	591	64
Q16637	SMN1	Survival	7	2	2	2	294	31.8
P28676	GCA	Grancalci	14	3	3	3	217	24
E9PE15	CLCN3	Chloride	3	2	2	2	725	80.5
Q9NRZ7	AGPAT3	1-acyl-sn	5	2	3	2	376	43.4
C9J6A7	RPE	Ribulose-	11	2	2	2	159	17.4
F8WCZ3	CCDC115	Coiled-cc	11	2	2	2	193	21.1
Q9UBV8	PEF1	Peflin OS	8	2	2	2	284	30.4
O94888	UBXN7	UBX domai	5	3	4	3	489	54.8
A0A2R8YDF	TSC2	Tuberin (	2	2	2	2	1781	197.7
O95396	MOCS3	Adenylylt	6	2	2	2	460	49.6
E9PPY3	RRP8	Ribosomal	7	2	2	2	306	34.2
Q8NBP0	TTC13	Tetratric	4	2	2	2	860	96.8
Q7Z6B7	SRGAP1	SLIT-ROBO	1	2	2	2	1085	124.2
Q9UK39	NOCT	Nocturnin	4	1	1	1	431	48.2
Q8N122	RPTOR	Regulator	2	2	2	2	1335	148.9
Q9NX08	COMMD8	COMM doma	11	2	2	2	183	21.1
Q8TCE6	FAM45A	Protein F	7	2	2	2	357	40.5
Q5SY16	NOL9	Polynucle	3	2	3	2	702	79.3
Q9NRN7	AASDHPPT	L-aminoad	6	2	2	2	309	35.8
O75909	CCNK	Cyclin-K	4	2	2	2	580	64.2
Q8N5G0	SMIM20	Small int	33	2	2	2	67	7.7
A0A096LP6	CD99	CD99 anti	9	2	2	2	184	18.8
Q9H7M9	VSIR	V-type im	5	2	2	2	311	33.9
Q6UWP7	LCLAT1	Lysocardi	7	3	3	3	414	48.9
Q9P2C4	TMEM181	Transmemb	3	1	1	1	612	69.3
Q86UE8	TLK2	Serine/th	2	2	2	1	772	87.6
B9ZVN9	POLR1A	DNA-direc	2	2	2	2	1659	187.7
P04217	A1BG	Alpha-1B-	4	1	1	1	495	54.2
P19256	CD58	Lymphocyt	10	2	2	2	250	28.1
Q6UVY6	MOXD1	DBH-like	4	2	2	2	613	69.6
R4GN18	CD46	Membrane	27	2	2	2	78	8.5
Q9ULP9	TBC1D24	TBC1 doma	4	2	3	2	559	62.9
Q9C0I1	MTMR12	Myotubula	3	2	2	2	747	86.1
H0Y555	PSMF1	Proteasom	20	2	3	2	107	11.6
Q8TCC3	MRPL30	39S ribos	15	3	3	3	161	18.5
Q9BPX5	ARPC5L	Actin-rel	14	3	4	3	153	16.9
Q86VX2	COMMD7	COMM doma	10	2	2	2	200	22.5
A0A0B4J25	LYZ	Lysozyme	15	2	2	2	137	15.3
O43318	MAP3K7	Mitogen-a	4	2	2	2	606	67.2
Q8NC60	NOA1	Nitric ox	4	2	3	2	698	78.4
Q9Y613	FHOD1	FH1/FH2 d	2	3	3	3	1164	126.5
O00217	NDUFS8	NADH dehy	9	2	2	2	210	23.7
Q5JTJ3	COA6	Cytochrom	21	2	2	2	125	14.1
P02792	FTL	Ferritin	18	3	3	3	175	20
Q5JSL0	HMGN5	High mobi	16	2	2	2	148	16.5
Q6ZUT6	CCDC9B	Coiled-cc	3	2	2	2	534	57.3

Q9BTY2	FUCA2	Plasma al	4	2	2	2	467	54
A0A2U3TZV	PLCH1	1-phospha	0	1	10	1	1685	188.6
G3V150	B3GAT3	Galactosy	5	2	2	2	319	34.9
A8MQ02	AFDN	Afadin OS	1	2	2	2	1781	201.7
Q5TAW7	CAB39L	Calcium-b	9	3	3	1	280	32.4
Q7Z2X4	PID1	PTB-conta	8	1	1	1	250	28.3
Q15172	PPP2R5A	Serine/th	6	2	2	2	486	56.2
Q96BZ9	TBC1D20	TBC1 doma	6	2	2	2	403	45.8
A0A0S2Z4R	TAP1	Antigen p	10	2	2	2	323	34.6
Q9NY61	AATF	Protein A	4	2	2	2	560	63.1
Q5VWJ9	SNX30	Sorting n	4	2	2	2	437	49.6
Q96L58	B3GALT6	Beta-1,3-	4	1	1	1	329	37.1
P62341	SELENOT	Thioredox	10	2	2	2	195	22.3
Q8NI22	MCFD2	Multiple	12	1	2	1	146	16.4
A6XND1	IGFBP3	Insulin-l	7	2	2	2	263	29
F6SYF8	DKK3	Dickkopf-	10	2	2	2	364	39.9
Q9GZT8	NIF3L1	NIF3-like	9	3	3	3	377	41.9
Q9NY93	DDX56	Probable	3	2	2	2	547	61.6
P78524	ST5	Suppressi	2	2	2	2	1137	126.4
Q9NXE4	SMPD4	Sphingomy	2	2	2	2	866	97.7
Q9UQ03	CORO2B	Coronin-2	5	2	2	2	480	54.9
Q00403	GTF2B	Transcrip	8	3	3	3	316	34.8
P80217	IFI35	Interfero	7	2	2	2	286	31.5
O14646	CHD1	Chromodom	1	3	3	3	1710	196.6
Q9UL54	TAOK2	Serine/th	1	2	3	1	1235	138.2
Q13424	SNTA1	Alpha-1-s	4	2	2	1	505	53.9
O75884	RBBP9	Putative	16	2	2	2	186	21
F8WF16	PPHLN1	Periphili	6	2	2	2	303	34.8
Q96IP4	TENT5A	Terminal	5	2	2	2	442	49.6
Q5T1B5	INPP5A	Type I in	4	2	3	2	387	44.9
Q9UBI1	COMMD3	COMM doma	12	2	3	2	195	22.1
P34059	GALNS	N-acetylg	3	2	3	1	522	58
Q96CN4	EVI5L	EVI5-like	3	2	2	1	794	91.3
Q9H974	QTRT2	Queuine t	5	1	1	1	415	46.7
Q96H79	ZC3HAV1L	Zinc fing	7	2	2	2	300	32.9
O75815	BCAR3	Breast ca	3	3	4	3	825	92.5
O00479	HMGN4	High mobi	19	2	2	2	90	9.5
O95628	CNOT4	CCR4-NOT	3	2	2	2	575	63.5
Q6ICB0	DESI1	Desumoyla	10	2	2	2	168	18.3
Q96DX4	RSPRY1	RING fing	5	2	2	2	576	64.1
P57723	PCBP4	Poly(rC)-	5	2	4	1	403	41.5
Q86U38	NOP9	Nucleolar	3	2	2	2	636	69.4
J3KRR1	C17orf75	Protein N	10	2	2	2	275	30.6
Q9UET6	FTSJ1	Putative	8	2	2	2	329	36.1
Q96AJ9	VTI1A	Vesicle t	7	2	2	2	217	25.2
O15397	IPO8	Importin-	2	2	2	1	1037	119.9
A0A088AWN	DOCK9	Dedicator	1	2	2	1	2081	237.8
Q14118	DAG1	Dystrogly	3	1	1	1	895	97.4
Q96EY1	DNAJA3	DnaJ homo	5	2	2	2	480	52.5
Q6P6C2	ALKBH5	RNA demet	7	2	2	2	394	44.2
Q9UBV7	B4GALT7	Beta-1,4-	8	3	3	3	327	37.4

E9PIQ7	HAX1	HCLS1-ass	13	2	2	2	151	17.2
P40937	RFC5	Replicati	5	2	2	2	340	38.5
Q9P0P0	RNF181	E3 ubiqui	10	2	2	2	153	17.9
Q16656	NRF1	Nuclear r	4	2	2	2	503	53.5
Q8IXM6	NRM	Nurim OS=	7	2	2	2	262	29.4
Q9BX40	LSM14B	Protein L	8	2	2	2	385	42
J3QRZ6	MIF4GD	MIF4G dom	15	2	2	2	204	23.3
Q96QU8	XPO6	Exportin-	2	2	2	2	1125	128.8
Q9NPJ6	MED4	Mediator	8	2	2	2	270	29.7
O95786	DDX58	Probable	2	2	2	2	925	106.5
Q9NYV4	CDK12	Cyclin-de	2	3	3	2	1490	164.1
Q9P013	CWC15	Spliceosc	8	2	2	2	229	26.6
Q9UKU7	ACAD8	Isobutyry	5	2	2	2	415	45
Q9Y3B2	EXOSC1	Exosome c	12	2	2	2	195	21.4
Q15154	PCM1	Pericentr	1	3	3	2	2024	228.4
H0YJM2	UBR7	Putative	8	2	2	2	174	20.5
O14879	IFIT3	Interferc	5	2	2	2	490	56
H3BQV3	COG8	Conservec	3	2	2	2	534	60.4
Q32P41	TRMT5	tRNA (gua	4	2	2	2	509	58.2
Q7Z674	FEZ2	Fascicula	11	2	2	2	182	20.9
O75146	HIP1R	Huntingti	2	3	3	2	1068	119.3
Q9UJY1	HSPB8	Heat shoc	12	2	2	2	196	21.6
P56589	PEX3	Peroxisom	5	2	2	2	373	42.1
O96033	MOCS2	Molybdopt	25	2	2	2	88	9.7
Q8WVC0	LEO1	RNA polym	2	1	1	1	666	75.4
Q8TDQ4	TMEM222	Transmemb	11	1	1	1	175	19.8
E9PG73	PPIG	Peptidyl-	2	2	2	2	739	87
Q9P2P6	STARD9	StAR-rela	0	2	2	2	4700	516
P42356	PI4KA	Phosphati	1	2	2	2	2102	236.7
Q9HAN9	NMNAT1	Nicotinam	7	2	2	2	279	31.9
Q16134	ETFDH	Electron	5	2	2	2	617	68.5
P21953	BCKDHB	2-oxoisov	6	2	2	2	392	43.1
Q70JA7	CHSY3	Chondroit	3	3	3	3	882	100.2
Q92995	USP13	Ubiquitir	2	2	2	2	863	97.3
Q92597	NDRG1	Protein N	8	2	2	2	394	42.8
Q9NWX6	THG1L	Probable	6	2	2	2	298	34.8
E7EM50	PIGG	GPI ethar	5	2	2	2	463	51.2
Q8TCD1	C18orf32	UPF0729 p	13	1	2	1	76	8.7
Q9Y294	ASF1A	Histone c	13	2	2	2	204	23
Q99496	RNF2	E3 ubiqui	6	2	2	1	336	37.6
L0R819	ASDURF	ASNSD1 up	19	2	2	2	96	11.2
Q9UMZ2	SYNRG	Synergin	1	2	2	2	1314	140.6
H7C224	IRAK1	Interleuk	5	2	2	2	392	42.2
Q6P1L8	MRPL14	39S ribos	11	2	2	2	145	15.9
Q8WY22	BRI3BP	BRI3-binc	9	2	2	1	251	27.8
Q08426	EHHADH	Peroxisom	4	2	2	2	723	79.4
O43184	ADAM12	Disintegr	3	3	4	3	909	99.5
F5GYT8	MCCC1	Methylcrc	4	2	2	2	575	64
Q8N511	TMEM199	Transmemb	10	2	2	2	208	23.1
Q9NRG9	AAAS	Aladin OS	3	2	2	2	546	59.5
K7EQH1	C18orf25	Uncharact	15	1	1	1	146	15.5

Q86X76	NIT1	Deaminate	6	2	2	2	327	35.9
Q9UQ13	SHOC2	Leucine-r	3	2	2	2	582	64.8
Q8N3P4	VPS8	Vacuolar	2	2	2	2	1428	161.7
P45877	PPIC	Peptidyl-	9	3	7	2	212	22.7
Q9P2Q2	FRMD4A	FERM doma	1	2	2	2	1039	115.4
Q8NE86	MCU	Calcium u	5	2	2	2	351	39.8
Q9BVM4	GGACT	Gamma-glu	14	2	2	2	153	17.3
Q8IWR0	ZC3H7A	Zinc fing	2	2	2	2	971	110.5
Q8N5I4	DHRSX	Dehydroge	5	1	1	1	330	36.4
Q15427	SF3B4	Splicing	3	1	1	1	424	44.4
Q4LDG9	DNAL1	Dynein li	11	2	2	2	190	21.5
Q02040	AKAP17A	A-kinase	4	3	3	3	695	80.7
Q6XQN6	NAPRT	Nicotinat	4	2	2	2	538	57.5
E5RH51	SMIM12	Small int	26	2	2	2	74	8.7
P51397	DAP	Death-ass	16	2	3	2	102	11.2
Q9UHR4	BAIAP2L1	Brain-spe	4	2	2	2	511	56.8
P52657	GTF2A2	Transcrip	15	2	2	2	109	12.4
O15226	NKRF	NF-kappa-	3	2	2	2	690	77.6
Q92733	PRCC	Proline-r	4	2	2	2	491	52.4
G3V4K3	VIPAS39	Spermatog	3	2	2	2	519	59.7
A5PLN9	TRAPPC13	Trafficki	4	2	2	2	417	46.5
Q9NP92	MRPS30	39S ribos	10	3	4	3	439	50.3
H7BY55	CD55	Complemen	3	2	2	2	550	58.9
B1APR7	EYA3	Eyes abse	3	1	1	1	416	45.5
Q8WXH0	SYNE2	Nesprin-2	0	3	3	1	6885	795.9
H3BPK1	KATNB1	Katanin p	8	2	2	2	210	22.9
P01024	C3	Complemen	1	1	1	1	1663	187
A0A0A0MSY	TK2	Thymidine	6	2	2	2	289	33.3
A0A087WU8	TNFAIP3	Tumor nec	3	3	3	3	749	84.9
A0A087WZK	DHPS	Deoxyhypu	6	2	2	2	370	41.1
Q9Y2Q5	LAMTOR2	Ragulator	8	1	2	1	125	13.5
P12074	COX6A1	Cytochrom	27	1	2	1	109	12.1
O14880	MGST3	Microsoma	5	1	2	1	152	16.5
Q9NVM6	DNAJC17	DnaJ homo	5	2	2	2	304	34.7
O75084	FZD7	Frizzled-	3	2	3	2	574	63.6
P25490	YY1	Transcrip	4	2	2	2	414	44.7
Q969T9	WBP2	WW domain	7	2	2	2	261	28.1
P15291	B4GALT1	Beta-1,4-	4	2	2	2	398	43.9
Q14289	PTK2B	Protein-t	2	2	2	2	1009	115.8
A0A0U1RQI	SORBS1	Sorbin an	3	1	1	1	507	58
A0A2R8Y5F	KSR1	Kinase su	2	2	2	2	928	103
Q14692	BMS1	Ribosome	1	2	2	2	1282	145.7
Q9UKN8	GTF3C4	General t	2	2	2	2	822	91.9
O43292	GPAA1	Glycosylp	3	2	2	2	621	67.6
H0Y3M3	EML3	Echinoder	3	2	2	2	911	97.5
Q96BW9	TAMM41	Phosphati	4	1	1	1	452	51
P35251	RFC1	Replicati	1	1	1	1	1148	128.2
E9PI99	NFYC	Nuclear t	18	3	3	3	141	16.2
Q99808	SLC29A1	Equilibra	4	2	2	2	456	50.2
Q06587	RING1	E3 ubiqui	5	2	2	1	406	42.4
Q96I51	RCC1L	RCC1-like	5	1	1	1	464	50

Q8NHG7	SVIP	Small VCF	14	1	1	1	77	8.4
Q5R3B4	MPC2	Mitochond	11	1	1	1	105	11.7
Q96JP5	ZFP91	E3 ubiqui	4	1	1	1	570	63.4
Q68DK2	ZFYVE26	Zinc fing	1	2	3	1	2539	284.4
Q5JTZ9	AARS2	Alanine--	2	2	2	2	985	107.3
O14682	ENC1	Ectoderm-	3	2	2	2	589	66.1
H9KVA9	BRCC3	Lys-63-sp	3	1	1	1	317	36.2
Q86UV5	USP48	Ubiquitin	2	3	3	3	1035	119
Q96P47	AGAP3	Arf-GAP w	4	2	2	2	875	95
O43301	HSPA12A	Heat shoc	3	2	2	2	675	74.9
H0YAG5	MRPS18C	28S ribos	16	1	1	1	113	12.5
A0A096LNX	SCARF2	Scavenger	3	2	2	2	871	92.3
P62328	TMSB4X	Thymosin	32	2	4	2	44	5.1
A2IDA3	MPG	DNA-3-met	5	1	1	1	251	27.3
Q99797	MIPEP	Mitochond	3	2	2	2	713	80.6
Q9ULF5	SLC39A10	Zinc tran	2	2	2	2	831	94.1
Q9Y6K8	AK5	Adenylate	3	2	5	1	562	63.3
G3V1J9	TMED3	Transmemb	8	1	1	1	146	16.7
Q9BXP2	SLC12A9	Solute ca	2	1	1	1	914	96
O60508	CDC40	Pre-mRNA-	3	2	2	2	579	65.5
Q9BQ51	PDCD1LG2	Programme	4	1	1	1	273	30.9
H7BXY6	TSPAN14	Tetraspan	9	2	2	2	213	23.9
Q9NWZ8	GEMIN8	Gem-assoc	8	2	2	2	242	28.6
Q5T5Y3	CAMSAP1	Calmoduli	1	3	3	2	1602	177.9
Q96SK3	ZNF607	Zinc fing	4	2	2	2	696	80.5
Q9BQQ3	GORASP1	Golgi rea	4	2	2	1	440	46.5
Q8IVH4	MMAA	Methylmal	4	2	2	2	418	46.5
Q8WWH5	TRUB1	Probable	5	2	2	2	349	37.2
Q7Z6U0	TBPL1	TATA box-	14	1	1	1	91	10.2
O95639	CPSF4	Cleavage	4	1	1	1	269	30.2
Q9C0H2	TTYH3	Protein t	3	2	2	2	523	57.5
Q96EC8	YIPF6	Protein Y	5	1	1	1	236	26.2
E5RGA1	PLAT	Tissue-ty	7	1	1	1	178	20.1
Q96AA3	RFT1	Protein R	4	2	2	2	541	60.3
P55899	FCGRT	IgG recep	4	2	2	2	365	39.7
Q9NTJ4	MAN2C1	Alpha-man	2	2	2	2	1040	115.8
O94923	GLCE	D-glucuro	4	3	3	3	617	70.1
G3V5T0	GSTZ1	Maleylace	8	2	2	2	202	22.6
J3QQK6	MBP	Myelin ba	9	2	2	2	253	27.7
Q9Y221	NIP7	60S ribos	9	2	2	2	180	20.4
H0YDK7	RAB30	Ras-relat	11	2	2	1	167	19.2
C9JWV9	TMBIM1	Protein l	8	1	1	1	171	19
A0A0C4DG9	KLHL13	Kelch-lik	3	2	2	2	658	74.6
Q99717	SMAD5	Mothers a	4	2	2	2	465	52.2
K7EM09	TMEM205	Transmemb	11	1	1	1	120	13.4
B3KRD8	SEC14L2	SEC14-lik	5	2	2	2	329	37.4
Q9ULI3	HEG1	Protein H	2	2	2	2	1381	147.4
P52179	MYOM1	Myomesin-	1	1	3	1	1685	187.5
P46100	ATRX	Transcrip	1	2	3	1	2492	282.4
Q16566	CAMK4	Calcium/c	4	2	2	1	473	51.9
H7C5U3	MFSD1	Major fac	19	1	1	1	64	6.8

Q15118	PDK1	[Pyruvate	5	2	2	2	436	49.2
Q9Y312	AAR2	Protein A	5	1	2	1	384	43.4
A0A0C4DFF	NGLY1	Peptide-N	4	2	2	2	633	72
Q6PI78	TMEM65	Transmemb	8	2	2	2	240	25.5
F5H303	NOC4L	Nucleolar	8	2	2	2	270	31.1
Q7L4I2	RSRC2	Arginine/	5	1	1	1	434	50.5
H0YFW5	MSRB3	Methionin	9	2	2	2	174	19.5
R4GMU8	LAMTOR5	Regulator	25	1	1	1	79	8.2
Q9BVL4	SELENOO	Selenopro	2	1	1	1	669	73.4
Q9Y657	SPIN1	Spindlin-	6	1	1	1	262	29.6
G3V2M2	ZFYVE21	Zinc fing	22	1	2	1	93	10.5
Q5T4B2	CERCAM	Inactive	4	2	2	2	595	67.5
Q9UFW8	CGGBP1	CGG tripl	5	1	1	1	167	18.8
H3BRM1	ZFYVE19	Abscissic	5	2	2	1	307	33.2
A0A087WYY	ATP13A3	Cation-tr	2	1	1	1	701	77.3
Q8IYS2	KIAA2013	Uncharact	2	2	2	2	634	69.1
F2Z328	DYNLT3	Dynein li	8	1	1	1	122	13.4
Q5T0V2	SLIT1	Slit homo	2	1	3	1	409	45
Q8WVT3	TRAPPC12	Trafficki	2	1	1	1	735	79.3
Q9H977	WDR54	WD repeat	4	1	1	1	334	35.9
Q8IV48	ERI1	3'-5' exo	5	2	2	2	349	40
Q01658	DR1	Protein D	9	2	2	2	176	19.4
Q9NXH8	TOR4A	Torsin-4A	5	2	2	2	423	46.9
Q86YZ3	HRNR	Hornerin	2	2	2	2	2850	282.2
B7Z7D2	ACP2	cDNA FLJ5	5	2	2	2	391	44.5
F5GZ28	LIG1	DNA ligas	2	2	3	1	851	93.9
C9JBX7	WDR45	WD repeat	5	1	1	1	213	23.5
A0A2R8YFQ	CRYL1	Lambda-cr	8	2	2	2	229	25.3
Q9Y6M5	SLC30A1	Zinc tran	4	2	3	2	507	55.3
P12277	CKB	Creatine	4	2	2	2	381	42.6
O75940	SMNDC1	Survival	4	1	1	1	238	26.7
A0A087X09	WDR13	WD repeat	10	2	2	2	187	20.6
Q96A00	PPP1R14A	Protein p	12	2	2	2	147	16.7
H7C024	GPC1	Glypican-	5	1	1	1	294	32.9
C9J236	NDUFAF7	Protein a	9	1	1	1	230	25.4
Q9BUH6	PAXX	Protein P	7	1	1	1	204	21.6
Q96JH7	VCPIP1	Deubiquit	1	1	1	1	1222	134.2
C9JKF1	SAMD9	Sterile a	2	2	6	1	1283	148.7
D6RCB6	RIOX2	Ribosomal	13	1	1	1	112	12.4
A0A2R8Y4J	RMND1	Required	3	1	1	1	444	50.5
E9PIN5	TP53I11	Tumor pro	8	1	1	1	123	13.3
Q5JPH6	EARS2	Probable	3	2	2	2	523	58.7
O14777	NDC80	Kinetocho	2	2	2	2	642	73.9
H0YCT3	POGZ	Pogo tran	6	1	1	1	316	35.4
Q7Z2K6	ERMP1	Endoplasm	1	1	1	1	904	100.2
E9PI02	HSF1	Heat shoc	7	1	1	1	138	16.1
E7EUH7	PUS7	Pseudouri	4	2	2	2	448	50.5
Q8N4H5	TOMM5	Mitochond	43	3	4	3	51	6
Q9HBM6	TAF9B	Transcrip	4	1	1	1	251	27.6
K7ELY2	STX10	Syntaxin-	5	1	1	1	201	22.5
Q9NXU5	ARL15	ADP-ribos	9	2	2	2	204	22.9

Q5JXX2	MORF4L2	Mortality	13	2	2	2	177	19.8
P22413	ENPP1	Ectonucle	3	1	1	1	925	104.9
Q9H9A5	CNOT10	CCR4-NOT	2	2	2	2	744	82.3
P61024	CKS1B	Cyclin-de	20	1	1	1	79	9.7
P98170	XIAP	E3 ubiqui	3	1	1	1	497	56.6
A0A087WYUMETTL26		Methyltra	5	1	1	1	184	20.4
Q8IWB9	TEX2	Testis-ex	2	2	2	2	1127	125.2
Q9Y2U5	MAP3K2	Mitogen-a	2	1	1	1	619	69.7
F8VQD9	ATG101	Autophagy	13	2	2	2	190	21.9
J3KNN7	BRAP	BRCA1-ass	2	1	1	1	562	64.2
O95801	TTC4	Tetratric	5	2	2	2	387	44.7
P36639	NUDT1	7,8-dihyd	7	1	1	1	197	22.5
Q969M3	YIPF5	Protein Y	5	1	1	1	257	28
Q9P003	CNIH4	Protein c	14	1	1	1	139	16.1
J3QQW2	SS18	Protein S	13	1	1	1	94	10.6
O43741	PRKAB2	5'-AMP-ac	7	1	1	1	272	30.3
Q9H788	SH2D4A	SH2 domai	4	2	2	2	454	52.7
Q99595	TIMM17A	Mitochond	13	1	1	1	171	18
Q09328	MGAT5	Alpha-1,6	1	1	1	1	741	84.5
O95633	FSTL3	Follistat	7	1	1	1	263	27.6
I3L0T6	MRM3	rRNA meth	11	1	1	1	110	12.6
A0A0U1RRLSPATA20		Spermatog	1	1	1	1	786	88
Q969Z0	TBRG4	FAST kina	2	2	3	2	631	70.7
Q8NHZ8	CDC26	Anaphase-	13	1	2	1	85	9.8
H0YI10	CERS5	Ceramide	5	2	2	2	259	30.5
P63272	SUPT4H1	Transcrip	8	1	1	1	117	13.2
Q9UF94	KLHDC4	Kelch dom	9	2	2	2	258	29.2
Q9H501	ESF1	ESF1 homo	1	1	1	1	851	98.7
E7EPJ2	ITSN2	Intersect	3	1	1	1	609	68.9
Q15742	NAB2	NGFI-A-bi	2	1	1	1	525	56.6
O14531	DPYSL4	Dihydropy	3	1	3	1	572	61.8
Q9Y679	AUP1	Ancient u	4	1	1	1	476	53
H7BXQ8	ARMC10	Armadillo	6	1	1	1	193	20.9
A1L188	NDUFAF8	NADH dehy	18	1	1	1	74	7.8
Q9BU61	NDUFAF3	NADH dehy	6	1	1	1	184	20.3
P61812	TGFB2	Transform	6	2	2	2	414	47.7
Q9BXJ0	C1QTNF5	Complemen	6	1	1	1	243	25.3
Q9ULE0	WWC3	Protein W	2	2	2	1	1092	122.6
Q08554	DSC1	Desmocoll	2	1	1	1	894	99.9
Q16832	DDR2	Discoidin	2	2	2	2	855	96.7
E9PEC4	CCM2	Cerebral	6	2	2	2	271	29.8
P22670	RFX1	MHC class	1	1	2	1	979	104.7
A8MXF6	RABL2B	Rab-like	7	1	1	1	165	18.5
Q8TF68	ZNF384	Zinc fing	2	1	1	1	577	63.2
P09661	SNRPA1	U2 small	8	2	2	2	255	28.4
H0YCP5	RNASEH2C	Ribonucle	12	1	1	1	129	13.9
H3BU43	SIRPB1	Signal-re	19	1	1	1	77	8.2
O95104	SCAF4	Splicing	1	2	2	2	1147	125.8
Q9BVS4	RIOK2	Serine/th	3	2	2	2	552	63.2
Q9NP84	TNFRSF12ATumor nec		8	1	1	1	129	13.9
Q9Y5T5	USP16	Ubiquitin	3	2	2	2	823	93.5

H0YME2	ZFAND6	AN1-type	16	1	1	1	159	17
Q9BRQ0	PYGO2	Pygopus h	4	1	1	1	406	41.2
Q8NG68	TTL	Tubulin--	3	1	1	1	377	43.2
P56378	MP68	6.8 kDa m	16	1	4	1	58	6.7
Q96EV2	RBM33	RNA-bindi	2	1	1	1	1170	129.9
C9J3D7	CROT	Peroxisom	3	1	1	1	574	65.8
O94992	HEXIM1	Protein H	4	2	2	2	359	40.6
Q8N2E6	TOR2A	Prosalusi	4	1	1	1	242	26.2
O15379	HDAC3	Histone d	6	2	2	2	428	48.8
Q9UKL0	RCOR1	REST core	2	1	1	1	485	53.3
F5H2Z7	CCDC92	Coiled-cc	11	1	1	1	96	11
A6NMN0	PHKA1	Phosphory	1	1	1	1	1240	139
Q9BRX5	GINS3	DNA repli	6	1	1	1	216	24.5
Q9P266	JCAD	Junctiona	1	2	2	2	1359	148.3
Q96HR3	MED30	Mediator	7	1	1	1	178	20.3
H7C3I5	CRYZL1	Quinone c	5	1	1	1	241	26.3
Q8N4V1	MMGT1	Membrane	7	1	1	1	131	14.7
P18440	NAT1	Arylamine	3	1	1	1	290	33.9
Q7L2J0	MEPCE	7SK snRNA	2	1	1	1	689	74.3
O43150	ASAP2	Arf-GAP w	2	2	2	2	1006	111.6
H0Y7V5	CCDC186	Coiled-cc	3	1	1	1	436	50.9
P27658	COL8A1	Collagen	1	1	1	1	744	73.3
O60870	KIN	DNA/RNA-b	3	1	1	1	393	45.3
A0A087WVZ	POLR2E	DNA-direc	9	2	2	2	184	21.4
P78345	RPP38	Ribonucle	4	1	1	1	283	31.8
O95772	STARD3NL	STARD3 N-	4	1	1	1	234	26.6
B4DUA7	MED29	Intersex-	5	1	1	1	221	23.5
C9J2Q2	ATPAF2	ATP synth	6	1	1	1	205	23.1
Q9ULK4	MED23	Mediator	1	1	1	1	1368	156.4
X6RLT1	NELFCD	Negative	3	2	2	2	593	66.5
F8WBT0	ARAP1	Arf-GAP w	5	1	1	1	307	34.9
Q96NT1	NAP1L5	Nucleosom	5	1	1	1	182	19.6
H0YJ92	MNAT1	CDK-activ	5	1	1	1	169	19.4
A0A0A0MTQ	KIAA0391	Mitochond	5	1	1	1	185	21.7
J3KRP9	TANC2	Protein T	1	1	1	1	1254	138.9
Q9NSY2	STARD5	StAR-rela	4	1	1	1	213	23.8
E7EV41	SLC8A1	Sodium/ca	2	2	2	2	664	74.4
Q6GMV2	SMYD5	SET and M	3	1	1	1	418	47.3
Q96FX2	DPH3	DPH3 homo	26	1	1	1	82	9.2
Q9NQW6	ANLN	Anillin C	1	1	1	1	1124	124.1
Q5F1R6	DNAJC21	DnaJ homo	4	2	2	2	531	62
Q6UWJ1	TMCO3	Transmemb	1	1	1	1	677	75.5
Q96NC0	ZMAT2	Zinc fing	8	2	2	2	199	23.6
O95999	BCL10	B-cell ly	4	1	1	1	233	26.2
Q96AY4	TTC28	Tetratric	0	1	1	1	2481	270.7
P60520	GABARAPL2	Gamma-ami	13	2	2	1	117	13.7
A0A087WZC	ARHGEF18	Rho guani	1	2	2	1	1122	126.1
A0A087WTT	TMEM230	Transmemb	9	1	1	1	127	13.6
X6R717	DHX32	Putative	4	1	1	1	367	41.5
Q96IW7	SEC22A	Vesicle-t	4	1	1	1	307	34.9
F8VV52	CNOT2	CCR4-NOT	4	2	2	2	531	58.6

H7BYP4	SP140L	Nuclear b	3	2	2	1	520	60.5
A0A087WYWHIKESHI		Protein H	7	1	1	1	131	14.4
Q8N5W9	RFLNB	Refilin-B	6	1	1	1	214	22.9
Q53TN4	CYBRD1	Cytochrom	3	1	1	1	286	31.6
Q96CW5	TUBGCP3	Gamma-tub	2	2	2	2	907	103.5
Q9NXD2	MTMR10	Myotubula	2	1	1	1	777	88.2
Q5TIH2	SFT2D2	Vesicle t	9	1	1	1	108	11.7
O14763	TNFRSF10E	Tumor nec	2	1	1	1	440	47.8
Q9UKZ1	CNOT11	CCR4-NOT	2	1	1	1	510	55.2
Q96G74	OTUD5	OTU domai	3	1	1	1	571	60.6
Q9UJJ9	GNPTG	N-acetylg	3	1	1	1	305	34
P49903	SEPHS1	Selenide,	3	1	2	1	392	42.9
Q9NWT1	PAK1IP1	p21-activ	4	2	2	2	392	43.9
F5GWI4	ADA	Adenosine	3	1	1	1	339	38.3
P42336	PIK3CA	Phosphati	1	2	2	2	1068	124.2
Q53SF7	COBLL1	Cordon-bl	1	1	1	1	1204	131.7
F8WDM6	MED15	Mediator	19	1	1	1	54	6.1
P08047	SP1	Transcrip	2	2	2	2	785	80.6
Q9BXJ8	TMEM120A	Transmemb	3	1	1	1	343	40.6
P62875	POLR2L	DNA-direc	13	1	1	1	67	7.6
Q5HYK7	SH3D19	SH3 domai	1	1	1	1	790	86.5
P17706	PTPN2	Tyrosine-	4	2	2	2	415	48.4
P24385	CCND1	G1/S-spec	6	2	2	2	295	33.7
A0A0A0MTEBTAF1		TATA-bind	1	1	1	1	1849	206.8
Q96B45	BORCS7	BLOC-1-re	16	1	1	1	105	11.6
A0A087WXDSLC9A7		Sodium/hy	1	1	1	1	726	80.2
U3KQ69	MTG1	Mitochond	3	1	1	1	293	32.6
P18887	XRCC1	DNA repai	3	2	2	2	633	69.4
Q5C9Z4	NOM1	Nucleolar	1	1	1	1	860	96.2
Q96BN8	OTULIN	Ubiquitin	5	2	2	2	352	40.2
K7ENP1	TRMT11	tRNA (gua	4	1	1	1	230	26.4
Q9BYC9	MRPL20	39S ribos	13	2	2	2	149	17.4
Q8WXA9	SREK1	Splicing	2	1	1	1	508	59.3
Q9H0N5	PCBD2	Pterin-4-	10	2	3	1	130	14.4
Q9BVJ7	DUSP23	Dual spec	7	1	1	1	150	16.6
O94817	ATG12	Ubiquitin	6	1	1	1	140	15.1
Q9Y4R8	TELO2	Telomere	2	2	2	2	837	91.7
Q8N108	MIER1	Mesoderm	4	1	1	1	512	57.9
P98155	VLDLR	Very low-	1	1	1	1	873	96
K7EQ17	NOL4	Nucleolar	2	1	1	1	558	61.3
A0A1W2PNWKCNMA1		Calcium-a	1	1	1	1	1230	137
Q7Z4H3	HDDC2	HD domain	5	1	1	1	204	23.4
Q14331	FRG1	Protein F	5	1	1	1	258	29.2
O95905	ECD	Protein e	2	1	1	1	644	72.7
Q8NBN3	TMEM87A	Transmemb	3	2	2	2	555	63.4
P82663	MRPS25	28S ribos	10	1	1	1	173	20.1
Q8IW45	NAXD	ATP-depen	4	1	1	1	347	36.6
P04066	FUCA1	Tissue al	2	1	2	1	466	53.7
A0A087WVZZMYND8		Protein k	1	2	2	2	1136	125.7
Q96GM8	TOE1	Target of	3	1	1	1	510	56.5
Q8TD43	TRPM4	Transient	1	1	1	1	1214	134.2

Q49B96	COX19	Cytochrom	11	1	1	1	90	10.4
A0A1W2PQ7	GOSR2	Golgi SNA	15	2	2	2	164	19.4
Q8IUR7	ARMC8	Armadillo	1	1	1	1	673	75.5
E5RFJ9	SARAF	Store-ope	12	1	1	1	147	16.1
Q8IX04	UEVLD	Ubiquitin	2	1	1	1	471	52.2
J3KPT4	TRABD	TraB doma	2	1	1	1	379	42.7
C9JE98	NCOR2	Nuclear r	1	1	1	1	2458	267.9
K7EPS8	C19orf12	Protein C	9	1	1	1	106	11
Q9Y6D0	SELENOK	Selenopro	18	2	2	2	94	10.6
Q96BI3	APH1A	Gamma-sec	5	1	1	1	265	29
Q04941	PLP2	Proteolip	9	1	2	1	152	16.7
I3L213	PHKB	Phosphory	8	1	1	1	210	23.5
Q8N9T8	KRI1	Protein K	3	2	2	2	703	82.5
P49069	CAMLG	Calcium s	3	1	1	1	296	32.9
Q96MG7	NSMCE3	Non-struc	6	2	2	2	304	34.3
C9JVK8	AZI2	5-azacyti	5	1	1	1	215	25
Q9Y625	GPC6	Glypican-	3	1	1	1	555	62.7
C9JBY7	MRPS33	28S ribos	9	1	1	1	96	11.4
C9JQZ0	SERF2	Small EDR	13	1	1	1	71	7.7
E5RK75	ZFAND1	AN1-type	10	1	1	1	97	10.9
O95297	MPZL1	Myelin pr	4	1	1	1	269	29.1
Q9P1F3	ABRACL	Costars f	16	1	1	1	81	9.1
Q8IY37	DHX37	Probable	1	1	1	1	1157	129.5
Q9NRG4	SMYD2	N-lysine	5	2	2	2	433	49.7
C9JWF7	SRPK2	SRSF prot	6	2	2	2	231	25.8
Q9H5V9	CXorf56	UPF0428 p	6	1	1	1	222	25.6
P48651	PTDSS1	Phosphati	4	2	2	2	473	55.5
P58004	SESN2	Sestrin-2	3	1	1	1	480	54.5
Q8IY33	MICALL2	MICAL-lik	2	1	1	1	904	97.4
Q9UHW5	GPN3	GPN-loop	7	2	2	2	284	32.7
Q6P161	MRPL54	39S ribos	7	1	1	1	138	15.8
Q9H2P9	DPH5	Diphthine	6	2	2	2	285	31.6
B0QY95	MIEF1	Mitochond	2	1	1	1	478	53.3
Q9H1A4	ANAPC1	Anaphase-	1	2	2	2	1944	216.4
J3KQ43	ALS2	Alsin (Fr	8	1	1	1	106	11.2
A6NHK2	SNRPE	Small nuc	23	1	1	1	52	5.9
F8VRX4	DDX54	ATP-depen	10	2	2	2	208	22.9
Q15628	TRADD	Tumor nec	6	1	1	1	312	34.2
Q14146	URB2	Unhealthy	1	1	1	1	1524	170.4
O95857	TSPAN13	Tetraspan	7	2	2	1	204	22.1
P58557	YBEY	Endoribon	7	1	1	1	167	19.3
P52435	POLR2J	DNA-direc	13	1	1	1	117	13.3
A0A024QZ3	NSRP1	Coiled-co	2	1	1	1	504	60.4
M0R176	MRPL51	39S ribos	34	1	1	1	32	4
P63218	GNG5	Guanine n	13	1	2	1	68	7.3
Q8NHV4	NEDD1	Protein N	1	1	1	1	660	71.9
F8VVY2	RNF41	E3 ubiqui	7	1	1	1	130	15
H3BNT2	COQ9	Ubiquinon	4	2	2	2	303	33.9
Q9BV81	EMC6	ER membra	9	1	1	1	110	12
Q8N5A5	ZGPAT	Zinc fing	2	1	1	1	531	57.3
Q8N653	LZTR1	Leucine-z	2	1	1	1	840	94.7

Q13287	NMI	N-myc-int	3	1	1	1	307	35
Q8N1Q1	CA13	Carbonic	3	1	1	1	262	29.4
J3QT51	CRBN	Protein c	4	1	1	1	228	26.2
C9JS61	ANKZF1	Ankyrin r	10	1	1	1	115	13.5
A0A087WUTMYEF2		Myelin ex	3	2	2	2	547	58.6
P58397	ADAMTS12	A disinte	1	2	2	2	1594	177.6
Q14249	ENDOG	Endonucle	3	1	1	1	297	32.6
O60942	RNGTT	mRNA-capp	1	1	1	1	597	68.5
A0A096LP2PTRH1		Probable	6	1	1	1	172	18.5
Q0VDG4	SCRN3	Secernin-	3	2	2	2	424	48.5
Q8TBQ9	TMEM167A	Protein k	13	1	1	1	72	8.1
Q9BQC6	MRPL57	Ribosomal	13	1	1	1	102	12.3
Q9NX47	MARCH5	E3 ubiqui	5	1	1	1	278	31.2
O95379	TNFAIP8	Tumor nec	7	2	3	2	198	23
Q86UL3	GPAT4	Glycerol-	2	1	1	1	456	52
Q9NYP7	ELOVL5	Elongatic	3	1	1	1	299	35.3
C9JUN5	CCDC12	Coiled-cc	11	1	1	1	89	10.5
E9PL46	FBXO3	F-box onl	5	2	3	2	129	15.2
Q9BQ48	MRPL34	39S ribos	11	1	1	1	92	10.2
P51178	PLCD1	1-phospha	2	2	2	2	756	85.6
Q86WC4	OSTM1	Osteopetr	3	1	1	1	334	37.2
Q8N584	TTC39C	Tetratric	2	1	1	1	583	65.8
E9PNG8	RNF170	E3 ubiqui	20	1	1	1	45	4.8
Q13322	GRB10	Growth fa	2	1	1	1	594	67.2
Q01831	XPC	DNA repai	2	2	2	2	940	105.9
D6RD44	TCEAL4	Transcrip	16	1	1	1	129	14.3
E7EW18	POLB	DNA polym	3	1	1	1	280	31.3
O43934	MFSD11	UNC93-lik	2	1	1	1	449	49.2
Q8TDB6	DTX3L	E3 ubiqui	1	1	1	1	740	83.5
P10915	HAPLN1	Hyalurona	3	1	1	1	354	40.1
P27144	AK4	Adenylate	4	1	1	1	223	25.3
E9PCT3	CAV2	Caveolin	16	2	2	2	113	12.9
G3V3R7	ATXN3	Ataxin-3	4	1	1	1	329	37.7
O75600	GCAT	2-amino-3	4	1	1	1	419	45.3
Q9H3L0	MMADHC	Methylmal	4	1	1	1	296	32.9
Q12923	PTPN13	Tyrosine-	0	1	1	1	2485	276.7
Q9Y2S6	TMA7	Translati	14	1	1	1	64	7.1
H0YG25	ETV6	Transcrip	9	1	1	1	88	10.3
H0YF29	C8orf82	UPF0598 p	7	1	1	1	261	28.6
A0A140T99PSMB8		Proteasom	5	1	1	1	252	27.8
Q8TF74	WIPF2	WAS/WASL-	2	1	1	1	440	46.3
Q8IW41	MAPKAPK5	MAP kinas	2	1	1	1	473	54.2
G3V4M9	ITPK1	Inositol	3	1	1	1	295	31.7
Q9BSY9	DESI2	Deubiquit	12	2	2	2	194	21.4
Q9NRY2	INIP	SOSS comp	13	1	1	1	104	11.4
A0A0U1RQYCSNK1G1		Casein ki	2	1	1	1	459	52.6
Q7L5Y9	MAEA	Macrophag	2	1	1	1	396	45.3
R4GNH2	FBXO2	F-box onl	13	1	1	1	120	12.8
Q9H490	PIGU	Phosphati	2	1	1	1	435	50
Q92604	LPGAT1	Acyl-CoA:	2	1	1	1	370	43.1
Q9BYC5	FUT8	Alpha-(1,	1	1	1	1	575	66.5

E9PGM9	RBM6	RNA-bindi	1	1	1	1	991	113.9
G5E994	GPR107	G proteir	2	1	1	1	571	63.9
E9PHH9	POLR3C	DNA-direc	2	1	1	1	411	46.2
Q8TAF3	WDR48	WD repeat	1	1	1	1	677	76.2
Q8WUX9	CHMP7	Charged m	2	1	1	1	453	50.9
Q96HH9	GRAMD2B	GRAM doma	4	2	2	2	432	47.8
P05204	HMGN2	Non-histc	9	1	3	1	90	9.4
E5RI96	PDP1	[Pyruvate	6	1	1	1	147	16.8
O75592	MYCBP2	E3 ubiqui	0	2	2	2	4678	513.3
Q9NUN5	LMBRD1	Probable	3	1	1	1	540	61.3
P60604	UBE2G2	Ubiquitir	9	1	1	1	165	18.6
Q86UY6	NAA40	N-alpha-a	5	1	2	1	237	27.2
A2ACR1	PSMB9	Proteasom	5	1	1	1	196	20.9
Q14457	BECN1	Beclin-1	2	1	1	1	450	51.9
Q96SZ5	ADO	2-aminoet	3	1	1	1	270	29.7
F8VPW2	ZDHHC17	Palmitoyl	7	1	1	1	126	14.4
Q8WZA1	POMGNT1	Protein C	2	1	1	1	660	75.2
Q8N201	INTS1	Integratc	1	2	2	2	2190	244.1
Q8NB46	ANKRD52	Serine/th	1	1	1	1	1076	115
A0A0G2JHL	BRD2	Bromodoma	2	1	1	1	613	67.2
Q03393	PTS	6-pyruvoy	6	1	1	1	145	16.4
Q9BVT8	TMUB1	Transmemb	5	1	1	1	246	26.2
Q96B26	EXOSC8	Exosome c	4	1	1	1	276	30
Q9Y5B8	NME7	Nucleosid	3	1	1	1	376	42.5
Q9P032	NDUFAF4	NADH dehy	9	2	2	2	175	20.3
Q9BPX7	C7orf25	UPF0415 p	3	1	1	1	421	46.4
Q96CU9	FOXRED1	FAD-deper	4	2	2	2	486	53.8
A0A087WYW	SIRT2	NAD-deper	4	2	2	2	389	43.3
F8WF50	RABL3	Rab-like	14	2	2	2	129	14
F8VXD5	ORMDL2	ORM1-like	9	1	1	1	119	13.6
Q9UPY3	DICER1	Endoribor	1	1	1	1	1922	218.5
Q8NAV1	PRPF38A	Pre-mRNA-	3	1	1	1	312	37.5
O43598	DNPH1	2'-deoxyr	10	1	1	1	174	19.1
C9JJG2	POLR1B	DNA-direc	9	1	1	1	114	12.7
H0YEP3	NPEPL1	Probable	6	1	1	1	180	19.4
Q9ULW3	ABT1	Activator	3	1	1	1	272	31.1
Q9Y619	SLC25A15	Mitochond	3	1	1	1	301	32.7
F5H0Y3	MLF2	Myeloid l	4	1	1	1	230	24.7
F8WDV0	IPO11	Importin-	1	1	1	1	863	99.6
A0A087X0W	OTUD6B	Deubiquit	4	2	2	1	323	37.3
J3QRG5	CYBC1	Cytochron	5	1	1	1	148	16.7
I3L2J0	CIC	Protein c	0	1	1	1	2514	257.6
Q9NVN8	GNL3L	Guanine n	1	1	1	1	582	65.5
Q96T58	SPEN	Msx2-inte	0	1	1	1	3664	402
Q9UBN7	HDAC6	Histone d	1	1	1	1	1215	131.3
H3BRK1	PARN	Poly(A)-s	3	1	1	1	261	29.4
A0A1B0GUW	CDKL5	Cyclin-de	1	1	1	1	881	98.8
E9PEY4	DTNB	Dystrobre	2	1	1	1	590	67
Q9BXX3	ANKRD30A	Ankyrin r	1	2	2	1	1397	158.7
A0A087X2C	AGRN	Agrin OS=	1	1	1	1	1930	202.2
Q5VTL8	PRPF38B	Pre-mRNA-	2	1	1	1	546	64.4

G3V5E8	GALC	Galactoce	4	1	1	1	211	23.5
C9JWL3	UBP1	Upstream-	9	1	1	1	148	16.8
Q9UBP9	GULP1	PTB domai	3	1	1	1	304	34.5
P19447	ERCC3	General t	1	1	1	1	782	89.2
P36959	GMPR	GMP reduc	3	1	1	1	345	37.4
Q8IWT6	LRRC8A	Volume-re	1	1	1	1	810	94.1
Q5W111	SPRYD7	SPRY doma	6	1	1	1	196	21.7
K7ELL0	GLYR1	Putative	15	1	1	1	52	5.9
Q8IXQ5	KLHL7	Kelch-lik	2	1	1	1	586	65.9
Q9UER7	DAXX	Death dom	1	1	1	1	740	81.3
K7ENL9	RMC1	Regulator	2	1	1	1	609	69.3
Q96B54	ZNF428	Zinc fing	7	1	1	1	188	20.5
Q96S15	WDR24	GATOR com	1	1	1	1	920	101.9
Q1RLN5	ARHGAP12	ARHGAP12	1	1	1	1	799	90.8
E7EPI0	IBTK	Inhibitor	1	2	2	2	1338	148.9
D6R9T3	HMCES	Embryonic	3	1	1	1	282	32.1
P49795	RGS19	Regulator	5	1	1	1	217	24.6
G3XAN8	TIMM8B	Mitochond	8	1	1	1	98	11.1
Q9NRK6	ABCB10	ATP-bindi	2	2	2	2	738	79.1
B8ZZC8	METTL5	Methyltra	3	1	1	1	244	27.8
O43734	TRAF3IP2	Adapter p	3	2	3	2	574	64.6
Q147X3	NAA30	N-alpha-a	3	1	1	1	362	39.3
E9PF16	ACSF2	Acyl-CoA	2	1	1	1	572	63.6
P62273	RPS29	40S ribos	27	2	4	2	56	6.7
C9JCU6	MAP4K2	Mitogen-a	4	1	1	1	307	34.7
Q9ULG6	CCPG1	Cell cycl	2	1	1	1	757	87.3
Q9NVH6	TMLHE	Trimethyl	2	1	1	1	421	49.5
Q6B0J5	HFE	HFE prote	6	1	1	1	345	39.7
P48553	TRAPPC10	Trafficki	1	1	1	1	1259	142.1
F8VSI7	TMBIM6	Bax inhib	6	1	1	1	138	15.1
Q5JPI3	C3orf38	Uncharact	4	1	1	1	329	37.5
H7C5U2	ITGB5	Integrin	2	1	1	1	401	43.7
J3KNE1	CDKN2AIP	CDKN2A-in	8	1	1	1	126	14
A0A087WUCMINOS1		MICOS com	13	1	1	1	62	7
Q9H1K6	TLNRD1	Talin roc	2	1	1	1	362	37.7
Q96Q05	TRAPPC9	Trafficki	1	1	1	1	1148	128.4
Q9C005	DPY30	Protein d	11	1	1	1	99	11.2
K7EIX4	TIMP2	Metallopr	7	1	1	1	109	12.2
Q9NRA2	SLC17A5	Sialin OS	2	1	1	1	495	54.6
Q9NQ92	COPRS	Coordinat	7	1	1	1	184	20.1
Q9BZM5	ULBP2	UL16-binc	4	1	1	1	246	27.4
Q9H2H9	SLC38A1	Sodium-cc	2	1	1	1	487	54
Q9NUI1	DECR2	Peroxisom	4	1	1	1	292	30.8
O95229	ZWINT	ZW10 inte	4	1	1	1	277	31.3
F8W681	DPAGT1	UDP-N-ace	14	1	1	1	57	6.4
Q9UL42	PNMA2	Paraneopl	4	2	2	1	364	41.5
Q9BSR8	YIPF4	Protein Y	4	1	2	1	244	27.1
O14967	CLGN	Calmegin	1	1	1	1	610	70
H3BSB3	MPHOSPH6	M-phase p	8	1	1	1	131	15.5
Q9Y2K7	KDM2A	Lysine-sp	1	1	1	1	1162	132.7
O95684	FGFR1OP	FGFR1 onc	2	1	1	1	399	43

Q96BJ3	AIDA	Axin inte	2	1	1	1	306	35
Q9Y3D3	MRPS16	28S ribos	6	1	1	1	137	15.3
P10109	FDX1	Adrenodox	5	1	1	1	184	19.4
P61962	DCAF7	DDB1- anc	4	1	1	1	342	38.9
P10588	NR2F6	Nuclear r	4	2	2	1	404	43
A0A0A0MTJ	SPOCK3	Sparc/ost	3	1	1	1	344	39.4
Q8N4Q1	CHCHD4	Mitochonc	6	1	1	1	142	16
M0R2A0	EMC10	ER membra	2	1	1	1	371	39
F5H5A3	MAP4K3	Mitogen-a	1	1	1	1	810	91.9
Q9NUD5	ZCCHC3	Zinc fing	2	1	1	1	404	43.6
X6RM59	NT5C3A	5'-nuclec	3	1	1	1	331	37.4
R4GN70	ANKS1B	Ankyrin r	55	1	1	1	20	2.3
Q13888	GTF2H2	General t	2	1	1	1	395	44.4
Q8N3Y1	FBXW8	F-box/WD	2	1	1	1	598	67.4
S4R3I5	NDUFA3	NADH dehy	27	1	2	1	41	4.6
Q9BTT4	MED10	Mediator	7	1	1	1	135	15.7
Q13772	NCOA4	Nuclear r	1	1	1	1	614	69.7
Q14790	CASP8	Caspase-8	3	1	1	1	479	55.4
Q15022	SUZ12	Polycomb	1	1	1	1	739	83
Q9BVG9	PTDSS2	Phosphati	2	1	1	1	487	56.2
Q86YS7	C2CD5	C2 domair	1	1	1	1	1000	110.4
P29083	GTF2E1	General t	3	1	1	1	439	49.4
Q9BTX1	NDC1	Nucleopor	1	1	1	1	674	76.3
P50747	HLCS	Biotin--p	1	1	1	1	726	80.7
Q6ZTI6	RFLNA	Refilin-A	4	1	1	1	216	23.6
Q9H1C3	GLT8D2	Glycosylt	3	1	1	1	349	40
P05230	FGF1	Fibroblas	8	1	1	1	155	17.4
U3KQI2	MEIS2	Homeobox	5	1	1	1	235	26
A0A087WWF	RBM15	RNA-bindi	1	1	1	1	933	102.1
H0Y3V5	THADA	Thyroid a	1	1	1	1	1193	133.9
F8VRQ4	SMARCD1	SWI/SNF-r	4	1	1	1	313	36.8
Q14156	EFR3A	Protein E	1	1	1	1	821	92.9
Q07617	SPAG1	Sperm-ass	2	2	2	1	926	103.6
P20585	MSH3	DNA misma	1	1	1	1	1137	127.3
D6RG18	CCNH	Cyclin-H	3	1	1	1	255	29.5
Q96PX6	CCDC85A	Coiled-cc	2	1	1	1	553	59.9
E7EVJ3	NDST1	Bifunctic	1	1	1	1	825	94.3
Q86V87	FAM160B2	Protein F	1	1	1	1	743	82.3
Q99943	AGPAT1	1-acyl-sn	3	1	1	1	283	31.7
Q9H6E4	CCDC134	Coiled-cc	3	1	1	1	229	26.5
Q14393	GAS6	Growth ar	2	1	1	1	721	79.6
Q99959	PKP2	Plakophil	1	1	1	1	881	97.4
C9JFZ1	SYNJ1	Synaptoja	1	1	1	1	1350	149.2
O43156	TTI1	TELO2-int	1	1	1	1	1089	122
Q96CB9	NSUN4	5-methylc	3	1	1	1	384	43.1
P33947	KDELR2	ER lumen	4	1	1	1	212	24.4
Q96G28	CFAP36	Cilia- an	3	1	1	1	342	39.4
Q6YHK3	CD109	CD109 ant	1	1	1	1	1445	161.6
Q96B96	TMEM159	Promethir	5	1	2	1	161	17.5
Q9HAC8	UBTD1	Ubiquitir	6	2	2	2	227	25.9
A0A1B0GUU	IGHM	Immunoglc	2	1	1	1	474	51.9

K7EQF2	PLCD3	1-phospha	4	1	1	1	195	23
A0A0U1RRC	DOCK7	Dedicator	7	2	2	1	248	27
A0A0A0MRV	FAM213B	Prostamid	5	1	1	1	192	20.7
Q86UP3	ZFHX4	Zinc fing	0	2	2	2	3567	393.5
O75683	SURF6	Surfeit l	5	2	2	2	361	41.4
Q8TAC2	JOSD2	Josephin-	6	1	1	1	188	20.7
E9PS63	RABGAP1L	Rab GTPas	5	1	1	1	190	22.5
O14494	PLPP1	Phospholi	3	1	1	1	284	32.1
Q7L3B6	CDC37L1	Hsp90 co-	3	1	1	1	337	38.8
P08590	MYL3	Myosin li	8	1	2	1	195	21.9
A0A0A0MSZ	CSTF1	Cleavage	4	1	1	1	345	38.4
A0A0D9SGH	SLC9A6	Sodium/hy	1	1	1	1	679	75.8
Q5MNZ6	WDR45B	WD repeat	4	2	2	2	344	38.1
Q9NR48	ASH1L	Histone-l	0	1	3	1	2969	332.6
O94885	SASH1	SAM and S	1	1	1	1	1247	136.6
C9JVH1	VOPP1	Vesicular	5	1	1	1	170	19.1
P78504	JAG1	Protein j	1	1	1	1	1218	133.7
Q99541	PLIN2	Perilipin	2	1	1	1	437	48
F6RY50	SIPA1	Signal-in	1	1	1	1	940	101.8
A0A096LP1	ALG13	Putative	4	1	1	1	162	17.8
F8WDR3	AP4M1	AP-4 comp	11	1	1	1	64	6.8
A0A2R8Y6L	DYRK1A	Dual-spec	1	1	1	1	605	68.4
Q9BX59	TAPBPL	Tapasin-r	2	1	1	1	468	50.2
Q6ZWT7	MBOAT2	Lysophosp	2	1	1	1	520	59.5
O60220	TIMM8A	Mitochond	11	1	1	1	97	11
Q9P2X0	DPM3	Dolichol-	13	1	1	1	92	10.1
Q9P0U1	TOMM7	Mitochond	44	2	2	2	55	6.2
A0A0G2JH3	TAPBP	Tapasin C	2	1	1	1	466	49.5
Q8N573	OXR1	Oxidation	1	1	1	1	874	97.9
Q9Y6V7	DDX49	Probable	2	1	1	1	483	54.2
Q68D91	MBLAC2	Metallo-b	4	1	1	1	279	31.4
Q9P1Y5	CAMSAP3	Calmoduli	1	2	2	1	1249	134.7
Q9NQZ2	UTP3	Something	2	1	1	1	479	54.5
Q70CQ2	USP34	Ubiquitin	0	1	1	1	3546	404
Q9H1C7	CYSTM1	Cysteine-	10	1	1	1	97	10.6
O60285	NUAK1	NUAK fami	1	1	1	1	661	74.3
Q96I36	COX14	Cytochrom	12	1	1	1	57	6.6
Q9HAU0	PLEKHA5	Pleckstri	1	1	1	1	1116	127.4
O95251	KAT7	Histone a	1	1	1	1	611	70.6
Q9Y5Y2	NUBP2	Cytosolic	4	1	1	1	271	28.8
C9JWG4	SLC38A5	Sodium-co	3	1	1	1	272	29.3
Q7Z7F0	KHDC4	KH homolo	2	1	1	1	614	64.8
Q7RTN0	RTN2	Reticulon	2	1	1	1	461	50.4
A0A087WXF	ING1	Inhibitor	6	1	1	1	111	12.6
Q9ULR0	ISY1	Pre-mRNA-	3	1	1	1	285	33
Q969J3	BORCS5	BLOC-1-re	6	1	1	1	196	22.2
F8WDB4	LRWD1	Leucine-r	5	1	1	1	154	16.7
O43704	SULT1B1	Sulfotran	4	1	1	1	296	34.9
H0YAA3	WDR41	WD repeat	4	1	1	1	394	44.5
Q8WZ19	KCTD13	BTB/POZ d	2	1	1	1	329	36.3
E9PKE9	RNF121	RING fing	4	1	1	1	167	19.5

P62877	RBX1	E3 ubiqui	7	1	2	1	108	12.3
C9J0E4	CSTA	Cystatin-	11	1	1	1	63	7.1
Q04844	CHRNE	Acetylcho	1	1	1	1	493	54.7
Q9BWW4	SSBP3	Single-st	3	1	1	1	388	40.4
Q9NVT9	ARMC1	Armadillo	3	1	1	1	282	31.3
B5MDL5	MAPK12	Mitogen-a	3	1	1	1	277	31.8
Q9Y6Y0	IVNS1ABP	Influenza	2	1	1	1	642	71.7
Q96EL2	MRPS24	28S ribos	5	1	1	1	167	19
Q6XYQ8	SYT10	Synaptota	2	1	1	1	523	59.1
Q8WWC4	MAIP1	m-AAA pro	3	1	1	1	291	32.5
B8ZZW5	AVL9	Late secr	1	1	1	1	630	69.8
J3QL63	TVP23B	Golgi app	5	1	1	1	141	16.3
Q17RB0	RTL8B	Retrotran	6	1	1	1	113	13.2
Q14966	ZNF638	Zinc fing	1	1	1	1	1978	220.5
Q13356	PPIL2	RING-type	2	1	1	1	520	58.8
Q8IY63	AMOTL1	Angiomoti	1	1	1	1	956	106.5
Q9Y3M8	STARD13	StAR-rela	1	1	1	1	1113	124.9
Q96NL6	SCLT1	Sodium ch	1	1	1	1	688	80.9
Q92935	EXTL1	Exostosin	1	1	1	1	676	74.6
B1ALY0	PALM2-AKA	PALM2-AKA	2	1	1	1	433	47.6
A0A0C4DGW	C5orf51	UPF0600 p	5	1	1	1	152	17
Q5QPE8	MGME1	Mitochond	3	1	1	1	264	30
Q68DQ2	CRYBG3	Very larg	0	1	1	1	2970	330.4
F8W8Y1	NTM	Neurotrim	5	1	1	1	179	19.7
Q96DY7	MTBP	Mdm2-bind	1	1	1	1	904	102.1
X1WI34	MARC2	Mitochond	5	1	1	1	172	19.8
E9PNL8	DGKZ	Diacylgly	1	1	1	1	707	78.9
Q9NV96	TMEM30A	Cell cycl	3	1	1	1	361	40.7
A0A087WYF	DTNBP1	Dysbindin	3	1	1	1	316	35.5
L0R6Q1	SLC35A4	SLC35A4 u	7	1	1	1	103	11.1
P15407	FOSL1	Fos-relat	3	1	1	1	271	29.4
Q96HQ2	CDKN2AIPN	CDKN2AIP	6	1	1	1	116	13.2
O60563	CCNT1	Cyclin-T1	1	1	1	1	726	80.6
P16220	CREB1	Cyclic AM	2	1	1	1	341	36.7
O43715	TRIAP1	TP53-regu	11	1	1	1	76	8.8
Q5VTE6	ANGEL2	Protein a	2	1	1	1	544	62.3
O43861	ATP9B	Probable	1	1	1	1	1147	129.2
A0A0S2Z43	NEFL	Neurofila	2	1	3	1	284	32.6
C9J719	EBP	3-beta-hy	7	1	1	1	145	16.6
C9J2P0	UBE2E1	Ubiquitin	5	1	1	1	147	16.2
A0A087WVS	MOB2	MOB kinas	3	1	1	1	234	26.6
P13498	CYBA	Cytochrom	5	1	1	1	195	21
E9PHT3	GRAMD1C	GRAM doma	2	1	2	1	495	56.5
Q96AT1	KIAA1143	Uncharact	5	1	1	1	154	17.5
Q8TEA7	TBCK	TBC domai	1	1	1	1	893	100.6
P23229	ITGA6	Integrin	1	1	1	1	1130	126.5
P49662	CASP4	Caspase-4	2	1	1	1	377	43.2
Q3LIE7	DHCR24	Delta(24)	2	1	1	1	427	49.4
U3KQ50	TARS2	Threonine	7	1	1	1	143	15.4
Q5JUR7	TEX30	Testis-ex	5	1	1	1	227	25.6
O14548	COX7A2L	Cytochrom	8	1	1	1	114	12.6

H0YCA5	SPATA5L1	Spermatog	3	1	1	1	258	28.6
B5MD58	SREBF1	Sterol re	1	1	1	1	893	95.8
G3V5K2	NEMP1	Nuclear e	8	1	1	1	110	12
A2ABF8	EHMT2	Histone-l	1	1	1	1	1233	135.3
Q7Z2T5	TRMT1L	TRMT1-lik	1	1	1	1	733	81.7
O15120	AGPAT2	1-acyl-sn	3	1	1	1	278	30.9
G3V1J0	WDR55	WD repeat	4	1	1	1	222	24.5
M0QZR8	POLD1	DNA polym	1	1	1	1	1009	111.6
Q9Y4D1	DAAM1	Dishevele	1	1	1	1	1078	123.4
C9JD84	LTBP1	Latent-tr	1	1	1	1	1340	146.9
Q02487	DSC2	Desmocoll	1	1	1	1	901	99.9
G5E9F5	MPV17	MpV17 tra	7	1	1	1	113	13
Q9Y5T4	DNAJC15	DnaJ homo	6	1	1	1	150	16.4
P05496	ATP5MC1	ATP synth	5	1	3	1	136	14.3
H9KV31	NCAM2	Neural ce	1	1	1	1	819	91.1
C9JPV1	SLC6A6	Transport	8	1	1	1	109	12.1
G3V2H7	TRAPPC6B	Trafficki	8	1	1	1	102	11.7
Q2KHT3	CLEC16A	Protein C	1	1	2	1	1053	117.6
Q8IYK4	COLGALT2	Procollag	1	1	1	1	626	72.9
P42685	FRK	Tyrosine-	2	1	1	1	505	58.2
Q9H2K0	MTIF3	Translati	3	1	1	1	278	31.7
H0YAB2	PLXND1	Plexin-D1	3	1	1	1	380	43.6
H0Y9C8	FAT1	Protocadh	4	1	1	1	285	31.4
P57764	GSDMD	Gasdermin	1	1	1	1	484	52.8
Q6PJF5	RHBDF2	Inactive	1	1	2	1	856	96.6
A4FU01	MTMR11	Myotubula	1	1	2	1	709	79.5
O95503	CBX6	Chromobox	2	1	1	1	412	43.9
Q13206	DDX10	Probable	1	1	1	1	875	100.8
X6RK58	LRRC20	Leucine-r	6	1	1	1	174	19.5
K7ELQ4		Uncharact	2	1	1	1	463	49.9
P53803	POLR2K	DNA-direc	12	1	1	1	58	7
F8VRE5	YAF2	YY1-assoc	10	1	1	1	72	8.1
A0A0U1RQJATRIP		ATR-inter	2	1	2	1	604	65.2
Q53F19	NCBP3	Nuclear c	2	1	1	1	620	70.5
H7C3Y7	NBEAL2	Neurobeac	1	1	1	1	1093	121.6
K7EL74	VMP1	Vacuole m	9	1	1	1	75	9
H3BQG1	CLK3	Dual-spec	2	1	1	1	306	36.4
G5EA09	SDCBP	Syndecan	2	1	1	1	318	34.8
Q9H0C5	BTBD1	BTB/POZ d	2	1	1	1	482	52.7
F5H450	FZD10	Frizzled-	2	1	1	1	454	48.7
Q5BJD5	TMEM41B	Transmemb	3	1	1	1	291	32.5
C9J5X1	IGF1R	Tyrosine-	1	1	1	1	1366	154.7
Q96B70	LENG9	Leukocyte	2	1	1	1	501	53.1
D6REA0	GATB	Glutamyl-	1	1	1	1	516	57.6
F5GWH5	TMEM258	Transmemb	16	1	1	1	49	5.7
P50461	CSRP3	Cysteine	4	1	2	1	194	21
Q9BWJ5	SF3B5	Splicing	13	1	1	1	86	10.1
P17152	TMEM11	Transmemb	4	1	1	1	192	21.5
Q9BW60	ELOVL1	Elongatio	5	1	1	1	279	32.6
Q9HCJ1	ANKH	Progressi	2	1	1	1	492	54.2
O60934	NBN	Nibrin OS	1	1	1	1	754	84.9

Q9H0J9	PARP12	Poly [ADF	1	1	1	1	701	79
Q8N697	SLC15A4	Solute ca	1	1	1	1	577	62
Q9Y2X9	ZNF281	Zinc fing	1	1	1	1	895	96.9
Q86X02	CDR2L	Cerebella	2	1	1	1	465	53
O60637	TSPAN3	Tetraspar	3	1	1	1	253	28
E9PKQ5	CCDC90B	Coiled-cc	3	1	1	1	208	24.3
Q9BZA7	PCDH11X	Protocadh	1	1	1	1	1347	147.5
Q6Y1H2	HACD2	Very-long	4	1	1	1	254	28.4
P50151	GNG10	Guanine r	10	1	1	1	68	7.2
Q9H5V8	CDCP1	CUB domai	1	1	1	1	836	92.9
O15344	MID1	E3 ubiqui	1	1	1	1	667	75.2
A0A2R8Y4I	SPAST	Spastin C	1	1	1	1	583	63.5
Q8NEC6	MTMR1	MTMR1 prc	2	1	1	1	363	39.8
Q9UK59	DBR1	Lariat de	1	1	1	1	544	61.5
A0A087WW6	ABCB7	ATP-bindi	1	1	1	1	713	78.2
Q69YN4	VIRMA	Protein v	0	1	1	1	1812	201.9
O43657	TSPAN6	Tetraspar	3	1	1	1	245	27.5
A0A0G2JJD	ABHD16A	HLA-B ass	1	1	1	1	601	67.5
Q9H147	DNTTIP1	Deoxynucl	2	1	1	1	329	37
P28347	TEAD1	Transcrip	2	1	1	1	426	47.9
Q9BRV8	SIKE1	Suppressc	3	1	1	1	207	23.7
Q9H8H2	DDX31	Probable	1	1	1	1	851	94
H0YEU7	CREB3L1	Cyclic AM	7	1	1	1	123	13.9
A0A2R8Y5Q	SLC11A2	Natural r	3	1	1	1	331	36.4
Q9P000	COMMD9	COMM doma	4	1	1	1	198	21.8
A0A087X0T	NTAN1	Protein N	3	1	1	1	205	23.4
H0YCP6	TMEM63B	CSC1-like	2	1	1	1	520	60.5
P00451	F8	Coagulati	0	1	1	1	2351	266.8
Q8TE73	DNAH5	Dynein he	0	1	1	1	4624	528.7
C9JE50	UXS1	UDP-glucu	4	1	1	1	170	18.8
Q9BTC0	DIDO1	Death-ind	0	1	1	1	2240	243.7
C9JTA8	C2orf76	UPF0538 p	8	1	1	1	85	9.6
Q9Y244	POMP	Proteasom	5	1	1	1	141	15.8
H0YLH9	IL16	Pro-inter	1	1	4	1	611	64.6
O60239	SH3BP5	SH3 domai	2	1	1	1	455	50.4
E9PEE2	NCK2	Cytoplasm	6	1	1	1	110	12.6
A0A2R8YEX	OSBPL2	Oxysterol	2	1	1	1	413	47.9
A0A087WT9	LAMTOR4	Ragulator	10	1	1	1	72	7.9
Q7Z422	SZRD1	SUZ domai	5	1	1	1	152	17
Q6V1X1	DPP8	Dipeptidy	1	1	1	1	898	103.3
Q06190	PPP2R3A	Serine/th	1	1	1	1	1150	130.2
Q5T5C0	STXBP5	Syntaxin-	1	1	1	1	1151	127.5
I3L2S8	NDE1	Nuclear d	6	1	1	1	127	15.2
Q9BXT8	RNF17	RING fing	0	1	1	1	1623	184.5
A0A0J9YWW	APOO	MICOS com	8	1	1	1	90	10
H0YCN4	DCUN1D5	DCN1-like	4	1	2	1	195	22.9
P55789	GFER	FAD-linke	6	1	1	1	205	23.4
B1AL33	UHRF2	E3 ubiqui	7	1	1	1	136	15.3
Q9NXF7	DCAF16	DDB1- and	4	1	1	1	216	24.2
Q96SK2	TMEM209	Transmemb	1	1	1	1	561	62.9
Q9H1K0	RBSN	Rabenosyr	1	1	1	1	784	88.8

Q8TCU6	PREX1	Phosphati	0	1	1	1	1659	186.1
Q9BZL1	UBL5	Ubiquitir	8	1	1	1	73	8.5
Q5BJF2	TMEM97	Sigma int	5	1	1	1	176	20.8
O75030	MITF	Microphth	1	1	1	1	526	58.8
Q9BW61	DDA1	DET1- and	8	1	1	1	102	11.8
Q8WVX9	FAR1	Fatty acy	2	1	2	1	515	59.3
Q8NI51	CTCFL	Transcrip	1	1	1	1	663	75.7
Q9UJX6	ANAPC2	Anaphase-	1	1	1	1	822	93.8
P0C860	MSL3P1	Putative	1	1	1	1	447	51
K7ELS8	SYNGR2	Synaptogy	6	1	1	1	135	14.7
Q9Y2J4	AMOTL2	Angiomoti	1	1	1	1	779	85.7
Q5JR91	KIF2C	Kinesin-l	2	1	1	1	336	37.9
O43663	PRC1	Protein r	1	1	1	1	620	71.6
Q9BZI7	UPF3B	Regulator	2	1	1	1	483	57.7
Q9H7P9	PLEKHG2	Pleckstri	1	1	1	1	1386	147.9
A2A2F0	RALGAPB	Ral GTPas	1	1	1	1	1323	147.3
Q15648	MED1	Mediator	1	1	1	1	1581	168.4
J3QS80	C19orf47	Uncharact	6	1	1	1	168	17.7
I3L130	URI1	Unconvent	16	1	1	1	50	5.5
O75164	KDM4A	Lysine-sp	1	1	1	1	1064	120.6
Q9UJA3	MCM8	DNA helic	1	1	2	1	840	93.6
Q86V42	FAM124A	Protein F	1	1	1	1	546	60.1
Q6ZWJ1	STXBP4	Syntaxin-	1	1	1	1	553	61.6
Q9ULD2	MTUS1	Microtubu	1	1	1	1	1270	141.3
G3V515	ZFP36L1	mRNA deca	4	1	1	1	189	20.4
K7EK57	WIPI1	WD repeat	2	1	1	1	369	41.1
Q9P2K3	RCOR3	REST core	2	1	1	1	495	55.5
H3BNK2	LYRM1	LYR motif	7	1	1	1	107	12.4
Q3KRB8	ARHGAP11B	Rho GTPas	2	1	1	1	267	30.2
E9PGH5	CYP2U1	Cytochrom	2	1	1	1	335	38.7
O95926	SYF2	Pre-mRNA-	2	1	1	1	243	28.7
E9PR71	UVRAG	UV radiat	1	1	1	1	598	67
H7BYP1	TRPM3	Transient	1	1	1	1	1556	177.7
K7EIY6	RNF126	E3 ubiqui	3	1	1	1	283	30.8
K7EPC4	KAT2A	Histone a	9	1	2	1	70	8.4
Q9UHA4	LAMTOR3	Ragulator	6	1	1	1	124	13.6
E9PCW1	GOSR1	Golgi SNA	3	1	1	1	248	28.3
O75818	RPP40	Ribonucle	2	1	1	1	363	41.8
A0A2R8Y42	CCDC9	Coiled-cc	1	1	1	1	587	65.3
Q8N2G8	GHDC	GH3 domai	2	1	1	1	530	57.5
O95239	KIF4A	Chromosom	1	1	1	1	1232	139.8
J3KNF5	CEP290	Centrosom	0	1	1	1	2481	290.4
Q9P0B6	CCDC167	Coiled-cc	6	1	1	1	97	11.5
O95210	STBD1	Starch-bi	2	1	1	1	358	39
Q96IG2	FBXL20	F-box/LRR	2	1	1	1	436	48.4
Q01974	ROR2	Tyrosine-	1	1	1	1	943	104.7
Q6AWC2	WWC2	Protein W	1	1	1	1	1192	133.8
H0YC48	HACE1	E3 ubiqui	4	1	1	1	175	19.9
A0A087WYI	INTS11	Integratc	1	1	1	1	502	56.3
Q8IUA7	ABCA9	ATP-bindi	0	1	1	1	1624	184.2
Q8WZ42	TTN	Titin OS=	0	1	2	1	34350	3813.7

E9PFH2	KDM5D	Lysine-sp	0	1	1	1	1476	166.8
P24311	COX7B	Cytochrom	9	1	1	1	80	9.2
Q96NW7	LRRC7	Leucine-r	0	1	1	1	1537	172.5
P18615	NELFE	Negative	3	1	1	1	380	43.2
Q8NA19	L3MBTL4	Lethal(3)	1	1	1	1	623	71.1
O94887	FARP2	FERM, ARH	1	1	1	1	1054	119.8
Q8WWI5	SLC44A1	Choline t	1	1	1	1	657	73.3
O95644	NFATC1	Nuclear f	1	1	1	1	943	101.2
Q9UKK6	NXT1	NTF2-rela	5	1	1	1	140	15.8
F8VWZ8	ARHGAP29	Rho GTPas	1	1	1	1	1035	117.4
Q14520	HABP2	Hyalurona	2	1	1	1	560	62.6
A0A087X0C	GLS2	Glutamina	2	1	1	1	337	37.1
Q15835	GRK1	Rhodopsin	2	1	1	1	563	63.5
P01008	SERPINC1	Antithrom	2	1	1	1	464	52.6
Q5T2S9	ARMC4	Armadillo	2	1	1	1	389	44.3
Q8IWV8	UBR2	E3 ubiqui	0	1	1	1	1755	200.4
Q12933	TRAF2	TNF recep	2	1	1	1	501	55.8
Q9Y227	ENTPD4	Ectonucle	1	1	1	1	616	70.2
A6NC98	CCDC88B	Coiled-co	1	1	1	1	1476	164.7
Q96FI4	NEIL1	Endonucle	2	1	1	1	390	43.7
Q8TD26	CHD6	Chromodom	0	1	1	1	2715	305.2
Q7Z7E8	UBE2Q1	Ubiquitin	1	1	1	1	422	46.1
E9PKV2	MRPL17	39S ribos	6	1	1	1	142	16.4
Q3MIP1	ITPRIPL2	Inositol	2	1	1	1	535	58.4
Q9BZ68	FRMD8P1	Putative	2	1	1	1	369	41.1
P46019	PHKA2	Phosphory	1	1	1	1	1235	138.3
Q9C0D5	TANC1	Protein T	0	1	1	1	1861	202.1
O15235	MRPS12	28S ribos	6	1	1	1	138	15.2
A0A0A0MSC	SLIT3	Slit homo	1	1	1	1	1393	153.7
H7BZN3	RIF1	Telomere-	1	1	1	1	845	93.6
Q9UBQ6	EXTL2	Exostosin	2	1	1	1	330	37.4
Q8N2G6	ZCCHC24	Zinc fing	4	1	1	1	241	26.9
Q9BXY5	CAPS2	Calcyphos	1	1	1	1	557	63.8
H0YBS1	INTS8	Integrato	1	1	1	1	800	91.2
P51854	TKTL1	Transketo	1	1	1	1	596	65.3
H0YBT8	TACC1	Transform	2	1	1	1	454	50.5
Q9BYN0	SRXN1	Sulfiredo	5	1	1	1	137	14.3
Q86YF9	DZIP1	Zinc fing	1	1	1	1	867	98.6
H0YMT3	WDR73	WD repeat	3	1	1	1	178	20.1
O60673	REV3L	DNA polym	0	1	1	1	3130	352.6
M0R228	KXD1	KxDL moti	5	1	1	1	131	15
Q9P2G4	MAP10	Microtubu	1	1	1	1	905	100.3
Q6ZW61	BBS12	Bardet-Bi	1	1	1	1	710	79
O00635	TRIM38	E3 ubiqui	1	1	1	1	465	53.4
Q16654	PDK4	[Pyruvate	1	1	1	1	411	46.4
K7ERQ2	FAM210A	Protein F	4	1	1	1	178	20
P38936	CDKN1A	Cyclin-de	4	1	1	1	164	18.1
Q8N1G2	CMTR1	Cap-speci	1	1	1	1	835	95.3
A6NF31	OFD1	Oral-faci	1	1	1	1	872	100.9
Q6TFL3	CCDC171	Coiled-co	1	1	2	1	1326	152.7
P55199	ELL	RNA polym	1	1	1	1	621	68.2

Q02410	APBA1	Amyloid-b	1	1	2	1	837	92.8

calc. pI	Score Se(	UCA2	UCA3	UCA4	WJ1	WJ2	WJ3
6.15	2040.52	90.7	92.4	91	104.7	103.3	103.8
5.96	1836.84	90.9	90.5	89.6	112.4	112	112
6.06	2706	97.7	98.1	97.4	93.8	93	93.1
5.6	2625.4	101.3	102.1	101.5	90.5	90.1	89.7
6.07	1495.51	102	102.5	101.5	93.8	93.7	93.6
5.73	1261.9	96.4	96.9	96.2	102.6	102.9	102.7
5.97	1330.66	92.8	93.6	93.3	104.9	104.4	104.7
6.4	922.24	95.5	95.2	95.3	97.7	97.3	98
5.35	777.91	101.5	103.5	101.7	106.4	105.6	105.1
5.34	762.21	96.4	94.1	109.1	104.5	111.1	109.9
5.54	857.63	101.5	100.9	100.2	89.2	89.4	89.1
5.39	601.03	97.9	96.3	96.3	95.6	96.4	97.1
5.39	593.09	98.2	97.5	106.4	98.2	105.5	100.9
5.57	645.15	101	101.3	102.2	106.2	105.6	105.8
5.41	1110.66	95.9	96.5	97	92.7	92.3	93.2
5.66	843.7	100.5	101.5	100.5	91.5	90.5	91.1
5.8	753.37	92.7	91.8	92.6	119.9	120.6	121.1
6.18	564.45	99.6	100	98.4	93.5	93	93.8
5.69	664.25	96.4	97.5	96.6	99.3	98.9	97.6
4.81	562.95	99.2	100.3	99.9	111.7	112.3	111.2
7.12	457.43	100.5	101.8	100.8	106	107.4	106.8
5.69	998.92	102	100.9	101.6	88.2	83.2	84
6.48	562.2	99.3	99.7	100.7	95.1	94.9	95.9
5.71	593.87	106.8	107.6	106.1	87.9	88.4	87.9
5.44	911.8	100.8	101.4	100.9	91.4	91.1	92.1
8.6	556.35	108.9	110.1	109	118.7	116.8	117.1
5.53	478.19	71.7	70.1	69.8	98.5	98.7	98.5
6.44	447.88	96.2	96.8	96	102.2	102.5	103.2
5.16	963.45	100.7	101.9	100.9	100	99.6	99.9
7.84	1025.25	100.9	101.2	101.3	96.8	96.5	96.9
5.03	785.47	101.7	102.4	102.6	104.1	103.3	103.7
5.52	692.49	99.9	100.7	101.1	102	102.2	102.3
9.01	474.45	90.9	91	90	118.5	118	119.5
7.33	388.27	99.9	98.7	99.7	99.3	99	100.4
5.02	663.78	99.1	99.2	99.8	102.9	102	101.4
5.12	1700.09	102.4	101.9	106.9	98.3	105.1	100.5
6.83	622.13	96.6	97.9	96.6	105.9	105	104.8
5.43	376.29	117.3	86.8	101.1	137.9	138.1	139.3
6.23	372.1	101.5	101.6	102.2	116.1	116.3	116
7.02	619.15	108.5	109.5	108.5	96.7	96.8	96.6
7.33	497.24	93.1	92.4	91.7	105.3	105.1	105.4
5.19	364.47	98.2	98.3	98.5	99.7	100.2	101.1
5.17	3029.45	96.4	98.4	100.7	92	97.9	94.6
5.6	463.35	99	99.6	98.1	91.8	91.9	90.5
6.11	574.02	89.1	91.9	90.5	86.6	87.3	85.4
7.02	580.84	103.6	96.1	91.1	88.9	109	105.4
5.92	576.4	101.5	100.9	100.7	97.2	95.8	96.2
5.66	580.7	94.3	100.2	95	77.7	79.5	75.6
7.47	311.28	102.3	100.8	99.5	99.9	98.1	100
5.59	665.25	102.1	102.8	102.7	73.6	73.6	74.1

6.4	539.52	105.5	106.7	106.6	96.5	96.4	95.1
4.84	656.67	105.8	106.9	105.1	93.2	94.1	93.3
6.68	295.45	114	113.6	113.1	102.3	101.7	103.5
8.69	588.65	101.6	101.4	105.2	93.9	97.1	94.8
5.26	364.14	97.5	97.9	97.8	99.2	99.7	98.8
5.39	1684.6	96.1	97.4	102.6	88.5	97.9	92
5.07	408.05	104.3	105.6	104.6	97.5	96.4	96.1
5.22	320.72	104.2	104.2	103	96.7	95.8	95.9
5.48	356.81	98.2	99.7	97.7	102.5	100.5	102.4
8.46	1270.02	102.9	103.3	107	91.3	97.2	94
5.6	435.62	99.6	100.4	100.4	94.5	93.7	93.8
4.87	607.95	100.5	101.6	102.1	93.6	94	93.1
6.87	339.37	99.6	100.3	99.3	101.4	100.7	100.5
5.76	325.11	96	96.5	95.6	100.6	100.4	99.2
8.03	669.63	107.4	105.2	101.8	86.9	90	86.9
5.64	258.95	102.9	102.6	103.4	97.7	98.1	97.9
6.35	492.27	105.7	105.3	104.4	94.3	94.7	95.2
6.51	233.29	98.5	99.4	99.3	98	96.4	97.8
4.94	259.52	99.8	99.7	98.9	99.4	98.4	97.6
7.66	291.12	99.9	99.4	99.2	99.2	99.6	99.4
8.12	358.14	129.8	131.4	130.4	81.5	79.5	80.8
5.87	349.5	97.8	99.1	99.2	106.3	106.2	105.9
5.6	333.38	100.4	99.7	99.4	103.7	101.9	102.6
5.39	244.39	99	97.1	98.1	96.6	95.8	96.4
7.55	314.77	108	104.2	104.2	94.5	95	92.4
9.01	1043.15	99.1	102.1	100.5	98.9	100	98.5
7.39	497.84	98.3	98.9	100.3	100.3	102.9	100.6
7.17	310.44	99.3	98.7	95.4	98.8	96.7	96.5
6.13	236.22	98.6	96.4	95.9	104.9	105	104.8
5.02	215.04	105.4	104.2	105.5	105.3	105.4	106.3
6.16	316.63	94.5	94.7	95.2	106.1	105.3	105.9
5.31	238.78	104.1	98.2	102	90.6	91.9	93.7
5.33	238.26	100.1	101.1	99.9	102.8	101.5	102.6
8.1	402.02	98.6	98.8	96	98.2	97.8	97.2
4.44	517.21	101.4	104.3	104.3	97.7	98.7	97.1
5.67	223.09	99.5	97.8	99.2	97	96.2	96.9
7.75	698.07	93.1	93.3	94.3	98.5	102	99.6
5.06	672.79	97.6	98.9	99.5	99.4	98.9	100.3
6.61	305.5	97.3	96.5	97.5	110.8	112.4	113.7
4.89	780.04	95.9	96.7	96.4	101.1	100.3	101.8
5.07	677.98	95.7	98	98	100.4	103.4	101.6
7.83	267.22	98.9	99.7	100.5	102.3	100.3	102.9
6.52	238.44	101.2	102.2	101.4	95	95.6	94.2
6.65	383.58	101.3	104.7	104.9	91	92.9	92
5.45	393.54	108.3	108.6	106	63.5	63	64.6
5.22	324.77	111.4	111.1	110.5	66.6	66.1	65.8
5.66	270.38	97.7	98.4	98.7	103	102.7	104.5
4.89	710.34	98.1	96.2	97.8	102.6	102	104.5
8.7	274.79	102.6	103.3	104.1	104.9	104.5	105.2
6.49	257.74	99.4	98.4	97.7	102.1	102.2	104.2
5.39	224.59	96.7	97.5	97.1	107	106.7	109.6

5.48	326.16	97.2	100.1	98	107.3	106.5	105.6
5.34	239.52	98.3	97.1	97.2	101.6	101.9	101.1
6.46	259.17	99.4	99.2	100.4	102.4	102.4	102.1
6.65	346.92	84.8	91.7	85.6	105.4	103	105.6
6.29	216.55	96.8	97.1	97	98.6	97.4	99.2
5.66	346.73	92.4	92.1	91.3	124.5	123.5	123.4
7.02	395.08	94.9	96.3	96.1	102	100.4	100.1
6.27	341.63	101.9	103.8	102.1	98.8	95.6	97.9
5.68	185.02	98.2	99.5	102.5	101.9	105.1	102.8
5.53	201.2	100	100.8	100.1	96.4	93.9	93.2
8.06	298.41	101.8	104.1	103.3	92.5	92.8	91.2
6.79	211.79	99.1	98.8	97.7	105.6	105.1	106.4
4.72	423.85	98.1	100.1	98.9	92.9	92.3	91.6
4.93	176.58	92.9	93.4	92.9	108.9	108	109.2
6.11	222.33	100.5	98.9	98.6	102.1	102.4	105.3
5.12	203.95	104.5	101	100.5	102.4	101.3	102.7
6.05	215.75	97.8	98.8	100.1	100.7	100.4	99.1
8.84	751.36	98	99.3	100.8	78.5	81.6	80
7.18	174.58	93.5	92.2	93.3	90	87.8	89.1
6.64	234.67	102.2	104	102.1	108.4	107.3	106.6
6.47	246	99	98.4	98.3	98.6	98.2	99.8
8	198.57	111.4	112.7	111.4	90.1	91.7	91.7
5.74	274.59	92	93.4	93.2	80.7	79.6	81.1
6.05	263.41	98.7	95.6	96	94.8	89.7	84.8
5.47	224.15	98.3	99.3	98.7	98.4	99.8	99.7
6.71	292.31	86.9	87.4	85.6	105.2	105.3	104.8
8.09	575.72	99.6	99.4	97.5	93.5	91.8	91.4
5.49	183.56	102.7	102.5	100.8	99.2	98.3	99.2
5.21	221.86	109.7	111.3	110.6	88.2	89.5	88.8
7.65	222.86	99.7	100.8	101	101.8	101.5	100.5
4.74	398.46	96.9	98.7	97.2	84.4	86	84.8
7.3	168.03	109.9	124	97.6	68.1	83.3	83.6
4.69	485.33	100	101	99	92.6	92	91.3
6.21	177.91	99.1	98.4	99	96.7	95.5	97.3
5.38	229.89	97.2	97.3	97.2	98.3	97.4	98.3
6.8	214.08	104.3	102	102.3	97	95.9	97.4
5.4	265.07	100.1	98.5	100.6	102.4	102	102.1
4.89	660.26	91.4	84.9	93.5	107	91.3	111.3
8.75	136.1	101.5	98.6	101.7	96.1	94.3	97
5.14	247.87	81.2	77.9	76.7	115.9	117.3	116.8
6.15	181.08	97.7	99.7	98.5	94.6	97.3	94.7
6.49	240.3	103.2	103.5	102.6	94.8	94.4	95.6
5.27	217.99	100.7	100.7	100.3	100.2	101.3	99.6
7.3	186.63	100.9	99.5	98.5	102.3	101.6	102
4.94	183.4	107.9	104.9	106.3	95.3	93.5	95.4
5.62	298.64	101.8	104.5	101.3	99.3	98.9	98.5
6.8	218.09	99.3	102.2	101.5	105.6	102.8	103.4
9.41	180.84	98.1	95.6	96.5	95.7	96.5	98.6
6.84	209.91	102	99.6	102.2	108.6	106.1	105.9
6.01	228.31	101.2	103.3	101.4	95.2	94.4	94.1
6	263.37	102.4	104.2	104	113.2	113.1	112.9

5.78	263.12	97.9	102.8	97.4	137.8	125.6	128.8
7.8	147.9	99.4	101	100.2	105.7	104.4	105.4
8.34	412.54	94.3	95.9	96.1	100.5	100.2	99.1
8.84	150.19	103.8	103.5	103.2	107.5	106.8	107.6
9.1	180.2	96.1	95.5	95	97.8	99.9	98.3
5.63	160.24	101.4	100.6	101.6	98	97.5	98.2
4.93	400.1	103.2	103.1	101.5	97.8	98.2	102.8
5.36	151.4	90.4	90.4	89.8	116.7	117.8	118
5.78	215.14	101	99.9	100.7	97	96	97.3
6.68	196.89	101.2	99.9	99.5	98.4	97.2	97.1
7.46	178.37	99.1	98.6	99.3	98	97.6	97.2
4.45	202.45	94.9	93.2	97	113.5	110.7	112.6
4.64	211.95	96	96.8	97.2	111.1	110.3	109.6
5.92	305.63	104.1	104.8	103.4	97.8	98.2	96.7
7.66	213.96	101.3	101.1	100.8	100.1	100.6	100.2
6.86	186.91	103.3	102.7	103.5	86.8	86	87.8
5.53	117.9	99	98.7	99.6	95.5	97	97.4
5.39	286.77	101.8	102.9	103.5	98.9	98.8	99.6
5.06	169.7	94.5	95.6	94.1	98.3	96.9	98.5
6.9	163.53	103.4	103.7	103.9	98.9	97.9	98
4.74	244.87	99.7	100.9	101.6	100.3	100.3	101
5.44	272.3	96.7	97.9	95.8	95	95.4	96.3
5.24	210.61	136.7	140.5	141.3	83.2	80.3	81.9
4.7	223.95	102.7	103.2	103.2	117.4	115.6	116.2
6.61	163.36	96.8	97.6	98.5	110.7	111.1	110.2
4.6	283.46	99	101	98.8	103.1	102.7	101.8
7.23	164.66	98.5	96.9	97.8	99.7	98.3	98.4
6.14	203.64	103.4	103.4	102	97.6	97	97.7
8.37	204.34	102.9	105.5	105.6	105.6	105	105.2
7.03	189.21	96.1	95.5	94.6	93.8	93.2	92.4
8.59	184.19	101	102.5	99.8	103.5	105.3	104.6
4.77	332.78	100.8	103.2	101	104.1	103.3	102.1
5.95	194.55	100.9	101	100.2	100.2	97.3	99.2
7.3	139.4	100.3	98.2	100.8	97.4	95.5	98.2
7.24	289.5	92.9	93.2	93.7	107.9	107.1	107.5
6.2	144.85	100.6	100	102.7	105.3	103.7	105.1
5	134.53	99.6	99.7	99.2	100.4	100	102.3
5.88	240.25	97.1	99.5	97.7	97.6	96	97
5.05	265.5	97.9	98.1	98.4	99.8	98.2	98.1
5.44	132.55	102.4	100.1	102.2	100.5	96.9	100.8
5.2	170.67	100.4	100.5	98.4	104.8	103.3	104.4
6.06	135.17	102.4	102.7	102.7	106.1	105.1	106.3
7.03	179.5	97.8	98.4	97.1	97.6	95.3	94.5
5.6	158.18	102.3	100.8	99.6	94.7	92.8	94.7
7.11	149.48	99.3	99.2	96.7	96.6	96	96.2
4.41	145.36	134.3	132.3	136.1	91.7	92.4	90.5
9.13	195.67	101.8	99.1	99.6	102.2	103.2	102.4
7.12	200.65	100.8	103.5	103.2	97.9	96.5	97.6
5.39	160.1	99.7	96.4	97.4	101.7	101.4	102.2
4.91	155.01	99.2	96.6	98.5	95.9	97.9	97.5
7.18	181.32	100.4	99	97.5	105.7	106.3	105.6

4.68	407.93	99.8	94.5	94	92.7	94.9	91
5.22	140.45	99.4	98	97.4	101.3	100.4	102.6
4.69	413.4	105.4	108.8	107	98.5	98.2	97.6
4.79	396.09	99.8	101.2	98.8	101.2	101	99.3
7.05	172.43	94.9	96.8	96.2	98.9	98	98.5
5.4	167.21	90.9	91.3	89.4	104.4	104.5	104.7
6.95	131.07	101.3	100	99.9	99.5	99.7	99.4
8.88	133.11	103.2	103.4	101.9	110.7	111	111.7
6.04	128.86	99.8	98.1	96.5	99.4	98.6	97.8
6.67	188.83	100.5	99.7	99.9	99.9	100	99.9
6.98	164.28	100.4	100.2	97.5	97.4	95.9	97
8.21	170.13	106.2	107.5	107.1	91.3	90.2	88.3
5.33	121.58	98.6	97.5	99	96.1	96	94
7.18	254.42	97.1	97.2	98.7	99.2	98.5	98.7
4.78	203.02	98	97.9	97.9	99.6	102.3	100.8
6.21	165.67	99.4	98.1	98.3	100.6	101.6	99.6
5.24	153.13	102.1	102.9	101.5	98.3	99.3	99.9
6.38	187.28	101.6	101.7	102.1	100.5	103.3	101.5
7.93	140.77	103.3	99.8	99.8	98	100.2	96.6
4.36	120.75	124.2	125.6	121.6	119.5	118.5	118.7
7.39	183.82	92.3	92.1	89.7	80.6	80.5	82.5
5.81	204.39	101.9	103.1	104.9	107.1	106.8	106.2
5.67	160.88	100.4	98.6	100.6	96.9	96.2	96.2
6.52	146.29	102.9	102.6	102.6	88.8	88.7	88.2
5.48	134.9	113.8	114.9	117.4	92.1	91.2	89.8
8.25	262.51	101	102	101.3	94.4	97.2	95.5
8.68	258.17	98.7	99	98.3	96.5	95.3	97.6
4.98	180.61	97.3	98.4	98.5	102.8	103.6	102.3
9.26	187.6	100.5	100.4	103.8	102.2	106.4	103.3
8.76	158.93	102.3	102.5	103.2	106.2	109.4	106.4
4.63	187.39	87.3	89.8	88.1	125.5	125.6	124.4
7.33	285.96	124	80.2	102.7	94.5	87.3	102.2
8.85	185.18	101.9	101.8	104.5	109.3	107.1	107.2
8.18	150.81	103.3	105.1	104.9	103	100.6	101.7
6.25	131.65	97.5	95.9	94.9	105.8	105.3	106.9
5.94	170.09	99	103	117.4	108.1	78.4	103.4
6.67	229.56	98.7	99.3	99.9	102.5	101.7	100.2
6.37	254.11	100.6	102.5	102.3	104.7	101.3	102.5
5.11	154.75	99.8	99.3	100	99	96.1	97.1
5.16	154.89	105.7	108.2	107.9	111.1	108.5	108
7.69	194.83	98.6	101.8	99.9	106	102.7	103.9
4.68	119.84	98.3	94.4	96.7	107.6	107.9	107.4
6.71	154.28	99.8	100.5	101.9	94.6	94.7	93
6.6	131.08	95.4	93.5	92.5	93.1	92.3	92.9
5.59	165.91	97.8	98.7	98.3	86.8	86	86.3
7.08	177.28	98.4	98	99.9	98	100.7	98
6.28	159.93	113.4	112.9	117.1	105	104.7	106
5.1	123.29	104.4	106.5	106.3	126.2	127.2	123.9
4.78	252.57	100.7	100.5	100	109.8	109.5	108.3
7.59	127.1	101	97.9	98.1	100.5	101.5	100.6
9.16	158.76	68.8	104.9	97.8	85.7	93.2	183.9

10.61	214.53	99	99.9	99.6	104.8	104.2	105.3
4.41	175.92	104.3	104.5	103.3	97.7	95.4	95.6
4.78	232.13	99.8	102.4	100.4	104.6	104.2	102
7.01	153.51	92	92.6	90.6	94.6	94.9	96.7
11.06	212.79	99.2	100	101.4	104	102.8	103.4
5.94	122.38	90.7	89.2	91.4	108.9	110.1	112.1
6.46	115.06	103.1	99.1	98.2	105.6	106.6	107.5
4.78	296.44	102.6	105	103.2	103.4	98.5	100.3
7.25	148.38	96.2	96.3	94.8	108.7	107.8	109.1
4.63	352.18	158.2	156	146.1	58.2	54.3	60.8
6.84	152.81	102.8	99.2	99.1	96.5	96.1	96.3
6.71	133.08	97.9	101.1	99.3	103.4	100.7	100.9
5.59	153.03	105.8	105.1	106.5	107.9	104.9	105.8
8.13	290.96	97.8	98.4	97.6	98.5	100	100.2
5.96	144.16	98.8	97.7	97.4	97.8	97.1	99.4
6.84	144.67	93	91.5	91.3	100.9	99.8	100.5
5.97	228.12	102.6	102.8	102.4	97.2	97.4	96.4
5.14	153.7	98.6	97.5	98.4	100.8	100.6	101.6
6.7	119.72	97.4	98.5	99.6	102.5	104.1	104
6.61	129.01	100.5	97.9	101.2	103.2	100.9	103.1
6.38	156.41	97.1	98.8	98.1	104.7	104.6	104.8
7.11	118.36	100.5	99.5	99.7	111.3	110.3	111.4
6.16	119.91	100.3	98.8	97.9	100.6	99.6	101.1
5.57	164.29	99.8	96.7	99.8	99.6	102.8	101.3
4.88	272.88	94.8	98.3	96.3	101.2	100.6	101.8
6.92	124.83	104.8	103.5	101.6	101.5	101.1	103.5
6.05	189.8	97.6	100.7	100.2	102.4	101.4	101.1
9.26	148.2	102.1	103.1	103.6	104.9	105.3	104
6.95	128.8	95.2	93.9	92.4	97.9	94.4	95.3
5.26	126.17	101.3	100.8	101.3	103.6	102.3	103.9
7.55	152.96	98.2	101.1	100.2	90	88.4	89.7
7.23	140.25	97.1	97.4	97.6	104.8	103.9	103.3
7.55	142.11	104.4	101.6	101.3	100.1	99.7	100.2
8.95	139.18	104.4	105.6	104.7	108.3	107.4	104.7
5.71	175.28	100.2	103.3	100.7	97.5	94.2	95.6
6.93	116.76	104.1	104.8	104.5	101.4	102.5	104
6.15	120.23	96.9	99.3	98.8	108.1	107.6	107.2
6.6	155.98	97.1	98.3	97.6	98.4	98.1	99.9
8.72	172.46	104.5	107.1	106.3	111	110.3	107.8
8.75	116.01	105	105.5	108.1	98.9	98.1	97.6
9.66	253.97	102.4	100.9	101.5	105.2	103.8	104.3
10.18	160.08	103.4	101.9	99.7	103.9	105.6	101
8.27	328.51	104.2	107.7	105.7	99.5	98.7	96
8.57	246.09	92.3	95.8	93.8	77.2	79.7	77.1
7.61	131.97	106.4	100.4	100.8	102.3	107.7	102.7
8.13	147.84	103.8	103.7	103.9	106.4	105.6	104.2
8.15	158.89	90.7	91.2	91.2	97.7	96.5	97.4
6.7	147.84	101.6	100.3	99.2	92.7	91.7	91.4
4.86	87.82	96.9	98.4	99.2	109.9	114.1	115.6
5	118.7	97.6	99.5	98.6	105.2	105.3	104.9
5.07	114.87	99.2	102.1	98.3	108.4	104.2	124.5

7.05	130.65	96.7	98.3	97.1	99.5	96.7	98.9
5.49	125.13	98.3	98.7	98.4	104.8	106.4	105.9
9.31	123.81	91.5	91.8	90.9	96.9	95.4	93.6
5.68	105.61	104.9	106.4	105.6	95.9	93.3	92.9
8.65	153.15	99.6	102.2	103.9	108.2	110.9	107
7.77	149.56	86.1	88.5	88	94.5	94.8	93.9
7.14	129.08	100.4	99.9	101.4	104.3	105.9	105.8
7.09	145.97	101.1	99.8	99.4	105.3	104.4	103.5
9.13	185.14	102.8	102.8	102.7	102.8	103	101.6
5.1	136.19	96.2	96.9	96.5	88.9	88	88.4
7.55	143.71	119.4	126.4	131.3	69.5	78.3	79.1
5.31	117.76	101.7	99.6	99.2	104.6	105.6	103.1
4.82	284.93	98.5	100.5	100	91.9	91.7	89.8
8.56	107.65	101.1	103.4	101.6	98.1	98	96.2
9.31	105.67	101.6	103.7	103.5	110.5	107.7	107.7
7.17	119.67	92.1	92.2	91.4	96.3	95.7	93.9
7.12	124.8	100.7	100.7	99.8	96.7	96.5	95.6
5.83	106.5	100.8	101.1	101.2	97.3	97.3	100.4
5.8	101.8	96.1	96.5	98.6	95.9	96.1	96.4
6.76	131.05	91.4	91.4	90.4	93.5	92.6	91.5
5.36	114.22	101.4	101.8	102.5	99.9	96.4	100.8
5.62	123.45	95.8	98.3	96.5	104	104.1	104.2
6.71	134.4	98.7	100.3	102.4	95.9	94.3	94.9
5.08	187.44	103.2	103.1	103.2	94.3	96.1	96.4
6.34	106.66	99.1	98.4	97.7	105	107.1	106.9
7.12	120.46	101.5	102.7	101	107.2	106.8	106.3
6.33	149.03	88.7	91	90.4	104.5	105.3	104.2
5.77	129.14	104.4	104	102.9	108	110.1	107
9.41	269.76	102.1	105.9	102.2	97	96.3	96.5
4.82	167.98	96.3	96.6	95.5	99	97.5	98.1
6.4	263.27	97.1	99.3	97.7	90.9	90.5	89.7
5.77	119.71	98.7	97.1	99.5	108.8	107.5	108.2
4.89	192.3	100.2	96.9	96.9	97.1	99	97.6
5.88	135.56	98.1	98.5	97.3	104.9	103	102.4
4.82	118.32	99.7	96.1	97.2	104.3	102.8	106
9.04	151.21	102.6	100	100.4	96.5	97	95.1
5.64	258.92	96.6	99.2	99.8	93.3	93.8	92.8
9.44	152.74	105.6	105.6	105.5	109.3	112	110.8
5.54	168.73	103.1	104.2	101.8	106.4	106.9	106.7
9.41	120.1	97.6	97.4	97.9	106.4	105.3	104.4
7.09	140.42	110.6	110.9	110.7	80.3	80.5	81.8
6.76	110.99	121.2	102.1	99.4	94.2	105.1	91.5
9.28	116.01	98	98.4	99	116.3	117.8	118.5
6.38	192.64	101.7	105	102.3	88	87.1	87.6
4.64	272.11	100.3	102.7	109.9	82	86.2	81.9
6.48	129.14	99.7	97.8	97.6	105.3	99.7	103.1
6.48	111.63	101.1	101.5	98.9	100.7	101.5	100.9
8.95	184.64	102.5	102.6	103.4	108.9	108.5	107
5.06	139.17	93.6	93.4	93.7	108	107.4	106.4
8.76	122.64	105.7	103.5	102.1	95.6	94.2	94.5
8.09	109.38	100.8	100.9	99.8	96.6	95.4	97.9

5.01	106.06	99	98.6	99.7	99.2	98.6	99.1
5	115.79	103.8	102	104.3	105.3	106.3	106.7
6.23	96.99	103	97.4	99.3	103	103.1	103.7
7.15	100.94	100	99.3	99.3	100.5	99	99.2
5.69	137.06	98.4	100	97.5	98.9	99.1	100
8.78	91.56	103.7	99.7	102.1	100.3	97.6	104.4
7.65	141.73	97.5	99.5	98.1	100.2	99.2	99.3
6.04	109.1	104.4	103.1	105.1	105.3	107.2	108.3
5.83	138.83	99.7	100.3	100.4	82.9	83.3	82.7
7.09	104.21	95.4	92.8	91.8	101.4	101.2	102.1
8.84	110.09	99.1	102.1	99.5	96.6	93.1	93.4
6.06	88.24	98.3	98.1	99.6	100.1	104.7	101.1
7.8	125.06	99.4	100.3	100.9	97.8	98.5	100
6.98	199.16	92.8	94.4	92.1	91.6	90.8	91.5
6.86	109.32	93.6	94.2	94.8	98.7	97.7	96.7
6.25	148.05	97.8	97.5	95.9	95.9	95.8	95.2
8.97	138.04	101.8	100.8	101.5	110.5	109.9	108.8
8.48	172.32	98.6	99.1	101	97.8	98.5	98.3
6.32	88.68	100.8	101.2	102.1	101	97.8	100.8
8.27	345.28	102	102.9	102.3	94.2	93.6	93.1
7.81	277.83	104.5	104.3	106.5	92.5	95	93.8
9.13	169.77	106.9	106.3	106.8	107.5	110.3	105.1
5.78	102.53	103.4	102.6	103.6	109.8	111.9	108.8
6.81	148.63	97.9	98.8	98.1	102	100	101.6
8.48	98.54	97.8	98.8	97.2	104.2	103.6	104.4
5.73	88.61	96.6	101.2	95.8	98.1	100.2	100.9
7.37	135.16	97.3	101	100.3	117.8	116.2	117.7
6.27	88.45	100.6	97.3	101.8	95.4	102.1	99
7.08	87.66	98.7	101.4	98.5	97.4	94.8	96.8
5.3	95.79	99.1	97.8	99.6	92.8	95.3	95.5
4.77	149.42	93.5	93.3	92.1	106.9	105.5	104.2
6.55	108.07	99.5	97	97.6	100.3	103.8	101.8
7.25	105.42	100.8	103.5	99.4	98.7	96.7	96
5.17	179.15	99.8	99.9	100.8	96.8	97.9	96.8
6.34	134.74	98.7	102.9	99.3	96.1	95.8	96.1
6.55	157.28	98.2	98.5	99.1	107.9	107.2	108.4
5.43	104.95	97.8	98.3	98.7	113.7	112.2	111.4
6.46	115.28	100.5	99.4	98.9	95.2	96.4	94.8
7.81	112.3	99.6	100.5	101.3	99.9	98.7	98.9
7.03	149.63	114	118.2	115.6	78.2	78.5	79.1
4.92	198.62	103.8	96.2	103.3	89	87.8	87.4
5.76	92.38	100.1	97.9	97.7	101.5	101.3	101.1
5.4	104.82	99.9	99.2	97.9	100.1	102.8	100.3
5.64	125.21	99.4	99.3	99.1	105.4	106.1	105
5.59	158.3	95.7	103.7	110	109	105.2	116.3
6.05	93.56	97	96.8	97	95.2	91.9	93.3
6.05	87.93	100	101.8	100.5	98.4	97.4	99.4
5.66	113.01	98.2	98.7	95.3	99.7	96.4	99.1
8.22	121.34	103.7	99.6	101.9	108	109.1	108
7.3	105.82	104.8	97.6	99.1	108.1	114.3	109.3
5.12	108.51	96.9	98.5	99.5	101.1	100	100.6

6.21	95.54	104.5	103.3	101.3	97.3	99.3	99.4
9.16	129.09	105.3	106.4	105.9	105.9	105	103.1
5.64	103.2	97.9	99.8	99	108.6	107.6	107.6
6.42	92.81	100.9	101	100.4	107	105.4	106
4.84	143.45	110	111.5	110.4	89.8	89.4	90.2
6.34	122.06	100.3	101.8	108.4	107.1	104.6	107.7
8.34	110.36	102.7	103.5	103.2	92.3	93.7	94
5.67	110.91	102.9	104.2	102.7	110.2	108.6	112.2
8.95	91.13	100.6	97.9	99.6	102.1	102.2	100.9
4.93	95.93	99.2	101	101.8	92.3	93.1	94.6
8.63	123.45	98.1	97.3	97.3	99.3	100.4	101
8.13	93.95	99	96.8	97.7	100.7	98.3	98.8
5.48	150.2	95.7	109.1	106.4	94.4	95	96.6
8.54	153.77	103	100.4	99.1	103.6	105.6	103.9
8.9	205.45	81.8	73.4	95.4	87	88.3	100.6
6.73	113.98	92.5	90.7	91.8	96.8	96.6	98.5
4.89	132.36	98	96.1	97.1	97.8	96.7	100.5
5.48	282.29	92.9	93.7	93.6	89.3	91	90.9
6.8	108.64	98.9	99.5	98.9	76.8	76.2	76.7
6.14	99.22	96.8	98.5	96.5	99.3	98.2	99
4.94	95.28	107.5	107	105.7	66.9	66.1	67.1
5.43	116.88	97	96.2	98.4	108.3	103.6	105.1
6.19	138.11	105.4	104	104.1	99.4	99.4	97.6
7.85	130.96	97.1	96.1	96.4	91.3	93.1	92.1
5.44	86.93	102.5	102.6	105	107.7	102.7	104.8
6.46	119.51	103.4	102.3	103.7	78.5	81.3	80.5
9.57	123.78	102	107.3	103.2	104.7	102.7	103.2
5.86	116.37	94.8	96.8	94.8	103.7	102.3	103.1
6.87	88.7	101	95.9	95.8	102.3	103.7	101.8
5.62	87.14	116.4	116.1	119.8	68.2	68.6	72.3
6.23	103.19	95.8	97.1	96	93.7	92.6	91.9
5.62	89.55	99.1	101.8	101.8	97.1	97.2	96.9
5.03	105.79	100.9	99.2	99.3	95.7	95.7	100.1
7.68	89.55	95.9	98.6	97.8	97.1	97.9	96.6
6.68	94.27	95.6	95.3	96.5	101.7	98.9	101.2
8.44	96.99	98.8	100.1	97.4	105.2	103.8	101.7
5.24	138.91	103.8	104.8	103	100.4	98.3	96.3
5.92	108.74	92.7	92.7	94.5	102.7	100.5	102.5
10.24	196.91	100.9	102.4	103	103.6	101.6	103
6.68	106.07	98.7	95.9	95.4	110	109.2	107.8
7.37	97.82	99.7	97.6	100.5	96.2	95.6	97.9
4.83	162.9	97.6	97.7	96.1	95.8	98.7	98.9
5	105.01	94.8	96	95.5	99.3	99.1	100.2
5.21	109.85	103	102.8	101.9	96	92.1	94.6
8.94	109.59	100.5	101.5	101.5	90.3	89.8	89.7
7.03	99.96	97	97.2	97.1	105	102.4	102.3
9.73	161.44	102.9	103.8	103	103.9	100.2	101.8
6.04	87.02	97.4	98.6	97.6	102	97.9	98.8
5.25	114.05	96.1	95.8	94.2	106.5	102.5	103.6
7.27	83.77	106.6	100.6	103.2	98.9	104.9	100.6
4.65	79.01	105.7	104.6	105.8	111.2	109.4	109.7

8.34	91.41	98.3	99.4	100.2	103.4	101	103.1
5.97	97.05	70.4	71.5	69.4	89.5	85.2	86.2
6.79	167.92	99.6	102.7	100.7	98.1	97.5	97.3
9.13	71.61	100.2	100.1	100.4	115.4	114.6	114.1
6.46	89.49	94.6	93.1	93.6	106.7	102.7	107.5
5.8	107.91	97.1	97.2	95.8	109	107.7	106.6
6.77	163.02	101.9	104.7	103.1	94.6	97.4	96.8
9.2	94.21	97.8	94.3	97.8	106.3	104.7	103.3
5.5	513.6	101.5	102.7	102.4	96.3	97.2	95.3
6.35	115.11	101.4	101.6	102.8	103.1	101.2	102.1
7.66	100.16	95	96.1	94.3	93.8	91.6	91.5
5.31	89.48	95.7	92.8	94.4	97.8	98.9	94.8
4.98	85.13	95.5	94.4	95.1	101.9	100.8	101.7
9.26	80.41	100.4	99	99	109.7	110.9	110.9
7.44	90.06	104.5	99.6	99.5	97.6	98.5	98
5.26	85.97	103.3	100.9	102.7	106.6	105.5	106.8
5.05	85.35	95.9	90.6	92.1	106.2	105.4	103.7
4.84	200.56	101	103.1	101.6	92.3	92.4	92.8
6.79	129.41	97.7	100	100.2	108	108	104.8
5.97	168.36	101.1	103.4	102.1	102.5	102.1	101.1
6.04	76.56	95.7	95.2	98.4	86	85.1	88.4
5	70.07	100	101.3	100	102.8	99.8	100.7
9.01	98.78	96.6	97	96.9	103.5	105.2	103.5
10.65	149.12	101.1	104.5	101.4	104.7	104.5	103.8
6.86	76.98	96.5	100.1	100.1	97.6	96.2	97.2
4.93	96.03	104.9	106	105.1	102.5	103.9	103.4
5.97	89.51	94.9	93.8	94.8	99.1	99.7	98.5
6.55	75.56	92.5	96.1	95.4	106.6	111	109.5
9.41	101	117.8	84	100.9	114	106	130.2
5.22	76.35	105.3	103.1	101.7	104.6	105.9	108.5
9.01	109.01	103.6	103	105.2	106.3	105	107.4
5.03	97.3	97.7	95.9	98.2	96.2	93.8	92.6
6.43	167.2	99	100.4	98.5	98.5	97.1	97.9
7.53	137.57	98.3	96.5	99.6	96.8	98.3	96.9
4.45	169.48	99.8	104.6	100.3	105.1	102.3	102.5
7.78	89.62	101.4	104.3	101.2	97.4	96.8	96.8
6.7	129.38	97.2	95.1	97.2	107.2	110.1	107.2
10.15	120.45	102.1	101.9	101.7	104.3	101.7	101.1
8.03	142.5	92.1	95	92.1	95.7	95.6	95.5
6.27	73.26	102	97.1	96.7	97.2	94.7	96
8.27	108.96	108.1	109.7	108.6	50.5	48.7	51.2
5.59	625.09	132.8	87.3	115.2	80.4	84.9	70
5.86	76.51	102.2	99.1	98.3	100.4	100	102.5
9.26	85.74	99.2	100.1	98.1	99.4	100.3	102.3
5.35	106.06	102.6	104.1	100.4	110	106.9	106.7
8.38	96.21	102.1	100.2	99.3	97.3	97.1	102.5
6.21	134.71	99.6	102.2	101.4	99.4	99.2	100.1
6.37	76.95	96.4	96.7	98.2	102	99.9	101.4
6	63.46	104.9	102.3	108.3	107.7	109.4	111.3
5.36	89.75	95.6	96.7	94.7	103.4	99.1	100.2
4.87	129.78	100.6	100.9	101.7	103.5	103.6	104.7

7.42	92.66	99.5	96.8	98.6	100.8	101.3	103.8
6.05	128.02	93.2	94.4	93.1	95.4	93.7	95.3
7.59	118.84	97.8	99.1	97.8	102.8	99.5	101.8
7.06	72.92	98.8	97.4	100.4	99.5	98.3	100.3
6.9	91.64	114	113.7	111.8	75.3	75.5	78.2
8.75	84.36	99.3	102.7	103	98.7	97.4	99.6
6.6	142.19	94.9	98.1	96.6	106	103.4	103.8
6.02	91.39	108.4	105.6	110	92.5	92.6	93.2
5.12	82.61	103.2	102.5	101.6	92	93.3	93.7
8.88	76.2	99.2	98.3	96.7	100.1	99.5	100.4
9.7	135.95	108.1	112.7	110.6	111.5	108.5	105.5
5.73	93.6	99.9	100.4	98.9	100.6	99.6	100.2
9.69	141.37	100.9	101.4	99.7	102.3	101.2	100.5
6.84	80.71	99	99	99.4	96.9	98.5	97.9
5.6	203.77	90	100.4	90	78.3	89.8	99.1
8.69	86.75	103.5	102.2	103.6	100.1	98.7	97.3
6.25	77.81	117.5	122.7	122.9	78.6	78.9	77.8
7.65	79.93	100.8	100	98.9	101.8	101.4	104.5
7.99	88.29	98.4	99	99.8	100.3	99.4	100.7
6.3	89.12	102.5	101.6	106	102.8	106	104.9
6.61	106.36	104.1	101.2	103.3	100.8	101.5	101.3
6.77	75.47	99.7	98.9	99.4	105.2	103.7	103
5.49	85.01	97.3	97.5	96.4	100.8	99.4	100.2
8.41	121.5	98.2	97.3	98.1	94.6	94.9	94.3
7.36	140.59	97.1	98.5	97.3	93.7	95.2	94
7.9	80.36	97.6	94.7	96.1	100.3	100.5	99.2
5.87	83.41	91.9	93.1	90.7	90.7	91.4	89.1
5.97	155.44	98.4	100.4	99.7	96	95.4	94.9
6.62	79.74	100.6	98.2	99.3	98.8	97.7	97.7
5.73	85.09	101.3	99	102	105	107.4	105.4
6.21	97.98	101.3	100.4	99.8	111.8	111.2	111.4
5.19	93.19	99.7	102.1	102.6	95.3	98	99.9
5.58	79.06	98.8	97.7	97.4	100.9	99.8	100.2
8.4	106.43	98.6	98.7	99.7	102.7	100.9	103.2
5.83	86.31	103.1	95.4	95.7	98.1	100.4	99.9
6.68	81.44	96.7	95.6	93.7	106.9	106	106.1
5.38	88.48	101	99.8	101.8	111	108.2	108.2
5.74	85.27	96.4	99	97.5	97.2	99.5	103
8.38	67.62	97.7	97.7	97.8	102.5	101.8	106.4
6.37	67.22	95.5	99.3	100.1	96.9	95.8	97.3
6.81	71.66	116.9	112.8	113.6	95.2	96.5	98.5
6.05	87.33	98.6	96.8	97.6	103.9	105.6	105.7
6.8	67.83	99.1	99.5	99.7	112.3	108.9	115.9
9.94	120.43	100.7	102.5	102.3	104	103	102.1
7.9	108.97	104.1	104.1	102.4	95.4	96	94.8
6.74	144.26	101.3	103.3	99.9	94.1	92	93.7
10.84	103.44	102.1	101.1	101.5	102.4	102.8	103.4
6.39	79.66	101	99.7	100	107.7	107.1	109.4
11.03	172.28	112.3	114.2	113.9	100.8	100	99.3
5.25	84.61	99.4	99	100.8	107.3	107.4	107.6
5.69	86.52	100.5	97.4	101.1	102	104.6	105.9

7.05	112.57	93.5	90.3	93.6	103.4	101.6	102.5
10.93	172.8	108.3	112.6	105.5	137.3	133.2	126.3
5.54	96.69	99.9	100.4	100	100.4	100.7	100.9
7.05	75.89	101.6	104.1	106.8	95.9	93.2	95.5
6.68	104.94	103.4	100.9	99.6	100.6	99.8	99.4
7.36	97.83	98.9	99.7	97.9	96.5	95.2	94.8
6.14	70.6	103.5	100.9	101.8	96.4	95.3	94.5
4.53	67.59	95.5	95	95.6	106.9	107.5	107.3
7.5	115.35	98.3	100.5	103.8	97.3	104.1	99
10.32	165.35	104.8	106.1	101.6	102.4	99.7	99.6
5.95	84.56	100.3	98.2	100.7	103.9	99.6	101.6
6.92	80.19	99.9	100.2	99.8	99.8	100.1	99.8
4.67	154.35	100.7	100.9	98.6	103.7	103.4	100.8
6.24	75.83	97.4	96.2	99.4	96.2	94.1	95.2
5.25	62.81	100.4	96.9	99.6	104.2	101.3	107.2
8.65	71.06	102.9	101.7	102.1	103.8	102.2	103.1
9.29	82.37	105.3	101.5	103.1	103.5	103	104.7
6.51	61	101.9	96.2	114.3	94.6	102.1	102.7
7.74	100.58	120.2	120.9	116	84.6	79.3	81.6
7.49	135.05	99.4	100.6	97.9	92.6	93.7	92.7
6.87	167.37	101.4	102.5	101.9	91.4	92.2	92.9
6.71	62.19	100.6	100.1	98.2	103.4	102.3	102.5
8.76	76.96	98.6	105.1	104.2	83.9	82.9	84.7
6.29	92.39	102.8	102.4	99.2	94.2	94.7	95.6
9.54	90.86	96.8	94.4	96.7	101.9	100.5	99.9
4.68	58.06	98.9	100.9	101.7	94.1	94.1	94.7
10.08	139.23	104.4	103.7	104.2	109.4	108.2	109.5
6.95	74.15	99.8	101.3	99.8	97.7	98.5	97
6.13	82.78	99.6	100.1	102.6	103.7	108.2	105.6
5.39	70.46	100.7	98.5	98.3	96	98.7	100.7
5.27	92.23	95.9	100	96	104.9	108.3	103.9
5.03	81.66	87	84.4	102	107.4	104.8	152.1
6.87	68.43	104.3	97.3	101.3	108.1	109.9	107.1
5.83	76.04	102.6	100.7	101.7	97.1	98.6	96.3
7.37	74.95	97.6	95.4	99.9	110.9	111.7	110.9
5.14	102.34	99.2	97.1	98.9	100.8	104.2	101.7
6.04	69.3	114.2	111.7	113.2	85.8	83.3	84.1
8.38	77.46	100.9	100.3	99.2	106.9	104.1	106.3
8.35	77.59	98	97	96.7	104.2	104.3	102.7
5.69	66.76	104.9	103.3	101.4	108.1	108.9	111.2
4.84	136.77	101.9	102.7	102.6	101.6	99.7	100.9
6.76	85.06	102	100.7	102.5	97	98.7	97.4
8.92	62.22	101.8	100	102	109.1	108.1	106.4
6.29	138.92	102.6	104.3	102.8	96.8	97.8	97
7.31	73	98.3	99.5	97.5	104.8	101.5	103.9
4.3	64.46	105.5	101.5	103.5	119.4	119.9	121
7.97	106.56	104.7	104.5	103.9	93.1	90.5	91.6
8.78	71.43	99.8	99.4	101.4	106.1	106.8	107.5
7.09	61.12	99.3	103.5	100	106.9	107.4	109.2
9.17	96.1	99.9	97.6	96.8	104.5	103.3	102.9
7.37	77.96	99.7	101.8	99.9	97.5	97.9	97.5

8.13	64.28	96.3	96	95.6	99.6	96.4	97.4
8.92	62.46	99.9	99.7	101.9	97.2	94.2	95.7
5.73	113.6	97.5	98.5	97.9	103.1	104.5	100.7
8.03	67.31	101.2	100.4	100.9	94.7	98.8	96.7
5.96	70.95	96.7	95.2	93.7	103.7	101.3	105.5
8.12	62.82	103.1	99.7	100	113.5	111.9	109.5
6.19	124.3	101.8	106.1	98.2	102.6	103.6	94.4
9.74	123.72	99.5	96.8	99.4	103.3	100.6	104.7
8.78	73.08	105.8	105.9	101.2	96.5	96.1	98.5
11.36	290.23	111	112.3	113.1	106.8	110	107.4
8.46	79.06	101.6	97.4	99.1	101.9	101	100.8
5.38	66.09	101.1	99.7	100.4	96.5	95.2	100.2
8.5	77.54	100.4	101.1	100.7	101.1	102.4	101.9
9.64	318.71	107.4	105.4	106.1	94.5	95.8	95.9
8.05	100.19	106.7	103.9	104.2	94.3	94.7	94.7
6.29	98.65	74.3	78.9	72.2	118.8	117.5	117.2
6.04	77.48	98.1	99.4	96.2	107.5	108.5	107.5
5.22	83.08	102	103.6	104	111	111.3	109.3
4.36	312.51	98.1	101.5	97.2	98.6	99	98.2
6.02	63.47	100.7	99.9	102.6	98.5	104	101.1
7.77	82.21	96.5	94.2	94.7	97.5	95.4	97.3
5.97	63.54	106	102	104.4	98.4	97.4	96
6.21	122.24	98.5	98.3	98.7	100	99.1	101.6
10.32	102.77	100.9	100.7	101.1	105.4	105.4	103.2
7.88	114.79	97.2	95.6	90.8	98.3	95.2	94.4
9.04	66.21	95.3	97	95.2	111	110.5	109
5.67	59.97	100.7	98	101.1	93.3	92.1	93.8
7.49	57.98	102.6	102.4	103.8	103.2	101.7	102.3
6.32	84.36	110	107.8	105.3	96.9	94.8	95.9
9.11	74.69	93.5	93.5	96.7	91.9	91.6	95.6
8.25	81.9	100.1	99.4	100	108.4	109.4	105.4
10.43	125.76	97.4	109.2	103.9	107.3	110.2	109.5
6	95.14	102.1	102.3	100.7	100.5	100.9	102.4
7.88	81.6	105.6	103.6	106.1	102.5	102.5	99.6
6.25	78.06	99.9	101.5	99.2	94.8	94.3	93.4
4.54	119.13	102.2	102.6	100.5	102.2	101.8	102.4
7.93	72.42	100.6	109.4	110.2	95.5	94.7	94.6
6.02	86.01	95.4	92.6	90.7	92.5	93.9	95.2
4.79	103.98	99.3	100.7	99.7	100.6	98	96.6
5.21	77.9	100.9	100.7	102.8	97.8	98.3	101.3
6.73	74.75	105.3	107.5	111	114.4	113	113.9
5.66	64.12	105.3	101.8	105.4	96.2	96.1	98.6
11.02	168.58	98	105.6	106.8	136	140.9	148.2
6.16	67.28	100.8	100.5	100.1	94.5	91.5	95.5
6.67	125.2	97.6	97.2	97.8	109.1	107.3	105.6
6.43	68.57	102.2	98.9	104.3	92.3	94.2	92.9
9.76	106.53	95.2	94.6	92.9	85.5	85.4	84.3
6.18	64.17	95.3	98.2	99.7	91.3	89.4	91.3
8.5	109.94	99.6	97.9	97.8	97.4	97	96.9
5.33	86.89	102.6	104.7	102.6	98.2	95.4	94.6
8.41	90.29	97.2	97.7	98.9	99	102.2	102.1

10.58	139.25	97.4	99.7	100.1	109.7	111	111
5.92	113.98	100.7	100.7	98.9	104.3	103.5	104.5
4.84	79.73	106	101.5	102.4	99.9	103.9	102
7.17	74.89	97.5	98.7	95.8	97.6	96.7	97.9
5.41	82.28	100.5	94.5	91.3	116.7	111	114.9
7.56	126.78	99.3	98	97.7	103.4	103.4	102.1
7.21	114.97	100.5	104.6	103	104	101.6	101.8
5.26	78.74	99	100	97.1	100.2	96.1	97.6
5.68	76.74	101.2	99.4	98.7	93.7	92.6	94.2
6.96	71.22	98.7	98.7	100.2	97.9	100.2	106
9.42	137.79	102.7	100.1	102.9	104.7	101.4	105.2
6.13	71.14	97.3	98.4	99.3	93.2	92.1	92.3
7.2	71.25	97.2	96.4	94.6	100.5	99.4	98.6
5.67	74.54	105.7	104	104.8	125	120.9	122.6
9.28	64.19	95.1	97.8	96.4	99.7	101	99.8
8.85	71.62	98.8	98.6	99.1	102.4	99.8	100.3
7.93	78.42	98.5	97.4	95.1	90.2	93	92.6
5.55	53.31	97.8	95	95.8	99.5	98.7	99.7
8	127.73	115.6	118.1	126.5	88.6	80.4	86.9
6.89	61.75	99.8	102.3	97.1	95.9	96.3	97.5
6.73	61.69	101	102.4	102.2	95.9	97.2	94.8
5.96	64.84	107.3	101.1	101.1	112.9	110.6	113.7
7.09	59.01	99.9	96.8	99	100.7	98.8	101.4
5.9	65.43	98.1	101.5	103.2	98.2	102.3	100.5
7.06	69.13	95	96.6	95.3	103	104	103.7
10.05	92.38	104	104.6	101.2	102	102	101
6.05	56.14	106.7	106.7	111	92.7	93.7	95.6
5.2	61.89	102	101.7	100.5	105.4	104.1	105.3
5.49	70.33	96.1	96.4	97.5	100.4	101.7	100.6
5.22	69.7	92.4	95.4	95.6	102.2	100.5	104.5
5.2	81.59	99.6	97.6	100.1	103.4	100.6	104.2
5.43	75.02	97.8	99	97.8	99.1	101.4	102.2
6.87	67.5	102.5	98.8	101.2	100.3	101.9	99.2
7.23	102.09	96.9	97.2	96.8	92.8	92.4	92.4
8.53	73.51	102.9	101.3	103.3	104.5	103.8	101.7
5.81	89.04	100.5	99.2	100.6	99.8	99.8	101.1
6.7	53.43	101.2	99.8	107.6	99.2	101.6	102.3
6.61	59.82	101.3	97.7	96.4	101.5	101.8	103
6.65	72.26	97.6	98.6	99.3	97.6	99.4	101
5.85	77.06	93.2	93.6	93.5	101.2	101.1	99.9
6.33	99.22	102.4	99.7	102.9	97.5	97.8	97.9
8.25	79.89	95.6	100	99	96	95.9	97.1
5.47	63.04	98.6	98.8	97.1	96.9	95.8	94.9
8.7	85.45	102	101	99	90.1	91	91.7
5.14	103.07	96.8	94.9	95.5	107.1	103.2	106.9
5.16	71.38	97	95.7	94.1	101.9	104.7	103.7
6.38	75.42	99.2	97	96.5	98.8	100.2	100.4
5.54	62.73	98.4	98.5	99.3	97.5	97.5	103.1
5.57	89.59	97.2	97	95.7	101.1	104.5	103.8
5.47	71.83	103.9	103.2	105.6	115.1	109.8	112.2
5.24	53.39	99.2	103.9	102.6	102.2	101.3	101.2

7.46	88.04	104.8	101.4	101.8	107.9	109.5	109
6.19	93.85	98.2	102.2	96.2	95.9	94.8	95.3
8.44	81	99.3	98.5	97.9	101.2	101.5	99.8
5.47	64.39	98.7	101.4	98.5	77.3	79.2	78.5
11.25	110.03	100.1	101.2	99.6	107.4	106.1	103.8
7.14	97.83	94.4	96.6	95.2	101.9	102	100.3
6.18	80.6	100.1	100.2	98.4	100.8	101.5	101.8
5.62	58.29	100.6	96.3	98	98.5	102.3	102.6
5.76	99.85	103.9	102.4	100	108.7	107.8	106.9
5.2	74.09	96.2	99.7	95.2	105	103.9	101.7
5.06	53.13	95.9	99.9	100.2	99.1	102.2	100.5
5.36	114.11	96.9	99.9	99.8	100	98	100
5.05	71.84	129.9	129.8	126.1	73.8	69	69.8
5.11	112.49	99.2	103	100	101.2	101.3	101.1
7.01	96.62	95.9	94.2	100	112.3	112.8	110.8
6.04	69.07	102.9	103.2	104.3	84.3	83.9	86.2
9.44	79.55	100.2	102.6	99	107	107.6	106.3
6.74	43.28	102.3	98.4	94.7	95.7	97.6	98.6
8.21	70.19	106	105.3	102.3	102.5	103	103.1
4.94	67.37	103.2	106.6	106.2	107	104.4	102.8
8.38	57.79	98.9	97.2	95.2	106.5	106.1	108.3
5.07	63.94	100.4	103.2	100.8	100.9	101	97.9
10.32	217.53	111.2	111	113.2	106	111.3	105.9
10.45	111.77	101.6	103.7	102.1	106.3	104.1	103.3
5.62	61.08	99.7	99.2	95.9	98.9	96.1	95.9
6.95	77.17	106.7	106.9	105.7	109	108.5	107.9
7.47	64.62	102.6	100.9	103	104.7	106.1	106
5.16	76.86	128.1	132.1	128.7	78.7	77.3	81
7.53	64.55	98.7	97.1	97.8	97.9	98	97.6
5.48	87.31	98.5	98.2	101.1	101.8	97.3	104.3
8.87	71.78	103.8	94.7	100.9	112.4	114.4	114.4
8.69	95.8	111.4	110.1	111.4	95.3	96.9	95.5
7.61	89.12	102.8	104.8	101.7	111.9	109.9	110.2
5.52	68.33	94.7	97.4	96.6	94.4	92.6	95.4
10.92	146.65	113.2	113.7	113	99.8	99.4	98.2
10.08	98.44	99.7	102.3	99.1	103.6	105.3	105.1
5.25	74.35	100.3	99.9	99.8	107.8	106.7	107
7.3	61.74	98.6	98.9	99.6	96	95.1	95.1
6.29	61.09	103.4	98.6	96.7	101.8	104	100
8.46	80.22	101.1	103	103.6	99.5	99.1	99.4
5.08	99.56	86.5	86.7	86.6	104.6	106.2	104
6.54	89.77	99.3	96	95	88.9	90.6	90.2
9.38	93.35	94.7	93.9	95.8	102.3	100.3	104.4
6.73	82.87	102.7	101.2	106.2	113	103.2	113.7
6.1	93.54	102.9	103.4	102.6	98.1	98.4	96.8
8.9	68.64	103	97.2	98.9	98.1	99.1	98.6
5.17	66.75	99	101.4	103.5	95.4	94.8	92.2
5.83	69.95	105.2	104.6	104.5	106.7	104.1	100.3
5.48	131.84	85	84.3	82.5	131.4	128.9	127
5.1	64.21	101.9	98.1	97.5	104.3	103.3	103.4
10.13	72.4	97	97.7	97.5	114	114.9	113.8

6.37	57.54	99.2	97.6	97.6	96.7	93.1	93.9
5.08	161.93	103.1	101.7	102.1	90.2	89.5	90
7.31	111.23	101.8	102.4	101.9	99.2	99.5	97.2
7.21	66.51	99.6	99.4	98.4	96.8	96.1	95.8
6.05	82.08	102.5	98.4	100.4	100.3	100.2	101.8
4.4	56.07	98.8	97.3	96.9	110.6	109.3	109.7
5.54	74.28	108.7	96.6	103.3	103.7	103.9	100.6
6	80.75	105.4	94.9	104	98.9	103.6	102.8
5.27	212.53	74.3	66	68.7	91.1	85.5	95
6.54	85.96	106.7	96.9	100.3	107.9	96.1	95.2
6.68	54.66	102.2	102.3	99.2	107.2	106.2	102.6
8.43	66.35	99.7	100.3	99.4	101	100.4	100
5.15	56.39	105.4	102.9	99.8	95.8	97.6	93.9
6.89	49.15	104.1	101.2	102	103.1	105.4	108.6
6.37	73.77	102.6	101.4	102.4	106.3	105.2	106.2
5.67	69.32	99.1	99.7	98.7	96.6	98.7	97.7
4.78	58.61	100.7	97.7	105.1	87.3	93	88.2
5.66	54.35	106.5	103.8	102.5	111.3	110.9	109.6
5.1	66.3	102.9	98.5	97.8	105.9	103.7	105.6
7.37	65.36	94	92.4	95.3	103.5	106.6	106.6
8	55.5	105.9	101.8	103	105.5	104.3	105.1
5.69	48.41	97.2	96.5	97.8	95.5	95.8	97.1
6.2	60.59	104.6	102.6	102.6	100.6	101.6	98.9
5.54	61.67	103.7	99.7	101.4	95.3	98.2	96.7
9	92.58	100.3	104.3	102.3	108.6	104.9	107.1
7.37	67.19	107.8	106.4	107.2	96.1	98	97.5
4.67	51.87	97.8	97.2	99.6	111.4	107.3	113
5.33	62.33	105.5	104.2	107.8	115.5	113.7	112.2
7.77	56.46	98.1	99.5	99.7	106.6	104.9	106.5
6.65	63.72	98.8	98.5	100.8	98.9	99.5	99
10.32	161.83	108.3	113.7	110.2	107.2	106.7	103.3
5	73.5	97.1	96.7	99.3	101.3	99.6	100.3
4.79	73.68	100.8	102	100.4	101.3	100.8	100.9
7.12	77.37	98.7	98.8	97.6	102.6	101.2	102.5
6.16	73.03	100.3	100.8	99.3	98.9	100.2	101.2
10.15	57.65	101.5	103.9	104.5	120.8	122.7	122.6
5.62	61.42	97.9	98	96.7	105.2	105.3	109.6
6.57	67.36	107.4	102.4	102	92.3	96.9	106.6
11.03	96.25	101.4	101.7	101.1	107.9	101.9	102.6
5.35	67.3	97.1	93.6	96.9	101.1	101.5	102.5
6.86	81.22	96	97.1	95.8	102.9	110.5	105
8.5	64.9	102.4	102.8	103	96.3	97	98.5
6.92	50.23	102.8	97.8	100.8	98.3	100.5	98.2
6.9	60.19	103.7	96.6	97.6	97.4	95.6	97.6
6.23	52.25	93.6	96.4	94.1	97.2	96.2	94.5
4.68	53.23	99	99.1	105	103	103.1	105.9
8.54	60.52	102.2	99.6	99.5	100.8	100.9	101.3
5.54	57.59	94.9	91.5	93.8	104.2	101.1	105.8
7.87	43.84	100.1	101.2	97.2	102.3	98.3	99.5
4.84	55.43	99.1	100.7	101.3	99.8	105.5	103.9
10.15	99.5	101	101.9	102.1	105.4	103.3	105.8

9.07	71.29	103.2	100.9	102.2	67.6	66.1	67.4
6.4	54.15	102.8	103.1	100.7	104.9	105.6	104.4
10.05	89.43	104.1	102.5	105.5	118.2	116.8	117.5
9.83	84.37	101.8	102.2	103.3	106	105.6	103.3
5.29	75.94	99.1	98.5	103.1	106.8	106.9	104.2
7.21	55.32	94.9	97.7	95.9	95.3	97.2	98
5.78	69.74	101.1	101.1	102.3	95.2	94.3	97.2
7.61	63.5	99.3	99.5	98.3	107.6	107.1	105.6
6.15	66.8	114.2	110.7	114.9	72.8	76.2	77.3
10.51	123.15	100.3	99.2	98.6	106.2	107.3	106.6
6.84	77.56	102.4	102.6	99.8	98.1	97.2	97.7
5.25	57.14	93.3	93.9	91.6	103.2	104.6	103.7
9.09	67.16	100.1	98.3	98.6	108	106.6	108.5
9.57	76.79	102.5	105.3	106.2	106.4	109.6	106.6
6.18	70.39	97.6	96	94.4	103.8	102	102.3
8.1	51.56	100.1	99.7	99.6	97.2	96.2	96.2
6.52	57.24	99.3	98	99.2	105.4	103.9	106
9.19	62.51	101.4	103.2	102	103.4	105.5	107.4
5.01	131.34	101.6	101.3	99.8	104	102.2	100.7
9.38	83.35	101.3	102.2	103.9	104.1	105.7	106.6
6.95	71.84	100	98.1	100.8	102.6	101.5	102.1
5.49	121.03	97.3	100.8	98.3	97.1	97.5	95.9
5.25	63.33	101.5	105.4	102.3	89.4	94.8	92
5.6	115.12	95.8	96.8	95.5	96.4	94.5	96
5.48	53.46	101.7	98.5	96	100.8	99.3	98
6.05	75.38	96.7	97.4	99.6	95.2	92.2	97.8
9.03	74.26	98	99.9	99.7	99.1	98.2	95.8
4.73	55.53	104.5	102.2	102.2	108	111.6	109.4
5.17	51.11	98.8	100.6	98	100.8	98	100.5
5.52	65.41	99	98.4	100.5	108.4	110.2	112.8
7.62	50.42	95.7	92.2	92.5	92.6	90.2	93.2
8.32	84.5	98.6	101.2	99.5	96.1	93.3	95.5
6.7	70.23	64.6	62.9	61.2	81.9	82.2	82.3
5.63	65.82	99	94.4	94.9	110.3	111.1	108.2
6.79	63.01	102.3	99.8	99.7	101.1	101	99.9
8.07	67.59	105	97.9	99.8	98.9	104.8	100.2
5.08	67.19	100.4	97.9	97.5	102.4	103.8	105.4
9.36	90.32	101.5	101	102.7	108.7	111.2	109.3
10.3	97.03	99.7	104	99.2	103.2	104.5	102.2
5.39	62.57	103.1	100.8	98.7	100.9	101.8	98.3
8.95	72.78	102.4	102.8	103.9	104.3	104.3	100.1
6.4	45.82	99.8	100.5	100.4	110.5	105.9	107.5
8.57	95.54	99	102	109.5	84.6	93.7	95.6
5.31	60.89	97.6	97.1	98.1	102.4	99.1	100.1
4.69	69.57	105.5	105.8	106.2	105.3	105.7	105.9
7.18	53.73	108.6	104.4	110.7	102.6	102.9	106.3
8.21	47.33	103.7	102.8	99.9	100.2	100.3	100.5
7.34	58.72	105.9	102.5	106.2	103.7	102.1	104
8.07	72.46	94.6	99.3	94.7	98.1	97.4	96.7
5.55	43.9	114.3	107	110.8	90.7	91.3	90.3
6.15	53.59	108.3	99.3	107.8	91.3	90.6	82.8

6.06	52.44	104	97.6	98.3	99	97	99.9
7.5	49.61	108	108.3	106.2	94.4	92	97.5
8.44	58.69	102.5	97.1	99.2	83.4	84.9	88.8
5.48	55.17	103.6	105.8	104	100.1	97.3	96.5
8.06	86.38	93.9	103.8	99.9	99	110.2	99.1
6.38	62.29	93.1	94	93	106.6	108.5	106.2
5.66	61.02	96.2	94.4	96	108.9	108.5	108.2
7.8	55.45	95.8	96	92.5	114.4	109.6	112.7
7.91	48.36	103.4	102	101.5	91.8	89.7	93
8.15	40.8	95.3	94.5	93.2	107.1	105.3	108.9
9.96	83.37	101.4	101.8	102	99.5	97.2	99
9.79	58.82	103.6	103.4	110	99.9	106.9	106.7
5.63	63.68	93.5	95.3	92.3	88.4	83.8	87.2
5.43	66	98	100	102.4	102.9	101.2	100.3
6.74	65.02	103.4	103.8	100.9	91.8	88.7	88.7
7.72	66.6	100.8	102.9	99.6	97.8	97.5	95.1
6.04	52.3	102.5	105.3	99.9	140.2	123.4	135.4
9.32	57.55	99	98	99.5	120.5	122	119.7
6.39	51.53	98.8	96.6	97.4	101.8	104.3	105.8
6.61	50.46	105.1	106.8	107.4	93.3	95	96.6
6.54	68.88	101	100.5	97.6	103	101.3	102.7
8.72	51.14	99.7	95.5	93.7	94.7	91.4	96.8
5.77	56.52	104.1	106	108	110.5	116.3	114.2
4.81	48.26	101.5	97.2	101.4	104.8	105.5	104.3
5.87	63.45	121.7	121.9	121.5	85	84.7	85.4
4.96	52.61	96.6	92.9	94	107.1	102.2	106.4
6.32	50.46	106.7	107.1	107.1	98.3	94.1	95.6
8.48	58.44	101.6	96.8	97.4	102.7	103.9	102
8.87	50.21	100.9	100.1	103.6	98.9	103	102.4
7.18	41.49	104.2	105.5	104.5	95.1	92.7	92.8
5.39	44.58	111.5	96.3	98.6	91	96.6	95.1
10.54	105.87	104.5	103.6	103.4	101.7	102.9	102.1
6.05	56.15	98.3	100.2	99.1	104.2	103.8	104.1
6.55	64.2	105.6	103.7	105.6	86.9	89.5	90.6
8.51	84.46	95.3	102.1	99.1	99.7	98.7	99.8
8.28	38.92	97.1	101	101.5	100.8	101.6	97.6
7.58	49.12	92.2	90.9	90.7	103.7	106.1	109.4
5.38	52.46	96.5	96	98.7	99.3	105.7	102.9
6.73	56.01	108	104.8	105.4	95.7	96.5	102.3
5.97	75.74	102.3	105.6	104.6	111.6	116	112.7
5.43	54.15	123.2	98.1	107.7	91.4	90.4	92.8
8.95	53.19	105.2	101.8	100.7	104.3	100.7	104.5
5.68	55	97.4	101.3	102.3	106.5	107.6	107
9.23	43.36	102.3	101.5	109.5	109.2	104.9	110
6.68	51.91	101.4	101	98.8	108.9	109.7	107.9
8.32	89.96	103.4	102.2	100.9	102.8	100.3	101.5
6.19	49.91	108.1	101.1	102.1	106.5	104	103.7
6.7	61.84	97.5	96.3	99.1	109.4	104.1	105.4
4.83	48.02	95.5	98.3	96.2	102.9	105.2	103.1
8.75	47.63	102	102	99.1	102.4	100.6	99.2
7.03	53.21	100.7	99.1	99.9	95.1	92.7	93.9

6.34	53.14	131.2	123.5	126.5	92.2	95.5	92.5
8	63.14	103	95.8	97.9	88.6	89	90.2
5.12	50.06	95.6	97.5	100	94.5	95.5	98.3
5.52	42.41	106.5	103.6	104.3	111.8	114.5	114.4
4.75	62.93	82.4	81.2	82.4	106.6	110.7	105.5
7.94	59.11	99.6	102.5	100.4	108.4	106.7	105.8
6.48	70.09	106.9	103.2	102.1	102	100.6	99.6
10.18	60.06	101.5	103.9	104.6	109.4	110.2	110.4
9.28	41.73	106	101.2	105.6	95.3	95.5	98
7.78	65.88	96.7	99.3	98.3	103.5	100.8	107.4
8.56	67.64	86.4	85.6	84.7	72.6	72.9	71.6
7.01	93.74	99.4	98.9	100.3	104.4	104.9	104.9
5.29	59.43	104.3	106.2	104.4	108.9	108.8	108.4
5.92	60.44	100.4	100.1	98.3	97.3	96.1	97.8
4.98	57.24	96.4	94.2	95.8	101	103.9	105
7.77	58.07	113.2	113.8	109.2	91.9	92.2	93.9
6.92	100.61	100.5	100.4	101.6	92	92.9	91.9
8.65	40.21	106.1	103.6	108	94.8	93.1	94.5
10.62	86.3	96.6	101.8	100.3	118.7	117.3	116.8
5.3	64.02	94.7	97.6	96.6	100	98.5	99.2
8.57	46.55	111.5	103.9	106.3	96.9	98.4	97.6
8.13	67.42	99.9	98.8	100.6	104.7	103.9	103.5
6.74	46.38	89.8	88.2	90.4	105.8	104.6	101.4
11.47	81.83	98.7	101.5	97.7	105.6	104.4	103.1
7.96	58.02	97.7	94.3	97.1	106.9	108.1	105.6
6.8	44.76	103	94.8	101.1	103.6	101.9	102.5
5.3	54.87	103.4	100.9	103.5	108.5	109.3	105.9
6.3	51.57	95.1	97.9	100.5	93.8	96.6	98.7
6.92	50.05	100	100.4	97.4	98.5	98.7	97.2
8.59	50.08	97.8	100.3	97.7	116.1	113.3	113.9
6.67	45.86	102	99.8	103.9	108.1	103.8	105.9
6.47	42.74	124.9	125.8	125.1	62	60.7	59.7
7.18	54.43	95.8	103.2	102	104.2	100.8	106.1
6.47	48.25	151.3	148.5	142.5	92.7	114.5	121.7
6.57	59.55	102.1	102.9	99.7	100.2	100.2	98.7
8.13	67.54	100.1	98.1	100.4	102	98.8	100.4
8.06	66.4	97.5	96.7	97.1	102	105.2	104.2
5.64	70.56	99.6	98	98.3	106.6	102.9	106.9
5.55	41.48	102.3	101.4	99.9	103.1	101	101.5
11.65	116.54	98.9	99.8	97.3	107.2	105.8	105.9
7.11	45.27	101.4	102.1	103.1	103.9	98.9	103.8
8.37	43.11	108.2	99.9	103.6	115.6	108.6	118.6
8.09	57.1	102	102.7	101.3	100.3	100.7	102.4
8.66	53.97	102.4	101.5	103.9	107	105.7	106.8
6.67	51.52	105.2	107.8	103.4	89.6	92.9	95
5.44	53.7	98.8	98.6	96.3	103.6	101	100.5
8.32	75.44	98.1	100.7	100.2	96.4	97.2	100.1
5.36	42.1	96.1	98.6	99.6	95	96.2	102.1
11.62	64.6	98.9	96.7	96.6	105.8	109.7	106.5
6.33	55.57	101.8	103.2	103	100.9	100.3	96.7
6.64	40.08	95.1	96.4	95.7	102.8	103.4	102.6

8.28	49.73	99.1	96.9	99.2	103.6	101.4	99.1
6.3	52.49	102.8	94.2	100.7	99.8	97	100.9
7.06	42.83	100.7	98.5	96.9	102.9	100	105.3
8.32	49.47	101.6	100.6	101.4	95.9	101.2	98.7
5.58	64.86	113	103.8	102.1	104.7	111.9	103.1
5.67	91.53	88.6	91.5	87.8	97.3	99.9	96.9
5.41	47.12	106.1	101.9	109	111.6	111.1	114.7
5.81	50.22	99.5	96.3	96.4	96.2	95	100.7
5.64	50.24	98.7	100.1	100.9	114.6	116.8	116.1
7.74	40.3	95.8	98.7	96.8	93.3	97.6	97.3
7.43	82.83	100.8	99.1	99.5	99.8	97.7	99.2
6.77	42.66	107.3	97.6	101.9	104.1	107.2	105
6.16	73.21	103.8	102.2	105.3	96.3	93.9	96
9.28	62.38	97.3	100.5	98.9	104.6	101	101.9
8.87	51.39	97.4	102	100.4	108.9	106.3	110
6.52	40.96	96.3	98.5	103.7	106.2	105.2	107.6
7.42	49.11	96.2	95.2	99.5	95.2	91.1	95.5
6.37	63.84	106	101.1	101.7	87.6	80.5	84.6
9.03	44.65	107	106.1	104.4	110.7	112.3	112
8.09	46.84	102.6	99.6	101	109.5	108.1	111
9.98	51.39	98.7	98.9	99.7	111.8	112.7	112.3
8.35	76.31	97.6	97	97.9	95.4	93.6	94.7
8.48	76.95	88.3	89	86.7	98.2	97.9	98
8.65	73.17	105.9	102.2	98.7	91.9	91.6	91.8
6.39	50.52	124.2	115.1	123.2	82.6	84.1	83.4
7.2	45	93.9	95.3	99.3	106.4	110	108.4
8.87	62.67	98.6	101.6	100.7	112.6	111.3	113.8
9.67	43.1	104.9	103.8	101.7	97.9	99.9	98.4
5.77	56.7	107.9	108.3	108.5	96.2	93.9	98.5
6.76	68.23	97.6	98.1	99.1	101.1	101.2	97.1
6.42	51.66	94.7	94.6	96	105.3	104.6	104.3
7.15	54.14	103.8	94.2	101.5	97.5	96.5	100.7
6.64	82.01	99.9	101.1	101.4	98.6	100.4	97.2
7.39	55.27	102.6	103.2	102	102.5	104	107.9
8.25	45.53	102.4	100.8	102.7	98.4	99.3	101.4
6.57	39.74	93.6	92.2	94.1	89.1	92.2	89.8
6.68	51.32	104.7	104.8	103.2	105.9	111.3	105.1
7.01	50.44	97.5	97.9	99	101.9	109.1	106
5.11	57.56	102.8	103.2	99.9	101.6	99.8	104.2
5.17	46.21	99.9	102.2	102.3	97.8	98	98.3
6.32	42.65	97.9	100.2	91.9	113	109.7	113.7
8.7	53.48	101.2	101.7	101	104.5	101.3	101.7
5.15	47.11	99.5	98	101.9	105.2	97.6	105.2
7.62	48.29	101.9	101.5	101.2	103.1	99	100
8.73	48.17	96.9	93.8	93.6	96	90.5	98.3
7.64	48.74	107.8	105.3	108.9	103.3	103.9	109.3
6.96	46.35	100	98.8	99.3	110	105.5	109.2
8.59	74.67	94.2	88.9	94.4	51.2	53.9	51.3
6.61	48.81	105.2	103.7	97.9	97.3	90.9	94.2
6.8	55.04	101.7	99.7	97.4	99.2	102.1	98.3
6.38	53.66	94.9	96.3	96.5	100.9	99.6	99.2

6.39	70.62	99.6	100.1	100	109.2	108.9	109.2
8.12	48.19	93.9	100.1	100.5	95.9	99.7	96.3
4.98	43.08	95.4	94.2	98	100.4	102.7	106.1
4.49	62.86	97.5	100.5	103.9	99.9	100.5	102.4
7.34	42.58	97	97.1	97.7	104.2	100.7	103
4.69	70.62	105.5	99.6	98.9	101.4	99.3	100.5
9.66	45.14	110	104.7	108	105	106.3	108.1
8.7	35.6	99.6	99	99.8	108.3	108.6	107.3
7.12	46.16	99	102.8	99.5	95.4	96.2	94.4
6.62	48	100.2	100.8	102.2	114.2	108.7	111.5
6.01	56.47	97.4	97.1	100.3	94.9	97	99
5.66	41.36	105.1	101.5	99.2	99.5	96.9	101.3
10.9	58	103.5	99.8	98.9	111.2	108.6	113.9
6.68	70.44	102.7	94.6	96.6	106.7	103.9	104.6
8.98	57.17	108.3	98.8	95.7	107.4	110.3	108.1
9.64	46.95	91	90	93.9	117	117.9	117.6
8.82	52.45	95.8	96.6	96.2	107.2	108	106.9
6.74	63.13	114.8	96.4	104	99.5	110.5	111.1
8.5	44.58	97.7	95.3	96	106	100.8	102.1
12.06	43.54	108.5	100.7	103.9	117.8	117.7	118.2
6.52	59.02	98.1	104.3	100.8	100.3	97.7	95.5
5.53	53.01	77.3	78.6	77.9	110.7	115.6	113.1
8.44	44.14	93.4	90.3	91.2	100.7	102.3	103.2
5.33	58.9	96	92.3	94.4	93.2	92.4	94.2
7.84	57.85	102.7	96.4	102	100.7	99.7	104.1
6.16	46.74	103.5	99.9	101.7	99	105.4	116.5
5.03	42.2	102.2	101.5	100.7	92	89.9	96.2
5.19	46.43	100.3	100.6	100.9	101.7	100.6	103
5.85	71.06	98.1	99.8	99	96.6	92.6	97.9
8	43.88	102.5	99.4	100.6	98.5	97.1	96.9
7.66	56.42	97	97	101.5	103.2	102.1	101.3
5.38	47.57	102.3	104.7	100.8	106.9	104	105.8
5.86	44.43	105.2	99.3	100.2	100.9	97.9	102.1
5.3	62.57	100.4	99.7	100.3	102.4	100.5	102.8
7.3	93.38	108.7	109.4	108.3	148.4	156.7	145.4
6.79	65.27	104.2	103.7	102.1	85.3	86.5	88.8
9.63	74.45	102.1	107.3	102.9	127.5	129.6	124.7
10.23	87.09	103	103.5	100.5	104.5	105.2	105
6.33	46.46	100.5	93.5	91.1	96.1	95.5	99.5
5.29	75.15	93.1	94.5	93	117.6	117.4	117.4
7.3	46.22	93.1	91	88	103.1	107.1	106.1
9.36	48.51	109	108.1	108.5	113.9	113.7	117.8
10.1	81.94	98.8	97.9	98.3	108.3	109.1	110.5
10.93	113.08	101.8	100.7	100	104.2	104.3	105.8
5.74	94.33	102	103.7	102.8	82.7	82.9	81.1
6.24	41.76	100.1	95.4	97.1	98.7	97	99.4
6.74	62.17	100.5	98.6	100.5	103.7	98.9	103.8
5.45	57.61	96.4	97	99.8	105.3	107.9	109
8.43	43.59	104.6	103.5	104.3	108	105.4	110
10.77	85.67	100.9	99.2	97.7	103.9	104.5	103.5
6.6	42.11	107.3	100.3	100.9	96.9	99	98.8

8.85	48.03	106	96.4	104.5	127.1	119.7	127.1
8.13	48.14	103.6	100.8	98.7	101.5	97.9	98.4
5.12	49.82	99.1	99.5	97.2	100	98.5	103.2
6.61	47.85	95.6	94.9	95.2	98	99.5	100.7
7.37	51.94	97.2	97.2	96.5	108.8	111.7	108.9
6.42	45.05	95.4	98.6	100.2	103.4	102.8	102.4
8.79	53.04	98.2	103.9	103.7	93.8	92.7	94.7
6.55	61.83	98.8	94.1	95	100.8	102.7	101.3
6.24	52.82	103.2	104.4	101.9	99.3	100.9	106.1
6.4	49.25	104	108	112	91.8	89.4	88.8
5.67	69.4	100.4	102.1	99.5	96.5	95.5	93.9
9.72	52.52	102.3	107	102.5	104	102.8	103
6.7	46.65	101.9	101	99.1	96.6	98.7	95.5
6.92	43.4	91.5	92.4	91.3	94.5	90.4	92.4
7.01	49.86	97.9	96	94	92.6	92.4	93.6
4.77	43.75	108.4	101.1	102.7	111.5	103.2	109.5
7.46	49.85	99.8	100.9	102.8	99.2	99.7	103.4
9.07	41.01	103.9	96.6	110.6	97.7	105.3	103.2
5.31	45.15	101.5	97.9	97.8	114.1	111.8	114.2
6.15	51.99	109.7	98	96.2	101	103.6	103.5
5.69	43.03	115.7	106.2	109.3	98	100.9	104.6
9.67	44.4	104.8	102.4	102.3	110.9	106.2	108.1
7.91	77.48	90.5	92	89.6	107.1	104.7	106.1
4.32	70.2	110.6	107.7	107.9	100	101.8	101
5.26	36.38	101.2	101.9	99.8	99.6	102	102.2
5.68	55.03	101.6	102.8	104.5	108.3	112.2	106.7
7.39	42.99	99.4	98	98.6	105.3	102.9	101.4
6.98	74.76	90.5	93.4	92.9	100	99.4	100.1
8.62	75.85	100.5	101.7	103.4	104.5	103.8	104.2
5.76	43.7	102.9	101.2	102.8	118.6	123.6	127.9
8.82	48.75	101	101.2	99.7	98.8	100.4	97.9
10.1	48.49	105.9	102.9	105.4	105.5	108.1	106.9
7.44	57	102.1	106.2	100.8	95.3	96.2	96.5
9.95	76.13	89.8	96.1	99.4	113.1	116.9	107.2
5.41	40.9	98.2	103.6	105.3	86.4	85.7	85
9.07	50.12	101.3	100.3	103.9	97.2	97	97.2
8.27	44.51	100.7	100.5	100.8	110.8	109.9	112.2
9.83	283.69	94.6	97.3	113.9	112.1	131.3	136.8
9.03	39.74	100.5	101.4	93.1	100.5	100.1	105.1
7.46	37.21	95.1	95.1	94.1	100.3	97.9	97.9
5.02	46.5	101.6	102	99.6	97.7	97.8	99.9
8.56	43.25	110.4	105.7	111.7	109.8	109.6	111.1
8.56	38.3	98.5	98.6	98.3	94.4	93.6	96.7
7.5	37.9	98.9	106.4	100.5	104.2	100.5	104.5
11.52	96.87	115.6	115.7	116.9	104.5	111.9	101.2
6.32	53.32	88	87	87.3	89.8	90.1	91.5
6.4	84.23	96.5	100.4	102.4	92	91.4	98
6.7	42.56	101.3	101.8	102.6	100.9	98.6	100.3
8.4	36.87	102.1	98.8	101.6	107.4	111.6	108.9
5.69	46.54	102.5	103.4	99	96.7	98.3	98
6.4	45.26	105.7	102	106.6	132.2	130.2	138.7

5.47	37.69	102.3	98.1	100.8	99.7	98.2	103
5.39	37.21	99.9	100.2	98.3	99.3	99.5	105
5.53	56.24	97.1	99.2	97.9	102.5	106.3	104.3
9.44	40	100.5	99.9	101.8	104.2	103	104.4
5.4	42.59	94.5	94.1	97.1	117.2	118.7	121.9
5.88	45.57	129.7	129.9	132.2	84.8	91.3	87.8
6.46	41.7	108.2	108.3	105.5	112.4	113.4	114.6
5.27	51.05	92.5	94	98.7	148.3	144.2	144.1
7.61	57.92	97.8	97.9	100.5	103	101	102.4
8.66	56.69	106.9	106.4	105.2	105.8	103.5	105.6
6.7	71.74	95.4	95.4	93.5	110	108.7	107.1
5.68	45.01	105.5	100.2	104.9	87.2	88.2	91.6
9.94	48.45	102.9	104.2	104.6	107	108.7	105.9
7.94	46.66	89.3	108.5	97	105.8	103.8	117.3
6.93	42.84	90.2	94	93.4	101.2	100.9	100.4
5.53	41.33	99.9	94.2	98.9	108.6	112.6	113.8
6.92	45.67	100.8	99.1	97.8	106.2	104.9	103.6
6.44	40.96	101.4	103.9	103.9	96.7	93.3	95.2
4.36	51.09	108	104.6	104.6	109.9	108.7	107.3
6.13	79.67	100.4	102.3	99.9	93.7	92.3	94.1
8.4	40.62	101.5	105.1	102.5	96	96.5	97.7
6.29	41.18	96	91.6	94.7	96.6	94.4	100.8
9.55	41.96	101.9	103.3	101.4	106.5	108.6	104.9
5.87	45.82	95	93.3	92.2	117.7	115.2	114.4
6.8	38.99	98.1	98.4	98.5	100.7	100.9	100.2
6.92	46.13	101	99.7	99.8	106.7	108.8	107.6
4.74	98.41	94.7	97.9	98.5	114	113.6	103.5
7.39	49.97	97.9	102.4	100.1	100.3	97.5	103.1
9.32	55.44	95.7	96	97	97.6	96.3	98
6.73	43.08	104.8	106.3	105.6	98.3	100.2	100
6.55	55.36	103.7	105.1	104.1	98.9	96.3	98.5
9.7	46.7	106.7	99.1	100.1	96.7	99.4	100.1
5.41	51.05	100.4	103.4	102.9	100.6	99	98.6
5.43	59.02	114.7	114.2	89.8	114	90.9	77.8
6.74	45.58	107.9	105.4	106	114.6	113.5	116
6.38	48.19	99.2	96.7	100.6	97.2	95.9	98.7
5.6	41.48	102.4	102.4	104.8	105	105.9	106.4
7.02	39.11	99.3	103.1	107.1	101.5	105.5	104.3
8.43	74.91	103.1	100.5	98.2	94.7	94.1	92.2
9.2	54.62	107.4	101.1	98.7	114.2	113.8	111.1
6.19	40.1	90.3	98.2	95.6	97.9	94.6	94.1
5.88	48.95	97.7	97.5	97.3	95.9	98.3	97.4
6.89	42.98	95.9	96.2	95.6	105.5	104.6	104.9
6.79	68.58	104.9	104.7	108.6	93.6	95	96.8
9.32	63.42	100.5	101.3	101.4	104.6	103.7	103.5
6.33	52.91	98.7	101.1	100	99.1	95.7	97.2
5.03	79.46	98	104.8	107	109.7	109.7	107.8
8.24	56.34	105.1	101.1	98.2	92.1	89.9	94
7.05	46.01	103.5	104.4	100.1	95.2	92.1	91.9
9.16	44.82	99.3	95.6	96.6	99.5	98.6	99.2
6.29	36.97	104.5	98.2	99.5	98.8	95.1	100.7

9.22	56.78	99.1	101.5	99.7	101.1	103	99.3
6.27	50.16	94.5	97.6	94.1	102.4	101.6	100.3
4.06	58.63	108.2	108.9	107.6	104	104.3	102.7
7.69	39.68	102.2	102.1	103.9	98.8	96.7	98.1
5.41	38.93	101.8	101.3	98.9	98	97.8	96.3
7.27	51.14	101.2	98.5	101	97.6	99.7	98.4
4.93	43.96	97.6	101.8	101.4	102.7	101.1	106.5
10.49	73.22	100.1	103.5	101.4	103.3	102.1	101
4.92	40.91	96.1	96	95	93.3	92.5	93.5
5.94	47.91	88.3	87.2	110.2	108.4	140.2	116
5.17	42.37	83.5	78.6	80.9	90.8	91.1	93
6.05	38.9	97.9	98.5	100.8	103.6	101	104.1
7.02	36.05	114	112.1	119	101.5	101.8	104.5
7.52	38.37	109	100.4	99.3	101.3	98.9	98.2
8.25	37.75	97.8	101	99.7	103.2	98.9	98.1
4.64	39.01	102.1	102.4	105.4	93.9	93.4	100.4
8.48	37.93	100.4	100.8	101.5	96.6	100.5	96.2
5.2	61.42	100.3	103.9	100.4	100.1	96.2	96.6
8.72	32.06	98.7	102	102.6	131.6	131.9	127.5
7.49	38.09	98.2	95.3	95.6	101.6	105.1	110.1
5.27	30.75	118.9	106.2	108.5	84.4	84.3	88.2
5.02	50.37	101.3	98.8	100.9	89.4	87.9	88.1
7.49	41.3	117.9	95.4	94.9	96.3	108.5	96.3
5.91	44.36	98.3	98.7	95.2	97.8	99.1	100.9
4.91	37.87	97	96.5	95.1	102.1	101.9	102.2
8.81	40.51	101.9	103.5	98.4	107.9	106	104.5
7.68	36.55	103.9	96.9	100.6	101.5	94	104.9
5.33	33.16	98.1	100.4	98.3	98.9	92.7	92.4
5.57	34.74	106.6	108.9	103.4	105.8	101.8	106.9
6.55	34.68	102.2	97.6	98.4	99.5	96.1	99.7
6.87	57.4	105	105.8	105.5	120.2	106.3	108.2
6.58	68.58	99.2	102.7	99.7	97.6	96	93.5
5.53	32.99	99.7	100.8	100.8	104.4	98	100.3
6.58	48.71	100.5	103.8	101.7	100.8	98.8	99
4.91	36.83	101.5	99.3	101.4	100.6	98.9	101.6
8.82	42.45	102.4	98.5	102.3	93.3	92.6	94
10.32	86.57	110.7	111.8	111.4	117.8	118.7	117
8.18	40.72	100.7	99.8	100.6	109.5	108.1	105.7
7.39	33.05	95.7	97.2	94.6	104.4	106.5	108.6
9.44	43.9	99.8	97.9	97.7	100.1	101.2	104
5.15	74.47	102	99.9	97.9	106.3	106.3	106.2
9.42	46.85	111.9	103.2	106	103.8	106.8	103.7
7.64	36.7	95.7	94.2	94.8	107.7	127.2	111.1
8.02	45.86	100.8	104.2	104.8	104.8	108.9	106.7
8.05	53.49	90.5	98	97.7	85.2	89.1	98.3
8.63	34.36	94.3	102.2	97.6	101.6	105.9	101.9
8.09	44.49	102	99.8	99.3	94.3	93.9	96.3
6.33	49.68	103.1	100.1	100.1	97	96.3	97.6
7.66	31.49	98.6	97	101.8	101.9	103.3	98.6
9.13	45.74	108.8	106.5	105.9	113.7	108.5	107.5
5.73	37.14	76.4	71.5	72.6	99.9	96.3	99.1

6.89	47.12	101.1	100.2	98.9	100.9	99.2	102.5
8.19	84.98	96.9	96.9	98.6	120.3	122.2	121.5
4.89	54.1	105.8	105.3	105.1	111.9	106.8	105.8
7.23	57.04	95.6	100.5	96.8	103.7	103.5	104.4
6.57	44.52	100.5	99.7	103	94.3	95.6	97
6.44	37.43	109.1	109.8	114.4	83.1	86.7	85.3
8.18	39.71	103.1	100.5	100.7	111.9	107.1	110.7
7.15	41.17	98.9	98.8	98.5	101.4	97.4	102.4
5.92	49.92	105.8	111.6	108.6	86.8	86.9	87
4.73	46.94	99.4	99.6	101.2	98.8	96.8	100.4
7.44	33.43	97.8	98.8	93.5	97.4	97.3	95.8
6.65	50.38	99.7	103.3	99.8	100.7	103.8	101.1
7.17	59.94	95.7	95.1	91.2	96.5	97.4	96.8
11.44	51.46	96.9	99.7	100.4	110.2	110.8	106.5
8.44	47.64	97.5	97	98.9	99.6	96.8	100.8
7.96	37.11	96.2	100	99.8	106.1	101.6	102.4
6.13	42.47	92.7	92.5	94.1	89.9	87.8	87.8
8.22	40.06	101.2	101.2	99.7	96.3	97.7	95.4
8	47.8	98.9	97.8	97.3	98.9	99.3	100.6
7.02	52.87	100.5	98.7	100.3	111.5	110.6	110.4
8.73	39.26	98.2	100.1	99.5	95	95.4	96.4
7.84	45.84	105.1	103.2	100	99.1	98	107.8
6.35	39.66	96.3	93.5	95.7	105.1	104.6	106.8
6.07	41.01	96.1	97.2	100.6	99.4	106.6	100.5
4.82	43.8	96.7	102.3	100	95.9	94.9	111.2
5.24	47.29	97.8	99.6	97.2	101.2	103.1	101.7
5.73	47.98	96.5	100.6	96.7	104.9	100.7	101.6
8.09	38.74	103.3	102.6	101.9	98.1	96.1	103.7
7.3	45.26	99.1	98.9	96.1	99.5	99.5	96
7.75	37.74	96.1	97.2	99.1	95.7	94.7	97.7
5.6	33.67	101.5	96.3	97.7	113.3	105.8	112.4
6.19	37.75	87.6	85.7	89.4	115.1	116.5	114
6.44	43.4	97.1	96	94.1	104.4	103.2	103.3
6.05	93.01	109.1	96.6	88.6	81.4	87.4	79.6
10.58	80.21	104.9	104.5	105	103.6	101.6	101.1
7.69	46.38	98.9	100.4	101.5	108	104.8	106.9
8.25	58.69	103.7	104.1	103	78.1	77.1	79.2
6.54	36.71	103	96.8	102.6	100.7	96	99.9
7.87	42.07	97.8	97.5	100.6	106.9	107	104.7
10.93	64.54	104.5	102.7	101.2	99.1	96.7	96.7
8.13	36.8	102	93.8	99.5	97.7	97.8	95.4
6.54	35.27	100.2	100.9	102.9	98.3	100.3	99.2
9.89	38.89	95.4	91.5	93.5	101.9	99.2	100.3
5.6	37.23	101.1	102	99.7	107.2	104.9	110
5.14	43.97	94.4	92	92.5	102.1	103.4	106.4
6.04	39.48	102	99.9	102.1	102	99.3	101.3
8.92	70.98	102	104.9	104.7	109.6	103.5	107.4
8.9	36.8	101.6	94.1	101.8	104.7	106	103.4
8.15	42.82	103.1	97.1	100.5	99.7	90.9	98.4
5.45	33.99	99.1	99.4	99.8	97.5	99.7	100.7
7.66	34.55	105.8	96.6	100	105.9	101.5	110

9.38	41.14	99.5	100.9	99.3	107.7	110.9	105.6
7.84	34.99	98.4	94.8	95.6	95.6	95.7	98.7
5.16	35.33	110.3	109	110.2	92.5	86.1	89
9.51	55.36	104.1	99.2	100	104.9	106.8	106.8
6.04	41.36	97.2	98.5	100.2	91.8	94.7	93.1
7.21	44.04	96.7	93.1	96.3	103	102.9	101.8
6.18	50.72	110.1	115.1	117.5	91.9	89.4	96.8
8.38	30.15	104.9	111.6	93.2	96.6	102	108.2
6.44	45.03	95.2	100.2	101.6	101.7	99.8	99.5
5.8	34.46	91.7	89.1	89.5	97.2	99	100.7
5.76	48.45	100.5	101	95.4	105.2	96.8	104.2
6.68	39.11	101.8	102	100.8	96	98.8	98.2
6.09	42.67	103.1	86.4	92.1	98.4	105.6	109.5
5.25	42.03	99.3	95.1	97	97.5	93.2	97.4
7.23	33.19	107.1	97.6	99.6	103.5	96.5	99.5
5.24	48.85	101.2	103.4	98.3	105.7	106.1	104.3
5.94	64.38	99.4	98.5	95.8	99.2	102.1	100.4
8.29	33.75	100.2	104.7	107.4	97.6	89.2	94.1
5.01	33.12	99.3	91.6	94.1	95.7	97.6	99.3
6.49	34	94.7	96.8	98.7	99.3	98.5	101
6.93	39.62	102.2	98.1	103.7	86.7	88.9	95.6
4.75	36.79	104.5	103	99.1	98.5	96.6	101.1
5.02	60.36	111.7	105.6	104.6	97.5	100.9	102.6
6.2	40.52	97.8	96.3	99.7	97.5	97.5	103.5
9.5	33.98	99.4	103.9	106	110.8	111.8	110
5.08	28.3	101.6	98.2	99.1	99	100.9	103.4
6.4	34.51	100.9	102	102.6	100	99.9	98.8
6.48	36.52	97.5	96.9	96.3	100.1	97.1	98.7
7.15	43.07	101	100.4	102.7	106.1	102.6	105.4
4.87	40.7	96.4	96.4	106.3	100.4	107.1	100.7
8.66	35.75	100	100.8	98.7	99.9	99.4	103.7
6.32	41.24	103.4	88.2	94.7	84.8	116.5	87.1
7.15	33.65	93.6	96.7	96.9	118.7	121.5	121.7
5.17	29.33	91.8	82	89.4	81.6	80.8	80.2
5.73	28.74	108.1	107.2	104.7	109.6	108.1	106.4
8.6	28.97	99.5	97.1	102.6	101.3	102.2	102
8.65	35.51	106.1	107.8	107.2	105.1	104.8	107.9
4.78	29.18	101.6	103.9	97.5	97.1	101.8	96.7
7.17	42.69	96.7	94.4	98.9	98	96.1	98
7.5	41.69	100.4	100.8	98.3	115.5	112.1	113.4
4.92	31.22	106.4	94.2	93.7	96.7	95.7	99.1
4.96	27.72	95.6	96.9	98.1	105.3	112.9	113.7
5.1	32.64	104.7	96.1	99.2	108.4	108.5	109.4
7.33	62.91	129.7	129	127.9	86.6	82.1	88
6.84	35.2	100.4	90.7	103.1	107.2	106.3	114.3
9.36	71.66	110.3	105.6	108.4	103.9	106.7	104.9
6.74	54.21	94.9	94.2	100.1	100.9	98.6	101.4
8.72	44.52	130.9	133.1	128.6	85.1	82.1	80.5
4.84	34.59	101.2	103.2	100.4	104.2	101.9	107.5
6.55	68.75	94.7	97.5	97.7	100.6	97	97.9
7.33	47.38	99.1	99.6	100	99.5	96.9	98.1

10.49	48.31	103.2	105.8	106.3	103.7	105.9	104.4
8.56	45.9	95	96.6	90.5	98.7	92.3	89.5
5.07	51.68	102.9	95.7	98.9	105.6	115.2	114.5
5.35	35.47	100	98.7	101.6	104.7	103.4	104.9
8.46	33.21	101	95.6	105.5	107.4	115.7	112.6
6	41.51	101.9	95.3	103.1	100.3	104.3	105.5
4.83	30.14	109.6	110.9	108.1	75.4	76.2	86
6.98	44.5	98.5	97.8	97	101.2	98.6	97.7
7.81	47.37	103.7	104.7	100.8	102.2	97.7	97.9
8.13	30.23	100.8	106.5	100.9	90.6	87.6	90.5
8.63	30.76	103.3	100.1	98.1	105.2	99.6	100.1
5.91	43.62	102	101.8	105	109.8	108.6	109.9
8.85	32.8	102.2	101.3	98	94.5	95.3	93.1
6.54	30.29	97	89.9	97.6	100.2	104.5	104.7
10.9	82.08	116.2	111.7	112.7	106.5	105.2	105.3
8.62	32.98	97	96.5	103.2	107.4	106.9	103.7
7.24	36.34	100.8	102.9	98.4	102.1	95.4	100.9
9.52	44.45	96.3	103.7	99.8	107.1	105.5	107.9
6.06	28.71	94.5	94.3	94	95.7	100.9	106.1
5.44	54.48	102.8	100.4	100	100	97.8	100.3
5.9	31.5	101.5	103	104.2	103.5	96.6	99.2
6.83	35.72	103	101.1	97.5	106.1	102.9	101.6
4.79	42.45	99.2	97.5	97.7	101.4	101	100.9
8.65	44.65	103.7	101.9	104.6	105.6	109.1	105.4
10.65	55	100	99.8	101.5	105.5	104	104
12.02	90.86	100.9	100.3	100.7	105.2	105.2	106.6
7.4	32.65	109.6	105.3	107.8	106.4	95.6	103.4
10.46	64.75	102.2	107	104.8	98.6	100.5	97.6
6.37	37.24	89.2	91	92.2	99.6	101.8	101
7.01	40.5	102.2	100.7	100.3	99.5	94.1	97.6
9.95	58.17	99.5	99.1	102.1	105.3	103.7	103.1
6.3	72.06	94.4	94.4	93.1	108.1	104.5	105
6.14	36.19	94.2	93.4	98.6	100.1	97.9	107.1
5.16	40	97.1	96.8	94.2	106.5	111.2	111.8
5.96	25.34	94.5	94.1	99.9	97.3	99.3	101.6
5.6	42.63	103.2	98.9	101	136.5	142	144.2
8.66	44.18	97.2	92.6	93.5	98.6	96.6	98.1
7.71	31.56	99.6	91.5	92.9	110.1	104.9	110.7
6.24	36.42	89.2	94.5	92.5	96.7	97.1	100.6
9.38	43.03	101.9	104.7	104.7	106	100.8	102.5
5.59	46.99	93.9	94.4	94.3	95.6	97.4	96.5
5.24	47.12	103.4	103.6	101.1	102.2	101	101.8
6.37	24.49	99.1	96.5	93.5	97.6	95.8	101.8
4.91	80.7	100.8	102.1	103	107.5	106.1	108.5
5.73	32.15	101.3	98.2	100.6	135.2	136.5	135.6
9.29	30.62	102.2	101.9	104.9	95.5	98.1	97.7
7.15	36.34	102.7	102	103.1	91.3	87.7	90
4.97	33.61	102.6	99.2	98	100.7	101.8	99.9
4.94	49.2	98.7	98	97	108.4	99.4	107.5
9.77	46.25	109.5	109.1	110.3	98.8	99.3	98.5
6.19	26.6	107.5	102.5	102.6	106.3	105.9	109.6

4.92	39.66	100.9	99.4	101.8	97.7	100	102.8
6.13	28.96	104.4	106.3	102.9	106.8	106.9	113.1
7.85	29.71	94.2	98.4	93.7	97.4	98	102.2
5.5	43.93	94.9	92.2	98.1	99.6	102.5	104
9.52	47.9	99.3	102.1	104.4	100.4	99.4	100.6
8.12	33.37	100.1	101.3	101	93.1	95.1	93
5.59	45.66	102.6	100.2	100.6	96.7	98.8	95.4
5.48	114.46	104.1	103.2	112.5	95.6	104.9	95.1
5.07	33.52	97.4	103.7	101	102.4	104.6	104.1
6.95	43.37	102.5	102.2	103.3	95.6	93.5	94.1
7.58	29.59	101	100	96.5	109.2	107	109.7
7.69	35.62	95.9	96.6	94.8	93.7	98.1	93.9
7.65	38.81	92.8	100.7	92.4	95.1	92.4	98.2
6.2	38.4	103.7	102.8	102.7	90.9	91.7	93.2
6.84	34.52	107.3	101.5	102.5	109.7	111.1	108.6
4.64	55.02	97.2	96.8	96	99.1	98.6	98.9
5.14	25.76	97.8	93.8	98.1	106.8	100	100.6
8.97	47.91	103.2	101.3	102	95.8	94.8	98.9
6.2	36.87	99.7	104.7	106.2	103.1	99.3	100.8
4.09	55.65	109.8	107.9	113.2	99.6	99.2	97.8
4.82	52.67	101.8	99.3	99	98.2	103.5	102.1
7.43	53.35	109.4	95.9	102.6	95.8	93.9	95.8
5.57	52.98	95.1	95.6	91.4	91.4	88.9	89.5
8.07	33.65	104.3	104	106.1	93.5	97.5	92.2
5.82	39.77	106.7	99	103.1	99.4	100.9	104
7.44	46.28	101.3	96	97.7	102.5	105.5	102.5
9.38	32.35	97	94.7	94.3	94.1	90.2	94
5.44	37.31	103.8	103	99.9	109.9	108.3	107.3
5.35	38.08	102.2	101.4	101.1	96.6	100.2	101
8.48	28.67	103.6	96.6	100.3	106.8	103.7	103.5
6.32	34.38	98.8	96.1	96	104.2	105.6	104.4
9.16	32.11	106	101	95.1	100.3	93.2	97.5
6.65	38.74	118.4	122.7	124.4	101.8	97.3	95.5
5.62	36.49	99.8	96.3	95.9	100.2	98.5	99.3
6.93	31.92	97.4	96.9	97.5	95.3	95	94.8
8.79	35.85	97.8	98.7	96.2	102.4	100.4	101.3
5.15	31.89	107.1	105	103.7	100.1	97.9	95.9
6.77	37.55	116.6	113.9	114.3	73.4	71.3	73.5
4.86	39.78	103	100	97.3	109.5	102.9	108.4
6.96	34.43	97.9	94.3	96.2	98.6	99.2	98.5
9.19	47.57	100	102.5	97.8	97.6	100.4	102.9
7.3	35.71	96.4	103.8	102.3	102.6	107.6	103.5
6.77	36.95	100.3	103.7	102.6	97.8	90.2	95
6.48	26.3	97.4	99.5	107.4	103.6	99.7	102.5
5.01	30.32	97.7	93.3	101.6	88.8	95.2	100.1
6.95	31.46	94	94.9	91.7	100.3	100.7	101.6
7.33	28.33	93.6	101.9	101.3	102.5	104.1	103.7
8.59	33.48	107	103.2	100.7	101.9	102.7	106.5
8.63	25.78	104.7	106.3	104.6	106.4	103.9	101.3
6.9	29.5	96.1	105.4	107.7	106.8	111	110.8
5.66	24.28	102.6	103.2	105.6	106.6	104.3	104.1

9.57	26.59	98.4	101.1	100	104.1	102.1	104.2
8.88	25.73	90.2	101.8	93	103.9	113.5	108.7
6.74	28.93	100.5	92.8	102.7	99.1	100.9	107.5
5.4	38.05	101.7	100.9	102.8	97.3	98.8	94.8
6.62	45.58	97.6	102.2	102.2	99.4	97.4	98.7
6.32	39.97	102.7	100.5	100.6	109.9	103.4	105.3
8.13	31.48	105.6	100.1	101.7	100.9	96.3	101.9
5.83	30.25	101	94.7	97.9	103.4	104.4	102
10.1	69.52	96.5	99.9	99.2	103.5	107.6	107
9.54	41.18	102.9	100.9	103	122.9	124.5	120.4
8.81	52.07	99.9	100.8	100.4	109	107.7	106.9
5.74	32.71	98.1	99.2	103.9	96.2	97.1	96.5
7.44	41.27	93.9	97.3	101.7	104.8	102.9	102.9
9.6	53.48	100.3	101.1	102.4	112.9	112.6	110.7
4.91	35.58	100	96.8	98.3	98.2	97.6	97.6
4.7	45.74	100.3	99.4	96.1	106.8	104.5	104.7
4.86	92.61	59.8	56.8	57.3	168.3	166.9	167.7
4.26	44.97	93.9	96.3	96.8	97.7	98	97.6
8.63	38.65	97.1	97.7	94.9	95.3	94.1	96.6
7.77	39.09	99.4	96.1	100.5	100.8	104.1	101.2
8.97	44.56	96	97.9	100.4	108.9	111.5	110.4
8.24	33.46	104.8	101.2	105.5	100.8	107.1	105.2
8.87	40.34	119	116.2	107.5	105.6	82.6	89.2
5.16	31.59	95.5	92.8	92.9	91.5	92.2	91.8
6.43	32.94	109.8	93.3	91.8	90.2	93.2	104.5
9.5	31.69	95.9	99.2	95.9	111.4	113.5	115.5
6.67	43.62	100.7	95.7	103.9	99.2	94.2	85.4
9.14	36.13	101	105	104	102.4	101.6	102.3
7.01	41.22	102.4	98.7	99.9	97.5	101.7	100.8
6.64	24.02	93.3	101.2	103.2	101.7	103.1	100.9
6.01	36.32	103.3	101.7	107.1	94.1	95.7	93.2
7.99	25.85	73.8	69.5	69.6	91.4	91.2	93.4
5.4	34.68	104.1	100.2	101	97.5	93.3	97.3
6.3	41.17	134.8	122.1	119.6	61.4	77	73.3
8.48	33.77	99.9	103.5	102.5	98.5	100.9	102.5
7.87	45.59	104.1	96.7	104.4	107.2	104.5	108.3
6.28	33.69	90.3	89.6	88.7	102.6	102.2	105.8
8.91	27.04	96.3	103.7	97.8	101.1	100.7	101.8
8.47	33.08	97.7	100.4	100.2	99.4	99.1	103.3
7.3	34.41	100.1	99.3	99	101.7	97.9	101.3
8.66	40.84	102.9	101.3	105.6	103.2	104.4	101
8.4	28.79	103.1	98.4	98.3	110.8	108.9	108.4
8.09	28.29	98.6	99.6	94.1	96.1	99.1	95.3
5.97	140.03	93.7	89.8	108.4	82.3	118.8	97.8
5.17	41.74	96.8	102	100.1	90.1	91.2	93.4
8.28	30.89	104.6	102.1	110.6	95.5	102.2	95.1
4.32	72.44	98.1	101.1	97.4	104.3	101.6	100.8
9.8	28.34	98.3	93.5	101.5	105.4	104.3	105.8
7.75	31.29	103.6	104	107.3	115.5	114.3	116.1
8.21	28.73	92.7	94.7	92.7	102.7	102.4	104.4
7.43	28.82	92.3	90.7	92	114.2	119.2	124.3

6.47	51.82	94.3	94.5	93.3	101	96.5	97.7
8.56	45.01	102.7	99.6	97.7	102	100.5	101.5
4.92	35.04	96.6	96.2	104.9	106.6	107.6	109.4
4.87	36.59	102.3	102.8	98	103.6	104.5	104.8
4.44	36.12	97.2	93.1	102	104.8	98.4	99.9
7.36	35.02	94.3	91.3	96.7	106.5	103.9	99.8
5.11	24.83	100.9	99	104.3	99.4	98.7	103.4
7.12	95.06	102.6	100.8	99.8	94.8	95.4	94.3
6.76	32.04	99.3	95.1	97.1	101.1	102.7	104.8
6.44	38.19	95.5	94.1	94.8	97.7	100.3	100.5
6.52	31.51	104	102.2	100.8	108.8	106.4	105.8
6.6	25.27	105.1	100.2	99.6	109.5	107.4	111.7
7.17	36.87	106.5	106.5	107.1	96.4	96.3	90.9
8.95	25.33	94	93.7	99.8	102.5	100.2	103.5
6.71	28.98	97.5	97.3	95.7	104.9	103.6	107.2
5.31	31.31	100.3	98.4	95.4	110.1	99.6	103.1
10.11	56.1	101.7	106.9	103.8	110.3	108	106.2
8.02	50.27	101.1	104	101.9	100.2	96.4	95.2
8.73	34.13	97.1	97.5	94.6	114.8	118	114.7
8.78	37.46	103.4	101.1	105.4	107.7	110.2	110.8
8.02	36.67	78.4	73.8	77.6	96.2	101.1	95.6
9.95	40.86	98.1	98.6	99.8	110.5	110.5	109.1
6.18	25.86	97.2	89.2	92.8	115.2	112.5	113
7.61	41.95	85.1	89.3	87.2	79.8	95.1	96.6
5.11	41.94	97.8	99.5	99.7	100.1	99.5	98
6.34	29.63	98.8	96.2	98.8	100.1	100.6	101.9
6.77	26.16	102	93	91.5	99.4	102.9	101.3
5.14	28.61	102.8	96.1	99.5	96.2	105.7	100.7
8.22	42.39	99.8	97.6	104.9	100.3	105.7	97
5.57	31.69	75.8	81.4	81.8	121.9	125.1	129.8
6.47	31.02	97	98.8	100.3	102.4	105.4	102
7.46	27.84	107	105.9	104	110.3	102.7	107.7
6.44	34.6	105	98	102.7	94.9	95.4	93.6
5.92	37.55	92.2	94.3	94.7	96.9	100.7	100.4
5.17	28.85	108.7	104.4	104.8	90.8	91.5	92.3
8	24.02	105.2	99.8	99	100.2	115.3	106.8
5.4	29.15	102.8	99.8	105.6	96.3	99.9	98.6
8.09	31.75	94.4	97.5	94.5	106.2	117.2	102.7
11.59	59.47	97.1	99.4	99.6	105.6	104.8	107.4
6.65	41.21	97.9	98.1	97.6	96.1	93.8	97.5
5.81	32.8	106.7	107.2	110.5	100.1	94.5	97.1
5.71	27.88	97.3	98.7	100.9	100.8	103.7	106.3
6.18	24.27	101.4	91.1	91.6	94.6	94.6	94.8
5.59	30.61	107	99.7	98.8	101.1	99.9	99.7
6.55	28.85	103.6	97.2	101.9	106.2	112.1	111.4
7.39	35.21	100.3	99	103.5	98	101.4	102.2
6.67	31.16	104.6	102.5	107.3	100.7	99.5	104
6.01	26.92	102.6	98.8	98.3	99.5	99.3	109.8
7.56	36.31	102.9	101.5	106.7	105	105.3	107.9
6.55	41.11	95.6	98.1	99.4	102.5	101.2	102.4
5.83	38.98	105.3	107.2	107.5	102.8	102.1	106.1

5.86	29.19	99	96.2	97.9	100.7	97	98
6.02	42.49	104	101.4	102.4	108.6	107.6	108.8
8.12	21.15	100.4	100.9	102.1	101.7	102.1	110.5
10.55	47.52	104.8	104.5	105	107.1	107.9	106.8
7.34	22.57	99.1	101.5	96.8	99	98.3	102.6
7.71	33.41	111.3	114.7	108.7	88.2	85.1	87.2
6.15	46.76	97.9	99.8	94.5	97.9	95.4	96.5
7.96	27.59	100.3	95.1	94.6	100.5	101.9	105
4.77	34.1	106	103.1	104.6	96.8	96.9	96
5.81	28.78	103.6	106.1	104.2	99.8	99.1	100.5
5.55	30.71	106.2	102.8	103.6	104.2	102.8	106.4
7.68	35.02	100.6	100.8	100.3	104.3	99.9	100.3
11.82	46.49	101.9	102.9	103.6	112.2	107.3	108.6
11.59	49.43	100.4	101	100.4	114.8	118.3	116.3
9.55	64.49	103.2	93	99	103.7	100.3	112.5
5.55	31.42	95.6	99.9	99.1	100	96.7	97.4
4.63	32.49	101.4	97.7	99	96.6	97.5	97
6.6	38.21	99	99.3	96.4	100.5	98.1	100.5
7.81	31.38	111.1	114.1	113	107	105	102.9
6.16	29.13	102.7	103	98.7	110.4	104	106.4
7.53	27.54	110.8	106.9	106.2	99.1	96.7	95.8
6.6	35.2	102.4	100.9	98.3	90.8	89.1	94.8
9.91	35.14	104.1	101.7	104.6	98.2	98.5	100.2
6.23	26.23	97.1	100.8	95.3	94.5	103.3	129.3
6.95	28.48	99.4	94.3	94.3	100	102.3	99.7
8.88	36.71	102.3	103.6	101.7	103.6	102.2	103.2
7.03	31.21	96.1	98.6	94.9	101.9	97	100.6
4.81	45.79	78.6	76.4	77.3	126.1	119.2	124.9
7.74	28.32	95	98.8	98.7	113.5	107.7	107.3
5.73	35.65	100.4	101.2	100.5	102	101.8	103.4
5.3	33.07	98.5	97.2	101.8	98.5	98.2	100.8
5.62	27.41	98.1	92.6	87.2	111.1	107.4	107.8
10.13	66.84	100.5	97.6	101	105.5	104.5	107.4
6.46	21.97	102.9	99.9	100.6	97.7	88.9	93.4
5.55	34.79	99.1	99.3	98	103.5	104.1	101.3
8.48	30.44	96.3	96.2	97.7	108.8	102.4	106.2
11.25	28.26	104.4	109.3	103.9	109.4	104.9	99.6
9.5	27.34	102.5	102.1	102	104.3	103.1	104
7.28	34.99	117.3	115.6	118.4	75.8	74.7	73.8
8.29	38.32	101.1	102.5	99.7	102.8	102.6	101.2
7.8	24.32	97.5	91.1	95.5	100.8	105.8	100.7
7.99	27.37	99.6	102.3	106.1	99.3	96.8	96.8
7.31	28.03	95.5	94.3	96.1	99.5	100.8	106.8
6.89	25.4	98.8	104.7	100	91.7	96.1	92.6
8.68	31.5	111.6	105.3	104.9	101.3	97.4	101.4
6.23	29.47	99.7	107.1	97.2	101.2	100.5	101.7
10.49	66.28	101.8	104.5	101.5	104.3	102.8	102.6
5.22	25.8	102.8	99.6	96	99.2	94	94.3
5.25	31.7	99.6	98.8	102.9	101.2	102.7	106.1
8.78	28.32	107.9	100.5	104.1	104.5	102.1	104.3
6	38.22	96.8	96.6	95.5	99.4	98.4	101.5

7.78	53.92	96.4	98.5	97.7	105.1	107.8	103.6
5.92	45.57	106.5	106.5	104.2	104.6	101	102.4
5.14	23.04	98.4	95.4	97.4	97.3	98.6	96.8
6.48	25.42	95.3	94.4	98.5	104.4	104.5	103.8
5.45	28.29	101.7	99.7	100.7	102.7	103.4	100.8
9.09	24.26	101.8	100.9	108	96.3	96.8	118.8
5.97	30.73	108.4	89.3	88.3	98.1	127.4	110.5
7.8	33.33	102.1	102.2	101	102.9	99	98.9
8.38	29.76	106.4	102.5	100.5	95.1	102.5	99
7.08	38.52	74.7	87.3	106.8	117.6	91.1	94.9
8.24	25.56	103.3	104	101	91.5	86.3	86.2
6.7	33.02	101.5	101.1	99.9	97.6	97.1	96.3
11	44.64	99.2	95.1	98.2	105.7	106.8	105
8.15	27.73	89.2	90.1	89.9	106	106.2	106.3
8.91	40.53	101.8	100.7	98	99.2	103.7	102.8
7.36	24.52	102.6	101.3	102.2	105.1	104.1	102.9
8.66	25.92	106.2	103	104.2	88.6	93.7	94.8
6.28	30.63	90.4	87.3	86	87.8	93.5	91.2
7.21	29.14	103.8	96.4	103.8	97	98.9	99.6
6.2	39.42	102.7	103.2	98.6	99.7	97.9	98
8.82	29.6	102.4	101.1	100.1	99.8	96.1	93.1
6.54	35.32	95.5	87.2	86	98.1	98.5	99.9
5.76	32.79	127.1	126.6	129.4	69.3	71.9	71.7
7.08	27.54	97.7	100.9	101	109.6	110.2	113
7.24	28.52	100.1	100.4	97.5	103.5	102	103.1
6.11	36.96	109.5	105.3	111	102.7	102.7	102.8
7.9	38.97	103.4	99.8	110.4	71.1	80.9	87.3
8.43	29.23	93.6	98.6	97.8	104.9	106.7	103.7
9.76	49.29	104.7	102.2	99.1	105.4	104	103.7
6.79	31.39	101.9	103.3	103.8	104.3	106.9	106.8
8.21	26.56	94.6	95.1	94.1	109.6	105.5	105.7
6.71	67.21	98	97.7	99.6	95.7	98.2	96.8
6.16	44.16	104.4	102.6	104	106.6	103.3	104.8
7.4	48.71	99.6	101.2	98	101.7	102.2	99.7
7.88	25.82	96.4	95.5	90.3	98.2	100.7	96.7
6.74	30.38	96.9	93	95	101.1	101.6	100.6
5.96	20.87	94.6	99.7	103.4	97.5	101.6	102
5.33	28.87	98.1	100.2	97.9	98.6	97.4	100.6
5.4	32.35	99.2	100.4	100.2	106.2	97.9	99.2
6.89	26.19	106.3	101.1	98.3	103	98.2	99.5
6.87	28.35	98.8	100.5	96.4	96	93.8	91.8
4.84	36.11	95	101.1	99.4	114.1	113.5	113.7
7.43	28.99	96	91.7	92.5	100.4	101.8	99.4
5.48	33.9	105	99.6	102.8	110.7	105.9	105.4
6.42	26.88	105.7	100.1	97.6	108.2	103.5	104.2
5.53	30.78	100.3	101.9	98.2	101.1	106.6	103.3
6.84	29.83	83.7	83.5	82.3	100.2	95.1	98.9
9.25	28.37	111.9	111.4	107.7	99	99	98.1
9	27.52	101.2	99.6	102	96.1	119.9	99
5.71	21.05	124.1	106.5	112	80.9	78.9	86.8
5.71	25.34	104.5	97.9	97.8	97.2	98.7	95.9

7.88	24.1	99.3	97.7	100.6	116.1	107.1	116.8
11.05	23.47	100.2	102.4	112.3	106.3	106.3	112.2
9.17	37.2	93.6	100.6	98.8	98.3	95.7	100
6.14	30.11	99.6	95.6	98.7	114	111.8	109.7
6	31.18	92	88.9	93.4	98.3	105.3	104
9.91	84.14	103.8	106.2	103.2	104.9	105.1	103.2
9.16	57.33	95.7	98	96.7	90.5	91.9	92.6
4.84	27.73	98.8	97.3	97.4	101.5	105.4	106.5
4.94	41.12	102.4	95.1	91.7	100	98.6	106
6.68	38.73	100	101.2	99.3	102.5	102.7	102.9
7.99	42.76	102.9	104	98.1	105.5	100.8	104.1
5.97	48.23	102.7	96.7	98.5	100.8	101.1	106.7
6.43	30.1	105.6	98.2	101.7	113.9	102	108.9
9.22	27.27	107.7	94.5	109.9	109.4	116.6	109.9
4.4	28.69	87.8	112.4	98.9	105.7	113.8	98.9
9.13	36.02	102.9	102.7	96.8	96.7	96.3	93.4
5.76	30.11	100.1	98.7	100	112.1	108.8	110.3
6.44	21.11	95.8	102.3	94.5	97.8	100.2	99.5
6.3	27.08	97.2	110.5	92.3	70.5	65.1	78.7
8	28.61	99.4	94.8	90.6	101.4	103.5	104
5.73	28.24	109.6	98.5	97.1	94	103.3	98.1
9.17	34.19	101.6	99.5	105.1	99.3	100.5	103
4.77	26.37	99.7	96.8	94.3	110.9	110.9	110.3
9.16	30.71	101.6	104.5	105.1	96.1	97.6	100.7
7.3	42.9	93.4	91.2	90.8	105.4	104.2	104
6.61	33.71	107.3	102	102.3	102.5	104.6	104.8
6.9	31.86	100.6	96.7	100.1	103.8	104.9	105.5
6.52	27.16	101.7	99.6	111.8	103.3	104.8	101.2
8.18	35.25	95.4	96.4	99.1	100.7	102.4	100.9
4.96	48.54	94.9	93.5	94.8	102.6	100.4	100.4
5.9	26.58	99.8	96.1	95.2	100	101.1	99.8
8.06	26.46	96.6	98	94.7	103.3	104.4	103.7
6.92	38.1	98.3	96.6	97.5	101.3	97.7	102.1
5.01	29.53	95.8	95	98.5	97.1	100.7	103.1
5.06	24.91	97.9	100.9	100	101.4	106.3	104.1
5.22	33.44	103.3	100.1	97.8	109.4	101.6	108
9.28	26.42	94.6	99.3	95	106.7	102.7	103.2
9.94	55.54	101.3	100.9	103.2	102.9	103.6	106.9
8.68	49.26	84.1	82.7	85.1	111.9	115.1	114.2
6.87	26.73	104.9	103.2	101.3	107.4	107.5	109.4
7.49	19.93	104.4	105.9	100	94.8	89.4	96.8
6.99	30.4	96.2	103.4	93.8	92.2	85.8	87.4
9.67	27.91	102.1	102.3	98.6	110.1	110.1	109.8
7.78	46	97.9	98.7	96.2	105.1	104.7	103.2
9.44	30.09	109.3	102.4	107.2	106.8	107.3	110.2
8.32	37.35	105	105.9	102.9	110.7	109.8	109.1
9.16	26.5	105.2	96.7	102	103.6	112.4	106.4
5.58	25.46	101.9	103.5	98.9	101.1	104.9	99.7
5.67	24.35	101.5	98.1	94.9	110	107.6	109.7
5.83	30.64	90.4	87.7	110.9	102.2	133.7	114.1
5.57	36.95	98.5	94.5	97.8	89.5	88.4	89.3

9.04	27.05	102.4	96	98.4	114.3	115.3	108.4
10.55	40.28	97.6	103.2	101.7	133.4	132.8	127.4
6.76	26.93	97.3	98.8	99.3	90	90.2	93
7.71	34.32	107.8	102.1	104.4	126.9	129.3	120.6
5.24	24.86	100.8	102.3	102.8	85.2	93.2	94.8
4.91	25.85	99.7	95.2	99.4	102.6	102.1	109.4
4.81	21.32	88.7	95	98.6	102	118.1	117.3
8.09	60.98	101.6	107.2	95.2	95.2	89.6	99.5
8.02	29.51	113	103.6	103.9	98.7	103.2	97.6
8.98	29.87	101.4	100.8	101.6	108.1	107.3	108.4
10.32	46.2	104.1	96.6	103.5	104.7	104.7	108.6
5.53	30.92	105.4	109.3	108.7	102.9	99.2	100.7
8.07	25.99	100.8	96.8	95.5	107	104.9	104.8
7.8	28.72	102.6	95.7	98.2	94.2	91.7	95.9
5.95	22.46	104.8	103.6	105	93.4	88.4	96.1
8.29	33.31	102.6	101.8	104.4	107.8	108.7	111.2
6.44	22.69	103.9	104.9	99	105.2	104.4	102.3
8.54	49.61	95.2	99.2	100.4	98.7	98.4	101.3
8.18	42.55	102.2	96.8	98.9	101.1	98.4	98.3
7.77	27.01	102.1	96.1	99.9	101.3	97.1	92.6
7.42	25.68	101.6	97.4	97.7	96.5	96.4	97.3
6.74	25.29	102	100.8	96	97.1	98.5	110.9
7.39	31.59	98.2	95.8	97.5	108	99.1	106.3
10.74	63.59	110.6	104.8	119.3	101.3	116.9	107.8
5.86	27.63	89.6	88.9	89.2	95.2	94.1	91.2
6.38	26.02	92.8	92.6	90.1	99.8	100.6	102.4
4.63	27.47	101.2	97.1	100.9	106.5	103.3	104.8
8.4	48.28	105.9	104.5	105.7	90.9	92.3	91.3
5.26	24.2	99.4	96.5	98.8	98.2	96.1	106.5
8.05	28.42	87	77.7	124.4	89.8	109.7	95.2
7.3	30.6	92.8	93.1	96.5	100.5	102.1	101.8
7.39	28.37	105.6	102.6	106	125	129.5	122.1
6.96	36.22	100.2	99.7	99.1	97.3	96.7	94.7
5.17	48.03	99.2	102	99.4	102.7	106.6	107.5
5.33	23.31	99.6	96.2	98.7	101.2	106.3	105.1
8.48	19.39	100.1	103	103.5	109.2	106.1	109.2
6.95	26.96	100.2	106.3	96.4	104.8	102.5	105.1
6.47	19.7	97.1	92.5	97.3	102.9	105.6	110.1
4.91	48.29	112	110.2	124.7	94.1	88.7	91.3
9.66	36.9	96.5	96.6	97	104.8	108.2	108.2
5.4	42.57	94.4	96.8	93.5	114.9	113	114.6
9.91	24.83	108	108.6	100.1	111.6	105.3	102.9
6.28	23.13	103.8	114.3	100.5	103.3	108.6	107.5
7.46	37.33	108.2	106.5	106.5	91.4	96.3	95.5
8.59	34.02	95.6	97.6	94.5	99.4	96.6	98.1
5.52	37.42	94.1	94	93.8	103.2	101.3	105.4
10.01	26.06	104.1	103.8	102.2	106.4	105.9	103.5
4.94	29.09	106.8	88.7	101.8	102.5	111.4	104.8
8.31	27.28	96	99.1	99	96.3	97.4	94.4
5.27	29.82	99.1	98.4	92.6	88.2	88.6	89.6
6.67	19.24	98.6	97	100.6	101.4	100.1	101.5

4.84	26.98	97.1	99.5	100	100.5	105.4	105.2
5.54	25.95	102.6	101.5	100	98	97.7	98.4
6.11	36.52	101.3	99.4	92.4	98.9	98.4	102.5
8.41	24.38	96.8	99.8	98.7	100	99	96
6.74	27.26	104.6	102.8	105.3	82.6	81.4	85.9
7.39	27.11	103.7	104.4	100.6	96.5	94.9	95.8
6.11	36.09	101.4	100.5	94.8	104.5	102.8	105.9
5.07	26.12	96.9	97.9	100.4	89.6	97.8	100
10.58	56.17	113.3	110	108	111.4	110.3	110.6
7.44	19.46	93.5	97.2	94.6	116.3	110.4	109
5.33	21.74	100.8	100.7	102.7	100.3	97.7	97.6
7.78	33.64	94.4	95.8	97.2	101.2	103.5	102.6
6.9	26.33	97.6	95.6	95.9	104.6	92.9	101.9
8.09	22.88	101.2	96.9	93.4	104.2	105.1	104.3
8.38	24.31	112.4	110.5	110.7	108.4	111.2	112.9
8.44	23.9	105.4	102.5	100.4	108.5	105.1	104
9.07	35.05	97.2	96.7	102.5	101.3	103.4	98.3
8.1	28.25	103	99.4	100.1	99.1	100.7	98.9
8.1	20.7	103.1	99.2	101.8	107.1	108.3	106.9
8.53	34.19	95.4	97.3	99.5	102.3	100.8	100.6
6.58	26.05	92.2	90	90.9	97.9	89.7	97.9
7.78	37.59	100.2	96.1	102.5	96.3	101.4	100.9
7.78	31.04	95.7	95.4	101.8	104.2	106.9	103.1
5.66	33.38	89	95.6	89.2	119	114.9	121.8
7.5	23.59	99.3	99.9	104.4	93.1	91.4	94.1
5.73	21.16	99.1	96.6	98.6	101.9	98.4	99.2
3.85	22.81	102.6	113.3	111.3	105	94.1	98.8
8.56	26.15	99	101.3	94.6	100.5	99.4	101
8.56	24.72	105.9	97.6	102.3	100.5	102.4	107.5
7.87	18.26	102.3	100.7	101.8	109.6	107.4	108.8
6.44	19.45	96.8	93.5	97.5	105	105.4	104
4.79	26.38	105.9	100.5	104	103.6	101.2	103
6.89	21.29	93.7	97.8	100	95.6	94.4	96.9
9.36	26.65	95.1	94.8	94.8	97.7	97.6	98.7
6.1	20.9	99.6	99.2	95.2	98.8	97.7	100.4
8.29	24.14	101	98.8	104.8	102.2	113.6	109.6
6.96	31.27	105.3	102.5	104.9	104	104.3	103.8
6.29	29.41	108.6	102.3	98.8	98.8	95.2	96.5
6.77	36.26	100.1	99.3	101.2	106.8	102.3	104
8.38	23.31	99.4	103.8	97.2	102.7	109.6	99.6
8.12	24.21	93.8	98.4	95.7	110.4	105.7	106.2
6.29	24.26	107	105.5	111.4	86.1	79.7	81.4
6.71	24.94	96.3	98.1	98.9	101.1	97.5	100.9
5.85	20.95	100.9	102.7	105.4	91.5	95.2	96.7
6.7	24.97	112.9	100.1	103.9	101.7	99	100.2
9.45	25.75	105.2	104.6	107.2	95.3	96.9	96.1
6.29	25.3	114.4	111.7	114	90.5	81.5	80.6
5.5	21.78	97.9	105	101.3	103.5	104.9	108.4
7.14	29.98	105.2	105.5	104.7	102.9	101.7	108.4
4.7	23.13	108.6	106	112.1	107.3	102.5	97.7
8.82	27.13	102.2	93.8	104.7	100.6	96.6	100.8

5.71	23.35	95.4	91.2	92.2	104.9	105	104.4
11.05	49.99	106.9	98.1	101	105.1	110.6	104.8
4.7	34.44	94.3	92.9	94.5	107.1	108.4	106.8
7.52	26.79	130.3	128.6	124.5	77.5	73.1	74.7
5.31	20.49	96.7	86.9	95.9	93.4	92.2	96
5.12	26.74	99.5	98.8	98.4	95	96	96.2
9.36	33.7	102.3	100	103.2	99.1	100.7	101.4
7.4	23.65	94.2	96.1	95.8	94	95.6	96.4
10.43	42.86	102.8	101	99.9	97.4	96.7	98.7
7.88	27.99	99	102.8	100.5	102.9	102	99.2
7.25	21.64	96.1	91.6	95.6	101	97.5	98.8
6.28	33.01	97.9	95.2	98.8	97.3	100	101.8
5.01	31.38	94.9	99	97.7	97.4	96.1	101.7
8.4	26.95	101.8	100.4	95.1	106.7	106.3	102.9
6.68	41.51	97	98.4	92.7	102.8	99.8	99.8
6.29	25.21	99.4	94	99.8	102	99.9	106.8
6.1	25.79	101.2	98.9	99.6	102.4	96.9	97.9
5.06	25.15	111.1	107.9	109.3	61.5	59	62.8
7.8	23.57	95.3	94.8	97	96.2	97.1	98.8
5.74	23.43	101.3	103	97.2	97.5	97.4	96.4
6.21	24.43	98.2	95.4	101.8	101.9	97	103.8
7.47	24.37	105	101.4	102.6	103	102.3	107
7.49	31.6	101.7	100.3	104.1	109.2	110	110.9
6.96	22.73	93.2	87.8	88	103.9	106.2	105.6
6.25	25.11	100	100.3	100.4	98.6	98.4	98.3
6.24	19.99	103.8	97.6	96	103.5	107.3	102.4
6.54	23.54	87.9	83.3	89	107.9	111.8	106.9
6.55	24.14	102.6	95.1	100.2	94.2	90.1	95.1
11.47	66.31	102.6	100.9	97.4	105.3	107.8	102.8
8.56	25.51	99.9	106.5	107.2	109.3	102.6	106.6
5.88	23.21	90	89.8	93.6	106.7	104.2	112.9
11.59	28.59	101.9	98.1	104.5	111.7	106	107.9
6.55	29	110.2	106	107.3	88.6	89	92.2
8.4	22.53	94.7	102.1	97.8	121.8	120.7	119.2
8.12	36.26	102.3	100	98.3	105.6	104.3	102.1
7.31	21.38	96.2	99.5	100.1	102.8	99.4	111.6
5.2	21.76	105.7	99.3	98.2	98.1	97.2	100.7
6.8	24.62	95.3	97.3	97.2	93.6	93.7	96.5
6.37	20.71	94.2	96.2	97	107.4	99	111.5
5.74	18.92	101	99.7	99.1	103.7	105.5	108.8
9.13	26.05	98.9	97.8	99.8	112.5	108.7	107.8
9.45	21.81	111.2	109.7	111.9	120.4	118.8	117.6
8.72	36.73	63.1	60.2	58.7	112	110.2	113
5.77	26.54	100.3	103.1	101.7	99.4	99.3	100.7
5.76	29.46	101.5	106	102	100.1	101.1	104.7
5.67	22.8	100.8	96.9	97.9	99.5	95.4	97.9
4.54	52.39	102.3	101.1	99.6	110.6	107	109.1
7.37	66.43	107.8	110.8	107.4	96.5	98.2	96.2
11	38.99	100.2	101.1	102.6	105	112.1	106.3
8.79	25.61	103.1	102	106.7	101	96.7	97
6.49	19.72	99.5	93.2	100.9	101.8	97.9	100.6

4.98	34.1	98.5	101.3	100.6	105.8	107.3	108
6.29	24.53	103.2	100	96.9	99.3	96.9	101.9
5.66	24.26	100.5	96.5	103.9	106.3	107.4	108.7
6.14	25.26	95.1	90.2	94	99.6	104.7	101.9
5.52	29.77	98	102.9	99.2	101.2	97.6	96.8
4.82	21.76	94.9	101.6	91	117.1	118.6	113.8
8.31	22.73	111.1	115.4	119.1	61.7	70	63.5
6.57	25.03	105.4	103	98.2	93.6	93.2	92.6
5.58	29.44	98.6	100.5	96.5	101.7	104.5	110.9
4.92	19.59	104.3	98.2	100.6	107.3	100.7	109.2
8.79	33.11	98.3	96.9	94	104.7	101	101.5
7.14	22.4	104.5	99.4	100.5	104.7	102.4	105.5
6.27	22.99	102.5	106.2	104.5	95.7	97.3	97.7
6.16	44.92	105.9	106.2	105.3	89.4	88.8	89.2
6.15	21.42	90.1	96.1	93.4	107.1	100.5	104.2
8.47	38.95	98.9	95.3	93.2	103	102.8	99.3
7.11	33.55	99.4	100.7	100.1	98	101.8	99.8
6.44	26.8	97.1	101.1	98.9	101.2	98.4	100.2
7.88	23.58	103.8	100.5	103.1	84.1	90.8	91.9
10.01	29.44	100.4	100.9	107.2	111.9	110.9	109.6
7.28	50.3	68	67.3	69.6	131	125.4	124
10.23	19.04	102.2	93.9	102.5	103.8	115.5	109.3
8.47	27.95	105.7	108	105.9	107.2	109.2	108.1
5.76	19	104.7	98	104.9	98.2	95.5	97.1
9.83	29.12	102.5	96.4	104	109.6	108.8	110.1
8.51	22.12	109.5	102.3	107.2	98.6	101.7	104.3
7.03	29.74	129.7	132.7	128.3	50.8	50.8	50.2
4.58	36.43	104.9	99.3	98.9	106.6	104.7	103.8
10.1	22.69	102.9	102	100.1	108	110	105.6
5.62	23.53	102.9	104.5	99.1	100.1	97.8	100.6
8	22.9	105.2	109.4	104.1	106	99.4	97.1
5.94	18.27	98.6	97.8	101.6	93.9	101.7	113.1
5.55	20.65	106.6	103.1	97.6	98.1	105.3	102.9
8.63	20.34	105.3	99.5	100.5	101.5	101.6	110.5
11.66	47.99	98.1	100.4	103.3	126.2	124.3	123
8.82	20.01	104.5	106.4	106.7	95.5	94.7	91.1
6.4	30.43	102.9	93.9	88.7	105	88.7	108.3
8.76	30.85	98.6	106.4	104	99.7	93.1	93.9
5.83	36.6	102.7	102.5	101.6	98.7	97.1	98.1
8.15	48.1	88.7	127.4	109.5	82.9	76.3	108.3
7.85	26	106	106.1	104.2	112.4	110.3	112
6.68	24.57	96.7	96.4	97.6	101.9	99.7	97.6
10.24	23.94	114.2	110.8	98.9	88.5	95.7	97.3
8.19	26.99	99.2	99.2	101.4	96.9	94.2	96.2
7.14	18.44	101.4	96.6	98	104.2	106.6	103.3
8.7	32.62	90.6	95.3	95.5	92.8	96.8	97.6
7.46	23.06	96.1	95.3	98.7	102.1	105.3	101.7
5.01	43.23	140.9	145.7	138.8	71	63.9	69.4
9.09	33.85	97.8	90.2	93.6	87	87.5	87.3
5.83	20.13	105.1	98.3	102.1	101	100.6	104.6
6.02	24.18	96.5	96.9	95.5	96.2	95.6	100.6

8.27	32.55	103.5	101.5	105.4	109.2	101.2	105.7
7.34	25.85	105.5	98.7	103.1	95.8	97.3	97.6
11.06	51.95	102.2	99.6	104.9	99.7	109.4	103.8
5.26	22.06	100	102	100.9	94.8	94	95.3
6.38	13.37	101	101.9	111.9	105.7	107.9	105.4
6.76	24.93	100.6	97.8	96.4	101.9	100.2	101.3
5.5	29.34	97.3	96.8	95.6	117.2	115.3	114.5
6.62	22.56	99	108.1	99.6	101	100.5	99.4
6.34	25.53	94.1	90.7	95.8	102.7	104	110
5.91	32.43	93.2	97.5	92.4	100.6	99.5	100.6
6.7	22.52	95.2	89.1	97.3	95.1	102	101.8
5.77	24.18	85.5	83.2	86	101.4	101.6	100.6
6.81	33.09	103.8	100.4	99.4	99.7	99.6	97.1
5.49	21.87	102.7	106.8	109.3	99.4	99.4	98.9
6.46	25.2	106.4	99.2	94.1	94.7	96.3	100
8.25	18.77	97.7	93.4	97.4	101.8	106.5	113
7.11	26.9	97.6	102.1	94.5	92.5	93.1	93.6
9.6	26.01	97	98.5	99.5	107.2	101.3	104.3
6.55	33.23	99.6	106.6	101	96.8	99.8	100
6.35	18.14	95.4	97.2	93.8	101.2	95.1	93.5
6.83	22.22	111.6	103.2	101.5	104.8	105	108.1
4.78	27.93	103.8	100.8	95.8	103.1	102	103.2
6.74	26.67	96.9	95.5	94.5	103.8	99.6	101.2
8.66	27.71	100.2	97.4	97.6	89.7	97.9	116.5
7.39	23.02	101.9	108.4	108.5	97.2	101.7	100.3
5.47	28.66	102.6	100	100.7	97.5	97.9	99.1
7.55	18.29	106.5	106.6	105.5	100.9	99.6	98.9
9.51	22.97	102.3	100.2	101.4	105.5	105.2	102.6
6.62	28.78	150.9	152.2	152.1	62.2	63.5	64.1
5.94	21.18	85	82.6	77.9	87	89.3	87.5
5.97	37.29	102.9	103.5	103.2	99.2	99.6	99
5.21	19.93	94.8	98.2	98.1	97.8	96.9	102.4
5.07	20.31	96.7	87.7	112.5	94.9	115.2	101.1
5.22	24.39	93.1	94.3	99.9	103.7	130.5	109.3
4.49	33.5	105.1	103.2	101.4	98.5	102.4	98.6
7.25	22.32	100.5	95.3	101.8	109.5	105.9	108.7
7.99	19.9	112.7	115.1	103.1	111.8	109.7	108.3
6.86	35.29	89.1	92.3	90.4	99.2	97.6	97.6
7.99	23.27	100.4	101.2	101.5	95	95.4	97.4
8.75	25.48	100.7	95.8	100.2	108.9	119.4	111.1
7.56	39.14	98.4	103.2	102.8	91.4	93.5	95.1
7.56	23.37	95.1	98.4	96.4	97.7	102.5	98.4
8.5	29.73	69.1	67.5	65.7	88.6	90.1	86.9
5.58	22.22	98.1	99.7	97.6	99	102.9	95.9
5.97	22.01	104.2	102.3	100.3	109.2	111.8	108.6
5.66	31.93	103.7	100.8	111.2	95.3	110.5	102.3
6.92	26.72	90.1	95.1	99.6	98.3	96.1	99.1
7.39	22.68	100.2	99.3	100.9	100	100.6	96.4
7.43	41.93	100.4	93.4	94.6	101.1	105	101.5
7.68	18.22	97.2	100	96.4	95.1	94.1	99.3
6.55	32.01	97.6	98.6	91.3	103.5	100.4	104.5

6.92	25.85	103.8	105.1	102.8	93.4	92.2	97.1
10.89	43.85	117.1	114.9	118.8	106.1	99.8	103.7
6.9	19.6	100	93.4	98.4	100	96.4	104.8
4.83	19.82	106.7	104.4	103.7	95.7	94.6	97.6
8.65	17.54	106.3	117.8	111.4	100	98.5	100.8
6.28	30.1	94.7	94.1	95	98.8	99	101.4
8.5	24.61	101.1	101.4	100.6	96.9	98.1	102.9
7.21	26.2	99.4	99.9	96.9	100.8	112.8	104.3
5.01	23.88	102.4	106.2	103.6	104.9	101.6	101.3
7.42	16.38	100.8	96.7	103	112.3	106.2	101.3
9.8	54.17	102.9	102.4	101.9	102.6	102.8	100
5.86	23.29	111.2	112.4	107.3	115.7	115.9	115.1
6.34	27.59	102.5	101.9	98.3	103.3	98.3	99
8.5	18.39	89.7	95	95.8	105.7	106.8	109.9
5.53	22.49	104	98.5	99.9	98.5	100.7	100.6
6.99	20.08	94	94.6	97.5	100.4	100.2	106.3
6.52	28.99	97.7	94.5	98.7	93.1	92.4	101.1
5.52	35.24	101.8	102.2	101.1	99.7	96	97.1
7.18	26.13	94.2	96	92.7	99.3	103.5	98.7
4.78	23.08	104.3	102.8	94.7	103.7	98.3	104.1
5.31	21.63	94.7	98.4	98.4	108.9	109.6	109.1
8	13.98	103.9	101.1	107.4	99.3	111.3	103.9
9.31	34.03	96	94.1	97.6	111	94.7	113.1
8.62	27.27	109.5	105.9	106.7	105.4	104.6	103.5
7.06	31.51	97.7	99.6	100.3	97.8	95.9	96.7
8.51	22.46	107	96.9	95.8	96.8	95.8	100.3
9.32	25.21	98.2	97.1	103	96	94.8	90.5
9.41	27.7	110.6	111.3	112.8	88.4	91.4	93.9
8.53	18.46	108.9	112.6	110	89.2	94.5	92.8
7.64	16.32	74.2	74.4	75.2	105.3	98.4	102.4
5.49	19.97	97.9	96.9	97.5	95.9	92.6	99.2
6.83	19.83	108.6	96.2	102.5	93.4	106.7	95.8
8.97	24.3	98.9	105.8	101.2	101.6	105.6	107.4
7.75	24.81	100.6	100.1	100.2	98.8	96.9	96.4
5.53	20.35	80.6	78.7	85.7	126.7	122.1	121.1
5.76	19.1	97.9	102.7	103.5	110.6	103.9	109
6.55	22.85	99.6	96.7	97.2	103.5	101.3	104.3
8	21.29	104.3	104.5	103.4	96.9	103.4	96.4
6.3	19.81	96.7	95.3	94.7	100.3	98.5	96.2
6.89	23.46	102.5	102.6	93.9	98.5	99.5	97.6
5.22	28.05	98.2	88.6	88.9	105.7	106.5	111.4
9.35	24.06	103.4	104.9	107.7	97.7	98	100.1
4.73	21.03	101.9	93.4	99.3	106.4	107.3	106
7.25	26.61	102.3	100.6	98	104.7	100.9	105.4
5.62	25	98	100.6	98.8	105.2	103	102.4
7.94	21.34	100.3	97.4	100.9	91.4	92.7	98.1
6.34	21.29	103.3	99.4	101.4	94.3	89.3	89.9
5.02	20.73	91.6	87.8	95.1	113	121.8	112.1
6.42	19.83	108.1	98.6	96.3	102.1	108.7	100.6
4.34	23.26	106.2	102.6	98.6	105.5	103.4	107.2
5.66	47.01	94.7	93.5	96.4	105.3	103.7	104.2

5.12	20.95	98.8	95.2	98.5	101.8	99.1	106.6
9.7	24.66	106.3	83.9	121.6	160.8	176.9	153.8
6.04	33.63	105.3	106.2	106.1	104.2	103.4	107.7
9.35	30.38	100.6	100.3	98.1	91	97.2	93.8
5.08	28.29	95.3	93.2	92.2	103.1	103.6	105.9
6.09	20.97	102.8	104	112.4	106.5	111.5	105.1
6.55	18.65	96.1	98.7	101.6	102.1	95.9	100.6
5.47	19.61	105.1	114.2	115.9	109.3	114.9	112.5
6.43	39.65	93.4	95.2	95.6	103	105	105.6
6.46	39.71	148.2	146.1	146.7	42.9	43.7	43.9
5.21	22.76	103.2	94.1	95	109.8	104.2	105.4
9.19	18.8	100.1	98.4	106.2	94.8	97.5	101.6
5.55	18.92	97.5	95.3	97.9	89.8	85.9	91.9
9.09	20.02	98.3	103.8	105.2	102.5	96.5	96
4.51	51.59	98.7	99.8	100.1	104.6	102.7	105.5
10.04	47.05	105.2	105.1	107.8	104.8	105.5	103.6
6.81	21.77	94.5	93.2	101.2	102.6	93.7	107.1
8.69	27.67	100	101.9	100.9	104.4	103.9	101.1
5.83	25.95	97.1	104.6	102.7	93.2	94.5	96
11.43	30.04	105.8	104.4	104.9	106.5	109.4	105.6
9.14	25.29	98.8	97.2	96.5	99.6	102.2	101.6
11.08	31.34	111.7	106	102.4	112.9	108.8	103.2
8.53	22.6	123.7	129.6	123.9	94.5	94.3	93.4
5.55	30.51	102.8	102.1	99	102.3	105.2	106.5
5.24	18.46	100.7	89.7	91.6	107.9	104.5	107.9
6.83	23.66	105.1	113.1	108.9	109.1	107.3	104.3
6.2	21.55	98.4	91.3	106.8	99.2	95.4	100.2
7.08	25.9	106.7	101.1	100.8	110.1	114.1	110.6
5.01	19.9	110.8	100.6	127.1	110.7	107.2	111.6
5.41	18.61	107.5	104.5	116	93.2	97.8	94.6
8.22	43.17	111.1	109.9	106.2	90.2	96	94.7
6.25	58.36	111.2	116.3	113.9	94.4	92.8	92.3
8.27	26.45	102	106.9	102.3	97.1	96.7	96.6
8.15	16.93	104.9	95	103.3	84.3	80.6	88.8
7.99	31.7	101.8	98.9	104.2	100.2	96.1	94.7
4.86	25.21	99.5	99.4	99.6	117.2	114.6	111.8
6.9	26.56	106.1	95.1	104.5	97.7	91.1	104.1
7.4	22.15	95.7	92.1	100.8	99.9	101.6	100.9
6.3	18.7	100.7	103.5	96.3	104.6	99.4	108.7
7.55	20.71	94.8	93.2	111.3	105.5	98.9	102.1
6.6	18.61	98.7	101	107.4	108.9	103.3	103.3
7.96	20.12	94.9	96.1	96.8	102.2	102.8	105.9
6.54	17.62	94.1	88.6	94.3	92.8	99.9	88.6
8.62	22.64	108.2	101.7	98.7	101.6	103.7	98
6.79	24.44	98.9	95.4	105.2	105.3	104.8	103.2
8.94	29.28	94.4	96.4	96.5	108.7	105.7	102.2
6.32	19.54	97.1	97.6	96.2	97.1	89.4	90.1
8.35	34.34	107.3	100	104.4	96.3	101.4	97.6
7.72	21.88	102	100.9	100.7	101.4	100.9	100.5
8.78	27.28	109.5	107.5	108.2	106.6	104.9	106.1
6.79	18.82	97.1	96.1	95.5	94.7	89.9	92.8

6.65	24.33	86.7	105	107.2	90.3	97.7	99.2
6.65	21.08	90	85.3	85.6	103.6	101.6	97.6
10.43	44.08	99.3	98	100.1	104.2	100.9	104.7
6.62	23.37	95	99.7	97.9	107.4	109.7	111.2
5.57	20.49	98.5	94.4	91.2	103.2	90.9	106.2
5.06	18.03	90.8	88.6	89.6	104.5	109	109.2
8.12	23.26	102.5	100.7	99.8	106	114.6	113
4.81	30.26	96.7	101.9	104.9	106.7	102.6	97
6.77	27.91	96.3	105.7	102.4	104.2	100.3	95.7
6.42	12.41	101	97.4	96.3	106.3	109.2	99.9
5.82	25.29	104	99.7	95.9	93.9	99.9	94.7
8.63	20.6	93.4	100.1	99.8	96.5	99.1	98.2
4.92	49.75	96.7	95.6	95.9	102.3	102.7	100.8
5.63	21.46	104.7	105.2	104	103	96.3	98.5
5.44	16.35	93.2	96.3	89.3	106.9	108.5	112.2
9.36	21.46	97.3	96.5	95.5	94.2	91.3	96.4
5.21	22.64	94.2	93.9	95.1	100.2	104.9	103
7.09	19.87	89.6	99.7	96	97.9	101.4	101.4
6.37	22.77	92.9	95.3	94.8	100.2	105.6	109.3
4.69	22.99	69.9	62.6	63.6	117.5	134.9	127.7
7.72	24.59	106.9	106.5	111.7	92.5	91.6	88.6
5.43	21.83	98.2	100.7	100.2	97.2	94.9	100.3
5.05	21.59	99.4	102.1	93.8	99.3	96.1	96.4
8.38	39.64	102.2	100.8	98.2	99.7	99.2	100.2
6.39	23.13	132.5	124	117.8	84.9	82.7	82.9
8.48	30.05	109.1	114.9	79.4	119.8	80	119.6
10.99	49.48	104	104	103.4	102.6	102	100.2
6.98	21.76	104.1	101.7	99.8	99.5	105.5	102.5
10.02	19.05	101.6	103.2	108	101.2	106.9	117.3
6.81	20.77	111.3	111	112.1	82.6	83.5	80.5
9.42	16.87	100.1	102.6	108.9	103.8	95.8	97.1
5.39	23.01	100.6	91.1	96.7	98.8	99.2	99
6.84	22.03	101	96.4	96.4	98.6	104	105.4
6.34	19.16	101.2	102.6	99.4	97.4	97.4	98.7
5.69	18.88	101.5	97.2	94.9	99.4	101	103.5
8	19.4	97.7	99.2	104.6	104.3	110	104.3
9.47	24.48	105.8	110	115.5	124.4	117.5	121.1
9.45	22.16	109.4	103	110.3	100.1	101.6	104.3
9.16	29.09	111.7	125.9	90.9	103.9	102.4	107.5
5.9	15.58	101.9	99.7	100.1	121.5	123.1	112.7
6.99	17.44	95.7	103.7	96.3	92.4	87.8	86.5
8.06	18.59	102.2	97.9	101.5	103.3	106.1	107.1
6.77	20.95	95.4	103.6	95.2	99.5	95.4	103
7.75	25.64	98.9	98.5	103.2	101.2	98.6	98.4
7.39	17.9	99.8	96.9	101.4	106.9	104.1	117
6.74	21.5	93.9	103.8	99.2	108.4	102	103.1
7.15	17.84	103.2	95.5	95.3	102.8	109.8	106.2
6.73	19.06	90.8	91.6	95.6	101.7	107.7	104.9
4.83	36.32	97.6	96.8	100.4	98.2	99.8	99.5
5.43	19.87	96.2	101.2	108.5	106.3	106.7	105.1
8.82	25.33	109.9	103.6	105.9	96.4	95.2	91.5

5.1	24.15	99.5	98.8	103.4	100.2	102.4	103
4.93	23.31	94.6	101.1	97.1	108.3	109.6	109.8
5.21	21.48	98	100	93.7	102.3	104.2	93.7
5.36	16.59	105	85.1	103.7	110.7	86.5	112.4
6.65	18.95	99.3	99	102.9	85.6	92.5	88.2
10.54	41.65	100.5	101	105.9	104.1	104.9	105.4
7.37	26.04	94.7	101.4	102	100.3	103	105.3
4.46	22.42	98.8	100.9	99.4	101.7	96	99.5
8.63	21.21	95	94	99.8	120.3	112.1	114.2
9.23	23.07	101.9	107	103.4	102.5	96.9	97.4
9.64	35.26	100.5	99.9	101.1	98.2	99.2	103.6
4.72	20.7	115.2	125.3	130.8	109.2	106.4	114.4
5.45	19.6	103.2	101	104.2	97.7	95.2	103.1
9.06	23.98	96.3	98.6	92.8	80	78.3	77
5.17	20.11	94.3	92.4	96.5	104.2	116.6	118.9
4.86	17.98	103.5	97.9	106	103.5	101.8	100.9
7.43	20.91	93.7	101	95.9	100.2	104.3	108.5
5.4	22.25	101.4	99.7	100.4	98.7	95.6	96
5.78	27.69	101.5	99.3	104.2	106.3	108.3	108.1
8.72	20.46	95.8	94.4	95.6	102.2	104.6	99.9
7.9	18.89	99.7	100.4	101.7	104.4	107.1	108.3
10.99	47.96	96.9	101.5	94.5	152.9	171.4	158.5
8.25	17.12	95.4	92.7	95.3	116.4	107.6	111.1
5.8	18.8	96.6	105.5	101.4	106.3	103.7	105.2
6.77	18.7	103.6	107.2	100.2	96.1	100.4	97.4
6.28	22.07	101.1	100.6	99.4	116.9	113.3	115.4
7.87	23.94	95.4	97	85.4	104.3	98.6	91.1
8.19	22.33	97.3	94.1	99.6	100.5	103	99.8
9.06	19.2	97.8	100.7	94.8	114.4	112.6	111.4
5.31	35.68	92.4	93.4	92.6	105	101.6	102.5
4.97	18.48	111	96.8	98.6	102.9	104.1	102
4.7	19.69	80.5	79.1	79	108.4	100.8	106.2
9.64	20.01	100.6	100.3	97.8	109.5	109.9	114.6
6.55	29.48	103.6	98.9	96.7	83.8	79.4	85
8.82	24.9	102.2	105.5	105	112	110.1	109.2
5.68	20.97	100.8	102.3	98.8	96.6	91.5	89.5
4.96	19.46	90.8	93	96.7	105.6	104.1	106.3
7.97	23.2	94	90.8	94.1	103.4	108.6	106.4
11.65	37.35	103.9	102.6	105	108.3	105	106.5
8.79	25.73	105.3	106.2	91.9	105.4	97.3	104.3
7.96	20.05	96.7	94.5	89	102.4	97.8	101
9.17	18.26	97.4	96.6	103.5	108.4	104.8	106.1
7.72	23.24	92.4	95.6	90.5	89.9	89.3	92.4
8.24	35.32	100.3	99.5	101.8	104.2	104	106.2
6.65	25.7	100.6	100.7	97.3	97.6	99.8	104.8
7.52	19.55	108.7	108.2	115.6	105	109.5	104.8
5.41	19.44	100.5	100.4	95.1	100.3	95.3	96.9
6.43	15.4	100.5	102.7	99.1	108.1	102	116.9
5.39	21.54	99.3	95.9	96.4	104	101.6	104.4
9.22	14.69	101.5	96	98.8	113.3	101.9	99.1
6.18	10.23	93.1	88.2	94.4	103.7	88.4	102.6

9.1	31.04	101.9	102.1	104.4	94.4	89.2	91.6
10.08	21.68	98	103.4	103.8	98.3	100.9	97.7
6.35	11.47	103.7	100	97.8	100.1	103.3	106
5.85	20.04	94.6	99.5	97.6	99.3	106	99.6
5.94	18.21	95.5	101.8	102.3	111.3	113.7	109.7
6.93	13.12	107.5	90.6	104.8	97.4	92.9	103.4
8.31	22.83	95.5	112.8	108.5	102.5	100.3	109.9
6.92	23.67	98.4	98.6	99.3	108.3	100.3	104.2
4.92	20.41	102.6	90.7	95.6	104.8	106.2	111.7
5.47	25.58	107.8	97.1	103.2	96.5	94.4	97.6
6.58	16.46	98	102.7	97.7	97.9	96.1	98.3
6.73	22.7	101.2	102.2	98.5	105.2	104.9	104
4.92	19.74	97.3	100.4	97.8	104.3	102.2	102.3
7.21	17.86	97.9	100.9	101.9	105.4	103.1	105.5
7.72	24.99	109.3	112.7	112.8	81.7	77.6	78.9
8.31	20.18	94.4	99.3	99.4	105.9	97.9	101.1
6.86	35.92	87.5	82.8	84.6	106.4	103.5	107.2
5.64	25.72	99.6	100.4	100.1	101.9	97.1	100.8
7.97	25.28	105.3	105.2	106.3	109.3	105.1	108
8.57	18.28	100.9	91.2	95.8	105.9	111.5	106.2
8.05	24.65	86.1	86.9	81.5	90	87.4	86.8
5.08	17.05	100.3	97.9	97.7	98.1	97.2	102.2
6.07	16.05	94.6	101.6	104.6	108.9	112.3	110.6
6.76	16.93	97.7	90.8	97.4	106.2	105.3	107.6
5.62	29.12	98.8	97.4	97.8	103.7	99.7	102
10.1	43.04	104.2	104.2	103.6	103.2	102.5	100.7
6.35	26.21	99.2	105.6	96.8	97.6	100.7	93.2
9.89	17.45	101.7	107.1	105.8	113.1	111.2	108.5
5.1	15.9	95.8	97.3	100.4	101.3	98.3	100.6
7.33	13.37	95.4	101.8	94.9	104.3	100.9	102.5
6.55	16.8	95.7	99.2	98	101.6	102.9	103.8
6.37	17.49	98.9	93.5	99.7	101.5	103.4	110
7.88	15.76	112.8	109	108	87.9	88.8	86.7
6.64	19.28	99.4	98.5	101.2	112.5	107.2	112.8
9.1	17.41	102.6	100.5	105.5	103	104.6	110.8
6.43	20.26	100.1	101	101.7	109.9	99.3	107.6
10.11	11.47	103.6	110	106.6	112.4	116.7	119.3
6.33	13.19	103.7	98.7	97.3	96.5	107.4	95.9
7.09	17.28	114.7	102.7	104.8	99	94.4	96
5.52	18.69	98.6	98.4	94	79.3	78	81
5.69	27.24	99.2	98.6	102.4	98.9	98.9	101.6
6.02	14.2	112.8	111.4	116.6	109.8	113.4	109.8
9.01	18.99	106.7	98.6	96.8	109.1	108.5	102.5
6.18	16.71	101.5	92.6	91.9	85.8	101.9	101.5
7.25	15.86	104	91.7	96.7	89.5	85.1	95.9
9.28	17.06	100.3	99.7	106.5	105.6	112.7	113.3
6.95	22.23	97.4	98.4	103.6	93.5	96.6	100.8
6.02	22.26	101.4	101.6	105.4	96	96.3	91
7.11	24.26	106.1	107.6	105.6	86.2	87.7	89.6
5.92	21.99	100.5	98.6	100.9	96.1	93.3	96.3
4.83	22.24	109.2	103.1	101.6	59.4	65.9	60.7

8.78	22.96	94.2	108.5	90.3	90.7	96.8	98.3
9.33	18.33	104	104.6	100	100.7	103.1	107.9
8.05	17.63	96.4	94	94.2	97.3	95.3	98.4
6.79	15.78	101.2	99.4	98.4	104.7	112.7	102.3
8.81	30.46	150	98.1	118.4	104.5	92.8	120.6
6.48	17.84	94.5	98	98.2	94.5	102.8	94.9
5.36	19.48	100.8	97.8	100.1	100	98.4	101
5.22	19.5	92.7	95.8	105.6	100.7	101.2	96.6
6.7	23.27	104.8	124.2	103.7	106.7	102.7	119.9
5.07	23.02	96.3	97.3	99.3	109.2	103.3	102.9
9.45	14.97	105.6	98.6	105.4	109.8	109.8	110.6
9.64	16.54	107.5	106.3	113.1	107	129	114.7
7.3	12.3	101.3	104.1	103.5	107.6	106.7	115.3
6.99	31.45	100.8	106.1	107.6	91.6	94.4	96.8
6.8	17.37	93.5	100.6	107.7	110.9	110.5	109.8
6.46	26.92	99.4	97.6	99.5	102.4	105.5	104.3
7.2	14.61	99.6	83.7	124.5	101.1	97.3	133.3
4.72	27.21	107.3	98.4	100.9	105.7	106.2	105.3
4.81	23.7	100.6	100.6	103	100.3	97.6	107.2
9.36	23.28	105.4	98.7	96.1	102.7	103.5	106.2
6.01	18.68	121.8	115.9	125.9	92	99.9	98.3
5.94	12.28	134.6	91.3	87.8	94.8	123.3	95.1
11.62	26.07	102.6	111.2	100.3	99.6	103.6	105.6
6.02	31.49	99.4	98.5	98.2	94.7	94.2	96.7
5	12.58	103.1	102	100.5	107.3	99	102.2
6.8	15.28	104.3	107.6	102.3	103.6	100.8	95.7
7.05	14.89	116	101.6	114.7	88.2	98.8	93.2
6.15	25.82	107.6	95.4	101.2	101.3	102.5	106.8
6.61	15.34	103.7	95.5	97.1	94.6	93.5	97.1
7.15	16.24	88	92.1	88.6	109.9	102	106
6.64	15.73	100.4	94	99.4	100.7	100.3	106.8
7.18	17.31	104.6	104.5	105.4	106.5	107.9	102.8
6.07	23.82	98	97	96.6	101.2	100.7	99.2
9.36	19.32	99	92.6	103.5	102	108.5	104
7.2	12.96	92.5	98.9	95	98.1	121.3	116.8
4.91	19.11	104.3	93.5	108.6	101.8	116.8	99.8
7.14	15.5	110.6	108.4	117.9	80.4	79.5	83.6
8.27	22.81	103.2	94.6	90.7	95.1	102.1	100.7
5.38	26.72	95.6	98.7	102.6	98	97.4	97.8
8.69	15.28	100.1	91.2	97.8	108	100	104.1
6.43	13.73	86.9	83.9	81	103.9	102.4	99.6
8.16	19.86	101	95.6	94.8	105.7	94.5	102.4
5.36	16.79	92.6	97.7	96.4	106.5	99	102.7
8.73	17.87	97.4	91.1	101.4	102.5	105.1	98.6
8.27	16.54	101	100.5	97.2	104.1	94.4	95.2
9.61	34.6	115.7	98.9	93.2	97.6	103.2	103.2
8.4	15	92.8	96.9	95.7	113.3	107.1	114.6
7.11	21.55	98.4	88.6	106.4	96.4	115.2	103.2
6.99	16.27	110.3	119.3	114.9	102.8	107.1	101.5
5.02	22.97	95	102	94.7	105.4	102.3	103.2
6.55	14.23	99.5	105.8	101.5	99.6	100.8	112.8

7.09	19.25	105.3	102.2	104.3	97.3	107.2	97.6
10.65	37.49	111.2	102.3	95.5	100.6	106.8	97.1
5.86	17.6	108	88.7	97.1	92.2	103.6	98.2
4.34	17.48	96.8	99.4	94	107.9	113.2	105.6
8.47	18.63	106.4	102.5	103.5	99	104.7	108
9.92	20.82	103.4	102.7	102.2	112.6	109.6	108.9
7.34	17.11	99.3	107.1	106.3	114	105.9	103.7
7.23	17.81	86.8	89.1	89.9	116	119.7	119.2
9.72	18.25	102	104.6	108.4	104.9	101	107.7
4.67	34.36	102	101.2	104.2	127.8	126.1	123.4
7.46	22.7	97.5	99	96.2	105.1	102	104
4.93	19.79	86.8	95.8	90.8	113.7	108.2	111.7
6.05	24.09	96.1	95.9	98	101.2	102.1	105.8
5.82	15.09	98.5	100.2	102.1	98.1	105	103
4.44	15.13	100.5	106	101.1	97.3	98	92.8
6.55	19.27	100.6	97.6	96.3	94.6	96.9	95.5
6.2	17.42	100.3	98.1	96.7	98.6	97.7	98.1
9.23	20.75	100.6	100	103.3	106.8	100.4	104.3
6.92	16.27	93.1	89.6	89	98	104.8	113.8
5.8	20.37	104.5	100.5	102.3	97.1	99.1	93.2
8.07	18.96	108	106.8	107.5	97.7	100.2	102
5.02	18.31	111	107.5	107.5	118.5	103.6	114.9
7.28	20.67	95.6	95.4	93.8	93.7	97.9	97.7
5.76	14.69	101.7	89.3	98.9	96.2	92.3	93.5
4.98	19.36	106.4	94.4	95.8	110.9	101.6	104
8.29	30.57	102.9	97.6	101.5	102	96	105.6
8.57	15.65	108	129.9	82	99.6	102.3	86.7
6.84	19.81	104.7	99.8	98.1	102.8	100.1	104.2
5.45	19.59	99.4	99.5	102.9	88.9	100.7	99.7
5.35	21.8	101.6	102	108	101.7	96.6	99.6
6.73	21.86	99.8	97.3	105.5	84.6	89.1	86.6
5.25	15.79	106.8	105.9	100.5	96.7	97	97.5
7.37	15.68	95.2	100.2	98.1	101.1	104.1	97.3
6.87	10.28	94.4	99.4	86.5	99.1	99.6	96.6
7.06	14.8	90.9	101.9	97.8	96.9	104.3	97.6
7.31	17.68	104.1	89.5	109.7	109.4	79.8	117.6
6.38	17.52	99.1	90.8	98.4	105.6	103.4	104
6.93	14.58	99.1	105.1	107.5	96.1	96.3	103.5
5.22	16.71	99.8	100.5	99.6	100.3	103.2	97.8
8.46	27.56	101.6	105.7	102.5	103.7	104.7	100.5
6.64	18.12	106.8	102.4	100.1	99.8	93.1	94.1
8.09	17.65	102.6	94.9	102.9	97.4	93.2	105.8
5.06	19.12	83.9	87.2	87.4	96.4	92.2	171
4.81	22	106.8	101.8	104.3	96.3	96.8	95.6
5.05	19.39	101.7	95.6	102.1	105.6	110.9	110.8
6.43	22.8	92.5	95.4	97.4	99.5	97.9	105
9.16	21.61	99.8	97	93.9	113.6	111.9	110.1
7.28	18.88	100.9	106.7	103.2	111.4	103.5	106
5.16	18.8	95.5	100.6	97.8	103.3	97	98.9
8.51	16.9	100.6	98.9	97.7	93.5	98.4	97.6
7.33	16.07	95.1	95.7	93.1	101.5	106.1	102.6

7.02	16.62	101.8	103.4	102.5	102.4	100.4	107.2
6.32	19.02	111.1	100.3	103.2	104.9	117.6	110.5
7.37	17.41	99.9	104.2	104	98.2	105.1	100.2
5.66	15.87	103.9	99.4	106.8	99.3	96.2	94
5.76	15.86	94.9	100.2	101.3	96.7	96.7	97.3
7.12	20.91	80	74.9	74.8	120.7	118.1	120.4
5.1	18.85	103.7	97.7	102.3	101.1	98.5	98.7
7.66	18.01	93.6	96.9	93.1	98	101.2	100
6.13	11.8	96.2	99.4	99.5	110.6	106.2	110.8
6.96	20.03	99.5	97.3	98.3	99.2	103	107.7
10.1	46.01	91.9	82.1	86.2	95.2	91.4	97.7
5.82	13.24	102.7	97.7	110.6	102.2	102.4	103.9
11.19	22.27	106.5	95.1	101	105.2	105.9	107.5
7.06	15.94	105.7	102.4	102	102.1	108.9	117.4
6.96	18.86	95.1	90.3	98.7	98.9	93.5	101.9
6.44	23.08	100.2	103.3	100.8	105.6	110.6	107.2
6.84	20.02	88.3	92.2	92.4	94.8	95.3	94.8
8.15	18.39	98.4	92.3	87.3	109	110.8	116.9
7.99	19.73	99.9	98.5	96	95.1	91.6	90.6
6.19	14.61	114.9	123.9	112.3	74.1	67.7	76.4
5.69	17.41	104.9	102.4	104.2	109.7	107.1	107.9
6.35	17.47	107.5	106.4	105.3	97.9	93.5	96.5
11.27	55.37	106.2	114.6	116.7	104.8	102.2	101.4
8.53	14.4	92.5	91.2	93.4	83.1	82.6	85.2
4.94	19.44	100.8	92	97	98.2	99.4	101.4
9.45	16.1	100.2	104.9	102.3	108.5	102.7	104.8
5.27	17.37	100.6	93.3	92.6	98.9	104.9	104.7
8.72	14.84	107	108.4	105.9	97.1	100.3	99.4
6.73	24.23	99.5	100.6	94.2	101.1	104.9	101.3
5.78	13.6	96.5	90.3	92.3	114.1	133.2	110.3
5.41	23.1	99.2	97.8	94.5	100.7	102.2	102.9
6.64	14.64	97.4	99.5	108.4	107.3	94.3	103.5
5.44	11.97	96.8	100	100.6	95.7	96.2	92.6
5.54	19.6	102.3	99.8	103.9	105.4	110.1	106.5
6.14	18.6	91.8	90.9	87.8	86.9	84.7	91.5
6.61	23.18	104.9	98	106.7	101.9	101.6	102.1
8.54	22.18	94	95.7	97	100.4	97.2	98.4
4.88	22.87	99.9	98.8	96.5	105.7	99	101.3
5.05	14.26	100.8	105.6	103.9	105.3	100.6	99.9
5.99	19.51	100.9	98.5	99.5	101.6	104.2	107.2
7.05	22.44	102.1	95.1	101.8	96.1	93.7	95
11.84	20.88	101.6	98.4	105	117.3	121	121.7
6.7	15.8	96.3	91.4	107.5	96.6	91.9	98.3
8.88	18.84	106	102.4	103.6	105	108.3	104.8
7.02	19.28	89.1	97.6	100.1	106.5	106.5	106.2
9.17	25.76	102	104	104.8	111.9	104.3	113
5.49	16.74	109.2	101.1	105.3	106.4	93.8	104.5
6.8	17.56	94.7	102.2	98.4	103.2	100.5	108.9
7.05	29.05	102.4	99.3	102.3	112.4	106.1	106.6
6.93	18.17	95.7	95.5	99.6	99.5	108.4	106.2
10.26	17.18	105.1	105.2	103.4	104	105.2	103.1

4.82	16.55	95.6	104.2	101.6	95.9	91.8	88.6
5.81	17.55	92.7	94.9	90	101.6	101.9	106
5.81	14.21	101.2	93.5	95.1	103.2	98.7	98.3
4.73	16.74	91	97.3	100.2	105.5	107.9	105.3
6.64	12.68	104.8	104.6	104.5	109.8	106.6	105
5.03	17.23	102.7	99.1	102.4	102.8	100.4	101.9
6.81	16.89	113.3	89.5	96.8	122.1	102.3	118.4
7.31	15.36	121.2	125.9	104.2	85.1	83.5	81.1
5.69	21.29	107.2	102.8	100.5	105.7	104.1	104.7
6.73	24.13	97	98	99.6	104.9	100.1	94.7
4.59	26.73	93.5	90.8	100.3	104.1	104.5	106.7
6.48	17.78	107.1	97.9	97.8	87.6	93.9	87.4
7.43	17.68	103.4	102.4	99.5	94.5	97.6	94.2
7.34	9.67	102.4	98.2	99.5	111.1	106.5	106.8
4.98	14.33	101.5	95.7	95.9	92.6	103.5	91.4
7.83	28.06	86.9	84.5	87	92	92.6	91
7.31	16.95	95.1	99.9	99.6	108	93.2	98.3
9.29	24.05	106.7	102.2	99.8	110	112.1	114.1
8.1	25.24	94.7	96.3	91.6	100	98.1	98.2
6.32	15.97	91.8	95.2	94.9	104	104.4	102.5
6.86	20.67	97	96	103.2	103.9	103.8	106.4
6.42	18.14	97.3	96	97	105	98.9	106.2
6.8	15.98	93.9	94.2	90.8	102.9	97.3	95.5
6.73	15.34	96.9	97.1	93.7	114.3	109.4	107.3
4.93	20.43	97.9	97	95.8	115.6	105.5	110.9
6.62	15.35	101.9	95.9	100.9	87.2	86.5	94.8
6.07	15.7	102.2	94.4	99.5	101.4	102.6	106.3
7.42	17.67	103.4	109.3	101.6	112.2	109.4	113.2
5.64	17.29	103.9	99.6	104.2	113.3	104.9	105.3
6.18	15.53	105.2	102.6	106.2	101.7	106	111.1
9.31	34.98	92.5	93	99.6	100.2	101.3	106.3
5.07	14.72	98.2	99.8	105	94.1	98.5	100.7
6.35	14.97	97.8	99	100.7	102.5	99.4	105.5
4.92	10.15	90.1	83.6	81.2	103.2	106.1	105.9
6.65	16.93	94.9	103.4	95	98.3	103.1	102.2
4.96	15.2	96.7	100.2	85.9	106	109.3	100.8
8.46	25.34	107	102.8	106.5	104.3	100	104.5
6.7	15.24	101	95.8	97.5	103.8	102.1	102.8
7.77	15.06	99.4	105	103.3	98.4	95.2	100.5
6.61	26.78	94.7	94.9	98.7	96.4	94.7	93.6
8.24	13.15	94.2	102.2	93.2	97.3	110.4	109.2
5.3	17.12	104.9	101.7	104.6	100	104.9	106.5
7.17	13.7	98.8	100.6	99.5	99.7	99.4	102.4
5.3	22.66	101.3	103.5	99	98.3	98.3	101
6.84	13.94	102.3	103.9	97.5	96.1	102.9	105.8
9.33	18.55	95.9	96.3	93.7	82.4	79.7	83.8
8.47	15.27	92.3	98.4	89.4	109.8	108.4	110.1
5.5	14.97	94.1	95.4	97.1	105.6	102.8	93.7
9.99	19.8	100.3	99	95.3	105	103	102.7
7.11	14.78	95	98.1	97.9	101.8	104	102.4
8.82	29.04	94.2	98.3	100.2	100.7	105.6	102.9

8.65	21.02	100.7	101.1	99.9	105	99.3	102.2
6.34	18.91	106.4	96.7	104.8	101.5	102.5	100.7
8.24	25.38	102.4	100.6	100.4	102.4	100.1	101
7.25	14.93	114	100.1	91.7	89.7	90.1	90.2
8.38	16.56	112.7	99.9	99.7	83.5	86.1	89.9
6.95	20.43	105.5	102.2	103.3	90.2	89.1	87.1
7.94	15.92	101.8	102.1	96.9	98.1	92.7	95.1
10.3	17.93	100.5	98.3	104	103.8	101.6	99
6.23	17.05	106	96.5	100.8	111.7	109.3	115.4
5.14	10.77	100	101.3	113.4	98.1	91.9	88.3
7.37	15	100.1	100.1	105.1	93.6	100.4	94.8
6.46	16.74	96.1	92.6	99.5	96.6	98.2	99.1
6.54	16.98	99.6	93.6	85.2	96.6	87.1	95.7
9.48	15.46	116.2	102.5	99.8	99.3	95.4	107.4
4.78	14.98	97.9	98.2	98.9	104.4	108.2	103.4
8.75	23.07	99.4	103.9	99.8	99.6	102	96.9
8.18	17.46	101.7	94.5	99.7	96.1	92.4	91.5
6.61	11.86	100.7	104.6	99.5	116.3	112.6	108.6
9.92	20.72	105.3	101.7	96.7	104.7	104.9	108.7
5.57	18.12	97.9	100.5	106.8	108.8	113.1	114.8
8.51	14.51	96.3	99.3	94.8	101.9	105.5	102.9
7.25	15.42	107.3	128.8	120.7	127.4	128	130.3
5.14	16.32	100.9	98.2	99.4	99.1	94.8	102.4
5.55	16.86	106.9	97.9	106.1	105.1	105.4	106.3
9.94	15.21	97.9	101.6	102.2	110.5	107.5	112.4
6.24	17.14	107.5	92.9	87.3	101.3	108.5	108.1
7.81	25.72	94	99.8	95.5	101.1	99.2	99.9
6.52	15.48	89.9	109.5	96.9	105.7	89.6	88.2
4.61	14.2	83.4	88.8	79.5	104.8	99.4	96.3
6.29	13.68	100.8	96.3	97	106.3	116.3	115.5
9.6	16.29	103.3	99.7	96.5	137.5	141.2	135.8
8.32	16.17	98.6	94.4	91.1	94.4	94.4	93.4
5.72	16.08	92.4	96.9	94.6	106.5	105.5	106.6
9.11	16.75	106.1	94.5	89.5	99.1	114.3	113
8.9	13.94	108.1	106.1	105.7	96.2	98.8	97.1
10.18	16.18	102.2	98.8	98.1	113	117.7	116.3
8.43	16.53	99.5	89.4	92.1	96.8	96.6	101.2
8.09	24.51	83.1	86.2	81.6	107.4	102.8	106.1
9.38	32.3	99.3	100.3	101.1	106.8	106.5	106.3
8.95	15.02	97.6	94	94.7	98.2	98.8	99.6
8.22	17.5	101.9	97.8	100.9	98.2	101.8	96.4
5.97	14.83	94.1	93.6	92.1	104.9	98.9	106
10.7	42.41	100.5	94.4	97.4	107.1	108	110.4
9.99	15.87	92.2	96.7	96.8	107.5	106.9	104
5.97	14.72	102.7	96.3	100	96.9	95.6	101.4
8.79	16.83	94.5	100.4	93.4	110.7	113.4	106.7
9.16	9.2	110	110.8	103.9	107	99.3	110.3
5.1	14.48	103.8	99.3	101.6	97.9	94.4	95
4.64	11.68	92.4	96.8	89.9	114.7	116.1	112.8
8.43	13.21	93.5	90	90.3	104.9	98.6	109.8
8.37	18.84	100.4	103.5	103	105.9	105	107.7

5.54	25.59	94.3	96.2	91.7	103.3	102.9	103.6
6.77	26.68	102.8	102.2	102.1	97.6	95.1	95.3
7.02	14.12	101.3	94.7	94.9	102.5	100	104.3
5.95	18.79	88.3	98.1	88.7	101.4	92.3	100.8
9.45	36.85	102.5	101.4	97.2	107.3	107	107.5
4.77	14.45	95.3	94.6	93.5	102.2	91.6	96.2
5.08	25	111.8	105.3	101.7	129.5	132	133.5
6.54	18.17	113.9	110.4	110.2	77.7	73.1	74.6
5.07	12.38	99.3	102.3	99.9	107.4	107.7	100
5.55	15.79	99.6	97.3	100.2	102.6	104.7	98.4
10.92	18.67	103.8	101	100	105.9	106.8	108.8
7.34	20.99	102	98.3	97	91.9	92.5	94
8.37	15.52	102.2	114.7	112.8	107.9	104.7	107
9.36	21.75	103.9	100.7	100.1	100.9	97	96.8
10.95	44.07	109.5	103.6	101.8	102.6	104.6	106.7
5.59	13.37	102.2	94.9	98.4	109.8	112.7	109.3
5.38	13.4	102.3	102.4	98	92.5	92.5	92
5.67	17.8	98.3	97.9	99.4	101.1	99.6	106.5
10.35	18.33	105.8	103.6	108.5	108.3	105.5	105.1
7.62	16.53	102.2	108.5	104.4	99.7	95.5	102.2
6.18	14.62	100.1	109.8	101.2	101.2	106.5	101.9
8.66	18.4	105	97.3	97.3	100.7	100.9	103.7
8.1	23.41	99.9	99.2	101.7	102	101.3	102.7
7.05	14.69	98.8	102	97	104.4	102.1	105.8
6.84	10.57	108.2	104.6	96.7	102.7	102.6	102.9
4.88	18.51	98.4	94.2	101.1	99.4	101.6	95.1
4.36	19.22	95.3	92.1	90.1	101.9	97.6	109.9
6.6	13.85	107.6	107.1	105.8	91.1	88.4	91.1
9.33	11.19	107.9	101.7	98.9	90.3	102.2	100.3
7.05	18.44	95.4	94.2	93.2	101	107.4	108
5.94	24.86	104.2	110.7	107.8	84.8	91.9	100.4
6.35	8	99.8	99.5	114.8	102.3	95.1	100.7
6.61	23.67	88.1	83.6	96.2	112.2	115	115.3
8.24	16.15	103.1	102.8	104	104	109.3	106.7
6.61	7.53	104.6	96.6	102.2	113.2	110.6	112.4
6.68	15.21	99.6	108.1	94.7	103.9	107.1	95.5
7.39	11.64	85.6	90.4	99.8	110.6	107.1	110.9
8.79	20.06	107.4	113	103.8	109.1	97.8	109.7
7.74	17.96	97.5	101.5	106	107.6	105.5	126.9
6.3	14.42	96.9	99.4	96.1	99.9	97.4	100.3
5.24	14.85	93.2	95.3	95	104.2	105.7	103.6
9.79	15.3	105.9	99.5	102	108.2	107.9	111.9
5.17	14.5	95	89.6	107.6	93.8	117.2	104.7
4.79	13.54	101.1	99.4	102.3	96.3	95.5	101.6
5.91	12.98	104.4	99.3	100	105.7	104.7	88.5
8.43	9.48	100.3	103.9	98.7	99.3	96	105.9
9.63	12.8	97.7	109.7	101.7	97.3	104	97.4
6.54	12.38	101.3	102.2	103.1	88.3	92.8	87.3
7.39	15.47	94.5	85.1	95.3	116.3	114.9	120.3
4.81	14.25	108.3	91.5	102.5	100.3	98.5	101.3
4.94	15.29	100.1	100.1	99.7	97.8	106.1	108.3

8.47	16.04	100.8	103.3	98.4	99.6	105.7	111.1
8.68	16.76	100.2	99.6	107	109.2	106.4	100.2
7.33	14.86	98	96.7	94.9	95.1	98.7	104.9
8.47	12.26	103.1	99.9	97.8	87.1	87	92.6
7.17	15.25	105	95.6	96.5	105.7	105.5	105.9
6.8	17.35	97.3	97.3	96.3	109.9	106.1	110.2
9.61	15.2	99	106.8	104.7	105.2	102.8	107.1
9.19	14.26	98.9	97.4	94.6	98.8	100.1	98.8
8.12	18.42	106.5	99.2	100.3	92	95.8	91.7
10.07	16.59	102.9	95.1	102.2	102.9	104.6	107.4
9.72	13.17	95.9	96	91.2	103.5	88	122.9
5.52	13.82	94.5	98.3	102	105.9	96.3	100.2
4.94	11.78	99.9	100.8	101.1	100.5	97.6	98.3
5.63	12.27	98.3	98.5	101.1	95.6	103.7	98.2
9.52	16.35	100.7	92.7	102.5	91.4	96.5	98.8
9.73	12.43	103	99.3	90.3	118.4	111.7	110.2
5.21	15.44	95.8	97	91.2	92.2	91.3	95.3
6.83	20.93	101.7	96.8	98.7	96.5	99.6	94.1
5.01	14.61	93.5	93.1	100.5	105.2	115.2	101.7
6.98	11.94	99.9	96.4	100.9	121.1	106.6	129.9
7.91	11.38	105.3	106.7	102.5	114.3	107.9	105.2
9.72	18.23	106.1	106.4	105.3	105.4	109.2	109.4
6.47	19.02	101.4	95.9	108.5	97.4	99.3	103.5
5.16	26.19	95.4	102.1	95	99	94.8	99.8
5.45	10.65	102.1	84.4	95.1	107.1	112.2	106.9
5.72	25.02	104.3	107.8	100.8	109	105.2	106.8
7.75	15.09	85.3	95	95.9	98.1	103.2	102.4
5.36	11.46	104.3	92.6	99.9	99.9	92.2	95.9
6.62	10.22	100.2	99.1	95.2	100.2	93.6	94.8
12.26	14.77	95.8	107.2	103.8	100.4	99.2	101.3
5.38	17.34	97.4	94.4	96.4	105.1	107.3	113
7.15	12.19	105.7	102	103.6	96.9	96.7	102
5.57	15.86	94	98.6	92.5	108.3	108.4	111.7
8.76	13.49	101.1	94.8	106.4	98.2	105.1	112.6
8.48	20.33	85.4	94.4	83.8	99.3	98.9	97.5
5.92	19.09	104	104.1	104	102.8	107.2	101.7
5.41	15.69	106.5	98	97.7	101.3	103.6	98.5
9.07	19.42	90.6	96.8	107.9	100	106.4	105.6
8.79	30.41	95.1	104.6	97.4	109.9	110.1	107.8
9.23	15.81	97.6	99.4	103.2	105.4	96.1	99.2
9.38	17.56	103	102.1	105.1	104.3	109.6	110.9
8.46	15.88	96.1	94.2	100	108	107.1	108.9
7.39	13.6	107.8	102.4	97.8	98	98.2	107.4
9.94	10.88	111.1	105.6	99.3	118.5	118.3	111.4
10.7	12.47	100.1	109.9	91.1	116.9	108.6	110.6
8.94	10.92	97.8	96.9	96.9	103.9	100.8	102.5
7.87	11.33	97.4	97.1	91.4	97.9	98.5	100.7
8.69	12.09	105.6	100.5	99.7	116.3	97.7	120.7
5.11	15.19	97.2	95.6	92.8	110.6	106	105.2
5.31	25.64	94.8	98.4	94.1	100.2	92.7	97.1
5.74	18.76	96.4	86.5	94.1	82.1	86.9	93.3

5.77	10.24	93.4	94.1	102.4	93.2	109.7	100.3
6.51	10.35	107.8	100.2	101.1	104.3	108.8	105.2
4.98	15.77	99.4	102.9	98.6	100.4	101.6	105.1
8.9	19.8	104	96.3	103.6	102.7	109	106.8
7.71	16.52	97	91.3	100.3	107.7	112.7	101.8
9.85	22.21	104.4	103.1	101.5	100.8	103.8	103.6
4.78	14.18	91.6	95.6	98.4	96	97.6	101.2
4.83	16.36	102.9	95.8	102.5	113.2	107.2	106.4
7.43	18.83	97.4	97.8	92.4	98.3	96.8	95.1
9.03	12.78	105.3	100.8	100.3	100.1	113.7	110.1
6.07	11.96	105.7	101	99.1	107.2	99.3	103.5
7.14	19.94	116.9	117.4	118.2	70.6	73.5	71.8
9.66	26.9	104.6	101.1	108.4	95.3	107	99.4
6.73	12.57	103.5	98.7	107.7	116.8	97.6	108.5
5.41	16.39	101.3	100.1	98	91.6	94	96.6
7.88	16.46	106.6	102.2	103	102	101.7	104.4
7.84	16.45	104	103.2	93.3	99.7	98	104.9
8.53	14.01	92.7	89.2	94.9	107.3	108.6	101.7
7.4	15.9	95.8	92.6	97.4	102.7	97.6	95.6
4.61	14.15	104.7	97.3	99.9	110	102.4	103.3
5.1	26.42	94	95.6	94.5	97.9	90.3	96.6
6.79	13.25	103.1	96.9	109.6	100.2	104.5	103.7
5.29	16.47	99.3	98.9	104	101	98	104.3
7.49	20.78	101.4	101.6	99.3	100.2	95.3	100.1
9.16	15.11	93.9	92.3	95	97.7	98.8	105
8.09	10.53	106.8	100.5	100.7	95.2	88.5	94.7
5.58	18.02	103.4	105.2	99.4	99.9	102.8	99.2
5.45	9.88	101.3	95.2	98.7	100.9	100.7	107.9
9.57	17.38	107.4	101.5	97	93.9	99.4	102.2
4.86	15.56	92.9	100.2	102.4	100.9	101.7	101
4.74	23.8	97.7	92.8	97.6	102.2	100.2	107.9
8.92	16.26	105.8	106.3	103.4	82.5	99.7	89.1
6.79	16.8	116.5	101.3	104.8	87.8	88.1	94.5
6.9	11.06	111.2	97.2	97.8	102.1	96.2	102.6
5.9	15.34	98.5	98.3	96.2	100.1	100.2	107.6
8.02	13.56	106.4	104.5	100.5	109	94.3	112.4
6.16	15.3	101.4	102.4	102.2	100.1	96.2	102.1
8.24	18.35	94.9	99.2	97.9	96.6	99.5	97.9
5.71	18.02	106.7	96.2	94.8	104.2	98.6	101.6
7.81	13.37	105.2	98.7	98.8	93.8	98.9	102.4
8.87	18.81	99.7	97.9	99.5	102	106.6	110.5
8.75	17.46	101.8	101	102.3	98.4	98.9	101
8.56	16.63	107.4	95.9	99	107.1	110	116.5
11.27	18.24	101	105.5	108.9	108	107	116.8
6.21	12.61	97.7	97.8	100.9	101.7	100.6	93.9
5.68	11.33	91.2	88.6	107.1	106.4	99.2	99.5
8.41	15.1	107.3	98.5	104.9	105.4	104.2	107.8
8.19	13.75	100.5	96.1	102	98.2	99.9	100
8.38	14.98	97.2	97.5	91.7	100	96.5	96.6
6.8	8.57	104.8	102.2	100.7	102.7	107	112.3
9.94	16.2	101.2	105.1	103.6	98.6	101.8	99.3

5.49	15.49	97.5	102.2	102.9	105	95.6	100.4
6.95	13.13	82.4	77.5	78.2	116.8	109.8	117.8
7.72	16.64	107.7	100.7	103.5	107.5	102.9	100.4
5.9	32.03	101.1	104.8	101	99.5	94	96.1
6.54	8.05	101	97.5	101	99.4	96	100.4
8.09	11.72	102.6	100.7	98.2	116.8	102.7	111.3
8.53	13.18	104.5	104.5	103.7	97.6	104.8	103.1
10.76	13.64	109.4	95.4	130.8	104.9	131.8	106.4
6.05	9.96	103.8	96.7	95	92.5	97.4	100.3
7.93	13.58	107.1	107.6	103	92.2	93.5	90.9
6.15	13.51	106.9	98.2	104.6	112	108.1	108.9
8.7	13.35	103.6	95.9	100.5	100.2	104.3	104.1
7.93	16.73	121.9	113.8	108.7	94.9	100.3	100.9
7.74	26.46	95.3	97.3	98.5	106.9	107.5	109.5
5.99	13.22	97	93.1	108.1	98.9	100.3	96.5
9.55	21.29	99.8	98.5	101	102.8	98.3	97.8
8	16.89	95.7	85.6	90.4	102.9	110.1	109.7
6.93	12.13	92.9	87.5	97.1	100	96.8	95.2
7.25	16.77	93.6	90.2	100.7	105.6	113.1	115
8.05	17.06	104.7	98.5	98.9	96.7	94.4	96.8
5.97	13.63	101.6	100.6	97.5	103.7	116.5	104.4
6.2	13.76	94.1	88.7	94.8	103.9	105.9	108.9
9.61	11.66	108	103.5	94.1	104.7	102.5	111.8
5.07	16.71	95.3	91.6	101.8	97.1	106.7	106.4
7.72	12.16	101	95	101	94.5	97.6	91.7
5.92	11.73	106.1	98.6	97.2	108.6	95.4	96.3
8.5	12.87	97	91.7	88.4	103.2	103.4	106
6.58	12.9	109.2	108.9	98.3	97.4	88.4	97.8
10.37	9.41	101.8	95.4	104	111.4	107.9	116.3
5.49	12.75	101.8	98.2	100.7	97.3	97.2	103.6
8.15	12.62	100.9	111	105.6	109.9	101.1	106.6
7.87	15.11	103.5	98.5	104.7	112.7	113.9	116.2
7.06	15	112	103.9	105.1	100.8	91.9	99.7
6	12.76	93.5	102.9	101.1	101.9	95	102.6
6.4	24.1	96.2	94.4	94.7	92.6	93.8	98.5
8.21	14.97	99.6	100.4	98.7	127.8	113.2	103.2
6.11	16.07	63.8	65.1	63.4	135.7	136.4	131.3
7.83	14.78	107.4	97.2	97.9	101	101.3	97.2
10.01	14.43	99.4	108.2	103.6	103.2	109.5	104.1
7.68	14.94	105.7	99.9	90.3	101	105.3	107.5
7.49	22.12	92	90.9	93.2	106.7	112.8	112.5
4.58	18.05	103.9	97.2	94.9	96.1	101.7	100.6
5.99	11.5	106.8	98.4	99.2	101.7	98.6	106.8
5.55	10.14	99.9	92.5	107.2	92.1	92	110.2
9.14	12.55	109.5	98.9	98	97	95.9	95.1
8.48	13.78	93	102	98.2	103.4	103.3	99.4
7.14	13.43	109.2	107.5	98.5	108.5	119.1	111.4
9.44	33.82	98.9	102.2	105.7	107.3	101.8	108.5
4.93	13.07	99.9	103.2	104.3	97.9	99.1	106.5
9.99	12.75	102.1	96.9	102.5	111.3	108.6	113.9
4.83	8.94	97.8	90.6	97.7	111.2	97	96

8.19	14.21	101.5	107	111.3	84.5	89.2	94.4
5.71	14.29	87.2	85.4	84.1	100.4	100.2	105.8
9.16	17.72	103.5	101.5	100.9	94.6	94.7	96.7
7.47	12.64	105.1	97.8	101.3	92.8	96.4	97.1
9	18.3	96.3	88.4	86.7	110.6	108.6	105
6.3	17.53	98.9	88	83.7	105.8	106.8	105.4
5.52	19.39	96.6	94.4	88.8	108.3	103.5	105.7
9.04	14.63	96.8	96	95.1	105.5	104.7	110.8
6.15	16.23	100	95.2	99.9	86.1	95.5	95.2
5.87	14.14	118	74.7	111.6	148.4	120.7	131.5
6.16	14.63	98.8	92.9	92.9	104	100.2	101.2
5.4	14.19	104.5	102.6	97.5	106	104.8	106.7
7.83	13.85	92.7	97.4	99.7	96.1	98	102.6
8.25	14.38	108.9	96.6	96.6	95.5	96.5	96
8.1	15.72	102.3	100.5	97.2	105.8	103.2	103.1
7.34	10.03	94.4	98.6	103	105.2	99.2	103.7
9.16	12.75	101.3	114.8	107.4	97.3	103.1	109.7
5.26	14.36	97.9	97.6	107.4	96.6	99.8	103.3
8.13	15.36	113.6	110.2	107.1	127.7	127.4	134.6
5.41	13.87	102.7	94.3	104.8	95.5	102.9	102.9
9.38	14.44	110.4	106.1	105.1	106.6	104.8	109.6
8.27	16.59	108	107.3	102.9	101.3	105	101.7
9.63	12.92	101.8	104	99	101.2	106.6	102.6
7.05	10.45	101.3	82.9	100.3	90	109.9	98.5
6.92	12.52	107.1	105.9	103.7	105.5	100.6	102.9
6.64	8.03	100.9	84.8	99.2	91.4	105.2	92.9
7.4	16.15	107.4	98.8	102.2	95	95.1	96.7
5.24	16.04	94.6	96.1	95.2	104.2	98.7	105.2
5.6	11.93	100.3	100.7	104.5	99.9	96.8	96.4
9.92	11.81	97.9	95.9	100.6	109.7	109.7	112.5
5.1	11.68	102.6	94.8	100.8	98.7	101.5	99.3
8.22	12.76	96.9	94.1	95.7	96.7	99.5	99.9
6.46	12.13	95.9	99.7	100.2	98.5	100.6	91.4
6.54	11.06	97.7	89.2	86.9	103.7	115.5	109
9.61	10.03	103.2	103.2	108.6	103.3	100.1	103.8
7.37	20.91	102.6	102	83	100.7	99.5	99.2
4.87	10.88	96.3	96.8	93.9	97.3	93.7	91.7
9.16	14.87	95.2	90.4	93.3	106.9	115.5	114
7.65	12.59	102.8	95.2	90.5	98.3	101.1	98
8.84	13.27	76	89.1	69.2	103.9	97.3	104.2
7.64	12.81	99.9	91.9	97.5	96.5	96.4	104.5
5.54	15.2	94.2	103.9	110.1	98.6	97.2	94.6
5.19	13.1	101.6	103	98.5	104	101.4	93.5
6.48	11.97	94.5	96.9	93.8	109.5	101.2	101
5.3	21.84	104.6	94.6	107.5	91.7	92.1	91.2
7.05	11.99	113	110.6	111.8	90.2	89.8	95
7.75	10.14	91.3	106.5	96.8	93.6	97.7	104.6
8.19	15.84	54.5	54.5	62.9	135.7	140	138.2
5.31	11.35	97.8	95.7	94.4	92.5	91.1	94.8
7.99	16.27	100.6	97.6	99.6	107.2	112.8	108.3
7.65	13.11	98.4	100.4	101.9	97.9	96.2	111.9

5.91	17.31	101.9	71.5	69.2	122.2	104.4	92
8.79	18.16	98.2	94.5	94.8	106.5	101.5	98.7
6.99	17.73	97.2	94.4	99	103.4	102.7	106.8
7.39	9.46	95.6	94.5	93	111.6	111.5	118
4.97	16.58	95.9	98.7	104.8	98.3	93.3	91.2
7.15	18.36	97.4	99.9	99.3	98.9	100.7	100.9
5.57	11.82	96.4	95.7	96.6	101.7	99.1	96.9
5.31	17.81	102.6	95	98.6	100.3	101.7	100.6
9.11	14.57	103.9	109.6	93.1	97.5	93.9	86.8
6.57	13.05	99.2	103.9	103.7	100.1	94	95.9
5.29	15.57	96.7	95	99	93.8	106.5	101.5
6.18	13.94	98.2	90.9	90.1	99	102.5	114
7.94	14.43	95.8	93.6	101.1	102.6	104.2	96.3
6.6	15.84	106.9	102.3	99.1	97.7	97.4	95.3
7.31	16.17	107.1	102.2	97.9	83.5	93.1	92.1
6.13	14.25	107.4	109.8	103.5	96.6	98.3	98.4
8	20.61	96.1	97	118	101.6	101	76.9
7.44	15.13	96.3	96.3	97.5	105.3	101.9	107.1
9.29	13.5	104.2	96.8	95.3	105.7	111.6	110.6
5.69	17.88	102.2	99.5	95.2	89.4	87.5	91.1
7.4	11.95	108.8	108.2	108.8	97.4	101.8	98.9
9.29	12	108.7	97.2	98.4	103.6	108.1	105.4
6.77	13.75	105.7	99.6	104.2	102.7	102.5	103.2
6.27	15.54	108.8	94.8	94	101.4	105.1	99.3
7.8	16.86	107.9	108.7	109.6	82.1	76.4	78.3
9.35	13.08	101.9	102.4	99.5	114.5	112.3	108.4
9.79	13.07	103.9	95.1	98	105.4	105.4	105.8
8.02	13.18	90.7	96.5	96.7	98.8	97.2	105.8
8.65	13.54	104.7	101.6	97.6	98.7	98.9	97.9
5.35	15.35	92	100.6	94.4	110.5	108.7	107.5
4.54	12.29	105.2	100.7	102.3	106.3	105.6	113.9
10.3	14.5	99.4	103.4	104	105	107.3	108.9
7.96	20.05	105.2	74.9	69.6	101.3	103.2	105.9
4.61	11.63	102.6	99.1	100.9	110	115.6	110.8
5.4	11.57	110.7	98.1	104.1	108	110.5	109
8.79	12.28	98.9	98.2	105	99	97.2	105.5
5.17	9.6	96.2	97.8	104.8	93.6	98	91
6.07	16.49	101.9	100.1	99.3	105.1	97.7	101.4
5.27	11.57	99.4	100.4	100.9	97.5	104.9	98.1
9.41	11.6	98.3	94.1	93.3	99.4	99	98.5
6.52	15.68	108.4	103.2	106.5	97.9	95.7	105.7
5.57	8.26	96.8	90.4	102.8	93.8	100.1	97.6
7.28	16.78	100.6	109	107	116	116.7	116.4
9.67	13.82	120.1	92.3	94.6	100.5	122.5	102.1
4.77	11.71	102.4	105.8	101.1	96.7	100.3	105.9
5.6	15.9	95.1	87.5	93.5	99.4	102.3	101.5
9.01	12.34	89.7	86.1	87.8	103.5	110.4	113.5
6.38	8.62	108	114.4	96.9	102.3	94.9	85.2
6.95	9.55	99.6	100.9	105.6	97	103.9	105.9
8.09	10.55	137.2	94.6	88.7	98.3	124.9	90
5.88	11.62	97.4	99.4	97.9	105.4	103.9	106.5

7.83	13.37	103.1	107.8	110.2	86.7	95.5	101.2
5.25	13.29	97.3	98.5	101.9	106	110.8	108.6
7.36	15.78	92.7	105	102.9	105.6	90.2	105
8.68	10.62	99.5	106.8	102.4	104	117.6	117
9.16	11.6	107.4	98.1	97	110.6	110.2	117.3
6.29	13.14	102.4	97	98.9	100.7	97.4	102.1
10.84	15.36	112.3	109.9	111.4	91.9	90.4	85.6
9.29	12.57	100.6	94.6	95.4	92.4	101.7	96.2
10.48	17.83	102.7	100.6	95.9	109.6	106.2	106.2
8.57	19.36	94.1	99.5	99	96.3	95.1	92.3
6.76	10.87	95	93.3	99	105.8	101.1	95.9
7.01	11.45	101	97	109.6	81.6	110	90.2
9.54	9.7	90.1	103.6	97.7	103	103	112.1
8.34	10.67	97.5	90.3	105.3	106.8	120.6	110.8
4.86	16.45	96.8	96.3	95.1	96	96.5	97.8
6.15	14.34	89	99.7	90	110.1	102.9	106.1
7.42	16.58	97.5	101.8	103.4	99.2	100.7	91.9
4.97	12.94	104.4	93.6	103.7	104	103.4	105.8
6.73	14.22	119.4	104.3	103.7	105	104.8	95.3
6.43	8.19	93.6	89.9	85.1	100.4	101.5	106.4
7.66	23.09	94.7	113.1	105.8	119.3	112	122.5
8.03	13.61	118.9	92.5	97	87.5	102.1	109.6
8.37	13.22	102.7	98.2	98.5	96.3	99.8	95.9
6.33	14.95	95.8	98.6	104.8	109.5	107.1	114.6
10.39	13.04	104.3	102.6	101	111	108.2	102.8
5.85	8.19	87.8	85.6	95.5	95.6	100.2	97.4
7.39	6.61	106.1	101.3	91.3	71.4	97	97.9
7.37	7.93	93.8	106.6	105.3	104.1	103.9	114.7
9.33	12.09	98.7	94.5	104.9	98.9	113.1	111.1
8.48	18.58	103	96.1	97.4	98.1	93.6	99.1
6.9	11.86	94.6	93.7	89.8	95.5	100.4	112.6
6	14.65	110.4	106.6	104.4	91.8	94.9	88.3
8.95	13.5	107.1	97.3	93.8	107	106.5	110.4
8.82	14.24	101.2	102.2	107.5	106	106.8	102.8
5.43	18.36	92.2	84	94.1	98.3	98.3	99
8.21	13.02	94.7	99.4	108	103.4	104.5	105.7
9.35	15.48	101	106.2	104.6	107	112.1	108.2
11.27	14.94	106	105.2	99	106.4	112.9	106.6
9.39	16.37	73.7	79.8	66.8	132.1	127.1	126
6.16	13.32	96.2	94.6	96	103.3	109.7	103.1
7.91	10.29	104.3	96.3	94.2	114.9	114	107.4
5.21	12.54	101.5	93.3	94.1	89.5	83.2	87.6
9.88	10.94	94.9	92.6	94.6	117.9	112.2	116.2
10.13	11.59	94.4	96.7	102.6	103.4	108.1	102
7.33	11.67	103.9	98.8	92.4	101.8	99.6	103.7
8.85	13.81	94.6	95.3	101.2	89.1	102.5	85.6
7.49	11.74	116.1	109.3	113.6	115.4	113.9	112
4.82	21.66	94.3	94.6	93.8	105	104.9	106.3
6.49	12.5	103.7	92.4	105.4	101.3	100.8	101.6
5.12	13.12	103.3	91.5	93.3	97.4	101.6	110.6
6.55	9.69	90.5	89.7	99	95	104	101.4

9.26	12.86	100.9	113	103.4	101.7	105.9	108.9
5.2	13.01	121.5	121.4	117.9	73.8	79.5	77.3
5.83	9.93	96.1	100.6	100.4	104.7	105.8	107.2
7.94	9.35	118.8	114.7	96	82.6	76.7	81.6
6.79	13.42	99	93	92.1	89.5	85.7	90.9
5.22	13.31	97.5	94.5	99.9	117.3	108.4	117.4
9.94	8.97	100.6	101.4	98.9	103.7	102	99.7
5.86	17.14	111.8	108.8	111.2	94.7	86.3	88.6
5.9	16.21	98.7	94.3	98.1	95.8	98.2	102.1
8.21	15.98	107.7	100.7	102.2	107.6	110.2	114.1
5.06	11.61	110	94	97.5	97.8	110.6	105.4
6.01	9.02	95.8	98.6	98.8	104.2	105.4	107.8
4.97	9.93	98.7	98.8	89.1	117.5	107.7	104.3
9.36	9.34	102	97.3	101.1	106.2	102.2	108.1
5.73	12.9	102.5	103.5	103.6	95.8	100.5	97.3
5.12	15.96	99.6	99.3	103.6	101	101.9	101.7
4.84	11.01	112.7	84.9	101.1	108.3	101.6	101.2
6.55	13.11	93.2	97	87.1	101.1	106.6	103
8.73	13.09	103.3	94.5	101.5	103.5	100.9	97.1
4.51	12.32	106.6	99.6	101.3	99.4	101.8	104.5
8.75	15.22	96.4	103.7	93.4	93	99.7	100.7
5.96	14.4	95.6	98.6	104.9	97.3	105.9	105.6
8.54	12.66	111.6	99.2	102.1	92	91.5	99.9
9.64	12.57	103.4	106.6	105.6	103.6	106.2	108.6
5.64	8.77	116.8	107	115.4	96.6	90	94.5
5.85	10.83	106.9	90.1	95	104.4	100.7	107
5.34	13.44	99.6	97.1	92.5	93.2	104.4	100.8
7.46	11.01	99.1	100	107.6	95.7	91.9	104.8
10.39	14.37	103.1	108.2	104.9	100.1	103.2	103.8
7.31	10.73	103.2	99.7	104.8	111.5	112.8	117.7
4.7	12.66	98	99	98.8	95.8	107.9	109.2
5.5	23.14	109.3	96.1	114.1	113.4	122.8	109.1
7.01	10.25	100.9	101.7	101	130.6	127	111.4
7.33	14.17	97.8	101.1	105	101.7	91.7	105.3
7.24	13.57	100	97.8	104.1	94.6	100.9	104.4
8.5	26.98	112.2	98.4	109.5	96.8	105.8	98.2
9.77	16.85	99.7	97.6	101.9	95	94.7	93.7
6.89	9.24	91.2	83.5	91.3	114.7	107.9	108
5.71	13.12	98.3	99.9	100.5	100.6	111.3	115.7
9.76	14.64	100.2	91.3	99.1	99.4	102.6	106.6
6.18	11.08	101	99.1	92.5	89.2	95.9	89
4.83	15.98	96.6	102.2	102.6	103.3	100.7	107.8
6.01	8.03	109.9	107.7	112.3	102.5	108.1	120.3
7.55	16.38	103.7	99.4	97.6	95.7	100.2	95.5
8.5	11.52	92.6	84.2	75.5	106.5	102.1	109
7.72	11.43	97.2	101.4	103.6	101.4	103.1	103.5
8.54	10.71	108.8	102.6	104.4	100	106.5	118.5
5.11	11.31	102.6	101.6	99.9	104	100.7	99.3
8.92	16.58	101.5	104.9	96.9	107.6	106.7	107.6
8.5	11.99	94.1	91.7	109.3	93.4	74	87.3
11.09	11.76	100.3	97.8	101.1	105.9	103.2	105.2

5.15	9.41	112.6	105.9	99.1	101.6	99	112.1
6.8	17.44	103	96.7	102.3	90.3	84.6	85.6
11.41	6.54	110.7	99.9	102.1	105.2	105.2	106.9
6.24	6.29	103.1	109.7	80.9	100.5	96.2	93.5
8.76	18.09	100.2	95.5	98.1	110.2	114.6	113.9
11.65	12.54	105.8	102.6	103.1	104.1	110.9	105.8
9.7	9.88	97.9	101.7	108.7	111.3	95.1	112.5
6.48	18.07	88	92.8	94.3	102.7	94.6	95.9
6.11	12.69	101.3	97.8	96.6	97.8	95.7	102.7
5.22	11.22	106.9	96.1	99.3	101.9	99.3	101.2
5.9	13.51	110.2	99.4	98.6	110	111.5	114.2
9.5	9.89	113.3	101.5	83.5	106.1	112.6	111.1
8.44	10.24	95.3	90.9	98.6	102.1	98.1	104.5
8.44	13.11	103.9	104.3	94.4	102.4	94	98.6
9.7	9.61	107.7	97.9	110.6	104	94.9	97.1
6.46	13.68	99.6	94.1	102.5	93.2	95.6	100.9
4.84	13.35	110.1	100.6	107.4	96.2	96	95
5.44	11.65	135.1	71.2	87.4	72.5	98.9	98
9.58	10.47	100.4	95.8	99.6	106.5	106.4	104.8
7.59	12.66	94.7	111.4	98	100.3	99.8	103.4
9.99	11.8	118.4	103.5	103.3	106	103	108.8
5.52	8.5	101.1	103.9	113.5	104.6	113.5	110.9
6.6	8.94	112.3	105.7	104.6	101.3	94.3	108.6
9.45	10.46	98.7	114	101.9	101.7	100.7	98.1
6.68	13.12	107.1	101.5	106.1	96.1	99	104.7
7.61	12.08	105.4	107.9	94.1	95.2	99.4	103.4
7.17	8.75	96.2	101.7	112.1	93	98.3	106.4
6.35	10.93	91.5	91	102.3	114.8	104.8	96
6.89	9.66	92.2	95.9	91.6	102.7	101	99.3
9.94	12.66	102.3	99.8	99.3	103.7	105.7	111.6
6.73	15.24	97.6	99.1	106	95.7	94.6	103.8
4.88	12.15	105.7	100.2	98.1	103.6	97.9	97.6
9.85	10.02	119.9	95.2	115.9	107.7	88.9	92.3
9.83	16.33	117.7	77.1	88.1	115.9	59.9	101.9
6.7	10.65	95.4	91.9	106.9	103.1	111.8	103.2
8.07	8.45	104.2	99	94.4	101.7	99	90.3
6.74	16.77	91.1	94	98.6	110.4	108.2	94.1
9.52	16.27	100.4	98.8	98.7	102.5	104.4	106.4
7.65	12.27	99.5	102.6	97.9	98	91.7	99.2
7.01	10.71	101.2	102.8	108.1	102.6	109.7	113.2
4.84	13.8	98.8	102	100.3	99.8	105	113.8
9.63	12.9	89.7	95.2	95.5	97	97.8	100.5
7.85	7.33	104.8	66.9	110.6	100.6	97	96.9
11.56	21.9	93.3	91.6	93.5	122	123.5	123.3
6.29	14.55	108.7	106.6	105	110.9	109.4	112.5
9.16	11.49	99.8	97.7	94.1	108.7	99.1	115.8
5.45	11.98	99	93.9	93	105.7	102.7	105.9
8.54	12.99	86.6	92.6	100.4	106.4	106.3	102.8
5.11	8.19	102.6	103.6	107.3	110.9	103.8	93.7
8.72	11.91	111.2	104.4	88.7	97.4	140.9	111.5
7.21	11.67	101.6	104.9	104.7	96	102.1	98.6

6.16	11.19	98.9	105	100.2	100.9	98.7	100.9
6.61	12.54	104.2	97	94.2	102	101.7	107.7
6.2	15.81	103.7	105.1	97.5	105.6	106.4	105.7
9.57	11.87	109.5	105.7	101.3	109.1	111.9	108.8
6.05	8.67	95.1	99.5	100.8	100.6	93.1	97
7.93	12.4	116.1	111.9	108.7	103.7	100.5	99.3
8.76	10.92	101.6	94.6	105.1	104.3	103.3	97.2
6.54	10.25	88.4	89.9	89	107.9	105.7	119.1
6.07	10.76	106.7	100.4	104.9	105	104.5	101.8
9.16	9.97	111.2	88.1	99.9	117.7	105.2	129.7
5.24	8.51	107.3	102.3	94.4	104.3	89.5	98.6
5.01	10.67	106.7	95.2	100.1	114.9	116.3	116.8
8.31	19.88	100.5	110.5	107.2	92.1	102.4	97.1
6.52	16.65	98.2	99	98.4	102.4	102.6	104.6
5.15	12.68	99.8	101.4	97	109.6	104.3	108.3
5.6	13.17	98.9	94.9	97.3	102.8	106.9	106.3
7.08	11.56	95.5	97.5	98.4	105.5	102.7	106.7
9.69	11.91	105	113.2	105.7	94.6	91.4	100.5
8.81	14.96	101.7	106.7	101.4	106.7	111.1	106.1
9.77	9.09	93.6	105.5	95.6	105.4	107.1	102.4
5.92	11.71	100.1	102.2	99	99.1	98.2	96.4
9.25	10.09	101.6	97.2	99.3	113.9	112	121.5
6.58	10.99	92.6	99.9	102.8	95.6	91.3	97.6
5.34	17.4	77.8	77.6	79	125.2	125.6	123.1
8.19	12.93	93.1	95.3	102	107.4	112.9	111.4
7.37	9.24	95.3	90.3	96.3	128.2	128.1	120.2
7.12	14.55	98.4	98.1	94.7	100.2	99	102.7
8.41	10.72	99.2	95.7	101.3	102.1	97.1	96.8
9.63	10.31	110.8	108.4	107.4	106.1	105.7	103.8
6.9	9.49	100.1	89.8	98.4	102.7	106.1	114.5
9.58	16.7	104.7	104.4	102.9	101.4	103.9	100.4
6.51	13.63	95.8	90.8	102.2	96.6	93.8	97.5
5.43	10.97	101.9	106.4	114.8	104.9	99.3	103.6
8.53	9.82	97.8	102.3	106.5	109.8	105.8	111.1
5.81	10.81	90.6	108.6	105	96.8	92.4	100.5
8.43	8.53	103.3	103.6	108.5	105.9	89.8	94.4
10.64	10.58	103.3	100.1	98.9	113.1	111.3	103.5
7.24	10.33	96.9	93.4	95.4	98.4	102.6	109.9
6.16	8.68	101.5	94.4	101.5	95.3	99.7	97.1
7.88	11.21	86.1	86.7	83	118.2	116.9	120.9
9.03	8.77	98	95.9	95.2	100.7	98.1	108.2
6.46	11.72	99.3	98.5	99.4	95.9	98.2	89.4
5.14	9.7	106.4	101.8	93.7	86.8	102.1	95.7
5.49	21.29	102.4	105	100.7	104.5	107	105.2
4.97	10.42	105.2	104	95.4	103.7	105.8	98.4
5.68	12.39	99.5	102.3	108.1	96.7	92.9	99.9
7.96	15.46	92.8	91.8	93.9	114.6	109.2	108.7
7.21	17.55	103.8	96.7	101.2	104.1	107.1	101.1
9.06	15.06	103.2	105.4	104	108.6	106.5	105.3
6.13	11.64	90.8	96.4	103.6	101.5	108.6	108.1
7.43	13.6	101.2	107.9	99.1	91.3	96.1	101.3

6.54	9.62	102.9	102.3	96.3	96.2	93.1	103
7.66	5.3	106.2	110.3	97.9	96.2	109.1	83.8
9.8	18.23	78.9	93.4	86.9	106.9	110.2	102.8
7.61	14.98	109.6	101	107.9	93.3	105.3	104.4
8.62	10.79	95	88.4	90.5	114	105.8	103.9
5.11	10.88	55.9	53.2	60.1	171.4	166.1	164.5
8.32	12.49	105	108.5	102.9	106.7	103.1	101.8
5.85	6.49	102.7	101	92.6	111.1	108.5	109.7
7.27	15.03	109.3	96.3	97.5	109.3	105.4	113.7
4.48	11.12	102.5	93.3	101.5	108.6	102.1	100.5
7.72	9.28	95.9	103.4	100.1	90.7	93.7	96.6
9.7	11.37	108.9	103.1	98.9	103.8	100	100.1
6.57	12.31	99.3	95	96.1	97.5	100.5	110.3
8.85	17.69	102.4	101.9	96.8	102.8	100.3	104.5
5.06	15.57	100.1	101.5	103.6	100	103.9	105.1
7.49	11.97	92.7	95	94.3	112.6	107.6	112.7
11.74	35.5	95.8	99.6	96.3	117.3	111.6	114.4
7.84	11.88	83.4	85.6	83.2	87.6	85.2	86.1
9.38	28.23	90.9	95	90.4	98.7	98.2	94.7
9.76	17.98	98.3	105.5	102.6	105.4	106.7	105.9
8.21	11.19	99.8	101.4	96.4	86.2	84.9	92.1
7.11	11.89	107	93.7	94.9	85.4	80.5	107.5
5.78	10.44	118.7	106.2	91.6	112.3	79.7	91.8
4.49	10.83	96.8	93.6	94.7	100.9	93.4	104.3
9.13	10.49	96.8	97.9	98.6	103.5	101.5	101.7
6.24	17.35	97.3	94.5	96.5	99.8	95.7	96.7
9.29	13.65	104.3	96.2	94.5	110.8	109.2	107.7
7.93	9.21	97.2	110.3	124.1	95.5	95.9	100.1
6.62	9.99	91.4	98.1	103.3	103.4	98.9	102.4
5.03	10.01	94.7	93.8	105.7	97.4	114.1	108.9
8.19	9.97	90.8	92.8	90.9	112	110.4	118
6.87	10.69	102.2	97.6	102.6	97.6	100.2	100.9
9.04	13.2	102.4	93.7	100.7	109.4	112.7	111
7.47	8.18	99.9	103.2	100.4	99.2	100.5	102.9
9.26	15.03	105.1	93.1	99.2	96.2	96.8	100.5
5.83	13.95	84.5	77.5	79.7	89.3	89.2	89.5
5.31	11.65	108.2	83.5	104.8	97.5	111.6	114.8
10.15	11.88	101.1	93.5	97.5	103.5	111.9	110.5
7.02	9.57	96.7	98.4	99.8	103.6	106.6	102
4.88	14.01	119.2	102.8	100.3	103.7	105.4	101.5
8.48	11.61	101.8	105.1	103.7	100.1	99	104.1
8.09	6.96	77.9	88.8	93.4	112	108.8	110.1
5.02	11.41	95.3	98.6	94.4	98.8	96.7	101.9
8.72	8.65	94.5	95.4	103.1	111.7	118.8	102
9.01	5.62	98.8	104.3	109.4	107.3	98.8	111
8.06	17.95	100.6	100.9	100.7	106.2	102.9	103.9
9.67	22.34	101	105.2	101.9	107.5	105.2	98.9
4.25	15.37	97.4	102.9	100.2	104.4	103.5	105.9
6.38	9.48	101.4	96	95.3	103.8	101	108.8
10.27	10.76	102.8	102.4	97.2	110.2	115.5	108.2
6.71	13.6	101.6	99.7	103.1	102.5	97	99.2

7.55	9.12	99.5	94.3	89	98.4	103.7	102.2
7.15	10.11	103.1	83.8	91.3	104.6	119	120.5
7.94	11.52	99.4	99.9	98.1	107.6	107.2	108.3
6.16	10.73	93.2	103.5	98.7	104.1	97.4	101.3
5.19	10.14	104	101.7	99.7	95.3	107.9	102
10.81	10.8	99.6	106.1	100.6	102.2	104.4	111.7
5.08	7.47	103.3	88.4	92.8	110.2	112.4	109.4
6.1	11.11	103.4	89.8	94.9	99.4	104.2	102.9
9.38	19.94	101.5	97.2	101.4	103.3	104.1	100.8
5.11	12.72	101.4	98.9	80.4	116.9	101.7	84
9.67	8.91	98.9	97.8	104.4	106.4	105.1	108
7.05	7.89	99.8	98.6	96.5	109	104.6	96.1
10.1	8.82	129.3	99.1	96.4	100.1	102.8	91
5.11	8.77	103.5	107.5	89.7	98.2	98.1	101.2
6.79	10.6	95.7	87.5	94.9	104.4	111.8	109.1
5.9	9.98	101.7	107	106.8	123.2	122.5	116
11.56	14.07	106.9	95.2	107.1	95.5	92.8	95.6
4.89	10.65	100.5	104.9	101.1	112.6	106.9	107
5.39	10.53	102.2	100.6	110.3	101.5	103.6	103.9
6.4	13.14	96.4	97.9	102.7	99.9	98.9	100.4
9.26	9.23	104.6	107.2	112.6	112.5	99.1	99.4
4.2	6.85	97.7	98	99.1	101.2	100.7	102.5
7.24	23.01	106.6	102	105.4	97.3	91.3	95
6.4	10.35	100.2	90.5	100.8	103.2	104.3	104.5
4.35	9.73	99	88.2	95.7	104.4	98.9	105.8
8.9	11.66	91.4	92.7	91.9	101.2	109.9	99.6
5.52	13.07	95.3	100.8	106.3	95.3	102.1	99.5
5.1	11.08	97	104.1	96.7	107.7	116.9	111.6
9.89	12.44	108.6	97.8	102.2	102.9	102.4	108.1
5.31	10.95	111.3	104.2	120	100.2	103.5	101.8
7.21	12.41	94.9	91.1	92.6	108.8	108.9	107.2
7.71	15.49	91	94.4	86.8	91.3	99	100.2
8.91	7.89	97	99.3	102.1	115.4	104.6	104
7.2	10.26	91.9	99.4	108.1	103.1	94.7	94.2
7.52	9.13	95.7	93.6	100.5	103.9	96.5	116.1
7.85	11.37	104.8	93.5	104.9	102.8	98.7	112.6
6.6	10.07	105.4	109.7	107.9	94.4	103.2	91.9
7.83	12.12	107.6	94.4	88.9	82.1	98.6	116.9
6.2	11.18	108.1	99.8	102.2	103.6	103.8	109.4
7.84	9.83	123.5	120.3	122.9	76.6	70.9	72
8.88	10.11	98.9	97.7	106.8	99.3	93.1	110.6
6.6	7.01	111.9	108.4	98.1	122.7	111.6	121.4
8.69	9.53	105.5	97.7	98.1	97.3	113.2	98.3
9.04	13.05	102.4	107.8	107	103.2	103.3	102.1
8.98	16.73	100.3	98.5	94.9	103.4	102.7	115.3
8.22	8.21	100.9	95.5	95.3	118.6	107.1	113.3
7.85	9.18	99	102.2	95.2	103.7	111.3	113.9
8.91	8.17	107.4	94.8	106.8	106.5	109.2	104.2
4.54	12.01	94.1	94.2	97.5	102.7	97.3	103.2
7.94	10.7	107.5	103.8	103.5	106.2	103.5	102.6
8.46	10.5	100.6	110.9	98.7	110.2	110.7	106

5.69	6.8	107.8	99.9	103.5	103.4	102.8	103.6
8.28	11.23	104.3	99.2	107.5	96.7	100.1	95
8.94	11.59	98.1	98.7	107.3	102.5	100.6	101.3
7.08	7.77	98.7	99.1	109.8	105	115.8	108.2
5.9	11.55	101.5	96.2	96.4	110.7	108	114.1
8.43	11.42	102.8	98.5	104	102.1	100.3	101.8
8.44	7.69	107.6	99.3	100.1	116.5	112.8	124.2
4.63	9.91	105.9	101.2	95.3	103.9	97.1	94.9
7.68	8.01	104.7	94.4	110.1	101.8	94.9	104.8
7.85	14.06	98.9	104.8	98.2	101.9	103.1	101
8.31	10	99.1	88.4	98.6	103.7	106.1	113.2
5.99	12.28	99.5	95.1	96.1	106	103.7	103.7
6.52	11.42	89.1	91.7	85.5	94.3	97.4	93.8
5.91	10.1	91	91.6	90.8	103.8	104.3	107
7.69	10.43	99.7	103.7	99.3	97.7	105.1	108.4
8.28	11.05	105.8	102.4	102.1	102.2	98.8	93
6.14	9.69	94.2	95.7	98.3	102.1	100.9	104.8
5.55	10.56	105	88.2	104.1	109.3	92.2	92
7.03	8.78	100.2	101.1	103.9	106.3	97.2	104.2
9.5	9.28	92.4	91	100.1	106.4	100.7	104.2
9.38	10.54	104.3	101.4	100.3	106.3	107.6	105.4
5.5	7.27	98.3	95.1	105.5	112.5	111.9	109.2
8.15	10.78	101.7	102.2	101.8	102.8	105.2	101.4
6.74	10.36	95.7	103.4	115.4	92.2	107.3	98.6
7.14	12.16	103.6	93.8	96.6	93.3	100.4	103.3
5.38	9.78	110.4	109.4	105.6	110.4	100.4	99.9
11.27	50.19	112	117.1	112.7	108.9	108.8	107.4
9.52	10.18	98.4	101.5	98.3	101.3	100.4	106.3
4.44	10.33	104	102.5	99.1	133	124.1	114.2
6.73	9.85	100.2	98.2	99.3	94.7	90.7	101.4
7.09	9.32	101.8	100.6	78.4	106.2	107	109.9
9.77	8.32	101.1	102.4	110.6	108.8	110.5	107.2
5.38	9.84	101.4	95.9	95.2	98.3	100.4	101.9
9.31	8.58	105.1	102.7	103	101	96.5	90.5
8.91	10.87	102.1	102.4	100.2	113.2	104.3	113.2
8.73	8.35	85.6	92	94.7	109.8	103.9	92.7
5.21	9.47	102.6	93	102.2	98.6	97.1	98.9
5.4	71.41	83.4	99	102.3	97.8	93.9	87.5
9.17	10.45	99.7	84	105.3	117.4	116.4	122.3
8.31	9.49	105.2	102.1	100.3	98.8	100.3	107.2
6.7	9.53	104.2	111	99.6	112.2	106.2	106.4
8.51	11.59	98.7	96.6	96.3	96.6	100.7	99.1
6.7	10.7	84.2	85	89.6	104.4	99.6	106.3
4.81	10.55	105.7	104.6	103.8	112.3	110.3	110.5
7.42	10.73	80.5	80.9	72.8	84.2	80.3	84.3
5.35	8.54	104.9	99.8	106.8	104.6	104.2	103.4
7.33	6.62	100.4	94	103.4	94.2	81.7	97.7
5.48	7.69	100.8	93.7	89.8	99.1	101	94.9
6.47	5.43	111	101.2	95.7	93.2	94.4	107.5
6.18	12.26	104.3	100.1	100.8	98.1	99.9	94.6
8.13	5.14	110.6	108	80.7	125.8	104.6	123.7

8.92	7.77	102.3	93	94.2	105.7	117.8	113.9
6.79	13.27	101.9	96.3	100.4	94.6	96.9	104.6
6.23	14.05	105.3	90.7	110.2	89.6	98.9	91.9
5.85	8.21	99.8	99.2	98.3	98.2	107	102
9.14	9.18	102.2	90.1	94.3	102.5	112.2	101.7
6.1	8.3	97.2	96.8	101.7	94.4	103.5	103.8
4.54	9.4	104.1	95	100.6	113.5	115.6	110.7
5.34	6.74	101.9	99.5	95.9	99.8	99.6	107.6
5.97	8.09	95.9	100.5	113.3	107.2	105.6	102.7
10.11	8.03	103.9	99.5	105.2	113.3	110.5	109.5
5.92	10.27	96.8	100.5	112.1	105.8	116.2	126.7
8.03	8.35	94.4	91.5	107.7	100	113.9	103.7
4.94	10.49	93.6	83.6	92.8	99.5	98.4	104.4
4.58	7.89	104.7	89.4	99.8	110.1	107.4	108.1
8.22	9.64	94.5	90	96.7	99.5	103.2	99.1
4.93	13.23	49.6	50.4	53.1	150.1	144.6	142.1
8.12	24.76	96.3	100.1	97.7	123.1	123.4	121.2
8.32	8.08	109.7	97.6	99.5	104.7	100.7	110.6
9.92	6.83	101.1	106.7	108.8	105.3	101.3	109.7
5.29	14	110.6	96.2	105.6	108.5	102.5	114.4
8.13	8.92	96.5	90.6	98.8	108.1	112.2	105.3
9.66	6.23	100.9	106.3	100.2	96	102.7	102.2
9.42	13.43	94.6	98.5	102.2	104.6	108	105.1
7.28	7.37	124.9	80.8	94.3	119.2	89.4	108
9.77	9.78	101.1	99.2	96.6	113	102.5	104.8
8.63	7.73	110.1	102.8	102.1	84.2	81.1	91.5
4.2	9.96	89.9	97.5	106.7	112	103.5	101.8
9.48	10.12	125.2	106.9	93.6	105.8	95.6	99.8
4.65	9.5	112.7	104.7	118.3	108.1	112.7	113.4
5.55	4.82	102.1	96.8	90.4	101.5	93.3	108.2
7.58	6.41	103.5	97.3	106.4	122.5	119.4	130.3
7.15	11.01	157.3	160.2	167.1	59.9	53.5	66.7
9.13	9.71	101.4	94.2	100.5	106.4	101.4	105.7
6.92	9.18	115.8	97.7	94	88.4	115.9	100.9
6.64	8.54	109.4	82.3	104.7	81	98.2	95.8
8.56	10.34	108.1	102.3	100.5	112.9	114.8	112.1
9.57	8.95	104.8	109.2	100.5	94.2	107.5	109
10.27	9.47	113.4	97.2	96.9	101.2	94.7	105.5
7.12	8	101.4	114.7	106.9	97.5	98.4	101.4
8.87	9.22	109.2	110.6	110.7	99.9	98.5	99.6
7.12	6.74	88.7	104.4	101.1	115.9	89.8	116.4
6.52	23.15	98.9	104.4	99.8	100.1	93.2	91.6
8.87	13.79	96.8	101.4	99.9	103.7	100.8	104.4
4.82	12.63	97.4	107.6	102.7	104.7	104.6	109.4
9.47	8.41	83	72	71.6	101.3	107.5	104.1
5.66	12.02	100.1	102.5	100.1	94.2	103.4	97.6
4.42	9.77	100.2	101.3	95.9	98.7	106	101
5.03	6.9	96.1	88.8	103.9	97.2	91.1	90.2
5.64	8.21	95.7	90.4	111.3	100.3	112.4	107.6
8.54	6.34	100.4	92.3	97.7	102.3	102.2	109.9
7.56	8.97	95.7	96.4	97.1	101.4	96.3	102.7

7.4	7.14	91.2	86.8	84.1	101.5	91.5	91.5
5.69	13.17	103.9	95.7	128.6	103.2	101	107.5
6.29	11.12	107.6	99.7	95.7	103.2	92.1	98.6
5.8	6.35	98.3	104.2	102	96.2	97.2	104.4
5.3	7.55	98.9	104.7	102.7	86	92.3	98.4
5.08	7.5	102.6	96.7	102.1	108.7	107.4	111.3
6.7	9.87	85.7	95.8	96	88.4	87.3	92.1
6.49	6.74	106.9	104.2	98.6	101.1	96	98.6
8.43	5.46	97.3	94.7	97	106.2	96.2	103.3
6.95	8.64	102	99.7	98.3	94.5	102	109.2
8.65	4.93	98.5	87	100.4	90	99.7	109.9
7.53	4.1	94.6	103.9	112.1	97	109.6	91.6
6.19	9.92	98	102.6	100.7	100	97.5	99.4
7.66	5.01	89.9	102.9	99.2	104.9	105	112.8
6.84	10.3	95.6	90.5	123.7	106.8	115.8	130.3
8.51	8.89	103.7	104.3	108.4	106.1	104.5	99.2
7.46	8	109.8	82.7	97.8	96.3	89.1	93.2
5.24	9.27	84.7	93	98.7	106.7	107.3	99.8
10.15	9.23	100.8	99.6	101.2	105.6	110.8	109.2
8.76	11.29	96.5	92.4	96.3	110.5	112	112.5
4.88	14.05	91	95.5	90.1	100.7	97.1	104
9.25	6.89	97.5	97.1	94.7	99.3	102.7	116.9
8.79	5.95	92.4	86.6	89.4	91.5	77.8	243.8
4.97	6.59	98.2	101.8	91.1	99.9	99.6	100.5
6.33	4.99	100	109.9	113.9	93.4	96.7	92.9
7.33	7.87	95.1	89.7	93.1	108.4	98.7	108.7
7.23	6.28	99.6	110.4	100.6	85.8	96.6	100.8
8.88	5.42	84.6	88.2	93.8	105.8	119.5	103
5.58	8.28	100.9	100.3	106.9	103.9	94	98.4
8.44	7.22	88.2	88.1	91	117.3	110.8	113.9
7.24	9.87	112.9	100.2	106.3	66	63.2	60.8
8.53	8.94	105.5	87.6	100.3	91.5	101.9	100.8
6.74	8.39	88.6	87.7	93.6	99.1	104.9	103.4
6.61	9.45	100	95.1	87.7	90.2	89.2	128.2
4.92	5.73	94.2	94.4	94	71.5	111.6	119.8
7.31	10.15	102.6	103.5	97	91.5	98.6	96.3
9.85	7.53	96.4	86	98.7	96	94.1	108.9
5.68	5.17	128.3	120.1	104.2	101.4	97.8	90.7
5.3	10.92	103.9	101.4	105.5	100.9	97.6	101
6.95	10.12	89.6	90.8	99.1	103.6	102.1	106.8
9.54	6.95	110.7	99.5	103.2	102.2	103.3	102.7
5.27	11.53	103.2	100.8	102.2	103.8	108.8	108
5.88	6.14	105.3	103.2	103.1	97.5	98.1	100
7.99	9.25	89.6	88.2	92.7	122.9	126.7	128.2
6.01	9.12	96.1	106.3	98.2	106.5	114.9	93
7.43	9.96	100.9	98.1	93.5	99.2	101.7	94.6
7.97	11.78	94.8	94	93.7	105.9	106.9	108.4
8.09	7.47	102.1	105.1	111.3	97.7	90.8	94.3
7.55	7.98	115.2	105.7	99.8	110	100.5	91.4
9.5	12.28	102.8	102.4	101.7	110.6	108	106.8
7.93	9.08	101.2	105.1	103.5	98	99	97.4

4.82	6.37	99.2	95.8	102.8	101.1	99.8	102.8
8.02	10.18	102.8	95	98.3	105.4	105.7	100.8
9.14	9.16	109.6	105.8	98	112.7	92.6	94.9
7.12	8.91	93.9	103.4	110.7	94.4	99.5	104.1
4.55	10.33	99.1	95.7	96	108.1	109.1	109.6
6.14	12.65	108	104.3	95.8	103.5	102.3	106.2
7.23	9.93	96.8	98.7	100.9	111.3	100.3	113.4
7.81	12.44	98.6	99.1	94.5	108	109.9	106.7
6.04	10.1	105.2	102	105.6	84.3	108.2	101.8
6.77	9.59	105.8	98.7	103.1	94.1	98.1	95.4
8.34	6.81	101	94.6	97.6	108.5	118.5	121
7.56	8.27	95.9	85.2	95.4	114	139.8	142.1
9.07	6.59	98.2	88.2	105.6	97.2	111	104.4
6.89	7.93	115	109.2	98.6	103.9	102.8	115.3
7.18	10.27	100.4	98.7	98.7	102.4	107.6	105.5
7.56	7.75	83.6	105.3	100.9	97.2	103.3	98.4
5.49	6.95	87.4	97.5	91	107.4	98.7	116.1
8.53	9.14	101.8	99.5	101.7	102.1	104	106.6
5.34	9.03	105.1	102.4	105.4	95.4	90.2	95.8
4.98	11.18	98.8	104.6	97	106.5	96.3	102
7.2	7.9	98.4	98.7	99.5	94.7	98.6	104.8
5.19	8.57	106.3	102.8	111.2	103.1	101.6	104.8
9.69	10.96	106.7	96.5	115.1	94.8	102.1	113
6.51	11.79	102.7	97.5	109	91.3	95.7	100.5
5.49	8.49	100.8	97.5	94.8	101.9	103.4	106.8
6.43	7.51	96.6	100.7	87.2	115.3	100.7	109.2
8.63	8.56	100.7	99.9	105.2	106.1	102.2	99.8
7.2	8.39	93.5	91	79.7	82	78.5	79.8
6.81	9.77	100.1	104.1	100.8	89.1	88	95.9
7.11	11.98	99.9	107.9	105.5	94.3	98.1	90.3
6.55	7.97	90.7	95.3	101.2	107.1	104	99.2
7.03	10.34	101.1	97.3	105.1	97.4	95.9	91.3
5.07	8.43	102.8	88.4	96.9	118	103.3	119.7
9.72	9.23	89.1	90.8	104.7	110.1	116	127.5
7.01	5.52	112.9	121	119.9	90.2	92.9	97.2
8.62	5.96	105	102.3	98.4	104.7	98.5	103.4
5.54	8.87	95.6	96.4	100.9	100.3	103.5	103.1
9.6	10.4	109	100.8	105.9	96.2	99.8	114.4
6.73	9.91	100	99	97.1	102.9	103	101.7
6.74	8.97	101.8	98.4	108	97.2	97.2	93.8
7.37	7.44	114.3	100.9	101.4	98.3	98.2	100.3
4.96	13.1	105.4	104.7	100.2	80.5	95.9	87.2
8.68	10.89	97.5	97.1	98.2	101.4	101.5	100.8
10.1	7.54	95.6	98.2	92.7	105.5	103.4	105.3
5.17	8.76	85.9	92.6	86.3	72	82.7	255.5
9.67	8.82	102.2	104	99	94.3	95.5	103
5.03	8.68	107.1	95.8	92.5	101.4	93.2	112.7
10.32	8.3	103.6	96.3	106.6	109.3	112.2	111.6
6.8	7.42	102.8	94.6	107.2	94.5	112.7	100.3
5.74	7.82	104.7	100.6	101.7	108.7	100.9	107.7
5.67	6.95	102.9	93.3	108.8	89.1	96.9	87.9

6.07	9.16	91.4	90.9	92.8	109.9	110.2	117.3
7.24	7.84	107.8	103.1	102.1	112.6	112.1	105.6
7.17	7.49	96.5	99.4	89	106	94.5	104
9.6	7.33	103.2	101.1	104	107.2	102.9	99.2
4.98	8.22	101.8	95	87.5	100.9	120	110.1
5.01	8.24	95.5	94.4	85.8	125.5	92.1	129.8
6.55	9.02	107.6	104	72.2	110	114.2	107
9.03	12.23	97.3	104.6	100.8	94.8	98.8	99.6
8.02	9.07	109.5	90.2	114	106.9	107.9	106.8
5.43	5.01	105.8	102.7	89.1	124	135.8	129
5.47	11.86	103.2	94.1	100.9	94.5	96.7	97.3
9.35	13.42	101.7	104	103.4	98.9	93.4	97.5
6.25	5.17	99.1	99.6	102.2	95.5	95.4	94.1
8.29	10.12	92.1	103	98.7	90.7	91.7	87.5
6.23	8.04	100.2	101.9	87.6	106.5	91.3	96.3
5.38	18.12	89.3	88.9	85.2	89.6	88.8	81.9
5.27	10.46	103.8	106.2	106.6	99.9	107.3	101
9.26	7	112.5	94.3	102.6	106.7	103.2	105.2
8.91	7.87	101.1	99.9	99.3	87.8	91.9	104.6
5.12	11.44	104.3	102	101	92.2	94.6	102.8
8.72	6.6	105.4	100.3	94.3	104	108.6	95.9
4.48	7.58	105.8	98.2	106.5	119	103.1	99.9
6.39	8.62	105.6	98.6	106.2	103.7	91.5	102.3
8.4	4.63	109.2	94.5	106.4	97.7	95	93.6
9	7.76	110.4	114.4	110.9	92.2	93.8	96.3
7.36	8.09	98.1	99.3	103.5	117.1	107.6	115.5
8.82	12.09	97.9	96.6	93.4	102.2	108.9	102.4
8.25	8.79	105.1	95.8	96.6	102.8	103.5	96.1
8.34	6.12	98.6	99.9	104.7	99	103	100.7
9.54	16.69	96.3	98.4	100.1	98.7	101.4	106.5
10.15	12.47	99	100.6	102.7	103.5	106.5	99.5
5.85	9.63	94.2	100.1	97.4	114	114.1	100.8
7.71	9.23	97.4	97	104.1	108.6	102.4	94.3
7.3	12.23	107.3	92.9	97.3	89.5	89.6	96.9
5.94	8.02	92.8	99.9	77.3	97	82.3	96.1
5	5.83	98.9	79.7	99.6	106.1	105.7	109.9
6.32	16.55	91.3	79.3	104.4	92.7	104.3	99
6.8	14.87	95.5	95.1	94.1	93.6	96.2	103.7
9.04	8.33	89.3	98.6	95.3	122.1	97.8	121.8
5.74	8.45	100.9	91	100	99	102.8	98.6
8.13	8.01	98.4	102.1	94.1	104.8	94	94.3
8.68	8.68	91.4	100.7	105.4	118.1	81.8	137.4
9.09	9.04	101.2	103.3	99.3	115.5	113.4	111.4
7.31	6.76	95.4	96.2	95.9	103.3	98.7	110.2
6.34	9.86	97.6	100.5	99.9	104.4	98	105.9
10.33	11.39	106.1	104.2	108.5	111.1	115	110.2
7.61	6.3	95.1	100.1	111.4	111.6	103.9	105.3
6.38	8.25	96.7	91.6	94.7	111.7	105.1	116.6
5.87	7.13	100.2	92.2	91.3	100.3	103.3	102.6
4.87	8.06	100.6	90.5	118.3	127.8	109	107.9
4.63	3.56	102.8	121.5	104.1	100.8	112.8	101.9

8.66	5.5	106.9	99.6	106.4	98.2	101.8	95.2
8.72	8.78	112.2	102.7	110.1	97.1	99	102.5
7.25	8.82	114.2	109.4	97.1	91.6	95.6	84.6
5.03	6.02	93.7	109.7	89.1	117.4	103.9	108.7
8.88	6.5	108.4	96.5	97.4	106.7	94.4	94.6
4.96	10.76	96.5	100.3	95.9	99.7	102.1	103.3
5.76	10.54	115.5	104.6	110.9	88.1	86.8	92.4
9.66	8.62	108.5	99	101.7	101.5	102	110.2
5.77	10.75	98.4	101.2	100.2	109.4	110.7	115.8
8.03	12	102.2	98.1	92.9	102.2	98.4	100.8
7.56	5.48	112.5	99.7	104.4	99.9	88.5	97.3
8.6	6.59	93.1	95.3	89.8	105.8	99.1	102.5
4.97	5.54	66.8	83.5	97.4	125.2	120.9	117.4
8.6	7.19	99	97.7	93.8	112.5	107.4	111.9
8.76	7.43	95.1	102.7	107.4	104.4	115.7	111.2
5.24	4.4	93.4	112.5	83.9	103	114.1	92.7
9.33	6.32	102.3	101.1	104.2	96.2	102.4	110.7
5.96	4.93	103.7	103.8	102.7	104.7	95.9	100.8
8.24	7.96	94.2	95.3	93.9	95.6	103.6	92.3
8.63	5.3	96.1	92.1	92.9	113.6	126.1	115.3
6.1	6.32	90.7	90.7	91.8	111.5	111	114.4
8.22	5.78	92.2	109.8	108	99.5	84.8	99.2
9.73	6.43	106.3	100.5	109	106.8	111.3	113.3
6.02	14.55	95.7	98.5	96.2	102	98.7	100.8
9.13	13.02	102.5	110.7	99.7	100.3	99.8	96.7
9.17	9.55	133.8	92.7	91.4	98.8	128.7	102.4
8.68	9.56	92.3	112.8	112.9	117	110.9	108.4
8.56	11.98	95.9	92.8	92	110.4	111.5	112
8.18	9.14	91.5	93.4	109.8	85.6	86.4	86.9
5.78	7.93	113.3	109.2	109.2	105.9	93.8	99.8
5.82	9.24	112.8	108.9	103.2	96.2	93.8	95.2
5.06	4.32	108.3	115.6	97.1	87.7	118.7	98.1
5.34	10.11	108.3	103	97.3	70.7	77.4	74.1
5.12	7.1	109.4	99.2	103.7	113.6	115.1	110.5
9.82	9.35	92	88.7	95.5	98.3	101.2	98.7
4.98	7.39	133.4	125.8	123	76.3	75	76.2
7.25	7.54	107	113.1	105.5	89.3	100.4	100
5.07	7.45	112.9	100.1	94.1	95.3	95	102.5
4.4	7.48	104.8	110	95.6	101.7	112.6	106.8
5.24	8.96	102.3	89.2	103.9	112.7	103.9	107.6
5.19	7.91	114.1	107.3	117.3	94.4	86.6	99.5
5.12	5.23	113.4	94.2	103.1	107.1	120.2	109.4
8.03	8.61	122.1	111.3	117.1	97.4	90.8	95.7
6.58	6.33	65.1	86	71.3	120.4	107.6	125.6
5.27	8.3	94.5	106	99.5	98.6	105.4	98.6
8.15	6.37	103.7	106.1	92.8	112.8	103.5	116.1
5.94	12.01	100.3	107.1	105.1	110.1	103.9	105.8
7.61	8.19	108.4	94.6	98.7	100.4	102.5	106.9
6.87	7.01	96.4	107.7	98.9	105.4	101.6	102.8
8.91	10.88	112.1	86.7	105	93.9	100.5	104.9
7.18	8.65	85.3	83	79.5	119.9	114.4	116.4

8.02	4.75	101	104.4	94.1	104.6	97.4	123.3
8.47	8.07	106.4	107.8	100.9	109.1	113	102
4.61	6.93	100.7	95.9	96.6	105.2	105.2	104.3
9.41	10.09	100.2	99.9	105.4	114.5	108.3	112.6
5.29	7.65	100.3	98.3	104	93.8	91.2	101
8.76	8.24	102.6	115.4	109.1	91.8	87.2	90.9
6.32	7.26	96.8	100.3	106.5	95.1	98.9	99.2
5.52	9.21	102.1	92.9	103.3	105.6	106	93.1
7.34	7.45	94.7	88.7	84.4	115.8	113.5	112.8
5.39	6.86	101.4	101.2	109.9	96.6	96.3	92.4
5.59	9.89	107.1	99.5	97.7	99.9	92.6	104.8
6.14	12.24	72.6	151	114.4	116.5	111.8	90.7
8.66	7.86	113.8	99.8	95.5	94.9	84	105.8
4.58	8.23	91.5	98.6	98.7	111.8	106.3	108.5
7.65	9.29	100.4	97.8	104.4	106.5	99.2	101.1
5.47	7.94	106.1	104.5	105.4	103.8	100.4	99.8
7.33	11.16	132.7	131.2	126.7	61.2	62.9	63.1
5.36	9	101.5	100.5	102.1	95.9	101.2	103.9
6.49	3.05	110.4	98.8	110.2	99.9	77.4	95
7.05	8.01	107.8	114.4	109.3	104.7	101.2	105.6
8.54	5.47	109.1	92.1	107.8	110.4	114.1	106.9
5.02	10.23	99.2	98.2	101.4	103.6	100.7	106.6
9.57	16.3	97.8	92.2	105.4	100.9	107.9	105.5
6.04	7.54	124.8	98.6	123.6	82.2	85	84.2
5.25	4.78	113.3	93.3	110.9	106.4	86.4	100.3
6.04	5.51	99.6	105.3	93.9	92.7	92.4	105.4
4.48	7.38	69.3	81.8	84	108.4	113.2	119
7.36	2.93	91.5	94.4	111.6	92.3	116.6	107.8
5.67	3.42	95.9	115.8	122.7	117.7	103.3	104.7
5.41	4.7	102.4	111.1	89.8	92.9	101.5	99.6
8.69	11.2	110.1	109.9	101.2	99.6	105.5	99.1
5.76	4.5	98.5	104.6	91	98.7	113.1	107.1
8.63	6.15	99.1	104.2	88.5	110.4	92.5	105.2
5.47	6.96	101.6	97.3	103.2	100.5	101.8	100.3
9.57	7.17	101.9	102.4	101.4	101.6	103.8	115.1
5.88	7.08	93.8	104.5	91.5	115.7	61.7	118.8
5.41	5.13	107.4	78.8	122.1	85.6	95.4	98.4
10.26	10.11	108.9	88	99.7	99.9	102.7	105.6
11.15	8.99	91.6	110.4	113.1	108	96.7	110.2
8.95	8.77	90.2	90.5	120.1	101.4	97.2	103
7.02	7.67	100.1	96.9	95	110.2	108.3	107.7
6.21	7.28	79.3	74.3	80.7	109.7	107.1	105.3
7.61	5.88	96.8	111.6	94.3	85.4	85.1	76.5
6.74	6.71	98.9	99.2	91.1	89.3	84.5	88.7
8.16	7.06	97.9	95.5	97.8	98.4	103.3	111.6
6.98	7.77	116.1	105.8	111	95.3	99.9	94.9
7.84	7.09	93.7	98.5	103.4	93.1	105.5	97.3
10.56	5.84	109.2	113.8	105.9	91.8	95.4	90.4
7.01	4.22	98.9	92.2	109.8	107.2	97.4	102.7
7.64	7.16	117.9	91.7	107.7	108	107.5	96
9.25	12.91	115.7	99.4	97	105.9	102.3	111.1

8.35	7.07	103.8	97.9	113.1	89.1	92.6	72.5
7.21	13.32	103.8	99.7	100.2	93.8	95.4	97.5
6.04	7.92	108	102.9	107.2	105.8	106.7	106
6.21	9.49	98.5	98.6	95.1	97.8	106	101
9.89	7.28	104.2	92.2	100.9	100.3	105.5	107.1
5.82	7.41	102.3	99.7	100.5	94.7	86.6	88.4
4.68	8.35	103.1	99.7	109.2	107.3	105	104.1
9.2	7.8	96.3	98.7	101.2	103.5	103.1	105.9
5.62	6.68	108.6	97	95.9	118.8	105.8	100
4.44	8.99	90.8	87	79.1	100.2	99.2	94.5
9.26	7.34	103.5	99.5	97	113.1	105.9	105.4
5.57	5.1	94.8	89.9	93.6	105.7	95.9	111.8
7.52	5.86	107.8	102	99.5	96.1	101.8	92.8
6.14	6.51	78.7	91.6	105.5	115.1	156.7	95
6.28	8.03	97.6	100.4	107.3	101.5	101.1	100.1
8.13	6.31	115.5	106	94.5	91.6	91.7	103.9
5.08	10.48	95.7	100	104.9	121.7	117.5	117.5
8.79	8.74	94.2	100.6	91.6	109.5	108.6	115.8
6.87	8.09	92.7	79.5	100.6	115.8	102.4	110.8
6.54	5.12	93.2	82.1	97.3	119.3	110.1	127.6
5.73	7.11	93.1	87.6	95	101.6	101.6	108.6
5.38	5.24	105.5	110.5	115.2	80	83.5	82.4
8.02	8.16	101	91	101.7	99.8	94.6	98.9
7.58	8.86	119.1	92.4	100.1	133.3	129.8	92.4
5.64	6.31	96.5	92.5	98.4	102.3	106.4	103.9
8.72	16.5	94.6	106.7	103.3	99.7	91.2	103.6
5.08	6.02	106.8	101	103.1	109.7	102.8	103.5
7.15	6.96	107.3	105.6	106.9	100.9	100.8	96.7
8.98	4.49	96	98.5	87.6	107	108.7	105.6
9.67	5.97	94.2	110.9	95.3	133.2	133.2	123.2
6.9	7.16	101.2	96.6	92.4	105.5	103.2	93.3
5.55	5.91	108.3	106.9	106.6	104.7	110	106.1
9.7	5.45	106.8	128.2	85.1	88.9	119.8	88.1
9.64	6.85	96.9	117.5	100.5	106.8	106.7	103.5
5.43	6.93	103.6	90.2	89.8	83.3	91.2	79.5
8.76	2.8	99.8	94.6	94.9	112.9	106	104.7
5.68	7.17	100.4	111.2	106.5	87	86.2	86.4
5.21	4.36	99.7	103.2	85.6	77.9	89.2	103.7
6.09	6.89	96.2	88.6	98	108.7	110.3	100.6
6.64	7.11	91	96.7	102.2	114	115.3	116.8
6.65	9.4	90	90	94.4	124.2	123.2	122.3
8.38	7.85	105.8	108.4	105.5	115.6	121.4	116
5.73	8.11	102	96.4	93.9	107.6	100.6	103
6.35	7.76	103.6	97.1	85.9	105.9	107.1	108.5
4.37	7.13	106	96.4	103.1	100.3	101.1	103.4
6.29	5.52	91.6	105.2	97.3	99.6	100.9	87.6
6.33	7.66	87.9	99.4	120.8	101.3	98.5	134.6
9.31	8.21	98.4	103.6	109.3	97.7	101.9	108.9
7.28	8.55	86.1	95.1	96.3	96.6	91.1	103.7
5.85	6.01	101.9	97.1	94.4	89.8	117.1	99.4
6.1	5.28	104.2	99.8	123.9	92.4	104	94.1

8.02	6.34	110.6	100.5	107.6	102.2	93.5	91.9
8.94	10.27	96.3	100.9	107.3	98.9	97.4	97.6
5.3	6.7	90.3	94.8	102.3	119.9	113.5	105.3
6.07	5.33	104.8	89.4	94.1	98.2	96.9	106.1
11.18	8.06	88.8	86	88.2	111.2	111.1	106.7
8.24	7.93	99.8	98.2	96.7	104.8	103.6	102.9
6.37	8.06	107.6	97.2	93.8	100.4	95.9	120.7
9.25	6.57	102	101.1	99.8	93.9	104.4	92.5
9.11	10.11	93	99.4	91.6	105.8	107.8	103.2
5.45	5.53	108.5	93.2	90.1	107.8	108.5	105.3
5.44	8.58	92	98.2	101.2	97	98.2	103.1
7.83	18.99	99.8	98.3	96.3	99.1	97.4	93.1
6.39	4.61	87.3	73	100.1	76.6	105.4	94.4
8.02	4.97	106.7	102.1	102.9	101.8	110.3	111.7
4.23	7.64	105.9	121.6	114.3	134.3	138.4	131.6
5.07	6.83	73.8	74.1	71.6	115.6	121.4	118
6.48	4.73	75.8	105.2	89.8	113.4	116.7	108
5.54	6.86	101.8	106.6	107.9	93.7	90.7	99.8
6.83	6.94	100.6	102.5	114.4	71.3	80.9	85.5
6.23	5.99	89.3	95.6	101.4	114.1	102.6	118.8
9.48	6	132.3	87.1	102	97.5	144.2	99.9
8.46	6.75	98.7	110.1	108.7	95.8	95	88.4
7.23	9.5	90.1	115.1	109.6	100.2	106	107.5
6.3	6.27	110.2	92.7	82.6	115.6	100.8	106.2
9.2	18.2	97.3	98	102.7	102.4	101.5	106.7
8.81	6.58	98.4	107.4	91.2	105.9	94.6	95.1
11.96	7.72	88.3	102.3	104.7	99.3	98.4	106.7
5.47	4.31	105.5	96.4	102.3	114	108.9	114.5
6.62	4.75	92.3	94.9	94.9	92.9	93.3	98.5
7.53	6.13	104.7	90.5	109.8	103.9	106.1	96.3
6.27	5.4	86.3	106.3	93.8	110.4	99.5	124.3
9.17	6.66	93.2	114.8	97.6	121	115.9	108.5
9.22	9.53	101.8	96	98.8	104.9	100.2	103.2
8	6.17	109.3	110.3	93.8	108.1	105.6	105.9
5.03	5.74	108.9	104.7	99.7	100.2	113.2	123.6
5.68	7.72	79.2	115.1	107	114.4	113.9	119.3
6.44	7	90.1	105.5	98.4	107.5	111.2	117.8
7.28	6.86	102.9	112.8	94.9	103	88.8	99.8
6.28	5.73	103.8	90.2	93.6	108.8	117	118.6
4.64	7.68	104.2	103	95	101	101.2	99.1
7.71	7.22	100.9	93.2	113.2	99.5	97.7	96.9
9.51	7.88	106.8	101.1	102.4	113.4	105.5	112.4
7.88	7.6	95.2	107.2	99.8	103.9	114.9	107.5
8.21	6.07	98.1	85	95	101.8	107.8	122.8
6.64	9.64	103	96.5	95	108.9	104.8	102.9
10.95	6.26	100.9	100.7	101.9	105.7	104.2	116.3
6.83	8.2	104.9	88.6	87.9	116	115.1	103.6
5.95	5.8	80.8	94.5	95.1	94.3	109.1	98.7
7.18	6.71	99.1	96.5	99.2	109.6	99.7	99.4
9.73	8.7	93.3	98.3	103	112.8	105.3	108
5.96	8.35	94	105.5	104.1	106	107.3	107.7

6.61	7.63	81.1	87	92.2	109.2	109	112.4
8.1	7.2	90.6	93.2	89.5	114.4	102.8	114.6
10.08	6.33	130.9	114.8	122.4	90.8	96.2	100.4
7.96	4.87	91.3	92.8	99.9	101.7	108	103.8
4.74	6.85	103.3	104.1	109.3	104.4	118.3	123.8
9.92	14.44	104.7	101.6	104.8	96.1	98.5	95.3
8.38	8.8	103.9	102.1	98.8	96.7	99	104.4
4.96	8.86	101.9	92.3	96.5	93.6	95.7	100.9
6.96	6.89	112.8	92.1	96.8	111.4	84.1	77.7
6.61	4.66	111	100.8	115.9	100.4	90.8	107.2
9.39	8.69	84.2	101.5	90	125.6	121.2	114.7
5.52	9.14	101.6	106	101.5	103.7	120.7	99.3
7.18	7.94	102.2	99.2	101.2	88.2	96	99.4
5.33	8.66	94.6	99.6	99.5	100.2	99.8	99.6
8.92	9.29	105.9	109	88.1	100	91.4	95
8.02	5.44	109.9	90.3	98.6	93.7	83.6	104.3
7.42	6.57	99.7	98.7	103.6	128.2	125.2	124
7.55	7.11	91.6	98.6	104.9	104.2	99.3	105.9
6.65	5.2	87.9	84.6	100.4	103.2	88.2	99.1
5.99	6.85	102	99.1	102.7	86.5	86	105.5
5.33	7.74	103.2	84.8	101.5	91.8	98.3	98.4
4.97	7.91	98.6	99.2	95.4	115.2	116.4	119.4
9.55	10.18	107.4	104.4	105	108	112.8	106.3
8.47	6.64	98.8	94.2	97.2	111.2	106.1	119
9.76	7.6	95	113.9	94	113.2	104.8	102.9
6.34	7.22	101.8	94.7	98.7	89.2	105.4	120
5.74	7.4	78.7	78.9	79.4	106.5	103.4	99.3
6.98	6.43	112.6	74.4	89.4	95.3	123.3	114.1
7.59	2.89	94.1	91.9	106.4	103.1	91.1	95.8
7.02	8.97	104.4	109.7	106.7	96.4	90	97.6
8.76	7.83	103.1	107.2	112.3	103.7	114.2	103.1
8.34	8.51	83.7	78.9	94	111.8	109.4	134
9.36	11.56	115.2	112	116.8	99.9	101.3	100
8.66	5.62	93.5	99.6	100.7	91.9	105.3	96.2
5.52	3.84	82.3	91.7	113.5	104.3	101.1	111.3
6.6	8.61	97.3	94.9	99.4	100.8	97.5	102.7
5.33	6.28	106.6	101.7	105.7	87.5	113.3	113.5
5.94	5.57	112.9	92.5	105.1	92.8	96.2	102.7
7.53	7.78	97.2	99.4	100.3	111.1	113.6	105.3
7.44	20.18	119.8	119	136.7	80.1	76	85.9
9.32	10.35	105.1	110	104.3	111.8	106.4	113.4
5.59	7.96	73.8	77	74.4	125.4	119.8	116.9
8.6	6.84	105.1	89.4	102.2	110.1	90.4	135.9
5.72	6.25	100.3	95.8	109.5	88.7	108	102.8
9.07	2.85	104.3	97.2	100.8	97.6	108.3	94.8
11.3	6.59	106.6	91.8	103.4	103.6	104	108.3
5.29	7.33	114.6	117	117.1	88	93.5	100
8.13	7.95	99.8	106.1	100.5	102.5	103.6	110
5.15	6.91	86.6	100.5	109.8	101.5	108.4	102.6
7.65	7.82	94.9	84.6	110.4	87.6	110.3	92.8
7.99	6.74	95	98.2	95.1	109.3	112	112.1

5.69	11.22	97.3	101.9	104.4	106.9	111	112.2
4.63	5.94	113.1	103.1	90.6	115.5	111.1	102.8
8.84	5.99	99.1	98.5	105.7	93.4	67.3	108.5
7.84	11.39	97.2	92.8	106.5	89.5	103.5	96.1
5.87	8.34	99.6	91.4	95.8	98.9	100.9	97.4
6.09	9.79	104.3	106.2	108.7	103.7	104	99.9
6.92	5.05	92.3	104	91.6	109.5	112.4	125.4
9.11	7.91	106.2	102	97.2	103.2	106.8	108
5.33	5.32	97.3	94.4	109.4	95.1	122.4	101.2
9.55	8.8	94.8	98.9	98.9	114.4	110.7	110.4
6.79	7.79	89.3	102.6	99.2	95.9	97.5	99.4
9.54	9.3	100.5	99.7	103.2	101	104.1	108.3
7.09	7.7	96.8	103.3	108.7	108.6	102.9	107.4
6.52	3.49	112.7	107.6	94.6	90.9	118.8	87.8
6.73	6.2	104.4	104.5	96.9	99.6	103.6	100.6
6.44	8.07	112.9	100.7	106.6	109.4	113.3	114.9
7.36	5.15	93.7	101.2	105.4	97.9	91.4	91.2
6.07	4.11	99.6	95.4	94.9	94.7	100.2	89.4
8.82	6.67	105.4	111.8	91.4	90.4	96.1	99.1
5.92	8.51	111.6	101.5	100.5	111.3	117	113.9
8.28	5.4	114.8	106.5	87.2	96.8	106	97.8
7.58	6.93	100.1	104.9	92.9	98.4	100.2	131
7.03	5.78	97	100	97.5	98	95.8	97.6
6.92	7.25	95.8	92.7	91.3	102.6	98.3	114.6
7.34	5.46	104	96.8	85.5	103.3	112.7	119.2
6	3.54	86.8	124.5	112.9	83.6	90.4	103.5
9.03	6.21	104.3	103.1	90.7	111.3	109.9	99.2
6.83	7.1	97	93.5	101.8	87.4	100.8	101.2
6.09	5.51	90.1	96	103.9	101.2	107.4	103.6
6.89	6.25	103.7	101.7	104.9	89.5	105	111.3
8.46	3.08	96.5	105	107.9	98.4	100.7	103.4
5.76	7.32	108.3	86.8	99.7	100.3	102.9	98.3
9.22	5.55	102.7	104.6	106.2	106.4	104.1	109.9
8.87	6.53	103.6	95.4	103	100	109.1	107.3
5.26	6.96	87.6	102.3	89	99.6	95.5	104.3
6.21	6.24	79.8	104.4	102	90.9	98	94.1
5.31	7.9	93.4	99.9	96.4	104.4	106.7	108.1
4.92	1.9	120.6	101.1	71.3	119.5	112.5	107.9
6.71	4.71	98.8	103.2	99.7	110.6	97.3	102.3
5.6	6.31	91.5	122.3	82.5	102.4	96.7	125.9
6.8	4.19	110	110.1	111.1	102	106.3	98.8
7.21	6.47	97.7	86.5	89.5	103.6	99.3	100.3
5.68	7.22	92	100.9	97.4	103.3	98.4	101.7
8.1	5.33	101	92.5	98.6	99.4	102.7	99.8
8.78	6.42	92.6	97.3	97.7	97.8	102.3	132
5.76	4.64	104.8	101.6	99.3	99.7	103.2	104.5
8.05	6.11	105.9	83.2	96	102.2	99.1	114.6
4.93	5.67	91.4	94.9	105.1	97	100.7	90.9
6.14	6.88	98.9	93.4	100.1	99.4	95.2	100.9
9.57	5.9	111.8	103.6	97.9	96	91.7	95.3
5.08	5.12	101.5	97.4	92.5	104.7	101.2	108.8

6.4	5.49	97.1	100.9	98.3	97.9	109	114.6
7.64	3.14	118.6	92	87.7	91.3	99.6	115.4
5.69	3.09	106.1	93.8	114.9	105.7	98.2	100.5
5.77	7.48	108.7	102.4	109.4	94.2	101.3	102
5.25	6.37	97.4	105.6	110.1	110	104.7	112
5.19	4.29	119.5	117.4	120.2	90.8	86.2	89.4
10.35	6.76	103.3	108.7	102	103.7	95.9	102.3
7.15	1.81	74.9	111.6	93.7	118.8	99	96.9
8.15	7.07	98	88.6	102	107.5	89.8	114.6
4.65	7.59	87.8	99	105.7	114.5	101.6	112.1
8.88	9.59	98.7	112.2	112.2	97.2	119.4	99.1
6.28	5.4	82.1	109.2	104.9	98.6	120.3	109.1
6.13	5.8	81.1	81.9	84.2	94	93.6	88.1
7.88	5.18	88.1	92	118.6	97.8	90.7	103.4
6.21	7.5	106.9	108.5	101	106.7	101.8	102.4
7.85	6.17	80.4	110.3	105.2	113.1	97.5	99.6
5.11	8.62	99.5	89.9	94	111.1	101.4	110.5
7.36	3.2	100.3	124	111.2	103.1	109.2	103.4
6.64	6.7	101.9	97.7	101.4	96.4	101.9	100.9
7.06	6.2	102.6	93.5	99.1	110.8	103.6	98
5.99	5.57	113.2	92.6	96.1	96.6	92.4	96.5
5.34	5.85	114.8	95.5	86.8	105.8	95.9	113.8
5.77	4.6	76.7	74.9	82.7	108.5	143.2	124.9
6.28	5.79	102.4	105.4	91.7	106.7	102.9	106
8.32	4.34	104.1	99.3	107.4	108.6	97.6	95.3
6.71	4.18	98.7	91.8	100.4	99.2	136	112.1
11.08	5.5	107.8	91.5	113.8	112.6	101	105.1
9.48	8.9	111.4	106.9	107.8	123.8	122.5	113.9
5.33	6.33	76	70.1	54.1	138.7	136.4	138.3
9.54	6.35	91.2	95.1	108.7	100.1	98.2	103.8
8.29	11.87	95.9	98.8	96.3	107.6	114.4	111.6
9.03	2.42	103.5	108.8	123.1	87.2	96.4	80.2
9.98	6.56	99.2	112.5	111.4	101.5	111.6	100.5
9.57	7.57	95.7	95.3	99.5	101.2	105.6	106
5.69	7.47	110.4	91.2	101.3	106.4	100.8	98.6
9.38	4.62	104.9	99.3	105	84.8	104.6	102.9
8.97	6.44	88.9	90.6	84.9	84.2	71.4	88.9
8.88	2.76	103.9	106	98.4	110	107.6	106.6
5.15	7.77	120.5	118.2	110.6	85	78.3	87.6
9.26	7.86	96.6	107	107.6	96.1	98.8	101.3
5.12	6.61	103.1	101.7	97.3	108.2	91.1	97.1
7.85	4.17	82	79.2	76.6	97.1	98.4	97.6
6.73	8.09	96.4	99.1	108.4	102.5	95.5	94.7
8.37	8.21	100.7	100.1	94.2	107.3	103	103.9
5.07	6.76	103.6	104.3	109.2	115.8	122.6	120.1
6.13	9.94	85.4	89.5	86.8	65	58	56.2
6.6	5.21	72.6	48.7	69.9	140.9	117.9	101
9.55	6.29	101.4	101.3	113.2	100.5	77.3	101.6
6.57	5.41	90.7	91	82.8	120.3	124.6	120
6.11	6.31	90.2	107.6	111.6	89.6	91.4	99
9.82	4.76	106.2	91.3	106.2	105	102.1	106.8

6.54	4.2	118.6	78.2	112	108.8	100.4	122.4
6.38	4.86	92.5	91.9	99.9	91	95.5	95.6
8.59	5.71	103.8	97.1	96.4	110.6	97.8	109.5
5.11	7.54	103.2	100.7	94.3	97	99.2	102.8
7.4	3.18	71.9	68.1	66.1	80.8	83.4	101.3
8.76	7.37	109	110.4	103.4	103	98.5	106.9
6.95	6.8	114.8	89.9	102.6	97.7	97.3	93.4
5.59	4.54	94.2	81.4	108.8	95.5	106.1	133.5
8.06	7.39	107.7	106.7	90.1	98.9	98.1	101.8
8.02	7.36	102.4	105.7	95.8	114	96.6	108.5
6.58	6.53	105.6	99	87.8	100.4	110.8	102.1
6.95	6.35	96.8	101.9	98.4	103.1	104.5	108.5
9.73	7.08	91.4	105	100.9	113.7	102.7	120.9
4.7	8.28	94.9	84.2	88.7	109.4	116	115.7
6.48	6.41	102.4	110.2	98.5	105	107.5	104.4
9.83	4.76	102.2	63.5	114	105.5	94.2	131.7
5.92	6.37	110.6	105.4	103	84.8	86.6	97.5
7.4	4.22	107	105.9	98.9	97.8	102.9	106.9
8.35	6.73	109.2	106.4	110.7	104	95.9	94.2
5.85	5.77	106.1	97.9	104.8	109.9	105.7	111.9
10.1	6.83	102	101.9	91.2	114.2	102.8	112
5.6	3.72	110.5	117.2	99.9	80.5	76.8	85
6.23	6.74	107.6	101.7	100.9	104.2	97.7	95.3
9	7.79	96.5	102.5	103.8	107.6	120.5	117
4.92	6.09	95.7	89.7	90.5	106.1	104.7	108.4
4.75	6.46	87	82.3	81.4	117.8	118.6	136.1
9.04	5.24	108.7	93.4	96.3	94.6	100.6	112.8
8.27	5.19	99.6	92.2	101.9	91.3	89.8	96.4
5.59	5.96	95.7	97.5	101.4	99	101.1	97.1
5.43	6.8	119.1	116.8	114	97.5	99.7	101.5
10.07	3.99	102.5	106.8	96.4	114.7	92	98.7
6.55	5.88	98.4	102.2	105.3	102.4	106.3	98
5.87	7.96	101.1	106.4	109.8	107.2	112.8	108.3
4.65	4.76	90.7	90.3	97.3	128.1	119.6	113.4
6.19	6.65	97.6	100.2	102	107.8	104.6	110.9
5.54	7.67	93.7	90.1	93.3	119	115.7	116.4
9.36	8.73	98.8	93.6	97.2	91.8	89.5	93.2
5.35	6.68	97	95.8	98.3	97.9	92.2	89.5
6.52	7.88	96.3	98.2	109.7	106	104	104.8
6.34	6.51	99.5	98.6	101.7	103.2	101.9	107.4
8.28	5.6	104.1	96.6	102.6	139.6	147.6	147.2
6.74	4.98	101.6	93.6	92.3	101	104.3	97.8
8	5.56	93.9	111.8	75.5	90.3	116.3	115.1
7.3	7.65	98.9	95.4	100.8	92	85.9	104.5
8.1	5.37	114.8	113.9	95.3	108	121.3	102.4
8.73	5.84	76.4	110.1	77.8	112.7	114.9	107.1
7.42	6.81	103.2	91.8	90.6	89.5	110.9	98.5
6.87	6.62	119.6	91.6	87.6	87.7	133.6	100.7
6.4	8.21	90.3	105.8	99.6	94.7	88.9	98.2
8.59	8.9	95.9	104	106.8	94	94.4	92.8
9.17	6.81	103	103.3	107.6	109.9	111.9	106.9

5.99	5.02	114.3	109.2	96	92.3	86.5	101.6
6.16	5.47	94	100.2	108.3	105.9	104.2	116.8
8.6	5.48	100.7	105.9	103.7	100.5	97.5	99.6
7.58	5.63	101.2	85.5	109.4	90.7	104.2	102.9
6.99	5.39	111.2	86.5	102.4	97.3	111.1	108.2
8.84	5.33	105.7	109.3	98.4	99.7	110.6	97.8
7.55	4.5	98.6	101.4	103.1	103.5	112.7	119.3
6.06	5.91	83.1	94.3	120.6	115.6	68.5	115.2
7.02	5.83	109.5	99.6	91.7	100.5	86.3	94.3
4.82	5.6	91.6	104.3	83.6	101.7	95.1	104.7
5.85	12.1	102.7	100.7	97.6	96.6	100.5	98.5
6.05	4.14	115.8	121.9	113.5	106.7	96.2	96.3
9.67	5.12	96.5	88.1	112	98	103.3	100.5
7.18	7.35	97	95.8	103.5	105.6	102.5	108
7.18	5.31	103.3	84.8	91.2	101.6	94.5	121.6
8.37	3.9	94.2	96.8	93.5	115.7	106.4	111.7
9.51	8.48	106.3	106.6	98.4	91.1	100.4	93.8
6.9	5.53	103.7	95.3	102.1	88.8	101.1	106.1
4.93	4.85	103.4	105.2	96.1	95.8	103.2	96
6.83	7.49	100.4	93.3	95.9	103.2	95.2	88.4
6.06	4.87	100.3	106.5	108.6	96.8	94.3	99
4.91	6.57	105.6	107.3	89.2	109.6	110.3	99.5
6.14	6.71	102.8	89.6	99.6	110.8	106.1	108.5
5.8	5.81	100.5	106.7	115.6	97.8	100.5	103.2
7.02	15.72	116.2	95.7	100.2	95.8	107.5	95.9
6.68	4.81	107.2	115.1	65.9	91.5	87.8	70.7
5.19	7.03	103.2	110.4	94.6	110.1	102.7	101.4
10.48	5.59	105.6	99.9	92	92.5	93.3	100.8
7.03	5.88	77.4	115.4	107.5	123.6	103.1	94.2
11.55	5.97	118.1	109.9	114.4	108.4	116.4	115.3
9.96	4.99	105	108.6	105.9	111.6	92.4	106.3
6.54	3.27	96.2	92.6	115.4	104	87.7	114.4
4.75	5.48	112.2	94.9	88.9	87.8	91.4	110.5
9.11	5.73	96.1	104.4	88.8	107.1	88.2	99.2
9.58	5.17	116.4	96.2	107.1	100.8	105.4	97
5.8	3.96	107.4	99.4	100.6	95.9	104.9	110.2
7.43	6.11	90.6	101.7	95.3	102.8	109.2	105.8
5.96	5.32	96.9	88.6	102.6	96.9	98.3	105.3
7.88	11.64	100.1	95.6	104.4	96.2	95.2	100.2
6.71	4.32	90.2	96.2	100.4	122.2	120.6	122.4
9.64	5.97	94.3	97.9	94	106.3	101.1	95.6
5.69	6.31	120.6	70.7	99.4	91.5	98.1	91.9
7.46	6.13	115.8	99.6	97.6	96	98.2	112.1
5.73	3.94	118.3	99.7	115.2	75.9	92.7	120.1
5.48	8.41	96	97.5	100.5	127.5	126.2	121.1
6.28	9.07	98.2	98.3	96.6	103.8	99.1	106.9
7.03	6.55	95.3	93.8	87.3	150.4	96	116.9
4.84	6.07	89.7	109.6	104.5	94.8	102.1	98.6
8.27	5.85	128.7	85.1	101.9	87.3	101.7	106.6
8.76	5.42	104.5	103.2	110.4	106	104.4	99
6.42	2.57	98.3	94.9	90.5	106.1	99.8	114.7

8	4.26	97	101.1	105.9	121.6	132.3	111.6
4.59	4.92	104.7	95.4	91.2	104	98.3	106.3
9.01	6.28	91.5	94.3	99.5	98.4	98.2	110.9
6.65	5.03	109.1	116.2	101.8	109	96.6	105.2
7.14	5.58	103.3	105.7	96.5	89.3	86.2	85.9
5.64	6.03	87.3	83.6	94.3	94.5	121.9	108.3
4.37	9.44	102.5	91.3	100.9	95.7	98.7	92.7
6.11	3.87	112	102.5	91.7	98.9	102.7	101.1
6.67	3.18	97.4	102.3	107.5	101.2	98.2	113.2
10.37	7.38	87.5	97.5	90.8	103.4	103.5	118
7.06	5.62	96.6	96	93.5	112.1	109.2	116.6
8.28	5.7	107.5	88.3	106.8	110.3	98	95.4
5.48	3.51	86.5	93.4	105.1	99.3	65.5	78.2
7.77	4.99	82.1	108.8	98.9	106.4	83	91.7
7.69	5.11	97.9	93.8	98.1	103.5	92.1	104.9
9.1	6.12	85.3	75.2	144.9	90.3	186.5	98.7
6.67	4.87	96.8	100.9	100.9	113.2	99.6	94.7
5.86	4.81	89.7	121.3	119.6	103.5	111	102.3
9.19	5.59	101.7	98.9	97.3	101.9	103.4	106.5
7.11	5.77	104.8	106.5	104.9	104.3	111.1	104.2
8.18	4.96	108.7	109.7	109.9	120	114.7	89
6.99	5.23	101.1	108.9	97.7	102.6	90.6	97.6
8.21	2.59	89.9	101.9	76.8	110.1	116.4	104.9
5.73	2.2	117.7	98.9	109.4	81	82.7	85.5
6.48	6.82	109.9	93	101.6	107.8	95.4	97.6
6.25	3.97	93.5	96.6	99.9	114	100.5	99.3
6.32	6.78	100.8	105.1	100.6	102.9	101.2	107.9
6.6	6.73	97.4	100.3	107.5	99.6	93.6	98.7
6.44	5.94	109	88.9	113.1	100.2	88.3	101.6
6.29	5.66	98.3	97.9	103.2	93.7	97.7	110.4
7.02	5.04	98.7	93.4	99	82.9	91.2	96.8
8.73	4.73	102.3	104.1	89.7	94.8	95.3	101
4.93	5.43	88.7	92.2	90.8	117.1	107.8	100
8.54	2.59	107.1	87.9	78	104.4	103.6	110.5
4.89	4.81	90.9	106.3	97.4	89.4	84	108.5
6.2	5.92	105.4	94.2	99.6	87.8	84.4	99.3
7.88	4.11	105.7	94.9	94.1	99.1	97.8	99.2
4.7	5.58	99.7	88.9	86.5	102	106.1	94.3
5.73	4.48	76.2	91.9	100.6	148.9	108.9	114.8
7.77	4.72	126.6	113.2	127.7	97.6	94	62.3
6.61	3.59	95.7	84.8	91.2	105	111.4	99.1
5.03	6.11	105.8	91.8	100.3	118.2	113.9	117.2
7.37	4.15	94	99.5	106.8	109.2	125.5	117
4.97	5.86	95.1	86.5	116.1	119.7	104.3	84.3
6.23	4.61	100.7	88.9	109.8	89	88.7	119.7
7.77	9.28	97.9	102.4	105.4	90.1	86.1	94.8
6.54	5.74	100.4	92.3	110.6	105.6	104.5	104.6
6.4	5.08	104.1	89.3	89.2	111.1	112.8	111.6
7.2	2.86	95.7	99.5	105.4	84.3	97.9	96
10.7	5.53	100.8	101.7	98.8	118.5	113.3	117.6
4.89	6.51	105.4	110	100.3	100.4	97.2	105.4

5.27	8.33	87.6	95.3	91	107.9	105.2	109.1
5.4	2.16	135.3	100.5	93.7	92.5	114.9	111.1
6.51	6.76	93.1	94.3	97.5	93.9	107.3	104.4
9.63	5.64	97.5	99.1	99.1	107.5	106.7	107.2
12.15	12.34	91.6	94.3	97.1	107.6	106	106.3
7.05	4.87	86.7	78	87.2	120.6	128	127.8
9.57	6.16	108.6	93.3	93.6	100.8	94.7	99.1
8.65	5.72	90.5	88.9	100.2	86.4	94.7	97.7
6.73	3.53	109.3	114.6	104.1	104.4	117.7	101.8
8.47	6	97.5	104	96.1	97.9	96.6	104.4
9.67	6.37	107.4	100	106.1	88.8	98.1	87.9
6.46	3.57	157.5	71.2	83.5	79.9	81.8	96.2
6.43	4.04	102.5	101.4	99.4	99.2	91.4	101.8
6.18	4.1	103.8	89.2	99.6	93.3	99.6	104.9
10.08	7.55	108.7	102.5	94.5	108.9	105.9	103.7
8.25	5.23	103.7	106.3	103.5	113	92.1	96.1
4.39	4.89	102.8	102.4	96.8	107.8	94.9	96.1
5.39	6.03	136	89.4	87.8	91.3	87.1	93
8.34	4.48	104.2	97.7	101.1	116.9	116.9	93.3
8.22	5.38	104.6	92.2	98.2	74.5	79.1	84.3
8.18	4.23	97.1	89.3	110.1	110.7	104.6	116
7.25	5.14	95.5	111.1	88.6	98.4	96	100.6
7.78	5.05	113.9	105.8	106.4	114.9	112.9	104.7
6.19	5.35	110.4	113.4	94	80.4	95.6	100.7
8.72	5.68	105.8	100.9	98.1	101	90.8	92.9
6.95	3.52	109	114	97.6	102.6	103.1	85.8
8.98	5.65	106.7	95.3	107.7	110.5	99.7	106.1
5.29	5.23	98.7	97.9	109.3	102.7	104.2	103.8
5.71	13.59	86.9	74.4	102.7	100.9	95.5	98.7
9.5	5.58	104.8	77.5	92.9	118.4	122	127.9
9.95	6.11	103	95.5	99.5	111.2	97.9	112
7.14	5.31	90.9	104.2	108.9	88.6	109	93.4
6.62	3.92	98.2	101.2	100.8	94.8	87.7	104.2
6.55	5.39	109.7	95.3	111.8	102.3	81.3	89.2
4.75	5.73	96.5	101.9	100.4	99.4	124	106.4
8.75	4.81	103.8	99	112.6	86.1	90.7	89.5
6.19	8.69	94	96.9	98.9	103.4	98.4	103.6
7.52	5.07	110.6	99.4	102.8	101.6	107.1	99.8
4.68	4.7	110.7	103.9	117.2	65.8	78.4	70.2
4.73	4.59	94	94.5	95.7	94.4	98.1	100.8
7.71	5.08	97.8	96.2	84.1	99.1	101.7	95.8
5.72	6.07	89.7	94.3	85.3	62	65	63.3
8.29	4.2	100.1	98.1	96.6	108.8	102.3	95.6
5.6	4.97	120.3	97.3	93.5	104.6	90.9	109.1
8.94	3.02	107.5	94.6	100.9	94	108.5	105
9.22	2.76	98.2	96	74.5	104.3	99.6	103.6
8.5	2.07	125.3	105	115.7	89.9	105.9	97.6
5.07	6.23	102.8	97.1	90.9	101.5	105.1	114.6
9.36	6.17	119.8	98.9	105.2	91.6	88.3	89
4.79	2.94	108.7	105.2	101.3	89.1	79.8	77.1
6.02	5.77	93	103.8	99.4	100.3	102.4	108.9

7.21	3.77	126.2	87.8	80.2	121.4	102	98
7.25	2.22	101.7	104.5	100.1	98.2	95.1	90.2
6.93	2.38	94.1	88.8	103.1	100.7	98.3	103.7
7.03	4.69	95.3	85.1	81.6	44.7	53.2	49.9
9.73	5.71	101.7	102.7	96.4	101.7	99.8	104.8
6.55	4.53	100.3	98.6	98.9	107.6	108.5	108
5.21	7.09	100.5	101.1	109	88.5	85.5	86.4
6.83	5.04	106.9	92.6	100.1	98.2	100.9	108
8.72	4.98	101.9	100.3	104.9	94.1	95.3	97.5
5.82	5.48	84.3	107.4	103.2	101.6	101.5	104.8
5.48	3.31	89	83.7	93.3	102.9	114.1	118.7
6.54	3.04	106.4	97.5	102.9	102.6	97.2	96.8
5.16	4.47	98.3	110.1	97	109.3	115.8	103.8
7.21	3.01	86.2	82	95.6	102.1	106.2	107.9
6.21	4.65	81.6	100.5	100.3	93	94.1	95.3
9.76	5.12	102.5	109.8	94.6	114.4	112.6	113.3
7.01	5.79	101.4	83.1	111.3	59	98.7	93.8
6.83	3.05	91.2	100.5	104.2	119.9	115	119.2
7.23	3.64	83.6	92.3	106.8	101.7	110.9	107.3
6.89	4.47	111.9	110.3	104.2	91.8	99.3	90.8
5.43	6.54	97.7	90.9	104.1	98.4	97.1	101.8
6.61	5.99	96.8	96.9	93.8	96	92.5	96.2
9.13	2.3	113.8	113.5	111.7	111.3	105.1	103.5
6.8	5.47	90.6	97.3	95.4	112.8	110.4	105.4
8.41	4.79	101.8	104.8	108.2	116.3	105.7	106.9
9.86	3.25	117.4	112.8	89.1	99.8	101.4	100.8
4.75	6.04	96.9	95.2	102.2	128.8	123.8	121.2
6.95	5.62	78.5	74.1	62.5	97.3	82.4	89.5
8.62	5.81	107.9	101.5	103.1	99.1	89.1	96.3
8.92	4.93	95.1	98.4	114.9	98.4	98.3	104.6
8.41	5.78	99.6	102.9	98.8	100.1	99.5	109.8
7.39	2.27	103.3	100.2	103.7	123.1	116	101
5.86	4.27	114.6	84.5	126.1	106.3	80.3	87.4
6.76	3.3	100.5	86.5	92.6	100.6	81.2	102.8
6.43	5.88	109.7	115	109.6	77	83.8	95.8
9.07	5.6	110.8	106.3	98.3	139.2	127.1	123.1
7.36	4.87	100	100.8	105.5	98	82.7	98.8
6.62	4.98	93.4	105.5	95.2	102.3	96.1	94.2
10.45	7.93	94.2	94.3	98	100.7	99.6	104.5
9.99	6.75	110.3	98.1	112.1	96.8	108.3	108
6.6	8.06	99.7	104.8	101.8	102.4	100.2	104.5
5.92	3.04	105.8	95.3	100.6	111.3	97.9	97
8.69	2.6	166.8	85.1	108.1	103.4	87	81
7.11	2.66	95.1	100.3	86	110.4	100.8	102.6
8.66	2.02	81.3	108.9	96.6	126.6	96.1	119.5
6.39	2.67	102.8	105.3	93.3	108	118.3	117.6
6.34	6.35	96.7	95	99.5	111.5	112.5	114.4
8.25	6.35	106.5	99.7	108.7	97.7	104.3	102.8
5.78	5.14	75.1	106.1	96.9	98.9	91.2	103.9
4.72	3.3	111.4	110.9	100.4	111.8	84.2	106.7
9.13	4.94	101.7	104.4	113.8	78.6	77.7	74.8

6.25	4.76	94.5	104	97.7	91.4	94.2	92.4
7.84	27.16	108.2	109.2	103.5	87.4	87.7	86.7
7.85	5.16	92.6	100.3	95.4	97.7	106.1	106.4
6.51	5.27	103.4	112.6	118.4	85.8	92.5	95.8
7.49	4.26	95.4	108.7	82.2	109.6	116.9	104.3
7.01	4.42	81.6	104.4	100.4	97.8	87.6	94.7
6.71	4.28	126.2	115.1	101.6	79.1	112	95.6
6.86	2.86	99.6	111.5	101.8	87.2	100.3	98.7
7.77	5.54	105	108.4	106.9	102.2	93.3	92.7
4.94	4.71	93.6	123.9	112.3	114.1	104.2	111.4
5.35	5.21	100.3	95.8	93	89.4	87.9	100.9
9.66	3.4	97	100.8	96.8	89.2	97.2	103.9
8.6	5.54	107.4	112.2	116.3	91.2	98.8	95.9
4.63	9.45	98.8	103.7	104.2	107.1	116.4	109.9
8.46	3.84	89.7	85.5	78.7	101.9	109.3	98.7
4.7	5.47	108.1	102.7	100.2	100.1	106.7	122.4
6.65	3.48	99.4	103.7	100.4	101.5	101.4	107.1
9.26	5.04	98.5	102.9	105.8	106.4	112.5	116.7
9.25	4.65	101.6	95.2	95	85.8	89.6	93.6
8.27	5.38	100.7	87.9	105.7	107	105.8	99.1
8.27	2.7	103.6	104.4	110.7	93.5	90.5	85.4
8.35	6.13	106.4	109.9	103.5	105.9	104	107.6
6.09	4.43	102.7	101.2	107.2	94.1	83	84.6
7.14	4.14	108.2	106.4	110.6	106.9	98.9	100.5
7.27	8	94.1	103.4	77.9	103.4	93	140.6
6.8	5.33	97.3	102.9	110.5	76.4	104.4	85.1
6.2	2.18	99.2	102.3	108.6	86.6	100.4	100.4
8.02	2.78	107	98.1	96.7	116.2	101	109.2
5.17	5.86	84.7	100.4	90.8	116	106.2	120.8
7.06	5.19	93.8	110.9	96	78.3	86	91.8
5.99	1.71	103.9	91.7	110.6	111.2	94.4	93.3
6.74	4.59	96	104	94.4	107.1	82.1	105.6
5.34	4.98	100.9	104.4	66.8	121.4	128.2	108.7
6.81	4.32	104.7	82.6	106.4	64.1	111.9	88.8
8.13	3.93	88.9	112.1	109.2	115.4	99.5	105.8
7.96	2.01	94.5	87.7	85.3	118.6	111.8	114.1
10.48	5.67	99.9	113.7	109.9	159.4	150.1	159.8
7.03	5.55	101.2	99.6	101.4	101.1	106.2	99.5
4.94	5.47	89	83.8	93.6	109.2	103.1	113
5.57	3.15	110.6	107.3	103.5	102.8	92.5	91
8.16	9.15	95.1	93.8	96.1	143.2	144.1	95.7
7.28	4.38	105.3	98.9	112.1	102.5	91.6	115.1
4.84	4.6	86.7	128.2	71.5	102.7	89.7	99.5
5.69	2.31	105	113.9	114.5	111.7	116.1	91.6
6.4	5.96	96.4	98.9	96.6	106.5	98.8	103.4
5.16	3.42	84.4	96.4	96.5	94.3	96.3	111.3
7.4	2.75	95.5	117.5	102.2	75.3	102.1	101.8
8.56	3.89	109.9	94	87.5	84.1	107.6	114.5
9.26	5.21	101.3	115.2	92.1	95	96.1	112.6
9.09	5.18	102	92.8	103.8	110.7	96.9	96.9
8.98	2.6	107.5	89.5	89.7	102	97.5	111.3

5.41	4.76	101.4	93.4	105.1	99.6	100.3	95.1
7.2	2.72	96.8	90	104.5	102.6	105.9	128.1
5.06	5.15	93.7	100.6	99.6	102.1	104.1	112.4
5.05	5.42	93.1	97.5	100.1	110.4	111.4	117
8.63	5.36	104.1	109.5	110.2	97.7	107	101.3
9.69	2.78	112	112.6	81.7	108.3	83.7	100.8
5.17	2.18	105.9	97.5	115	73.8	90.4	81.1
6.35	5.04	94.6	89.5	101.9	99.7	114	122.8
5.1	4.96	112.5	91.8	110.4	91.4	86.4	122.6
6.4	4.68	105.5	86	93.6	79.4	91.4	89.8
9.44	2.05	106.4	95.9	96.5	142.4	123	126.4
5.71	4.76	105	107.5	105.7	109	119.2	114.7
7.85	2.94	104.2	97.9	103.2	88.1	101.5	98
8.24	5.87	90.5	100.7	106.6	111	110.9	116.4
5.02	6.36	105.7	113.5	106.6	116.9	104.2	114.3
6.52	2.82	92.7	126	117.9	97.7	122.9	97.1
5.2	3.4	101.6	94.6	88.1	76.2	89.2	95.8
5.81	4.68	120.4	98.8	105.4	95.5	86.1	85
8.62	4.97	103.4	113.9	100.4	99	119.8	110.3
5.26	4.45	100.8	94.3	91.4	94.4	98.9	82.6
6.67	4.09	99.6	94.9	98.7	100	87.9	99
5.12	2.58	92.6	97.2	102.3	86.5	100.3	93.4
8.15	2.69	90.9	87.4	93.7	97.4	103.4	113.8
4.72	4.27	96.7	100.6	111.3	96.2	89.4	107.9
4.51	3.45	111.4	86.2	91.9	98.8	119.3	107.3
7.96	3.86	111.5	90.4	118.3	86.4	92.8	90
10.3	4.34	113.6	108.4	105.1	114.2	118.6	131.8
6.32	5	94.8	92.1	102.2	105.7	105.2	107
7.06	5.1	93.7	101.3	86.3	93.2	95.8	102.2
8.87	5.57	95.7	105.7	107	106	105.1	103.4
7.55	1.99	97.9	82	91.3	92.8	97.4	88.6
6.29	5.53	90.7	104.4	91.6	102.6	109.4	101.2
8.75	4.78	102.1	109.7	107.6	101.6	104.3	99.7
5.53	6.13	86.3	98.3	89	93.8	110.4	112.3
5.82	2.24	84.2	77.3	79.8	105.7	116	103.9
8	5.62	100.9	104.7	94.9	103.4	95.2	105.4
7.62	4.99	91.1	88.2	88.1	116.5	108.9	106.5
9.13	2.06	78.9	104.3	105.2	112.7	102	101.2
4.41	3.42	115.6	110.8	115.8	107.4	103.1	98.4
6.84	3.55	123.8	105.7	125.1	90.9	89.8	110.7
8.88	3.08	103.7	92.5	103.7	109.8	107.8	103.4
5.03	2.4	94.2	101.6	101.3	107.5	110.1	114.9
6.61	4.9	95	92.5	104.2	93.3	86.9	110.9
10.24	5.5	102.1	98.3	101.3	108.5	106.6	122.9
9.44	3.38	65.1	91.9	114.7	101.9	130.3	169.6
9.14	2.32	96.7	78.7	85.8	102.9	101.4	92.9
8.4	2.19	108.9	107.1	110.5	98.3	77.9	103.1
6.9	5.04	109.5	114	90.2	95.4	86	98.5
9.01	5.27	90	114.2	81.2	105.1	89.2	120.8
7.5	5.53	106.3	111.3	104.9	109.4	113.5	105.2
4.44	4.27	79.3	108	99.8	111.7	108.2	110.4

7.74	2.43	100.8	99.5	94.7	99.2	99	97.3
8.46	4.79	97	82.2	119	102.2	114.4	104.3
5.64	2.67	77.9	104.8	113.8	97.6	101.1	84.5
8.4	6.59	103.8	111	109.1	89.8	87.5	98
8.87	5.72	120.7	111.7	121	80.3	86.4	97.8
8.65	2.67	92.7	87.2	92.7	99.1	107.4	110.1
6.87	5.08	109.7	103.6	104.4	102.7	97.1	97.4
7.3	5.27	103.7	94.1	99.6	101.4	102.6	97.9
9	4.8	97.4	99.7	97.7	86.2	115	117.8
8.56	4.65	94.6	89.4	102.9	114.1	112.1	114.8
6.1	5.05	99.5	93	92.8	108.2	100.7	113.7
9.73	3.86	98.5	120.2	88.8	136.6	178.3	169.6
5.68	3.89	99.6	86.6	109.8	91.4	103.2	105.6
6.54	4.89	101.1	106.4	114.7	98.4	111.6	109.2
9.32	7.48	97.6	105.6	96.6	121.3	111	123.6
8.68	4.19	97.1	86.4	104.5	94.2	101.1	119.1
6.62	5.3	97.3	113.9	103.5	106.6	106	112
8.79	4.23	103.8	102.1	103.2	110.4	109.4	108.6
5.1	5.08	114.8	94.2	96.9	109.2	93.7	118.3
8.1	4.55	120.2	109.5	97.1	90.5	97.8	90.4
5.6	4.82	102.1	101.5	101.8	101.3	91.2	102.3
7.97	5.04	109.9	102.2	102.7	108.5	95.5	105.4
8.78	6.17	73.1	79.5	78.5	97.4	89	91.9
5.85	4.13	89.9	91.2	101.1	110.6	117	112.5
5.36	5.12	102.7	107.6	97.7	96.8	89.7	102.6
8.12	5.06	105.9	107	98	89.6	94.4	100.8
6.4	4.72	85.2	88.1	96.7	111.4	117.8	110.1
9.32	4.28	95.8	95.8	94.6	103.9	94.9	102.1
8.12	4.24	110.6	110.9	114.6	94	90	97.6
5.47	3.2	112.7	119.7	105.2	105.3	105.7	100
5.4	2.75	91.2	93	90.7	89.1	111.2	107.2
9.32	8.53	95.6	63.2	104.8	96.8	102.3	133.4
9.38	2.7	96	97.7	102.8	102.4	94.6	94.2
8.53	4.7	108.3	106.1	108.2	107.2	105.7	108.3
7.81	7	93.8	95.6	100.2	102.4	98.4	110.8
6.25	4.5	116.1	102.8	107.8	99.1	105.1	114.1
5.91	6.23	96.2	89.7	98.4	91.6	96.9	98.7
8.65	4.58	106.2	101.1	93.9	110	113.3	104.8
6.25	5.5	95.6	86	98.1	83.7	80.1	85.2
6.99	4.03	144.2	126.8	120.8	59.5	57.7	47
8.79	2.58	92.7	87.7	97.5	103.5	113.8	151.9
6.44	5.52	104.3	99.9	103.5	104.6	107.9	105.9
6.65	2.26	114.1	101	94.7	118.3	123.2	101.9
8.06	3.69	115.6	98.9	105.6	96.9	92.8	90
9.03	2.08	105.4	133.2	89.2	99.7	103.6	77.7
7.94	4.38	98.6	90.8	118.5	89.3	114.8	93.8
9.36	3.36	115.8	83.9	102.7	119.6	120.3	97.8
7.27	1.69	99.3	108.5	99.9	112.3	102	108
8.29	4.9	98.2	116.4	107.3	111.8	99.8	108.3
5.62	5.26	95.6	103.6	97.5	111.2	125.5	103.2
8.4	4.06	89.9	85.9	101.6	107.1	113.5	110.4

8.91	3.6	118.9	107	109	98.9	100.7	111.7
10.2	4.63	104.8	111.5	106.4	113.4	108.4	94.1
7.36	3.4	110.2	101.4	129.4	104.4	102.6	120.5
6.39	7.56	115.7	50.4	88.8	178.4	51.8	196.4
6.27	2.44	104.1	89.3	88.6	102.5	103.4	110.5
6.84	3.76	85.6	73.6	83.8	117.8	100.4	119.9
6	3.81	108.8	99.3	93.8	90.4	102.6	97
6.05	4.31	97.8	102.5	99.2	108.1	108.9	103.8
7.97	1.83	93.2	77	102.7	109.9	82.9	105
6.77	5.14	119.5	120.5	111.1	87.7	114.4	115.1
9.77	3.49	91	88.3	83.5	121.2	112.7	123.8
8.32	2.5	83.7	119.2	74	91	98.7	97.7
5.06	10.11	83.8	88.2	88.4	169.6	171.8	171.8
9.03	3.74	114.7	104.4	99	110.5	109.7	115
7.05	4.19	124.4	125.8	94.4	118.1	99.8	95.7
6.76	1.99	79.8	91.6	98.6	92.6	100.7	113.3
5.07	11.81	98	124.1	91	116.6	72.8	95.8
5	4	107	96.9	107.2	97.1	94.7	97.1
8.07	4.65	93.8	101.7	109.1	97.6	85.4	88.6
7.06	4.65	99.8	102.4	91.3	105	109.8	103.5
8.03	2.95	65.8	92.9	102.6	81.5	108.8	105.9
7.77	2.78	100.2	87.4	86.5	110.9	102.3	108.5
6.8	1.96	104.1	92	101.3	91.4	99.7	111.7
6.73	3.91	121.7	95.2	107.6	98.6	92.9	107
8.6	4.98	101.1	99	105.1	112.3	109.8	117.9
4.5	2.07	92.7	100.1	95.1	97.6	99.2	94.5
9.29	3.93	94.4	95.2	97.2	105.7	106.2	94.7
8.25	2.35	94.6	87.5	78.9	103.1	105.8	93.8
9.91	2.99	99.1	107.2	117	109	107.4	88.1
8.31	3.58	107.1	105.5	100.8	100.8	96.6	116
5.39	5.38	93.3	96.3	95.8	107	109.4	109.7
5.64	3.91	94.4	107.2	103.3	96.3	99.5	98.2
8.19	3.57	95.3	98.1	84.7	77.6	71.8	78.9
8.85	2.17	110.8	124	97.1	96.3	101	94.9
6.54	2.26	116.4	124.9	105.8	70.3	88.6	94.1
6.57	2.49	91.3	93.1	104.9	96.6	94	93.1
8.97	3.95	94.1	101	96.2	99.6	105.4	110.6
7.69	5.53	129.9	88.2	103.5	82.9	130.9	85.8
9.36	2.19	97.3	86.7	93	88.5	77.5	96.1
8.51	6.1	102.6	98.7	100.2	106.8	111.9	110.3
4.92	4.74	116.1	102.4	89.6	102.2	123.2	71.2
8.41	3.42	91.8	93.5	99.4	121.1	115.2	107.8
6.83	1.82	104.2	105.9	97	106.7	116.8	107.2
7.71	3.98	101.9	103.6	97.6	104.7	103.5	101.1
8.1	3.15	89.8	83.7	83.8	116.5	102.5	119.2
7.84	2.49	120.4	111	116.6	102.2	91.3	89.2
6.18	2.88	90.8	94.3	101	119.2	111.4	98.3
6.93	4.79	104.4	108.3	100.5	105.3	94.4	103.1
6.58	7.86	88.2	98.2	116.1	99.3	113.8	125
5.82	4.88	101.8	79.9	101.7	108.9	132.7	167.2
4.83	4.04	95.2	92.7	89.5	120.4	114.3	111.4

8.81	2.05	100	118.5	102.6	83.6	96.1	99.9
5.96	7.69	95.7	92.9	97	97.2	108	100.3
8.12	2.46	94.9	106.2	117.7	92.2	91.5	94.4
8.6	4.48	94.4	97.2	98.4	107.8	97.4	110.6
9.55	2.74	104.1	95.1	100.7	105.7	113.5	116.2
11.33	3.37	109.7	115.6	88.9	117.9	112.4	86
8.03	4.1	97.1	92.4	132	82.6	95.1	81.6
5.68	3.9	98	89.9	127.8	107.7	108	96.6
5.97	3.23	98.5	95.1	115.3	82.3	89.5	98
6.96	3.01	121.4	97.6	137	112.4	97	113.9
10.77	7.42	112.1	107.4	93.5	101.9	91.2	102.2
6.06	1.78	87.8	85.3	100	97.6	97.2	109.3
8.95	3.41	138.1	99.2	119.7	97.3	105.4	98.6
7.88	4.7	103.1	104.5	101.3	97.2	92.7	106.8
5.66	3.41	101.1	99.1	80.5	89.1	87.2	128.8
8.19	6.11	97	99.4	96.7	93.1	97.7	100.6
6.49	2.91	112.1	88.6	97.9	89.9	95.8	95.8
8.31	3.84	95.2	110.9	102.3	96.6	93.9	97.9
4.91	3.12	101.9	101.2	97.5	80.6	92.4	103.3
6.2	2.48	106.3	136.6	74.4	96.4	101	121.8
6.7	3.64	100.4	104.1	114	105.8	105.5	108.3
4.75	6.2	104.5	105.5	104.3	113.1	104.3	105.7
9.94	4.17	95.5	93.5	96.7	125.4	122.8	125.7
10.04	4.09	137.2	129.8	167.8	84.7	57.1	78.8
6.68	4.53	107.1	97.2	103	104	94.3	102.6
6.01	6.92	103.9	108.7	113.4	110.5	106.8	118.1
6.98	3.34	92.1	98.3	95.8	99.8	105.6	95.9
7.31	3.93	107.8	86.1	103.7	89.9	111	109
6.48	3.38	93.1	101.6	91.2	108.1	103.6	93.5
5.59	2.53	81.5	89	88.7	93.7	98.1	103
7.24	3.89	113.6	103.5	100.4	107.9	106.8	103.5
9.31	2.53	93.7	85.9	98.9	107.4	114.1	94.8
9.38	4.92	121.4	122.8	125.3	36.4	28.8	39.7
7.4	2.87	121.4	98.9	105	95.7	88	99.1
5.31	4.15	115.4	117.8	86.8	82.3	115.9	107.2
5.48	2.93	110.2	90	105.8	105.5	97.4	108.7
7.2	3.22	91.6	100.4	103.1	107.3	97.7	84.6
7.14	6.01	133.9	113.1	150	72.5	67.8	68.2
9.07	3.44	102.2	79	99.9	100.2	101.5	108.6
9.2	3.8	100.4	95	96.8	105.4	92.6	107.4
6.52	3.26	112	106.6	95.8	95.8	86.6	85.4
8.76	3.78	96.2	93.6	105	92.3	106.1	119.2
5.6	4.62	98.9	96.5	106	114.6	117.1	133.3
9.57	3.46	107.5	83.4	89	119	94.4	104.1
7.52	3.16	101.7	97.2	99.4	106.2	100.1	96.1
9.58	3.46	104.3	102.2	106.5	104.3	107.3	108.2
5.81	2.55	96.7	101.2	112.4	117.9	93.2	105.9
9.7	4.95	105.5	103.7	104.4	105.8	102.7	101.2
9.55	3.57	111.6	96.7	100.7	104	113.8	105.8
4.82	2.8	101.1	120.6	120.7	100.6	89.1	77.5
5.63	2.3	102.2	105	94.2	84.8	93.3	104.2

10.02	3.87	107.2	104.9	90.6	104.4	109.6	109.8
7.14	3.6	126.3	108.2	109.9	71.5	126.9	78.9
7.78	3.02	105.4	105.8	101.8	103.9	100.4	104.3
8.94	4.12	106.8	110	102.8	127	110.1	124.4
6.65	3.06	102.9	102.6	97.8	118.1	105.9	116.1
6.77	3.8	106.2	111.7	103.5	93	93	92.9
6.01	3.47	100.1	76.1	90.5	109.1	89.9	104.3
8	2.97	125.5	95.5	101.8	86.3	99	89.8
5.66	4.27	102.4	99.2	97.3	93.4	93.2	103.3
6.01	3.36	92.5	100.7	98.3	108.7	102	103.7
5.6	3.95	108	93.4	95.1	103.6	98.2	107.3
5.27	2.67	101.4	100.4	99.5	104.4	91.1	89.3
4.36	3.87	89.5	87.8	92.5	127.1	132.3	113.6
6.65	3.78	131.7	121.2	88.8	90.6	65.8	95.7
8.02	3.5	80.2	108.2	97.8	112.3	122.2	108.1
6.46	2.84	105.7	102.1	101.3	96.1	89.4	93.4
8.06	2.01	124.8	86.3	96.2	96.6	120.9	93.2
7.87	3.28	108.3	114.4	82.2	127.9	115.2	94.4
8.12	2.73	83.7	92.7	70.4	127.3	156.2	139.8
6.77	2.97	108.1	96	93.2	81.6	91.5	95.7
10.37	3.77	103	105.7	97.1	123	136.2	126.5
7.27	3.1	111.9	110.1	101.8	89.2	88.4	97.7
7.42	8.24	101	102.8	103.7	108.3	104.3	104.7
6.81	5.49	99.9	102.6	104.6	118.3	113.5	110.8
8.65	4.82	96.8	95.2	102.5	99.2	101.2	102.9
8.06	3.63	108.3	106.4	102.8	99.5	98.5	98.9
9.72	2.1	104	87.9	101.3	97.9	88.9	108.2
5.11	3.15	104.1	113.7	90.5	115.5	113.4	117.5
9.35	3.71	115.3	94.6	76.7	77.7	98.4	81.8
6.96	2.72	101.5	90.1	123.6	102.1	107.8	93.3
7.09	5.03	101.9	102.7	109.4	95.2	94.1	99
8.09	3.36	108.8	117.5	106.3	89.3	104.2	106.1
7.84	3.41	94.9	102.8	94	94.5	97.4	98.7
9.32	2.87	91.8	90.5	82.8	119.1	101	93
8.22	3.91	103.7	94.6	87.7	107.2	114.4	102.6
8.53	3.41	113.5	103.4	105.9	99.1	107.4	102.3
6.54	3.55	97.4	87.2	111.7	93.2	96.4	114.1
6.37	4.45	118.3	146.7	103.9	95.9	54.4	85.9
5.43	3.61	122.7	105.6	97.2	78.8	76.5	103
5.36	4.66	94.9	106.8	104.4	85.5	82.1	96.5
6.99	3.27	98.5	92.1	105.3	99.2	81.1	93.9
6.29	2.28	118	98.4	104.6	99	87.3	99.2
4.55	4.24	94.8	85.9	79	104.6	103.4	99.1
8.95	2.95	93.6	102.1	87.2	110.2	117.4	108
8.62	2.05	111.3	97.9	111.7	105.9	104.1	107.8
5.12	3.88	103.2	104.5	104.7	117	113.4	98.9
8.78	4.14	91.2	86.8	88.9	116.2	104.5	124.8
9.55	4.66	108.3	102.4	100.6	102.6	101.9	103.5
5.94	1.74	101.8	104.4	115	114.1	99	114.7
8.95	3.02	98.1	107.3	98.5	116.5	106.3	101.1
6.93	2.99	89.1	96.8	119.9	95.1	114.3	111.3

5.94	3.65	90	118.6	88.3	148.1	114.6	98.3
7.28	2.04	138.7	103.2	62.5	101.7	145.5	125.4
6.74	2.99	111.3	105.1	80.8	99.2	97.4	111.8
10.08	4.25	93.1	108.2	114.8	133.1	114.6	101.7
6.93	3.17	94.1	112.1	98.1	107.7	100.2	103.6
7.28	3.87	92.3	100.9	108.9	100.8	109.4	101.8
4.89	4.76	96.7	106.2	107.3	109.3	103.8	111.8
10.14	3.46	84	88.4	81.1	121	127.3	124.9
5.16	2.46	119.1	83.6	83.4	102.9	90.4	106.9
7.03	3.01	122.3	98.5	89.9	119.3	121.9	130
6.52	2.6	85.1	99.5	88	86.6	79	98.7
6.18	2.38	117.6	87.2	79.3	79.8	107.3	95.5
5.34	3.94	102.8	109.8	119.3	103	107.4	108
7.09	4.78	83.1	67.8	82.7	112.8	136.2	120.1
8.27	3	93	110.2	100.3	105.6	104.7	93.9
5.96	3.23	99.8	95.9	94.3	102.8	94.6	96.6
9.16	3.23	110.7	96.7	93.5	92.1	112.7	96
6.54	3.64	101.7	100.6	102.7	73.2	74.8	82.6
9.57	3.46	97.4	136.4	120.5	131.2	127.7	131.7
6.68	5.65	117.5	102.4	105.9	95	90.7	85.1
7.47	3.15	107	113.4	86.5	102.5	116	104.9
9.61	2.61	101	110.9	96.8	61.9	53.4	40.8
8.95	3.06	108.8	102.2	107	145.1	143.2	130.7
7.44	2.91	96.1	95.2	98.8	103.2	100.9	116.7
9.92	3.63	131.4	91.3	94.7	106.1	114.4	94.5
4.81	3.24	98.6	92.5	95	111.3	108.2	100.8
8.16	3.98	103.6	95.4	119.9	106	115.9	113.7
8.27	3.29	97.8	110.2	96.9	100.3	98.5	106.5
7.4	3	112	111.3	99.2	92.9	102.8	99.2
5.07	2.31	113.8	117.3	93.5	135.7	105	84.5
6.13	3.4	93.2	91.9	101.1	99	91.7	104.8
4.21	3.37	99.3	100.7	96	94.4	94.1	99.5
5.58	2.85	95.6	106.6	115.1	84.5	122.6	106.5
9.45	3.03	98.3	80.5	126.2	119.6	112.6	110.1
7.66	2.97	89.8	105.9	106.4	98.3	113.4	73.1
6.67	2.48	91.8	112.4	98.8	72.9	60	68.5
5.07	4.38	98.2	84.6	87.3	113.5	112.1	113
5.05	2.95	84.8	69.9	112.3	86.2	121.5	109.2
4.09	3.9	101.5	72.1	88.1	123.8	106	110.1
8.07	2.67	116.2	117.7	126	134.1	144.1	130.7
5.47	2.73	110.9	89.6	100.3	110.1	102.6	115.2
6.9	3.27	107.6	113.1	110.2	100.8	92.7	79.2
9.01	2.16	110.7	103.6	102.7	102.3	97.5	110.1
5.74	2.81	91.9	100.9	93.4	89	113.8	120
6.89	3.11	118.4	94.6	85.7	95.3	79.9	93.4
8.1	4.74	100.7	101.8	100.9	108.6	104.6	103
8.41	5.86	122.2	91.1	95.9	116.3	91.1	113.6
9.94	3.09	101.3	93.6	101.6	105.5	114.6	108.3
4.97	2.39	84.9	109	80.8	94.2	92.6	108.1
8.24	3.64	105.7	99.4	99.3	88.9	102.2	103.9
7.37	2.96	107	98.1	108.4	106.8	99.2	100.1

8.46	2.56	113.3	139.9	134.2	81.1	84.6	82.7
9.19	3.72	95	102.6	99.5	104.5	102.7	97.6
5.44	2.62	79.8	86.5	70.7	118.8	114.7	128.1
8.76	3.64	103.9	81.8	91.9	133.2	129.1	136.1
8.12	1.9	97.8	111.4	83.5	138.3	125.5	134.3
8.53	3.59	104.1	96.3	101.4	101.4	88.7	84.8
8.12	3.21	87.9	88.3	90.1	101.8	105.3	128.5
5.55	3.9	97.3	104.6	103.7	104.4	83.9	105.3
6.4	2.2	92.7	114.9	92.4	124.4	114.5	110.2
6.54	3.27	128.9	100.7	119.4	90.8	87.6	119
6.95	3.32	89.5	103.2	99.9	98	88.2	107.8
5.97	2.15	112.1	94.8	94.4	104.9	94.7	112.7
8.91	4.85	111.1	107.6	101.3	102.8	99.1	103.1
5.85	3.81	86.8	79.3	82.1	96.1	99.6	97.8
7.23	4.06	95.2	95.5	113.2	102.3	108.3	102.3
6.67	3.31	67.8	81	91.7	92.3	104	129.1
9.7	3.47	111.6	109.5	98.7	107.8	99.4	96.5
7.34	2.43	116.1	101.9	97.4	98.7	120.4	114.8
8.98	2.86	80.6	86.4	105.5	90.5	109.6	101.9
7.77	3.71	93.8	98.5	102.4	109.8	102.7	105.6
8.32	3.08	92.2	94.9	91.6	101.5	112.3	108.8
8.29	2.3	102	99.3	94.7	100.8	107.7	115.3
5.02	4.83	86.6	76.9	93.6	118.8	118.4	117.4
6.52	2.69	108.2	100.5	81.5	108.6	102.6	112.8
6.79	3.44	101.3	106.2	102.1	99.3	94.6	114.8
6.42	3.36	104.9	102.5	74.1	107.4	93.1	91.1
9.11	2.33	100.2	86.9	110.8	116.3	96.6	92.6
6.39	1.9	88	106.5	118.9	101.4	124	105
8.1	3.34	94	99.5	107.7	104.8	114.8	119.1
5.47	2.21	95.8	108.3	108.6	79.2	81	92.9
7.21	3.45	109.7	94.7	103.2	82.9	98.8	108.5
10.86	1.74	91.5	84.8	110.8	80.3	81.1	119
10.39	3.74	105.1	99.4	103.7	103	112.4	109
9.11	8.27	94.2	95.1	99.5	110.4	99.2	97.8
8.21	2.79	96.3	98.3	101	98.5	121	113
5.1	3.32	94.2	99.7	107.5	95.9	99.8	94.5
5.76	1.61	97.1	100.6	93.9	102.2	96	105.9
4.42	3.23	115.1	117.9	98.3	112.9	96.6	86.1
4.79	2.99	83.8	87	100.5	104.2	103.5	111.8
5.21	2.69	101.7	115.2	79.3	104.4	119.7	105
6.92	2.58	92.8	59.3	81.5	98.4	120.7	128.7
5.49	2.95	76.1	87.6	81.5	120.2	104.5	121.6
9.01	3.09	105.8	85.8	116.7	111.1	115.5	123
4.87	2.84	106.9	83.4	103.1	118.4	112.5	104.1
6.74	5.55	139.5	100.8	91.7	99	98.7	97.7
8.82	3.6	80.2	107.8	98.2	107.9	88.5	103.5
8.06	2.56	100.7	92.1	91.4	89.2	102.4	90.7
6.84	4.37	109.1	92.2	105.3	95.7	80.6	84.7
6.8	1.84	104.8	91.6	103.5	92.5	101.8	129.3
7.18	2.49	112	79.5	108	120.5	127.4	115
8.15	2.61	97.6	113.9	100	103.9	102.5	80.8

8.72	3.67	105.1	104.1	104	107	99.6	105.4
9.36	2.18	92.4	109	95.1	90.2	105.9	92.4
6.73	2.46	123.4	114.9	118.9	78.5	72.4	87.3
5.49	3.01	123.9	57.5	93.1	106	99.8	120.1
7.09	3.07	110.8	96.6	98.6	104.2	94.2	103.2
8	3.96	97.7	82	98.9	120	120.4	111.3
7.83	2.7	117.4	136.8	101.3	120	85.1	119.7
8.21	2.63	94.9	117.1	95.8	89.2	109.4	95.1
10.86	4.5	103.1	78.7	68.7	83.7	132.5	157
7.9	2.06	125.7	105.5	71.4	106.4	93	109.7
7.24	5.25	115.1	85.4	94.5	106.2	96	126.6
9.41	2.79	95.8	110.5	114.1	91.6	95.3	80.5
5.14	1.61	112.3	122.9	101.8	106.2	98.8	116.4
8.05	2.04	107.6	109.6	100.8	117.3	112	90.8
9.28	2.21	112.4	89.1	95.8	115.2	106	123.4
5.6	2.81	84.1	97.8	116.4	107.7	106	101.4
5.41	3.22	89.2	102.6	91.2	100.2	94.7	109.5
10.17	3.8	99.6	96	109.6	104.3	105.9	100.4
10.14	3.33	108.6	110.8	111.7	136.4	118.7	135.1
10.05	2.98	105.8	87.2	103.8	94.1	102	106.8
8.72	2.77	111.9	105.6	89.5	108.5	157.3	108.1
6.29	2.54	80.2	81.3	96.4	95.5	103.1	107.8
8.1	3.23	108	101.4	112.5	100.8	105.1	104.9
6.71	4.88	96.5	101.8	106.2	92.2	107.9	107
8.59	2.35	91.2	89.7	108.2	106.7	120.8	118.5
8.73	2.94	92.3	92	86.6	103.5	115.3	127.8
8.43	2.19	91.4	83.5	85.2	106.4	113.1	114.5
5.9	2.44	82.8	99.9	100.4	114.2	103.6	97.8
9.57	2.56	95	104.4	121.3	84.5	121.6	102.5
4.5	4.02	103.1	98.3	84.2	112.8	108.3	103.4
9.6	2.81	116.7	86.9	101.9	112.3	98.4	91.7
5.31	1.99	122.5	94	98.8	103.3	122	103.5
9.16	2.87	95.4	95	113.2	105	99.1	108.1
6.3	1.91	102.1	93.2	103.2	94.8	93.7	109.3
6.77	2.13	99.2	87.3	103.9	86	100.2	101.5
5.95	3.01	96.3	118.1	118.2	101.4	105	116
9.83	2.24	115.8	95.6	85.4	116.8	104.3	120.9
6.27	2.93	69.7	101.9	123.2	104	88.8	117.5
7.31	2.67	108.2	105.2	127.2	112	98.9	100.8
7.93	2.45	101.6	95.5	106.1	99.7	104.6	91.8
7.55	1.93	93	85.5	90.1	100.8	121.8	99.8
5.86	4.05	96.5	104.1	106.8	100.4	104.3	103.3
8.94	2.52	98.3	103.3	98.3	123.5	103.8	136.5
11	2.2	63.5	100.9	97.6	110.8	126.9	87.8
9.85	6.72	115.8	105.9	102	99.5	106	95.1
7.97	3.6	98.4	101.5	95.5	112.6	106.5	107.6
6.77	3.15	89.4	92.1	95.7	101.2	100.6	103.4
5.5	4.39	247.4	83.7	86.3	91.1	83.3	85.2
10.07	2.58	97.3	106.9	102	99.9	99.4	115.3
5.43	2.87	98.5	111.2	120.5	102	90.9	111.9
6.57	2.59	88.6	70.8	92.3	105.1	102.7	67.3

5.34	3.31	89	96.9	91.6	106	89	103.5
6.96	2.93	79.8	96.2	84.8	92.6	98.3	92.9
5.07	2.38	93.7	90.7	84	106.8	91.2	122.1
5.82	3.59	105.2	99	113.5	104.1	96.9	108.1
9.22	1.65	138.6	102.2	138.7	67.6	76.8	81.2
7.87	2.53	105.9	97.3	93.3	91	92.9	85.8
9.5	2.65	108.7	105.8	94.6	100.5	94.2	95.3
8.13	3.11	93.7	97	100.5	118.7	102.5	100.4
9.98	2.87	104.9	127.4	88.6	105.4	98.6	120.8
5.55	1.99	120.4	94.3	89.9	102.3	81.2	117.1
8.95	3.29	89.5	90.3	95.4	113.2	130.7	115.5
11.44	4.02	104.8	99.9	99.9	107.1	103.1	105.7
8.7	3.19	94.4	92.3	101.6	108.9	107.4	114.4
7.93	7.31	103.3	96.6	99.6	76.5	81.4	79.8
9.19	2.46	87.5	94.4	103.8	106.3	92.8	101.3
9.42	2.58	87.3	94.4	93.2	103.9	101.6	102.2
8.37	2.43	101.7	108.2	103.4	108	105.2	112.3
8.32	6.89	99.4	105.3	102.5	99	96.3	95.9
12.25	2.74	98.6	94.2	99.2	125.6	133.4	117.4
6.7	4.05	83.9	100.4	106.4	97.3	107.7	107.4
6.01	2.4	97.4	97.7	90.9	116.3	107.2	107.7
6.99	2.49	93.6	97.7	74	106.5	95.8	97.5
4.17	3.42	104.4	100.8	97.9	108.2	87.1	93.4
7.87	2.29	120.3	91.1	90.9	120.2	100.7	117.6
8.9	2.22	109.4	103.1	104.8	116.2	106.7	108.7
4.74	2.62	78.7	114	132.3	119.9	123.5	109.3
9.16	2.46	98.6	105.5	108.5	77.9	105.6	105.3
5.68	2.47	104.4	95.8	101.5	104.9	100.2	102.7
8.06	2.23	105.6	95.6	110.7	84.7	75.6	96.1
7.42	2.82	133.1	144.6	126.2	105.8	104.5	79.1
8.4	2.82	107.3	102	109.7	103.6	95.8	92.8
5.06	2.37	98.6	92.5	102.7	91.7	102.6	98.8
4.82	1.85	117.7	87.5	90.3	100.5	95.5	120.5
8.05	2.74	114.3	150.2	104.3	100.1	81	77.8
5.29	2.21	118.1	74.5	94.3	102.8	85.9	116.2
6.42	3.19	98.2	103.1	106.1	102	100.6	111
9.99	3.01	103.6	109.7	110	106.5	114	109
5.82	3.13	102.5	120.5	110.1	97	99.5	89.6
9.72	2.28	82.2	108.9	96.6	92.6	109.9	74.3
7.8	2.67	120.2	102.9	100	105.4	106.5	94.4
10.93	2.45	100.6	89	103.8	91.8	86.7	106.3
7.78	2.97	94.5	109.2	108.8	91.3	86.9	86.4
5.74	2.47	108.6	89.5	94.6	102.3	94.4	86.7
4.92	2.09	100.4	99.5	96.2	95.4	97.8	95.7
9.25	2.9	85	87.8	126.6	95.4	128.8	115.7
9.01	2.82	100.6	91.8	93.6	107.1	104.3	97.8
8.69	3.19	100.2	88.8	92.1	100.1	106.6	108
4.02	1.8	101.4	78.7	57.5	133.7	100.1	108.3
7.72	2.89	116.3	94.8	96.6	91.1	79.8	98.6
8.92	2.91	99	99.4	109	95	94.6	100.1
7.66	3.21	116.6	110.5	120.1	103.1	79.5	93.4

6.34	2.39	116.1	87.2	92.9	108.1	133.4	102.4
7.59	2.5	90.7	54.2	139.9	87.5	79.2	91.7
7.77	2.8	107.5	86.1	106.9	108.2	95.9	102.5
7.03	2.61	103.4	94	93.2	99.5	97.8	99.3
5.35	2.12	112.6	102.8	89.3	109.8	110.1	104.3
7.9	4.1	97.1	87.3	97.6	94.1	89.4	92.6
9.99	7.27	112.8	104.9	106	155.6	141.3	140.7
8.9	2.87	93.3	87	93.7	112.6	107.7	117.6
7.02	2.24	93.9	91	84.9	101.1	99.5	96.2
7.77	2.2	99.3	90.4	89.6	134.8	93.3	87.1
4.7	2.84	101.5	86.7	89.3	129.4	93.5	104.7
7.39	2.32	112.3	119	103.4	114.5	105.6	95.6
4.89	2.08	147.1	123.7	137.8	83.7	71.2	82.9
4.89	2.87	100.5	94.6	88.1	99.5	101.2	111.4
6.04	2.37	107.3	125.3	80.7	108	89.6	97.9
6.79	2.6	101.9	97.7	90.4	92.7	84.4	98.5
6.83	3.51	100.2	102.1	98.1	115.2	99.7	93.6
6.13	1.76	116.2	88.6	95	97.3	105.7	96
6.48	2.7	95.9	97.7	99	106.3	101.1	124.2
9.09	3.04	128.7	98.6	93.6	83.3	112.1	105.3
6.68	3.17	92.6	84.5	90.7	104.9	103.2	99.2
5.72	3.56	82.5	107.4	97.2	104.6	106.3	116.9
5.3	2.92	96.9	108.2	109.2	102.8	117.8	97
6.47	2.56	110.4	90.6	93	110.1	97.1	121.9
8.82	2.79	107.7	97.1	102.3	97.7	115.6	108.7
6.42	1.97	102.4	95.5	94.8	110.1	86.1	90.4
7.78	1.91	93.6	97.6	105.1	93.9	122.5	124.7
5.12	1.61	98.1	106.8	114.4	86.7	87.4	104.9
7.39	2.53	92.8	100.5	93.3	102.4	95.8	105.3
9.52	3.2	100.6	95.4	90.2	113.9	114.7	110.7
5.68	2.08	90.4	50.5	104.5	113.6	106.2	121.7
9.96	2.51	118.4	77.8	129.2	89.8	96.9	106.4
5.05	2.9	99.5	96.1	108.2	97.8	100.5	104.8
8.88	2.06	93.1	98.1	103.5	92.6	128.3	120.4
8.18	3.08	97.8	97	86.1	103.4	90.2	94.3
9.88	2.88	100.2	91.8	103.9	109.3	111.4	111.9
9.13	2.7	108.2	110.5	101.6	95.3	94.9	105.4
10.29	2.88	99.8	100.5	98.1	118.4	121.1	114.2
5.58	2.52	82	87.9	101.1	107.4	110.6	113.6
6.74	4.56	102.1	109.3	97	101.3	101.6	102.1
9.54	2.87	93.9	97.7	92.8	120	111.5	114.7
8.1	2.28	102.1	111.2	94.3	121.3	73.8	95.8
8.44	3.25	104.9	88.9	95.6	104.5	107.3	111.7
7.64	2.37	111.9	113.3	110.7	126.8	109.4	123.8
5.3	2.79	98.5	92.1	96.2	101.2	102.6	100.4
5.36	2.9	95.6	93.8	108.4	103.7	98.9	111.9
9.41	2.56	101.6	82.3	80	101.5	107.5	120.6
8.15	2.03	117	92.7	73.3	86.9	99.3	115.3
6.48	4.35	96.8	92	109.4	97.2	98.5	100.5
6.37	2.2	72.2	83.2	76.1	122.2	114.5	119
10.54	2.21	92.2	96	123.1	106.6	99.7	91.6

6.19	3.11	153.1	81.8	77.5	94.4	128.4	96.2
5.06	3.63	98.6	106.5	104.6	90.5	110.5	87.4
7.9	3.21	111.1	122.4	123.9	83.7	82.4	86
7.23	2.96	110.4	99.8	109.1	100.3	99.6	104.8
7.06	2.03	136.4	124.6	126	75.7	50.6	82.5
7.94	2.5	82.8	96	104.4	116.3	106.6	98.5
6.7	3.5	87.1	98.3	97.1	102.8	91.8	110.2
10.1	2.59	117	93.2	103.4	108.4	111	103.2
6.48	1.9	113.5	102.2	97.2	89.3	115.6	85.9
4.87	2.54	102	106.7	106.5	108.3	116	116.7
8.47	1.72	104.1	84.2	94.5	95.5	101.5	103.1
4.17	2.59	82.9	112.5	106.3	123.1	102.4	120.1
6.58	2.09	106	86.6	106.1	89.7	106.9	116
8.51	2.34	122.7	93.7	95	89.9	94.8	92.7
7.65	2.35	96.5	73	88.9	101.6	111.9	122.8
8.05	2.13	84.5	76.6	83.7	96.2	116.2	99.5
5.62	2.91	72.7	117.9	94.7	97.7	99.3	97.5
8.1	3.35	103.4	98.5	107.9	111	112.3	111.6
9.85	1.81	95.3	113.8	90.8	94.8	93.3	97
8.22	2.82	100.5	100.2	102.1	107.1	100.2	103.3
6.76	3.86	98.5	105.2	111.9	110.6	110.3	103.6
5.52	2.27	83.8	102	106.9	100.4	116.4	120.9
7.42	2.24	89.7	80.5	80.2	102.9	110.5	107
10.13	9.57	112.9	120.2	112.7	88.6	89.9	87.5
8.5	2.23	100	100.2	98.7	99.9	100.2	100.1
5.95	2.94	104.9	93.5	96.8	97.8	94.2	89.6
7.72	2.1	88.5	86.4	100.6	105.5	103.1	102.3
6.19	2.05	100.6	96	112.5	109.6	69	92.3
6.04	2.24	112.7	98.8	98.6	103.8	100.8	111.9
9.33	3.47	96.3	99.9	97.8	112.1	106.1	108.8
6.47	2.01	89.9	87.8	126.4	87.8	124.6	119
5.17	2.77	116.4	99.8	119.4	93.6	93.8	82.1
6.79	2.55	107	123.7	107.4	91.9	97.1	90.8
7.28	3.04	100.6	96.2	102.1	96.4	93.2	92
8.15	2.49	123.7	102.2	87.8	96.5	84.9	89.8
6.62	3.12	112	110.6	94.6	91.3	103.4	101.8
4.88	2.49	111.9	97.8	104.5	111.6	81.9	90.5
7.62	2.84	108.8	95.7	112.6	88.2	95.4	94.4
8.27	2.91	107.6	89.8	98.5	93.1	95.6	106.3
4.18	2.5	161.7	88.8	98.8	100.5	102	87.3
7.3	2.15	67.2	105.5	109.6	92.2	105.1	96
7.02	3.23	89.6	95.7	95.7	113.2	108.8	103.4
9.22	2.03	99.2	88.4	91	76.3	65.2	76.3
5.15	2.42	111.8	103.7	117.4	108.1	91.4	113.7
9.32	3.05	103.7	98.3	101.9	97.5	97	101.3
4.86	3.91	127.4	94.5	85.9	93.5	77.6	90.5
4.65	4.77	146.1	91.6	101.5	94.3	92.9	92.6
4.69	3.11	122.1	118.9	107.7	89.6	97.3	92.5
4.88	2.83	106.5	97.2	115.4	102.2	100	96.3
7.58	2.23	104.1	100.4	101.4	125.7	110.6	130.2
4.81	1.81	86.7	84.9	71.1	135	123.3	143.9

6.55	3.03	92	101.4	114.9	84.3	87.8	86.4
9.5	3.43	94.4	94.8	121.1	103.7	101.8	103.2
5.83	2.23	118.4	83.2	111.7	106.9	99.1	116
5.52	2.81	102.4	97.6	125.9	80	121.4	98.7
7.78	1.9	97.3	96.3	93.2	103.5	142.3	91.5
5.02	2.7	103.9	97.8	111.8	87.3	98.7	96.1
4.31	2.19	97	122.3	98.5	105.1	94.4	112.9
6.9	3.52	107.7	98.1	97.7	95.5	95	102.5
8.12	2.49	87.1	100	82.4	116.9	101.5	101.8
8.53	2.13	107.9	103.8	111.6	84.8	96.9	120.2
6.87	2.15	103.4	63.3	125.7	100.1	95.4	87.2
8.22	1.86	93.8	98.9	93.1	109.1	121.2	100.4
6.76	1.75	134.1	98	89.4	99	83.1	98.5
5.67	2.4	87.6	86.2	107.8	120.6	94	100.3
5.83	4.05	94.4	108.8	103.3	107.8	112.4	110.4
6.19	2.03	101.9	88.3	104	106.5	112.8	110.8
6.01	1.87	101.5	92.9	106.6	100.6	87	93.6
5.1	2.36	121.1	89.1	100.9	102.1	97.3	101.9
8.81	2.1	88.2	103.4	116.2	93.3	111.5	91.7
6.25	2.15	101.9	84.1	100.1	110.9	121.1	124.1
5.69	2.18	88.5	90.2	114.2	100	103.6	87.7
4.82	1.71	87.4	85.6	78	124	96.9	104.5
9.09	2.56	106.3	102.1	104.1	119	105.4	96.8
5.62	2.15	102.4	108.5	98.6	94.1	76.3	90.2
8.48	1.91	86.3	65.9	111.8	104.1	129.8	117.1
7.06	3.59	99.5	101.3	98.1	99.8	118	112.5
7.02	2.16	61.7	94.8	92.7	111.9	120.9	95.3
6.32	2.7	93	102.1	98	111.1	122.8	123
9.94	1.95	107	97.7	95.5	105.2	95	109.5
5.91	2.26	85.4	105.3	123.4	95.4	105	109.3
6.62	1.66	84.1	106.4	88.8	103.9	88.8	117.1
6.7	2.3	121.3	100.1	102.1	75.2	85.7	124.3
6.86	4.32	104.1	99.5	99.8	98.2	96.9	105.1
8.02	1.82	105	93.8	71.2	112.8	126.7	125.2
8.19	2.88	98.7	98.6	102.9	106.1	108.1	105.1
8.79	2.34	90.3	98.7	102.4	93.4	108.1	113
7.88	3.02	96.3	83.6	105.6	92.2	95.5	100.2
5.47	2.43	100.8	102.1	95.5	94.5	104.6	100.1
9.38	2.2	72.6	65.9	82.3	92.1	103	249.4
8.85	2.9	102.8	108.5	105.7	115.5	106.1	102.8
6.21	2.55	98.8	98.8	108.7	122.6	85.2	93.8
9.33	2.93	85.3	82.8	79.9	87.7	99.6	102.6
8.25	1.94	99.2	86.9	114.4	110.8	68.3	94.4
5.97	2.52	95.7	97.4	111.7	96.5	112.3	100.7
8.18	2.17	100.2	94.8	110.2	109.8	98.8	110.6
8.72	3	89.1	88.7	92.2	127	124.9	118.1
4.97	2.77	116.9	112.2	89.2	91.8	84.8	113.2
5.85	2.6	107.1	103.6	99.5	124.4	116.2	116.3
5.06	4.33	110.6	93.5	88.1	95.3	88.2	96.8
7.83	4.75	100.7	105.3	98.2	99.9	94.7	89
6.15	2.54	119.8	110.5	114.3	89.9	85	72.2

10.64	2.21	81.5	90.2	98.7	76	85	87.3
7.02	2.08	108.7	94.9	77.9	95	118.3	130.7
6.21	2.96	105.2	97.8	104.4	113.3	109.9	94.4
6.37	2.02	121	109.7	120.2	139.8	147.4	129.2
10.64	1.73	112.2	104	100.4	99	104.8	113.8
7.42	1.95	65.6	103.3	89.9	105.7	118	116
5.25	2.28	109.7	109.6	105.8	116.8	102.5	107.6
7.97	2.95	95.3	96.7	109.8	88.9	94.4	93.4
5.34	1.8	90.6	90.4	91.2	113.3	107.4	84.1
5.1	2.44	111.1	101	118.1	88.6	97.4	87.8
5.97	2.47	96.5	106.5	105.6	104.2	90.9	102.1
5.9	2.62	103.9	90.4	112.2	98.9	93.2	105.2
7.59	4.14	100.6	96.4	105.6	97.8	103.8	100.2
9.39	2.32	98.2	100.4	95.4	95.5	97.4	97
6.09	2.7	102.2	95.4	90.8	107.9	105.4	96.7
8.27	2.49	95.4	90.2	86.3	119	89.4	91.9
6.06	2.33	146	124.3	164.2	52.5	61.1	66.3
6.8	2.73	84.8	82	75	139.7	135	147.8
6.48	1.69	114.3	101.2	99.1	107.5	97.1	114.4
6.52	2.72	102.3	105.4	86.6	92.4	97.7	96.9
5.19	2.25	98.8	109.2	104.8	100.6	101.5	92.7
8.95	2.27	102.1	94.7	103.4	108.6	98	113.1
5.26	1.93	93.7	83	92.3	86.2	63.9	104.3
8.03	2.25	109.6	84.5	94.9	98.7	83.4	97.2
5.16	1.96	102.6	97.9	110.7	120.2	110.9	95.5
5.94	2.65	108	107.6	87.6	99.2	68.8	98.1
10.29	2.25	123.4	97.8	101.5	90.5	89.8	97
6.92	2.92	108	110.6	106.5	94.5	94.1	96.5
5.47	1.98	86.7	84.2	101.3	110.7	74.4	118.6
9.06	2.82	108	96	116.5	108.8	99.8	100.3
6.92	2.06	92.6	90.4	108.4	99.7	99.7	95.8
8.35	3.95	108.9	108.2	106.2	117	113.4	112.6
5.62	3	94.9	93.2	97.3	109.6	120.5	124.9
5.82	2.37	114.7	97.8	99.2	109.2	104.7	111.3
4.32	1.97	141.6	87.9	83.7	76.3	105.8	87.9
8.82	2.14	79.9	99.9	92	102.4	83.9	98.1
9.55	2.49	106.9	98.8	84.3	105.1	109.7	106.2
7.53	1.64	89.2	101.3	102	127.2	154.6	102
8.85	2.36	114.4	103.6	103.8	93.5	93.9	102.8
5.83	2.04	132.5	97.5	68	99.2	106.9	98.7
6.51	2.25	106.6	94.9	91.2	121.4	99.2	120.6
8.73	1.78	106.7	115.8	105.7	111.9	80.8	125.3
4.94	2.24	87.7	131.9	113	91.1	70.5	74.9
4.83	2.41	115.7	99.3	100.3	104	88.6	117
5.17	2.41	101.1	109.2	105.7	117.3	113.8	102.9
6.35	2.24	105	91.9	118.4	108.4	90.9	82.3
5.59	2.65	102.2	102.2	120.1	105.3	111.2	112.5
7.06	1.94	131	143.6	110.1	74.7	84.5	82.1
5.48	2.64	101.1	97.1	93.7	93.6	92.7	109
7.23	3.09	97.4	106.1	110.2	112.4	102.9	101.2
8.21	1.66	105.4	96.5	100.1	109.3	101.3	98.2

6.96	5.58	101.1	104	102.5	103.4	91.4	106.7
7.24	2.78	118.8	100.5	118.7	83.3	93.2	86.4
5.26	2.65	93.4	93.7	98.9	104.3	111.6	107.6
6.9	1.79	107.5	68.3	97.9	104.1	132.8	104.9
5.74	1.95	97.5	85.2	87.7	104.8	115.2	106.6
4.97	1.74	90.8	86.7	78.6	100.6	101	114.8
5.53	2.24	88.6	90.9	101.4	95.3	104.8	105.1
9.38	2.26	89.7	103.1	98.4	104.3	109.4	114
7.64	3.44	106.4	101.6	104.7	88.8	98.7	96.8
9.17	2.35	91.9	98.8	87	108.9	100.6	94
6.04	2.4	91.1	82.8	95.3	96.9	116.7	99
9.42	2.36	105	98.7	105.4	92	101.5	102.4
5.27	2.95	91.1	94.4	90.6	113.9	112.1	106.9
6.38	2.12	104.4	102.8	115.5	112	102.8	110.8
8.78	2.67	111.1	90.4	91.1	116.8	116.5	123.7
7.11	2.04	100.8	116.9	117.2	114.8	103.8	102
7.02	2.69	100.2	99.4	95.8	98.4	102	103.8
6.07	2.24	76.2	86.3	128.7	119.4	132.9	104.4
8.19	1.81	87.9	101.4	93.9	95.2	118.7	106
4.89	2.96	107.6	112.8	104.1	88	95.9	100.5
4.56	2.47	96.3	101.4	99.3	95.1	100.8	98.5
6.86	2.26	83.2	114.5	102.5	109.5	117.2	107.4
5.2	2.07	101.4	105.8	99.1	103.2	95.4	105.5
7.78	1.98	138.7	101.4	113.5	111.4	105.3	107.9
7.77	2.51	101.9	101.4	98.1	97.3	85.8	94.9
7.66	1.74	110.1	108.9	91.8	83.3	87.9	91.9
6.83	1.82	90.7	110.4	102.6	104.1	92.4	85.4
8.59	1.86	92.4	85.4	86.2	105.5	109.9	104.3
4.46	2.04	124.5	85.7	88.5	87.4	101.1	84.5
8.1	2.36	108.7	117.3	92.7	109.1	107.8	95.7
8.02	2.63	108.9	112.2	111.1	93.4	107.6	109.9
5	2.27	97.8	107.8	106.6	105.3	115.1	109.2
8.78	1.77	107.9	109.4	99	123.9	104.1	90.1
5.57	3.15	114.2	110	99.9	101.8	104.8	98.3
5.48	2.6	107.2	123.9	120.6	98.5	90.3	113
7.81	1.65	70	121.6	87	98.8	105.4	94.9
7.61	1.94	100.5	103.7	95.4	84.7	96.3	110.3
5.54	3.76	95.2	106.2	66.7	142.7	105.2	117.6
7.01	2.36	110.4	105.6	102.8	105.2	97.1	99
8.56	2.01	90.3	98.7	101.9	101.5	113.6	112
7.12	2.25	95.1	117	95.1	86.8	91.6	86.3
9.54	2.09	90.6	70.7	91.6	111.2	109.1	114.1
8.38	4.09	103.2	98.4	105.5	104.5	112.1	109
6.11	2.62	108.3	103.5	112.8	104	114.7	90.5
6.58	1.8	83.5	65.9	107.7	93.1	110.5	110.3
6.61	2.62	105.3	86.2	110	96	100.4	107.9
6	2.33	98.7	78.4	97.3	107.6	105.5	92.3
7.55	2.28	99.2	91.2	97.5	83	107.3	105.1
5.07	2.51	93.6	92.9	118.4	110.9	105.2	106.6
8.66	2.13	112.9	86.9	53.9	128.3	103.5	94.5
9.42	3.18	92.9	98.7	109.3	107.6	103.9	115.5

5.43	1.95	93.5	89.8	88	106.1	90.5	122.2
9.25	2.05	102.2	99.8	89.5	96.1	105.4	98.1
7.87	2.02	108.6	81.6	98.1	106.3	130.8	83.6
6.16	2.27	119.2	93.8	105.8	109.2	111.6	112.2
7.88	1.98	124.8	107	104.1	124.2	113.6	99.3
9.01	2.02	88.5	93.9	85.4	119.1	101.4	110.3
5.39	1.74	78.6	115.6	128	100.8	92.2	112.9
7.68	2.2	106.3	103.8	106.2	117.5	114.4	118.5
7.23	1.68	109	89.5	108.7	79.2	87.7	87.2
4.91	1.87	129.9	101.3	94.6	88.1	100.1	91.4
5.34	2.11	84.8	102.6	105.3	101.9	95.2	94.6
9.51	2.59	104.9	97.2	100.7	106	84.6	89.1
10.08	2.2	111	99.2	102.7	107	117.7	106.6
9.74	7.56	100.2	102.3	103	100	108.8	103.8
5.6	1.79	100.7	114.9	93.1	92.7	106.4	83.8
8.43	2.03	121.6	90.9	100.1	77.1	82.1	80.5
7.24	1.83	77.1	87.8	106.5	99.8	101.6	87.4
5.86	4.71	97.3	93.1	96.2	106	109.5	109
6.2	3.03	103.3	100.7	96.1	108.2	106.9	100.1
6.67	2.93	97	103.7	97.6	93.3	105.7	99.5
9.69	2.11	130.4	90.4	91.3	78	90.9	104.3
6.8	1.76	104.5	103.1	108.2	100	106.1	98.3
4.67	2.14	105.8	94.3	85.1	100.1	97.8	112.3
5.08	1.93	101.6	106.6	128.8	91.1	83.6	102
8.82	1.64	101.3	99.5	97.1	95.1	92.9	94.1
7.03	4.82	103	105.3	95.9	102.8	95.9	97.9
10.01	1.89	85.2	137.1	105.1	96	114.8	87.3
8.63	2.09	109.9	82.1	113.1	108.9	94.2	115.7
6.55	1.83	93.8	108.1	84.1	89.6	111.2	95.4
7.52	2.09	118.2	128.4	107.4	95.2	96.8	82
9.06	2.53	103.7	110.5	98.2	116.5	114.7	118
9.6	1.73	130	99.3	88.6	87.9	111.2	97
6.32	4.17	107.5	105.4	110.6	90.7	92.3	103.7
5.73	1.61	108.5	95.1	90.4	142.8	103.8	109.8
6.28	1.97	108	95.8	101.5	96.1	88.2	98.5
8.54	2.15	89.7	93.4	89	113.6	108.1	107.1
10.18	2.64	104.2	104.1	108.8	97.7	99.7	106.2
8.51	2.84	102.4	97.7	99.8	95.4	97.3	101.7
6.1	1.96	94.8	83.5	101	107.4	95.2	101
11.84	2.41	112.3	102.8	116	96.9	101.6	88.7
9.58	2.33	99.7	93.1	96.2	104.8	94.9	110.7
5.83	1.82	96.9	122	101.1	80.9	117.1	116.4
7.84	2.39	102.1	86.8	89.5	88.5	95	84.8
9.17	2.47	105.7	98.9	104	101	104.7	99.3
4.6	2.22	108.6	93.1	106.3	113	109.5	89.5
8.54	5.48	91.6	89.8	90.8	90.6	87.5	90.6
6.35	2.39	99.1	104.1	107.2	103.9	94.8	89.2
7.36	2.18	107.7	107.1	101.1	101.7	95.2	123.9
9.6	1.62	88.6	90.7	92.7	121.4	115.7	120.1
7.88	1.96	95	97.8	92.5	94.8	70	109.7
6.9	1.98	88.7	98.8	86.6	108	110	98.6

8.51	1.74	111.1	101.3	117.2	96.1	98.1	99.7
9	1.97	92.5	104.9	99.8	105.9	103.4	117.5
8.48	2.13	121.4	109.6	106.8	95.2	105.1	101.3
6.01	2.66	101.1	101.2	121.8	98.2	98.2	111.8
5.81	2.43	103.2	107	91.4	92.2	103.6	99.4
9.73	3.16	95.7	103.7	96.6	95.9	101.5	95.1
5.21	2.35	94	101.1	99.5	99	100.1	107.6
9.55	1.86	127.9	78.3	82.3	129.2	118.2	160.8
7.85	2.08	106.9	113.9	123.4	104.9	101.7	103.4
7.96	3.47	93.6	88.2	89	101.8	105.7	117.5
6.8	1.88	92	111.3	100.7	102.6	99.6	86.3
9.72	1.67	86	114.6	117.9	86.7	89.1	105.9
7.64	2.53	95.9	90.4	92.3	120.1	121.1	116.9
5.47	1.95	93.4	102	112.7	99.6	108.5	98.9
9.13	1.78	111.9	96.1	116.6	95.2	89.2	115.8
5.01	2.25	91.2	97.6	97.3	106.8	114.5	113.6
8.1	2.3	102.2	105.6	103.2	104.8	113.9	103.6
8.84	1.77	96.2	86.9	99.7	75.5	122.2	76.5
8.97	1.73	88.6	91.4	105.5	115.7	107.7	103.3
8.15	2.79	95.9	103.2	96.6	104.5	110.3	112.1
5.21	1.84	95.3	96.5	86.7	97.1	91.5	92.3
9.99	1.85	106.7	89.1	82.2	120.9	109.8	112.2
9.35	1.66	89.9	93.3	101.2	112.8	107.3	104.6
7.75	2.14	104.8	92.3	91.5	99.8	97.5	88.6
5.88	2.32	113.7	82.1	108.4	92.9	105.2	97.2
5.85	2.14	90.7	116.5	106	95.2	84.8	87.5
8.76	1.7	83.3	77.7	127.5	77.6	75.1	118.6
7.36	3.05	89.6	91.3	90.2	116.6	116.9	113
6.1	2.79	107	106.6	106.6	102.3	96.4	95.3
9.33	2.15	104.6	107.2	90.8	89.7	101.3	109.7
7.88	1.65	119.1	94.7	83.2	98.4	108.2	85.1
6.28	1.7	36	106.3	119	131.8	79.9	113.8
5.11	2.36	129.3	97	99	109	79.6	111.5
6.6	11.15	105.8	103.8	105.1	93.3	88.1	87.4
4.97	2.84	104.6	93.6	97.2	85.6	86.6	84.9
9.52	2.75	105.5	99.1	97.7	101	98.4	95.7
6.8	2.18	92.4	100.7	93.3	97.5	113.8	100.1
8.25	2.19	103.3	102.4	92.4	106	103.4	101.1
8.95	2.44	99.5	83.4	96.6	120.1	118.3	115
5.8	2.03	108.4	107.2	109.2	82	82.9	105.6
5.21	2.02	101.8	98.1	105.6	108.9	111.2	97.6
7.28	2.09	109	94.2	92	103.7	90.7	87.7
4.54	2.22	101	94.1	93.5	103.2	122.1	127.4
5.4	2.73	106	101.5	113.5	109.9	106.2	110.9
9.85	2.08	97.5	117.9	86.6	90.3	102.5	96.7
7.72	5.73	114.1	102.9	104.3	99.6	97.5	91.4
7.62	2.16	99.8	95.6	109.7	102.9	99.8	116.8
8.92	1.61	96.9	100.7	117.2	98.6	93.4	96
6.04	1.85	96.3	92.6	92	109.2	93.8	127.8
8.63	1.91	110.1	75.7	82.6	118.5	104.9	119
5.5	1.74	93.2	100.5	98.4	105.3	98.8	93.3

6.44	2.17	102.7	102.3	105.2	97.5	96.3	98.4
8.44	2.48	79	85.9	65.9	114.7	124.1	119.5
9.38	1.88	80.5	89.4	113.7	99.8	71.7	95.8
6.33	1.84	114.1	106.4	101.3	85.3	94.9	86.2
8.68	2.74	110.6	91	100.8	99.8	95.4	108.5
9.17	4.1	99.4	107.4	110.2	96.2	103.3	107.8
8.24	2.07	108.1	103.4	108.2	94.7	98.5	102.6
5.22	1.82	102.9	102.4	97.7	110.9	100.1	101.8
8.37	2.63	100.3	99.8	91.7	81.8	84.7	99.7
8.09	1.75	116.4	100.8	97.1	98.6	84.7	95.3
7.3	2.06	107.5	102.6	106.7	93	94	97.7
9.07	1.92	95.4	101.1	92.9	120.7	98.6	124
6.68	2.35	94.1	106.3	106.7	105.4	107.2	119.6
9.48	1.68	112.1	98	113.9	104.8	113.9	98.4
5.86	2.22	102.7	107.4	109.6	93.2	88	114.2
6.76	2.07	121.5	84.7	108.5	89.2	78.9	105.4
8.73	1.87	79.2	86.9	120.1	91.8	112.3	122.1
9.88	1.67	103.1	90.7	101.9	100.9	109.6	124.7
4.88	1.69	103.2	104.8	92.6	98.3	92.5	89
5.85	2.81	99.5	90.1	92.8	106.1	94.6	97
7.75	5.34	102.9	101.1	100	86.1	85.2	81.8
6.6	1.9	124.5	71.1	107	88	83.9	107.4
5.2	1.81	85.4	99	99.9	88.4	99	122.5
7.5	1.82	102.8	94.4	100.3	99.6	102.9	100
11.15	1.79	106.7	108	95.9	99.4	119	119.1
7.42	2.51	87.9	96	94.3	98.8	98.1	101.8
8.27	1.82	96.3	108.4	87.4	97.2	99.8	106.9
9.52	1.93	108.9	97.6	98.5	95.9	96.3	99.1
9.14	2.52	96.2	88.7	104.1	98.7	94.7	111.8
5.55	1.67	99.4	88.5	77.3	92.2	101.3	81.6
8.4	2	117.1	93.7	104.7	92.3	105.8	108
7.65	2.35	104.2	99.3	95.6	99.5	89.4	90
6.87	1.79	118.2	85.4	88.4	115.8	88.1	102
5	2.35	97.5	99.3	103.4	106.8	104.6	105.1
7.61	4.32	114.4	118.3	114.3	75	71.5	69.9
7.34	2.54	97.3	88.5	89.8	104.2	100.4	110.3
9.32	1.83	92.3	102.2	84.9	113.5	107.9	93
6.67	1.8	96.9	94.7	112	85	120	112.9
5.29	2.62	91	87.2	110.3	110.4	111.8	110
7.88	2.07	94.8	96	95.1	97.7	99.7	93.2
6.27	1.85	93.4	101	104.8	99.2	102.3	128.6
5.95	2.53	106	108.5	96.6	92.2	94.1	86.9
9.5	2.16	103.8	106.3	119.2	107.9	99.2	98.2
5.73	1.67	101.8	84.3	92.7	98.1	92.3	106.2
7.49	2.09	94.2	103.7	98.4	89	88.1	99.3
6.55	1.89	105.4	96	103.9	96.8	82.7	98.2
5.53	2.07	99	94.3	97.7	112.4	114.7	109
6.51	2.13	89	92.9	114.2	101.2	103.3	96.9
8.9	2.19	110.2	104.1	86.4	115.8	111.2	115.7
6.93	1.88	92.3	86.6	112.3	127.7	94.6	87.8
6.35	4.47	91.6	97.3	90.6	107.4	107.4	96.4

5.81	1.86	75.5	96.4	112.1	90.3	116.1	104
10.27	2.1	104.7	96.2	105.6	108.5	124	116.4
6.81	2.02	128.7	106.8	100.1	118.8	108.3	122
9.33	2.02	132	113.7	94	130.4	85.2	121.8
7.43	2.14	105	90.8	97.1	101.1	97.7	89.2
8.79	2.37	112.9	93.5	104.7	106.3	104.2	89.7
8.6	2.81	102.8	102.5	105.8	90.9	85.1	85.5
6.99	2.56	100.6	98.8	105.3	105.2	101.8	116
5.03	2.53	99.4	110.1	105.9	103.5	91.2	111.3
6.64	1.71	103.2	99.7	122.4	76.4	84.9	98.3
6.54	2.59	103.5	109.6	94	93.7	80.5	84.7
6	2.45	85.5	82.4	87.5	84.1	78.3	85.2
5.87	1.63	101.4	101	101.7	94.7	96.2	90.3
6.71	1.99	112.6	85.6	100.4	92.8	78.6	102.9
9.03	2.26	85.4	92.3	90.7	115.4	102.4	105.6
6.24	2.31	81.9	103.6	98	97.1	98.3	94.9
7.53	1.67	100.7	112.9	96.4	120.4	97.9	134.7
8.29	1.69	95	106.2	88.8	98.7	98	110.5
5.11	1.82	95	85.6	126.3	99.7	139.3	85
9.92	1.8	110.9	98.5	95.6	103.5	92.1	107.8
6.27	2.44	88.8	99.3	95.4	106.6	100.5	93.4
5.1	1.93	90.2	92.2	90.9	101.5	109.9	102.2
10.48	2.02	98.6	110.5	103.5	104.1	102.8	117.4
9.42	1.67	86.8	102.3	103.1	98.8	98.5	99.8
5.82	1.76	94.4	100.2	126	70.3	109	102.1
6.44	2.45	96.5	95.9	97.3	104.8	98.2	92
8.32	2.01	99.9	102.6	92.5	98	99.2	101.9
10.29	2.31	105.1	95.4	92	109.5	122	121.3
7.39	1.69	74.1	90.5	101.8	73.6	82.9	74.8
8.21	2.44	91.7	96.6	90.4	118.8	116.6	112.5
8.95	2.13	99.7	97.6	96.4	93.7	103.1	99.2
8.7	2.82	98.8	94.6	100.3	106.6	102.7	110.4
8.66	2.43	103.6	98.9	104.7	110.2	108.3	101.5
6.54	1.7	104.9	99.3	97.6	113.8	112.3	115.7
5.86	2.1	113.4	89.4	81.8	94.7	121.5	99.1
4.96	1.62	121.6	104.6	113.4	94.8	94.4	117.2
8.19	1.94	90.9	82	101.6	123.4	118	113.4
6.15	2.03	109.8	102.3	100	100.6	99.3	112.1
6.54	1.82	96.9	103.3	94.3	110	108.4	110.7
8.47	2.28	99.6	96	102.1	110.1	98.8	98.2
5.83	1.67	89.1	78.9	108.7	118.3	97.5	74
7.17	2.33	99.1	92.4	92.5	98.2	92.2	97.2
6.2	1.89	101	91.9	94.2	125.1	125.3	122.8
7.02	2.45	96.7	90.6	102	94	83.8	81.8
6.65	2.36	112.1	112.6	99.3	93.7	92.5	87.3
9.99	2.03	107.7	106.7	104.7	105.3	83.9	89.5
8.37	1.94	87.6	105.4	96.8	111.8	116.5	102.8
7.05	1.61	91.4	77.4	100.4	122.3	114.8	105.5
5.96	2.51	95.7	97.4	104.6	102.9	108.4	96.8
6.81	4.26	104.9	92.9	92.7	104	92	110.7
9.33	2.14	91.2	106.1	103.4	100.1	96.8	98.5

4.93	5.54	91.3	98	94.6	97.2	99.1	97.7

Accessior	Gene Name	Descripti	Coverage	# Peptide	# PSMs	# Unique	# AAs	MW [kDa]
P04264	KRT1	Keratin,	50	40	121	33	644	66
P05783	KRT18	Keratin,	71	34	124	33	430	48
P21980	TGM2	Protein-g	57	30	121	21	687	77.3
P35527	KRT9	Keratin,	50	27	70	25	623	62
K7ENT6	TPM4	Tropomyos	61	22	127	2	247	28.5
O15523	DDX3Y	ATP-deper	41	27	47	3	660	73.1
P07996	THBS1	Thrombosp	24	27	34	27	1170	129.3
P58107	EPPK1	Epiplakir	23	26	34	20	5088	555.3
Q5JXI8	FHL1	Four and	60	12	36	4	257	29.1
Q13740	ALCAM	CD166 ant	29	16	28	16	583	65.1
Q99536	VAT1	Synaptic	32	10	34	10	393	41.9
P08779	KRT16	Keratin,	39	18	27	6	473	51.2
P02533	KRT14	Keratin,	33	18	26	4	472	51.5
P08729	KRT7	Keratin,	32	16	47	12	469	51.4
P51911	CNN1	Calponin-	45	11	21	10	297	33.2
Q15746	MYLK	Myosin li	10	15	23	15	1914	210.6
Q9UPQ0	LIMCH1	LIM and c	16	15	19	15	1083	121.8
Q5JXI2	FHL1	Four and	43	9	29	1	210	23.7
P35237	SERPINB6	Serpin B6	36	11	16	11	376	42.6
Q9UI15	TAGLN3	Transgeli	32	6	22	4	199	22.5
P52943	CRIP2	Cysteine-	39	5	11	5	208	22.5
P26006	ITGA3	Integrin	7	7	12	7	1051	116.5
E7ESP9	NEFM	Neurofila	11	11	32	10	877	98.3
Q95365	HLA-B	HLA class	30	8	14	1	362	40.4
Q15555	MAPRE2	Microtubu	28	8	11	6	327	37
K7ERE3	KRT13	Keratin,	16	7	15	1	415	45.2
O43854	EDIL3	EGF-like	22	9	11	9	480	53.7
P43121	MCAM	Cell surf	14	8	11	8	646	71.6
P21810	BGN	Biglycan	20	6	8	5	368	41.6
A0A140TA6	KRT34	Keratin,	18	8	9	8	436	49.4
P35625	TIMP3	Metallopr	32	7	17	7	211	24.1
O94875	SORBS2	Sorbin ar	9	7	9	7	1100	124
P68871	HBB	Hemoglobi	38	5	15	5	147	16
E9PR44	CRYAB	Alpha-cry	30	5	16	5	174	20
K7ELP0	TPM4	Tropomyos	57	6	17	1	69	8
P49888	SULT1E1	Estrogen	27	8	11	8	294	35.1
P19971	TYMP	Thymidine	18	6	8	6	482	49.9
D6RBR1	CAST	Calpastat	50	6	10	1	150	15.5
P01023	A2M	Alpha-2-m	4	6	15	5	1474	163.2
Q16799	RTN1	Reticulon	13	7	9	7	776	83.6
Q02539	HIST1H1A	Histone H	24	5	16	2	215	21.8
P01584	IL1B	Interleuk	21	5	8	5	269	30.7
P02787	TF	Serotrans	4	2	7	2	698	77
H0YC42		Uncharact	18	4	5	2	278	31.2
P15090	FABP4	Fatty aci	25	3	5	3	132	14.7
P26447	S100A4	Protein S	26	3	6	3	101	11.7
V9HW50	ADH1B	Alcohol d	12	2	4	2	375	39.8
P10620	MGST1	Microsoma	9	2	4	2	155	17.6
H0YGX7	ARHGDIB	Rho GDP-d	29	4	4	4	195	22.4
Q9GZX9	TWSG1	Twisted g	17	3	5	3	223	25

P09455	RBP1	Retinol-b	30	4	4	4	135	15.8
Q07092	COL16A1	Collagen	3	3	3	3	1604	157.7
A0A0C4DG3	SFTPA1	Pulmonary	9	1	3	1	158	16
Q13336	SLC14A1	Urea tran	9	4	4	4	389	42.5
H7C5K4	CCDC80	Coiled-cc	8	2	3	2	261	30.5
P55290	CDH13	Cadherin-	7	5	6	5	713	78.2
J3QR68	HP	Haptoglob	3	1	2	1	404	45
F5GXS0	C4B	Complemen	1	3	4	3	1698	187.6
Q9BY67	CADM1	Cell adhe	6	3	3	3	442	48.5
F8W1S1	KRT74	Keratin,	5	3	10	1	543	59.4
A0A024R6I	SERPINA1	Alpha-1-a	5	2	2	2	418	46.7
Q14410	GK2	Glycerol	3	2	3	1	553	60.6
Q9UJ72	ANXA10	Annexin A	8	2	2	2	324	37.3
E5RHP7	CA1	Carbonic	5	1	3	1	251	27.7
E5RFR7	TPD52	Tumor prc	20	2	2	1	111	12.4
P05109	S100A8	Protein S	19	2	2	2	93	10.8
Q02040	AKAP17A	A-kinase	4	3	3	3	695	80.7
A0A0U1RQI	SORBS1	Sorbin an	3	1	1	1	507	58
P62328	TMSB4X	Thymosin	32	2	4	2	44	5.1
Q86YZ3	HRNR	Hornerin	2	2	2	2	2850	282.2
Q96A00	PPP1R14A	Protein p	12	2	2	2	147	16.7
C9JKF1	SAMD9	Sterile a	2	2	6	1	1283	148.7
Q09328	MGAT5	Alpha-1,6	1	1	1	1	741	84.5
Q9P266	JCAD	Junctiona	1	2	2	2	1359	148.3
P27658	COL8A1	Collagen	1	1	1	1	744	73.3
Q9NSY2	STARD5	StAR-rela	4	1	1	1	213	23.8
H7BYP4	SP140L	Nuclear b	3	2	2	1	520	60.5
Q8N5W9	RFLNB	Refilin-B	6	1	1	1	214	22.9
A0A087WU1	MYEF2	Myelin ex	3	2	2	2	547	58.6
A2ACR1	PSMB9	Proteasom	5	1	1	1	196	20.9
A0A087X2C	AGRN	Agrin OS=	1	1	1	1	1930	202.2
P36959	GMPR	GMP reduc	3	1	1	1	345	37.4
O95684	FGFR1OP	FGFR1 onc	2	1	1	1	399	43
P78504	JAG1	Protein j	1	1	1	1	1218	133.7
Q99541	PLIN2	Perilipin	2	1	1	1	437	48
O43704	SULT1B1	Sulfotran	4	1	1	1	296	34.9
Q9BZL1	UBL5	Ubiquitin	8	1	1	1	73	8.5
K7EPC4	KAT2A	Histone a	9	1	2	1	70	8.4

calc. pI	Score Sec	UCA2	UCA3	UCA4	WJ1	WJ2	WJ3	P-value
8.12	358.14	129.8	131.4	130.4	81.5	79.5	80.8	3.03E-07
5.45	393.54	108.3	108.6	106	63.5	63	64.6	1.29E-06
5.22	324.77	111.4	111.1	110.5	66.6	66.1	65.8	2.3E-08
5.24	210.61	136.7	140.5	141.3	83.2	80.3	81.9	3.97E-06
4.63	352.18	158.2	156	146.1	58.2	54.3	60.8	2.15E-05
7.55	143.71	119.4	126.4	131.3	69.5	78.3	79.1	0.000413
4.94	95.28	107.5	107	105.7	66.9	66.1	67.1	3.39E-07
5.62	87.14	116.4	116.1	119.8	68.2	68.6	72.3	1.11E-05
8.27	108.96	108.1	109.7	108.6	50.5	48.7	51.2	3.06E-07
6.25	77.81	117.5	122.7	122.9	78.6	78.9	77.8	1.88E-05
6.29	98.65	74.3	78.9	72.2	118.8	117.5	117.2	3.07E-05
5.05	71.84	129.9	129.8	126.1	73.8	69	69.8	7.61E-06
5.16	76.86	128.1	132.1	128.7	78.7	77.3	81	6.68E-06
5.48	131.84	85	84.3	82.5	131.4	128.9	127	6.79E-06
9.07	71.29	103.2	100.9	102.2	67.6	66.1	67.4	1.75E-06
6.15	66.8	114.2	110.7	114.9	72.8	76.2	77.3	3.57E-05
6.47	42.74	124.9	125.8	125.1	62	60.7	59.7	9.31E-08
8.59	74.67	94.2	88.9	94.4	51.2	53.9	51.3	3.6E-05
5.27	51.05	92.5	94	98.7	148.3	144.2	144.1	2.66E-05
7.33	62.91	129.7	129	127.9	86.6	82.1	88	2E-05
8.72	44.52	130.9	133.1	128.6	85.1	82.1	80.5	1.34E-05
6.77	37.55	116.6	113.9	114.3	73.4	71.3	73.5	2.81E-06
4.86	92.61	59.8	56.8	57.3	168.3	166.9	167.7	4.36E-08
6.3	41.17	134.8	122.1	119.6	61.4	77	73.3	0.001175
5.57	31.69	75.8	81.4	81.8	121.9	125.1	129.8	0.000106
4.81	45.79	78.6	76.4	77.3	126.1	119.2	124.9	3.23E-05
7.28	34.99	117.3	115.6	118.4	75.8	74.7	73.8	1.85E-06
5.76	32.79	127.1	126.6	129.4	69.3	71.9	71.7	1.2E-06
7.52	26.79	130.3	128.6	124.5	77.5	73.1	74.7	1.64E-05
5.06	25.15	111.1	107.9	109.3	61.5	59	62.8	4.82E-06
8.72	36.73	63.1	60.2	58.7	112	110.2	113	4.8E-06
8.31	22.73	111.1	115.4	119.1	61.7	70	63.5	0.000126
7.28	50.3	68	67.3	69.6	131	125.4	124	1.29E-05
7.03	29.74	129.7	132.7	128.3	50.8	50.8	50.2	4.43E-07
5.01	43.23	140.9	145.7	138.8	71	63.9	69.4	1.56E-05
6.62	28.78	150.9	152.2	152.1	62.2	63.5	64.1	2.34E-08
5.53	20.35	80.6	78.7	85.7	126.7	122.1	121.1	0.000105
9.7	24.66	106.3	83.9	121.6	160.8	176.9	153.8	0.009729
6.46	39.71	148.2	146.1	146.7	42.9	43.7	43.9	1.22E-08
4.69	22.99	69.9	62.6	63.6	117.5	134.9	127.7	0.000379
10.99	47.96	96.9	101.5	94.5	152.9	171.4	158.5	0.000414
4.83	22.24	109.2	103.1	101.6	59.4	65.9	60.7	0.000153
7.12	20.91	80	74.9	74.8	120.7	118.1	120.4	2.24E-05
6.19	14.61	114.9	123.9	112.3	74.1	67.7	76.4	0.000535
7.14	19.94	116.9	117.4	118.2	70.6	73.5	71.8	1.01E-06
6.11	16.07	63.8	65.1	63.4	135.7	136.4	131.3	1.93E-06
8.19	15.84	54.5	54.5	62.9	135.7	140	138.2	1.24E-05
9.39	16.37	73.7	79.8	66.8	132.1	127.1	126	0.000196
5.2	13.01	121.5	121.4	117.9	73.8	79.5	77.3	2.88E-05
5.34	17.4	77.8	77.6	79	125.2	125.6	123.1	8.03E-07

5.11	10.88	55.9	53.2	60.1	171.4	166.1	164.5	2.77E-06
7.84	9.83	123.5	120.3	122.9	76.6	70.9	72	1.65E-05
4.93	13.23	49.6	50.4	53.1	150.1	144.6	142.1	3.36E-06
7.15	11.01	157.3	160.2	167.1	59.9	53.5	66.7	2.94E-05
7.24	9.87	112.9	100.2	106.3	66	63.2	60.8	0.000405
4.98	7.39	133.4	125.8	123	76.3	75	76.2	8E-05
6.58	6.33	65.1	86	71.3	120.4	107.6	125.6	0.005916
7.33	11.16	132.7	131.2	126.7	61.2	62.9	63.1	3.69E-06
5.07	6.83	73.8	74.1	71.6	115.6	121.4	118	1.7E-05
7.44	20.18	119.8	119	136.7	80.1	76	85.9	0.00231
5.59	7.96	73.8	77	74.4	125.4	119.8	116.9	6.99E-05
5.77	4.6	76.7	74.9	82.7	108.5	143.2	124.9	0.009977
5.33	6.33	76	70.1	54.1	138.7	136.4	138.3	0.000417
6.6	5.21	72.6	48.7	69.9	140.9	117.9	101	0.015241
4.68	4.7	110.7	103.9	117.2	65.8	78.4	70.2	0.001828
7.03	4.69	95.3	85.1	81.6	44.7	53.2	49.9	0.001365
9.73	3.86	98.5	120.2	88.8	136.6	178.3	169.6	0.019939
6.99	4.03	144.2	126.8	120.8	59.5	57.7	47	0.0007
5.06	10.11	83.8	88.2	88.4	169.6	171.8	171.8	9.25E-07
10.04	4.09	137.2	129.8	167.8	84.7	57.1	78.8	0.007606
9.38	4.92	121.4	122.8	125.3	36.4	28.8	39.7	1.35E-05
7.14	6.01	133.9	113.1	150	72.5	67.8	68.2	0.004326
8.12	2.73	83.7	92.7	70.4	127.3	156.2	139.8	0.005124
7.09	4.78	83.1	67.8	82.7	112.8	136.2	120.1	0.006163
9.61	2.61	101	110.9	96.8	61.9	53.4	40.8	0.002369
6.67	2.48	91.8	112.4	98.8	72.9	60	68.5	0.008994
8.46	2.56	113.3	139.9	134.2	81.1	84.6	82.7	0.004724
5.44	2.62	79.8	86.5	70.7	118.8	114.7	128.1	0.002367
9.22	1.65	138.6	102.2	138.7	67.6	76.8	81.2	0.015999
4.89	2.08	147.1	123.7	137.8	83.7	71.2	82.9	0.001976
6.37	2.2	72.2	83.2	76.1	122.2	114.5	119	0.000454
7.06	2.03	136.4	124.6	126	75.7	50.6	82.5	0.004632
4.81	1.81	86.7	84.9	71.1	135	123.3	143.9	0.002349
6.06	2.33	146	124.3	164.2	52.5	61.1	66.3	0.002254
6.8	2.73	84.8	82	75	139.7	135	147.8	0.000221
7.06	1.94	131	143.6	110.1	74.7	84.5	82.1	0.009418
8.44	2.48	79	85.9	65.9	114.7	124.1	119.5	0.002767
7.61	4.32	114.4	118.3	114.3	75	71.5	69.9	2.64E-05

FC
1.61952
1.689691
1.677582
1.705379
2.656088
1.661966
1.6002
1.68484
2.170213
1.543136
0.637624
1.814675
1.640928
0.650142
1.523123
1.501547
2.060307
1.774297
0.65323
1.506038
1.584982
1.580202
0.345794
1.77846
0.634289
0.627499
1.566206
1.799436
1.701731
1.791053
0.542959
1.770492
0.538644
2.573781
2.082232
2.398314
0.662341
0.634385
3.37931
0.515917
0.606669
1.687634
0.639477
1.609074
1.6327
0.476698
0.415318
0.571911
1.564614
0.626906

0.337052
1.670615
0.350504
2.690727
1.681053
1.68
0.628959
2.086538
0.61831
1.551653
0.621928
0.622146
0.484277
0.531406
1.547575
1.772666
0.634675
2.386114
0.507405
1.970988
3.522402
1.904077
0.583038
0.632891
1.977578
1.504469
1.559581
0.65542
1.682181
1.718251
0.650829
1.853448
0.603431
2.415231
0.572308
1.594281
0.644153
1.603512
