# Supplementary material for: Umbilical cord artery-derived perivascular stem cells for treatment of ovarian failure through CD146 signaling
Source: Signal Transduct Target Ther. 2022 Jul 13;7:223. doi: 10.1038/s41392-022-01029-4 (PMC9276707; doi:10.1038/s41392-022-01029-4)
Supplement: Supplementary file 10 — Supplementary Table 3 [file 41392_2022_1029_MOESM10_ESM.pdf]

Gene Name（TPM4.FHL1重复，存在空缺值）差异蛋白
KRT1
KRT18
TGM2
KRT9
TPM4
DDX3Y
THBS1
EPPK1
FHL1
ALCAM
VAT1
KRT16
KRT14
KRT7
CNN1
MYLK
LIMCH1
FHL1
SERPINB6
TAGLN3
CRIP2
ITGA3
NEFM
HLA-B
MAPRE2
KRT13
EDIL3
MCAM
BGN
KRT34
TIMP3
SORBS2
HBB
CRYAB
TPM4
SULT1E1
TYMP
CAST
A2M
RTN1
HIST1H1A
IL1B
TF

FABP4
S100A4
ADH1B
MGST1
ARHGDIB
TWSG1

RBP1
COL16A1
SFTPA1
SLC14A1
CCDC80
CDH13
HP
C4B
CADM1
KRT74
SERPINA1
GK2
ANXA10
CA1
TPD52
S100A8
AKAP17A
SORBS1
TMSB4X
HRNR
PPP1R14A
SAMD9
MGAT5
JCAD
COL8A1
STARD5
SP140L
RFLNB
MYEF2
PSMB9
AGRN
GMPR
FGFR1OP
JAG1
PLIN2
SULT1B1
UBL5
KAT2A

gene_symbol（存在空缺值）差异基因,SYT15存在两个

RILPL1
AP000892.6
PFN2
IL32
B3GAT2
PKP2
ZMAT3
PKMYT1
FAM89B
GUCY1A3
FBXO45
AMZ2
GCNT4
RBM15B
TRABD2B
ME1
KRTAP2-3
RP11-77403.3
PHLDA2
CBL
XXbac-BPG252P9.9
CENPH
C2orf27A
GAS1
PWAR6
TREH
CYP2E1
HSDL2
NTM
RP11-535A19.1
PIGA
CTSD
RP11-1102P22.3
ZFYVE28
PRDM10
VPS18
SH3RF1
LAMA1
TAF9B
MPLKIP
RMI2
GALNT16
ZNF850
HOOK3
CHORDC1
N4BP1
CTD-2066L21.3
RGS20
PDE1C
FARSB

43个共有[illegible]	Accessio[illegible]	Gene Name
KRT18	P05783	KRT18
DDX3Y	O15523	DDX3Y
THBS1	P07996	THBS1
EPPK1	P58107	EPPK1
FHL1	Q5JXI8	FHL1
	Q5JXI2	FHL1
KRT14	P02533	KRT14
KRT7	P08729	KRT7
CNN1	P51911	CNN1
MYLK	Q15746	MYLK
LIMCH1	Q9UPQ0	LIMCH1
CRIP2	P52943	CRIP2
ITGA3	P26006	ITGA3
NEFM	E7ESP9	NEFM
HLA-B	Q95365	HLA-B
EDIL3	O43854	EDIL3
MCAM	P43121	MCAM
BGN	P21810	BGN
TIMP3	P35625	TIMP3
SORBS2	O94875	SORBS2
CRYAB	E9PR44	CRYAB
SULT1E1	P49888	SULT1E1
RTN1	Q16799	RTN1
IL1B	P01584	IL1B
S100A4	P26447	S100A4
MGST1	P10620	MGST1
TWSG1	Q9GZX9	TWSG1
RBP1	P09455	RBP1
CDH13	P55290	CDH13
CADM1	Q9BY67	CADM1
ANXA10	Q9UJ72	ANXA10
AKAP17A	Q02040	AKAP17A
SORBS1	A0A0U1RQI	SORBS1
PPP1R14A	Q96A00	PPP1R14A
SAMD9	C9JKF1	SAMD9
MGAT5	Q09328	MGAT5
COL8A1	P27658	COL8A1
SP140L	H7BYP4	SP140L
PSMB9	A2ACR1	PSMB9
AGRN	A0A087X2C	AGRN
GMPR	P36959	GMPR
JAG1	P78504	JAG1
PLIN2	Q99541	PLIN2
SULT1B1	O43704	SULT1B1

ABCA1
ANKRD1
MUT
BCAR1
SIM2
C14orf169
CREM
CSPG4
B3GNT4
RPA1
NR2F1
PTGS2
CDK5RAP2
NPPB
CTD-2192J16.15
MARK2
TMEM30A
RP11-253E3.3
DDR1
SCPEP1
TOR4A
CH507-513H4.3
FCGRT
ZFHX2
ANXA3
HS3ST3A1
NPIPB3
FGF10
RASD2
GNG7
ARNT
NOTCH2
HAND1
RP11-705C15.2
FXYD6
PAQR3
DPYSL3
ANTXR2
APBA2
KCNJ2-AS1
TNFAIP8L3
LLGL1
ULK1
PBX1
INSIG1
SORBS2
CFB
TNPO3
MARVELD1
GLOD4
MAFK

RNASET2
LSAMP
AKAP17A
MAP7D1
GPR68
IL18BP
STAT1
ZNF598
ZNF532
STRA6
RP11-172H24.4
ZNF528
CTHRC1
C9orf16
RAN
ABCF2
RAC2
TNFAIP3
HSPB2
ZFHX3
ECE1
PABPC4L
PXDN
FOXL1
ECM1
NRN1
AKR1C1
MMP11
MAP3K3
CCDC85A
SFRP2
P3H3
MUC1
EPB41L4B
MYB
RASSF2
RP11-890B15.2
FPR3
NBPF25P
RPA3
XXYLT1
GTPBP2
STRN4
KLF16
ADNP2
SLC9A7
NLRP1
SUSD1
CCDC42B
ENO1
DGKQ

LTK
HOXC13
MORC2
CLMP
EPHA7
SRRM2
IFITM1
RP11-231I16.1
IRF2BPL
GLT8D2
DCAF13
PIEZO1
PLOD3
ILF3
CAMKK2
BCL11A
MT1L
SMAD6
RP11-335O4.3
TWF1
UBE2Q1
ATP10A
PPFIBP2
TJP2
NPR3
SHROOM2
IFITM2
CSNK1E
RHOJ
TCF7L1
LINC00960
JSRP1
HIST2H2BE
MICALCL
YARS
CUL1
ADGRL2
SMURF1
PHC2
RELB
COTL1
PRRX2
CPEB2
PTX3
CD200
HEY2
IPO4
TRIM46
INA
CADPS2
HLA-B

PROCR
RUNDC1
DKFZP434K028
SDHAP2
GNPDA1
BNIP2
MAP4K5
SNX7
FIBIN
ERCC8
TNFRSF14
IL21R-AS1
CYBRD1
PIGZ
N4BP2L1
CACNA1C
DCLK1
RHBDF1
CTBS
H19
KCTD15
OXTR
LAMA5
GTF2E1
HSPA5
HCG4
MLPH
PARM1
ADGRL1
CDR2L
GALC
ZNF451
HLA-F
RP11-4204.2
TRIM7
SEMA3A
LONP1
ITPRIPL2
CACNA2D1
PTPRZ1
ITGA10
TSPAN8
NDST3
IFI16
BRD1
RPUSD3
S1PR2
HTR7
MSX2
CLSTN1
EHBP1L1

RGL1
GNAI2
CTD-2154I11.2
RP1-95L4.4
FAM91A1
TBX18
SPRY1
DDX3Y
ELN
WDR26
TRAPPC10
CUEDC1
CNTNAP3P2
ERVK3-1
NAALAD2
TNFRSF11B
OCLN
TMEM65
LINC01592
TANK
BRD7
STX4
WDR60
RNF6
GABRA3
HDAC7
RHOU
NPPC
FYN
DBNDD2
UXS1
MC1R
STK39
GBP2
LACC1
ANXA1
RP11-2C24.3
HDAC1
TMEM2
RHOT1
CERS1
TOB1
SQRDL
DSP
ABR
ARHGAP22
ARHGEF16
AC009506.1
LPAR1
ZYX
RP3-512B11.3

EMP1
CST6
SDCBP
SMC5
POGK
JAKMIP2
SORL1
LGR4
IRX3
RBM38
AKAP9
GFPT2
NR2C1
RP11-736K20.5
SELO
POLI
ERP44
RP11-221N13.3
SMAP2
SRF
VIPAS39
DENND5A
COX11
SYNPO
SLC33A1
SRSF3
HK1
HN1L
RGMB-AS1
PPM1L
ERAP2
TIMMDC1
GLUD2
CENPA
CCDC33
ZACN
TMEM99
LINC01503
MTURN
SLC25A24
PTER
GAB2
PCNXL3
CRADD
AC093616.4
ZHX2
GNA11
MYCBP
TNKS1BP1
TBC1D3L
C3

LRWD1
NUDT12
LMCD1
RELT
C4orf26
OGFRL1
TMEM259
NDUFB5
CALM2
TRA2B
ARRDC3
AADAC
PMPCB
FAM213B
GGT5
YWHAE
BBS4
KLC2
CRYZ
BPNT1
WWC3
SERTAD4
ANKRD11
TCHP
HEPH
ALDH3A2
PRSS21
AC004540.5
ARRDC1
SH2D5
CHD8
CXCL14
GSC
INPP5E
RFC5
LCAT
TNFRSF10C
ADCY4
DEPDC7
FXR2
LPCAT4
JUP
CDKN1A
TMEM70
PDGFRL
PCDH18
ANXA2P2
TMSB4Y
MMP8
RNF152
CHST2

MTRR
NECAP1
FBXO42
CAPN12
RP5-857K21.4
ZNF284
EPHX2
PRR19
RP11-706O15.3
ABI3BP
PLGRKT
PSMD1
NAPRT
MOXD1
GLIS2
ADCY6
SPEN
B3GALT6
CHKB
TRIM23
NBEA
FAIM2
CRABP2
AGAP11
KIAA1755
SRP9P1
TMEM173
KIAA1549L
SENP8
ADSL
SCN1B
SEPT10
RP11-87C12.5
RP11-489E7.4
CNOT11
KANK2
PNPT1
LINC00856
XRCC1
NPDC1
CXCL2
SYNPO2L
SERPINF1
FOXO1
EIF1AY
ATL1
ALDH1A1
SYNE3
ZNF316
NAMPT
FAM107B

AK4
NSMF
EPPK1
MOB1B
CCDC134
COX7A1
BIN1
KIF26B
AGO2
HMGN2
HERC2P2
MPZL1
LMNB2
JMJD7-PLA2G4B
PRLR
C11orf73
GNGT1
AL513523.2
KDELC1
PHB2
TCF21
TMEM121
RP11-574K11.31
RP5-1054A22.4
SNX8
ARAP1-AS1
FEM1A
RP11-69I8.3
CHST7
MIRLET7BHG
FERMT3
SLC45A4
RXRB
AXL
ZNF672
PREX1
ALDOC
MLH1
RP11-642D21.1
F2RL2
ANKRD52
CCNJL
ANKIB1
NOP10
PAX8-AS1
CHKB-CPT1B
SHQ1
COL6A6
PKN2
FAM122C
HOXA9

ENDOD1
TRIM11
CTD-2540B15.13
FIZ1
GIT1
ZDHHC11
VWCE
DVL3
HADH
ARHGEF10L
TMTC4
SCN2A
COL21A1
MME-AS1
TNRC18
LINC00578
HPS6
ADGRL4
TTC39C
PMEPA1
ABCA5
RP11-343L5.2
MMP2
NCOR2
SLCO4A1
TEX29
RP11-513I15.6
CPOX
SERPING1
TCEAL3
MOB3B
LRRC47
FRY
SEMA3B
IL13RA1
F3
BST1
STEAP1
PERP
HSPE1-MOB4
PGF
RARA
FHOD1
METTL7A
RBMXL1
FGD5
FBN1
RP2
GALNTL6
TANC2
MECP2

CSNK1G2
TAF12
FAM124A
SEC11C
ITPR3
TCEA3
SEC24B
ZNF420
KREMEN1
MOB1A
PLXNA4
MEIS3
KDM5D
TSN
TCF12
ZDHHC8
ADAM19
OBSL1
COL4A2-AS1
CADM1
RP11-473E2.4
NFE2L1
COL19A1
FGF1
SLC25A36
PARP10
CTD-2587H24.14
NPTXR
GULP1
IFT46
SLC22A23
PICALM
AP1S3
SHROOM4
PLCH2
TMPO-AS1
RGL2
HOXA11
PPAPDC3
SUN2
NHS
COL6A2
KRT80
RCC2
TAP1
CYP27C1
RFTN1
MAP3K8
TMEM237
GID4
TET3

PLXNA1
RBM33
TIMP2
MAGI1
TXNRD3
HMCN1
SLC12A7
RP11-138A9.2
MAP2K6
GPRC5B
ATP13A2
RP11-245P10.8
MYL9
RP11-110I1.6
BAIAP2
ORAOV1
TDO2
RP11-295K3.1
JOSD1
MOV10L1
SLC40A1
NDUFS1
COG2
ANKRD31
UBE2Q2
PGAM4
RP11-295P9.3
ZNF560
TMTC2
SYNC
IARS2
ACAP3
PENK
ATP6V1B2
TULP3
DNAJC12
TSPAN12
SKI
SLC16A4
RCN2
ARC
FHDC1
FAM84B
GPR37L1
CIC
RP3-416H24.1
KCNMB1
EVC
IL7R
WEE1
TMEM168

MUC12
AC005082.12
SH3BP4
CTD-2298J14.2
CRIP2
PLK1
HIST2H2AA4
HERPUD1
GALNT2
SNX2
ARHGAP18
SUSD5
NBPF8
DYNLT3
TYW1B
KRT14
CASP3
CNTN6
EPHB1
PAPSS1
STEAP1B
TMEM88
SP1
ARHGEF39
PTPN14
RHOBTB3
KIF3C
SLC2A3
WDFY2
GLUL
PLXND1
DNAJC14
PHLDA3
ADAMTS9
ACTC1
HSBP1L1
CFI
ANXA8
N6AMT1
PALM2
S100A4
FAM212B
AC009404.2
GLUD1
RBBP6
PDLIM1
MTPN
ZNF775
ADPGK
NDUFA6
MATN2

IL21R
RABL6
ANXA7
CIRH1A
RP11-96O20.4
PDE3A
GATA6
MGAT5
CENPV
HSPB8
RP11-45M22.3
SUSD3
POLR2A
ASB9
HPS1
RILPL2
CH17-302M23.1
PPP2CA
RP11-579D7.2
ANKRD35
RIPK2
AP3D1
MGST1
FAM212B-AS1
PRR7
ELOVL3
RAB32
PQLC3
BAX
COL14A1
ZDHHC18
FTL
ALDH9A1
C19orf47
COL6A3
CTC-347C20.2
FOPNL
UFL1
CHI3L1
MXD4
PIP5K1A
BEX4
AC005262.2
ADORA1
RP5-890E16.4
MRC2
ABHD17A
B4GALNT1
NADK2
ZBTB2
AC008063.2

C15orf52
IL17RE
RP11-44M6.7
SMARCC1
RP11-147L13.11
RNFT1
AC068831.15
CECR1
SCUBE3
RBPMS
GHDC
ZNF281
NFIB
HOXA-AS3
TPRG1L
CFL2
TMEM120B
DLX2
DES
SMPDL3A
XRCC5
RP11-571M6.7
IGSF8
SDCCAG8
SNAI2
RP11-63M22.2
RMDN1
RXFP1
HES1
HDAC3
PGM3
ZNF154
COPB2
CPA4
ISCA2
AC093673.5
TTTY14
CTC-548K16.5
TNFRSF1B
ACVR2A
NELFCD
RSPH9
NPIPP1
AL035610.1
COPS8
TCP11L1
TCF3
C2orf27B
ZNF276
HPS3
CALCOCO2

EPHA4
CAMK2N1
CH507-24F1.2
GALNT4
TMX4
ANKRD37
STK10
TMX1
BICD2
UNC5B
MRPS9
SYTL5
PHF23
TMEM131
CH507-513H4.4
TLR4
OXSR1
IDH3B
ADGRF4
HSF2
GGH
ZADH2
PREPL
RP11-527D7.1
SNX18
KDM5A
OCRL
GABRB3
CD81
RBKS
ABLIM1
VAT1L
TMEM154
LINC00869
PTPRN
SLC16A10
PACS1
EEF1DP3
NEDD4
KDM4B
ATN1
LYRM9
TAF13
CHST3
FERMT1
MLXIP
ST3GAL6-AS1
GSTT2B
MYO18B
GLT8D1
SPG7

TAOK1
ADAM12
DCTD
PAWR
SLC30A6
NACAD
GPN1
NFKBIZ
OTUD4
PANO1
NRSN2
COPG2
ARPP21
SCRIB
CYTH2
PABPC1L
TMEM98
ENC1
UTS2B
CLN8
NOM1
FADS3
IGFBP3
ARHGEF7
FUCA1
NCAPG2
ZNF467
NAA50
CEP41
FEM1B
EBF1
ZEB2
NUTM2B-AS1
PVRL1
FBLN2
TNIK
ITGA3
SNTB2
ZNF543
MAN1A1
ZNF883
SP100
SLC9B2
LINC01013
TLN1
EMX2
MYO18A
SYNPO2
LATS2
ADAMTS5
SLC31A2

PGM2L1
ACSF2
CLNS1A
RP11-361D15.2
MAFB
ERF
ABCC10
GMPR
SOX13
TMCC1
HNRNPUL2
SOX5
PDE4DIP
LMO3
ATP10D
GSTM4
NUAK1
CA12
TGFBRAP1
FGGY
PLXDC2
TTC8
CTD-2033A16.1
PDCD6IP
NUDT16P1
AC021224.1
UBE2M
PTGIS
SLC1A4
TELO2
FAM171B
TSTD1
ELK3
ADAMTS10
ATHL1
AC005540.3
EML3
GABRE
STAT2
UBP1
IMPACT
GLRX
RAB3B
SSR1
H2AFJ
C1orf101
FBXO8
ADD2
TSPAN7
STX1A
SOWAHC

PRIM1
GYG2
SYNJ2
SUCLG2
ARL5A
ZDHHC13
TMEM8A
UBE2L6
PCOLCE-AS1
HSPA13
S1PR1
HERC1
CTB-174D11.3
PNRC1
MID1
FZD7
SLC25A28
HPRT1
RP11-366L20.2
LRRC40
CHSY3
GSTM2
EGFL7
HAGH
CLU
ANKMY2
C9orf69
UTY
MGAT4B
SAMD9L
DVL1
HSPBAP1
TTI1
CH507-9B2.9
MBNL2
ZNF528-AS1
RIT1
GPSM1
QSOX1
ART4
PGM2
GABPB2
CAPN15
TOR1AIP1
FBXO31
FAT1
MICALL2
KIAA1919
ZNF212
PLCB3
EYA2

SIMC1
IGF2BP3
TUSC3
MCHR1
EHD1
NCOA4
CARD16
GPR89A
NANOS3
RASGRF2
SLC44A1
RRS1
TBL1XR1
PTHLH
C2CD2L
POLR3C
CTSB
INPP5F
AC147651.4
DTNB
DGKA
MBD2
RP11-423H2.1
SEMA3D
EIF3B
RP11-1100L3.7
SDC1
DHX35
FAM129B
POLD1
MBTPS2
LXN
NUDT10
CLEC18B
QSOX2
STMN3
ODF3B
CTNNB1
MFSD10
HIST2H2AA3
RP11-61J19.5
TRIQK
BRI3
KLF13
TCTN3
DISC1
HKDC1
FN1
CRISPLD2
LRTOMT
NFIA

ARL10
ERLIN1
KCTD6
SLC16A9
CHMP4A
WNT2
HHEX
AXIN1
AC104654.2
EDN1
RAB39B
ADIPOR1
LINC00205
TOM1L2
RGS12
TRIL
BAMBI
DUSP5
NRGN
LITAF
CCDC144B
EXTL3
OAT
ATG5
CLK4
JKAMP
NAP1L1
SLC24A3
MDC1
ARF3
LZTS1
KMT2A
C14orf80
CACNB3
RPL9P25
PIK3C2A
DGKG
FOXH1
AIM1
PTGES
SLC25A32
VCAN-AS1
MATN3
HS6ST1
CCND1
FAM131B
FOXC2
GALNT11
ZBED3
PLXNB1
MPHOSPH6

GSAP
DUSP8
BMPER
OXCT1
DMWD
PPP1R3C
CTD-2269F5.1
MAP2
STXBP5L
CDC25C
STK32B
UBQLN4
NPY
NTN4
XYLB
LINC01341
ESPL1
SDHA
SLC35B3
FAM83G
DDX46
GPCPD1
RASL11B
RP1-228H13.5
PM20D2
RP11-1L9.1
YWHAQ
SCARF2
KIT
TMEM9
LL22NC03-2H8.5
BCKDHA
DOCK4
CAPZA2
ANKRD9
RECK
GLO1
MIDN
PF4V1
RDH13
PTGER2
VEPH1
CHSY1
NBPF15
MKRN2
MAP3K7CL
CDC7
PPP2R3B
HIST1H2AC
SOD2
OTUD6B-AS1

RP11-421F16.3
PRPF38B
HECW2
ANGPTL4
SYMPK
DGKH
CYTL1
STT3A
LUZP1
CNOT2
GTPBP3
ZNF646
CH507-513H4.5
FOXF2
PPARG
LRP10
SYNDIG1
CNPPD1
NSFP1
CTTNBP2NL
HIST1H2BL
SMN2
PHRF1
UBL3
TEAD2
ZFHX4
KHDRBS3
LIMS2
SAMD4A
DNAJA3
FNIP2
EXOC2
CRYAB
GPR161
ACIN1
PYGB
SORT1
ASNS
ADPGK-AS1
FPGS
KLF3
NKIRAS2
CTC-575D19.1
AC079135.1
BTG1
MALAT1
FANCE
MX2
RP11-295D4.3
ADGRL3
HSPB6

PHKA2
SATB2
FANK1
ABL1
LRIG3
FHL2
MXRA7
BORA
DTD1
AC002398.12
MFSD1
DCP1B
BCOR
RPL7L1P9
VIM
LRRFIP1
MIR503HG
OBSCN
RNF213
RP11-147L13.13
CD93
ATG9B
ASB18
AP2A1
MIR210HG
NARS
KLHL30
GCNT1
SLC9B1
CRK
NEDD9
PPP1R9A
ANXA2
UNC13A
NCKAP5L
GBA2
GNAS
SUGCT
STK38L
IFFO2
KRTAP7-1
NUMBL
CLK1
PTPRF
CACNG8
RFT1
HTRA1
LMLN
PPP1R14A
HMG20A
YBX3

SPOCD1
DIRAS3
ADAMTSL3
NBPF26
WSB2
KIAA1467
SYCP2
AARSD1
VPS37C
HBS1L
FIGN
DCBLD2
ZNF428
LSM11
STK32C
TRHDE
YLPM1
FMNL3
C19orf68
SLCO3A1
HNRNPK
TNFAIP6
COL4A5
RP11-1223D19.3
DPYD
TNFRSF10A
CASC15
AGRN
ARMCX7P
HPCAL1
ZFPM2
COPZ1
FAM214B
RASSF5
EBLN3
TMBIM6
FGF13
KLF2
KIAA1462
FLNC
GPD2
ASTN1
KDM1A
CTD-2369P2.8
MYO5A
TRBC2
AKIRIN1
AEBP1
CERK
ADAM23
PCDH7

C2orf88
DUSP6
SLC38A5
C10orf107
CAPS2
VTN
MAP3K5
HMGA2
KIAA0355
ITGA7
COL6A1
GPS2
GHITM
AC007743.1
FLRT2
BIRC3
CCNG2
FAM189A2
SRSF6
DLK1
PRKD2
UBE2D1
ANKRA2
NUP62
AC097662.2
PI16
ATRNL1
HOXA13
TMEM132A
CCDC28A
LOXL1
MASP1
C6orf1
NR2F2
MARK4
TYRP1
NKX3-1
PLEKHH3
FBLIM1
MITF
GEMIN8
H1FX
SERPINB2
ADRM1
RNF167
RP5-956O18.3
XXbac-BPG283O16.9
EIF3E
DIRC3
LRRC8A
NELFA

TBC1D10B
ZWINT
DLD
ITIH3
PPP1R9B
HEXIM1
ISG15
CTC-444N24.11
RP11-462G12.2
SDC4
IDH3A
HMGCR
SPOCK1
SMG1
B4GALT2
CLIP1
SMIM3
PCDH9
MAT2A
RP11-498C9.3
ABCD1
VEGFB
PKIA
QPRT
ATF1
PXK
HIBADH
FBXL4
KRT8
MRPL34
ZNF805
SH3PXD2B
EDN2
PRELP
TGFB1
KITLG
PRUNE2
UNC50
RP11-265D17.2
NINJ1
JADE3
EMC2
SCAF1
CPNE7
JAG1
MAD2L2
PNN
COLEC12
STON1
IDUA
HAPLN3

HLA-C
TRAF1
DAW1
STX12
AC005606.15
MAT2B
CPTP
STOX2
SEMA6A
IGSF1
ZNF677
RP11-286H15.1
PPP1R18
PPP1R12C
WDR82
PODNL1
LY96
ADM2
TUBE1
RAB33A
ABHD4
EPHA2
ZNF629
IRF2BP1
BAZ1A
ZNF185
ZKSCAN8
LYPD6B
MYOF
TPM3P9
WASH1
FGFRL1
ZKSCAN1
OSBPL3
PGR
MSMO1
FGF7
RP5-857K21.6
ADAR
HBEGF
SETD1A
TSPAN13
NBPF10
CDR2
RPL36A-HNRNPH2
C1orf198
SLC30A5
SLC35D1
MKLN1
ATXN7L3
SETD3

SEMA5A
PI4KAP1
HOXD-AS2
RABEP2
COL4A1
CARHSP1
RAVER2
FKBP1B
ST6GAL1
RALGPS2
AATF
CCDC152
IDI1
ZNF768
POM121
PKNOX2
KCNK1
GABRA5
XIST
RP11-307C12.12
ERAL1
NOL6
COQ5
S1PR5
RERE
RP11-566K19.6
EMILIN1
MBOAT2
CLDN14
SYNGR2
EFEMP1
RP11-706O15.5
TMEM102
KIF5B
XXbac-BPG248L24.12
GRAMD1A
ZBTB4
KLHL17
GATAD2A
ANOS1
DERA
TMEM209
BACH2
ARL4A
MAGI3
SOX11
KIF7
MTM1
CLCN4
MARCKSL1
UBE2O

KDR
SRGN
LIMD1
ZCCHC9
CYB5B
ADM
CDR1
MEF2BNB
CAPRIN2
FBXL6
RIMS3
FLRT3
MX1
NOTCH3
MME
ARID1A
NR3C1
FBXO2
RP11-627D16.1
PYGL
MPP6
CDR1-AS
DDX39A
C11orf95
FBF1
API5
RP11-175K6.1
TENM4
PNMA2
PLAC9
KLF5
ARCN1
SH3GL1
THOC6
COLEC10
ARHGEF18
G3BP1
MALL
MXRA5
SYT15
RAET1G
TMEM38A
USP5
ANKRD44
SLC41A2
C19orf81
PDCD1LG2
PRR16
ZNF592
HNRNPLL
ZEB1

PRSS35
C1orf162
ZNF175
C5orf15
CCBL2
NRAV
TBX3
CLDN11
DYSF
UNKL
TFPI
ZNF582
CNN1
SLC9A3R2
ZNF142
GCLM
FLNA
PDGFD
GSK3B
SLC7A2
LUM
CTA-390C10.10
RIPK4
AMPD3
GSTM1
ADAMTS1
GNB3
PRSS23
TIGD5
JPX
KLF8
SCRN1
FDX1
F2R
EREG
MSN
TRAC
TRIB2
VAX2
DPF2
CDK11B
RP11-356I2.4
RABL3
DPP10
TSHZ2
FOXC1
DDX3X
SLC17A9
SIDT2
PDZRN4
SCFD1

MMP3
PROSC
ZNF702P
PTGER1
TSPYL4
SMURF2
PRPF18
PWWP2B
LYSMD4
RIC8A
CD72
TINAGL1
IL33
OR2A20P
TFDP1
ERGIC2
ISLR
CYHR1
MYLK
SRP68
IL17RC
CLIP2
MAP3K14
PPIL3
SOX6
PLCL1
ZMYND19
MEST
HAND2-AS1
CTD-2531D15.4
PODXL
LINC01583
MED13
ZNF671
DSC3
SLC26A10
PRDM16
PLXNA2
CTD-2033D15.2
DBF4B
JUND
ANKRD28
SDC3
NTSR1
NALCN
SHMT1
GPR85
3-Mar
HGF
DKFZp434P228
RP11-357H14.17

ATP8B4
SLC29A1
REXO1
GEM
MICA
RRAGC
DBN1
RP11-894P9.1
KCNN2
ALG2
RP11-809N8.2
TPI1
ARSB
DHRS3
ACTG2
IL1B
TSPYL5
GALNT5
COL18A1
RP11-201K10.3
NSF
SLC43A3
HPSE
WEE2-AS1
WDR11
DLG5
FAM101A
GBP3
FBRS
KDM2A
NACC2
RP5-875H18.9
RP11-408A13.4
SLC9A9
MAP4K4
JAKMIP2-AS1
MRPS22
RP11-999E24.3
USP32P1
NKX6-2
RP11-203B9.4
ARL6IP6
KCNG1
TBC1D23
FBXL21
FRMD6
IFI35
BMP1
TMEM119
PTTG1IP
GGPS1

GAP43
CDKN1C
PI4KAP2
PLEKHG3
LAMP1
SLC3A1
UGP2
TIMM21
C8orf58
BZW1P2
NAV3
DLC1
UHRF1BP1
TNFRSF10B
ITGA5
PKP4
KAT7
LPXN
CNTNAP3B
ELL3
OLFML3
ABHD14B
SEC23B
PLD5
ZNF215
AKAP12
PSD3
ZCCHC4
GSN
CPXM2
BCO2
RAB9B
MARVELD2
CARNMT1
DHRS1
CCDC85C
RP11-24B13.2
AFAP1
ZFHX4-AS1
TGFBR3
NFE2L2
RRP7A
CCDC106
AC012462.2
ADAMTSL2
MAST4
CH507-254M2.1
ELOVL2
AC007319.1
DAB2
LARGE

RP11-334J6.7
ERICD
TECR
SH2B3
PPP1R14B
CTD-2373N4.3
TMEM246
C1R
FRK
RASA4B
TCEANC2
ACAN
PCSK7
TPD52L1
NSMAF
FAM86C2P
FHL1
SLC19A1
CATSPER1
CST2
FNDC4
SUCO
ZC3H12C
ADAMTS15
KBTBD11
RP11-482H16.1
GPR143
NBPF1
ECM2
LMO7

DKK2
RP3-394A18.1
KIF21A
LPIN3
TACC2
INTS1
SLC11A2
FECH
PPP2R3A
CD46
SIX2
GPC1
AC002044.1
FBXL2
RNF41
MEGF8
RP11-433J22.2
LINC00619
GREM1
TVP23A

STC2
SYS1-DBNDD2
CDYL2
PDE9A
RP11-555G19.1
EMC10
RAB27B
ZFP37
TWSG1
ARHGEF6
PTDSS2
SLC25A29
SPRTN
THBS1
C9orf64
AC007191.4
STK17A
IP6K3
GMPS
HOXA10
LRFN5
SH2B1
UGDH
STT3B
STK24
EPHB4
RPS4Y1
RP11-982M15.2
ANAPC2
CBLN2
SLC6A1
EBF2
PRDM1
PAK1
ZBED6
TBX2
TIMP3
SSBP2
ALPK3
AMPH
RP11-792A8.4
SMS
ZNF496
LINC01006
RP11-1151B14.4
LAMA2
ZGPAT
TNC
NRG1
PTGFR
CITED4

LMO7-AS1
DPP4
ZNFX1
HOXB7
SMO
ARHGEF3
GRB14
CTXN1
SDHD
PLBD2
CNIH1
HNRNPL
IPP
CNTNAP3
PARD3B
TM2D3
FAM46C
PDE10A
SERPINB9
RP11-865I6.2
DIAPH1
PRKAR1A
BOLA3
LLNLR-284B4.1
XPO6
GALNT10
ELMSAN1
SIAH2
RP11-43F13.3
RBM6
C1orf54
HHIP-AS1
MT1E
MKX
BAG2
APOL4
SKP2
MLKL
PPP1R14C
UBE2W
CTA-228A9.3
ALDH1A3
GPX1P1
ADAM15
ZBTB47
ITPK1
DCAF17
DDX51
BTBD11
RUNX1T1
LZTS2

CCNYL1
RAPGEF3
MINK1
FAM155A
CREB3L2
PIM3
BRAT1
SLC4A7
RP11-486O12.2
ESAM
ABLIM2
ROCK1
NUTF2
SASH1
NSDHL
CHCHD10
TBC1D15
SPON2
BRD4
INPP5A
CTD-3014M21.1
ARL4C
LDB2
METTL10
OSBP
GFM2
GOT1
MB21D2
KLF12
FUCA2
SPTAN1
GZMK
AURKC
TOMM34
WWTR1
HHIP
LCA5L
GINS2
TTTY15
RTN1
TMEM38B
ALKBH1
STK11IP
RAPH1
SIPA1L1
BRD3
DHX33
THOC3
DUSP4
PLCXD1
TBC1D12

SLC5A3
KIAA2013
MFSD12
SEC24D
SPAG16
RAI14
MYO1D
MSL1
TEX41
RP1-56K13.3
SOCS7
RP1-152L7.5
HIAT1
ACADSB
HLA-A
VPS9D1
RP11-51F16.1
RP11-175K6.2
KIAA1217
EEPD1
ARMC10
ZC3H13
LRFN4
C1orf115
NRP2
ZNF652
GJA5
LRRN1
BEND3
RADIL
HEYL
CHST15
MIR143HG
HTATSF1P2
COQ3
NDUFA5
FADD
CPSF3
MRPL37
PPAP2B
TANC1
PCDH10
CXCL1
F8A3
MAB21L2
USP4
LPCAT1
SEMA3C
EFHD1
BET1
SLC41A1

ANKRD34A

NEFM
HACD4
LINGO2
IGFBP7-AS1
PSPH
NUCB1
LIPA
ADAMTS7
NET1
IFT57
SWI5
SSH1
FEM1C
TFPI2
PCDH1
UHRF1BP1L
HRAS
IFIT1
PAGR1
LRRC8D
BST2
KLHDC2
MEX3B
PKP1
VRK1
SLIT2
RHBDD2
DPYSL2
TNK2
UFM1
AC005562.1
IGFBP5
PHF11
CTD-2545M3.2
PRKCSH
THSD7B
AP001062.7
WTIP
LINC01091
CD83
SUCLA2
SPEF1
CD151
APLP1
ZNF431
NPIPB11
SNCA
ORC4
RASA4CP

ARMC9
TUBGCP3
FAM65A
ACKR3
RNF17
RP4-541C22.5
PLS3
RP11-490M8.1
TTC13
FOS
DSE
GPRC5A
STK40
MCU
PEX6
ULBP2
SLC39A8
VWA5A
CYFIP2
ARHGEF11
AUH
WIZ
KIF3B
DNM3
STARD7-AS1
PRCP
FAM20C
ZNF704
KIAA1324L
HIST1H2AG
RAB40C
CCDC3
KAZALD1
ETV6
PTRF
PDHX
RIN3
NCK1
HOXD11
CA13
BLID
RNF13
BMP2K
NEU1
CTD-2006M22.2
GCN1
CPSF1
HDGF
AC092171.4
PXMP4
AREG

BCKDK
UBFD1
CHN1
PCGF3
YBX1
KLHL12
B3GALNT1
RGS17
SLC7A7
RP11-736K20.4
AGT
IMMP2L
SCN9A
ANKRD36
GPR183
TVP23B
ARRDC2
ASXL2
RGS4
EMBP1
LENG9
MCAM
DLEU2
FNDC1
ADAMTS4
HSD17B7
ATP6V1C1
C7orf43
LRRTM3
SKA3
PECR
NOVA1
COL5A1
OGDH
RP11-488C13.5
SARDH
TMEM97
PLEKHM2
RP11-1017G21.4
MYH9
POT1
KCNK3
VRK3
RP11-863P13.3
KCNMA1-AS1
SLC16A1
MFAP3L
CNTROB
ARAP2
PAN3
ACSL3

RSL24D1
ROR1
PPP1R3G
CTSO
PTPRD-AS1
DPT
GLI3
RHBDF2
ZNF282
ALPK2
SLC4A2
PLXDC1
PQLC2L
HERC2
NIPSNAP3B
LTBP2
TCEANC
PSKH1
CDH13
RP11-244F12.2
COG6
PHF20
SETD8
CXCL3
EDIL3
SSBP4
MAPRE1
THNSL1
RAP1GDS1
GALM
RNF149
ATMIN
EGFL8
TNPO2
PTPN3
KCNMA1
OTUD6B
IGFBP2
VIL1
LAMC1
TGFBR2
RP3-329E20.2
LAYN
EPB41L4A
ADGRB2
TRIP10
RP11-801F7.1
PLAC1
SLC39A4
DBR1
FAM174B

RCHY1
FBXL7
TNS2
CCDC81
CC2D1A
PIDD1
CAMK1D
NCK2
PINK1
EFS
ATP8B1
IL1RL1
EIF4EBP3
TRANK1
SLC6A6
CRHBP
CHRNB1
SLC35B1
CTD-2162K18.4
RNF215
SLC35A3
OSER1
HTT
CHRM3
PDAP1
GPX4
LTBP1
ATPAF1
ERRFI1
RP11-274H2.5
CYP27A1
EHD3
ATP9A
WDR62
TRAM2
GPR162
NFKBIA
TSPAN2
RP13-463N16.6
CTD-2031P19.5
SNRNP40
ECD
PDGFRA
TP53INP2
NCDN
CTD-2026K11.1
AVPI1
PDZRN3
ASCC1
SVEP1
BEND7

DNAJB9
ELL2
CACNA1H
IPO8
ASS1
KMT2D
FSTL1
TMEM106B
SLC22A3
VPS53
MAN1C1
PPP1R37
MFGE8
DTX4
FZD2
F8A1
GOLGA8B
SWAP70
SPIRE1
EPG5
DSC2
HID1
NID1
TBX5
SURF6
ITFG3
NEURL1B
TUBA4A
PIK3R1
SLC46A3
RP11-367J11.2
RP11-259N19.1
MECOM
B3GNT2
NDUFV3
WI2-1896014.1
DIXDC1
AES
STXBP6
RP11-157P1.4
AP1AR
CD109
KIF20A
RP11-12J10.3
FAM86FP
AHSA2
CASP1
ICAM2
AC159540.1
CD4
GAREML

MFRP
SYT13
VCAN
FOXK1
TMEM116
PRKG1
PGRMC2
RP11-25K19.1
SGCA

EMX2OS
CAP2
TTC26
CXCL6
SLC22A17
RP11-45M22.2
HAS1
ARFIP1
C6orf62
RAD9A
XRCC4
MIB2
THBD
ARHGEF17
PPAP2C
FAM120A
COPS6
HNRNPD
CYTH3
HSPA8P11
VBP1
RHBDD3
HSPG2
SYNGR1
TECPR1
DLGAP4-AS1
TMEM184C
LPAR3
TNFRSF25
FAM101B
KIAA1644
TENM3
RP11-757G1.6
FKBP9P1
AC006547.13
MT2A
GIPC1
GBGT1
PALM
RP11-347C12.1
PIP4K2B

BDNF
HOXB2
DCLK2
EIF4G3
KIAA2026
GABRB2
TBC1D16
GPR173
XAF1
TSEN15
PITPNM3
SLC4A4
GOLGA2P7
QPCT
SEC23A
HOXB3
DYRK2
FRMD4A
RGS5
C10orf54
AFF3
TRIM28
NT5E
AR
WBSCR17
C6orf47-AS1
VAPA
RP11-397A15.4
MFAP2
LAMB1
AC004988.1
RASA4DP
PPM1F
INF2
ANGPTL1
COQ2
CHRM2
MEIS2
ENOX1
RRAS
NUDT21
ZNF358
RP11-290L1.3
DOK5
FUT1
C15orf39
RP11-169K16.4
SH3TC2
SEMA7A
SOCS1
WARS

C1D
FZR1
CXCL5
ARHGAP20
ZSCAN18
SPRY4
ZNF800
PDLIM3
EXOSC2
PPP1R16A
LARP1
ANKH
ETS2
PTPN23
GADD45B
INHBA
ALG6
PRMT5
ARHGAP12
HBP1
ROBO2
MMP17
POLRMT
RBM24
FDFT1
LIN7C
CNTN1
RAB11FIP3
FEZ2
C12orf29
DCN
ARMCX2
RP11-114H24.6
STAU1
AKR1B1
MIER2
C5orf46
UBN1
LIMCH1
CH507-254M2.2
SLC5A2
TMEFF2
ALDH6A1
RGS2
GMPPA
RP11-707G14.1
ATL2
VLDLR
RANGAP1
ITGB5
CD59

TSIX
ERG
RAD51B
CEMIP
YWHAG
RP11-543C4.1
PNPLA6
BCCIP
PPP3CB
PANK3
CEP170
POLM
CH17-360D5.2
HEXB
FBXO7
TPRN
ACP1
SIPA1L3
GNAZ
PPP1R13L
FENDRR
PURA
COMMD8
SPTLC1
KLHL3
AMOTL1
TMEM184A
PCDHB16
DYNC1H1
DOT1L
SLC47A1
IGFBP7

ZFX
TTL
UACA
PKD1
CUL2
RP11-22H5.2
B2M
FDCSP
RP11-60C6.5
C1S
MFSD11
KCNK6
SGIP1
HOXA5
RASA4
PPP2R1B
SRRM5
RASGRP2

NLRP2
PLOD2
GTPBP1
TMSB15A
LRRN4CL
PRPS2
NNT
PRKAA2
LYSMD2
VCL
RABGEF1
MGST2
PTBP3
DTWD1
PIM1
MFAP4
LOH12CR1
LSP1
BCL7A
SMARCA4
RP11-568K15.1
NUPR1
WFDC21P
TATDN3
PAM
DAGLA
TMEM183A
GUCY1B3
GOLGA8A
CHRNE
RPN1
MEF2D
SVIL
CCDC102B
FAM219A
TBX4
6-Mar
GAS7
AP5M1
SGSM2
COL11A1
CTD-3252C9.4
MAFG
RP11-861E21.2
DGAT2
MARS
ICAM1
ALDH16A1
PRMT3
TPMT
CNTNAP2

MYCT1
KRT19
MEIS1
CXADR
GFRA1
SLC38A4
GIPC3
OSBPL8
KIF1C
KRT79
BEX1
MYD88
KCNS2
PACS2
KLHL4
NCAM1
EDNRA
PGRMC1
REP15
SORBS1
TLE2
KLF3-AS1
TUT1
DMRT2
SMN1
TRAM1
RGS7BP
NQO1
HSD17B14
STXBP2
ST3GAL2
CLEC18A
RP11-148K1.12
FZD4
RP11-367G6.3
NEK10
APCDD1L
ANO1
FAM120B
ANKRD17
CLCF1
SNHG14
BTBD1
DEAF1
PROS1
TESK1
KLF4
EFNB2
CCNB3
IQGAP2
CPNE8

NID2
GRIA3
YY1
LRP12
HTR1B
DBNDD1
ABHD3
SDHB
WASF2
RHOB
ZMIZ2
TUBB2B
DAG1
ABCA2
TRIM56
GPC2
CD164
NDRG2
SLC20A2
PNP
SH3PXD2A
PKN1
RP13-1032I1.7
HYOU1
HAS2-AS1
TGFBI
RP11-89N17.4
SULT1B1
CEP131
KLHL21
C21orf58
RP11-380J14.1
PARL
SRCAP
PRSS12
CYB5R2
CEP57
LINC00643
C6orf89
FOXC2-AS1
BAALC
C20orf27
BAZ2A
USP9Y
GALNT6
RP11-632K5.2
FOXP4
DHX37
THAP1
MTCL1
NLGN4Y

CALD1
SCD
CXCR5
PQLC1
SURF4
UBR4
CNNM2
TAF1A
GDF6
ZNF736
RP11-697N18.3
TXNIP
ADGRE5
RP11-5407.3
SMG9
HOXC8
ARL8A
DAAM2
C19orf54
ADCY1
C6orf120
TATDN2
WNT5A
PRICKLE2
DDIT4L
KLHL35
NOV
IREB2
SCAF8
NCAM2
FAM189B
SPRED1
TXNRD1
DENR
ISL2
GALNT14
HIST1H1C
MICAL2
POLD2
SHISA3
CHRNA1
RNF19B
ADCK2
EPSTI1
NXT2
BMPR2
ST6GAL2
ZNF526
SLC27A2
DFNA5
SH3RF3

ISOC1
MIPEP
VPS37B
CDH8
KRT18
DCUN1D4
SNX1
RNF168
KIAA1671
PSAT1
RAMP1
FAM127C
ANXA10
CD3EAP
NFE4
GART
BTBD2
CD74
ATP2C1
RBM7
OLFM2
SERPIND1
RAPGEF1
CCDC88B
CBX3
TMPRSS2
CNN3
AC000032.2
LIN9
AC005786.7
ODC1
YTHDF1
CREG1
MICAL1
ATP13A3
TMEM120A
MLLT1
NOTCH1
FURIN
GRP
RP11-463J10.3
FERMT2
JAM3
RBM47
COL1A1
RP11-185E8.2
MMP24
OR7E38P
FAM46A
SARAF
C15orf48

ZC3H14
CENPM
ZMIZ1-AS1
MICU1
LZTFL1
PTGES3L-AARSD1
ZNF521
SYT15
AC005944.2
SPAG5
RP11-77H9.2
IVNS1ABP
RCN1
CSMD2
SPSB4
SPATA2L
FMOD
KIZ
MEPCE
GSK3A
YIPF5
MEX3A
DDX1
HOMER1
SEPP1
FAM109A
TMEM39A
RBBP7
AC003092.1
TOX
RAB31
VASH1
RP11-745O10.4
LINC00665
IQCG
PLIN2
KRT7
ANXA8L1
DNAJC6
TSPAN6
KIAA0319L
UNC13D
SPEF2
PLRG1
JADE1
STK11
AC002117.1
MAML1
ZNF484
CCL26
XYLT1

KCNE4
IGHMBP2
TECPR2
CTGF
RP11-638I2.6
EEF2
CTD-2192J16.20
HIST1H2AI
PHLDB1
VSNL1
LTBP3
BZW1
CDC23
PPP1R3B
KB-1471A8.1
ZNF853
MDGA1
SEPN1
RIMS2
ERCC6
MYO1C
CSK
IER2
GATSL2
BOC
MAP3K11
BMP6
CDCP1
SLC9A7P1
SIAE
CTB-43P18.1
DISP1
GRID1
HAPLN1
TPGS1
TRIM29
COL7A1
LGMN
LINC00702
ZFY
OSGEPL1
GCKR
C11orf87
MRPS18B
SLC7A5
HDHD1
TAGLN
RHNO1
SV2A
ERMARD
LINC01094

C1QTNF1
APLP2
MESDC1
4-Mar
RP11-9G1.3
GPC3
BCKDHB
VEGFC
NES
EPB41L3
AAK1
DSG2
LRP3
BNC2
CNKSR2
F8A2
FOXS1
GATA3
LINC00460
PRKY
PDE7B
HES4
SPTBN5
NAV1
BACH1
TNFSF4
CTSK
SEL1L3
DPAGT1
PID1
MAPK1IP1L
PRR15
RP11-483P21.2
ZNF502
TNFRSF12A
ACSF3
FLYWCH1
HACD1
COL8A1
SFTA1P
TXLNGY
KDM6B
DBH-AS1
KRT17
C1orf110
ACAA2
BAG6
UTP6
CDK13
TNFRSF10D
NPIPB4

DUS1L
BNC1
LAT
MRPL36
PLXNA3
CTB-47B11.3
MCM6
CXCL12
CCDC50
CDC42BPB
CH507-338C24.1
ZMYM6NB
PRICKLE1
TNS1
SLC36A4
MT1F
7-Mar
TRIM8
EP300
RALBP1
IL6ST
NRXN3
SBSPON
ACAT2
GOT2
SYTL4
TTC38
FAM168B
AK4P1
PCDHGA1
CKLF-CMTM1
SYT16
HMGN4
NCSTN
RP11-734K23.9
SP140L
DAB2IP
TTYH3
PLN
RAB5A
KCNK2
RBM20
NDFIP2
PCGF2
NBPF14
SERPINB7
GBX2
CHN2
HCFC1R1
LIN7A
A1BG

SLC35F5
FAH
PLEKHA7
MBNL1-AS1
CDK6
AL035610.2
LURAP1L
TNFSF13B
SLC38A9
CAPG
ZNF814
RP11-552F3.12
LINC00900
PDXDC1
HAND2
LINC01197
AC007563.5
PDLIM4
RP11-278I4.2
GNG11
MMP24-AS1
C2CD5
TMEM176A
AC005943.6
DENND4B
MROH7-TTC4
FBXO6
COL12A1
SLC39A14
COL4A2
SMTN
PHLDA1
KIAA2012
9-Mar
HOXD3
RP11-328C8.4
DLX1
SAPCD2
SAP30L-AS1
GLIPR1
PRSS3
CNTN4
GK
CHD4
RP11-138C9.1
RBBP4
DMPK
FBXO22
PLEKHG5
RTF1
PGM1

AGA
TMEM128
THUMPD2
ALDH1L1
ABCA3
WASF1
LIN52
SPAG9
RUFY1
PDPR
MCM9
PREP
IL17D
RP11-620J15.3
GRB10
NACC1
CCNC
CDCA3
CRLF3
C14orf1
OSTN-AS1
GSTT2
IFI44
NPAS1
SENP3-EIF4A1
ZNF587
LINC01085
CNRIP1
PGAM1
ARHGEF10
PAMR1
SNTA1
STK26
RICTOR
RASA3
NUCKS1
IDH1
PRPS1
CHML
DRAM1
GP1BB
CTB-109A12.1
WDR45
LMO2
RPS6KA6
VPS26A
ASAP2
ST6GALNAC3
RP11-475I24.9
IARS
MGARP

PEAK1
ABT1
MARK1
AHDC1
MCM3AP-AS1
IL10RB
HRASLS
BCAR3
TMED2
MEGF6
LINC01123
TWIST1
RGMB
LINC01116
KY
GALNT13
MICAL3
ELOVL5
TRPC1
CH507-513H4.6
SGMS2
P2RX5-TAX1BP3
CABLES1
SAMD1
CELSR2
UBASH3B
E2F3
RUSC2
CPEB1
PXDC1
ASAH1
SFRP1
KCNAB1
PKDCC
TNFAIP2
PSMB9
RP5-1172A22.1
LACTB2
RP11-154D6.1
CCDC74A
SLC38A2
SPOCK3
PHACTR2
TRABD2A
PSMB8-AS1
NMT1
G0S2
MUM1
HNRNPA1
SERPINB1
RP11-462G12.1

RP11-54A9.1
LRRC17
COL25A1
FASN
ESRRA
DSEL
MOGS
PITPNM1
KLF10
EXOC3
F8
SHC3
CAT
ALG3
GOLPH3
NHSL1
MMP16
CTH
PFKP
RBP1
APBB2
GATB
C16orf52
MALT1
BCL9L
CHCHD3
PDPN
CENPB
RP3-509I19.11
TCN2
ZFPM1
PPP2R2B
QRSL1
ACTR3C
KPNB1
PCMTD2
FAM219B
POSTN
NGEF
IL13RA2
ZNF330
CHST14
GLB1
NTRK3
LRRC37A4P
NOS3
RUNX3
SERPINE2
CALU
PIEZO2
SLC35E4

LINC00475
DUSP10
RSPO2
RP11-1060J15.4
GPBAR1
WRNIP1
RNF212
SEZ6L2
STRADB
IFNAR1
MICB
RRN3P1
SAPCD1
CNN2
CARD11
CTD-2561J22.5
SH3BP5L
CDH10
SLC12A8
NR2F6
RP11-96D1.11
PVRL3
GDI1
ATXN1
BAP1
RP4-665J23.1
ZPLD1
SGCE
ZNF264
CYB561
MAGEF1
ZSWIM8
ID2
SNTG2
KLHDC8B
TBC1D2B
CTD-2228K2.7
TMEM262
CST3
FAM43A
C8orf48
SAMD9
MRPS6
BCR
RP11-311C24.1
ITFG1
RP1-20B21.4
CAMK2B
MPRIP
ZFAND1
FAM20A

ARAP1
CTD-2033D15.3
KCTD2
FUNDC1
FGD4
LDOC1L
HSPB2-C11orf52
PDGFA
TIFA
ABHD10
CAPN5
CCDC122
ERBB2
UBE2V2
ZNF804A
PPP3CA
FAM118A
RP11-542C16.2
MAATS1
NCS1
IRS2
ZNF214
MTPAP
SULF2
IRAK1
LANCL3
LINC00839
ARHGDIA
AKAP2
CD44
FAM168A
UNC119B
AQP11
ZBED4
RP11-400K9.4
LMOD1
RP11-366M4.11
FBRSL1
B3GAT1
PCOLCE2
LRRC61
BLVRB
BCL2L1
B3GALT2
BDH2
SOD3
KIAA0196
CMIP
CERKL
RPL39L
ITGA11

UBE2D3
FARP1
AC004076.5
KMT2B
ABALON
MAP3K7
LMTK2
SESTD1
NAB2
PPP1R26
ABCC4
FGF5
TMEM251
RP11-70L8.5
THAP5
DDIAS
TNFRSF9
NIPA2
CPED1
ABCC9
LINC00506
GNAI3
OCIAD2
C8orf4
MROH8
TPST1
IPO7
TNFRSF11A
NELFB
PKD1P6
TRAV39
GNAQ
STAT6
EPHB6
SLC7A8
BGN
SOGA1
CTD-2231E14.2
SULT1E1
MYO6
GUCA1A
PARP2
MMEL1
PLEC
NBPF20
FOXF1
TIMP1
ARHGAP21
EIF5B
PPP6R1

Descripti	Coverage	# Peptide	# PSMs	# Unique	# AAs	MW [kDa]	calc. pI	Score Sec
Keratin,	71	34	124	33	430	48	5.45	393.54
ATP-depen	41	27	47	3	660	73.1	7.55	143.71
Thrombosp	24	27	34	27	1170	129.3	4.94	95.28
Epiplakin	23	26	34	20	5088	555.3	5.62	87.14
Four and	60	12	36	4	257	29.1	8.27	108.96
Four and	43	9	29	1	210	23.7	8.59	74.67
Keratin,	33	18	26	4	472	51.5	5.16	76.86
Keratin,	32	16	47	12	469	51.4	5.48	131.84
Calponin-	45	11	21	10	297	33.2	9.07	71.29
Myosin li	10	15	23	15	1914	210.6	6.15	66.8
LIM and c	16	15	19	15	1083	121.8	6.47	42.74
Cysteine-	39	5	11	5	208	22.5	8.72	44.52
Integrin	7	7	12	7	1051	116.5	6.77	37.55
Neurofila	11	11	32	10	877	98.3	4.86	92.61
HLA class	30	8	14	1	362	40.4	6.3	41.17
EGF-like	22	9	11	9	480	53.7	7.28	34.99
Cell surf	14	8	11	8	646	71.6	5.76	32.79
Biglycan	20	6	8	5	368	41.6	7.52	26.79
Metallopr	32	7	17	7	211	24.1	8.72	36.73
Sorbin an	9	7	9	7	1100	124	8.31	22.73
Alpha-cry	30	5	16	5	174	20	7.03	29.74
Estrogen	27	8	11	8	294	35.1	6.62	28.78
Reticulon	13	7	9	7	776	83.6	4.69	22.99
Interleuk	21	5	8	5	269	30.7	4.83	22.24
Protein S	26	3	6	3	101	11.7	6.11	16.07
Microsoma	9	2	4	2	155	17.6	9.39	16.37
Twisted g	17	3	5	3	223	25	5.34	17.4
Retinol-b	30	4	4	4	135	15.8	5.11	10.88
Cadherin-	7	5	6	5	713	78.2	4.98	7.39
Cell adhe	6	3	3	3	442	48.5	5.07	6.83
Annexin A	8	2	2	2	324	37.3	5.33	6.33
A-kinase	4	3	3	3	695	80.7	9.73	3.86
Sorbin an	3	1	1	1	507	58	6.99	4.03
Protein p	12	2	2	2	147	16.7	9.38	4.92
Sterile a	2	2	6	1	1283	148.7	7.14	6.01
Alpha-1,6	1	1	1	1	741	84.5	8.12	2.73
Collagen	1	1	1	1	744	73.3	9.61	2.61
Nuclear b	3	2	2	1	520	60.5	8.46	2.56
Proteasom	5	1	1	1	196	20.9	4.89	2.08
Agrin OS=	1	1	1	1	1930	202.2	6.37	2.2
GMP reduc	3	1	1	1	345	37.4	7.06	2.03
Protein j	1	1	1	1	1218	133.7	6.06	2.33
Perilipin	2	1	1	1	437	48	6.8	2.73
Sulfotran	4	1	1	1	296	34.9	7.06	1.94

UCA2	UCA3	UCA4	WJ1	WJ2	WJ3	P-value	FC
108.3	108.6	106	63.5	63	64.6	1.29E-06	1.689691
119.4	126.4	131.3	69.5	78.3	79.1	0.000413	1.661966
107.5	107	105.7	66.9	66.1	67.1	3.39E-07	1.6002
116.4	116.1	119.8	68.2	68.6	72.3	1.11E-05	1.68484
108.1	109.7	108.6	50.5	48.7	51.2	3.06E-07	2.170213
94.2	88.9	94.4	51.2	53.9	51.3	3.6E-05	1.774297
128.1	132.1	128.7	78.7	77.3	81	6.68E-06	1.640928
85	84.3	82.5	131.4	128.9	127	6.79E-06	0.650142
103.2	100.9	102.2	67.6	66.1	67.4	1.75E-06	1.523123
114.2	110.7	114.9	72.8	76.2	77.3	3.57E-05	1.501547
124.9	125.8	125.1	62	60.7	59.7	9.31E-08	2.060307
130.9	133.1	128.6	85.1	82.1	80.5	1.34E-05	1.584982
116.6	113.9	114.3	73.4	71.3	73.5	2.81E-06	1.580202
59.8	56.8	57.3	168.3	166.9	167.7	4.36E-08	0.345794
134.8	122.1	119.6	61.4	77	73.3	0.001175	1.77846
117.3	115.6	118.4	75.8	74.7	73.8	1.85E-06	1.566206
127.1	126.6	129.4	69.3	71.9	71.7	1.2E-06	1.799436
130.3	128.6	124.5	77.5	73.1	74.7	1.64E-05	1.701731
63.1	60.2	58.7	112	110.2	113	4.8E-06	0.542959
111.1	115.4	119.1	61.7	70	63.5	0.000126	1.770492
129.7	132.7	128.3	50.8	50.8	50.2	4.43E-07	2.573781
150.9	152.2	152.1	62.2	63.5	64.1	2.34E-08	2.398314
69.9	62.6	63.6	117.5	134.9	127.7	0.000379	0.515917
109.2	103.1	101.6	59.4	65.9	60.7	0.000153	1.687634
63.8	65.1	63.4	135.7	136.4	131.3	1.93E-06	0.476698
73.7	79.8	66.8	132.1	127.1	126	0.000196	0.571911
77.8	77.6	79	125.2	125.6	123.1	8.03E-07	0.626906
55.9	53.2	60.1	171.4	166.1	164.5	2.77E-06	0.337052
133.4	125.8	123	76.3	75	76.2	8E-05	1.68
73.8	74.1	71.6	115.6	121.4	118	1.7E-05	0.61831
76	70.1	54.1	138.7	136.4	138.3	0.000417	0.484277
98.5	120.2	88.8	136.6	178.3	169.6	0.019939	0.634675
144.2	126.8	120.8	59.5	57.7	47	0.0007	2.386114
121.4	122.8	125.3	36.4	28.8	39.7	1.35E-05	3.522402
133.9	113.1	150	72.5	67.8	68.2	0.004326	1.904077
83.7	92.7	70.4	127.3	156.2	139.8	0.005124	0.583038
101	110.9	96.8	61.9	53.4	40.8	0.002369	1.977578
113.3	139.9	134.2	81.1	84.6	82.7	0.004724	1.559581
147.1	123.7	137.8	83.7	71.2	82.9	0.001976	1.718251
72.2	83.2	76.1	122.2	114.5	119	0.000454	0.650829
136.4	124.6	126	75.7	50.6	82.5	0.004632	1.853448
146	124.3	164.2	52.5	61.1	66.3	0.002254	2.415231
84.8	82	75	139.7	135	147.8	0.000221	0.572308
131	143.6	110.1	74.7	84.5	82.1	0.009418	1.594281
