# Supplementary material for: Umbilical cord artery-derived perivascular stem cells for treatment of ovarian failure through CD146 signaling
Source: Signal Transduct Target Ther. 2022 Jul 13;7:223. doi: 10.1038/s41392-022-01029-4 (PMC9276707; doi:10.1038/s41392-022-01029-4)
Supplement: Supplementary file 11 — Supplementary Table 4 [file 41392_2022_1029_MOESM11_ESM.pdf]

Accession	Gene Name	Descripti	Coverage	# Peptide	# PSMs	# Unique	# AAs	MW [kDa]
P10915	HAPLN1	Hyaluronar	46	11	19	10	354	40.1
P18065	IGFBP2	Insulin-li	23	6	7	6	325	34.8
Q9GZM7	TINAGL1	Tubulointe	9	3	3	3	467	52.4
P03956	MMP1	Interstiti	26	11	16	11	469	54
Q8N436	CPXM2	Inactive c	7	5	7	5	756	85.8
P05231	IL6	Interleuki	26	5	6	5	212	23.7
Q8IUX7	AEBP1	Adipocyte	15	15	24	15	1158	130.8
P02462	COL4A1	Collagen a	14	13	42	11	1669	160.5
P07996	THBS1	Thrombospc	52	55	155	54	1170	129.3
Q14766	LTBP1	Latent-tra	14	18	27	17	1721	186.7
Q14767	LTBP2	Latent-tra	19	26	64	26	1821	194.9
O60565	GREM1	Gremlin-1	15	3	5	3	184	20.7
Q76M96	CCDC80	Coiled-coi	27	24	53	24	950	108.1
Q07092	COL16A1	Collagen a	14	13	19	13	1604	157.7
Q9H013	ADAM19	Disintegri	5	5	9	5	955	104.9
P08476	INHBA	Inhibin be	42	20	51	20	426	47.4
P05534	HLA-A	HLA class	31	8	11	3	365	40.7
P08572	COL4A2	Collagen a	45	53	168	52	1712	167.4
P13611	VCAN	Versican c	8	27	56	27	3396	372.6
O95084	PRSS23	Serine prc	17	5	6	5	383	43
Q16769	QPCT	Glutaminyl	10	3	5	3	361	40.9
Q92743	HTRA1	Serine prc	13	8	14	8	480	51.3
P24821	TNC	Tenascin (	13	22	30	22	2201	240.7
P26006	ITGA3	Integrin a	2	2	3	2	1051	116.5
P01130	LDLR	Low-densit	2	2	3	2	860	95.3
P39060	COL18A1	Collagen a	8	9	10	9	1754	178.1
P37837	TALDO1	Transaldol	24	8	10	8	337	37.5
P34059	GALNS	N-acetylga	3	2	3	2	522	58
Q9Y287	ITM2B	Integral m	8	2	3	2	266	30.3
O00584	RNASET2	Ribonuclea	28	5	7	5	256	29.5
P06865	HEXA	Beta-hexos	23	12	18	12	529	60.7
P13647	KRT5	Keratin, t	26	16	23	7	590	62.3
Q14103	HNRNPD	Heterogene	8	2	2	2	355	38.4
Q9P2B2	PTGFRN	Prostaglar	9	8	8	8	879	98.5
P17936	IGFBP3	Insulin-li	47	12	35	12	291	31.7
P07910	HNRNPC	Heterogene	11	3	3	3	306	33.7
Q16610	ECM1	Extracellu	51	22	39	22	540	60.6
P04062	GBA	Lysosomal	13	6	6	6	536	59.7
P07339	CTSD	Cathepsin	31	9	16	9	412	44.5
P06748	NPM1	Nucleophos	11	3	3	3	294	32.6
P04264	KRT1	Keratin, t	51	32	74	28	644	66
P51884	LUM	Lumican OS	33	10	72	10	338	38.4
Q9BTY2	FUCA2	Plasma alp	8	4	5	4	467	54
P05362	ICAM1	Intercellu	11	4	4	4	532	57.8
P17900	GM2A	Gangliosic	35	6	10	6	193	20.8
P19338	NCL	Nucleolin	7	4	5	4	710	76.6
P09871	C1S	Complement	34	20	49	20	688	76.6
Q9HCU0	CD248	Endosialir	11	7	10	7	757	80.8
P00751	CFB	Complement	12	8	10	8	764	85.5
P06702	S100A9	Protein S1	25	2	2	2	114	13.2

P08727	KRT19	Keratin, t	22	9	14	5	400	44.1
P10909	CLU	Clusterin	26	12	23	12	449	52.5
P07858	CTSB	Cathepsin	33	11	22	11	339	37.8
P25391	LAMA1	Laminin su	6	16	17	16	3075	336.9
P35908	KRT2	Keratin, t	47	29	50	24	639	65.4
P19883	FST	Follistati	25	7	11	7	344	38
Q08431	MFGE8	Lactadheri	27	9	15	9	387	43.1
P42785	PRCP	Lysosomal	4	2	2	2	496	55.8
P13521	SCG2	Secretogra	9	5	6	5	617	70.9
P36222	CHI3L1	Chitinase-	21	7	14	7	383	42.6
P13645	KRT10	Keratin, t	52	30	64	27	584	58.8
Q9UI42	CPA4	Carboxypep	37	14	42	14	421	47.3
Q8N8U9	BMPER	BMP-bindir	10	5	5	5	685	75.9
P07585	DCN	Decorin OS	30	12	54	11	359	39.7
P98095	FBLN2	Fibulin-2	9	8	9	8	1184	126.5
P24593	IGFBP5	Insulin-li	52	12	31	12	272	30.6
Q6UXH9	PAMR1	Inactive s	14	9	9	9	720	80.1
P24043	LAMA2	Laminin su	13	34	38	34	3122	343.7
Q9BUD6	SPON2	Spondin-2	7	3	13	3	331	35.8

calc. pI	Score Seq	UCA-PSCs-	UC28-UCA-	WJ-MSCs-S	UC28-WJ-M	P-value	FC
7.42	37.99	176	180.5	51.5	47.8	0.000512668	3.5901309
7.5	13.8	173.5	158.3	58.5	66.4	0.006785397	2.6565252
6.99	5.94	155.9	148.4	59.5	62.7	0.001999091	2.49018
6.96	32.49	155	160.3	72.9	68.6	0.001538498	2.2282686
6.87	7.55	117.3	113.6	51.3	55.7	0.002145997	2.1579439
6.57	8.03	116.2	118.3	59.6	52.2	0.003907161	2.0974955
5.11	46.72	161.7	160.4	78.7	83.4	0.000927614	1.987045
8.28	93.92	145.7	144.2	76.5	77.1	0.000140461	1.8873698
4.94	334.78	121.8	121.8	65.6	64.8	4.99407E-05	1.8680982
5.96	51.33	140.3	138.2	78.2	73.8	0.001482115	1.8322368
5.19	128.53	148.5	148	83.4	83.7	2.03047E-05	1.7743866
9.39	5.6	116	126.6	70.6	69.2	0.010645297	1.7353362
9.72	106.92	136.5	136.1	82.2	77.3	0.001884181	1.7090909
7.84	28.7	148.6	138.6	78.5	91.4	0.018817402	1.6904061
8.35	17.43	126.3	121.6	76.5	70.9	0.005250308	1.6818182
8.03	116.58	132	133.1	79	78.7	0.000112684	1.6810399
6.34	21.02	156	144	90.5	90	0.00995084	1.6620499
8.66	415.88	137.8	137.3	83.1	83.2	2.19635E-05	1.6542393
4.51	133.79	130.1	133.7	83.9	75.9	0.007040331	1.6508135
9.42	9.78	115.4	125	78.3	68.9	0.020156092	1.6331522
6.61	5.41	137	133.5	79.7	87.1	0.006173703	1.6217026
7.83	25.48	141.9	135.5	86.6	88	0.004036803	1.5887743
4.89	57.41	124.4	126.7	76.9	81.8	0.003414248	1.5822306
6.77	5.13	127.1	128.7	82.5	82.9	0.000332671	1.5465538
5.05	1.71	114.2	117.3	73.5	77.3	0.003672573	1.5351459
6.01	19.09	125.1	122.8	87.8	76.7	0.017977743	1.5069909
6.81	19.58	87.6	85.1	128.8	130.4	0.001175378	0.6662809
6.74	4.28	70.3	70.2	103	108.5	0.005949262	0.6643026
5.14	6.34	91.7	80.3	126.5	133.3	0.022101927	0.6620477
7.08	16.19	81.7	75.6	125.2	117.8	0.012291904	0.6473251
5.16	31.69	76	73.5	114.3	116.9	0.001943416	0.6466263
7.74	35.73	71	71.7	110.5	112.6	0.000757165	0.6396235
7.81	2.42	92.6	103.6	153.9	155.1	0.009486172	0.6349515
6.61	9.31	69.4	65.7	107.5	105.9	0.002640021	0.6330834
8.69	71.51	59.2	57.8	91.8	95.2	0.002747816	0.6256684
5.08	4.95	89.8	91.5	149.8	141.4	0.006026383	0.6225962
6.71	66.03	78.7	79.9	126	130.8	0.00252894	0.6176012
7.61	6.19	68.6	70.4	113.7	112.8	0.00052856	0.6136865
6.54	30.5	72.4	73.2	121.9	117	0.00281977	0.60946
4.78	7.81	85.9	89	139.5	152.6	0.012937743	0.5987675
8.12	154.76	66.6	67.3	113.4	112.2	0.00022944	0.5935284
6.61	166.77	72.9	71.8	124.9	119.2	0.003393455	0.5927898
6.25	10.35	71.7	69.8	120.8	118.7	0.000834024	0.5908142
7.99	6.71	58.9	62.8	102.8	108.8	0.006276701	0.5751418
5.31	24.27	71.8	73.2	133.5	128.8	0.00174334	0.5528021
4.7	4.61	80.4	89.8	154.8	153.4	0.004709226	0.5522388
4.96	107.25	69.6	69.3	130.1	129.1	7.53089E-05	0.5358796
5.35	15.25	78.7	76.5	142.9	149.4	0.00249589	0.5309613
7.06	11.49	67.5	67.1	127.3	127.6	1.72742E-05	0.5280502
6.13	4.85	61.5	63.9	114.8	131.3	0.018553539	0.509549

5.14	25.43	72.8	71.3	148.3	136	0.007720989	0.506859
6.27	50.62	56.7	58.2	117.9	110.1	0.004895948	0.5039474
6.3	41.01	65.9	67.1	133.7	134.6	0.000122887	0.4957138
6.35	33.71	61.7	54.8	119.1	117.3	0.003518486	0.4928088
8	94.14	67.3	68.3	133.7	145.6	0.006835428	0.4854995
5.67	12.84	70.1	65.9	137.5	142.9	0.00223693	0.4850214
8.15	25.58	56	57.7	119.2	115.7	0.001029082	0.4840358
7.21	5.05	65.5	51	111.4	130.3	0.034347234	0.4820025
4.75	4.62	64.1	62.5	138.4	127.6	0.006078228	0.4759398
8.46	27.33	43.1	37.2	86.2	83.8	0.005004383	0.4723529
5.21	139.02	63.1	64	136	134	0.000235465	0.4707407
6.7	85.08	51.9	52.1	118.1	121.7	0.000704182	0.4336947
7.77	10.37	58.3	54.9	145.3	144.9	0.000373885	0.3900758
8.54	115.92	58	56.2	152.1	147.7	0.000655429	0.3809206
4.82	10.66	52.2	54.8	145.6	137.9	0.002113513	0.377425
8.21	67.94	43.7	45.5	138.4	141.5	0.00035316	0.3186852
7.46	10.37	39.6	36	125.5	137.4	0.004377145	0.2875618
6.4	74.46	42.2	37.5	153.5	153.4	0.000427855	0.2596937
5.52	18.38	26.5	29.8	160.4	160.9	0.000158595	0.1752256
