# Supplementary material for: Umbilical cord artery-derived perivascular stem cells for treatment of ovarian failure through CD146 signaling
Source: Signal Transduct Target Ther. 2022 Jul 13;7:223. doi: 10.1038/s41392-022-01029-4 (PMC9276707; doi:10.1038/s41392-022-01029-4)
Supplement: Supplementary file 14 — Supplementary Materials [file 41392_2022_1029_MOESM14_ESM.docx]

Supplementary Materials for

Umbilical cord artery-derived perivascular stem cells for treatment of ovarian failure through CD146 signaling

Lu Xu^1,2,3†^, Yanjun Yang^1,2†^, Lingling Zhang^1,2,4†^, Guijun Yan^1,2†^, Shiyuan Li^1,2†^, Yifan Li^1,2^, Yali Hu^1,2,3^, Lijun Ding^1,2,5*^, Bruno Péault^6*^, Haixiang Sun^1,2,7*^

Correspondence to: dinglijun@nju.edu.cn, bpeault@mednet.ucla.edu, and stevensunz@163.com

**This PDF file includes:**

Materials and Methods

Supplementary Fig. S1 to S7

Supplementary Tables S1 to S8

Supplementary Data file 1 to 2

Supplementary Videos

Materials and Methods

Immunohistochemistry

Tissues were fixed in 10% (v/v) buffered formaldehyde, embedded in paraffin, and then sectioned into 5-µm slices ^1^. In summary, these slices were incubated with 0.03% H_2_O_2_ in PBS for 10 min at room temperature (RT), autoclaved in 10 mM sodium citric buffer (pH 6.0) for antigen retrieval, and then incubated in 5% normal goat serum in PBS for 15 min. Primary antibodies and respective dilution are listed in Supplementary Table 5, and they were incubated with tissue slices at 4 ℃ overnight, and then, secondary goat anti-rabbit antibody (PV6001, ZSGB-BIO, Beijing, China) was incubated with the tissues for 30 min at RT. Horseradish peroxidase conjugated to the secondary antibody was detected by diaminobenzidine. Images were captured using a Leica DM 2000 microscope (Leica, Wetzlar, Germany). Samples to which the IgG isotype was added were used as negative controls. The number of capillaries and ratio of capillary number per unit area were used to compare differences in capillary density among groups.

Immunofluorescence staining was performed as previously described ^2^. UC tissues were embedded with optimal cutting temperature (OCT) compound, sectioned into 5-µm slices and then fixed with 4% paraformaldehyde, and then, the UC tissue sections were permeabilized for 10 min in PBS containing 0.25% Triton X-100 and rinsed with PBS. Then, the sections were blocked for 1 h in PBS containing 3% bovine serum albumin (BSA; Sigma, Steinheim, Germany). Prediluted primary antibodies were incubated with the tissue at 4 ℃ overnight, and then secondary antibodies were added and allowed to the react with the primary antibodies in the dark at RT for 1 h. The antibodies and respective dilution are listed in Supplementary Table 5. The sections were mounted with ProLong Gold mounting medium with DAPI (Molecular Probes, Invitrogen, Eugene, USA). Images were captured with a Leica DM 3000 LED microscope (Leica).

Isolation and culture of UCA-PSCs and WJ-MSCs

Adherent cells were isolated and cultured using the umbilical tissue culture method ^3^. In summary, two umbilical arteries were extracted from human full-term umbilical cords (n=10). The umbilical arteries were cut perpendicular to the long axis and seeded on the tissue culture dishes. Wharton’s jelly was then manually minced into 1-2-mm^3^ fragments and plated in culture dishes (Supplementary Fig. 1a). These tissues were submerged slowly and gently into culture medium containing low-glucose DMEM (LG-DMEM, Gibco, MA, USA) supplemented with 10% fetal bovine serum (FBS, Gibco), 1% penicillin/streptomycin (Gibco), and 10 ng/ml basic fibroblast growth factor (bFGF, Gibco) and cultured at 37°C and 5% CO_2_. The culture medium was replaced every 3-5 days for 2 weeks. Then, adherent cells grown from the tissue fragments were rinsed once with PBS and detached with a 0.05% trypsin/EDTA solution (TE, Gibco). The cells were subpassaged every 4-5 days at a 1:4 ratio.

Flow cytometry analysis

Cultured cells (from the third passage [P3]) were analyzed by flow cytometry using a FACScan flow cytometer (Becton Dickinson, USA). Single cells were harvested with 0.05% trypsin-EDTA (TE) and resuspended in PBS. The cells were passed through a 100-μm filter and incubated for 1 h with fluorescein isothiocyanate (FITC)- or phycoerythrin (PE)-conjugated antibodies, which are listed in Supplementary Table 6.

Multilineage differentiation

Cells at P3 were assessed for multipotency in adipogenic and osteogenic differentiation conditions in culture, respectively. Cells were seeded at 5 × 10^3^/cm^2^ in 24-well plates and grown in a monolayer in LG-DMEM supplemented with 10% FBS until reaching ~90% confluency. On day 14, cells cultured in adipogenic induction medium (Gibco) were stained with Oil red O (Sigma) as an indicator of intracellular lipid accumulation. For cells grown in osteogenesis induction medium (Gibco) for 21 days, calcium deposition was assessed by alizarin red staining (Sigma). For assessing neurogenic differentiation, cells were treated with preinduction medium containing 10^-7^ mol/L all-trans-retinoic acid (ATRA; Sigma) and 10 ng/ml bFGF (Gibco) for 18 h and then with modified neuronal medium (MNM) for 36 h. The expression of neurofilament medium polypeptide (1:100; sc-16143, Santa Cruz Biotechnology, California, USA) in induced cells was detected by immunofluorescence staining.

*In vitro* angiogenesis

A tube formation assay was performed as described previously ^3^. UCA-PSCs or WJ-MSCs (1×10^4^) were plated onto Matrigel-coated 96-well plates and cultured in DMEM without serum. HUVECs (1×10^4^/well) seeded on the Matrigel bed and cultured in PBS served as the negative controls, while HUVECs in serum-free DMEM containing VEGF (0.3 nmol/L) served as the positive controls. To detect proangiogenic potential, UCA-PSCs or WJ-MSCs (5×10^3^) labeled with CM-Dil (C7000, Molecular Probes) were cocultured with HUVECs (5×10^3^). Following incubation at 37 ℃ for 2–24 h, each well was digitally photographed under a microscope (Leica) with phase contrast. The tubes and branching points were counted. In addition, total tubular length was quantified by ImageJ software (National Institutes of Health, MA, USA) and calculated as the average of the total tubule length in three to five random fields per well of three wells.

Matrigel plug assay

Liquid Matrigel (356234, BD Biosciences, USA) was mixed at 4 ℃ with 5×10^5^ cells or bFGF dissolved in PBS at a concentration of 150 ng/ml and injected subcutaneously (250 μl/site, 2 sites/mouse) into the flank areas of female BALB/C nude mice. Matrigel with PBS alone was used as the negative control ^4^. Two weeks after injection, the mice were sacrificed, and the plugs were harvested, weighed, and embedded in OCT compound (Leica). The plugs were sliced into to 6-μm-thick sections using a cryostat (Leica). Neovasculature was assessed using anti-mouse CD31 (550274, BD Pharmingen), and anti-vWF antibody for immunohistochemistry.

Single cell-colony RNA sequencing

Colonies of primary cells obtained from UCA and WJ tissues were cultured *in vitro* for ten days and digested with 0.05% TE to obtain single cells. The total RNA of each UCA- or WJ-derived cell colony was reverse transcribed to cDNA and amplified with Discover-sc^TM^ WTA Kit V2 (N711, Vazyme, China) according to the manufacturer’s instructions. The amplified cDNA product was purified with VAHTS DNA Clean Beads (N411, Vazyme) and analyzed with an Agilent 2100 Bioanalyzer with a High Sensitivity DNA Chip. Input DNA was subjected to tagmentation with TruePrep Tagment Enzyme Mix, and a cDNA library was prepared using TruePrep^TM^ DNA Library Prep Kit V2 for Illumina® (TD502, Vazyme). Size-selected 450 bp fragments were loaded on an Illumina X ten platform for sequencing.

Raw sequencing data were converted to raw reads using CASAVA base calling. Quality control of the raw reads was performed by calculating the Qphred score with Fast QC. Clean read alignment to reference genome sequences was performed using TopHat (V2.1.1), and the mapped reads of each colony were assembled using Cufflinks (V2.2.1) ^5^. Gene fragments per kilobase of exon per million fragments mapped (FPKMs) were computed using Cuffdiff (V1.3.0). DEGs were measured with the help of Cuffdiff analysis. Genes with an FDR <0.05 and **|**log2 (FC)| >0.58 were considered to be significantly differentially expressed. Unsupervised hierarchical clustering was performed with the heatmap package in R. Statistically significantly different ensemble gene IDs were loaded into the GO database (http://www.geneontology.org/) for assessment of genes enriched in the molecular function, cellular component and biological process categories. Pathways enriched with genes were identified by KEGG pathway analyses of upregulated and downregulated genes.

LC-MS/MS analysis

A total of 1× 10^7^ cultured UCA-PSCs and WJ-MSCs at P4 were lysed with 300 μL of SDS lysis buffer containing protease inhibitor and 1 μM PMSF in 1.5-mL tubes. Each sample was ultrasonicated on ice for 3 min. After centrifugation twice at 2000 ×g for 10 min at 4°C, the supernatant was collected for total protein measurement.

Cell supernatants (5 mL/10^6^ cells) of P4 UCA-PSCs and WJ-MSCs cultured in basic DMEM without serum or antibiotics for 48 h were collected. After concentration in 3-KD ultrafiltration centrifuge tubes, the cell supernatants were frozen and resuspended in SDS solution. After centrifugation, the supernatants from UCA-PSC and WJ-MSC cultures were obtained.

Total protein in the cells or supernatant was quantified using the bicinchoninic acid (BCA) method. Protein samples were separated by SDS-PAGE for quality control ^6^. One hundred micrograms of protein extracted from each sample was mixed with 120 μL of reducing buffer (10 mM DTT, 8 M urea, and 100 mM tetraethylammonium bromide [TEAB], pH=8.0) in a 10-KD ultrafiltration tube. After 50 mM iodoacetamide (IAA) treatment, 200 μL of 300 mM TEAB was added, followed by 3 μL of sequencing-grade trypsin (1 μg/μL), and then, the solutions were incubated at 37 ℃ for 12 h.

After tandem mass tag (TMT) labeling of each sample, reversed-phase (RP) separation was performed on a 1100 HPLC System (Agilent Technologies, Santa Clara, CA) using an Agilent ZORBAX Extend RP column. Then, all mass analyses were performed with a Q Exactive mass spectrometer (Thermo Fisher Scientific, MA, USA) equipped with a Nanospray Flex source (Thermo Fisher Scientific). Samples were loaded and separated on a C18 column in an EASY-nLC^TM^ 1200 system (Thermo Fisher Scientific) according to manufacturer’s instructions.

Proteome Discoverer software (v2.3, Thermo Fisher Scientific) was used to search all of the Q Exactive raw data thoroughly against the sample protein database. The global FDR was set to 0.01, and for protein groups to be considered for quantification at least 2 peptides were required. The identity of proteins in a set was verified on the basis of the following two values: a Sequest HT score >0 and unique peptide >1. Missing values were replaced on the basis of the normal distribution using the data imputation feature in Perseus ^7^. Differentially expressed or secreted proteins in the UCA-PSCs and WJ-MSCs were identified on the basis of a FC >1.5 and p-value < 0.05. A GO enrichment analysis was carried out online (https://www.ebi.ac.uk/QuickGO). A KEGG pathway analysis was based on the KEGG database ^8^. Functional annotation enrichment analyses were performed using STRING ^9^.

Quantitative real-time PCR (qRT-PCR)

Total RNA was prepared from tissues or cells using TRIzol reagent (Invitrogen, Grand Island, NY, USA) according to the manufacturer’s instructions. After quality control, a 1-μg aliquot of purified total RNA was converted to cDNA in a total volume of 20 μL using a PrimeScript RT reagent kit (Bio-Rad Laboratories, Hercules, CA, USA). The primers used for quantitative PCR analysis are listed in Supplementary Table 7. PCR was performed on a MyiQ Single Color Real-time PCR Detection System (Bio-Rad Laboratories) by the following procedure (40 cycles for 95°C for 3 min, 94°C for 10 s, 60°C for 30 s, 72°C for 30 s). Human 18S rRNA was used as the internal control for gene detection. The samples were processed in duplicate using RNA preparations in three independent experiments. The Fold Change (FC) in gene expression was calculated using the 2^−ΔΔCT^ method.

Western blot analysis

P3 cells were rinsed twice and then harvested in cell lysis buffer (50.0 mmol/L Tris, pH=7.6; 150.0 mmol/L NaCl; 0.1% SDS; 1.0% NP-40; and protease inhibitor cocktail). Protein concentrations were measured using BCA protein assay reagent (Thermo Fisher Scientific, Rockford, IL, USA), after which 25 μg of total protein was loaded onto 10% SDS-PAGE gels, and after electrophoresis, these proteins were transferred to a PVDF membrane (Millipore, Danvers, USA) using the conventional method. The membrane was treated with 5% nonfat milk in TRIS-buffered saline and Tween 20 for 1 h at RT. Then, the membrane was incubated with primary antibodies overnight at 4°C (the antibodies are listed in Supplementary Table 8). Secondary species-specific antibodies conjugated to horseradish peroxidase were added and incubated for 1 h at RT. The band intensity was measured using an enhanced chemiluminescence kit (Amersham Biosciences, Piscataway, NJ, USA). All experiments were repeated at least three times.

Small interfering RNA (SiRNA) transfection

UCA-PSCs were transfected with CD146 siRNA, control siRNA (50 nM, RiboBio, Guangzhou, China), Jagged1 siRNA or control siRNA (50 nM) separately using Lipofectamine™ 3000 transfection reagent (Life Technologies, Carlsbad, CA, USA). The cells of each group were seeded in separate 6-well plates and cultured at 37°C with 5% CO_2_ until 80% confluency. Cell transfection was performed strictly according to the reagent manufacturer’s manual. The knockdown efficiency was confirmed 48 h and 72 h post-transfection by qRT-PCR and Western blot analysis, respectively. The *in vitro* and *in vivo* angiogenic properties of UCA-PSCs were determined 72 h after transfection.

*In vitro* activator and inhibitor assays

IGF-1 was administered at specific concentrations for different periods after cells were starved by culture in serum-free medium overnight, and 0.1% BSA/PBS was used as the control medium. Stock MK2206 aliquots (1-100 mM, Selleck Chemicals, Shanghai, China) were prepared in dimethyl sulfoxide (DMSO; Sigma Aldrich) and diluted in the appropriate medium before use. Treatment with matching DMSO concentrations were used as controls ^10^. For Western blotting, cells were harvested after treatment and processed as described previously.

Chromatin immunoprecipitation (ChIP)-PCR assay

ChIP was performed according to the manufacturer’s instructions (Millipore, Danvers, MA) ^11^. In summary, UCA-PSCs (70-80% confluency) were treated with IGF-1 or MK2206 for 6 h. The cells were then crosslinked with 1% formaldehyde for 15 min at RT. After incubation with glycine (0.125 M final concentration) for 10 min, the cells were collected, washed with cold PBS and added to lysis buffer (20 mM Tris-HCl, pH = 8.0; 85 mM KCl; 1 mM EDTA; 0.5 mM EGTA; 0.5% Nonidet P40; and protease inhibitor cocktail [Sigma]). Cell pellets were lysed in nuclear lysis buffer (50 mM Tris-HCl, pH = 8.0;10 mM EDTA; 1% SDS; and protease inhibitor cocktail) and sonicated on ice to yield genomic DNA fragments of 100-500 bp. Next, precleared sonicates were immunoprecipitated using anti-FHL1 (10991-1-AP, Proteintech, Chicago, USA), and nonspecific IgG was used as the control. Beads were collected and washed extensively. Immune complexes were eluted by incubation with fresh elution buffer (1% SDS and 0.1 M NaHCO_3_) at 65°C for 30 min followed by incubation at RT for 15 min. Crosslinks were broken by incubation with 0.3 M NaCl for 5 h at 65°C. The eluates were incubated with proteinase K, and DNA was purified by phenol-chloroform extraction and ethanol precipitation. Finally, the purified DNA fragments were used as templates for PCR amplification. The primers used to amplify the Jagged1 promoter DNA fragments containing FHL1-binding sequence 1 (ACGCA) were 5′-CCAGGAAAGTTTTTCAAAGTTCCC-3′ and 5′-TACTTGGAAGGGATCGTTGCTCA-3′ (-628 to -262; expected size, 367 bp).

Luciferase assays to determine Jagged1 promoter activity

Using the GenBank sequence, an 864-bp sequence (including the FHL1-binding site ACGCA) of the sequence upstream of the Jagged1 transcription start site was amplified. The following primers with restriction enzyme site linkers were used (restriction sites are underlined): fwd, 5′-CCGCTCGAGCTTCTACCCCCGGTTTCC-3′, and rev, 5′-CCCAAGCTTGCAGGTAACACAATGACGCGTG-3′. PCR was performed on an S-1000 thermocycler (Bio-Rad, USA) using genomic DNA and Phusion HS DNA polymerase (Finnzymes, Espoo, Finland). Purified PCR products were digested with XhoI and HindⅢ restriction enzymes (NEB, Ipswich, MA, USA) and ligated into a pGL3-basic luciferase reporter vector (Promega, Madison, WI). After ligation reactions, all vectors were transformed into *E. coli* DH5α, amplified and purified by a SanPrep column plasmid mini prep kit (Sangon Biotech, Shanghai, China). All constructs were verified by sequencing (GenScript, Nanjing, China).

A dual-luciferase reporter assay system was used to measure the activity of the Jagged1 promoter. Preconfluent (60-70%) UCA-PSCs in 24-well plates were transfected with plasmid overexpressing FHL1 or FHL1 siRNA (RiboBio, Guangzhou, China), and then, 300 ng of luciferase reporter plasmids and 5 ng of a pRL-RSV Renilla luciferase reporter plasmid were transfected using Lipofectamine 3000 (Life Technologies) for 48 h. Cell lysates were assayed for luciferase activity using a luciferase assay system (Promega, Madison, WI, USA), and the activity was measured using a luminescence counter (Centro XS3 LB 960, Berthold, Germany) ^12^.

Hind limb ischemia (HLI) in mice

BALB/c nude mice (n=6 in each group) were anesthetized with isoflurane using a tabletop laboratory animal anesthesia system (VWR, 89012-492). Ischemia was induced by ligating the proximal portion of the femoral artery, including the superficial and deep branches, and the distal portion of the saphenous artery with 7-0 silk sutures ^13^. All arterial branches between the ligations were obliterated. The femoral artery was obliterated at the bifurcation of the epigastric artery, and the entire femoral artery was removed above the popliteal artery (Supplementary Fig. 6a). After induction of ischemia, UCA-PSCs (1×10^6^ cells/50 µL of PBS), WJ-MSCs (1×10^6^ cells/50 µL of PBS), or PBS (50 µL of PBS) were injected into the ischemic muscle with a 30-gauge needle at five different sites (n=6 mice in each group). Four weeks after treatment, all the mice were sacrificed, and ischemic hind limbs were collected.

Chemical-induced ischemic ovary failure in mice

Six-week-old C57BL/6 female mice were administered CTX (40 mg/kg) dissolved in 0.9% normal saline daily for 2 weeks ^14^. Mice in the control group were given an equal volume of normal saline. After an ischemic ovary failure mouse model was established, UCA-PSCs, WJ-MSCs, or PBS was transplanted into the ovaries of the CTX-treated mice following a modified approach of a previously reported method (n=6 mice in each group) (Supplementary Fig. 7a) ^15^. In summary, a suspension of 2×10^5^ cells in 10 μL of sterilized PBS was injected through 0.33-mm (29G) needles of BD Ultra-Fine^TM^ 1.0-ml disposable insulin syringes (BD, Franklin Lakes, NJ, USA) into each ovary of mice anesthetized with isoflurane. Four weeks after cell transplantation, all the mice were sacrificed, and ovaries were collected.

Laser Doppler perfusion imaging (LDPI)

Blood flow in the hind legs of the anesthetized animals was measured under standardized conditions with a laser Doppler imager (Moor Instruments, UK). The mice (n=6 mice in each group) were maintained on a heating pad (37°C) to minimize variation between flow measurements. To quantify blood flow from the knee joint to the toe, digital color-coded images obtained by LDPI were assessed, and the mean perfusion volume was evaluated. In the digital color-coded images, red indicates areas with maximum perfusion, yellow indicates medium perfusion, and blue represents areas with the lowest perfusion. To prevent data variation due to ambient conditions, hind limb perfusion was measured as the ratio of perfusion in ischemic to that in nonischemic areas.

Estrous cycle analysis

To detect estrous cycles, vaginal smears were obtained from mice by inserting a cotton-tipped swab wetted with saline into the vagina. Gently rotate the swab against the vaginal wall. Remove and wipe it onto a clean glass microscope slide. The stage of the estrous cycle was determined based on the relative quantities of leukocytes, nucleated epithelial cells, and cornified epithelial cells by examining the slides under the microscope. Vaginal smears showed mostly leukocytes and nucleated epithelial cells during diestrus. Mostly nucleated epithelial and some cornified epithelial cells appeared during proestrus. Increased number of cornified epithelial cells was presented in the stage of estrus. A large number of cornified epithelial cells and reappearing leukocytes were indicative of metestrus. Vaginal smears from the mice were analyzed for 15 consecutive days to follow the progression through the cycle.

Mice hormone detection

Four weeks after transplantation, blood was collected from the mice during diestrus. The serum was separated using centrifugation and kept at − 80°C. The concentrations of serum estradiol (E_2_) were measured with ELISA kit (YANYU, Shanghai, China).

Ovarian morphology and follicle counting

The number of follicles in each ovary was estimated according to a modified published protocol ^16^. Ovaries (n=6 mice in each group) were fixed with 10% (v/v) buffered formaldehyde at 4 ℃ overnight, embedded in paraffin, serially sectioned (to a 5-µm thickness), and stained with hematoxylin-eosin. The number of primordial, primary, secondary, and antral follicles in every randomly selected field of the tenth section was recorded. The different categories of follicles were determined as described previously. Only follicles containing an oocyte with a clearly visible nucleus were counted.

Ovarian superstimulation

Mice in each group were superovulated by i.p. injection of 10 IU of pregnant mare serum gonadotropin (PMSG) (Ningbo Sansheng Pharmaceutical Co., Ltd., Ningbo, China) followed by i.p. injection 48 h later with 10 IU of Human Chorionic Gonadotropin (hCG) (Ningbo Sansheng Pharmaceutical Co., Ltd.). Unfertilized oocytes were released from the swollen oviducts at 13-15 h post hCG injection, and cumulus cells were dissolved away from the oocytes with M2 medium (Sigma Aldrich) containing 0.3 mg/ml of hyaluronidase at 37°C for less than 5 min. The remaining cumulus cells were removed by repeated aspiration with a narrow-bore pipette. Cumulus-free oocytes were washed three times in M2 medium and incubated in M16 medium at 37°C in 5% CO_2_ in compressed air and high humidity for viable observation.

Statistical analysis

All experiments were performed at least three times. The data are presented as the mean ± standard error of the mean (SEM). Two-tailed Student's *t*-test was performed to evaluate the differences between two groups, and nonparametric one-way ANOVA followed by Dunn’s multiple comparison test was performed for comparisons between more than two groups. Statistical analyses were performed using Statistics Package for Social Science (SPSS 25.0, SPSS, Chicago, IL, USA). A value of *P* < 0.05 was considered to be statistically significant.

REFERENCES

1. Li, H. et al. Immunohistochemistry Microarrays. Anal Chem 89, 8620–8625 (2017).

2. Fang, S. et al. Umbilical Cord-Derived Mesenchymal Stem Cell-Derived Exosomal MicroRNAs Suppress Myofibroblast Differentiation by Inhibiting the Transforming Growth Factor-β/SMAD2 Pathway During Wound Healing. Stem Cells Transl Med 5, 1425–1439 (2016).

3. Xu, L. et al. Different Angiogenic Potentials of Mesenchymal Stem Cells Derived from Umbilical Artery, Umbilical Vein, and Wharton’s Jelly. Stem Cells International 2017, 1–15 (2017).

4. D, C. et al. Matrigel plug assay: evaluation of the angiogenic response by reverse transcription-quantitative PCR. Angiogenesis 16, (2013).

5. Kim, D. et al. TopHat2: accurate alignment of transcriptomes in the presence of insertions, deletions and gene fusions. Genome Biol 14, R36 (2013).

6. Wiśniewski, J. R., Zougman, A., Nagaraj, N. & Mann, M. Universal sample preparation method for proteome analysis. Nat Methods 6, 359–362 (2009).

7. Tyanova, S. et al. The Perseus computational platform for comprehensive analysis of (prote)omics data. Nat Methods 13, 731–740 (2016).

8. M, K. et al. KEGG for linking genomes to life and the environment. Nucleic acids research 36, (2008).

9. Szklarczyk, D. et al. The STRING database in 2017: quality-controlled protein-protein association networks, made broadly accessible. Nucleic Acids Res 45, D362–D368 (2017).

10. Fan, C.-D., Lum, M. A., Xu, C., Black, J. D. & Wang, X. Ubiquitin-dependent regulation of phospho-AKT dynamics by the ubiquitin E3 ligase, NEDD4-1, in the insulin-like growth factor-1 response. J Biol Chem 288, 1674–1684 (2013).

11. Hu, X. et al. MiR-211/STAT5A Signaling Modulates Migration of Mesenchymal Stem Cells to Improve its Therapeutic Efficacy. Stem Cells 34, 1846–1858 (2016).

12. Hu, L. et al. IGF1 Promotes Adipogenesis by a Lineage Bias of Endogenous Adipose Stem/Progenitor Cells. Stem Cells 33, 2483–2495 (2015).

13. Padgett, M. E., McCord, T. J., McClung, J. M. & Kontos, C. D. Methods for Acute and Subacute Murine Hindlimb Ischemia. J Vis Exp (2016) doi:10.3791/54166.

14. Li, J. et al. Human chorionic plate-derived mesenchymal stem cells transplantation restores ovarian function in a chemotherapy-induced mouse model of premature ovarian failure. Stem Cell Res Ther 9, 81 (2018).

15. Su, J. et al. Transplantation of adipose-derived stem cells combined with collagen scaffolds restores ovarian function in a rat model of premature ovarian insufficiency. Hum Reprod 31, 1075–1086 (2016).

16. Myers, M., Britt, K. L., Wreford, N. G. M., Ebling, F. J. P. & Kerr, J. B. Methods for quantifying follicular numbers within the mouse ovary. Reproduction 127, 569–580 (2004).


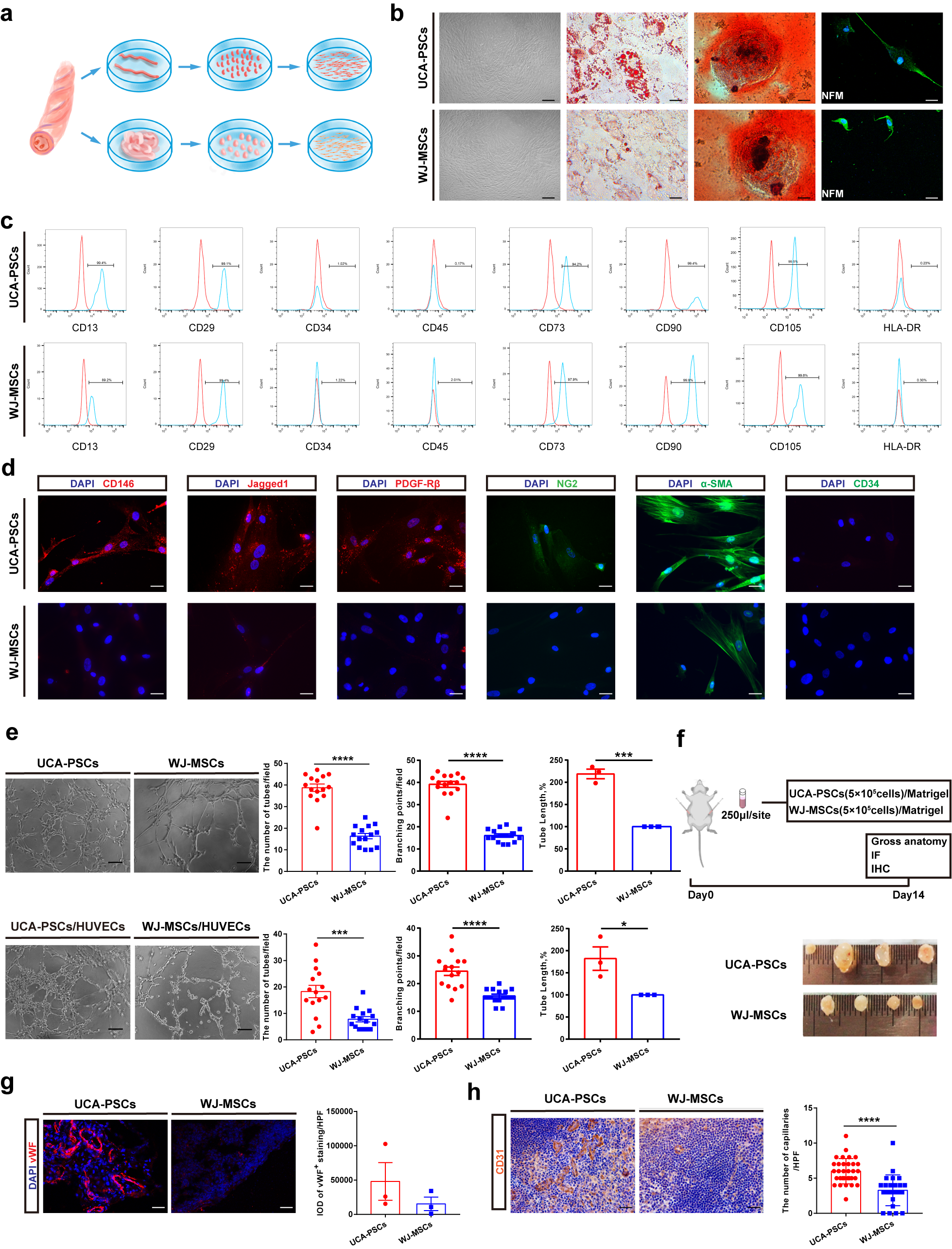


**Supplementary Fig. 1** Angiogenic capacity of UCA-PSCs *in vitro* and *in vivo.* **a** Flowchart of the isolation of UCA-PSCs and WJ-MSCs from the human umbilical cord. **b** Morphology of cultured UCA-PSCs and WJ-MSCs. The cells at third passage (P3) showed fibroblastic morphology. Scale bars, 500 μm. UCA-PSCs and WJ-MSCs were induced to differentiate into adipogenic cells, osteogenic cells, and neuron-like cells. NFM, neurofilament medium polypeptide. Scale bars, 50 μm. **c** Flow cytometry analysis of UCA-PSCs and WJ-MSCs. Cells were negative for hematopoietic cell markers such as CD34, CD45, and HLA-DR, while positive for CD13, CD29, CD73, CD90 and CD105. **d** Fluorescence imaging revealed a high incidence of CD146^+^, Jagged1^+^, PDGF-Rβ^+^, NG2^+^, α-SMA^+^, staining in UCA-PSCs, followed by WJ-MSCs. While both cells were negative for CD34. Scale bars, 10 µm. n=3 in each group. **e** Cells were treated with basic medium and plated on Matrigel in 96-well tissue culture plates. Tube formation was microscopically compared after 3 h. (magnification: 100×). The number of tubes per field, branching points, and the total length of tubes were quantified 3 h after treatment by counting 3-5 random fields/well under the microscope. The data are shown as the mean ± SEM. n=3 per group. **P* < 0.05, ****P* < 0.001, *****P* < 0.0001 (t-test). **f** Flowchart of Matrigel plug assay. Gross anatomy of freshly removed Matrigel plugs harvested 2 weeks after subcutaneous injection into nude mice. **g** Sections of cryopreserved Matrigel plugs were stained for the endothelial marker, vWF (red), and cell nuclei visualized with DAPI (blue). Scale bars, 100 µm. The integrated optical density (IOD) values of positive staining in 3-5 randomly selected high-power field (HPF) of view were counted. The data are shown as the mean ± SEM. n=3 per group. **P* < 0.05 (t-test). **h** The neovasculature formed after transplantation of UCA-PSCs or WJ-MSCs in Matrigel plugs was quantified by immunohistochemistry staining with anti-mouse CD31 IgG. Density of CD31-positive blood vessels was shown as the number of capillaries per HPF from 3-5 random sections of each group. The data are shown as the mean ± SEM. n=3 per group, *****P* <0.0001 (t-test).


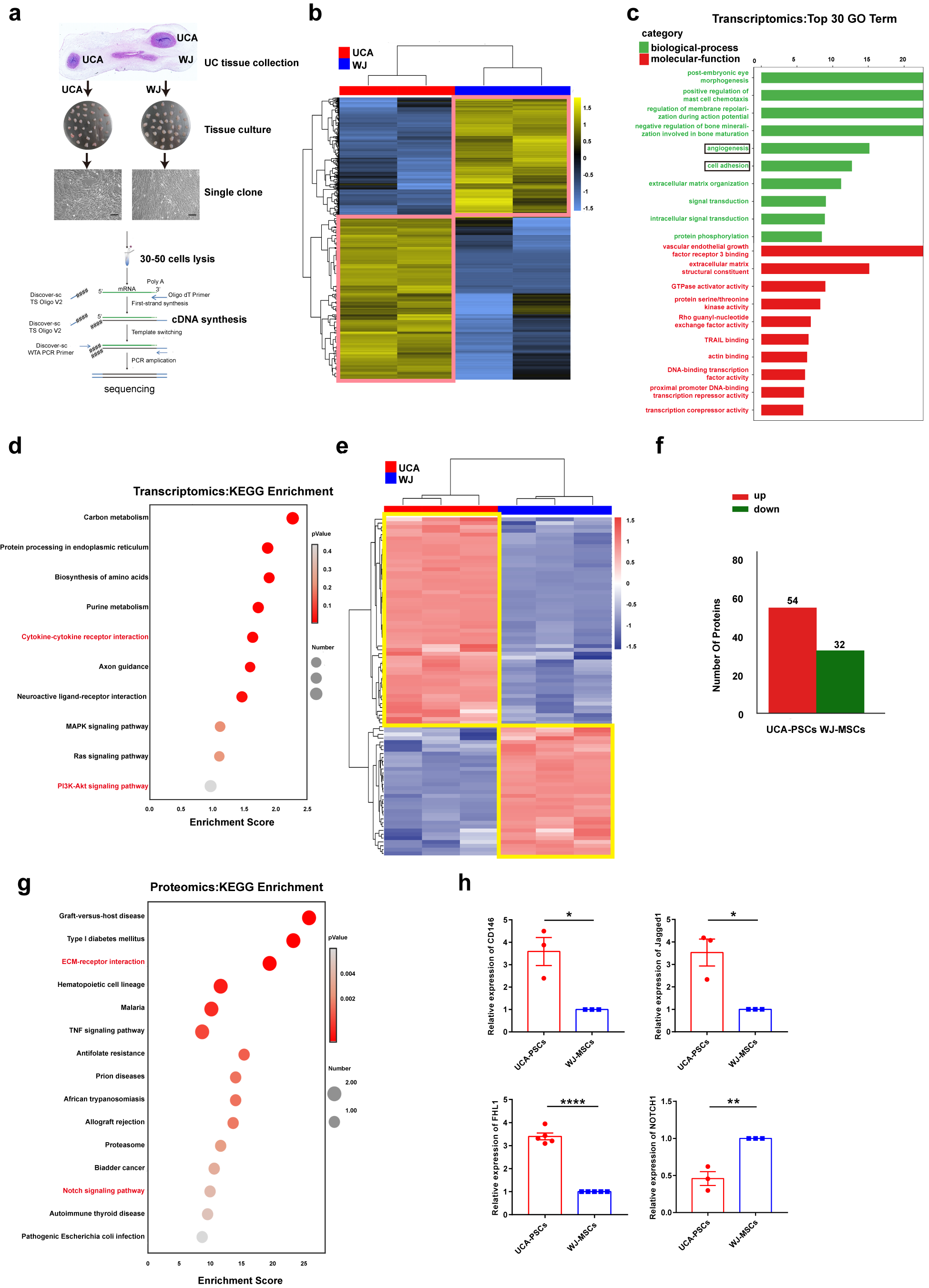


**Supplementary Fig. 2** Angiogenic genes expression in UCA-PSCs. **a** Flowchart of single cell colony RNA sequencing. **b** Differentially expressed genes (DEGs) among UCA-PSCs and WJ-MSCs. RNA-Seq data was assessed as log2 (Fold Change, FC) with corresponding FDR by DESeq software using log2(FC)> 0.58 or <-0.58 and FDR < 0.05 as the statistical cutoffs. Among these DEGs, 3363 genes were differentially expressed between UCA-PSCs and WJ-MSCs. **c** Gene Ontology analysis showed that “Biological Process” category contains several interesting GO processes such as “Cell adhesion” and “Angiogenesis”. **d** KEGG pathway enrichment of RNAs was performed to explore the possible signaling pathways modulated by UCA-PSCs. **e** Comparison of protein expression patterns in UCA-PSCs and WJ-MSCs. Red indicates upregulated proteins while blue indicates downregulated proteins. **f** 54 proteins were up-regulated in UCA-PSCs compared with WJ-MSCs. **g** KEGG pathway enrichment of proteins was performed to explore the possible signaling pathways modulated by UCA-PSCs. **h** Transcript levels of CD146, Jagged1, Notch1, and FHL1 in UCA-PSCs and WJ-MSCs were measured by qRT-PCR. Results were normalized to 18s rRNA expression (mean ± SEM. n=3). **P* < 0.05, ***P* < 0.01, and *****P* < 0.0001 (t-test).


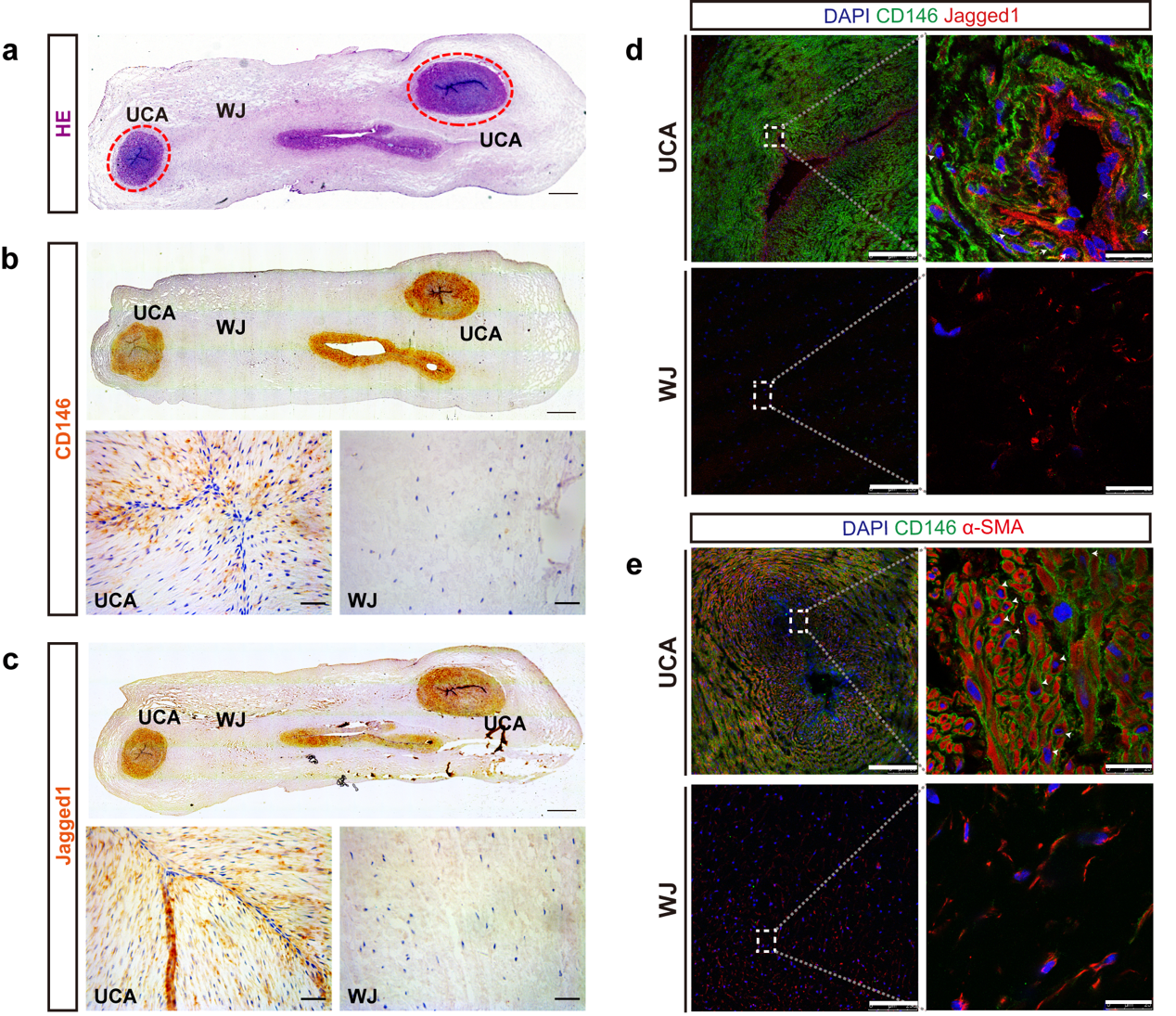


Supplementary Fig. 3 Expression of CD146, Jagged1 and α-SMA in human UC. a H&E staining of human umbilical cord. The two UCAs and the single vein are surrounded by WJ. UCA, umbilical cord arteries; WJ, wharton’s jelly. Scale bar, 1 mm. b Immunohistochemical staining revealed that expression of CD146 is low in WJ, as compared with UCAs. Scale bar, 1 mm. Scale bars, 50 μm. c Expression of Jagged1 is also lower in WJ, as compared with UCAs. Scale bar, 1 mm. Scale bars, 50 μm. d Representative images of co-staining for Jagged1 and CD146 in UCA and WJ. Scale bars, 100 µm. White arrowheads mark Jagged1^+^CD146^+^ perivascular cells. Scale bars, 25 µm. e Representative images of co-staining for α-SMA and CD146 in UCA and WJ. Scale bars, 100µm. White arrowheads mark α-SMA^+^CD146^+^ perivascular cells. Scale bars, 25 µm. All experiments were performed three times.


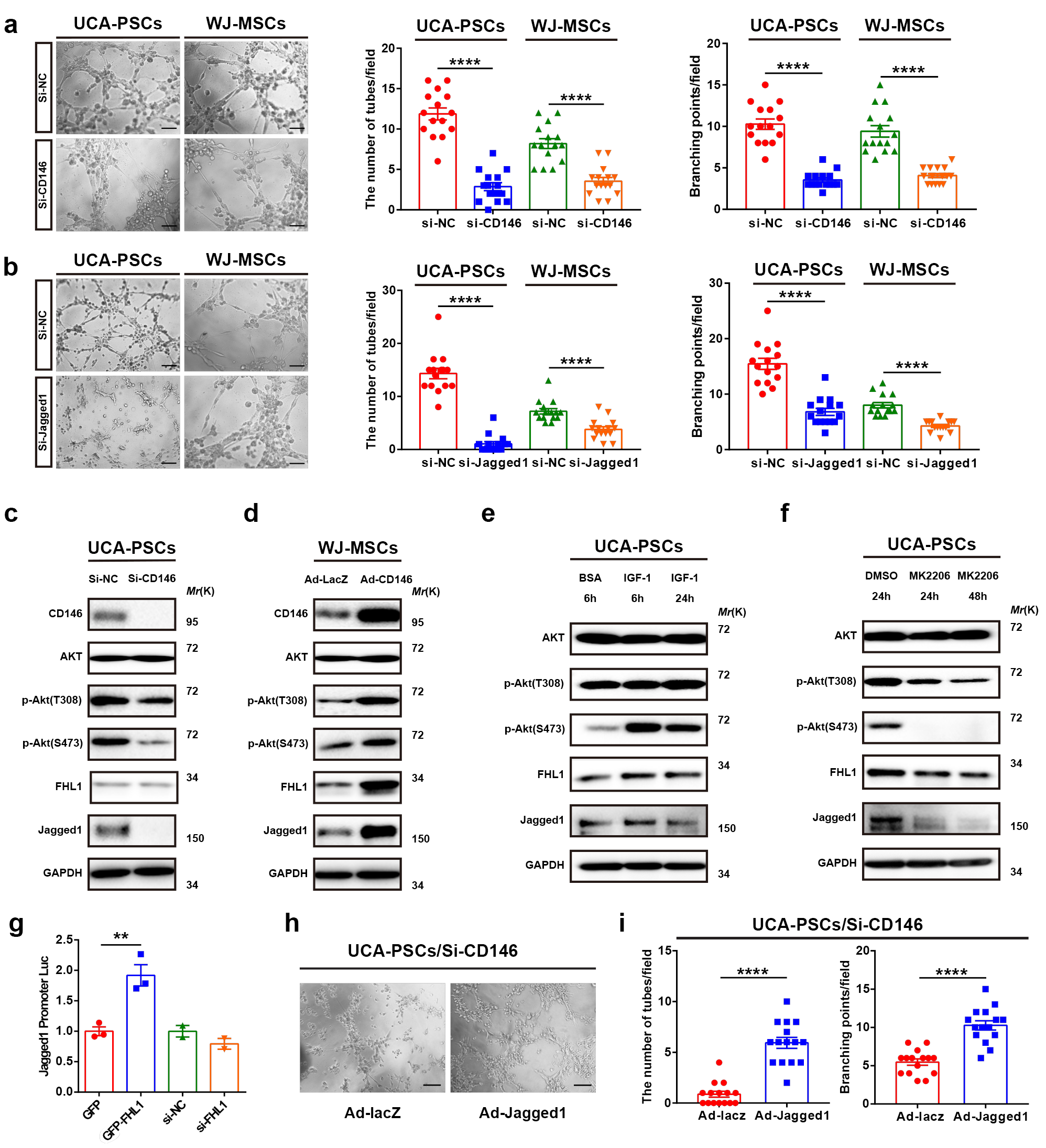


**Supplementary Fig. 4** CD146/AKT/FHL1/Jagged1 in angiogenesis from UCA-PSCs. **a-b** Knockdown of CD146 (a) and Jagged1 (b) decreased angiogenesis in UCA-PSCs and WJ-MSCs. Tube formation assays were performed 72 h after transfection of Si-NC or Si-CD146, Si-NC or Si-Jagged1, respectively. Scale bars, 50 µm. The number of tubes per field and branching points in UCA-PSCs and WJ-MSCs was quantified 3 h after treatment by counting 3-5 random fields/well under the microscope (magnification: 100×). The data are shown as the mean ± SEM, n=3 in each group. *****P* < 0.0001 (t-test). **c-d** UCA-PSCs were transfected with si-NC or si-CD146, while WJ-MSCs were infected with Ad-LacZ or Ad-CD146 for 48 h. Protein levels of CD146, AKT, p-Akt (T308), p-Akt (S473), FHL1, and Jagged1 were measured by Western blot. GAPDH was used as an internal loading control. **e-f** UCA-PSCs were treated with an AKT agonist, IGF-1, for 0 h, 6 h, 24 h, or inhibitor MK2206, for 0 h, 24 h, 48 h. The AKT, p-Akt (T308), p-Akt (S473), FHL1 and Jagged1 expressions were determined by western blot (n=3). **g** UCA-PSCs were infected with the FHL1-expressing plasmids or transfected with si-RNAs for 24 h and then transfected with Jagged1-Luc. After 48 h, luciferase assays were performed, and data were plotted after normalization to Renilla luciferase activity. The data are shown as mean ± SEM, n=3 per group. ***P* < 0.01 (t-test). **h-i** Jagged1 reversed CD146 knockdown-induced decreased angiogenesis in UCA-PSCs. Tube formation assay was performed in CD146-knockdown UCA-PSCs with Ad-lacZ or Ad-Jagged1. The number of tubes and branching points per field was quantified 3 h after treatment by counting 3-5 random fields/well under the microscope (magnification: 100×). The data are shown as the mean ± SEM. n=3 in each group. *****P* < 0.0001 (t-test).


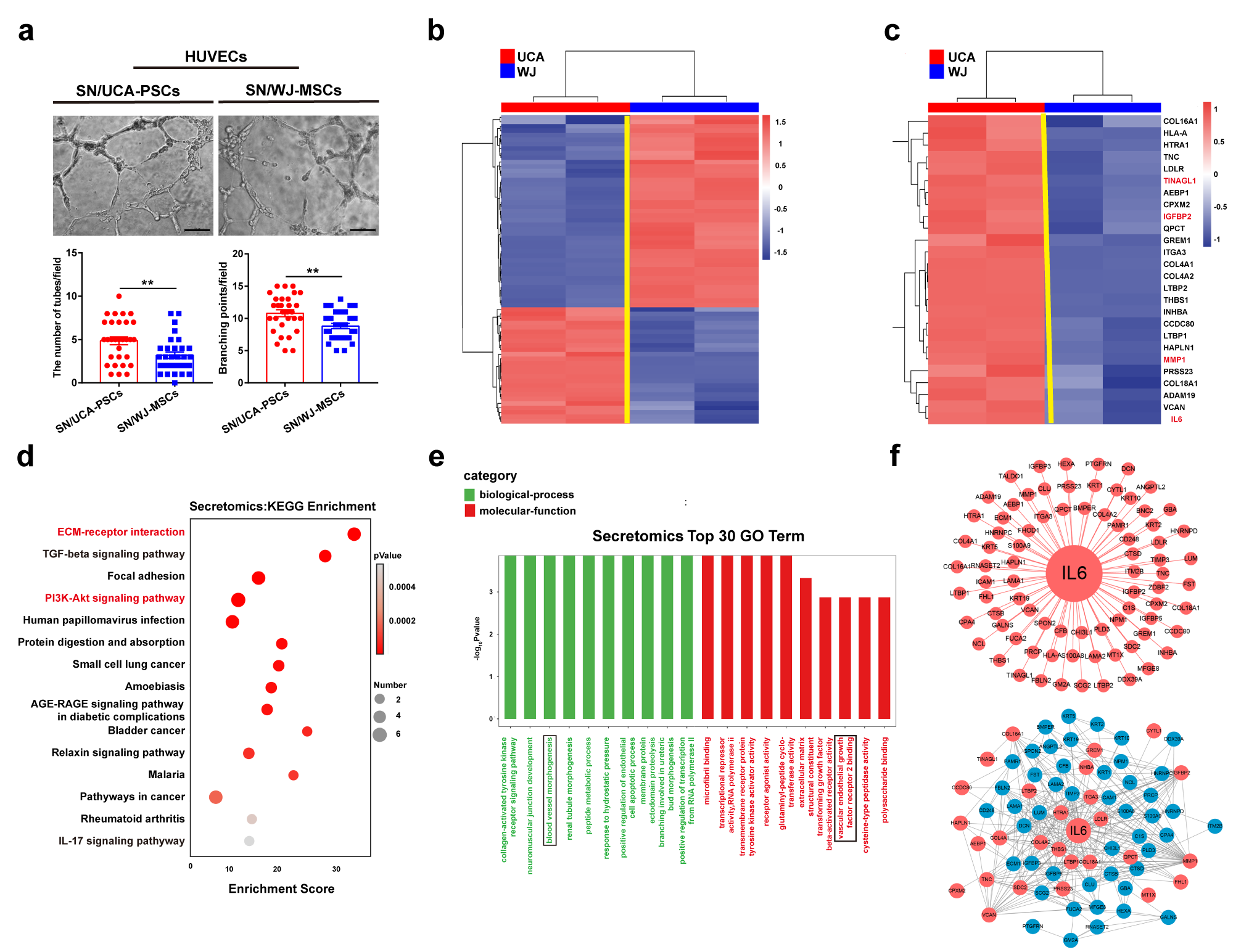


**Supplementary Fig. 5** Secretory proteins for angiogenesis from UCA-PSCs. **a** The number of tubes and branching points per field were quantified 3 h after treatment by counting 3-5 random fields/well under the microscope (magnification: 200×). SN, supernatant. The data are shown as the mean ± SEM. n=3 in each group. ***P* < 0.01(t-test). **b** Heatmaps showed fold change in the expression of secretory proteins from UCA-PSCs and WJ-MSCs. Red indicates upregulated proteins while blue indicates downregulated proteins (n=2 in each group). **c** Heat map and hierarchical clustering indicated that 26 secretory proteins were up-regulated in UCA-PSCs supernatants. **d** KEGG pathway enrichment was performed to explore the possible signaling pathways modulated by secretory proteins in the cell supernatant of UCA-PSCs. **e** Gene Ontology analysis annotated that secretory proteins in UCA-PSCs are involved with blood vessel morphogenesis and VEGF receptor 2 binding. **f** Protein-protein interactions (PPI) analyses revealed functional relationships within IL6 and other differently expressed secretory proteins from UCA-PSCs.


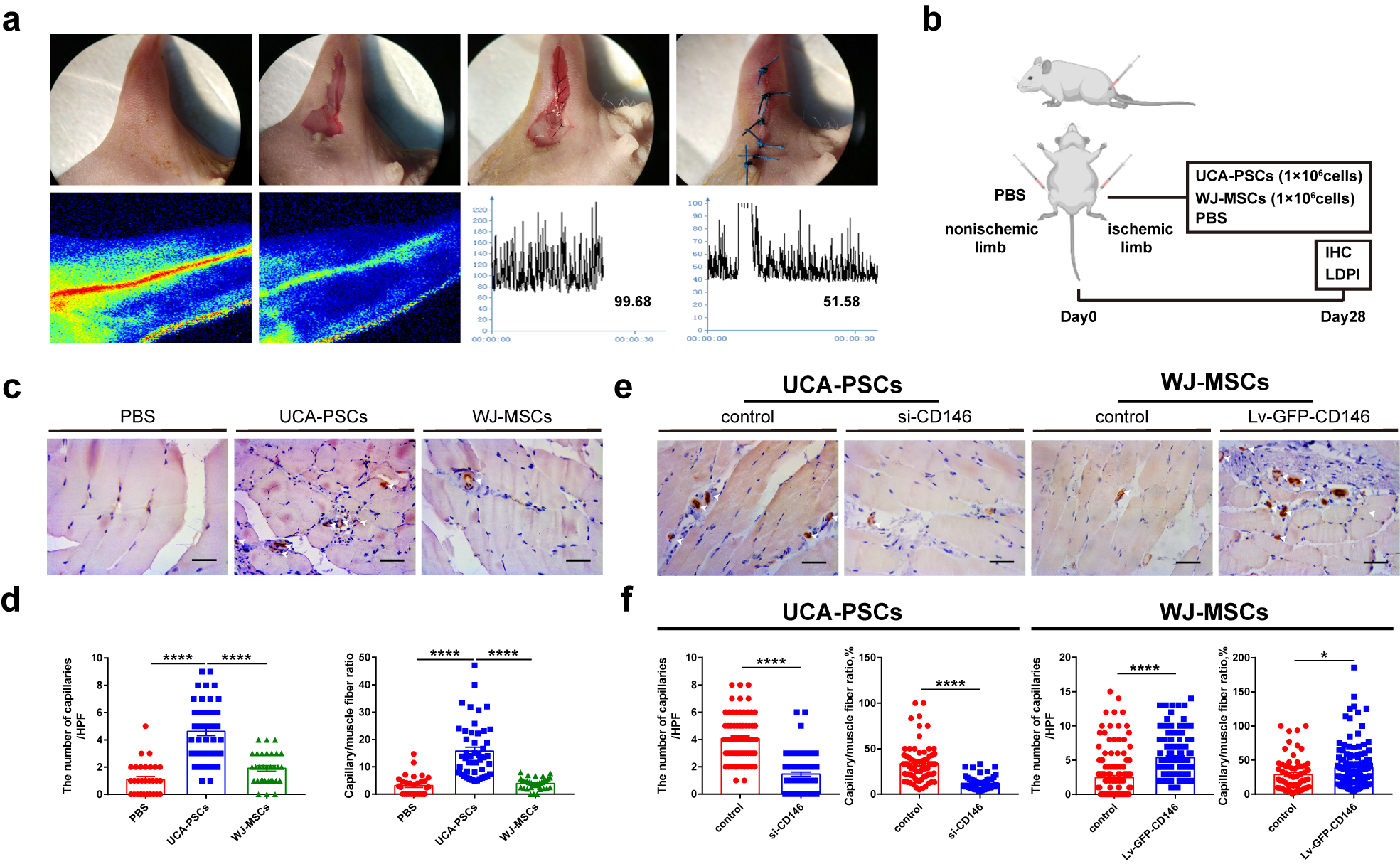


**Supplementary Fig. 6** UCA-PSCs transplantation into hindlimb ischemia (HLI) mice. **a** The steps for the establishment of HLI model. The blood perfusion ratio of mice limbs was measured by LDPI**.** And the decreased blood supply in ischemic limb compared with the other limb in the same mouse indicated the successful establishment of ischemia hindlimb. **b** Flowchart of cell transplantation into HLI mice. **c-d** Representative photographs of immunohistochemistry with anti-mouse CD31 antibody in the control, UCA-PSCs, and WJ-MSCs groups. Scale bars, 100 µm. Quantitative analysis of capillary density in ischemic hindlimb muscle among above three groups. Capillary density is shown as the number of capillaries and the capillary-to-muscle-fiber ratio. The data are shown as the mean ± SEM. n=6 per group. *****P* < 0.0001 (one-way ANOVA). **e**-**f** Representative photographs of immunohistochemistry with anti-mCD31 antibody in the UCA-PSCs and si-CD146 UCA-PSCs groups, WJ-MSCs and Lv-GFP-CD146 WJ-MSCs groups, respectively. Scale bars, 100 µm. Quantitative analysis of capillary density in ischemic hindlimb muscle in above four groups. Capillary density is shown as the number of capillaries and the capillary-to-muscle-fiber ratio. The data are shown as the mean ± SEM. n=6 per group. **P* < 0.05, *****P* < 0.0001 (t-test).


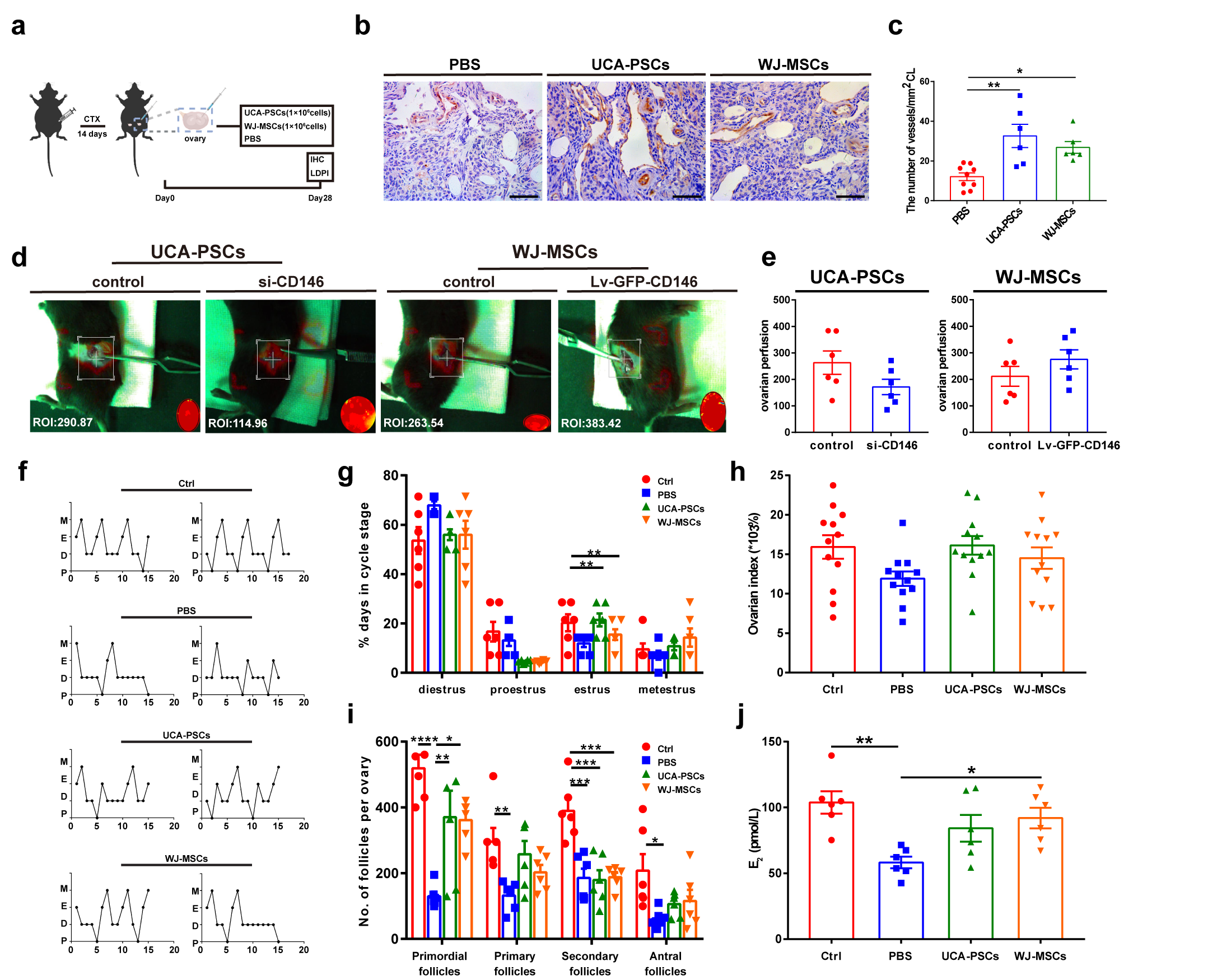


**Supplementary Fig. 7** UCA-PSCs transplantation into ischemic premature ovary failure (POF) mice. **a** Flowchart of cell transplantation into POF mice. **b** The expression of mouse CD31 in POF mice ovaries among PBS, UCA-PSCs and WJ-MSCs groups. Scale bars, 50 μm. **c** The number of vessels per 1mm^2^ in ovaries of UCA-PSCs group was significantly increased compared with PBS group. The data are shown as the mean ± SEM. n=6 per group. **P* < 0.05, ***P* < 0.01 (one-way ANOVA). **d-e** The blood perfusion flow in ovaries in different groups through LDPI. The data are shown as the mean ± SEM. n=6 per group. **f-g** State of the estrous cycles 4 weeks after transplantation in mice given different treatments. ***P* < 0.01 (one-way ANOVA). **h-j** The ovarian index (h), the number of follicles (i) and the level of serum estradiol (E_2_) (j) were measured 4 weeks after transplantation of PBS, UCA-PSCs and WJ-MSCs in POF mice. The mice without CTX treatment were used as normal control. The data are shown as the mean ± SEM. n=6 per group. **P* < 0.05, ***P* < 0.01, ****P* < 0.001, *****P* < 0.0001 (one-way ANOVA).

**Supplementary Table 1.** Genes differently expressed in UCA-PSCs and WJ-MSCs.

See Excel sheet.

**Supplementary Table 2.** Proteins differently expressed in UCA-PSCs and WJ-MSCs.

See Excel sheet.

**Supplementary Table 3.** Common genes and corresponding proteins differently expressed in UCA-PSCs and WJ-MSCs.

See Excel sheet.

**Supplementary Table 4.** Secreted proteins differently expressed in the supernatant harvest from UCA-PSCs and WJ-MSCs.

See Excel sheet.

Supplementary Table 5. Antibodies used for IHC and Immunofluorescence.

| Antibody | Source | Concentration |
| --- | --- | --- |
| CD146 | Abcam ab24577 | 1:1000 |
| Jagged1 | Abcam ab109536 | 1:300 |
| PDGF-Rβ | Abcam ab32570 | 1:100 |
| NG2 | Abcam ab139406 | 1:500 |
| α-SMA | Abcam ab8207 | 1:500 |
| CD34 | Abcam ab81289 | 1:200 |
| vWF | Abcam ab11713 | 1:200 |
| NFM | Santa Cruz Biotechnology sc-16143 | 1:100 |
| CD31 | Abcam ab28364 | 1:100 |
| Alexa Fluor® 488 conjugated goat anti-mouse IgG | Abcam ab150117 | 1:200 |
| Alexa Fluor® 488 conjugated goat anti-rabbit IgG | Abcam ab150077 | 1:200 |
| Alexa Fluor® 594 conjugated goat anti-rabbit IgG | Abcam ab150084 | 1:200 |
| Alexa Fluor® 555 conjugated goat anti-mouse IgG | Abcam ab150118 | 1:200 |
| Alexa Fluor® 555 conjugated goat anti-rabbit IgG | Abcam ab150082 | 1:200 |
| Goat anti-rabbit IgG | ZSGB-BIO PV6001 | 1:200 |

Supplementary Table 6. Antibodies used for Flow cytometry analysis and Multilineage differentiation.

| Antibody | Source | Concentration |
| --- | --- | --- |
| anti-CD13-FITC | ebioscience 12-0138-42 | 1:100 |
| anti-CD29-PE | ebioscience 12-0299-42 | 1:100 |
| anti-CD34-PE | BD 555822 | 1:100 |
| anti-CD45-FITC | BD 560976 | 1:100 |
| anti-CD73-PE | BD 561014 | 1:100 |
| anti-CD90-APC | BD 559869 | 1:100 |
| anti-CD105-PE | BD 560839 | 1:100 |
| anti-CD146-FITC | BD 560846 | 1:100 |
| anti-HLA-DR-PE | Beckman Coulter IM0464U | 1:100 |

Supplementary Table 7. Quantitative RT-PCR primers.

| Species | Genes | Primer Sequences 5’-3’ | Length |
| --- | --- | --- | --- |
| Human | *CD146* | Forward: accacatgaaggagtccagg  Reverse: ggttgtctcttcctctgcct | 20  20 |
| Human | *Jagged1* | Forward: cctgaaggggtgcggtatat  Reverse: ggagttgacaccatcgatgc | 20  20 |
| Human | *Notch1* | Forward: atgcagaacaacagggagga  Reverse: accaggttgtactcgtccag | 20  20 |
| Human | *FHL1* | Forward: aacgtggagtacaaggggac  Reverse: atggcaagtcacgcagtagaa | 20  21 |
| Human | *18S RNA* | Forward: cggctaccacatccaaggaa  Reverse: ctggaattaccgcggct | 20  17 |

Supplementary Table 8. Antibodies used for Western blot.

| Antibody | Source | Concentration |
| --- | --- | --- |
| CD146 | Abcam ab75769 | 1:500 |
| FHL1 | Proteintech 10991-1-AP | 1:1000 |
| Jagged1 | Abcam ab109536 | 1:500 |
| DLL4 | Abcam ab7280 | 1:500 |
| Notch1 | Abways CY5244 | 1:500 |
| PI3K-110α | Abways CY5224 | 1:500 |
| PI3K-85β | Bioworld BS3006 | 1:500 |
| PTEN | Abways AY0570 | 1:1000 |
| AKT | Bioworld BS3006 | 1:1000 |
| p-AKT(T308) | Bioworld BS4009 | 1:1000 |
| p-AKT(S473) | Bioworld BS4006 | 1:1000 |
| IL6 | Proteintech 66146-1-lg | 1:1000 |
| GAPDH | Bioworld AP0063 | 1:10000 |
| Goat anti-mouse IgG | Bioworld BS12471 | 1:10000 |
| Goat anti-rabbit IgG | Bioworld BS13271 | 1:10000 |

**Supplementary Data file 1.** RNA-seq data.

See SRA (NCBI) under the accession number (PRJNA613338) or the following link after the indicated release date: https://www.ncbi.nlm.nih.gov/sra/PRJNA613338 .

**Supplementary Data file 2.** Proteome and secretory proteomic data.

See iProX under the accession number (IPX0002083000, IPX0002090000) or the following link after the indicated release date: <https://www.iprox.org/page/PCV010.html?submitPage=1.>

**Supplementary Video1** Bipedal stepping as well as climbing abilities were tested at 4 weeks after UCA-PSCs transplantation.

**Supplementary Video2** Bipedal stepping as well as climbing abilities were tested at 4 weeks after WJ-MSCs transplantation.
