# Supplementary material for: Umbilical cord artery-derived perivascular stem cells for treatment of ovarian failure through CD146 signaling
Source: Signal Transduct Target Ther. 2022 Jul 13;7:223. doi: 10.1038/s41392-022-01029-4 (PMC9276707; doi:10.1038/s41392-022-01029-4)
Supplement: Supplementary file 8 — Supplementary Table 1 [file 41392_2022_1029_MOESM8_ESM.pdf]

gene_id	baseMean	baseMean_c	baseMean_c	foldChange	log2FoldCh	pval	padj
ENSG000001	173.54851	117.27526	229.82177	1.9596782	0.9706167	0.0181386	0.2096746
ENSG000002	1223.2619	407.91155	2038.6123	4.9976821	2.3212591	2.00E-09	2.16E-07
ENSG000000	2190.2708	3465.0542	915.48751	0.2642058	-1.920266	3.50E-05	0.0014909
ENSG000000	170.31845	257.41165	83.225256	0.3233158	-1.628984	0.0099106	0.1366755
ENSG000001	244.36582	142.83108	345.90057	2.4217458	1.2760475	0.0064484	0.1005766
ENSG000000	91.189989	179.29505	3.0849255	0.0172059	-5.860956	3.35E-06	0.0001906
ENSG000001	874.38048	297.98924	1450.7717	4.8685373	2.2834884	2.02E-07	1.52E-05
ENSG000001	422.74565	319.65669	525.83461	1.644998	0.7180858	0.0207452	0.2290431
ENSG000001	782.09917	425.99425	1138.2041	2.6718767	1.4178534	0.0001597	0.0053698
ENSG000001	27.680447	49.251621	6.1092733	0.1240421	-3.011099	0.0379331	0.3371123
ENSG000001	223.87795	129.22101	318.53488	2.4650394	1.3016107	0.0192096	0.2170483
ENSG000001	1147.7243	1488.1589	807.2898	0.5424756	-0.88237	0.027748	0.2759602
ENSG000001	32.520059	4.7381535	60.301965	12.726891	3.6698082	0.0321714	0.3042956
ENSG000002	485.21186	281.14992	689.2738	2.4516237	1.2937376	0.0007059	0.0182461
ENSG000002	34.455689	3.7905228	65.120855	17.179914	4.1026509	0.016377	0.1959234
ENSG000000	1031.1777	1485.3714	576.98406	0.3884443	-1.36422	0.000269	0.0082255
ENSG000002	68.209893	1.8952614	134.52452	70.979404	6.1493285	2.65E-06	0.000155
ENSG000002	40.440715	1.2635076	79.617923	63.01341	5.977587	0.0003419	0.0100795
ENSG000001	5939.8729	1982.1702	9897.5755	4.9933025	2.3199943	8.67E-08	7.05E-06
ENSG000001	590.15403	412.04816	768.25989	1.8644905	0.8987815	0.0249707	0.259088
ENSG000002	8034.4374	9738.5593	6330.3155	0.6500259	-0.621431	0.0332789	0.3113967
ENSG000001	253.46419	332.5076	174.42077	0.5245618	-0.930815	0.0471615	0.3860371
ENSG000001	110.06835	40.422173	179.71452	4.4459391	2.1524882	0.0147177	0.1813642
ENSG000001	118.65589	194.97091	42.340868	0.2171651	-2.203136	0.002046	0.0422005
ENSG000002	146.83096	67.33935	226.32256	3.3609259	1.7488587	0.0174328	0.2053759
ENSG000001	588.46917	197.35257	979.58577	4.9636332	2.3113965	2.37E-06	0.0001411
ENSG000001	28.296167	4.9712849	51.62105	10.383845	3.3762688	0.0035907	0.0651698
ENSG000001	563.8183	733.70081	393.93579	0.5369161	-0.897231	0.0194867	0.2188778
ENSG000001	913.46511	322.73053	1504.1997	4.6608535	2.2205942	4.76E-06	0.0002609
ENSG000002	12.153708	24.307416	0	0	#NAME?	0.0268625	0.27114
ENSG000001	105.30813	163.47603	47.140222	0.2883617	-1.794049	0.0187657	0.2142175
ENSG000001	3216.9199	2473.6144	3960.2254	1.6009873	0.6789619	0.009717	0.1349855
ENSG000002	22.526583	0.3158769	44.737289	141.62887	7.1459716	0.0033578	0.0619404
ENSG000001	69.376772	34.886775	103.86677	2.9772534	1.573982	0.0344061	0.3184211
ENSG000001	53.066689	11.371568	94.76181	8.3332225	3.0588745	0.0282828	0.2801371
ENSG000001	253.92693	163.68399	344.16986	2.1026483	1.0722076	0.0068761	0.1053641
ENSG000001	986.79206	625.60831	1347.9758	2.1546642	1.107463	0.0126335	0.1621792
ENSG000001	145.7713	37.574246	253.96835	6.7591071	2.7568327	6.68E-05	0.0026204
ENSG000001	525.94796	766.25627	285.63965	0.372773	-1.423631	0.0047444	0.079992
ENSG000001	1401.1532	858.61754	1943.689	2.2637424	1.1787098	0.0163955	0.1960209
ENSG000001	199.838	102.15848	297.51752	2.9123134	1.5421656	0.0331674	0.3112144
ENSG000001	52.698104	0	105.39621	Inf	Inf	2.06E-07	1.54E-05
ENSG000002	109.52276	31.660365	187.38515	5.9186034	2.5652568	0.004635	0.0788353
ENSG000001	939.25019	666.48667	1212.0137	1.8185115	0.8627581	0.04073	0.3499034
ENSG000001	738.98983	958.4771	519.50256	0.5420083	-0.883613	0.0114616	0.1517427
ENSG000001	196.58774	103.57741	289.59807	2.7959577	1.4833425	0.0352962	0.3231224
ENSG000002	38.511744	0	77.023488	Inf	Inf	1.01E-05	0.000517
ENSG000001	60.359842	107.29023	13.429459	0.1251695	-2.998046	0.0004215	0.011931
ENSG000001	65.464785	0	130.92957	Inf	Inf	8.07E-09	7.97E-07
ENSG000001	1512.3178	1882.5186	1142.1169	0.6066962	-0.720954	0.0168648	0.2001656

ENSG000001	177.44783	308.80677	46.088894	0.1492483	-2.744213	2.12E-06	0.0001302
ENSG000001	4593.4296	8.5286763	9178.3306	1076.1729	10.071694	6.74E-58	3.65E-54
ENSG000001	521.33567	684.40891	358.26242	0.5234625	-0.933842	0.0293052	0.2869128
ENSG00000(	1299.1857	978.65446	1619.717	1.6550448	0.7268703	0.0452594	0.3769782
ENSG000001	321.1217	6.9492918	635.29411	91.41854	6.5144149	1.08E-17	3.78E-15
ENSG000001	146.55414	79.565733	213.54254	2.6838505	1.4243043	0.0409643	0.3512217
ENSG00000(	337.30166	468.82326	205.78006	0.4389289	-1.187941	0.0111376	0.1483187
ENSG000001	953.1672	234.3951	1671.9393	7.132996	2.8345082	1.22E-11	1.91E-09
ENSG000001	473.99735	943.87409	4.120617	0.0043656	-7.83959	4.99E-19	1.93E-16
ENSG000001	968.61699	1353.1297	584.10426	0.4316691	-1.212002	0.0034683	0.0634592
ENSG000001	453.8159	117.85951	789.77229	6.7009634	2.7443685	1.90E-07	1.43E-05
ENSG00000(	155.40901	205.02643	105.79159	0.51599	-0.954585	0.0430461	0.3632278
ENSG000001	544.28172	298.23748	790.32597	2.6499888	1.4059862	0.0030939	0.058141
ENSG000001	456.54756	4.4222766	908.67285	205.47626	7.6828279	2.02E-15	5.07E-13
ENSG000001	1109.0176	1504.1959	713.8392	0.4745653	-1.075321	0.0045698	0.0779223
ENSG00000(	167.40009	90.455934	244.34424	2.7012516	1.433628	0.0078053	0.1152314
ENSG000001	2104.5364	2912.6984	1296.3743	0.4450767	-1.167874	0.0033725	0.062138
ENSG000002	18.46005	0	36.9201	Inf	Inf	0.0043326	0.0751087
ENSG000002	189.32327	120.75494	257.8916	2.1356608	1.0946825	0.0061046	0.0966064
ENSG000001	681.86201	957.80222	405.9218	0.4238054	-1.238526	0.0100832	0.1382973
ENSG000001	177.35582	81.621451	273.09019	3.345814	1.7423573	0.0048066	0.0808201
ENSG000002	3161.5822	1848.1823	4474.982	2.4212881	1.2757748	0.0002766	0.0084286
ENSG000001	1054.0787	806.55257	1301.6049	1.613788	0.6904511	0.0418282	0.3563954
ENSG000001	28.498596	2.5270152	54.470176	21.555144	4.4299603	0.018113	0.2096746
ENSG000001	179.0009	325.83676	32.165044	0.0987152	-3.340584	0.0083535	0.1208852
ENSG000001	108.55685	199.44572	17.667976	0.0885854	-3.496787	0.0400001	0.3464927
ENSG000001	178.96307	93.785229	264.14092	2.8164448	1.4938752	0.0271323	0.2730012
ENSG00000(	10.180736	20.361472	0	0	#NAME?	0.0298582	0.2907493
ENSG000001	11.32616	0	22.652319	Inf	Inf	0.0370972	0.3326329
ENSG000001	10.615696	21.231393	0	0	#NAME?	0.0164069	0.1960209
ENSG000001	501.98068	702.10305	301.8583	0.4299345	-1.217811	0.0030161	0.0569969
ENSG000001	1175.8275	796.18402	1555.471	1.9536576	0.9661776	0.0207393	0.2290431
ENSG000001	53.523512	107.04702	0	0	#NAME?	1.83E-10	2.32E-08
ENSG000002	87.201952	132.86618	41.53772	0.3126282	-1.67748	0.0123189	0.1593503
ENSG000001	288.77711	416.38769	161.16654	0.3870588	-1.369375	0.0146469	0.1807162
ENSG000001	180.25641	286.08377	74.429054	0.2601652	-1.9425	0.0010869	0.0254809
ENSG000001	2563.4567	1712.8546	3414.0587	1.9931982	0.9950852	0.0052683	0.0858832
ENSG000001	2166.3238	1332.9532	2999.6944	2.2504124	1.1701894	0.0024339	0.0480749
ENSG00000(	189.4741	99.445838	279.50236	2.8105988	1.4908776	0.0012282	0.0280703
ENSG000002	6.9467743	13.893549	0	0	#NAME?	0.0492096	0.3959515
ENSG000001	28.173372	8.2854748	48.06127	5.8006657	2.5362185	0.0097449	0.1351645
ENSG000001	80.988815	127.79201	34.185617	0.2675098	-1.902336	0.0336439	0.3139441
ENSG000001	202.80787	59.364717	346.25101	5.8326062	2.5441407	8.93E-05	0.0033614
ENSG000001	194.55696	297.68847	91.425449	0.3071179	-1.703136	0.001061	0.0249991
ENSG000001	1594.6295	2481.2106	708.04845	0.2853641	-1.809124	0.0191046	0.2161386
ENSG000001	223.78233	134.97947	312.58519	2.315798	1.2115094	0.0210269	0.2306596
ENSG000002	266.34737	430.03301	102.66173	0.2387299	-2.066549	0.0004696	0.0130595
ENSG00000(	800.70522	1038.3587	563.05173	0.5422517	-0.882966	0.0084172	0.1216756
ENSG000001	1431.654	1061.7251	1801.583	1.6968452	0.7628549	0.0167473	0.1992957
ENSG000001	911.77388	1240.0206	583.52715	0.4705786	-1.087492	0.003166	0.0592093
ENSG000001	64.351061	7.8969225	120.8052	15.297757	3.9352482	0.0017895	0.0377919

ENSG00000(	713.69013	392.62929	1034.751	2.6354401	1.3980439	0.0008414	0.0208879
ENSG000001	227.22625	450.89599	3.5565239	0.0078877	-6.986183	9.49E-23	5.35E-20
ENSG000001	339.23143	244.50316	433.95971	1.7748634	0.827708	0.048808	0.3939138
ENSG000001	327.75898	228.25312	427.26484	1.8718904	0.904496	0.0078803	0.1159985
ENSG000001	35.637539	60.477839	10.797239	0.1785322	-2.485744	0.0264197	0.2683722
ENSG000001	337.51226	197.80876	477.21576	2.4125107	1.2705353	0.0048305	0.0808898
ENSG000001	1284.9115	889.94407	1679.8789	1.887623	0.9165707	0.0163998	0.1960209
ENSG000001	168.60176	81.947398	255.25612	3.1148777	1.6391755	0.0276436	0.2753268
ENSG00000(	284.35716	179.90163	388.81269	2.1612516	1.111867	0.0492214	0.3959515
ENSG000001	697.75782	1340.0925	55.423141	0.0413577	-4.5957	2.15E-06	0.0001308
ENSG00000:	43.662875	4.4222766	82.903474	18.746786	4.2285714	0.0048247	0.0808898
ENSG000001	150.83617	43.585977	258.08636	5.9213163	2.5659179	0.0032455	0.0604034
ENSG000001	12366.076	18731.164	6000.9892	0.3203746	-1.642168	5.87E-06	0.0003139
ENSG000001	956.24736	518.00724	1394.4875	2.6920231	1.4286908	0.0010288	0.0244847
ENSG000001	6895.2678	8885.2571	4905.2784	0.5520694	-0.857079	0.0166339	0.1983825
ENSG00000(	1386.1452	1726.5128	1045.7775	0.6057166	-0.723285	0.0422968	0.3592263
ENSG000001	321.27762	521.77544	120.77979	0.2314785	-2.11105	0.0061305	0.0969026
ENSG000001	351.06033	150.74814	551.37251	3.6575742	1.8708871	0.002172	0.0442584
ENSG000001	501.07769	61.254943	940.90044	15.3604	3.9411439	1.29E-13	2.47E-11
ENSG000001	645.54482	358.47212	932.61752	2.6016459	1.3794246	0.0035662	0.0648113
ENSG000001	3383.5458	865.4381	5901.6534	6.8192669	2.7696167	2.08E-14	4.78E-12
ENSG00000:	64.885812	116.19235	13.579276	0.1168689	-3.097037	0.0008954	0.0219153
ENSG000001	5911.3802	4268.6316	7554.1289	1.769684	0.8234917	0.0033756	0.062138
ENSG000001	140.73161	34.425547	247.03768	7.1759987	2.8431796	0.0011495	0.0266557
ENSG000001	2347.4133	959.28157	3735.545	3.8941069	1.9612925	1.61E-06	0.0001011
ENSG000001	13.773712	0	27.547423	Inf	Inf	0.0177983	0.2076811
ENSG000001	149.00702	272.42335	25.590684	0.0939372	-3.41216	7.56E-08	6.24E-06
ENSG00000(	134.18243	43.731328	224.63354	5.1366732	2.3608343	0.0007587	0.0192952
ENSG000001	289.16363	200.16525	378.16201	1.889249	0.9178129	0.0365868	0.3309076
ENSG00000(	196.39012	332.23923	60.54102	0.1822212	-2.456238	6.20E-05	0.0024573
ENSG000001	42.141873	84.283747	0	0	#NAME?	0.0204676	0.2269043
ENSG000001	1049.5116	644.48254	1454.5406	2.2569124	1.1743504	0.0041141	0.0723095
ENSG000001	57.601913	31.56755	83.636277	2.6494383	1.4056866	0.0209079	0.2299305
ENSG00000(	140.46477	0	280.92953	Inf	Inf	6.00E-16	1.62E-13
ENSG000001	23.67368	0	47.34736	Inf	Inf	0.0007876	0.0198593
ENSG000001	63.220756	108.29543	18.146086	0.167561	-2.577242	0.0076769	0.1139571
ENSG00000:	18.678263	0	37.356525	Inf	Inf	0.0036422	0.065922
ENSG000001	34.520423	0	69.040846	Inf	Inf	0.0057269	0.092138
ENSG00000:	31.940641	0	63.881281	Inf	Inf	0.0094652	0.1325498
ENSG000001	1240.1055	1530.2863	949.92464	0.6207496	-0.687917	0.0490491	0.3952702
ENSG000001	790.01579	551.33192	1028.6997	1.8658446	0.8998288	0.0060321	0.0957203
ENSG000001	466.80391	332.39968	601.20815	1.8086905	0.8549455	0.0441569	0.3705207
ENSG00000(	383.17514	176.75293	589.59735	3.3357146	1.7379959	0.0011727	0.0269621
ENSG000001	129.03201	76.717806	181.34622	2.3638087	1.2411133	0.0174991	0.2056308
ENSG000001	186.99443	112.87816	261.1107	2.3132084	1.2098952	0.0312379	0.2985247
ENSG00000(	277.98965	141.60785	414.37145	2.9261898	1.5490234	0.0048621	0.081218
ENSG00000(	1432.2572	400.34058	2464.1738	6.1551937	2.6218043	3.96E-11	5.83E-09
ENSG000001	330.75593	218.96245	442.54942	2.0211202	1.0151551	0.0209295	0.2300458
ENSG000001	77.178266	42.146908	112.20962	2.6623453	1.4126977	0.0491162	0.3955752
ENSG00000(	45685.624	57510.736	33860.513	0.5887686	-0.764227	0.0229434	0.2448236
ENSG000001	115.96356	31.013506	200.91361	6.4782618	2.6956068	3.01E-06	0.0001737

ENSG00000(	28.288366	0	56.576732	Inf	Inf	0.0002211	0.007005
ENSG000001	30.923842	0	61.847684	Inf	Inf	9.41E-05	0.0035186
ENSG000001	404.98847	278.63297	531.34397	1.9069673	0.9312801	0.0238102	0.2508092
ENSG000001	1054.8142	1690.0186	419.60985	0.2482871	-2.009919	2.18E-06	0.0001323
ENSG000001	7.4205896	14.841179	0	0	#NAME?	0.0381488	0.3378143
ENSG000001	1961.9422	1409.0465	2514.838	1.78478	0.8357463	0.0084227	0.1216909
ENSG000001	753.00066	1403.0081	102.99328	0.0734089	-3.767901	8.31E-11	1.11E-08
ENSG000002	41.670648	7.4205896	75.920707	10.231088	3.3548877	0.0299307	0.2911408
ENSG000001	251.27711	139.79534	362.75889	2.5949284	1.3756947	0.0071771	0.1088677
ENSG000001	1104.1808	1874.0295	334.3321	0.1784028	-2.48679	5.14E-10	6.13E-08
ENSG000001	2455.9006	3158.5419	1753.2593	0.555085	-0.849219	0.0246281	0.2569453
ENSG000001	760.54189	536.53321	984.55058	1.8350226	0.8757979	0.0117755	0.154463
ENSG000001	1875.1214	1448.6333	2301.6094	1.5888144	0.6679506	0.0359321	0.3268641
ENSG000001	1388.7883	954.5456	1823.031	1.9098417	0.9334531	0.0230422	0.245684
ENSG000001	533.88485	335.4131	732.35659	2.1834466	1.1266072	0.0136495	0.1713467
ENSG000001	61.988513	26.834431	97.142594	3.6200728	1.8560187	0.0149114	0.1829795
ENSG000002	435.40017	755.48625	115.31409	0.1526356	-2.711837	1.16E-09	1.28E-07
ENSG000001	151.01298	75.381623	226.64435	3.006626	1.5881454	0.0072832	0.1096776
ENSG000002	23.340468	9.9425698	36.738366	3.6950574	1.8855968	0.0412873	0.3527868
ENSG000001	1797.3616	2569.2595	1025.4636	0.3991281	-1.325076	0.0105181	0.1423157
ENSG000001	392.88311	166.95068	618.81554	3.706577	1.8900875	0.0001108	0.004
ENSG000002	261.56118	117.43572	405.68664	3.4545422	1.7884945	1.60E-05	0.0007593
ENSG000001	98.043977	59.670524	136.41743	2.2861778	1.1929376	0.042662	0.3611753
ENSG000001	389.06309	272.01466	506.11153	1.8606039	0.8957709	0.0452848	0.3770618
ENSG000001	64.712275	7.5810456	121.8435	16.072124	4.0064887	0.016219	0.1948084
ENSG000001	66.855804	0	133.71161	Inf	Inf	5.40E-09	5.57E-07
ENSG000001	4909.9488	6123.3975	3696.5001	0.6036682	-0.728172	0.0272207	0.2731653
ENSG000002	1026.6906	553.37253	1500.0087	2.7106671	1.438648	2.28E-05	0.0010237
ENSG000001	549.28955	154.33574	944.24335	6.1181119	2.6130865	0.0324361	0.3061577
ENSG000001	282.23883	99.77682	464.70083	4.6574027	2.2195256	4.73E-05	0.0019441
ENSG000002	225.74841	128.98788	322.50894	2.5003042	1.3221036	0.0242725	0.2542106
ENSG000001	1366.3345	1915.6907	816.97839	0.4264668	-1.229495	0.0021615	0.0441126
ENSG000001	39.364388	63.310661	15.418116	0.2435311	-2.037822	0.0484465	0.3919313
ENSG000001	16.81239	0	33.62478	Inf	Inf	0.006822	0.1047732
ENSG000001	2431.0591	3286.6284	1575.4898	0.4793635	-1.060808	0.008077	0.1182105
ENSG00000(	1052.6009	1404.4846	700.71721	0.4989141	-1.003137	0.0044936	0.0770124
ENSG000001	227.62058	47.594526	407.64664	8.5649899	3.0984515	6.89E-08	5.76E-06
ENSG000001	313.20428	201.7245	424.68407	2.1052677	1.0740037	0.0257186	0.2638287
ENSG000001	820.38929	427.34553	1213.433	2.8394658	1.5056196	0.0001134	0.0040751
ENSG000001	116.17955	194.91334	37.445764	0.1921149	-2.379958	0.00023	0.00722
ENSG000001	11593.846	15952.226	7235.4657	0.4535709	-1.1406	0.000681	0.0177541
ENSG000001	220.97928	423.04125	18.917318	0.0447174	-4.483019	5.23E-14	1.10E-11
ENSG000001	83.475769	18.952614	147.99892	7.8088924	2.9651179	0.0111427	0.1483187
ENSG000001	28575.928	10181.55	46970.306	4.6132768	2.2057919	1.50E-07	1.16E-05
ENSG00000(	1654.8377	2330.2961	979.37928	0.420281	-1.250574	0.0008182	0.0204637
ENSG000001	206.56142	9.476307	403.64653	42.595341	5.4126237	4.84E-11	6.90E-09
ENSG000001	265.56633	109.18045	421.9522	3.864723	1.950365	0.0014345	0.0318442
ENSG000001	143.06492	61.244873	224.88498	3.6718988	1.8765263	0.0003392	0.0100092
ENSG000001	59.967131	8.2854748	111.64879	13.475243	3.7522394	1.88E-05	0.0008724
ENSG00000(	54.898948	93.87301	15.924887	0.1696429	-2.559427	0.0095736	0.1334729
ENSG000002	2685.6487	565.02255	4806.2749	8.5063417	3.0885388	8.95E-14	1.77E-11

ENSG000001	1617.8298	2270.5422	965.11737	0.4250603	-1.234261	0.0009973	0.0240109
ENSG000001	278.7769	207.58081	349.97299	1.6859602	0.7535705	0.0249491	0.259088
ENSG000001	16.559004	0	33.118008	Inf	Inf	0.0071281	0.1081845
ENSG000002	64.401845	13.26683	115.53686	8.7087015	3.1224576	0.0128548	0.164009
ENSG000001	658.57572	864.54149	452.60996	0.523526	-0.933667	0.0191914	0.2169334
ENSG000001	1264.6497	1560.2594	969.03997	0.6210762	-0.687158	0.027934	0.2775042
ENSG000000	1219.7574	878.08106	1561.4337	1.7782341	0.8304452	0.0206887	0.228793
ENSG000001	1245.2759	1538.8423	951.70941	0.618458	-0.693252	0.0359465	0.3268641
ENSG000001	240.90743	472.44544	9.3694202	0.0198318	-5.656044	0.0033795	0.062138
ENSG000000	262.23583	338.19842	186.27324	0.5507809	-0.860449	0.0386026	0.340302
ENSG000001	93.869734	57.692517	130.04695	2.254139	1.1725765	0.0206645	0.2286192
ENSG000002	65.576623	31.407094	99.746153	3.1759116	1.6671708	0.0023514	0.0468907
ENSG000000	1222.7914	1851.4994	594.0834	0.3208661	-1.639957	7.06E-05	0.0027527
ENSG000001	70.027056	22.499935	117.55418	5.2246451	2.385333	0.0181386	0.2096746
ENSG000001	8.8395181	17.679036	0	0	#NAME?	0.037215	0.3335176
ENSG000001	37.748774	3.158769	72.338778	22.90094	4.5173349	0.0047766	0.0804346
ENSG000001	96.936575	9.155395	184.71775	20.175837	4.3345566	3.03E-07	2.19E-05
ENSG000000	588.87568	319.38831	858.36304	2.6875218	1.4262765	0.0003143	0.0094105
ENSG000001	585.90094	734.24982	437.55205	0.5959171	-0.746816	0.0404212	0.3482456
ENSG000001	165.60339	323.43714	7.7696356	0.0240221	-5.379495	0.0029337	0.0557889
ENSG000001	359.42806	139.62481	579.23131	4.1484841	2.0525843	4.67E-06	0.0002563
ENSG000001	457.72268	34.493187	880.95217	25.539889	4.6746803	3.46E-17	1.14E-14
ENSG000001	1764.3356	29.361447	3499.3097	119.18043	6.8970035	1.56E-39	2.35E-36
ENSG000001	476.81935	610.40879	343.22992	0.5622952	-0.8306	0.0194608	0.2187905
ENSG000000	30892.436	39602.785	22182.086	0.5601143	-0.836207	0.0095221	0.1330308
ENSG000001	14.403702	28.807403	0	0	#NAME?	0.0044255	0.0761822
ENSG000001	73.080883	12.003322	134.15844	11.176776	3.4824322	0.0444737	0.3726014
ENSG000001	1029.2975	1956.8094	101.78562	0.0520161	-4.264898	1.28E-23	7.55E-21
ENSG000000	94.686997	41.831031	147.54296	3.5271175	1.8184896	0.0047151	0.0796477
ENSG000001	997.96077	565.49888	1430.4227	2.529488	1.3388454	0.0008738	0.0215364
ENSG000000	235.33935	361.36251	109.3162	0.3025112	-1.72494	0.0038493	0.0685303
ENSG000001	701.39468	913.72765	489.06171	0.5352379	-0.901748	0.0258178	0.2644421
ENSG000002	101.92235	18.709413	185.13529	9.895302	3.3067437	0.0001214	0.0043204
ENSG000002	130.92336	195.55013	66.296595	0.3390261	-1.560532	0.0054921	0.0891559
ENSG000001	155.24079	58.163815	252.31776	4.3380538	2.1170479	2.26E-05	0.001017
ENSG000000	57.595978	106.97938	8.2125731	0.0767678	-3.703355	0.0025475	0.0497022
ENSG000001	594.09342	457.86257	730.32428	1.5950731	0.6736226	0.0235322	0.2489462
ENSG000002	407.73993	200.02997	615.44988	3.0767883	1.6214252	0.0009577	0.0232231
ENSG000001	342.00892	196.49272	487.52512	2.4811358	1.3110007	0.0234376	0.2483336
ENSG000001	97.943793	0	195.88759	Inf	Inf	3.59E-12	5.79E-10
ENSG000001	113.60876	4.9712849	222.24624	44.705997	5.4823965	2.28E-11	3.45E-09
ENSG000001	57.936323	19.807429	96.065218	4.8499589	2.2779725	0.0011911	0.027339
ENSG000001	38.034635	74.526808	1.5424628	0.0206968	-5.594452	5.13E-06	0.0002799
ENSG000001	1503.2051	1996.166	1010.2442	0.5060923	-0.982528	0.0066697	0.1033137
ENSG000001	145.81072	74.356282	217.26516	2.9219476	1.5469303	0.0099841	0.1373561
ENSG000001	1083.0834	1402.5943	763.57257	0.5444002	-0.877261	0.0094774	0.1326101
ENSG000002	532.54721	814.84089	250.25353	0.3071195	-1.703128	2.33E-05	0.0010424
ENSG000001	30.66101	0	61.32202	Inf	Inf	0.0001121	0.0040347
ENSG000001	110.99719	174.31873	47.675654	0.273497	-1.870403	0.0073775	0.1106668
ENSG000001	999.90024	699.90417	1299.8963	1.857249	0.8931672	0.0097397	0.135162
ENSG000001	134.04237	39.391797	228.69294	5.805598	2.5374447	0.0071892	0.1089901

ENSG000001	395.67544	556.61186	234.73902	0.4217284	-1.245614	0.0052244	0.0854718
ENSG000001	3239.3462	1876.2976	4602.3948	2.452913	1.2944961	7.58E-05	0.0029109
ENSG000002	88.930109	134.27001	43.59021	0.3246459	-1.623061	0.036937	0.3323883
ENSG000002	24.334083	4.5776975	44.090469	9.631582	3.2677728	0.0261779	0.2670188
ENSG000001	309.0735	508.98713	109.15987	0.2144649	-2.221187	2.58E-05	0.0011448
ENSG000001	397.83832	79.259927	716.41671	9.0388263	3.1761355	1.97E-08	1.84E-06
ENSG000001	160.41036	10.103026	310.71769	30.754914	4.9427451	6.99E-11	9.51E-09
ENSG00000(	257.23859	0	514.47718	Inf	Inf	4.92E-24	3.17E-21
ENSG00000(	826.40049	24.229706	1628.5713	67.213828	6.0706862	0.0006369	0.0168469
ENSG000001	462.71082	222.9861	702.43554	3.1501315	1.6554121	0.0001289	0.0045484
ENSG000001	214.99437	89.212567	340.77618	3.8198226	1.9335056	0.0058309	0.0933703
ENSG000001	141.68303	68.815849	214.55021	3.1177442	1.6405026	0.0002064	0.0066256
ENSG000002	79.302902	1.2635076	157.3423	124.52818	6.9603284	5.64E-08	4.80E-06
ENSG000001	1033.4332	746.92451	1319.9418	1.767169	0.82144	0.0077349	0.1145425
ENSG00000(	23.426588	45.310712	1.5424628	0.0340419	-4.876545	0.0015807	0.0344132
ENSG000001	2369.4407	526.16028	4212.7211	8.0065356	3.0011781	1.81E-05	0.0008459
ENSG000001	12.647154	0	25.294307	Inf	Inf	0.0254635	0.2624022
ENSG000001	161.72735	229.54684	93.907852	0.409101	-1.289471	0.0414446	0.3537957
ENSG000002	56.007001	1.5793845	110.43462	69.922566	6.1276862	1.60E-05	0.0007593
ENSG000001	573.54718	895.15571	251.93865	0.2814467	-1.829066	0.0297703	0.2901021
ENSG000001	686.13296	415.31485	956.95106	2.304158	1.2042397	0.0103604	0.1407218
ENSG000001	218.84763	162.59101	275.10426	1.6920017	0.758731	0.0386988	0.3408942
ENSG000001	332.29655	141.15166	523.44144	3.708362	1.8907821	0.0008967	0.02192
ENSG000001	525.61818	774.23594	277.00042	0.3577726	-1.482885	0.0059613	0.094936
ENSG00000(	10.173504	20.050631	0.296377	0.0147814	-6.08007	0.0289027	0.2844127
ENSG00000(	870.81014	397.40487	1344.2154	3.3824835	1.7580829	1.22E-06	7.91E-05
ENSG000001	147.24934	261.28492	33.213759	0.127117	-2.975771	2.68E-06	0.0001566
ENSG000001	21.420564	0	42.841127	Inf	Inf	0.0015651	0.0341563
ENSG00000(	316.89885	183.73462	450.06307	2.4495279	1.2925037	0.0021316	0.0436335
ENSG000002	1332.684	273.19039	2392.1776	8.7564488	3.1303459	1.98E-13	3.67E-11
ENSG000001	859.20716	1240.6696	477.74468	0.38507	-1.376807	0.0010876	0.0254809
ENSG000002	63.30488	35.75166	90.858099	2.5413673	1.3456049	0.0346389	0.3195923
ENSG000001	404.78192	208.00461	601.55923	2.8920477	1.5320913	0.0024798	0.0486282
ENSG000001	537.14272	926.06699	148.21844	0.1600515	-2.643392	1.34E-09	1.47E-07
ENSG000001	205.24093	310.14295	100.33891	0.3235247	-1.628052	0.0049715	0.082523
ENSG000001	28646.718	40789.232	16504.203	0.4046216	-1.305355	0.0009636	0.0233246
ENSG000002	399.64078	263.14997	536.13159	2.0373614	1.0267019	0.004478	0.0768423
ENSG000001	1376.6667	1695.8807	1057.4528	0.623542	-0.681441	0.031785	0.3021189
ENSG000001	436.42217	586.382	286.46233	0.4885251	-1.033495	0.011499	0.1520148
ENSG000001	385.89923	541.55266	230.24581	0.4251587	-1.233927	0.0027478	0.0529608
ENSG000002	12.25991	0	24.51982	Inf	Inf	0.0274554	0.2744626
ENSG000001	335.81307	482.81969	188.80645	0.3910496	-1.354576	0.0020515	0.0422811
ENSG000001	711.94036	1094.7525	329.12824	0.3006417	-1.733883	1.68E-05	0.0007915
ENSG00000(	4235.005	0	8470.0099	Inf	Inf	4.00E-72	1.08E-67
ENSG000001	406.4429	249.90831	562.97748	2.2527361	1.1716783	0.0067941	0.1044638
ENSG000001	541.01278	306.42007	775.60548	2.5311837	1.3398122	0.0008487	0.0210312
ENSG000001	24.848114	0	49.696227	Inf	Inf	0.0006004	0.0160476
ENSG000002	41.515264	64.734624	18.295903	0.2826293	-1.823017	0.0480129	0.3898223
ENSG000001	536.34683	1048.9705	23.723183	0.0226157	-5.466533	7.25E-28	5.77E-25
ENSG000001	8154.5888	6104.2767	10204.901	1.6717625	0.7413699	0.0092238	0.1297324
ENSG000002	148.52296	0	297.04592	Inf	Inf	1.52E-16	4.42E-14

ENSG000001	450.39262	298.31015	602.47509	2.0196265	1.0140885	0.0270271	0.2723951
ENSG000001	89.854878	1.5793845	178.13037	112.78468	6.8174273	1.54E-08	1.47E-06
ENSG000001	2355.191	3197.8661	1512.5159	0.4729766	-1.080159	0.047624	0.3879832
ENSG000001	1233.7961	797.33742	1670.2548	2.0947905	1.0668059	0.025584	0.2633142
ENSG000001	317.09164	195.76815	438.41513	2.239461	1.1631515	0.0212233	0.2320498
ENSG000001	46.633325	90.538679	2.7279707	0.0301304	-5.052634	1.52E-05	0.0007264
ENSG000001	96.380552	178.56041	14.200691	0.0795288	-3.652379	2.86E-06	0.0001662
ENSG000002	106.67093	40.412103	172.92976	4.2791578	2.0973269	0.0019899	0.0412644
ENSG000001	28.379996	56.759991	0	0	#NAME?	3.99E-06	0.0002227
ENSG000001	69.230491	36.694256	101.76673	2.7733694	1.4716398	0.0050714	0.0835793
ENSG000001	2638.9205	1649.6893	3628.1516	2.1992939	1.1370404	0.0014377	0.0318909
ENSG000001	2110.7068	3823.8667	397.54703	0.1039647	-3.265835	7.79E-17	2.35E-14
ENSG000001	282.1973	361.41286	202.98174	0.561634	-0.832298	0.0401252	0.3468208
ENSG000002	39.068997	9.7094384	68.428555	7.0476327	2.8171387	0.0223864	0.2405872
ENSG000000	206.11469	114.59786	297.63152	2.5971822	1.3769472	0.0062061	0.0977553
ENSG000001	189.74013	307.34537	72.134896	0.234703	-2.091092	0.0030322	0.0572209
ENSG000000	2094.6206	2833.7478	1355.4934	0.4783395	-1.063893	0.0014163	0.0315697
ENSG000002	133.47967	59.369752	207.58959	3.4965548	1.8059341	0.0266962	0.2701666
ENSG000000	497.15103	317.16995	677.13211	2.1349188	1.0941812	0.0139684	0.1740321
ENSG000001	383.12924	233.8137	532.44478	2.2772181	1.1872725	0.0139429	0.1737943
ENSG000001	387.3243	526.77912	247.86949	0.4705378	-1.087617	0.0089902	0.1275071
ENSG000001	706.8194	425.71865	987.92015	2.320594	1.2144941	0.0065372	0.1015524
ENSG000001	645.16272	834.17199	456.15345	0.5468338	-0.870826	0.0286444	0.2824881
ENSG000001	1520.5734	458.09066	2583.0561	5.6387442	2.4953739	1.36E-11	2.10E-09
ENSG000001	365.91018	510.84496	220.9754	0.4325684	-1.209	0.0030266	0.0571539
ENSG000001	4467.9281	5583.6464	3352.2097	0.6003621	-0.736095	0.0445509	0.372902
ENSG000001	1926.4754	1514.4688	2338.482	1.5440938	0.6267604	0.0215184	0.2342356
ENSG000002	1032.6132	769.5374	1295.6891	1.6837246	0.7516562	0.0306305	0.2950843
ENSG000002	56.662048	15.162091	98.162005	6.4741732	2.694696	0.0488302	0.3939758
ENSG000001	57.446414	17.756747	97.136082	5.470376	2.45164	0.0018916	0.0394976
ENSG000001	117.00992	191.37609	42.643757	0.222827	-2.166004	0.0127578	0.163079
ENSG000001	1836.5632	2532.0876	1141.0389	0.4506317	-1.149979	0.0037164	0.0668674
ENSG000001	24.885131	45.471168	4.2990944	0.0945455	-3.402847	0.0121459	0.1582472
ENSG000001	429.43613	567.54738	291.32488	0.5133049	-0.962112	0.0307379	0.2956984
ENSG000001	12.425276	24.07932	0.7712314	0.0320288	-4.964487	0.0161809	0.1945907
ENSG000001	239.98243	163.92216	316.04271	1.9280048	0.9471087	0.0127539	0.163079
ENSG000001	472.59939	694.10106	251.09772	0.3617596	-1.466897	0.0050093	0.0829085
ENSG000002	79.409393	133.31734	25.501445	0.1912838	-2.386214	0.0057949	0.0929
ENSG000001	40.529517	63.237985	17.821048	0.2818092	-1.827209	0.0388251	0.3411188
ENSG000000	973.10523	1199.4682	746.74227	0.6225611	-0.683713	0.0421522	0.3584792
ENSG000001	145.75691	216.07709	75.436728	0.3491195	-1.518207	0.0036714	0.066367
ENSG000000	113.96026	38.371491	189.54902	4.93984	2.3044643	0.0106234	0.1433818
ENSG000001	340.7883	219.13801	462.43859	2.1102619	1.0774221	0.0425402	0.3604196
ENSG000001	249.81538	328.17311	171.45765	0.522461	-0.936605	0.0246301	0.2569453
ENSG000002	142.91424	194.20891	91.619563	0.4717578	-1.083882	0.031934	0.3029935
ENSG000001	106.47405	26.917177	186.03093	6.9112348	2.7889435	0.0003274	0.0097353
ENSG000000	841.82655	307.82893	1375.8242	4.4694441	2.1600954	6.38E-08	5.36E-06
ENSG000002	559.40034	726.1097	392.69099	0.540815	-0.886793	0.0183244	0.2111013
ENSG000001	281.92589	168.45235	395.39943	2.3472479	1.2309702	0.0174933	0.2056308
ENSG000002	34.834342	5.6857842	63.982901	11.253136	3.4922551	0.0370223	0.332455
ENSG000001	49.84452	99.689039	0	0	#NAME?	6.16E-09	6.24E-07

ENSG000001	540.32312	316.94404	763.70221	2.4095806	1.2687821	0.0082278	0.1195777
ENSG000001	165.95828	279.54317	52.373388	0.1873535	-2.416165	9.02E-06	0.0004678
ENSG00000(	1385.4137	2523.6979	247.12953	0.0979236	-3.3522	0.0197461	0.2211716
ENSG00000(	140.04198	47.040483	233.04348	4.9541049	2.3086244	0.000106	0.0038703
ENSG000001	37.446772	73.351081	1.5424628	0.0210285	-5.571511	0.0078536	0.1157091
ENSG000001	438.98621	742.10931	135.8631	0.1830769	-2.449478	0.0005902	0.015828
ENSG000001	649.22915	444.09926	854.35904	1.923802	0.9439603	0.0378494	0.3367002
ENSG000001	970.15985	1247.3585	692.96124	0.555543	-0.84803	0.0189511	0.2152057
ENSG000001	32707.99	40182.072	25233.908	0.6279892	-0.671188	0.0356818	0.3255311
ENSG000001	1882.6427	2418.325	1346.9603	0.5569807	-0.844301	0.0142187	0.1764197
ENSG000001	292.48837	489.71141	95.265325	0.1945336	-2.361909	0.0005422	0.0147917
ENSG000001	23.954349	43.580942	4.3277553	0.0993038	-3.332007	0.0116644	0.1533026
ENSG000001	1266.0943	1595.8556	936.33298	0.5867279	-0.769237	0.0175406	0.2060183
ENSG000001	319.42945	190.79687	448.06204	2.3483721	1.231661	0.0178828	0.2080974
ENSG00000(	293.01556	62.280284	523.75084	8.4095769	3.0720332	5.19E-08	4.47E-06
ENSG000001	3473.9063	4388.9251	2558.8874	0.5830328	-0.778351	0.0176969	0.2073139
ENSG000001	309.41671	483.42627	135.40714	0.2800989	-1.835992	0.00022	0.0069949
ENSG000001	233.75162	150.58769	316.91556	2.1045251	1.0734947	0.0060345	0.0957203
ENSG000001	941.86441	1267.5774	616.15141	0.4860858	-1.040717	0.0242708	0.2542106
ENSG000001	215.85344	293.59718	138.10971	0.4704054	-1.088023	0.0182427	0.2103619
ENSG00000(	523.48663	256.70722	790.26604	3.0784722	1.6222145	0.0004818	0.0133585
ENSG00000(	86.328558	22.743137	149.91398	6.591614	2.7206318	0.0189478	0.2152057
ENSG000001	2025.1711	1347.054	2703.2881	2.0068149	1.0049075	0.0011159	0.0260536
ENSG000001	459.33583	318.18522	600.48644	1.8872229	0.9162648	0.0192524	0.2172087
ENSG00000(	288.12693	0	576.25387	Inf	Inf	6.37E-26	4.66E-23
ENSG00000(	605.29055	401.61919	808.9619	2.0142511	1.0102436	0.0201806	0.2249205
ENSG00000(	14.010114	28.020228	0	0	#NAME?	0.0036104	0.0654834
ENSG000002	15.137697	0	30.275394	Inf	Inf	0.0116413	0.1530729
ENSG000001	289.75095	186.65522	392.84668	2.1046648	1.0735905	0.0041772	0.0730889
ENSG000001	24.302885	8.2854748	40.320295	4.8663831	2.2828499	0.0217509	0.2359499
ENSG000001	415.30133	303.97076	526.63189	1.7325084	0.7928623	0.0375803	0.3357417
ENSG000001	134.5988	265.10565	4.0919561	0.0154352	-6.017633	2.69E-09	2.87E-07
ENSG000001	44.841932	89.683864	0	0	#NAME?	3.24E-09	3.38E-07
ENSG000001	210.30533	153.03699	267.57367	1.7484248	0.8060558	0.0248229	0.2583887
ENSG000001	509.94386	675.85791	344.02981	0.5090268	-0.974186	0.0139035	0.1734636
ENSG000002	124.31052	75.143457	173.47758	2.3086185	1.2070298	0.036838	0.3323387
ENSG000001	13.816703	0	27.633406	Inf	Inf	0.0168966	0.200214
ENSG000001	26.047952	0	52.095904	Inf	Inf	0.0004097	0.0116945
ENSG000001	298.03065	486.34972	109.71158	0.2255817	-2.148278	0.0358567	0.3264867
ENSG000001	302.5824	148.63485	456.52995	3.0714865	1.618937	0.001105	0.0258459
ENSG000001	339.47406	166.71755	512.23057	3.0724455	1.6193874	0.0016929	0.0361211
ENSG000001	1269.3682	470.1868	2068.5496	4.3994208	2.1373136	1.44E-08	1.38E-06
ENSG000001	7296.936	5484.3874	9109.4845	1.6609849	0.7320389	0.0290975	0.2851896
ENSG000001	745.99534	952.9266	539.06408	0.5656932	-0.821908	0.017592	0.2065324
ENSG000001	275.74305	159.28689	392.19922	2.4622191	1.2999591	0.0049452	0.0823011
ENSG000001	268.73996	406.22206	131.25786	0.3231185	-1.629865	0.0015449	0.0338232
ENSG000002	708.17066	957.33159	459.00974	0.4794679	-1.060494	0.0462397	0.3813753
ENSG000001	15.118483	0	30.236965	Inf	Inf	0.010492	0.1420332
ENSG000001	14.383772	26.839466	1.9280785	0.0718374	-3.79912	0.036878	0.3323387
ENSG000001	328.98105	62.891898	595.07021	9.4617945	3.2421138	4.02E-14	8.77E-12
ENSG000001	258.93382	57.459385	460.40825	8.0127598	3.0022992	2.13E-08	1.98E-06

ENSG000001	321.23942	458.33168	184.14715	0.401777	-1.315533	0.0055366	0.0896083
ENSG00000(	514.32818	679.87149	348.78487	0.5130159	-0.962925	0.0087478	0.1250525
ENSG00000(	345.00219	459.84846	230.15593	0.5005039	-0.998547	0.0192324	0.2172087
ENSG000001	166.98644	83.200835	250.77204	3.0140567	1.5917066	0.0103124	0.1404445
ENSG000002	774.57266	1128.5441	420.60124	0.3726937	-1.423938	0.0045019	0.0771054
ENSG000001	44.63662	9.9425698	79.33067	7.97889	2.9961881	0.00338	0.062138
ENSG000001	43.000332	78.705883	7.2947812	0.0926841	-3.431535	0.0008843	0.0217538
ENSG000001	131.39296	179.67857	83.107357	0.4625335	-1.11237	0.0433061	0.3651029
ENSG000002	11.288823	22.577646	0	0	#NAME?	0.0066086	0.1024851
ENSG000001	49.902519	85.57243	14.232608	0.1663224	-2.587946	0.005964	0.094936
ENSG000001	523.2117	668.48482	377.93859	0.565366	-0.822743	0.0299674	0.2911839
ENSG000001	3570.3462	4313.7974	2826.8949	0.6553147	-0.60974	0.0336777	0.3141508
ENSG000001	296.18971	214.84598	377.53344	1.7572283	0.8133017	0.0249583	0.259088
ENSG00000(	380.48712	741.43224	19.541989	0.0263571	-5.245666	2.25E-23	1.29E-20
ENSG000001	137.32029	36.150282	238.4903	6.5971905	2.7218518	4.29E-05	0.0017795
ENSG000001	318.20577	114.94898	521.46256	4.5364696	2.18157	0.0003873	0.0112092
ENSG00000(	797.29304	483.75222	1110.8339	2.2962869	1.1993029	0.0088375	0.1259351
ENSG000001	767.80469	430.29853	1105.3108	2.568707	1.3610423	5.04E-05	0.0020339
ENSG000001	487.585	651.41236	323.75764	0.4970087	-1.008657	0.0239146	0.2517086
ENSG000001	194.01139	258.70537	129.31741	0.4998636	-1.000394	0.0246984	0.2575115
ENSG000001	128.74703	42.156979	215.33708	5.1079818	2.3527534	0.0024666	0.048438
ENSG000001	183.63082	365.77975	1.481885	0.0040513	-7.947398	1.89E-21	9.46E-19
ENSG000001	587.86337	1043.497	132.22972	0.1267179	-2.980308	0.0368845	0.3323387
ENSG000002	12.393768	0	24.787536	Inf	Inf	0.0266139	0.269636
ENSG000001	141.04968	5.3699073	276.72944	51.533375	5.6874352	2.26E-09	2.42E-07
ENSG000001	90.819689	125.21246	56.426915	0.4506494	-1.149923	0.0450313	0.3759926
ENSG000001	840.05413	537.64414	1142.4641	2.1249448	1.0874254	0.0082123	0.1194158
ENSG000001	702.34254	185.4795	1219.2056	6.5732635	2.7166098	5.49E-11	7.67E-09
ENSG000001	72.554034	131.67535	13.432715	0.1020139	-3.293162	5.92E-05	0.0023619
ENSG000002	1873.2476	2330.9753	1415.5198	0.607265	-0.719602	0.0181149	0.2096746
ENSG000001	45.507455	6.317538	84.697373	13.406706	3.7448829	0.0259435	0.2654285
ENSG000001	863.09931	1058.6626	667.53601	0.6305465	-0.665325	0.0411695	0.3523345
ENSG000002	15.330505	0	30.66101	Inf	Inf	0.0109858	0.1470376
ENSG000002	168.15473	261.38277	74.9267	0.2866551	-1.802612	0.0009546	0.0231684
ENSG000001	379.4246	273.25803	485.59117	1.7770427	0.8294783	0.0218689	0.2368129
ENSG000001	453.8714	237.24303	670.49978	2.8262149	1.4988712	1.63E-05	0.0007698
ENSG000001	674.83794	924.06163	425.61424	0.4605908	-1.118443	0.0061105	0.0966431
ENSG000002	72.786968	145.57394	0	0	#NAME?	1.35E-13	2.57E-11
ENSG00000(	274.90348	200.78694	349.02003	1.7382606	0.7976444	0.0377346	0.3361675
ENSG000001	933.37003	578.95353	1287.7865	2.2243349	1.153374	0.0006072	0.0161873
ENSG00000(	193.76401	375.55179	11.976235	0.0318897	-4.970766	5.69E-06	0.0003066
ENSG000001	31.703213	0	63.406427	Inf	Inf	0.0078461	0.1157072
ENSG000001	563.56216	1109.1821	17.942204	0.0161761	-5.949995	4.08E-20	1.78E-17
ENSG000001	160.06462	69.545453	250.58379	3.6031657	1.849265	0.0041419	0.0726408
ENSG000001	662.71027	0	1325.4205	Inf	Inf	4.75E-40	7.56E-37
ENSG000001	334.48541	508.27263	160.69819	0.3161654	-1.661249	0.0013865	0.0311355
ENSG000001	2417.8061	4768.6337	66.978587	0.0140457	-6.153732	5.70E-46	1.40E-42
ENSG000001	84.323969	17.37323	151.27471	8.7073453	3.1222329	0.0057796	0.092775
ENSG000002	52.84457	20.599639	85.089501	4.1306307	2.0463621	0.014855	0.1826327
ENSG000001	660.86452	1004.7203	317.0087	0.3155193	-1.6642	1.35E-05	0.0006549
ENSG00000(	435.90095	254.4083	617.39359	2.4267824	1.2790448	0.0034063	0.0624943

ENSG000001	356.57821	482.22537	230.93106	0.4788862	-1.062245	0.0339122	0.3154687
ENSG000001	174.98347	83.511677	266.45526	3.1906347	1.6738435	0.0036828	0.0664407
ENSG000002	75.917037	1.2635076	150.57057	119.16871	6.8968616	1.39E-07	1.08E-05
ENSG000001	184.06015	93.153475	274.96682	2.9517613	1.561576	0.0196179	0.219917
ENSG000001	167.44866	228.54164	106.35569	0.4653668	-1.10356	0.0331619	0.3112144
ENSG000001	848.82776	275.72747	1421.9281	5.1570054	2.3665335	9.09E-08	7.37E-06
ENSG000001	442.42821	307.66847	577.18794	1.8760062	0.9076646	0.008445	0.1218829
ENSG000001	175.57623	267.37939	83.773069	0.3133116	-1.67433	0.0034	0.0624194
ENSG000001	653.49531	295.72053	1011.2701	3.4196817	1.773862	0.0003102	0.0093256
ENSG000001	8631.5545	12532.064	4731.0445	0.3775152	-1.405393	0.0317834	0.3021189
ENSG000002	509.81782	314.06372	705.57192	2.2465884	1.1677359	0.0016196	0.0349988
ENSG000001	1397.3376	1791.6209	1003.0543	0.5598585	-0.836866	0.0237616	0.2504914
ENSG000001	672.4109	475.01274	869.80907	1.8311279	0.8727326	0.0200886	0.224234
ENSG000001	43.348742	8.6063868	78.091097	9.0736216	3.1816785	0.0215982	0.2348207
ENSG000001	29.249482	54.94244	3.5565239	0.0647318	-3.949381	0.0038222	0.0681825
ENSG000001	1319.4128	1684.2033	954.62237	0.5668095	-0.819064	0.010991	0.1470376
ENSG000001	69.232954	17.052318	121.41359	7.1200639	2.8318902	0.005331	0.0867484
ENSG000002	260.70421	188.40513	333.00329	1.7674852	0.8216982	0.0312356	0.2985247
ENSG000001	1215.0533	766.93553	1663.1711	2.1685931	1.1167594	0.0167958	0.1996483
ENSG000002	2246.3248	2809.9391	1682.7105	0.5988423	-0.739752	0.0236189	0.2497664
ENSG000001	135.76387	209.67681	61.85094	0.2949823	-1.7613	0.0022774	0.045795
ENSG000001	96.941584	39.304016	154.57915	3.9329098	1.9755971	0.0019794	0.0411088
ENSG000002	31.563147	0.3158769	62.810417	198.8446	7.6354976	0.0022694	0.0457623
ENSG000002	14.854023	0	29.708045	Inf	Inf	0.0120813	0.1577101
ENSG000001	714.42568	1018.4103	410.44105	0.4030213	-1.311072	0.0070692	0.1075919
ENSG000002	129.44663	67.091113	191.80214	2.8588308	1.5154252	0.0092136	0.129657
ENSG000001	267.71571	174.13814	361.29328	2.074751	1.0529382	0.0477932	0.3884039
ENSG000002	901.06837	345.42835	1456.7084	4.2171073	2.0762537	1.20E-06	7.77E-05
ENSG000001	95.092478	28.428921	161.75603	5.6898408	2.5083883	0.0387675	0.3411188
ENSG000001	161.82051	25.73138	297.90965	11.577679	3.5332741	1.55E-07	1.19E-05
ENSG000001	64.335864	1.2635076	127.40822	100.83692	6.6558802	1.57E-06	9.85E-05
ENSG000000	50.344503	7.5810456	93.10796	12.281678	3.6184358	0.0104342	0.1415337
ENSG000002	225.38232	120.37142	330.39322	2.7447812	1.4566911	0.003295	0.0609895
ENSG000001	11675.723	7596.4906	15754.956	2.0739782	1.0524008	0.0136515	0.1713467
ENSG000001	205.0234	158.16873	251.87808	1.5924644	0.6712611	0.0458291	0.3791447
ENSG000001	29.422994	10.574324	48.271664	4.5649884	2.1906112	0.020704	0.2288683
ENSG000001	408.33263	550.09862	266.56664	0.4845797	-1.045194	0.0474717	0.3871706
ENSG000000	776.59755	985.64755	567.54755	0.5758119	-0.796331	0.0413815	0.3533686
ENSG000002	7.5785281	15.157056	0	0	#NAME?	0.035058	0.3217228
ENSG000001	248.37803	418.53404	78.22202	0.1868952	-2.419698	0.0359046	0.3268128
ENSG000001	538.19794	317.65132	758.74456	2.3886082	1.2561702	0.0066965	0.1036693
ENSG000001	184.27575	82.962669	285.58884	3.4423777	1.7834054	0.0028627	0.0546312
ENSG000000	699.2233	489.87406	908.57255	1.8547064	0.8911908	0.0206229	0.2283773
ENSG000001	4856.8658	3272.2246	6441.507	1.9685406	0.9771265	0.0283808	0.2807063
ENSG000001	236.71695	335.50592	137.92798	0.4111044	-1.282423	0.0090818	0.1284021
ENSG000002	11.079762	21.863146	0.296377	0.013556	-6.204924	0.0318052	0.302135
ENSG000001	445.63558	579.58813	311.68304	0.5377664	-0.894948	0.0318161	0.302135
ENSG000002	59.162888	118.32578	0	0	#NAME?	6.89E-11	9.41E-09
ENSG000000	398.21928	532.77574	263.66281	0.4948852	-1.014834	0.0217136	0.2357912
ENSG000001	43.344922	68.597822	18.092021	0.2637405	-1.922809	0.0444929	0.3726469
ENSG000000	451.24353	590.26534	312.22173	0.5289515	-0.918793	0.0158784	0.1920806

ENSG000001	866.53369	563.17764	1169.8897	2.0773015	1.0547106	0.02638	0.2680691
ENSG000001	231.36288	148.22616	314.4996	2.121755	1.0852581	0.0212507	0.2322562
ENSG000002	61.20212	15.395223	107.00902	6.9507938	2.7971777	0.0125834	0.1616902
ENSG000001	77.27843	47.273614	107.28325	2.2694107	1.1823177	0.026827	0.2709884
ENSG000001	315.57582	151.77852	479.37312	3.1583727	1.6591814	0.0001041	0.0038158
ENSG000001	10.390781	0	20.781562	Inf	Inf	0.0481792	0.3908202
ENSG000001	11.401068	0	22.802136	Inf	Inf	0.0369735	0.332455
ENSG000001	947.23427	528.74202	1365.7265	2.5829733	1.3690327	0.0016921	0.0361211
ENSG000001	667.81548	855.06518	480.56579	0.5620224	-0.8313	0.0272575	0.273191
ENSG000000	179.68672	33.540522	325.83292	9.714605	3.2801553	7.16E-09	7.17E-07
ENSG000001	207.88438	285.76286	130.00591	0.4549433	-1.136241	0.0250166	0.2593767
ENSG000001	34.008175	64.10287	3.9134787	0.06105	-4.033865	0.0008063	0.0202225
ENSG000001	12.510958	25.021915	0	0	#NAME?	0.0056512	0.0911908
ENSG000002	33.829986	66.474465	1.185508	0.017834	-5.809223	2.11E-05	0.0009641
ENSG000001	136.98455	49.10627	224.86283	4.5791062	2.195066	0.0059456	0.0947556
ENSG000002	27.794297	0	55.588594	Inf	Inf	0.0002272	0.0071659
ENSG000001	271.32177	161.09437	381.54918	2.3684824	1.243963	0.0033767	0.062138
ENSG000001	534.21366	806.89647	261.53085	0.3241195	-1.625402	0.000151	0.0051226
ENSG000001	243.44965	380.67128	106.22802	0.2790545	-1.841381	0.000176	0.0058145
ENSG000001	402.54293	147.2886	657.79726	4.4660432	2.1589972	4.16E-06	0.0002305
ENSG000001	37.316047	65.055536	9.5765585	0.1472059	-2.764093	0.0062949	0.0986942
ENSG000002	11.844005	0	23.68801	Inf	Inf	0.0306801	0.2954196
ENSG000000	4000.6512	6470.8307	1530.4717	0.2365186	-2.079975	0.0058714	0.0938538
ENSG000001	1685.1235	527.94696	2842.3	5.3836847	2.4285939	3.08E-11	4.58E-09
ENSG000001	38.951393	73.335976	4.5668105	0.0622724	-4.005262	0.0004149	0.0117805
ENSG000001	11.92295	22.660391	1.185508	0.0523163	-4.256596	0.0334631	0.3125805
ENSG000002	352.11357	195.32706	508.90008	2.6053741	1.3814905	0.0485927	0.3929971
ENSG000000	202.91648	115.09433	290.73863	2.5260899	1.336906	0.0249622	0.259088
ENSG000001	246.95262	478.42981	15.475437	0.0323463	-4.950255	8.67E-16	2.28E-13
ENSG000001	604.71253	301.41857	908.00649	3.0124437	1.5909343	0.0001693	0.0056112
ENSG000001	37.728336	14.520267	60.936404	4.1966448	2.0692364	0.0137179	0.1718617
ENSG000001	723.73256	555.98733	891.47779	1.6034139	0.6811469	0.0209684	0.2302859
ENSG000000	266.93376	30.319147	503.54837	16.608263	4.0538293	4.39E-09	4.57E-07
ENSG000000	44.771722	28.170614	61.372829	2.1786117	1.1234091	0.0241751	0.2535798
ENSG000001	1702.316	1175.7522	2228.8798	1.8957053	0.9227347	0.0308039	0.2960502
ENSG000001	617.37205	1089.225	145.51913	0.1335988	-2.904021	1.18E-05	0.0005856
ENSG000001	1534.5018	689.87382	2379.1299	3.448645	1.7860296	1.61E-06	0.0001011
ENSG000001	220.07992	80.68389	359.47595	4.4553621	2.1555427	0.0024389	0.0481231
ENSG000001	477.66519	667.3868	287.94357	0.4314493	-1.212737	0.0031245	0.0585145
ENSG000002	94.039503	28.744798	159.33421	5.5430624	2.4706832	0.0302552	0.2930338
ENSG000001	148.85488	14.214461	283.49531	19.944148	4.3178936	1.47E-06	9.31E-05
ENSG000001	294.92609	207.58584	382.26635	1.8414856	0.8808701	0.0224029	0.2406686
ENSG000001	214.06558	102.23116	325.90001	3.1878735	1.6725944	0.0091778	0.1292865
ENSG000001	77.998069	148.88309	7.1130478	0.0477761	-4.387568	7.63E-07	5.15E-05
ENSG000002	257.67927	355.7019	159.65663	0.4488495	-1.155696	0.0176699	0.2070874
ENSG000001	16.511129	0	33.022258	Inf	Inf	0.0238337	0.2509587
ENSG000001	8312.6642	3448.4473	13176.881	3.8211055	1.9339901	2.49E-07	1.82E-05
ENSG000001	411.29226	667.89335	154.69118	0.2316106	-2.110227	0.0007311	0.0187345
ENSG000001	11.405389	22.810777	0	0	#NAME?	0.0104483	0.1415824
ENSG000001	194.70261	356.55168	32.853548	0.0921425	-3.43999	2.17E-08	1.99E-06
ENSG000001	304.14566	160.42015	447.87118	2.7918636	1.4812285	0.0187709	0.2142175

ENSG000001	240.05865	85.256553	394.86074	4.6314415	2.2114613	0.0002556	0.007868
ENSG000001	918.79642	1120.1457	717.44718	0.6404945	-0.642742	0.0457576	0.3791325
ENSG000001	13.101339	26.202678	0	0	#NAME?	0.0075582	0.1126287
ENSG000001	1105.402	1394.0959	816.70807	0.5858335	-0.771437	0.0184202	0.2117546
ENSG00000(	240.19616	91.185538	389.20678	4.2682951	2.0936599	0.0015201	0.0334431
ENSG000002	271.84365	90.455934	453.23137	5.0105212	2.3249607	7.14E-06	0.0003736
ENSG000001	369.43124	514.57287	224.28961	0.4358753	-1.198013	0.0086751	0.1244117
ENSG000001	79.185352	117.54364	40.827066	0.3473354	-1.525599	0.0411947	0.3523345
ENSG000001	61.852304	116.8241	6.8805047	0.0588963	-4.08568	2.03E-05	0.0009361
ENSG000001	1878.2835	2422.2889	1334.278	0.5508335	-0.860312	0.0183439	0.2112364
ENSG000002	91.832272	39.863095	143.80145	3.607383	1.8509526	0.016939	0.2004681
ENSG000001	163.87594	83.025274	244.7266	2.9476157	1.5595484	0.0004648	0.0129465
ENSG00000(	151.30174	0	302.60348	Inf	Inf	7.82E-17	2.35E-14
ENSG000002	1306.8874	1829.5815	784.19326	0.4286189	-1.222233	0.0007892	0.0198664
ENSG000001	1164.9901	1627.5987	702.38147	0.4315446	-1.212418	0.0004136	0.0117692
ENSG00000(	148.09414	75.6975	220.49077	2.9127881	1.5424007	0.0105243	0.1423284
ENSG000001	2888.3974	914.06519	4862.7297	5.3198937	2.4113974	8.71E-14	1.75E-11
ENSG000001	305.06263	205.39484	404.73042	1.9704994	0.9785613	0.0469985	0.3852043
ENSG000002	81.103562	15.234767	146.97236	9.6471683	3.2701055	0.0002651	0.0081317
ENSG000001	943.72161	1849.4588	37.984452	0.0205381	-5.60555	1.90E-33	2.05E-30
ENSG000002	14.690329	28.491526	0.889131	0.0312069	-5.001993	0.0369137	0.3323387
ENSG00000(	692.58396	319.41852	1065.7494	3.3365297	1.7383484	1.97E-06	0.0001219
ENSG00000(	13.607937	0	27.215873	Inf	Inf	0.0174973	0.2056308
ENSG000001	953.61034	662.57816	1244.6425	1.8784841	0.9095689	0.0125617	0.1614884
ENSG000001	439.91217	243.07701	636.74734	2.6195292	1.3893076	0.0152695	0.1862357
ENSG000001	73.976406	29.920525	118.03229	3.9448602	1.9799742	0.0429697	0.3626958
ENSG000002	12.060794	23.053979	1.0676084	0.0463091	-4.432561	0.0258139	0.2644421
ENSG000002	81.559552	6.9492918	156.16981	22.472767	4.4901058	7.49E-05	0.002888
ENSG000001	361.32025	533.23479	189.40572	0.3552014	-1.493291	0.046502	0.3826057
ENSG000001	517.35291	695.42717	339.27866	0.4878709	-1.035429	0.0047069	0.079558
ENSG000001	47.123667	4.1063997	90.140934	21.95133	4.4562364	0.0022004	0.0446683
ENSG00000(	1430.995	1005.9486	1856.0415	1.8450659	0.8836724	0.0437824	0.3680628
ENSG000001	91.310358	28.108009	154.51271	5.497106	2.4586723	0.0144698	0.1787164
ENSG000001	69.646413	32.204339	107.08849	3.3252814	1.7334764	0.0276774	0.2754613
ENSG000001	12.036813	0	24.073626	Inf	Inf	0.0289268	0.2844433
ENSG000002	123.8308	74.82758	172.83401	2.3097635	1.2077451	0.0389009	0.3415327
ENSG000002	245.18823	110.82244	379.55401	3.424884	1.7760551	0.0005795	0.0156051
ENSG00000(	391.23922	532.05621	250.42224	0.4706688	-1.087216	0.0124365	0.1606415
ENSG000001	52.899436	10.418903	95.379969	9.154512	3.194483	0.0018417	0.0386339
ENSG000001	677.19127	424.94373	929.43881	2.1872044	1.1290881	0.006008	0.095469
ENSG000001	48.471264	2.2888488	94.653679	41.354274	5.3699645	3.04E-05	0.001309
ENSG000001	3589.0798	2101.479	5076.6807	2.4157655	1.2724804	7.31E-05	0.0028303
ENSG000001	73.321219	17.37323	129.26921	7.4407127	2.8954408	0.0167242	0.1991084
ENSG000001	827.98739	523.63764	1132.3371	2.1624441	1.1126629	0.0020285	0.0418709
ENSG000001	701.6309	497.70837	905.55343	1.8194458	0.8634991	0.0389621	0.341819
ENSG000001	22.64904	6.6283798	38.669701	5.8339597	2.5444754	0.0239315	0.2517086
ENSG000001	983.91229	459.04115	1508.7834	3.2868152	1.7166904	0.0001215	0.0043204
ENSG000001	171.6372	296.01627	47.258121	0.159647	-2.647042	2.06E-05	0.0009443
ENSG000001	1322.85	1641.9384	1003.7617	0.6113272	-0.709983	0.0285291	0.2817613
ENSG000001	85.215657	38.599587	131.83173	3.4153662	1.7720403	0.0251924	0.260402
ENSG000001	163.07375	104.49987	221.64762	2.1210326	1.0847668	0.0178919	0.2080974

ENSG000001	1235.0892	516.02638	1954.152	3.7869227	1.921026	3.82E-08	3.38E-06
ENSG000001	571.70583	409.75428	733.65739	1.7904813	0.8403475	0.0321486	0.3042927
ENSG00000(	2233.8968	1323.823	3143.9707	2.3749177	1.2478775	0.0019548	0.0407234
ENSG000001	432.43641	230.07571	634.79711	2.7590792	1.4641869	0.0001401	0.0048611
ENSG000001	142.03615	228.5215	55.550809	0.2430879	-2.04045	0.0009835	0.0237362
ENSG000001	695.3728	148.16859	1242.577	8.3862376	3.0680237	2.46E-10	3.09E-08
ENSG000001	142.5863	76.712771	208.45983	2.7174071	1.4422307	0.0018471	0.0386879
ENSG000002	129.85647	197.03669	62.676237	0.3180942	-1.652474	0.008971	0.1273013
ENSG000001	38.820053	73.341011	4.2990944	0.0586179	-4.092515	0.0002514	0.0077576
ENSG000001	388.58426	634.94145	142.22707	0.2240003	-2.158427	0.0001231	0.0043528
ENSG000001	483.56833	223.59268	743.54399	3.3254398	1.7335451	5.60E-06	0.0003023
ENSG000002	19.10523	3.3141899	34.89627	10.529351	3.3963447	0.0186047	0.2133315
ENSG000001	10549.922	7569.3166	13530.527	1.7875494	0.8379831	0.035062	0.3217228
ENSG000002	515.93437	285.8456	746.02314	2.6098814	1.3839843	0.0052288	0.0854718
ENSG000001	289.35018	211.22094	367.47941	1.7397868	0.7989105	0.0473783	0.3868387
ENSG000001	1345.1547	403.3065	2287.0029	5.6706324	2.5035096	1.23E-08	1.19E-06
ENSG000001	177.67067	341.13413	14.207203	0.041647	-4.585645	8.68E-07	5.78E-05
ENSG000002	152.53184	1.9729719	303.09072	153.62141	7.2632355	3.57E-15	8.62E-13
ENSG000001	310.08384	127.73444	492.43325	3.8551328	1.9467806	0.001155	0.0267598
ENSG00000(	50.122494	86.194113	14.050874	0.1630143	-2.616929	0.0192983	0.2175058
ENSG000001	47.690336	83.754879	11.625793	0.1388073	-2.848844	0.003121	0.058488
ENSG00000(	1454.3364	1928.8194	979.85349	0.5080069	-0.97708	0.0037453	0.0672539
ENSG000001	290.59353	396.87382	184.31325	0.4644127	-1.106521	0.0363951	0.3294811
ENSG000001	8.565014	17.130028	0	0	#NAME?	0.0241763	0.2535798
ENSG000001	888.83223	1206.3448	571.31965	0.4735957	-1.078272	0.0074027	0.1108604
ENSG000002	93.003154	139.42692	46.579385	0.3340774	-1.581746	0.036947	0.3323883
ENSG000002	489.57995	111.85282	867.30709	7.7540031	2.9549413	7.09E-07	4.82E-05
ENSG000001	15.598221	0	31.196442	Inf	Inf	0.0103415	0.1406287
ENSG000001	31.695948	1.5793845	61.812511	39.137088	5.2904645	0.0032196	0.0600034
ENSG000001	331.01954	459.88589	202.15319	0.4395725	-1.185827	0.0234844	0.2486857
ENSG00000(	1045.945	1392.2431	699.64699	0.5025322	-0.992712	0.0046494	0.0788817
ENSG000001	311.46094	192.51153	430.41034	2.2357639	1.1607679	0.0051559	0.0845593
ENSG000001	63.377345	114.77846	11.976235	0.1043422	-3.260605	0.0003665	0.010697
ENSG000001	1211.9564	1460.0839	963.82895	0.6601189	-0.599202	0.0499935	0.4010082
ENSG00000(	943.25541	1214.4497	672.06114	0.5533874	-0.853638	0.0181079	0.2096746
ENSG000001	143.38541	245.40833	41.362498	0.1685456	-2.568789	0.0001464	0.0050301
ENSG000001	34.807171	2.8428921	66.771449	23.487156	4.5538001	0.0068399	0.1049066
ENSG000001	248.21087	125.96943	370.45231	2.9408112	1.5562142	0.000582	0.0156558
ENSG000001	114.22054	172.87463	55.566445	0.3214263	-1.63744	0.0165902	0.1979487
ENSG000001	2828.625	3557.4003	2099.8496	0.5902765	-0.760537	0.0249791	0.259088
ENSG000001	12.302901	0	24.605802	Inf	Inf	0.0260997	0.2665218
ENSG000001	22.396211	43.24996	1.5424628	0.0356639	-4.809391	0.0282181	0.2798131
ENSG000001	99.576372	190.3155	8.837244	0.0464347	-4.428653	2.99E-08	2.70E-06
ENSG000001	23.523717	2.2111383	44.836296	20.277472	4.3418059	0.0378635	0.3367147
ENSG00000(	90.582924	41.038822	140.12703	3.4144993	1.771674	0.0023813	0.0473125
ENSG000002	65.579158	26.440844	104.71747	3.9604436	1.985662	0.0109137	0.1464227
ENSG000001	1184.8305	3.7905228	2365.8704	624.15412	9.2857585	9.27E-42	1.57E-38
ENSG00000(	99.351244	58.562437	140.14005	2.3930024	1.2588218	0.0224275	0.2407917
ENSG000001	112.66801	52.731302	172.60473	3.2732878	1.7107404	0.0225536	0.2418081
ENSG000001	412.82527	152.98949	672.66105	4.3967795	2.1364472	5.70E-05	0.0022889
ENSG000001	277.98541	356.6596	199.31122	0.5588276	-0.839525	0.0470729	0.3855611

ENSG000002	137.43018	230.26637	44.593984	0.1936626	-2.368383	0.0002147	0.0068426
ENSG000002	9.6317279	19.263456	0	0	#NAME?	0.0118367	0.1549498
ENSG000001	526.81301	396.87097	656.75505	1.6548327	0.7266854	0.0332107	0.31123
ENSG000002	18.468729	36.937457	0	0	#NAME?	0.0008289	0.0206742
ENSG000001	4690.7533	787.51939	8593.9872	10.912731	3.4479403	5.66E-20	2.40E-17
ENSG000001	2136.6966	2853.1321	1420.2612	0.4977902	-1.00639	0.0181929	0.2100335
ENSG000002	78.916296	119.43386	38.398729	0.3215062	-1.637081	0.0364047	0.3294811
ENSG000000	2797.7874	3747	1848.5748	0.493348	-1.019323	0.0459869	0.3798687
ENSG000001	2183.9862	1613.3628	2754.6095	1.7073714	0.771777	0.0112243	0.1490382
ENSG000002	1110.1157	1565.2357	654.99569	0.4184646	-1.256823	0.0004561	0.0127634
ENSG000001	1631.9919	952.89421	2311.0895	2.425337	1.2781852	0.0105937	0.1431233
ENSG000001	65.505609	20.837805	110.17341	5.2871889	2.4025009	0.0052335	0.0854718
ENSG000002	238.66942	108.46092	368.87793	3.4010217	1.7659682	0.0017322	0.0368518
ENSG000001	1443.1156	972.36562	1913.8656	1.9682572	0.9769188	0.0448524	0.3749509
ENSG000002	78.105484	25.265117	130.94585	5.1828714	2.3737516	0.0263206	0.2679777
ENSG000001	926.90456	1.2635076	1852.5456	1466.1927	10.517859	9.44E-08	7.60E-06
ENSG000001	777.09912	1016.8114	537.3868	0.5285019	-0.920019	0.0087257	0.1249341
ENSG000001	12.195081	24.390162	0	0	#NAME?	0.0067247	0.1039868
ENSG000001	130.70381	0.9476307	260.45998	274.85389	8.1025211	7.58E-13	1.32E-10
ENSG000001	1726.8367	2325.0413	1128.6321	0.4854245	-1.042681	0.0019833	0.0411574
ENSG000001	247.35763	3.158769	491.5565	155.61647	7.281851	1.84E-17	6.23E-15
ENSG000001	420.28479	778.50498	62.06459	0.0797228	-3.648864	3.15E-14	7.11E-12
ENSG000001	281.43321	118.85464	444.01177	3.7357544	1.9013996	2.82E-06	0.000164
ENSG000001	182.48958	322.38947	42.589691	0.1321063	-2.920228	2.92E-07	2.12E-05
ENSG000001	1147.2641	407.14167	1887.3865	4.6356996	2.2127871	1.28E-11	1.99E-09
ENSG000001	1782.7507	2503.0376	1062.4638	0.4244698	-1.236266	0.0003251	0.0096791
ENSG000000	188.26456	89.83425	286.69487	3.1913761	1.6741786	0.0083163	0.1204745
ENSG000000	554.37055	685.54217	423.19893	0.6173201	-0.695909	0.0477581	0.3883676
ENSG000001	556.81664	754.40552	359.22777	0.4761733	-1.070441	0.0221661	0.2387891
ENSG000001	1572.7258	2176.0352	969.41646	0.4454967	-1.166513	0.0009432	0.0229324
ENSG000000	728.15613	249.70758	1206.6047	4.8320708	2.2726416	7.07E-08	5.87E-06
ENSG000001	175.95089	281.21034	70.69144	0.2513828	-1.992042	0.0012408	0.0283108
ENSG000001	895.91836	646.32024	1145.5165	1.7723667	0.8256772	0.0163725	0.1959234
ENSG000001	37.003417	64.341037	9.6657972	0.1502276	-2.734779	0.0086711	0.1244117
ENSG000001	49.603732	0	99.207465	Inf	Inf	5.15E-07	3.63E-05
ENSG000002	209.24075	361.45532	57.026181	0.1577683	-2.664121	2.20E-06	0.0001331
ENSG000002	164.03763	328.07526	0	0	#NAME?	9.31E-25	6.46E-22
ENSG000002	112.93993	224.33739	1.5424628	0.0068756	-7.184291	4.02E-15	9.54E-13
ENSG000001	294.24572	404.66282	183.82862	0.454276	-1.138359	0.0111284	0.1482743
ENSG000002	107.52262	23.991539	191.0537	7.9633783	2.9933806	7.05E-06	0.0003699
ENSG000001	6150.4446	9978.9621	2321.9272	0.2326822	-2.103567	4.51E-08	3.95E-06
ENSG000001	136.43629	13.339505	259.53307	19.455974	4.2821413	2.41E-08	2.18E-06
ENSG000002	23.631818	43.021864	4.2417726	0.0985957	-3.342331	0.0143152	0.177174
ENSG000001	1524.3974	1974.4309	1074.3639	0.5441385	-0.877954	0.0091143	0.1287278
ENSG000001	600.16803	429.26094	771.07513	1.7962854	0.8450166	0.0455847	0.3785114
ENSG000001	1176.4124	543.80626	1809.0185	3.3265864	1.7340425	3.14E-05	0.0013477
ENSG000001	4582.9922	2831.9001	6334.0843	2.2366906	1.1613657	0.0058717	0.0938538
ENSG000001	33.91046	12.075998	55.744923	4.6161754	2.206698	0.0499974	0.4010082
ENSG000001	1275.1198	1660.3218	889.91773	0.5359911	-0.899719	0.0072294	0.1093028
ENSG000001	2618.9896	3145.8666	2092.1126	0.6650354	-0.588497	0.0359372	0.3268641
ENSG000001	1072.4982	1867.0371	277.95925	0.1488772	-2.747805	2.03E-08	1.88E-06

ENSG000001	82.173191	25.255047	139.09133	5.5074669	2.4613889	0.00048	0.0133371
ENSG000001	349.63804	222.0284	477.24767	2.1494893	1.1039939	0.003981	0.0703664
ENSG000001	2946.1308	3778.5279	2113.7338	0.5594067	-0.838031	0.0473951	0.3868387
ENSG000001	1412.3443	1851.0662	973.62242	0.5259793	-0.926922	0.0060918	0.0964592
ENSG000002	7.3015064	14.603013	0	0	#NAME?	0.0481057	0.3904584
ENSG000001	561.50366	169.61801	953.38931	5.6208024	2.4907761	6.31E-09	6.37E-07
ENSG000001	1031.1079	767.54932	1294.6664	1.6867533	0.754249	0.0219892	0.2373828
ENSG000001	268.73969	119.84256	417.63683	3.4848792	1.8011086	0.0046895	0.0794133
ENSG000001	1458.9165	1818.0869	1099.7461	0.6048919	-0.725251	0.0455175	0.3780688
ENSG000001	492.07712	272.14994	712.0043	2.6162207	1.3874842	0.0115098	0.1520839
ENSG000002	44.140041	13.417216	74.862867	5.5796127	2.480165	0.0101565	0.139091
ENSG000001	53.836871	107.67374	0	0	#NAME?	2.75E-10	3.38E-08
ENSG000001	1015.9286	689.09168	1342.7654	1.948602	0.9624395	0.0057888	0.0928568
ENSG000001	13.891031	27.782062	0	0	#NAME?	0.0047578	0.0801694
ENSG000001	997.41618	1325.9636	668.86873	0.5044397	-0.987246	0.0276768	0.2754613
ENSG000001	441.24721	168.29693	714.19748	4.243675	2.0853142	2.07E-05	0.0009478
ENSG000002	7.814177	15.628354	0	0	#NAME?	0.0436661	0.3672
ENSG000001	4795.2575	5946.6711	3643.844	0.6127536	-0.706621	0.034566	0.3193548
ENSG000002	100.49072	14.214461	186.76699	13.139225	3.7158082	0.0003306	0.0097996
ENSG000001	152.84801	260.98414	44.711884	0.1713203	-2.545232	2.10E-05	0.0009577
ENSG000001	588.21211	774.23091	402.19331	0.5194746	-0.944875	0.0273696	0.2738106
ENSG000000	1600.1021	1187.6743	2012.5299	1.6945132	0.7608709	0.0448188	0.3747963
ENSG000000	994.65443	1844.0672	145.24165	0.0787616	-3.666364	2.95E-05	0.0012755
ENSG000002	36.690491	0	73.380982	Inf	Inf	1.79E-05	0.0008377
ENSG000001	134.56874	52.809013	216.32847	4.0964309	2.0343675	0.0028955	0.0551413
ENSG000001	17.244076	34.488152	0	0	#NAME?	0.0178773	0.2080974
ENSG000001	3006.8154	3833.7926	2179.8382	0.5685853	-0.814551	0.0091898	0.1293881
ENSG000001	331.65466	437.79968	225.50965	0.5150978	-0.957082	0.047762	0.3883676
ENSG000000	992.46309	724.79584	1260.1303	1.7386004	0.7979264	0.03515	0.322329
ENSG000001	89.051188	146.50143	31.600951	0.215704	-2.212875	0.0097811	0.1354591
ENSG000002	150.00601	88.716093	211.29593	2.3817092	1.2519973	0.0131799	0.1671324
ENSG000000	108566.97	166717.64	50416.312	0.3024054	-1.725444	2.74E-07	1.99E-05
ENSG000001	1368.4381	1888.0389	848.83729	0.4495868	-1.153329	0.0008896	0.0218659
ENSG000001	159.29904	247.76985	70.828232	0.285863	-1.806604	0.0111191	0.148224
ENSG000001	7176.2182	2451.3036	11901.133	4.8550219	2.2794778	5.92E-09	6.02E-07
ENSG000002	14.048969	28.097939	0	0	#NAME?	0.0043442	0.0751312
ENSG000001	796.28358	1078.1642	514.40293	0.47711	-1.067606	0.0115933	0.1528878
ENSG000000	625.5608	803.48947	447.63212	0.5571101	-0.843966	0.0189906	0.215474
ENSG000001	714.62885	1426.1441	3.1135864	0.0021832	-8.839327	9.74E-49	2.93E-45
ENSG000001	223.33119	148.55211	298.11027	2.0067724	1.004877	0.0342731	0.3177338
ENSG000001	382.474	515.10678	249.84122	0.485028	-1.04386	0.0137099	0.1718617
ENSG000001	253.34288	337.63431	169.05146	0.500694	-0.997999	0.0294526	0.2877316
ENSG000002	80.183008	36.782036	123.58398	3.3599005	1.7484185	0.0188143	0.2143716
ENSG000001	89.696702	171.27007	8.1233344	0.04743	-4.398057	0.008674	0.1244117
ENSG000002	69.001998	23.996574	114.00742	4.7509874	2.2482274	0.0120879	0.1577193
ENSG000000	1203.6457	773.03437	1634.2571	2.1140808	1.0800305	0.0070743	0.10761
ENSG000001	219.57435	135.28024	303.86846	2.2462146	1.1674958	0.0041187	0.072327
ENSG000001	166.61005	5.6857842	327.53432	57.605831	5.8481429	8.25E-11	1.11E-08
ENSG000001	417.94505	256.06539	579.8247	2.2643618	1.1791045	0.0082573	0.119749
ENSG000001	286.66726	377.67297	195.66155	0.5180714	-0.948777	0.0230751	0.2458411
ENSG000002	10.618214	21.236428	0	0	#NAME?	0.0081193	0.1187018

ENSG00000115.856467	1.9729719	29.739962	15.073688	3.9139605	0.0413371	0.3531012
ENSG00000117.787504	0	35.575007	Inf	Inf	0.0046453	0.0788817
ENSG0000043.278717	8.6063868	77.951048	9.057349	3.1790888	0.0237717	0.2505011
ENSG000001406.2776	936.82913	1875.7261	2.0022073	1.0015914	0.0085547	0.1232037
ENSG00000235.53534	159.12643	311.94424	1.9603547	0.9711147	0.0268275	0.2709884
ENSG00000294.10881	455.88238	132.33524	0.2902837	-1.784464	0.0002226	0.0070366
ENSG00000104.48131	44.124915	164.8377	3.7357058	1.9013809	0.0074239	0.1109938
ENSG00000	15.04049	30.080981	0	0	#NAME?	0.0008247	0.0206056
ENSG00000593.05805	20.847875	1165.2682	55.89386	5.8046179	1.43E-19	5.95E-17
ENSG000002434.7922	1403.4866	3466.0979	2.4696338	1.3042971	0.0014653	0.0323968
ENSG0000028.522087	1.8952614	55.148913	29.098315	4.8628637	0.0098583	0.136249
ENSG00000668.21371	492.04491	844.38251	1.716068	0.7791067	0.0093358	0.130968
ENSG00000	422.7196	246.60923	598.82997	2.4282545	1.2799197	0.0099024	0.1366755
ENSG0000098.951535	171.95217	25.950895	0.1509193	-2.728151	0.0023629	0.0470839
ENSG00000369.27423	183.37124	555.17722	3.0276133	1.598181	0.0013246	0.0299457
ENSG000002267.9834	2988.3066	1547.6603	0.5179054	-0.949239	0.0034788	0.0635322
ENSG00000203.54207	105.36979	301.71435	2.8633857	1.517722	0.0015219	0.0334554
ENSG0000085.883203	0	171.76641	Inf	Inf	6.39E-11	8.78E-09
ENSG0000012.195081	24.390162	0	0	#NAME?	0.0067247	0.1039868
ENSG00000361.70148	665.78728	57.615679	0.0865377	-3.530528	6.55E-13	1.16E-10
ENSG000006530.8276	7954.1891	5107.4661	0.6421102	-0.639107	0.0467093	0.3836504
ENSG00000649.05577	384.69208	913.41946	2.3744171	1.2475734	0.0001776	0.0058554
ENSG00000359.51085	240.24134	478.78037	1.9929142	0.9948796	0.0091655	0.1291805
ENSG000001065.6661	737.5964	1393.7358	1.8895642	0.9180536	0.0195672	0.2195308
ENSG000002060.4438	3206.5524	914.33528	0.2851459	-1.810228	1.70E-06	0.0001062
ENSG00000362.97429	195.77319	530.17539	2.7081103	1.4372865	0.0062948	0.0986942
ENSG00000792.72535	1024.5954	560.85529	0.547392	-0.869354	0.0173998	0.205166
ENSG00000130.61137	0	261.22273	Inf	Inf	7.07E-08	5.87E-06
ENSG00000710.82953	476.60219	945.05687	1.982905	0.9876156	0.0084386	0.1218551
ENSG000001310.3095	1847.752	772.8671	0.4182743	-1.257479	0.0008029	0.0201742
ENSG000003027.2572	4206.1806	1848.3337	0.4394328	-1.186286	0.000565	0.015262
ENSG0000055.230333	0	110.46067	Inf	Inf	3.11E-07	2.25E-05
ENSG000004390.8116	5977.789	2803.8342	0.469042	-1.092211	0.0007747	0.0195926
ENSG000002564.0797	3776.2283	1351.931	0.3580109	-1.481924	0.0128747	0.1641861
ENSG00000813.30528	995.48724	631.12333	0.6339844	-0.657481	0.0405559	0.3488504
ENSG00000	495.5719	263.41331	727.73049	2.7626945	1.466076	0.0014049	0.0313835
ENSG0000064.639488	0	129.27898	Inf	Inf	1.11E-08	1.08E-06
ENSG0000035.850837	5.3699073	66.331768	12.352498	3.6267309	0.0296025	0.2888832
ENSG00000	28.20905	55.64687	0.7712314	0.0138594	-6.172993	0.0001511	0.0051226
ENSG00000495.64829	907.20434	84.092238	0.0926938	-3.431383	1.26E-12	2.15E-10
ENSG000001117.9063	1387.8006	848.012	0.6110474	-0.710644	0.0422714	0.3591545
ENSG00000408.11148	761.69872	54.524242	0.0715824	-3.804251	8.89E-05	0.0033519
ENSG00000	116.1871	29.516867	202.85733	6.87259	2.7808539	1.33E-05	0.0006515
ENSG0000025.218709	50.437418	0	0	#NAME?	3.77E-05	0.0015759
ENSG000001983.9601	2853.5193	1114.4008	0.3905356	-1.356474	0.0004575	0.012789
ENSG00000	510.9287	694.89042	326.96697	0.4705303	-1.087641	0.020465	0.2269043
ENSG00000353.24563	206.95409	499.53717	2.4137584	1.2712813	0.001805	0.0380411
ENSG0000025.294307	0	50.588614	Inf	Inf	0.0005451	0.0148548
ENSG00000126.08941	67.489736	184.68909	2.7365508	1.4523587	0.0242741	0.2542106
ENSG00000283.99239	466.66751	101.31728	0.2171081	-2.203515	0.0040726	0.0718448
ENSG00000973.53935	1396.3315	550.7472	0.3944244	-1.342179	0.0010345	0.0245384

ENSG000001	122.4174	208.2176	36.617211	0.1758603	-2.507498	0.0114049	0.1511398
ENSG000001	114.10415	165.1432	63.065109	0.3818814	-1.388804	0.0344712	0.3189141
ENSG000002	50.293624	81.548775	19.038473	0.2334612	-2.098745	0.0154415	0.1879722
ENSG000002	175.98239	132.12147	219.84331	1.6639484	0.7346107	0.0471785	0.3860593
ENSG000001	835.99616	1286.0581	385.93426	0.3000908	-1.736529	3.27E-05	0.0014005
ENSG000001	769.66061	473.08004	1066.2412	2.2538283	1.1723776	0.0074213	0.1109938
ENSG000000	285.90561	120.92547	450.88576	3.7286253	1.8986438	0.0001341	0.0046886
ENSG000001	1469.7308	2131.6045	807.85715	0.3789902	-1.399768	9.69E-05	0.0036102
ENSG000001	476.54558	309.89975	643.19142	2.0754822	1.0534466	0.0113963	0.1511001
ENSG000001	914.17535	481.0295	1347.3212	2.8009118	1.4858965	0.0001333	0.0046653
ENSG000001	674.59163	882.3407	466.84256	0.5290956	-0.9184	0.0142085	0.1763747
ENSG000001	59.590861	1.8952614	117.28646	61.884055	5.9514958	1.37E-05	0.0006661
ENSG000000	517.36237	326.14475	708.57999	2.1725936	1.1194183	0.0027852	0.0535684
ENSG000000	637.46088	395.08866	879.83311	2.2269257	1.1550535	0.0090596	0.1282225
ENSG000002	2015.348	1201.942	2828.7539	2.3534862	1.2347994	0.0005421	0.0147917
ENSG000001	10.5533	0	21.1066	Inf	Inf	0.0451359	0.3763531
ENSG000001	841.83311	458.32665	1225.3396	2.6735072	1.4187336	0.0015745	0.0343328
ENSG000001	1419.6032	1728.6239	1110.5824	0.6424662	-0.638308	0.0424095	0.3596499
ENSG000001	17.717892	35.435783	0	0	#NAME?	0.0100645	0.1381111
ENSG000000	145.52647	207.55345	83.499484	0.4023035	-1.313644	0.0134331	0.1693284
ENSG000001	2592.188	3160.4444	2023.9315	0.6403946	-0.642967	0.0305936	0.2949644
ENSG000001	501.91332	706.9592	296.86744	0.4199216	-1.251808	0.0027643	0.0532049
ENSG000001	519.25518	698.0794	340.43096	0.487668	-1.036029	0.0158343	0.1917585
ENSG000002	42.691311	0	85.382622	Inf	Inf	3.09E-06	0.0001775
ENSG000001	255.16234	117.93722	392.38746	3.3270875	1.7342598	0.0026096	0.0506579
ENSG000000	918.05325	651.39726	1184.7092	1.81872	0.8629235	0.033275	0.3113967
ENSG000001	149.6999	253.41317	45.986631	0.181469	-2.462205	2.51E-05	0.0011172
ENSG000001	11.48799	21.790471	1.185508	0.0544049	-4.20012	0.0446633	0.3737269
ENSG000001	1182.0304	807.50523	1556.5555	1.9276104	0.9468135	0.008962	0.1273013
ENSG000001	60.60366	99.476048	21.731271	0.2184573	-2.194577	0.0064238	0.1002498
ENSG000000	77.413919	15.711099	139.11674	8.8546788	3.14644	0.0019307	0.0402826
ENSG000001	493.38504	975.82953	10.940544	0.0112115	-6.478873	3.92E-10	4.73E-08
ENSG000001	234.35185	446.52624	22.177465	0.0496667	-4.331579	2.61E-10	3.25E-08
ENSG000002	31.711445	6.7888358	56.634054	8.3422335	3.0604337	0.0319962	0.3033808
ENSG000000	119.92534	12.635076	227.21559	17.982923	4.1685556	0.0169349	0.2004681
ENSG000001	13.652865	27.305729	0	0	#NAME?	0.0425939	0.3607621
ENSG000001	577.50524	257.6045	897.40598	3.483658	1.800603	3.36E-07	2.42E-05
ENSG000002	22.779665	0	45.55933	Inf	Inf	0.0474024	0.3868387
ENSG000000	455.36961	648.42412	262.3151	0.4045425	-1.305637	0.0022883	0.045971
ENSG000001	167.49088	105.68567	229.2961	2.1696046	1.1174321	0.0187967	0.214352
ENSG000001	215.39918	62.057223	368.74114	5.9419535	2.5709373	0.0003575	0.010481
ENSG000002	58.244327	96.710867	19.777788	0.2045043	-2.289797	0.0042091	0.073427
ENSG000001	437.36473	300.95734	573.77211	1.9064898	0.9309188	0.0072475	0.109346
ENSG000001	450.5205	79.405277	821.63573	10.347369	3.3711921	7.52E-10	8.48E-08
ENSG000001	36.546289	63.305626	9.7869528	0.1545985	-2.693402	0.0192907	0.2175058
ENSG000001	1350.0332	643.47012	2056.5962	3.1961021	1.6763135	8.12E-06	0.0004226
ENSG000002	13.652556	0	27.305112	Inf	Inf	0.0173159	0.2042656
ENSG000001	218.57709	329.82013	107.33406	0.3254321	-1.619572	0.0015801	0.0344132
ENSG000001	45.585057	0	91.170113	Inf	Inf	1.49E-06	9.42E-05
ENSG000000	1100.1129	1425.6203	774.60561	0.5433464	-0.880056	0.034154	0.3172818
ENSG000001	825.33781	594.92293	1055.7527	1.7746041	0.8274972	0.0179153	0.2080974

ENSG000001	1160.8625	804.16872	1517.5562	1.8871117	0.9161798	0.0345446	0.3192662
ENSG000001	2563.8378	1866.0067	3261.6688	1.7479406	0.8056561	0.0102526	0.1399829
ENSG000001	1602.2087	2200.6909	1003.7265	0.4560961	-1.13259	0.0037595	0.0674615
ENSG000001	987.12924	418.14264	1556.1158	3.7214952	1.8958824	1.18E-05	0.0005848
ENSG000001	371.54252	480.69634	262.38871	0.5458513	-0.87342	0.0208276	0.229672
ENSG000001	65.455968	22.650321	108.26162	4.7796945	2.2569184	0.002534	0.0494752
ENSG000001	1210.1212	1526.9771	893.26518	0.5849892	-0.773518	0.0412178	0.3523867
ENSG000001	1046.6927	1790.52	302.86533	0.1691494	-2.56363	6.93E-10	7.87E-08
ENSG000001	333.67594	198.31027	469.04161	2.3651907	1.2419565	0.0402555	0.3474812
ENSG000002	36.536945	8.5236412	64.55025	7.5730838	2.9208809	0.0081438	0.1187981
ENSG000001	279.51049	425.85393	133.16705	0.3127059	-1.677122	0.0023158	0.0464206
ENSG000001	234.26045	354.22036	114.30054	0.322682	-1.631815	0.0008641	0.0213553
ENSG000001	85.475116	0	170.95023	Inf	Inf	5.98E-11	8.26E-09
ENSG000001	166.92018	65.511729	268.32863	4.0958868	2.0341758	0.0003076	0.0092605
ENSG000001	1322.6155	969.50192	1675.729	1.7284432	0.7894732	0.0073495	0.1103516
ENSG000001	162.59273	99.611329	225.57412	2.2645429	1.1792199	0.0377522	0.3361675
ENSG000000	2330.8953	3474.7586	1187.0321	0.3416157	-1.549554	1.43E-05	0.000688
ENSG000001	975.13543	260.04155	1690.2293	6.4998433	2.7004049	1.73E-08	1.64E-06
ENSG000001	382.31637	200.92507	563.70767	2.8055617	1.4882896	0.0163523	0.1958109
ENSG000001	531.97857	313.2089	750.74825	2.3969569	1.261204	0.0016939	0.0361211
ENSG000001	260.44002	190.12987	330.75017	1.7396013	0.7987567	0.0219917	0.2373828
ENSG000002	616.56923	447.75451	785.38396	1.7540504	0.8106902	0.0102217	0.139702
ENSG000001	7272.9378	12295.659	2250.2163	0.183009	-2.450014	0.0017694	0.0375246
ENSG000001	569.51473	370.06675	768.96271	2.0779028	1.0551281	0.0235225	0.2489415
ENSG000001	377.80822	210.9101	544.70634	2.582647	1.3688505	0.0126702	0.1624289
ENSG000001	620.43787	875.5094	365.36634	0.4173186	-1.260779	0.003195	0.0596282
ENSG000001	39.746583	7.5810456	71.912121	9.4857787	3.2457662	0.0458071	0.3791447
ENSG000001	1745.366	2164.5161	1326.2159	0.6127078	-0.706729	0.0204336	0.2268068
ENSG000001	526.98148	723.99641	329.96656	0.4557572	-1.133663	0.0026073	0.0506502
ENSG000001	587.59101	343.21217	831.96985	2.4240686	1.2774305	0.002388	0.0473519
ENSG000001	121.9011	13.25676	230.54544	17.390784	4.1202511	6.53E-10	7.55E-08
ENSG000001	638.33695	388.23218	888.44171	2.2884288	1.1943574	0.0126827	0.1624289
ENSG000002	514.79967	319.33359	710.26575	2.2242125	1.1532946	0.0375211	0.3354343
ENSG000001	170.81289	75.614755	266.01103	3.5179779	1.8147464	0.0348016	0.3208002
ENSG000001	3259.9887	2211.5611	4308.4162	1.9481334	0.9620925	0.020248	0.2253145
ENSG000001	446.13666	185.89826	706.37507	3.7997939	1.9259212	7.41E-05	0.0028617
ENSG000000	1178.4648	112.63999	2244.2895	19.924447	4.3164678	0.0090909	0.1284639
ENSG000001	778.729	506.22194	1051.236	2.0766307	1.0542447	0.0087086	0.1247555
ENSG000001	61.548065	93.862939	29.233191	0.3114455	-1.682948	0.0401118	0.3468208
ENSG000001	394.69392	657.55937	131.82847	0.2004815	-2.318459	1.04E-06	6.76E-05
ENSG000002	33.627691	6.6283798	60.627003	9.1465795	3.1932323	0.0016579	0.0355789
ENSG000000	1550.9908	914.5249	2187.4567	2.391905	1.2581601	0.0024976	0.0489401
ENSG000001	285.88975	413.14618	158.63332	0.3839642	-1.380956	0.013433	0.1693284
ENSG000002	28.10826	0	56.216521	Inf	Inf	0.0002207	0.007005
ENSG000001	7952.1929	4234.3816	11670.004	2.7560115	1.4625819	6.50E-06	0.0003433
ENSG000001	106.55175	0	213.1035	Inf	Inf	6.61E-13	1.16E-10
ENSG000001	81.272913	135.14496	27.400863	0.2027516	-2.302215	0.0023047	0.0462316
ENSG000001	699.22726	259.79835	1138.6562	4.3828461	2.131868	9.70E-06	0.000496
ENSG000001	394.04756	246.52648	541.56864	2.196797	1.1354016	0.0211354	0.2316313
ENSG000001	77.903386	143.82402	11.982747	0.0833153	-3.585274	1.25E-05	0.0006169
ENSG000001	191.43289	268.73068	114.13509	0.4247192	-1.235419	0.008374	0.1211164

ENSG000001	811.43921	411.71215	1211.1663	2.9417793	1.556689	4.87E-05	0.0019845
ENSG000001	117.59701	66.697526	168.49649	2.526278	1.3370134	0.0261563	0.2668991
ENSG00000(	1830.5591	2451.544	1209.5743	0.4933929	-1.019191	0.0110272	0.1473619
ENSG000002	19.71468	36.315773	3.1135864	0.0857365	-3.543947	0.0158376	0.1917585
ENSG000002	120.61118	65.822571	175.39979	2.6647362	1.4139927	0.0152713	0.1862357
ENSG000001	60.301568	24.93917	95.663965	3.8358921	1.9395621	0.0090726	0.1283396
ENSG000001	200.80031	145.30556	256.29507	1.7638353	0.8187158	0.0317012	0.301535
ENSG000001	242.40817	353.11228	131.70406	0.3729807	-1.422827	0.0033578	0.0619404
ENSG000001	62.760397	14.209425	111.31137	7.833629	2.9696808	0.0040303	0.0711441
ENSG000001	152.54251	91.398529	213.68648	2.3379641	1.2252528	0.0117578	0.1543051
ENSG000002	193.69555	88.420357	298.97074	3.3812434	1.7575539	0.0125486	0.1614323
ENSG000001	18.553992	37.107983	0	0	#NAME?	0.0001662	0.0055251
ENSG000001	1144.8328	1760.4614	529.20417	0.3006054	-1.734057	0.000701	0.0181356
ENSG00000(	142.53939	282.35082	2.7279707	0.0096616	-6.693517	3.01E-16	8.39E-14
ENSG000001	325.37616	223.68549	427.06682	1.909229	0.9329901	0.010633	0.1434392
ENSG000001	243.63623	377.72047	109.552	0.2900346	-1.785703	0.0010035	0.0241158
ENSG00000(	1147.398	557.52928	1737.2666	3.1160096	1.6396997	4.87E-05	0.0019845
ENSG00000(	799.1819	212.44199	1385.9218	6.523766	2.705705	0.0004388	0.0123433
ENSG000001	331.74932	228.43875	435.05988	1.9044924	0.9294066	0.0376723	0.3359033
ENSG000001	190.0224	280.22242	99.822368	0.3562255	-1.489137	0.0189453	0.2152057
ENSG000001	381.49821	146.5115	616.48493	4.207758	2.0730517	5.28E-05	0.0021299
ENSG000001	239.72784	373.09023	106.36545	0.2850931	-1.810495	0.0001507	0.0051226
ENSG000002	303.4548	471.58844	135.32116	0.2869476	-1.801141	0.0002494	0.007729
ENSG000001	903.94048	1155.1951	652.68589	0.5650006	-0.823676	0.0376537	0.3359033
ENSG000002	11.565845	23.131689	0	0	#NAME?	0.0049434	0.0823011
ENSG000002	171.76551	224.8389	118.69213	0.5278986	-0.921667	0.0464221	0.3821113
ENSG000001	385.87013	223.24944	548.49083	2.4568519	1.2968109	0.0180152	0.2089622
ENSG000001	273.73104	108.86457	438.59751	4.0288359	2.0103631	0.0009695	0.0234248
ENSG000001	1002.7263	611.48885	1393.9638	2.2796226	1.188795	0.0018598	0.0388932
ENSG000001	210.56902	120.18579	300.95224	2.5040584	1.3242682	0.0139118	0.1734878
ENSG000001	360.48578	525.46307	195.50848	0.3720689	-1.426358	0.0014234	0.0317017
ENSG000002	108.82829	42.069198	175.58739	4.1737755	2.061353	0.0016614	0.0356243
ENSG000001	1370.363	840.43699	1900.289	2.2610725	1.1770072	0.0101076	0.1385619
ENSG000001	68.709655	12.391874	125.02744	10.089469	3.3347784	0.0002353	0.0073534
ENSG000001	156.34677	97.550576	215.14296	2.2054505	1.1410734	0.0081313	0.1187981
ENSG000002	66.27179	117.83937	14.704206	0.1247818	-3.002521	0.0275597	0.2748962
ENSG000001	205.95258	101.75483	310.15034	3.048016	1.6078705	0.0097255	0.1350337
ENSG000001	217.10222	371.77638	62.428057	0.1679183	-2.574169	4.25E-05	0.0017622
ENSG000001	619.7141	845.4781	393.9501	0.4659495	-1.101754	0.0166705	0.1985568
ENSG000001	497.93443	272.18015	723.6887	2.6588592	1.4108074	0.0023963	0.0474376
ENSG000001	566.87956	738.2483	395.51082	0.5357423	-0.900389	0.0296194	0.2889442
ENSG000001	2249.9739	3537.2483	962.69944	0.2721605	-1.87747	2.64E-06	0.000155
ENSG000001	1462.6425	506.97672	2418.3083	4.7700579	2.2540068	5.58E-07	3.88E-05
ENSG000001	4227.2714	5352.896	3101.6467	0.5794334	-0.787285	0.0425395	0.3604196
ENSG000002	1694.9455	973.37585	2416.5151	2.4826125	1.3118591	0.0037734	0.067579
ENSG000001	8.6840972	17.368194	0	0	#NAME?	0.0194876	0.2188778
ENSG000001	406.76601	545.18776	268.34426	0.4922052	-1.022668	0.0125367	0.1613971
ENSG00000(	36.117346	69.535383	2.6993098	0.0388192	-4.687085	0.0012604	0.0287103
ENSG000001	16.222147	30.309077	2.1352168	0.0704481	-3.827295	0.0467527	0.3838515
ENSG000001	170.06407	62.280284	277.84786	4.4612491	2.1574477	0.0004925	0.0135444
ENSG000001	161.66767	94.87821	228.45714	2.4078989	1.2677748	0.0357431	0.3257811

ENSG000001	423.72842	537.85495	309.60189	0.5756234	-0.796803	0.0302129	0.2927285
ENSG00000(	156.32866	243.11948	69.537849	0.2860234	-1.805795	0.0050397	0.083208
ENSG00000(	1246.4381	836.55366	1656.3225	1.9799358	0.9854536	0.0312808	0.2987966
ENSG000001	888.78481	1195.8885	581.68115	0.4864008	-1.039782	0.0247694	0.2580003
ENSG000001	1359.5568	1745.6684	973.44523	0.5576347	-0.842608	0.0247034	0.2575115
ENSG000001	469.42645	616.24496	322.60794	0.523506	-0.933722	0.0107287	0.1444723
ENSG000001	458.7604	268.77315	648.74765	2.4137369	1.2712684	0.0120224	0.157016
ENSG000001	578.6406	979.59051	177.69069	0.1813928	-2.462811	7.68E-09	7.66E-07
ENSG000002	223.57428	322.20384	124.94471	0.3877816	-1.366684	0.0367131	0.3316066
ENSG000001	1207.2393	1623.6002	790.87833	0.487114	-1.037669	0.0081512	0.1187981
ENSG000001	234.36096	444.94468	23.777249	0.0534387	-4.225972	2.09E-07	1.55E-05
ENSG000001	459.50887	317.56857	601.44917	1.893919	0.9213747	0.0392773	0.3434319
ENSG000002	27.047285	3.158769	50.935801	16.125206	4.0112457	0.0343124	0.3179889
ENSG000001	876.13012	1264.8368	487.4235	0.3853648	-1.375703	0.0007626	0.0193766
ENSG000001	366.80111	589.20475	144.39746	0.2450718	-2.028724	1.94E-05	0.0008983
ENSG000001	344.53694	113.17893	575.89495	5.0883583	2.3472003	1.82E-07	1.39E-05
ENSG000001	111.94447	58.489762	165.39918	2.8278314	1.4996961	0.0239433	0.251721
ENSG000001	1223.2944	1734.4529	712.13587	0.4105824	-1.284256	0.0069055	0.1057551
ENSG000001	517.63684	185.05351	850.22017	4.5944557	2.199894	4.94E-05	0.0019995
ENSG00000(	525.28718	689.60611	360.96824	0.5234412	-0.933901	0.0366552	0.331194
ENSG000001	191.83281	107.03192	276.63369	2.5845906	1.3699358	0.0075506	0.112577
ENSG000002	521.45296	748.72477	294.18116	0.3929096	-1.347731	0.0025247	0.0493285
ENSG000001	212.93536	149.63502	276.2357	1.8460632	0.8844519	0.0379641	0.3371662
ENSG00000(	845.37092	589.59331	1101.1485	1.8676408	0.901217	0.0295897	0.2888624
ENSG000001	1175.3995	2170.2084	180.59063	0.0832135	-3.587039	3.44E-17	1.14E-14
ENSG000001	510.95707	733.36479	288.54935	0.3934595	-1.345713	0.0014401	0.0319176
ENSG000002	264.99585	143.79885	386.19285	2.6856463	1.4252693	0.0024482	0.048218
ENSG000001	48.473918	0	96.947836	Inf	Inf	6.51E-07	4.46E-05
ENSG000001	734.31613	436.39301	1032.2393	2.3653891	1.2420775	0.000183	0.005996
ENSG000001	187.37534	82.26831	292.48237	3.5552252	1.8299409	0.0194369	0.2186131
ENSG000001	157.1453	44.601248	269.68936	6.0466773	2.5961426	0.000165	0.0055123
ENSG000001	122.51513	172.64653	72.383719	0.4192596	-1.254084	0.0324596	0.3062726
ENSG000001	268.55223	362.88432	174.22015	0.4800983	-1.058598	0.038808	0.3411188
ENSG000002	66.315004	12.319199	120.31081	9.7661226	3.2877859	0.0075913	0.1129345
ENSG000001	1585.599	2188.3645	982.83354	0.4491178	-1.154834	0.0008372	0.0208237
ENSG000002	41.286525	6.317538	76.255513	12.070448	3.5934073	0.0187688	0.2142175
ENSG000001	577.51064	431.05835	723.96293	1.679501	0.7480327	0.0381368	0.3378143
ENSG000001	340.97625	178.05891	503.89359	2.8299263	1.5007645	0.0001875	0.0061282
ENSG000001	5472.6268	2746.9876	8198.2661	2.9844569	1.5774684	6.64E-07	4.54E-05
ENSG000001	56.971933	2.8428921	111.10097	39.080264	5.2883683	0.0001035	0.0038053
ENSG000001	692.29389	883.00266	501.58512	0.5680449	-0.815923	0.0312166	0.2985247
ENSG000001	112.69398	156.62459	68.763361	0.439033	-1.187599	0.0421441	0.3584792
ENSG000001	109.71681	48.231315	171.20231	3.5496091	1.8276601	0.0138938	0.1734636
ENSG000001	607.00316	934.85179	279.15453	0.2986083	-1.743674	0.0252552	0.2609507
ENSG000001	263.11341	174.03525	352.19158	2.0236795	1.0169809	0.0080691	0.1181586
ENSG00000(	1144.5597	387.10899	1902.0105	4.913372	2.2967135	8.56E-08	6.97E-06
ENSG000001	105.59198	61.716171	149.46779	2.4218577	1.2761141	0.0076932	0.1141371
ENSG000001	120.03894	190.38817	49.689715	0.2609916	-1.937925	0.0106158	0.1433506
ENSG000001	106.85627	57.775262	155.93727	2.6990318	1.432442	0.0152411	0.1861185
ENSG000001	184.88591	61.332654	308.43917	5.0289552	2.3302587	0.0001435	0.0049468
ENSG00000(	61.021812	122.04362	0	0	#NAME?	1.50E-11	2.30E-09

ENSG000001	166.53625	238.93537	94.137139	0.3939858	-1.343784	0.0472123	0.3862188
ENSG000001	351.39681	135.53855	567.25507	4.1851936	2.0652944	9.36E-05	0.0035089
ENSG000001	2177.4115	2632.863	1721.9599	0.6540256	-0.612581	0.0365131	0.3303512
ENSG000001	8.6815797	17.363159	0	0	#NAME?	0.0409391	0.3511422
ENSG000001	644.91803	445.31024	844.52582	1.8964887	0.9233307	0.0023926	0.0473975
ENSG000002	1680.4088	2068.868	1291.9495	0.6244717	-0.679292	0.0353501	0.3235062
ENSG000002	102.2345	155.13299	49.336016	0.318024	-1.652792	0.0077645	0.1147252
ENSG000001	513.9622	645.68411	382.2403	0.5919927	-0.756349	0.0390785	0.3424558
ENSG000001	11.918913	0	23.837827	Inf	Inf	0.0305603	0.294933
ENSG000001	42.56286	76.173833	8.9518875	0.1175192	-3.089032	0.0065372	0.1015524
ENSG000000	692.72691	1050.9485	334.50535	0.318289	-1.651591	1.98E-05	0.0009174
ENSG000001	295.67742	164.72444	426.6304	2.5899643	1.3729322	0.001058	0.024961
ENSG000001	919.5246	1184.3486	654.70063	0.5527939	-0.855186	0.0354659	0.3241283
ENSG000000	85.200474	150.59776	19.803193	0.1314973	-2.926895	0.0087318	0.1249555
ENSG000001	144.42504	40.417138	248.43294	6.1467227	2.6198174	0.0002128	0.0068067
ENSG000001	717.4641	887.09396	547.83424	0.6175606	-0.695347	0.0370056	0.332455
ENSG000001	9466.0506	13039.478	5892.6227	0.4519063	-1.145904	0.0010341	0.0245384
ENSG000001	462.03413	261.12446	662.9438	2.5388039	1.344149	0.0032889	0.0609598
ENSG000002	55.446918	0	110.89384	Inf	Inf	1.15E-07	9.17E-06
ENSG000001	107.86152	154.64658	61.076452	0.3949421	-1.340287	0.0412275	0.3523867
ENSG000000	489.00738	335.08715	642.9276	1.9186877	0.9401199	0.0320527	0.3037711
ENSG000001	303.53523	209.96247	397.10799	1.8913284	0.9193999	0.0361111	0.3277006
ENSG000001	527.0143	683.5066	370.52201	0.5420899	-0.883396	0.0158622	0.1919706
ENSG000001	429.18528	183.08054	675.29001	3.688486	1.8830288	0.001083	0.0254183
ENSG000001	1036.5465	770.65052	1302.4425	1.690056	0.7570711	0.036917	0.3323387
ENSG000002	753.2476	497.33493	1009.1603	2.0291362	1.0208657	0.0338462	0.315378
ENSG000001	731.09519	1106.4644	355.72595	0.3214979	-1.637119	0.0263314	0.2679777
ENSG000001	89.050376	135.22771	42.873044	0.3170433	-1.657248	0.0190456	0.2157357
ENSG000001	187.64896	75.138422	300.15951	3.994754	1.9981066	0.0001222	0.0043389
ENSG000000	257.87546	157.60965	358.14127	2.2723308	1.1841729	0.0012083	0.027662
ENSG000000	494.5908	659.65537	329.52623	0.499543	-1.001319	0.0095647	0.1334178
ENSG000000	912.19288	1309.792	514.59379	0.3928821	-1.347832	0.0052207	0.0854667
ENSG000001	39.524383	69.322392	9.726375	0.1403064	-2.833347	0.0335999	0.3136598
ENSG000001	50.691839	6.6283798	94.755298	14.295393	3.8374784	3.53E-05	0.001495
ENSG000001	263.7866	192.11291	335.46029	1.7461621	0.8041875	0.0278555	0.2769276
ENSG000001	24.865344	9.9425698	39.788119	4.0017943	2.000647	0.0284262	0.2810532
ENSG000001	59.086073	111.67726	6.494889	0.0581577	-4.103887	0.0387995	0.3411188
ENSG000001	2387.1064	3750.958	1023.2548	0.2727983	-1.874094	0.0001399	0.0048611
ENSG000001	1146.3297	892.31281	1400.3466	1.5693449	0.6501625	0.0200941	0.224234
ENSG000002	86.517891	130.48452	42.551262	0.326102	-1.616605	0.0322345	0.3046792
ENSG000002	13.254238	0	26.508476	Inf	Inf	0.0206258	0.2283773
ENSG000002	255.50821	375.22366	135.79276	0.3618982	-1.466344	0.0017274	0.036808
ENSG000001	661.92669	356.77474	967.07865	2.7106141	1.4386197	0.0003211	0.0096011
ENSG000001	100.82831	44.9898	156.66682	3.4822741	1.8000298	0.0058629	0.0938238
ENSG000001	1179.4931	1567.041	791.94529	0.5053762	-0.98457	0.0454512	0.3776344
ENSG000001	41.401334	3.7905228	79.012144	20.844656	4.3816057	0.0041785	0.0730889
ENSG000001	21.959252	0	43.918504	Inf	Inf	0.0015082	0.0332355
ENSG000001	44129.82	21871.905	66387.735	3.0352973	1.6018378	1.30E-05	0.0006395
ENSG000001	1951.4737	3888.6862	14.261269	0.0036674	-8.091037	7.41E-60	6.69E-56
ENSG000001	429.39169	634.70613	224.07725	0.3530409	-1.502093	0.0116042	0.1528881
ENSG000001	442.18489	102.23619	782.13358	7.650261	2.935509	1.81E-07	1.38E-05

ENSG000001	239.19507	124.58574	353.80439	2.8398465	1.5058129	0.046714	0.3836504
ENSG000001	1425.6645	2007.7685	843.56047	0.4201483	-1.25103	0.0002799	0.0085005
ENSG000001	244.24669	331.34195	157.15144	0.4742878	-1.076165	0.0217544	0.2359499
ENSG000001	25.779432	46.102922	5.4559415	0.1183426	-3.078958	0.0168984	0.200214
ENSG000002	728.35792	1069.8781	386.83774	0.3615718	-1.467646	0.0175	0.2056308
ENSG000001	229.56866	449.35688	9.7804407	0.0217654	-5.521818	1.11E-13	2.16E-11
ENSG000001	46.51569	2.8428921	90.188487	31.724203	4.987512	0.0006906	0.0179533
ENSG000001	108.36688	44.285371	172.4484	3.8940262	1.9612626	0.0111556	0.1484181
ENSG000002	20.776359	38.439131	3.1135864	0.0810004	-3.625926	0.0312192	0.2985247
ENSG00000(	599.1122	228.98054	969.24385	4.2328656	2.0816347	0.0007726	0.0195758
ENSG000001	30.543578	56.045492	5.0416649	0.0899567	-3.474626	0.0059068	0.0943032
ENSG000001	2604.5084	3440.2078	1768.809	0.5141576	-0.959717	0.0047029	0.0795402
ENSG000002	75.350938	18.315825	132.38605	7.22796	2.8535885	0.0042112	0.073427
ENSG000001	539.37565	319.07528	759.67602	2.3808676	1.2514874	0.0092422	0.1299236
ENSG000001	283.03815	156.76994	409.30635	2.6108726	1.3845321	0.007113	0.1080155
ENSG000002	70.591009	23.053979	118.12804	5.1239762	2.3572638	0.0294911	0.2880035
ENSG00000(	2495.5723	4640.8368	350.3078	0.0754838	-3.72769	9.54E-07	6.27E-05
ENSG000001	44.531606	7.5810456	81.482167	10.748144	3.4260156	0.0224321	0.2407917
ENSG000001	28.404187	1.8952614	54.913113	28.9739	4.856682	0.0102698	0.1400764
ENSG000001	1277.6273	1784.1528	771.10186	0.432195	-1.210246	0.0013703	0.0308492
ENSG000001	219.5416	157.54201	281.54118	1.7870863	0.8376093	0.0293306	0.2869538
ENSG00000(	184.5382	71.58103	297.49537	4.1560644	2.055218	1.67E-05	0.0007883
ENSG00000(	2678.7287	3580.4399	1777.0176	0.4963127	-1.010679	0.0015525	0.0339633
ENSG00000(	891.44266	1190.7013	592.18398	0.4973405	-1.007694	0.0137935	0.1724716
ENSG000001	181.70486	241.57534	121.83438	0.5043329	-0.987552	0.0328084	0.3090252
ENSG00000(	1118.7842	1407.6635	829.90498	0.5895621	-0.762284	0.0149901	0.183674
ENSG000001	4858.3054	2598.1216	7118.4892	2.7398599	1.4541021	0.0006402	0.0169177
ENSG000001	426.16962	30.00327	822.33597	27.408211	4.7765363	4.99E-14	1.06E-11
ENSG000001	190.43776	110.66702	270.20851	2.4416353	1.2878477	0.0158966	0.1922031
ENSG000001	1063.2241	808.05928	1318.3889	1.6315498	0.706243	0.0395892	0.3446984
ENSG00000(	99.240842	34.570898	163.91079	4.7412939	2.2452808	0.0012985	0.0295026
ENSG000001	559.18817	308.75138	809.62497	2.6222554	1.3908082	0.0155208	0.1888467
ENSG000001	176.48616	111.13328	241.83904	2.1761171	1.1217562	0.016195	0.1946059
ENSG000001	213.31679	101.98292	324.65067	3.1833826	1.6705606	0.0020883	0.0429088
ENSG000002	10.637569	20.682384	0.592754	0.0286598	-5.124825	0.0401842	0.3470124
ENSG00000(	633.09008	347.97047	918.20969	2.6387575	1.3998588	0.0028484	0.0544739
ENSG00000(	60.606431	10.739815	110.47305	10.286309	3.3626534	0.0090181	0.1277156
ENSG000001	36.061682	3.4746459	68.648718	19.75704	4.3042949	0.0099234	0.1366755
ENSG000001	21.044085	39.003245	3.0849255	0.0790941	-3.660287	0.0097774	0.1354591
ENSG000001	321.58209	621.99049	21.17369	0.0340418	-4.876548	2.71E-10	3.36E-08
ENSG000001	802.14343	1013.0735	591.21341	0.583584	-0.776988	0.0297437	0.2899471
ENSG000002	844.19269	303.44189	1384.9435	4.5641143	2.1903349	1.01E-06	6.63E-05
ENSG000001	75.363003	120.92547	29.80054	0.2464373	-2.020708	0.0227006	0.242807
ENSG000001	272.95524	99.76675	446.14373	4.4718679	2.1608776	1.06E-05	0.0005373
ENSG000001	10585.554	3253.9024	17917.206	5.5063747	2.4611028	9.90E-11	1.31E-08
ENSG000001	20.301801	6.6283798	33.975222	5.1257205	2.3577548	0.0352482	0.3227928
ENSG000001	771.04458	143.33259	1398.7566	9.7588177	3.2867064	5.56E-14	1.16E-11
ENSG000001	448.83877	591.79437	305.88316	0.5168741	-0.952115	0.0444623	0.3726014
ENSG000001	99.079662	61.327619	136.83171	2.2311596	1.1577937	0.0429065	0.3622755
ENSG000001	153.53284	62.756617	244.30907	3.892961	1.9608679	0.0031005	0.0582244
ENSG000001	1123.6894	1525.3373	722.04136	0.473365	-1.078975	0.0021596	0.0441063

ENSG00000128.679785	54.631599	2.7279707	0.0499339	-4.323836	0.0008507	0.0210612	
ENSG00000113.250982	0	26.501964	Inf	Inf	0.0190286	0.215633	
ENSG000001	1079.2827	1945.6624	212.90287	0.1094244	-3.191994	0.0001509	0.0051226
ENSG00000(	681.30108	929.42868	433.17349	0.4660643	-1.101399	0.0081468	0.1187981
ENSG000001	229.94042	122.50485	337.37598	2.7539806	1.4615184	0.0044708	0.076766
ENSG000001	1161.7339	2181.6577	141.81018	0.0650011	-3.943392	8.32E-21	3.88E-18
ENSG00000	110.86269	24.317486	197.4079	8.1179402	3.0211137	0.0007224	0.0185649
ENSG00000(	18.828079	0	37.656158	Inf	Inf	0.0036173	0.065564
ENSG000001	252.66184	124.87141	380.45227	3.0467524	1.6072722	0.0010236	0.0244695
ENSG000001	171.11978	246.55669	95.682858	0.3880765	-1.365587	0.0213598	0.2330714
ENSG000001	223.98341	15.793845	432.17297	27.363379	4.7741745	1.62E-09	1.76E-07
ENSG000001	254.36368	164.2481	344.47926	2.0973105	1.0685404	0.0064931	0.1010404
ENSG000001	379.60675	759.2135	0	0	#NAME?	5.95E-10	6.97E-08
ENSG00000(	1557.5937	1.5793845	3113.608	1971.406	10.945009	2.02E-50	7.80E-47
ENSG00000(	53.428039	83.604493	23.251585	0.2781141	-1.846251	0.017649	0.2069762
ENSG00000	16.912256	32.282049	1.5424628	0.0477808	-4.387425	0.0215108	0.2342356
ENSG000001	109.45785	40.412103	178.50361	4.4170828	2.1430939	0.0014915	0.0329217
ENSG00000(	1564.5651	2026.9838	1102.1464	0.5437372	-0.879019	0.0254363	0.2622212
ENSG000001	801.37259	1051.2593	551.48587	0.5245955	-0.930723	0.0155752	0.1891746
ENSG000001	165.50789	63.694178	267.3216	4.196955	2.069343	0.0001481	0.0050687
ENSG000001	1533.7502	1181.2747	1886.2257	1.5967714	0.6751578	0.0428249	0.361856
ENSG000001	532.37695	720.01018	344.74372	0.478804	-1.062493	0.01313	0.1667506
ENSG000001	67.794442	122.42714	13.161743	0.1075067	-3.217501	0.0006809	0.0177541
ENSG00000	69.052393	20.604674	117.50011	5.7025951	2.5116186	0.0121718	0.1584327
ENSG000001	81.510521	132.00633	31.014709	0.2349486	-2.089583	0.0194928	0.2188778
ENSG00000	14.262897	0	28.525793	Inf	Inf	0.0152518	0.1861651
ENSG000001	12192.651	15153.561	9231.741	0.6092127	-0.714982	0.0195087	0.2189648
ENSG00000	508.46311	256.11289	760.81333	2.970617	1.5707626	1.42E-05	0.0006872
ENSG00000125.275093	0	50.550185	Inf	Inf	0.0004915	0.0135315	
ENSG000001	2042.5437	2537.874	1547.2134	0.6096494	-0.713948	0.03765	0.3359033
ENSG00000	22.234051	1.2635076	43.204594	34.19417	5.0956785	0.0203338	0.2260689
ENSG00000	536.01449	856.53883	215.49015	0.2515825	-1.990897	2.15E-05	0.0009772
ENSG000001	154.04215	51.773601	256.31071	4.9506061	2.3076052	2.21E-05	0.000997
ENSG000001	3208.693	4155.5381	2261.8479	0.5442973	-0.877533	0.0039104	0.0694346
ENSG000001	157.78198	97.954234	217.60973	2.2215449	1.1515633	0.0288991	0.2844127
ENSG000001	1508.6795	1888.7282	1128.6308	0.5975613	-0.742841	0.0168884	0.200214
ENSG000001	3241.6544	4331.0181	2152.2908	0.496948	-1.008833	0.0191563	0.2166266
ENSG000001	538.03521	299.94711	776.12331	2.5875339	1.3715778	0.0008806	0.0216837
ENSG000001	43.578425	87.156849	0	0	#NAME?	5.76E-09	5.90E-07
ENSG000001	104.13148	68.349586	139.91338	2.0470259	1.0335294	0.0361409	0.3278603
ENSG000001	396.29753	744.22764	48.367415	0.0649901	-3.943637	6.10E-08	5.15E-06
ENSG000001	1212.8934	468.32964	1957.4571	4.1796567	2.0633845	3.47E-05	0.0014822
ENSG000001	1142.3504	590.61865	1694.0822	2.8683182	1.5202051	0.0002968	0.0089538
ENSG00000	337.72227	217.48091	457.96363	2.1057647	1.0743443	0.0402975	0.3476224
ENSG00000(	895.57712	1344.8666	446.28767	0.3318453	-1.591417	2.07E-05	0.0009485
ENSG000001	1181.7798	130.84571	2232.7138	17.063714	4.0928598	1.16E-13	2.24E-11
ENSG00000(	181.85364	276.0937	87.613589	0.3173328	-1.655931	0.0466012	0.3831816
ENSG000001	114.04128	30.008306	198.07426	6.6006478	2.7226076	0.0091485	0.1290092
ENSG000001	135.15813	231.11112	39.205133	0.1696376	-2.559472	0.0021239	0.0435081
ENSG000001	803.12475	1285.9077	320.3418	0.2491173	-2.005103	1.71E-06	0.0001069
ENSG00000	608.95581	755.19555	462.71608	0.6127103	-0.706723	0.0394508	0.3440471

ENSG000002	106.20581	46.180633	166.23099	3.5995824	1.8478296	0.0211508	0.2316313
ENSG000001	671.05598	534.59548	807.51648	1.5105187	0.5950441	0.0350328	0.3217228
ENSG000001	91.790493	35.989826	147.59116	4.1009134	2.0359453	0.0226547	0.2424111
ENSG000001	206.13971	104.95606	307.32336	2.9281145	1.549972	0.0003881	0.0112203
ENSG000001	142.11329	92.574256	191.65232	2.0702551	1.0498085	0.0106877	0.1441051
ENSG000001	249.45349	141.93883	356.96814	2.5149434	1.330526	0.0253586	0.26172
ENSG000001	260.31328	79.570768	441.0558	5.5429375	2.4706507	0.0367945	0.3321813
ENSG000001	4037.4292	5145.8202	2929.0381	0.5692072	-0.812974	0.0121675	0.1584327
ENSG000001	1542.4402	813.42197	2271.4584	2.7924724	1.481543	0.0007703	0.0195425
ENSG000001	966.46937	1267.4767	665.46202	0.525029	-0.929531	0.0119249	0.155893
ENSG000001	126.01577	46.56415	205.4674	4.4125663	2.141618	0.000141	0.0048786
ENSG000001	68.112133	21.863146	114.36112	5.2307714	2.3870237	0.0036768	0.0664133
ENSG000002	219.99	136.63657	303.34344	2.2200751	1.1506085	0.0283623	0.2806256
ENSG000001	15.061161	0	30.122322	Inf	Inf	0.0112091	0.1489707
ENSG000001	12.704475	0	25.408951	Inf	Inf	0.023753	0.2504914
ENSG000001	3107.7623	2358.5172	3857.0074	1.6353526	0.7096018	0.0198817	0.2225058
ENSG000001	120.59723	50.214357	190.9801	3.8032967	1.9272505	0.0349525	0.321512
ENSG000001	435.62892	347.04516	524.21267	1.5105028	0.5950288	0.0433223	0.3651029
ENSG000002	135.5229	207.30521	63.740589	0.3074722	-1.701472	0.0048407	0.0809609
ENSG000001	622.02541	414.0312	830.01962	2.0047272	1.0034059	0.0224631	0.2410287
ENSG000001	7.4205896	14.841179	0	0	#NAME?	0.0381488	0.3378143
ENSG000002	559.224	709.08759	409.36042	0.5773059	-0.792592	0.0256932	0.2638287
ENSG000000	149.11877	95.976227	202.26132	2.1074106	1.0754714	0.0200768	0.224234
ENSG000001	359.63678	500.33827	218.93529	0.4375745	-1.192399	0.0060669	0.0961213
ENSG000000	331.33967	192.61442	470.06492	2.4404452	1.2871443	0.0168538	0.200123
ENSG000000	414.70261	700.85968	128.54553	0.1834112	-2.446846	7.66E-08	6.28E-06
ENSG000001	183.13903	77.996419	288.28164	3.6960881	1.8859991	0.0049529	0.0823733
ENSG000000	1313.4973	401.68179	2225.3128	5.5399892	2.4698832	4.50E-10	5.41E-08
ENSG000000	109.26762	181.23278	37.302459	0.2058262	-2.280501	0.0022753	0.045795
ENSG000001	651.10979	874.31572	427.90386	0.4894157	-1.030868	0.0299488	0.2911544
ENSG000000	344.88854	51.487935	638.28916	12.396868	3.6319038	2.88E-05	0.0012545
ENSG000001	413.81346	598.97176	228.65515	0.3817461	-1.389315	0.0052425	0.0855134
ENSG000001	6711.5849	297.10925	13126.061	44.179239	5.4652967	5.18E-33	5.40E-30
ENSG000001	203.05398	60.462734	345.64524	5.7166657	2.5151739	1.42E-05	0.000688
ENSG000001	537.30304	406.10407	668.502	1.6461347	0.7190824	0.0318201	0.302135
ENSG000001	926.57318	407.45536	1445.691	3.5480966	1.8270453	3.67E-06	0.0002066
ENSG000001	1883.6206	912.34616	2854.895	3.1291796	1.6457845	1.79E-05	0.0008392
ENSG000000	1901.475	2628.7682	1174.1817	0.4466661	-1.162731	0.0042934	0.0745726
ENSG000002	64.586664	113.3696	15.803731	0.1394001	-2.842697	0.0003773	0.0109678
ENSG000001	1297.9873	933.68547	1662.2891	1.7803523	0.8321628	0.0373923	0.3347712
ENSG000001	1140.0537	1656.9199	623.1876	0.3761121	-1.410765	0.0001196	0.00427
ENSG000001	1201.0747	556.83996	1845.3094	3.3138954	1.7285281	5.73E-05	0.0022951
ENSG000002	471.20042	923.21907	19.181778	0.0207771	-5.588865	1.14E-26	8.81E-24
ENSG000002	26.137191	0	52.274381	Inf	Inf	0.0004018	0.0115394
ENSG000001	1803.9198	1057.5114	2550.3283	2.411632	1.2700098	0.0006809	0.0177541
ENSG000002	45962.767	34338.545	57586.99	1.6770364	0.745914	0.0107598	0.1447898
ENSG000001	91.308919	48.858034	133.7598	2.7377239	1.4529769	0.0303985	0.2938454
ENSG000001	91.312614	175.8053	6.8199268	0.0387925	-4.688079	7.81E-09	7.74E-07
ENSG000002	21.910926	1.8952614	41.926592	22.121799	4.4673968	0.0386064	0.340302
ENSG000001	10.821016	0	21.642032	Inf	Inf	0.0423509	0.3593784
ENSG000000	1546.2984	2713.2304	379.36641	0.1398209	-2.838348	2.77E-13	4.97E-11

ENSG00000(	125.47676	84.920535	166.03298	1.955157	0.9672845	0.0177649	0.2075715
ENSG000001	55.658567	14.913855	96.40328	6.4640083	2.692429	0.0002337	0.0073119
ENSG000002	24.471944	0	48.943889	Inf	Inf	0.0008055	0.0202204
ENSG00000(	560.40972	253.27	867.54944	3.4253936	1.7762698	0.0005632	0.0152409
ENSG000001	137.20485	267.29665	7.1130478	0.0266111	-5.23183	1.91E-12	3.12E-10
ENSG000001	6185.1809	3078.4517	9291.9101	3.0183712	1.5937703	0.0001051	0.0038468
ENSG000001	1264.8122	778.27688	1751.3475	2.2502884	1.1701099	0.0057206	0.0920919
ENSG000001	279.48125	375.93313	183.02937	0.4868668	-1.038401	0.0156566	0.1899072
ENSG000001	1841.1122	2266.7847	1415.4397	0.6244262	-0.679397	0.0271504	0.2730012
ENSG000002	428.70111	731.04859	126.35364	0.1728389	-2.5325	0.0001154	0.004136
ENSG000001	1235.9815	1622.4821	849.48086	0.5235687	-0.933549	0.0088932	0.1264625
ENSG000001	268.48505	395.60024	141.36986	0.3573553	-1.484569	0.002328	0.0465952
ENSG000001	197.06787	136.14513	257.9906	1.8949676	0.9221732	0.0126271	0.1621744
ENSG000002	33.793767	5.6857842	61.90175	10.887108	3.4445489	0.0405535	0.3488504
ENSG00000(	68143.295	46646.165	89640.425	1.9217105	0.942391	0.017424	0.205362
ENSG000001	2022.3232	1479.2661	2565.3803	1.734225	0.7942911	0.0076057	0.1130252
ENSG000002	100.83217	166.79022	34.874122	0.2090897	-2.257806	0.00234	0.0467322
ENSG000001	50.431746	20.837805	80.025687	3.8404086	1.9412598	0.0370032	0.332455
ENSG000001	631.75175	446.70684	816.79666	1.8284848	0.8706486	0.0360651	0.3273931
ENSG000002	17.435433	0	34.870866	Inf	Inf	0.0056838	0.091662
ENSG000001	12.365107	0	24.730214	Inf	Inf	0.0275591	0.2748962
ENSG000001	50.338321	94.567369	6.1092733	0.0646023	-3.95227	0.0001118	0.0040297
ENSG000001	20.236684	0	40.473367	Inf	Inf	0.0023645	0.0470839
ENSG000001	964.09815	638.33838	1289.8579	2.0206492	1.0148188	0.0049031	0.0817688
ENSG000002	53.053792	17.047282	89.060301	5.2243108	2.3852407	0.0046062	0.0784439
ENSG000001	3109.7975	4277.6738	1941.9211	0.4539666	-1.139342	0.0008924	0.0218952
ENSG000001	20.845396	0	41.690792	Inf	Inf	0.0020092	0.0415678
ENSG000001	142.93706	272.82701	13.0471	0.0478219	-4.386185	5.68E-11	7.89E-09
ENSG000001	119.82498	177.15156	62.498403	0.3527962	-1.503093	0.0408503	0.350761
ENSG000001	1576.8071	1232.0129	1921.6014	1.559725	0.6412917	0.0437995	0.3680924
ENSG000001	578.42565	232.62286	924.22844	3.9730765	1.9902566	6.58E-06	0.0003469
ENSG000001	22.950609	0.6317538	45.269465	71.656815	6.163032	0.0059786	0.0951134
ENSG000001	83630.726	100376.47	66884.982	0.6663412	-0.585667	0.0453964	0.3774111
ENSG000001	22.473842	0	44.947683	Inf	Inf	0.0011578	0.0268007
ENSG000001	199.13234	129.52178	268.7429	2.0748857	1.0530319	0.0142538	0.1766939
ENSG00000(	328.46039	223.14152	433.77926	1.9439648	0.9590021	0.021324	0.2328685
ENSG00000(	6141.4075	3908.9133	8373.9017	2.1422583	1.0991324	0.0001578	0.0053098
ENSG000001	385.90978	28.501596	743.31796	26.079871	4.7048648	1.59E-13	2.99E-11
ENSG000002	1363.9196	537.78227	2190.057	4.072386	2.0258743	1.50E-05	0.0007195
ENSG000001	90.339277	45.150256	135.5283	3.0017171	1.585788	0.0284638	0.2812187
ENSG000002	31.618344	10.81249	52.424198	4.8484852	2.2775341	0.0383651	0.3388373
ENSG000001	273.69336	104.58261	442.80411	4.2340126	2.0820256	2.49E-05	0.0011099
ENSG00000(	327.61648	443.39769	211.83526	0.4777546	-1.065658	0.027186	0.2730012
ENSG000001	1074.4938	635.46025	1513.5274	2.3817815	1.252041	0.0001227	0.0043483
ENSG000001	26.545848	1.2635076	51.828188	41.019293	5.3582307	0.0073032	0.1099178
ENSG000001	774.64235	1042.2241	507.06059	0.4865178	-1.039435	0.0153678	0.1871598
ENSG000001	6640.8041	2686.0067	10595.601	3.9447412	1.9799307	4.87E-08	4.24E-06
ENSG000001	165.17792	234.52316	95.832674	0.4086278	-1.291141	0.0352224	0.3226655
ENSG000001	454.49278	4.7381535	904.24741	190.84384	7.5762488	1.26E-24	8.50E-22
ENSG000001	407.55254	552.11187	262.9932	0.4763404	-1.069935	0.0203833	0.2263533
ENSG00000(	781.55742	617.93159	945.18325	1.529592	0.6131469	0.0310555	0.2974841

ENSG000001	1933.3383	1016.1243	2850.5523	2.8053185	1.4881646	0.0003529	0.0103803
ENSG000001	1494.3845	2654.3217	334.44739	0.1260011	-2.988492	0.0345209	0.3191881
ENSG000001	37.533322	10.258447	64.808198	6.3175449	2.659364	0.0027643	0.0532049
ENSG000002	355.16264	203.82553	506.49976	2.4849672	1.3132268	0.0124434	0.1606544
ENSG000001	1785.8086	1002.5142	2569.1029	2.5626598	1.357642	0.0029816	0.0565022
ENSG000000	74.52907	107.1348	41.923336	0.3913139	-1.353602	0.040014	0.3465024
ENSG000001	53.320774	14.913855	91.727694	6.1505021	2.6207042	0.0002845	0.0086131
ENSG000002	657.34399	1085.5417	229.14629	0.2110893	-2.244074	3.47E-07	2.49E-05
ENSG000001	126.50962	64.88501	188.13423	2.8995022	1.5358052	0.0192559	0.2172087
ENSG000001	1397.9803	1833.7952	962.16533	0.5246853	-0.930476	0.0275025	0.2746293
ENSG000001	102.4879	23.374891	181.60091	7.7690593	2.9577399	0.004834	0.080898
ENSG000000	3392.9988	2064.5134	4721.4843	2.286972	1.1934387	0.0050362	0.083208
ENSG000001	1069.0506	577.38925	1560.712	2.7030499	1.4345882	6.73E-05	0.0026341
ENSG000001	96.105818	39.158665	153.05297	3.9085339	1.9666275	0.033215	0.31123
ENSG000001	109.33446	73.481327	145.18758	1.9758433	0.9824685	0.0472996	0.3864657
ENSG000000	85.043946	138.2987	31.789196	0.229859	-2.121179	0.0055144	0.0894055
ENSG000001	673.53217	435.65115	911.4132	2.0920711	1.0649319	0.0110466	0.1475481
ENSG000001	201.83459	82.501441	321.16774	3.8928744	1.9608358	0.0064785	0.1009317
ENSG000001	31.6887	2.8428921	60.534508	21.293284	4.4123265	0.0330056	0.3103417
ENSG000001	55.756367	17.130028	94.382706	5.5097812	2.461995	0.0200127	0.2236941
ENSG000001	6113.6124	8150.0941	4077.1308	0.5002557	-0.999262	0.0016493	0.0354223
ENSG000001	1219.0052	1543.7661	894.24419	0.5792614	-0.787713	0.012915	0.1645144
ENSG000001	157.47921	77.520086	237.43833	3.0629265	1.6149108	0.0168134	0.1997314
ENSG000002	76.548444	12.55233	140.54456	11.19669	3.4850005	0.0004869	0.0134596
ENSG000001	265.72242	522.43237	9.0124654	0.017251	-5.857179	0.0004072	0.0116482
ENSG000001	123.20765	34.172275	212.24303	6.2109715	2.6348189	1.08E-05	0.0005446
ENSG000002	35.110113	17.834457	52.385769	2.9373346	1.5545076	0.0304762	0.294331
ENSG000001	131.37814	37.258369	225.49791	6.0522754	2.5974776	0.0434677	0.3659859
ENSG000002	301.79556	161.23972	442.3514	2.7434394	1.4559857	0.0001037	0.0038069
ENSG000001	992.30006	450.2592	1534.3409	3.4076837	1.7687914	2.24E-05	0.0010116
ENSG000001	50.289416	95.597745	4.9810871	0.0521047	-4.262444	3.92E-05	0.0016331
ENSG000001	4012.5948	4984.6538	3040.5358	0.6099793	-0.713168	0.0161175	0.194192
ENSG000000	411.24121	204.04859	618.43383	3.0308164	1.5997065	0.000699	0.0181347
ENSG000002	52.501185	90.145092	14.857279	0.1648152	-2.601079	0.0070001	0.106781
ENSG000002	311.42267	537.17284	85.672487	0.1594877	-2.648483	5.58E-07	3.88E-05
ENSG000001	10975.269	13863.521	8087.016	0.5833306	-0.777614	0.0142418	0.1766254
ENSG000001	24.341342	0	48.682685	Inf	Inf	0.0006532	0.017151
ENSG000001	983.16612	380.66339	1585.6688	4.1655407	2.0585038	1.27E-08	1.23E-06
ENSG000001	805.75583	1395.6991	215.81258	0.1546269	-2.693137	0.0189607	0.2152246
ENSG000001	2135.0962	1206.2988	3063.8935	2.5399126	1.3447788	3.62E-05	0.001525
ENSG000001	283.34168	149.19897	417.4844	2.7981722	1.4844847	0.0091334	0.1289306
ENSG000001	10.460275	20.920551	0	0	#NAME?	0.0088228	0.1257923
ENSG000000	1033.5748	757.9327	1309.2169	1.7273524	0.7885625	0.0228262	0.2439575
ENSG000002	32.897612	55.418773	10.376451	0.1872371	-2.417062	0.0304042	0.2938454
ENSG000001	399.86571	282.51127	517.22014	1.8307947	0.87247	0.0383144	0.3385002
ENSG000002	1527.0557	2.8428921	3051.2685	1073.2973	10.067834	1.85E-47	5.00E-44
ENSG000001	950.55636	1285.2759	615.83678	0.4791475	-1.061458	0.0071061	0.1079713
ENSG000001	2427.9685	608.05667	4247.8804	6.9859943	2.8044655	3.38E-14	7.52E-12
ENSG000001	598.0642	412.43671	783.69168	1.9001501	0.9261134	0.0189245	0.2152057
ENSG000001	865.65373	96.224464	1635.083	16.992384	4.0868163	9.73E-17	2.89E-14
ENSG000001	295.20271	470.97398	119.43145	0.253584	-1.979465	0.0471134	0.3857598

ENSG000001	68.590224	121.49462	15.685832	0.1291072	-2.953358	0.0002771	0.0084355
ENSG000001	525.7627	316.7965	734.72889	2.3192456	1.2136556	0.0187743	0.2142175
ENSG00000(	414.36157	667.25371	161.46943	0.2419911	-2.046974	2.75E-05	0.001211
ENSG000001	14.340487	26.606335	2.0746389	0.0779754	-3.680838	0.0398294	0.3456775
ENSG000001	20.98099	38.99821	2.9637699	0.0759976	-3.717903	0.0163504	0.1958109
ENSG000001	227.72134	386.87083	68.57186	0.1772474	-2.496163	1.50E-05	0.0007203
ENSG000001	131.82691	13.898584	249.75524	17.969834	4.1675052	6.69E-06	0.0003516
ENSG000001	764.64134	449.27917	1080.0035	2.4038584	1.2653519	0.0132054	0.1672457
ENSG000001	461.65198	188.04679	735.25717	3.9099693	1.9671573	0.0002451	0.0076163
ENSG000001	76.191679	0	152.38336	Inf	Inf	7.65E-08	6.28E-06
ENSG000001	3299.2269	1555.663	5042.7907	3.24157	1.6966927	8.70E-07	5.78E-05
ENSG000001	871.61531	1131.151	612.07963	0.5411122	-0.886	0.0126641	0.1624289
ENSG000001	6338.7805	7989.4552	4688.1057	0.5867867	-0.769092	0.0178297	0.2078804
ENSG000002	54.503169	86.286929	22.719409	0.2633007	-1.925217	0.0139769	0.1740584
ENSG000001	837.9755	1442.0308	233.92023	0.1622158	-2.624013	6.10E-10	7.12E-08
ENSG00000(	59.712643	90.077452	29.347834	0.3258067	-1.617912	0.0444404	0.3725531
ENSG000001	182.85884	251.11425	114.60343	0.4563796	-1.131694	0.0293207	0.2869538
ENSG000001	16.248297	0	32.496594	Inf	Inf	0.0079725	0.1170616
ENSG000001	1900.684	2364.9598	1436.4082	0.6073711	-0.71935	0.0220523	0.2377515
ENSG000001	19.302311	38.604622	0	0	#NAME?	0.0001606	0.0053931
ENSG000001	222.76528	123.92378	321.60678	2.5951983	1.3758448	0.0050395	0.083208
ENSG00000(	538.76648	699.90702	377.62593	0.5395373	-0.890205	0.020353	0.2261901
ENSG000001	189.47562	280.7189	98.232351	0.3499314	-1.514856	0.0041644	0.0729881
ENSG000002	741.08328	484.56172	997.60484	2.0587777	1.041788	0.0029866	0.0565446
ENSG000002	9.3521887	18.704377	0	0	#NAME?	0.0349539	0.321512
ENSG000001	268.22462	109.77192	426.67731	3.8869439	1.9586363	0.002202	0.0446683
ENSG000001	12.258282	0	24.516564	Inf	Inf	0.0263764	0.2680691
ENSG000001	84.096673	151.71088	16.482468	0.1086439	-3.20232	0.0002211	0.007005
ENSG00000(	271.59103	118.86471	424.31735	3.5697503	1.8358232	6.50E-05	0.0025514
ENSG00000(	310.92813	445.5009	176.35536	0.3958586	-1.336943	0.0232257	0.2468472
ENSG000001	2986.0298	1929.6908	4042.3687	2.0948271	1.0668312	0.0010311	0.0245187
ENSG000001	723.92681	165.60443	1282.2492	7.7428437	2.9528635	9.00E-11	1.20E-08
ENSG000001	666.4948	329.88777	1003.1018	3.0407366	1.6044208	0.0004318	0.0121727
ENSG000001	1094.5515	652.51038	1536.5927	2.3548939	1.2356621	0.0033891	0.0622617
ENSG00000(	134.39312	70.871566	197.91467	2.7925822	1.4815997	0.0016843	0.0360315
ENSG000001	73.537805	147.07561	0	0	#NAME?	8.78E-14	1.75E-11
ENSG000001	114.25479	177.4624	51.047188	0.2876507	-1.79761	0.0072109	0.109136
ENSG00000(	41.655262	13.339505	69.971018	5.2453983	2.3910523	0.0263654	0.2680691
ENSG000001	900.96752	491.55347	1310.3816	2.6657966	1.4145667	0.0015067	0.0332297
ENSG000001	139.86979	248.47931	31.260276	0.1258064	-2.990723	6.20E-05	0.0024573
ENSG00000(	228.02828	339.42669	116.62987	0.3436084	-1.541163	0.0094578	0.1325411
ENSG000001	294.74475	128.1856	461.30389	3.5987185	1.8474833	0.0001349	0.0047081
ENSG000001	336.78535	64.744694	608.826	9.4034886	3.2331961	0.0095124	0.1330306
ENSG000001	3295.5726	2461.5967	4129.5485	1.6775894	0.7463897	0.0211655	0.2316992
ENSG000001	560.17885	321.35406	799.00363	2.4863654	1.3140383	0.0025562	0.049837
ENSG000002	320.57239	136.70924	504.43553	3.6898422	1.8835591	7.12E-05	0.0027721
ENSG000002	106.15759	54.466107	157.84907	2.8981154	1.5351151	0.0287036	0.2829681
ENSG000001	3345.2803	4094.6918	2595.8687	0.6339595	-0.657537	0.0219749	0.2373828
ENSG000002	38.468092	75.454299	1.481885	0.0196395	-5.670098	0.0011147	0.0260501
ENSG000001	796.54486	501.5464	1091.5433	2.1763556	1.1219143	0.0024698	0.0484657
ENSG000001	237.91456	165.26841	310.56072	1.8791294	0.9100644	0.0360242	0.3272442

ENSG000001	397.80735	243.40514	552.20955	2.2686848	1.1818562	0.0005292	0.0144956
ENSG000001	1329.4013	1798.8867	859.91592	0.4780267	-1.064837	0.0193165	0.2176203
ENSG00000(	1601.5934	2247.5666	955.62028	0.42518	-1.233854	0.0011739	0.0269669
ENSG000001	25.679923	0	51.359846	Inf	Inf	0.0004879	0.0134739
ENSG000001	166.53094	53.518477	279.5434	5.2233063	2.3849633	0.0001804	0.0059243
ENSG000001	229.97132	124.09934	335.84329	2.7062455	1.4362927	0.0265802	0.2693961
ENSG000001	2167.034	996.48237	3337.5856	3.3493675	1.7438887	2.93E-05	0.0012694
ENSG000002	222.22526	133.17199	311.27853	2.3374174	1.2249154	0.0399554	0.3463601
ENSG000002	10.654552	21.309103	0	0	#NAME?	0.0202492	0.2253145
ENSG000001	1353.7263	1967.9954	739.45726	0.3757414	-1.412188	0.0001656	0.005515
ENSG000001	938.97574	1178.6628	699.28871	0.5932899	-0.753191	0.0338559	0.315378
ENSG000001	288.78576	474.20827	103.36325	0.2179702	-2.197797	5.21E-06	0.0002829
ENSG000001	8558.8995	4728.919	12388.88	2.6198123	1.3894634	2.88E-05	0.0012545
ENSG000001	623.24827	362.59362	883.90292	2.4377233	1.2855344	0.0139003	0.1734636
ENSG000001	636.76532	386.23404	887.2966	2.297303	1.1999411	0.0035268	0.0642687
ENSG000001	1132.1572	848.48649	1415.8279	1.6686511	0.7386823	0.041107	0.3519128
ENSG000002	1698.3082	2326.42	1070.1963	0.4600185	-1.120236	0.0010806	0.0254135
ENSG000001	198.64269	295.70039	101.58499	0.3435403	-1.541449	0.0072491	0.109346
ENSG000001	2841.5948	3714.5523	1968.6374	0.5299797	-0.915991	0.0122016	0.1585917
ENSG000002	1649.7023	1050.4995	2248.9052	2.140796	1.0981473	0.0219676	0.2373828
ENSG000001	72.079653	31.888462	112.27084	3.5207357	1.8158769	0.0260857	0.2664803
ENSG000001	291.55907	98.601093	484.51705	4.9139116	2.2968719	0.0001087	0.0039428
ENSG000001	175.99494	307.89942	44.090469	0.1431976	-2.80392	0.0006409	0.016921
ENSG000001	891.67296	1743.8379	39.508022	0.0226558	-5.463977	9.98E-30	8.71E-27
ENSG000001	281.31222	385.89584	176.7286	0.4579697	-1.126676	0.0248353	0.2583887
ENSG000001	863.90658	1337.7518	390.06139	0.2915798	-1.778037	3.88E-06	0.000217
ENSG000001	692.74163	930.6749	454.80836	0.4886866	-1.033019	0.0048884	0.0816068
ENSG000001	194.12871	253.96218	134.29524	0.5288001	-0.919206	0.0488915	0.3943525
ENSG000001	20071.774	14501.491	25642.057	1.7682359	0.8223107	0.0086305	0.1240966
ENSG000001	489.56323	789.8888	189.23765	0.2395751	-2.06145	0.0007563	0.0192952
ENSG000002	63.353183	99.222777	27.48359	0.2769887	-1.852101	0.0457429	0.3791266
ENSG000001	877.21141	433.77099	1320.6518	3.0445831	1.6062447	0.0002282	0.0071811
ENSG000001	36.439608	1.5793845	71.299831	45.144061	5.4964643	0.0011406	0.0264935
ENSG000001	93.169627	146.84751	39.491742	0.2689303	-1.894696	0.019436	0.2186131
ENSG000001	346.35289	139.39671	553.30907	3.9693122	1.988889	3.91E-05	0.0016305
ENSG00000(	103.16516	152.60597	53.724349	0.3520462	-1.506163	0.0117905	0.1545101
ENSG000001	122.18144	225.0821	19.280785	0.0856611	-3.545216	8.02E-05	0.0030566
ENSG000001	1054.341	1296.9238	811.75825	0.6259105	-0.675972	0.0422702	0.3591545
ENSG000002	552.41684	156.99804	947.83565	6.0372452	2.5938904	1.82E-08	1.72E-06
ENSG000001	1163.8102	1727.7533	599.86695	0.3471948	-1.526183	0.0002329	0.0072961
ENSG000001	62.845299	22.101313	103.58929	4.6870196	2.2286708	0.0116406	0.1530729
ENSG000001	806.09418	1072.5159	539.67248	0.5031837	-0.990843	0.0064144	0.1001614
ENSG000001	172.00321	98.119725	245.8867	2.5059865	1.3253786	0.0282714	0.2801365
ENSG000001	127.90124	216.50811	39.294372	0.1814915	-2.462026	0.0042348	0.0737907
ENSG000001	1440.6116	4.7381535	2876.4849	607.08986	9.2457663	9.12E-44	1.64E-40
ENSG000001	1152.617	1460.7459	844.48803	0.5781211	-0.790556	0.0350082	0.3217228
ENSG000001	1461.3093	1123.7851	1798.8336	1.6006917	0.6786955	0.0323507	0.3054583
ENSG000001	116.6712	233.3424	0	0	#NAME?	4.89E-19	1.92E-16
ENSG000002	239.66689	416.65104	62.682749	0.1504442	-2.732699	2.44E-07	1.79E-05
ENSG000001	195.33678	127.94743	262.72612	2.0533911	1.0380084	0.025533	0.2630177
ENSG000001	331.00663	67.013403	594.99987	8.8788189	3.1503678	3.22E-09	3.38E-07

ENSG000002	3336.2383	1795.6439	4876.8327	2.7159242	1.4414432	1.86E-05	0.000867
ENSG00000(	321.58777	13.261795	629.91374	47.498378	5.5698063	4.92E-08	4.27E-06
ENSG00000l	28.842319	53.057249	4.6273883	0.087215	-3.51928	0.0127424	0.1630363
ENSG00000l	1021.4884	1317.9292	725.04746	0.5501414	-0.862126	0.0150113	0.1838103
ENSG000002	107.60088	34.658678	180.54307	5.2091736	2.3810545	0.0067325	0.1040488
ENSG00000(	1596.3125	2429.2505	763.37456	0.3142428	-1.670048	2.66E-06	0.0001557
ENSG000002	178.09557	106.06918	250.12196	2.3581021	1.2376262	0.0024278	0.0479902
ENSG00000l	86.824979	49.562463	124.0875	2.5036588	1.324038	0.0124361	0.1606415
ENSG00000(	27.430796	9.9425698	44.919022	4.5178484	2.1756358	0.0170848	0.2016304
ENSG00000l	87.637688	0	175.27538	Inf	Inf	4.74E-11	6.79E-09
ENSG00000l	269.05629	157.48444	380.62813	2.4169253	1.2731729	0.0215584	0.2345766
ENSG000002	14.78237	0	29.564741	Inf	Inf	0.0131712	0.1671324
ENSG00000l	1059.4186	572.56113	1546.2761	2.7006306	1.4332963	0.00117	0.0269621
ENSG00000l	203.7584	104.02354	303.49326	2.9175441	1.5447545	0.0011231	0.0262004
ENSG00000l	720.05313	528.54128	911.56498	1.7246808	0.7863293	0.0254074	0.2620234
ENSG00000l	142.42263	254.30541	30.539854	0.1200912	-3.057797	0.0217442	0.2359499
ENSG00000l	121.59513	231.29675	11.893509	0.051421	-4.281499	1.05E-09	1.17E-07
ENSG00000l	163.4553	116.3377	210.5729	1.8100143	0.8560011	0.032662	0.3077528
ENSG00000(	412.71451	541.76565	283.66337	0.5235905	-0.933489	0.0457106	0.3790499
ENSG00000l	191.15298	59.685629	322.62032	5.4053267	2.4343818	0.0006355	0.0168274
ENSG00000l	577.30232	959.25639	195.34825	0.2036455	-2.295868	8.21E-07	5.51E-05
ENSG00000l	868.00758	277.30182	1458.7133	5.2603813	2.3951674	3.34E-08	2.97E-06
ENSG00000l	131.35235	48.396806	214.3079	4.4281414	2.1467013	0.0097892	0.1355017
ENSG00000l	145.33286	27.155343	263.51038	9.7038132	3.2785518	9.07E-06	0.0004694
ENSG00000l	1153.1145	669.71812	1636.5109	2.4435816	1.2889973	0.0015342	0.0336448
ENSG00000l	292.75769	65.506694	520.00869	7.9382527	2.9888215	1.42E-09	1.56E-07
ENSG00000l	78.512134	42.618206	114.40606	2.684441	1.4246217	0.0410034	0.3512478
ENSG00000l	140.69593	16.109722	265.28213	16.467207	4.041524	9.01E-06	0.0004678
ENSG00000l	2210.4019	1599.9642	2820.8397	1.7630643	0.8180851	0.0453076	0.3771364
ENSG000002	119.04431	36.859747	201.22888	5.4593127	2.4487193	0.0002011	0.0064869
ENSG00000l	100.77314	57.770227	143.77604	2.4887568	1.3154252	0.008101	0.1184986
ENSG00000l	317.72398	135.28024	500.16771	3.6972709	1.8864608	1.40E-05	0.0006813
ENSG00000l	795.36486	539.70993	1051.0198	1.947379	0.9615337	0.0282922	0.2801371
ENSG00000(	153.95269	234.46055	73.444815	0.3132502	-1.674613	0.0029729	0.0563761
ENSG00000(	12.822375	0	25.64475	Inf	Inf	0.0224883	0.2412035
ENSG00000(	1898.9393	2644.0439	1153.8346	0.4363901	-1.19631	0.0004148	0.0117805
ENSG00000l	917.48636	1820.2685	14.704206	0.008078	-6.951779	5.15E-14	1.09E-11
ENSG000002	9535.024	12658.426	6411.6224	0.5065103	-0.981337	0.017085	0.2016304
ENSG00000l	1563.7318	1147.278	1980.1857	1.7259859	0.7874207	0.0337605	0.3148148
ENSG00000l	824.21548	403.72526	1244.7057	3.0830514	1.6243589	0.001026	0.0244715
ENSG00000(	144.83164	73.646818	216.01646	2.9331404	1.5524461	0.0140693	0.1749675
ENSG00000l	1341.4989	1983.1625	699.83524	0.3528885	-1.502716	4.92E-05	0.0019959
ENSG000002	347.83814	123.23949	572.43679	4.6449136	2.2156518	0.0002598	0.007988
ENSG00000l	443.74444	233.90148	653.5874	2.7942851	1.4824792	0.0033017	0.061071
ENSG000002	181.05674	264.20552	97.907957	0.370575	-1.432163	0.0251546	0.2603088
ENSG00000l	2403.8365	1229.6312	3578.0418	2.9098494	1.5409445	1.45E-05	0.0006972
ENSG00000l	1055.668	1446.5084	664.82758	0.4596085	-1.121523	0.0029409	0.055887
ENSG00000l	274.17953	372.44337	175.91568	0.4723287	-1.082137	0.0241344	0.2534347
ENSG00000l	779.52784	996.65793	562.39775	0.5642836	-0.825508	0.0204504	0.2268999
ENSG00000(	185.04433	52.43053	317.65813	6.058648	2.5989959	0.001352	0.0304894
ENSG00000l	564.33698	738.16052	390.51345	0.5290359	-0.918562	0.0357785	0.3259937

ENSG000001	1280.978	395.67291	2166.283	5.4749338	2.4528415	1.26E-07	9.94E-06
ENSG000002	155.94597	62.834328	249.05761	3.9637189	1.9868546	0.0021455	0.0438521
ENSG000002	165.64427	65.040431	266.24812	4.0935786	2.0333626	0.0006817	0.0177541
ENSG000001	158.53104	96.048902	221.01318	2.3010485	1.2022914	0.0040947	0.0720927
ENSG000001	7832.494	1008.3526	14656.635	14.535228	3.8614818	5.68E-20	2.40E-17
ENSG000001	1250.9597	1845.8287	656.09063	0.355445	-1.492302	8.26E-05	0.0031384
ENSG000001	49.173464	10.423938	87.92299	8.4347195	3.0763401	0.0368013	0.3321813
ENSG000001	166.30819	72.621477	259.9949	3.5801379	1.8400151	0.002698	0.052187
ENSG000000	204.82352	9.7921839	399.85485	40.834083	5.3517019	1.04E-10	1.37E-08
ENSG000001	343.54278	588.29237	98.793188	0.1679321	-2.57405	3.06E-08	2.74E-06
ENSG000002	893.23026	655.06979	1131.3907	1.7271301	0.7883767	0.02912	0.285306
ENSG000001	204.2973	342.29475	66.299851	0.1936923	-2.368162	1.02E-05	0.0005209
ENSG000000	1425.3356	1978.3941	872.27712	0.4409016	-1.181471	0.001684	0.0360315
ENSG000001	33.016067	0	66.032135	Inf	Inf	5.38E-05	0.0021624
ENSG000001	433.63451	230.39662	636.87239	2.7642436	1.4668848	0.0002754	0.0084033
ENSG000001	54.391847	2.5270152	106.25668	42.048294	5.3939754	0.0001011	0.003733
ENSG000001	57.189332	111.29374	3.0849255	0.0277188	-5.172993	1.50E-06	9.46E-05
ENSG000001	58.106499	114.67053	1.5424628	0.0134513	-6.216115	0.0316277	0.3010473
ENSG000002	444.09515	886.64784	1.5424628	0.0017397	-9.166982	7.23E-18	2.61E-15
ENSG000002	476.77234	251.55534	701.98935	2.7905961	1.4805733	0.0002825	0.0085713
ENSG000001	933.44745	1283.5534	583.34151	0.4544739	-1.137731	0.0187114	0.2140109
ENSG000001	596.67615	410.36086	782.99143	1.9080559	0.9321034	0.0079561	0.1169301
ENSG000001	583.52862	771.69886	395.35839	0.5123221	-0.964877	0.0209065	0.2299305
ENSG000001	111.08664	0	222.17329	Inf	Inf	2.43E-13	4.42E-11
ENSG000001	564.09331	336.97738	791.20923	2.3479595	1.2314075	0.0034604	0.0633579
ENSG000002	38.996354	65.449124	12.543584	0.191654	-2.383424	0.0110081	0.1471785
ENSG000001	1771.5944	1101.681	2441.5078	2.2161659	1.1480659	9.90E-05	0.0036692
ENSG000001	1105.27	1725.3025	485.23747	0.2812478	-1.830086	0.001051	0.0248167
ENSG000001	103.11932	0	206.23863	Inf	Inf	1.19E-12	2.05E-10
ENSG000001	1016.2095	781.35006	1251.069	1.6011633	0.6791204	0.0237479	0.2504914
ENSG000001	1830.1836	3040.1544	620.21277	0.204007	-2.293309	1.98E-08	1.84E-06
ENSG000002	12.075998	24.151995	0	0	#NAME?	0.0087715	0.1253246
ENSG000001	71.550827	6.6334149	136.46824	20.572848	4.3626696	0.0002328	0.0072961
ENSG000001	6603.6486	4887.5106	8319.7866	1.7022544	0.7674467	0.0460056	0.3798687
ENSG000002	73.748111	3.7905228	143.7057	37.911841	5.2445766	1.33E-05	0.0006515
ENSG000000	1102.3281	852.32451	1352.3316	1.5866393	0.6659742	0.0321235	0.3041615
ENSG000001	306.37439	195.92357	416.8252	2.1274887	1.0891515	0.0348054	0.3208002
ENSG000001	141.20914	85.71778	196.7005	2.2947456	1.1983342	0.0220199	0.2374977
ENSG000001	521.20534	182.8172	859.59349	4.701929	2.2332528	2.14E-06	0.0001307
ENSG000000	245.35911	56.283658	434.43456	7.7186625	2.9483509	9.35E-07	6.16E-05
ENSG000000	758.56428	1136.0295	381.09909	0.3354658	-1.575762	0.0001327	0.0046518
ENSG000001	404.5325	616.8616	192.20339	0.3115827	-1.682313	0.00014	0.0048611
ENSG000001	37.574246	75.148492	0	0	#NAME?	1.70E-07	1.30E-05
ENSG000001	598.57688	891.96236	305.1914	0.3421573	-1.547269	0.0001465	0.0050301
ENSG000000	139.72347	79.648479	199.79845	2.508503	1.3268267	0.0477952	0.3884039
ENSG000001	1646.242	900.76948	2391.7145	2.6551904	1.4088153	0.0027907	0.0535973
ENSG000001	120.49936	63.227915	177.7708	2.8115873	1.4913848	0.0305963	0.2949644
ENSG000001	110.66733	165.31876	56.015895	0.3388357	-1.561342	0.0163144	0.1956933
ENSG000000	103.49156	44.586143	162.39699	3.6423197	1.8648575	0.0001505	0.0051226
ENSG000001	3519.7922	1818.7935	5220.7909	2.8704692	1.5212866	4.56E-05	0.0018829
ENSG000001	221.94325	121.86806	322.01845	2.642353	1.4018232	0.0033556	0.0619404

ENSG000001	91.76162	176.52484	6.9984042	0.0396454	-4.656701	1.45E-08	1.38E-06
ENSG000001	1855.7735	0	3711.5469	Inf	Inf	1.22E-58	8.24E-55
ENSG000001	237.54493	110.36121	364.72865	3.3048626	1.7245903	0.0045059	0.077124
ENSG000001	390.82899	513.25902	268.39897	0.5229308	-0.935308	0.0469834	0.3852043
ENSG000001	786.23941	1013.4246	559.05424	0.5516486	-0.858179	0.0232203	0.2468472
ENSG000001	1118.2204	1893.9053	342.53555	0.180862	-2.467039	5.73E-06	0.000308
ENSG000001	333.03497	202.53181	463.53812	2.2887176	1.1945394	0.002622	0.0508629
ENSG000002	550.02808	421.97563	678.08053	1.6069187	0.6842969	0.0395135	0.3442612
ENSG000001	759.15546	284.5972	1233.7137	4.3349467	2.1160143	1.84E-05	0.00086
ENSG000001	225.58643	107.66871	343.50415	3.1903806	1.6737285	0.0023878	0.0473519
ENSG000001	10.793983	0	21.587967	Inf	Inf	0.0457196	0.3790499
ENSG000001	284.03305	546.36349	21.70261	0.0397219	-4.653921	4.64E-17	1.46E-14
ENSG000001	46.428715	86.748157	6.1092733	0.0704254	-3.82776	0.0004629	0.0129133
ENSG000000	1006.0005	29.439157	1982.5619	67.344383	6.0734857	2.91E-31	2.81E-28
ENSG000001	1381.6414	2732.5391	30.743736	0.011251	-6.473806	4.59E-44	9.55E-41
ENSG000001	763.02838	541.12382	984.93294	1.8201619	0.8640667	0.039063	0.3424306
ENSG000001	1972.141	3088.5799	855.70216	0.2770536	-1.851763	1.43E-07	1.11E-05
ENSG000001	19.297276	38.594552	0	0	#NAME?	0.0007411	0.0189374
ENSG000002	22.062821	0	44.125642	Inf	Inf	0.0014537	0.0321797
ENSG000001	560.55127	778.81582	342.28673	0.4394964	-1.186077	0.0057542	0.0924857
ENSG000001	258.94211	366.59713	151.28709	0.4126794	-1.276907	0.0151754	0.1855455
ENSG000002	297.03851	185.65002	408.42699	2.1999835	1.1374927	0.0099245	0.1366755
ENSG000001	1681.2999	2181.6483	1180.9514	0.5413116	-0.885469	0.0095292	0.1330592
ENSG000001	112.28901	60.77861	163.7994	2.6950172	1.4302945	0.0290701	0.2851352
ENSG000001	53.410863	26.518554	80.303171	3.0281881	1.5984548	0.0393238	0.3434928
ENSG000001	766.02222	1152.2924	379.75203	0.3295622	-1.601377	0.0006309	0.0167371
ENSG000002	17.139056	0	34.278112	Inf	Inf	0.006249	0.0983728
ENSG000001	398.04729	178.84105	617.25354	3.4514087	1.7871853	3.12E-06	0.0001791
ENSG000002	94.337295	163.47603	25.198556	0.1541422	-2.697666	0.0007584	0.0192952
ENSG000001	1389.114	823.65524	1954.5728	2.3730473	1.2467408	0.002462	0.0484039
ENSG000001	70.812337	120.00805	21.616628	0.1801265	-2.472918	0.0220697	0.2378444
ENSG000000	3300.3532	4060.1533	2540.553	0.6257283	-0.676392	0.0202158	0.2252202
ENSG000001	876.96856	562.21708	1191.72	2.1196795	1.0838461	0.0008547	0.0211428
ENSG000001	543.50388	726.13202	360.87575	0.4969837	-1.00873	0.0397081	0.3450102
ENSG000001	232.22054	3.7905228	460.65056	121.52692	6.9251321	6.22E-16	1.67E-13
ENSG000001	241.35763	135.20253	347.51274	2.5703123	1.3619437	0.0013446	0.0303481
ENSG000001	3563.5897	4501.9256	2625.2537	0.5831402	-0.778085	0.035833	0.3263808
ENSG000001	817.08927	60.006541	1574.172	26.23334	4.7133296	5.17E-17	1.61E-14
ENSG000001	303.22467	604.07833	2.3710159	0.003925	-7.993086	9.07E-21	4.16E-18
ENSG000002	96.983253	169.80364	24.162865	0.1422989	-2.813004	0.0001145	0.0041106
ENSG000002	53.630267	88.493032	18.767501	0.2120789	-2.237327	0.0143187	0.177174
ENSG000000	69.674276	18.393536	120.95502	6.5759525	2.7171999	0.0061917	0.0976412
ENSG000001	443.0488	333.0113	553.0863	1.6608635	0.7319335	0.035072	0.3217228
ENSG000000	94.348879	25.580994	163.11676	6.3764826	2.6727608	0.0089554	0.12728
ENSG000001	103.87936	149.35942	58.399291	0.3909984	-1.354765	0.0463119	0.3817102
ENSG000002	27.047164	0	54.094328	Inf	Inf	0.0103639	0.1407218
ENSG000001	425.65585	234.64115	616.67056	2.6281433	1.3940439	0.0299304	0.2911408
ENSG000001	344.62286	447.30116	241.94456	0.5408986	-0.88657	0.0257332	0.2638753
ENSG000001	236.33362	119.49143	353.17582	2.955658	1.5634794	0.0002	0.0064653
ENSG000001	1004.0919	1329.108	679.07582	0.510926	-0.968814	0.0042612	0.0741561
ENSG000001	827.91064	1340.3342	315.4871	0.2353794	-2.08694	0.0001482	0.0050687

ENSG000001	262.34124	371.95194	152.73054	0.410619	-1.284128	0.0269905	0.2721275
ENSG000001	38.725394	0	77.450789	Inf	Inf	1.10E-05	0.0005537
ENSG000001	196.50281	35.440818	357.56479	10.089067	3.3347209	1.29E-07	1.01E-05
ENSG000001	1241.5452	1586.67	896.42045	0.5649697	-0.823755	0.0167802	0.1995992
ENSG000001	880.75993	1400.0292	361.49065	0.2582022	-1.953427	4.40E-06	0.0002429
ENSG000002	220.00785	298.55336	141.46235	0.473826	-1.077571	0.036284	0.328608
ENSG000001	777.20978	1430.5614	123.85821	0.0865801	-3.52982	1.78E-15	4.55E-13
ENSG000000	1593.1695	3088.9255	97.413566	0.0315364	-4.986839	0.0035524	0.0646906
ENSG000001	15.014913	0	30.029827	Inf	Inf	0.0108899	0.1462488
ENSG000000	191.669	125.65355	257.68446	2.0507535	1.0361541	0.0153257	0.1867305
ENSG000000	1843.6577	1092.5111	2594.8044	2.3750828	1.2479778	0.0015544	0.0339712
ENSG000000	33.032574	7.8918874	58.17326	7.3712734	2.8819139	0.009016	0.1277156
ENSG000001	1538.2203	1219.3778	1857.0629	1.5229593	0.6068774	0.0468074	0.3841844
ENSG000000	1361.02	242.3798	2479.6603	10.230474	3.3548011	6.89E-17	2.12E-14
ENSG000001	144.27086	89.896855	198.64486	2.2096975	1.1438489	0.0202451	0.2253145
ENSG000000	848.07144	1183.7794	512.36346	0.43282	-1.208161	0.0030352	0.0572365
ENSG000001	10570.429	6470.2758	14670.582	2.2673812	1.181027	0.0006812	0.0177541
ENSG000001	16.221253	32.442505	0	0	#NAME?	0.0010814	0.0254135
ENSG000000	467.79382	300.20038	635.38725	2.1165438	1.0817103	0.0181545	0.2097684
ENSG000000	69.191647	0	138.38329	Inf	Inf	0.0001021	0.0037609
ENSG000001	12415.787	24430.97	400.60329	0.0163974	-5.930393	7.79E-21	3.70E-18
ENSG000002	11.32616	0	22.652319	Inf	Inf	0.0370972	0.3326329
ENSG000001	59.192598	21.542234	96.842961	4.4954929	2.1684793	0.0005324	0.0145662
ENSG000001	31.814618	57.459385	6.1698511	0.1073776	-3.219235	0.0165749	0.1979009
ENSG000001	158.35643	227.05004	89.662823	0.3949034	-1.340428	0.0085153	0.1227016
ENSG000001	2083.5048	3323.7703	843.23937	0.2536997	-1.978807	0.0354365	0.3240782
ENSG000001	90.483232	35.440818	145.52565	4.1061593	2.0377896	0.0064066	0.1000972
ENSG000001	11436.808	6951.6138	15922.002	2.2904037	1.1956019	0.0042046	0.0734069
ENSG000001	106.77924	34.570898	178.98758	5.1774063	2.3722295	0.0004889	0.0134858
ENSG000002	459.0026	576.9359	341.0693	0.5911736	-0.758346	0.0299536	0.2911544
ENSG000001	24.153418	0	48.306837	Inf	Inf	0.0007796	0.0196984
ENSG000001	1011.0119	1327.7214	694.30244	0.5229278	-0.935316	0.0109921	0.1470376
ENSG000001	779.46372	1059.7153	499.21213	0.4710814	-1.085952	0.0037243	0.066965
ENSG000001	858.55024	504.93108	1212.1694	2.4006631	1.2634329	0.0101163	0.1386111
ENSG000001	10.615506	0	21.231012	Inf	Inf	0.047643	0.3879832
ENSG000001	5760.8233	3860.4775	7661.1691	1.9845133	0.9887853	0.002813	0.0539449
ENSG000002	35.55123	0	71.10246	Inf	Inf	2.61E-05	0.001156
ENSG000000	170.77205	70.420408	271.12369	3.8500726	1.9448857	0.007203	0.1090775
ENSG000001	30.261938	1.5793845	58.944492	37.321179	5.2219227	0.004819	0.0808898
ENSG000001	1130.0791	1432.5422	827.61605	0.5777254	-0.791544	0.0309933	0.2972042
ENSG000002	346.87749	211.54186	482.21312	2.2795164	1.1887278	0.0069693	0.1065495
ENSG000002	41.407092	5.3699073	77.444277	14.421902	3.8501895	0.0118413	0.1549498
ENSG000001	623.44271	862.67862	384.2068	0.4453649	-1.16694	0.003052	0.0575135
ENSG000001	24.46582	45.461098	3.4705412	0.0763409	-3.7114	0.0289558	0.2846253
ENSG000001	18.626667	37.253334	0	0	#NAME?	0.0007579	0.0192952
ENSG000000	53.571277	0	107.14255	Inf	Inf	3.29E-07	2.37E-05
ENSG000002	1821.9936	2853.312	790.67513	0.2771078	-1.851481	4.10E-07	2.91E-05
ENSG000001	1693.8949	1328.0338	2059.756	1.5509816	0.6331816	0.0372205	0.3335176
ENSG000001	258.47268	158.57742	358.36794	2.2598926	1.1762542	0.013743	0.172096
ENSG000001	86.954091	147.84264	26.065538	0.176306	-2.503847	0.002167	0.0441907
ENSG000000	2016.706	2623.3644	1410.0476	0.5374959	-0.895674	0.0131739	0.1671324

ENSG000001	409.34159	814.70913	3.9740565	0.0048779	-7.679529	0.0483232	0.3914023
ENSG000001	1183.7741	1459.4069	908.14135	0.6222674	-0.684393	0.0492945	0.3964219
ENSG000002	50.724671	1.2635076	100.18583	79.291834	6.3091004	3.03E-05	0.0013053
ENSG000001	169.56529	62.270214	276.86037	4.4461123	2.1525444	3.39E-05	0.0014502
ENSG000001	214.59964	108.15008	321.0492	2.9685527	1.5697597	0.0109115	0.1464227
ENSG000001	1340.9139	604.87339	2076.9544	3.433701	1.7797644	9.80E-05	0.003644
ENSG000001	549.26923	703.77022	394.76824	0.5609334	-0.834099	0.0301131	0.2920755
ENSG000001	63.201469	1.5793845	124.82355	79.033037	6.3043839	3.80E-06	0.0002126
ENSG000001	243.76536	126.46087	361.06986	2.8551905	1.513587	0.0072004	0.1090775
ENSG000001	1295.1657	1023.7701	1566.5613	1.5301885	0.6137094	0.0463127	0.3817102
ENSG000001	98.982499	51.939092	146.0259	2.8114836	1.4913316	0.0338908	0.315378
ENSG000001	2427.7121	118.55387	4736.8704	39.955425	5.3203195	2.27E-30	2.12E-27
ENSG000001	936.56625	3.7905228	1869.342	493.16205	8.945918	9.38E-38	1.34E-34
ENSG000001	40.737064	67.805613	13.668515	0.2015838	-2.310548	0.0394856	0.3441291
ENSG000001	1635.908	2084.8698	1186.9461	0.5693143	-0.812703	0.0435973	0.3668488
ENSG00000(	1745.706	2192.6184	1298.7935	0.5923482	-0.755483	0.0420667	0.3579769
ENSG000001	2361.1333	4572.3526	149.91398	0.0327871	-4.93073	1.26E-31	1.26E-28
ENSG000001	256.30275	158.422	354.18349	2.2356963	1.1607242	0.018301	0.2109215
ENSG00000(	1454.1044	87.161884	2821.0469	32.365602	5.0163894	3.42E-22	1.81E-19
ENSG000001	1068.2025	1423.4444	712.96052	0.50087	-0.997492	0.0130472	0.1658767
ENSG000001	178.94192	124.5505	233.33335	1.8734036	0.9056617	0.0345883	0.3194514
ENSG000001	220.37431	70.808961	369.93967	5.2244753	2.3852862	0.0001104	0.0039956
ENSG00000(	47.497671	21.54727	73.448072	3.4086951	1.7692195	0.0477255	0.3883676
ENSG000002	441.31863	574.86508	307.77217	0.5353816	-0.901361	0.0272745	0.2732253
ENSG000001	141.1382	224.3676	57.9088	0.2580979	-1.95401	0.0009656	0.0233521
ENSG000001	79.79887	37.253334	122.34441	3.2841197	1.7155067	0.0122077	0.1585948
ENSG000001	272.08261	135.82422	408.34101	3.0063933	1.5880337	4.22E-05	0.0017524
ENSG000001	11621.362	18903.527	4339.1958	0.2295442	-2.123156	0.0083223	0.1204974
ENSG000002	389.14547	686.81793	91.473002	0.1331838	-2.90851	7.69E-09	7.66E-07
ENSG000002	19.613153	39.226306	0	0	#NAME?	0.0005382	0.0147106
ENSG000001	883.15128	61.48304	1704.8195	27.728289	4.7932867	1.69E-17	5.85E-15
ENSG000002	65.8745	0	131.749	Inf	Inf	8.98E-09	8.77E-07
ENSG000001	624.71335	465.95519	783.47151	1.681431	0.7496896	0.0463226	0.3817102
ENSG00000(	65.094619	17.689106	112.50013	6.3598539	2.6689936	0.0448917	0.3750584
ENSG000001	612.3897	939.28632	285.49309	0.3039468	-1.718109	5.35E-05	0.0021526
ENSG000001	24.290182	8.2854748	40.29489	4.863317	2.2819406	0.0231318	0.246251
ENSG000001	65.11874	2.5270152	127.71047	50.538068	5.6592986	0.0061994	0.097707
ENSG00000(	364.73147	204.81059	524.65236	2.5616466	1.3570715	0.0001928	0.0062631
ENSG000002	122.20578	35.68402	208.72755	5.8493283	2.548271	0.0016009	0.0347419
ENSG000001	99.931073	40.184007	159.67814	3.9736739	1.9904735	0.0210069	0.2306157
ENSG000001	273.38658	127.56392	419.20924	3.2862681	1.7164502	0.0103415	0.1406287
ENSG000002	415.15503	596.55267	233.75739	0.391847	-1.351638	0.0026526	0.0514196
ENSG000001	327.78669	184.41672	471.15665	2.5548477	1.3532373	0.0409959	0.3512478
ENSG000001	28.888203	1.2635076	56.512898	44.726995	5.4830739	0.0043014	0.0746161
ENSG000001	231.22774	55.65694	406.79855	7.3090355	2.869681	1.14E-05	0.0005724
ENSG000001	331.53852	475.22354	187.85349	0.395295	-1.338998	0.0046989	0.0795221
ENSG000001	66.64249	123.47262	9.8123576	0.0794699	-3.653448	2.15E-05	0.0009772
ENSG000001	2143.7614	2720.5387	1566.9841	0.575983	-0.795902	0.0136246	0.171247
ENSG00000(	573.39981	915.36242	231.43719	0.2528367	-1.983722	4.64E-06	0.0002552
ENSG000002	417.73603	241.7984	593.67367	2.4552423	1.2958654	0.0121934	0.1585609
ENSG000002	93.85731	0.9476307	186.76699	197.08837	7.6226988	1.18E-09	1.30E-07

ENSG000001	21.539893	41.5979	1.481885	0.035624	-4.811005	0.0061828	0.0976157	
ENSG000001	3113.5592	890.30526	5336.8131	5.9943631	2.5836065	7.08E-10	8.01E-08	
ENSG00000(	51.946815	3.7905228	100.10311	26.408787	4.7229461	0.000725	0.0185955	
ENSG000001	1620.6143	1117.4877	2123.7409	1.9004602	0.9263488	0.0284421	0.2811069	
ENSG000002	1259.1566	1626.1971	892.11613	0.5485904	-0.866199	0.0132579	0.1676216	
ENSG000001	322.41033	200.2005	444.62016	2.2208744	1.1511278	0.0459303	0.3797494	
ENSG000001	1289.0589	865.88423	1712.2336	1.9774394	0.9836335	0.0210993	0.2313485	
ENSG000002	47.533952	5.3699073	89.697996	16.703826	4.0621067	0.0049775	0.082523	
ENSG00000(	17.836975	35.673949	0	0	#NAME?	0.0016337	0.0351455	
ENSG000001	1093.0703	1467.2987	718.84179	0.4899083	-1.029416	0.004115	0.0723095	
ENSG000002	49.458857	16.731406	82.186309	4.9120983	2.2963394	0.0070373	0.1072887	
ENSG000001	25106.147	30534.615	19677.68	0.6444384	-0.633886	0.023719	0.2504332	
ENSG000001	475.59928	251.35745	699.84111	2.7842465	1.4772869	0.0049587	0.0823747	
ENSG000001	1268.9716	2502.3297	35.613436	0.0142321	-6.134706	1.18E-35	1.45E-32	
ENSG000001	22149.357	3224.3888	41074.325	12.738639	3.6711392	3.04E-15	7.48E-13	
ENSG000001	332.22358	643.20963	21.237524	0.033018	-4.920602	1.90E-20	8.41E-18	
ENSG000001	266.04513	177.29187	354.79839	2.0012107	1.0008731	0.0464278	0.3821113	
ENSG000001	346.04331	240.59246	451.49415	1.8765931	0.9081159	0.031377	0.2995033	
ENSG000001	1635.3852	294.71248	2976.0579	10.098174	3.3360225	0.0004021	0.0115394	
ENSG000002	58.772526	0	117.54505	Inf	Inf	5.40E-08	4.62E-06	
ENSG00000(	905.29962	1187.5426	623.05667	0.5246605	-0.930544	0.0088787	0.126323	
ENSG000001	729.42289	1063.077	395.76876	0.3722861	-1.425516	0.0005935	0.0159029	
ENSG000001	31.045904	57.46442	4.6273883	0.0805261	-3.634399	0.0041885	0.0731722	
ENSG000002	8.0523434	16.104687	0	0	#NAME?	0.0272348	0.2731653	
ENSG000001	440.82628	560.17932	321.47325	0.5738756	-0.80119	0.0392449	0.3434319	
ENSG000001	438.57925	274.00274	603.15576	2.2012764	1.1383403	0.026765	0.2707082	
ENSG000001	206.52855	117.11984	295.93727	2.5267903	1.3373059	0.0134936	0.1699956	
ENSG000001	708.5286	219.61938	1197.4378	5.4523323	2.4468735	5.71E-07	3.96E-05	
ENSG000001	256.56433	105.4475	407.68116	3.8662004	1.9509164	3.60E-05	0.0015186	
ENSG000001	379.79709	238.70224	520.89195	2.182183	1.1257721	0.0127406	0.1630363	
ENSG000001	520.97811	244.84421	797.11201	3.2555885	1.7029184	3.63E-05	0.0015268	
ENSG000002	32.692657	0	65.385315	Inf	Inf	6.32E-05	0.0024899	
ENSG000002	11.265582	0	22.531163	Inf	Inf	0.0366149	0.3310514	
ENSG000001	101.52827	183.37124	19.685293	0.1073521	-3.219577	2.71E-05	0.0012017	
ENSG00000(	2533.4156	1145.0892	3921.742	3.4248354	1.7760346	1.01E-06	6.60E-05	
ENSG000002	13.810803	27.621606	0	0	#NAME?	0.0356882	0.3255311	
ENSG000001	1358.2248	1998.1497	718.29985	0.3594825	-1.476007	0.0052055	0.0852694	
ENSG000002	14.737751	0	29.475502	Inf	Inf	0.013305	0.1680896	
ENSG000001	80.928793	9.9425698	151.91502	15.279251	3.9335019	2.33E-07	1.71E-05	
ENSG000001	84.417233	39.469507	129.36496	3.2775925	1.7126365	0.0394458	0.3440471	
ENSG000002	84.838938	130.50466	39.173216	0.3001672	-1.736162	0.0230144	0.2454844	
ENSG000001	500.97648	703.17371	298.77924	0.424901	-1.234801	0.0054116	0.0879534	
ENSG00000(	409.87385	114.59786	705.14984	6.153255	2.6213498	9.65E-08	7.73E-06	
ENSG00000(	1012.378	1440.2765	584.47947	0.4058106	-1.301122	0.001166	0.0269267	
ENSG000001	46.821326	78.788629	14.854023	0.18853	-2.407134	0.0084723	0.1221467	
ENSG000001	1705.1168	2654.3693	755.86416	0.2847622	-1.81217	2.92E-06	0.000169	
ENSG00000(	297.91697	439.83022	156.00372	0.3546908	-1.495366	0.007621	0.1131895	
ENSG000001	1080.8667	678.03314	1483.7002	2.1882414	1.1297719	0.0045253	0.0773586	
ENSG000001	161.10281	319.5063	2.6993098	0.0084484	-6.88711	3.69E-07	2.64E-05	
ENSG000001	8335.27	10432.712	6237.8276	0.5979104	-0.741999	0.0476749	0.3881255	
ENSG000001	616.51875	786.70553	446.33197	0.5673431	-0.817707	0.039367	0.3436488	

ENSG000001	548.59063	141.18187	955.99938	6.7714034	2.7594549	5.84E-06	0.0003138
ENSG000001	59.931985	103.971	15.89297	0.1528596	-2.709721	0.0012263	0.0280525
ENSG000001	143.21957	72.38331	214.05582	2.9572538	1.5642581	0.0098461	0.1362193
ENSG000001	88.74816	22.971233	154.52509	6.7268956	2.7499409	0.0011662	0.0269267
ENSG000001	504.07731	326.65633	681.49829	2.0862853	1.0609364	0.0315025	0.3003834
ENSG000001	103.12733	150.93377	55.320878	0.3665242	-1.44802	0.0399334	0.346358
ENSG000001	2806.559	3709.535	1903.583	0.5131595	-0.962521	0.0046474	0.0788817
ENSG00000(	733.03044	932.01108	534.04979	0.573008	-0.803373	0.0256326	0.2635437
ENSG00000:	373.91114	273.79697	474.02531	1.7313023	0.7918576	0.0231747	0.2465135
ENSG000001	97.539407	133.34252	61.736296	0.4629903	-1.110946	0.0472485	0.3862788
ENSG00000(	865.79295	533.99175	1197.5941	2.2427203	1.1652497	0.0273699	0.2738106
ENSG000001	3116.507	1417.7658	4815.2482	3.3963636	1.7639909	2.09E-06	0.0001284
ENSG00000(	221.45353	135.912	306.99507	2.2587783	1.1755427	0.0044375	0.0762914
ENSG000001	828.44809	489.33512	1167.5611	2.3860153	1.2546033	0.0028092	0.0539143
ENSG000001	5699.3416	3138.0554	8260.6277	2.6324034	1.3963806	2.21E-05	0.000997
ENSG000001	289.28442	91.263249	487.3056	5.3395601	2.4167209	9.84E-05	0.0036542
ENSG000001	310.96296	436.85709	185.06884	0.423637	-1.239099	0.0065441	0.1015757
ENSG000001	903.95673	1678.6729	129.24055	0.0769897	-3.69919	1.17E-12	2.03E-10
ENSG000001	166.31013	5.0540304	327.56624	64.812875	6.0182085	3.52E-11	5.20E-09
ENSG000001	40.883715	16.337818	65.429613	4.004795	2.0017284	0.0350321	0.3217228
ENSG000001	5081.1962	9881.6797	280.71263	0.0284074	-5.13759	1.58E-37	2.14E-34
ENSG000001	474.33349	741.04873	207.61825	0.2801682	-1.835635	1.50E-05	0.0007203
ENSG000001	1293.1446	1800.8518	785.43738	0.4361477	-1.197111	0.0007006	0.0181356
ENSG000001	134.09759	267.03834	1.1568471	0.0043321	-7.850705	5.82E-18	2.13E-15
ENSG000001	68.429401	105.78352	31.075286	0.293763	-1.767275	0.0207198	0.2289498
ENSG000001	3687.2515	246.9726	7127.5304	28.859599	4.8509794	1.95E-28	1.65E-25
ENSG000001	434.15889	663.07967	205.23811	0.3095226	-1.691884	0.0003144	0.0094105
ENSG000001	188.4431	303.24401	73.642186	0.2428479	-2.041875	0.0099164	0.1366755
ENSG000001	2438.2711	1558.3801	3318.1622	2.1292381	1.0903373	0.0024424	0.0481391
ENSG000001	633.70774	70.415373	1197.0001	16.99913	4.087389	7.58E-15	1.77E-12
ENSG000001	53.073369	97.487971	8.6587666	0.0888188	-3.492991	0.0003988	0.0114699
ENSG000001	28.263532	1.2635076	55.263556	43.738206	5.4508222	0.0047239	0.0797471
ENSG000001	33.219582	13.25676	53.182405	4.0117198	2.0042208	0.0287307	0.2831327
ENSG000001	358.26775	497.54288	218.99261	0.4401482	-1.183939	0.0189156	0.2152057
ENSG000001	348.98441	229.36624	468.60258	2.043032	1.0307118	0.0232859	0.247075
ENSG00000:	436.57897	287.96393	585.19402	2.0321782	1.0230269	0.0261468	0.2668991
ENSG00000:	22.413264	0	44.826528	Inf	Inf	0.001143	0.0265281
ENSG000001	1263.077	566.38391	1959.7702	3.4601445	1.7908323	1.61E-05	0.0007622
ENSG00000:	44.241527	84.241282	4.2417726	0.0503527	-4.311788	0.0007337	0.018785
ENSG00000(	94.678193	153.68888	35.667502	0.232076	-2.107331	0.0067606	0.1042592
ENSG000001	2367.9337	3326.5396	1409.3279	0.4236618	-1.239015	0.0024627	0.0484039
ENSG000001	2316.68	3269.8422	1363.5177	0.416998	-1.261888	0.0006294	0.0167143
ENSG000001	74.005168	40.484778	107.52556	2.6559502	1.4092281	0.0328366	0.3090751
ENSG00000:	11.742064	0	23.484128	Inf	Inf	0.0332091	0.31123
ENSG000001	42.058822	0	84.117643	Inf	Inf	4.13E-06	0.0002289
ENSG00000(	139.56544	91.481275	187.64961	2.0512352	1.0364929	0.0458254	0.3791447
ENSG00000:	63.056857	92.842633	33.271081	0.3583599	-1.480519	0.0348571	0.3210828
ENSG000001	260.08916	512.82296	7.355359	0.0143429	-6.123521	1.29E-21	6.60E-19
ENSG00000:	104.88211	55.729615	154.0346	2.763963	1.4667383	0.0428301	0.361856
ENSG000001	2341.7865	3053.4471	1630.126	0.5338642	-0.905455	0.0063763	0.0997819
ENSG000001	106.32026	21.469559	191.17096	8.9042797	3.1544989	3.52E-05	0.0014948

ENSG000001	680.72774	387.34716	974.10833	2.5148199	1.3304551	0.0022778	0.045795
ENSG000002	19.242356	0	38.484711	Inf	Inf	0.0031206	0.058488
ENSG00000(	2163.3886	2762.8259	1563.9512	0.5660694	-0.820949	0.0121326	0.158151
ENSG000001	817.04228	116.52837	1517.5562	13.023063	3.7029969	1.32E-12	2.24E-10
ENSG000001	2244.9564	1202.4989	3287.4138	2.7338186	1.4509175	0.0004188	0.0118671
ENSG000002	30.850701	2.8428921	58.858509	20.703744	4.3718197	0.0149164	0.1829795
ENSG000001	58.083664	13.339505	102.82782	7.7085185	2.9464536	0.002505	0.0490482
ENSG000001	834.9747	1550.4385	119.51092	0.077082	-3.697462	0.0001496	0.005106
ENSG000001	19.867027	0	39.734053	Inf	Inf	0.0027132	0.0523695
ENSG000001	122.31086	0	244.62172	Inf	Inf	2.45E-14	5.56E-12
ENSG000001	154.75909	204.07377	105.44441	0.5166975	-0.952608	0.0484121	0.39177
ENSG000001	1157.9268	21.542234	2294.3115	106.50295	6.7347495	1.51E-05	0.0007203
ENSG000001	27390.931	13744.982	41036.881	2.9855899	1.578016	5.46E-06	0.0002959
ENSG000001	814.95143	1318.2322	311.67066	0.2364308	-2.08051	1.46E-06	9.29E-05
ENSG00000(	422.09358	571.13213	273.05502	0.4780943	-1.064633	0.0155519	0.1889766
ENSG000001	62.644163	97.565682	27.722645	0.2841434	-1.815309	0.0456016	0.3785356
ENSG00000(	6316.0996	4601.3723	8030.8268	1.7453112	0.8034843	0.0051401	0.0843512
ENSG000001	1702.1641	411.19838	2993.1298	7.2790408	2.8637484	4.06E-11	5.90E-09
ENSG000001	26.946529	0	53.893059	Inf	Inf	0.0034764	0.0635322
ENSG000001	15.430818	0	30.861636	Inf	Inf	0.0097541	0.1352231
ENSG000001	567.79794	216.80384	918.79203	4.2378955	2.083348	3.01E-08	2.71E-06
ENSG00000(	275.24814	381.91968	168.5766	0.4413928	-1.179865	0.0102818	0.1401689
ENSG000001	128.95465	63.621503	194.2878	3.0538072	1.610609	0.0114876	0.1520129
ENSG000001	87.369807	2.5270152	172.2126	68.14862	6.0906125	2.07E-07	1.55E-05
ENSG000001	70.04267	0	140.08534	Inf	Inf	2.42E-09	2.59E-07
ENSG000002	51.219878	102.14338	0.296377	0.0029016	-8.428947	8.71E-09	8.57E-07
ENSG000001	12.779384	0	25.558767	Inf	Inf	0.0236689	0.2500994
ENSG000002	714.8079	974.2681	455.34769	0.4673741	-1.09735	0.0078548	0.1157091
ENSG000001	35.619942	59.913725	11.32616	0.1890411	-2.403228	0.037444	0.334966
ENSG000001	5351.1748	2880.5703	7821.7793	2.7153579	1.4411424	0.000735	0.0187988
ENSG000002	523.76899	150.6805	896.85748	5.9520474	2.573386	0.0276433	0.2753268
ENSG000001	84.354607	162.78167	5.9275398	0.036414	-4.779361	3.19E-08	2.85E-06
ENSG000002	180.65935	94.261562	267.05714	2.8331499	1.5024069	0.0356303	0.3253011
ENSG000001	78.250199	129.1282	27.372202	0.211977	-2.238021	0.0384822	0.3395393
ENSG000001	310.27928	207.07426	413.48429	1.9967923	0.9976843	0.0298989	0.2910408
ENSG000001	175.22538	36.709361	313.7414	8.546632	3.095356	1.21E-05	0.0005984
ENSG000001	589.64613	403.4015	775.89077	1.9233711	0.9436371	0.0057158	0.0920682
ENSG000001	495.59669	623.826	367.36738	0.588894	-0.76392	0.0293519	0.2870586
ENSG00000(	327.47938	489.64881	165.30995	0.3376092	-1.566574	0.0294102	0.2874306
ENSG00000(	303.73834	430.20353	177.27316	0.4120681	-1.279045	0.0183975	0.2115835
ENSG000001	1569.2744	1924.2618	1214.287	0.6310404	-0.664196	0.0288326	0.2840333
ENSG000001	12.811301	0	25.622601	Inf	Inf	0.0248921	0.2587806
ENSG00000(	1135.1374	487.34922	1782.9256	3.6584148	1.8712186	8.26E-08	6.75E-06
ENSG000002	11.918913	0	23.837827	Inf	Inf	0.0305603	0.294933
ENSG000001	142.43131	210.06032	74.802289	0.3560991	-1.489649	0.0159027	0.1922031
ENSG000001	754.94408	499.73388	1010.1543	2.0213844	1.0153437	0.0067654	0.1042592
ENSG000001	164.03742	82.797178	245.27767	2.9623916	1.5667624	0.0027193	0.0524506
ENSG000002	14.191244	0	28.382489	Inf	Inf	0.0396885	0.345008
ENSG000002	17.225038	0	34.450077	Inf	Inf	0.0056458	0.091158
ENSG000001	1695.5512	877.04347	2514.0589	2.8665157	1.5192982	0.0004129	0.0117626
ENSG000001	46.071176	88.021734	4.120617	0.0468136	-4.416928	4.75E-05	0.0019464

ENSG000001	9987.913	3217.5393	16758.287	5.2084171	2.380845	6.34E-14	1.31E-11
ENSG000002	124.9603	75.68743	174.23317	2.3020094	1.2028937	0.0102001	0.1394774
ENSG000001	253.61313	106.08429	401.14198	3.7813515	1.918902	0.0001496	0.005106
ENSG000001	42.058822	0	84.117643	Inf	Inf	4.13E-06	0.0002289
ENSG000002	12.422429	0	24.844858	Inf	Inf	0.0257189	0.2638287
ENSG000001	438.36867	213.26659	663.47075	3.1109924	1.6373749	0.0001765	0.0058248
ENSG000000	40.048396	75.469404	4.6273883	0.0613148	-4.027622	0.0001897	0.0061782
ENSG000001	35.341597	60.156927	10.526267	0.1749801	-2.514737	0.0155343	0.1888467
ENSG000001	2116.6807	3337.2621	896.09931	0.2685133	-1.896934	1.27E-07	1.00E-05
ENSG000001	55.275786	8.5236412	102.02793	11.969994	3.5813505	0.0002496	0.007729
ENSG000001	388.96056	290.21753	487.7036	1.680476	0.7488699	0.0159803	0.1928398
ENSG000001	244.90762	81.834442	407.9808	4.9854412	2.3177212	2.17E-08	1.99E-06
ENSG000000	183.39345	241.41488	125.37201	0.5193218	-0.945299	0.0376729	0.3359033
ENSG000001	24819.228	8670.8004	40967.655	4.7247836	2.2402483	1.61E-08	1.52E-06
ENSG000001	266.78696	374.66962	158.9043	0.4241184	-1.237461	0.0052371	0.0854778
ENSG000002	32.371282	3.7905228	60.952041	16.080115	4.0072058	0.0190853	0.2160956
ENSG000001	1414.0984	872.52116	1955.6756	2.2414077	1.1644051	0.0220028	0.2374069
ENSG000001	18.157887	36.315773	0	0	#NAME?	0.0002503	0.0077328
ENSG000001	1296.6056	1699.9943	893.21698	0.5254235	-0.928447	0.0129187	0.1645144
ENSG000002	505.82543	686.26674	325.38411	0.4741365	-1.076626	0.0051121	0.0840055
ENSG000001	49.621759	93.459282	5.7842354	0.0618904	-4.01414	0.0001061	0.0038703
ENSG000001	111.86689	29.050605	194.68318	6.7015192	2.7444882	0.0006283	0.0167017
ENSG000001	3018.9725	4147.3498	1890.5951	0.4558562	-1.133349	0.0010454	0.0247295
ENSG000001	1222.3896	1584.1754	860.60378	0.5432503	-0.880311	0.0177619	0.2075715
ENSG000001	1088.223	640.1034	1536.3426	2.4001475	1.2631231	0.0108219	0.1454798
ENSG000001	382.04085	150.26677	613.81492	4.0848346	2.0302777	2.14E-07	1.59E-05
ENSG000001	1608.9224	0	3217.8448	Inf	Inf	4.52E-56	2.04E-52
ENSG000002	528.9655	198.56354	859.36746	4.3279216	2.1136744	0.0002502	0.0077328
ENSG000001	203.11514	127.15522	279.07506	2.1947589	1.1340624	0.0122648	0.1590305
ENSG000001	79.579172	0	159.15834	Inf	Inf	0.0012303	0.0280953
ENSG000001	18.520628	0	37.041255	Inf	Inf	0.0115496	0.1524926
ENSG000002	11.526989	23.053979	0	0	#NAME?	0.0044117	0.0759938
ENSG000000	148.84853	275.07339	22.623658	0.0822459	-3.603912	0.0082492	0.11974
ENSG000001	474.58597	648.43922	300.73272	0.4637794	-1.108489	0.0060658	0.0961213
ENSG000002	866.02801	633.07136	1098.9847	1.735957	0.7957312	0.034605	0.3194619
ENSG000001	293.43704	133.01153	453.86255	3.4122045	1.7707041	0.0010215	0.0244415
ENSG000001	12874.214	21917.106	3831.3223	0.1748097	-2.516143	0.0005521	0.0150017
ENSG000001	369.04535	566.66235	171.42834	0.3025229	-1.724884	8.30E-05	0.0031512
ENSG000001	254.10202	9.155395	499.04865	54.508696	5.7684145	3.94E-17	1.29E-14
ENSG000000	109.4772	172.57889	46.375502	0.2687206	-1.895821	0.0016216	0.0349988
ENSG000002	154.74505	102.28873	207.20136	2.0256519	1.0183863	0.0210279	0.2306596
ENSG000001	4141.4767	5287.3035	2995.65	0.5665742	-0.819663	0.0058311	0.0933703
ENSG000001	347.44353	239.4117	455.47536	1.9024775	0.9278794	0.0404686	0.3484538
ENSG000001	100.37504	44.290406	156.45968	3.5325862	1.8207248	0.0460149	0.3798687
ENSG000002	112.83516	195.36953	30.300799	0.1550948	-2.688778	0.0001945	0.0063104
ENSG000001	229.65155	443.64592	15.657171	0.035292	-4.824513	5.69E-16	1.55E-13
ENSG000001	219.38954	161.0843	277.69479	1.7239097	0.7856842	0.0311503	0.2982863
ENSG000000	1873.132	2652.8519	1093.4121	0.4121648	-1.278707	0.032947	0.3098986
ENSG000001	210.03626	377.46719	42.605328	0.1128716	-3.147245	1.99E-07	1.49E-05
ENSG000001	319.51225	624.67075	14.353763	0.0229781	-5.443595	1.30E-22	7.18E-20
ENSG000001	80.649051	24.472907	136.82519	5.5908844	2.4830765	0.007384	0.1107034

ENSG000002	559.82674	207.9895	911.66399	4.3832212	2.1319915	1.36E-06	8.75E-05
ENSG000001	1206.3901	2371.3285	41.451737	0.0174804	-5.838119	1.78E-17	6.08E-15
ENSG000001	447.65582	322.34919	572.96245	1.7774589	0.8298162	0.0135178	0.1702206
ENSG000002	183.42474	59.053875	307.79561	5.2121153	2.381869	0.0011718	0.0269621
ENSG000001	106.12115	45.78201	166.46028	3.6359321	1.8623253	0.0086754	0.1244117
ENSG000001	794.16908	1462.6125	125.72571	0.0859597	-3.540196	7.32E-06	0.0003824
ENSG000001	23.053893	0	46.107786	Inf	Inf	0.0010184	0.0244103
ENSG000001	45.784021	9.9425698	81.625472	8.2096956	3.0373287	0.0003604	0.0105441
ENSG000002	2400.0947	3352.53	1447.6594	0.431811	-1.211528	0.0003359	0.0099349
ENSG000001	760.5839	474.76953	1046.3983	2.2040131	1.1401328	0.0009841	0.0237362
ENSG000001	1964.821	2936.2762	993.36568	0.338308	-1.563591	0.0001018	0.0037519
ENSG000001	357.40081	239.32895	475.47267	1.986691	0.9903675	0.0213376	0.2329228
ENSG000001	130.89244	197.18708	64.597803	0.3275965	-1.610008	0.0192442	0.2172087
ENSG000001	175.12713	0.9476307	349.30663	368.61051	8.5259534	3.94E-12	6.31E-10
ENSG000001	163.69895	81.922222	245.47568	2.996448	1.5832533	0.0007109	0.0183401
ENSG000001	883.89831	1175.8602	591.93645	0.5034072	-0.990202	0.0063263	0.0991284
ENSG000001	43.473698	81.548775	5.3986197	0.0662011	-3.917001	0.0001319	0.0046282
ENSG000001	173.27542	45.227967	301.32287	6.6623129	2.7360231	1.09E-05	0.0005498
ENSG000001	455.65625	690.39832	220.91418	0.3199808	-1.643943	0.0067624	0.1042592
ENSG000002	81.839122	0	163.67824	Inf	Inf	1.76E-10	2.25E-08
ENSG000001	683.32259	391.11032	975.53486	2.4942703	1.3186178	0.0182312	0.2103619
ENSG000001	848.40382	1203.4566	493.35104	0.409945	-1.286498	0.0009079	0.0221548
ENSG000001	1338.6779	1661.8062	1015.5497	0.611112	-0.710491	0.0303446	0.2936274
ENSG000002	134.78453	92.4138	177.15526	1.9169784	0.9388341	0.0271831	0.2730012
ENSG000001	812.85399	549.86549	1075.8425	1.9565558	0.9683162	0.0116159	0.1529216
ENSG000001	1382.6598	831.32121	1933.9984	2.3264153	1.2181087	0.0004371	0.0123079
ENSG000001	571.2145	754.9171	387.51189	0.5133171	-0.962078	0.0147365	0.1815127
ENSG000001	433.87712	314.70051	553.05374	1.757397	0.8134402	0.0223099	0.2399559
ENSG000002	33.386615	62.917073	3.8561569	0.0612895	-4.028216	0.0006463	0.0170125
ENSG000000	873.59693	629.67946	1117.5144	1.7747353	0.8276039	0.0345244	0.3191881
ENSG000001	283.93148	110.11298	457.74998	4.1570939	2.0555753	5.71E-05	0.0022909
ENSG000002	225.98547	19.418877	432.55207	22.274824	4.4773422	5.15E-11	7.26E-09
ENSG000001	3933.3453	5641.0677	2225.6229	0.3945393	-1.341759	0.0002493	0.007729
ENSG000001	52.755098	101.26842	4.2417726	0.0418864	-4.577373	2.28E-05	0.0010217
ENSG000001	2852.4986	4735.0844	969.91282	0.2048354	-2.287463	0.0003668	0.010697
ENSG000001	8.5236412	17.047282	0	0	#NAME?	0.0450828	0.3761901
ENSG000001	326.367	500.0699	152.6641	0.3052855	-1.711769	0.0007733	0.0195758
ENSG000001	273.48244	183.27843	363.68645	1.9843385	0.9886582	0.0178529	0.2080613
ENSG000001	264.86209	30.464498	499.25969	16.388246	4.0345895	1.60E-12	2.66E-10
ENSG000001	472.61472	633.78871	311.44073	0.4913952	-1.025044	0.0265337	0.2691259
ENSG000002	18.735584	0	37.471169	Inf	Inf	0.0034114	0.0625439
ENSG000001	1653.7207	2798.9784	508.46301	0.1816602	-2.460686	1.41E-10	1.84E-08
ENSG000001	80.528342	121.41187	39.644814	0.3265316	-1.614705	0.026323	0.2679777
ENSG000001	676.9252	474.94224	878.90816	1.850558	0.8879603	0.0360246	0.3272442
ENSG000001	66.393105	25.891836	106.89437	4.1284973	2.0456168	0.0290101	0.2849075
ENSG000001	165.01464	50.281997	279.74728	5.5635674	2.4760102	0.0001109	0.004
ENSG000001	112.85379	172.27309	53.434485	0.3101731	-1.688854	0.0461942	0.3811161
ENSG000001	137.64144	50.68062	224.60227	4.431719	2.1478664	0.0407077	0.3498224
ENSG000001	91.788561	135.95228	47.624844	0.3503056	-1.513314	0.0362345	0.3282694
ENSG000000	298.31968	539.26599	57.373368	0.1063916	-3.232544	1.60E-05	0.0007605
ENSG000001	252.90002	149.80051	355.99954	2.3764908	1.2488328	0.0013887	0.0311355

ENSG000001	306.90894	107.96948	505.84839	4.6851055	2.2280815	0.0103017	0.1403703
ENSG00000(	13.488409	0	26.976818	Inf	Inf	0.0177583	0.2075715
ENSG000001	208.13642	68.530182	347.74267	5.0742995	2.3432087	0.0011317	0.0263155
ENSG000002	259.71469	91.879897	427.54948	4.6533517	2.2182702	0.0079593	0.1169301
ENSG000001	1435.1282	927.21755	1943.0389	2.0955588	1.067335	0.0075686	0.1127211
ENSG000001	400.36195	319.34584	481.37806	1.5073879	0.5920507	0.0433673	0.3652541
ENSG000001	360.06474	212.31393	507.81555	2.3918146	1.2581056	0.0017812	0.037687
ENSG00000(	629.6715	364.25072	895.09228	2.4573522	1.2971046	0.0128424	0.163928
ENSG000002	729.92559	1097.4076	362.44362	0.3302726	-1.598271	2.86E-05	0.0012477
ENSG000001	827.10273	14.913855	1639.2916	109.91737	6.7802755	1.02E-13	2.00E-11
ENSG000001	11.160385	0	22.320769	Inf	Inf	0.0483486	0.3914385
ENSG00000(	1317.3942	810.31792	1824.4705	2.251549	1.1709179	0.000652	0.0171459
ENSG000001	3338.5134	4125.4146	2551.6122	0.6185105	-0.69313	0.016308	0.1956933
ENSG000001	813.22662	452.53798	1173.9153	2.5940702	1.3752175	0.0006242	0.016608
ENSG000001	847.15938	1198.781	495.53771	0.413368	-1.274501	0.0012064	0.0276421
ENSG000002	75.520965	27.709387	123.33254	4.4509301	2.1541069	0.04763	0.3879832
ENSG000001	467.96002	592.39373	343.5263	0.5798952	-0.786136	0.0369069	0.3323387
ENSG000001	1812.1019	3605.1973	19.006556	0.005272	-7.567437	0.0003489	0.0102741
ENSG000001	860.88295	496.13906	1225.6268	2.4703293	1.3047033	0.0037026	0.0667084
ENSG00000(	312.11937	119.99798	504.24077	4.2020773	2.0711027	0.0005555	0.0150772
ENSG000002	28.98221	54.704274	3.2601469	0.0595958	-4.068645	0.0037013	0.0667084
ENSG000001	642.09638	149.80051	1134.3922	7.5726861	2.9208051	5.79E-12	9.11E-10
ENSG000001	169.81037	329.92302	9.6977141	0.0293939	-5.088341	1.37E-07	1.07E-05
ENSG000002	457.06534	578.57789	335.55278	0.5799613	-0.785971	0.0378153	0.3365074
ENSG000001	390.52298	237.75964	543.28632	2.2850233	1.1922089	0.01186	0.1551195
ENSG000001	485.28818	694.1687	276.40766	0.3981851	-1.328489	0.0015554	0.0339712
ENSG000001	1480.7545	1951.9539	1009.555	0.5172023	-0.951199	0.0214763	0.2339659
ENSG000001	296.15565	467.8382	124.47311	0.2660602	-1.910176	0.0007073	0.0182645
ENSG000001	248.17553	40.81576	455.5353	11.16077	3.4803646	1.28E-07	1.01E-05
ENSG00000(	2458.4848	3101.4855	1815.4841	0.5853595	-0.772605	0.0185095	0.2124197
ENSG000001	2308.8338	1549.0425	3068.6252	1.9809818	0.9862157	0.0049792	0.082523
ENSG000001	12.779384	0	25.558767	Inf	Inf	0.0236689	0.2500994
ENSG000001	31.558991	4.1063997	59.011582	14.370638	3.8450522	0.031607	0.300956
ENSG00000(	2519.9409	3140.85	1899.0318	0.6046235	-0.725891	0.018356	0.2112858
ENSG00000(	1404.6648	856.75971	1952.5699	2.2790169	1.1884116	0.006379	0.0997819
ENSG000001	1071.8314	65.108071	2078.5548	31.924687	4.9966006	6.54E-28	5.36E-25
ENSG000001	33.770764	58.650218	8.8913097	0.1515989	-2.721669	0.0331825	0.31123
ENSG000001	1292.3788	1627.9844	956.77323	0.5877042	-0.766838	0.0428272	0.361856
ENSG000002	146.91341	0	293.82682	Inf	Inf	1.76E-16	5.08E-14
ENSG000001	186.68132	29.526938	343.8357	11.644814	3.5416157	1.38E-06	8.83E-05
ENSG00000(	158.20653	242.19702	74.216047	0.3064284	-1.706378	0.0022691	0.0457623
ENSG000001	257.28939	381.45342	133.12537	0.3489951	-1.518721	0.0015852	0.0344449
ENSG000001	148.46786	89.591049	207.34467	2.3143458	1.2106045	0.0389101	0.3415327
ENSG000001	420.53269	239.81032	601.25506	2.5072109	1.3260834	0.0038135	0.0681163
ENSG000001	1413.2569	880.03171	1946.482	2.2118317	1.1452416	0.0073524	0.1103516
ENSG000001	846.77565	587.91607	1105.6352	1.8806005	0.9111934	0.0153016	0.1865211
ENSG00000(	366.10876	214.56031	517.65721	2.412642	1.2706138	0.0143176	0.177174
ENSG00000(	1214.4259	1529.3236	899.52817	0.588187	-0.765653	0.0141268	0.1756023
ENSG000001	276.37462	26.285423	526.46382	20.028737	4.3239995	2.29E-06	0.0001371
ENSG000001	292.51913	214.05377	370.98448	1.7331369	0.7933856	0.0228352	0.2439575
ENSG000001	193.99967	131.81567	256.18368	1.9434995	0.9586567	0.0401326	0.3468208

ENSG000001	464.66345	663.0221	266.3048	0.401653	-1.315979	0.0028428	0.0544052
ENSG000001	690.90724	411.81503	969.99945	2.3554251	1.2359874	0.0032746	0.0607777
ENSG000001	254.53485	123.04882	386.02088	3.1371359	1.649448	0.0016338	0.0351455
ENSG000001	2747.6312	3568.9072	1926.3551	0.5397605	-0.889609	0.011499	0.1520148
ENSG000001	444.08583	622.45961	265.71204	0.4268744	-1.228117	0.0038977	0.0693014
ENSG00000(	2313.4204	2875.4055	1751.4354	0.609109	-0.715228	0.0392914	0.3434319
ENSG000001	253.04109	5.3699073	500.71227	93.244117	6.5429408	9.52E-16	2.48E-13
ENSG000001	458.7278	187.34236	730.11324	3.8972138	1.9624431	0.0004162	0.0118069
ENSG000002	32.517722	0.6317538	64.403689	101.94428	6.6716371	0.0005976	0.0159963
ENSG000002	322.31805	126.9372	517.69889	4.078386	2.0279983	0.0002418	0.0075377
ENSG000002	114.38492	57.542131	171.22772	2.975693	1.5732257	0.0147559	0.1816691
ENSG000002	63.2016	9.476307	116.92689	12.338867	3.625138	0.0167991	0.1996483
ENSG000001	886.6162	1200.3683	572.86409	0.4772403	-1.067212	0.0060289	0.0957203
ENSG000001	176.75292	282.96747	70.538367	0.2492808	-2.004156	0.0177947	0.2076811
ENSG000002	4741.6478	2634.2806	6849.0149	2.5999565	1.3784875	4.91E-05	0.0019959
ENSG00000(	96.369236	43.021864	149.71661	3.4800122	1.7990923	0.025642	0.2635437
ENSG000002	45.312997	12.945918	77.680076	6.0003529	2.5850474	0.0225713	0.2418522
ENSG000002	11.891881	0	23.783761	Inf	Inf	0.033035	0.3104837
ENSG000001	69.047801	120.1534	17.942204	0.1493275	-2.743448	0.0004251	0.0120212
ENSG000001	20.10575	37.097913	3.1135864	0.0839289	-3.574689	0.0336019	0.3136598
ENSG000001	678.81497	821.97582	535.65412	0.6516665	-0.617794	0.0486402	0.3932631
ENSG000001	1393.6396	1010.0824	1777.1968	1.7594573	0.8151305	0.027629	0.2753268
ENSG000001	160.79041	28.097939	293.48288	10.444997	3.3847402	1.40E-07	1.09E-05
ENSG000001	14.734495	0	29.46899	Inf	Inf	0.0122544	0.1589727
ENSG000001	2361.3975	3942.7465	780.04853	0.1978439	-2.337565	8.98E-10	1.00E-07
ENSG000001	149.15382	62.43067	235.87697	3.7782227	1.9177077	0.0004854	0.0134349
ENSG000002	67.472978	0	134.94596	Inf	Inf	0.0201725	0.2249205
ENSG000001	12.124424	0	24.248848	Inf	Inf	0.0272271	0.2731653
ENSG000001	64.933151	38.19593	91.670372	2.4000037	1.2630366	0.0160676	0.1937632
ENSG000001	82.457169	25.570924	139.34341	5.4492914	2.4460686	0.0017314	0.0368518
ENSG000001	39.48523	0	78.970459	Inf	Inf	0.0051805	0.0849114
ENSG000001	92.520874	15.86652	169.17523	10.662403	3.4144606	7.73E-05	0.0029535
ENSG000002	300.29755	106.39009	494.205	4.6452163	2.2157458	7.45E-07	5.04E-05
ENSG000002	48.578613	12.153708	85.003518	6.9940398	2.806126	0.0021035	0.0431558
ENSG000001	423.27443	585.41927	261.1296	0.4460557	-1.164704	0.0091485	0.1290092
ENSG000001	1205.3572	1500.2125	910.50192	0.6069153	-0.720433	0.0308128	0.2960502
ENSG000001	610.94584	391.49099	830.4007	2.1211234	1.0848286	0.0013261	0.0299553
ENSG000001	1074.319	1386.8508	761.78715	0.5492928	-0.864353	0.0380812	0.3376529
ENSG000001	3434.4727	4532.5167	2336.4288	0.5154816	-0.956007	0.002191	0.0445451
ENSG000001	589.52005	892.55887	286.48122	0.3209662	-1.639507	0.0001758	0.0058145
ENSG000001	780.18352	477.49225	1082.8748	2.2678374	1.1813172	0.0039348	0.0697314
ENSG000001	2324.1644	649.57971	3998.7492	6.1559022	2.6219703	2.04E-10	2.56E-08
ENSG000001	6140.4979	11977.889	303.107	0.0253055	-5.304403	0.0007571	0.0192952
ENSG000002	253.8455	145.22785	362.46315	2.495824	1.3195162	0.0022688	0.0457623
ENSG000001	47.118193	94.236386	0	0	#NAME?	0.0002055	0.0066151
ENSG000001	818.23983	1089.4876	546.99202	0.5020635	-0.994058	0.0286065	0.2822168
ENSG000001	455.12752	342.07891	568.17612	1.6609504	0.732009	0.0208051	0.2295176
ENSG00000(	778.51359	1108.8792	448.14802	0.404145	-1.307055	0.001129	0.0263076
ENSG000001	168.52515	0	337.0503	Inf	Inf	3.35E-18	1.24E-15
ENSG000001	564.14795	737.99284	390.30305	0.5288711	-0.919012	0.0276135	0.2753268
ENSG000001	259.60215	191.63154	327.57275	1.7093885	0.7734803	0.0208742	0.2298113

ENSG000002	81.847079	2.8428921	160.85127	56.580152	5.8222241	9.30E-07	6.14E-05
ENSG000002	21.76156	0	43.52312	Inf	Inf	0.0014122	0.0315053
ENSG000001	86.595289	139.2564	33.934181	0.2436813	-2.036933	0.0018109	0.0380766
ENSG000001	210.72106	319.15299	102.28913	0.3205019	-1.641595	0.0028556	0.0545341
ENSG000001	15.157056	30.314112	0	0	#NAME?	0.0011714	0.0269621
ENSG000002	301.48901	591.91958	11.058443	0.0186823	-5.742181	2.42E-13	4.42E-11
ENSG000001	928.25049	1329.9772	526.52376	0.3958893	-1.336831	0.0027011	0.05221
ENSG000001	823.21952	587.77794	1058.6611	1.8011243	0.8488977	0.0062931	0.0986942
ENSG000001	956.51117	1321.2032	591.81919	0.4479396	-1.158624	0.0039567	0.0699832
ENSG000001	300.72779	21.309103	580.14649	27.225289	4.7668754	2.02E-15	5.07E-13
ENSG000001	641.5189	1028.1478	254.89004	0.2479119	-2.012101	5.26E-07	3.69E-05
ENSG000001	1464.0719	1833.2843	1094.8595	0.5972121	-0.743685	0.0216809	0.2355305
ENSG000001	1148.0122	1466.3108	829.71348	0.565851	-0.821506	0.029092	0.2851896
ENSG000000	871.81388	532.09867	1211.5291	2.276888	1.1870633	0.0016198	0.0349988
ENSG000001	218.84884	152.80386	284.89382	1.8644413	0.8987434	0.0181305	0.2096746
ENSG000001	7374.2692	2341.713	12406.825	5.2981837	2.4054979	2.35E-08	2.15E-06
ENSG000001	45.84007	0	91.680141	Inf	Inf	1.49E-06	9.42E-05
ENSG000001	488.50976	671.66591	305.3536	0.4546213	-1.137263	0.0245172	0.2561626
ENSG000001	726.83882	569.48225	884.19539	1.5526303	0.6347143	0.0241169	0.2533495
ENSG000001	53.035862	99.962451	6.1092733	0.0611157	-4.032314	0.0459825	0.3798687
ENSG000002	877.42412	449.64255	1305.2057	2.9027628	1.5374267	0.0005484	0.0149295
ENSG000001	206.3318	339.6526	73.011003	0.2149579	-2.217874	0.0403753	0.3479604
ENSG000001	22.458563	44.917125	0	0	#NAME?	5.78E-05	0.0023104
ENSG000001	1152.7354	1465.0473	840.42345	0.5736494	-0.801759	0.0392612	0.3434319
ENSG000001	405.22317	85.080991	725.36534	8.5255864	3.0917991	4.36E-11	6.27E-09
ENSG000000	35.743097	10.73478	60.751415	5.6593071	2.5006254	0.0341973	0.3174863
ENSG000001	790.53048	964.63922	616.42174	0.6390179	-0.646072	0.045985	0.3798687
ENSG000001	529.52772	737.13014	321.92531	0.4367279	-1.195193	0.0082523	0.11974
ENSG000000	920.84252	1348.9522	492.73288	0.3652708	-1.452962	0.0290709	0.2851352
ENSG000000	2247.774	2777.6318	1717.9161	0.6184823	-0.693196	0.036198	0.3280821
ENSG000000	61.043705	21.863146	100.22426	4.5841647	2.1966589	0.0098524	0.1362367
ENSG000001	1979.1054	2404.9531	1553.2576	0.6458577	-0.630712	0.0381769	0.3378371
ENSG000002	24.237912	2.8428921	45.632932	16.051588	4.0046441	0.0488042	0.3939138
ENSG000001	1773.2493	3144.9658	401.53282	0.1276748	-2.969455	4.74E-14	1.03E-11
ENSG000001	335.73654	222.74793	448.72514	2.0144974	1.01042	0.0149934	0.183674
ENSG000002	106.86359	40.883401	172.84378	4.2277252	2.0798816	0.0010273	0.0244715
ENSG000001	383.62328	287.46242	479.78414	1.6690326	0.7390121	0.025302	0.2612356
ENSG000001	18.126872	0	36.253744	Inf	Inf	0.0159782	0.1928398
ENSG000001	67.559698	24.467872	110.65152	4.522319	2.1770628	0.0067526	0.1042592
ENSG000001	139.794	60.302278	219.28573	3.6364419	1.8625275	0.0014268	0.0317532
ENSG000002	13.741795	0	27.48359	Inf	Inf	0.0169595	0.2005861
ENSG000001	66.173849	121.01828	11.329416	0.0936174	-3.41708	4.92E-05	0.0019959
ENSG000001	1116.8593	1462.1022	771.61643	0.5277445	-0.922088	0.0341995	0.3174863
ENSG000001	8.7618077	17.523615	0	0	#NAME?	0.0257478	0.2639244
ENSG000001	2021.4757	1344.5148	2698.4366	2.0069966	1.0050382	0.0251803	0.260402
ENSG000001	301.28403	199.92205	402.64601	2.014015	1.0100744	0.0050692	0.0835793
ENSG000001	88.593689	40.655304	136.53207	3.3582844	1.7477244	0.0405259	0.348814
ENSG000002	61.596178	11.055692	112.13666	10.14289	3.3423969	0.0092634	0.1301544
ENSG000001	26.880839	53.761678	0	0	#NAME?	6.31E-06	0.0003343
ENSG000001	531.19016	689.78889	372.59142	0.5401528	-0.88856	0.0219733	0.2373828
ENSG000002	30.584474	0	61.168947	Inf	Inf	0.000108	0.003929

ENSG000001	1109.7661	760.77844	1458.7537	1.9174488	0.9391881	0.0466145	0.3831816
ENSG000001	364.22648	502.55445	225.89852	0.4495006	-1.153605	0.0066398	0.102909
ENSG00000(	487.41879	250.86098	723.9766	2.8859674	1.529055	0.0008957	0.0219153
ENSG000001	30.319147	60.638295	0	0	#NAME?	0.000813	0.0203713
ENSG000001	13.136338	0	26.272677	Inf	Inf	0.0218039	0.2363923
ENSG000002	33.730851	1.5793845	65.882318	41.71392	5.382457	0.0022636	0.0457468
ENSG000001	15521.414	22619.738	8423.0899	0.3723779	-1.425161	1.17E-05	0.0005823
ENSG000002	96.424635	42.307364	150.54191	3.5582908	1.8311844	0.00929	0.1304603
ENSG000001	305.75576	418.61897	192.89254	0.4607831	-1.11784	0.0161554	0.1944756
ENSG000001	856.79834	1497.8453	215.75136	0.1440411	-2.795447	1.78E-06	0.0001108
ENSG000001	860.51181	1216.8213	504.20234	0.4143602	-1.271043	0.0006542	0.0171531
ENSG00000(	171.52766	286.89612	56.159199	0.1957475	-2.352934	0.0007143	0.0183908
ENSG000001	309.83834	233.70074	385.97594	1.651582	0.7238486	0.0458232	0.3791447
ENSG000001	780.55363	1082.0872	479.02007	0.4426816	-1.175659	0.0046243	0.0787041
ENSG000001	60.588506	19.88514	101.29187	5.0938477	2.3487558	0.0002955	0.0089247
ENSG000001	514.79728	321.60733	707.98723	2.2014026	1.138423	0.0219325	0.2372864
ENSG000001	123.68614	231.98607	15.386199	0.0663238	-3.914329	2.27E-07	1.68E-05
ENSG000001	109.73065	190.35578	29.105523	0.1529007	-2.709334	0.0342731	0.3177338
ENSG00000(	351.90888	111.24624	592.57153	5.3266657	2.4132327	0.0001569	0.0052941
ENSG000001	332.07845	234.82393	429.33297	1.8283186	0.8705175	0.0289195	0.2844433
ENSG000001	139.62996	198.13471	81.125212	0.4094447	-1.288259	0.0433144	0.3651029
ENSG00000(	242.97631	89.357917	396.5947	4.4382716	2.1499979	0.0002063	0.0066256
ENSG000001	496.54654	207.95207	785.141	3.7755864	1.9167007	0.0006534	0.017151
ENSG000001	107.27741	14.530337	200.02448	13.76599	3.7830365	0.0001807	0.0059288
ENSG000002	182.2764	256.00782	108.54497	0.4239908	-1.237895	0.0226029	0.2420488
ENSG000001	1079.4857	1424.8208	734.15049	0.5152581	-0.956633	0.0100008	0.1375166
ENSG000001	273.70957	116.81403	430.6051	3.6862446	1.8821518	6.20E-05	0.0024573
ENSG000001	56.485343	15.86652	97.104165	6.1200668	2.6135474	0.0105743	0.1429322
ENSG000001	25.982434	46.180633	5.7842354	0.1252524	-2.99709	0.0160865	0.193905
ENSG000001	42.117866	74.83765	9.398081	0.1255796	-2.993326	0.0040793	0.0719155
ENSG000001	179.60987	120.82258	238.39716	1.9731176	0.980477	0.0453358	0.3771385
ENSG000001	71.72236	8.2127994	135.23192	16.465996	4.0414179	0.0006641	0.0173964
ENSG000001	222.63415	105.92383	339.34446	3.2036649	1.6797232	0.0015846	0.0344449
ENSG000001	512.21269	277.24929	747.17608	2.6949612	1.4302645	0.0056019	0.0906122
ENSG000001	12150.035	6714.4994	17585.571	2.6190443	1.3890404	1.34E-05	0.0006523
ENSG000001	584.90846	868.17878	301.63814	0.3474378	-1.525173	0.0003278	0.0097379
ENSG000001	189.7991	109.09267	270.50553	2.479594	1.3101039	0.0280934	0.2786784
ENSG000001	366.22568	512.05308	220.39828	0.4304208	-1.21618	0.0212024	0.2319153
ENSG000001	574.99417	743.69592	406.29242	0.5463152	-0.872194	0.0302087	0.2927285
ENSG000001	59.812789	95.520034	24.105543	0.2523611	-1.986438	0.0180546	0.2093301
ENSG000002	40.64679	4.1841102	77.109471	18.429121	4.2039153	0.0021348	0.0436657
ENSG00000(	316.19208	435.40291	196.98124	0.4524114	-1.144293	0.0397142	0.3450102
ENSG000001	167.65666	102.7701	232.54322	2.2627518	1.1780784	0.0242922	0.2543024
ENSG000002	980.25264	1407.2138	553.29147	0.3931822	-1.34673	0.0433546	0.3652541
ENSG000002	18.536689	36.777001	0.296377	0.0080588	-6.955227	0.0315192	0.3004367
ENSG00000(	1031.4574	723.818	1339.0969	1.8500464	0.8875615	0.0079879	0.1170973
ENSG00000(	495.91479	374.10769	617.72189	1.6511874	0.7235038	0.0309121	0.296741
ENSG000001	958.36078	747.32817	1169.3934	1.5647656	0.6459465	0.0310231	0.2972791
ENSG000002	106.24391	47.754982	164.73283	3.4495422	1.7864049	0.0096473	0.1341552
ENSG000001	307.08718	426.67132	187.50305	0.4394555	-1.186211	0.014418	0.1781578
ENSG000001	135.88467	3.7905228	267.97883	70.697062	6.1435784	2.25E-06	0.0001355

ENSG000001	1804.9745	2412.0572	1197.8918	0.4966266	-1.009766	0.0096222	0.1340132
ENSG000001	716.10068	985.6922	446.50916	0.4529905	-1.142447	0.0133194	0.1681928
ENSG000001	516.66754	854.2003	179.13479	0.2097105	-2.253529	0.0001312	0.0046107
ENSG000001	689.85941	423.93634	955.78248	2.2545424	1.1728347	0.0248786	0.258739
ENSG00000(	4266.1332	3127.5897	5404.6768	1.7280645	0.7891571	0.0271881	0.2730012
ENSG000001	488.68426	620.64206	356.72647	0.5747701	-0.798943	0.04289	0.3622492
ENSG000001	356.42505	512.02572	200.82437	0.3922154	-1.350282	0.0017508	0.0371878
ENSG00000(	57.83093	96.151788	19.510072	0.2029091	-2.301095	0.0075807	0.112839
ENSG000001	67.89176	133.08421	2.6993098	0.0202827	-5.623605	2.37E-08	2.16E-06
ENSG000002	12.658228	0	25.316456	Inf	Inf	0.0230665	0.2458411
ENSG000001	11.252879	0	22.505759	Inf	Inf	0.0388139	0.3411188
ENSG000001	120.91056	182.43368	59.387429	0.3255289	-1.619143	0.0109632	0.1468689
ENSG000001	112.03319	9.7871488	214.27924	21.893939	4.4524596	5.93E-08	5.03E-06
ENSG000001	688.9375	381.67648	996.19852	2.61006	1.384083	0.0024157	0.0477851
ENSG000001	50.951302	6.9492918	94.953312	13.663739	3.7722804	0.0146584	0.1807162
ENSG000001	1158.3733	1460.3969	856.34962	0.5863814	-0.770089	0.0359998	0.327239
ENSG000001	135.43702	50.986426	219.88761	4.3126696	2.1085812	0.0053146	0.0865857
ENSG000001	250.93602	163.79191	338.08013	2.0640832	1.0455011	0.0374751	0.3351336
ENSG000001	1889.71	707.6032	3071.8169	4.3411574	2.1180797	2.26E-07	1.67E-05
ENSG000002	10.180736	20.361472	0	0	#NAME?	0.0298582	0.2907493
ENSG000002	70.63154	11.133402	130.12968	11.688222	3.5469836	0.0017593	0.0373397
ENSG00000(	1938.3424	122.68041	3754.0044	30.599867	4.9354535	1.11E-23	6.70E-21
ENSG000002	224.24401	302.51944	145.96858	0.4825098	-1.05137	0.018777	0.2142175
ENSG000001	25.020079	0	50.040158	Inf	Inf	0.0016218	0.0349988
ENSG000001	127.77407	181.24285	74.305286	0.4099764	-1.286387	0.0481943	0.3908261
ENSG000001	170.10873	247.65471	92.562759	0.3737573	-1.419826	0.0055174	0.0894055
ENSG000001	692.38411	960.76092	424.0073	0.4413245	-1.180088	0.0022442	0.0453884
ENSG000001	186.34665	90.922197	281.77111	3.0990354	1.6318193	0.0006058	0.016166
ENSG000001	23.533259	44.04217	3.0243477	0.0686694	-3.86419	0.0187086	0.2140109
ENSG000001	708.82176	978.84865	438.79488	0.4482765	-1.157539	0.004345	0.0751312
ENSG000001	97.274264	149.59759	44.950939	0.300479	-1.734664	0.008821	0.1257923
ENSG000001	11.781994	22.971233	0.592754	0.0258042	-5.276251	0.028268	0.2801365
ENSG000001	9553.4464	5916.9523	13189.941	2.2291781	1.1565119	0.0003562	0.0104541
ENSG000001	465.42595	313.84066	617.01123	1.9660016	0.9752645	0.0134344	0.1693284
ENSG000002	85.310163	19.890175	150.73015	7.5781211	2.9218402	0.0008401	0.0208765
ENSG000001	187.21556	54.937405	319.49371	5.8155953	2.5399269	2.00E-05	0.0009266
ENSG000001	432.03034	647.87226	216.18842	0.3336899	-1.58342	0.0280122	0.2779747
ENSG000001	336.514	173.96761	499.06038	2.8686971	1.5203957	0.0483566	0.3914385
ENSG000002	117.32223	63.461047	171.18342	2.6974566	1.4315998	0.0213987	0.2334016
ENSG000001	6298.8019	2916.2407	9681.3631	3.3198093	1.7311004	2.53E-06	0.0001492
ENSG000001	165.87517	228.54667	103.20367	0.451565	-1.146995	0.0260555	0.2663722
ENSG000001	48.80384	0	97.60768	Inf	Inf	6.48E-07	4.45E-05
ENSG000001	611.01222	849.96584	372.0586	0.4377336	-1.191875	0.0026049	0.0506398
ENSG000002	39.945007	4.5776975	75.312316	16.452008	4.0401918	0.0010252	0.0244715
ENSG000002	17.384603	32.841127	1.9280785	0.0587093	-4.090268	0.0116046	0.1528881
ENSG000001	1143.6803	1538.8395	748.52118	0.4864193	-1.039728	0.0081522	0.1187981
ENSG000001	314.26064	25.891836	602.62945	23.274883	4.540702	3.24E-13	5.77E-11
ENSG000001	159.38009	103.15865	215.60154	2.0899996	1.0635027	0.0179206	0.2080974
ENSG00000(	28.584803	51.477865	5.6917406	0.1105668	-3.17701	0.0199767	0.2233845
ENSG000001	244.10171	438.00042	50.202999	0.1146186	-3.125087	3.77E-05	0.0015759
ENSG000001	1264.5585	1633.9134	895.20367	0.5478893	-0.868044	0.0178991	0.2080974

ENSG000001	1758.5733	2122.3966	1394.75	0.657158	-0.605688	0.0469933	0.3852043
ENSG000001	1646.2242	815.27695	2477.1714	3.0384416	1.6033315	7.60E-05	0.0029112
ENSG000002	11.563587	0	23.127173	Inf	Inf	0.034613	0.3194619
ENSG000002	246.83504	351.34726	142.32282	0.4050774	-1.303731	0.0196478	0.2201616
ENSG000002	8.5261588	17.052318	0	0	#NAME?	0.0211844	0.231812
ENSG000001	31.424717	62.849433	0	0	#NAME?	0.001045	0.0247295
ENSG000001	505.43749	678.63316	332.24182	0.489575	-1.030398	0.0290562	0.2851352
ENSG000001	135.67238	52.084443	219.26033	4.2097086	2.0737204	2.05E-05	0.0009425
ENSG000001	115.12723	52.42046	177.83399	3.3924539	1.7623292	0.0330436	0.3104837
ENSG000001	515.25966	676.1033	354.41603	0.524204	-0.9318	0.0417883	0.3561681
ENSG000001	1129.5835	551.80322	1707.3638	3.0941534	1.6295447	5.51E-06	0.0002981
ENSG000001	13.784786	0	27.569572	Inf	Inf	0.0161334	0.1942972
ENSG000001	74.686416	8.8445532	140.52828	15.88868	3.9899274	0.0005621	0.0152266
ENSG000001	600.11597	372.80675	827.42519	2.219448	1.1502009	0.0037795	0.0676
ENSG000001	23.787823	46.418799	1.1568471	0.024922	-5.326439	0.0005652	0.015262
ENSG000001	2947.6041	1598.6302	4296.5781	2.6876623	1.4263519	0.0001072	0.0039033
ENSG000001	26.34262	47.672237	5.013004	0.1051556	-3.249402	0.0278877	0.2771461
ENSG000001	265.13541	182.24805	348.02276	1.9096103	0.9332783	0.0161641	0.194494
ENSG000001	3252.1884	1296.6655	5207.7114	4.0162336	2.0058432	0.0263118	0.2679777
ENSG000002	6144.0262	3844.8102	8443.2422	2.1960101	1.1348847	0.0035745	0.0649194
ENSG000001	712.27565	1102.0651	322.48614	0.2926199	-1.7729	3.56E-05	0.0015043
ENSG000000	919.64138	557.23355	1282.0492	2.3007395	1.2020976	0.0051128	0.0840055
ENSG000001	469.22081	206.93898	731.50263	3.5348711	1.8216576	1.09E-06	7.11E-05
ENSG000001	574.53998	1124.4103	24.669636	0.0219401	-5.510288	1.68E-05	0.000791
ENSG000001	2738.7801	743.30233	4734.2579	6.3692224	2.6711173	1.49E-10	1.91E-08
ENSG000001	336.17651	222.4997	449.85332	2.0218154	1.0156513	0.005921	0.0944742
ENSG000001	2949.7532	3769.0212	2130.4852	0.5652622	-0.823008	0.0482641	0.3912656
ENSG000001	96.823113	137.83747	55.808756	0.4048881	-1.304405	0.0312413	0.2985247
ENSG000001	1068.843	1368.1084	769.57761	0.5625122	-0.830044	0.0265697	0.2693901
ENSG000001	304.98048	526.54095	83.420014	0.1584302	-2.65808	3.38E-08	3.00E-06
ENSG000001	619.92945	901.58402	338.27488	0.3752006	-1.414266	0.000429	0.0121042
ENSG000001	434.92225	298.38283	571.46167	1.9151962	0.9374922	0.0383037	0.3385002
ENSG000002	23.825125	0	47.650249	Inf	Inf	0.0008144	0.0203864
ENSG000001	222.47119	117.39829	327.54409	2.7900245	1.4802778	0.0423756	0.3594753
ENSG000000	162.3709	16.736441	308.00536	18.403277	4.2018908	5.22E-08	4.49E-06
ENSG000001	4113.3417	7950.1103	276.57312	0.0347886	-4.845242	6.80E-34	7.67E-31
ENSG000001	137.04305	205.47256	68.613545	0.3339305	-1.58238	0.0163532	0.1958109
ENSG000001	8813.7243	43.167214	17584.281	407.3527	8.6701347	3.23E-64	4.37E-60
ENSG000001	60.230895	31.251673	89.210118	2.854571	1.5132739	0.0131867	0.1671407
ENSG000001	2969.1999	1809.2395	4129.1602	2.2822629	1.190465	0.0012471	0.0284316
ENSG000001	1158.4534	1938.3885	378.51832	0.1952747	-2.356423	3.04E-09	3.20E-07
ENSG000002	10.733406	0	21.466811	Inf	Inf	0.0450548	0.3760729
ENSG000002	1172.9447	2181.395	164.49442	0.0754079	-3.729141	1.80E-20	8.13E-18
ENSG000001	92.776241	37.967833	147.58465	3.8870969	1.9586931	0.011552	0.1524926
ENSG000001	82.032015	16.498274	147.56576	8.9443146	3.1609709	0.0381619	0.3378143
ENSG000001	689.57171	511.88255	867.26086	1.6942575	0.7606532	0.0264694	0.268675
ENSG000002	74.308501	36.072572	112.54443	3.1199447	1.6415205	0.0340596	0.3165136
ENSG000001	16.685628	32.214409	1.1568471	0.0359109	-4.799436	0.0146563	0.1807162
ENSG000001	507.99203	349.45704	666.52701	1.9073218	0.9315483	0.0403476	0.3478323
ENSG000001	346.47723	446.44635	246.50812	0.5521562	-0.856852	0.0425093	0.3603832
ENSG000001	77.67018	1.8952614	153.4451	80.962498	6.3391819	4.09E-07	2.91E-05

ENSG000001	437.06478	602.17081	271.95875	0.4516306	-1.146785	0.0056991	0.0918539
ENSG000001	119.23992	45.466133	193.0137	4.2452191	2.085839	0.0028384	0.0543591
ENSG000001	186.02889	111.76	260.29779	2.3290782	1.2197591	0.0055146	0.0894055
ENSG000001	285.71803	12.945918	558.49014	43.140251	5.4309627	9.68E-16	2.49E-13
ENSG000001	180.2217	125.25493	235.18847	1.8776784	0.90895	0.019348	0.2177937
ENSG000001	124.0683	85.624965	162.51163	1.8979468	0.9244396	0.0328844	0.3094174
ENSG000001	99.179419	24.706039	173.6528	7.0287593	2.8132701	0.0002522	0.0077732
ENSG00000(	215.50877	128.672	302.34553	2.3497383	1.2325001	0.0375592	0.3356637
ENSG000001	487.41596	299.30025	675.53168	2.2570368	1.1744299	0.0011319	0.0263155
ENSG000001	178.21189	80.455794	275.96798	3.4300573	1.7782327	0.0368997	0.3323387
ENSG00000(	439.26884	92.433941	786.10373	8.5044923	3.0882251	1.58E-09	1.72E-07
ENSG000001	30.856752	0	61.713504	Inf	Inf	0.0461202	0.3806219
ENSG000002	39.796391	66.634921	12.957861	0.1944605	-2.362451	0.0135526	0.1705794
ENSG000001	72.398382	21.158717	123.63805	5.8433621	2.5467987	0.0143469	0.1774419
ENSG000001	244.347	108.15511	380.53889	3.518455	1.8149421	0.0044413	0.0763086
ENSG000001	24.757247	0	49.514494	Inf	Inf	0.0005882	0.015792
ENSG000001	182.48877	111.2261	253.75144	2.281402	1.1899207	0.0475821	0.3879537
ENSG000001	1876.7038	2375.5061	1377.9014	0.5800454	-0.785762	0.020578	0.2280349
ENSG000002	27.246162	0	54.492325	Inf	Inf	0.0002696	0.0082338
ENSG00000(	60.334003	36.461124	84.206882	2.3094977	1.2075791	0.0378895	0.3368354
ENSG000001	389.7317	523.97147	255.49192	0.4876065	-1.036211	0.0219388	0.2372864
ENSG000001	1070.6938	1343.6333	797.75429	0.5937292	-0.752123	0.0229178	0.2446461
ENSG000001	361.89419	264.5689	459.21949	1.7357274	0.7955404	0.016895	0.200214
ENSG000001	112.15183	193.38145	30.922214	0.1599027	-2.644734	0.0025982	0.0505459
ENSG000001	2050.4466	1497.5748	2603.3185	1.7383563	0.7977238	0.0196058	0.2198731
ENSG000001	2769.3207	3380.6251	2158.0164	0.6383483	-0.647584	0.041527	0.3542752
ENSG00000(	2364.3904	1534.4273	3194.3535	2.0817888	1.0578237	0.0069731	0.1065495
ENSG000001	603.50952	761.1821	445.83694	0.5857165	-0.771725	0.0398072	0.345596
ENSG000001	1853.9193	823.57031	2884.2683	3.5021518	1.8082416	9.21E-05	0.0034574
ENSG000002	26.069735	47.040483	5.0989867	0.1083957	-3.20562	0.0491468	0.3957044
ENSG000001	144.8285	259.44219	30.214816	0.1164607	-3.102085	0.0034939	0.0637554
ENSG00000(	584.86246	218.95741	950.7675	4.3422486	2.1184423	8.52E-07	5.70E-05
ENSG00000(	179.49838	86.665411	272.33134	3.1423303	1.6518348	0.0016816	0.0360291
ENSG00000(	124.99408	83.662063	166.3261	1.9880708	0.9913691	0.0311856	0.2985193
ENSG00000(	2198.7745	1019.4385	3378.1104	3.3136971	1.7284418	2.79E-05	0.0012211
ENSG000002	12.511667	0	25.023335	Inf	Inf	0.025188	0.260402
ENSG000001	1146.1904	1693.0824	599.29832	0.3539688	-1.498306	0.0001803	0.0059243
ENSG000001	304.84772	186.51994	423.17549	2.2687949	1.1819262	0.0078904	0.1160449
ENSG000002	31.130878	55.263352	6.9984042	0.1266373	-2.981225	0.0074379	0.1111407
ENSG000002	152.14872	258.99607	45.301382	0.1749115	-2.515303	8.78E-05	0.0033124
ENSG00000(	1709.0635	2128.6918	1289.4352	0.6057407	-0.723228	0.0391839	0.3430462
ENSG000001	891.6364	1169.267	614.00578	0.5251202	-0.92928	0.0161865	0.1945907
ENSG000001	2233.9714	2739.1603	1728.7824	0.6311359	-0.663977	0.0343639	0.3182391
ENSG00000(	207.90519	98.435602	317.37478	3.2241869	1.6889354	0.0037796	0.0676
ENSG00000(	248.07361	156.34614	339.80107	2.1733895	1.1199467	0.0039564	0.0699832
ENSG000002	44.864618	4.9712849	84.757951	17.049506	4.091658	0.0001198	0.0042722
ENSG000001	253.1914	405.2694	101.11339	0.2494967	-2.002907	0.0001407	0.004876
ENSG000001	252.14268	461.60056	42.684799	0.0924713	-3.434851	2.48E-06	0.0001469
ENSG000001	341.16425	459.38723	222.94126	0.4853014	-1.043047	0.0094682	0.1325498
ENSG000001	178.88516	347.68699	10.08333	0.0290012	-5.107745	7.57E-06	0.0003946
ENSG000001	69.676567	31.101287	108.25185	3.4806228	1.7993455	0.0401158	0.3468208

ENSG000001	996.6583	1362.0441	631.2725	0.4634744	-1.109439	0.0037302	0.0670278
ENSG000001	1706.0733	2310.1325	1102.0142	0.4770351	-1.067833	0.0010078	0.0241996
ENSG000001	84.665456	0	169.33091	Inf	Inf	0.00018	0.0059243
ENSG000001	297.66243	191.19045	404.13441	2.1137792	1.0798247	0.0401394	0.3468208
ENSG000001	748.61637	1431.9048	65.327993	0.0456231	-4.45409	1.84E-07	1.39E-05
ENSG000001	183.81946	92.190739	275.44819	2.9878075	1.5790872	0.0023484	0.0468656
ENSG000001	11468.774	8568.9492	14368.598	1.6768215	0.7457291	0.0380604	0.3375789
ENSG000001	581.49751	765.94762	397.04741	0.5183741	-0.947934	0.0222548	0.2395529
ENSG000001	210.03812	353.61094	66.465304	0.1879617	-2.41149	0.0088623	0.1261567
ENSG000001	402.50941	262.60599	542.41283	2.0655006	1.0464915	0.00723	0.1093028
ENSG000001	9.8648593	19.729719	0	0	#NAME?	0.0491089	0.3955752
ENSG000001	50.1729	21.231393	79.114407	3.7262938	1.8977414	0.0316613	0.301261
ENSG000001	1885.3889	1239.4659	2531.312	2.0422603	1.0301667	0.0010994	0.0257359
ENSG000001	58.011365	24.307416	91.715313	3.7731412	1.9157661	0.008742	0.1250353
ENSG000001	223.15852	114.62303	331.69401	2.8937815	1.532956	0.0138106	0.1725434
ENSG000002	296.35956	140.74297	451.97616	3.2113588	1.6831839	0.0010465	0.0247322
ENSG000002	417.68438	154.95239	680.41637	4.3911318	2.1345928	6.96E-05	0.0027161
ENSG000001	1271.2758	630.77244	1911.7792	3.0308541	1.5997244	3.52E-05	0.0014948
ENSG000001	289.21948	169.09418	409.34478	2.4208095	1.2754895	0.0262338	0.2674876
ENSG000001	544.39294	313.09376	775.69211	2.4775074	1.3088894	0.0126829	0.1624289
ENSG000001	54.456068	106.24474	2.6673929	0.0251061	-5.315817	3.61E-06	0.0002039
ENSG000001	29.249149	0	58.498298	Inf	Inf	0.0001554	0.0052517
ENSG000001	786.11706	588.29455	983.93958	1.6725288	0.742031	0.0328291	0.3090751
ENSG00000(	15.232249	30.464498	0	0	#NAME?	0.0043896	0.0757079
ENSG000001	361.88244	246.64162	477.12326	1.9344799	0.9519457	0.0058827	0.0939737
ENSG000001	1308.2295	951.42426	1665.0347	1.7500444	0.8073915	0.0046697	0.0791766
ENSG000002	66.415424	21.707726	111.12312	5.1190588	2.3558786	0.0104459	0.1415824
ENSG000001	190.80079	268.07879	113.5228	0.423468	-1.239675	0.030008	0.2913691
ENSG000001	278.43688	414.26434	142.60943	0.3442474	-1.538482	0.0041033	0.0721966
ENSG000001	216.27637	80.67382	351.87892	4.3617486	2.1249066	0.0002653	0.0081317
ENSG000002	291.90029	148.72767	435.0729	2.925299	1.5485841	0.0077668	0.1147252
ENSG000002	40.913708	19.020254	62.807161	3.3021199	1.7233925	0.0472593	0.3862788
ENSG00000(	43.198915	74.832615	11.565215	0.1545478	-2.693875	0.0098734	0.1363884
ENSG000001	470.48061	294.14618	646.81503	2.1989577	1.1368199	0.0381571	0.3378143
ENSG000001	438.459	108.06733	768.85068	7.1145524	2.830773	5.43E-08	4.64E-06
ENSG000002	280.01671	388.05662	171.9768	0.4431745	-1.174053	0.0255909	0.2633142
ENSG000001	239.06393	95.432254	382.69562	4.0101287	2.0036485	0.0001908	0.0062057
ENSG000001	1264.1842	755.63882	1772.7296	2.3460012	1.2302038	0.0007141	0.0183908
ENSG000001	135.97977	257.01303	14.946517	0.0581547	-4.10396	5.43E-09	5.59E-07
ENSG000002	25.4679	0	50.935801	Inf	Inf	0.000465	0.0129465
ENSG000001	256.60227	347.36892	165.83561	0.4774049	-1.066715	0.0243682	0.2549994
ENSG000001	456.22556	606.03904	306.41208	0.5055979	-0.983938	0.0152122	0.1858498
ENSG000001	1150.8861	1677.9814	623.79076	0.3717507	-1.427593	0.0228324	0.2439575
ENSG000002	18.106351	0	36.212702	Inf	Inf	0.045829	0.3791447
ENSG000001	14.149592	26.756721	1.5424628	0.0576477	-4.116594	0.0487878	0.3939138
ENSG000001	330.29047	211.96784	448.61311	2.1164206	1.0816264	0.021662	0.2354198
ENSG000001	172.07659	307.01943	37.13375	0.1209492	-3.047527	8.53E-07	5.70E-05
ENSG000001	34.888452	0	69.776904	Inf	Inf	3.50E-05	0.0014912
ENSG000002	129.23685	32.364795	226.10891	6.9862614	2.8045206	0.0001389	0.0048363
ENSG00000(	58.666103	10.103026	107.22918	10.613571	3.4078382	0.001021	0.0244415
ENSG000001	149.93315	100.16034	199.70596	1.9938627	0.995566	0.036153	0.3278603

ENSG000002	71.481455	26.829396	116.13351	4.3285921	2.1138979	0.0394268	0.3440471
ENSG00000(	10.49435	0	20.988701	Inf	Inf	0.0463838	0.3820979
ENSG00000(	5124.2083	3399.2806	6849.1359	2.0148781	1.0106925	0.0032417	0.0603742
ENSG000001	227.43816	157.54705	297.32927	1.8872412	0.9162788	0.0340596	0.3165136
ENSG000001	144.18153	203.98095	84.382103	0.4136764	-1.273425	0.0369978	0.332455
ENSG000001	515.20971	276.05342	754.36599	2.7326812	1.4503172	0.0025909	0.0504407
ENSG000001	545.95492	329.94031	761.96953	2.3094163	1.2075283	0.0020811	0.0427948
ENSG000001	18.504669	0	37.009338	Inf	Inf	0.00429	0.0745611
ENSG000001	62.506659	25.891836	99.121482	3.828291	1.9367005	0.0414961	0.3541239
ENSG000002	75.454268	37.729667	113.17887	2.9997315	1.5848334	0.0383994	0.3390069
ENSG000002	101.79213	0	203.58426	Inf	Inf	1.83E-12	3.00E-10
ENSG000001	1263.405	536.73394	1990.0762	3.7077516	1.8905446	4.58E-07	3.24E-05
ENSG000001	239.24928	339.75767	138.74089	0.4083525	-1.292113	0.0109367	0.1466586
ENSG000001	4074.574	7985.658	163.49	0.020473	-5.610137	2.31E-06	0.0001382
ENSG00000(	359.03048	246.15522	471.90573	1.9171063	0.9389304	0.0393217	0.3434928
ENSG000002	21.90158	8.2854748	35.517685	4.2867411	2.0998813	0.0366507	0.331194
ENSG000001	82.133984	8.2127994	156.05517	19.001459	4.2480383	0.0001683	0.0055883
ENSG000001	517.90261	694.84795	340.95727	0.4906933	-1.027106	0.0125115	0.1612258
ENSG000001	2521.1121	3107.5627	1934.6615	0.6225655	-0.683702	0.0206537	0.228593
ENSG000001	41.35784	15.944231	66.771449	4.1878125	2.0661968	0.0490189	0.3951443
ENSG000001	1411.6501	949.10805	1874.1921	1.9746879	0.9816246	0.004181	0.0730889
ENSG000001	211.83587	142.39503	281.27672	1.9753269	0.9820914	0.0349497	0.321512
ENSG000001	84.319897	0	168.63979	Inf	Inf	8.01E-11	1.08E-08
ENSG000001	596.90493	297.65322	896.15664	3.0107406	1.5901184	7.91E-05	0.00302
ENSG000001	141.68584	199.71913	83.652557	0.418851	-1.255491	0.0306072	0.2949644
ENSG00000(	1285.8168	763.36018	1808.2733	2.3688337	1.2441769	0.0004028	0.0115462
ENSG000001	5377.3422	6845.6065	3909.0779	0.5710346	-0.80835	0.0123931	0.1602334
ENSG000001	1236.3097	877.94075	1594.6787	1.8163854	0.8610703	0.0451413	0.3763531
ENSG00000(	388.24673	130.3917	646.10176	4.9550834	2.3089093	5.51E-07	3.85E-05
ENSG000002	11.13498	0	22.269959	Inf	Inf	0.041053	0.3515618
ENSG000001	1756.826	2228.3981	1285.254	0.5767614	-0.793953	0.0174815	0.2056308
ENSG000001	395.82638	277.82566	513.8271	1.8494588	0.8871032	0.0078158	0.1153234
ENSG000001	1144.7517	534.9941	1754.5093	3.2794927	1.7134727	2.39E-06	0.0001419
ENSG000001	330.28509	495.42959	165.14059	0.3333281	-1.584985	0.0010373	0.0245809
ENSG000002	177.20151	94.261562	260.14146	2.759783	1.4645548	0.0406687	0.3496379
ENSG000002	117.7897	31.494874	204.08452	6.4799281	2.6959778	0.0002138	0.0068236
ENSG000001	588.64872	735.2802	442.01724	0.6011548	-0.734191	0.0362017	0.3280821
ENSG000001	24.788495	47.205974	2.3710159	0.050227	-4.315392	0.0044022	0.075877
ENSG000002	39.214103	7.2651687	71.163038	9.7950978	3.2920599	0.0401883	0.3470124
ENSG000001	538.90244	342.45521	735.34967	2.1472871	1.1025151	0.0332619	0.3113967
ENSG000001	117.09309	61.249908	172.93628	2.8234536	1.4974609	0.0146051	0.1803045
ENSG000002	199.63004	390.24762	9.0124654	0.0230942	-5.436324	4.78E-14	1.03E-11
ENSG000002	12.483007	0	24.966013	Inf	Inf	0.0260693	0.2664129
ENSG000001	64.6458	103.73283	25.558767	0.2463903	-2.020982	0.0102418	0.1399057
ENSG000002	26.009376	2.2111383	49.807615	22.52578	4.4935052	0.020759	0.2291028
ENSG000001	21253.639	30562.208	11945.069	0.3908445	-1.355334	2.72E-05	0.0012029
ENSG000001	760.61454	481.57062	1039.6585	2.1588909	1.1102904	0.0064876	0.1010136
ENSG000001	83.750286	41.437444	126.06313	3.0422515	1.6051394	0.015801	0.1915489
ENSG00000(	10.615506	0	21.231012	Inf	Inf	0.047643	0.3879832
ENSG000001	73.772721	20.847875	126.69757	6.0772412	2.6034165	0.0382754	0.3384871
ENSG000002	415.04263	186.30695	643.7783	3.4554712	1.7888825	0.0009624	0.0233164

ENSG000001	789.66256	482.22033	1097.1048	2.275111	1.1859369	0.0028889	0.0550541
ENSG000001	271.67029	454.63397	88.706602	0.1951165	-2.357592	2.27E-06	0.0001367
ENSG000001	189.25712	81.854582	296.65966	3.6242279	1.8576737	0.0009492	0.0230586
ENSG00000(	667.01413	446.76945	887.25881	1.9859434	0.9898245	0.0096298	0.1340356
ENSG000001	219.75469	123.62301	315.88638	2.5552394	1.3534585	0.0274158	0.2741677
ENSG000001	18.825978	37.651956	0	0	#NAME?	0.0003816	0.0110668
ENSG000001	266.19207	158.01834	374.36579	2.3691286	1.2443565	0.0043437	0.0751312
ENSG000001	35.616363	5.0540304	66.178695	13.094242	3.7108606	0.0249189	0.2589599
ENSG000001	246.3243	355.47884	137.16977	0.3858732	-1.373801	0.0038354	0.068372
ENSG000001	1035.9796	1342.6151	729.34398	0.5432264	-0.880375	0.0285851	0.2821086
ENSG00000(	73.056265	0	146.11253	Inf	Inf	0.0001656	0.005515
ENSG00000(	85.832816	128.04025	43.625383	0.3407162	-1.553358	0.0262624	0.2676785
ENSG000002	31.056603	12.863172	49.250034	3.8287626	1.9368782	0.0319322	0.3029935
ENSG000001	624.67605	174.00789	1075.3442	6.1798588	2.6275739	1.21E-06	7.84E-05
ENSG000001	3595.5874	4908.5779	2282.597	0.4650221	-1.104629	0.0034365	0.0629627
ENSG000001	17.299947	0	34.599893	Inf	Inf	0.0056245	0.0908679
ENSG000001	314.85371	170.19219	459.51522	2.6999783	1.4329478	0.0051177	0.0840355
ENSG000001	1472.3348	324.19695	2620.4726	8.0829647	3.0148846	4.66E-12	7.38E-10
ENSG000001	2198.2821	72.947424	4323.6168	59.270315	5.8892378	3.31E-30	2.99E-27
ENSG000001	399.76395	718.97981	80.548095	0.1120311	-3.158029	1.42E-10	1.84E-08
ENSG000001	44.46482	7.8969225	81.032718	10.261303	3.3591421	0.0244347	0.2555414
ENSG000001	1287.3609	812.72694	1761.9949	2.1680036	1.1163671	0.0003225	0.0096323
ENSG000001	2986.7579	1950.3667	4023.1491	2.0627655	1.0445798	0.017805	0.2076811
ENSG000001	14.246938	0	28.493876	Inf	Inf	0.0148833	0.1827391
ENSG000001	10.983535	0	21.96707	Inf	Inf	0.0396738	0.3449939
ENSG000002	27.700152	6.6283798	48.771923	7.3580459	2.8793227	0.0067938	0.1044638
ENSG000001	4170.038	5218.0352	3122.0407	0.5983173	-0.741017	0.0310065	0.2972256
ENSG000002	33.413341	2.6047257	64.221956	24.655939	4.6238633	0.0016235	0.035007
ENSG000001	4884.1174	2364.5051	7403.7296	3.1311963	1.6467139	1.24E-05	0.0006153
ENSG00000(	1918.2825	1170.522	2666.043	2.2776531	1.187548	0.0001002	0.0037037
ENSG000002	14.825362	0	29.650723	Inf	Inf	0.0125005	0.1611608
ENSG000002	47.038258	6.6283798	87.448136	13.192988	3.7216995	9.79E-05	0.003644
ENSG000001	378.95492	297.00133	460.90851	1.5518736	0.634011	0.0419428	0.35726
ENSG000002	742.50902	404.7657	1080.2523	2.6688336	1.4162094	0.008192	0.1192484
ENSG000001	77.290522	145.47608	9.1049601	0.0625873	-3.997985	4.62E-06	0.0002547
ENSG000001	360.9909	506.06434	215.91745	0.4266601	-1.228841	0.0380451	0.3375789
ENSG000001	96.662152	191.39623	1.9280785	0.0100738	-6.633255	0.007239	0.109346
ENSG000001	481.37157	691.12507	271.61808	0.3930086	-1.347367	0.0013624	0.0306984
ENSG000001	66.708239	120.22607	13.190404	0.1097133	-3.188189	0.0003836	0.0111131
ENSG000001	676.76298	448.99065	904.5353	2.0145972	1.0104914	0.0137127	0.1718617
ENSG000001	1255.3448	1525.0265	985.66313	0.6463252	-0.629668	0.0486786	0.3934566
ENSG000001	197.45853	75.065746	319.85131	4.2609489	2.0911748	0.0002669	0.0081699
ENSG000002	449.33141	182.51643	716.14638	3.9237366	1.9722282	0.0002056	0.0066151
ENSG000001	934.32627	1322.0709	546.58164	0.4134284	-1.274291	0.0003708	0.0108015
ENSG000001	19.255058	0	38.510116	Inf	Inf	0.0029512	0.0560441
ENSG000001	156.55499	90.621425	222.48856	2.455143	1.295807	0.0396356	0.3449916
ENSG000002	52.049657	0	104.09931	Inf	Inf	2.67E-07	1.95E-05
ENSG000001	21.036576	0	42.073152	Inf	Inf	0.0018231	0.0382747
ENSG000001	93.537651	144.14494	42.930366	0.2978278	-1.74745	0.0162387	0.1949579
ENSG000001	253.42987	349.73045	157.12929	0.4492868	-1.154291	0.0073285	0.1101753
ENSG000001	2220.4802	3204.1521	1236.8084	0.3860018	-1.373321	0.0004086	0.0116774

ENSG000001 546.68603	718.49626	374.87581	0.5217505	-0.938568	0.0274874	0.2745801
ENSG000001 310.63775	213.19895	408.07655	1.9140645	0.9366395	0.0279934	0.2779747
ENSG000001 418.79513	827.89255	9.6977141	0.0117137	-6.415655	4.77E-05	0.0019539
ENSG000001 43.855323	78.93398	8.7766662	0.11119	-3.168902	0.0045481	0.0776015
ENSG000001 242.13163	171.72626	312.53699	1.819972	0.8639163	0.0402738	0.3475288
ENSG000001 524.18309	267.60245	780.76372	2.9176254	1.5447947	0.0011296	0.0263076
ENSG00000(397.31783	234.54834	560.08731	2.3879398	1.2557665	0.0325561	0.3069047
ENSG000001 3977.9768	1583.1025	6372.8512	4.0255454	2.0091843	8.76E-07	5.81E-05
ENSG000001 735.03505	980.82665	489.24345	0.4988073	-1.003446	0.0122873	0.1591357
ENSG000001 97.541004	40.572559	154.50945	3.8082254	1.9291189	0.0093245	0.1308772
ENSG000001 1999.9476	1488.2639	2511.6312	1.6876248	0.7549942	0.0307041	0.2954781
ENSG000001 868.15016	608.37036	1127.93	1.8540186	0.8906557	0.0209653	0.2302859
ENSG000001 507.85986	820.07771	195.64202	0.2385652	-2.067544	1.25E-05	0.0006157
ENSG00000(220.8305	113.84593	327.81506	2.8794623	1.5257994	0.0374355	0.334966
ENSG00000(778.64666	392.26809	1165.0252	2.969972	1.5704493	7.51E-05	0.0028899
ENSG000001 4337.9934	648.86302	8027.1238	12.371061	3.6288973	1.32E-12	2.24E-10
ENSG00000(252.7913	351.548	154.0346	0.4381609	-1.190467	0.0064786	0.1009317
ENSG000001 1193.3657	1530.1655	856.56589	0.5597864	-0.837052	0.0291824	0.2858146
ENSG000001 475.40667	597.60822	353.20512	0.5910312	-0.758694	0.0351924	0.3226091
ENSG000001 539.8633	705.84607	373.88052	0.5296913	-0.916776	0.0255486	0.2630789
ENSG000001 30.390038	0	60.780075	Inf	Inf	0.0001097	0.0039737
ENSG000001 187.76023	72.942389	302.57808	4.1481789	2.0524781	0.0016456	0.0353708
ENSG00000(226.95025	163.12995	290.77055	1.7824474	0.8338595	0.0368768	0.3323387
ENSG000001 135.81612	26.513519	245.11873	9.2450468	3.2086806	5.91E-09	6.02E-07
ENSG00000(2009.019	2655.9365	1362.1016	0.5128517	-0.963386	0.0069361	0.1061643
ENSG000001 905.78682	1155.7045	655.86918	0.567506	-0.817293	0.0415409	0.3542822
ENSG00000(594.67118	790.39031	398.95205	0.5047532	-0.98635	0.0439922	0.3694015
ENSG00000(121.81759	20.604674	223.0305	10.824267	3.4361975	3.02E-05	0.0013013
ENSG000001 560.18056	221.82044	898.54068	4.0507568	2.0181915	2.74E-05	0.0012094
ENSG000001 348.88908	487.98886	209.78929	0.4299059	-1.217907	0.0143623	0.1775505
ENSG00000(8964.1711	17561.762	366.58051	0.0208738	-5.582164	1.41E-25	1.00E-22
ENSG000001 563.28701	354.12251	772.4515	2.1813115	1.1251958	0.0150604	0.1843281
ENSG000002 65.86352	8.5286763	123.19836	14.445192	3.8525174	0.0018471	0.0386879
ENSG000001 1380.0459	1056.2429	1703.849	1.6131224	0.6898559	0.0482919	0.3912656
ENSG00000(3110.4412	4022.8943	2197.9882	0.5463699	-0.87205	0.0103336	0.1406287
ENSG000001 385.05418	291.56378	478.54457	1.6413032	0.7148418	0.0244389	0.2555414
ENSG000001 353.46831	17.047282	689.88934	40.469168	5.3387513	2.78E-19	1.12E-16
ENSG000001 378.31025	158.41193	598.20856	3.7762847	1.9169675	1.26E-05	0.0006197
ENSG00000(1555.7493	246.03504	2865.4636	11.646567	3.5418328	2.45E-16	6.91E-14
ENSG000002 52.426856	95.369649	9.4840637	0.0994453	-3.329953	0.0004897	0.013496
ENSG000001 343.37844	228.8597	457.89719	2.0007768	1.0005603	0.023281	0.247075
ENSG000001 43.184837	86.369674	0	0	#NAME?	5.36E-09	5.56E-07
ENSG000001 304.74409	480.86905	128.61913	0.2674723	-1.902539	0.0005601	0.0151885
ENSG000001 855.23866	1410.4963	299.98103	0.2126776	-2.23326	6.82E-08	5.72E-06
ENSG000001 1749.2955	2207.7906	1290.8005	0.5846571	-0.774337	0.034056	0.3165136
ENSG000002 201.26248	274.87769	127.64728	0.4643784	-1.106627	0.0267697	0.2707082
ENSG000001 211.0662	289.25261	132.8798	0.4593901	-1.122208	0.0214169	0.2334124
ENSG000001 305.35116	141.45747	469.24485	3.3172152	1.7299726	0.0009003	0.0219884
ENSG000001 1047.1365	768.73512	1325.5378	1.7243102	0.7860193	0.0188645	0.2148541
ENSG00000(2794.5055	1760.9449	3828.066	2.1738704	1.1202659	0.0002188	0.0069648
ENSG00000(9617.5814	4950.1909	14284.972	2.8857417	1.5289422	0.000394	0.0113671

ENSG000002	603.81517	1206.4448	1.185508	0.0009826	-9.991041	2.29E-49	7.76E-46
ENSG000001	102.49614	26.368169	178.62412	6.774233	2.7600576	0.0012984	0.0295026
ENSG000001	137.44761	200.35088	74.544341	0.3720689	-1.426358	0.0178776	0.2080974
ENSG000001	220.11817	362.37778	77.858553	0.2148547	-2.218567	0.001969	0.0409232
ENSG000001	862.61972	556.47658	1168.7629	2.1002912	1.0705893	0.0343364	0.3181028
ENSG000002	7.5785281	15.157056	0	0	#NAME?	0.035058	0.3217228
ENSG000000	489.17059	304.17872	674.16247	2.2163367	1.1481771	0.00363	0.0657507
ENSG000001	1913.2582	2450.7625	1375.7538	0.5613575	-0.833008	0.011416	0.1512125
ENSG000001	481.80217	302.34891	661.25542	2.1870607	1.1289932	0.0314329	0.2999313
ENSG000001	449.66221	301.68191	597.6425	1.9810352	0.9862545	0.0137985	0.1724716
ENSG000001	2157.5535	1572.8815	2742.2256	1.7434406	0.8019372	0.040903	0.3509436
ENSG000001	128.5163	31.261743	225.77085	7.2219534	2.8523891	0.0002216	0.0070135
ENSG000002	17.596322	32.525251	2.6673929	0.0820099	-3.608058	0.0308034	0.2960502
ENSG000000	1522.0274	2041.9307	1002.1241	0.4907728	-1.026873	0.0024399	0.0481231
ENSG000001	1817.48	2244.969	1389.991	0.6191582	-0.69162	0.035461	0.3241283
ENSG000001	57.623506	28.729693	86.51732	3.0114252	1.5904464	0.0411953	0.3523345
ENSG000001	1594.8472	1925.0742	1264.6203	0.6569203	-0.60621	0.0357026	0.3255311
ENSG000001	87.483507	45.305677	129.66134	2.8619225	1.5169846	0.0237177	0.2504332
ENSG000001	19.263456	38.526912	0	0	#NAME?	0.000143	0.0049423
ENSG000001	580.5966	116.03189	1045.1613	9.0075347	3.1711323	2.40E-11	3.60E-09
ENSG000002	159.58819	312.59225	6.5841277	0.021063	-5.569146	4.49E-15	1.06E-12
ENSG000001	367.19617	183.94543	550.44692	2.9924469	1.5813257	0.0070427	0.1073106
ENSG000001	851.84852	1146.1879	557.50916	0.4864029	-1.039776	0.0032779	0.0607974
ENSG000000	1038.1992	1428.2905	648.10802	0.4537649	-1.139983	0.002683	0.0519337
ENSG000001	23.503295	41.60797	5.3986197	0.1297497	-2.946197	0.0130413	0.1658767
ENSG000001	835.89568	329.63954	1342.1518	4.0715742	2.0255867	3.02E-06	0.000174
ENSG000001	62.339773	11.68241	112.99714	9.6724163	3.2738763	0.0010272	0.0244715
ENSG000002	10.969205	0	21.938409	Inf	Inf	0.0403443	0.3478323
ENSG000001	2546.0621	1760.1886	3331.9355	1.8929423	0.9206304	0.0179252	0.2080974
ENSG000001	261.03387	149.87822	372.18953	2.4832796	1.3122467	0.0011675	0.0269353
ENSG000001	11.593876	0	23.187751	Inf	Inf	0.0348598	0.3210828
ENSG000001	50808.446	15608.248	86008.645	5.5104613	2.4621731	5.50E-11	7.67E-09
ENSG000002	18.32573	34.337766	2.3136942	0.0673804	-3.891526	0.0177503	0.2075715
ENSG000000	462.08475	669.28206	254.88743	0.3808371	-1.392754	0.0008351	0.0207905
ENSG000001	538.27823	363.75424	712.80222	1.9595709	0.9705378	0.0360525	0.327388
ENSG000001	3667.5543	2509.1616	4825.947	1.9233305	0.9436067	0.0132073	0.1672457
ENSG000000	503.88574	321.05044	686.72105	2.1389818	1.0969242	0.0059936	0.0952957
ENSG000001	926.21616	1346.2732	506.15908	0.3759705	-1.411308	0.0032637	0.0606163
ENSG000002	27.327418	2.5270152	52.127821	20.628218	4.3665473	0.0226191	0.2421266
ENSG000001	13127.944	19043.832	7212.057	0.3787083	-1.400841	0.0031873	0.0595249
ENSG000001	19.025289	38.050579	0	0	#NAME?	0.0002134	0.0068167
ENSG000002	15.548126	31.096252	0	0	#NAME?	0.0028514	0.0544923
ENSG000001	1048.1558	2000.5552	95.75646	0.0478649	-4.384887	1.10E-23	6.70E-21
ENSG000000	1298.1595	1676.7251	919.59389	0.5484464	-0.866577	0.0474322	0.386965
ENSG000000	600.54608	448.04521	753.04695	1.6807388	0.7490955	0.0482777	0.3912656
ENSG000001	121.61726	11.128367	232.10616	20.857162	4.382471	2.03E-06	0.0001253
ENSG000001	31.472309	54.461072	8.4835452	0.1557726	-2.682486	0.0404712	0.3484538
ENSG000001	217.56149	16.653695	418.46928	25.127713	4.6512075	1.69E-12	2.80E-10
ENSG000001	560.45937	724.69077	396.22798	0.5467546	-0.871035	0.022273	0.2396536
ENSG000002	107.86495	51.146882	164.58301	3.2178503	1.6860972	0.0079784	0.1170833
ENSG000000	19.552328	1.2635076	37.841148	29.949284	4.9044496	0.0395701	0.3446428

ENSG00000(	11.888625	0	23.777249	Inf	Inf	0.0303599	0.2936274
ENSG000001	2551.2781	4027.9174	1074.6387	0.2667976	-1.906182	6.45E-07	4.44E-05
ENSG000001	395.43071	241.45735	549.40408	2.275367	1.1860993	0.0041371	0.0726031
ENSG000001	683.30774	361.13223	1005.4832	2.7842523	1.47729	0.0331044	0.3109474
ENSG000001	18.942544	37.885088	0	0	#NAME?	0.0006344	0.0168141
ENSG000001	532.88227	719.49139	346.27316	0.4812749	-1.055067	0.0067747	0.1042841
ENSG000001	601.61017	748.86794	454.3524	0.606719	-0.7209	0.0439962	0.3694015
ENSG000001	9.3521887	18.704377	0	0	#NAME?	0.0349539	0.321512
ENSG000001	228.34926	339.95337	116.74516	0.3434152	-1.541974	0.0166656	0.1985568
ENSG00000(	4351.9315	3094.1722	5609.6908	1.812986	0.8583678	0.0225756	0.2418522
ENSG000001	524.27946	783.37842	265.18051	0.3385088	-1.562735	0.0004622	0.0129079
ENSG00000(	272.24183	349.63763	194.84603	0.5572799	-0.843526	0.0497493	0.3996039
ENSG000001	239.28056	325.26258	153.29854	0.471307	-1.085261	0.0203062	0.2258556
ENSG000001	646.79316	882.08743	411.49889	0.4665058	-1.100033	0.0067568	0.1042592
ENSG000001	506.56745	856.41077	156.72414	0.1830011	-2.450076	0.0015278	0.0335309
ENSG000001	560.44737	904.58451	216.31022	0.2391266	-2.064153	0.0002244	0.0070866
ENSG000001	144.31039	203.52476	85.096013	0.4181114	-1.258041	0.0151599	0.1854621
ENSG000001	156.44566	299.84422	13.0471	0.0435129	-4.522412	0.002988	0.0565446
ENSG000001	279.54907	22.743137	536.35501	23.583159	4.5596851	6.30E-10	7.31E-08
ENSG000001	1364.1431	1039.0496	1689.2366	1.6257517	0.701107	0.0388713	0.3414139
ENSG000002	86.719109	137.05029	36.387923	0.2655078	-1.913174	0.0033557	0.0619404
ENSG000001	4816.8055	6435.3063	3198.3047	0.4969934	-1.008701	0.0116184	0.1529216
ENSG000002	530.89496	1028.5508	33.239164	0.0323165	-4.951585	7.83E-07	5.27E-05
ENSG000002	208.88024	300.44862	117.31187	0.3904557	-1.356769	0.0132197	0.1673233
ENSG000001	1429.1972	1766.0226	1092.3719	0.6185492	-0.69304	0.0392798	0.3434319
ENSG000001	49.107351	19.652008	78.562695	3.997693	1.9991677	0.0158061	0.1915489
ENSG000001	1438.0951	1804.8129	1071.3773	0.5936224	-0.752383	0.0186583	0.213765
ENSG00000(	109.23722	175.24119	43.233255	0.2467072	-2.019129	0.0040132	0.0708879
ENSG000001	379.31284	159.1365	599.48917	3.7671381	1.9134689	8.70E-05	0.0032892
ENSG000001	74.109163	15.793845	132.42448	8.3845625	3.0677355	0.0112112	0.1489707
ENSG000001	7862.1221	9943.1067	5781.1374	0.5814216	-0.782343	0.0315816	0.3008197
ENSG000001	307.40638	195.68037	419.13238	2.1419235	1.0989069	0.0208463	0.2297852
ENSG000001	427.35096	94.883245	759.81868	8.007933	3.0014299	8.85E-09	8.68E-07
ENSG000001	158.14601	6.317538	309.97448	49.065708	5.6166432	5.61E-10	6.65E-08
ENSG000001	80.735423	37.408755	124.06209	3.3163919	1.7296145	0.0050858	0.0837655
ENSG000001	41.94508	83.890159	0	0	#NAME?	0.0148065	0.1822097
ENSG000001	672.63912	880.99948	464.27876	0.526991	-0.92415	0.0401405	0.3468208
ENSG00000(	252.21744	488.09897	16.335907	0.0334684	-4.901055	9.01E-05	0.0033856
ENSG00000(	471.70922	612.5595	330.85895	0.5401254	-0.888634	0.0496244	0.3988378
ENSG000001	152.20755	98.663698	205.7514	2.0853809	1.060311	0.0391699	0.3430462
ENSG00000(	287.62498	539.33363	35.916325	0.0665939	-3.908466	6.32E-05	0.0024899
ENSG000002	150.92732	101.03026	200.82437	1.9877647	0.991147	0.0417443	0.3559047
ENSG000001	418.27596	272.30033	564.25159	2.0721664	1.0511399	0.0211479	0.2316313
ENSG000002	154.75801	53.912064	255.60395	4.7411272	2.2452301	0.0004489	0.0125887
ENSG00000(	21.510932	43.021864	0	0	#NAME?	9.39E-05	0.0035147
ENSG000001	2786.4311	3629.4872	1943.375	0.5354407	-0.901201	0.0232914	0.247075
ENSG00000(	165.24572	326.31024	4.1811948	0.0128136	-6.286185	3.49E-15	8.50E-13
ENSG000001	277.62744	146.24815	409.00672	2.7966624	1.4837061	0.0002128	0.0068067
ENSG000001	357.26622	520.59183	193.94061	0.3725387	-1.424538	0.004432	0.0762451
ENSG000001	742.07161	1069.1067	415.03652	0.3882087	-1.365096	0.0108756	0.146129
ENSG000001	83.043781	163.38825	2.6993098	0.0165208	-5.91957	6.98E-09	7.02E-07

ENSG00000137.893724	2.8428921	72.944556	25.658574	4.6813691	0.0040922	0.0720927
ENSG0000019059.7052	16944.155	1175.2552	0.0693605	-3.849742	7.65E-18	2.72E-15
ENSG000001	376.1471	511.69474	240.59947	0.4702012	-1.08865	0.016644	0.1984159
ENSG00000173.458648	135.06222	11.85508	0.087775	-3.510047	0.0001084	0.0039378
ENSG000001555.99304	1106.5875	5.3986197	0.0048786	-7.679311	5.47E-35	6.44E-32
ENSG000001300.34752	574.27579	26.419237	0.0460044	-4.442083	8.63E-16	2.28E-13
ENSG00000126.272677	0	52.545354	Inf	Inf	0.0004065	0.0116402
ENSG000000	1589.1372	1087.0484	2091.226	1.9237653	0.9439328	0.0148611	0.1826327
ENSG000001616.83379	360.66026	873.00731	2.4205808	1.2753533	0.0023028	0.0462295
ENSG00000116.202049	0	32.404099	Inf	Inf	0.0077375	0.1145425
ENSG000001127.27241	0	254.54482	Inf	Inf	2.16E-08	1.99E-06
ENSG000001772.28033	993.88771	550.67295	0.5540595	-0.851887	0.0209105	0.2299305
ENSG000001114.09991	5.0540304	223.14579	44.152047	5.4644084	0.0001249	0.0044108
ENSG000001191.44773	63.859669	319.03578	4.9958885	2.3207413	0.0002404	0.007502
ENSG00000160.869277	104.60275	17.1358	0.1638179	-2.609835	0.001959	0.040778
ENSG00000153.198971	92.190739	14.207203	0.1541066	-2.697999	0.0081695	0.1189852
ENSG000001	202.3303	72.39338	332.26723	4.589746	2.1984143	0.0011619	0.0268732
ENSG000001	2278.706	2983.739	1573.6731	0.5274165	-0.922985	0.0048275	0.0808898
ENSG00000147.507547	85.795491	9.2196036	0.1074602	-3.218125	0.0112719	0.1495976
ENSG000000	159.24483	34.819134	283.67053	8.1469724	3.026264	0.0001571	0.0052946
ENSG000000	31.36529	2.8428921	59.887688	21.065762	4.3968282	0.0122905	0.1591357
ENSG000002	102.55736	146.75973	58.354994	0.3976227	-1.330528	0.0256704	0.2637304
ENSG000001141.60248	72.533696	210.67126	2.9044606	1.5382703	0.0085657	0.1232306
ENSG00000175.451957	149.42203	1.481885	0.0099174	-6.655816	6.74E-10	7.77E-08
ENSG000001839.86251	1081.3504	598.37466	0.5533587	-0.853713	0.0186306	0.2135376
ENSG000000	4345.6994	5909.6373	2781.7616	0.4707161	-1.087071	0.0009256	0.0225443
ENSG00000154.232299	1.8952614	106.56934	56.22936	5.8132517	4.97E-05	0.0020112
ENSG0000014804.2763	6922.6459	2685.9067	0.3879885	-1.365914	2.79E-05	0.0012211
ENSG000000	89.104286	137.3561	40.852471	0.2974201	-1.749426	0.019928	0.2229317
ENSG000000	79.363194	29.760069	128.96632	4.3335356	2.1155446	0.0133681	0.168729
ENSG000001245.70936	129.52682	361.8919	2.7939535	1.482308	0.0018022	0.0380129
ENSG00000126.653408	0	53.306817	Inf	Inf	0.0003231	0.0096414
ENSG000002	275.91637	127.4711	424.36165	3.3290812	1.7351241	0.000188	0.0061356
ENSG000001550.94298	274.20347	827.68249	3.0184975	1.5938306	0.0087958	0.1256061
ENSG000002	12.718806	0	25.437612	Inf	Inf	0.0233534	0.2475389
ENSG00000114.882683	0	29.765367	Inf	Inf	0.0116827	0.1534689
ENSG0000011473.6543	383.93512	2563.3734	6.6765798	2.7391092	2.38E-11	3.57E-09
ENSG00000145.204325	0	90.40865	Inf	Inf	1.85E-06	0.0001146
ENSG000001335.20513	499.59356	170.8167	0.3419113	-1.548306	0.0022157	0.0448795
ENSG000001897.11455	518.74188	1275.4872	2.4588091	1.2979597	0.0074139	0.110966
ENSG000001292.59519	65.672185	519.5182	7.9107799	2.9838199	4.02E-08	3.54E-06
ENSG000002	892.52636	476.96338	1308.0893	2.7425362	1.4555107	0.0013225	0.0299232
ENSG000000	1072.167	1427.1147	717.21922	0.5025659	-0.992615	0.0063945	0.0999668
ENSG000001394.31785	272.43561	516.20008	1.89476	0.9220151	0.0419896	0.3575456
ENSG000001125.69574	183.78497	67.606514	0.3678566	-1.442785	0.0101715	0.1392267
ENSG000001191.13434	58.810674	323.458	5.4999881	2.4594285	0.0001175	0.0041993
ENSG000001212.23188	133.69582	290.76794	2.1748468	1.1209138	0.0102675	0.1400764
ENSG000001540.81541	218.15513	863.4757	3.9580811	1.9848012	6.70E-07	4.57E-05
ENSG00000110.460275	20.920551	0	0	#NAME?	0.0088228	0.1257923
ENSG000001474.12528	941.90549	6.3450724	0.0067364	-7.213802	3.17E-16	8.76E-14
ENSG00000178.527577	120.22104	36.834117	0.3063866	-1.706575	0.0268835	0.2712507

ENSG00000(	1821.7451	1394.4111	2249.0791	1.612924	0.6896784	0.0389679	0.341819
ENSG000001	183.68327	112.15862	255.20792	2.2754195	1.1861326	0.0100615	0.1381111
ENSG000001	839.83228	455.59386	1224.0707	2.6867586	1.4258667	0.0001174	0.0041993
ENSG000001	493.2407	213.12124	773.36017	3.6287334	1.8594661	2.93E-05	0.0012694
ENSG000001	33.201913	1.8952614	64.508565	34.036764	5.089022	0.0050947	0.0838607
ENSG00000(	87.959845	44.11988	131.79981	2.9873111	1.5788475	0.013029	0.1658406
ENSG000001	133.599	196.40494	70.793059	0.3604444	-1.472151	0.0215857	0.2347791
ENSG000001	3494.7888	4275.431	2714.1466	0.6348241	-0.655571	0.0268445	0.2710597
ENSG000001	1726.1196	1102.4012	2349.8381	2.1315635	1.091912	0.0079827	0.1170833
ENSG000001	786.20825	394.12592	1178.2906	2.9896297	1.5799668	0.0001228	0.0043483
ENSG000001	277.16633	117.91205	436.42061	3.7012384	1.8880081	1.05E-05	0.0005329
ENSG000001	320.44846	623.91093	16.985983	0.027225	-5.198923	3.83E-19	1.52E-16
ENSG000001	494.01481	194.65	793.37962	4.0759293	2.027129	5.87E-06	0.0003139
ENSG000001	85.516498	28.812438	142.22056	4.936082	2.3033664	0.0054582	0.0886585
ENSG000001	390.85297	218.17024	563.53571	2.5830091	1.3690527	0.0014693	0.0324572
ENSG000002	15.268299	0	30.536598	Inf	Inf	0.0104015	0.1411605
ENSG000001	4148.974	5769.4591	2528.4889	0.4382541	-1.190161	0.0020257	0.041846
ENSG000001	153.28961	284.45907	22.120143	0.0777621	-3.684788	0.003081	0.0579801
ENSG000001	636.95367	360.36738	913.53997	2.5350241	1.3419994	0.00425	0.0740077
ENSG000001	1106.7625	695.98122	1517.5438	2.1804379	1.1246179	0.0245546	0.2564534
ENSG000001	639.2083	347.0178	931.3988	2.6840087	1.4243894	0.0014543	0.0321797
ENSG000001	397.76866	214.37468	581.16264	2.7109668	1.4388075	0.0004855	0.0134349
ENSG000002	528.2797	306.43517	750.12422	2.4479051	1.2915476	0.0038816	0.0690597
ENSG000001	1299.1612	871.85634	1726.4661	1.9802186	0.9856597	0.009019	0.1277156
ENSG000002	105.68433	156.9304	54.438259	0.3468943	-1.527432	0.0285535	0.2818991
ENSG000001	35443.348	64538.216	6348.4805	0.0983678	-3.34567	2.53E-19	1.04E-16
ENSG000002	20.369025	40.73805	0	0	#NAME?	8.20E-05	0.003122
ENSG000001	436.86024	11.998287	861.72219	71.820434	6.1663225	3.27E-22	1.77E-19
ENSG000001	53.567931	18.942544	88.193319	4.6558329	2.2190393	0.0479936	0.3897826
ENSG000001	293.03099	171.06715	414.99484	2.4259178	1.2785307	0.022907	0.2446281
ENSG000001	170.56039	80.969557	260.15123	3.2129511	1.683899	6.89E-05	0.0026925
ENSG000002	28.757436	3.7905228	53.724349	14.173335	3.8251074	0.0377332	0.3361675
ENSG000001	1005.4886	1321.5464	689.43077	0.5216849	-0.938749	0.0338158	0.3152218
ENSG00000(	294.74362	180.60103	408.88621	2.2640304	1.1788933	0.0111182	0.148224
ENSG000001	506.84642	824.54245	189.15038	0.2294004	-2.12406	0.0001451	0.0049945
ENSG000001	527.39484	697.71602	357.07366	0.5117751	-0.966418	0.0085653	0.1232306
ENSG000001	431.41924	549.30423	313.53426	0.5707844	-0.808982	0.0303568	0.2936274
ENSG000001	90.884057	1.5793845	180.18873	114.08794	6.8340025	6.16E-05	0.0024495
ENSG000001	1509.4893	1883.2777	1135.7009	0.6030448	-0.729663	0.0472624	0.3862788
ENSG000002	51.797945	20.045595	83.550294	4.1680126	2.0593596	0.0077243	0.1144734
ENSG000001	30.28903	52.575881	8.0021787	0.1522025	-2.715936	0.0296801	0.2894321
ENSG000001	1919.0512	2325.3572	1512.7451	0.6505431	-0.620283	0.0390281	0.3422358
ENSG00000(	472.9454	321.96064	623.93017	1.9379082	0.9545002	0.0086971	0.124657
ENSG000001	137.93254	0	275.86508	Inf	Inf	1.82E-10	2.32E-08
ENSG000001	237.33708	14.214461	460.4597	32.393752	5.0176437	1.44E-10	1.86E-08
ENSG000002	11.176343	0	22.352686	Inf	Inf	0.0373975	0.3347712
ENSG000001	147.60725	56.361369	238.85312	4.2378872	2.0833452	0.0016094	0.0348431
ENSG000001	148.06653	67.557376	228.57568	3.3834304	1.7584867	0.000379	0.0110034
ENSG000001	279.38296	379.15953	179.60639	0.4736961	-1.077966	0.0159839	0.1928398
ENSG000001	365.97044	187.46254	544.47834	2.9044648	1.5382723	0.000444	0.0124629
ENSG000001	103.39517	0	206.79034	Inf	Inf	1.34E-12	2.24E-10

ENSG000001	23961.626	15669.157	32254.094	2.0584447	1.0415547	0.0025122	0.049121
ENSG00000(	1495.3157	2317.2819	673.34955	0.2905773	-1.783006	2.22E-06	0.0001339
ENSG000001	319.1693	130.71262	507.62597	3.883527	1.9573675	0.0003124	0.0093842
ENSG000001	203.38703	87.31227	319.4618	3.658842	1.8713871	0.0039153	0.0694774
ENSG000001	8455.6221	12089.011	4822.2329	0.3988939	-1.325923	0.0001536	0.005197
ENSG000001	1146.9514	758.84794	1535.0548	2.0228754	1.0164075	0.0148746	0.1827149
ENSG000001	449.85201	287.19186	612.51216	2.132763	1.0927236	0.0408688	0.350761
ENSG000001	125.90416	191.10771	60.700605	0.3176251	-1.654603	0.0117452	0.1542145
ENSG000001	176.72446	27.466185	325.98273	11.868512	3.5690671	1.41E-08	1.36E-06
ENSG000002	64.037605	22.733067	105.34214	4.633873	2.2122185	0.0130502	0.1658767
ENSG000002	20.03217	38.521877	1.5424628	0.0400412	-4.64237	0.0047961	0.080714
ENSG000002	746.84996	394.29645	1099.4035	2.7882662	1.4793683	0.0007212	0.0185522
ENSG000001	638.33463	244.92696	1031.7423	4.2124489	2.0746592	0.0170415	0.2014677
ENSG000002	55.362753	21.231393	89.494114	4.2151787	2.0755938	0.0165789	0.1979009
ENSG000001	327.89151	196.92374	458.85928	2.3301369	1.2204147	0.0013194	0.0298787
ENSG00000(	303.03768	420.12065	185.95472	0.4426222	-1.175852	0.0109561	0.1468462
ENSG000001	100.42021	51.700926	149.13949	2.884658	1.5284003	0.0230953	0.245959
ENSG000001	66.040768	17.37323	114.70831	6.6025897	2.723032	0.0332446	0.3113967
ENSG000001	160.59589	234.51813	86.673649	0.3695819	-1.436034	0.0245962	0.2567893
ENSG000001	12.213662	0	24.427325	Inf	Inf	0.0266565	0.2699673
ENSG000001	882.00022	1144.0919	619.90856	0.5418346	-0.884076	0.0183959	0.2115835
ENSG000001	165.99743	53.057249	278.93762	5.2572952	2.3943207	0.0023327	0.0466213
ENSG000001	120.79689	2.5270152	239.06677	94.604407	6.5638355	4.76E-10	5.70E-08
ENSG000001	92.726354	152.5959	32.856804	0.215319	-2.215452	0.0013117	0.029778
ENSG000001	559.33843	1078.8536	39.823292	0.0369126	-4.759743	4.53E-17	1.44E-14
ENSG000001	107.60967	75.449264	139.77007	1.8525041	0.8894767	0.0301395	0.2922267
ENSG000001	328.67184	31.58769	625.75598	19.810122	4.3081658	8.23E-10	9.24E-08
ENSG000001	352.18032	230.89813	473.46251	2.0505255	1.0359937	0.0491717	0.3957865
ENSG000002	251.50194	174.43387	328.57001	1.8836365	0.9135206	0.018899	0.2151563
ENSG000001	123.8658	243.16479	4.5668105	0.0187807	-5.734604	3.13E-06	0.0001791
ENSG000001	248.87715	139.16358	358.59072	2.5767569	1.3655564	0.0080679	0.1181586
ENSG000001	581.00727	358.47212	803.54242	2.2415758	1.1645133	0.0155291	0.1888467
ENSG000001	3010.2639	4160.41	1860.1178	0.4470996	-1.161332	0.0338732	0.315378
ENSG000001	1905.089	2377.9985	1432.1794	0.6022625	-0.731536	0.0271037	0.2729641
ENSG000001	37.766764	9.8648593	65.668668	6.6568276	2.7348348	0.0061915	0.0976412
ENSG000001	160.41883	254.8472	65.99045	0.2589412	-1.949303	0.0125514	0.1614323
ENSG000001	503.99183	687.42233	320.56132	0.4663237	-1.100596	0.0089708	0.1273013
ENSG000001	482.99381	308.24769	657.73994	2.1338033	1.0934272	0.0128912	0.1643181
ENSG000001	1870.8024	2488.0605	1253.5443	0.5038239	-0.989009	0.0124729	0.160881
ENSG000001	71.668182	0	143.33636	Inf	Inf	1.99E-09	2.16E-07
ENSG000001	19.776592	36.082642	3.4705412	0.0961831	-3.378072	0.0240009	0.2522281
ENSG000001	62.178691	19.579333	104.77805	5.3514617	2.419933	0.0264608	0.268675
ENSG000001	122.74134	68.26684	177.21583	2.5959285	1.3762506	0.0032513	0.0604703
ENSG000001	125.14852	241.8135	8.4835452	0.035083	-4.833084	2.93E-09	3.10E-07
ENSG000001	175.11179	299.39592	50.827669	0.1697674	-2.558369	0.0394799	0.3441291
ENSG000002	896.11758	510.65429	1281.5809	2.5096839	1.3275057	0.0017904	0.0377919
ENSG000001	244.58038	105.04384	384.11692	3.6567296	1.8705539	0.0002448	0.0076143
ENSG000001	139.96561	103.5472	176.38403	1.7034166	0.7684313	0.0386633	0.3406924
ENSG000001	16.337818	32.675636	0	0	#NAME?	0.0018075	0.0380549
ENSG000001	729.21406	1060.3874	398.04077	0.375373	-1.413603	0.0003358	0.0099349
ENSG000001	187.39992	62.192504	312.60734	5.0264473	2.3295391	9.24E-06	0.000477

ENSG00000(	906.54024	1325.9794	487.10107	0.3673519	-1.444765	0.0026599	0.0515235
ENSG00000(	286.97971	383.7473	190.21212	0.4956703	-1.012547	0.0177526	0.2075715
ENSG000001	335.2968	143.10449	527.4891	3.6860416	1.8820723	7.21E-05	0.0027959
ENSG000001	56.077412	13.25676	98.898064	7.4601989	2.8992141	0.0014328	0.0318322
ENSG000001	9709.6944	5276.2478	14143.141	2.6805301	1.4225183	0.0007882	0.0198593
ENSG000001	745.40147	937.85732	552.94561	0.5895839	-0.762231	0.0251183	0.2602319
ENSG00000(	1400.3511	1777.5971	1023.105	0.5755551	-0.796974	0.0272811	0.2732253
ENSG000001	738.63027	510.95506	966.30549	1.8911751	0.9192829	0.0184627	0.212062
ENSG000001	94.166363	6.317538	182.01519	28.811095	4.8485526	6.20E-06	0.0003297
ENSG000001	3919.6368	5375.8743	2463.3993	0.4582323	-1.125849	0.001586	0.0344449
ENSG000001	74.470116	9.476307	139.46393	14.717118	3.8794233	0.0008287	0.0206742
ENSG000002	1590.8619	1184.1529	1997.5709	1.6869198	0.7543914	0.0498988	0.4005673
ENSG000001	259.78222	506.07441	13.490037	0.0266562	-5.229383	7.14E-05	0.0027734
ENSG000001	425.08667	234.91171	615.26163	2.6191186	1.3890814	0.0018722	0.0391233
ENSG000002	10.792355	0	21.584711	Inf	Inf	0.0438212	0.3681605
ENSG000001	1212.5423	1525.8691	899.21551	0.5893137	-0.762892	0.0333312	0.3116711
ENSG000001	284.21318	156.06048	412.36587	2.6423466	1.4018197	0.0072539	0.1093581
ENSG00000(	36.973826	71.280259	2.6673929	0.0374212	-4.74	0.000263	0.0080786
ENSG00000(	1323.1657	1940.0802	706.2513	0.364032	-1.457863	0.0001475	0.0050573
ENSG00000(	758.85672	1083.1701	434.54334	0.4011774	-1.317688	0.003172	0.0592799
ENSG000001	98.955424	25.099626	172.81122	6.8850118	2.7834591	0.0004985	0.013682
ENSG00000(	26.357031	0	52.714063	Inf	Inf	0.0003539	0.0103974
ENSG000001	178.01401	77.976279	278.05175	3.5658504	1.8342462	0.0001311	0.0046107
ENSG000001	64.289527	20.206051	108.373	5.3633934	2.4231461	0.0050998	0.0838949
ENSG000001	1808.5207	2379.6018	1237.4396	0.5200196	-0.943362	0.0274777	0.2745801
ENSG000001	33.66464	67.32928	0	0	#NAME?	1.39E-06	8.91E-05
ENSG000001	11657.57	15172.796	8142.3446	0.536641	-0.897971	0.0029589	0.0561497
ENSG000002	74.754904	149.50981	0	0	#NAME?	2.15E-13	3.96E-11
ENSG000001	163.70965	233.99933	93.419973	0.3992318	-1.324701	0.0137945	0.1724716
ENSG000002	70.346885	33.467846	107.22592	3.2038489	1.6798061	0.0426828	0.3611753
ENSG000001	3310.6405	3988.1262	2633.1549	0.6602486	-0.598919	0.0325122	0.3066617
ENSG000001	414.48784	248.8103	580.16538	2.3317579	1.221418	0.0097016	0.1348403
ENSG000001	619.54098	833.26179	405.82018	0.487026	-1.037929	0.0232334	0.2468472
ENSG000001	826.97343	453.79863	1200.1482	2.6446713	1.4030884	0.0009868	0.0237779
ENSG000001	1160.3245	539.77254	1780.8764	3.299309	1.7221639	2.80E-05	0.0012225
ENSG000001	544.26158	695.45957	393.06358	0.5651854	-0.823204	0.0466378	0.3832571
ENSG000001	524.58061	272.46078	776.70043	2.8506871	1.5113097	7.14E-05	0.0027734
ENSG000001	173.98721	77.660402	270.31403	3.4807189	1.7993853	0.0001694	0.0056112
ENSG000001	1108.8209	320.28341	1897.3584	5.9239984	2.5665713	1.48E-11	2.28E-09
ENSG000001	29.053122	58.106244	0	0	#NAME?	1.92E-06	0.0001186
ENSG000002	74.689008	121.18377	28.194243	0.2326569	-2.103724	0.004173	0.0730889
ENSG00000(	3574.2494	2412.9825	4735.5163	1.9625158	0.9727043	0.0142953	0.1771265
ENSG000001	402.56659	720.60451	84.528664	0.1173024	-3.091695	3.16E-10	3.87E-08
ENSG000001	55.928926	111.85785	0	0	#NAME?	1.24E-10	1.62E-08
ENSG000001	127765.2	95040.446	160489.95	1.6886489	0.7558694	0.0184859	0.2122394
ENSG000002	109.95409	50.841076	169.0671	3.3254036	1.7335294	0.0399592	0.3463601
ENSG000001	133.08506	59.90869	206.26142	3.4429299	1.7836368	0.0036789	0.0664133
ENSG000001	444.89731	261.46048	628.33414	2.4031706	1.2649391	0.0176528	0.2069762
ENSG000001	1298.2123	1756.5262	839.89843	0.4781588	-1.064438	0.0032946	0.0609895
ENSG000001	1178.3565	1611.2005	745.51247	0.4627062	-1.111832	0.0023684	0.0471269
ENSG000001	21.531942	37.579281	5.4846024	0.1459475	-2.776479	0.0397539	0.3452444

ENSG000001	721.76052	889.69868	553.82236	0.6224831	-0.683894	0.0399294	0.346358
ENSG000001	886.40274	1291.4913	481.31423	0.372681	-1.423987	0.0048532	0.0811197
ENSG000002	32.556553	65.113106	0	0	#NAME?	0.0004123	0.0117572
ENSG000001	918.22832	1226.122	610.33461	0.4977764	-1.00643	0.0073211	0.1101264
ENSG000001	234.82215	327.28305	142.36125	0.434979	-1.200982	0.0190136	0.2155539
ENSG000001	452.36521	21.630015	883.10041	40.827545	5.3514709	1.71E-18	6.44E-16
ENSG000001	58.361249	116.12974	0.592754	0.0051042	-7.614088	6.81E-10	7.81E-08
ENSG000002	110.97362	183.99796	37.949279	0.2062484	-2.277545	0.0023885	0.0473519
ENSG000002	503.67245	266.41162	740.93328	2.7811597	1.4756866	0.0010615	0.0249991
ENSG000000	586.9608	728.03517	445.88642	0.6124518	-0.707332	0.044865	0.3749509
ENSG000002	429.49522	569.02891	289.96153	0.5095726	-0.97264	0.0115684	0.1526342
ENSG000001	1027.5091	1399.1945	655.82359	0.4687151	-1.093217	0.0074876	0.1118217
ENSG000000	6279.2318	9312.0449	3246.4188	0.3486258	-1.520249	9.25E-06	0.000477
ENSG000001	45.554085	83.045414	8.0627566	0.0970885	-3.364555	0.0015376	0.0336913
ENSG000001	11.504637	0	23.009274	Inf	Inf	0.0355878	0.3250221
ENSG000001	139.90686	72.621477	207.19224	2.8530436	1.5125018	0.0453566	0.3771958
ENSG000001	667.03279	1246.3497	87.715852	0.0703782	-3.828728	0.0048302	0.0808898
ENSG000000	206.49108	294.90818	118.07397	0.4003754	-1.320575	0.0218421	0.236617
ENSG000001	232.79494	107.51832	358.07156	3.3303307	1.7356654	0.0043615	0.0753678
ENSG000001	131.06757	62.435705	199.69944	3.1984814	1.6773871	0.0069777	0.1065599
ENSG000001	7436.328	10745.563	4127.0934	0.3840742	-1.380543	0.0039568	0.0699832
ENSG000002	166.08633	61.239838	270.93283	4.4241271	2.1453928	1.17E-05	0.0005837
ENSG000000	3773.2249	4696.3287	2850.1211	0.6068828	-0.72051	0.0132448	0.1675628
ENSG000001	121.49298	54.466107	188.51985	3.4612322	1.7912857	0.0095222	0.1330308
ENSG000002	192.98471	375.35391	10.615506	0.0282813	-5.144006	2.61E-15	6.48E-13
ENSG000001	103.88631	19.418877	188.35375	9.6995182	3.2779131	6.28E-05	0.0024804
ENSG000001	711.7894	958.34904	465.22975	0.4854492	-1.042608	0.007883	0.1159985
ENSG000001	2293.1227	2973.6309	1612.6144	0.5423048	-0.882824	0.0062697	0.098472
ENSG000002	19.734796	0	39.469593	Inf	Inf	0.0029144	0.0554614
ENSG000001	12.304529	0	24.609058	Inf	Inf	0.0271715	0.2730012
ENSG000001	1485.5758	832.54511	2138.6066	2.5687576	1.3610708	0.0432717	0.3650176
ENSG000000	146.28875	60.374953	232.20255	3.846008	1.9433618	0.0001526	0.0051674
ENSG000002	95.451084	151.50292	39.399247	0.260056	-1.943106	0.0110945	0.148041
ENSG000002	159.71575	54.952511	264.47898	4.8128644	2.2668958	0.0053248	0.0866993
ENSG000001	520.60788	152.68872	888.52705	5.8192056	2.5408222	4.80E-06	0.0002626
ENSG000001	1396.1644	2255.1771	537.15164	0.238186	-2.069839	4.78E-08	4.17E-06
ENSG000001	827.2895	476.92025	1177.6587	2.4692991	1.3041016	0.0016095	0.0348431
ENSG000002	221.55283	423.12399	19.98167	0.0472241	-4.404331	1.57E-13	2.97E-11
ENSG000001	12.422429	0	24.844858	Inf	Inf	0.0257189	0.2638287
ENSG000000	750.1915	932.86086	567.52214	0.6083674	-0.716985	0.0497944	0.3998476
ENSG000001	439.85044	322.74278	556.95809	1.7257027	0.787184	0.0169421	0.2004681
ENSG000000	19.732092	38.278675	1.185508	0.0309705	-5.012964	0.0148149	0.1822304
ENSG000001	57.435709	97.021708	17.849709	0.1839764	-2.442407	0.0043739	0.0754869
ENSG000001	1533.1686	1968.4846	1097.8526	0.5577146	-0.842401	0.0135684	0.1706206
ENSG000000	423.82303	266.0281	581.61796	2.1863027	1.1284932	0.0232727	0.247075
ENSG000001	235.124	131.25659	338.99141	2.582662	1.3688588	0.0016883	0.0360877
ENSG000002	40.179182	6.317538	74.040825	11.719886	3.5508866	0.0242524	0.2542106
ENSG000001	191.95133	106.57069	277.33197	2.6023287	1.3798032	0.0322745	0.3049516
ENSG000001	93.733137	139.40175	48.064526	0.3447914	-1.536204	0.0456466	0.378793
ENSG000000	91.543564	44.9898	138.09733	3.0695252	1.6180155	0.0174455	0.2054366
ENSG000001	494.87494	272.02977	717.72012	2.6383882	1.3996568	0.0044835	0.0768869

ENSG000001	1665.8787	2932.8053	398.95205	0.1360309	-2.877994	0.0280045	0.2779747
ENSG000001	228.07668	131.72285	324.4305	2.4629781	1.3004038	0.0008342	0.020787
ENSG000001	171.89811	77.520086	266.27614	3.4349309	1.7802811	0.0062623	0.0984683
ENSG000001	58695.008	14353.238	103036.78	7.1786436	2.8437113	8.33E-14	1.70E-11
ENSG000002	58.176666	28.175649	88.177682	3.1295705	1.6459647	0.0222331	0.2394155
ENSG000001	4315.4192	3316.9786	5313.8597	1.6020181	0.6798905	0.0179104	0.2080974
ENSG000002	10.346162	0	20.692324	Inf	Inf	0.0486939	0.3934622
ENSG000001	70.140142	107.84427	32.436016	0.3007672	-1.733281	0.0136596	0.1713692
ENSG000000	986.48591	415.78833	1557.1835	3.7451351	1.9050178	0.019345	0.2177937
ENSG000001	24.347854	0	48.695709	Inf	Inf	0.0050393	0.083208
ENSG000001	511.94834	223.14152	800.75516	3.5885529	1.8434022	2.02E-05	0.0009318
ENSG000000	3743.0186	5008.2483	2477.7889	0.4947416	-1.015253	0.0028851	0.0550202
ENSG000000	733.16778	965.68974	500.64582	0.5184334	-0.947769	0.042333	0.359339
ENSG000001	187.31361	98.513312	276.1139	2.802808	1.4868729	0.0096338	0.1340356
ENSG000002	7.7364665	15.472933	0	0	#NAME?	0.032223	0.3046769
ENSG000002	59.373662	6.317538	112.42979	17.796456	4.153518	0.0012889	0.0293354
ENSG000001	144.63297	55.403668	233.86227	4.2210611	2.0776057	8.60E-05	0.0032533
ENSG000001	486.63007	261.1295	712.13064	2.7271168	1.4473765	0.0023731	0.0471851
ENSG000001	51.714109	98.357891	5.0703258	0.0515498	-4.27789	3.69E-05	0.0015456
ENSG000002	623.40852	216.07205	1030.745	4.7703762	2.2541031	9.69E-06	0.000496
ENSG000001	3034.1809	2013.8091	4054.5527	2.0133749	1.0096158	0.0014056	0.0313835
ENSG000001	298.92146	140.90846	456.93445	3.2427752	1.697229	0.0019659	0.0408912
ENSG000001	1527.0574	803.55577	2250.559	2.8007502	1.4858133	0.0016031	0.0347606
ENSG000002	13.062484	26.124967	0	0	#NAME?	0.0061676	0.0974328
ENSG000001	69.551001	123.15171	15.950292	0.1295174	-2.948782	0.0003583	0.0104929
ENSG000001	237.32631	127.16529	347.48733	2.7325642	1.4502554	0.0053934	0.0877114
ENSG000001	130.98542	214.7733	47.197544	0.2197552	-2.186031	0.0065051	0.1011692
ENSG000001	99.607403	5.0540304	194.16078	38.417018	5.2636736	9.10E-07	6.02E-05
ENSG000002	17.964505	33.793793	2.1352168	0.0631837	-3.984304	0.031484	0.3003131
ENSG000001	511.00246	640.30917	381.69574	0.5961116	-0.746346	0.0426761	0.3611753
ENSG000002	731.5374	915.80855	547.26625	0.5975771	-0.742803	0.0453314	0.3771385
ENSG000001	147.54381	228.69203	66.395602	0.2903276	-1.784246	0.0020981	0.0430772
ENSG000001	12.197598	24.395197	0	0	#NAME?	0.0035584	0.0647138
ENSG000001	542.79793	57.489596	1028.1063	17.883345	4.1605447	3.99E-11	5.84E-09
ENSG000001	327.4789	222.02336	432.93443	1.9499499	0.963437	0.0069478	0.106283
ENSG000001	22.453527	44.907055	0	0	#NAME?	0.0039306	0.0697021
ENSG000001	276.20073	113.83586	438.56559	3.8526137	1.9458375	0.0010346	0.0245384
ENSG000001	2555.9031	1482.6638	3629.1424	2.4477178	1.2914372	0.0013991	0.0313417
ENSG000002	103.36942	40.893471	165.84538	4.0555466	2.0198964	0.0186034	0.2133315
ENSG000000	123.80838	0	247.61677	Inf	Inf	3.39E-14	7.52E-12
ENSG000001	136.42765	188.76633	84.088982	0.445466	-1.166613	0.0342652	0.3177338
ENSG000000	47.476334	0	94.952668	Inf	Inf	0.0003243	0.0096664
ENSG000001	27.929783	0	55.859566	Inf	Inf	0.0002294	0.0072093
ENSG000002	1966.2894	2418.4279	1514.1508	0.6260889	-0.675561	0.026797	0.270883
ENSG000001	1934.1368	1470.4713	2397.8023	1.6306352	0.7054341	0.0354944	0.3242788
ENSG000001	426.50347	588.69603	264.31092	0.4489769	-1.155287	0.0124546	0.1607217
ENSG000001	43429.936	25798.752	61061.119	2.3668245	1.2429527	0.0003138	0.0094105
ENSG000001	508.63644	801.30787	215.96501	0.2695156	-1.891559	0.0247578	0.257979
ENSG000001	170.29251	62.606231	277.97879	4.4401137	2.1505966	0.0038405	0.0684191
ENSG000001	257.2503	367.78293	146.71767	0.3989246	-1.325812	0.0072472	0.109346
ENSG000002	13.417216	26.834431	0	0	#NAME?	0.0062698	0.098472

ENSG00000172.960297	132.99139	12.9292	0.0972183	-3.362628	0.0256425	0.2635437	
ENSG0000005976.2958	3826.4578	8126.1339	2.1236701	1.0865596	0.0005502	0.0149649	
ENSG000001	158.978	59.510068	258.44592	4.3428941	2.1186568	9.59E-05	0.003578
ENSG000001204.34674	4.4222766	404.2712	91.416987	6.5143904	7.11E-14	1.46E-11	
ENSG000002	105.3936	24.074285	186.71292	7.7556997	2.9552569	9.91E-05	0.0036692
ENSG000001544.42145	1071.5827	17.260211	0.0161072	-5.956149	4.07E-11	5.90E-09	
ENSG000000280.71297	374.82504	186.60089	0.4978346	-1.006261	0.0265288	0.2691259	
ENSG000001713.38396	226.89461	1199.8733	5.2882406	2.4027878	3.28E-06	0.0001868	
ENSG000001595.82227	154.45088	1037.1937	6.7153624	2.7474653	4.90E-11	6.94E-09	
ENSG00000066.184566	129.99812	2.3710159	0.0182388	-5.776842	3.36E-06	0.0001906	
ENSG0000011098.0384	758.76519	1437.3116	1.8942772	0.9216475	0.0289872	0.2848305	
ENSG000000984.35318	608.8618	1359.8446	2.2334208	1.1592551	0.0071004	0.1079465	
ENSG00000177.810401	28.097939	127.52286	4.5385131	2.1822197	0.0030759	0.0579238	
ENSG000001604.19355	1010.15	198.2371	0.1962452	-2.349271	1.18E-07	9.43E-06	
ENSG000001	42.10218	82.661897	1.5424628	0.0186599	-5.743915	6.44E-05	0.0025331
ENSG000002176.75094	111.13328	242.36861	2.1808823	1.1249119	0.0191051	0.2161386	
ENSG000001583.96779	0	1167.9356	Inf	Inf	5.58E-19	2.13E-16	
ENSG00000114.758434	29.516867	0	0	#NAME?	0.009923	0.1366755	
ENSG00000213.772084	0	27.544167	Inf	Inf	0.0170718	0.2016304	
ENSG000000209.67921	0	419.35841	Inf	Inf	4.89E-21	2.40E-18	
ENSG000001229.08206	363.67653	94.487582	0.2598122	-1.944459	0.0003282	0.0097388	
ENSG000001220.86299	19.967885	421.75809	21.121821	4.4006623	2.67E-12	4.33E-10	
ENSG000001	18.06955	0	36.1391	Inf	Inf	0.0042973	0.0745922
ENSG0000012522.0295	1632.7348	3411.3242	2.0893314	1.0630414	0.0036443	0.065922	
ENSG000001719.38185	423.51758	1015.2461	2.3971759	1.2613357	0.005622	0.0908679	
ENSG000001337.77201	0	675.54402	Inf	Inf	2.36E-06	0.0001409	
ENSG000001438.76609	828.46388	49.0683	0.059228	-4.077576	6.00E-08	5.07E-06	
ENSG000000663.01856	400.86223	925.17489	2.3079623	1.2066196	0.0084713	0.1221467	
ENSG000001928.11068	1203.8905	652.3309	0.5418524	-0.884028	0.0120039	0.1568502	
ENSG000001552.12806	795.04572	309.2104	0.3889215	-1.362449	0.0032616	0.0606163	
ENSG0000013017.4151	3646.5107	2388.3196	0.6549603	-0.610521	0.0449829	0.375704	
ENSG000001126.50618	8.2127994	244.79956	29.807079	4.8975831	7.38E-07	5.01E-05	
ENSG00000210.390781	0	20.781562	Inf	Inf	0.0481792	0.3908202	
ENSG00000177.571268	43.726293	111.41624	2.5480377	1.3493866	0.0377697	0.3362128	
ENSG00000011570.316	7161.2742	15979.357	2.2313567	1.1579212	0.0021901	0.0445451	
ENSG000001183.79738	78.136735	289.45802	3.7045062	1.8892813	0.000198	0.0064159	
ENSG000000230.75154	141.2668	320.23628	2.2668899	1.1807143	0.0068214	0.1047732	
ENSG0000011582.1515	1096.6477	2067.6553	1.8854325	0.9148955	0.0302663	0.2930358	
ENSG0000013052.7306	1097.1004	5008.3609	4.5650889	2.1906429	4.26E-12	6.78E-10	
ENSG000002310.28159	0	620.56318	Inf	Inf	1.11E-10	1.45E-08	
ENSG000001149.42805	0	298.8561	Inf	Inf	1.30E-16	3.83E-14	
ENSG00000175.470956	38.123254	112.81866	2.9593134	1.5652625	0.0306018	0.2949644	
ENSG00000228.734559	0	57.469119	Inf	Inf	0.0002011	0.0064869	
ENSG000001225.77282	90.947372	360.59826	3.9649113	1.9872886	0.0027878	0.0535799	
ENSG00000117.053073	0	34.106146	Inf	Inf	0.0277245	0.2758281	
ENSG0000012455.6445	3018.6056	1892.6835	0.6270059	-0.673449	0.0338907	0.315378	
ENSG000002612.05253	329.62443	894.48064	2.7136357	1.4402271	0.0003386	0.0100029	
ENSG000001892.17059	1085.7201	698.62107	0.6434633	-0.63607	0.0465467	0.3828569	
ENSG000000	344.4566	220.14321	468.77	2.1293866	1.0904379	0.0082455	0.11974
ENSG000001964.79911	471.15457	1458.4436	3.0954675	1.6301573	2.78E-05	0.0012211	
ENSG000001209.23952	119.42883	299.05021	2.5040036	1.3242366	0.0128423	0.163928	

ENSG000001	207.89687	108.84947	306.94426	2.8198967	1.4956423	0.0047309	0.0798145
ENSG000001	120.608	216.00441	25.21158	0.1167179	-3.098902	0.0005686	0.0153263
ENSG000002	46.371624	9.476307	83.266941	8.7868556	3.135347	0.0376187	0.3358625
ENSG000001	825.81217	1119.7046	531.91976	0.4750537	-1.073838	0.0253003	0.2612356
ENSG000001	785.17353	322.76292	1247.5841	3.8653267	1.9505904	3.38E-06	0.0001913
ENSG000002	20.590383	0	41.180765	Inf	Inf	0.002015	0.0416569
ENSG00000(	1220.9942	1654.3677	787.62079	0.4760857	-1.070707	0.0037618	0.0674615
ENSG000001	4946.2711	59.209296	9833.3329	166.07752	7.375713	2.57E-44	5.80E-41
ENSG000001	5329.5062	2832.562	7826.4504	2.7630288	1.4662506	0.0004535	0.0127047
ENSG000001	364.38099	248.1584	480.60357	1.9366806	0.9535861	0.0121145	0.1579903
ENSG000002	38.343746	67.499806	9.1876867	0.1361143	-2.87711	0.0074016	0.1108604
ENSG000002	651.42389	841.95881	460.88897	0.5474009	-0.86933	0.0271633	0.2730012
ENSG000001	181.48519	351.58039	11.389993	0.0323966	-4.948016	3.98E-14	8.76E-12
ENSG00000(	1731.8257	882.70123	2580.9502	2.9239228	1.5479052	1.15E-05	0.0005751
ENSG000001	451.13219	574.78737	327.477	0.5697359	-0.811635	0.0370875	0.3326329
ENSG000001	163.2603	21.081007	305.43958	14.488852	3.8568714	1.27E-06	8.17E-05
ENSG000001	924.53732	1174.0628	675.01188	0.5749368	-0.798525	0.0179762	0.2085998
ENSG000001	636.93386	419.36083	854.50688	2.0376411	1.0269	0.0031348	0.0586654
ENSG000001	476.11425	294.3016	657.92689	2.2355532	1.1606319	0.0266829	0.270133
ENSG00000(	990.35278	672.67615	1308.0294	1.9445158	0.959411	0.0072597	0.1093846
ENSG000001	5981.1463	9072.7534	2889.5392	0.3184854	-1.650701	1.43E-06	9.11E-05
ENSG00000(	317.49287	88.503102	546.48263	6.1747286	2.6263757	2.45E-05	0.0010921
ENSG000001	394.17209	113.189	675.15519	5.9648481	2.5764854	9.43E-08	7.60E-06
ENSG000001	2839.3265	4119.5936	1559.0594	0.3784498	-1.401826	0.0004814	0.0133585
ENSG000001	2374.8667	3209.0514	1540.6821	0.4801051	-1.058578	0.0025067	0.0490482
ENSG000001	132.27341	48.96092	215.5859	4.4032241	2.1385603	0.0456699	0.3788696
ENSG00000(	275.43624	373.3759	177.49658	0.4753831	-1.072838	0.0382701	0.3384871
ENSG000001	964.62535	601.1354	1328.1153	2.2093447	1.1436185	0.0099482	0.1369314
ENSG000002	16.67336	31.893497	1.4532241	0.0455649	-4.455933	0.010354	0.1407218
ENSG000002	13.429459	0	26.858919	Inf	Inf	0.0182447	0.2103619
ENSG000002	64.235173	114.23448	14.235864	0.1246197	-3.004396	0.0001841	0.006025
ENSG000001	49.095333	0	98.190666	Inf	Inf	5.36E-07	3.75E-05
ENSG000001	627.26524	833.04661	421.48386	0.5059547	-0.98292	0.0214088	0.2334124
ENSG000001	2401.4626	3124.2812	1678.6439	0.5372896	-0.896228	0.0073373	0.110247
ENSG000002	88.003783	31.728006	144.27956	4.5473882	2.1850382	0.0015164	0.0333902
ENSG000001	285.13979	74.273537	496.00605	6.6780993	2.7394375	1.85E-07	1.40E-05
ENSG000001	137.52664	45.155292	229.89798	5.0912744	2.3480268	0.001402	0.0313658
ENSG000001	868.96791	619.22028	1118.7155	1.806652	0.8533186	0.0088593	0.1261567
ENSG000001	58.334308	2.5270152	114.1416	45.168546	5.4972466	0.0028148	0.0539449
ENSG000001	841.68324	1280.8162	402.55026	0.314292	-1.669823	2.75E-05	0.001211
ENSG00000(	45.936009	87.24463	4.6273883	0.0530392	-4.236796	0.0063751	0.0997819
ENSG000002	23.500087	0	47.000173	Inf	Inf	0.0009237	0.0225196
ENSG000001	1148.3958	1682.9448	613.84684	0.3647457	-1.455037	0.000165	0.0055123
ENSG000002	268.56	157.31392	379.80609	2.4143197	1.2716167	0.0075062	0.1120383
ENSG000002	785.17873	542.95144	1027.406	1.8922613	0.9201113	0.0458485	0.3791888
ENSG000001	485.38804	921.81591	48.960169	0.0531127	-4.234798	0.0163411	0.1958109
ENSG000001	216.64211	0	433.28423	Inf	Inf	1.24E-21	6.44E-19
ENSG000001	76.410781	128.74468	24.076882	0.1870126	-2.418792	0.003909	0.0694346
ENSG000001	1173.0868	1761.7177	584.45603	0.3317535	-1.591816	0.0001887	0.0061516
ENSG000001	101.43536	184.33901	18.531702	0.1005305	-3.314294	2.13E-06	0.0001302
ENSG000001	341.04123	129.68727	552.39518	4.2594401	2.0906638	2.19E-05	0.000991

ENSG000001	685.41933	895.16863	475.67004	0.5313748	-0.912198	0.0319441	0.3029935
ENSG000001	492.36339	725.98449	258.7423	0.356402	-1.488423	0.0013148	0.0297994
ENSG000001	17.679036	35.358073	0	0	#NAME?	0.0022069	0.0447349
ENSG000002	392.2174	190.64648	593.78831	3.1146041	1.6390488	0.0021121	0.0433006
ENSG000001	642.16263	422.34185	861.9834	2.0409613	1.0292489	0.0370405	0.332455
ENSG000002	125.31867	248.26632	2.3710159	0.0095503	-6.710239	3.79E-15	9.07E-13
ENSG000001	244.39516	425.67115	63.119174	0.1482815	-2.753589	1.68E-07	1.29E-05
ENSG000001	44.790236	86.763262	2.8172094	0.0324701	-4.944746	5.96E-06	0.0003179
ENSG000001	160.62417	222.9235	98.324846	0.4410699	-1.180921	0.0452608	0.3769782
ENSG000000	1054.8661	1466.2001	643.53209	0.4389115	-1.187998	0.0132618	0.1676216
ENSG000002	238.47522	151.87637	325.07407	2.1403861	1.0978711	0.0391827	0.3430462
ENSG000002	61.735413	94.737895	28.732931	0.3032887	-1.721236	0.0186996	0.2140109
ENSG000002	28.141829	56.283658	0	0	#NAME?	6.04E-06	0.0003217
ENSG000001	867.9038	1121.5042	614.30344	0.5477496	-0.868412	0.0213216	0.2328685
ENSG000001	1054.1422	1767.0652	341.21912	0.1930993	-2.372585	2.88E-09	3.06E-07
ENSG000002	19.363511	0	38.727022	Inf	Inf	0.0032093	0.0598524
ENSG000002	31.79229	62.042118	1.5424628	0.0248615	-5.32994	0.000161	0.0053969
ENSG000001	1618.8262	2205.3233	1032.3291	0.4681078	-1.095087	0.0082795	0.120006
ENSG000002	15.714493	0	31.428985	Inf	Inf	0.0093859	0.1316021
ENSG000001	3644.371	2416.5276	4872.2144	2.0162047	1.0116421	0.021939	0.2372864
ENSG000001	463.8188	309.32556	618.31203	1.9989037	0.999209	0.0049556	0.0823733
ENSG000001	251.01585	378.77602	123.25569	0.3254052	-1.619691	0.0006007	0.0160476
ENSG000000	9.1942503	18.388501	0	0	#NAME?	0.0385889	0.340302
ENSG000002	102.69612	43.648582	161.74365	3.7055878	1.8897024	0.005004	0.0828715
ENSG000001	250.59101	111.47434	389.70769	3.4959409	1.8056808	0.0065462	0.1015757
ENSG000002	7.3428792	14.685758	0	0	#NAME?	0.0301119	0.2920755
ENSG000001	94.423513	175.62471	13.222321	0.0752874	-3.731448	9.77E-06	0.0004989
ENSG000001	4263.9849	6586.2552	1941.7146	0.2948131	-1.762127	3.26E-06	0.0001864
ENSG000001	1297.9555	1686.6246	909.28646	0.5391161	-0.891332	0.0190126	0.2155539
ENSG000001	8315.2413	1240.9899	15389.493	12.400982	3.6323824	5.32E-21	2.57E-18
ENSG000001	1126.3637	783.71377	1469.0136	1.8744261	0.906449	0.0128131	0.1637093
ENSG000001	2818.5924	1283.9376	4353.2472	3.3905443	1.7615169	4.60E-05	0.0018928
ENSG000001	15.066045	0	30.13209	Inf	Inf	0.0321623	0.3042956
ENSG000001	44.14597	10.81249	77.47945	7.1657361	2.8411149	0.0046734	0.0791895
ENSG000001	64.634519	20.682384	108.58665	5.2501999	2.3923724	0.0156462	0.1898657
ENSG000002	44.591178	89.182356	0	0	#NAME?	0.001771	0.0375295
ENSG000001	96.361273	0	192.72255	Inf	Inf	6.06E-05	0.0024142
ENSG000001	154.82103	32.364795	277.27726	8.5672491	3.098832	9.68E-06	0.000496
ENSG000002	111.39012	58.722893	164.05735	2.7937545	1.4822053	0.0122271	0.158771
ENSG000001	6090.5258	4115.9434	8065.1082	1.9594799	0.9704708	0.0331237	0.3110202
ENSG000000	1599.294	400.78955	2797.7985	6.9807172	2.8033753	3.29E-10	4.01E-08
ENSG000001	16.811634	33.623267	0	0	#NAME?	0.0013822	0.0310652
ENSG000001	293.21298	429.59695	156.82901	0.3650608	-1.453791	0.0035157	0.0641102
ENSG000001	1073.8507	794.13552	1353.5659	1.7044521	0.769308	0.0131313	0.1667506
ENSG000002	359.25038	161.67359	556.82717	3.4441443	1.7841456	0.0010137	0.0243191
ENSG000001	1693.7164	2039.6094	1347.8234	0.6608243	-0.597661	0.0406733	0.3496379
ENSG000001	299.37243	179.61311	419.13174	2.3335252	1.2225111	0.0057802	0.092775
ENSG000001	875.69855	1136.5432	614.85387	0.5409859	-0.886337	0.0409743	0.3512217
ENSG000001	44.190782	0	88.381565	Inf	Inf	2.15E-06	0.0001308
ENSG000001	1646.8867	1172.4654	2121.308	1.8092713	0.8554087	0.0251326	0.2602678
ENSG000000	1297.903	1781.1387	814.66731	0.4573857	-1.128517	0.0294112	0.2874306

ENSG00000(	496.53446	679.14189	313.92703	0.4622407	-1.113284	0.0048084	0.0808201
ENSG000001	732.24672	930.37916	534.11427	0.5740824	-0.80067	0.0271011	0.2729641
ENSG000001	289.99118	103.48963	476.49272	4.6042556	2.2029679	8.52E-05	0.0032308
ENSG000001	23.466542	0	46.933083	Inf	Inf	0.0008466	0.0209987
ENSG000001	53.647813	0	107.29563	Inf	Inf	1.64E-07	1.26E-05
ENSG000001	674.9301	1020.6746	329.18556	0.3225176	-1.63255	2.98E-05	0.0012875
ENSG000002	321.46802	457.09335	185.84268	0.4065749	-1.298407	0.0023312	0.0466213
ENSG00000(	857.68916	406.26956	1309.1088	3.2222664	1.6880758	1.71E-05	0.0008026
ENSG000001	477.268	265.08047	689.45553	2.6009291	1.3790271	0.0045339	0.0774577
ENSG00000(	184.96694	105.6907	264.24318	2.5001555	1.3220178	0.0113344	0.1503526
ENSG000001	158.24038	236.89475	79.586006	0.3359551	-1.57366	0.0086386	0.1241472
ENSG00000(	654.75372	840.16358	469.34386	0.5586339	-0.840025	0.0399789	0.3464198
ENSG000001	86.048461	14.209425	157.8875	11.111462	3.4739768	0.0001611	0.0053969
ENSG000002	290.87937	372.85207	208.90667	0.5602937	-0.835745	0.0380486	0.3375789
ENSG000001	825.44217	392.5264	1258.3579	3.2057919	1.6806808	1.09E-05	0.0005498
ENSG000001	79.309509	43.094539	115.52448	2.680722	1.4226216	0.0325606	0.3069047
ENSG000001	735.65745	971.62594	499.68896	0.5142812	-0.959371	0.0118267	0.1549093
ENSG000001	972.18434	1215.37	728.99873	0.5998163	-0.737407	0.0288688	0.2842858
ENSG000001	257.49549	348.61733	166.37365	0.4772386	-1.067217	0.0231737	0.2465135
ENSG000001	1218.3996	1619.2456	817.55354	0.5048978	-0.985937	0.0100645	0.1381111
ENSG000002	161.91138	0	323.82276	Inf	Inf	4.16E-08	3.65E-06
ENSG00000(	204.86769	320.81512	88.920253	0.2771698	-1.851158	0.0006439	0.0169705
ENSG000001	106.35788	188.25978	24.455986	0.1299055	-2.944465	0.0001657	0.005515
ENSG000001	154.55269	11.055692	298.0497	26.958937	4.7526917	9.58E-08	7.69E-06
ENSG000002	380.47373	760.94745	0	0	#NAME?	0.0176189	0.2067583
ENSG000001	166.89472	105.37986	228.40958	2.167488	1.116024	0.0479933	0.3897826
ENSG000002	109.87498	1.5793845	218.17057	138.13645	7.1099502	3.66E-10	4.44E-08
ENSG000001	2671.6875	1603.8554	3739.5197	2.3315815	1.2213089	0.0077022	0.1142084
ENSG000001	11427.063	14674.2	8179.9272	0.557436	-0.843122	0.0046452	0.0788817
ENSG000001	504.87161	171.26285	838.48038	4.8958684	2.2915648	0.0002877	0.0086998
ENSG000001	590.43367	1172.1193	8.7480053	0.0074634	-7.06595	3.21E-37	4.13E-34
ENSG000001	182.49098	81.222828	283.75912	3.4935883	1.8047096	0.0013128	0.029778
ENSG000001	295.22802	493.44437	97.01167	0.196601	-2.346657	0.0139886	0.1741238
ENSG000001	343.63194	218.589	468.67489	2.1440918	1.1003667	0.0423064	0.3592263
ENSG000001	394.50411	231.66516	557.34306	2.405813	1.2665245	0.0019546	0.0407234
ENSG00000(	2584.7003	1822.1257	3347.2748	1.8370164	0.8773645	0.0384093	0.3390069
ENSG000001	2191.7438	3376.8201	1006.6675	0.2981111	-1.746078	0.0002434	0.0075804
ENSG000001	447.54877	580.62858	314.46897	0.5416009	-0.884698	0.0186887	0.2140109
ENSG000002	272.09773	138.15838	406.03708	2.9389247	1.5552884	0.0177499	0.2075715
ENSG000001	478.35613	752.43036	204.28189	0.2714961	-1.880997	0.0001295	0.0045615
ENSG000002	56.579666	0	113.15933	Inf	Inf	2.58E-06	0.0001522
ENSG000002	18.324564	0	36.649127	Inf	Inf	0.0042804	0.0744418
ENSG000001	923.31724	1250.9533	595.68121	0.4761818	-1.070416	0.0082078	0.1194151
ENSG000001	167.88189	6.6283798	329.13539	49.655482	5.6338811	1.28E-05	0.0006276
ENSG00000(	44.379258	76.42207	12.336446	0.1614252	-2.631062	0.0037661	0.0674932
ENSG000001	1102.2754	1402.5267	802.02408	0.5718423	-0.806311	0.0299874	0.2912743
ENSG000001	1343.0615	649.3365	2036.7865	3.1367195	1.6492565	4.33E-05	0.0017924
ENSG000001	591.14097	1025.864	156.41799	0.1524744	-2.713361	2.73E-10	3.37E-08
ENSG000002	12.780008	24.788784	0.7712314	0.0311121	-5.00638	0.0159206	0.1923336
ENSG000001	3467.4727	4667.2149	2267.7306	0.4858852	-1.041313	0.0018565	0.0388552
ENSG000001	678.93467	448.21355	909.6558	2.0295143	1.0211345	0.0181175	0.2096746

ENSG000001	786.64925	521.58696	1051.7115	2.0163686	1.0117594	0.0043376	0.0751312
ENSG000001	1247.9395	1757.5616	738.31734	0.4200805	-1.251262	0.0004944	0.0135839
ENSG000001	118.49661	40.417138	196.57609	4.8636816	2.2820488	0.0022772	0.045795
ENSG000001	65.090965	8.8445532	121.33738	13.718881	3.7780909	0.002572	0.0501092
ENSG000002	125.15853	59.209296	191.10777	3.2276649	1.6904908	0.0382924	0.3385002
ENSG000002	693.28946	993.08543	393.4935	0.3962333	-1.335578	0.0006905	0.0179533
ENSG000001	49.265104	92.035318	6.494889	0.0705695	-3.824811	0.0003767	0.0109609
ENSG000001	700.46801	936.8716	464.06443	0.4953341	-1.013526	0.0370284	0.332455
ENSG000000	7454.0536	11153.788	3754.3196	0.3365959	-1.57091	0.0333055	0.311538
ENSG000001	206.29917	6.9442567	405.65408	58.415766	5.8682859	4.18E-17	1.35E-14
ENSG000002	27.077109	6.6283798	47.525837	7.1700534	2.8419839	0.0077445	0.1145844
ENSG000001	123.70757	197.01152	50.403625	0.255841	-1.966681	0.022363	0.240431
ENSG000001	1175.1307	612.29398	1737.9675	2.8384527	1.5051047	0.0013879	0.0311355
ENSG000001	111.63854	47.361395	175.91568	3.7143265	1.8931006	0.0075249	0.112256
ENSG000001	40.122603	0	80.245206	Inf	Inf	0.0049041	0.0817688
ENSG000001	108.43465	216.8693	0	0	#NAME?	1.33E-12	2.24E-10
ENSG000002	276.20709	196.85106	355.56311	1.8062545	0.8530012	0.025141	0.2602678
ENSG000000	1268.3383	1655.6189	881.05769	0.5321621	-0.910062	0.0049232	0.0820368
ENSG000001	73.091162	115.96425	30.218072	0.2605809	-1.940197	0.010814	0.1454467
ENSG000002	742.14002	474.39609	1009.884	2.128778	1.0900255	0.0045445	0.077589
ENSG000001	360.73739	194.98098	526.49381	2.7002317	1.4330832	0.0045111	0.0771644
ENSG000002	2398.2625	1154.9648	3641.5602	3.1529621	1.6567078	0.0001993	0.0064508
ENSG000001	24.683967	0	49.367933	Inf	Inf	0.0006139	0.0163518
ENSG000001	259.50428	87.623112	431.38546	4.9231926	2.2995942	7.52E-05	0.0028899
ENSG000001	25.629234	3.158769	48.099699	15.227356	3.9285935	0.0496049	0.3987994
ENSG000001	1083.9084	398.11215	1769.7046	4.4452414	2.1522618	2.94E-06	0.0001697
ENSG000001	383.95386	190.63641	577.27131	3.0281272	1.5984258	0.0013816	0.0310652
ENSG000001	356.5997	210.88996	502.30944	2.3818556	1.252086	0.0005881	0.015792
ENSG000002	104.53331	173.18547	35.881152	0.2071834	-2.27102	0.0018226	0.0382747
ENSG000001	772.13549	457.02286	1087.2481	2.3789797	1.250343	0.000869	0.0214366
ENSG000001	1111.032	1451.5725	770.4915	0.5307978	-0.913766	0.0122782	0.159128
ENSG000001	67.2677	6.2347925	128.30061	20.578168	4.3630426	6.65E-06	0.0003499
ENSG000001	156.7427	69.141796	244.3436	3.5339492	1.8212813	0.0184528	0.212039
ENSG000001	133.15775	85.950912	180.36459	2.0984605	1.0693313	0.0234892	0.2486857
ENSG000001	118.72488	197.65838	39.791375	0.2013139	-2.312481	0.0008915	0.0218927
ENSG000002	193.50616	113.28182	273.7305	2.4163675	1.2728399	0.048916	0.3944329
ENSG000002	230.20207	314.104	146.30013	0.4657697	-1.102311	0.0290161	0.2849075
ENSG000001	279.87685	414.61042	145.14329	0.3500715	-1.514279	0.0010023	0.0241105
ENSG000002	47.27285	75.153527	19.392172	0.2580341	-1.954366	0.0320738	0.3037973
ENSG000001	507.88276	356.63946	659.12607	1.848158	0.8860881	0.0420176	0.3576715
ENSG000001	7706.1009	11496.672	3915.5298	0.3405794	-1.553937	2.59E-06	0.0001525
ENSG000001	35.127459	70.254917	0	0	#NAME?	4.11E-07	2.91E-05
ENSG000001	1292.5007	860.7409	1724.2606	2.0032283	1.0023269	0.0468574	0.3844776
ENSG000001	326.62484	576.25095	76.998727	0.1336201	-2.903791	0.0271821	0.2730012
ENSG000002	85.444508	38.439131	132.44988	3.4457044	1.784799	0.0126781	0.1624289
ENSG000001	924.31377	1285.2788	563.34875	0.4383086	-1.189981	0.0006441	0.0169705
ENSG000001	39.721043	64.734624	14.707462	0.2271962	-2.137989	0.0275343	0.2748454
ENSG000001	374.96037	207.13469	542.78606	2.6204499	1.3898145	0.0052336	0.0854718
ENSG000001	5524.0828	7072.7768	3975.3888	0.562069	-0.831181	0.0117891	0.1545101
ENSG000000	314.52973	452.32498	176.73447	0.3907245	-1.355776	0.0059278	0.0945274
ENSG000002	56.805723	94.72279	18.888657	0.1994098	-2.326191	0.0141462	0.1757621

ENSG000002	343.10844	48.629937	637.58694	13.110997	3.7127054	2.75E-08	2.49E-06
ENSG000001	1212.4738	2038.8798	386.06779	0.1893529	-2.400851	6.87E-10	7.85E-08
ENSG000001	63.994235	6.0016611	121.98681	20.325508	4.3452195	0.0454367	0.37763
ENSG000001	481.79342	296.39475	667.19208	2.2510253	1.1705823	0.0037846	0.0676455
ENSG000001	225.14762	105.36979	344.92546	3.2734758	1.7108233	0.0003971	0.011433
ENSG000001	595.31589	365.32356	825.30822	2.2591158	1.1757582	0.0062568	0.0984391
ENSG000001	423.61164	311.5216	535.70167	1.7196293	0.7820976	0.0203844	0.2263533
ENSG000001	123.75377	24.633363	222.87417	9.047655	3.1775439	0.0002128	0.0068067
ENSG000001	911.56231	1144.4279	678.69672	0.5930445	-0.753788	0.0320598	0.3037711
ENSG000001	396.66679	300.96741	492.36616	1.6359451	0.7101243	0.0447127	0.374025
ENSG000001	50.46398	81.232898	19.695061	0.2424518	-2.04423	0.0136309	0.1712474
ENSG000001	187.49323	269.66321	105.32325	0.3905733	-1.356335	0.0107309	0.1444723
ENSG000001	824.64175	1200.1604	449.12313	0.3742193	-1.418044	0.0106991	0.1441872
ENSG000002	1775.4735	2272.3072	1278.6399	0.5627056	-0.829548	0.0181795	0.2099677
ENSG000001	1487.3771	1938.98	1035.7743	0.5341851	-0.904588	0.0076012	0.1130195
ENSG000001	184.00807	299.81686	68.199268	0.2274698	-2.136253	0.000135	0.0047081
ENSG000001	866.44048	369.07665	1363.8043	3.6951791	1.8856443	0.0001434	0.0049468
ENSG000001	209.0259	314.91131	103.14048	0.3275223	-1.610335	0.0007008	0.0181356
ENSG000000	5730.9846	4492.7229	6969.2463	1.5512299	0.6334125	0.0260139	0.2660481
ENSG000001	177.87653	295.18663	60.566425	0.2051801	-2.285037	0.0022357	0.0452509
ENSG000001	694.414	381.04473	1007.7833	2.64479	1.4031532	0.002194	0.0445729
ENSG000000	501.41377	637.69437	365.13316	0.5725833	-0.804442	0.0436524	0.3671984
ENSG000001	89.901289	56.827632	122.97495	2.1639991	1.1136999	0.0442782	0.3713405
ENSG000001	333.87646	490.80155	176.95137	0.3605355	-1.471787	0.0241458	0.2534562
ENSG000001	335.20682	122.51492	547.89872	4.4720978	2.1609518	0.001193	0.0273598
ENSG000001	1667.8208	2215.3543	1120.2873	0.5056922	-0.983669	0.0037094	0.0667861
ENSG000001	15.198284	29.211061	1.185508	0.0405842	-4.622937	0.013787	0.1724716
ENSG000001	147.47037	43.26003	251.6807	5.8178578	2.540488	0.0004281	0.0120941
ENSG000002	14.882552	29.765104	0	0	#NAME?	0.0008938	0.0219094
ENSG000001	57.310043	19.97292	94.647166	4.7387746	2.244514	0.0393641	0.3436488
ENSG000001	29.505424	13.25676	45.754088	3.4513779	1.7871725	0.0239328	0.2517086
ENSG000001	37.151873	74.303747	0	0	#NAME?	0.01231	0.1593115
ENSG000001	423.51407	595.08624	251.94191	0.4233704	-1.240008	0.0017845	0.0377278
ENSG000001	23.44846	44.761704	2.1352168	0.0477019	-4.389811	0.002463	0.0484039
ENSG000001	3364.7175	2245.3871	4484.0479	1.9970043	0.9978375	0.0068423	0.1049066
ENSG000002	268.93679	370.46033	167.41325	0.451906	-1.145905	0.0233215	0.2472974
ENSG000001	365.77485	529.12554	202.42416	0.3825636	-1.386229	0.003819	0.0681702
ENSG000001	8176.5855	4919.4703	11433.701	2.3241732	1.2167176	0.0035573	0.0647138
ENSG000000	15.896226	0	31.792452	Inf	Inf	0.0208608	0.2297953
ENSG000001	277.89375	546.77503	9.0124654	0.0164829	-5.922882	0.0049799	0.082523
ENSG000001	553.28445	690.69405	415.87484	0.6021115	-0.731897	0.0308183	0.2960502
ENSG000001	531.13815	304.23914	758.03716	2.4915833	1.3170628	0.0070616	0.1075374
ENSG000001	1504.3762	2123.6874	885.06495	0.4167586	-1.262716	0.0002791	0.008485
ENSG000001	12.722062	0	25.444124	Inf	Inf	0.0254009	0.2620234
ENSG000002	64.316069	29.04557	99.586568	3.428632	1.7776331	0.0198783	0.2225058
ENSG000001	231.65844	448.72731	14.589562	0.0325132	-4.94283	5.19E-08	4.47E-06
ENSG000000	35.334931	0.6317538	70.038108	110.86298	6.7926339	0.0002974	0.0089612
ENSG000001	21174.379	34320.552	8028.2053	0.2339183	-2.095923	9.52E-06	0.0004898
ENSG000001	15118.662	20721	9516.3241	0.4592599	-1.122617	0.0003932	0.0113561
ENSG000001	242.43875	103.73283	381.14467	3.6742915	1.8774661	0.0030071	0.0568656
ENSG000001	240.36799	121.645	359.09097	2.9519583	1.5616724	0.0057554	0.0924857

ENSG000002	45.996583	80.66375	11.329416	0.1404524	-2.831847	0.0377476	0.3361675
ENSG000001	602.21083	1092.0427	112.37898	0.1029071	-3.280585	5.71E-10	6.72E-08
ENSG000001	218.16772	436.33544	0	0	#NAME?	2.35E-16	6.71E-14
ENSG000002	69.475803	120.29875	18.652858	0.1550545	-2.689153	0.0017393	0.0369727
ENSG000001	28.103439	52.736337	3.4705412	0.0658093	-3.925565	0.0015264	0.0335274
ENSG000001	255.80063	142.47777	369.12349	2.5907444	1.3733667	0.0067737	0.1042841
ENSG000001	18.882291	35.450888	2.3136942	0.0652648	-3.937552	0.0135627	0.1706206
ENSG000001	196.47656	80.73139	312.22173	3.8674142	1.9513693	3.74E-06	0.00021
ENSG000000	564.54941	822.30176	306.79706	0.3730955	-1.422383	0.0034794	0.0635322
ENSG000001	836.96732	1044.7238	629.21089	0.6022749	-0.731506	0.0387253	0.3410173
ENSG000002	112.35712	30.324182	194.39006	6.4103975	2.6804138	0.0104887	0.1420332
ENSG000002	111.00311	175.50957	46.496658	0.2649238	-1.916351	0.0309678	0.2970646
ENSG000002	25.655819	1.657095	49.654542	29.964814	4.9051975	0.00437	0.0754668
ENSG000000	3210.1907	4503.4172	1916.9642	0.4256688	-1.232197	0.000693	0.0179974
ENSG000001	82.798632	18.704377	146.89289	7.8533962	2.9733167	7.63E-05	0.0029198
ENSG000002	24.935724	0	49.871449	Inf	Inf	0.0005657	0.015262
ENSG000001	186.14001	48.780323	323.49969	6.631766	2.7293931	6.31E-06	0.0003343
ENSG000000	27.425443	53.694038	1.1568471	0.0215452	-5.536492	0.0396741	0.3449939
ENSG000002	731.21942	1423.0904	39.348437	0.02765	-5.176577	2.74E-13	4.95E-11
ENSG000001	553.96333	333.56534	774.36133	2.3214682	1.2150375	0.0016309	0.0351386
ENSG000002	215.74973	127.56392	303.93555	2.3826138	1.2525451	0.0251168	0.2602319
ENSG000001	177.90268	70.420408	285.38496	4.0525888	2.0188438	0.0045917	0.0782471
ENSG000002	1862.7795	1433.8453	2291.7136	1.5982991	0.6765374	0.0334355	0.3124307
ENSG000001	676.46045	491.28795	861.63296	1.7538247	0.8105046	0.0263534	0.2680691
ENSG000001	256.71364	99.330697	414.09658	4.1688682	2.0596558	0.0068306	0.104845
ENSG000002	22.649318	41.442479	3.8561569	0.0930484	-3.425875	0.0156344	0.1898086
ENSG000001	36.037151	5.0540304	67.020272	13.260758	3.7290913	0.0379551	0.3371662
ENSG000001	1103.7209	1549.8203	657.62139	0.4243211	-1.236772	0.0005214	0.0142954
ENSG000000	93.538689	50.675585	136.40179	2.6916669	1.4284999	0.0463994	0.3821098
ENSG000000	257.14873	401.44363	112.85383	0.28112	-1.830742	0.0244797	0.2558692
ENSG000001	723.5727	503.46683	943.67857	1.874361	0.9063988	0.0283275	0.2803838
ENSG000002	174.28492	81.786942	266.78291	3.2619255	1.7057238	0.0142007	0.1763588
ENSG000001	2915.5397	4262.1893	1568.89	0.3680949	-1.44185	0.0008659	0.0213808
ENSG000001	14.189926	27.787097	0.592754	0.021332	-5.550838	0.0112137	0.1489707
ENSG000001	301.24331	199.62632	402.86031	2.0180721	1.0129777	0.0315305	0.3004386
ENSG000001	469.46886	160.25969	778.67803	4.8588513	2.2806153	2.26E-05	0.0010169
ENSG000002	28.70498	4.1063997	53.303561	12.980607	3.6982859	0.0422676	0.3591545
ENSG000001	312.10072	205.19411	419.00732	2.0420047	1.0299862	0.0110877	0.1480242
ENSG000001	7485.1965	4659.6835	10310.709	2.2127489	1.1458397	0.0031042	0.0582539
ENSG000001	843.59034	52.643521	1634.5372	31.049161	4.9564824	5.39E-24	3.39E-21
ENSG000001	316.9977	160.79863	473.19676	2.942791	1.5571851	0.0027049	0.0522458
ENSG000002	226.48795	79.585874	373.39003	4.6916621	2.2300991	0.0018085	0.0380549
ENSG000002	2436.1299	3590.7012	1281.5587	0.3569104	-1.486366	0.0014025	0.0313658
ENSG000001	293.88098	167.95084	419.81112	2.4996071	1.3217013	0.0014308	0.0318152
ENSG000002	138.3046	23.836118	252.77307	10.604624	3.4066216	3.67E-06	0.0002066
ENSG000001	1496.2628	2135.1065	857.4192	0.4015815	-1.316235	0.0004412	0.0123974
ENSG000002	113.71469	43.026899	184.40248	4.2857489	2.0995473	0.0149668	0.1835139
ENSG000000	11.549256	0	23.098513	Inf	Inf	0.0352216	0.3226655
ENSG000001	845.21866	446.5061	1243.9312	2.7859221	1.4781549	3.63E-05	0.0015268
ENSG000001	330.14441	441.42975	218.85907	0.4957959	-1.012182	0.0308679	0.2964212
ENSG000001	46.865417	91.952573	1.7782619	0.0193389	-5.69235	4.81E-05	0.0019646

ENSG000001	434.91782	309.6213	560.21434	1.8093534	0.8554742	0.02373	0.2504511
ENSG000002	244.68795	80.270163	409.10573	5.0966102	2.349538	4.02E-06	0.0002236
ENSG000001	157.70134	93.92051	221.48217	2.3581875	1.2376784	0.0323435	0.3054583
ENSG000000	447.69218	558.85321	336.53115	0.6021817	-0.731729	0.0396552	0.3449939
ENSG000001	105.38072	30.314112	180.44732	5.9525847	2.5735162	0.0017746	0.0375772
ENSG000001	385.49101	207.19226	563.78976	2.7210947	1.4441872	0.000396	0.011411
ENSG000002	33.490922	0	66.981843	Inf	Inf	4.74E-05	0.0019464
ENSG000001	1329.6461	48.712683	2610.5794	53.59137	5.7439288	2.95E-26	2.22E-23
ENSG000001	172.07763	235.40315	108.75211	0.4619824	-1.11409	0.0445468	0.372902
ENSG000001	693.884	886.20893	501.55907	0.5659603	-0.821227	0.0353693	0.3235729
ENSG000001	345.68953	230.00807	461.37098	2.0058904	1.0042428	0.0140649	0.1749675
ENSG000001	63.25622	107.27008	19.242356	0.1793823	-2.47889	0.0101991	0.1394774
ENSG000001	319.08631	209.95744	428.21519	2.0395333	1.0282391	0.0188137	0.2143716
ENSG000001	2086.4394	2688.4523	1484.4265	0.5521491	-0.85687	0.0125252	0.1613255
ENSG000001	70.355571	132.14161	8.5695279	0.0648511	-3.946725	1.04E-05	0.0005304
ENSG000001	933.64048	1262.0766	605.20435	0.4795306	-1.060305	0.0081405	0.1187981
ENSG000001	577.71733	730.20603	425.22863	0.5823406	-0.780065	0.045144	0.3763531
ENSG000002	89.394785	0	178.78957	Inf	Inf	6.26E-05	0.0024785
ENSG000001	24.000346	0	48.000692	Inf	Inf	0.0007231	0.0185649
ENSG000001	722.8172	275.48931	1170.1451	4.2475154	2.0866192	2.31E-06	0.0001381
ENSG000001	32.871179	53.052214	12.690145	0.239201	-2.063704	0.0470749	0.3855611
ENSG000001	33.83846	55.107931	12.568989	0.2280795	-2.132391	0.0350372	0.3217228
ENSG000001	385.03503	541.54259	228.52748	0.4219936	-1.244707	0.0050666	0.0835793
ENSG000001	263.89435	461.98911	65.799592	0.1424267	-2.811708	0.0002277	0.007173
ENSG000001	718.28391	409.54347	1027.0243	2.5077297	1.3263819	0.0009332	0.0227094
ENSG000001	55.606908	3.7905228	107.42329	28.339968	4.8247662	0.0003648	0.0106608
ENSG000001	166.60446	1.5793845	331.62953	209.97391	7.7140662	1.70E-14	3.92E-12
ENSG000001	2877.0826	2227.5973	3526.5679	1.5831263	0.6627764	0.0272517	0.273191
ENSG000002	1510.2137	926.49801	2093.9293	2.2600473	1.1763529	0.0023264	0.0465952
ENSG000000	1913.9312	2886.2532	941.60912	0.3262393	-1.615998	5.17E-06	0.0002818
ENSG000000	488.52146	249.17367	727.86925	2.9211323	1.5465277	7.40E-05	0.0028617
ENSG000001	243.10926	179.00654	307.21198	1.7162054	0.7792222	0.0258405	0.2645744
ENSG000001	68.151557	122.03859	14.264525	0.1168854	-3.096834	0.0002844	0.0086131
ENSG000001	211.91092	118.72943	305.0924	2.5696442	1.3615686	0.0300321	0.2914992
ENSG000002	90.322345	0	180.64469	Inf	Inf	1.94E-11	2.95E-09
ENSG000001	455.32636	343.91157	566.74114	1.647927	0.7206523	0.0435773	0.366795
ENSG000002	51.473204	84.153501	18.792906	0.223317	-2.162835	0.0208642	0.2297953
ENSG000001	120.73454	35.042196	206.42688	5.8908089	2.5584657	4.55E-05	0.0018805
ENSG000001	49.638905	0	99.277811	Inf	Inf	5.80E-07	4.02E-05
ENSG000001	646.96025	227.807	1066.1135	4.6798979	2.2264771	6.30E-07	4.35E-05
ENSG000001	51.119742	96.783542	5.4559415	0.0563726	-4.148862	3.67E-05	0.0015413
ENSG000000	1103.692	1413.0931	794.2909	0.5620938	-0.831117	0.0347546	0.3205501
ENSG000001	448.82831	239.80529	657.85132	2.7432728	1.4558981	0.0010819	0.0254135
ENSG000001	118.6822	35.052266	202.31213	5.7717276	2.5290032	0.0020705	0.0426079
ENSG000001	878.99128	1131.0632	626.91934	0.5542744	-0.851328	0.0258722	0.2647982
ENSG000001	148.94935	58.567472	239.33123	4.0864191	2.0308372	0.0007916	0.0199089
ENSG000001	675.16794	911.13585	439.20003	0.4820357	-1.052788	0.0309327	0.2968335
ENSG000001	54.350389	23.126654	85.574123	3.700238	1.8876181	0.0343747	0.3182391
ENSG000001	231.95757	89.109681	374.80547	4.2061139	2.0724879	3.09E-05	0.0013269
ENSG000001	924.29119	536.19785	1312.3845	2.4475751	1.2913531	0.009542	0.1331694
ENSG000001	2849.2308	1265.3671	4433.0945	3.5034058	1.8087581	1.22E-07	9.70E-06

ENSG000001	8676.017	10722.324	6629.7102	0.6183091	-0.6936	0.0189362	0.2152057
ENSG000001	1807.0929	1143.7257	2470.4602	2.1600112	1.1110388	0.0030878	0.0580674
ENSG000002	10.747736	0	21.495472	Inf	Inf	0.044282	0.3713405
ENSG000002	100.74923	52.094513	149.40395	2.8679403	1.520015	0.0187657	0.2142175
ENSG000002	103.46655	55.569159	151.36395	2.7238841	1.4456653	0.0306871	0.2954196
ENSG000001	713.78906	965.52643	462.05169	0.478549	-1.063261	0.0096185	0.1340132
ENSG000001	122.2794	59.84105	184.71775	3.0868067	1.6261151	0.0451691	0.3764467
ENSG000001	304.28625	172.1076	436.46491	2.5360003	1.3425549	0.0401272	0.3468208
ENSG000001	370.00662	224.86122	515.15201	2.2909776	1.1959633	0.0007705	0.0195425
ENSG000001	116.82934	63.135099	170.52358	2.7009314	1.433457	0.0019974	0.0413867
ENSG000001	382.95291	655.03739	110.86843	0.1692551	-2.562729	7.13E-08	5.90E-06
ENSG000001	88.791929	19.900245	157.68361	7.9237023	2.9861747	0.0077643	0.1147252
ENSG000001	350.70899	195.03351	506.38447	2.5963972	1.3765111	0.0021904	0.0445451
ENSG000002	43.962933	7.8969225	80.028943	10.134194	3.3411594	0.0273176	0.2734896
ENSG000001	631.91926	840.25136	423.58716	0.5041196	-0.988162	0.0121933	0.1585609
ENSG000001	152.84684	223.64806	82.045617	0.3668514	-1.446732	0.0477438	0.3883676
ENSG000000	20.490481	39.795454	1.185508	0.02979	-5.069026	0.0333795	0.3120143
ENSG000001	1610.624	2001.892	1219.356	0.6091018	-0.715245	0.0357036	0.3255311
ENSG000001	110.23369	197.13958	23.3278	0.1183314	-3.079095	0.0048153	0.0808863
ENSG000000	120.25434	40.733015	199.77566	4.9045144	2.2941103	0.0020664	0.0425553
ENSG000002	9.1579126	18.315825	0	0	#NAME?	0.0151804	0.1855455
ENSG000000	3332.0994	4089.2068	2574.9921	0.6297045	-0.667253	0.0388242	0.3411188
ENSG000001	376.14625	218.35083	533.94166	2.4453383	1.2900341	0.0122444	0.1589189
ENSG000001	61.995872	0	123.99174	Inf	Inf	1.95E-08	1.83E-06
ENSG000001	97.919812	139.65502	56.184604	0.40231	-1.313621	0.0342541	0.3177338
ENSG000001	2707.3333	4503.5928	911.07385	0.2022993	-2.305436	5.63E-10	6.65E-08
ENSG000002	927.34883	572.21789	1282.4798	2.2412437	1.1642996	0.0375948	0.33576
ENSG000001	24.462498	0	48.924996	Inf	Inf	0.000671	0.0175607
ENSG000001	228.0408	99.932241	356.14935	3.5639084	1.8334603	0.0007854	0.019827
ENSG000002	79.530659	19.584368	139.47695	7.121851	2.8322523	0.017078	0.2016304
ENSG000002	67.283225	3.158769	131.40768	41.600915	5.3785434	2.13E-05	0.0009699
ENSG000001	688.04669	399.21017	976.88321	2.4470399	1.2910376	0.0069928	0.1067293
ENSG000001	435.96593	343.73601	528.19586	1.5366323	0.619772	0.0388128	0.3411188
ENSG000001	374.35455	2.5270152	746.18208	295.28199	8.2059496	3.22E-24	2.13E-21
ENSG000000	28.074474	51.939092	4.2098557	0.0810537	-3.624978	0.0100332	0.137891
ENSG000001	7256.2411	2165.1934	12347.289	5.7026264	2.5116265	1.60E-13	2.99E-11
ENSG000001	295.22625	138.30373	452.14877	3.2692449	1.7089575	0.0055342	0.0896083
ENSG000002	701.38709	872.56362	530.21056	0.6076469	-0.718695	0.0373812	0.3347712
ENSG000001	1740.7334	7.5810456	3473.8858	458.23307	8.8399378	8.06E-44	1.56E-40
ENSG000001	350.2728	217.55862	482.98697	2.2200314	1.1505801	0.0248339	0.2583887
ENSG000000	43.86771	81.450925	6.2844946	0.0771568	-3.696062	0.0427354	0.361508
ENSG000001	684.34063	874.59416	494.0871	0.564933	-0.823848	0.0313516	0.2993672
ENSG000001	92.007606	47.594526	136.42069	2.8663104	1.5191949	0.0201025	0.2242346
ENSG000001	3130.5908	2250.365	4010.8166	1.7822961	0.833737	0.002009	0.0415678
ENSG000001	140.39878	80.435654	200.3619	2.4909588	1.3167012	0.0304459	0.2941436
ENSG000001	201.22751	387.75082	14.704206	0.0379218	-4.720829	8.54E-14	1.72E-11
ENSG000001	37162.395	26004.109	48320.681	1.8581941	0.8939012	0.0177955	0.2076811
ENSG000001	523.69322	365.62433	681.76211	1.8646519	0.8989064	0.0200929	0.224234
ENSG000001	2753.6727	1992.2222	3515.1232	1.7644233	0.8191967	0.0218308	0.2365894
ENSG000001	211.18072	91.55395	330.80749	3.613252	1.8532979	0.0001059	0.0038703

<table>
<tr><th>Case</th><th>Control</th><th>Up_diff</th><th>Down_diff</th><th>Total_diff(pvalue<0.05&|log2FC|>0.58)</th></tr>
<tr><td>WJ1,WJ2(WJ</td><td>UCA3,UCA4(</td><td>1432</td><td>1932</td><td>3364</td></tr>
<tr><td></td><td></td><td></td><td></td><td></td></tr>
<tr><td>Note: one gene tested twice</td><td></td><td></td><td></td><td>1</td></tr>
<tr><td></td><td></td><td></td><td></td><td>3363</td></tr>
</table>

up_down	expressior	expressior	expressior	expressior	gene_symbc	descriptic	GO_id
Up	0.0445547	0.218571	0.257443	0.239588	RILPL1	Rab interε	GO:0003382
Up	3.59299	7.2075	28.6788	24.0389	AP000892.6		GO:0004252
Down	2.89903	2.70969	1.26127	0.212084	PFN2	profilin 2	GO:0003779
Down	0.924617	1.20698	0.312821	0.364954	IL32	interleuki	GO:0005125
Up	0.0092807	0.196028	0.240448	0.234043	B3GAT2	beta-1,3-g	GO:0000139
Down	0.153233	0.0791471	0.0040256	0	PKP2	plakophili	GO:0001533
Up	0.34219	0.413978	1.78422	1.84281	ZMAT3	zinc finge	GO:0003723
Up	1.04523	2.56865	2.7513	3.03057	PKMYT1	protein kinase, memb	
Up	11.5339	20.8795	47.0969	37.6511	FAM89B	family wit	GO:0001222
Down	0.0328948	0.0731028	0.0097603	0.0030902	GUCY1A3	guanylate	GO:0004383
Up	0.401651	0.481036	1.09914	1.04548	FBXO45	F-box prot	GO:0001764
Down	10.3293	11.3068	5.37721	6.20504	AMZ2	archaelysi	GO:0006508
Up	0.114342	0	0.557527	0.934938	GCNT4	glucosaminyl (N-acet	
Up	1.9954	3.97397	7.06642	7.22042	RBM15B	RNA bindir	GO:0000381
Up	0.0021494	0	0.0252973	0.0127005	TRABD2B	TraB domai	GO:0004222
Down	0.491507	0.443565	0.174184	0.185665	ME1	malic enzyme 1, NADP	
Up	0.29031	0	10.9217	10.2462	KRTAP2-3	keratin as	GO:0005829
Up	0.052123	0	2.05402	1.32353	RP11-77403.3		
Up	136.709	94.9132	637.633	514.793	PHLDA2	pleckstrir	GO:0005737
Up	0.232428	0.3031	0.438408	0.543408	CBL	Cbl proto-oncogene,	
Down	966.519	779.971	534.226	592.769	XXbac-BPG252P9.9		GO:0000226
Down	1.20236	1.01456	0.490011	0.662804	CENPH	centromere	GO:0000776
Up	0.0808566	0.042624	0.292846	0.256756	C2orf27A	chromosome	GO:0005802
Down	4.05847	5.60124	1.05756	1.0045	GAS1	growth arr	GO:0005886
Up	1.13681	0.883203	3.47982	3.26789	PWAR6	Prader Willi/Angelma	
Up	0.626303	0.600469	3.04461	2.98251	TREH	trehalase (brush-bor	
Up	0	0.0176347	0.0876394	0.0864725	CYP2E1	cytochrome P450, fam	
Down	0.607076	0.492946	0.247675	0.338231	HSDL2	hydroxyste	GO:0005739
Up	0.0272945	0.0188689	0.105191	0.109065	NTM	neurotrimi	GO:0005576
Down	0.233445	1.13392	0	0	RP11-535A19.1		
Down	0.601063	0.818077	0.165493	0.236413	PIGA	phosphatidylinositol	
Up	10.1026	19.3811	23.5193	22.6	CTSD	cathepsin D	
Up	0.0309029	0	1.67568	2.81308	RP11-1102P22.3		GO:0003677
Up	0.0105074	0.0225304	0.0504938	0.0454433	ZFYVE28	zinc finge	GO:0005829
Up	0.0147787	0	0.0631566	0.0633314	PRDM10	PR domain	GO:0000981
Up	0.48342	1.96037	2.32657	2.63481	VPS18	vacuolar p	GO:0003779
Up	0.251843	0.240123	0.516522	0.532642	SH3RF1	SH3 domair	GO:0001764
Up	0.0134654	0.0163516	0.0855077	0.112898	LAMA1	laminin, a	GO:0002011
Down	6.43195	4.19179	1.81195	2.13449	TAF9B	TAF9B RNA	GO:0000122
Up	8.18112	5.55562	14.9897	15.9837	MPLKIP	M-phase sp	GO:0005634
Up	0.0862124	0.0516744	0.213251	0.187857	RMI2	RecQ media	GO:0003677
Up	0	0	0.0573437	0.0908814	GALNT16	polypeptide N-acetyl	
Up	0.0536066	0.0205259	0.210107	0.231148	ZNF850	zinc finge	GO:0003677
Up	0.337788	0.357344	0.593787	0.654628	HOOK3	hook micro	GO:0000242
Down	3.49487	2.47429	1.62773	1.5931	CHORDC1	cysteine a	GO:0005524
Up	0.105032	0.070718	0.255063	0.234667	N4BP1	NEDD4 binc	GO:0005730
Up	0	0	0.0108993	0.0175446	CTD-2066L21.3		
Down	0.0862524	0.0513335	0.0054632	0.011699	RGS20	regulator	GO:0005096
Up	0	0	0.0148043	0.0180579	PDE1C	phosphodie	GO:0004117
Down	1.74475	1.29184	0.960092	0.872796	FARSB	phenylalar	GO:0000287

Down	0.136296	0.156431	0.035535	0.0077148	ABCA1	ATP-binding cassette	
Up	0.124507	0	72.6824	64.9805	ANKRD1	ankyrin re	GO:0000122
Down	1.42748	1.47611	0.749631	0.752486	MUT	methylmalonyl CoA mu	
Up	0.794342	2.69606	2.62876	2.96215	BCAR1	breast cancer anti-e	
Up	0.0183403	0	0.797904	0.923423	SIM2	single-mir	GO:0000122
Up	1.83013	2.76564	4.19807	7.88303	C14orf169	chromosome	GO:0005506
Down	0.370954	0.387021	0.179679	0.149242	CREM	cAMP respo	GO:0000122
Up	0.15824	0.709913	2.59686	3.37264	CSPG4	chondroiti	GO:0000187
Down	14.188	9.93382	0.0296052	0.0749862	B3GNT4	UDP-GlcNAc	GO:0000139
Down	1.56701	1.09084	0.631879	0.511266	RPA1	replication protein	
Up	0.750721	0.701955	4.97954	4.66136	NR2F1	nuclear re	GO:0000122
Down	2.02445	1.25002	0.84066	0.84545	PTGS2	prostaglandin-endope	
Up	0.109105	0.107856	0.2601	0.308335	CDK5RAP2	CDK5 regul	GO:0000086
Up	0.403758	0	50.8423	34.4603	NPPB	natriuretic peptide	
Down	158.649	166.27	86.7453	65.7257	CTD-2192J16.15		GO:0000027
Up	0.0634243	0.113151	0.22735	0.239156	MARK2	MAP/microt	GO:0000226
Down	5.64668	7.0602	2.83892	2.73066	TMEM30A	transmembr	GO:0005783
Up	0	0	0.987542	0.461782	RP11-253E3.3		GO:0016021
Up	0.176582	0.545823	0.710761	0.784483	DDR1	discoidin	GO:0004714
Down	1.9526	2.72864	0.86981	1.07844	SCPEP1	serine car	GO:0004185
Up	1.05561	1.27486	4.25533	3.43245	TOR4A	torsin fam	GO:0002576
Up	114.856	165.838	360.76	306.959	CH507-513H4.3		GO:0005524
Up	2.32964	3.46641	5.03026	4.15486	FCGRT	Fc fragmen	GO:0002416
Up	0.0095845	0	0.119352	0.0929869	ZFHX2	zinc finge	GO:0000122
Down	0.533764	0.223908	0.0226478	0.0523159	ANXA3	annexin A3	GO:0005509
Down	0.0697386	0.196472	0.0060303	0.0168015	HS3ST3A1	heparan su	GO:0000139
Up	0.156259	0.124281	0.349152	0.435174	NPIPB3	nuclear po	GO:0005654
Down	0.0058963	0.0278449	0	0	FGF10	fibroblast growth fa	
Up	0	0	0.113816	0.12355	RASD2	RASD famil	GO:0001963
Down	0.0044218	0.0112761	0	0	GNG7	guanine nu	GO:0001662
Down	0.747449	0.70834	0.358234	0.261925	ARNT	aryl hydrocarbon rec	
Up	0.340679	0.361229	0.702501	0.652369	NOTCH2	notch 2	
Down	2.46847	2.04001	0	0	HAND1	heart and	GO:0000122
Down	1.05373	0.717491	0.242064	0.309168	RP11-705C15.2		GO:0005886
Down	0.615547	0.823204	0.226673	0.320447	FXYD6	FXYD domai	GO:0005215
Down	0.393327	0.366906	0.0275554	0.167506	PAQR3	progestin	GO:0004674
Up	0.891502	1.11664	1.83831	2.10142	DPYSL3	dihydropyr	GO:0005615
Up	0.386552	0.442818	0.955866	0.885481	ANTXR2	anthrax to	GO:0004888
Up	0.0152087	0.0451453	0.0963498	0.0683548	APBA2	amyloid be	GO:0001540
Down	0.398751	0.392989	0	0	KCNJ2-AS1	KCNJ2 antisense RNA	
Up	0	0.024643	0.062514	0.0733364	TNFAIP8L3	tumor necr	GO:0005654
Down	0.34679	0.584593	0.11348	0.130395	LLGL1	lethal gia	GO:0000137
Up	0.154401	0.134611	0.816257	0.854967	ULK1	unc-51 lik	GO:0000045
Down	0.0624498	0.0579359	0.0250077	0.0116847	PBX1	pre-B-cell	GO:0000978
Down	9.88088	18.1576	4.34046	3.48698	INSIG1	insulin induced gene	
Up	0.0212136	0.0297307	0.063857	0.0521289	SORBS2	sorbin and SH3 domai	
Down	3.71065	5.6671	0.672018	1.51931	CFB	complement	GO:0004252
Down	0.812833	0.610525	0.424487	0.343439	TNPO3	transporti	GO:0005634
Up	8.41637	21.7758	24.2712	25.5217	MARVELD1	MARVEL dom	GO:0005634
Down	3.44891	3.10233	1.60404	1.45182	GLOD4	glyoxalase	GO:0005739
Up	0.0858413	0	0.814027	0.536294	MAFK	v-maf avia	GO:0000122

Up	0.857809	1.12646	2.69177	2.45415	RNASET2	ribonuclease T2	
Down	0.0219438	0.025122	0	0.0003641	LSAMP	limbic sys	GO:0005829
Up	0.682228	2.5042	2.29253	3.17201	AKAP17A	A kinase (	GO:0003723
Up	0.331713	0.949387	1.20419	1.12371	MAP7D1	MAP7 domai	GO:0000226
Down	0.275069	0.112143	0.0698731	0	GPR68	G protein-coupled re	
Up	1.52826	2.34068	4.50171	4.65325	IL18BP	interleuki	GO:0005576
Up	1.00077	1.1953	1.92979	2.15441	STAT1	signal transducer an	
Up	0.574001	0.354852	1.41696	1.47003	ZNF598	zinc finge	GO:0003723
Up	0.0960331	0.106518	0.268921	0.163736	ZNF532	zinc finge	GO:0000981
Down	1.99539	3.75949	0.151365	0.0816248	STRA6	stimulated	GO:0001523
Up	0.134342	0	1.35527	1.23211	RP11-172H24.4		GO:0016021
Up	0.240878	0.0466679	0.955398	0.773316	ZNF528	zinc finge	GO:0000981
Down	106.681	120.931	33.5355	38.3772	CTHRC1	collagen triple heli	
Up	9.84284	9.80865	24.0239	28.2723	C9orf16	chromosome 9 open re	
Down	103.083	110.446	55.3579	61.0331	RAN	RAN, membe	GO:0000055
Down	6.263	6.12355	3.85394	3.57063	ABCF2	ATP-binding cassette	
Down	2.42928	1.31288	0.559256	0.308675	RAC2	ras-related C3 botul	
Up	0.343946	0.981996	2.94007	1.77365	TNFAIP3	tumor necrosis facto	
Up	2.03455	2.59092	36.0629	33.8866	HSPB2	heat shock 27kDa pro	
Up	0.070842	0.0670198	0.178372	0.176716	ZFHX3	zinc finger homeobox	
Up	0.272326	0.68277	3.0907	3.24267	ECE1	endothelin convertin	
Down	1.23509	1.77193	0.15772	0.187258	PABPC4L	poly(A) bi	GO:0003723
Up	1.90311	3.4151	4.49256	4.71156	PXDN	peroxidasi	GO:0004601
Up	0.643129	0.165662	3.56658	2.31094	FOXL1	forkhead b	GO:0000981
Up	11.5894	11.7279	42.6881	47.062	ECM1	extracellular matrix	
Up	0	0	0.23896	0.15624	NRN1	neuritin 1	GO:0005576
Down	0.946879	0.946216	0.109276	0.0668328	AKR1C1	aldo-keto	GO:0001523
Up	0.171021	0.208742	1.002	0.920213	MMP11	matrix metallopeptic	
Up	0.122618	0.259762	0.427024	0.27836	MAP3K3	mitogen-ac	GO:0000165
Down	0.0961769	0.134179	0.0169108	0.0242942	CCDC85A	coiled-coil domain c	
Down	0.1787	1.23771	0	0	SFRP2	secreted f	GO:0001569
Up	1.68726	6.33243	7.80617	9.68274	P3H3	prolyl 3-h	GO:0005506
Up	0.0955013	0.541197	0.654837	0.966574	MUC1	mucin 1, cell surfac	
Up	0	0	0.123625	0.135869	EPB41L4B	erythrocyt	GO:0005200
Up	0	0	0.0690989	0.102868	MYB	v-myb avian myelobla	
Down	0.129079	0.219994	0.0293724	0.0278987	RASSF2	Ras association (Ral	
Up	0	0	0.0999262	0.140471	RP11-890B15.2		GO:0005829
Up	0	0	0.234015	0.0732195	FPR3	formyl pep	GO:0002430
Up	0	0	0.192422	0.0559725	NBPF25P	neuroblast	GO:0005737
Down	1.52452	1.05215	0.723851	0.86828	RPA3	replication protein	
Up	0.131045	0.251876	0.387078	0.311321	XXYLT1	xyloside x	GO:0000287
Up	0.834417	1.11675	1.88505	1.58718	GTPBP2	GTP bindir	GO:0002576
Up	0.482081	0.410223	1.3246	1.62636	STRN4	striatin,	GO:0005516
Up	0.284268	0.687326	1.20446	1.03144	KLF16	Kruppel-li	GO:0000978
Up	0.173778	0.236847	0.515947	0.418396	ADNP2	ADNP homec	GO:0000981
Up	0.049241	0.0750119	0.219667	0.13749	SLC9A7	solute car	GO:0000139
Up	0.165822	0.303903	1.42637	1.40062	NLRP1	NLR family	GO:0005524
Up	0.0893131	0.139007	0.251823	0.200993	SUSD1	sushi doma	GO:0005509
Up	0.175299	0.458494	0.538472	1.09828	CCDC42B	coiled-coi	GO:0003341
Down	217.545	221.302	127.601	127.834	ENO1	enolase 1, (alpha)	
Up	0.0136039	0.145619	0.51846	0.469273	DGKQ	diacylglyc	GO:0003951

Up	0	0	0.447939	0.312667	LTK	leukocyte	GO:0004672
Up	0	0	0.49408	0.599649	HOXC13	homeobox C13	
Up	0.340312	0.566884	0.672777	1.01962	MORC2	MORC famil	GO:0005634
Down	0.803752	1.08274	0.231572	0.22901	CLMP	CXADR-like	GO:0005881
Down	0.0061314	0.0053456	0	0	EPHA7	EPH receptor A7	
Up	3.81682	5.97128	8.3371	8.78997	SRRM2	serine/arg	GO:0000398
Down	63.1819	46.7076	2.96097	5.05085	IFITM1	interferon	GO:0001503
Up	0.0556164	0.0484882	0.30751	0.746088	RP11-231I16.1		GO:0000209
Up	1.99616	2.71259	5.58667	6.42132	IRF2BPL	interferon	GO:0000122
Down	1.69798	1.77387	0.299598	0.312348	GLT8D2	glycosyltr	GO:0005794
Down	8.97266	6.21921	3.78583	4.60725	DCAF13	DDB1 and C	GO:0000462
Up	0.272676	0.817256	0.941392	0.99809	PIEZO1	piezo-type	GO:0005261
Up	4.71544	11.7608	11.7811	13.6692	PLOD3	procollage	GO:0001701
Up	1.36807	2.1441	2.61644	3.95288	ILF3	interleuki	GO:0003677
Up	0.37156	0.399747	0.745978	0.916179	CAMKK2	calcium/ca	GO:0000165
Up	0.0090957	0.0281153	0.0792471	0.0515012	BCL11A	B-cell CLL/lymphoma	
Down	42.6522	35.3716	6.47971	5.34658	MT1L	metallothionein 1L	
Up	0.0509381	0.0803492	0.190717	0.196243	SMAD6	SMAD famil	GO:0000978
Up	0	0.120674	0.237201	0.187041	RP11-33504.3		
Down	17.2453	10.7701	5.36708	5.78712	TWF1	twinfilin actin binc	
Up	0.972145	1.26249	4.34174	3.81145	UBE2Q1	ubiquitin-	GO:0001967
Up	0.0175747	0.0715034	0.160249	0.137096	ATP10A	ATPase, cl	GO:0000287
Up	0.0185443	0.0400339	0.0619885	0.0685386	PPFIBP2	PTPRF inte	GO:0005615
Up	0.122954	0.161206	0.276217	0.244111	TJP2	tight junction prote	
Up	0.0098981	0	0.0520073	0.111046	NPR3	natriureti	GO:0001501
Up	0	0	0.0458582	0.0669538	SHROOM2	shroom fam	GO:0002089
Down	57.3781	54.0306	32.3738	34.2026	IFITM2	interferon	GO:0005886
Up	0.89947	1.95306	3.63859	3.8977	CSNK1E	casein kin	GO:0004672
Up	0.134937	0.105008	0.465541	0.990218	RHOJ	ras homolo	GO:0003924
Up	0.0354046	0.0433804	0.175266	0.186128	TCF7L1	transcript	GO:0000981
Up	1.27997	1.31881	3.3131	3.1102	LINC00960	long inter	GO:0003964
Down	8.85525	6.27617	3.19572	3.2277	JSRP1	junctional	GO:0003009
Down	2.04545	1.96835	0.486673	0.480746	HIST2H2BE	histone cl	GO:0000786
Up	0	0	0.0308509	0.0249075	MICALCL	MICAL C-te	GO:0005737
Down	4.77853	5.92574	2.41209	2.6401	YARS	tyrosyl-tR	GO:0000049
Down	1.04361	0.844533	0.413135	0.521635	CUL1	cullin 1	GO:0006513
Up	0.0031462	0.0066423	0.0448092	0.0370044	ADGRL2	adhesion G protein-c	
Up	0.0489914	0.197426	0.299288	0.20238	SMURF1	SMAD speci	GO:0000209
Up	0.214778	0.343923	0.823954	0.731447	PHC2	polyhomeot	GO:0000792
Down	0.414515	0.319727	0.0595314	0.0805415	RELB	v-rel avian reticulo	
Down	21.5169	20.7628	9.58125	9.39753	COTL1	coactosin-	GO:0003779
Down	0.515163	0.517413	0.0243379	0.0213386	PRRX2	paired related homec	
Up	0.037644	0	0.187507	0.114825	CPEB2	cytoplasmi	GO:0000900
Up	121.285	84.498	456.281	488.839	PTX3	pentraxin	GO:0001550
Down	6.09637	4.48875	2.40076	2.02665	CD200	CD200 molecule	
Up	0.0933491	0	1.91484	2.16762	HEY2	hes-relate	GO:0000122
Up	0.987506	0.740662	3.33904	3.30255	IPO4	importin 4	GO:0000790
Up	0.177902	0.600603	1.21716	1.54871	TRIM46	tripartite	GO:0001578
Up	0	0.0909544	0.412888	0.75051	INA	internexin	GO:0005200
Down	0.0154945	0.0071765	0.0027242	0.0011421	CADPS2	Ca++-deper	GO:0005654
Up	0.382344	0.519792	3.3032	4.23586	HLA-B	major hist	GO:0000139

Down	3.49581	3.66659	1.52302	1.48519	PROCR	protein C receptor,
Up	0.664437	1.57192	1.61881	2.04908	RUNDC1	RUN domair GO:0005096
Up	0	0	0.186521	0.22168	DKFZP434K(	uncharacterized LOC2
Up	0.0639415	0	0.228458	0.342816	SDHAP2	succinate dehydroger
Down	3.3662	2.24224	1.46677	1.45939	GNPDA1	glucosamir GO:0004342
Down	4.04575	3.06324	2.10994	2.27874	BNIP2	BCL2/adenovirus E1B
Up	0.364221	0.497919	0.784953	0.72246	MAP4K5	mitogen-ac GO:0004672
Down	1.18612	0.973763	0.612234	0.713217	SNX7	sorting ne GO:0015031
Down	8.6072	25.9975	0.330576	0.334923	FIBIN	fin bud ir GO:0005576
Down	0.374384	0.28288	0.20533	0.154884	ERCC8	excision r GO:0003723
Up	0.178142	0.664802	0.9424	0.895117	TNFRSF14	tumor necrosis facto
Up	0.0369755	0.754332	1.28691	1.11251	IL21R-AS1	IL21R antisense RNA
Down	3.35633	3.84412	0.792929	1.48199	CYBRD1	cytochrome GO:0000293
Up	0.0838613	0.0528039	0.293754	0.417854	PIGZ	phosphatic GO:0000026
Down	0.0214838	0.0695696	0	0	N4BP2L1	NEDD4 binding protei
Up	0.0005822	0	0.0051394	0.0085357	CACNA1C	calcium ch GO:0005245
Up	0.0009339	0.0026463	0.0338593	0.0362435	DCLK1	doublecort GO:0001764
Up	0.409555	2.08489	3.15129	3.3058	RHBDF1	rhomboid 5 GO:0000139
Down	2.23301	1.86149	1.24268	1.17911	CTBS	chitobiase GO:0004568
Down	1.87254	5.40085	0.11849	0.051449	H19	H19, imprinted mater
Up	0.305289	0.689598	2.14026	1.88201	KCTD15	potassium GO:0007275
Up	0.0903082	0.162268	3.297	3.01391	OXTR	oxytocin receptor
Up	0.0213974	0.0485029	4.05641	4.05894	LAMA5	laminin, a GO:0001525
Down	1.23242	0.854461	0.58362	0.58494	GTF2E1	general tr GO:0000993
Down	443.923	405.582	218.728	252.454	HSPA5	heat shock 70kDa pro
Down	0.9757	3.12521	0	0	HCG4	HLA comple GO:0002474
Up	0.0230187	0	0.181803	0.0830029	MLPH	melanophilin
Down	1.21633	1.10712	0.0552034	0.0644804	PARM1	prostate a GO:0000139
Up	0.0275268	0.0738909	0.184522	0.163117	ADGRL1	adhesion (GO:0004930
Up	1.87346	2.48252	6.31759	4.53033	CDR2L	cerebellar degenerat
Down	0.131211	0.193973	0.0441914	0.0523213	GALC	galactosyl GO:0004336
Down	0.746161	0.778997	0.427289	0.379604	ZNF451	zinc finge GO:0000981
Up	0.0886834	0.0761456	0.806548	0.811145	HLA-F	major hist GO:0000139
Down	28.6992	19.1086	7.74836	8.40071	RP11-4204.2	
Up	0.119154	0.611932	1.45561	1.59844	TRIM7	tripartite GO:0005634
Down	0.0188445	0.0084896	0.0008949	0.0012088	SEMA3A	sema domai GO:0001755
Up	0.674704	1.57781	1.81496	1.68402	LONP1	lon peptic GO:0000002
Up	1.69392	1.93232	5.54132	5.46486	ITPRIPL2	inositol 1 GO:0016021
Up	0.0310703	0.0236372	0.0634403	0.0714523	CACNA2D1	calcium ch GO:0005245
Up	0	0	0.0618794	0.080728	PTPRZ1	protein ty GO:0002244
Up	0	0.037918	0.708766	0.901881	ITGA10	integrin, GO:0005518
Up	0.0006682	0.0083302	0.0202219	0.0215121	TSPAN8	tetraspanin 8
Down	0.028597	0.0170586	0.000949	0	NDST3	N-deacetyl GO:0000139
Down	2.51775	2.5171	1.36111	1.15995	IFI16	interferon GO:0000122
Up	0.0772885	0.115027	0.242181	0.308951	BRD1	bromodomai GO:0005634
Down	16.0284	12.7526	7.59638	7.96455	RPUSD3	RNA pseudo GO:0001522
Down	6.08932	5.41392	2.34977	1.15872	S1PR2	sphingosir GO:0000187
Up	0	0	0.0438521	0.0284621	HTR7	5-hydroxyt GO:0004930
Down	2.41602	1.35782	0.47001	0.561223	MSX2	msh homeob GO:0000122
Up	0.398539	0.630567	0.995076	0.879308	CLSTN1	calsynteni GO:0000139
Up	0.170744	0.158858	0.668435	1.2234	EHBP1L1	EH domain GO:0005768

Down	0.134345	0.130441	0.0545844	0.0559032	RGL1	ral guanir	GO:0005829
Up	2.91047	5.0721	9.92549	9.24008	GNAI2	guanine nu	GO:0003924
Down	0.257728	0.347743	0.120371	0.0731723	CTD-2154I11.2		GO:0005886
Up	0.148812	0.421651	2.76792	2.56666	RP1-95L4.4		GO:0004252
Down	0.676533	0.83722	0.118143	0.201124	FAM91A1	family wit	GO:0005802
Up	0.0800994	0.0621803	0.660009	0.61822	TBX18	T-box 18	
Up	0.0668542	0.137766	3.39519	2.74876	SPRY1	sprouty RT	GO:0000132
Up	0	0	2.33823	1.98984	DDX3Y	DEAD (Asp-	GO:0003677
Up	0.0190983	0.0626586	1.53627	3.77368	ELN	elastin	GO:0003677
Up	0.229263	0.374506	0.91259	0.950649	WDR26	WD repeat	GO:0005634
Up	0.0844636	0.045828	0.261179	0.236774	TRAPPC10	traffickir	GO:0000139
Up	0.0130501	0.091805	0.160026	0.153938	CUEDC1	CUE domain containir	
Up	0.000724	0	0.038573	0.0539376	CNTNAP3P2	contactin	GO:0007155
Up	3.51546	6.65606	9.3513	8.21938	ERVK3-1	endogenous	GO:0003677
Down	0.0482826	0.0547226	0.0034792	0	NAALAD2	N-acetylat	GO:0004180
Up	1.90371	0.603066	8.419	11.8074	TNFRSF11B	tumor necrosis facto	
Up	0	0	0.0332595	0.019725	OCLN	occludin	
Down	0.208807	0.274177	0.111586	0.0829602	TMEM65	transmembr	GO:0003231
Up	0.0011003	0	0.0291377	0.0497637	LINC01592	long intergenic non-	
Down	0.436525	0.83344	0.17686	0.172377	TANK	TRAF famil	GO:0005829
Up	0.551278	0.488943	0.990772	1.38278	BRD7	bromodomai	GO:0002039
Up	0.663154	1.61738	2.04301	1.71327	STX4	syntaxin 4	GO:0000149
Up	0.109044	0.0862245	0.356027	0.363273	WDR60	WD repeat	GO:0000242
Down	0.727952	0.453936	0.226834	0.195254	RNF6	ring finge	GO:0003677
Down	0.0047515	0.0050487	0	0.0001423	GABRA3	gamma-aminobutyric a	
Up	0.338453	0.775761	1.82369	1.84687	HDAC7	histone de	GO:0000118
Down	1.44982	1.69701	0.276312	0.118977	RHOU	ras homolo	GO:0000082
Up	0	0	0.524097	0.761084	NPPC	natriureti	GO:0001503
Up	0.0280099	0.0945625	0.125253	0.165224	FYN	FYN proto-	GO:0001664
Up	3.30961	5.11028	34.1618	38.1267	DBNDD2	dysbindin	GO:0005737
Down	0.790698	0.923708	0.312285	0.338474	UXS1	UDP-glucur	GO:0005737
Up	0.114695	0.460394	0.89191	0.522034	MC1R	melanocortin 1 recep	
Up	0.0472134	0.05139	0.166814	0.115032	STK39	serine thr	GO:0000187
Down	1.52251	1.38025	0.220418	0.23979	GBP2	guanylate	GO:0000139
Down	1.30632	1.65417	0.502939	0.440319	LACC1	laccase (m	GO:0005507
Down	175.67	127.111	59.7619	62.1301	ANXA1	annexin A1	
Up	4.68718	10.1645	14.5953	14.9007	RP11-2C24.3		GO:0000151
Down	3.19335	2.45983	1.55731	1.94392	HDAC1	histone de	GO:0000976
Down	0.322945	0.287922	0.162977	0.132968	TMEM2	transmembr	GO:0001525
Down	0.374052	0.302817	0.161241	0.124715	RHOT1	ras homolo	GO:0003924
Up	0	0	0.0549357	0.0647745	CERS1	ceramide s	GO:0005125
Down	6.45792	5.16853	2.0962	2.41742	TOB1	transducer	GO:0003714
Down	1.28742	1.24453	0.408308	0.34533	SQRDL	sulfide qu	GO:0005743
Up	0	0	12.2723	13.5489	DSP	desmoplakin	
Up	0.0638328	0.0914976	0.157675	0.185834	ABR	active BCR	GO:0002692
Up	0.0791431	0.125533	0.220935	0.286554	ARHGAP22	Rho GTPase	GO:0001525
Up	0	0	0.144033	0.112485	ARHGEF16	Rho guanir	GO:0005085
Down	2.86669	2.14813	0.866398	0.544835	AC009506.1		
Down	0.453108	0.429891	0.0129272	0.006876	LPAR1	lysophosphatidic aci	
Up	32.162	53.3553	71.514	68.5345	ZYX	zyxin	GO:0001725
Up	0	0	25.0106	21.0604	RP3-512B11.3		

Up	0.851332	0.940091	1.46436	2.10314	EMP1	epithelialGO:0016021
Up	0.127138	0	6.60537	8.11351	CST6	cystatin E/M
Down	8.77511	5.98657	2.19352	4.7469	SDCBP	syndecan binding pro
Up	0.650984	0.500213	1.15261	1.24375	SMC5	structuralGO:0000722
Up	0.764159	0.851886	1.57656	1.99261	POGK	pogo transGO:0003677
Down	0.0265933	0.0376754	0.0010818	0.000822	JAKMIP2	janus kinaGO:0005794
Down	0.0657829	0.0713846	0.0038241	0.0069292	SORL1	sortilin-rGO:0000139
Up	0.0174397	0.035938	0.126954	0.0960875	LGR4	leucine-riGO:0001649
Down	1.23585	1.04011	0	0	IRX3	iroquois hGO:0000981
Up	0.0141698	0.281046	0.458625	0.323599	RBM38	RNA bindinGO:0003723
Up	0.586659	0.772854	1.45318	1.48789	AKAP9	A kinase (PRKA) anch
Down	4.76489	5.35856	0.512626	0.525781	GFPT2	glutamine-GO:0004360
Down	0.548658	0.385845	0.25159	0.270829	NR2C1	nuclear reGO:0000978
Up	0.0193472	0.0219278	0.123614	0.16314	RP11-736K20.5	
Up	0.229013	0.749839	0.972267	1.48733	SELO	selenoprotGO:0005739
Down	0.515916	0.300692	0.0833819	0.107944	POLI	polymerase (DNA dire
Down	1.79751	1.48121	0.747848	0.808562	ERP44	endoplasmiGO:0003756
Up	0.522472	0.284237	1.07525	1.74221	RP11-221N13.3	
Up	0.253017	0.311834	0.618395	0.569663	SMAP2	small ArfGGO:0005096
Up	1.45975	1.73906	3.86461	3.31804	SRF	serum respGO:0000790
Down	1.22769	1.11223	0.670807	0.421525	VIPAS39	VPS33B intGO:0005737
Up	0.224453	0.244534	0.584642	0.490403	DENND5A	DENN/MADD GO:0000139
Down	3.53058	3.33932	1.72332	1.99426	COX11	COX11 cytcGO:0004129
Up	0.307291	0.808797	3.37733	2.74213	SYNPO	synaptopocGO:0001725
Down	1.17863	0.940833	0.565748	0.346098	SLC33A1	solute carrier famil
Down	41.2792	27.281	20.424	20.6011	SRSF3	serine/arginine-rich
Up	0.584183	1.03135	1.15237	1.28747	HK1	hexokinase 1
Up	1.84326	2.64636	3.76216	3.66248	HN1L	hematologiGO:0005634
Up	0.527563	0	1.3444	2.15897	RGMB-AS1	RGMB antisense RNA 1
Up	0.0011786	0.0066787	0.0217974	0.0195744	PPM1L	protein phGO:0000165
Down	0.403273	0.197293	0.0926834	0.0416999	ERAP2	endoplasmiGO:0002250
Down	6.07978	7.2358	2.85042	3.06254	TIMMDC1	translocasGO:0005634
Down	1.37557	1.15485	0.107076	0.130181	GLUD2	glutamate GO:0004352
Down	1.28925	0.841503	0.467412	0.622096	CENPA	centromereGO:0000132
Down	0.0158248	0.0089678	0.0007982	0	CCDC33	coiled-coil domain c
Up	0.415919	1.57815	1.88359	1.83327	ZACN	zinc activGO:0004888
Down	3.40047	2.17994	1.20029	0.815178	TMEM99	transmembrGO:0016021
Down	0.256455	0.431589	0.0783994	0.0506368	LINC01503	long interGO:0005654
Down	0.199924	0.111579	0.0515679	0.0362819	MTURN	maturin, rGO:0007275
Down	1.37566	1.11317	0.704767	0.833039	SLC25A24	solute carGO:0005347
Down	0.23538	0.153068	0.0820253	0.0533484	PTER	phosphotriGO:0008270
Up	0.0187653	0.0070894	0.0607364	0.0677284	GAB2	GRB2-associated binc
Up	0.699525	0.708675	1.58191	1.35743	PCNXL3	pecanex-liGO:0016021
Down	0.119535	0.0893633	0.0697222	0.0389882	CRADD	CASP2 and GO:0002020
Down	0.437945	0.284372	0.172581	0.167201	AC093616.4	GO:0005654
Up	0.0083306	0.0111811	0.0713245	0.061339	ZHX2	zinc fingeGO:0000122
Up	0.493537	0.972811	3.51771	2.88544	GNA11	guanine nucleotide b
Down	4.7877	4.26351	2.92767	1.92971	MYCBP	MYC bindinGO:0003713
Up	0.418101	0.511778	1.17047	0.980736	TNKS1BP1	tankyrase GO:0000289
Up	0.068797	0	0.366299	0.428492	TBC1D3L	TBC1 domaiGO:0005096
Down	0.0889044	0.177016	0	0	C3	complement component

Up	0.950525	4.54217	5.26754	7.47841	LRWD1	leucine-ri	GO:000077(
Down	1.58851	1.18764	0.390672	0.128056	NUDT12	nudix (nu	GO:000021(
Down	1.38789	3.50836	0.223161	0.242959	LMCD1	LIM and cy	GO:000012
Up	0.0878235	0.226222	0.70476	0.807105	RELT	RELT tumor	GO:000563
Down	0.79613	0.219483	0.0217069	0	C4orf26	chromosome	GO:000557
Down	3.17296	1.90526	0.51406	0.414776	OGFRL1	opioid gro	GO:000498
Up	1.1748	4.30657	4.11568	6.07176	TMEM259	transmembr	GO:000578
Down	3.94454	3.60538	2.13245	2.02325	NDUFB5	NADH dehyc	GO:000396
Down	175.065	163.049	98.6936	111.504	CALM2	calmodulin	GO:000550
Down	8.08612	7.01488	3.39574	4.93613	TRA2B	transforme	GO:000037
Down	2.86084	1.75399	0.441374	0.454569	ARRDC3	arrestin c	GO:000165
Down	0.281231	0.132809	0.0073883	0.0336846	AADAC	arylacetam	GO:000382
Down	3.87616	3.01301	1.91325	2.10233	PMPCB	peptidase	GO:000422
Up	2.59344	2.74662	5.83312	6.55077	FAM213B	family wit	GO:000151
Up	0.144506	0.197666	1.48294	1.34647	GGT5	gamma-glut	GO:000004
Down	5.6827	5.18692	3.11249	3.16546	YWHAE	tyrosine 3	GO:000016
Down	0.627387	0.660607	0.162314	0.193925	BBS4	Bardet-Biedl syndrom	
Up	0.480783	1.54391	1.834	2.29193	KLC2	kinesin li	GO:000377
Down	3.85667	2.40547	1.64406	1.39452	CRYZ	crystallin	GO:000373
Down	0.715008	0.516488	0.2849	0.291516	BPNT1	3'(2'), 5'	GO:000582
Up	0.129063	0.148883	0.422824	0.421041	WWC3	WWC family	GO:000012
Up	0.220361	0	0.779613	0.712637	SERTAD4	SERTA doma	GO:000563
Up	0.24498	0.610198	0.831229	0.837973	ANKRD11	ankyrin re	GO:000170
Up	0.215286	0.318637	0.401155	0.586831	TCHP	trichoplei	GO:000573
Up	0	0	0.332423	0.413234	HEPH	hephaestin	
Up	0.771643	1.14003	1.48949	2.28565	ALDH3A2	aldehyde c	GO:000156
Down	0.14383	0.289218	0	0	PRSS21	protease,	GO:000425
Up	0	0	0.0262297	0.0173866	AC004540.5		
Up	0.632415	2.10087	2.98648	2.58693	ARRDC1	arrestin c	GO:000588
Up	0	0.0915304	0.162942	0.260009	SH2D5	SH2 domain	GO:001406
Up	0.226806	0.37544	0.54718	0.47526	CHD8	chromodoma	GO:000012
Down	2.63344	1.61787	0.0372432	0.0282996	CXCL14	chemokine (C-X-C mot	
Down	3.76095	2.38134	0	0	GSC	goosecoid	GO:000097
Up	0.502527	1.43459	1.80104	1.48802	INPP5E	inositol p	GO:000172
Down	2.85091	1.92741	1.20473	1.2185	RFC5	replication factor (	
Up	0.879201	1.53303	2.52198	2.92545	LCAT	lecithin-cholesterol	
Up	0	0	0.0548679	0.0600855	TNFRSF10C	tumor necr	GO:000488
Up	0	0	0.219993	0.208349	ADCY4	adenylate	GO:000309
Down	2.67607	1.08322	0.482086	0.370898	DEPDC7	DEP domain	GO:000562
Up	0.363522	0.517549	1.37524	1.2835	FXR2	fragile X	GO:000372
Up	1.2465	1.44046	4.53068	3.61599	LPCAT4	lysophosph	GO:000384
Up	0.678375	1.37296	4.74131	4.07081	JUP	junction p	GO:000153
Up	18.4874	53.0116	58.6876	56.582	CDKN1A	cyclin-dependent kin	
Down	7.04183	5.72728	3.97302	3.20322	TMEM70	transmembr	GO:000565
Up	0.125185	0.205729	0.367818	0.430032	PDGFRL	platelet-d	GO:000499
Down	2.01441	2.15587	0.625227	0.705158	PCDH18	protocadhe	GO:000550
Down	82.6815	46.5726	35.0686	26.9257	ANXA2P2	annexin A2	GO:000152
Up	0	0	0.434402	1.25433	TMSB4Y	thymosin b	GO:000378
Down	0.119329	0.12581	0.0174974	0	MMP8	matrix metallopeptid	
Up	0.0024562	0.103	0.516009	0.434929	RNF152	ring finge	GO:000484
Up	0.506163	1.22931	6.79319	6.73816	CHST2	carbohydra	GO:000013

Down	0.593674	0.568737	0.201519	0.26039	MTRR	5-methyltetrahydrofo	
Down	2.57207	1.9266	1.22746	1.06843	NECAP1	NECAP endo	GO:0005829
Down	0.337522	0.26578	0.121824	0.17784	FBXO42	F-box protein 42	
Up	0.135374	0.157055	0.397877	0.47086	CAPN12	calpain 12	GO:0004198
Down	0.391447	0.253094	0.129204	0.110428	RP5-857K21.4		GO:0000281
Up	0	0.0823994	0.189471	0.434237	ZNF284	zinc finge	GO:0000981
Down	0.107415	0.0919635	0.0116938	0.0066643	EPHX2	epoxide hydrolase 2,	
Down	1.64029	1.21005	0.618087	0.692866	PRR19	proline rich 19	
Down	0.0577821	0.0491169	0	0	RP11-706O15.3		GO:0003676
Down	0.0251222	0.0235639	0.0043696	0.0036523	ABI3BP	ABI family	GO:0005201
Down	0.620965	0.540454	0.36213	0.289384	PLGRKT	plasminoge	GO:0005887
Down	2.98057	2.16615	1.70665	1.64944	PSMD1	proteasome	GO:0000502
Up	2.50845	5.33844	6.64415	6.80149	NAPRT	nicotinate	GO:0004514
Down	0.529357	0.445765	0.016701	0.0088449	MOXD1	monooxygen	GO:0004500
Up	0.0698882	0.135237	0.754488	0.568406	GLIS2	GLIS famil	GO:0000122
Up	0.441087	0.252061	1.61024	1.53256	ADCY6	adenylate	GO:0003091
Up	0.374003	0.352377	0.810072	0.841168	SPEN	spen famil	GO:0000122
Up	5.41217	16.5879	26.396	28.3841	B3GALT6	UDP-Gal:betaGal beta	
Down	1.98451	2.04668	0.830223	1.14815	CHKB	choline ki	GO:0004103
Down	0.582051	0.403215	0.304478	0.186654	TRIM23	tripartite	GO:0000139
Up	0.0046929	0.0032833	0.0176076	0.0229281	NBEA	neurobeach	GO:0005802
Down	0.53996	0.835688	0	0.0054348	FAIM2	Fas apopto	GO:0002931
Down	6.74828	17.035	1.2034	1.72433	CRABP2	cellular r	GO:0001972
Up	0	0	0.0433763	0.0432599	AGAP11	ankyrin re	GO:0005096
Up	0.014313	0	0.393894	0.363845	KIAA1755	KIAA1755	
Down	42.1747	23.9	16.5687	13.2116	SRP9P1	signal recognition p	
Up	4.60433	5.54259	10.9416	10.309	TMEM173	transmembr	GO:0002218
Up	0.0570773	0.141737	0.551547	0.718676	KIAA1549L	KIAA1549-l	GO:0016021
Down	0.327981	0.29347	0.0380942	0.0248111	SENP8	SUMO/sentr	GO:0005829
Down	4.10041	3.24702	2.40807	2.02635	ADSL	adenylosuccinate lya	
Up	0.0848095	0	0.368968	0.796398	SCN1B	sodium cha	GO:0001518
Down	1.2126	0.842119	0.628139	0.661839	SEPT10	septin 10	GO:0000281
Up	0	0	0.2029	0.131692	RP11-87C12.5		
Down	28.3836	22.6403	7.44425	7.08503	RP11-489E7.4		GO:0005783
Up	0.746909	1.45198	2.00872	1.80892	CNOT11	CCR4-NOT t	GO:0000289
Up	0.219735	0.787208	1.3345	1.4182	KANK2	KN motif a	GO:0000122
Down	1.26842	0.872859	0.535259	0.447531	PNPT1	polyribonu	GO:0000175
Down	0.0309418	0.0232074	0	0	LINC00856	long intergenic non-	
Up	0.19679	0.564004	0.547658	0.734754	XRCC1	X-ray repair complem	
Up	4.03628	8.08758	14.4054	11.9341	NPDC1	neural pro	GO:0005886
Down	7.92763	15.5061	0.189068	0.538745	CXCL2	chemokine (C-X-C mot	
Up	0	0	0.355488	0.107191	SYNPO2L	synaptopoc	GO:0003779
Down	3.94469	6.02257	0.119658	0.0389673	SERPINF1	serpin pep	GO:0001822
Up	0.0236826	0.0256214	0.0716746	0.103562	FOXO1	forkhead box O1	
Up	0	0	5.78907	4.68517	EIF1AY	eukaryotic	GO:0003743
Down	0.308601	0.398434	0.100872	0.119008	ATL1	atlastin (	GO:0000137
Down	1.92561	1.76022	0.0246478	0.0266267	ALDH1A1	aldehyde dehydrogena	
Up	0.0339579	0	0.151045	0.152636	SYNE3	spectrin r	GO:0005635
Up	0.0398757	0.112986	0.251422	0.360864	ZNF316	zinc finge	GO:0003677
Down	2.05107	1.60821	0.655387	0.492671	NAMPT	nicotinamide phospho	
Up	0.0576403	0.0814267	0.158493	0.172974	FAM107B	family with sequence	

Down	0.351819	0.445257	0.218356	0.157768	AK4	adenylate	GO:0001889
Up	0.385143	0.611936	1.82469	1.29684	NSMF	NMDA recep	GO:0005634
Up	0.0080104	0	0.545472	0.435591	EPPK1	epiplakin	GO:0003723
Up	0.0595629	0.0479344	0.145777	0.169195	MOB1B	MOB kinase	GO:0005634
Down	0.684106	0.552486	0.289643	0.28184	CCDC134	coiled-coi	GO:0005576
Up	8.82381	10.9614	52.4014	48.1063	COX7A1	cytochrome	GO:0002082
Up	0.238341	0.493354	0.638004	0.700867	BIN1	bridging integrator	
Down	0.0361941	0.030723	0.0050071	0.015731	KIF26B	kinesin fa	GO:0003777
Up	0.193234	0.162074	0.634383	0.571636	AGO2	argonaute	GO:0000340
Down	320.741	168.55	99.6991	85.2459	HMGN2	high mobility group	
Up	0.155131	0.30983	0.539228	0.480868	HERC2P2	hect domain and RLD	
Down	1.7371	1.79105	1.02129	0.931479	MPZL1	myelin pro	GO:0005198
Up	0.821964	1.4546	1.66571	2.40843	LMNB2	lamin B2	GO:0005198
Up	0.046429	0.0119595	0.20758	0.327598	JMJD7-PLA2	JMJD7-PLA2	GO:0004622
Down	0.020955	0.0211111	0	0.0026772	PRLR	prolactin receptor	
Down	3.03963	2.29378	1.41526	1.58945	C11orf73	chromosome	GO:0005622
Up	0.0043702	0.0030019	0.0242147	0.0280439	GNGT1	guanine nu	GO:0001750
Up	5.0289	10.5289	13.6516	13.1744	AL513523.2		GO:0005634
Up	3.9787	3.24194	7.83402	7.71298	KDELC1	KDEL (Lys-	GO:0005788
Down	37.348	33.6385	22.1067	20.0292	PHB2	prohibitin	GO:0005634
Down	2.58007	1.94449	0.682329	0.644933	TCF21	transcript	GO:0000122
Up	0.352808	1.1996	2.19484	3.70742	TMEM121	transmembr	GO:0016021
Up	0.0027967	0	0.409104	0.163471	RP11-574K1	Bifunction	GO:0000139
Up	0	0	3.30636	4.13804	RP5-1054A22.4		
Down	0.55569	0.837563	0.266032	0.284826	SNX8	sorting ne	GO:0005829
Up	0.3684	0.71421	1.42425	1.59781	ARAP1-AS1	ARAP1 antisense RNA	
Up	1.18277	1.35053	2.69918	2.48657	FEM1A	fem-1 homo	GO:0004842
Up	0.169417	0.212506	0.750274	0.835104	RP11-69I8.3		
Up	0.154734	0	0.351223	0.551883	CHST7	carbohydra	GO:0000139
Up	0.0204424	0.0399467	0.381343	0.301926	MIRLET7BHC	MIRLET7B H	GO:0005829
Up	0.0098407	0	0.524294	0.495042	FERMT3	fermitin f	GO:0002102
Up	0.0100463	0	0.0739008	0.0529456	SLC45A4	solute car	GO:0008506
Up	0.864505	1.5204	3.24042	3.16496	RXRB	retinoid X	GO:0000977
Up	13.6359	11.1099	26.2588	24.7042	AXL	AXL receptor tyrosin	
Up	0.539133	1.44274	1.53441	1.53187	ZNF672	zinc finge	GO:0000981
Up	0.0004158	0.0070692	0.0173038	0.0153399	PREX1	phosphatic	GO:0005085
Down	7.65125	10.9265	4.88855	3.959	ALDOC	aldolase C	GO:0004332
Down	1.42625	0.937397	0.634126	0.722068	MLH1	mutL homolog 1	
Down	0.126435	0.106147	0	0	RP11-642D21.1		
Down	5.23822	1.96087	0.778702	0.576403	F2RL2	coagulation factor I	
Up	1.03246	1.11889	2.51574	2.55801	ANKRD52	ankyrin repeat domai	
Up	0.0546458	0.0691005	0.207299	0.212044	CCNJL	cyclin J-l	GO:0000079
Up	0.185555	0.256696	0.406387	0.39979	ANKIB1	ankyrin re	GO:0000151
Up	172.931	136.582	310.708	294.661	NOP10	NOP10 ribo	GO:0000454
Down	0.383935	0.33189	0.134578	0.157132	PAX8-AS1	PAX8 antisense RNA 1	
Down	0.0660717	0.153173	0	0.0028777	CHKB-CPT1B	CHKB-CPT1B	GO:0004095
Down	0.370229	0.345061	0.191488	0.189443	SHQ1	SHQ1, H/AC	GO:0000493
Down	0.061086	0.0794153	0	0	COL6A6	collagen,	GO:0005576
Down	0.252015	0.235132	0.102187	0.136351	PKN2	protein ki	GO:0003723
Down	0.0783447	0.0871349	0.0369327	0.0063143	FAM122C	family wit	GO:0004865
Down	3.61056	2.80465	1.75922	1.61366	HOXA9	homeobox A9	

Up	0.984396	0.840133	1.8184	1.94049	ENDOD1	endonuclea	GO:0002576
Up	0.46298	1.12963	1.28118	2.0025	TRIM11	tripartite	GO:0005634
Up	2.73409	1.40853	14.9403	13.893	CTD-2540B15.13		GO:0005905
Up	0.115827	0.52058	0.836077	0.55876	FIZ1	FLT3-inter	GO:0000981
Up	0.308488	0.734234	1.51974	1.68489	GIT1	G protein-	GO:0005096
Up	0	0	0.0163882	0.0351608	ZDHHC11	zinc finge	GO:0005783
Up	0	0	0.0474382	0.0371387	VWCE	von Willet	GO:0005509
Up	2.01195	2.02753	4.99222	5.32278	DVL3	dishevelle	GO:0000790
Down	1.54868	1.0502	0.820867	0.635346	HADH	hydroxyacy	GO:0003857
Up	0.0045541	0.0258076	0.149651	0.13425	ARHGEF10L	Rho guanir	GO:0005085
Down	0.276031	0.283182	0.171041	0.0809447	TMTC4	transmembr	GO:0016021
Down	0.0255329	0.0194545	0.0010823	0.0016448	SCN2A	sodium cha	GO:0001518
Down	0.0040141	0.0063978	0	0	COL21A1	collagen,	GO:0003429
Down	0.232788	0.134808	0	0.0065127	MME-AS1	MME antisense RNA 1	
Up	0.0367604	0.020225	0.125116	0.135767	TNRC18	trinucleot	GO:0003682
Up	0	0	0.0094603	0.0151613	LINC00578	long intergenic non-	
Up	2.701	5.88704	10.601	9.23451	HPS6	Hermansky-	GO:0005765
Down	0.550592	0.404123	0.135576	0.172045	ADGRL4	adhesion (	GO:0004930
Down	0.211035	0.157888	0.0414881	0.0607783	TTC39C	tetratricopeptide re	
Up	0.122696	0.205173	0.739798	0.694489	PMEPA1	prostate t	GO:0000139
Down	0.073	0.0347148	0.0070812	0.0088048	ABCA5	ATP-binding cassette	
Up	0	0	0.772766	1.27651	RP11-343L5.2		
Down	2.78864	4.99559	0.845597	0.954616	MMP2	matrix metallopeptic	
Up	0.0891395	0.218041	0.809466	0.799589	NCOR2	nuclear re	GO:0000118
Down	0.119174	0.116248	0.0098545	0.00468	SLCO4A1	solute car	GO:0005886
Down	0.0844747	0.0256452	0	0.0057817	TEX29	testis exp	GO:0016021
Up	0.487638	0.213535	0.979687	0.854869	RP11-513I15.6		GO:0000184
Up	0.108478	0.112371	0.251525	0.29954	CPOX	coproporph	GO:0004109
Down	1.62396	2.21143	0.0427911	0.0789658	SERPING1	serpin peptidase inh	
Up	0.508271	1.4132	3.25033	2.37495	TCEAL3	transcript	GO:0005634
Up	0.0008278	0.0093823	0.0247925	0.0162609	MOB3B	MOB kinase	GO:0035330
Up	1.47909	2.88754	3.29901	3.53754	LRRC47	leucine ri	GO:0003723
Up	0.0118558	0.0035833	0.111232	0.147382	FRY	furry homo	GO:0000902
Up	0	0.408312	0.374128	0.471102	SEMA3B	sema domain, immunog	
Up	1.2587	1.18226	2.26489	2.31648	IL13RA1	interleuki	GO:0004896
Down	7.21101	4.75032	0.747161	0.845157	F3	coagulation factor I	
Up	1.03197	1.70964	4.91549	4.34882	BST1	bone marro	GO:0001931
Up	0.65196	0.413061	1.86386	2.86359	STEAP1	six transmembrane ep	
Down	2.67293	2.19635	0.881983	1.20164	PERP	PERP, TP53	GO:0002934
Up	0.0765723	0	0.199486	0.236242	HSPE1-MOB4	HSPE1-MOB4	GO:0005737
Up	0.136561	0	1.51925	1.27946	PGF	placental	GO:0001525
Up	0.198351	0.406039	0.59942	0.487293	RARA	retinoic acid recept	
Up	0.421701	0.354522	1.3645	1.09234	FHOD1	formin hom	GO:0003779
Down	1.17473	1.11393	0	0.10763	METTL7A	methyltran	GO:0005576
Down	1.7869	1.88312	1.05946	0.570739	RBMXL1	RNA bindir	GO:0003723
Up	0	0	0.0077679	0.0288566	FGD5	FYVE, Rho(	GO:0001726
Up	0.904149	1.12514	3.98212	3.65587	FBN1	fibrillin	GO:0001501
Down	1.2472	0.78179	0.257425	0.211685	RP2	retinitis pigmentosa	
Down	0.000861	0.0017565	0	0	GALNTL6	polypeptic	GO:0000139
Down	0.0500173	0.0694265	0.0058732	0.0049479	TANC2	tetratrico	GO:0001701
Up	0.116899	0.0747601	0.274956	0.25879	MECP2	methyl Cp(	GO:0000122

Up	0.151692	0.143271	0.716003	0.636927	CSNK1G2	casein kir	GO:0000287
Down	1.64818	1.23612	1.10722	0.731925	TAF12	TAF12 RNA polymerase	
Down	0.0136774	0.0465051	0	0	FAM124A	family with sequence	
Down	5.80821	4.1515	3.11063	2.69891	SEC11C	SEC11 homo	GO:0005787
Up	0.104995	0.0598146	0.328553	0.374216	ITPR3	inositol 1	GO:0000822
Up	0.104679	0.186751	0.711352	0.71699	TCEA3	transcript	GO:0003677
Down	0.399163	0.264275	0.16845	0.120046	SEC24B	SEC24 fami	GO:0000139
Down	0.0687995	0.0646536	0.0213625	0.0245145	ZNF420	zinc finge	GO:0000981
Down	0.0711127	0.100747	0.0078466	0.0021294	KREMEN1	kringle co	GO:0005886
Down	7.46848	5.23127	3.2473	3.71509	MOB1A	MOB kinase	GO:0005730
Up	0.004674	0.0059368	0.018292	0.019382	PLXNA4	plexin A4	GO:0002116
Up	0.136821	0.58517	0.9701	1.08345	MEIS3	Meis homeo	GO:0000981
Up	0	0	0.483515	0.530365	KDM5D	lysine (K)	GO:0000981
Down	4.47862	3.77617	1.31426	2.19123	TSN	translin	GO:0003677
Down	0.336774	0.255261	0.126647	0.127353	TCF12	transcript	GO:0005634
Up	0.223643	0.349171	0.926636	0.710675	ZDHHC8	zinc finge	GO:0005739
Up	0.135061	0.588345	2.00707	1.70853	ADAM19	ADAM metal	GO:0001890
Up	0.609078	0.749358	1.36433	1.27283	OBSL1	obscurin-l	GO:0000226
Up	0.166175	0.214023	1.70499	1.90353	COL4A2-AS1	COL4A2 antisense RNA	
Down	0.364556	0.403749	0.0090639	0.0065248	CADM1	cell adhes	GO:0001889
Down	0.0515646	0.172671	0	0.0067377	RP11-473E2.4		
Up	0.974853	2.46137	5.63133	5.51834	NFE2L1	nuclear fa	GO:0000122
Up	0	0	0.0040421	0.0068517	COL19A1	collagen,	GO:0001501
Up	0.356229	0.521106	0.883306	0.735551	FGF1	fibroblast growth fa	
Up	0.534434	0.346304	1.06765	1.2318	SLC25A36	solute car	GO:0000002
Up	0.0689371	0.0471487	0.205339	0.250557	PARP10	poly (ADP-	GO:0003950
Down	0.719216	0.313518	0.0348828	0.013253	CTD-2587H24.14		GO:0005737
Up	0.0364816	0	0.437041	0.405183	NPTXR	neuronal p	GO:0005886
Down	0.163357	0.0772756	0.0431648	0.0425324	GULP1	GULP, engu	GO:0005737
Down	1.95735	1.42553	0.946018	0.697448	IFT46	intraflage	GO:0005737
Up	0.0029266	0	0.0369053	0.0291215	SLC22A23	solute car	GO:0006811
Up	0.641474	0.60515	1.18435	1.09338	PICALM	phosphatidylinositol	
Up	0.0333431	0.0111149	0.134178	0.112293	AP1S3	adaptor-re	GO:0000139
Up	0.0074157	0.0129304	0.0273347	0.0388082	SHROOM4	shroom fam	GO:0001725
Up	0	0	0.0161068	0.0254978	PLCH2	phospholip	GO:0004435
Up	1.10444	1.94717	3.60563	3.29244	TMPO-AS1	TMPO antis	GO:0003677
Up	0.778191	1.25105	3.45898	3.35286	RGL2	ral guanin	GO:0005088
Down	9.87177	10.0774	5.8218	3.47347	HOXA11	homeobox A11	
Up	0.0259616	0.0490407	0.297373	0.373149	PPAPDC3	phosphatic	GO:0005635
Up	0.285718	0.726797	0.901197	1.25009	SUN2	Sad1 and U	GO:0000784
Up	0.0002348	0.0006654	0.0228018	0.0133884	NHS	Nance-Hora	GO:0002088
Up	2.23788	6.33747	10.1129	9.99684	COL6A2	collagen,	GO:0003429
Up	0.101218	0	0.348107	0.424985	KRT80	keratin 80	GO:0005198
Up	0.821951	1.41193	2.64956	2.08222	RCC2	regulator	GO:0003723
Up	3.73163	4.1856	6.15454	8.04875	TAP1	transporter 1, ATP-b	
Up	0	0.0266881	0.100959	0.0473827	CYP27C1	cytochrome P450, fam	
Up	0.161784	0.156792	0.547323	0.489151	RFTN1	raftlin, l	GO:0001765
Down	0.673821	0.808418	0.0918603	0.141056	MAP3K8	mitogen-activated pr	
Down	5.31678	4.42149	3.06125	2.84824	TMEM237	transmembr	GO:0016020
Up	0.0785283	0.107133	0.352094	0.271723	GID4	GID comple	GO:0000151
Up	0.0347671	0.0866897	0.142487	0.108271	TET3	tet methyl	GO:0000981

Up	0.425113	1.07179	2.75589	2.75665	PLXNA1	plexin A1	GO:000211
Up	0.175483	0.24336	0.361544	0.375376	RBM33	RNA bindir	GO:000372
Up	1.25413	1.29219	3.09515	2.88299	TIMP2	TIMP metallopeptidas	
Up	0.0142089	0.0332585	0.0607647	0.0667138	MAGI1	membrane a	GO:000552
Down	0.290437	0.374064	0.134291	0.0252999	TXNRD3	thioredoxi	GO:0004791
Up	0.0216138	0.0236556	0.197574	0.177328	HMCN1	hemicentir	GO:0005201
Up	0.0370774	0.140076	0.205205	0.25988	SLC12A7	solute car	GO:000588
Down	4.36872	4.20122	1.13521	1.56029	RP11-138A9.2		
Down	0.0470652	0.0317073	0.0020752	0.002523	MAP2K6	mitogen-ac	GO:000018
Down	1.16912	1.85574	0.306089	0.357823	GPRC5B	G protein-	GO:000166
Up	0.290169	0.933036	2.20369	1.7401	ATP13A2	ATPase typ	GO:000552
Up	0	0.179178	0.777496	1.01495	RP11-245P10.8		
Up	53.5372	52.1543	98.4979	88.4921	MYL9	myosin, li	GO:000172
Up	15.5603	14.8802	42.5142	36.1567	RP11-110I1.6		
Up	0.128074	0.233184	0.357386	0.257764	BAIAP2	BAI1-assoc	GO:0001221
Up	1.25641	1.25646	7.25032	6.84387	ORAOV1	oral cance	GO:0016021
Down	0.449357	0.266703	0.0268286	0.0030888	TDO2	tryptophar	GO:000483
Up	0.0014532	0.008235	0.588233	0.84243	RP11-295K	Uncharacterized prot	
Up	0.653005	0.449456	2.15366	2.0798	JOSD1	Josephin c	GO:000484
Down	0.061903	0.10691	0.0059475	0.0209018	MOV10L1	Mov10 RIS(	GO:000028
Down	0.244743	0.258757	0.0552767	0.0140009	SLC40A1	solute car	GO:000226
Down	3.26788	2.70524	1.52462	1.4873	NDUFS1	NADH dehyc	GO:000573
Down	0.675003	0.386712	0.271637	0.221375	COG2	component	GO:000013
Down	0.0070309	0.0071149	0	0	ANKRD31	ankyrin repeat domai	
Down	1.62553	1.26614	0.907137	0.456257	UBE2Q2	ubiquitin-	GO:000484
Down	16.8794	8.15413	3.97793	4.40144	PGAM4	phosphogly	GO:000408
Up	0.181591	0.237971	1.90779	1.29632	RP11-295P9.3		GO:0005737
Up	0	0	0.0898089	0.0442311	ZNF560	zinc finge	GO:0000981
Up	0.0004725	0	0.0065545	0.0124059	TMTC2	transmembr	GO:000578
Down	1.21889	1.54873	0.588172	0.60929	SYNC	syncoilin,	GO:000519
Down	1.96065	1.61903	0.870006	0.915289	IARS2	isoleucyl-	GO:000004
Up	0.530624	1.0441	1.80294	1.63598	ACAP3	ArfGAP wit	GO:000176
Down	0.778484	0.810781	0.0424515	0.120965	PENK	proenkepha	GO:000151
Down	3.93715	2.92436	2.04105	2.46135	ATP6V1B2	ATPase, H	GO:000172
Down	1.40686	1.23117	0.675724	0.771137	TULP3	tubby like	GO:000184
Down	0.337659	0.491378	0.0707519	0.0664689	DNAJC12	DnaJ (Hsp4	GO:0005737
Up	0.0053604	0	0.0630896	0.0662019	TSPAN12	tetraspani	GO:000152
Up	0.0655137	0.153219	0.319968	0.306405	SKI	SKI proto-	GO:000012
Down	0.395854	0.458851	0.147486	0.123563	SLC16A4	solute car	GO:0005887
Down	11.1057	10.6191	6.32246	6.36996	RCN2	reticuloca	GO:000550
Up	0	0	0.311391	0.674351	ARC	activity-r	GO:000166
Down	0.0312558	0.110702	0.0049268	0	FHDC1	FH2 domair	GO:000377
Down	1.93475	2.53947	0.143516	0.061342	FAM84B	family wit	GO:0005737
Up	0.0276067	0	0.368022	0.207845	GPR37L1	G protein-	GO:000308
Up	0.0388255	0.176017	0.35643	0.351195	CIC	capicua tr	GO:000012
Up	0.458459	1.09917	2.53488	3.4637	RP3-416H24.1		GO:000519
Up	0.0338537	0	10.4876	11.2103	KCNMB1	potassium	GO:000551
Up	0.0201196	0.0604982	0.114948	0.0723935	EVC	Ellis van	GO:0001501
Up	0.130587	0.142655	0.509894	0.37388	IL7R	interleukin 7 recept	
Up	0.565149	0.509512	2.48894	2.19484	WEE1	WEE1 G2 ch	GO:000008
Down	0.922945	0.833438	0.675607	0.299105	TMEM168	transmembr	GO:0016021

Down	0.314993	0.335606	0.0627751	0.0616815	MUC12	mucin 12, cell surfa	
Down	0.749658	0.424824	0	0	AC005082.12		
Up	0.195887	0.342273	0.442576	0.428974	SH3BP4	SH3-domair	GO:0005092
Down	0.0822028	0.22586	0	0	CTD-2298J14.2		GO:0003964
Up	3.98175	11.5151	86.3511	77.8037	CRIP2	cysteine-r	GO:0005938
Down	18.7605	12.23	7.93765	7.44525	PLK1	polo-like kinase 1	
Down	13.812	15.7394	5.20628	4.17184	HIST2H2AA4	histone cl	GO:0000786
Down	16.2067	24.8231	8.4981	11.3416	HERPUD1	homocystei	GO:0005783
Up	0.29788	0.720396	0.840356	0.851405	GALNT2	polypeptide N-acetyl	
Down	2.24694	1.6847	0.801116	0.834714	SNX2	sorting ne	GO:0005829
Up	0.604615	0.37573	1.23343	1.13963	ARHGAP18	Rho GTPase	GO:0005096
Up	0.0146888	0.0277468	0.0941588	0.124915	SUSD5	sushi doma	GO:0005540
Up	0.222563	0.259893	0.811358	0.806517	NBPF8	neuroblast	GO:0005737
Up	7.13981	5.22571	11.3341	12.8701	DYNLT3	dynein, li	GO:0000777
Up	0.0092701	0.0035616	0.040023	0.0269492	TYW1B	tRNA-yW sy	GO:0008033
Up	0.0364738	0	18.2023	36.6182	KRT14	keratin 14, type I	
Down	3.72248	2.72761	1.52964	1.8597	CASP3	caspase 3, apoptosis	
Down	0.0040755	0.0069287	0	0	CNTN6	contactin	GO:0005112
Up	0.0001917	0	0.0229612	0.031099	EPHB1	EPH recept	GO:0001525
Down	1.36335	1.11319	0.558582	0.634486	PAPSS1	3'-phospho	GO:0000103
Up	0.0019832	0	0.148299	0.168555	STEAP1B	STEAP family member	
Down	51.4588	53.3155	5.20985	3.05726	TMEM88	transmembr	GO:0005829
Up	0.0840412	0.383649	0.846355	0.838842	SP1	Sp1 transc	GO:0000790
Down	1.28815	1.35548	0.28906	0.0572988	ARHGEF39	Rho guanir	GO:0005089
Up	0.0421802	0.243742	0.618896	0.656036	PTPN14	protein tyrosine pho	
Down	1.68986	1.44347	0.674683	0.644765	RHOBTB3	Rho-relate	GO:0003924
Up	0.111584	0.111086	0.373642	0.32943	KIF3C	kinesin fa	GO:0003774
Down	3.045	2.53347	2.01701	1.40413	SLC2A3	solute car	GO:0005355
Down	0.259678	0.315523	0.122943	0.146771	WDFY2	WD repeat	GO:0001934
Down	15.1442	13.6372	6.43015	6.27651	GLUL	glutamate-ammonia li	
Up	0.131985	0.557477	1.39456	1.81927	PLXND1	plexin D1	GO:0001525
Down	2.42477	1.50786	0.584482	0.403002	DNAJC14	DnaJ (Hsp4	GO:0005789
Up	4.80487	14.0838	15.67	16.8036	PHLDA3	pleckstrir	GO:0005546
Down	0.0307062	0.0208811	0.0037173	0.0040015	ADAMTS9	ADAM metal	GO:0003179
Up	0	0	0.809715	0.887604	ACTC1	actin, alp	GO:0005524
Down	3.65766	4.40606	0.861098	0.39518	HSBP1L1	heat shock	GO:0003714
Down	0.368381	0.374594	0	0	CFI	complement factor I	
Down	0.568091	0.739845	0.0088991	0	ANXA8	annexin A8	GO:0005509
Down	2.32867	1.9294	0.931587	0.987951	N6AMT1	N-6 adenir	GO:0003676
Up	0.0017991	0.0094112	0.0525301	0.0335503	PALM2	paralemmir	GO:0005886
Down	90.112	124.115	24.3618	24.6303	S100A4	S100 calcium binding	
Up	0.0090085	0.0159532	0.249208	0.22605	FAM212B	family wit	GO:0005634
Down	0.372819	0.403905	0.0760514	0	AC009404.2		
Down	3.30541	2.87054	1.74187	1.5914	GLUD1	glutamate dehydrogen	
Up	0.764378	0.941369	1.61662	1.40258	RBBP6	retinoblas	GO:0000209
Up	0.661353	0.758543	2.34115	2.3178	PDLIM1	PDZ and LIM domain 1	
Up	4.1439	3.62774	9.42651	7.81715	MTPN	myotrophir	GO:0005634
Up	0.0263276	0.062165	0.221332	0.176591	ZNF775	zinc finge	GO:0000981
Down	3.59418	3.08963	2.05017	1.50607	ADPGK	ADP-depenc	GO:0005576
Down	45.8604	34.3247	27.3576	25.6806	NDUFA6	NADH dehyc	GO:0005743
Down	0.626692	0.933228	0.103356	0.12445	MATN2	matrilin 2	GO:0001764

Up	0.0144289	0.0577181	0.200148	0.183803	IL21R	interleukin 21 recep	
Up	0.300542	0.642287	0.982809	0.993291	RABL6	RAB, membe	GO:0003924
Down	5.88194	7.68215	3.5025	3.96037	ANXA7	annexin A7	GO:0003723
Down	1.41717	1.15346	0.623502	0.718383	CIRH1A	cirrhosis,	GO:0000462
Down	0.0095178	0.0134841	0	0	RP11-9602(	Uncharacte	GO:0005743
Up	0.0235103	0.052531	0.202448	0.213949	PDE3A	phosphodiesterase 3A	
Up	1.25657	2.00138	2.80446	2.58344	GATA6	GATA binding protein	
Up	0.0307432	0.0186407	0.0784933	0.0932241	MGAT5	mannosyl (alpha-1,6-	
Down	11.5444	11.1947	6.25893	7.34541	CENPV	centromere	GO:0000776
Up	0.293937	0.609822	0.869488	1.43404	HSPB8	heat shock	GO:0005634
Up	0.026447	0.081749	0.257708	0.327139	RP11-45M22.3		
Down	0.2769	0.290251	0	0	SUSD3	sushi doma	GO:0005886
Up	1.24697	1.95102	3.0514	3.05889	POLR2A	polymerase	GO:0000398
Down	0.0300859	0.0818373	0	0	ASB9	ankyrin re	GO:0005739
Down	2.55811	3.47981	1.60772	1.38787	HPS1	Hermansky-Pudlak syn	
Up	0.4228	0.479194	1.60564	2.16744	RILPL2	Rab intera	GO:0003382
Down	0.242773	0.458591	0	0	CH17-302M23.1		
Down	15.2676	10.5746	7.82971	7.93904	PPP2CA	protein ph	GO:0000775
Up	1.68598	0	11.4808	11.2727	RP11-579D7.2		
Down	1.00651	0.869859	0.154852	0.163892	ANKRD35	ankyrin repeat domai	
Down	1.94803	1.2602	0.777177	0.883994	RIPK2	receptor-i	GO:0000165
Up	0.688297	1.96538	1.86713	2.49369	AP3D1	adaptor-re	GO:0000139
Down	0.609086	0.360633	0.0291262	0.0470495	MGST1	microsomal glutathio	
Up	0	0	0.570242	0.698385	FAM212B-AS	FAM212B antisense RN	
Up	0.335529	0.414411	1.52971	1.49624	PRR7	proline ri	GO:0002250
Down	0.259576	1.25035	0	0	ELOVL3	ELOVL fatt	GO:0005783
Down	25.7221	21.4529	14.0136	12.6133	RAB32	RAB32, mem	GO:0003924
Down	1.25882	1.31729	0.59077	0.719617	PQLC3	PQ loop re	GO:0016021
Up	6.34084	8.17595	13.0948	11.7509	BAX	BCL2-associated X pr	
Down	0.0258965	0.0399537	0.0037614	0.0101335	COL14A1	collagen,	GO:0003429
Up	0.12608	0.279581	0.361186	0.579105	ZDHHC18	zinc finge	GO:0005783
Down	7575.38	7113.36	2295.01	2104.22	FTL	ferritin, light poly	
Down	4.06872	3.06381	1.51942	1.66817	ALDH9A1	aldehyde d	GO:0004028
Down	0.448311	0.753059	0.134428	0.201494	C19orf47	chromosome	GO:0005654
Up	1.82422	1.95657	8.16656	9.95144	COL6A3	collagen,	GO:0003429
Down	0.0127182	0.0332644	0	0	CTC-347C20.2		GO:0003964
Down	3.85553	2.64995	1.35183	1.73739	FOPNL	FGFR1OP N-	GO:0005634
Down	1.83893	1.46735	0.933602	0.896094	UFL1	UFM1-speci	GO:0001649
Down	11.0675	14.4522	0.03414	0.0207533	CHI3L1	chitinase	GO:0005201
Up	0.532665	0.871672	1.46554	1.29648	MXD4	MAX dimeri	GO:0000122
Down	0.765689	0.618541	0.268037	0.397906	PIP5K1A	phosphatid	GO:0000285
Down	11.4143	10.3538	6.61467	4.19794	BEX4	brain expr	GO:0000922
Up	1.79712	3.31577	8.74869	8.04943	AC005262.2		
Down	0.0849474	0.231317	0.0055647	0.0089854	ADORA1	adenosine	GO:0000186
Up	5.01552	6.68765	28.8333	25.8671	RP5-890E16.4		
Up	0.525078	1.12354	1.40654	1.98863	MRC2	mannose receptor, C	
Up	0.600799	1.59251	2.09569	2.6881	ABHD17A	abhydrolas	GO:0002084
Up	0.0766202	0	2.08807	2.44319	B4GALNT1	beta-1,4-N-acetyl-ga	
Up	0.297367	0.42491	0.954126	0.653973	NADK2	NAD kinase	GO:0003951
Down	1.1004	0.80463	0.492388	0.489496	ZBTB2	zinc finge	GO:0000122
Down	0.906904	0.626749	0	0	AC008063.2		

Up	0.004449	0.0252122	0.230024	0.200365	C15orf52	chromosome	GO:0003723
Up	0	0	0.116137	0.238269	IL17RE	interleuki	GO:0005576
Up	0.143981	0.0370876	0.85005	0.808968	RP11-44M6.7		GO:0005634
Up	0.299403	0.365666	0.654755	0.657044	SMARCC1	SWI/SNF re	GO:0000790
Up	2.73936	4.92817	7.07332	7.62464	RP11-147L13.11		GO:0016021
Down	2.55604	2.50994	0.814336	0.641461	RNFT1	ring finge	GO:0005789
Up	2.66517	3.71393	8.81537	14.5691	AC068831.15		
Down	0.0676215	0.0277685	0	0	CECR1	cat eye sy	GO:0004000
Up	0.0722753	0	1.8974	2.24973	SCUBE3	signal pep	GO:0005509
Up	0.525088	0.51489	1.34449	1.19726	RBPMS	RNA bindir	GO:0000398
Up	0.0443009	0	0.651755	0.672116	GHDC	GH3 domair	GO:0005576
Up	4.29409	9.28358	11.5394	11.1785	ZNF281	zinc finge	GO:0000122
Up	0.0537987	0.0544686	0.122721	0.137126	NFIB	nuclear fa	GO:0000122
Down	0.597076	0.314321	0.113145	0.0250108	HOXA-AS3	HOXA clust	GO:0000122
Up	2.23826	2.76146	8.65499	6.26794	TPRG1L	tumor prot	GO:0005737
Down	27.9408	23.5303	14.0645	12.3944	CFL2	cofilin 2	GO:0007519
Up	0.0650874	0.146855	0.28271	0.308371	TMEM120B	transmembr	GO:0005637
Up	0	0	3.56229	3.26931	DLX2	distal-les	GO:0000122
Down	0.151872	0.258194	0	0	DES	desmin	
Down	2.27536	2.20514	0.235019	0.149145	SMPDL3A	sphingomye	GO:0005615
Down	6.58184	4.54158	3.59143	3.52236	XRCC5	X-ray repair complem	
Up	0.569049	1.41959	2.22101	2.37072	RP11-571M6.7		GO:0000836
Up	0.963215	3.5338	4.11434	4.55078	IGSF8	immunoglobulin super	
Up	0.193939	0.228065	0.373845	0.41206	SDCCAG8	serologica	GO:0000086
Down	62.8926	48.9139	17.6303	14.0705	SNAI2	snail family zinc fi	
Up	1.2675	1.23386	3.36832	3.33437	RP11-63M22.2		
Down	1.65395	1.43116	0.812963	0.861491	RMDN1	regulator	GO:0000922
Up	0	0	0.0699603	0.0366001	RXFP1	relaxin/ir	GO:0004930
Up	10.0935	15.7598	28.1381	22.1648	HES1	hes family	GO:0000122
Down	8.84589	7.16984	2.47998	4.16201	HDAC3	histone de	GO:0000118
Down	9.3501	8.47369	3.59861	4.15858	PGM3	phosphoglu	GO:0000287
Up	0	0	0.835035	0.452732	ZNF154	zinc finge	GO:0000981
Down	13.1465	11.155	5.64334	5.66445	COPB2	coatomer p	GO:0000139
Down	6.42274	10.6719	3.01272	2.98177	CPA4	carboxypep	GO:0004181
Down	22.9997	17.7089	13.7923	11.8696	ISCA2	iron-sulfu	GO:0005198
Up	20.4312	25.3646	67.7975	56.8652	AC093673.5		
Up	0	0	0.0468965	0.0399902	TTTY14	testis-spe	GO:0005654
Up	0.119815	0	0.879877	0.641571	CTC-548K16.5		
Down	0.0661734	0.119318	0.0025287	0	TNFRSF1B	tumor necrosis facto	
Down	0.613642	0.858941	0.0754645	0.0587526	ACVR2A	activin A receptor,	
Down	7.29632	6.54671	4.05222	4.32883	NELFCD	negative e	GO:0003723
Down	2.44766	1.35833	0.122068	0.150174	RSPH9	radial spo	GO:0003341
Up	0.0441308	0.17506	0.651108	0.803443	NPIPP1	nuclear po	GO:0005654
Down	0.0284752	0.0510994	0	0	AL035610.1		GO:0005829
Down	11.9733	14.3271	5.38284	4.74336	COPS8	COP9 signa	GO:0000338
Down	0.668843	0.78979	0.301741	0.374411	TCP11L1	t-complex	GO:0005874
Up	0.219914	0.454183	0.87386	0.714586	TCF3	transcription factor	
Up	0	0	0.653309	0.387453	C2orf27B	chromosome 2 open re	
Up	0.192028	0.269126	0.606683	0.633126	ZNF276	zinc finge	GO:0000776
Down	0.938817	0.51626	0.168089	0.147939	HPS3	Hermansky-Pudlak syn	
Down	2.51031	2.97747	1.1839	0.950971	CALCOCO2	calcium bi	GO:0000421

Down	0.0620784	0.125969	0.015383	0.0168841	EPHA4	EPH recept	GO:000154(
Down	2.99564	3.00683	1.10122	1.16512	CAMK2N1	calcium/ca	GO:0005622
Down	0.163378	0.13137	0.0473298	0.0211554	CH507-24F1.2		
Up	0.893945	2.57739	2.42268	3.17838	GALNT4	polypeptide N-acetyl	
Down	1.94533	2.28161	0.656009	0.594924	TMX4	thioredoxi	GO:0005623
Up	6.77133	7.54955	17.7613	14.1124	ANKRD37	ankyrin re	GO:0005634
Up	0.0439822	0.0721494	0.207621	0.216543	STK10	serine/thr	GO:0004674
Down	9.10055	7.92142	3.00644	3.38821	TMX1	thioredoxi	GO:0005789
Up	0.336636	0.475799	0.800072	0.85613	BICD2	bicaudal D	GO:0005635
Up	0.305705	0.441073	0.899311	1.1533	UNC5B	unc-5 netr	GO:0001525
Down	1.0588	0.917438	0.521149	0.515783	MRPS9	mitochondr	GO:0000462
Up	0.0020781	0	0.0532841	0.078654	SYTL5	synaptotag	GO:0005543
Up	3.44466	6.33653	10.4161	10.3601	PHF23	PHD finger	GO:0003682
Up	0.108865	0.121182	0.249157	0.256385	TMEM131	transmembr	GO:0016021
Up	74.6744	107.873	223.501	198.53	CH507-513H4.4		GO:0005524
Up	0	0	0.0596039	0.171458	TLR4	toll-like receptor 4	
Up	0.35031	0.355633	1.01874	0.848149	OXSR1	oxidative stress res	
Down	22.6548	18.4934	13.3081	12.9398	IDH3B	isocitrate	GO:0000287
Down	0.0269304	0.1128	0	0	ADGRF4	adhesion G	GO:000493(
Down	0.530882	0.32157	0.194	0.148621	HSF2	heat shock transcrip	
Down	10.1884	7.98637	5.6661	5.89663	GGH	gamma-glutamyl hydro	
Down	4.04908	2.81347	1.55204	1.31892	ZADH2	zinc bindi	GO:0005777
Down	1.10249	1.02638	0.36426	0.662153	PREPL	prolyl end	GO:0004252
Up	0	0	0.464194	0.811262	RP11-527D7.1		
Up	0.286388	0.282973	0.922304	0.951318	SNX18	sorting ne	GO:0000281
Up	0.384156	0.447685	0.757901	0.733975	KDM5A	lysine (K)-specific	
Down	0.383107	0.282189	0.05773	0.062337	OCRL	oculocerebrorenal sy	
Down	0.0051318	0.0024235	0	0.0004098	GABRB3	gamma-aminobutyric a	
Up	2.18829	3.13975	5.59189	4.50114	CD81	CD81 molec	GO:0000187
Down	0.0810202	0.0437271	0.0102169	0.0170056	RBKS	ribokinase	GO:0004747
Up	0.0056696	0.0028349	0.0365886	0.0388273	ABLIM1	actin binc	GO:0001725
Down	0.252592	0.464616	0.0036228	0.0042351	VAT1L	vesicle am	GO:000827(
Down	0.391486	0.627735	0.0225581	0.0270301	TMEM154	transmembr	GO:0016021
Up	0.006363	0.0065561	0.0583556	0.0482221	LINC00869	long inter	GO:0005802
Up	0.0845639	0	1.07704	0.488204	PTPRN	protein ty	GO:0000302
Down	0.0038303	0.0233755	0	0	SLC16A10	solute car	GO:0005886
Up	0.0451568	0.165096	0.367993	0.340573	PACS1	phosphofur	GO:0005794
Up	0	0	0.0095128	0.0490773	EEF1DP3	eukaryotic	GO:0003746
Down	0.278651	0.261744	0.108488	0.107969	NEDD4	neural pre	GO:0000151
Up	0.0250144	0.0559214	0.095789	0.0754252	KDM4B	lysine (K)	GO:0001078
Up	0.341369	0.134341	1.53058	1.31295	ATN1	atrophin 1	GO:0000122
Down	0.504777	0.306485	0.0941818	0.0715651	LYRM9	LYR motif containing	
Up	0.996015	2.14769	3.06892	2.77613	TAF13	TAF13 RNA	GO:0003677
Up	0.0808876	0.146292	1.34881	0.952347	CHST3	carbohydra	GO:0000139
Down	0.073894	0.108383	0.0219253	0.0058311	FERMT1	fermitin f	GO:0001954
Up	0.518832	0.797098	1.92778	2.19608	MLXIP	MLX intera	GO:0000977
Up	0	0	0.0786747	0.126206	ST3GAL6-AS	ST3GAL6 antisense RN	
Down	6.41842	5.72297	1.981	1.93537	GSTT2B	glutathion	GO:0004364
Up	0	0	0.0194039	0.0240121	MYO18B	myosin XVI	GO:000157(
Down	7.93198	9.31	4.55498	4.68046	GLT8D1	glycosyltr	GO:0005794
Up	0.485103	0.764418	1.15957	1.01522	SPG7	spastic paraplegia 7	

Up	0.349833	0.343292	0.649489	0.644543	TAOK1	TAO kinase	GO:000018б
Up	0.277612	0.4185	0.560179	0.63331	ADAM12	ADAM metallopeptidas	
Down	5.43392	5.55971	1.66425	3.28301	DCTD	dCMP deami	GO:0004132
Up	0.243552	0.258269	0.82069	1.02293	PAWR	PRKC, apoptosis, WT1	
Down	0.656689	0.47952	0.349299	0.268191	SLC30A6	solute car	GO:0000139
Up	0.0699206	0.311327	1.05176	0.708269	NACAD	NAC alpha	GO:0005634
Down	5.41815	3.65108	2.40065	2.88273	GPN1	GPN-loop (	GO:0003924
Down	3.31831	4.26353	0.724081	0.539148	NFKBIZ	nuclear fa	GO:0005634
Up	0.229627	0.166121	0.479787	0.451765	OTUD4	OTU deubiquitinase 4	
Up	0.151203	0.571237	2.8283	2.46303	PANO1	proapoptot	GO:0005730
Down	1.89676	2.73843	0.816635	0.60793	NRSN2	neurensin	GO:0005886
Down	0.167773	0.134346	0.0476354	0.049177	COPG2	coatomer p	GO:0000139
Up	0	0	0.0624655	0.088671	ARPP21	cAMP-regul	GO:0003676
Up	0.122891	0.255025	0.72028	0.789458	SCRIB	scribbled	GO:0001843
Up	3.44547	6.84556	7.84965	9.50739	CYTH2	cytohesin	GO:0005086
Up	0.100279	0.186159	0.402258	0.232766	PABPC1L	poly(A) bi	GO:0001556
Down	14.4635	13.6304	4.64123	4.85952	TMEM98	transmembr	GO:0005783
Up	1.44052	1.12952	9.25789	7.35013	ENC1	ectodermal-neural co	
Up	0.281271	0.155229	0.609966	0.614572	UTS2B	urotensin	GO:0001664
Up	0.372363	0.584991	1.04256	1.20641	CLN8	ceroid-lipofuscinosi	
Up	0.281524	0.869274	0.989932	0.951072	NOM1	nucleolar	GO:0003723
Up	0.973157	2.44671	3.01321	2.82154	FADS3	fatty acid	GO:0005789
Down	63.9318	115.799	18.7329	13.4635	IGFBP3	insulin-like growth	
Up	0.129328	0.140554	0.31584	0.238297	ARHGEF7	Rho guanir	GO:0000322
Up	0.490509	0.785334	1.22099	2.00409	FUCA1	fucosidase	GO:0004560
Down	0.973785	0.672254	0.380519	0.30404	NCAPG2	non-SMC co	GO:0000796
Up	0.10928	0	0.717736	0.349043	ZNF467	zinc finge	GO:0000981
Down	5.80694	4.229	3.02892	3.08868	NAA50	N(alpha)-a	GO:0004596
Down	1.15884	0.902231	0.533189	0.400982	CEP41	centrosome	GO:0000086
Up	1.12036	1.54076	3.3192	3.02297	FEM1B	fem-1 homo	GO:0004842
Up	0	0.0047527	0.0456087	0.033049	EBF1	early B-ce	GO:0000978
Up	0.172286	0.164718	0.416855	0.346882	ZEB2	zinc finge	GO:0000122
Up	0.157171	0.112985	0.295499	0.302445	NUTM2B-AS1	NUTM2B ant	GO:0003964
Up	0.0328513	0.0681093	0.111649	0.234168	PVRL1	poliovirus	GO:0001618
Up	0.566123	2.97441	3.29027	3.35155	FBLN2	fibulin 2	GO:0005201
Up	0.0295988	0.0354706	0.129902	0.113854	TNIK	TRAF2 and	GO:0000165
Up	0.186452	0.271103	2.5554	6.37416	ITGA3	integrin,	GO:0001764
Up	0.255916	0.324684	0.580191	0.606563	SNTB2	syntrophin	GO:0003723
Down	0.68445	0.584144	0.166383	0.2252	ZNF543	zinc finge	GO:0000981
Down	0.250754	0.280863	0.056311	0.0489012	MAN1A1	mannosidas	GO:0000139
Up	0	0.0103949	0.0318055	0.058442	ZNF883	zinc finge	GO:0003677
Up	0.497211	0.496131	1.15901	1.1906	SP100	SP100 nucl	GO:0000122
Down	0.361355	0.516488	0.178063	0.153138	SLC9B2	solute car	GO:0005451
Up	0	0	0.102615	0.103964	LINC01013	long intergenic non-	
Up	6.04813	10.7615	23.0869	22.2373	TLN1	talin 1	GO:0001726
Up	0	0	2.24736	1.88474	EMX2	empty spir	GO:0000981
Down	0.0844499	0.0878111	0.0234482	0.0111358	MYO18A	myosin XVI	GO:0000139
Up	0.0904807	0.0808999	0.396556	0.348146	SYNPO2	synaptopodin 2	
Up	0.185101	0.203285	0.437281	0.405144	LATS2	large tumor suppress	
Down	0.186414	0.227075	0.0307573	0.0033388	ADAMTS5	ADAM metal	GO:0004222
Down	1.65228	1.1636	0.740377	0.451912	SLC31A2	solute car	GO:0005375

Up	0.314315	0.535063	1.193	1.25293	PGM2L1	phosphoglu	GO:0004614
Up	0.0695644	0.123192	0.220403	0.255797	ACSF2	acyl-CoA s	GO:0003996
Down	1.23355	1.55625	0.752134	0.603874	CLNS1A	chloride c	GO:0000387
Down	3.42638	2.12789	0.295943	0.1799	RP11-361D15.2		
Up	0.636364	2.12131	4.48441	2.64233	MAFB	v-maf avia	GO:0000978
Up	0.0892948	0.37952	1.14011	0.598941	ERF	Ets2 repre	GO:0000122
Up	0.256977	0.634515	0.759215	0.77037	ABCC10	ATP-binding cassette	
Down	0.468181	0.391654	0.153689	0.164492	GMPR	guanosine	GO:0003920
Up	0.0185998	0.0175672	0.154411	0.126242	SOX13	SRY (sex c	GO:0000977
Up	0.0155027	0.0370932	0.0584307	0.0612346	TMCC1	transmembr	GO:0005789
Up	0.503326	0.325978	1.49066	1.30632	HNRNPUL2	heterogene	GO:0003723
Down	0.0086375	0.003415	0	0	SOX5	SRY (sex c	GO:0000981
Down	0.402728	0.626513	0.177659	0.126042	PDE4DIP	phosphodiesterase 4D	
Down	0.261961	0.374019	0.0034321	0.0026079	LMO3	LIM domain	GO:0005737
Up	0.0923439	0.199565	0.25066	0.292449	ATP10D	ATPase, cl	GO:0000287
Down	1.31372	1.49662	0.355807	0.447722	GSTM4	glutathion	GO:0004364
Up	0.444379	0.572474	1.63379	1.4853	NUAK1	NUAK famil	GO:0001650
Up	0.282088	0.201301	1.9288	1.21401	CA12	carbonic a	GO:0004089
Up	0.202513	0.285079	0.464393	0.448045	TGFBRAP1	transforming growth	
Down	0.0324522	0.0438755	0.0102827	0.0164161	FGGY	FGGY carbo	GO:0005623
Up	0.020828	0.022289	0.0790194	0.100044	PLXDC2	plexin dom	GO:0016021
Down	0.511678	0.396171	0.158497	0.0990564	TTC8	tetratrico	GO:0001103
Down	45.8551	46.6323	12.6327	13.5987	CTD-2033A16.1		GO:0005829
Down	1.08044	1.1768	0.69133	0.568523	PDCD6IP	programmed	GO:0000281
Down	0.617003	0.371968	0	0	NUDT16P1	nudix (nuc	GO:0000287
Down	17.617	11.8254	8.14737	7.34445	AC021224.1		GO:0003723
Up	5.10806	4.43065	12.7514	10.5009	UBE2M	ubiquitin-	GO:0004842
Up	0.133717	0.100786	0.50171	0.438036	PTGIS	prostaglandin I2 (pr	
Up	0.891266	1.53895	2.2614	3.15585	SLC1A4	solute car	GO:0005254
Up	0.0717407	0.939267	0.957703	1.46018	TELO2	telomere m	GO:0000723
Down	0.595698	0.413224	0.176489	0.197217	FAM171B	family wit	GO:0016021
Up	1.36972	2.99899	8.79333	8.97855	TSTD1	thiosulfat	GO:0005737
Up	0.862965	0.680452	1.56441	1.90058	ELK3	ELK3, ETS-	GO:0000978
Up	0.0180488	0.0393388	0.243357	0.320613	ADAMTS10	ADAM metal	GO:0001527
Up	0.473581	1.51391	2.1591	2.08859	ATHL1	ATH1, acid	GO:0004555
Down	3.88843	9.30804	0.50725	1.09208	AC005540.3		GO:0001967
Up	0.682336	0.65962	2.17642	1.873	EML3	echinoderm	GO:0000226
Down	0.930913	1.49136	0.309575	0.0903299	GABRE	gamma-amir	GO:0004890
Down	2.67129	3.71016	1.88422	1.04266	STAT2	signal transducer ar	
Up	0.336071	0.379992	0.901948	0.976019	UBP1	upstream b	GO:0000981
Down	1.89352	1.92506	1.05705	0.965965	IMPACT	impact RWD	GO:0000122
Down	2.87081	4.04289	1.03444	0.815459	GLRX	glutaredoxin (thiolt	
Up	0.471893	0.376644	2.03536	1.98505	RAB3B	RAB3B, mem	GO:0003924
Down	4.23845	5.21426	2.89064	2.5066	SSR1	signal sec	GO:0005783
Up	20.7006	16.569	48.5654	43.3579	H2AFJ	H2A histor	GO:0000786
Down	0.0077058	0.0051374	0	0	C1orf101	chromosome	GO:0036128
Down	0.891079	0.695463	0.321851	0.453335	FBXO8	F-box prot	GO:0000151
Down	0.0179251	0.0433208	0.0023269	0	ADD2	adducin 2	GO:0003779
Down	0.0109534	0.0225716	0.0016743	0.0006361	TSPAN7	tetraspani	GO:0005887
Up	0.179963	0.246168	0.905148	0.963298	STX1A	syntaxin 1	GO:0000149
Up	1.10911	1.75161	2.43038	4.31199	SOWAHC	sosondowah ankyrin r	

Down	2.09056	1.48954	0.986669	1.06407	PRIM1	primase, I	GO:000008
Down	0.257784	0.37298	0.113627	0.0638173	GYG2	glycogenir	GO:000582
Up	0.529888	0.460243	0.980458	0.963604	SYNJ2	synaptojar	GO:000372
Down	0.342547	0.218587	0.122147	0.14991	SUCLG2	succinate-	GO:000028
Down	2.98756	3.16986	1.82424	1.56894	ARL5A	ADP-ribosy	GO:000552
Down	0.830695	0.606634	0.413973	0.335077	ZDHHC13	zinc finge	GO:000013
Up	0.940475	1.36543	3.35207	2.12011	TMEM8A	transmembr	GO:000462
Down	4.30269	3.88107	0.709529	0.761144	UBE2L6	ubiquitin-	GO:000015
Down	1.14671	1.91223	0.569761	0.591867	PCOLCE-AS1	PCOLCE ant	GO:000520
Down	10.7331	7.409	4.66552	4.13874	HSPA13	heat shock	GO:000552
Down	8.08159	5.18073	0.276682	0.42924	S1PR1	sphingosir	GO:000152
Up	0.0913158	0.105412	0.159222	0.207946	HERC1	HECT and R	GO:000484
Up	0.423793	0	2.99272	4.02021	CTB-174D11.3		
Down	18.9508	21.0542	7.78425	7.43278	PNRC1	proline-ri	GO:000563
Down	0.102696	0.108733	0.0261204	0.025071	MID1	midline 1	GO:000022
Up	1.05321	3.10857	11.5381	9.01968	FZD7	frizzled c	GO:000493
Up	0.314757	0.499436	1.30189	0.957085	SLC25A28	solute car	GO:000538
Down	2.38685	1.58195	0.727927	0.895213	HPRT1	hypoxanthine phospho	
Up	0.516371	0.311301	1.75259	2.04233	RP11-366L20.2		GO:001602
Down	0.968341	0.595696	0.428397	0.388892	LRRC40	leucine ri	GO:000573
Up	0.0193557	0.0340115	0.0662235	0.0687237	CHSY3	chondroitin sulfate	
Down	1.1489	1.36915	0.574632	0.401475	GSTM2	glutathione S-transf	
Up	0.294028	1.24967	1.55263	1.20033	EGFL7	EGF-like-d	GO:000152
Up	1.22955	1.37531	2.25388	2.54519	HAGH	hydroxyacy	GO:000441
Down	7.36426	9.39017	0.610065	0.761411	CLU	clusterin	
Down	1.14113	1.07344	0.410481	0.452003	ANKMY2	ankyrin re	GO:000592
Up	1.07745	3.6971	7.32948	5.0958	C9orf69	chromosome	GO:000028
Up	0	0	0.0229829	0.0344037	UTY	ubiquitous	GO:000097
Up	1.95485	4.64145	7.57796	7.60966	MGAT4B	mannosyl (	GO:000013
Up	0.432269	0.183395	1.21847	0.98124	SAMD9L	sterile al	GO:000573
Up	0.21958	0.221595	1.22138	1.41518	DVL1	dishevelled segment	
Down	0.235983	0.20951	0.116057	0.0693436	HSPBAP1	HSPB (heat shock 27k	
Down	0.484095	0.518236	0.224293	0.250824	TTI1	TELO2 inte	GO:000573
Up	0.165544	0	0.674443	0.984293	CH507-9B2.	Protein LO	GO:000013
Down	0.940946	0.818317	0.410875	0.372817	MBNL2	muscleblir	GO:000038
Up	0.0948726	0	0.502476	0.672716	ZNF528-AS1	ZNF528 ant	GO:001602
Up	1.7499	2.69969	3.60842	3.7205	RIT1	Ras-like w	GO:000392
Up	0.217416	0.567504	1.17643	0.98357	GPSM1	G-protein	GO:000013
Up	2.54037	5.34	11.5714	11.3695	QSOX1	quiescin Q	GO:000257
Up	0.0212547	0	0.443734	0.40959	ART4	ADP-ribosyltransfera	
Down	1.95679	1.4076	0.788211	1.11182	PGM2	phosphoglu	GO:000028
Down	0.220292	0.174598	0.100045	0.07233	GABPB2	GA binding	GO:000563
Up	0.116379	0.133685	0.452172	0.423457	CAPN15	calpain 15	GO:000370
Down	2.00159	0.988519	0.441183	0.453279	TOR1AIP1	torsin A i	GO:000167
Up	0.109163	0.268803	0.390029	0.354272	FBXO31	F-box protein 31	
Up	0.17521	0.214121	1.06246	0.82338	FAT1	FAT atypical cadheri	
Up	0.0518333	0.231896	0.338857	0.324143	MICALL2	MICAL-like	GO:000172
Down	0.881124	1.38025	0.234871	0.343211	KIAA1919	KIAA1919	GO:000535
Up	0.151213	0.360804	0.746008	0.599908	ZNF212	zinc finge	GO:000098
Up	0.201742	0.285813	1.37498	1.03559	PLCB3	phospholip	GO:000307
Down	0.0299301	0.027725	0	0	EYA2	EYA transc	GO:000028

Down	0.126273	0.185025	0.0570465	0.0633249	SIMC1	SUMO-inte	GO:003218
Up	0.0565341	0.06138	0.262178	0.225292	IGF2BP3	insulin-li	GO:000372
Down	0.585921	0.457957	0.352167	0.326342	TUSC3	tumor sup	GO:000573
Down	0.135395	0.472166	0	0	MCHR1	melanin-co	GO:000493
Up	0.501275	1.21743	1.58904	1.58264	EHD1	EH-domain	GO:000550
Down	6.14891	5.04611	3.11449	3.82136	NCOA4	nuclear receptor coa	
Down	0.22399	0.131723	0.0515174	0.0614189	CARD16	caspase re	GO:000486
Down	0.803374	0.624123	0.395347	0.444139	GPR89A	G protein-	GO:000830
Up	0	0	0.0799607	0.0954785	NANOS3	nanos homo	GO:000093
Down	0.0172813	0.0222572	0.0007924	0.0037634	RASGRF2	Ras protei	GO:000016
Down	0.38953	0.361328	0.161907	0.0752756	SLC44A1	solute car	GO:000565
Up	4.2188	9.4224	16.6799	17.7394	RRS1	ribosome b	GO:000002
Down	0.399912	0.389784	0.14277	0.288322	TBL1XR1	transducin	GO:000011
Down	0.469059	0.984487	0.0584194	0.127623	PTHLH	parathyroid hormone-	
Up	0.168343	0.17612	0.989575	1.10185	C2CD2L	C2CD2-like	GO:000554
Down	3.74224	2.93093	2.2088	1.88763	POLR3C	polymerase	GO:000369
Down	33.3491	34.0922	14.0544	16.0613	CTSB	cathepsin B	
Up	0.158381	0.195317	0.436182	0.448208	INPP5F	inositol p	GO:0001921
Up	0	0	1.51155	1.37063	AC147651.4		
Down	0.0377026	0.0348003	0.0126064	0.0157371	DTNB	dystrobrev	GO:0005737
Up	0.775123	0.979117	1.80099	1.51431	DGKA	diacylglyc	GO:0003951
Up	0.153872	0.249138	0.447891	0.299872	MBD2	methyl-CpG binding d	
Down	0.387343	0.281807	0.162061	0.198638	RP11-423H2.1		GO:000034
Up	0.0829906	0.0489112	0.249773	0.236234	SEMA3D	sema domai	GO:000175
Up	1.71666	2.3308	3.54852	3.17875	EIF3B	eukaryotic	GO:000173
Up	6.9512	6.22217	13.795	12.7025	RP11-1100L3.7		GO:000004
Down	2.25114	4.07056	1.08133	0.907282	SDC1	syndecan 1	
Down	0.127954	0.114653	0.0358564	0.0403448	DHX35	DEAH (Asp-	GO:000039
Up	0.0402382	0.104241	0.305174	0.256126	FAM129B	family wit	GO:000371
Up	0.145234	0.51794	0.715209	0.742608	POLD1	polymerase (DNA dire	
Down	1.116	0.880266	0.475711	0.514747	MBTPS2	membrane-b	GO:000079
Down	3.99548	2.74097	1.05099	1.58212	LXN	latexin	GO:0005737
Down	1.33527	0.453106	0.161324	0.0919377	NUDT10	nudix (nuc	GO:000029
Up	0	0.0701314	0.397953	0.554379	CLEC18B	C-type lec	GO:000561
Up	0.213244	0.473657	0.578352	0.590405	QSOX2	quiescin Q	GO:000375
Up	0	0.0800711	0.130664	0.173751	STMN3	stathmin-l	GO:000579
Down	1.26819	4.26189	0.241743	0.0706505	ODF3B	outer dense fiber of	
Down	4.49773	3.46314	0.629105	1.52423	CTNNB1	catenin (cadherin-as	
Up	9.99581	19.2538	23.5459	21.3076	MFSD10	major faci	GO:000691
Down	15.2691	17.9175	6.32099	4.35972	HIST2H2AA3	histone cl	GO:000078
Up	0	0	1.06832	0.854503	RP11-61J19.5		
Down	0.220926	0.166333	0.0636785	0.0756044	TRIQK	triple Qxx	GO:000582
Up	0.318641	0.545431	1.34061	0.954399	BRI3	brain prot	GO:000588
Up	0.0186762	0.0396886	0.0922421	0.105882	KLF13	Kruppel-li	GO:000097
Down	4.42536	2.59832	1.71895	1.82599	TCTN3	tectonic f	GO:000563
Up	0.0012258	0	0.0109492	0.0152694	DISC1	disrupted	GO:000022
Up	0	0	0.0869899	0.0412054	HKDC1	hexokinase	GO:000167
Up	18.9214	21.3743	55.5057	65.1117	FN1	fibronectin 1	
Down	2.41457	2.97802	0.0090053	0.0104654	CRISPLD2	cysteine-r	GO:000553
Down	1.59744	1.29209	0.88536	0.131886	LRTOMT	leucine ri	GO:000578
Up	0.0143831	0.0092349	0.0961376	0.0841175	NFIA	nuclear fa	GO:000012

Up	0.324523	0.145014	0.753928	0.585141	ARL10	ADP-ribosy	GO:0005525
Down	4.01105	3.26869	1.57759	1.45971	ERLIN1	ER lipid r	GO:0005783
Down	2.75167	1.7092	1.14623	0.965988	KCTD6	potassium	GO:0005829
Down	0.0412234	0.0337748	0.0050105	0.0038073	SLC16A9	solute car	GO:0005887
Down	14.0728	20.9307	3.64887	8.74374	CHMP4A	charged mu	GO:0000815
Down	0.776708	0.555334	0	0.0286918	WNT2	wingless-t	GO:0001938
Up	0.0668947	0	0.881053	1.29622	HHEX	hematopoie	GO:0000122
Up	0.0467281	0.0478118	0.189871	0.174126	AXIN1	axin 1	
Down	0.8567	1.69355	0.125619	0.0763624	AC104654.2		
Up	3.71192	0.842809	9.29915	10.225	EDN1	endothelin 1	
Down	0.553467	0.680194	0.0840889	0.0255584	RAB39B	RAB39B, me	GO:0003924
Down	14.1274	12.8002	6.58403	7.13074	ADIPOR1	adiponectin receptor	
Up	0.384881	0.235793	2.16438	2.31245	LINC00205	long intergenic non-	
Up	0.169792	0.174944	0.455558	0.356231	TOM1L2	target of	GO:0005622
Up	0.0674458	0.0816686	0.176281	0.207138	RGS12	regulator	GO:0000794
Up	0.446103	0.194464	1.70928	1.58653	TRIL	TLR4 inter	GO:0001530
Down	43.0705	73.5016	4.05322	4.55823	BAMBI	BMP and ac	GO:0005109
Up	0.074129	0	0.451814	0.366993	DUSP5	dual speci	GO:0000165
Up	0.0344904	0	0.536418	0.490225	NRGN	neurograni	GO:0005516
Down	1.44211	1.35574	0.471496	0.724322	LITAF	lipopolysaccharide-i	
Up	0.078582	0.174849	0.189677	0.251301	CCDC144B	coiled-coil domain c	
Up	0.0117352	0.0541296	0.148328	0.115847	EXTL3	exostosin-like glyco	
Down	12.939	9.94038	6.28124	5.01245	OAT	ornithine	GO:0004587
Down	0.322847	0.266131	0.0999709	0.190272	ATG5	autophagy	GO:0000045
Down	0.71339	0.480663	0.311328	0.288814	CLK4	CDC-like k	GO:0004674
Down	5.3224	3.97044	2.93573	2.51516	JKAMP	JNK1/MAPK8	GO:0005789
Up	4.14831	3.28331	10.3918	9.83813	NAP1L1	nucleosome	GO:0003723
Up	0.0061395	0.0018807	0.116968	0.104938	SLC24A3	solute car	GO:0005262
Up	0.35353	0.504172	0.820764	1.23387	MDC1	mediator c	GO:0005634
Up	2.14313	3.04447	4.12107	4.1926	ARF3	ADP-ribosy	GO:0005525
Up	0.0263695	0.058113	0.208753	0.181785	LZTS1	leucine zipper, puta	
Up	0.113876	0.371791	0.774734	0.460714	KMT2A	lysine (K)-specific	
Up	0.526979	1.08594	1.42244	1.99896	C14orf80	chromosome 14 open r	
Up	0.374515	0.570181	1.59657	1.35511	CACNB3	calcium ch	GO:0005245
Down	2.48611	2.48621	0	0.14013	RPL9P25	ribosomal	GO:0000184
Up	0.187544	0.184194	0.490212	0.480255	PIK3C2A	phosphatid	GO:0005524
Up	0.0056104	0	0.0274671	0.0317811	DGKG	diacylglyc	GO:0003951
Up	0.15927	0	2.09974	1.13767	FOXH1	forkhead b	GO:0000122
Down	0.0184448	0.0068168	0.0020226	0	AIM1	absent in	GO:0030246
Down	2.22372	3.7169	0.137851	0.0606432	PTGES	prostaglan	GO:0001516
Down	4.593	3.89842	2.35417	2.56074	SLC25A32	solute car	GO:0005739
Up	0.404992	0.441726	1.85906	1.95576	VCAN-AS1	VCAN antisense RNA 1	
Down	0.312632	0.512848	0.116715	0.0827748	MATN3	matrilin 3	GO:0001501
Up	0.0548205	0.117231	0.371086	0.379103	HS6ST1	heparan su	GO:0000139
Up	14.5592	19.4617	95.1199	89.1437	CCND1	cyclin D1	
Up	0	0.102409	0.182309	0.316021	FAM131B	family wit	GO:0005654
Up	2.37483	5.80919	41.5384	36.1964	FOXC2	forkhead box C2	
Down	0.386259	0.469049	0.238571	0.197227	GALNT11	polypeptide N-acetyl	
Up	0.174877	0.393259	0.577566	0.656975	ZBED3	zinc finge	GO:0000981
Up	0.159988	0.174001	0.590982	0.692178	PLXNB1	plexin B1	
Down	5.13426	4.30013	2.22321	2.20843	MPHOSPH6	M-phase ph	GO:0000176

Down	0.0440801	0.0272507	0.0020213	0.0015359	GSAP	gamma-seci	GO:000154C
Up	0	0	0.0537554	0.149771	DUSP8	dual speci	GO:000018E
Down	0.385982	0.705528	0.0680748	0.0488177	BMPER	BMP bindir	GO:0001657
Down	0.440988	0.481701	0.229205	0.195537	OXCT1	3-oxoacid	GO:0005654
Up	0.674401	1.07107	2.63257	2.08271	DMWD	dystrophia	GO:0005634
Down	29.2036	36.9267	2.93964	1.30154	PPP1R3C	protein pł	GO:0004722
Up	0.0222954	0.0090247	0.123506	0.131996	CTD-2269F5.1		
Up	0	0	0.0086093	0.0081119	MAP2	microtubule-associat	
Up	0.0126186	0.0213596	0.0448439	0.0564517	STXBP5L	syntaxin k	GO:0005096
Down	0.409087	0.23047	0.129722	0.118438	CDC25C	cell division cycle	
Up	0.0047087	0	0.0742232	0.0582041	STK32B	serine/thi	GO:0004674
Up	0.361697	0.895126	1.31637	1.24813	UBQLN4	ubiquilin	GO:0005634
Down	4.24304	9.68649	0	0	NPY	neuropeptide Y	
Up	0.0015875	0	1.59108	1.62305	NTN4	netrin 4	GO:0005604
Down	0.0936544	0.0611145	0.0343566	0.0087021	XYLB	xylulokina	GO:0004856
Down	0.0879164	0.190494	0.013043	0	LINC01341	long inter	GO:0003777
Up	0.0734987	0.151458	0.547678	0.422562	ESPL1	extra spir	GO:000007C
Down	3.44882	3.90509	1.93507	2.00939	SDHA	succinate	GO:0005739
Down	3.34298	3.17881	1.64337	1.7428	SLC35B3	solute car	GO:0000139
Up	0.0705302	0.17986	0.492138	0.529396	FAM83G	family wit	GO:0005634
Up	0.728	0.98843	1.24989	1.44356	DDX46	DEAD (Asp-	GO:000165C
Down	0.738416	0.767046	0.359804	0.35256	GPCPD1	glyceropho	GO:0005829
Down	1.50871	2.26937	0.0939517	0.303409	RASL11B	RAS-like, family 11,	
Up	0.67421	0.489832	3.07379	3.52833	RP1-228H13.5		
Down	0.633448	0.295044	0.114895	0.103934	PM20D2	peptidase	GO:0005654
Up	0	0	0.0909366	0.061247	RP11-1L9.1		
Down	25.6912	18.8853	12.9569	14.0511	YWHAQ	tyrosine 3	GO:0005737
Up	0.709303	2.08353	4.27942	3.77242	SCARF2	scavenger	GO:0005925
Up	0	0	0.0354639	0.0485058	KIT	v-kit Hardy-Zuckerma	
Down	4.89136	4.70022	2.9361	2.85162	TMEM9	transmembr	GO:0005764
Up	0.0514266	0	1.11867	0.689882	LL22NC03-2H8.5		
Down	1.88584	2.46487	0.496164	0.580251	BCKDHA	branched c	GO:0003826
Up	0.0033496	0.0119884	0.0316792	0.0417245	DOCK4	dedicator	GO:0005085
Down	2.91529	2.27208	1.60193	1.20591	CAPZA2	capping pr	GO:0003779
Up	0.610831	1.10411	2.01201	1.71554	ANKRD9	ankyrin re	GO:0005829
Down	1.65223	1.322	0.989286	0.777069	RECK	reversion-	GO:0001944
Down	13.3309	8.63876	5.71994	5.16913	GLO1	glyoxalase	GO:0004462
Up	1.53145	2.44723	5.52134	4.57477	MIDN	midnolin	GO:0005634
Down	7.48667	4.96214	0	0	PF4V1	platelet f	GO:0005615
Up	0.0776124	0.223639	0.270391	0.327727	RDH13	retinol de	GO:0005743
Down	4.31895	2.86857	0.324763	0.141825	PTGER2	prostaglandin E rece	
Up	0.154583	0.0805928	0.5002	0.483768	VEPH1	ventricula	GO:0005886
Up	0.50831	0.572961	1.70301	1.35899	CHSY1	chondroitin sulfate	
Up	0.376197	0.375163	0.73147	0.832717	NBPF15	neuroblast	GO:0005737
Down	3.75641	3.23487	1.29371	1.00887	MKRN2	makorin ri	GO:0003723
Up	0.166906	0.0121887	1.55902	1.56386	MAP3K7CL	MAP3K7 C-t	GO:0005634
Down	1.05025	0.472662	0.225384	0.259337	CDC7	cell division cycle	
Up	0.0758969	0	0.292162	0.222768	PPP2R3B	protein pł	GO:0000159
Down	0.76349	1.41016	0.263874	0.0975441	HIST1H2AC	histone cl	GO:0000786
Down	0.898164	1.02156	0.238745	0.233059	SOD2	superoxide	GO:0003964
Down	5.99989	4.15385	3.5052	2.69503	OTUD6B-AS1	OTUD6B ant	GO:000016C

Up	0.41294	0.390016	1.68235	1.18197	RP11-421F16.3		GO:0016021
Up	2.66141	5.28881	5.77183	5.95184	PRPF38B	pre-mRNA p	GO:0005681
Up	0.0031807	0.0096131	0.0346274	0.0163561	HECW2	HECT, C2 a	GO:0000209
Up	0.10694	1.25533	1.90242	1.91401	ANGPTL4	angiopoiet	GO:0001525
Up	0.0504	0.225484	0.254224	0.29654	SYMPK	symplekin	GO:0000398
Up	0.0488782	0.0421022	0.0986189	0.128142	DGKH	diacylglyc	GO:0003951
Up	1.09156	1.17824	8.53204	3.91812	CYTL1	cytokine-l	GO:0002062
Down	11.1655	10.1757	6.25442	5.78291	STT3A	STT3A, sub	GO:0004576
Up	0.641468	0.562795	1.80567	1.52994	LUZP1	leucine zi	GO:0003281
Down	0.802889	0.77117	0.443788	0.374525	CNOT2	CCR4-NOT t	GO:0000122
Up	0.173334	0.675311	1.63935	1.97751	GTPBP3	GTP bindin	GO:0002098
Up	0.0952851	0.220898	0.813792	0.796825	ZNF646	zinc finge	GO:0000981
Up	8.5316	12.2169	26.6073	18.6795	CH507-513H4.5		GO:0005524
Up	0	0	0.296395	0.422285	FOXF2	forkhead b	GO:0000977
Up	0	0	0.0105331	0.0132475	PPARG	peroxisome prolifera	
Up	9.74327	23.732	26.1102	27.1495	LRP10	low densit	GO:0005041
Up	0.025091	0.0097223	0.0695009	0.0637021	SYNDIG1	synapse di	GO:0005887
Up	2.30433	5.60199	5.76006	5.85975	CNPPD1	cyclin Pas	GO:0000079
Down	0.138176	0.113361	0.0280284	0.0486298	NSFP1	N-ethylmal	GO:0000139
Up	0.398977	0.46004	0.905461	0.79355	CTTNBP2NL	CTTNBP2 N-	GO:0005737
Down	2.25566	1.96655	0	0	HIST1H2BL	histone cl	GO:0000786
Down	1.98132	1.41075	0.866072	1.08186	SMN2	survival of motor ne	
Up	0.11136	0.268539	0.334637	0.443851	PHRF1	PHD and ri	GO:0006366
Down	0.432129	0.372468	0.171952	0.17719	UBL3	ubiquitin-	GO:0005622
Up	0.611923	0.614531	1.63854	1.323	TEAD2	TEA domain	GO:0000981
Down	0.287605	0.234676	0.0321361	0.0627916	ZFHX4	zinc finge	GO:0000122
Up	0.0301699	0.0240803	0.0940412	0.10505	KHDRBS3	KH domain	GO:0003723
Up	0.457449	0.845683	3.90269	3.15897	LIMS2	LIM and se	GO:0005634
Down	0.0445417	0.0678761	0.0132161	0.0095044	SAMD4A	sterile al	GO:0000289
Down	1.80442	2.13806	0.599885	1.29807	DNAJA3	DnaJ (Hsp40) homolog	
Up	0.0496783	0	0.258654	0.373224	FNIP2	folliculin	GO:0000122
Down	0.176693	0.226101	0.0703356	0.0809482	EXOC2	exocyst co	GO:0000145
Up	1.10325	1.6461	62.4927	56.7458	CRYAB	crystallin, alpha B	
Up	0.0553567	0.0908083	0.363728	0.454356	GPR161	G protein-	GO:0004930
Up	0.551908	0.990734	1.28531	1.19893	ACIN1	apoptotic	GO:0003676
Up	0.444171	0.701323	1.82785	2.15484	PYGB	phosphoryl	GO:0005576
Up	0.62463	0.819783	2.28028	2.1669	SORT1	sortilin 1	GO:0001503
Down	8.05075	9.93743	3.53132	4.37752	ASNS	asparagine synthetas	
Down	0.642011	0.374752	0.0729677	0.0686466	ADPGK-AS1	ADPGK anti	GO:0005576
Up	3.10054	3.50194	6.15298	5.44848	FPGS	folylpolyg	GO:0001889
Down	3.32059	2.92355	1.16782	1.16042	KLF3	Kruppel-li	GO:0000122
Up	2.64773	2.79231	9.96043	7.8615	NKIRAS2	NFKB inhib	GO:0003924
Down	102.659	96.3398	1.57267	2.51582	CTC-575D19.1		GO:0000027
Up	0	0	0.0297343	0.0267308	AC079135.1		GO:0000979
Up	11.428	14.8989	32.1183	30.3607	BTG1	B-cell tra	GO:0005634
Up	216.167	334.353	463.888	441.706	MALAT1	metastasis associate	
Up	0.157941	0.309196	0.713386	0.536571	FANCE	Fanconi anemia, comp	
Down	0.276607	0.237654	0.0112519	0.0085499	MX2	MX dynamin	GO:0000266
Up	0.129934	0	1.58389	1.36954	RP11-295D4.3		
Up	0	0	0.0012174	0.0021739	ADGRL3	adhesion G	GO:0001764
Down	52.273	55.4186	8.48492	6.40114	HSPB6	heat shock	GO:0005212

Up	0.0270629	0.103975	0.109901	0.137571	PHKA2	phosphoryl GO:0004689
Up	0	0.0107018	0.0415426	0.0243284	SATB2	SATB homec GO:0000118
Up	0	0	0.0174727	0.0419835	FANK1	fibronecti GO:0005634
Up	0.050932	0.156052	0.27473	0.41191	ABL1	ABL proto-oncogene 1
Down	0.400842	0.367016	0	0.0201269	LRIG3	leucine-ri GO:0005615
Up	2.85243	2.43477	7.97365	7.85725	FHL2	four and a half LIM
Up	1.42422	1.39089	2.89106	3.37377	MXRA7	matrix-rem GO:0000978
Down	1.03722	0.792704	0.608005	0.279481	BORA	bora, aurc GO:0000086
Down	0.883954	0.715613	0.521476	0.470585	DTD1	D-tyrosyl- GO:0000049
Down	66.5491	42.5371	11.9474	6.88552	AC002398.12	
Down	1.33711	0.964382	0.556753	0.641862	MFSD1	major faci GO:0016021
Down	0.561046	0.373323	0.16067	0.172032	DCP1B	decapping GO:0000184
Up	0.0368537	0.114772	0.145282	0.1333	BCOR	BCL6 corep GO:0000122
Up	1.02291	0	4.01306	7.40562	RPL7L1P9	ribosomal GO:0005840
Up	359.104	335.553	671.351	650.654	VIM	vimentin GO:0001701
Up	0.405382	0.712899	0.992562	0.905487	LRRFIP1	leucine ri GO:0000122
Down	2.94149	4.14909	1.00427	0.4554	MIR503HG	MIR503 host gene
Up	0.0059509	0.0112411	0.0322057	0.0323124	OBSCN	obscurin, GO:0004674
Up	0.203825	0.248755	0.401027	0.414143	RNF213	ring finger protein
Up	0	0	1.01506	0.741637	RP11-147L13.13	
Up	0	0	0.290153	0.197268	CD93	CD93 molec GO:0001849
Down	0.489126	0.585598	0.0521241	0.0165029	ATG9B	autophagy GO:0000045
Up	0	0	0.0422265	0.0379201	ASB18	ankyrin re GO:0005829
Up	0.875296	1.35659	2.40303	2.02201	AP2A1	adaptor-re GO:0005829
Up	0.181574	0.685975	1.67911	2.69677	MIR210HG	MIR210 hos GO:0003677
Down	14.2321	13.3548	6.31008	6.09134	NARS	asparaginy GO:0003676
Up	0	0	0.232677	0.171099	KLHL30	kelch-like family me
Down	0.234971	0.197269	0.0121936	0.0083389	GCNT1	glucosaminyl (N-acet
Down	0.103727	0.0783748	0.0630231	0.0012048	SLC9B1	solute car GO:0005886
Up	1.64966	2.41761	3.1336	3.09457	CRK	v-crk avian sarcoma
Up	0.0684874	0.0952201	0.273845	0.36481	NEDD9	neural pre GO:0000922
Up	0.0002176	0	0.0053496	0.0106314	PPP1R9A	protein ph GO:0005737
Down	133.697	116.254	84.5983	80.5784	ANXA2	annexin A2
Up	0	0	0.0318962	0.0391515	UNC13A	unc-13 hom GO:0001956
Up	0.14861	0.33814	0.465664	0.517814	NCKAP5L	NCK-associ GO:0001578
Up	0.928036	1.61072	2.77671	2.05742	GBA2	glucosidas GO:0000139
Up	2.4949	5.22301	7.49487	8.62659	GNAS	GNAS compl GO:0001726
Up	0.0037333	0.0016528	0.0813561	0.0597513	SUGCT	succinyl-C GO:0005739
Up	0.543205	0.362841	1.79584	1.88064	STK38L	serine/thr GO:0000287
Up	0.047664	0.0732495	0.170129	0.185784	IFFO2	intermedia GO:0005882
Up	0.469758	1.6638	5.62759	4.38872	KRTAP7-1	keratin as GO:0005882
Up	0.19591	0.392401	1.27358	1.1587	NUMBL	numb homol GO:0005737
Down	2.4711	2.8007	1.44661	1.04046	CLK1	CDC-like k GO:0004674
Up	0.289755	0.621238	1.01865	1.09658	PTPRF	protein ty GO:0004725
Up	0.0062315	0	0.129659	0.132857	CACNG8	calcium ch GO:0005245
Down	1.61865	1.84597	0.768137	0.893877	RFT1	RFT1 homol GO:0005319
Up	2.9161	4.13469	12.3969	14.9111	HTRA1	HtrA serine peptidas
Down	0.176909	0.215442	0.0856551	0.0723718	LMLN	leishmanol GO:0004222
Up	0.118591	0	10.9269	12.3104	PPP1R14A	protein ph GO:0004865
Down	0.596779	0.581436	0.194076	0.360277	HMG20A	high mobil GO:0000122
Up	1.09932	2.50577	2.65794	2.71299	YBX3	Y box binc GO:0003677

Up	2.78337	2.73338	8.14256	7.17331	SPOCD1	SPOC domai	GO:0006351
Down	48.2506	15.6561	4.73619	3.39136	DIRAS3	DIRAS family, GTP-bi	
Up	0.0001098	0.0037317	0.0085807	0.0145126	ADAMTSL3	ADAMTS-lik	GO:0005576
Up	0.122805	0.118369	0.277479	0.315405	NBPF26	neuroblast	GO:0003723
Up	2.64383	2.02508	6.31062	5.58644	WSB2	WD repeat	GO:0005622
Down	0.0986905	0.0512868	0.0260859	0.0326232	KIAA1467	KIAA1467	GO:0016021
Up	0	0.0308059	0.0670274	0.11286	SYCP2	synaptonem	GO:0000795
Down	4.81756	6.04392	1.24558	1.01324	AARSD1	alanyl-tRN	GO:0002196
Up	0.121495	0.1683	0.434091	0.391938	VPS37C	vacuolar p	GO:0000813
Down	0.808882	0.98706	0.41684	0.510936	HBS1L	HBS1-like	GO:0003746
Up	0.0219675	0	0.0804843	0.0947221	FIGN	fidgetin	GO:0005524
Up	1.46569	1.24328	3.11143	3.03521	DCBLD2	discoidin,	GO:0005887
Up	2.34164	4.07575	7.94325	9.0264	ZNF428	zinc finge	GO:0003676
Up	0.204033	0.112804	0.608715	0.629434	LSM11	LSM11, U7	GO:0005634
Up	0.0238315	0.0598082	0.0682613	0.0923205	STK32C	serine/thr	GO:0004674
Down	0.0150195	0.0180919	0.0049409	0.0025724	TRHDE	thyrotropi	GO:0004177
Up	0.277193	0.35712	0.749667	0.557397	YLPM1	YLP motif	GO:0003723
Up	0.101608	0.0569109	0.370238	0.247366	FMNL3	formin-lik	GO:0001525
Up	0.0141495	0	0.0872322	0.22145	C19orf68	chromosome	GO:0005634
Up	0.0037191	0.0037636	0.023617	0.017182	SLCO3A1	solute car	GO:0005886
Down	49.3019	40.8863	21.5955	23.174	HNRNPK	heterogeneous nuclea	
Down	5.46532	4.04905	2.71017	2.7711	TNFAIP6	tumor necr	GO:0005540
Up	0.0213572	0.0204819	0.0633939	0.0634404	COL4A5	collagen, type IV, a	
Up	0.0252343	0.0178751	0.251918	0.2287	RP11-1223D19.3		GO:0000139
Down	0.0606458	0.0241824	0.0005065	0.0009621	DPYD	dihydropyr	GO:0002058
Up	0.0231983	0.117625	0.455742	0.387249	TNFRSF10A	tumor necr	GO:0002020
Up	0.0002407	0.0034101	0.0037183	0.0065157	CASC15	cancer sus	GO:0005829
Up	0.0646886	0.0799821	0.605132	0.25921	AGRN	agrin	
Up	4.26284	15.4853	26.3265	26.0791	ARMCX7P	armadillo	GO:0005741
Up	0.227419	0.276988	0.814546	0.878488	HPCAL1	hippocalci	GO:0005509
Down	0.0056156	0.0053368	0.0002639	0.0003008	ZFPM2	zinc finge	GO:0000122
Down	7.81906	5.80283	4.04353	4.21951	COPZ1	coatomer p	GO:0000139
Up	0.98313	1.35359	4.1558	2.81286	FAM214B	family wit	GO:0005634
Down	0.0661522	0.0878623	0.0175964	0.0074282	RASSF5	Ras association (Ral	
Down	3.23559	3.80682	0.30075	0.803657	EBLN3	endogenous Bornaviru	
Down	17.8665	15.785	9.04452	10.4067	TMBIM6	transmembr	GO:0005634
Up	0	0	0.0047413	0.0062715	FGF13	fibroblast growth fa	
Up	4.84562	12.7884	34.5678	36.8	KLF2	Kruppel-like factor	
Down	0.595302	1.32464	0.174676	0.114954	KIAA1462	KIAA1462	GO:0005911
Up	2.00565	3.87717	7.44159	7.15526	FLNC	filamin C,	GO:0005737
Up	0.0597879	0.0564688	0.170534	0.151634	GPD2	glycerol-3	GO:0004367
Down	0.0055042	0.003899	0	0	ASTN1	astrotacti	GO:0001764
Up	0.675498	0.978471	1.36458	1.44041	KDM1A	lysine (K)	GO:0000122
Down	1.34152	1.04345	0.165853	0.277256	CTD-2369P2.8		
Up	0.0755989	0.102776	0.140832	0.180001	MYO5A	myosin VA (heavy cha	
Up	0.223087	0	131.657	114.363	TRBC2	T cell rec	GO:0003823
Down	5.65272	6.4358	3.54793	2.1737	AKIRIN1	akirin 1	GO:0005634
Up	2.41522	6.02033	27.5941	29.7119	AEBP1	AE binding	GO:0000122
Up	0.444981	0.62819	1.02809	0.975561	CERK	ceramide k	GO:0000287
Up	0.033852	0.0418797	0.684817	0.583184	ADAM23	ADAM metallopeptidas	
Down	0.0506386	0.10522	0.0195326	0.0190283	PCDH7	protocadhe	GO:0002576

Down	0.0290197	0.0229641	0.0037913	0.0028809	C2orf88	chromosome	GO:0005886
Up	3.90425	3.37635	9.13077	7.6232	DUSP6	dual specificity pho	
Down	3.51844	4.4422	0.874617	1.02111	SLC38A5	solute car	GO:0003333
Down	0.0236551	0.0115562	0	0.0027356	C10orf107	chromosome	GO:0005509
Down	0.0261498	0.0208716	0	0.0035292	CAPS2	calcyphosi	GO:0005509
Down	4.14197	5.15646	0.55995	1.06022	VTN	vitronectin	
Up	0.007911	0	0.0607631	0.0851072	MAP3K5	mitogen-ac	GO:0000165
Up	0.256407	0.180575	0.485192	0.560152	HMGA2	high mobil	GO:0003677
Up	0.148315	0.109216	0.489236	0.512263	KIAA0355	KIAA0355	
Up	0	0	0.237461	0.427895	ITGA7	integrin,	GO:0005886
Up	3.33226	6.06128	16.8518	12.946	COL6A1	collagen,	GO:0001649
Down	19.1469	17.9332	12.6767	7.222	GPS2	G protein	GO:0000122
Down	41.9935	38.4226	22.3475	24.3858	GHITM	growth hor	GO:0005739
Down	0.563939	0.411728	0.226868	0.0298364	AC007743.1		GO:0003964
Down	0.733077	0.887998	0.143046	0.116203	FLRT2	fibronecti	GO:0003007
Down	0.337477	0.229494	0.0486363	0.134892	BIRC3	baculoviral IAP repe	
Down	0.0663626	0.0599282	0.0318893	0.0252594	CCNG2	cyclin G2	
Up	0	0	0.0330313	0.032868	FAM189A2	family wit	GO:0016021
Down	30.0177	21.7522	15.0534	16.227	SRSF6	serine/arg	GO:0000380
Down	0.253616	0.172466	0	0	DLK1	delta-like	GO:0005509
Up	0.149181	0.257539	0.484632	0.54812	PRKD2	protein ki	GO:0001525
Down	1.46728	1.24724	0.734144	0.718852	UBE2D1	ubiquitin-	GO:0000151
Down	1.55371	1.36563	0.523804	0.489168	ANKRA2	ankyrin re	GO:0005634
Up	1.05939	1.914	3.17689	2.81191	NUP62	nucleopori	GO:0000922
Down	0.004878	0.0207324	0	0	AC097662.2		GO:0016021
Up	0.097062	0.854012	1.30128	2.23703	PI16	peptidase	GO:0005615
Up	0	0	0.0011855	0.0027499	ATRNL1	attractin-	GO:0007186
Down	1.26293	1.96179	0.0970104	0.245715	HOXA13	homeobox A	GO:0000981
Up	0.380122	0.944789	2.60696	1.99691	TMEM132A	transmembr	GO:0000139
Down	1.22025	1.93767	0.582092	0.642986	CCDC28A	coiled-coil domain c	
Up	4.02176	6.33999	10.3697	10.9079	LOXL1	lysyl oxic	GO:0001669
Up	0.124624	0.186188	1.11365	1.24735	MASP1	mannan-bir	GO:0001867
Up	7.19084	7.68426	22.7474	21.9309	C6orf1	chromosome	GO:0016021
Up	3.03504	3.31593	8.10175	6.67158	NR2F2	nuclear re	GO:0000122
Up	0.0086174	0.0360947	0.0602398	0.0603139	MARK4	MAP/microt	GO:0000226
Down	0.480404	0.337888	0	0	TYRP1	tyrosinase-related p	
Down	3.09904	2.71928	0.428617	1.22613	NKX3-1	NK3 homeobox 1	
Up	0.0745943	0.1321	0.470328	0.589683	PLEKHH3	pleckstrin	GO:0005615
Up	0.626837	1.69611	2.48532	3.52402	FBLIM1	filamin bi	GO:0001650
Down	0.0562265	0.0964278	0.0158599	0.0030128	MITF	microphtha	GO:0000122
Down	0.953878	1.24316	0.266755	0.474836	GEMIN8	gem (nucle	GO:0000387
Up	4.3264	7.64177	21.8229	20.3241	H1FX	H1 histone	GO:0000786
Up	0.214321	0.0596092	1.70283	0.904608	SERPINB2	serpin pep	GO:0004867
Up	21.1434	29.8039	44.0116	39.9803	ADRM1	adhesion r	GO:0000502
Up	3.87243	4.73858	9.34675	11.7307	RNF167	ring finge	GO:0000209
Up	4.28186	7.88611	24.7704	19.1555	RP5-956018.3		
Up	0.891063	1.16529	2.56424	3.29488	XXbac-BPG283016.9		GO:0003964
Down	1.37652	1.04528	0.782858	0.74371	EIF3E	eukaryotic	GO:0000184
Down	0.0063607	0.0162459	0	0.0004292	DIRC3	disrupted in renal c	
Up	0.770662	1.18865	1.96892	2.21197	LRRC8A	leucine rich repeat	
Up	0.0965616	0.29995	0.293143	0.428361	NELFA	negative e	GO:0005654

Up	0.656137	1.9685	2.95271	2.82305	TBC1D10B	TBC1 domai	GO:000509(
Down	37.505	23.826	15.9188	13.3388	ZWINT	ZW10 inter	GO:000007(
Down	4.41535	3.21376	1.59794	1.62923	DLD	dihydrolip	GO:000414(
Up	0	0	0.314885	0.1823	ITIH3	inter-alph	GO:000257(
Up	0.18793	0.255596	1.15649	1.12127	PPP1R9B	protein phosphatase	
Up	2.64606	2.13262	7.59297	5.26251	HEXIM1	hexamethyl	GO:000012
Up	5.4074	4.92225	15.5337	18.7251	ISG15	ISG15 ubic	GO:000557(
Up	2.15761	2.19748	5.4667	4.60172	CTC-444N24.11		GO:0016021
Down	0.0420872	0.159003	0	0	RP11-462G12.2		GO:000565
Down	6.00552	5.82767	2.07673	2.32145	SDC4	syndecan 4	GO:000152
Down	2.044	2.0106	1.50984	0.873487	IDH3A	isocitrate	GO:000028
Down	1.37148	1.1821	0.294115	0.258111	HMGCR	3-hydroxy-	GO:000442(
Up	0.41482	0.65074	1.34035	1.39643	SPOCK1	sparc/oste	GO:000155
Up	0.237097	0.175596	0.503578	0.497077	SMG1	SMG1 phosp	GO:000018
Up	1.89041	2.53105	4.81139	5.17545	B4GALT2	UDP-Gal:betaGlcNAc b	
Up	0.339365	0.445879	0.648632	0.640437	CLIP1	CAP-GLY do	GO:000027
Down	9.05369	8.14672	4.47245	3.37469	SMIM3	small inte	GO:0016021
Down	0.0202189	0.0243124	0.0081727	0.0069099	PCDH9	protocadhe	GO:000550
Down	49.4461	34.5208	23.3769	20.9471	MAT2A	methionine	GO:000447
Up	79.5344	96.8906	145.341	226.291	RP11-498C9.3		
Up	0.0808647	0.144711	0.51523	0.263042	ABCD1	ATP-binding cassette	
Up	1.80051	1.4093	7.99065	7.68267	VEGFB	vascular e	GO:000152
Down	0.30353	0.172277	0.0353409	0.0327712	PKIA	protein ki	GO:000012
Down	5.14699	7.26143	0.254849	0.0226611	QPRT	quinolinat	GO:000451
Down	0.575917	0.351022	0.251072	0.173115	ATF1	activating transcrip	
Down	1.05108	0.941699	0.312037	0.264078	PXK	PX domain	GO:000377
Down	0.53062	0.406395	0.197943	0.256876	HIBADH	3-hydroxyi	GO:000573
Down	0.246662	0.196237	0.136377	0.0964812	FBXL4	F-box and	GO:0000151
Up	15.898	22.6152	33.6873	33.2094	KRT8	keratin 8, type II	
Down	3.02173	4.76974	1.37643	0.458825	MRPL34	mitochondr	GO:000373
Down	0.413086	0.547875	0.107138	0.154399	ZNF805	zinc finge	GO:0000981
Up	0.22423	0.242654	0.707724	0.695941	SH3PXD2B	SH3 and PX	GO:0001501
Up	0.0358848	0	0.588273	1.07281	EDN2	endothelin 2	
Down	0.867191	0.432705	0.151309	0.198591	PRELP	proline/ar	GO:0001501
Up	0.144831	0.229259	0.751973	0.703972	TGFB1	transforming growth	
Down	0.149041	0.0899096	0.0388017	0.0451472	KITLG	KIT ligand	
Down	0.0669433	0.0382618	0.0090577	0	PRUNE2	prune homo	GO:000430
Down	10.6982	7.3398	5.56607	5.67872	UNC50	unc-50 hom	GO:000372
Up	4.7956	7.82347	40.9879	33.698	RP11-265D17.2		
Down	8.09694	10.7964	3.79556	2.66224	NINJ1	ninjurin 1	GO:000195
Up	0.0079577	0.0128844	0.038706	0.056916	JADE3	jade famil	GO:000012
Down	1.85208	1.58403	0.896004	0.819442	EMC2	ER membran	GO:000563
Up	0.379335	0.450267	1.00519	1.04378	SCAF1	SR-related CTD-assoc	
Down	0.492771	0.927107	0.18642	0.0661047	CPNE7	copine VII	GO:000521
Up	0.017435	0	5.32796	5.54206	JAG1	jagged 1	
Down	7.06889	4.68074	3.59734	3.17528	MAD2L2	MAD2 mitotic arrest	
Up	7.7709	11.8527	15.7852	15.0336	PNN	pinin, des	GO:000039
Down	0.0830709	0.0938903	0	0	COLEC12	collectin	GO:000504
Down	0.428691	0.40952	0.0679601	0.0569216	STON1	stonin 1	GO:000573
Up	0.357845	0.671391	0.997024	1.06805	IDUA	iduronidas	GO:000394(
Up	0.186994	0.327062	2.32603	2.1413	HAPLN3	hyaluronan	GO:000554(

Up	28.016	46.6077	95.2524	103.206	HLA-C	major hist	GO:000013[illegible]
Up	0.033198	0.0358343	1.23398	1.99952	TRAF1	TNF recept	GO:000516[illegible]
Down	0.1001	0.0360165	0.0120219	0	DAW1	dynein ass	GO:000592[illegible]
Down	1.85549	1.71739	1.10796	0.84031	STX12	syntaxin 1	GO:000004[illegible]
Up	4.28654	2.32932	17.9935	16.4858	AC005606.15		GO:000372[illegible]
Down	11.4722	9.2874	2.95851	3.51587	MAT2B	methionine	GO:000562[illegible]
Up	0.643946	3.01203	3.89261	4.41718	CPTP	ceramide-1	GO:000554[illegible]
Up	0.0058115	0.0254968	0.0390065	0.0366452	STOX2	storkhead	GO:000189[illegible]
Up	0	0.010962	0.0223604	0.0247139	SEMA6A	sema domai	GO:000175[illegible]
Up	0	0	0.0466761	0.0324122	IGSF1	immunoglob	GO:000557[illegible]
Up	0.546619	0.577734	1.41963	1.26579	ZNF677	zinc finge	GO:0000981
Up	0	0	3.07044	1.71412	RP11-286H15.1		GO:000367[illegible]
Up	3.5615	3.35683	9.12612	9.37069	PPP1R18	protein ph	GO:000377[illegible]
Up	0.139815	0.414167	0.931641	0.637888	PPP1R12C	protein ph	GO:000485[illegible]
Up	0.865639	1.34241	1.87176	1.86297	WDR82	WD repeat	GO:000078[illegible]
Down	0.474732	1.14525	0.0961687	0.0931705	PODNL1	podocan-li	GO:006202[illegible]
Down	0.433063	0.419618	0.038199	0.0053752	LY96	lymphocyte	GO:000153[illegible]
Up	0.918672	2.45568	2.88525	3.04775	ADM2	adrenomedu	GO:000152[illegible]
Down	2.2264	2.27704	0.87955	1.44806	TUBE1	tubulin, e	GO:000022[illegible]
Up	0.403453	0.217746	1.64339	1.71511	RAB33A	RAB33A, me	GO:000013[illegible]
Down	4.13505	4.80512	0.392061	1.39748	ABHD4	abhydrolas	GO:000578[illegible]
Up	0.520028	0.702734	3.08716	3.23636	EPHA2	EPH receptor A2	
Up	0.512647	0.246662	1.62226	1.74708	ZNF629	zinc finge	GO:0000981
Up	0.726531	0.748579	7.68338	6.47119	IRF2BP1	interferon	GO:000097[illegible]
Up	0.361657	0.398184	0.798756	1.0327	BAZ1A	bromodomai	GO:0006261
Up	0.035855	0.121912	0.536241	0.67545	ZNF185	zinc finge	GO:000573[illegible]
Up	0.0723172	0.27321	0.595801	0.302588	ZKSCAN8	zinc finge	GO:0000981
Up	0.012188	0	0.105175	0.100986	LYPD6B	LY6/PLAUR	GO:000557[illegible]
Up	0.633288	0.632914	1.1167	1.09117	MYOF	myoferlin	GO:000177[illegible]
Up	0.126807	0.28366	1.00573	1.1767	TPM3P9	tropomyosi	GO:000573[illegible]
Up	0.100146	0.441405	0.677042	0.623747	WASH1	WAS protei	GO:000377[illegible]
Up	0.309502	0.820383	2.02385	2.03531	FGFRL1	fibroblast	GO:0001501
Up	1.40053	1.47894	2.83927	2.70108	ZKSCAN1	zinc finge	GO:0000981
Down	0.0953802	0.0803638	0.028555	0.0260813	OSBPL3	oxysterol	GO:000578[illegible]
Up	0	0	0.0132278	0.0217108	PGR	progesterone recepto	
Down	12.9016	10.7005	5.10241	5.11989	MSMO1	methylster	GO:000550[illegible]
Down	2.30442	1.52324	0.0097542	0.0210002	FGF7	fibroblast	GO:000016[illegible]
Down	681.259	451.851	287.666	284.367	RP5-857K21.6		GO:000412[illegible]
Up	1.5778	1.9115	2.96936	2.96454	ADAR	adenosine	GO:000367[illegible]
Up	2.34574	1.70514	6.4775	5.95171	HBEGF	heparin-binding EGF-	
Up	0.157772	0.219137	0.633924	0.453533	SETD1A	SET domain	GO:000079[illegible]
Down	4.57619	4.31827	1.56882	1.53885	TSPAN13	tetraspani	GO:000524[illegible]
Up	0.136884	0.0748757	0.514513	0.469454	NBPF10	neuroblast	GO:000573[illegible]
Up	0.180522	0.176199	0.509712	0.47669	CDR2	cerebellar	GO:000573[illegible]
Down	0.797534	0.935469	0.569319	0.066701	RPL36A-HNF	RPL36A-HNF	GO:000039[illegible]
Up	2.07962	3.23627	7.6372	7.53231	C1orf198	chromosome	GO:000582[illegible]
Down	2.71167	2.66691	1.27536	1.17072	SLC30A5	solute car	GO:000538[illegible]
Down	0.481593	0.464349	0.22518	0.21703	SLC35D1	solute car	GO:0005461
Down	0.195616	0.161998	0.0975109	0.102746	MKLN1	muskelin 1	GO:000172[illegible]
Up	0.73882	0.115499	2.36452	2.89524	ATXN7L3	ataxin 7-1	GO:000012[illegible]
Down	0.594236	0.643487	0.306289	0.340079	SETD3	SET domain	GO:000079[illegible]

Up	0.0620494	0.0450685	0.286659	0.296805	SEMA5A	sema domaiGO:0001755
Up	0.0897879	0.10826	0.373402	0.399973	PI4KAP1	phosphaticGO:0004430
Up	0.218682	0.371776	1.38917	0.979614	HOXD-AS2	HOXD cluster antiser
Up	0.0896689	0.336273	0.478906	0.468777	RABEP2	rabaptin, GO:0005096
Up	0.407881	0.482305	6.19818	6.55443	COL4A1	collagen, GO:0001569
Down	7.61332	8.40487	3.6376	1.99165	CARHSP1	calcium reGO:0000177
Up	0.0158515	0	0.0878312	0.0497095	RAVER2	ribonucleoGO:0003723
Up	0.31493	0.41185	1.19523	1.36376	FKBP1B	FK506 bindGO:0000413
Up	0.0088637	0	0.210929	0.161099	ST6GAL1	ST6 beta-galactosami
Down	0.233752	0.180524	0.0336568	0.0354743	RALGPS2	Ral GEF wiGO:0005085
Up	0.329688	0.522062	0.586791	0.85382	AATF	apoptosis antagonizi
Down	0.558483	0.484473	0.107807	0.0926037	CCDC152	coiled-coil domain c
Down	14.6714	15.0743	6.4725	6.49045	IDI1	isopentenyl-diphosph
Up	0	0	1.68435	1.53872	ZNF768	zinc fingeGO:0000981
Up	0.148023	0.302912	0.646648	0.570161	POM121	POM121 traGO:0005635
Up	0.0012605	0	0.0214583	0.0329126	PKNOX2	PBX/knotteGO:0000977
Down	0.0955148	0.172223	0.0072998	0	KCNK1	potassium GO:0005242
Down	0.0327064	0.165072	0.0025777	0	GABRA5	gamma-aminobutyric a
Down	2.29732	1.51711	0.0066521	0	XIST	X inactiveGO:0005576
Up	15.7902	28.5261	58.934	61.9937	RP11-307C12.12	
Down	11.7963	17.5732	5.87878	7.21348	ERAL1	Era-like 1GO:0000028
Up	1.57619	3.05422	4.96693	3.67024	NOL6	nucleolar GO:0000794
Down	2.04341	1.37818	1.04682	0.701707	COQ5	coenzyme QGO:0005739
Up	0	0	3.0963	2.7983	S1PR5	sphingosirGO:0005886
Up	0.0431334	0.0582721	0.115326	0.118761	RERE	arginine-gGO:0000118
Down	0.281773	0.149339	0.053682	0.0291364	RP11-566K19.6	
Up	5.97101	13.8898	20.7303	22.1202	EMILIN1	elastin miGO:0003180
Down	0.956982	0.619151	0.234927	0.207235	MBOAT2	membrane bGO:0003841
Up	0	0	0.111428	0.133302	CLDN14	claudin 14GO:0005198
Up	8.06701	14.0476	16.7039	17.9411	SYNGR2	synaptogyrGO:0005811
Down	3.16364	4.14899	0.54147	0.924002	EFEMP1	EGF containing fibul
Down	0.0346681	0.0818587	0	0	RP11-706O15.5	GO:0003676
Up	0.397441	0	3.36509	5.02334	TMEM102	transmembrGO:0005622
Up	7.15376	7.19425	11.4101	12.7335	KIF5B	kinesin faGO:0003777
Up	0.512657	0	8.56579	11.3802	XXbac-BPG248L24.12	GO:0000139
Up	1.46031	2.31682	2.72433	3.14805	GRAMD1A	GRAM domaiGO:0016021
Up	0.501622	0.597438	1.37019	0.936876	ZBTB4	zinc fingeGO:0000122
Up	0.767661	1.59042	2.68554	2.5943	KLHL17	kelch-likeGO:0005615
Up	0.0907912	0.116933	0.488752	0.472551	GATAD2A	GATA zinc GO:0000122
Up	0.0185329	0.0200609	0.12368	0.170201	ANOS1	anosmin 1 GO:0004867
Down	0.624423	0.62971	0.176957	0.238687	DERA	deoxyribosGO:0004139
Down	1.08458	0.902708	0.236834	0.376882	TMEM209	transmembrGO:0016021
Down	0.0128024	0.0155464	0	0	BACH2	BTB and CNC homology
Down	15.8595	14.5281	5.2485	5.05229	ARL4A	ADP-ribosyGO:0005525
Up	0.017355	0.0203201	0.055346	0.0379322	MAGI3	membrane aGO:0004385
Up	8.39552	5.88861	19.7228	18.0481	SOX11	SRY (sex determining
Up	0.0816562	0.107972	0.326074	0.199086	KIF7	kinesin faGO:0003777
Down	0.1395	0.0779079	0.0474202	0.0263466	MTM1	myotubularin 1
Up	0.0052452	0.0743098	0.144853	0.132183	CLCN4	chloride cGO:0005247
Up	44.6797	55.0836	140.594	141.461	MARCKSL1	MARCKS-like 1
Up	0.0889822	0.180628	0.393569	0.302239	UBE2O	ubiquitin-GO:0003723

Down	0.31362	0.203697	0.0135983	0.0068886	KDR	kinase ins	GO:0001525
Up	0	0	14.138	16.4324	SRGN	serglycin	GO:0002576
Up	0.0620771	0.0549665	0.194479	0.188982	LIMD1	LIM domains containi	
Down	2.87839	3.23773	1.65983	1.49685	ZCCHC9	zinc finge	GO:0003723
Down	1.97171	1.38519	0.808329	1.03406	CYB5B	cytochrome	GO:0005741
Down	56.6206	39.7218	9.70335	7.65678	ADM	adrenomedullin	
Up	3.62103	7.97463	14.0882	11.7904	CDR1	cerebellar degenerat	
Up	1.42481	2.355	2.47391	3.47005	MEF2BNB	MEF2B neig	GO:0005765
Up	0.502634	0.364636	1.87329	1.86754	CAPRIN2	caprin fam	GO:0003723
Up	1.63082	2.18883	6.29129	5.69549	FBXL6	F-box and	GO:0004842
Up	0	0	0.0427304	0.02345	RIMS3	regulating	GO:0017137
Down	2.80433	2.37804	0.0875872	0.11647	FLRT3	fibronecti	GO:0003345
Down	0.121861	0.191142	0.0164647	0.0052129	MX1	MX dynamin	GO:0000266
Up	0.0252342	0.07436	3.61996	2.89091	NOTCH3	notch 3	
Down	1.21064	1.17099	0.0097005	0.0167754	MME	membrane metallo-enc	
Up	0.398744	0.45085	0.734717	0.790635	ARID1A	AT rich ir	GO:0000122
Down	1.49378	1.2241	0.372675	0.374829	NR3C1	nuclear receptor sub	
Down	0.0744415	0.217318	0	0	FBXO2	F-box prot	GO:0000209
Up	0	0	0.0363715	0.0181824	RP11-627D16.1		GO:0003964
Down	0.603518	0.646445	0.281553	0.26112	PYGL	phosphoryl	GO:0002060
Down	0.207963	0.214274	0.0622506	0.1097	MPP6	membrane p	GO:0005886
Up	6.58574	10.9862	18.5151	19.3413	CDR1-AS	CDR1 antisense RNA	
Down	15.2317	13.4437	7.47888	7.90616	DDX39A	DEAD (Asp-	GO:0000398
Up	0.366945	0.594128	1.49035	1.05026	C11orf95	chromosome	GO:0003676
Up	0.0281647	0.091204	0.230012	0.120802	FBF1	Fas (TNFRS	GO:0000922
Down	2.88729	2.00381	0.916386	0.690098	API5	apoptosis inhibitor	
Up	0	0	0.163343	0.114571	RP11-175K6.1		GO:0005829
Up	0.0073058	0.0249625	0.0532446	0.0545624	TENM4	teneurin t	GO:0001702
Down	1.03063	1.40274	0.113507	0.254436	PNMA2	paraneopla	GO:0005730
Up	4.07645	4.31497	9.90244	9.76966	PLAC9	placenta-s	GO:0005576
Down	0.515915	0.212629	0.07807	0.0539294	KLF5	Kruppel-like factor	
Down	10.1988	8.16591	6.39388	5.02659	ARCN1	archain 1	GO:0000139
Up	0.642778	1.33163	2.0078	2.07492	SH3GL1	SH3-domain GRB2-like	
Down	11.6259	15.6343	6.82921	6.49472	THOC6	THO comple	GO:0000346
Up	0.0045716	0	0.294009	0.276702	COLEC10	collectin sub-family	
Up	0.0705934	0.176765	0.273962	0.343984	ARHGEF18	Rho/Rac gu	GO:0005085
Down	7.31496	7.71357	3.63117	5.01882	G3BP1	GTPase act	GO:0003677
Up	0.191634	0.0587013	3.5432	3.08689	MALL	mal, T-cel	GO:0000139
Down	0.860592	1.3673	0	0.0085233	MXRA5	matrix-rem	GO:0005201
Down	0.967872	0.885869	0.104855	0.156164	SYT15	synaptotag	GO:0000149
Down	1.01028	0.960601	0.179556	0.233893	RAET1G	retinoic a	GO:0001913
Up	0.0468734	0.0415043	0.356499	0.220361	TMEM38A	transmembr	GO:0005267
Up	1.13249	2.10064	2.8746	2.37728	USP5	ubiquitin	GO:0004197
Up	0.007381	0.0027885	0.0238896	0.0412563	ANKRD44	ankyrin repeat domai	
Down	0.071288	0.0614422	0.0136724	0.0376606	SLC41A2	solute car	GO:0005886
Up	0	0	0.584422	0.172698	C19orf81	chromosome 19 open r	
Up	0.357197	0.173955	0.717882	0.681195	PDCD1LG2	programmed	GO:0005576
Down	0.159246	0.118317	0.0741983	0.0751133	PRR16	proline ri	GO:0045727
Up	0.0695873	0.224154	0.373175	0.467455	ZNF592	zinc finge	GO:0000981
Down	2.50914	1.91433	1.11757	1.12902	HNRNPLL	heterogene	GO:0003723
Down	0.511681	0.365579	0.0641708	0.140907	ZEB1	zinc finge	GO:0000122

Down	1.76203	2.18655	0.462234	1.13073	PRSS35	protease,	GO:000557(
Up	0	0	1.28918	0.971008	C1orf162	chromosome	GO:0016021
Up	0.0878235	0.147044	1.26605	1.05574	ZNF175	zinc finge	GO:000012:
Down	8.93517	7.63857	4.37199	4.91293	C5orf15	chromosome	GO:0016021
Down	1.52597	1.8965	0.423885	0.446249	CCBL2	cysteine c	GO:000372:
Down	3.72607	3.67595	1.77865	1.69122	NRAV	negative regulator c	
Down	6.19943	8.19221	0.72535	0.50159	TBX3	T-box 3	
Down	0.261108	0.730872	0.0199518	0.0104746	CLDN11	claudin 11	GO:000519{
Up	0	0	0.0048108	0.0128815	DYSF	dysferlin	
Up	0.102666	0.242028	0.328322	0.359659	UNKL	unkempt fa	GO:000020{
Up	0.707207	0.793974	1.80938	1.70931	TFPI	tissue factor pathwa	
Up	0.0095677	0.0542191	0.223204	0.229195	ZNF582	zinc finge	GO:0000981
Up	5.89077	8.84687	10.386	11.6317	CNN1	calponin 1	GO:000377{
Up	0.637949	1.8778	13.4183	11.561	SLC9A3R2	solute car	GO:000510:
Up	0.155794	0.430399	0.768651	0.490766	ZNF142	zinc finge	GO:0000981
Down	3.31859	3.48226	1.31423	1.59246	GCLM	glutamate-cysteine l	
Up	15.1434	19.5133	38.7669	38.5667	FLNA	filamin A,	GO:000166
Down	0.0074075	0.0102612	0	0	PDGFD	platelet c	GO:000013{
Up	0.0700719	0.0834595	0.16871	0.151667	GSK3B	glycogen synthase ki	
Up	0	0	0.0770097	0.181623	SLC7A2	solute car	GO:000006
Down	209.412	158.139	2.9992	2.99308	LUM	lumican	GO:0005201
Up	0	0	0.398206	0.432259	CTA-390C10.10		GO:0016021
Up	0	0.112443	0.273311	0.207679	RIPK4	receptor-i	GO:000200{
Down	0.0118979	0.0288963	0.0042868	0	AMPD3	adenosine	GO:000387(
Down	0.889582	0.587761	0.296796	0.284772	GSTM1	glutathione S-transf	
Down	16.4519	32.029	4.31502	7.67857	ADAMTS1	ADAM metallopeptidas	
Up	0.25038	0.419214	1.40646	1.28682	GNB3	guanine nu	GO:000392
Up	3.29484	2.65932	6.49324	7.04565	PRSS23	protease,	GO:000154(
Up	0.28277	0.623168	1.9711	2.59662	TIGD5	tigger tra	GO:000014
Down	0.373309	0.258499	0.190755	0.181264	JPX	JPX transc	GO:000219(
Up	0	0	0.0798862	0.0402245	KLF8	Kruppel-li	GO:000012:
Down	1.37409	1.25451	0.645796	0.715554	SCRN1	secernin 1	GO:000563
Down	2.20521	2.00167	1.0861	0.878602	FDX1	ferredoxin	GO:000550(
Up	2.0333	1.50717	3.83526	4.61392	F2R	coagulation factor I	
Up	0	0	0.072363	0.0515492	EREG	epiregulin	GO:000016
Up	1.45024	2.07335	3.64126	3.22918	MSN	moesin	
Up	0	0	1.10244	1.0035	TRAC	T cell rec	GO:000225(
Up	0.227688	0.148523	0.739493	0.704744	TRIB2	tribbles p	GO:000486(
Up	0.0064426	0	0.123489	0.123467	VAX2	ventral an	GO:0000981
Down	5.21177	5.00858	2.85069	2.99221	DPF2	D4, zinc and double	
Up	0.482355	0.766581	1.48363	1.30813	CDK11B	cyclin-dep	GO:000027{
Up	0.0161515	0	0.11262	0.126543	RP11-356I2.4		GO:000565
Down	1.12246	1.00547	0.470237	0.468976	RABL3	RAB, membe	GO:000392
Down	0.0011765	0.003419	0.0003424	0	DPP10	dipeptidyl	GO:000588(
Down	0.0027527	0.0073409	0	0	TSHZ2	teashirt z	GO:0000981
Up	0	0	1.33266	2.41793	FOXC1	forkhead box C1	
Down	6.89225	5.82744	1.25215	2.2392	DDX3X	DEAD (Asp-	GO:000367
Up	3.1917	7.90337	8.56024	8.18104	SLC17A9	solute car	GO:000521
Up	0.459274	0.730798	1.46073	1.17716	SIDT2	SID1 trans	GO:000090:
Down	0.0208317	0.0329301	0.0059452	0.0033619	PDZRN4	PDZ domain	GO:001656
Down	1.8774	1.31248	0.90388	0.803851	SCFD1	sec1 famil	GO:000090:

Down	13.2835	0.389723	0.053368	0.0152071	MMP3	matrix metallopeptic	
Down	6.2809	5.5178	3.23281	4.0414	PROSC	proline sy	GO:0005622
Up	0.0024315	0	0.0942846	0.103679	ZNF702P	zinc finge	GO:0000981
Up	0.657632	2.40168	7.53736	5.62932	PTGER1	prostaglar	GO:0004957
Up	1.87376	1.72191	5.18554	5.38636	TSPYL4	TSPY-like	GO:0005634
Up	0.398718	0.300465	1.14109	1.2454	SMURF2	SMAD speci	GO:0000122
Down	1.18122	1.04284	0.641502	0.595468	PRPF18	pre-mRNA p	GO:0000350
Up	0.0102283	0	0.386945	0.442997	PWWP2B	PWWP domai	GO:0005654
Up	0.476335	0.510324	1.30295	1.47828	LYSMD4	LysM, puta	GO:0016021
Up	7.35592	11.5495	14.7321	13.6381	RIC8A	RIC8 guani	GO:0001701
Up	0.0794995	0.115847	0.26495	0.274237	CD72	CD72 molecule	
Up	0.506441	0.985208	31.6029	26.6358	TINAGL1	tubulointe	GO:0005044
Up	0.0120395	0	2.8922	3.20422	IL33	interleuki	GO:0002282
Down	0.641882	0.890024	0.146387	0.157036	OR2A20P	olfactory receptor,	
Down	2.72448	2.37594	0.986856	1.88719	TFDP1	transcription factor	
Down	3.35987	3.61209	2.14463	1.935	ERGIC2	ERGIC and	GO:0005634
Down	84.5019	115.464	3.36802	3.07867	ISLR	immunoglob	GO:0002576
Up	0.560249	0.819805	1.64184	1.38871	CYHR1	cysteine/h	GO:0005635
Up	0.0296361	0.0139954	0.627344	0.787588	MYLK	myosin light chain k	
Down	2.87795	2.9743	1.12831	1.765	SRP68	signal rec	GO:0005047
Up	0.337529	0.724526	0.83837	1.10013	IL17RC	interleuki	GO:0005102
Up	0.0414394	0.0432588	0.253031	0.184605	CLIP2	CAP-GLY do	GO:0005875
Up	0.0164888	0.0400459	0.107925	0.0797507	MAP3K14	mitogen-ac	GO:0001650
Down	2.37724	1.95619	0.919933	1.3804	PPIL3	peptidylpr	GO:0000398
Down	0.0245323	0.0155159	0.0055933	0.0047224	SOX6	SRY (sex c	GO:0000902
Up	0.0018746	0.0049991	0.0126538	0.0092982	PLCL1	phospholip	GO:0004435
Up	0.44793	1.85387	3.32565	3.35651	ZMYND19	zinc finge	GO:0005737
Down	46.5546	85.6992	13.7595	15.9077	MEST	mesoderm s	GO:0005783
Down	0.629221	0.872707	0.104184	0.0924652	HAND2-AS1	HAND2 antisense RNA	
Down	0.303646	0.835785	0	0	CTD-2531D15.4		GO:0000122
Up	0.0387166	0.111772	2.34717	1.70583	PODXL	podocalyxi	GO:0001726
Up	0	0	1.23776	0.757772	LINC01583	long intergenic non-	
Up	0.221213	0.310954	0.434318	0.444769	MED13	mediator c	GO:0003712
Up	0.301101	0	1.13909	0.829486	ZNF671	zinc finge	GO:0000981
Down	1.41663	1.04495	0.346768	0.397164	DSC3	desmocolli	GO:0001533
Up	0	0.181071	0.427105	0.410268	SLC26A10	solute car	GO:0008271
Up	0.0009167	0	0.0138725	0.0336	PRDM16	PR domain containing	
Up	0.0325993	0.0983104	0.157463	0.167693	PLXNA2	plexin A2	GO:0001756
Up	4.10085	2.61849	17.4804	21.695	CTD-2033D15.2		
Up	0.077581	0.04946	0.185881	0.316868	DBF4B	DBF4 zinc	GO:0003676
Up	4.40868	5.15125	19.4581	11.5627	JUND	jun D proto-oncogene	
Down	0.215713	0.215576	0.0798592	0.0872302	ANKRD28	ankyrin re	GO:0000139
Up	0.380327	0.268646	0.643999	1.00451	SDC3	syndecan 3	GO:0001523
Up	0.0031399	0	0.0712699	0.0729591	NTSR1	neurotensin receptor	
Up	0.0126059	0.0085989	0.0765384	0.0778431	NALCN	sodium lea	GO:0005244
Down	0.937948	0.913877	0.317003	0.406766	SHMT1	serine hyd	GO:0000900
Down	1.09338	0.711023	0.0395551	0.10305	GPR85	G protein-	GO:0004930
Down	1.2634	1.05032	0.635046	0.687256	3-Mar	membrane-a	GO:0005764
Down	1.0117	0.762417	0.195815	0.24988	HGF	hepatocyte growth fa	
Up	6.03148	5.53153	13.4297	14.6858	DKFZp434P2	HCG201654	GO:0005793
Up	0.0114414	0	1.16866	1.14748	RP11-357H14.17		

Down	0.0069131	0.0110875	0	0.0006249	ATP8B4	ATPase, cl	GO:0000287
Up	4.41666	4.02951	22.0303	28.0929	SLC29A1	solute car	GO:0005337
Up	0.0152942	0	0.228223	0.186851	REXO1	REX1, RNA	GO:0003676
Up	4.97454	6.97888	8.80834	13.4853	GEM	GTP bindir	GO:0000278
Down	7.56951	6.98195	4.29318	3.61744	MICA	MHC class	GO:0001913
Up	0.706725	0.57689	1.31335	1.5157	RRAGC	Ras-relate	GO:0000287
Up	1.69167	5.21799	5.50262	7.73095	DBN1	drebrin 1	GO:0003779
Up	0.435144	0	4.35755	3.11492	RP11-894P9.1		GO:0005654
Down	0.0090573	0.0283183	0	0	KCNN2	potassium	GO:0005216
Down	19.5342	17.3075	9.05406	8.83816	ALG2	ALG2, alph	GO:0000033
Up	0.530417	2.18606	5.71581	7.16169	RP11-809N8.2		
Down	612.868	492.242	379.209	328.343	TPI1	triosephos	GO:0004807
Up	0.0932357	0.0735108	0.214187	0.246848	ARSB	arylsulfat	GO:0003943
Down	2.92913	4.10765	0.0610798	0.0374555	DHRS3	dehydroger	GO:0000166
Up	12.1224	3.84888	104.951	100.297	ACTG2	actin, gam	GO:0005524
Down	6.339	6.17157	0.388114	0.0226856	IL1B	interleukin 1, beta	
Up	2.42275	3.09849	5.61104	5.26628	TSPYL5	TSPY-like	GO:0005634
Up	0.240049	0.355976	0.496028	0.601197	GALNT5	polypeptide N-acetyl	
Up	0.106053	0.278401	1.32506	2.44151	COL18A1	collagen,	GO:0001525
Up	0	0	0.561854	0.345178	RP11-201K1	Uncharacte	GO:0008250
Down	0.540139	0.447343	0.245821	0.268268	NSF	N-ethylmal	GO:0000139
Down	3.47283	3.7104	1.30966	1.33147	SLC43A3	solute car	GO:0016021
Down	0.0763578	0.112393	0.0150061	0	HPSE	heparanase	GO:0004566
Down	0.0373462	0.0282184	0	0	WEE2-AS1	WEE2 antis	GO:0000287
Down	0.723392	0.608536	0.397094	0.361527	WDR11	WD repeat	GO:0005634
Up	0.141294	0.139636	0.356744	0.255535	DLG5	discs, lar	GO:0001837
Up	0.0095102	0.0391952	0.0732018	0.0458004	FAM101A	family wit	GO:0005737
Up	1.02001	0.857436	5.24702	4.9119	GBP3	guanylate	GO:0000139
Up	0.352124	0.852428	2.15553	2.37496	FBRS	fibrosin	GO:0005615
Up	0.102473	0.138263	0.256135	0.26038	KDM2A	lysine (K)	GO:0000981
Up	0.134526	0.254115	0.668336	0.568637	NACC2	NACC famil	GO:0000790
Up	0	0	9.61535	5.84506	RP5-875H18.9		
Up	0	0	0.841419	1.59841	RP11-408A13.4		
Down	0.0196693	0.0242671	0.0016475	0.0029905	SLC9A9	solute car	GO:0005886
Up	0.31436	0.498418	1.44318	1.2865	MAP4K4	mitogen-ac	GO:0000165
Down	0.00581	0.032925	0	0	JAKMIP2-AS	JAKMIP2 ar	GO:0005794
Down	0.480211	0.304492	0.138553	0.142799	MRPS22	mitochondr	GO:0003735
Up	0	0	2.61226	1.53007	RP11-999E24.3		
Up	0	0.0801201	0.469489	0.693173	USP32P1	ubiquitin	GO:0004843
Up	0.913322	1.00175	3.17653	3.02069	NKX6-2	NK6 homeob	GO:0000978
Down	0.201186	0.101073	0.0602767	0.0307625	RP11-203B9.4		GO:0000981
Down	1.33862	0.901891	0.45433	0.493984	ARL6IP6	ADP-ribosy	GO:0016021
Up	0.195662	0.64064	2.07806	2.90185	KCNG1	potassium	GO:0005249
Down	1.80149	1.29945	0.590782	0.661056	TBC1D23	TBC1 domai	GO:0005794
Down	0.308448	0.202987	0.0426603	0.0533909	FBXL21	F-box and	GO:0000151
Down	0.705767	0.828103	0.230673	0.200128	FRMD6	FERM domai	GO:0003383
Down	3.37294	4.59236	1.06334	1.71043	IFI35	interferon	GO:0005634
Up	0.458144	1.56784	2.37375	1.9205	BMP1	bone morph	GO:0001501
Down	1.69847	3.62356	0.0440963	0	TMEM119	transmembr	GO:0001649
Down	27.7708	32.1123	17.2574	18.0476	PTTG1IP	pituitary tumor-tran	
Down	3.84859	2.47138	1.86732	1.7093	GGPS1	geranylger	GO:0004161

Up	0.165609	0.0293279	0.653163	0.687235	GAP43	growth associated pı	
Down	3.14077	2.24728	0.440066	0.379988	CDKN1C	cyclin-dej	GO:000012
Up	0.0931652	0.134683	0.263739	0.397167	PI4KAP2	phosphatic	GO:000443
Up	0.0330284	0.036394	0.189738	0.270767	PLEKHG3	pleckstrir	GO:000508
Up	0.866536	0.853225	1.8946	1.65604	LAMP1	lysosomal-	GO:000161
Down	0.227148	0.229304	0.0603032	0.104862	SLC3A1	solute car	GO:000382
Down	5.26295	4.92417	2.63339	2.54353	UGP2	UDP-glucos	GO:000398
Down	5.99479	4.75049	2.53616	3.57413	TIMM21	transloca	GO:000574
Up	2.35412	6.16936	6.40657	7.92627	C8orf58	chromosome 8 open re	
Down	13.8389	8.42333	6.07564	4.22374	BZW1P2	basic leuc	GO:000573
Up	0.0495632	0.0334884	0.0973568	0.0882709	NAV3	neuron nav	GO:000552
Up	0.161307	0.219552	0.637482	0.634215	DLC1	DLC1 Rho GTPase acti	
Up	0.0585406	0.152708	0.227323	0.236396	UHRF1BP1	UHRF1 binc	GO:004280
Up	0.635308	0.758202	1.72964	1.54836	TNFRSF10B	tumor necr	GO:000562
Up	6.84389	11.3813	22.948	24.0354	ITGA5	integrin,	GO:000152
Up	0.0344818	0.0212397	0.152425	0.144553	PKP4	plakophili	GO:000092
Down	0.6577	0.65044	0.352658	0.196396	KAT7	K(lysine)	GO:000012
Down	2.56495	1.96234	0.159073	0.187235	LPXN	leupaxin	GO:000210
Up	0.0028319	0	0.088386	0.100063	CNTNAP3B	contactin	GO:000715
Up	0.128449	0.339692	0.637115	1.18158	ELL3	elongatior	GO:000563
Down	12.2402	12.2432	0.300285	0.387031	OLFML3	olfactomec	GO:000727
Down	3.87852	3.01107	0.720201	1.19524	ABHD14B	abhydrolas	GO:000563
Down	2.53207	2.10007	0.927705	1.07657	SEC23B	Sec23 homc	GO:000013
Down	0.0352719	0.0494275	0.0003626	0	PLD5	phospholip	GO:000382
Down	0.106692	0.090049	0.0329198	0.0244707	ZNF215	zinc finge	GO:000098
Up	0.0974871	0.196292	4.24866	4.02839	AKAP12	A kinase (PRKA) ancl	
Down	0.0765578	0.0895238	0.0252847	0.0253988	PSD3	pleckstrir	GO:000508
Down	0.470418	0.254086	0.0630287	0.11269	ZCCHC4	zinc finge	GO:000367
Up	0.446561	1.32191	1.6898	1.96139	GSN	gelsolin	
Up	0.0213438	0.0205006	0.327088	0.376625	CPXM2	carboxypep	GO:000418
Down	0.12555	0.151068	0.0151815	0.0090639	BCO2	beta-carot	GO:000152
Up	0.0170937	0	0.312558	0.454527	RAB9B	RAB9B, men	GO:000392
Up	0	0.0656887	0.0694324	0.180491	MARVELD2	MARVEL don	GO:000573
Down	0.661793	0.804726	0.306336	0.329476	CARNMT1	carnosine	GO:000563
Up	1.06805	2.4418	4.2255	2.76717	DHRS1	dehydroger	GO:000578
Up	0.174897	0.212549	0.417452	0.358614	CCDC85C	coiled-coi	GO:000592
Up	0	0	0.533638	0.866276	RP11-24B13.2		GO:000579
Up	0.204427	0.231694	0.730351	0.758236	AFAP1	actin fila	GO:000377
Down	0.0414378	0.018736	0.0030574	0	ZFHX4-AS1	ZFHX4 antisense RNA	
Down	0.0410269	0.0541428	0.0096857	0.012027	TGFBR3	transforming growth	
Down	1.39291	1.41246	0.349231	0.82313	NFE2L2	nuclear fa	GO:000078
Down	22.6557	23.6626	8.98939	10.0893	RRP7A	ribosomal	GO:000002
Up	0.0629805	0.416389	0.803027	0.422439	CCDC106	coiled-coi	GO:000563
Up	0	0	0.178573	0.139682	AC012462.2		
Up	0	0	0.134221	0.132867	ADAMTSL2	ADAMTS-lik	GO:000823
Up	0.0078288	0.0146488	0.020769	0.0242739	MAST4	microtubul	GO:000028
Down	0.208709	0.125157	0.0751964	0.0444413	CH507-254M2.1		
Down	0.634161	0.483191	0.0134403	0.0025532	ELOVL2	ELOVL fatt	GO:000578
Up	0.0062955	0.0078314	0.0198471	0.0186122	AC007319.1		
Down	2.40821	2.27673	1.48925	0.989725	DAB2	Dab, mitogen-respons	
Up	0.0014472	0.0025234	0.0144593	0.0201073	LARGE	like-glycosyltransfe	

Up	9.06662	10.8773	27.2352	22.2202	RP11-334J6.7		
Up	0	0	1.22609	1.41774	ERICD	E2F1-regulated inhil	
Down	4.18468	3.66042	2.28419	2.11981	TECR	trans-2,3-	GO:0005634
Up	0.225236	0.126063	2.29561	2.27813	SH2B3	SH2B adapt	GO:0005068
Up	33.9796	34.0097	100.683	83.2277	PPP1R14B	protein ph	GO:0004865
Up	0.267955	0	3.19127	2.51051	CTD-2373N4.3		
Up	0.0112212	0.0198718	0.120277	0.114243	TMEM246	transmembr	GO:0016021
Down	6.03008	11.5906	0.546841	0.778698	C1R	complement	GO:0004252
Up	0	0	0.0247148	0.0175278	FRK	fyn-relate	GO:0003964
Up	0	0	0.513681	0.439691	RASA4B	RAS p21 pr	GO:0005096
Down	0.279454	0.199482	0.146758	0.0998132	TCEANC2	transcript	GO:0005634
Up	0	0.0433718	1.34673	3.03557	ACAN	aggrecan	
Up	28.2431	40.0873	101.992	98.429	PCSK7	proprotein	GO:0004252
Down	0.543195	0.726234	0.167849	0.127542	TPD52L1	tumor prot	GO:0000086
Down	0.516865	0.524837	0.260853	0.23169	NSMAF	neutral sp	GO:0005737
Down	0.43608	0.560846	0.136502	0.142249	FAM86C2P	family wit	GO:0005737
Up	3.70214	6.3043	8.53856	8.55922	FHL1	four and a	GO:0003254
Up	0.555443	0.571012	3.78778	4.31369	SLC19A1	solute car	GO:0005542
Up	0	0	0.196802	0.560784	CATSPER1	cation cha	GO:0005227
Up	0	0	0.486493	0.944704	CST2	cystatin S	GO:0001580
Up	2.10237	7.06275	17.034	20.5552	FNDC4	fibronecti	GO:0005615
Down	0.347814	0.319953	0.177994	0.114325	SUCO	SUN domain	GO:0001503
Up	0.0463931	0.0672549	0.150339	0.190222	ZC3H12C	zinc finge	GO:0004519
Up	0.0122414	0	0.455385	0.401746	ADAMTS15	ADAM metal	GO:0004222
Up	0	0	0.234165	0.348504	KBTBD11	kelch repeat and BTI	
Down	0.0257349	0.0341964	0	0.0001701	RP11-482H16.1		
Up	0	0	0.0297807	0.0279537	GPR143	G protein-coupled re	
Down	1.27397	1.37648	0.644899	0.578699	NBPF1	neuroblast	GO:0005737
Down	0.0884025	0.10576	0.017341	0.018824	ECM2	extracellu	GO:0005178
Up	0.921402	0.746502	2.52027	1.97978	LMO7	LIM domain	GO:0000151
Up	0.176029	0.319437	2.00902	0.881264			
Down	0.0345006	0.0278356	0	0.0022413	DKK2	dickkopf W	GO:0005615
Up	0.157195	0.107092	0.367207	0.37862	RP3-394A18.1		GO:0004438
Down	0.0383438	0.0830818	0.0181322	0.0070241	KIF21A	kinesin fa	GO:0003777
Up	0.146413	1.36658	1.3087	1.58449	LPIN3	lipin 3	GO:0003713
Up	0.0110081	0.0081368	0.0712184	0.0914165	TACC2	transformi	GO:0000226
Up	0.497994	1.17528	1.73674	1.39822	INTS1	integrator	GO:0005654
Down	1.02299	0.731968	0.521219	0.507445	SLC11A2	solute car	GO:0001666
Down	1.16264	0.58593	0.33983	0.2519	FECH	ferrochelatase	
Down	0.143354	0.186965	0.0624065	0.0714644	PPP2R3A	protein ph	GO:0000159
Down	3.44584	2.68865	2.01601	1.83155	CD46	CD46 molecule, compl	
Up	0	0	0.600147	0.228014	SIX2	SIX homeob	GO:0000976
Up	0.459843	1.6805	4.10196	3.47493	GPC1	glypican 1	GO:0001523
Up	0	0	1.5411	1.84018	AC002044.1		GO:0016021
Down	0.115338	0.115566	0.034148	0.0470903	FBXL2	F-box and	GO:0004842
Up	1.42273	2.1249	3.54768	3.4903	RNF41	ring finge	GO:0000209
Up	0.0708788	0.148931	0.262115	0.372396	MEGF8	multiple E	GO:0003143
Up	0	0	2.51992	0.616626	RP11-433J22.2		GO:0001501
Up	0	0	0.341394	0.549344	LINC00619	long intergenic non-	
Up	2.35136	2.13977	6.69564	6.06255	GREM1	gremlin 1,	GO:0000902
Down	0.115579	0.0953444	0.0027674	0.0070094	TVP23A	trans-golg	GO:0009306

Up	6.75227	24.2012	74.9692	80.965	STC2	stanniocal	GO:000517[illegible]
Up	0.0500067	0.177115	0.283769	0.222471	SYS1-DBNDI	SYS1-DBNDI	GO:000573[illegible]
Up	0.0258422	0.0464906	0.137851	0.129707	CDYL2	chromodoma	GO:000371[illegible]
Up	0	0	0.0477485	0.0472668	PDE9A	phosphodie	GO:000411[illegible]
Up	0	0	1.61218	2.35584	RP11-555G19.1		
Up	0.655343	1.42582	3.70776	2.60993	EMC10	ER membran	GO:000152[illegible]
Down	0.0319331	0.0270093	0.0036061	0	RAB27B	RAB27B, me	GO:000257[illegible]
Down	0.269501	0.182746	0.0435702	0.0353145	ZFP37	ZFP37 zinc	GO:0000981
Down	3.58137	3.27321	0.942226	0.88146	TWSG1	twisted ga	GO:000150[illegible]
Up	0.0021797	0.0082346	0.0627599	0.0577835	ARHGEF6	Rac/Cdc42	GO:000508[illegible]
Up	0.278765	0.673056	0.734005	0.822035	PTDSS2	phosphatic	GO:000388[illegible]
Up	0.0630789	0.699383	1.76381	1.87372	SLC25A29	solute car	GO:000528[illegible]
Down	1.08677	0.776525	0.583932	0.380321	SPRTN	SprT-like	GO:000367[illegible]
Up	33.4519	32.1363	154.845	151.87	THBS1	thrombospondin 1	
Down	1.57097	1.20756	0.666064	0.506117	C9orf64	chromosome	GO:010103[illegible]
Up	0.16963	0	0.926938	1.86922	AC007191.4		
Up	1.66736	1.06803	3.09932	3.01568	STK17A	serine/thr	GO:000467[illegible]
Down	0.122468	0.0760563	0	0	IP6K3	inositol h	GO:000082[illegible]
Down	1.64068	1.57681	0.718466	0.953316	GMPS	guanine monphosphate	
Down	5.62975	4.19258	2.87614	1.7618	HOXA10	homeobox A	GO:000097[illegible]
Down	0.0182474	0.0258517	0.0026966	0	LRFN5	leucine ri	GO:000998[illegible]
Up	0.0766612	0.0695092	0.487227	0.483349	SH2B1	SH2B adapt	GO:000506[illegible]
Down	10.1126	9.39158	4.1454	4.65695	UGDH	UDP-glucos	GO:000170[illegible]
Down	1.0493	1.05123	0.577182	0.551411	STT3B	STT3B, sub	GO:000457[illegible]
Up	0.303281	0.370481	0.967979	0.627049	STK24	serine/thr	GO:000016[illegible]
Up	0.166247	0.692159	1.67713	1.7084	EPHB4	EPH recept	GO:000152[illegible]
Up	0	0	2.45308	2.44082	RPS4Y1	ribosomal	GO:000018[illegible]
Up	20.4116	9.09771	67.1544	61.0491	RP11-982M15.2		
Up	0.415188	0.888849	1.48925	1.30255	ANAPC2	anaphase p	GO:000565[illegible]
Up	0	0	0.0539951	0.16053	CBLN2	cerebellin	GO:000561[illegible]
Up	0	0	0.0837703	0.0261591	SLC6A1	solute car	GO:000533[illegible]
Down	0.0108094	0.004712	0	0	EBF2	early B-ce	GO:000097[illegible]
Down	1.14715	0.446929	0.070069	0.0618303	PRDM1	PR domain containing	
Down	0.321412	0.266701	0.126026	0.144572	PAK1	p21 protein (Cdc42/R	
Up	0.651985	0.841738	1.19949	1.35143	ZBED6	zinc finge	GO:0000981
Up	0.911232	1.02776	3.39707	3.13232	TBX2	T-box 2	
Down	18.3212	31.9382	4.35073	4.24768	TIMP3	TIMP metallopeptidas	
Down	0.131093	0.100563	0.0305726	0.0390375	SSBP2	single-str	GO:000097[illegible]
Up	0.0059626	0.0168949	0.539492	0.669186	ALPK3	alpha-kina	GO:000467[illegible]
Down	0.05927	0.036782	0.0146467	0.0111295	AMPH	amphiphysi	GO:000554[illegible]
Up	7.06882	18.9524	27.4129	23.8161	RP11-792A8.4		
Down	7.66095	5.83186	3.90992	3.69095	SMS	spermine s	GO:000582[illegible]
Up	0.409415	0.564353	1.00311	0.818773	ZNF496	zinc finge	GO:0000981
Up	0.0233726	0.0128178	0.0598979	0.06791	LINC01006	long inter	GO:0016021
Down	3.88561	4.81674	0.578456	0.749814	RP11-1151B14.4		GO:000467[illegible]
Down	0.048992	0.0484817	0.0021914	0.0012168	LAMA2	laminin, a	GO:000510[illegible]
Up	0.187488	0.610717	0.618064	0.714366	ZGPAT	zinc finge	GO:000012[illegible]
Down	1.41323	2.36564	0.818025	0.707917	TNC	tenascin C	
Down	0.0192445	0.0276362	0.002318	0.0028757	NRG1	neuregulin 1	
Down	0.19583	0.171904	0.0063378	0.0020639	PTGFR	prostaglan	GO:000495[illegible]
Up	1.47986	1.09386	5.88242	8.4156	CITED4	Cbp/p300-interacting	

Up	0.358501	0.574979	2.06251	1.94833	LMO7-AS1	LMO7 antis	GO:0004843
Down	1.57872	2.46619	0.0363264	0.033025	DPP4	dipeptidyl-peptidase	
Up	0.365	0.75862	0.986494	0.962052	ZNFX1	zinc finge	GO:0000775
Up	0.199263	0.109278	0.972682	0.637477	HOXB7	homeobox I	GO:0000978
Up	0.104667	0.155577	0.493332	0.435697	SMO	smoothened	GO:0000122
Down	0.212459	0.372241	0.0288246	0.0204041	ARHGEF3	Rho guanir	GO:0005085
Up	0	0	0.0268932	0.0223215	GRB14	growth fac	GO:0005070
Up	0	0.855837	3.20582	3.47308	CTXN1	cortexin 1	GO:0016021
Down	7.91182	6.05555	3.24591	2.75202	SDHD	succinate dehydrogen	
Up	0.69537	1.32805	2.10581	2.25048	PLBD2	phospholip	GO:0016042
Down	9.96595	13.0216	3.79679	3.85428	CNIH1	cornichon	GO:0000139
Up	0.840902	1.26273	2.08066	2.02075	HNRNPL	heterogene	GO:0000381
Down	0.224737	0.264083	0.0502347	0.107312	IPP	intraciste	GO:0003779
Up	0.0005892	0	0.0876802	0.135132	CNTNAP3	contactin	GO:0005576
Up	0.0023644	0.0084859	0.0160507	0.0154058	PARD3B	par-3 fami	GO:0000226
Down	2.88922	2.37997	1.16444	1.46682	TM2D3	TM2 domair	GO:0016021
Down	0.27924	0.224534	0.0333095	0	FAM46C	family wit	GO:0003723
Up	0.0034675	0.0061862	0.0359528	0.0270115	PDE10A	phosphodie	GO:0004114
Down	3.71158	2.23216	0.856743	1.0411	SERPINB9	serpin per	GO:0002020
Up	0	0	3.18576	2.42074	RP11-865I6.2		GO:0003677
Up	0.328799	0.189095	0.622963	0.667547	DIAPH1	diaphanous-related f	
Down	2.16396	2.06911	0.868197	0.849517	PRKAR1A	protein ki	GO:0001707
Down	10.7346	7.50403	5.58165	5.51657	BOLA3	bolA famil	GO:0000981
Up	2.83479	16.4137	16.7081	18.778	LLNLR-284B4.1		GO:0005783
Up	0.284248	0.391647	0.673782	0.626268	XPO6	exportin 6	GO:0005634
Up	0.190673	0.31676	0.584068	0.57225	GALNT10	polypeptide N-acetyl	
Down	0.722663	0.673501	0.385686	0.324372	ELMSAN1	ELM2 and M	GO:0000118
Up	0.704637	1.27737	1.62665	1.77872	SIAH2	siah E3 ubiquitin pr	
Down	0.459775	0.402669	0.0527081	0	RP11-43F13.3		
Up	0.242849	0.307322	0.5114	0.45019	RBM6	RNA bindir	GO:0003677
Up	0.479399	0.735776	2.23762	2.71471	C1orf54	chromosome	GO:0005576
Up	0.0688928	0.0780819	1.75489	1.4765	HHIP-AS1	HHIP antis	GO:0003824
Down	272.295	204.474	92.9239	94.1127	MT1E	metallothi	GO:0005634
Down	0.0671041	0.12785	0.0080243	0	MKX	mohawk hom	GO:0000981
Down	22.8273	14.4982	3.5098	4.11283	BAG2	BCL2-assoc	GO:0000774
Down	0.0323244	0.12212	0	0	APOL4	apolipopro	GO:0005615
Down	0.980803	1.1799	0.302187	0.347568	SKP2	S-phase ki	GO:0000082
Up	0.309825	0.577007	0.84743	0.87251	MLKL	mixed line	GO:0000165
Up	0.0090765	0.0313088	0.336406	0.304402	PPP1R14C	protein ph	GO:0004865
Down	0.544989	0.339919	0.209632	0.224157	UBE2W	ubiquitin-	GO:0004842
Up	0	0	1.04317	2.54786	CTA-228A9.3		GO:0003677
Down	4.9547	5.06191	0.868467	0.929927	ALDH1A3	aldehyde c	GO:0001758
Down	14.3918	13.4608	5.19785	3.81576	GPX1P1	glutathior	GO:0004602
Up	0.957002	4.59797	4.40747	5.49539	ADAM15	ADAM metal	GO:0001525
Up	0.13914	0.157699	0.679907	0.529968	ZBTB47	zinc finge	GO:0000981
Up	0.0165188	0.022734	0.105641	0.10899	ITPK1	inositol-t	GO:0000287
Down	0.323132	0.141584	0.088217	0.0566586	DCAF17	DDB1 and C	GO:0005654
Up	0.475757	0.433851	2.67561	1.3257	DDX51	DEAD (Asp-	GO:0003723
Down	0.036477	0.0182807	0.0128295	0.0064199	BTBD11	BTB (POZ)	GO:0016021
Down	0.187822	0.323516	0.0269964	0.0262573	RUNX1T1	runt-related transcr	
Up	0.483031	1.41142	2.04149	2.32507	LZTS2	leucine zi	GO:0000281

Up	0.0757519	0.228949	0.45746	0.924262	CCNYL1	cyclin Y-l	GO:0005886
Up	0	0	0.0291149	0.0719008	RAPGEF3	Rap guanir	GO:0001525
Up	0.100812	0.0445161	0.384681	0.355657	MINK1	misshapen-	GO:0000165
Up	0.0074051	0.0109174	0.0543578	0.0294784	FAM155A	family wit	GO:0005886
Up	0.485035	0.532362	1.02122	1.08306	CREB3L2	cAMP responsive elem	
Up	3.90382	8.74147	9.65115	8.92531	PIM3	Pim-3 prot	GO:0004674
Up	0.474337	1.20961	1.66286	2.24984	BRAT1	BRCA1-asso	GO:0001934
Up	0.258115	0.19331	0.571639	0.5317	SLC4A7	solute car	GO:0005452
Down	9.37149	7.10006	2.70847	2.7001	RP11-486O12.2		GO:0003677
Up	0	0.222422	9.0245	14.1911	ESAM	endothelia	GO:0005886
Up	0	0	0.0030346	0.0127872	ABLIM2	actin binc	GO:0005737
Up	0.251795	0.439269	0.857725	0.666017	ROCK1	Rho-associ	GO:0000139
Down	12.6588	9.44412	6.85845	6.7377	NUTF2	nuclear tr	GO:0000060
Up	0.0953442	0.131217	0.292755	0.284955	SASH1	SAM and SH	GO:0000209
Down	2.11668	2.19105	0.983388	0.777589	NSDHL	NAD(P) dep	GO:0000252
Up	0.827273	0.643463	4.35696	2.16049	CHCHD10	coiled-coi	GO:0005634
Down	0.521341	0.416575	0.269557	0.270629	TBC1D15	TBC1 domai	GO:0005096
Down	2.94125	9.24151	0.0342956	0.0279903	SPON2	spondin 2,	GO:0001530
Up	0.353437	0.358543	0.983828	0.756353	BRD4	bromodomain containi	
Up	0.0401549	0.0273456	0.148215	0.134421	INPP5A	inositol p	GO:0004445
Down	2.69213	3.08753	0	0.337807	CTD-3014M21.1		
Up	1.35039	3.94585	20.9668	17.9637	ARL4C	ADP-ribosy	GO:0005525
Down	0.071615	0.0428785	0.0025534	0.0008169	LDB2	LIM domair	GO:0000122
Down	1.01674	0.817621	0.536054	0.520525	METTL10	methyltrar	GO:0005634
Up	0.359002	0.436771	0.922049	0.869714	OSBP	oxysterol	GO:0000139
Down	1.22147	0.85745	0.444053	0.380474	GFM2	G elongati	GO:0003746
Down	4.82256	3.16292	2.04505	2.07194	GOT1	glutamic-c	GO:0004069
Down	0.322724	0.209576	0.0730337	0.0681993	MB21D2	Mab-21 dom	GO:0044877
Up	0.0112362	0.0069887	0.0964195	0.106484	KLF12	Kruppel-li	GO:0000122
Down	13.5739	11.9391	7.17007	7.63285	FUCA2	fucosidase	GO:0004560
Up	1.14684	1.5264	2.5705	2.63748	SPTAN1	spectrin,	GO:0000165
Up	0	0	0.175925	0.165132	GZMK	granzyme K	GO:0004252
Up	0.115943	0	1.12467	0.589669	AURKC	aurora kir	GO:0000780
Down	13.1321	10.5864	7.11376	7.127	TOMM34	transloca	GO:0005634
Up	0.287416	0.254699	0.559957	0.664111	WWTR1	WW domain	GO:0000122
Up	0.0140766	0.079771	1.426	1.45674	HHIP	hedgehog i	GO:0003824
Down	0.0911109	0.11407	0	0.0304546	LCA5L	Leber cong	GO:0005930
Down	8.122	8.21287	4.30921	5.17765	GINS2	GINS compl	GO:0000727
Up	0	0	0.642799	0.709593	TTTY15	testis-spe	GO:0016021
Up	0.0095454	0.0052348	0.0935778	0.0786298	RTN1	reticulon	GO:0005783
Down	0.22234	0.187097	0.0657032	0.0588229	TMEM38B	transmembr	GO:0005267
Down	0.811863	0.67361	0.239832	0.274497	ALKBH1	alkB homol	GO:0000049
Up	0.275536	0.400038	0.778193	0.75715	STK11IP	serine/thr	GO:0005200
Up	0.105042	0.132659	0.269843	0.316624	RAPH1	Ras associ	GO:0005829
Up	0.1473	0.143685	0.333564	0.303402	SIPA1L1	signal-inc	GO:0005096
Up	0.922462	1.23	1.78328	2.19545	BRD3	bromodomai	GO:0003682
Up	0.532426	0.528438	1.2385	1.29273	DHX33	DEAH (Asp-	GO:0000182
Down	1.04748	0.764075	0.53293	0.527203	THOC3	THO comple	GO:0000346
Up	0.0865736	0.122651	2.5716	1.55081	DUSP4	dual speci	GO:0000188
Up	0.338257	0.780948	0.955791	0.933346	PLCXD1	phosphatic	GO:0005737
Up	0.0491821	0.0899067	0.140045	0.124354	TBC1D12	TBC1 domai	GO:0005096

Down	1.4206	1.40167	0.496436	0.624154	SLC5A3	solute car	GO:0005367
Up	3.752	4.66647	9.64233	9.88381	KIAA2013	KIAA2013	GO:0016020
Up	0.143356	0.339603	0.897951	0.578732	MFSD12	major faci	GO:0005765
Down	2.16177	2.12459	1.08148	1.20658	SEC24D	SEC24 fami	GO:0000139
Down	0.0407533	0.0360053	0.0144123	0.0180481	SPAG16	sperm asso	GO:0005930
Down	1.136	1.13301	0.71218	0.655001	RAI14	retinoic a	GO:0001650
Up	0.0018711	0	0.0813335	0.0977655	MYO1D	myosin ID	GO:0000146
Up	1.0947	0.672871	3.19547	3.67686	MSL1	male-speci	GO:0003682
Up	0.0001423	0	0.0084341	0.0064769	TEX41	testis expressed 41	
Up	3.03731	2.9091	12.4075	11.6005	RP1-56K13.3		
Up	0.0571199	0.0944105	0.215089	0.226532	SOCS7	suppressor	GO:0005634
Up	0.316893	0	2.6774	1.34618	RP1-152L7.5		GO:0001837
Down	2.15513	1.51006	0.955022	0.787502	HIAT1	hippocampu	GO:0005215
Down	0.278052	0.530096	0.100644	0.0962115	ACADSB	acyl-CoA c	GO:0003995
Up	32.112	47.7764	91.7467	111.993	HLA-A	major hist	GO:0000139
Up	0.207819	0.225147	0.897958	0.591543	VPS9D1	VPS9 domai	GO:0005096
Up	0.464221	0.526139	2.63428	3.22494	RP11-51F16.1		GO:0003677
Up	0	0	1.04963	0.416914	RP11-175K6.2		GO:0003677
Down	0.0110668	0.008437	0.0021903	0.0007133	KIAA1217	KIAA1217	GO:0005737
Down	0.0125531	0.0226347	0.0017989	0.0010935	EEPD1	endonuclea	GO:0003677
Down	2.64223	1.9283	1.64129	1.32378	ARMC10	armadillo	GO:0002039
Up	0.645208	0.790823	1.18948	1.2988	ZC3H13	zinc finge	GO:0003723
Up	0.28748	0.751903	5.52146	5.03253	LRFN4	leucine ri	GO:0005886
Up	0	0	0.160514	0.274429	C1orf115	chromosome	GO:0016021
Down	2.22649	2.50984	0.438067	0.486207	NRP2	neuropilin	GO:0001525
Up	0.0323588	0.0884128	0.244104	0.199327	ZNF652	zinc finge	GO:0000981
Up	0	0	0.902036	0.184355	GJA5	gap juncti	GO:0001501
Up	0	0	0.0176976	0.0512694	LRRN1	leucine ri	GO:0005615
Up	0.013524	0.0957992	0.100193	0.151456	BEND3	BEN domain	GO:0000122
Up	0.0087932	0.0332203	0.143535	0.078172	RADIL	Ras associ	GO:0005874
Up	0	0	0.162263	0.524015	HEYL	hes-relate	GO:0000977
Up	0.0118229	0.0139582	0.119272	0.151523	CHST15	carbohydra	GO:0000139
Up	0.137631	0.440438	1.27819	1.32344	MIR143HG	MIR143 hos	GO:0003677
Up	0.469665	2.28133	8.37626	10.158	HTATSF1P2	HIV-1 Tat	GO:0000398
Down	1.62316	1.66362	0.729633	0.719601	COQ3	coenzyme Q	GO:0004395
Down	5.15396	4.64965	3.44553	2.45592	NDUFA5	NADH dehyd	GO:0005743
Up	4.21567	8.85228	11.9454	15.0737	FADD	Fas (TNFRSF6)-associ	
Down	2.32223	1.54509	0.91398	1.20145	CPSF3	cleavage a	GO:0000398
Down	8.60658	6.54474	4.10618	3.66071	MRPL37	mitochondrial ribos	
Down	0.388907	0.438231	0.132624	0.129338	PPAP2B	phosphatid	GO:0001933
Up	0.113705	0.13905	0.259064	0.305345	TANC1	tetratrico	GO:0007520
Up	0.700474	0.829514	4.83889	4.44729	PCDH10	protocadhe	GO:0005509
Down	633.795	252.759	10.7065	11.8413	CXCL1	chemokine (C-X-C mot	
Up	3.48997	8.18373	16.0255	12.3617	F8A3	coagulatio	GO:0005634
Down	1.1913	3.63515	0	0	MAB21L2	mab-21-lik	GO:0001654
Down	1.07185	1.35065	0.53743	0.65933	USP4	ubiquitin	GO:0000244
Up	0.225176	0.490517	0.62802	0.5313	LPCAT1	lysophosph	GO:0000139
Down	0.432499	0.425612	0.158838	0.184088	SEMA3C	sema domai	GO:0001755
Up	0	0	0.275551	0.328876	EFHD1	EF-hand do	GO:0005509
Down	1.48003	0.968417	0.600313	0.69008	BET1	Bet1 golgi	GO:0000139
Up	0.285039	0.817377	0.900772	0.927972	SLC41A1	solute car	GO:0010961

Up	0.0741164	0	2.12888	2.17791	ANKRD34A	ankyrin repeat domai
Up	0	0	0.368636	0.529777		
Down	1.75274	1.41894	0.771833	0	NEFM	neurofilament, mediu
Down	0.674818	0.709556	0.199445	0.238692	HACD4	3-hydroxyaGO:0005783
Down	0.0031656	0.0026576	0	0	LINGO2	leucine riGO:0005615
Down	0.329856	0.538728	0.0061334	0.0097448	IGFBP7-AS1	IGFBP7 antisense RNA
Down	1.89271	2.70364	0.9992	0.789263	PSPH	phosphoserGO:0000287
Up	1.06373	2.4997	2.93459	3.31178	NUCB1	nucleobincGO:0001965
Down	0.437927	0.478672	0.204533	0.20083	LIPA	lipase A, GO:0000902
Up	0.012159	0.0459361	0.894418	0.636137	ADAMTS7	ADAM metalGO:0004222
Down	1.66626	1.44225	0.444193	0.320737	NET1	neuroepithelial cell
Down	2.21285	1.90742	1.37276	1.06938	IFT57	intraflageGO:0001843
Down	7.40554	7.59663	4.35749	4.03529	SWI5	SWI5 homolGO:0000724
Up	0.380717	0.559014	0.977673	1.12201	SSH1	slingshot GO:0000902
Up	0.261219	0.640132	0.718904	0.914955	FEM1C	fem-1 homcGO:0005654
Up	34.8986	22.8985	145.355	159.819	TFPI2	tissue facGO:0004867
Up	0	0	0.253295	0.229721	PCDH1	protocadheGO:0005509
Down	0.350112	0.471895	0.164746	0.202494	UHRF1BP1L	UHRF1 bincGO:0005769
Up	5.31099	10.6029	11.0458	13.0643	HRAS	Harvey rat sarcoma v
Down	0.801732	0.172096	0.0459549	0.0145497	IFIT1	interferonGO:0003723
Up	2.4529	2.51006	7.58697	6.6589	PAGR1	PAXIP1 assGO:0000978
Down	0.282373	0.120377	0.0189355	0.0677307	LRRC8D	leucine riGO:0005225
Down	1.15606	1.15091	0	0	BST2	bone marrcGO:0002737
Down	5.35919	5.50673	2.73508	3.42203	KLHDC2	kelch domaGO:0005634
Up	0.679098	2.08913	11.5976	11.2853	MEX3B	mex-3 RNA GO:0000932
Up	0.0111094	0.019371	0.0786672	0.0900736	PKP1	plakophiliGO:0001533
Down	0.586775	0.405164	0.309798	0.321416	VRK1	vaccinia related kin
Down	0.132324	0.147622	0.0531988	0.0673729	SLIT2	slit guidance ligand
Down	1.49607	2.59988	0.756019	0.708454	RHBDD2	rhomboid cGO:0000139
Down	1.38385	1.29482	0.872537	0.768536	DPYSL2	dihydropyrGO:0005829
Up	0.019172	0.044446	0.164839	0.11941	TNK2	tyrosine kGO:0004674
Down	13.6009	10.5926	7.11789	8.40161	UFM1	ubiquitin-fold modif
Up	0.0039557	0	0.0349173	0.0303227	AC005562.1	GO:0016021
Down	9.19731	9.50623	0.838854	1.51732	IGFBP5	insulin-like growth
Up	0.348812	0.589412	0.92451	0.926133	PHF11	PHD fingerGO:0005654
Up	0.458333	1.33577	4.34304	3.02406	CTD-2545M3.2	GO:0001525
Up	0.842365	1.7442	1.95426	2.25591	PRKCSH	protein kiGO:0005080
Up	0	0	0.0012291	0.004225	THSD7B	thrombospcGO:0016021
Up	0.229195	0.519531	1.54916	1.74815	AP001062.7	GO:0001750
Up	0.0897349	0.187761	0.456115	0.527813	WTIP	Wilms tumcGO:0000932
Up	0	0	0.0055267	0.0079646	LINC01091	long intergenic non-
Down	0.452156	0.402652	0.0651637	0.014442	CD83	CD83 molecule
Down	1.91177	1.19841	0.821431	0.816179	SUCLA2	succinate-GO:0000287
Down	0.257563	0.364897	0	0	SPEF1	sperm flagGO:0005737
Up	9.44993	17.6447	32.2895	20.9252	CD151	CD151 molecule (Raph
Up	0.665686	1.71472	2.28941	2.37067	APLP1	amyloid beGO:0005604
Up	0.0556343	0.0488454	0.153158	0.194466	ZNF431	zinc fingeGO:0000122
Up	0.0653463	0	0.339032	0.3417	NPIPB11	nuclear pcGO:0005654
Down	0.0378084	0.0273073	0	0	SNCA	synuclein,GO:0000287
Down	0.541234	0.510311	0.34828	0.214829	ORC4	origin recGO:0003964
Up	0	0	0.289415	0.274215	RASA4CP	RAS p21 prGO:0000165

Up	0.330209	0.268102	0.510889	0.627525	ARMC9	armadillo	GO:0005737
Down	0.373043	0.302333	0.141281	0.160048	TUBGCP3	tubulin, g	GO:0000923
Up	0.543183	0.693434	1.97964	1.53571	FAM65A	family wit	GO:0005737
Down	0.435804	0.131715	0	0	ACKR3	atypical chemokine r	
Up	0	0	0.0193686	0.0119141	RNF17	ring finge	GO:0005634
Up	0.006724	0	0.14245	0.145611	RP4-541C22.5		
Down	19.1033	15.8791	6.17051	6.75447	PLS3	plastin 3	GO:0005509
Up	5.78373	10.4883	31.9453	24.7174	RP11-490M8.1		
Down	0.396364	0.406291	0.224187	0.141866	TTC13	tetratricopeptide re	
Down	23.6362	37.199	3.69099	4.89345	FOS	FBJ murine osteosarc	
Down	0.475571	0.410166	0.183384	0.180619	DSE	dermatan s	GO:0000139
Down	0.908912	0.547762	0.153346	0.131461	GPRC5A	G protein-	GO:0004930
Up	0.192137	0.523672	0.644953	0.504102	STK40	serine/thr	GO:0004674
Down	0.360017	0.412208	0.187002	0.150393	MCU	mitochondr	GO:0005262
Up	0	0.187547	0.528629	0.380544	PEX6	peroxisomal biogenes	
Up	3.19458	2.98407	6.75836	6.7108	ULBP2	UL16 bindi	GO:0001913
Down	0.0702413	0.110496	0.0038511	0.0078786	SLC39A8	solute car	GO:0005385
Down	0.575656	0.229836	0.0065993	0.116588	VWA5A	von Willeb	GO:0005634
Up	0.0859703	0.0314911	0.331828	0.298187	CYFIP2	cytoplasmi	GO:0000902
Up	0.10993	0.188843	0.265658	0.269029	ARHGEF11	Rho guanin	GO:0001558
Down	0.0863324	0.10044	0.0403748	0.035062	AUH	AU RNA bin	GO:0003730
Up	0.192107	0.224134	1.04204	0.780978	WIZ	widely int	GO:0000981
Up	0.323352	0.17571	0.872299	1.01157	KIF3B	kinesin fa	GO:0003777
Up	0.0033754	0	0.0251687	0.02257	DNM3	dynamin 3	GO:0001917
Down	0.542115	0.497991	0.198219	0.238381	STARD7-AS1	STARD7 ant	GO:0005576
Down	0.704929	0.641056	0.291835	0.39469	PRCP	prolylcarb	GO:0002155
Up	0.0737087	0.169912	0.499403	0.375886	FAM20C	family wit	GO:0004674
Up	0.0041249	0.0048699	0.0270916	0.0271734	ZNF704	zinc finge	GO:0001158
Down	0.0180656	0.0170627	0.004381	0	KIAA1324L	KIAA1324-l	GO:0016021
Down	2.48377	2.13262	0.213552	0.3606	HIST1H2AG	histone cl	GO:0000786
Up	0.0551538	0.378669	0.330365	0.490884	RAB40C	RAB40C, me	GO:0000139
Up	0.0054217	0	0.0439142	0.0477552	CCDC3	coiled-coi	GO:0005576
Up	0.773922	1.60175	2.90236	4.51395	KAZALD1	Kazal-type	GO:0001503
Up	0.0862087	0.0703493	0.204378	0.214445	ETV6	ets variant 6	
Up	11.4916	33.7341	57.5393	57.3773	PTRF	polymerase	GO:0003723
Down	0.563749	0.59085	0.209449	0.187033	PDHX	pyruvate c	GO:0005759
Up	0.0403506	0.0465542	0.11304	0.0995545	RIN3	Ras and Ra	GO:0005096
Down	0.339697	0.479204	0.189505	0.156962	NCK1	NCK adapto	GO:0000164
Down	9.2944	9.39927	5.8217	4.28453	HOXD11	homeobox D	GO:0000981
Down	0.110699	0.0982549	0.0260688	0.0261986	CA13	carbonic a	GO:0004089
Up	0.392918	0.278329	4.14965	8.14174	BLID	BH3-like m	GO:0005739
Down	0.164608	0.244043	0.107539	0.0741135	RNF13	ring finge	GO:0000139
Up	0.0380948	0.0651066	0.110946	0.11762	BMP2K	BMP2 induc	GO:0004674
Down	14.1015	23.5897	9.72542	4.81585	NEU1	sialidase 1 (lysosom	
Down	0.289528	1.34242	0	0.0126104	CTD-2006M22.2		GO:0004842
Up	0.575087	0.931133	1.31121	1.41879	GCN1	GCN1 eIF2	GO:0003723
Up	0.850888	2.44072	2.54339	2.73034	CPSF1	cleavage a	GO:0000398
Up	1.53013	2.7067	3.38367	3.10398	HDGF	hepatoma-d	GO:0000122
Up	0.654912	1.01762	2.69072	2.96564	AC092171.4		GO:0000981
Down	1.67506	1.70727	0.692957	0.77597	PXMP4	peroxisoma	GO:0005777
Up	0.0514422	0	2.37316	1.36786	AREG	amphiregulin	

Down	23.9075	26.4145	10.7894	13.8603	BCKDK	branched chain ketoa
Down	3.64991	4.43826	1.30742	2.29663	UBFD1	ubiquitin GO:0003723
Down	0.344438	0.228265	0.0743779	0.0454892	CHN1	chimerin 1GO:0005070
Up	0.526496	0.37711	0.964215	1.06275	PCGF3	polycomb gGO:0000805
Up	9.97063	11.958	19.8005	17.5563	YBX1	Y box binding protei
Down	1.21021	1.08656	0.678427	0.630068	KLHL12	kelch-likeGO:0000139
Down	1.80752	1.49517	0.672875	0.613173	B3GALNT1	beta-1,3-N-acetylgal
Down	0.0560971	0.0491984	0.0109479	0.0102386	RGS17	regulator of G-prote
Down	0.134643	0.194991	0.0066029	0	SLC7A7	solute carGO:0000821
Up	0	0	0.295779	0.774142	RP11-736K20.4	GO:0000978
Up	0	0	0.149623	0.113692	AGT	angiotensinogen (ser
Down	0.0145914	0.0136035	0.0042142	0.004871	IMMP2L	inner mitoGO:0001541
Up	0.0023415	0.0053077	0.0794285	0.0836888	SCN9A	sodium channel, volt
Up	0.175486	0.176334	0.435869	0.471953	ANKRD36	ankyrin repeat domai
Up	0.0723371	0	0.679997	0.337118	GPR183	G protein-GO:0002250
Down	3.94892	3.95513	2.44315	2.14194	TVP23B	trans-golgGO:0009306
Up	0.150142	0.406926	0.819075	1.51061	ARRDC2	arrestin dGO:0005886
Up	0.0672646	0.0911523	0.17112	0.150485	ASXL2	additionalGO:0003677
Up	5.72719	6.57478	27.5985	25.0894	RGS4	regulator GO:0005096
Down	0.0096255	0.0454557	0	0	EMBP1	embigin psGO:0005886
Up	0.662895	0.125219	4.26324	5.08153	LENG9	leukocyte GO:0046872
Up	0.7527	0.553959	19.5054	20.2635	MCAM	melanoma cell adhesi
Down	0.171513	0.120656	0.0712244	0.0691384	DLEU2	deleted inGO:0003677
Up	0	0	0.018712	0.0481851	FNDC1	fibronectiGO:0005576
Down	0.803738	0.910943	0.369217	0.324562	ADAMTS4	ADAM metalGO:0001501
Down	0.928441	0.618349	0.251056	0.324672	HSD17B7	hydroxysteGO:0000253
Down	1.47722	1.07987	0.500106	0.621907	ATP6V1C1	ATPase, H+GO:0005886
Up	0.768687	2.23389	4.73484	4.29661	C7orf43	chromosomeGO:0005815
Down	0.0109602	0.0248443	0.0012286	0.0011669	LRRTM3	leucine riGO:0005615
Down	3.47226	2.40879	1.3296	1.29595	SKA3	spindle anGO:0000278
Down	0.131467	0.108013	0.0382105	0.0332494	PECR	peroxisomaGO:0001561
Down	0.0098546	0.0108588	0	0.0005246	NOVA1	neuro-oncoGO:0000398
Up	1.59428	2.49643	4.76909	4.17562	COL5A1	collagen, GO:0001568
Up	0.167704	0.262169	0.401081	0.427464	OGDH	oxoglutaraGO:0004591
Up	0.1781	0.384487	2.68438	1.47902	RP11-488C13.5	GO:0016021
Up	0.0382382	0.0628095	0.252978	0.322327	SARDH	sarcosine GO:0005737
Down	3.40337	6.10368	1.70891	1.39582	TMEM97	transmembrGO:0001558
Up	0.180468	0.30443	0.453033	0.906452	PLEKHM2	pleckstrinGO:0007030
Up	0.106147	0.192487	0.465811	0.322761	RP11-1017G21.4	GO:0002177
Up	1.68097	2.13985	6.10268	6.38169	MYH9	myosin, heavy chain
Down	0.171161	0.122638	0.0530913	0.078799	POT1	protectionGO:0000781
Up	0	0	0.162533	0.167326	KCNK3	potassium GO:0005216
Down	1.24752	1.13947	0.545965	0.489467	VRK3	vaccinia rGO:0004672
Up	0.10301	0.291873	3.24745	3.05984	RP11-863P13.3	GO:0016021
Down	0.0283523	0.0207316	0.0028833	0	KCNMA1-AS1	KCNMA1 antGO:0001666
Down	2.27493	2.47531	1.15246	1.129	SLC16A1	solute carrier famil
Up	0.0364698	0.0413343	0.96348	0.823844	MFAP3L	microfibriGO:0005634
Up	0.239984	0.578996	0.701134	0.963075	CNTROB	centrobin,GO:0005813
Down	0.0172898	0.006478	0.0007208	0.0019169	ARAP2	ArfGAP witGO:0005096
Down	0.23837	0.146861	0.027574	0.016555	PAN3	PAN3 poly(A) specifi
Down	1.36915	1.34712	0.748782	0.723572	ACSL3	acyl-CoA sGO:0004467

Down	9.99455	8.09961	5.97505	5.83242	RSL24D1	ribosomal	GO:0000027
Up	0.13081	0.148376	0.45045	0.386782	ROR1	receptor t	GO:0001725
Up	0	0	0.326399	0.322424	PPP1R3G	protein ph	GO:0005737
Down	0.723137	0.925864	0.340394	0.317189	CTSO	cathepsin	GO:0004197
Down	0.338613	0.232593	0	0	PTPRD-AS1	PTPRD antisense RNA	
Down	0.196619	0.0567756	0	0	DPT	dermatopor	GO:0005201
Down	0.209757	0.128225	0.0896481	0.0755914	GLI3	GLI family	GO:0000122
Up	0.0246623	0.217403	0.475136	0.501514	RHBDF2	rhomboid 5	GO:0005789
Up	0.141747	0.0935846	0.357494	0.437798	ZNF282	zinc finge	GO:0000981
Down	0.293212	0.345965	0.173851	0.156545	ALPK2	alpha-kina	GO:0004674
Up	1.35659	2.67019	6.2486	5.91986	SLC4A2	solute carrier famil	
Up	0	0	0.0117218	0.0298382	PLXDC1	plexin dom	GO:0001525
Up	0.0088134	0	0.0555696	0.0880696	PQLC2L	PQ loop repeat conta	
Up	0.098867	0.148875	0.276154	0.263452	HERC2	HECT and RLD domain	
Down	0.267371	0.216453	0.0120415	0	NIPSNAP3B	nipsnap ho	GO:0005739
Up	0.864836	1.09237	2.42658	2.75035	LTBP2	latent tra	GO:0005201
Down	0.0676779	0.16675	0.0241189	0	TCEANC	transcript	GO:0005634
Up	0.225598	0.481065	0.610032	0.705338	PSKH1	protein se	GO:0004674
Up	0.0873184	0.0656089	0.216947	0.392771	CDH13	cadherin 13	
Up	143.501	139.985	288.018	327.7	RP11-244F12.2		
Down	0.535595	0.594336	0.140888	0.185185	COG6	component	GO:0000139
Up	0.210726	0.224578	0.50064	0.488653	PHF20	PHD finger	GO:0000123
Up	0.26806	0.881437	1.80283	2.13096	SETD8	SET domain	GO:0000122
Down	46.4962	23.1378	0.818023	0.712994	CXCL3	chemokine (C-X-C mot	
Up	0.116268	0.116655	0.642113	0.823879	EDIL3	EGF-like r	GO:0005178
Up	0.612171	1.39071	1.98603	1.95874	SSBP4	single str	GO:0000978
Down	10.4923	6.54977	4.82562	4.78626	MAPRE1	microtubule-associat	
Down	1.09153	0.815156	0.501497	0.267558	THNSL1	threonine synthase-l	
Down	0.514369	0.529859	0.32417	0.256773	RAP1GDS1	RAP1, GTP-	GO:0005096
Down	0.506393	0.46501	0.10702	0.0457763	GALM	galactose	GO:0004034
Down	1.70102	1.64499	0.734953	0.508677	RNF149	ring finge	GO:0006511
Up	1.62184	2.00072	3.62197	3.21383	ATMIN	ATM intera	GO:0006974
Up	0	0	0.995884	0.778668	EGFL8	EGF-like-d	GO:0005102
Up	0.42818	0.222345	0.901346	0.915377	TNPO2	transporti	GO:0005634
Up	0.0110281	0.0078119	0.172096	0.173038	PTPN3	protein ty	GO:0001784
Down	0.706837	0.733707	0.0240284	0.0254877	KCNMA1	potassium	GO:0001666
Down	0.749038	0.951161	0.309588	0.249646	OTUD6B	OTU domain	GO:0008283
Up	0.0214303	0.174575	38.5754	38.0084	IGFBP2	insulin-like growth	
Up	0.0185667	0.11223	0.145161	0.213488	VIL1	villin 1	GO:0001726
Up	0.923465	1.14722	2.47547	2.18029	LAMC1	laminin, g	GO:0005201
Down	1.71249	1.35234	0.305802	0.288839	TGFBR2	transforming growth	
Up	0	0	1.27766	1.24823	RP3-329E20.2		
Down	7.36054	6.76192	0.718772	0.338283	LAYN	layilin	GO:0001726
Up	0.0079513	0.0112648	0.0460847	0.027399	EPB41L4A	erythrocyt	GO:0005737
Up	0.0291384	0.0317547	0.384402	0.154637	ADGRB2	adhesion G	GO:0004930
Up	2.02316	3.26313	4.31752	4.45879	TRIP10	thyroid ho	GO:0001891
Up	3.45301	5.53006	11.5485	15.8961	RP11-801F7.1		
Down	0.0172773	0.004835	0.0008069	0	PLAC1	placenta-s	GO:0001890
Up	3.101	3.72254	7.58209	5.25665	SLC39A4	solute car	GO:0005385
Down	2.71554	1.70412	1.17976	1.25446	DBR1	debranchir	GO:0000375
Up	0.00132	0	0.0596462	0.0501713	FAM174B	family wit	GO:0016021

Down	1.23949	1.10129	0.558865	0.489381	RCHY1	ring finger and CHY	
Up	0.0057785	0.0087323	0.0380129	0.022517	FBXL7	F-box and	GO:0000086
Up	0.248125	0.661695	1.0215	1.03726	TNS2	tensin 2	GO:0001822
Up	0.017504	0.0198387	0.694197	0.893972	CCDC81	coiled-coi	GO:0005737
Up	0.185274	0.534689	0.679276	0.632866	CC2D1A	coiled-coi	GO:0000122
Up	0.140122	1.02093	1.04504	1.07029	PIDD1	p53-induce	GO:0005123
Up	0.0027002	0.0044424	0.0218577	0.0272918	CAMK1D	calcium/ca	GO:0004683
Up	0.0589516	0.0610327	0.142604	0.13606	NCK2	NCK adapto	GO:0001771
Up	0.745591	1.59442	2.41853	2.72966	PINK1	PTEN induc	GO:0000287
Up	0.891923	0.282228	2.27803	1.78738	EFS	embryonal	GO:0005737
Up	0.0351285	0.0474706	0.33428	0.356282	ATP8B1	ATPase, am	GO:0000287
Up	0	0	0.0316093	0.177138	IL1RL1	interleuki	GO:0002826
Down	3.27541	1.50498	0.530253	0.402918	EIF4EBP3	eukaryotic	GO:0005737
Up	0.016471	0.0081165	0.0677296	0.0761683	TRANK1	tetratricopeptide re	
Up	0.100507	0.0718869	0.318702	0.28518	SLC6A6	solute car	GO:0005328
Up	0	0	0.112609	0.126925	CRHBP	corticotro	GO:0001963
Up	0.599218	0.626026	1.06801	1.69045	CHRNB1	cholinergi	GO:0001941
Down	21.1737	19.7357	12.3483	11.1571	SLC35B1	solute car	GO:0005459
Up	0	0	0.761521	1.14697	CTD-2162K18.4		
Up	0.0167874	0.0815421	0.110887	0.108004	RNF215	ring finge	GO:0005770
Down	0.667432	0.423471	0.256925	0.273563	SLC35A3	solute car	GO:0000139
Down	7.37698	5.63885	3.35729	4.31895	OSER1	oxidative stress res	
Up	0.0677775	0.153069	0.184816	0.188604	HTT	huntingtin	
Down	0.017931	0.0335686	0.0031293	0.004909	CHRM3	cholinergi	GO:0003056
Up	5.3432	7.14812	10.4473	10.9045	PDAP1	PDGFA asso	GO:0003723
Down	87.1112	76.6805	51.2458	52.4009	GPX4	glutathion	GO:0004602
Up	0.229676	0.242815	0.473315	0.498244	LTBP1	latent tra	GO:0001527
Down	1.45443	1.10828	0.578908	0.911342	ATPAF1	ATP syntha	GO:0005739
Up	3.13299	2.05911	9.2177	8.90985	ERRFI1	ERBB receptor feedba	
Down	0.889176	2.2904	0.101934	0.232368	RP11-274H2.5		
Down	0.354648	0.736674	0.068436	0.0556299	CYP27A1	cytochrome	GO:0005506
Up	0.317126	0.56032	2.02994	1.69961	EHD3	EH-domain	GO:0001881
Up	0.0236133	0.0473926	0.103909	0.113839	ATP9A	ATPase, cl	GO:0000287
Up	0.0640626	0.171965	0.191332	0.264118	WDR62	WD repeat	GO:0000922
Up	0.897172	0.887094	3.00599	2.84331	TRAM2	translocat	GO:0005791
Up	0	0	0.177369	0.231044	GPR162	G protein-	GO:0004930
Down	34.6773	38.2212	11.7193	13.7396	NFKBIA	nuclear factor of ka	
Up	0.243879	0.387433	0.618936	0.784169	TSPAN2	tetraspani	GO:0005887
Down	4.20367	2.55234	0.567958	0.287713	RP13-463N16.6		
Down	2.99429	3.47959	0.439723	0.67552	CTD-2031P19.5		GO:0002675
Down	4.6586	3.24921	2.16622	2.60094	SNRNP40	small nucl	GO:0000375
Down	2.45853	1.69605	1.00598	1.1659	ECD	ecdysonele	GO:0003713
Down	2.97732	2.51886	1.72322	1.7183	PDGFRA	platelet-d	GO:0000165
Up	0.689421	0.812842	2.38058	2.39415	TP53INP2	tumor prot	GO:0000045
Up	0.483752	1.78679	2.55993	2.2142	NCDN	neurochond	GO:0005829
Up	0	0.768973	4.16381	8.2782	CTD-2026K11.1		GO:0000187
Down	2.65996	3.05352	0.80891	0.598269	AVPI1	arginine v	GO:0000187
Down	0.101256	0.166207	0.004403	0.0197394	PDZRN3	PDZ domain	GO:0004842
Down	0.312188	0.21198	0.129169	0.124279	ASCC1	activating	GO:0003723
Down	0.0753515	0.153143	0.0049749	0.0015121	SVEP1	sushi, von	GO:0003682
Up	0.0215215	0.0265131	0.0808275	0.083833	BEND7	BEN domain	GO:0070062

Down	18.1593	17.7113	7.95313	8.49284	DNAJB9	DnaJ (Hsp4	GO:0002377
Down	2.31722	1.84208	1.12708	0.845354	ELL2	elongation	GO:0005654
Up	0	0	0.0964301	0.242275	CACNA1H	calcium ch	GO:0005244
Up	0.190203	0.207695	0.42233	0.408103	IPO8	importin 8	GO:0005635
Down	1.28936	2.28653	0.105173	0.0547179	ASS1	argininosuccinate sy	
Up	0.10688	0.211694	0.457986	0.471299	KMT2D	lysine (K)	GO:0000981
Up	9.54934	10.7262	16.9904	16.5591	FSTL1	follistati	GO:0005509
Down	1.59849	1.72507	1.01369	0.689447	TMEM106B	transmembr	GO:0005764
Down	0.164779	0.312797	0.0276373	0.0598903	SLC22A3	solute car	GO:0005330
Up	0.0620001	0.111486	0.1699	0.180512	VPS53	vacuolar p	GO:0000938
Down	0.002509	0.0142183	0	0	MAN1C1	mannosidas	GO:0000139
Up	0.0149384	0.0380946	0.118678	0.0737175	PPP1R37	protein ph	GO:0004864
Up	4.23685	7.60693	12.0485	11.612	MFGE8	milk fat g	GO:0001525
Up	0.0159513	0.0774809	0.202587	0.137561	DTX4	deltex 4,	GO:0004842
Up	3.90895	3.63515	12.9426	8.70127	FZD2	frizzled c	GO:0003149
Up	4.9183	6.58273	19.9777	16.3644	F8A1	coagulatio	GO:0005634
Up	0.149156	0.193309	0.598852	0.879325	GOLGA8B	golgin A8	GO:0000137
Up	0.413775	0.580135	1.47468	1.4851	SWAP70	SWAP switc	GO:0003677
Up	0.0588171	0.0521508	0.129167	0.137063	SPIRE1	spire-type	GO:0003779
Up	0.205907	0.156383	0.42562	0.466473	EPG5	ectopic P-	GO:0005737
Down	0.133104	0.209219	0	0.0083786	DSC2	desmocolli	GO:0001533
Up	0	0	0.16904	0.189957	HID1	HID1 domai	GO:0000138
Up	0.363025	0.561307	0.811581	0.705242	NID1	nidogen 1	GO:0005201
Down	0.017863	0.0616172	0	0	TBX5	T-box 5	GO:0000978
Up	1.16045	3.42967	4.15902	4.45312	SURF6	surfeit 6	GO:0001652
Up	1.29128	2.60646	3.19311	3.46331	ITFG3	integrin a	GO:0009986
Up	0.0269474	0.033405	0.133802	0.170259	NEURL1B	neuralized	GO:0005769
Down	0.638178	0.674405	0.230735	0.317869	TUBA4A	tubulin, a	GO:0000226
Down	0.304366	0.367812	0.099209	0.128626	PIK3R1	phosphoinositide-3-k	
Up	0.266467	0.329926	1.2029	1.35819	SLC46A3	solute car	GO:0016021
Up	0.704805	0.612118	2.08063	1.74142	RP11-367J11.2		GO:0001533
Up	0.412888	1.61986	4.24541	2.25206	RP11-259N19.1		GO:0003677
Down	0.0081007	0.0099257	0.0018406	0.0009091	MECOM	MDS1 and EVI1 comple	
Up	0.803276	0.620359	1.53902	1.57201	B3GNT2	UDP-GlcNAc:betaGal b	
Up	0.212559	0.235208	1.68121	1.46429	NDUFV3	NADH dehyd	GO:0000978
Down	0.197758	0.240422	0.103548	0.0878617	WI2-1896014.1		
Up	0.0572234	0.0829554	0.227109	0.324316	DIXDC1	DIX domain	GO:0003779
Up	4.1598	6.23443	12.0723	11.8584	AES	amino-term	GO:0000122
Down	0.0619285	0.0877358	0.006212	0.0023601	STXBP6	syntaxin b	GO:0000145
Up	0	0	3.34422	4.49238	RP11-157P1.4		GO:0000502
Down	0.774665	0.475944	0.307973	0.288021	AP1AR	adaptor-re	GO:0001920
Down	0.336377	0.298696	0.168384	0.149987	CD109	CD109 molecule	
Down	16.5545	9.11998	5.51267	4.04007	KIF20A	kinesin fa	GO:0000281
Up	0	0	0.0235381	0.0051102	RP11-12J10	Uncharacte	GO:0005634
Down	0.109523	0.252862	0.0204611	0	FAM86FP	family wit	GO:0005737
Up	0.224184	1.98396	1.96342	2.51303	AHSA2	AHA1, acti	GO:0004197
Down	2.54246	1.7857	0.457239	0.0661787	CASP1	caspase 1, apoptosis	
Up	0	0	0.358071	0.175393	ICAM2	intercellular adhesi	
Up	0.121917	0.138179	0.867099	0.925228	AC159540.1		GO:0003964
Up	0.013717	0.0282666	0.286196	0.149361	CD4	CD4 molecule	
Up	0.260566	0.593538	0.950309	0.709864	GAREML	GRB2 associated, reg	

Up	0.0054756	0.496471	1.53287	0.540411	MFRP	membrane f	GO:0007601
Up	0	0	0.0185621	0.0440769	SYT13	synaptotag	GO:0000149
Up	1.80212	2.49359	4.92799	3.58611	VCAN	versican	
Up	0.0873954	0.161499	0.225208	0.233934	FOXK1	forkhead b	GO:0000976
Down	0.171097	0.175757	0.0664874	0.0752865	TMEM116	transmembr	GO:0016021
Up	0.0145443	0.0148689	0.0406029	0.0388606	PRKG1	protein ki	GO:0001764
Up	0.89674	1.46942	2.59679	2.75585	PGRMC2	progestero	GO:0003707
Up	0	0	1.52736	0.685797	RP11-25K19.1		
Up	0.144519	0.163796	0.596846	0.567765	SGCA	sarcoglycan, alpha (	
Up	0.095969	0.165519	0.418307	0.349841			
Up	0	0	0.209528	0.180666	EMX2OS	EMX2 opposite strand	
Up	0.161808	0.29739	0.908957	0.756495	CAP2	CAP, adeny	GO:0000902
Down	0.400523	0.411929	0.186079	0.14209	TTC26	tetratrico	GO:0005813
Down	57.0814	30.3336	0.883571	0.907024	CXCL6	chemokine (C-X-C mot	
Up	0.993223	4.32124	4.03218	5.7901	SLC22A17	solute car	GO:0004888
Up	0	0.683921	1.09576	1.68839	RP11-45M22.2		
Up	0.101145	0	1.08415	0.890898	HAS1	hyaluronan	GO:0005737
Down	0.367663	0.329067	0.146451	0.192109	ARFIP1	ADP-ribosy	GO:0000139
Down	15.2271	11.7137	8.53153	8.14093	C6orf62	chromosome	GO:0005622
Up	0.0099876	0.0178733	0.0556816	0.0584285	RAD9A	RAD9 checkpoint clam	
Up	0.193534	0.288296	0.435545	0.497899	XRCC4	X-ray repair complem	
Up	0.495918	0.820994	1.12074	1.42558	MIB2	mindbomb E3 ubiquiti	
Up	0	0	2.41669	3.22842	THBD	thrombomod	GO:0004888
Up	0.250667	0.437989	1.04312	0.986116	ARHGEF17	Rho guanine nucleoti	
Down	1.32695	1.3297	0.698917	0.403156	PPAP2C	phosphatid	GO:0004721
Up	0.352727	0.58519	1.13043	1.04623	FAM120A	family wit	GO:0003723
Down	153.91	138.986	81.5044	84.2085	COPS6	COP9 signa	GO:0000338
Up	2.50104	3.07449	3.92053	6.04291	HNRNPD	heterogeneous nuclea	
Up	0.053461	0.112528	0.438203	0.364441	CYTH3	cytohesin	GO:0000139
Up	0	0	0.546283	0.30291	HSPA8P11	heat shock	GO:0005524
Down	3.90527	3.31766	1.87434	2.25623	VBP1	von Hippel	GO:0005634
Up	1.37273	3.32982	4.61828	3.84901	RHBDD3	rhomboid d	GO:0000165
Up	0.22557	0.429571	0.995345	1.10328	HSPG2	heparan sulfate prot	
Down	0.965116	0.96872	0.269455	0.36741	SYNGR1	synaptogyr	GO:0016021
Up	0.205901	0.140274	0.437002	0.514264	TECPR1	tectonin b	GO:0000421
Up	0.0270688	0.0401753	0.220249	0.2075	DLGAP4-AS1	DLGAP4 ant	GO:0005886
Down	1.03089	0.833938	0.624108	0.48982	TMEM184C	transmembr	GO:0016021
Down	0.0394256	0.0411566	0	0.0039766	LPAR3	lysophosph	GO:0000187
Up	0.193013	0	1.05824	0.884525	TNFRSF25	tumor necrosis facto	
Up	4.34589	3.62173	8.39058	8.58709	FAM101B	family wit	GO:0001837
Up	0.0416118	0.0832271	0.211438	0.133104	KIAA1644	KIAA1644	GO:0016021
Down	0.0321845	0.0509663	0.0006481	0.0012311	TENM3	teneurin t	GO:0005887
Up	0	0	0.467954	0.41833	RP11-757G1.6		
Down	0.252608	0.19385	0.0564093	0.0529487	FKBP9P1	FK506 bind	GO:0003755
Up	0.023633	0	0.212871	0.33321	AC006547.13		GO:0003677
Down	1771.37	1492.89	622.244	643.49	MT2A	metallothionein 2A	
Up	0.635399	3.105	3.94241	3.84057	GIPC1	GIPC PDZ d	GO:0003779
Up	0.161679	0.370852	0.650482	0.925846	GBGT1	globoside alpha-1,3-	
Up	0	0	0.0433708	0.030896	PALM	paralemmin	GO:0005634
Up	0.088498	0	0.289142	0.263392	RP11-347C1	Putative N	GO:0005654
Up	0.415526	0.334402	1.35493	1.21922	PIP4K2B	phosphatid	GO:0005524

Up	0.429887	0.575098	1.1001	1.14818	BDNF	brain-derived neurot	
Down	8.42397	6.97032	1.22521	1.75304	HOXB2	homeobox B	GO:0000978
Up	0.0252816	0.0388297	0.0959402	0.131766	DCLK2	doublecort	GO:0004674
Up	0.0680869	0.100992	0.168262	0.161352	EIF4G3	eukaryotic	GO:0000339
Up	0.0713962	0.0645834	0.164003	0.180184	KIAA2026	KIAA2026	
Down	0.0082849	0.0119676	0	0	GABRB2	gamma-amir	GO:0004890
Up	0.07158	0.143712	0.253772	0.244175	TBC1D16	TBC1 domai	GO:0001919
Up	0.0214874	0	0.142255	0.146699	GPR173	G protein-	GO:0004930
Down	1.31406	1.12828	0.533854	0.401639	XAF1	XIAP assoc	GO:0003964
Down	4.12173	4.17326	1.6512	2.79739	TSEN15	TSEN15 tRN	GO:0000213
Up	0	0	0.136475	0.0551278	PITPNM3	PITPNM fam	GO:0004620
Down	0.0226335	0.02366	0.012054	0.0035795	SLC4A4	solute car	GO:0005452
Up	0.0054937	0.0544812	0.116038	0.103965	GOLGA2P7	golgin A2	GO:0000137
Up	0.564021	0.26705	2.36401	2.78268	QPCT	glutaminyl	GO:0005576
Down	5.07806	3.65769	1.91771	2.12368	SEC23A	Sec23 homo	GO:0000139
Up	0	0	0.0371524	0.047775	HOXB3	homeobox B	GO:0000122
Up	0.65977	0.730905	1.542	2.1604	DYRK2	dual-speci	GO:0000151
Up	0.0251403	0.0301938	0.236726	0.204216	FRMD4A	FERM domai	GO:0005737
Up	0.0295414	0.0182214	1.36872	1.45597	RGS5	regulator	GO:0005096
Down	1.8655	1.99779	0.142011	0.28463	C10orf54	chromosome	GO:0005886
Up	0.001772	0	0.0090273	0.009644	AFF3	AF4/FMR2 family, mem	
Up	5.27296	12.0466	16.9534	19.6048	TRIM28	tripartite	GO:0000122
Up	3.18663	2.66877	6.44821	5.54239	NT5E	5'-nucleotidase, ect	
Up	0	0	0.0105867	0.010436	AR	androgen receptor	
Up	0	0	0.001561	0.0036281	WBSCR17	Williams-Beuren sync	
Up	0	0.401036	1.22706	1.57659	C6orf47-AS	C6orf47 antisense RN	
Down	7.87584	7.90869	4.66594	4.67293	VAPA	VAMP (vesi	GO:0005783
Up	1.20963	2.28495	41.6935	42.4993	RP11-397A15.4		GO:0001527
Up	23.8755	28.257	81.2031	79.7132	MFAP2	microfibri	GO:0001527
Up	0.660707	1.41916	2.29055	2.32815	LAMB1	laminin, b	GO:0005198
Up	0	0	0.242618	0.221227	AC004988.1		GO:0003677
Up	0	0.111151	0.884236	0.512145	RASA4DP	RAS p21 pr	GO:0000165
Up	0.347128	0.911778	0.934587	0.96387	PPM1F	protein ph	GO:0004722
Up	1.06067	0.63386	2.15303	2.36161	INF2	inverted f	GO:0003779
Down	0.382754	0.573164	0.0475161	0.0114018	ANGPTL1	angiopoiet	GO:0005102
Down	1.93223	1.04674	0.671376	0.600383	COQ2	coenzyme Q	GO:0002083
Down	0.139925	0.0316544	0.001761	0	CHRM2	cholinergi	GO:0004930
Down	0.228356	0.225106	0.105721	0.0707698	MEIS2	Meis homeo	GO:0000122
Down	0.0145457	0.0146448	0.0004655	0.0026885	ENOX1	ecto-NOX c	GO:0003676
Up	5.56157	7.23179	13.6134	11.7571	RRAS	related RA	GO:0002521
Down	5.02897	4.13959	2.96485	2.91765	NUDT21	nudix (nuc	GO:0003682
Up	0.708691	1.12953	4.27291	3.40951	ZNF358	zinc finge	GO:0000981
Up	15.5953	13.0126	62.3766	49.0936	RP11-290L1.3		GO:0000086
Down	0.580407	0.463236	0.224408	0.204253	DOK5	docking pr	GO:0005829
Up	0	0	0.238727	0.478235	FUT1	fucosyltransferase 1	
Up	0.358635	0.406471	0.865735	0.986758	C15orf39	chromosome	GO:0005829
Up	0	0	0.688633	0.766358	RP11-169K16.4		
Up	0	0	0.02028	0.0212251	SH3TC2	SH3 domain and tetra	
Down	0.396819	0.400199	0.0975357	0.136949	SEMA7A	semaphorin 7A, GPI m	
Down	16.1477	11.0836	7.3089	4.89096	SOCS1	suppressor of cytoki	
Down	4.70137	5.70547	2.02417	1.93417	WARS	tryptophan	GO:0001525

Down	0.867125	0.553246	0.390743	0.348623	C1D	C1D nucle	GO:000017
Up	0.357892	0.576262	0.884305	0.868093	FZR1	fizzy/cell	GO:000565
Down	25.3104	10.8568	0.323255	0.103423	CXCL5	chemokine (C-X-C mot	
Down	0.0320291	0.0493415	0.0047056	0.0041715	ARHGAP20	Rho GTPase activatir	
Up	0.100365	0.610377	0.597317	0.646219	ZSCAN18	zinc finge	GO:000367
Up	1.10141	1.22736	3.82033	2.89027	SPRY4	sprouty R	GO:000582
Up	0.22577	0.154996	0.44524	0.460307	ZNF800	zinc finge	GO:000098
Up	3.31278	2.979	11.2846	13.7999	PDLIM3	PDZ and L	GO:000701
Down	7.23297	4.89544	3.24896	2.78098	EXOSC2	exosome c	GO:000017
Up	0.0964075	0.139067	0.457555	0.423261	PPP1R16A	protein p	GO:000588
Up	0.857203	1.13193	1.78562	1.518	LARP1	La ribonu	GO:000033
Up	0.228958	0.280188	0.386242	0.542763	ANKH	ANKH inor	GO:000150
Down	2.59852	3.23572	0.524398	0.844426	ETS2	v-ets avian erythrol	
Up	0.319834	0.162753	0.71116	0.680483	PTPN23	protein t	GO:000472
Up	7.34658	18.628	42.9502	32.1239	GADD45B	growth arrest and DN	
Up	1.40477	0.9121	12.4002	16.1559	INHBA	inhibin, beta A	
Down	0.410407	0.273815	0.154207	0.144613	ALG6	ALG6, alp	GO:000458
Down	11.4391	12.0555	6.54776	6.4461	PRMT5	protein a	GO:000038
Down	0.378887	0.291464	0.213599	0.180376	ARHGAP12	Rho GTPas	GO:000189
Down	1.6736	1.23644	0.583676	0.94745	HBP1	HMG-box transcriptio	
Up	0	0	0.0021438	0.0026529	ROBO2	roundabout	GO:000165
Up	0.228056	0.205138	0.878731	0.901847	MMP17	matrix met	GO:000182
Up	0.26362	1.15705	1.13744	1.30511	POLRMT	polymerase	GO:000101
Up	0	0.306362	1.3592	1.3339	RBM24	RNA bindir	GO:000038
Down	4.173	4.28418	2.37907	1.91072	FDFT1	farnesyl-	GO:000431
Down	7.98286	5.09421	3.80781	3.59514	LIN7C	lin-7 hom	GO:000201
Down	0.126238	0.164797	0.0635075	0.0809264	CNTN1	contactin	GO:000715
Up	0.0169534	0.0123171	0.161162	0.154118	RAB11FIP3	RAB11 fami	GO:000550
Up	0.131883	0.138557	0.573668	0.509033	FEZ2	fasciculat	GO:000573
Down	1.858	2.32346	1.03078	0.740982	C12orf29	chromosome	GO:000224
Down	31.8093	25.2692	0.652326	0.53183	DCN	decorin	
Up	5.18767	5.51539	11.7405	11.323	ARMCX2	armadillo repeat con	
Up	0.606417	0	4.67323	4.32577	RP11-114H24.6		
Up	0.841897	1.12879	1.63518	1.49177	STAU1	staufen d	GO:000372
Down	16.6648	16.3913	9.7287	8.14704	AKR1B1	aldo-keto	GO:000189
Up	0.296745	0.652737	0.726251	0.792232	MIER2	mesoderm i	GO:000012
Up	0.0196817	0.0743562	1.96278	1.71775	C5orf46	chromosome	GO:007006
Up	0.202813	0.423435	1.0948	1.21148	UBN1	ubinuclein	GO:000367
Up	0.0388004	0.0627217	0.614741	0.544335	LIMCH1	LIM and c	GO:000172
Down	0.217486	0.125124	0	0.0338512	CH507-254M2.2		
Up	0.511771	3.66336	4.2186	3.80433	SLC5A2	solute ca	GO:000536
Down	0.0307228	0.0175076	0	0	TMEFF2	transmemb	GO:000557
Down	0.981695	1.45806	0.301278	0.338992	ALDH6A1	aldehyde	GO:000006
Down	33.9609	26.247	6.89356	5.83822	RGS2	regulator of G-prote	
Down	18.6016	19.2574	11.16	10.7175	GMPPA	GDP-manno	GO:000573
Down	40.1701	34.9489	18.3916	16.211	RP11-707G14.1		GO:000013
Down	0.276403	0.209623	0.0868141	0.134887	ATL2	atlastin (	GO:000392
Up	0.240374	0.276203	0.724971	0.963771	VLDLR	very low density li	
Up	1.03235	1.62343	2.358	2.13314	RANGAP1	Ran GTPas	GO:000077
Up	0.606196	1.17318	1.77882	1.99826	ITGB5	integrin,	GO:000161
Up	10.4653	7.4643	26.2851	25.2191	CD59	CD59 molecule, compl	

Down	2.26166	2.27433	0	0.0043825	TSIX	TSIX trans	GO:000563
Up	0.0078136	0.0051091	0.0331598	0.0539933	ERG	v-ets avian erythrol	
Down	0.0153455	0.0152842	0.0063331	0.004946	RAD51B	RAD51 paralog B	
Down	0.107786	0.187835	0.0284845	0.0336428	CEMIP	cell migra	GO:000441
Up	1.34215	1.04934	2.71428	2.27892	YWHAG	tyrosine 3	GO:000008
Down	0.0539578	0.0452999	0	0	RP11-543C4.1		
Up	0.396513	1.15807	1.88852	1.45693	PNPLA6	patatin-li	GO:000462
Down	5.99597	5.29176	3.39275	2.89153	BCCIP	BRCA2 and	GO:000005
Up	0.374374	0.330107	0.839381	0.688866	PPP3CB	protein ph	GO:000191
Up	0.514478	0.848427	1.55328	1.0942	PANK3	pantothena	GO:000459
Up	0.82817	0.844807	1.25884	1.62244	CEP170	centrosoma	GO:000581
Up	0.234428	0.186454	1.27065	1.74581	POLM	polymerase (DNA dire	
Down	0.0308228	0.0229076	0	0.0043576	CH17-360D5.2		
Down	1.84463	1.58543	0.910424	0.760043	HEXB	hexosamini	GO:0001501
Down	6.92177	5.97961	3.70671	4.21228	FBXO7	F-box prot	GO:0000151
Up	0.0942541	0.228913	0.449959	0.496732	TPRN	taperin	GO:000760
Down	10.8776	7.95548	6.29227	6.01744	ACP1	acid phosp	GO:000399
Up	0.0068887	0.0143404	0.0303155	0.0289629	SIPA1L3	signal-ind	GO:000165
Down	0.0619448	0.0351035	0	0	GNAZ	guanine nu	GO:000166
Up	0.232951	0.377176	2.47197	2.91091	PPP1R13L	protein phosphatase	
Down	0.569456	0.693991	0.0216202	0.0046938	FENDRR	FOXF1 adja	GO:000396
Up	0.858934	0.564738	2.04959	2.19628	PURA	purine-ric	GO:000078
Down	6.88581	5.40209	2.93709	3.00082	COMMD8	COMM domai	GO:000565
Down	1.23833	1.14226	0.448176	0.620778	SPTLC1	serine pal	GO:000475
Down	0.0339149	0.0141616	0.0063026	0	KLHL3	kelch-like	GO:000377
Up	0.118567	0.15213	0.60747	0.478027	AMOTL1	angiomotin	GO:000336
Up	0.0364443	0.0516317	0.422233	0.414689	TMEM184A	transmembr	GO:000576
Up	0	0	0.21351	0.38937	PCDHB16	protocadhe	GO:000550
Up	1.37025	1.47403	2.71566	2.60198	DYNC1H1	dynein, cytoplasmic	
Up	0.0760988	0.234906	0.424421	0.324872	DOT1L	DOT1-like	GO:000007
Up	0	0	0.0216999	0.0164889	SLC47A1	solute car	GO:000588
Up	11.8816	15.55	69.8552	78.8115	IGFBP7	insulin-like growth	
Down	0.397493	0.485846	0.0589706	0			
Down	0.761122	0.622304	0.370071	0.154208	ZFX	zinc finge	GO:0000981
Up	0.415653	0.434856	0.776818	0.869411	TTL	tubulin ty	GO:000022
Up	1.54027	1.66782	3.09145	3.00164	UACA	uveal auto	GO:000557
Up	0.177641	0.793139	0.980895	1.02216	PKD1	polycystic kidney di	
Down	1.36203	0.903074	0.427276	0.421528	CUL2	cullin 2	GO:000565
Up	0.0060138	0	0.0720436	0.0554634	RP11-22H5.2		GO:000367
Down	213.286	142.149	73.3121	60.9034	B2M	beta-2-mic	GO:000247
Down	0.313538	0.261293	0	0	FDCSP	follicular	GO:000557
Down	0.454259	1.44158	0	0	RP11-60C6.5		GO:0000381
Down	1.44697	1.93619	0.111583	0.0480298	C1S	complement component	
Down	2.98592	2.09999	1.04937	1.7238	MFSD11	major faci	GO:0016021
Up	1.1144	4.09901	4.53447	3.94301	KCNK6	potassium	GO:000308
Up	0.0017729	0.0055817	0.055396	0.0930582	SGIP1	SH3-domain	GO:0002021
Down	0.922126	2.0169	0.448811	0	HOXA5	homeobox A	GO:000097
Up	0.0157712	0.0391012	0.734613	0.607318	RASA4	RAS p21 pr	GO:000016
Down	1.35624	1.18988	0.744353	0.636434	PPP2R1B	protein ph	GO:000015
Up	0.134959	0.268351	0.588191	0.678356	SRRM5	serine/arginine repe	
Up	0.0091312	0	0.207265	0.0743716	RASGRP2	RAS guanyl releasing	

Up	0	0	0.0266868	0.0414015	NLRP2	NLR family	GO:0005524
Down	2.64903	3.32833	0.886269	0.685067	PLOD2	procollagen-lysine,	
Up	0.400275	0.640728	0.945603	1.37355	GTPBP1	GTP bindir	GO:0000177
Up	11.4251	4.47582	24.7278	19.8205	TMSB15A	thymosin b	GO:0003785
Down	0.435714	1.0974	0	0	LRRN4CL	LRRN4 C-te	GO:0016021
Down	1.67709	1.3577	0.670843	0.778816	PRPS2	phosphorib	GO:0000287
Down	0.564012	0.426161	0.242177	0.354483	NNT	nicotinami	GO:0001933
Down	0.0072563	0.0308406	0	0	PRKAA2	protein ki	GO:0003682
Down	1.05046	0.58773	0.353118	0.210114	LYSMD2	LysM, putative pepti	
Up	1.63045	1.78935	3.17821	2.94359	VCL	vinculin	
Down	0.386039	0.460167	0.165572	0.117027	RABGEF1	RAB guanir	GO:0003677
Down	0.349523	0.297588	0.211479	0.146627	MGST2	microsomal glutathic	
Down	0.238061	0.148977	0.101295	0.0808188	PTBP3	polypyrimi	GO:0003723
Down	1.7786	1.70141	0.731468	0.874573	DTWD1	DTW domain containir	
Down	8.28261	14.7883	1.86	2.26756	PIM1	Pim-1 proto-oncogene	
Down	13.2059	20.2091	5.7337	2.12502	MFAP4	microfibri	GO:0001527
Down	0.151504	0.0979994	0.0521479	0.0518729	LOH12CR1	loss of he	GO:0031224
Down	0.304884	0.775352	0.0271709	0.0185815	LSP1	lymphocyte	GO:0003779
Up	0.0715167	0	0.91813	0.81472	BCL7A	B-cell CLL	GO:0045892
Up	0.35323	1.05947	1.12714	1.10051	SMARCA4	SWI/SNF related, mat	
Down	0.353972	0.241315	0.115787	0.0420785	RP11-568K15.1		GO:0005739
Down	60.8049	78.3903	32.6091	35.455	NUPR1	nuclear pr	GO:0002526
Down	6.02487	11.3904	0.301591	0.248671	WFDC21P	WAP four-c	GO:0005615
Down	0.853601	0.832013	0.234659	0.415507	TATDN3	TatD DNase	GO:0004518
Down	0.47344	0.411229	0.25449	0.287943	PAM	peptidylgl	GO:0001519
Up	0.0095398	0.0324367	0.0561397	0.106037	DAGLA	diacylglyc	GO:0005886
Down	7.9762	6.3473	4.21284	4.23261	TMEM183A	transmembr	GO:0016021
Down	0.227365	0.276451	0.0725023	0.0500834	GUCY1B3	guanylate	GO:0004383
Up	0.167466	0.213631	0.603369	0.808586	GOLGA8A	golgin A8	GO:0000137
Up	0.39933	0	1.93369	1.5076	CHRNE	cholinergi	GO:0005886
Down	10.6726	12.0301	6.62174	6.4032	RPN1	ribophorir	GO:0003723
Up	0.305814	0.433255	0.899346	0.657137	MEF2D	myocyte enhancer fac	
Up	0.0216651	0.0257568	0.189523	0.184804	SVIL	supervilli	GO:0002102
Up	0.0024906	0	0.0541762	0.0712358	CCDC102B	coiled-coil domain c	
Up	0.0168262	0.0715146	0.15737	0.125625	FAM219A	family with sequence	
Down	0.0517772	0.315423	0	0	TBX4	T-box 4	GO:0000981
Down	0.757499	0.653174	0.232186	0.503549	6-Mar	membrane-a	GO:0000835
Down	0.0760157	0.163311	0.003152	0.0046493	GAS7	growth arrest-specif	
Down	1.32259	1.54403	0.887594	0.640454	AP5M1	adaptor-re	GO:0005764
Up	0.109825	0.209291	0.359099	0.291491	SGSM2	small G pr	GO:0005096
Down	0.111485	0.216107	0.017717	0.0036716	COL11A1	collagen,	GO:0001502
Up	2.62518	5.40969	8.15567	7.43204	CTD-3252C9.4		GO:0016021
Up	1.18337	2.8958	3.24455	4.96947	MAFG	v-maf avia	GO:0000978
Up	0.322662	0.489316	2.23501	1.54589	RP11-861E21.2		GO:0003677
Down	0.0685063	0.0742184	0	0	DGAT2	diacylglyc	GO:0003846
Down	5.52164	6.50987	2.77428	3.57187	MARS	methionyl-	GO:0000049
Down	1.11067	1.79396	0.0236814	0.0128533	ICAM1	intercellular adhesi	
Up	0.288876	0.872191	1.41831	1.72888	ALDH16A1	aldehyde c	GO:0016020
Down	0.268419	0.329048	0.108298	0.110943	PRMT3	protein ar	GO:0003676
Down	3.41207	2.09764	1.0293	1.10498	TPMT	thiopurine	GO:0005829
Down	0.0035266	0.0064534	0.0001621	0	CNTNAP2	contactin	GO:0005769

Up	0.0142853	0	0.220173	0.156655	MYCT1	myc target	GO:000565
Down	209.519	296.525	14.5645	19.886	KRT19	keratin 19	GO:000520
Down	0.257486	0.249457	0.104557	0.131163	MEIS1	Meis home	GO:000097
Down	0.105323	0.127054	0	0.0199845	CXADR	coxsackie	GO:000161
Down	0.292096	0.424346	0.0034517	0	GFRA1	GDNF famil	GO:000016
Down	0.550115	0.634839	0.0292005	0.0245871	SLC38A4	solute car	GO:000333
Up	0	0	0.561347	0.345299	GIPC3	GIPC PDZ domain cont	
Up	0.329922	0.400225	0.809754	0.575516	OSBPL8	oxysterol	GO:000178
Up	0.259966	1.43381	2.07038	1.87692	KIF1C	kinesin fa	GO:000372
Up	0	0	0.128436	0.217225	KRT79	keratin 79	GO:000519
Up	0	0	7.78996	14.2114	BEX1	brain expressed, X-l	
Down	16.3107	14.0415	7.647	9.02393	MYD88	myeloid di	GO:000222
Up	0.11625	0	3.43582	1.84493	KCNS2	potassium	GO:000524
Up	0.0399139	0.05163	0.275188	0.175432	PACS2	phosphofur	GO:000004
Down	0.055596	0.0393822	0.0056087	0.0098554	KLHL4	kelch-like	GO:000377
Down	0.0137479	0.0272301	0.0055544	0.0006395	NCAM1	neural cell adhesion	
Up	0.092557	0.0636908	0.376829	0.337651	EDNRA	endothelin receptor	
Down	27.5689	22.7606	12.4152	13.9238	PGRMC1	progestero	GO:000154
Down	3.45607	7.7896	0.346676	0.827909	REP15	RAB15 effe	GO:000188
Up	0.0142455	0.0048052	0.0831903	0.0732882	SORBS1	sorbin and	GO:000172
Up	0.007621	0	0.0640682	0.100611	TLE2	transducin	GO:000371
Down	0.22798	0.160918	0.087986	0.0660798	KLF3-AS1	KLF3 antisense RNA 1	
Up	0.165562	0.434104	0.629451	1.06077	TUT1	terminal u	GO:000372
Down	1.07287	1.65247	0	0.0263597	DMRT2	doublesex	GO:000097
Down	2.75432	2.43961	1.4532	1.39607	SMN1	survival of motor ne	
Down	12.5729	10.9023	5.40817	5.54893	TRAM1	transloca	GO:000578
Up	0.0023952	0	0.0795364	0.0589066	RGS7BP	regulator	GO:000563
Down	26.6504	21.436	9.17357	9.35314	NQO1	NAD(P)H dehydrogenas	
Down	0.402578	0.405578	0.081226	0.156016	HSD17B14	hydroxyste	GO:000430
Up	0.181009	0.196423	0.631352	0.981621	STXBP2	syntaxin b	GO:000190
Up	0.102396	0.185837	0.347706	0.439278	ST3GAL2	ST3 beta-galactoside	
Up	0	0	0.217467	0.315735	CLEC18A	C-type lec	GO:000561
Up	3.21846	6.83952	17.1295	15.5331	RP11-148K1.12		GO:000545
Up	2.75475	1.11154	5.34265	6.38188	FZD4	frizzled c	GO:000154
Up	0	0	0.0509971	0.0775014	RP11-367G6.3		
Up	0	0	0.0057633	0.0100099	NEK10	NIMA-relat	GO:000467
Up	0.37931	0.58234	2.97474	3.32108	APCDD1L	adenomatos	GO:001602
Up	0	0	0.0638389	0.048144	ANO1	anoctamin	GO:000522
Down	0.268146	0.331953	0.104149	0.0979648	FAM120B	family wit	GO:000563
Up	0.218051	0.169501	0.471047	0.475855	ANKRD17	ankyrin re	GO:000078
Up	0.346816	0.575232	3.38142	3.76091	CLCF1	cardiotrop	GO:000263
Up	0.0774948	0.0729206	0.209372	0.19912	SNHG14	small nucleolar RNA	
Down	2.05479	1.83709	0.911936	1.02588	BTBD1	BTB (POZ)	GO:000093
Up	0.115188	0.506848	0.467379	0.668735	DEAF1	DEAF1 tran	GO:000012
Down	0.147082	0.104484	0.0528417	0.0393493	PROS1	protein S (alpha)	
Up	0.866937	0.861016	3.83195	5.55802	TESK1	testis-spe	GO:000467
Up	0.736817	2.64163	4.09583	3.01861	KLF4	Kruppel-like factor	
Up	0.194035	0.486352	1.43163	1.18885	EFNB2	ephrin-B2	GO:000161
Down	0.0113505	0.0080403	0	0	CCNB3	cyclin B3	GO:000007
Down	0.162747	0.271488	0.0014009	0.0014637	IQGAP2	IQ motif c	GO:000377
Down	0.0285919	0.0359038	0.0151596	0.0043586	CPNE8	copine VII	GO:000554

Up	1.31165	1.71674	2.09323	2.70887	NID2	nidogen 2	GO:0005201
Up	0.0164234	0.0351945	0.0462938	0.0681056	GRIA3	glutamate	GO:0001540
Up	0.513809	0.927434	1.98098	1.80705	YY1	YY1 transc	GO:0000122
Up	0.117091	0.182715	0.574571	0.492693	LRP12	low densit	GO:0001764
Up	0.125691	0	1.37367	3.01095	HTR1B	5-hydroxyt	GO:0002031
Up	0.110946	0.298642	0.647064	0.542176	DBNDD1	dysbindin	GO:0005737
Down	0.257592	0.249226	0.0554589	0.124918	ABHD3	abhydrolas	GO:0004623
Down	9.6679	7.01844	5.41577	5.12573	SDHB	succinate dehydrogen	
Up	0.889768	0.896212	1.89116	1.87323	WASF2	WAS protei	GO:0001525
Up	9.81674	13.4525	33.7614	34.6158	RHOB	ras homolo	GO:0000281
Up	0.17888	0.608218	1.41108	1.40932	ZMIZ2	zinc finge	GO:0005634
Down	5.01015	6.68049	0.078617	0.233521	TUBB2B	tubulin, b	GO:0000226
Up	0.157539	0.250977	0.818581	0.813582	DAG1	dystroglycan 1 (dyst	
Up	0.0858603	0.0995242	0.366649	0.534767	ABCA2	ATP-binding cassette	
Up	0.91574	1.53193	3.16178	3.03067	TRIM56	tripartite	GO:0003723
Up	0	0	0.206211	0.334276	GPC2	glypican 2	GO:0001523
Down	28.9141	21.157	9.83447	11.984	CD164	CD164 mole	GO:0005576
Down	0.228468	0.514335	0.0315731	0.0247409	NDRG2	NDRG family member 2	
Up	0.206197	0.193157	0.448546	0.553288	SLC20A2	solute car	GO:0001618
Up	6.875	4.95105	13.2273	12.4379	PNP	purine nucleoside ph	
Up	0.089525	0.0954112	0.20721	0.282636	SH3PXD2A	SH3 and PX	GO:0002020
Up	0.259595	0.519213	1.11411	0.947952	PKN1	protein ki	GO:0001782
Up	9.76512	11.8928	26.5455	25.6907	RP13-1032I1.7		GO:0005654
Up	4.11528	5.25412	9.47234	8.79368	HYOU1	hypoxia up	GO:0002931
Down	1.92049	2.13222	0.711709	0.676	HAS2-AS1	HAS2 antisense RNA 1	
Down	118.342	139.717	11.1151	13.8927	TGFBI	transforming growth	
Down	0.368737	0.192147	0	0	RP11-89N17.4		
Up	0.0107744	0.0143664	0.887936	0.88787	SULT1B1	sulfotrans	GO:0003964
Up	0.0228115	0.0574537	0.103877	0.258655	CEP131	centrosome	GO:0000086
Up	0.522919	0.472124	1.07071	1.31975	KLHL21	kelch-like	GO:0004842
Up	0.0643228	0.425264	0.76156	0.749887	C21orf58	chromosome 21 open r	
Up	0.0648428	0	0.436099	0.507418	RP11-380J14.1		
Down	1.41851	1.90512	0.956253	0.749941	PARL	presenilin	GO:0004175
Up	0.23128	0.355428	0.692051	0.611316	SRCAP	Snf2-relat	GO:0000812
Down	0.619684	0.966702	0.13902	0.217388	PRSS12	protease,	GO:0004252
Down	4.59933	3.36438	1.98614	2.06803	CYB5R2	cytochrome	GO:0004128
Down	1.0473	0.729939	0.443557	0.565851	CEP57	centrosomal protein	
Up	0.0127444	0	0.501417	0.989154	LINC00643	long intergenic non-	
Down	2.28536	2.30696	1.31658	1.42105	C6orf89	chromosome	GO:0000139
Up	0.177954	0.916773	1.95845	2.43374	FOXC2-AS1	FOXC2 anti	GO:0000981
Down	0.0439454	0.0374891	0	0.0122251	BAALC	brain and acute leuk	
Down	11.93	9.73867	7.59201	6.40956	C20orf27	chromosome 20 open r	
Up	0.384125	0.7159	1.05038	1.03335	BAZ2A	bromodomai	GO:0000183
Up	0	0	0.0886406	0.148943	USP9Y	ubiquitin	GO:0004197
Up	0.045774	0	0.850443	0.67351	GALNT6	polypeptide N-acetyl	
Up	0	0	0.131282	0.288185	RP11-632K5.2		
Up	0.0635519	0.0771735	0.269044	0.318253	FOXP4	forkhead b	GO:0000981
Up	0.0460423	0.181508	0.382443	0.361576	DHX37	DEAH (Asp-	GO:0000462
Down	4.2006	3.71493	2.23922	1.48195	THAP1	THAP domai	GO:0000122
Up	0.0736045	0.134004	0.311211	0.278603	MTCL1	microtubul	GO:0000922
Up	0	0	0.0492539	0.0389332	NLGN4Y	neuroligin	GO:0005886

Up	4.29319	5.37941	9.9194	9.68686	CALD1	caldesmon	GO:0003779
Down	8.97129	9.25216	2.84258	2.3936	SCD	stearoyl-(	GO:0004768
Up	0.488712	0.820589	3.01673	1.96854	CXCR5	chemokine (C-X-C mot	
Up	0.138415	0.107183	0.384867	0.507454	PQLC1	PQ loop re	GO:0005768
Down	56.2162	58.1949	22.138	22.9602	SURF4	surfeit 4	GO:0000139
Up	0.381051	0.397595	0.751446	0.804652	UBR4	ubiquitin	GO:0004842
Up	0.123123	0.108846	0.272951	0.217787	CNNM2	cyclin anc	GO:0005524
Down	0.376686	0.45689	0.165004	0.0962516	TAF1A	TATA box b	GO:0000120
Up	0.0550336	0.155935	1.29545	1.13597	GDF6	growth dif	GO:0005125
Up	0.0267944	0.0404911	0.13628	0.169452	ZNF736	zinc finge	GO:0000981
Down	2.2544	2.59841	0.192737	0	RP11-697N18.3		GO:0000045
Up	6.12798	7.08356	19.556	16.7879	TXNIP	thioredoxi	GO:0000122
Up	0.446994	0.773523	1.69102	3.33164	ADGRE5	adhesion G protein-c	
Up	0.203935	0.520057	1.87733	1.09431	RP11-5407.3		
Up	0.294801	0.737558	1.13108	1.20806	SMG9	SMG9 nonse	GO:0000184
Down	8.96913	8.98686	4.86836	3.00177	HOXC8	homeobox (	GO:0000122
Up	0.238939	0.401982	0.894509	0.91581	ARL8A	ADP-ribosy	GO:0003924
Up	0.0206833	0	0.0583295	0.0817981	DAAM2	dishevelle	GO:0003779
Down	1.29805	1.7717	0.653752	0.462632	C19orf54	chromosome 19 open r	
Up	0	0	0.0064506	0.0160661	ADCY1	adenylate	GO:0003091
Down	30.4311	29.2365	16.7951	15.2109	C6orf120	chromosome	GO:0005576
Up	0.160696	0.0578189	0.524295	0.631805	TATDN2	TatD DNase	GO:0005634
Up	0.0136433	0	0.647324	0.678168	WNT5A	wingless-t	GO:0000187
Down	0.0321211	0.0278475	0.0084639	0.0043641	PRICKLE2	prickle ho	GO:0005737
Down	17.226	13.1851	0.662868	0.454145	DDIT4L	DNA-damage	GO:0005737
Up	0.257024	1.0487	1.15385	1.18215	KLHL35	kelch-like family me	
Up	0.526055	0	5.14112	5.55989	NOV	nephroblas	GO:0001525
Up	0.244	0.258558	0.607876	0.411213	IREB2	iron-respo	GO:0001069
Up	0.0776093	0.166413	0.203141	0.244804	SCAF8	SR-relatec	GO:0000993
Down	0.0402384	0.02113	0.0007837	0.0003722	NCAM2	neural cel	GO:0005886
Up	0.992072	1.36203	3.01795	2.94493	FAM189B	family wit	GO:0016021
Up	0.244118	0.230947	0.517476	0.536653	SPRED1	sprouty-re	GO:0000165
Down	2.68888	1.57299	1.18779	0.718308	TXNRD1	thioredoxin reductas	
Down	10.5524	7.42412	5.28598	5.49151	DENR	density-regulated pr	
Up	0.0367955	0.208517	0.603203	0.966061	ISL2	ISL LIM ho	GO:0000981
Down	0.0923767	0.0509766	0.0113436	0.0256929	GALNT14	polypeptide N-acetyl	
Down	77.0901	71.7517	30.2699	38.4191	HIST1H1C	histone cl	GO:0000122
Up	0.111215	0.142723	0.277031	0.256381	MICAL2	microtubul	GO:0001947
Down	16.7633	19.1132	7.83334	9.98469	POLD2	polymerase (DNA dire	
Up	0	0	2.38862	1.85865	SHISA3	shisa fami	GO:0005789
Down	0.092347	0.0297342	0.0119099	0	CHRNA1	cholinergi	GO:0003009
Up	0.0614643	0.0339817	0.223073	0.287295	RNF19B	ring finge	GO:0000151
Up	0.0787783	0.342609	0.505949	0.549467	ADCK2	aarF domai	GO:0004674
Down	0.190725	0.124623	0.0110927	0	EPSTI1	epithelial stromal i	
Down	3.40691	1.18479	0.52729	0.259523	NXT2	nuclear tr	GO:0005654
Up	0.175945	0.197402	0.473122	0.451563	BMPR2	bone morph	GO:0001707
Up	0.0321826	0.143095	0.250991	0.36673	ST6GAL2	ST6 beta-galactosami	
Up	0.4057	1.45204	1.43382	1.62659	ZNF526	zinc finge	GO:0000981
Down	0.0234337	0.0619719	0	0	SLC27A2	solute car	GO:0001561
Down	1.41192	1.04381	0.424346	0.492151	DFNA5	deafness,	GO:0005546
Up	0.0056406	0.0179004	0.0598915	0.0548056	SH3RF3	SH3 domair	GO:0046330

Down	5.6704	3.83314	1.55916	1.91706	ISOC1	isochorismGO:0003824
Down	0.187714	0.146154	0.088851	0.0756671	MIPEP	mitochondrGO:0004222
Up	0.2381	0.408643	1.23532	1.09903	VPS37B	vacuolar pGO:0000813
Up	0	0.004924	0.0112311	0.0236249	CDH8	cadherin 8GO:0005509
Up	102.76	78.5051	242.372	240.628	KRT18	keratin 18, type I
Down	0.992513	0.766828	0.51553	0.515349	DCUN1D4	DCN1, defeGO:0000151
Down	2.43913	2.31757	1.25231	1.45665	SNX1	sorting neGO:0005154
Up	0.884591	1.15893	1.79608	2.00484	RNF168	ring fingeGO:0000151
Up	0.00346	0	0.0464682	0.0558651	KIAA1671	KIAA1671
Down	12.8009	9.79308	4.4378	5.84549	PSAT1	phosphoserGO:0004648
Up	0.0238643	0	0.148462	0.211901	RAMP1	receptor (GO:0001525
Up	37.8859	43.6971	71.1489	64.6157	FAM127C	family with sequence
Down	0.493314	0.236955	0.0084141	0.0110822	ANXA10	annexin A1GO:0005509
Up	3.00947	4.21096	8.98391	9.59451	CD3EAP	CD3e molecGO:0000120
Up	0	0	0.0900188	0.102603	NFE4	nuclear faGO:0000987
Down	3.17091	2.15898	1.50606	1.62225	GART	phosphoribGO:0003360
Up	0.202934	0.237765	0.546363	0.601408	BTBD2	BTB (POZ) GO:0000932
Down	0.39364	0.488635	0	0.0323304	CD74	CD74 molecGO:0000139
Down	0.772482	0.856453	0.33617	0.249582	ATP2C1	ATPase, CaGO:0000139
Down	4.69547	5.99207	2.00384	2.21527	RBM7	RNA bindinGO:0000381
Up	0.0137997	0.0289636	0.105055	0.181693	OLFM2	olfactomecGO:0005576
Up	0	0	0.21599	0.307729	SERPIND1	serpin pepGO:0004866
Up	0.0150365	0.0528894	0.109846	0.124456	RAPGEF1	Rap guanine nucleoti
Up	0.0538015	0.110869	0.447162	0.414767	CCDC88B	coiled-coiGO:0001819
Down	11.6197	15.6995	7.2585	6.71302	CBX3	chromobox GO:0000775
Down	0.0521532	0.0901059	0	0	TMPRSS2	transmembrane protea
Down	39.2813	29.9959	18.3153	18.6386	CNN3	calponin 3GO:0003779
Down	15.6904	17.6299	0	0	AC000032.2	
Down	0.251695	0.158481	0.0657848	0.0973979	LIN9	lin-9 DREAGO:0000003
Up	0.136152	0.215319	0.686766	0.418691	AC005786.7	
Down	36.9611	27.7101	21.5066	20.9562	ODC1	ornithine decarboxyl
Up	0.748822	0.922502	2.16057	1.68073	YTHDF1	YTH N(6)-mGO:0003723
Down	2.16509	2.66898	1.15016	1.16865	CREG1	cellular rGO:0003714
Up	0.566244	2.38748	3.32167	4.21471	MICAL1	microtubulGO:0001933
Up	0.35217	0.436249	1.38194	1.18108	ATP13A3	ATPase typGO:0005524
Down	6.06669	6.31771	3.90361	3.01806	TMEM120A	transmembrGO:0005637
Up	0.167352	0.386244	0.873302	0.664755	MLLT1	myeloid/lymphoid or
Up	0.0469322	0.167975	0.341599	0.381857	NOTCH1	notch 1
Up	0.825836	2.22701	8.63287	8.93375	FURIN	furin (paired basic
Down	0.482961	0.271428	0	0	GRP	gastrin-releasing pe
Down	3.66173	2.31516	0.515181	0.869926	RP11-463J10.3	
Up	1.78569	1.74202	3.22512	3.62247	FERMT2	fermitin family memb
Down	0.681349	0.519825	0.0623354	0.0776417	JAM3	junctionalGO:0001525
Down	0.0369106	0.0381359	0	0	RBM47	RNA bindinGO:0002244
Up	202.554	531.318	610.363	594.096	COL1A1	collagen, type I, al
Up	2.5222	1.75017	7.26986	6.88045	RP11-185E8.2	
Up	0.0580223	0.109602	0.330845	0.23367	MMP24	matrix metGO:0004222
Up	20.2444	16.563	41.9516	45.8392	OR7E38P	olfactory receptor,
Down	0.483576	0.450763	0.220428	0.221981	FAM46A	family witGO:0003723
Down	5.7876	5.29802	2.45304	2.62724	SARAF	store-operGO:0005783
Down	0.178794	0.105268	0.0146405	0.0266994	C15orf48	chromosomeGO:0005634

Down	1.06745	0.838205	0.632192	0.546854	ZC3H14	zinc finge	GO:0003723
Down	12.8455	8.26819	3.98956	3.85785	CENPM	centromere	GO:0000777
Down	0.018353	0.0558544	0	0	ZMIZ1-AS1	ZMIZ1 antisense RNA	
Down	0.343124	0.315117	0.153648	0.170817	MICU1	mitochondr	GO:0005509
Down	0.238333	0.253563	0.124939	0.0866239	LZTFL1	leucine zi	GO:0005829
Up	0.0522103	0.0479791	2.19047	1.86322	PTGES3L-AA	PTGES3L-AA	GO:0002196
Down	0.0329635	0.0223169	0	0.0002795	ZNF521	zinc finge	GO:0000981
Down	0.668603	0.947229	0.13383	0.193215	SYT15	synaptotag	GO:0000149
Up	20.1035	25.611	63.7381	61.4237	AC005944.2		
Down	2.71393	1.92245	1.69396	1.13585	SPAG5	sperm asso	GO:0000070
Down	3.5852	2.79699	1.78508	1.44837	RP11-77H9.2		GO:0000978
Down	4.44758	4.86896	1.86101	2.4466	IVNS1ABP	influenza	GO:0005654
Down	2.30341	2.10752	0.692453	0.830116	RCN1	reticuloca	GO:0001701
Down	0.0088981	0.0088335	0.0003276	0.0013692	CSMD2	CUB and Su	GO:0005886
Up	0	0	0.0175745	0.0150235	SPSB4	splA/ryanc	GO:0004842
Up	0.821003	1.07367	3.47426	1.85326	SPATA2L	spermatogenesis asso	
Down	4.7669	11.4637	0.717608	0.39589	FMOD	fibromodul	GO:0005576
Down	0.150902	0.190917	0.077002	0.0578383	KIZ	kizuna cen	GO:0005737
Up	1.45169	1.34862	5.12173	4.11984	MEPCE	methylphos	GO:0000122
Up	0.262053	0.443581	1.1802	1.03049	GSK3A	glycogen s	GO:0003073
Down	71.1898	47.0056	23.0203	22.232	YIPF5	Yip1 domai	GO:0005654
Up	0.11023	0.768819	2.18663	1.55185	MEX3A	mex-3 RNA	GO:0000932
Down	9.2606	7.02324	5.03747	4.78878	DDX1	DEAD (Asp-Glu-Ala-As	
Up	0.0233241	0.0305021	0.0965331	0.0868187	HOMER1	homer scaf	GO:0003009
Down	0.28301	0.315276	0.0097609	0.0069534	SEPP1	selenoprotein P, pla	
Up	0.149936	0.169935	1.26679	1.79108	FAM109A	family wit	GO:0001881
Down	1.75242	1.6348	0.769648	0.858157	TMEM39A	transmembr	GO:0016021
Down	7.23747	6.01109	3.57727	3.5535	RBBP7	retinoblas	GO:0006260
Up	0	0	0.0816126	0.042577	AC003092.1		
Up	0	0	0.0051042	0.0056884	TOX	thymocyte	GO:0000981
Up	0.420663	0.326343	1.17471	0.734691	RAB31	RAB31, mem	GO:0003924
Up	0.0792788	0.33407	0.863868	0.672003	VASH1	vasohibin	GO:0001525
Down	0.29132	0.161443	0.0382428	0.0792524	RP11-745O1	Uncharacte	GO:0000139
Up	0.299618	0.102904	0.97319	0.978736	LINC00665	long inter	GO:0005829
Up	0.194206	0.101277	0.863158	0.857953	IQCG	IQ motif c	GO:0002177
Down	3.86988	3.79968	0.942081	0.865565	PLIN2	perilipin	GO:0005576
Up	0.641501	2.81799	3.74615	4.49261	KRT7	keratin 7,	GO:0005198
Down	0.185278	0.177458	0.0059233	0.0110022	ANXA8L1	annexin A8	GO:0005509
Up	0	0	0.0082796	0.0120988	DNAJC6	DnaJ (Hsp4	GO:0004725
Down	5.34346	4.71161	2.88842	3.17411	TSPAN6	tetraspani	GO:0005887
Up	0.117356	0.248669	0.320858	0.295307	KIAA0319L	KIAA0319-l	GO:0000139
Down	0.0774471	0.233158	0	0.0092763	UNC13D	unc-13 homolog D (C.	
Down	0.0378684	0.0304825	0.0065118	0.0059789	SPEF2	sperm flag	GO:0005794
Down	10.089	7.57232	4.2316	5.55536	PLRG1	pleiotropi	GO:0000398
Up	0.279441	0.285262	0.611991	0.608494	JADE1	jade famil	GO:0000123
Up	0.10568	0.259357	0.462757	0.454489	STK11	serine/threonine kin	
Up	0.0762072	0	0.523742	0.39432	AC002117.1		
Up	0.171493	0.161973	0.481774	0.37795	MAML1	mastermind	GO:0002193
Down	0.256525	0.334352	0.108368	0.0921766	ZNF484	zinc finge	GO:0000981
Up	0.0997924	0.212068	0.600367	0.334676	CCL26	chemokine (C-C motif	
Up	0.051844	0.0506992	0.137696	0.130018	XYLT1	xylosyltra	GO:0000137

Down	0.836133	1.99352	0.164623	0.209776	KCNE4	potassium	GO:000524
Up	0.111713	0.398115	0.601253	0.613573	IGHMBP2	immunoglob	GO:000004
Up	0.0394484	0.0378316	0.117476	0.145093	TECPR2	tectonin b	GO:000691
Up	263.262	365.431	2023.19	2409.86	CTGF	connective tissue gr	
Up	0.251151	0.881061	1.62879	1.79918	RP11-638I2.6		
Up	15.3041	34.4818	38.1667	39.529	EEF2	eukaryotic	GO:000203
Up	0	0	0.0154591	0.0359745	CTD-2192J1	Uncharacte	GO:000012
Down	19.0222	10.4935	4.03329	4.83907	HIST1H2AI	histone cl	GO:000078
Up	0.322456	0.818346	1.37818	2.76824	PHLDB1	pleckstrin	GO:001047
Up	0	0	0.0425681	0.0144524	VSNL1	visinin-li	GO:000550
Up	0.570074	0.989433	3.16997	2.31198	LTBP3	latent tra	GO:000550
Down	21.1596	20.4458	10.6234	9.75123	BZW1	basic leucine zipper	
Down	3.2392	1.94206	1.36088	1.32141	CDC23	cell division cycle	
Up	0.397945	0.501138	1.04894	1.42993	PPP1R3B	protein ph	GO:000016
Down	0.167671	0.13574	0	0	KB-1471A8.1		GO:000097
Up	0.097618	0	0.824764	0.958767	ZNF853	zinc finge	GO:0000981
Up	0.0240593	0.0908945	0.191372	0.277236	MDGA1	MAM domain	GO:000176
Up	0.954686	1.06341	2.92541	2.50836	SEPN1	selenoprot	GO:000301
Down	0.0086316	0.0095289	0.0004948	0.0004297	RIMS2	regulating	GO:000688
Up	0.204213	0.15107	0.875942	0.810184	ERCC6	excision repair cros	
Up	3.66827	6.1912	10.475	8.97105	MYO1C	myosin IC	GO:000014
Up	0.462578	0.465268	1.45705	1.51778	CSK	c-src tyrosine kinas	
Up	0.885552	24.9316	32.0994	36.8123	IER2	immediate	GO:000097
Down	0.017646	0.0439992	0	0	GATSL2	GATS prote	GO:000582
Down	0.122929	0.100415	0.0111724	0.0175096	BOC	BOC cell a	GO:000565
Up	0.425098	0.585236	1.24172	1.47054	MAP3K11	mitogen-ac	GO:000016
Down	0.0698195	0.125681	0.015192	0.0267606	BMP6	bone morphogenetic p	
Up	0.0105603	0	0.238039	0.178979	CDCP1	CUB domain	GO:000557
Down	1.09966	0.289845	0.0644978	0.0245047	SLC9A7P1	solute car	GO:000013
Down	0.778493	0.640092	0.444367	0.395338	SIAE	sialic aci	GO:0001681
Down	13.3788	11.8508	6.94754	7.99118	CTB-43P18.1		
Down	0.121257	0.116618	0.0451139	0.0233591	DISP1	dispatched	GO:000722
Down	0.0028153	0.0015641	0	0	GRID1	glutamate	GO:000497
Up	0.091939	0	0.877814	0.811263	HAPLN1	hyaluronan	GO:000554
Up	0.748679	1.83521	2.62154	2.2823	TPGS1	tubulin po	GO:000573
Down	0.018245	0.0678515	0	0	TRIM29	tripartite	GO:000012
Up	0.275505	0.230729	1.02172	0.914216	COL7A1	collagen,	GO:000013
Up	2.31884	2.2765	5.43441	5.69171	LGMN	legumain	GO:000301
Up	0.0762167	0.0560927	0.313299	0.220991	LINC00702	long intergenic non-	
Up	0	0	0.287142	0.433799	ZFY	zinc finge	GO:0000981
Down	1.00425	0.564755	0.360904	0.337899	OSGEPL1	O-sialogly	GO:000040
Up	0	0	0.141212	0.344574	GCKR	glucokinase (hexokin	
Up	0	0	0.518998	0.57995	C11orf87	chromosome	GO:0016021
Down	21.1806	17.4271	13.2441	10.7641	MRPS18B	mitochondr	GO:000373
Up	1.39779	3.88457	3.91489	4.44571	SLC7A5	solute car	GO:000582
Down	0.479361	0.340771	0.247926	0.119327	HDHD1	haloacid d	GO:000028
Up	288.041	372.706	763.072	775.782	TAGLN	transgelin	
Down	2.93927	5.57027	1.0383	1.2018	RHNO1	RAD9-HUS1-	GO:000007
Up	0.377878	0.21414	1.41122	1.21717	SV2A	synaptic v	GO:000578
Down	0.852023	0.85677	0.384869	0.289739	ERMARD	ER membran	GO:000578
Down	0.0253122	0.078241	0	0	LINC01094	long intergenic non-	

Down	0.191477	0.506966	0.0435417	0.0225584	C1QTNF1	C1q and tı	GO:0005518
Up	2.76129	4.40639	6.82812	8.08483	APLP2	amyloid b€	GO:0002576
Up	0.45439	1.28749	3.18455	4.15304	MESDC1	mesoderm c	GO:0001725
Up	0.0051919	0	0.231486	0.25585	4-Mar	membrane-a	GO:0000139
Up	0.0166351	0.0292561	0.187349	0.161048	RP11-9G1.3		GO:0005829
Down	0.121701	0.21319	0.0040324	0.0012617	GPC3	glypican 3	
Down	0.120673	0.0961184	0.0418863	0.0651792	BCKDHB	branched c	GO:0003826
Up	0.176808	0.109863	0.832187	0.681389	VEGFC	vascular e	GO:0001525
Up	0.519712	2.02827	7.17817	9.34439	NES	nestin	GO:0000086
Down	0.0255812	0.0513423	0	0.0013618	EPB41L3	erythrocyte membrane	
Up	0.235995	0.252835	0.495501	0.419451	AAK1	AP2 associ	GO:0004674
Up	0.812414	0.852047	1.93163	1.74238	DSG2	desmoglein 2	
Up	0.0352074	0.0920849	0.268093	0.293244	LRP3	low densit	GO:0005905
Down	0.133157	0.173176	0.0387674	0.0204886	BNC2	basonuclir	GO:0000981
Down	0.0276428	0.0128401	0.0007619	0	CNKSR2	connector	GO:0005737
Up	4.59029	9.4592	16.1695	13.7406	F8A2	coagulatic	GO:0005634
Up	0	0	74.0309	48.0105	FOXS1	forkhead b	GO:0000978
Down	0.0392099	0.155539	0	0	GATA3	GATA binding protein	
Up	0	0	1.06454	1.22561	LINC00460	long intergenic non-	
Up	0	0	0.2933	0.243608	PRKY	protein ki	GO:0004674
Down	0.0726383	0.0746524	0.01537	0.0224144	PDE7B	phosphodie	GO:0004115
Up	0.559365	1.78305	25.9225	22.0425	HES4	hes family	GO:0000977
Up	0	0	0.0383552	0.0697704	SPTBN5	spectrin,	GO:0000165
Up	0.497666	0.62071	1.18687	1.11455	NAV1	neuron nav	GO:0001578
Up	0.0684478	0.0664794	0.1472	0.172701	BACH1	BTB and CN	GO:0000083
Up	0	0	2.76223	1.19053	TNFSF4	tumor necr	GO:0002215
Down	5.68173	3.76236	0.242353	0.314737	CTSK	cathepsin	GO:0000422
Up	0.244194	0.235106	0.465804	0.628038	SEL1L3	sel-1 supp	GO:0005634
Down	7.82029	6.28752	3.35791	4.22857	DPAGT1	dolichyl-p	GO:0005548
Down	0.113902	0.150534	0.0578595	0.0433867	PID1	phosphotyr	GO:0001933
Down	15.3341	11.8784	7.55003	10.1473	MAPK1IP1L	mitogen-activated pr	
Up	0.315856	0	3.81453	5.84359	PRR15	proline ri	GO:0007275
Up	0	0	0.0507946	0.108979	RP11-483P21.2		
Up	0.166487	0.385964	0.553491	0.815774	ZNF502	zinc finge	GO:0000981
Up	124.7	128.517	269.529	288.803	TNFRSF12A	tumor necr	GO:0001525
Up	0.0432957	0.120899	0.327583	0.263347	ACSF3	acyl-CoA s	GO:0005524
Up	0.0862435	0.427642	0.498916	0.622892	FLYWCH1	FLYWCH-typ	GO:0003677
Up	2.74071	2.64076	4.86305	5.17649	HACD1	3-hydroxya	GO:0005783
Up	0.200423	0.771614	2.18133	2.10797	COL8A1	collagen,	GO:0001525
Up	0	0	5.2102	2.90483	SFTA1P	surfactant associate	
Up	0	0	0.57962	0.476873	TXLNGY	taxilin ga	GO:0019905
Up	0.119395	0.241643	0.519792	0.522995	KDM6B	lysine (K)	GO:0000978
Up	0	0	1.87876	1.02608	DBH-AS1	DBH antisense RNA 1	
Up	1.43316	0.887605	4.38481	4.79516	KRT17	keratin 17, type I	
Up	0	0	0.0842501	0.0219492	C1orf110	chromosome 1 open re	
Down	7.06681	6.11231	4.07707	4.11749	ACAA2	acetyl-CoA	GO:0003723
Up	1.28144	2.10487	4.79704	4.20861	BAG6	BCL2-assoc	GO:0001822
Down	2.07712	1.60271	1.19404	1.15944	UTP6	UTP6, smal	GO:0000462
Up	0.0782855	0.132113	0.187641	0.250689	CDK13	cyclin-dependent kir	
Up	0.900532	1.42553	3.77093	3.29042	TNFRSF10D	tumor necr	GO:0004888
Up	0.14918	0.212644	0.440871	0.449006	NPIPB4	nuclear pc	GO:0000184

Up	0.707131	1.1163	2.63692	2.4054	DUS1L	dihydrouri	GO:0017150
Down	0.665647	0.366391	0.111179	0.0098561	BNC1	basonuclir	GO:0000981
Up	0.213177	0	1.21866	0.708127	LAT	linker for	GO:0000165
Down	31.6426	20.2012	15.3305	9.26752	MRPL36	mitochondr	GO:0003735
Up	0.93146	1.39037	4.85993	3.95294	PLXNA3	plexin A3	GO:0002116
Up	0	0	0.24926	0.365592	CTB-47B11.3		GO:0003677
Down	3.19474	3.05759	1.44782	1.49809	MCM6	minichromosome maint	
Up	0.295573	0.2057	37.6845	45.1711	CXCL12	chemokine (C-X-C mot	
Up	3.09037	2.55454	7.87542	7.60707	CCDC50	coiled-coi	GO:0005829
Up	0.0954479	0.184135	0.270176	0.258809	CDC42BPB	CDC42 binding protei	
Down	0.462942	0.291495	0	0.101862	CH507-338C24.1		GO:0000978
Down	18.0921	12.3761	7.33929	9.26287	ZMYM6NB	ZMYM6 neig	GO:0016021
Down	0.156546	0.211907	0.0111892	0.0006073	PRICKLE1	prickle ho	GO:0001843
Up	0.224803	0.386078	0.869365	0.878999	TNS1	tensin 1	GO:0003723
Down	0.816926	0.664535	0.392019	0.445689	SLC36A4	solute car	GO:0003333
Up	0.71853	0.23952	6.11165	7.87051	MT1F	metallothionein 1F	
Down	1.57939	1.1961	0.700951	0.884477	7-Mar	membrane-a	GO:0002643
Up	1.46986	2.77653	4.10618	4.34777	TRIM8	tripartite	GO:0005829
Up	0.255573	0.206513	0.43959	0.585352	EP300	E1A binding protein	
Up	0.591032	0.904812	1.53799	1.31651	RALBP1	ralA bindi	GO:0005096
Down	10.935	10.1176	3.35842	3.28271	IL6ST	interleuki	GO:0002675
Up	0.0047625	0.0024021	0.0222304	0.0220767	NRXN3	neurexin 3	GO:0005886
Up	0.0981124	0.169216	0.675991	0.883576	SBSPON	somatomedi	GO:0005044
Down	17.6351	12.7979	5.94943	5.51283	ACAT2	acetyl-CoA	GO:0003857
Down	8.49631	7.88658	3.54522	4.24047	GOT2	glutamic-o	GO:0004069
Up	0.113862	0	0.256598	0.258329	SYTL4	synaptotag	GO:0002576
Down	0.915068	1.08686	0.536996	0.401813	TTC38	tetratrico	GO:0070062
Up	0.934319	0.899995	1.99217	2.01836	FAM168B	family wit	GO:0005886
Down	3.71708	2.85619	0.23834	0.0603683	AK4P1	adenylate	GO:0001889
Up	0	0	0.006439	0.0137886	PCDHGA1	protocadhe	GO:0005509
Down	0.350828	0.243995	0.0603278	0.0137522	CKLF-CMTM1	CKLF-CMTM1	GO:0005125
Up	0	0	0.0166961	0.0282078	SYT16	synaptotag	GO:0005543
Down	8.90424	5.74585	3.84258	3.5503	HMGN4	high mobility group	
Down	14.5092	13.1733	7.68766	7.0523	NCSTN	nicastrin	GO:0002262
Up	0.115733	0.34007	1.03782	0.973423	RP11-734K23.9		GO:0003964
Up	0.0459048	0.0908917	0.485416	0.407005	SP140L	SP140 nucl	GO:0000981
Up	0.0155028	0.0131779	0.0654906	0.0792879	DAB2IP	DAB2 interacting pro	
Up	0.951073	1.70161	2.42141	2.2672	TTYH3	tweety fam	GO:0005229
Up	0.0272416	0	0.871694	0.39481	PLN	phospholam	GO:0002026
Down	2.37817	2.29862	0.685271	0.76882	RAB5A	RAB5A, mem	GO:0001726
Down	0.038069	0.0134833	0.0027696	0	KCNK2	potassium	GO:0003231
Up	0	0	0.0205262	0.0126856	RBM20	RNA bindir	GO:0003723
Down	1.78268	1.3252	0.563427	0.563979	NDFIP2	Nedd4 fami	GO:0000139
Up	0.529178	0.858955	1.82085	1.46513	PCGF2	polycomb g	GO:0000122
Up	0.253964	0.255497	0.540836	0.413148	NBPF14	neuroblast	GO:0005737
Down	1.85197	0.544419	0.0885695	0.0402256	SERPINB7	serpin pep	GO:0004867
Up	0	0	8.31314	10.705	GBX2	gastrulati	GO:0000979
Down	0.0196537	0.026314	0.00463	0.0038285	CHN2	chimerin 2	GO:0005070
Down	65.7022	82.0361	17.5505	30.6386	HCFC1R1	host cell	GO:0005654
Down	0.104237	0.0709499	0.0091791	0.0083699	LIN7A	lin-7 homo	GO:0005622
Up	0.834325	1.35499	5.40809	3.73892	A1BG	alpha-1-B glycoprote	

Down	1.14967	1.25949	0.608966	0.654555	SLC35F5	solute car	GO:0016021
Down	1.71525	1.19761	0.44778	0.585137	FAH	fumarylace	GO:0004334
Down	0.0049992	0.0161884	0	0	PLEKHA7	pleckstrir	GO:0005634
Up	2.04974	1.7631	5.53113	6.24296	MBNL1-AS1	MBNL1 antisense RNA	
Up	0.135047	0.118057	0.252107	0.260033	CDK6	cyclin-dependent kir	
Down	0.151561	0.155579	0	0.0028829	AL035610.2		GO:0005829
Down	0.720728	0.529635	0.0713941	0.112805	LURAP1L	leucine ri	GO:0043123
Down	0.129425	0.0796309	0.0046631	0.002126	TNFSF13B	tumor necrosis facto	
Down	0.100275	0.11072	0.0514496	0.0404712	SLC38A9	solute car	GO:0003333
Down	3.43021	5.25726	1.70184	2.03651	CAPG	capping protein (act	
Up	0.240312	0.285449	0.513449	0.59528	ZNF814	zinc finge	GO:0000981
Down	0.262211	0.152402	0.0729137	0.0528273	RP11-552F3	Uncharacte	GO:0000922
Down	0.590593	0.639284	0	0	LINC00900	long intergenic non-	
Down	0.471555	0.476201	0.297808	0.215951	PDXDC1	pyridoxal-	GO:0001667
Down	22.8544	23.9418	4.62744	4.30273	HAND2	heart and	GO:0000790
Up	0	0	0.028044	0.0171851	LINC01197	long inter	GO:0016021
Down	0.0114456	0.0176167	0.0007128	0	AC007563.5		
Down	8.45451	11.0961	4.8098	4.19708	PDLIM4	PDZ and LI	GO:0001725
Up	0	0	0.365609	0.375046	RP11-278I4.2		
Up	26.4544	22.0412	53.0559	44.0303	GNG11	guanine nu	GO:0003924
Up	0.185117	0.386324	0.594777	0.519926	MMP24-AS1	MMP24 antisense RNA	
Down	0.31147	0.235009	0.0884571	0.088352	C2CD5	C2 calcium	GO:0005509
Down	0.0987084	0.457668	0	0	TMEM176A	transmembr	GO:0016021
Up	14.017	27.5583	77.557	72.9119	AC005943.6		
Up	0.558879	0.334849	1.71068	1.41076	DENND4B	DENN/MADD	GO:0005634
Down	0.015162	0.0047734	0	0	MROH7-TTC4	MROH7-TTC4	GO:0005615
Down	0.889692	1.52427	0.0626192	0.11499	FBXO6	F-box protein 6	
Down	3.33823	4.22775	1.15876	1.0378	COL12A1	collagen,	GO:0001501
Down	1.71295	1.8008	0.949125	0.922582	SLC39A14	solute car	GO:0005384
Up	0.254983	0.591648	5.20545	5.01926	COL4A2	collagen,	GO:0001525
Up	0.627977	2.11279	2.4755	2.49874	SMTN	smoothelir	GO:0003779
Up	11.5895	9.38644	38.9935	31.6662	PHLDA1	pleckstrir	GO:0000086
Up	0	0	0.0250924	0.0064581	KIAA2012	KIAA2012	
Up	0.0637844	0.225913	1.11602	0.893861	9-Mar	membrane-a	GO:0000139
Up	0.0394469	0.0394486	0.23994	0.170092	HOXD3	homeobox D	GO:0000977
Down	0.388125	1.48996	0	0	RP11-328C8.4		GO:0001843
Up	0	0	1.58932	3.77086	DLX1	distal-les	GO:0000122
Up	0.25007	0.283425	2.04344	2.46329	SAPCD2	suppressor	GO:0000132
Up	0.0218205	0.049462	0.101352	0.0926947	SAP30L-AS1	SAP30L ant	GO:0000978
Up	14.0965	10.4876	23.9663	23.9424	GLIPR1	GLI pathog	GO:0005615
Up	0.605111	0.53626	3.985	3.91317	PRSS3	protease,	GO:0004252
Down	0.0014566	0.003502	0	0	CNTN4	contactin	GO:0005576
Down	0.390312	0.374788	0.0957045	0.180247	GK	glycerol k	GO:0004370
Up	1.08237	1.90555	2.52278	2.46048	CHD4	chromodoma	GO:0000790
Up	44.5568	39.5373	141.968	145.101	RP11-138C9.1		
Down	4.57775	3.47531	2.72345	2.568	RBBP4	retinoblas	GO:0000790
Up	0.214389	1.79435	2.21272	2.28184	DMPK	dystrophia	GO:0004674
Down	1.86098	2.247	1.08981	1.09994	FBXO22	F-box prot	GO:0000209
Up	0	0	0.0959541	0.129287	PLEKHG5	pleckstrir	GO:0005085
Up	1.00046	1.17795	2.04656	1.8401	RTF1	RTF1 homol	GO:0000122
Down	1.45211	2.28205	0.764509	0.909241	PGM1	phosphoglu	GO:0000287

Down	4.29359	3.74172	2.13388	1.55231	AGA	aspartylgl	GO:000394€
Down	6.03763	4.1088	2.85669	2.94548	TMEM128	transmembı	GO:0016021
Up	0.161674	0.172133	0.8155	0.703228	THUMPD2	THUMP doma	GO:000372ɜ
Up	0	0	0.028833	0.0395223	ALDH1L1	aldehyde (	GO:000382Ɛ
Up	0	0	0.0930048	0.134462	ABCA3	ATP-binding cassette	
Down	1.00528	0.756986	0.302274	0.263077	WASF1	WAS protei	GO:000377ç
Down	0.316327	0.22665	0.103622	0.116017	LIN52	lin-52 DRI	GO:0006351
Up	0.137395	0.222243	0.563177	0.572371	SPAG9	sperm ass(	GO:000166ç
Up	0.306124	0.31468	0.799892	0.796084	RUFY1	RUN and FY	GO:000563Ɛ
Up	0.112525	0.1913	0.37548	0.368253	PDPR	pyruvate (	GO:0004741
Down	0.12765	0.143888	0.051756	0.0383275	MCM9	minichrom(	GO:000072Ɛ
Down	0.46715	0.464471	0.233934	0.280534	PREP	prolyl enc	GO:000417ɛ
Up	0.0484496	0.04576	0.521865	0.514541	IL17D	interleukin 17D	
Down	6.21209	4.90369	3.84789	2.34782	RP11-620J15.3		
Up	0.0959554	0.175752	0.430464	0.421232	GRB10	growth factor recept	
Up	0.07718	0.187445	0.398574	0.292296	NACC1	nucleus accumbens as	
Down	2.72546	2.34561	1.08005	1.50408	CCNC	cyclin C	GO:0000307
Down	13.6312	9.46617	7.35425	6.44708	CDCA3	cell divis	GO:000582ç
Down	0.490717	0.381175	0.138798	0.273922	CRLF3	cytokine ı	GO:000008ʒ
Down	11.4208	8.22954	5.93409	3.95034	C14orf1	chromosome	GO:000578ç
Up	0	0	1.37759	0.717521	OSTN-AS1	OSTN antisense RNA 1	
Down	5.84903	5.72126	1.56373	1.60882	GSTT2	glutathior	GO:000436Ɛ
Down	0.755858	1.09184	0.0560681	0.178937	IFI44	interferor	GO:0005737
Up	0.0570886	0	0.830975	0.750209	NPAS1	neuronal I	GO:000012ʒ
Down	6.61026	0	0	0	SENP3-EIF4	SENP3-EIF4	GO:000563Ɛ
Up	0.416265	0.550419	0.867871	1.19129	ZNF587	zinc finge	GO:0000981
Up	0.0075373	0	0.56268	0.507004	LINC01085	long intergenic non-	
Up	3.61411	2.56763	6.96495	7.37951	CNRIP1	cannabinoi	GO:0005737
Down	156.72	122.464	76.3284	78.2939	PGAM1	phosphogly	GO:000408ʒ
Up	0.142416	0.030287	0.433318	0.423852	ARHGEF10	Rho guanir	GO:000508ɛ
Down	0.708926	0.957457	0.008132	0.0041195	PAMR1	peptidase	GO:000550ç
Up	0.123696	0.193602	0.441211	0.644556	SNTA1	syntrophir	GO:0002027
Down	0.876008	0.409893	0.115534	0.137846	STK26	serine/thı	GO:000013ç
Up	0.115388	0.107225	0.230387	0.242234	RICTOR	RPTOR inde	GO:000193€
Up	0.0757437	0.134813	0.226202	0.269089	RASA3	RAS p21 pı	GO:000016ɛ
Up	3.49586	3.26657	6.45651	5.84827	NUCKS1	nuclear ca	GO:000072Ɛ
Down	9.1956	6.44651	2.24299	2.39888	IDH1	isocitrate dehydroge	
Down	1.95923	1.60279	1.16919	0.748425	PRPS1	phosphorit	GO:0000287
Up	1.03967	0.631257	2.56592	2.33756	CHML	choroidere	GO:000509ʒ
Down	0.453273	0.318635	0.095067	0.113473	DRAM1	DNA-damage regulate(	
Up	0	0	2.89295	5.61773	GP1BB	glycoprote	GO:000488€
Up	0	0	0.0284842	0.0233089	CTB-109A12.1		
Down	2.8409	3.02919	1.27084	1.48879	WDR45	WD repeat	GO:000004ɛ
Up	0	0.0284653	0.907379	0.439199	LMO2	LIM domain only 2 (ı	
Down	0.0516296	0.0296284	0.0090655	0.0040705	RPS6KA6	ribosomal	GO:0000287
Down	2.23395	1.70156	0.880606	1.35389	VPS26A	VPS26 retı	GO:000576Ɛ
Up	0.203045	0.253285	0.777529	0.632826	ASAP2	ArfGAP wit	GO:000509€
Down	0.138077	0.116128	0.0144941	0.0239109	ST6GALNAC3	ST6 (alpha-N-acetyl-	
Down	4.41205	3.19184	0.236754	0	RP11-475I24.9		
Down	4.10042	3.65838	1.80708	1.9286	IARS	isoleucyl-	GO:000004ç
Up	2.07563	2.33556	4.29334	4.53866	MGARP	mitochondı	GO:000573ç

Up	0.0853393	0.144182	0.237288	0.215987	PEAK1	pseudopodi	GO:0004672
Down	33.7646	30.6146	14.6384	12.1713	ABT1	activator	GO:0000447
Up	0.0201931	0.021126	0.0940213	0.104486	MARK1	MAP/microt	GO:0000226
Up	0.0168393	0	0.139543	0.0979655	AHDC1	AT hook, I	GO:0003677
Up	0.159953	0.111317	0.504262	0.36838	MCM3AP-AS1	MCM3AP antisense RNA	
Down	1.63823	1.51079	0.641924	0.594213	IL10RB	interleukin 10 recep	
Down	0.130662	0.222791	0.0189557	0.0055399	HRASLS	HRAS-like	GO:0004623
Down	0.183967	0.276606	0.134703	0.0894187	BCAR3	breast car	GO:0002089
Down	72.9044	36.2091	17.9164	18.8701	TMED2	transmembr	GO:0001701
Up	0.0003482	0.0078937	0.203758	0.255265	MEGF6	multiple E	GO:0005509
Up	0	0.105715	0.299933	0.420065	LINC01123	long inter	GO:0000981
Down	0.0985272	0.188597	0.0331322	0.038603	TWIST1	twist family bHLH tr	
Up	1.61054	1.21584	4.1206	3.85739	RGMB	repulsive	GO:0005793
Up	0.33492	0.460112	1.4782	1.4247	LINC01116	long inter	GO:0005829
Up	0	0	0.0539396	0.173147	KY	kyphoscoli	GO:0005856
Down	0.0320108	0.019377	0	0	GALNT13	polypeptide N-acetyl	
Up	0.0382426	0.0789905	0.102309	0.104282	MICAL3	microtubul	GO:0003779
Down	1.58358	1.2204	0.808178	0.675613	ELOVL5	ELOVL fatt	GO:0005783
Down	0.0979939	0.0944622	0.0337597	0.0159724	TRPC1	transient	GO:0005261
Up	28.9432	43.1491	80.5737	70.1031	CH507-513H4.6		GO:0005524
Up	0.146449	0.153786	0.339852	0.460439	SGMS2	sphingomye	GO:0002950
Up	2.78183	2.04205	7.80228	7.33061	P2RX5-TAX1	P2RX5-TAX1BP3 readth	
Up	0	0	0.0275625	0.0264212	CABLES1	Cdk5 and A	GO:0005634
Up	1.86722	1.95118	9.56877	9.00937	SAMD1	sterile al	GO:0005576
Up	0.016453	0	0.174281	0.0835566	CELSR2	cadherin,	GO:0004930
Up	0.191957	0.155616	0.742664	0.791174	UBASH3B	ubiquitin	GO:0004725
Up	0.135574	0.15418	0.455319	0.410492	E2F3	E2F transcription fa	
Up	0.109128	0.307538	0.496152	0.467443	RUSC2	RUN and SH	GO:0005829
Down	0.0977675	0.131698	0.0303166	0.0165094	CPEB1	cytoplasmi	GO:0000900
Up	0.829091	1.35405	2.58291	2.50608	PXDC1	PX domain	GO:0035091
Down	3.58317	3.37461	1.8442	1.81251	ASAH1	N-acylsphi	GO:0003824
Up	0.0035622	0.0151401	0.201019	0.170667	SFRP1	secreted f	GO:0001649
Up	0.006079	0.013397	0.0227846	0.0441727	KCNAB1	potassium	GO:0004033
Up	0.314233	0.844707	1.22433	1.13878	PKDCC	protein ki	GO:0001501
Down	0.877104	1.09693	0.205955	0.185478	TNFAIP2	tumor necrosis facto	
Up	0.53709	0.481392	1.22326	1.21571	PSMB9	proteasome	GO:0000502
Down	6.47771	6.46129	3.28639	2.67729	RP5-1172A22.1		GO:0003677
Down	0.932845	0.765316	0.323258	0.267199	LACTB2	lactamase,	GO:0003727
Down	0.0527368	0.0449405	0.0140005	0.0110184	RP11-154D6.1		GO:0003677
Up	3.75182	4.56456	7.66251	7.48116	CCDC74A	coiled-coil domain c	
Down	58.6585	50.0302	18.9199	17.8062	SLC38A2	solute car	GO:0003333
Down	0.0090346	0.0105267	0	0	SPOCK3	sparc/oste	GO:0005509
Up	0.235748	0.168727	0.411918	0.394593	PHACTR2	phosphatas	GO:0002576
Down	0.277752	0.677382	0.113832	0.0109309	TRABD2A	TraB domai	GO:0004175
Up	0.755389	1.49328	3.96534	3.60226	PSMB8-AS1	PSMB8 anti	GO:0002250
Down	1.71244	1.3917	0.833273	0.519399	NMT1	N-myristoyltransfera	
Down	5.28665	3.96152	1.21211	0.879172	G0S2	G0/G1 swit	GO:0005739
Up	0.571132	0.647311	1.64749	1.50326	MUM1	melanoma a	GO:0005634
Down	74.2431	68.295	37.2385	42.0912	HNRNPA1	heterogene	GO:0003723
Down	3.19004	3.32292	1.08707	1.42563	SERPINB1	serpin pep	GO:0004867
Down	1.97055	2.70713	0.545929	0.371917	RP11-462G12.1		

Up	0.0850608	0.0635647	1.14808	0.791771	RP11-54A9.1		
Down	4.91606	3.92622	0.796837	0.865705	LRRC17	leucine ri	GO:000150[illegible]
Up	0.0016352	0	0.0092247	0.0248219	COL25A1	collagen,	GO:000154[illegible]
Up	0.798051	1.28357	2.33306	2.25961	FASN	fatty aci	GO:000164[illegible]
Up	0.409272	0.923428	2.203	2.04798	ESRRA	estrogen-related re	
Up	2.23104	2.67628	4.93887	5.98038	DSEL	dermatan s	GO:000013[illegible]
Up	2.70318	7.43894	8.93888	8.00356	MOGS	mannosyl-c	GO:000457[illegible]
Up	0.166474	0.066203	0.990714	1.12501	PITPNM1	phosphati	GO:000550[illegible]
Down	11.8976	10.4064	6.66976	6.44616	KLF10	Kruppel-li	GO:000012[illegible]
Up	0.529579	0.942005	1.15384	1.20096	EXOC3	exocyst c	GO:000014[illegible]
Down	0.0325578	0.0263575	0.0134062	0.0008489	F8	coagulation factor	
Down	0.11135	0.1054	0.0475255	0.0363473	SHC3	SHC (Src	GO:000016[illegible]
Down	3.09817	1.89688	0.84743	1.01731	CAT	catalase	
Down	22.0096	21.6153	12.9714	11.3229	ALG3	ALG3, alp	GO:000003[illegible]
Down	2.88147	2.5419	1.55281	1.32046	GOLPH3	golgi pho	GO:000573[illegible]
Down	0.0779354	0.0762694	0.0197347	0.0149956	NHSL1	NHS-like	GO:003015[illegible]
Up	0.104835	0.0672709	0.293257	0.340693	MMP16	matrix metallopepti	
Down	0.876783	0.64517	0.346797	0.150177	CTH	cystathio	GO:000009[illegible]
Up	2.98458	5.88875	6.06057	7.3724	PFKP	phosphofr	GO:000387[illegible]
Down	1.14179	0.674894	0.121575	0.24997	RBP1	retinol bi	GO:000152[illegible]
Up	0.0649595	0.0654828	0.172364	0.168779	APBB2	amyloid be	GO:000154[illegible]
Down	0.517285	0.46118	0.262458	0.292649	GATB	glutamyl-t	GO:000552[illegible]
Up	0.0286143	0.0720687	0.0775125	0.134045	C16orf52	chromosome	GO:000579[illegible]
Down	0.305202	0.525506	0.145209	0.147932	MALT1	MALT1 paracaspase	
Up	0.26549	0.272172	1.46197	0.917864	BCL9L	B-cell CLL	GO:000371[illegible]
Down	0.548404	0.486747	0.284525	0.234625	CHCHD3	coiled-coi	GO:000374[illegible]
Down	0.0748718	0.0417336	0	0.0047044	PDPN	podoplani	GO:000090[illegible]
Up	1.10315	1.01374	6.69231	5.51376	CENPB	centromere	GO:000077[illegible]
Down	0.05943	0.0247636	0	0	RP3-509I1[illegible]	Protein LOC10050746[illegible]	
Up	0.0766335	0.0586859	0.349983	0.287769	TCN2	transcobalamin II	
Up	0	0.0229314	0.0452874	0.03005	ZFPM1	zinc finge	GO:000012[illegible]
Down	0.017569	0.0023819	0	0	PPP2R2B	protein p	GO:000015[illegible]
Down	1.21866	0.90186	0.465977	0.427192	QRSL1	glutaminyl	GO:000404[illegible]
Down	0.0445596	0.0330675	0.0026758	0.0010166	ACTR3C	ARP3 acti	GO:000377[illegible]
Up	3.98223	4.72115	9.09212	8.04595	KPNB1	karyopher	GO:000006[illegible]
Down	0.764323	0.843867	0.402151	0.315681	PCMTD2	protein-L-	GO:000471[illegible]
Down	4.86563	5.48099	2.01281	1.89334	FAM219B	family with sequenc	
Up	10.5148	8.28358	18.807	24.5652	POSTN	periostin,	GO:0001501
Up	0	0	0.0253876	0.0072341	NGEF	neuronal	GO:000508[illegible]
Down	3.62177	1.03446	0.026689	0.0506999	IL13RA2	interleukin 13 rece	
Down	4.04946	2.86632	2.09517	2.05026	ZNF330	zinc finge	GO:000077[illegible]
Up	6.06505	5.77311	14.669	14.5289	CHST14	carbohydra	GO:000013[illegible]
Down	1.52647	1.40676	0.763498	0.449124	GLB1	galactosidase, beta	
Up	0	0	0.0061857	0.0026567	NTRK3	neurotrophic tyrosi	
Up	0.0281631	0.0660405	0.126138	0.187972	LRRC37A4P	leucine ri	GO:0016021
Down	0.958206	1.71851	0.0543068	0.0309492	NOS3	nitric oxide syntha	
Up	0.0012905	0	0.0846254	0.0624487	RUNX3	runt-rela	GO:000012[illegible]
Down	30.7729	44.1479	5.6882	11.4908	SERPINE2	serpin pe	GO:0004867
Down	48.2807	40.1096	19.6519	20.6306	CALU	calumenin	GO:000550[illegible]
Up	0.0168547	0.0129342	0.0617702	0.047105	PIEZO2	piezo-type	GO:0005261
Up	0.24236	0.266058	0.731256	0.750192	SLC35E4	solute ca	GO:0016021

Down	0.173245	0.433505	0.0680934	0.0150913	LINC00475	long intergenic non-
Down	2.12552	1.58365	0.209606	0.170222	DUSP10	dual specificity pho
Down	0.119661	0.213455	0	0	RSPO2	R-spondin GO:0001649
Down	0.503345	0.667231	0.122492	0.0564104	RP11-1060J15.4	GO:0001881
Down	1.00895	0.659724	0.110104	0	GPBAR1	G protein-GO:0005737
Up	0.381325	0.545803	1.24677	1.11312	WRNIP1	Werner helGO:0000731
Down	0.0635279	0.0209306	0.0055891	0	RNF212	ring fingeGO:0000795
Up	0.0447394	0.363399	0.789848	0.724497	SEZ6L2	seizure reGO:0005789
Down	0.726642	0.495376	0.187167	0.266556	STRADB	STE20-relaGO:0000165
Down	2.18708	1.91198	1.24772	1.20031	IFNAR1	interferon (alpha, b
Up	0.250206	0	0.861098	0.786676	MICB	MHC class GO:0001913
Down	0.684567	0.306007	0.166941	0.0968079	RRN3P1	RRN3 homolGO:0001042
Up	0	0.116863	0.910172	2.41073	SAPCD1	suppressor APC domai
Down	24.3417	25.917	10.4784	10.6519	CNN2	calponin 2GO:0001725
Up	0.0036867	0.0156691	0.0805828	0.0662363	CARD11	caspase reGO:0001772
Up	0	0	0.173726	0.175322	CTD-2561J22.5	
Up	0.15692	0.266775	1.24335	1.50612	SH3BP5L	SH3-bindinGO:0004860
Down	0.0399559	0.0060786	0.0010145	0	CDH10	cadherin 1GO:0005509
Down	0.589724	0.945095	0.0241874	0.0174445	SLC12A8	solute carGO:0005623
Up	1.18958	2.15037	4.34837	3.24067	NR2F6	nuclear reGO:0000122
Up	2.58526	3.02071	6.16727	6.99748	RP11-96D1.11	GO:0000978
Up	0.0286376	0.0186805	0.0974268	0.0937724	PVRL3	poliovirusGO:0002089
Up	12.3426	18.3371	25.4113	22.7314	GDI1	GDP dissocGO:0005093
Up	0.0597907	0.0892473	0.128165	0.128358	ATXN1	ataxin 1
Up	1.18786	0.256928	3.09877	3.00629	BAP1	BRCA1 associated pro
Down	0.0388384	0.0454162	0.007774	0	RP4-665J23.1	
Up	0.0017798	0	0.0168329	0.0074612	ZPLD1	zona pelluGO:0005576
Down	1.67487	1.35204	0.594204	0.680698	SGCE	sarcoglycaGO:0005794
Up	0.0891389	0.137766	0.357658	0.242005	ZNF264	zinc fingeGO:0000981
Down	1.39268	2.62505	0.614484	0.489981	CYB561	cytochromeGO:0000293
Up	19.7969	23.1707	37.0714	42.3033	MAGEF1	melanoma aGO:0016567
Up	0.402065	0.295905	1.04037	1.22333	ZSWIM8	zinc fingeGO:0008270
Down	61.5769	43.5012	18.732	19.7666	ID2	inhibitor GO:0000122
Down	0.0046731	0.0046056	0	0.0001947	SNTG2	syntrophinGO:0003779
Up	2.34433	3.39509	6.81037	4.57591	KLHDC8B	kelch domaGO:0005829
Up	0.139181	0.0973107	0.530524	0.613131	TBC1D2B	TBC1 domaiGO:0005096
Up	0.0719545	0	0.321068	0.636437	CTD-2228K2.7	GO:0005886
Up	1.20357	5.45644	6.24095	6.87351	TMEM262	transmembrGO:0003677
Up	27.4032	27.7269	62.2301	58.4037	CST3	cystatin CGO:0001540
Up	0.780873	2.21257	46.0028	44.2274	FAM43A	family with sequence
Up	7.38332	8.13568	20.3207	24.7336	C8orf48	chromosome 8 open re
Up	0.432266	0.155531	1.17672	1.59763	SAMD9	sterile alGO:0005737
Down	4.22453	2.88339	1.31078	1.21683	MRPS6	mitochondrGO:0003735
Up	0.0407045	0.131811	0.237737	0.180354	BCR	breakpoint cluster r
Up	0.613192	1.51083	8.47216	13.4118	RP11-311C24.1	
Down	0.482873	0.476334	0.172868	0.207969	ITFG1	integrin aGO:0016021
Up	6.04813	3.46593	22.5378	18.2326	RP1-20B21.4	
Up	0	0	0.0150033	0.0120914	CAMK2B	calcium/caGO:0000165
Up	0.117243	0.242474	0.506338	0.47161	MPRIP	myosin phoGO:0003779
Down	0.94544	1.00315	0.536079	0.418911	ZFAND1	zinc fingeGO:0008270
Down	0.0619624	0.133938	0	0.0036725	FAM20A	family witGO:0001934

Up	0.0979127	0.307275	0.317285	0.392851	ARAP1	ArfGAP wit	GO:0001921
Up	6.51956	15.052	51.3581	55.6839	CTD-2033D15.3		
Up	0.0978521	0.302465	0.581716	0.334866	KCTD2	potassium	GO:0005737
Down	2.31986	1.67273	1.21318	1.17954	FUNDC1	FUN14 doma	GO:0000422
Up	0.0092738	0.0077857	0.0480775	0.052659	FGD4	FYVE, RhoG	GO:0001726
Up	1.70767	3.39331	5.70517	7.83441	LDOC1L	leucine zipper, down	
Up	0	0	0.351237	0.301329	HSPB2-C11c	HSPB2-C11orf52 readt	
Up	0.196625	0.0937227	8.02941	7.57153	PDGFA	platelet-c	GO:0000139
Down	1.48675	1.39365	0.592052	0.724803	TIFA	TRAF-inter	GO:0002753
Down	4.59235	3.97805	1.69604	3.10601	ABHD10	abhydrolas	GO:0004553
Up	0.198016	0.348391	0.576934	0.495929	CAPN5	calpain 5	GO:0004198
Down	0.102393	0.167629	0.0219983	0.0254369	CCDC122	coiled-coi	GO:0003964
Up	0.243985	0.451475	0.837622	0.550595	ERBB2	erb-b2 receptor tyro	
Down	3.75257	2.85898	1.53217	2.09361	UBE2V2	ubiquitin-	GO:0000209
Down	0.0245736	0.0295392	0.0020346	0.0014271	ZNF804A	zinc finge	GO:0003676
Down	0.258163	0.283588	0.135906	0.120627	PPP3CA	protein phosphatase	
Down	1.57987	1.49822	0.96619	0.809186	FAM118A	family wit	GO:0016021
Up	0	0	1.12284	2.64679	RP11-542C1	Uncharacte	GO:0005814
Up	0	0	0.0441561	0.0588752	MAATS1	MYCBP-asso	GO:0003341
Up	0.261634	0.333597	1.2496	1.23908	NCS1	neuronal c	GO:0000287
Down	0.131244	0.085	0.0252195	0.0263495	IRS2	insulin re	GO:0000165
Down	0.245361	0.113971	0.020289	0.0616674	ZNF214	zinc finge	GO:0000981
Down	0.614281	0.549253	0.203153	0.283009	MTPAP	mitochondr	GO:0000287
Down	0.180373	0.318618	0.0422124	0.0274044	SULF2	sulfatase	GO:0001822
Up	1.54951	4.58026	6.47572	8.425	IRAK1	interleuki	GO:0000187
Up	0.0045001	0	0.0666789	0.0643216	LANCL3	LanC lanti	GO:0003824
Up	0.0106944	0	1.09236	1.2133	LINC00839	long intergenic non-	
Up	33.4583	51.3556	67.5824	64.142	ARHGDIA	Rho GDP di	GO:0005094
Up	0.484134	0.559551	1.01464	1.30985	AKAP2	A kinase (PRKA) anch	
Down	2.21251	2.07771	0.813381	0.573817	CD44	CD44 molecule (India	
Up	0.0509663	0.127421	0.218734	0.287682	FAM168A	family wit	GO:1905053
Up	0.538589	1.38073	1.68581	1.51752	UNC119B	unc-119 li	GO:0005829
Down	0.37763	0.434865	0.0687564	0.0251552	AQP11	aquaporin	GO:0005783
Up	0.243013	0.211867	0.545122	0.613489	ZBED4	zinc finge	GO:0000981
Up	0	0	1.16392	1.6716	RP11-400K9.4		
Up	0.365636	0.59816	0.708121	0.847163	LMOD1	leiomodin	GO:0003779
Down	4.51474	3.72756	0.301627	1.51842	RP11-366M4.11		GO:0005622
Up	0.0119523	0.040138	0.152955	0.14422	FBRSL1	fibrosin-l	GO:0003723
Up	0	0	0.249245	0.160255	B3GAT1	beta-1,3-g	GO:0000139
Up	0.180251	0.255366	1.09644	0.907319	PCOLCE2	procollage	GO:0005518
Down	0.463981	0.402134	0.0275338	0.0209219	LRRC61	leucine ri	GO:0005737
Down	5.2628	5.64845	2.96633	3.08998	BLVRB	biliverdin reductase	
Up	0.233949	0.330434	0.77027	0.750872	BCL2L1	BCL2-like 1	
Up	0.38387	0.252215	1.38907	2.2656	B3GALT2	UDP-Gal:betaGlcNAc b	
Down	3.89436	3.80136	2.08341	2.13659	BDH2	3-hydroxyb	GO:0003858
Up	0.271117	0.482868	1.43592	1.57728	SOD3	superoxide dismutase	
Down	1.15958	0.699371	0.500851	0.394384	KIAA0196	KIAA0196	GO:0001556
Up	0.004129	0.0080995	0.0244318	0.0197821	CMIP	c-Maf indu	GO:0005654
Up	0.0258744	0.0616503	0.17686	0.18144	CERKL	ceramide k	GO:0001750
Up	0.724432	0.512161	1.45221	1.56016	RPL39L	ribosomal	GO:0005634
Up	0.279374	1.04897	2.12127	2.37668	ITGA11	integrin, alpha 11	

Down	10.9675	8.98644	6.18254	6.06602	UBE2D3	ubiquitin-GO:000020ç
Up	0.231041	0.280123	0.518143	0.569423	FARP1	FERM, Rho(GO:000508ç
Up	0	0	0.401656	0.488324	AC004076.5	
Up	0.121774	0.230028	0.468361	0.517304	KMT2B	lysine (K)GO:0000981
Up	1.57957	2.52149	5.47066	5.47864	ABALON	apoptotic BCL2L1-ant
Down	0.930026	0.897988	0.330619	0.533921	MAP3K7	mitogen-activated pı
Up	0.0477969	0.0326901	0.119507	0.127922	LMTK2	lemur tyrosine kinas
Up	0.0924098	0.0529562	0.169232	0.198984	SESTD1	SEC14 and GO:000554€
Up	1.30554	3.5352	4.90654	5.86051	NAB2	NGFI-A bir GO:000195ε
Up	0.138032	0.782214	1.28915	1.10357	PPP1R26	protein pł GO:000486⁄
Down	0.155596	0.168683	0.0309009	0.0233364	ABCC4	ATP-binding cassette
Up	0.0380586	0	0.164548	0.145293	FGF5	fibroblast growth fa
Up	4.01795	8.86099	13.2285	19.326	TMEM251	transmembı GO:0016021
Up	0.633786	0	3.61249	2.98791	RP11-70L8.5	GO:000396⁄
Down	3.95865	3.72311	1.97291	1.86192	THAP5	THAP domaiGO:0000981
Down	0.296997	0.234558	0.0238082	0.169081	DDIAS	DNA damageGO:000563⁄
Down	0.163001	0.0351891	0	0.0059501	TNFRSF9	tumor necrosis facto
Down	4.93627	4.41218	2.62417	3.01692	NIPA2	non imprirGO:000576ç
Down	0.0589597	0.0287498	0.0015562	0.0088028	CPED1	cadherin-1GO:000578ʒ
Up	0.0192535	0.0198378	0.0868164	0.102587	ABCC9	ATP-binding cassette
Down	0.0230563	0.0141252	0	0	LINC00506	long interGO:000016€
Down	4.7207	3.39345	2.32282	2.75955	GNAI3	guanine nuGO:000166⁄
Up	0.706935	0.678636	1.46873	1.88226	OCIAD2	OCIA domaiGO:000576ε
Up	0	0	4.14665	5.05453	C8orf4	chromosomeGO:000226⁄
Down	0.145332	0.101034	0.044284	0.0543572	MROH8	maestro heat-like re
Down	1.42614	1.48735	0.279157	0.30311	TPST1	tyrosylpr(GO:000013ç
Up	0.788005	0.453356	1.44683	1.33383	IPO7	importin 7GO:000521£
Up	0	0	0.0566186	0.0454807	TNFRSF11A	tumor necıGO:000150ʒ
Up	0.317976	0.443957	1.26104	1.40642	NELFB	negative eGO:000372ʒ
Up	0.0895164	0	0.34229	0.312664	PKD1P6	polycystic kidney di
Up	0.846738	0	17.5113	18.6611	TRAV39	T cell rec GO:000225(
Up	0.0928971	0.0829219	0.213131	0.213275	GNAQ	guanine nuGO:000150ε
Up	0.41378	0.907896	0.893871	1.08454	STAT6	signal transducer ar
Up	0.0211236	0	3.26974	3.13732	EPHB6	EPH receptor B6
Down	0.0507637	0.0739732	0.003658	0.006254	SLC7A8	solute car GO:000573˜
Up	5.73819	15.2494	55.7039	60.5599	BGN	biglycan GO:000197⁄
Up	0.111923	0.111274	0.443224	0.279403	SOGA1	suppressorGO:000561£
Down	21.5846	17.3967	12.1125	11.4133	CTD-2231E14.2	GO:000367˜
Up	0.0536448	0	11.7312	13.5061	SULT1E1	sulfotransferase fan
Up	0.0877328	0.0901392	0.181778	0.208379	MYO6	myosin VI GO:000172€
Down	0.118504	0.350375	0.008663	0.0263308	GUCA1A	guanylate cyclase ac
Down	4.41576	4.07012	2.34067	2.40732	PARP2	poly (ADP-ribose) pc
Up	0.0509818	0.107633	0.223123	0.220309	MMEL1	membrane nGO:000422ʒ
Up	1.66975	3.48652	4.51373	4.45144	PLEC	plectin
Up	0.0412851	0.0559017	0.0995161	0.138284	NBPF20	neuroblastGO:000573˜
Down	5.35702	5.68902	0.13096	0.281949	FOXF1	forkhead box F1
Up	393.231	416.175	814.034	672.54	TIMP1	TIMP metalGO:000177£
Up	0.146907	0.219261	0.319429	0.350469	ARHGAP21	Rho GTPaseGO:000013ç
Up	1.95888	2.39267	3.80628	3.75783	EIF5B	eukaryoticGO:000372ʒ
Up	0.115928	0.328477	0.783936	0.774804	PPP6R1	protein pł GO:000013ç

GO_term pathway pathway_description
epithelial cell morphogenesis|nucleoplasm|cytoplasm|centrosome|cytosol|plasma m
serine-type endopeptidase activity|peptidase activity|protein processing|peptic
actin binc ko04015,kc Rap1 signaling pathway|Regulation of actin cytoskeleton|MAP
cytokine activity|extracellular space|cytosol|defense response|immune response|
Golgi memk ko00514 Other types of O-glycan biosynthesis
cornified envelope|desmosome assembly|protein kinase C binding|nucleus|nucleopl
RNA bindir ko04115 p53 signaling pathway
orane asso ko04914,kc Progesterone-mediated oocyte maturation|Oocyte meiosis|Cell
transcription corepressor binding|cytoplasm|establishment of cell polarity|lame
guanylate ko04921,kc Oxytocin signaling pathway|Renin secretion|Gap junction|Cir
neuron migration|ubiquitin-protein transferase activity|ubiquitin-dependent prc
proteolysis|peptidase activity|metallopeptidase activity|zinc ion binding
tyl) trans: ko00512 Mucin type O-Glycan biosynthesis
regulation of alternative mRNA splicing, via spliceosome|RNA methylation|RNA bi
metalloendopeptidase activity|integral component of plasma membrane|proteolysis
P(+)-depen ko00710,kc Carbon fixation in photosynthetic organisms|Carbon metaboli
cytosol|keratinization|keratin filament

cytoplasm|apoptotic process|membrane
E3 ubiqui ko04144,kc Endocytosis|Insulin signaling pathway|ErbB signaling pathwa
microtubul ko04540,kc Gap junction|Phagosome
kinetochore|condensed chromosome kinetochore|nucleolus|kinetochore binding|kine
trans-Golgi network|cytosol|plasma membrane|protein localization|endomembrane s
plasma men ko04340 Hedgehog signaling pathway
an region RNA 6
rder membr: ko00500 Starch and sucrose metabolism
mily 2, sul ko00982,kc Drug metabolism - cytochrome P450|Metabolism of xenobiotics
mitochondrion|peroxisome|membrane|oxidoreductase activity
extracellular region|plasma membrane|cell adhesion|neuron recognition|anchored

l glycan a ko00563 Glycosylphosphatidylinositol(GPI)-anchor biosynthesis
ko04071,kc Sphingolipid signaling pathway|Lysosome|Apoptosis
DNA binding|nucleus|metal ion binding
cytosol|negative regulation of epidermal growth factor-activated receptor activ
DNA-binding transcription factor activity, RNA polymerase II-specific|DNA bindi
actin binding|lysosome|lysosomal membrane|endosome|early endosome|late endosome
neuron migration|MAP-kinase scaffold activity|Golgi apparatus|cytosol|apoptotic
morphogen ko04151,kc PI3K-Akt signaling pathway|ECM-receptor interaction|Focal a
negative r ko03022 Basal transcription factors
nucleus|nucleoplasm|cytoplasm|Golgi apparatus|centrosome|cell cycle|midbody|int
DNA bindir ko03460 Fanconi anemia pathway
lgalactosa ko00512 Mucin type O-Glycan biosynthesis
DNA binding|nucleus|regulation of transcription, DNA-templated|metal ion bindir
pericentriolar material|cytoplasm|cis-Golgi network|centrosome|cytosol|microtub
ATP binding|zinc ion binding|regulation of centrosome duplication|ADP binding|F
nucleolus|PML body|negative regulation of protein ubiquitination|negative regul

GTPase activator activity|nucleus|cytoplasm|trans-Golgi network|regulation of G
calmodulir ko04924,kc Renin secretion|Olfactory transduction|Purine metabolism|Ca
magnesium ko00970 Aminoacyl-tRNA biosynthesis

e, sub-fam ko04975,ko Fat digestion and absorption|ABC transporters
negative regulation of transcription by RNA polymerase II|RNA polymerase II tra
utase ko00630,ko Glyoxylate and dicarboxylate metabolism|Propanoate metaboli
estrogen r ko04062,ko Chemokine signaling pathway|Regulation of actin cytoskeleto
negative regulation of transcription by RNA polymerase II|DNA-binding transcrip
iron ion binding|nucleus|nucleoplasm|nucleolus|oxidoreductase activity, acting
negative r ko04261 Adrenergic signaling in cardiomyocytes
activation of MAPK activity|angiogenesis|extracellular region|Golgi lumen|integ
Golgi memb ko00601 Glycosphingolipid biosynthesis - lacto and neolacto series
A1, 70kDa ko03430,ko Mismatch repair|DNA replication|Fanconi anemia pathway|Homo
negative regulation of transcription by RNA polymerase II|RNA polymerase II pro
eroxide sy ko04923,ko Regulation of lipolysis in adipocytes|Serotonergic synapse|
G2/M transition of mitotic cell cycle|establishment of mitotic spindle orientat
B ko04270,ko Vascular smooth muscle contraction|cGMP-PKG signaling pathw
ribosomal ko03010 Ribosome
microtubule cytoskeleton organization|magnesium ion binding|autophagy of mitoch
endoplasmic reticulum|Golgi apparatus|plasma membrane|drug transmembrane transp
integral component of membrane
transmembrane receptor protein tyrosine kinase activity|transmembrane signaling
serine-type carboxypeptidase activity|cytosol|retinoic acid metabolic process|r
platelet degranulation|ATP binding|extracellular region|integral component of m
ATP binding|cytoplasm|microtubule organizing center|cytosol|I-kappaB kinase/NF-
IgG immunoglobulin transcytosis in epithelial cells mediated by FcRn immunoglob
negative regulation of transcription by RNA polymerase II|RNA polymerase II pro
calcium ion binding|calcium-dependent phospholipid binding|cytoplasm|cytosol|pl
Golgi memb ko00534 Glycosaminoglycan biosynthesis - heparan sulfate / heparin
nucleoplasm|integral component of membrane
actor 10 ko04010,ko MAPK signaling pathway|Rap1 signaling pathway|Regulation of
synaptic transmission, dopaminergic|GTPase activity|GTP binding|plasma membrane
behavioral fear response|GTPase activity|heterotrimeric G-protein complex|plasm
ceptor nuc ko04066 HIF-1 signaling pathway
ko04919,ko Thyroid hormone signaling pathway|Notch signaling pathway|D
negative r ko04550 Signaling pathways regulating pluripotency of stem cells
plasma membrane|integral component of membrane|carbohydrate binding
transporte ko04978,ko Mineral absorption|Bile secretion|cAMP signaling pathway|Ca
protein serine/threonine kinase activity|ATP binding|nucleus|protein phosphoryl
extracellular space|cytosol|positive regulation of neuron projection developmen
transmembrane signaling receptor activity|extracellular region|endoplasmic reti
amyloid-beta binding|in utero embryonic development|cytoplasm|plasma membrane|c
1 (head to head)
nucleoplasm|cytoplasm|cytosol|plasma membrane|phospholipid metabolic process|ph
Golgi cis ko04530,ko Tight junction|Hippo signaling pathway|Hippo signaling path
autophagos ko04140,ko Regulation of autophagy|mTOR signaling pathway|Longevity re
RNA polymerase II proximal promoter sequence-specific DNA binding|proximal prom
e 1
in containing 2
serine-typ ko04610 Complement and coagulation cascades
nucleus|cytoplasm|protein import into nucleus|nuclear localization sequence bin
nucleus|cytoplasm|cytoskeleton|plasma membrane|cell cycle|integral component of
mitochondrion|cadherin binding|extracellular exosome
negative regulation of transcription by RNA polymerase II|RNA polymerase II pro

cytosol|plasma membrane|cell adhesion|anchored component of membrane|locomotory
RNA binding|nucleus|spliceosomal complex|cytosol|regulation of transcription, D
microtubule cytoskeleton organization|spindle|cytosol|microtubule cytoskeleton
eceptor 68
extracellular region|extracellular space|response to lipopolysaccharide|interle
d activat ko04062,kc Chemokine signaling pathway|Toll-like receptor signaling pa
RNA binding|protein monoubiquitination|protein ubiquitination|ribosome binding|
DNA-binding transcription factor activity, RNA polymerase II-specific|DNA bindi
retinoid metabolic process|blood vessel development|kidney development|pulmonar
integral component of membrane
DNA-binding transcription factor activity, RNA polymerase II-specific|DNA bindi
ix repeat containing 1
eading frame 16
ribosomal ko03008,kc Ribosome biogenesis in eukaryotes|RNA transport
e, sub-family F (GCN20), member 2
linum toxi ko04520,kc Adherens junction|Leukocyte transendothelial migration|Axon
or, alpha- ko04621,kc NOD-like receptor signaling pathway|TNF signaling pathway|N
otein 2
x 3 ko04550 Signaling pathways regulating pluripotency of stem cells
ng enzyme 1
RNA bindir ko03018,kc RNA degradation|mRNA surveillance pathway|RNA transport
peroxidase activity|interleukin-1 receptor antagonist activity|extracellular ma
DNA-binding transcription factor activity, RNA polymerase II-specific|DNA bindi
x protein 1
extracellular region|plasma membrane|nervous system development|cell junction|a
retinoid n ko00980,kc Metabolism of xenobiotics by cytochrome P450|Steroid hormon
dase 11
MAPK casca ko04722,kc Neurotrophin signaling pathway|MAPK signaling pathway|GnRH
containing 85A
branching ko04310 Wnt signaling pathway
iron ion binding|endoplasmic reticulum|negative regulation of cell proliferatio
ce associated
structural constituent of cytoskeleton|cytoplasm|cytosol|cytoskeleton|plasma me
astosis vi ko04151 PI3K-Akt signaling pathway
lGDS/AF-6) ko04392,kc Hippo signaling pathway -multiple species|Hippo signaling p
cytosol|membrane|protein homodimerization activity|extracellular exosome
complement ko04080 Neuroactive ligand-receptor interaction
cytoplasm
A3, 14kDa ko03430,kc Mismatch repair|DNA replication|Fanconi anemia pathway|Homo
magnesium ion binding|O-glycan processing|manganese ion binding|integral compor
platelet degranulation|translation elongation factor activity|GTPase activity|G
calmodulir ko04013 MAPK signaling pathway - fly
RNA polymerase II proximal promoter sequence-specific DNA binding|DNA-binding t
DNA-binding transcription factor activity, RNA polymerase II-specific|DNA bindi
Golgi membrane|trans-Golgi network|plasma membrane|ion transport|regulation of
ATP bindir ko04621 NOD-like receptor signaling pathway
calcium ion binding|integral component of membrane
cilium movement|motile cilium|inner dynein arm assembly|dynein complex binding|
ko04066,kc HIF-1 signaling pathway|Methane metabolism|RNA degradation|
NAD+ kinas ko00564,kc Glycerophospholipid metabolism|Phospholipase D signaling pa

protein kinase activity|protein tyrosine kinase activity|transmembrane receptor

nucleus|cytosol|fatty acid metabolic process|zinc ion binding
cytoplasmic microtubule|plasma membrane|bicellular tight junction|cell surface|
ko04360 Axon guidance
mRNA splicing, via spliceosome|RNA binding|nucleus|nucleoplasm|Cajal body|nucle
ossificati ko04662 B cell receptor signaling pathway
protein pc ko04120 Ubiquitin mediated proteolysis
negative regulation of transcription by RNA polymerase II|RNA polymerase II reg
Golgi apparatus|integral component of membrane|transferase activity, transferri
maturation of SSU-rRNA from tricistronic rRNA transcript (SSU-rRNA, 5.8S rRNA,
cation channel activity|endoplasmic reticulum|endoplasmic reticulum membrane|pl
in utero e ko00310,kc Lysine degradation|Other types of O-glycan biosynthesis
DNA binding|RNA binding|double-stranded RNA binding|single-stranded RNA binding
MAPK casca ko04920,kc Adipocytokine signaling pathway|Oxytocin signaling pathway|
11A (zinc finger protein)
(gene/pseudogene)
RNA polyme ko04350 TGF-beta signaling pathway

ding protein 1
suckling t ko04120 Ubiquitin mediated proteolysis
magnesium ion binding|phospholipid-translocating ATPase activity|ATP binding|en
extracellular space|intracellular|cytosol|identical protein binding|presynapse
ein 2 ko04530 Tight junction
skeletal system development|osteoclast proliferation|intracellular|integral com
lens morph ko04530 Tight junction
plasma membrane|immune response|response to virus|integral component of membran
protein ki ko04711,kc Circadian rhythm - fly|Hippo signaling pathway - fly|Hippo
GTPase activity|GTP binding|cytoplasm|cytosol|plasma membrane|cell cortex|endoc
DNA-bindir ko04916,kc Melanogenesis|Adherens junction|Hippo signaling pathway|Wnt
RNA-directed DNA polymerase activity|DNA recombination|type II site-specific de
skeletal muscle contraction|sarcoplasmic reticulum|sarcoplasmic reticulum membr
nucleosome|DNA binding|nucleus|defense response to bacterium|protein heterodime
cytoplasm|multicellular organism development|spermatogenesis|cell differentiati
tRNA bindi ko00970 Aminoacyl-tRNA biosynthesis
protein mc ko04114,kc Oocyte meiosis|Protein processing in endoplasmic reticulum|
coupled receptor L2
protein pc ko04120,kc Ubiquitin mediated proteolysis|Hedgehog signaling pathway -
heterochromatin|DNA binding|nucleus|nucleoplasm|multicellular organism developm
endotheli ko04010,kc MAPK signaling pathway|Osteoclast differentiation|NF-kappa
actin binding|nucleus|cytoplasm|cytoskeleton|enzyme binding|defense response to
box 2
translati ko04914,kc Progesterone-mediated oocyte maturation|Oocyte meiosis|Dors
ovarian cumulus expansion|complement component C1q binding|(1->3)-beta-D-glucan

negative regulation of transcription by RNA polymerase II|RNA polymerase II reg
nuclear chromatin|cytoplasm|DNA replication-dependent nucleosome assembly|DNA r
microtubule bundle formation|neuron migration|cytoskeleton|axonogenesis|zinc ic
structural constituent of cytoskeleton|extracellular space|nucleoplasm|neurofil
nucleoplasm|lipid binding|cellular response to starvation|protein transport|syr
Golgi mem ko04650,kc Natural killer cell mediated cytotoxicity|Endocytosis|Phago

endotheliako04610 Complement and coagulation cascades
GTPase activator activity|intracellular|intracellular protein transport|Rab GTF
26070
nase complko01200,kcCarbon metabolism|Oxidative phosphorylation|Citrate cycle (
glucosamirko00520 Amino sugar and nucleotide sugar metabolism
19kDa interacting protein 2
protein kinase activity|protein serine/threonine kinase activity|ATP binding|cy
protein transport|cytoplasmic vesicle membrane|phosphatidylinositol binding
extracellular region|endoplasmic reticulum|Golgi apparatus|response to manganes
RNA bindirko03008 Ribosome biogenesis in eukaryotes
or receptoko04060 Cytokine-cytokine receptor interaction
1 ko04060,kcCytokine-cytokine receptor interaction|Jak-STAT signaling p
ferric-cheko04978 Mineral absorption
alpha-1,2-ko00563 Glycosylphosphatidylinositol(GPI)-anchor biosynthesis
in 2-like 1
voltage-gako04924,kcRenin secretion|GABAergic synapse|Dopaminergic synapse|Adre
neuron migration|protein kinase activity|protein serine/threonine kinase activi
Golgi membrane|endoplasmic reticulum membrane|proteolysis|cell proliferation|pr
chitinase activity|extracellular space|lysosome|chitin catabolic process|chitir
rnally expressed transcript (non-protein coding)
multicellular organism development|identical protein binding|protein homooligom
ko04080,kcNeuroactive ligand-receptor interaction|cAMP signaling path
angiogeneko04151,kcPI3K-Akt signaling pathway|ECM-receptor interaction|Focal a
RNA polymeko03022 Basal transcription factors
otein 5 (gko04918,kcThyroid hormone synthesis|Protein processing in endoplasmic
antigen processing and presentation of peptide antigen via MHC class I|immune r

Golgi membrane|nucleus|early endosome|late endosome|Golgi apparatus|cytosol|pla
G protein-coupled receptor activity|intracellular|plasma membrane|integral comp
tion-related protein 2-like
galactosylko04142,kcLysosome|Sphingolipid metabolism
DNA-binding transcription factor activity, RNA polymerase II-specific|nucleic a
Golgi membko04650,kcNatural killer cell mediated cytotoxicity|Endocytosis|Phago
ko04610 Complement and coagulation cascades
nucleus|cytoplasm|zinc ion binding|protein ubiquitination|transferase activity
neural creko04360 Axon guidance
mitochondrial genome maintenance|mitochondrial promoter sequence-specific DNA b
integral component of membrane
voltage-gako04010,kcMAPK signaling pathway|Adrenergic signaling in cardiomyocyt
hematopoietic progenitor cell differentiation|protein tyrosine phosphatase acti
collagen ko04810,kcRegulation of actin cytoskeleton|PI3K-Akt signaling pathway

Golgi membko00534 Glycosaminoglycan biosynthesis - heparan sulfate / heparin
negative regulation of transcription by RNA polymerase II|RNA polymerase II pro
nucleus|nuclear speck|dendrite|response to immobilization stress|histone bindir
pseudouridine synthesis|RNA binding|mitochondrial matrix|mRNA processing|pseudo
activatiorko04071,kcSphingolipid signaling pathway|Neuroactive ligand-receptor
G protein-ko04726,kcSerotonergic synapse|Ras signaling pathway|Calcium signalin
negative regulation of transcription by RNA polymerase II|osteoblast differenti
Golgi membrane|amyloid-beta binding|regulation of cell growth|calcium ion bindi
endosome|membrane

cytosol|smko04014   Ras signaling pathway
GTPase ac1ko04924,kcRenin secretion|Gap junction|Leukocyte transendothelial mig
plasma membrane|integral component of membrane
serine-type endopeptidase activity|nucleus|mitochondrial inner membrane|integra
trans-Golgi network|intracellular protein transport|cytoplasmic vesicle|vesicle

establishment of mitotic spindle orientation|metanephros development|ureteric b
DNA binding|RNA binding|helicase activity|ATP binding|nucleus|cytosol|membrane
DNA bindirko04974   Protein digestion and absorption
nucleus|mitochondrion
Golgi membrane|cytosol|intra-Golgi vesicle-mediated transport|Rab guanyl-nucleo
ng 1
cell adhesion|integral component of membrane
DNA binding|RNA-directed DNA polymerase activity|RNA-DNA hybrid ribonuclease ac
carboxypeptidase activity|plasma membrane|proteolysis|serine-type peptidase act
or recepto:ko04060,kcCytokine-cytokine receptor interaction|Osteoclast different
ko04514,kcCell adhesion molecules (CAMs)|Leukocyte transendothelial m
cardiac ventricle development|mitochondrial inner membrane|plasma membrane|inte
-protein coding RNA 1592
cytosol|prko04622   RIG-I-like receptor signaling pathway
p53 binding|transcription coactivator activity|transcription corepressor activi
SNARE bincko04962,kcVasopressin-regulated water reabsorption|SNARE interactions
pericentriolar material|extracellular space|cytoplasmic dynein complex|cilium|m
DNA binding|ubiquitin-protein transferase activity|nucleus|nucleoplasm|cytoplas
acid (GABA`ko04723,kcRetrograde endocannabinoid signaling|GABAergic synapse|Neur
histone deacetylase complex|negative regulation of transcription by RNA polymer
G1/S transition of mitotic cell cycle|Golgi membrane|podosome|GTPase activity|G
ossificatiko04270   Vascular smooth muscle contraction
G protein-ko04650,kcNatural killer cell mediated cytotoxicity|Cholinergic synap
cytoplasm|negative regulation of protein kinase activity
cytoplasm|ko00520,kcAmino sugar and nucleotide sugar metabolism|Starch and sucr
otor (alphako04540,kcGap junction|Phagosome
activation of MAPK activity|protein serine/threonine kinase activity|ATP bindir
Golgi membrane|GTPase activity|GTP binding|nucleus|nucleoplasm|cytoplasm|Golgi
copper ion binding|peroxisome

ubiquitin ligase complex|ubiquitin-protein transferase activity|nucleus|histone
transcript ko04330,kcNotch signaling pathway|Cell cycle|Thyroid hormone signalin
angiogenesis|hyaluronoglucosaminidase activity|calcium ion binding|integral co
GTPase ac1ko04214   Apoptosis - fly
cytokine activity|transforming growth factor beta receptor binding|extracellula
transcript ko03018   RNA degradation
mitochondrko00920   Sulfur metabolism

negative regulation of cellular extravasation|guanyl-nucleotide exchange factor
angiogenesis|GTPase activator activity|nucleus|cytosol|focal adhesion|signal tr
guanyl-nucleotide exchange factor activity|Rho guanyl-nucleotide exchange facto

id recepto:ko04540,kcGap junction|Rap1 signaling pathway|Phospholipase D signali
stress fibko04510   Focal adhesion

integral component of membrane

otein (syntenin)
telomere maintenance via recombination|double-strand break repair via homologou
DNA binding|nucleus|regulation of transcription, DNA-templated|multicellular or
Golgi apparatus|microtubule binding|kinase binding
Golgi membrane|amyloid-beta binding|transmembrane signaling receptor activity|l
osteoblast differentiation|hair follicle development|transmembrane signaling re
DNA-binding transcription factor activity, RNA polymerase II-specific|metanephr
RNA bindirko04510,kcFocal adhesion|Regulation of actin cytoskeleton
nor protein 9
glutamine-ko00250,kcAlanine, aspartate and glutamate metabolism|Amino sugar and
RNA polymerase II proximal promoter sequence-specific DNA binding|DNA-binding t

mitochondrion
ected) iotako03460 Fanconi anemia pathway
protein disulfide isomerase activity|extracellular region|endoplasmic reticulum

GTPase actko04144 Endocytosis
nuclear chko04010,kcMAPK signaling pathway|cGMP-PKG signaling pathway
cytoplasm|early endosome|late endosome|Golgi apparatus|intracellular protein tr
Golgi membrane|trans-Golgi network|cytosol|negative regulation of neuron projec
cytochromeko00190 Oxidative phosphorylation
stress fiber|actin binding|nucleus|bicellular tight junction|visual learning|pc
ly 33 (ace ko00604 Glycosphingolipid biosynthesis - ganglio series
n splicing ko03040 Spliceosome
ko04066,kcHIF-1 signaling pathway|Insulin signaling pathway|Carbohydr
nucleus|cytosol|plasma membrane
l
MAPK cascade|protein serine/threonine phosphatase activity|endoplasmic reticulu
adaptive immune response|antigen processing and presentation of peptide antigen
nucleus|nucleoplasm|mitochondrion|mitochondrial inner membrane|integral compone
glutamate ko00910,kcNitrogen metabolism|D-Glutamine and D-glutamate metabolism|
establishment of mitotic spindle orientation|mitotic cytokinesis|chromosome, ce
containing 33
transmembrane signaling receptor activity|extracellular ligand-gated ion channe
integral component of membrane
nucleoplasm
multicellular organism development
ATP transmembrane transporter activity|calcium ion binding|mitochondrion|mitoch
zinc ion binding|catabolic process|hydrolase activity, acting on ester bonds|ep
ding prote ko04072,kcPhospholipase D signaling pathway|Osteoclast differentiatio
integral component of membrane
protease binding|nucleus|cytoplasm|cytosol|activation of cysteine-type endopept
nucleoplasko04914,kcProgesterone-mediated oocyte maturation|Oocyte meiosis|Cell
negative regulation of transcription by RNA polymerase II|DNA-binding transcrip
binding prko04725,kcCholinergic synapse|Gap junction|Long-term depression|Aldos
transcription coactivator activity|nucleus|cytoplasm|mitochondrion|regulation c
nuclear-transcribed mRNA poly(A) tail shortening|nucleus|nuclear heterochromati
GTPase activator activity|intracellular|plasma membrane|intracellular protein t
t 3 ko04610,kcComplement and coagulation cascades|Phagosome

kinetochore|condensed chromosome kinetochore|chromatin binding|nucleus|nuclear
NAD+ diphcko00760,kcNicotinate and nicotinamide metabolism|Peroxisome
negative regulation of transcription by RNA polymerase II|transcription corepre
nucleus|plko04060   Cytokine-cytokine receptor interaction
extracellular region|positive regulation of biomineral tissue development|posit
opioid receptor activity|membrane
endoplasmic reticulum|endoplasmic reticulum membrane|integral component of memb
RNA-directed DNA polymerase activity|DNA recombination|type II site-specific de
calcium ion binding|nucleus|calcium-mediated signaling|enzyme regulator activit
RNA splicko03040   Spliceosome
temperature homeostasis|lysosome|endosome|early endosome|plasma membrane|negati
catalytic activity|triglyceride lipase activity|endoplasmic reticulum membrane|
metalloendopeptidase activity|protein processing involved in protein targeting
prostaglarko00590   Arachidonic acid metabolism
peptidyltrko00460,kcCyanoamino acid metabolism|Glutathione metabolism|Arachidon
MAPK cascako04011,kcMAPK signaling pathway - yeast|Neurotrophin signaling pathw
ne 4
microtubule motor activity|nucleoplasm|mitochondrion|cytosol|kinesin complex|mi
mRNA 3'-UTR binding|NADPH:quinone reductase activity|cytosol|visual perception|
cytosol|nuko00920   Sulfur metabolism
negative regulation of transcription by RNA polymerase II|cytosol|kinase bindir
nucleus
in utero embryonic development|tissue homeostasis|nucleus|nucleoplasm|cytosol|p
cytoplasm|mitochondrion|centrosome|cytosol|plasma membrane|apoptotic process|ce
ko04978,kcMineral absorption|Porphyrin and chlorophyll metabolism
fatty acicko00561,kcGlycerolipid metabolism|Fatty acid degradation|Tryptophan m
serine-type endopeptidase activity|extracellular region|extracellular space|cyt

plasma membrane|ubiquitin-dependent protein catabolic process|extracellular tra
postsynaptic density|cell junction|postsynaptic membrane
negative rko04310   Wnt signaling pathway
tif) ligand 14
RNA polymerase II proximal promoter sequence-specific DNA binding|DNA-binding t
ruffle|phcko00562,kcInositol phosphate metabolism|Phosphatidylinositol signalin
C (activatko03430,kcMismatch repair|DNA replication|Nucleotide excision repair
l acyltranko00564   Glycerophospholipid metabolism
transmembrane signaling receptor activity|plasma membrane|cell surface|anchorec
renal watcko04540,kcGap junction|GABAergic synapse|Rap1 signaling pathway|Longe
intracellular|intracellular signal transduction
RNA bindirko03013   RNA transport
1-acylglycko00564,kcGlycerophospholipid metabolism|Ether lipid metabolism
cornified envelope|positive regulation of cell-matrix adhesion|desmosome assemb
nase inhibko04066,kcHIF-1 signaling pathway|p53 signaling pathway|Cell cycle|PI
nucleoplasm|mitochondrion|mitochondrial inner membrane|integral component of mi
platelet activating factor receptor activity|platelet-derived growth factor bet
calcium ion binding|integral component of plasma membrane|cell adhesion|homophi
angiogenesis|membrane raft assembly|phosphatidylserine binding|positive regulat
actin moncko04810   Regulation of actin cytoskeleton
dase 8
ubiquitin-ko04150   mTOR signaling pathway
Golgi memko00533   Glycosaminoglycan biosynthesis - keratan sulfate

olate-homocysteine methyltransferase reductase
cytosol|plasma membrane|clathrin-coated pit|endocytosis|protein transport|clath

calcium-dependent cysteine-type endopeptidase activity|calcium ion binding|cyto
mitotic cytokinesis|septin ring assembly|GTPase activity|GTP binding|septin rin
DNA-binding transcription factor activity, RNA polymerase II-specific|DNA bindi
cytoplasıko00625,kcChloroalkane and chloroalkene degradation|Arachidonic acid

nucleic acid binding|metal ion binding
extracellular matrix structural constituent|collagen binding|extracellular regi
integral component of plasma membrane|chemotaxis|inflammatory response|positive
proteasomeko03050 Proteasome
nicotinateko00760 Nicotinate and nicotinamide metabolism
dopamine beta-monooxygenase activity|copper ion binding|extracellular space|cyt
negative regulation of transcription by RNA polymerase II|RNA polymerase II reg
renal wateko04924,kcRenin secretion|Gap junction|GABAergic synapse|Rap1 signali
negative regulation of transcription by RNA polymerase II|RNA polymerase II tra
a 1,3-galaıko00534,kcGlycosaminoglycan biosynthesis - heparan sulfate / heparin|
choline kiko00564 Glycerophospholipid metabolism
Golgi membrane|nucleic acid binding|GTPase activity|ubiquitin-protein transfera
trans-Golgi network|cytosol|plasma membrane|protein localization|endomembrane s
response to ischemia|endoplasmic reticulum|Golgi apparatus|apoptotic process|ir
retinoic acid binding|retinoic acid biosynthetic process|retinoid binding|nucle
GTPase activator activity|nucleus|metal ion binding

particle 9 ko03060 Protein export
activatiorko04622,kcRIG-I-like receptor signaling pathway|Cytosolic DNA-sensing
integral component of membrane
cytosol|protein deubiquitination|NEDD8-specific protease activity|post-translat
ase ko00230,kcPurine metabolism|Alanine, aspartate and glutamate metaboli
voltage-gako04261 Adrenergic signaling in cardiomyocytes
mitotic cytokinesis|septin ring assembly|GTPase activity|GTP binding|septin rin

endoplasmic reticulum|calcium ion transport|integral component of endoplasmic r
nuclear-transcribed mRNA poly(A) tail shortening|nucleus|cytosol|regulation of
negative regulation of transcription by RNA polymerase II|cytoplasm|mitochondri
3'-5'-exorko00230,kcPurine metabolism|Pyrimidine metabolism|RNA degradation
-protein coding RNA 856
menting deko03410 Base excision repair
plasma membrane|integral component of membrane|regulation of immune response
tif) ligand 2
actin binding|nucleus|nucleoplasm|cytosol|actin cytoskeleton|nuclear speck|Z di
kidney devko04310 Wnt signaling pathway
ko04922,kcGlucagon signaling pathway|Longevity regulating pathway|AMP
translation initiation factor activity
Golgi cis cisterna|Golgi membrane|GTPase activity|GTP binding|endoplasmic retic
ase 1 famiko00830 Retinol metabolism
nuclear envelope|nuclear outer membrane|cytoplasm|rough endoplasmic reticulum|c
DNA binding|nucleus|regulation of transcription, DNA-templated|metal ion bindir
oribosyltrako00760 Nicotinate and nicotinamide metabolism
e similarity 107, member B

liver deveko00230   Purine metabolism
nucleus|nuclear envelope|nucleoplasm|nuclear euchromatin|cytoplasm|postsynaptic
RNA binding|structural molecule activity|cytoplasm|cytoskeleton|bicellular tigh
nucleus|nuko04392,kcHippo signaling pathway -multiple species|Cell cycle - yeas
extracellular region|nucleus|endoplasmic reticulum|membrane
regulatiorko00190,kcOxidative phosphorylation|Cardiac muscle contraction
1   ko04666,kcFc gamma R-mediated phagocytosis|Endocytosis
microtubule motor activity|ATP binding|cytoplasm|kinesin complex|microtubule|mi
RNA 7-methylguanosine cap binding|P-body|RNA polymerase II complex binding|core
nucleosomal binding domain 2
2 pseudogeko04120   Ubiquitin mediated proteolysis
structuralko04514   Cell adhesion molecules (CAMs)
structuralko04214,kcApoptosis - fly|Apoptosis
lysophosphko04921,kcOxytocin signaling pathway|Glycerophospholipid metabolism|a
          ko04060,kcCytokine-cytokine receptor interaction|Neuroactive ligand-r
intracellular|nucleus|nucleoplasm|cytosol|protein import into nucleus|Golgi org
photorecepko04062,kcChemokine signaling pathway|Cholinergic synapse|Serotonergi
nucleus|DNA repair|cellular response to DNA damage stimulus|mitotic cell cycle
endoplasmic reticulum lumen|glucosyltransferase activity
nucleus|cytoplasm|mitochondrion|mitochondrial inner membrane|response to woundi
negative regulation of transcription by RNA polymerase II|DNA-binding transcrip
integral component of membrane
Golgi membko00534   Glycosaminoglycan biosynthesis - heparan sulfate / heparin

cytosol|intracellular protein transport|early endosome membrane|early endosome
1
ubiquitin-protein transferase activity|cytosol|EP4 subtype prostaglandin E2 rec
          ko04390   Hippo signaling pathway
Golgi membko00532   Glycosaminoglycan biosynthesis - chondroitin sulfate / derm
cytosol|membrane|protein homodimerization activity|extracellular exosome
podosome|pko04611   Platelet activation
sucrose:proton symporter activity|sucrose transport|integral component of membr
RNA polymeko04920,kcAdipocytokine signaling pathway|PPAR signaling pathway|Thyr
ne kinase
DNA-binding transcription factor activity, RNA polymerase II-specific|DNA bindi
guanyl-nucko04062   Chemokine signaling pathway
fructose-bko00710,kcCarbon fixation in photosynthetic organisms|Methane metabol
          ko03460,kcFanconi anemia pathway|Mismatch repair

II (thrombko04080,kcNeuroactive ligand-receptor interaction|Axon guidance|Compl
in 52
regulation of cyclin-dependent protein serine/threonine kinase activity|mitotic
ubiquitin ligase complex|protein polyubiquitination|cytoplasm|ubiquitin-depende
snoRNA guiko03008   Ribosome biogenesis in eukaryotes
l
carnitine ko00071,kcFatty acid degradation|Glucagon signaling pathway|AMPK sign
box H/ACA snoRNP assembly|nucleoplasm|cytoplasm|cytosol|ribonucleoprotein compl
extracelluko04974,kcProtein digestion and absorption|PI3K-Akt signaling pathway
RNA bindirko04151   PI3K-Akt signaling pathway
protein serine/threonine phosphatase inhibitor activity|positive regulation of

platelet degranulation|nucleic acid binding|endonuclease activity|extracellular
nucleus|cytoplasm|cytosol|transcription factor binding|zinc ion binding|protein
clathrin-coated pit|receptor-mediated endocytosis|integral component of membran
DNA-binding transcription factor activity, RNA polymerase II-specific|positive
GTPase ac ko04144,kc Endocytosis|Regulation of actin cytoskeleton
endoplasmic reticulum|Golgi apparatus|protein targeting to membrane|integral cc
calcium ion binding|extracellular region|cytoplasm|cellular response to virus
nuclear ch ko04330,kc Notch signaling pathway|Melanogenesis|mTOR signaling pathwa
3-hydroxya ko00071,kc Fatty acid degradation|Tryptophan metabolism|Lysine degrada
guanyl-nucleotide exchange factor activity|Rho guanyl-nucleotide exchange facto
integral component of membrane
voltage-ga ko04742 Taste transduction
growth pla ko04974 Protein digestion and absorption

chromatin binding|nucleus|nucleoplasm|mitochondrion|cytosol|nuclear membrane
-protein coding RNA 578
lysosomal membrane|endoplasmic reticulum|organelle organization|blood coagulati
G protein-coupled receptor activity|calcium ion binding|plasma membrane|integra
epeat domain 39C
Golgi membrane|plasma membrane|endosome membrane|negative regulation of SMAD pr
e, sub-fam ko02010 ABC transporters

dase 2 ko04915,kc Estrogen signaling pathway|Leukocyte transendothelial migra
histone de ko04330 Notch signaling pathway
plasma membrane|integral component of plasma membrane|sodium-independent organi
integral component of membrane
nuclear-tr ko03010 Ribosome
coproporph ko00860 Porphyrin and chlorophyll metabolism
nibitor, c ko04610 Complement and coagulation cascades
nucleus|WW domain binding
regulation of hippo signaling|metal ion binding
RNA binding|phenylalanine-tRNA ligase activity
cell morphogenesis|spindle pole|enzyme inhibitor activity|microtubule organizin
globulin de ko04360 Axon guidance
cytokine r ko04060,kc Cytokine-cytokine receptor interaction|Jak-STAT signaling p
[II (throm ko04610 Complement and coagulation cascades
uropod|reg ko04972,kc Pancreatic secretion|Nicotinate and nicotinamide metabolism
pithelial a ko04978 Mineral absorption
desmosome ko04115 p53 signaling pathway
cytoplasm|Golgi apparatus|Golgi cisterna membrane|metal ion binding|perinuclear
angiogenes ko04014,kc Ras signaling pathway|PI3K-Akt signaling pathway|Focal adhe
tor, alpha
actin binding|nucleus|cytoplasm|cytosol|cytoskeleton|nuclear migration|intercal
extracellular region|endoplasmic reticulum|lipid droplet|methyltransferase acti
RNA bindir ko03040 Spliceosome
ruffle|guanyl-nucleotide exchange factor activity|Rho guanyl-nucleotide exchang
skeletal system development|microfibril|metanephros development|integrin bindir
a 2 (X-linked recessive)
Golgi mem ko00512 Mucin type O-Glycan biosynthesis
in utero embryonic development|glutamatergic synapse|postsynaptic density, intr
negative regulation of transcription by RNA polymerase II|nucleus|double-strand

magnesium ko04341,kcHedgehog signaling pathway - fly|Hedgehog signaling pathway
e II, TATA ko03022   Basal transcription factors
e similarity 124A
signal pepko03060   Protein export
inositol ko04924,kcRenin secretion|Gap junction|Glucagon signaling pathway|Lon
DNA binding|nucleus|transcription, DNA-templated|regulation of transcription, D
Golgi membko04141   Protein processing in endoplasmic reticulum
DNA-binding transcription factor activity, RNA polymerase II-specific|DNA bindi
plasma membrane|apoptotic process|cell communication|membrane|integral componen
nucleolus|ko04392,kcHippo signaling pathway -multiple species|Cell cycle - yeas
semaphorirko04360   Axon guidance
DNA-binding transcription factor activity, RNA polymerase II-specific|proximal
DNA-binding transcription factor activity, RNA polymerase II-specific|DNA-bindi
DNA binding|single-stranded DNA binding|mRNA binding|endoribonuclease activity|
nucleus|positive regulation of neuron differentiation|protein heterodimerizatic
mitochondrion|Golgi apparatus|cytosol|locomotory behavior|integral component of
placenta development|metalloendopeptidase activity|nucleus|Golgi apparatus|plas
microtubule cytoskeleton organization|cytoplasm|Golgi apparatus|centrosome|cytc
A 1
liver deveko04514   Cell adhesion molecules (CAMs)

negative regulation of transcription by RNA polymerase II|transcription regulat
skeletal system development|extracellular matrix structural constituent|extrace
actor 1 (ako04390,kcHippo signaling pathway|MAPK signaling pathway|Rap1 signali
mitochondrial genome maintenance|mitochondrion|mitochondrial inner membrane|pyr
NAD+ ADP-ribosyltransferase activity|nucleus|nucleolus|cytoplasm|cytosol|protei
cytoplasm|ko00565   Ether lipid metabolism
plasma membrane|cytoplasmic side of plasma membrane|integral component of membr
cytoplasm|lipid transport|phagocytosis, engulfment|apoptotic process
cytoplasm|centrosome|cilium|smoothened signaling pathway|protein C-terminus bir
ion transport|integral component of membrane|transmembrane transporter activity
l binding clathrin assembly protein
Golgi membko04142   Lysosome
stress fibko04530   Tight junction
phosphaticko00562   Inositol phosphate metabolism
DNA binding|lamin binding|nucleus|nuclear envelope|nuclear inner membrane|cytop
Ras guanylko04014   Ras signaling pathway

nuclear envelope|endoplasmic reticulum membrane|negative regulation of myotube
nuclear chromosome, telomeric region|condensed nuclear chromosome|lamin binding
lens development in camera-type eye|Golgi apparatus|bicellular tight junction|f
growth plako04974,kcProtein digestion and absorption|PI3K-Akt signaling pathway
structural molecule activity|cytoplasm|cytosol|intermediate filament|keratiniza
RNA binding|guanyl-nucleotide exchange factor activity|nucleolus|cytosol|microt
binding cako04145,kcPhagosome|Antigen processing and presentation|ABC transport
mily 27, subfamily C, polypeptide 1
membrane raft assembly|T cell antigen processing and presentation|double-stranc
rotein kinko04668,kcTNF signaling pathway|Toll-like receptor signaling pathway|
membrane|integral component of membrane|regulation of Wnt signaling pathway|cil
ubiquitin ligase complex|proteasome-mediated ubiquitin-dependent protein catabc
DNA-binding transcription factor activity, RNA polymerase II-specific|female pr

semaphorirko04360   Axon guidance
RNA binding
se inhibitor 2
ATP bindirko04015,kcRap1 signaling pathway|Tight junction
thioredoxiko00240,kcPyrimidine metabolism|Selenocompound metabolism
extracellular matrix structural constituent|calcium ion binding|basement membra
plasma menko04966   Collecting duct acid secretion

activatiorko04620,kcToll-like receptor signaling pathway|MAPK signaling pathway
G protein-coupled receptor binding|G protein-coupled receptor activity|extracel
ATP binding|lysosome|lysosomal membrane|late endosome|multivesicular body|autop

stress fiko04024,kccAMP signaling pathway|Oxytocin signaling pathway|Leukocyte

transcriptko04520,kcAdherens junction|Regulation of actin cytoskeleton
integral component of membrane
tryptopharko00380   Tryptophan metabolism
tein   ko04071,kcSphingolipid signaling pathway|Lysosome|Apoptosis
thiol-dependent ubiquitin-specific protease activity|cytoplasm|plasma membrane|
magnesium ion binding|RNA binding|ATP-dependent RNA helicase activity|ATP bindi
lymphocyteko04978   Mineral absorption
mitochondrko00190   Oxidative phosphorylation
Golgi membrane|Golgi stack|cytosol|ER to Golgi vesicle-mediated transport|intra
in 31
ubiquitin-ko04120   Ubiquitin mediated proteolysis
bisphosphcko00260,kcGlycine, serine and threonine metabolism|Methane metabolism
cytoplasm|cytoskeleton|adherens junction|bicellular tight junction|protein binc
DNA-binding transcription factor activity, RNA polymerase II-specific|DNA bindi
endoplasmic reticulum|endoplasmic reticulum membrane|integral component of memb
structural molecule activity|cytosol|intermediate filament|Z disc|neuromuscular
tRNA bindiko00970   Aminoacyl-tRNA biosynthesis
neuron migko04144   Endocytosis
opioid peptide activity|osteoblast differentiation|behavioral fear response|res
ruffle|ATFko04966,kcCollecting duct acid secretion|Phagosome|Oxidative phosphor
neural tube closure|phosphatidylinositol-4,5-bisphosphate binding|extracellular
cytoplasm
angiogenesis|integral component of plasma membrane|cell surface receptor signal
negative regulation of transcription by RNA polymerase II|DNA-binding transcrip
integral component of plasma membrane|monocarboxylic acid transmembrane transpc
calcium ion binding|nucleolus|endoplasmic reticulum|endoplasmic reticulum lumen
acrosomal vesicle|mRNA binding|cytoplasm|plasma membrane|cell cortex|endocytosi
actin binding|Golgi apparatus|microtubule|cytoplasmic microtubule|cilium|microt
cytoplasm|plasma membrane
negative regulation of systemic arterial blood pressure|G protein-coupled recep
negative rko04013   MAPK signaling pathway - fly
structural molecule activity|nucleus|cytoplasm|cytosol|intermediate filament|vi
detection ko04270,kcVascular smooth muscle contraction|cGMP-PKG signaling pathw
skeletal sko04340   Hedgehog signaling pathway
tor   ko04060,kcCytokine-cytokine receptor interaction|Jak-STAT signaling p
G2/M transko04110   Cell cycle
integral component of membrane|transport vesicle

ace associated

GDP-dissociation inhibitor activity|nucleus|cytoplasm|clathrin-coated pit|endoc
RNA-directed DNA polymerase activity|DNA recombination|type II site-specific de
cell cortex|zinc ion binding|positive regulation of cell proliferation|hemopoie
ko04068,kcFoxO signaling pathway|Progesterone-mediated oocyte maturat
nucleosome|nuclear chromatin|DNA binding|chromatin organization|protein heteroc
endoplasmiko04141 Protein processing in endoplasmic reticulum
lgalactosarko00512 Mucin type O-Glycan biosynthesis
cytosol|irko04144 Endocytosis
GTPase activator activity|cytoplasm|cytosol|plasma membrane|small GTPase mediat
hyaluronic acid binding|cell adhesion|Notch signaling pathway|integral componer
cytoplasm
condensed chromosome kinetochore|motor activity|nucleus|cytoplasm|cytoplasmic c
tRNA processing|FMN binding|metal ion binding|4 iron, 4 sulfur cluster binding|

s-related (ko04650,kcNatural killer cell mediated cytotoxicity|TNF signaling pat
Notch binding|plasma membrane|cell adhesion|homophilic cell adhesion via plasma
angiogenesko04360 Axon guidance
sulfate asko00230,kcPurine metabolism|Sulfur metabolism|Monobactam biosynthesis
1B ko04978 Mineral absorption
cytosol|plasma membrane|multicellular organism development|integral component c
nuclear chko04915,kcEstrogen signaling pathway|TGF-beta signaling pathway
Rho guanyl-nucleotide exchange factor activity|plasma membrane|positive regulat
osphatase, non-receptor type 14
GTPase activity|ATP binding|GTP binding|cytoplasm|cytosol|plasma membrane|cell
motor activity|microtubule motor activity|ATP binding|cytosol|kinesin complex|m
glucose transmembrane transporter activity|glucose binding|plasma membrane|inte
positive regulation of protein phosphorylation|early endosome|vesicle|positive
igase ko00910,kcNitrogen metabolism|Glyoxylate and dicarboxylate metabolism
angiogenesis|branching involved in blood vessel morphogenesis|semaphorin recept
endoplasmic reticulum membrane|protein transport|membrane|integral component of
phosphatidylinositol-4,5-bisphosphate binding|phosphatidylinositol-3,4,5-trisph
heart valve morphogenesis|ventricular cardiac muscle tissue development|metallc
ATP bindirko04261,kcAdrenergic signaling in cardiomyocytes|Cardiac muscle contr
transcription corepressor activity|nucleus|cytoplasm|cytosol|cellular heat accl
ko04610 Complement and coagulation cascades
calcium ion binding|calcium-dependent phospholipid binding|phosphatidylinositol
nucleic acid binding|nucleus|cytosol|protein methyltransferase activity|S-adenc
plasma membrane|regulation of cell shape
g protein A4
nucleus|protein kinase binding|protein serine/threonine kinase inhibitor activi

nase 1 ko00910,kcNitrogen metabolism|D-Glutamine and D-glutamate metabolism|
protein polyubiquitination|in utero embryonic development|RNA binding|ubiquitir
l
nucleus|cytosol|regulation of translation|catecholamine metabolic process|F-act
DNA-binding transcription factor activity, RNA polymerase II-specific|DNA bindi
extracellko01200,kcCarbon metabolism|Glycolysis / Gluconeogenesis
mitochondrko00190 Oxidative phosphorylation
neuron migration|growth plate cartilage chondrocyte morphogenesis|extracellular

ptor	ko04060,kcCytokine-cytokine receptor interaction|Jak-STAT signaling p
GTPase activity|GTP binding|nucleus|cytoplasm|centrosome|cytosol
RNA binding|integrin binding|calcium ion binding|calcium-dependent phospholipic
maturatioko03008	Ribosome biogenesis in eukaryotes
mitochondrko00920	Sulfur metabolism
A, cGMP-inlko00230,kcPurine metabolism|cAMP signaling pathway|cGMP-PKG signaling
1 6
-)-glycoprko00510	N-Glycan biosynthesis
kinetochore|condensed chromosome kinetochore|ameboidal-type cell migration|nucl
nucleus|cytoplasm

plasma membrane|integral component of membrane
mRNA splicko03020,kcRNA polymerase|Purine metabolism|Pyrimidine metabolism
mitochondrion|cytosol|protein ubiquitination|intracellular signal transduction|
ndrome 1
epithelial cell morphogenesis|cytoplasm|centrosome|cytosol|cilium|membrane|smal

chromosomeko03015,kcmRNA surveillance pathway|Hippo signaling pathway|Tight jun

in 35
MAPK cascako04621,kcNOD-like receptor signaling pathway|Neurotrophin signaling
Golgi membko04142	Lysosome
one S-tranko00982,kcDrug metabolism - cytochrome P450|Glutathione metabolism|Me
NA 1
adaptive immune response|nucleus|integral component of membrane|cell junction|T
endoplasmiko00062	Fatty acid elongation
GTPase activity|GTP binding|mitochondrion|mitochondrial outer membrane|early en
integral component of membrane
rotein	ko04722,kcNeurotrophin signaling pathway|Apoptosis - multiple species
growth plako04974	Protein digestion and absorption
endoplasmiko04391	Hippo signaling pathway - fly
ypeptide	ko04978	Mineral absorption
3-chloroalko00561,kcGlycerolipid metabolism|Fatty acid degradation|Tryptophan m
nucleoplasm
growth plako04974,kcProtein digestion and absorption|PI3K-Akt signaling pathway
RNA-directed DNA polymerase activity|DNA recombination|type II site-specific de
nucleus|centrosome|centriole|motile cilium|centriolar satellite|microtubule anc
osteoblast differentiation|cytoplasm|endoplasmic reticulum|endoplasmic reticulu
extracellular matrix structural constituent|extracellular region|extracellular
negative regulation of transcription by RNA polymerase II|DNA-binding transcrip
1-phosphatko00562,kcInositol phosphate metabolism|Endocytosis|MAPK signaling pa
spindle pole|nucleus|cytoplasm|microtubule|chromosome segregation|regulation of

activatioko04024,kccAMP signaling pathway|Regulation of lipolysis in adipocyte

type 2	ko04145	Phagosome
protein depalmitoylation|plasma membrane|palmitoyl-(protein) hydrolase activity
alactosamiko00604	Glycosphingolipid biosynthesis - ganglio series
NAD+ kinasko00760	Nicotinate and nicotinamide metabolism
negative regulation of transcription by RNA polymerase II|RNA polymerase II pro
	ko04974	Protein digestion and absorption

RNA binding
extracellular region|cytoplasm|plasma membrane|inflammatory response|integral c
nucleus|cytoplasm|regulation of transcription, DNA-templated
nuclear chromatin|DNA-binding transcription factor activity, RNA polymerase II-
integral component of membrane
endoplasmic reticulum membrane|integral component of membrane|protein ubiquitir

adenosine ko00230 Purine metabolism
calcium ion binding|extracellular space|plasma membrane|cell surface|extracellu
mRNA splicing, via spliceosome|P-body|transcription coactivator activity|RNA bi
extracellular region|nuclear envelope|endoplasmic reticulum|membrane|secretory
negative regulation of transcription by RNA polymerase II|RNA polymerase II prc
negative regulation of transcription by RNA polymerase II|RNA polymerase II prc
negative regulation of transcription by RNA polymerase II|RNA polymerase II prc
cytoplasm|synaptic vesicle|cell junction|synaptic vesicle membrane|identical pr
skeletal mko04666,kcFc gamma R-mediated phagocytosis|Axon guidance|Regulation o
nuclear inner membrane|integral component of membrane|fat cell differentiation|
negative regulation of transcription by RNA polymerase II|RNA polymerase II reg

extracellular space|phosphoric diester hydrolase activity|zinc ion binding|nucl
menting de:ko03450 Non-homologous end-joining
Hrd1p ubicko04141 Protein processing in endoplasmic reticulum
rfamily, member 8
G2/M transition of mitotic cell cycle|neuron migration|centrosome|centriole|cyt
inger 2 ko04520,kcAdherens junction|Hippo signaling pathway

spindle pole|cytoplasm|microtubule
G protein-ko04080 Neuroactive ligand-receptor interaction
negative rko03460,kcFanconi anemia pathway|Notch signaling pathway
histone deko04919 Thyroid hormone signaling pathway
magnesium ko00520 Amino sugar and nucleotide sugar metabolism
DNA-binding transcription factor activity, RNA polymerase II-specific|DNA bindi
Golgi membrane|protein kinase C binding|structural molecule activity|Golgi appa
metallocarboxypeptidase activity|extracellular space|proteolysis|zinc ion bindi
structural molecule activity|iron ion binding|mitochondrion|mitochondrial matri

nucleoplasm

or receptorko04060,kcCytokine-cytokine receptor interaction|Adipocytokine signal
type IIA ko04060,kcCytokine-cytokine receptor interaction|Signaling pathways r
RNA binding|nucleus|negative regulation of transcription, DNA-templated
cilium movement|axoneme|motile cilium|axoneme assembly|motile cilium assembly|c
nucleoplasm
cytosol|membrane|protein homodimerization activity|extracellular exosome
protein deneddylation|nucleotide-excision repair, DNA damage recognition|nucleu
microtubule
r 3 ko04550 Signaling pathways regulating pluripotency of stem cells
eading frame 27B
kinetochore|condensed chromosome kinetochore|DNA-binding transcription factor a
ndrome 3
autophagosome membrane|nucleus|cytoplasm|autophagosome|cytosol|cytoskeleton|mem

amyloid-bєko04360   Axon guidance
intracellկko04974,kcProtein digestion and absorption|PI3K-Akt signaling pathway

lgalactosaıko00512   Mucin type O-Glycan biosynthesis
cell|integral component of membrane|cell redox homeostasis|oxidation-reduction
nucleus|mitochondrion|cytosol
protein serine/threonine kinase activity|ATP binding|cytoplasm|plasma membrane|
endoplasmic reticulum membrane|disulfide oxidoreductase activity|integral compc
nuclear envelope|annulate lamellae|nuclear pore|cytoplasm|Golgi apparatus|centr
angiogenesko04360   Axon guidance
maturatiorko03010   Ribosome
phospholipid binding|intracellular protein transport|exocytosis|membrane|Rab GT
chromatin binding|nucleus|nucleoplasm|cytoplasm|autophagy|mitotic chromosome cc
integral component of membrane
ATP binding|cytoplasm|microtubule organizing center|cytosol|I-kappaB kinase/NF-
1   ko04620,kcToll-like receptor signaling pathway|HIF-1 signaling pathwa
sponsive 1
magnesium ko00020,kcCitrate cycle (TCA cycle)|Biosynthesis of amino acids|2-Oxo
G protein-coupled receptor activity|cell surface receptor signaling pathway|G p
otion factor 2
olase (con.ko00790   Folate biosynthesis
peroxisome|zinc ion binding|13-prostaglandin reductase activity|negative regula
serine-type endopeptidase activity|Golgi apparatus|cytosol|cytoskeleton|serine-

mitotic cytokinesis|phosphatidylinositol-4,5-bisphosphate binding|endocytosis|e
demethylase 5A
yndrome of ko00562,kcInositol phosphate metabolism|Phosphatidylinositol signalin
acid (GABA)ko04726,kcSerotonergic synapse|GABAergic synapse|Retrograde endocanna
activatiorko04662   B cell receptor signaling pathway
ribokinaseko00030   Pentose phosphate pathway
stress fibko04360   Axon guidance
zinc ion binding|oxidoreductase activity
integral component of membrane
trans-Golgi network|intracellular protein transport|cytoplasmic vesicle|vesicle
response to reactive oxygen species|luteinization|nucleus|endosome|Golgi appara
plasma menko04974,kcProtein digestion and absorption|Thyroid hormone signaling
Golgi apparatus|cytosol|COPI-coated vesicle|protein localization to Golgi appar
translation elongation factor activity
ubiquitin ko04120,kcUbiquitin mediated proteolysis|MAPK signaling pathway - yea
proximal promoter DNA-binding transcription repressor activity, RNA polymerase
negative regulation of transcription by RNA polymerase II|RNA polymerase II tra
g 9
DNA bindirko03022   Basal transcription factors
Golgi membko00532   Glycosaminoglycan biosynthesis - chondroitin sulfate / derm
positive regulation of cell-matrix adhesion|cytosol|cytoskeleton|focal adhesion
RNA polymerase II regulatory region sequence-specific DNA binding|DNA-binding t
NA 1
glutathiorko00982,kcDrug metabolism - cytochrome P450|Glutathione metabolism|Me
vasculogenesis|in utero embryonic development|motor activity|actin binding|ATP
Golgi apparatus|membrane|integral component of membrane|transferase activity, t
7 (pure and complicated autosomal recessive)

activatiorko04010 MAPK signaling pathway
se domain 12
dCMP deamiko00240 Pyrimidine metabolism
l, regulator
Golgi membrane|zinc ion transmembrane transporter activity|Golgi apparatus|inte
nucleus|nascent polypeptide-associated complex|protein transport
GTPase activity|GTP binding|nucleoplasm|mitochondrion|cytosol
nucleus|inflammatory response|nuclear speck|cytoplasmic ribonucleoprotein granu
4
nucleolus|apoptotic process|regulation of protein stability|negative regulatior
plasma membrane|nervous system development|integral component of membrane|trans
Golgi membrane|structural molecule activity|endoplasmic reticulum|endoplasmic r
nucleic acid binding|calmodulin binding|cytoplasm|cellular response to heat
neural tukko04390,kcHippo signaling pathway|Hippo signaling pathway - fly
ARF guanylko04072,kcPhospholipase D signaling pathway|Endocytosis
oocyte matko03018,kcRNA degradation|mRNA surveillance pathway|RNA transport
endoplasmic reticulum|integral component of membrane
ortex 1 (with BTB domain)
G protein-coupled receptor binding|hormone activity|extracellular region|G prot
is, neuronal 8
RNA binding|nucleus|nucleolus|ribosomal small subunit biogenesis|hair follicle
endoplasmic reticulum membrane|unsaturated fatty acid biosynthetic process|memb
factor biiko04115 p53 signaling pathway
storage vako04810 Regulation of actin cytoskeleton
alpha-L-fuko00511 Other glycan degradation
condensin complex|inner cell mass cell proliferation|nucleoplasm|transcription
DNA-binding transcription factor activity, RNA polymerase II-specific|DNA bindi
peptide alpha-N-acetyltransferase activity|nucleus|cytoplasm|cytosol|N-terminal
G2/M transition of mitotic cell cycle|centrosome|centriole|cytosol|cilium|regul
ubiquitin-protein transferase activity|death receptor binding|nucleus|nucleopla
RNA polymerase II proximal promoter sequence-specific DNA binding|proximal prom
negative regulation of transcription by RNA polymerase II|nuclear chromatin|DNA
RNA-directed DNA polymerase activity|DNA recombination|type II site-specific de
virus receko04514,kcCell adhesion molecules (CAMs)|Adherens junction
extracellular matrix structural constituent|calcium ion binding|extracellular r
MAPK cascako04013 MAPK signaling pathway - fly
neuron migko04640,kcHematopoietic cell lineage|Regulation of actin cytoskeleton
RNA binding|actin binding|structural molecule activity|calmodulin binding|intra
DNA-binding transcription factor activity, RNA polymerase II-specific|DNA bindi
Golgi memko04141,kcProtein processing in endoplasmic reticulum|N-Glycan biosyn
DNA binding|nucleus|metal ion binding
negative regulation of transcription by RNA polymerase II|telomere maintenance|
monovalent cation:proton antiporter activity|mitochondrial inner membrane|plasm
-protein coding RNA 1013
ruffle|phcko04015,kcRap1 signaling pathway|Focal adhesion|Platelet activation
DNA-binding transcription factor activity, RNA polymerase II-specific|nucleus|a
Golgi membrane|DNA binding|RNA binding|ATP binding|endoplasmic reticulum-Golgi

sor kinase ko04392,kcHippo signaling pathway -multiple species|Apoptosis - fly|H
metalloendopeptidase activity|integrin binding|extracellular region|extracellul
copper ion transmembrane transporter activity|late endosome|integral component

phosphoglu ko00500   Starch and sucrose metabolism
acyl-CoA ligase activity|ATP binding|mitochondrial matrix|fatty acid metabolic
spliceoson ko03013   RNA transport

RNA polymerase II proximal promoter sequence-specific DNA binding|DNA-binding t
negative regulation of transcription by RNA polymerase II|mitotic cell cycle|DN
e, sub-fam ko02010   ABC transporters
GMP reduct ko00230   Purine metabolism
RNA polymerase II regulatory region sequence-specific DNA binding|DNA-binding t
endoplasmic reticulum membrane|rough endoplasmic reticulum|cytosol|endoplasmic
RNA binding|nucleus|nucleoplasm|membrane
DNA-binding transcription factor activity, RNA polymerase II-specific|DNA bindi
) interacting protein
cytoplasm|positive regulation of peroxisome proliferator activated receptor sig
magnesium ion binding|phospholipid-translocating ATPase activity|ATP binding|nu
glutathion ko00982,kc Drug metabolism - cytochrome P450|Glutathione metabolism|Me
fibrillar center|p53 binding|protein serine/threonine kinase activity|ATP bindi
carbonate ko00910   Nitrogen metabolism
factor, beta receptor associated protein 1
cell|D-ribulokinase activity|pentose metabolic process|carbohydrate phosphoryla
integral component of membrane
RNA polymerase II repressing transcription factor binding|centrosome|cytosol|ci
cytosol|membrane|protein homodimerization activity|extracellular exosome
mitotic cy ko04144   Endocytosis
magnesium ko00230,kc Purine metabolism|RNA degradation
RNA binding|mRNA binding|nucleus|spliceosomal complex|cytoplasm|mRNA processing
ubiquitin- ko04120   Ubiquitin mediated proteolysis
rostacycli ko00590   Arachidonic acid metabolism
chloride channel activity|centrosome|microtubule organizing center|intermediate
telomere m ko03460,kc Fanconi anemia pathway|mTOR signaling pathway
integral component of membrane
cytoplasm|cytosol|cytoplasmic ribonucleoprotein granule|perinuclear region of c
RNA polymerase II proximal promoter sequence-specific DNA binding|DNA-binding t
microfibril|metalloendopeptidase activity|metal ion binding|collagen-containing
alpha,alpha-trehalase activity|cell wall|cytosol|carbohydrate metabolic process
suckling b ko00471,kc D-Glutamine and D-glutamate metabolism|Proximal tubule bica
microtubule cytoskeleton organization|cytoplasm|microtubule|microtubule binding
GABA-A rec ko04727,kc GABAergic synapse|Neuroactive ligand-receptor interaction|R
nd activat ko04062,kc Chemokine signaling pathway|Jak-STAT signaling pathway|Oste
DNA-binding transcription factor activity, RNA polymerase II-specific|angiogene
negative regulation of transcription by RNA polymerase II|negative regulation c
transferase)
GTPase act ko04530   Tight junction
endoplasmi ko04141   Protein processing in endoplasmic reticulum
nucleosome|nuclear chromatin|DNA binding|chromatin organization|protein heteroc
CatSper complex|sperm principal piece
ubiquitin ligase complex|ARF guanyl-nucleotide exchange factor activity|ubiquit
actin binding|structural molecule activity|calmodulin binding|cytosol|cytoskele
integral component of plasma membrane|cell surface receptor signaling pathway
SNARE binc ko04721,kc Synaptic vesicle cycle|SNARE interactions in vesicular tran
repeat domain family member C

G1/S trans ko00230,kc Purine metabolism|Pyrimidine metabolism|DNA replication
cytosol|glycogen biosynthetic process|glycogenin glucosyltransferase activity|U
RNA bindir ko00562,kc Inositol phosphate metabolism|Phosphatidylinositol signalin
magnesium ko00020,kc Citrate cycle (TCA cycle)|Propanoate metabolism|Carbon meta
GTP binding|cytoplasm|trans-Golgi network|intracellular protein transport|vesic
Golgi membrane|Golgi apparatus|magnesium ion transmembrane transporter activity
phospholipase A2 activity|lysosomal membrane|plasma membrane|integral component
ubiquitin ko04120 Ubiquitin mediated proteolysis
extracellular matrix structural constituent|collagen binding|extracellular regi
ATP binding|nucleus|cytoplasm|endoplasmic reticulum lumen|response to unfolded
angiogenes ko04068,kc FoxO signaling pathway|Neuroactive ligand-receptor interact
ubiquitin- ko04120 Ubiquitin mediated proteolysis

nucleus
microtubul ko04120 Ubiquitin mediated proteolysis
G protein- ko04390,kc Hippo signaling pathway|Melanogenesis|mTOR signaling pathwa
iron ion transmembrane transporter activity|mitochondrial inner membrane|integr
oribosyltr ko00230,kc Purine metabolism|Drug metabolism - other enzymes
integral component of membrane
cytoplasm|membrane
synthase ko00532 Glycosaminoglycan biosynthesis - chondroitin sulfate / derm
ferase mu 2 (muscle)
angiogenesis|blood vessel development|vasculogenesis|positive regulation of enc
hydroxyacy ko00620 Pyruvate metabolism
ko04610 Complement and coagulation cascades
cilium|enzyme binding|metal ion binding
mitotic cytokinesis|septin ring assembly|GTPase activity|nucleus|nucleoplasm|cy
RNA polymerase II proximal promoter sequence-specific DNA binding|chromatin bir
Golgi memb ko00510,kc N-Glycan biosynthesis|Various types of N-glycan biosynthesi
cytoplasm|early endosome|endosomal vesicle fusion
polarity ko04330,kc Notch signaling pathway|Melanogenesis|mTOR signaling pathwa
kDa) associated protein 1
cytoplasm| ko04150 mTOR signaling pathway
Golgi membrane|cytosol|intra-Golgi vesicle-mediated transport|Rab guanyl-nucled
regulation of alternative mRNA splicing, via spliceosome|RNA binding|nucleus|nu
integral component of membrane
GTPase activity|calmodulin binding|GTP binding|intracellular|plasma membrane|si
Golgi membrane|GDP-dissociation inhibitor activity|endoplasmic reticulum membra
platelet degranulation|protein disulfide isomerase activity|extracellular regic
ase 4 (Dombrock blood group)
magnesium ko00052,kc Galactose metabolism|Purine metabolism|Amino sugar and nucl
nucleus|protein homodimerization activity|transcription regulatory region DNA b
DNA-binding transcription factor activity|calcium-dependent cysteine-type endop
ATPase activator activity|nucleus|nuclear inner membrane|integral component of

in 1
stress fiber|cytosol|plasma membrane|cell-cell junction|bicellular tight juncti
glucose transmembrane transporter activity|sodium ion transport|symporter activ
DNA-binding transcription factor activity, RNA polymerase II-specific|DNA bindi
regulatior ko04924,kc Renin secretion|Gap junction|Glucagon signaling pathway|Rap
magnesium ion binding|protein tyrosine phosphatase activity|nucleus|nucleoplasm

SUMO polymer binding
RNA binding|mRNA 3'-UTR binding|nucleus|cytoplasm|cytosol|translation|anatomica
mitochondrko04141,kcProtein processing in endoplasmic reticulum|N-Glycan biosyn
G protein-ko04080 Neuroactive ligand-receptor interaction
calcium icko04144 Endocytosis
ıctivator 4
cysteine-type endopeptidase inhibitor activity|negative regulation of tumor nec
voltage-gated anion channel activity|protein transport|integral component of me
P-body|RNA binding|nucleus|cytoplasm|regulation of translation|multicellular or
MAPK cascako04014,kcRas signaling pathway|MAPK signaling pathway
nucleoplasm|mitochondrion|mitochondrial outer membrane|plasma membrane|phosphat
ribosomal large subunit assembly|ribosomal large subunit export from nucleus|er
histone deko04013,kcMAPK signaling pathway - fly|Wnt signaling pathway
-like hormone
phospholipid transporter activity|endoplasmic reticulum membrane|plasma membrar
single-stıko00230,kcPurine metabolism|Pyrimidine metabolism|RNA polymerase|Cyto
ko04142,kcLysosome|Renin secretion|Antigen processing and presentatio
positive regulation of receptor recycling|early endosome|clathrin-coated pit|ph

cytoplasm|zinc ion binding|synapse
NAD+ kinasko00564,kcGlycerophospholipid metabolism|Phospholipase D signaling pa
lomain protein 2
transcriptko03040,kcSpliceosome|RNA transport
neural creko04360 Axon guidance
formation ko03013 RNA transport
autophagosko04211 Longevity regulating pathway
ko04514,kcCell adhesion molecules (CAMs)|ECM-receptor interaction
mRNA splicing, via spliceosome|RNA binding|ATP binding|ATP-dependent 3'-5' RNA
transcription coactivator activity|nucleus|nucleoplasm|cytoplasm|cytosol|plasma
ected), deko03030,kcDNA replication|Mismatch repair|Homologous recombination|Nu
nuclear chromatin|RNA polymerase II proximal promoter sequence-specific DNA bir
cytoplasm|inflammatory response|metalloendopeptidase inhibitor activity|heparir
endopolyphosphatase activity|nucleus|cytoplasm|cytosol|diphosphoinositol-polyph
extracellular space|endosome|Golgi apparatus|sarcoplasmic reticulum|polysacchar
protein disulfide isomerase activity|extracellular space|nucleoplasm|Golgi appa
Golgi apparatus|tubulin binding|growth cone|regulation of microtubule polymeriz
f sperm tails 3B
ssociated ko04916,kcMelanogenesis|Adherens junction|Leukocyte transendothelial
apoptotic process|tetracycline transmembrane transporter activity|organic anior
nucleosome|nuclear chromatin|DNA binding|chromatin organization|protein heteroc

cytosol|membrane|protein homodimerization activity|extracellular exosome
plasma membrane|integral component of membrane|azurophil granule membrane|ident
RNA polymerase II proximal promoter sequence-specific DNA binding|DNA-binding t
nucleus|apoptotic process|smoothened signaling pathway|integral component of me
microtubule cytoskeleton organization|neuron migration|positive regulation of r
cellular gko04066,kcHIF-1 signaling pathway|Insulin signaling pathway|Carbohydr
ko04810,kcRegulation of actin cytoskeleton|PI3K-Akt signaling pathway
glycosaminoglycan binding|extracellular region|extracellular space|heparin binc
endoplasmic reticulum|plasma membrane|sensory perception of sound|integral comp
negative regulation of transcription by RNA polymerase II|RNA polymerase II prc

GTP binding|intracellular
endoplasmic reticulum|endoplasmic reticulum membrane|cholesterol metabolic proc
cytosol|ubiquitin-dependent protein catabolic process|protein ubiquitination|ar
integral component of plasma membrane|monocarboxylic acid transmembrane transpc
ESCRT III ko04144 Endocytosis
positive r ko04390,kc Hippo signaling pathway|Melanogenesis|mTOR signaling pathwa
negative regulation of transcription by RNA polymerase II|RNA polymerase II reg
ko04390,kc Hippo signaling pathway|Wnt signaling pathway|Signaling pat

ko04066,kc HIF-1 signaling pathway|Melanogenesis|TNF signaling pathway
GTPase activity|GTP binding|intracellular|Golgi apparatus|plasma membrane|intra
r 1 ko04920,kc Adipocytokine signaling pathway|Longevity regulating pathwa
-protein coding RNA 205
intracellular|intracellular protein transport|signal transduction|protein kinas
condensed nuclear chromosome|GTPase activator activity|nucleus|nucleolus|cytopl
lipopolysaccharide binding|regulation of cytokine production involved in immune
frizzled b ko04350,kc TGF-beta signaling pathway|Wnt signaling pathway
MAPK casca ko04010 MAPK signaling pathway
calmodulin binding|phosphatidylinositol-3,4,5-trisphosphate binding|nucleus|sig
induced TNF factor
containing 144B (pseudogene)
osyltransfe ko00534 Glycosaminoglycan biosynthesis - heparan sulfate / heparin
ornithine- ko00330 Arginine and proline metabolism
autophagos ko04140,kc Regulation of autophagy|Longevity regulating pathway|RIG-I-
protein serine/threonine kinase activity|protein serine/threonine/tyrosine kina
endoplasmic reticulum membrane|response to unfolded protein|integral component
RNA binding|nucleus|cytoplasm|DNA replication|nucleosome assembly|positive regu
calcium channel activity|plasma membrane|integral component of plasma membrane|
nucleus|nucleoplasm|chromosome|focal adhesion|double-strand break repair via nc
GTP bindir ko04144 Endocytosis
ative tumor suppressor 1
methyltrai ko00310 Lysine degradation
reading frame 80
voltage-ga ko04010,kc MAPK signaling pathway|Adrenergic signaling in cardiomyocyt
nuclear-tr ko03010 Ribosome
ATP bindir ko00562,kc Inositol phosphate metabolism|Phosphatidylinositol signalin
NAD+ kinas ko00564,kc Glycerophospholipid metabolism|Phospholipase D signaling pa
negative regulation of transcription by RNA polymerase II|nuclear chromatin|DNA
carbohydrate binding
prostaglar ko00590 Arachidonic acid metabolism
mitochondrion|mitochondrial inner membrane|glycine metabolic process|folic acic
l ko04514 Cell adhesion molecules (CAMs)
skeletal system development|growth plate cartilage chondrocyte morphogenesis|ex
Golgi memb ko00534 Glycosaminoglycan biosynthesis - heparan sulfate / heparin
ko04110,kc Cell cycle|Hippo signaling pathway|Hedgehog signaling pathw
nucleoplasm|cytosol

lgalactosa ko00512 Mucin type O-Glycan biosynthesis
DNA-binding transcription factor activity, RNA polymerase II-specific|negative
ko04360 Axon guidance
nuclear ex ko03018 RNA degradation

amyloid-beta binding|trans-Golgi network|regulation of proteolysis|positive reg
inactivati ko04010 MAPK signaling pathway
ureteric bud development|blood vessel endothelial cell proliferation involved i
nucleoplas ko00280,ko Valine, leucine and isoleucine degradation|Synthesis and de
nucleus|dendrite|perikaryon
protein se ko04910 Insulin signaling pathway

ted protein 2
GTPase activator activity|cytoplasm|plasma membrane|exocytosis|protein transpor
25C ko04914,ko Progesterone-mediated oocyte maturation|Oocyte meiosis|Cell
protein serine/threonine kinase activity|ATP binding|intracellular|peptidyl-ser
nucleus|cy ko04141 Protein processing in endoplasmic reticulum
ko04920,ko Adipocytokine signaling pathway|cAMP signaling pathway|Regu
basement m ko04360 Axon guidance
xylulokina ko00040 Pentose and glucuronate interconversions
microtubule motor activity|ATP binding|intracellular|kinesin complex|microtubul
mitotic si ko04113,ko Meiosis - yeast|Cell cycle - yeast|Oocyte meiosis|Cell cycl
mitochondr ko01200,ko Carbon metabolism|Oxidative phosphorylation|Citrate cycle (
Golgi membrane|integral component of Golgi membrane|integral component of endop
nucleus|cytosol|BMP signaling pathway
fibrillar ko03040 Spliceosome
cytosol|sh ko00564 Glycerophospholipid metabolism
member B

nucleoplasm|proteolysis|dipeptidase activity|regulation of cellular protein met

cytoplasm| ko04391,ko Hippo signaling pathway - fly|Longevity regulating pathway
focal adhesion|heterophilic cell-cell adhesion via plasma membrane cell adhesio
an 4 felin ko04060,ko Cytokine-cytokine receptor interaction|Endocytosis|Melanoge
lysosome|lysosomal membrane|late endosome|integral component of membrane|late e

alpha-keto ko00640,ko Propanoate metabolism|Valine, leucine and isoleucine degrad
guanyl-nuc ko04015 Rap1 signaling pathway
actin bind ko04144 Endocytosis
cytosol|membrane|axon initial segment|post-translational protein modification|i
vasculature development|blood vessel maturation|sprouting angiogenesis|endopept
lactoylglu ko00620 Pyruvate metabolism
nucleus|cytosol|kinase binding|negative regulation of glucokinase activity|nega
extracellu ko04060,ko Cytokine-cytokine receptor interaction|Chemokine signaling
mitochondrial inner membrane|response to high light intensity|retina layer form
eptor 2 (s ko04080,ko Neuroactive ligand-receptor interaction|cAMP signaling path
plasma membrane
synthase ko00532 Glycosaminoglycan biosynthesis - chondroitin sulfate / derm
cytoplasm
RNA binding|intracellular|protein ubiquitination|transferase activity|metal ion
nucleus|cytosol
7 ko04113,ko Meiosis - yeast|Cell cycle - yeast|Cell cycle
protein phosphatase type 2A complex|phosphoprotein phosphatase activity|protein
nucleosome|nuclear chromatin|DNA binding|nucleus|nucleoplasm|chromatin organiza
RNA-directed DNA polymerase activity|DNA recombination|type II site-specific de
nucleotide binding|single-stranded DNA binding|single-stranded RNA binding|nucl

integral component of membrane
spliceosomko03040   Spliceosome
protein polyubiquitination|cytoplasm|ubiquitin-dependent protein catabolic proc
angiogenesko03320   PPAR signaling pathway
mRNA splicko03015,kcmRNA surveillance pathway|Tight junction
NAD+ kinasko00564,kcGlycerophospholipid metabolism|Phospholipase D signaling pa
chondrocyte differentiation|signaling receptor binding|extracellular space|sigr
oligosacchko04141,kcProtein processing in endoplasmic reticulum|N-Glycan biosyn
ventricular septum development|nucleus|membrane|neural fold bending|artery deve
negative rko03018   RNA degradation
tRNA wobble uridine modification|GTPase activity|GTP binding|nucleus|cytoplasm|
DNA-binding transcription factor activity, RNA polymerase II-specific|DNA bindi
ATP binding|cytoplasm|microtubule organizing center|cytosol|I-kappaB kinase/NF-
RNA polymerase II regulatory region sequence-specific DNA binding|DNA-binding t
ator-activko04380,kcOsteoclast differentiation|Longevity regulating pathway|AMP
low-density lipoprotein particle receptor activity|clathrin-coated pit|lipid me
integral component of plasma membrane|intracellular protein transport|postsynap
regulation of cyclin-dependent protein serine/threonine kinase activity|integra
Golgi membko04727,kcGABAergic synapse|Vasopressin-regulated water reabsorption|
cytoplasm|protein dephosphorylation|actin cytoskeleton|negative regulation of t
nucleosome|DNA binding|nucleus|nucleoplasm|cytosol|nucleosome assembly|protein
euron 2, cko03013   RNA transport
transcription by RNA polymerase II|mRNA processing|membrane|protein domain spec
intracellular|plasma membrane|extracellular exosome
DNA-bindirko04011,kcMAPK signaling pathway - yeast|Hippo signaling pathway -mul
negative regulation of transcription by RNA polymerase II|RNA polymerase II pro
RNA binding|nucleus|nucleoplasm|mRNA processing|SH3 domain binding|protein doma
nucleus|cytosol|plasma membrane|focal adhesion|cell junction assembly|negative
nuclear-transcribed mRNA poly(A) tail shortening|P-body|fibrillar center|RNA bi
g, subfamily A, member 3
negative rko04150   mTOR signaling pathway
exocyst|exko04014   Ras signaling pathway

G protein-ko04340   Hedgehog signaling pathway
nucleic acko03040,kcSpliceosome|mRNA surveillance pathway|RNA transport
extracellko04910,kcInsulin signaling pathway|Glucagon signaling pathway|Starch
ossificatiko04722,kcNeurotrophin signaling pathway|Lysosome
se (glutamko00250   Alanine, aspartate and glutamate metabolism
extracellko01200,kcCarbon metabolism|Glycolysis / Gluconeogenesis
liver deveko00790   Folate biosynthesis
negative regulation of transcription by RNA polymerase II|RNA polymerase II reg
GTPase activity|GTP binding|cytoplasm|I-kappaB kinase/NF-kappaB signaling|membr
ribosomal ko03010   Ribosome
RNA polymerase II core promoter sequence-specific DNA binding|DNA-binding trans
nucleus|cyko03018   RNA degradation
ed lung adenocarcinoma transcript 1 (non-protein coding)
plementatiko03460   Fanconi anemia pathway
mitochondrial fission|dynamin family protein polymerization involved in mitocho

neuron migration|G protein-coupled receptor activity|calcium ion binding|intrac
structural constituent of eye lens|extracellular region|nucleus|cytoplasm|regul

phosphoryl ko04910,kc Insulin signaling pathway|Calcium signaling pathway|Glucago
histone deacetylase complex|negative regulation of transcription by RNA polymer
nucleus|nucleoplasm|cytoplasm|cytosol|cilium|ciliary basal body|positive regula
l, non-rec ko04722,kc Neurotrophin signaling pathway|Axon guidance|Cell cycle|Erb
extracellular space|plasma membrane|integral component of membrane|cytoplasmic
domains 2 ko04380 Osteoclast differentiation
RNA polymerase II proximal promoter sequence-specific DNA binding|chromatin bir
G2/M transition of mitotic cell cycle|cytosol|regulation of mitotic nuclear div
tRNA binding|aminoacyl-tRNA editing activity|DNA binding|nucleolus|cytoplasm|cy

integral component of membrane|transmembrane transport
nuclear-tr ko03018 RNA degradation
negative regulation of transcription by RNA polymerase II|negative regulation c
ribosome
in utero embryonic development|double-stranded RNA binding|structural constitue
negative regulation of transcription by RNA polymerase II|RNA polymerase II prc

protein serine/threonine kinase activity|guanyl-nucleotide exchange factor acti
213
ko04144 Endocytosis
complement component C1q binding|calcium ion binding|plasma membrane|phagocytos
autophagosome assembly|phagophore assembly site|autophagosome membrane|autophag
cytosol|protein ubiquitination|intracellular signal transduction|post-translati
cytosol|pl ko04961,kc Endocrine and other factor-regulated calcium reabsorption|E
DNA binding|nucleus|metal ion binding
nucleic ac ko00970 Aminoacyl-tRNA biosynthesis
ember 30
tyl) trans ko00512 Mucin type O-Glycan biosynthesis
plasma membrane|single fertilization|sodium:proton antiporter activity|integral
virus CT1(ko04062,kc Chemokine signaling pathway|Neurotrophin signaling pathway|
spindle pole|nucleus|nucleoplasm|cytoplasm|Golgi apparatus|spindle|cytosol|plas
cytoplasm|cytosol|actin filament organization|aging|protein C-terminus binding|

positive r ko04721 Synaptic vesicle cycle
microtubule bundle formation|cytoplasm|centrosome|microtubule depolymerization|
Golgi memb ko00511,kc Other glycan degradation|Sphingolipid metabolism
ruffle|cyt ko04924,kc Renin secretion|Gap junction|Glucagon signaling pathway|Rap
mitochondrion|succinate-hydroxymethylglutarate CoA-transferase activity
magnesium ion binding|actin binding|protein serine/threonine kinase activity|AT
intermediate filament
intermediate filament
cytoplasm| ko04330 Notch signaling pathway
protein serine/threonine kinase activity|protein serine/threonine/tyrosine kina
protein ty ko04514,kc Cell adhesion molecules (CAMs)|Adherens junction|Insulin si
voltage-ga ko04010,kc MAPK signaling pathway|Adrenergic signaling in cardiomyocyt
lipid transporter activity|endoplasmic reticulum membrane|carbohydrate transpor
se 1
metalloendopeptidase activity|cytoplasm|lipid droplet|cytosol|focal adhesion|ce
protein se ko04270 Vascular smooth muscle contraction
negative regulation of transcription by RNA polymerase II|DNA-binding transcrip
DNA bindir ko04530 Tight junction

transcription, DNA-templated|negative regulation of phosphatase activity
inding RAS-like 3
extracellular region|peptidase activity|intracellular membrane-bounded organell
RNA binding|cytoplasm
intracellular|protein ubiquitination|intracellular signal transduction
integral component of membrane
synaptonemal complex|lateral element|DNA binding|nucleus|synaptonemal complex a
Ser-tRNA(Ala) hydrolase activity|nucleic acid binding|alanine-tRNA ligase activ
ESCRT I complex|endosome membrane|protein transport|endosomal transport|macroau
translatiko03015 mRNA surveillance pathway
ATP binding|cytoplasm|microtubule organizing center|microtubule|cell cycle|prot
integral component of plasma membrane|cell surface|negative regulation of cell
nucleic acid binding|metal ion binding
nucleus|nucleoplasm|U7 snRNP|telomerase holoenzyme complex|termination of RNA p
protein serine/threonine kinase activity|ATP binding|intracellular|peptidyl-ser
aminopeptidase activity|cytoplasm|plasma membrane|integral component of plasma
RNA binding|nucleus|nucleoplasm|cytosol|nuclear speck
angiogenesis|actin binding|cytoplasm|Golgi apparatus|cytosol|plasma membrane|cy
nucleus|ubiquitin-dependent protein catabolic process|multicellular organism de
plasma membrane|integral component of plasma membrane|sodium-independent organi
ar ribonuc ko03040 Spliceosome
hyaluronic acid binding|extracellular region|extracellular space|inflammatory r
alpha 5 ko04974,kcProtein digestion and absorption|PI3K-Akt signaling pathway
Golgi membrane|plasma membrane|integral component of membrane|structural consti
uracil birko00770,kcPantothenate and CoA biosynthesis|Pyrimidine metabolism|Dru
protease bko04060,kcCytokine-cytokine receptor interaction|Apoptosis|Natural ki
cytosol|membrane|protein homodimerization activity|extracellular exosome
ko04512 ECM-receptor interaction
mitochondrial outer membrane|integral component of membrane
calcium ion binding|membrane
negative regulation of transcription by RNA polymerase II|DNA-binding transcrip
Golgi membrane|intracellular protein transport|intra-Golgi vesicle-mediated tra
nucleus
lGDS/AF-6) ko04014,kcRas signaling pathway|Leukocyte transendothelial migration|
us-like nucleoprotein 3
nucleus|cytoplasm|endoplasmic reticulum|endoplasmic reticulum membrane|integral
actor 13 ko04010,kcMAPK signaling pathway|Rap1 signaling pathway|Regulation of
2 ko04068 FoxO signaling pathway
cell-cell junction|adherens junction|cell adhesion|ruffle membrane|positive reg
cytoplasm|ko04010,kcMAPK signaling pathway|Focal adhesion
glycerol-3ko00564 Glycerophospholipid metabolism
neuron migration|endosome|neuron cell-cell adhesion|locomotory behavior|externa
negative regulation of transcription by RNA polymerase II|alternative mRNA spli

ain 12, myoxin)
antigen biko04015,kcRap1 signaling pathway|T cell receptor signaling pathway|Ce
nucleus|nucleoplasm|positive regulation of lamellipodium assembly|positive regu
negative regulation of transcription by RNA polymerase II|RNA polymerase II reg
magnesium ko00600 Sphingolipid metabolism
se domain 23
platelet degranulation|calcium ion binding|plasma membrane|integral component c

plasma membrane|protein kinase A regulatory subunit binding
osphatase (ko04010 MAPK signaling pathway
amino acicko04727 GABAergic synapse
calcium ion binding|cytoplasm|centrosome|sperm flagellum
calcium ion binding
ko04151,kcPI3K-Akt signaling pathway|ECM-receptor interaction|Focal a
MAPK cascako04214,kcApoptosis - fly|MAPK signaling pathway|TNF signaling pathwa
DNA binding|nucleus|metal ion binding

plasma menko04810,kcRegulation of actin cytoskeleton|PI3K-Akt signaling pathway
osteoblastko04974,kcProtein digestion and absorption|PI3K-Akt signaling pathway
negative regulation of transcription by RNA polymerase II|inactivation of MAPK
mitochondrion|mitochondrial inner membrane|apoptotic process|integral component
RNA-directed DNA polymerase activity|DNA recombination|type II site-specific de
heart morphogenesis|fibroblast growth factor receptor binding|endoplasmic retic
eat contaiko04215,kcApoptosis - multiple species|Apoptosis|TNF signaling pathwa
ko04068,kcFoxO signaling pathway|p53 signaling pathway
integral component of membrane
alternativko03040 Spliceosome
calcium ion binding|extracellular space|cytoplasm|integral component of membrar
angiogeneko04015,kcRap1 signaling pathway|Aldosterone synthesis and secretion
ubiquitin ko04120,kcUbiquitin mediated proteolysis|Protein processing in endopl
nucleus|cytosol|cytoskeleton|membrane|protein kinase binding|ubiquitin protein
spindle pcko03013 RNA transport
integral component of membrane
extracellular space|peptidase inhibitor activity
G protein-coupled receptor signaling pathway|integral component of membrane|car
DNA-binding transcription factor activity, RNA polymerase II-specific|skeletal
Golgi membrane|endoplasmic reticulum|endoplasmic reticulum lumen|endoplasmic re
containing 28A
acrosomal vesicle|protein-lysine 6-oxidase activity|copper ion binding|extracel
complementko04610 Complement and coagulation cascades
integral component of membrane
negative regulation of transcription by RNA polymerase II|DNA-binding transcrip
microtubule cytoskeleton organization|gamma-tubulin complex|microtubule bundle
protein 1 ko00350,kcTyrosine metabolism|Melanogenesis

extracellular space|cytoskeleton|signal transduction
fibrillar center|stress fiber|cytosol|focal adhesion|regulation of cell shape|c
negative rko04916,kcMelanogenesis|Osteoclast differentiation
spliceosonko03013 RNA transport
nucleosome|double-stranded DNA binding|RNA binding|nucleus|nucleolus|nucleosome
serine-typko04610 Complement and coagulation cascades
proteasome complex|protease binding|nucleoplasm|cytosol|plasma membrane|transcr
protein polyubiquitination|ubiquitin-protein transferase activity|cytoplasm|ubi

RNA-directed DNA polymerase activity|DNA recombination|type II site-specific de
nuclear-trko03013 RNA transport
carcinoma 3
containing 8 family, member A
nucleoplasm|cytosol|transcription by RNA polymerase II|transcription elongatior

GTPase activator activity|cytosol|plasma membrane|intracellular protein transpc
mitotic sister chromatid segregation|kinetochore|condensed chromosome kinetochc
dihydrolipko00260,kcGlycine, serine and threonine metabolism|Carbon metabolism|
platelet degranulation|endopeptidase inhibitor activity|serine-type endopeptida
1, regulatory subunit 9B
negative regulation of transcription by RNA polymerase II|activation of innate
extracellıko04622 RIG-I-like receptor signaling pathway
integral component of membrane
nucleoplasm
retinoid nko04514,kcCell adhesion molecules (CAMs)|ECM-receptor interaction
magnesium ko00020,kcCitrate cycle (TCA cycle)|Biosynthesis of amino acids|2-Oxo
hydroxymetko00900,kcTerpenoid backbone biosynthesis|Bile secretion|AMPK signali
regulation of cell growth|neuron migration|serine-type endopeptidase inhibitor
nuclear-tıko03015 mRNA surveillance pathway
ɔeta 1,4- ;ko00533,kcGlycosaminoglycan biosynthesis - keratan sulfate|Other type
mitotic ceko04150 mTOR signaling pathway
integral component of membrane
calcium ion binding|integral component of plasma membrane|cell adhesion|homophi
methionineko01230,kcBiosynthesis of amino acids|Cysteine and methionine metabol

ɔ, sub-fam ko04146,kcPeroxisome|ABC transporters
angiogeneseko04060,kcCytokine-cytokine receptor interaction|Rap1 signaling pathw
negative regulation of transcription by RNA polymerase II|cAMP-dependent protei
nicotinateko00760 Nicotinate and nicotinamide metabolism
ɔtion factcko04925 Aldosterone synthesis and secretion
actin binding|nucleus|cytoplasm|microtubule organizing center|cytosol|plasma me
mitochondıko00280 Valine, leucine and isoleucine degradation
ubiquitin ligase complex|protein polyubiquitination|ubiquitin-protein transfera

structuralko03010 Ribosome
DNA-binding transcription factor activity, RNA polymerase II-specific|DNA bindi
skeletal system development|eye development|osteoblast fate commitment|podosome
ko04080,kcNeuroactive ligand-receptor interaction|Vascular smooth mus
skeletal system development|extracellular matrix structural constituent|extrace
factor, bıko04110,kcCell cycle|Hippo signaling pathway|Osteoclast differentiati
ko04060,kcCytokine-cytokine receptor interaction|Melanogenesis|Rap1 s
exopolyphosphatase activity|cytoplasm|polyphosphate catabolic process|apoptotic
RNA binding|nuclear inner membrane|protein transport|integral component of Golg

positive regulation of cell-matrix adhesion|cell adhesion|nervous system develc
histone acetyltransferase complex|histone H3 acetylation|histone H4-K5 acetylat
nucleus|cytoplasm|endoplasmic reticulum|protein folding in endoplasmic reticulu
ɔiated factor 1
transporter activity|calcium-dependent phospholipid binding|nucleus|cytoplasm|p
ko04330 Notch signaling pathway
deficient·ko04914,kcProgesterone-mediated oocyte maturation|Oocyte meiosis|Cell
mRNA splicko03015,kcmRNA surveillance pathway|RNA transport
scavenger ko04145 Phagosome
cytoplasm|endocytosis|membrane|regulation of endocytosis
L-iduronicko00531,kcGlycosaminoglycan degradation|Lysosome
hyaluronic acid binding|extracellular space|cell adhesion|central nervous syste

Golgi memb ko04650,ko Natural killer cell mediated cytotoxicity|Endocytosis|Phago
tumor necr ko04210,ko Apoptosis|TNF signaling pathway|NF-kappa B signaling pathwa
cilium
autophagos ko04145 Phagosome
RNA binding|protein monoubiquitination|protein ubiquitination|ribosome binding|
intracellu ko01230,ko Biosynthesis of amino acids|Cysteine and methionine metabol
phospholipid binding|phospholipid transporter activity|nuclear outer membrane|C
maternal placenta development|response to bacterium|embryo development ending i
neural cre ko04360 Axon guidance
extracellular region|regulation of transcription, DNA-templated|coreceptor acti
DNA-binding transcription factor activity, RNA polymerase II-specific|DNA bindi
DNA binding|nucleus|metal ion binding
actin binding|cytoplasm|cytoskeleton|phosphatase binding
enzyme inh ko04921,ko Oxytocin signaling pathway|Vascular smooth muscle contracti
nuclear ch ko03015 mRNA surveillance pathway
collagen-containing extracellular matrix
lipopolysa ko04620,ko Toll-like receptor signaling pathway|NF-kappa B signaling p
angiogenesis|hormone activity|extracellular region|intracellular|protein phosph
microtubule cytoskeleton organization|pericentriolar material|mitotic cell cycl
Golgi membrane|GTPase activity|GTP binding|endoplasmic reticulum membrane|plasm
endoplasmic reticulum membrane|lipid droplet|lipid catabolic process|hydrolase
ko04014,ko Ras signaling pathway|PI3K-Akt signaling pathway|Axon guida
DNA-binding transcription factor activity, RNA polymerase II-specific|DNA bindi
RNA polymerase II regulatory region sequence-specific DNA binding|proximal prom
DNA-dependent DNA replication|chromatin remodeling|regulation of transcription
cytoplasm|focal adhesion|zinc ion binding|actin cytoskeleton|actin filament bin
DNA-binding transcription factor activity, RNA polymerase II-specific|DNA bindi
extracellular region|plasma membrane|acetylcholine receptor regulator activity|
plasma membrane repair|phospholipid binding|nuclear envelope|plasma membrane|ca
cytoplasm| ko04261,ko Adrenergic signaling in cardiomyocytes|Cardiac muscle contr
actin binc ko04212,ko Longevity regulating pathway - worm|Endocytosis
skeletal system development|heart valve morphogenesis|fibroblast growth factor-
DNA-binding transcription factor activity, RNA polymerase II-specific|DNA bindi
endoplasmic reticulum membrane|cytosol|plasma membrane|bile acid biosynthetic p
or ko04914,ko Progesterone-mediated oocyte maturation|Oocyte meiosis
iron ion b ko00100 Steroid biosynthesis
MAPK casca ko04010,ko MAPK signaling pathway|Rap1 signaling pathway|Regulation of
cytochrome ko00190,ko Oxidative phosphorylation|Cardiac muscle contraction
DNA bindir ko04623 Cytosolic DNA-sensing pathway
-like grow ko04915,ko Estrogen signaling pathway|GnRH signaling pathway|ErbB sign
nuclear ch ko00310 Lysine degradation
calcium channel regulator activity|integral component of plasma membrane|cell s
cytoplasm
cytoplasm
mRNA splic ko03010 Ribosome
cytosol
zinc ion transmembrane transporter activity|nucleus|nucleolus|Golgi apparatus|i
UDP-glucuronic acid transmembrane transporter activity|endoplasmic reticulum me
ruffle|regulation of receptor internalization|nucleoplasm|cytosol|cell cortex|c
SAGA complex|transcription coactivator activity|nucleus|zinc ion binding|histor
nuclear chromatin|RNA polymerase II activating transcription factor binding|tra

neural cre ko04360 Axon guidance
1-phosphatidylinositol 4-kinase activity|cytoplasm|plasma membrane|membrane|pho
ase RNA 2
GTPase activator activity|early endosome|cytosol|endocytosis|growth factor acti
branching ko04974,kc Protein digestion and absorption|PI3K-Akt signaling pathway
cytoplasmic exosome (RNase complex)|P-body|DNA binding|mRNA 3'-UTR binding|cytc
RNA binding|nucleus|cytoplasm
protein peptidyl-prolyl isomerization|peptidyl-prolyl cis-trans isomerase activ
ide alpha-: ko00510,kc N-Glycan biosynthesis|Other types of O-glycan biosynthesis
guanyl-nucleotide exchange factor activity|cytoplasm|plasma membrane|small GTPa
ing transcription factor
containing 152
nate delta ko00900 Terpenoid backbone biosynthesis
DNA-binding transcription factor activity, RNA polymerase II-specific|DNA bindi
nuclear er ko03013 RNA transport
RNA polymerase II regulatory region sequence-specific DNA binding|DNA-binding t
inward rectifier potassium channel activity|potassium channel activity|sodium c
acid (GABA) ko04723,kc Retrograde endocannabinoid signaling|GABAergic synapse|Neur
extracellular region

ribosomal small subunit assembly|RNA binding|GTP binding|mitochondrion|mitochon
condensed ko03008 Ribosome biogenesis in eukaryotes
mitochondr ko00130 Ubiquinone and other terpenoid-quinone biosynthesis
plasma mem ko04071,kc Sphingolipid signaling pathway|Neuroactive ligand-receptor
histone de ko04391 Hippo signaling pathway - fly

aortic valve morphogenesis|extracellular region|collagen trimer|extracellular s
1-acylglyc ko00564,kc Glycerophospholipid metabolism|Glycerolipid metabolism
structural ko04514,kc Cell adhesion molecules (CAMs)|Leukocyte transendothelial m
lipid droplet|integral component of membrane|cell junction|synaptic vesicle mem
lin-like extracellular matrix protein 1
nucleic acid binding|metal ion binding
intracellular|mitochondrion|plasma membrane|apoptotic process|signal transducti
microtubul ko04728,kc Dopaminergic synapse|Endocytosis
Golgi membrane|antigen processing and presentation of peptide antigen via MHC c
integral component of membrane|intrinsic component of endoplasmic reticulum mem
negative regulation of transcription by RNA polymerase II|RNA polymerase II reg
extracellular space|brain development|postsynaptic density|actin cytoskeleton|p
negative regulation of transcription by RNA polymerase II|DNA-binding transcrip
serine-type endopeptidase inhibitor activity|extracellular matrix structural cc
deoxyribos ko00030 Pentose phosphate pathway
integral component of membrane
y 1, basic leucine zipper transcription factor 2
GTP binding|nucleus|nucleoplasm|nucleolus|cytoplasm|cytosol|plasma membrane|int
guanylate ko04015,kc Rap1 signaling pathway|Tight junction
g region Y)-box 11
microtubul ko04340 Hedgehog signaling pathway
ko00562,kc Inositol phosphate metabolism|Phosphatidylinositol signalin
voltage-gated chloride channel activity|chloride channel activity|ATP binding|e
ko04666 Fc gamma R-mediated phagocytosis
RNA bindir ko04120 Ubiquitin mediated proteolysis

angiogeneskko04060,kcCytokine-cytokine receptor interaction|Endocytosis|Rap1 sig
platelet degranulation|extracellular region|extracellular space|Golgi apparatus
ing 1 ko04392,kcHippo signaling pathway -multiple species|Hippo signaling p
RNA binding|nucleolus|zinc ion binding|negative regulation of phosphatase activ
mitochondrial outer membrane|membrane|integral component of membrane|heme bindi
ko04270 Vascular smooth muscle contraction
tion-related protein 1, 34kDa
lysosomal membrane|heart development|BORC complex
RNA binding|signaling receptor binding|nucleus|cytoplasm|mitochondrion|centroso
ubiquitin-protein transferase activity|proteolysis|SCF ubiquitin ligase complex
Rab GTPase binding|calcium ion regulated exocytosis|cell junction|regulation of
proepicardium cell migration involved in pericardium morphogenesis|fibroblast g
mitochondrial fission|dynamin family protein polymerization involved in mitocho
ko04919,kcThyroid hormone signaling pathway|Notch signaling pathway|D
dopeptidaseko04974,kcProtein digestion and absorption|Hematopoietic cell lineage
negative regulation of transcription by RNA polymerase II|nuclear chromatin|DNA
family 3, ko04080 Neuroactive ligand-receptor interaction
protein pcko04120,kcUbiquitin mediated proteolysis|Protein processing in endopl
RNA-directed DNA polymerase activity|DNA recombination|type II site-specific de
purine nucko04910,kcInsulin signaling pathway|Glucagon signaling pathway|Starch
plasma membrane|membrane|PDZ domain binding|protein-containing complex assembly

mRNA splicing, via spliceosome|RNA binding|helicase activity|ATP binding|nucleu
nucleic acid binding|negative regulation of transcription, DNA-templated
spindle pole|centrosome|centriole|cytosol|cell junction|apical junction assembl
5
cytosol|membrane|protein homodimerization activity|extracellular exosome
gastrulation with mouth forming second|nucleus|cytoplasm|plasma membrane|integr
nucleolus|positive regulation of apoptotic process
extracellular region
5 (intestinal)
Golgi membrane|RNA binding|endoplasmic reticulum membrane|cytosol|intracellular
e 1 ko04144 Endocytosis
transcriptko03013 RNA transport
y member 10 (C-type lectin)
guanyl-nucleotide exchange factor activity|Rho guanyl-nucleotide exchange facto
DNA binding|mRNA binding|helicase activity|endonuclease activity|ATP binding|nu
Golgi membrane|plasma membrane|integral component of membrane|structural consti
extracellular matrix structural constituent|collagen-containing extracellular m
SNARE binding|phosphatidylserine binding|calcium ion binding|calcium-dependent
T cell meclko04650 Natural killer cell mediated cytotoxicity
potassium channel activity|integral component of membrane|nuclear membrane|sarc
cysteine-type endopeptidase activity|thiol-dependent ubiquitin-specific proteas
in 44
plasma membrane|magnesium ion transmembrane transporter activity|integral compo
reading frame 81
extracelluko04514 Cell adhesion molecules (CAMs)
positive regulation of translation|positive regulation of cell size
DNA-binding transcription factor activity, RNA polymerase II-specific|DNA bindi
RNA binding|mRNA binding|nucleus|mRNA processing|membrane|positive regulation o
negative regulation of transcription by RNA polymerase II|DNA-binding transcrip

extracellular region
integral component of membrane
negative regulation of transcription by RNA polymerase II|RNA polymerase II reg
integral component of membrane
RNA bindir ko00450,kc Selenocompound metabolism|Tryptophan metabolism
of antiviral response (non-protein coding)
ko04550 Signaling pathways regulating pluripotency of stem cells
structural ko04514,kc Cell adhesion molecules (CAMs)|Leukocyte transendothelial m

protein polyubiquitination|DNA-binding transcription factor activity, RNA polym
ay inhibit ko04610 Complement and coagulation cascades
DNA-binding transcription factor activity, RNA polymerase II-specific|DNA bindi
actin binding|calmodulin binding|cytoskeleton|focal adhesion|regulation of smoo
signaling ko04960 Aldosterone-regulated sodium reabsorption
DNA-binding transcription factor activity, RNA polymerase II-specific|DNA bindi
ligase, mo ko00480,kc Glutathione metabolism|Cysteine and methionine metabolism
G protein- ko04010,kc MAPK signaling pathway|Focal adhesion
Golgi memb ko04060,kc Cytokine-cytokine receptor interaction|Regulation of actin
inase 3 be ko04722,kc Neurotrophin signaling pathway|Axon guidance|Cell cycle|Hip
L-ornithine transmembrane transporter activity|nitric oxide production involved
extracellular matrix structural constituent|collagen binding|extracellular regi
integral component of membrane
morphogenesis of an epithelium|protein serine/threonine kinase activity|ATP bir
AMP deamir ko00230 Purine metabolism
ferase mu ko00982,kc Drug metabolism - cytochrome P450|Glutathione metabolism|Me
se with thrombospondin type 1 motif, 1
GTPase act ko04062,kc Chemokine signaling pathway|Cholinergic synapse|Serotonergi
amyloid-beta binding|vasculogenesis|G protein-coupled receptor activity|plasma
actin cortical patch assembly|DNA binding|nucleus|actin filament|endocytosis|ac
Ser-tRNA(Ala) hydrolase activity|nucleic acid binding|alanine-tRNA ligase activ
negative regulation of transcription by RNA polymerase II|RNA polymerase II prc
nucleus|cytoplasm|exocytosis|dipeptidase activity|nuclear membrane
iron ion binding|mitochondrion|mitochondrial matrix|C21-steroid hormone biosynt
II (thromb ko04024,kc cAMP signaling pathway|Endocytosis|Platelet activation|Rap1
MAPK casca ko04012 ErbB signaling pathway
ko04670,kc Leukocyte transendothelial migration|Regulation of actin cy
adaptive immune response|plasma membrane|integral component of membrane|regulat
protein kinase inhibitor activity|nucleus|cytoplasm|cytoskeleton|protein phosph
DNA-binding transcription factor activity, RNA polymerase II-specific|RNA polym
PHD fingers family 2
mitotic cell cycle|regulation of cell growth|RNA binding|protein kinase activit
nucleoplasm
GTPase activity|GTP binding|intracellular|intracellular protein transport|Rab p
plasma membrane|voltage-gated potassium channel complex|serine-type peptidase a
DNA-bindir ko04391 Hippo signaling pathway - fly

DNA bindir ko04622 RIG-I-like receptor signaling pathway
transporter activity|exocytosis|integral component of membrane|transmembrane tr
cell morphogenesis|type B pancreatic cell development|DNA binding|double-stranc
protein ubiquitination|metal ion binding|ubiquitin protein ligase activity
cell morphogenesis|response to hypoxia|endoplasmic reticulum membrane|Golgi-ass

dase 3	ko04668	TNF signaling pathway
intracellular|cytoplasm|pyridoxal phosphate binding
DNA-binding transcription factor activity, RNA polymerase II-specific|DNA bindi
prostaglarko04080,kcNeuroactive ligand-receptor interaction|Calcium signaling p
nucleus|nucleosome assembly
negative rko04120,kcUbiquitin mediated proteolysis|Hedgehog signaling pathway -
generatiorko03040	Spliceosome
nucleoplasm
integral component of membrane
in utero embryonic development|vasculature development|G-protein alpha-subunit
ko04662	B cell receptor signaling pathway
scavenger receptor activity|extracellular matrix structural constituent|extrace
microglialko04623	Cytosolic DNA-sensing pathway
family 2, ko04740	Olfactory transduction
r Dp-1	ko04350,kcTGF-beta signaling pathway|Cell cycle
nucleus|nucleolus|cytoplasm|endoplasmic reticulum|ER to Golgi vesicle-mediated
platelet degranulation|extracellular region|cell adhesion|platelet alpha granul
nuclear envelope|nucleoplasm|cytoplasm|zinc ion binding|perinuclear region of c
kinase	ko04971,kcGastric acid secretion|Oxytocin signaling pathway|Focal adh
signal recko03060	Protein export
signaling receptor binding|plasma membrane|cell surface|integral component of m
microtubule associated complex|cytoplasmic microtubule|microtubule binding|micr
fibrillar ko04010,kcMAPK signaling pathway|Apoptosis|TNF signaling pathway|Oste
mRNA splicing, via spliceosome|protein peptidyl-prolyl isomerization|peptidyl-p
cell morphogenesis|RNA polymerase II proximal promoter sequence-specific DNA bi
phosphaticko04727	GABAergic synapse
cytoplasm|plasma membrane|synapse|metal ion binding
endoplasmic reticulum|endoplasmic reticulum membrane|mesoderm development|regul
1 (head to head)
negative regulation of transcription by RNA polymerase II|extracellular space|r
ruffle|extracellular space|nucleolus|cytoplasm|microtubule organizing center|pl
-protein coding RNA 1583
transcriptko04919	Thyroid hormone signaling pathway
DNA-binding transcription factor activity, RNA polymerase II-specific|DNA bindi
cornified envelope|in utero embryonic development|calcium ion binding|extracell
secondary active sulfate transmembrane transporter activity|bicarbonate transme
g 16
somitogeneko04360	Axon guidance

nucleic acid binding|nucleus|nucleoplasm|cytoplasm|cell cycle|zinc ion binding|
e	ko04010,kcMAPK signaling pathway|Osteoclast differentiation
Golgi membrane|nucleoplasm|cytosol|COPII vesicle coating
retinoid metabolic process|Golgi lumen|plasma membrane|glycosaminoglycan biosyn
r 1 (high ako04080,kcNeuroactive ligand-receptor interaction|Calcium signaling p
voltage-gated ion channel activity|cation channel activity|sodium channel activ
translaticko00260,kcGlycine, serine and threonine metabolism|Methane metabolism
G protein-coupled receptor activity|endoplasmic reticulum|plasma membrane|integ
lysosome|endosome|endocytosis|zinc ion binding|integral component of membrane|p
actor (hepako04060,kcCytokine-cytokine receptor interaction|Rap1 signaling pathw
endoplasmic reticulum-Golgi intermediate compartment|plasma membrane|cell adhes

magnesium ion binding|phospholipid-translocating ATPase activity|ATP binding|Gc
nucleoside transmembrane transporter activity|plasma membrane|integral componer
nucleic acko03008 Ribosome biogenesis in eukaryotes
mitotic cell cycle|magnesium ion binding|GTPase activity|calmodulin binding|GTF
T cell mecko04650 Natural killer cell mediated cytotoxicity
magnesium ko04150 mTOR signaling pathway
actin binding|profilin binding|cytoplasm|cytoskeleton|gap junction|actin filame
nucleoplasm
ion channeko04726,kcSerotonergic synapse|Insulin secretion|Bile secretion
alpha-1,3-ko00510,kcN-Glycan biosynthesis|Various types of N-glycan biosynthesi

triose-phcko00710,kcCarbon fixation in photosynthetic organisms|Inositol phosph
N-acetylgako00531,kcGlycosaminoglycan degradation|Lysosome
nucleotideko00830 Retinol metabolism
ATP bindirko04270 Vascular smooth muscle contraction
ko04623,kcCytosolic DNA-sensing pathway|Hematopoietic cell lineage|TN
nucleus|nucleosome assembly|positive regulation of cell proliferation|positive
lgalactosarko00512 Mucin type O-Glycan biosynthesis
angiogeneško04974 Protein digestion and absorption
oligosaccharyltransferase complex|integral component of membrane|protein N-link
Golgi membko04727,kcGABAergic synapse|Vasopressin-regulated water reabsorption|
integral component of membrane|transmembrane transport
beta-glucuko00531 Glycosaminoglycan degradation
magnesium ko04110 Cell cycle
nucleus|cytoplasm|lysosomal membrane|trans-Golgi network|cytosol|axoneme|intrac
epithelial to mesenchymal transition|cytoplasm|plasma membrane|cell-cell adhere
cytoplasm|filamin binding|actin filament bundle|skeletal system morphogenesis|r
Golgi membrane|GTPase activity|GTP binding|cytoplasm|cytosol|protein homodimeri
extracellular space|growth factor activity|positive regulation of fibroblast pr
DNA-binding transcription factor activity, RNA polymerase II-specific|DNA bindi
nuclear chromatin|RNA polymerase II proximal promoter sequence-specific DNA bir

plasma membrane|ion transport|sodium:proton antiporter activity|potassium:protc
MAPK cascako04010 MAPK signaling pathway
Golgi apparatus|microtubule binding|kinase binding
structural constituent of ribosome|mitochondrion|mitochondrial inner membrane|m

thiol-dependent ubiquitin-specific protease activity|calcium ion binding|Golgi
RNA polymerase II proximal promoter sequence-specific DNA binding|DNA-binding t
DNA-binding transcription factor activity, RNA polymerase II-specific|nucleic a
integral cko03040 Spliceosome
voltage-gated potassium channel activity|delayed rectifier potassium channel ac
Golgi apparatus|trans-Golgi network|cytosol|brain development|vesicle-mediated
ubiquitin ligase complex|protein polyubiquitination|ubiquitin-protein transfera
apical corko04390,kcHippo signaling pathway|Hippo signaling pathway -multiple s
nucleus|cytosol|type I interferon signaling pathway
skeletal system development|cartilage condensation|ossification|metalloendopept
osteoblast differentiation|endoplasmic reticulum membrane|plasma membrane|sperm
nsforming 1 interacting protein
dimethylalko00900 Terpenoid backbone biosynthesis

rotein 43
negative rko04110   Cell cycle
1-phosphatidylinositol 4-kinase activity|cytoplasm|plasma membrane|membrane|pho
Rho guanyl-nucleotide exchange factor activity|regulation of Rho protein signal
virus recepko04142,koLysosome|Phagosome
catalytic ko04974   Protein digestion and absorption
UTP:glucosko00040,koPentose and glucuronate interconversions|Amino sugar and nu
TIM23 mitochondrial import inner membrane translocase complex|integral componen
eading frame 58
cytoplasm
ATP binding|nuclear outer membrane|negative regulation of microtubule depolymer
ivating protein
identical protein binding|histone deacetylase binding
intracelluko04060,koCytokine-cytokine receptor interaction|Apoptosis|Natural ki
angiogeneско04145,koPhagosome|Hematopoietic cell lineage|Regulation of actin cy
spindle pole|cornified envelope|nucleus|cytoplasm|cytoskeleton|plasma membrane|
histone acetyltransferase complex|nuclear chromatin|DNA-binding transcription f
podosome|transcription coregulator activity|nucleus|cytoplasm|cytosol|plasma me
cell adhesion|integral component of membrane
nucleus|nucleoplasm|nucleolus|DNA-templated transcription, elongation|transcrip
multicellular organism development|extracellular vesicle
nucleus|nucleolus|cytoplasm|cytosol|hydrolase activity|positive regulation of t
Golgi membko04141   Protein processing in endoplasmic reticulum
catalytic activity|integral component of membrane
DNA-binding transcription factor activity, RNA polymerase II-specific|DNA bindi
nor protein 12
ARF guanylko04144   Endocytosis
nucleic acid binding|methyltransferase activity|zinc ion binding|transferase ac
ko04666,koFc gamma R-mediated phagocytosis|Regulation of actin cytosk
metallocarboxypeptidase activity|extracellular space|peptide metabolic process|
retinoid metabolic process|beta-carotene 15,15'-monooxygenase activity|retinal
GTPase activity|GTP binding|cytosol|plasma membrane|intracellular protein trans
cytoplasm|bicellular tight junction|sensory perception of sound|integral compon
nucleus|cyko00340   Histidine metabolism
endoplasmic reticulum|oxidoreductase activity
bicellular tight junction|cerebral cortex development|apical junction complex
Golgi apparatus|carbohydrate metabolic process|acetyltransferase activity|integ
actin binding|cytosol|focal adhesion|regulation of signal transduction|actin cy
1
factor, beta receptor III
chromatin|ko04212,koLongevity regulating pathway - worm|Protein processing in e
ribosomal small subunit assembly|blastocyst formation|RNA binding|nucleoplasm|c
nucleus|cytosol

peptidase activity|extracellular matrix organization|negative regulation of tra
magnesium ion binding|protein serine/threonine kinase activity|ATP binding|cyto

endoplasmiko01040,koBiosynthesis of unsaturated fatty acids|Fatty acid elongati
ko00230,koPurine metabolism|Drug metabolism - other enzymes
sive phospko04144   Endocytosis
erase

oitor of cell death (non-protein coding)
nucleus|erko01040,kcBiosynthesis of unsaturated fatty acids|Fatty acid elongati
transmembrko04722   Neurotrophin signaling pathway
protein serine/threonine phosphatase inhibitor activity|cytoplasm|regulation of

integral component of membrane
serine-typko04610,kcComplement and coagulation cascades|Phagosome
RNA-directko04013   MAPK signaling pathway - fly
GTPase actko04014   Ras signaling pathway
nucleus|transcription, DNA-templated

serine-type endopeptidase activity|peptidase activity|protein processing|peptic
G2/M transition of mitotic cell cycle|cytoplasm|identical protein binding|prote
cytoplasm|ko04071   Sphingolipid signaling pathway
cytoplasm|cytosol|protein methylation|protein-lysine N-methyltransferase activi
regulatiorko04630   Jak-STAT signaling pathway
folic acicko04977   Vitamin digestion and absorption
calcium activated cation channel activity|voltage-gated calcium channel activit
detection ko04970   Salivary secretion
extracellular space|endoplasmic reticulum|plasma membrane|integral component of
ossification|cytoplasm|rough endoplasmic reticulum|multicellular organism devel
endonuclease activity|metal ion binding
metalloendopeptidase activity|extracellular space|heparin binding|zinc ion binc
3 (POZ) domain containing 11

eceptor 143
cytoplasm
integrin binding|interstitial matrix|cell-matrix adhesion|heparin binding|posit
ubiquitin ko04520   Adherens junction

extracellko04310   Wnt signaling pathway
phosphaticko00562,kcInositol phosphate metabolism|Phosphatidylinositol signalin
microtubule motor activity|ATP binding|cytosol|kinesin complex|microtubule|plas
transcriptko00564,kcGlycerophospholipid metabolism|Glycerolipid metabolism
microtubule cytoskeleton organization|nucleoplasm|cytoplasm|microtubule organiz
nucleoplasm|membrane|integral component of membrane|snRNA processing|nuclear me
response tko04978,kcMineral absorption|Lysosome
ko00860   Porphyrin and chlorophyll metabolism
protein phko03015,kcmRNA surveillance pathway|Dopaminergic synapse|Adrenergic s
lement regko04610   Complement and coagulation cascades
transcription regulatory region sequence-specific DNA binding|RNA polymerase II
retinoid metabolic process|copper ion binding|extracellular region|extracellula
integral component of membrane
ubiquitin-protein transferase activity|calmodulin binding|cytoplasm|cellular pr
protein pcko04144   Endocytosis
embryonic heart tube morphogenesis|calcium ion binding|nucleus|regulation of ge
skeletal system development|angiogenesis|negative regulation of glomerular filt
-protein coding RNA 619
cell morphogenesis|branching involved in ureteric bud morphogenesis|cell migrat
protein secretion|vesicle-mediated transport|integral component of Golgi membra

hormone activity|extracellular space|endoplasmic reticulum|endoplasmic reticulu
cytoplasm|negative regulation of protein kinase activity
transcription corepressor activity|catalytic activity|nucleus|methylated histor
3',5'-cyclko00230 Purine metabolism

angiogenesis|positive regulation of endothelial cell proliferation|extracellula
platelet cko04972 Pancreatic secretion
DNA-binding transcription factor activity, RNA polymerase II-specific|DNA bindi
ossification|mesoderm formation|negative regulation of cytokine production|extr
guanyl-nucko04810 Regulation of actin cytoskeleton
CDP-diacylko00564 Glycerophospholipid metabolism
high-affinity arginine transmembrane transporter activity|high-affinity lysine
DNA binding|nucleus|nucleoplasm|chromosome|cellular response to DNA damage stim
ko04145,kcPhagosome|Rap1 signaling pathway|p53 signaling pathway|TGF-
tRNA-guanine transglycosylation

protein serine/threonine kinase activity|ATP binding|nucleus|plasma membrane|pr
inositol-1ko04070 Phosphatidylinositol signaling system
e synthase ko00230,kcPurine metabolism|Drug metabolism - other enzymes
RNA polymerase II proximal promoter sequence-specific DNA binding|DNA-binding t
cell surface|glutamatergic synapse|GABA-ergic synapse|integral component of pos
transmembrko04722 Neurotrophin signaling pathway
gastrulatiko00040,kcPentose and glucuronate interconversions|Amino sugar and nu
dolichyl-cko04141,kcProtein processing in endoplasmic reticulum|N-Glycan biosyn
MAPK cascade|protein kinase activity|protein serine/threonine kinase activity|A
angiogeneско04360 Axon guidance
nuclear-trko03010 Ribosome

nucleoplasko04914,kcProgesterone-mediated oocyte maturation|Oocyte meiosis|Cell
extracellular space|positive regulation of synapse assembly|glutamatergic synap
gamma-amirko04727 GABAergic synapse
RNA polymerase II proximal promoter sequence-specific DNA binding|DNA-binding t
g 1, with ZNF domain
Rac)-activko04011,kcMAPK signaling pathway - yeast|cAMP signaling pathway|Natur
DNA-binding transcription factor activity, RNA polymerase II-specific|DNA bindi
ko04013 MAPK signaling pathway - fly
se inhibitor 3
RNA polymerase II proximal promoter sequence-specific DNA binding|proximal prom
protein serine/threonine kinase activity|ATP binding|nucleus|heart development|
phospholipko04666,kcFc gamma R-mediated phagocytosis|Endocytosis

cytosol|meko00330,kcArginine and proline metabolism|beta-Alanine metabolism|Glu
DNA-binding transcription factor activity, RNA polymerase II-specific|DNA bindi
integral component of membrane
protein serine/threonine kinase activity|ATP binding
signaling ko04151,kcPI3K-Akt signaling pathway|ECM-receptor interaction|Focal a
negative regulation of transcription by RNA polymerase II|RNA polymerase II prc
ko04151,kcPI3K-Akt signaling pathway|ECM-receptor interaction|Focal a
ko04012 ErbB signaling pathway
prostaglarko04072,kcPhospholipase D signaling pathway|Calcium signaling pathway
g transactivator, with Glu/Asp-rich carboxy-terminal domain, 4

thiol-dependent ubiquitin-specific protease activity|nucleus|cytoplasm|cytosol|
e 4 ko04974 Protein digestion and absorption
chromosome, centromeric region|DNA-binding transcription factor activity, RNA p
RNA polymerase II proximal promoter sequence-specific DNA binding|RNA polymeras
negative r ko04360,ko Axon guidance|Hedgehog signaling pathway - fly|Hedgehog sig
guanyl-nucleotide exchange factor activity|Rho guanyl-nucleotide exchange facto
SH3/SH2 adaptor activity|cytoplasm|cytosol|plasma membrane|signal transduction|
integral component of membrane
nase compl ko01200,ko Carbon metabolism|Oxidative phosphorylation|Citrate cycle (
lipid catabolic process|hydrolase activity|lysosomal lumen|extracellular exosom
Golgi membrane|endoplasmic reticulum membrane|integral component of membrane|ve
regulation of alternative mRNA splicing, via spliceosome|mRNA 3'-UTR binding|nu
actin binding|cytoplasm|actin cytoskeleton
extracellular region|plasma membrane|cell adhesion|cell recognition|integral co
microtubule cytoskeleton organization|nucleoplasm|cytoplasm|adherens junction|c
integral component of membrane
RNA binding|nucleus|cytoplasm|mRNA stabilization|RNA adenylyltransferase activi
3',5'-cycl ko00230 Purine metabolism
protease binding|mast cell mediated immunity|serine-type endopeptidase inhibito
DNA binding|nucleus|metal ion binding
formin 1 ko04510,ko Focal adhesion|Regulation of actin cytoskeleton
mesoderm f ko04910 Insulin signaling pathway
DNA-binding transcription factor activity, RNA polymerase II-specific|mitochond
endoplasmic reticulum|endoplasmic reticulum membrane|integral component of memb
nucleus|nucleolus|cytoplasm|cytosol|plasma membrane|protein export from nucleus
lgalactosa ko00512 Mucin type O-Glycan biosynthesis
histone deacetylase complex|DNA-binding transcription factor activity, RNA poly
rotein ligase 2

DNA binding|RNA binding|nucleus|RNA processing
extracellular region
catalytic activity|extracellular region|nucleus|cytoplasm|integral component of
nucleus|cytoplasm|cellular zinc ion homeostasis|detoxification of copper ion|me
DNA-binding transcription factor activity, RNA polymerase II-specific|nucleus|n
adenyl-nuc ko04141 Protein processing in endoplasmic reticulum
extracellular space|lipid metabolic process|lipid transport|lipid binding|lipop
G1/S trans ko04110,ko Cell cycle|Ubiquitin mediated proteolysis|FoxO signaling pa
MAPK casca ko04668 TNF signaling pathway
protein serine/threonine phosphatase inhibitor activity|cytoplasm|membrane|regu
ubiquitin- ko04120 Ubiquitin mediated proteolysis
DNA binding|nucleus|metal ion binding
retinal de ko00982,ko Drug metabolism - cytochrome P450|Metabolism of xenobiotics
glutathion ko00480,ko Glutathione metabolism|Thyroid hormone synthesis|Arachidoni
angiogenesis|acrosomal vesicle|negative regulation of cell-matrix adhesion|immu
DNA-binding transcription factor activity, RNA polymerase II-specific|DNA bindi
magnesium ko00562,ko Inositol phosphate metabolism|Phosphatidylinositol signalin
nucleoplasm|nucleolus|cytosol|integral component of membrane|protein ubiquitina
RNA binding|helicase activity|ATP binding|nucleolus|rRNA processing|membrane
integral component of membrane|protein heterodimerization activity|SMAD protein
ription factor 1; translocated to, 1 (cyclin D-related)
mitotic cytokinesis|kidney development|centrosome|cytosol|microtubule|plasma me

plasma membrane|spermatogenesis|cyclin-dependent protein serine/threonine kinas
angiogenesko04024,kccAMP signaling pathway|Serotonergic synapse|Leukocyte trans
MAPK cascade|regulation of cell-matrix adhesion|protein kinase activity|protein
plasma membrane|integral component of membrane|calcium ion import across plasma
nent bindiko04922,kcGlucagon signaling pathway|Longevity regulating pathway|Vas
protein serine/threonine kinase activity|ATP binding|cytoplasm|cytosol|protein
positive regulation of protein phosphorylation|nucleus|nucleoplasm|cytoplasm|gl
inorganic anion exchanger activity|plasma membrane|integral component of plasma
DNA binding|nucleus|metal ion binding
plasma menko04514,kcCell adhesion molecules (CAMs)|Leukocyte transendothelial m
cytoplasm|ko04360 Axon guidance
Golgi membko04062,kcChemokine signaling pathway|cAMP signaling pathway|Oxytocin
protein import into nucleus, translocation|nuclear inner membrane|nuclear outer
protein polyubiquitination|protein C-terminus binding|positive regulation of en
C-3 sterolko00100 Steroid biosynthesis
nucleus|mitochondrion|mitochondrial intermembrane space|oxidative phosphorylati
GTPase activator activity|extracellular region|cytoplasm|intracellular protein
lipopolysaccharide binding|mast cell mediated immunity|antigen binding|cell adh
ing 4
inositol-pko00562,kcInositol phosphate metabolism|Phosphatidylinositol signalin

GTP binding|cytoplasm|cytosol|plasma membrane|intracellular protein transport|v
negative regulation of transcription by RNA polymerase II|RNA polymerase II act
nucleus|cytoplasm|cytosol|protein methylation|methyltransferase activity|protei
Golgi membrane|nucleus|nucleoplasm|nucleolus|cytoplasm|endoplasmic reticulum me
translation elongation factor activity|GTPase activity|GTP binding|mitochondria
L-aspartatko00710,kcCarbon fixation in photosynthetic organisms|Phenylalanine,
protein-containing complex binding|cadherin binding
negative regulation of transcription by RNA polymerase II|RNA polymerase II reg
alpha-L-fuko00511 Other glycan degradation
MAPK cascako04210 Apoptosis
serine-type endopeptidase activity|extracellular region|serine-type peptidase a
condensed nuclear chromosome, centromeric region|condensed chromosome|protein k
nucleus|nucleoplasm|mitochondrial outer membrane|cytosol|protein targeting to m
negative rko04390,kcHippo signaling pathway|Hippo signaling pathway -multiple s
catalytic ko04024,kccAMP signaling pathway|Hedgehog signaling pathway
axoneme|intraciliary transport
double-strand break repair via break-induced replication|GINS complex|nucleopla
integral component of membrane
endoplasmic reticulum|endoplasmic reticulum membrane|integral component of endo
potassium channel activity|nucleus|integral component of membrane|nuclear membr
tRNA binding|in utero embryonic development|neuron migration|placenta developme
structural constituent of cytoskeleton|extracellular region|cytoplasm|lysosomal
cytosol|cytoskeleton|plasma membrane|signal transduction|nuclear body|lamellipo
GTPase actko04015 Rap1 signaling pathway
chromatin binding|nucleus|chromatin organization|regulation of transcription by
rDNA binding|RNA binding|double-stranded RNA binding|mRNA binding|ATP binding|r
transcriptko03040,kcSpliceosome|RNA transport
inactivatiko04010 MAPK signaling pathway
cytoplasm|lipid metabolic process|phosphoric diester hydrolase activity
GTPase activator activity|intracellular|intracellular protein transport|Rab GTF

myo-inositol:sodium symporter activity|plasma membrane|integral component of pl
membrane|integral component of membrane
lysosomal membrane|integral component of membrane
Golgi memb ko04141 Protein processing in endoplasmic reticulum
axoneme|sperm axoneme assembly|axoneme assembly|sperm flagellum|cilium assembly
fibrillar center|nucleus|cytosol|cytoskeleton|cell cortex|spermatogenesis|cell
microfilament motor activity|calmodulin binding|ATP binding|endosome|smooth enc
chromatin binding|nucleoplasm|histone H4-K16 acetylation|MSL complex
(non-prot ko00310 Lysine degradation

nucleus|cy ko04630,kc Jak-STAT signaling pathway|Prolactin signaling pathway
epithelial to mesenchymal transition|RNA binding|mRNA binding|nucleus|nucleopla
transporter activity|integral component of membrane|transmembrane transport
acyl-CoA c ko01212,kc Fatty acid metabolism|Valine, leucine and isoleucine degrad
Golgi memb ko04650,kc Natural killer cell mediated cytotoxicity|Endocytosis|Phago
GTPase activator activity|transporter activity|ATP synthesis coupled proton tra
DNA binding|nucleus|metal ion binding
DNA bindir ko04142,kc Lysosome|Apoptosis|Antigen processing and presentation|Phag
cytoplasm|embryonic skeletal system development
DNA binding|DNA repair
p53 binding|mitochondrion|endoplasmic reticulum|endoplasmic reticulum membrane|
RNA binding|nucleoplasm|mRNA processing|multicellular organism development|RNA
plasma membrane|cell surface|glutamatergic synapse|GABA-ergic synapse|integral
integral component of membrane
angiogenesis|positive regulation of endothelial cell proliferation|semaphorin r
DNA-binding transcription factor activity, RNA polymerase II-specific|DNA bindi
skeletal system development|angiogenesis|negative regulation of glomerular filt
extracellular space|integral component of membrane|extracellular matrix|positiv
negative regulation of transcription by RNA polymerase II|rDNA binding|chromati
microtubule|signal transduction|multicellular organism development|protein-cont
RNA polymerase II regulatory region sequence-specific DNA binding|RNA polymeras
Golgi memb ko00532 Glycosaminoglycan biosynthesis - chondroitin sulfate / derm
DNA binding|nucleus|metal ion binding
mRNA splicing, via spliceosome|RNA binding|nucleus|nucleoplasm|U2-type spliceos
hexaprenyl ko00130 Ubiquinone and other terpenoid-quinone biosynthesis
mitochondr ko00190 Oxidative phosphorylation
iated via ko04620,kc Toll-like receptor signaling pathway|Apoptosis - multiple s
mRNA splic ko03015 mRNA surveillance pathway
omal protein L37
negative r ko00564,kc Glycerophospholipid metabolism|Glycerolipid metabolism|Ethe
myoblast fusion|visual learning|postsynaptic density|cell junction|dendrite|neu
calcium ion binding|integral component of plasma membrane|cell adhesion|homophi
tif) ligan ko04060,kc Cytokine-cytokine receptor interaction|Chemokine signaling
nucleus
eye development|nucleus|cytoplasm|nervous system development|positive regulatic
spliceosomal tri-snRNP complex assembly|thiol-dependent ubiquitin-specific prot
Golgi memb ko00564,kc Glycerophospholipid metabolism|Ether lipid metabolism
neural cre ko04360 Axon guidance
calcium ion binding|mitochondrial inner membrane|neuron projection development
Golgi memb ko04130 SNARE interactions in vesicular transport
cellular magnesium ion homeostasis|magnesium ion transmembrane transporter acti

in 34A

um polypeptide
endoplasmiko01040,kcBiosynthesis of unsaturated fatty acids|Fatty acid elongati
extracellular space|integral component of membrane|extracellular matrix|positiv
A 1
magnesium ko00260,kcGlycine, serine and threonine metabolism|Carbon metabolism|
G-protein alpha-subunit binding|DNA binding|calcium ion binding|extracellular s
cell morphko04142,kcLysosome|Steroid biosynthesis
metalloendopeptidase activity|extracellular region|endoplasmic reticulum lumen|
l transforming 1
neural tube closure|heart looping|DNA binding|Golgi apparatus|centrosome|cilium
double-strand break repair via homologous recombination|DNA recombinase assembl
cell morphko04360,kcAxon guidance|Regulation of actin cytoskeleton
nucleoplasm|cytosol|protein ubiquitination|post-translational protein modificat
serine-type endopeptidase inhibitor activity|extracellular matrix structural cc
calcium ion binding|nucleus|nucleolus|plasma membrane|integral component of pla
early endosome|cytosol|protein homodimerization activity|GARP complex binding
viral oncogko04722,kcNeurotrophin signaling pathway|GnRH signaling pathway|Gap j
RNA binding|cytoplasm|cytosol|response to virus|viral process|intracellular tra
RNA polymerase II proximal promoter sequence-specific DNA binding|DNA-binding t
volume-sensitive anion channel activity|cytoplasm|endoplasmic reticulum membran
negative regulation of plasmacytoid dendritic cell cytokine production|RNA binc
nucleus|nuclear body|nuclear membrane
P-body|RNA binding|calcium ion binding|nucleoplasm|cytosol|protein phosphorylat
cornified envelope|lamin binding|nucleus|nucleoplasm|cytoplasm|intermediate fil
nase 1
d 2 ko04360 Axon guidance
Golgi membrane|serine-type endopeptidase activity|nucleus|nucleoplasm|Golgi app
cytosol|cyko04360 Axon guidance
protein serine/threonine kinase activity|protein serine/threonine/tyrosine kina
fier 1
integral component of membrane
factor binding protein 5
nucleoplasm|nuclear membrane|metal ion binding
angiogenesis|positive regulation of endothelial cell proliferation|extracellula
protein kiko04141 Protein processing in endoplasmic reticulum
integral component of membrane
photoreceptor outer segment|cytoplasm|mitochondrion|plasma membrane|cilium|cytc
P-body|resko04392,kcHippo signaling pathway -multiple species|Hippo signaling p
-protein coding RNA 1091

magnesium ko00020,kcCitrate cycle (TCA cycle)|Propanoate metabolism|Carbon meta
cytoplasm|axoneme|negative regulation of microtubule depolymerization|microtubu
n blood group)
basement membrane|plasma membrane|endocytosis|apoptotic process|cell adhesion|r
negative regulation of transcription by RNA polymerase II|RNA polymerase II prc
nucleoplasm|integral component of membrane
magnesium ion binding|microglial cell activation|positive regulation of recepto
RNA-directed DNA polymerase activity|DNA recombination|type II site-specific de
MAPK cascako04014 Ras signaling pathway

cytoplasm|ciliary basal body|cilium assembly|extracellular exosome
equatorial microtubule organizing center|gamma-tubulin complex|structural molec
cytoplasm|Golgi apparatus|Rho protein signal transduction|cellular response to
receptor 3 ko04060 Cytokine-cytokine receptor interaction
nucleus|cytoplasm|multicellular organism development|spermatid development|prot

calcium ion binding|cytoplasm|cytosol|actin filament|plasma membrane|actin fila

epeat domain 13
coma viral ko04620,kcToll-like receptor signaling pathway|T cell receptor signal
Golgi membko00532 Glycosaminoglycan biosynthesis - chondroitin sulfate / derm
G protein-coupled receptor activity|nucleolus|plasma membrane|integral componen
protein serine/threonine kinase activity|ATP binding|nucleus|cytoplasm
calcium channel activity|mitochondrion|mitochondrial inner membrane|mitochondri
sis factor ko04146 Peroxisome
T cell meck o04650 Natural killer cell mediated cytotoxicity
zinc ion transmembrane transporter activity|plasma membrane|integral component
nucleus|nucleoplasm
cell morphko03013,kcRNA transport|Regulation of actin cytoskeleton
regulatiorko04270 Vascular smooth muscle contraction
mRNA 3'-Ulko00280 Valine, leucine and isoleucine degradation
DNA-binding transcription factor activity, RNA polymerase II-specific|nucleic a
microtubule motor activity|ATP binding|centrosome|spindle|cytosol|kinesin compl
photorecepko04961,kcEndocrine and other factor-regulated calcium reabsorption|P
extracellular region
regulatiorko04974,kcProtein digestion and absorption|Renin-angiotensin system
protein serine/threonine kinase activity|calcium ion binding|ATP binding|extrac
enhancer sequence-specific DNA binding|nucleus|regulation of transcription by F
integral component of membrane
nucleosome|nuclear chromatin|DNA binding|nucleus|chromatin organization|enzyme
Golgi membrane|GTPase activity|GTP binding|plasma membrane|intracellular protei
extracellular region|endoplasmic reticulum|negative regulation of tumor necrosi
ossification|regulation of cell growth|insulin-like growth factor binding|inter
ko04320,kcDorso-ventral axis formation|MAPK signaling pathway - fly
RNA binding|nucleus|nucleoplasm|cytoplasm|mitochondrion|endoplasmic reticulum|c
mitochondrko00020,kcCitrate cycle (TCA cycle)|Pyruvate metabolism|Glycolysis /
GTPase activator activity|early endosome|cytosol|endocytosis|signal transductic
protein phko04660,kcT cell receptor signaling pathway|Axon guidance|ErbB signal
DNA-binding transcription factor activity, RNA polymerase II-specific|branching
carbonate ko00910 Nitrogen metabolism
mitochondrion|cytosol|apoptotic process|positive regulation of cysteine-type en
Golgi membrane|ubiquitin-protein transferase activity|nuclear inner membrane|nu
protein serine/threonine kinase activity|ATP binding|nucleus|protein phosphoryl
nal sialidko04142,kcLysosome|Other glycan degradation|Sphingolipid metabolism
ubiquitin-protein transferase activity|nucleus|nucleoplasm|cytosol|neuromuscula
RNA binding|cytoplasm|cytosol|ribosome|polysome|regulation of translation|trans
mRNA splicko03015 mRNA surveillance pathway
negative regulation of transcription by RNA polymerase II|nucleotide binding|tr
DNA-binding transcription factor activity, RNA polymerase II-specific|DNA bindi
peroxisomeko04146 Peroxisome
ko04390,kcHippo signaling pathway|ErbB signaling pathway

acid dehydrogenase kinase
RNA binding|cadherin binding
SH3/SH2 adaptor activity|GTPase activator activity|cytosol|motor neuron axon gu
X chromoscko04550 Signaling pathways regulating pluripotency of stem cells
in 1
Golgi membrane|microtubule organizing center|cytosol|protein monoubiquitinatior
lactosamin:ko00603 Glycosphingolipid biosynthesis - globo series
ein signaling 17
regulatiorko04974 Protein digestion and absorption
RNA polymerase II proximal promoter sequence-specific DNA binding|chromatin bir
rpin peptiko04270,kcVascular smooth muscle contraction|Renin-angiotensin system
ovarian fcko03060 Protein export
tage gated,ko04742 Taste transduction
in 36
adaptive immune response|mature B cell differentiation involved in immune respc
protein secretion|vesicle-mediated transport|integral component of Golgi membra
plasma membrane|cytoplasmic vesicle
DNA binding|chromatin binding|nucleoplasm|transcription, DNA-templated|animal c
GTPase activator activity|G protein-coupled receptor signaling pathway|negative
plasma membrane|integral component of plasma membrane|homophilic cell adhesion
metal ion binding
ion molecule
DNA binding|nucleus|metal ion binding
extracellular region|nuclear speck
skeletal system development|protease binding|metalloendopeptidase activity|extr
3-keto steko00140,kcSteroid hormone biosynthesis|Steroid biosynthesis|Ovarian s
plasma menko04966,kcCollecting duct acid secretion|Phagosome|Oxidative phosphor
microtubule organizing center|plasma membrane|intracellular membrane-bounded or
extracellular space|cell junction|extracellular matrix|positive regulation of s
mitotic cell cycle|kinetochore|condensed chromosome outer kinetochore|cytoplasm
fatty acicko01040,kcBiosynthesis of unsaturated fatty acids|Fatty acid metaboli
mRNA splicing, via spliceosome|RNA binding|nucleus
blood vesko04974 Protein digestion and absorption
oxoglutarako00020,kcCitrate cycle (TCA cycle)|Carbon metabolism|Tryptophan meta
integral component of membrane
cytoplasm|ko00260 Glycine, serine and threonine metabolism
regulation of cell growth|lysosome|rough endoplasmic reticulum|cytosol|plasma m
Golgi organization|endosome membrane|kinesin binding|lysosome localization|regu
manchette|ko04962,kcVasopressin-regulated water reabsorption|Phagosome
9, non-muko04530 Tight junction
chromosome, telomeric region|nuclear telomere cap complex|nuclear chromosome, t
ion channeko04925 Aldosterone synthesis and secretion
protein kinase activity|ATP binding|nucleus|nucleolus|peptidyl-serine phosphory
integral component of membrane
response tko04924,kcRenin secretion|Pancreatic secretion|Salivary secretion|Ins
ly 16 (monocarboxylate transporter), member 1
nucleus|cytoplasm|plasma membrane|integral component of membrane
centrosome|centriole|cytosol|centriole replication|protein domain specific binc
GTPase actko04144 Endocytosis
ic ribonucko03018 RNA degradation
long-chairko00071,kcFatty acid degradation|Fatty acid biosynthesis|Peroxisome|A

ribosomal ko03010 Ribosome
stress fiber|transmembrane receptor protein tyrosine kinase activity|transmembr
cytoplasm|protein phosphatase binding|glucose homeostasis|positive regulation c
cysteine-tko04142,kcLysosome|Apoptosis
1
extracellular matrix structural constituent|extracellular space|cell adhesion|r
negative rko04024,kccAMP signaling pathway|Hedgehog signaling pathway
endoplasmic reticulum membrane|plasma membrane|proteolysis|protein transport|ir
DNA-binding transcription factor activity, RNA polymerase II-specific|DNA bindi
protein serine/threonine kinase activity|ATP binding
ly 4 (aniorko04971,kcGastric acid secretion|Pancreatic secretion|Salivary secret
angiogenesis|extracellular region|extracellular space|intracellular|cytoplasm|p
aining 2-like
containingko04120 Ubiquitin mediated proteolysis
mitochondrion
extracellular matrix structural constituent|calcium ion binding|extracellular r
nucleus|transcription, DNA-templated
protein serine/threonine kinase activity|ATP binding|nucleoplasm|endoplasmic re

Golgi membrane|ER to Golgi vesicle-mediated transport|intra-Golgi vesicle-media
histone acetyltransferase complex|DNA-binding transcription factor activity, RN
negative rko00310 Lysine degradation
tif) ligand 3
integrin binding|extracellular matrix structural constituent|calcium ion bindir
RNA polymerase II proximal promoter sequence-specific DNA binding|proximal prom
ted protein, RP/EB family, member 1
like 1 (S. cerevisiae)
GTPase activator activity|cytoplasm|cytoskeleton|small GTPase mediated signal t
aldose 1-eko00052,kcGalactose metabolism|Glycolysis / Gluconeogenesis
ubiquitin-dependent protein catabolic process|membrane|integral component of me
cellular response to DNA damage stimulus|nuclear body|transcription regulatory
signaling receptor binding|calcium ion binding|extracellular region|cell surfac
nucleus|cytoplasm|NLS-bearing protein import into nucleus|ribosomal protein imp
phosphotyrosine residue binding|protein tyrosine phosphatase activity|cytoplasm
response tko04924,kcRenin secretion|Pancreatic secretion|Salivary secretion|Ins
cell proliferation|protein deubiquitination|negative regulation of translation|
factor binding protein 2, 36kDa
ruffle|intestinal D-glucose absorption|calcium ion binding|phosphatidylinositol
extracellko04151,kcPI3K-Akt signaling pathway|ECM-receptor interaction|Focal a
factor, bko04060,kcCytokine-cytokine receptor interaction|MAPK signaling pathw

ruffle|hyaluronic acid binding|focal adhesion|cell surface|integral component c
cytoplasm|cytoskeleton|cytoskeletal protein binding|actomyosin structure organi
G protein-coupled receptor activity|centrosome|plasma membrane|integral compone
phagocyticko04910 Insulin signaling pathway

placenta development|extracellular region
zinc ion tko04978 Mineral absorption
RNA splicing, via transesterification reactions|mRNA splicing, via spliceosome|
integral component of membrane

zinc fing‹ko04120,kcUbiquitin mediated proteolysis|p53 signaling pathway
G2/M transition of mitotic cell cycle|ubiquitin ligase complex|protein polyubiq
kidney development|phosphoprotein phosphatase activity|intracellular|plasma mem
cytoplasm|centrosome
negative regulation of transcription by RNA polymerase II|RNA polymerase II pro
death rece‹ko04115,kcp53 signaling pathway|NF-kappa B signaling pathway|Apoptosi
calmodulirko04925,kcAldosterone synthesis and secretion|Oxytocin signaling path
immunologiko04660,kcT cell receptor signaling pathway|Axon guidance|ErbB signal
magnesium ion binding|autophagy of mitochondrion|chromatin|positive regulation
cytoplasm|cell adhesion|cell migration|SH3 domain binding|protein domain specif
magnesium ion binding|phospholipid-translocating ATPase activity|ATP binding|en
negative regulation of T-helper 1 type immune response|cytokine receptor activi
cytoplasm|ko03013 RNA transport
epeat and ankyrin repeat containing 1
neurotransmitter:sodium symporter activity|taurine:sodium symporter activity|pl
synaptic transmission, dopaminergic|maternal aggressive behavior|extracellular
postsynaptko04080 Neuroactive ligand-receptor interaction
UDP-galactose transmembrane transporter activity|carbohydrate transport|integra

late endosome|Golgi apparatus|ubiquitin-dependent protein catabolic process|int
Golgi membrane|UDP-N-acetylglucosamine transmembrane transporter activity|Golgi
sponsive serine-rich 1

regulatiorko04971,kcGastric acid secretion|Cholinergic synapse|Pancreatic secre
RNA binding|extracellular region|cytosol|plasma membrane|signal transduction|ce
glutathiorko00480 Glutathione metabolism
microfibriko04350 TGF-beta signaling pathway
mitochondrion|mitochondrial proton-transporting ATP synthase complex assembly
ack inhibitor 1

iron ion bko03320,kcPPAR signaling pathway|Primary bile acid biosynthesis
receptor rko04144 Endocytosis
magnesium ion binding|phospholipid-translocating ATPase activity|ATP binding|en
spindle pole|nucleus|centrosome|centriole|microtubule organizing center|cytosol
rough endoplasmic reticulum|SRP-dependent cotranslational protein targeting to
G protein-coupled receptor activity|plasma membrane|integral component of membr
appa light ko04722,kcNeurotrophin signaling pathway|Toll-like receptor signaling
integral component of plasma membrane|inflammatory response|cell surface recept

positive rko04060,kcCytokine-cytokine receptor interaction|Jak-STAT signaling p
RNA splicirko03040 Spliceosome
transcription coactivator activity|nucleus|nucleoplasm|cytoplasm|cytosol|regula
MAPK cascako04060,kcCytokine-cytokine receptor interaction|Phospholipase D sign
autophagosome assembly|osteoblast differentiation|tissue homeostasis|nucleus|au
cytosol|membrane|dendrite|neuron projection development|neuronal cell body|bone
activation of MAPK activity|angiogenesis|extracellular region|Golgi lumen|integ
activation of MAPK activity|cell cycle
ubiquitin-protein transferase activity|nucleus|nucleoplasm|cytosol|neuromuscula
RNA binding|nucleus|nucleoplasm|transcription factor complex|DNA dealkylation i
chromatin binding|calcium ion binding|extracellular region|cytoplasm|cell adhes
extracellular exosome

immunoglobulin production|nucleolus|cytoplasm|endoplasmic reticulum|endoplasmic
nucleoplasm|transcription elongation from RNA polymerase II promoter|transcript
voltage-gako04713,kcCircadian entrainment|Aldosterone synthesis and secretion|C
nuclear envelope|nucleoplasm|cytosol|protein import into nucleus|signal transdu
ynthase 1 ko01230,kcBiosynthesis of amino acids|Alanine, aspartate and glutamat
DNA-bindirko00310 Lysine degradation
calcium ion binding|extracellular region|extracellular space|endoplasmic reticu
lysosome|lysosomal membrane|endosome|lysosome organization|lysosomal transport|
dopamine:sodium symporter activity|plasma membrane|integral component of plasma
GARP complex|Golgi apparatus|cytosol|lysosomal transport|endosome membrane|prot
Golgi membko04141,kcProtein processing in endoplasmic reticulum|N-Glycan biosyn
protein phosphatase inhibitor activity|negative regulation of phosphatase activ
angiogenesis|phosphatidylserine binding|integrin binding|extracellular matrix s
ubiquitin-ko04330 Notch signaling pathway
membranousko04390,kcHippo signaling pathway|Melanogenesis|mTOR signaling pathwa
nucleus
Golgi cis cisterna|Golgi apparatus|cis-Golgi network|cytosol|Golgi organization
DNA binding|calcium ion binding|ATP binding|nucleus|cytoplasm|cytoskeleton|plas
actin binko04320 Dorso-ventral axis formation
cytoplasm|autophagy|autophagosome maturation
cornified envelope|calcium ion binding|plasma membrane|cell-cell adherens junct
Golgi trans cisterna|cytoplasm|Golgi apparatus|Golgi medial cisterna|cytosol|cy
extracellular matrix structural constituent|calcium ion binding|collagen bindir
RNA polymerase II proximal promoter sequence-specific DNA binding|DNA-binding t
granular component|DNA binding|RNA binding|nucleoplasm|nucleolus|cytosolic larg
cell surface|integral component of membrane|extracellular exosome
early endosome|Notch signaling pathway|protein ubiquitination|transferase activ
microtubulko04540,kcGap junction|Apoptosis|Phagosome
kinase, reko04066,kcHIF-1 signaling pathway|Insulin signaling pathway|Leukocyte
integral component of membrane|transmembrane transport|extracellular exosome
cornified envelope|desmosome assembly|protein kinase C binding|nucleus|nucleopl
DNA binding|nucleus|metal ion binding
ex locus ko04010 MAPK signaling pathway
beta-1,3-N-ko00533,kcGlycosaminoglycan biosynthesis - keratan sulfate|Glycosphin
RNA polymerase II proximal promoter sequence-specific DNA binding|chromatin bir

actin binding|cytosol|cytoskeleton|focal adhesion|protein domain specific bindi
negative regulation of transcription by RNA polymerase II|skeletal system devel
exocyst|phosphatidylinositol-4,5-bisphosphate binding|plasma membrane|cell-cell
proteasome complex|nucleus|cytoplasm|ubiquitin-dependent protein catabolic proc
negative regulation of receptor recycling|endosome|early endosome|late endosome

mitotic cytokinesis|microtubule bundle formation|microtubule motor activity|tra
nucleus|Wnt signaling pathway|regulation of canonical Wnt signaling pathway|pos
cytoplasm|cytosol|protein methylation|protein-lysine N-methyltransferase activi
cysteine-type endopeptidase activity|thiol-dependent ubiquitin-specific proteas
s-related ko04621,kcNOD-like receptor signaling pathway|Cytosolic DNA-sensing p
ion molecuko04514,kcCell adhesion molecules (CAMs)|Natural killer cell mediated
RNA-directed DNA polymerase activity|DNA recombination|type II site-specific de
ko04514,kcCell adhesion molecules (CAMs)|T cell receptor signaling pa
gulator of MAPK1-like

visual perception|embryo development ending in birth or egg hatching|integral c
SNARE binding|phosphatidylserine binding|calcium ion binding|calcium-dependent
ko04514 Cell adhesion molecules (CAMs)
transcription regulatory region sequence-specific DNA binding|RNA polymerase II
integral component of membrane
neuron mig ko04923,kc Regulation of lipolysis in adipocytes|Gap junction|Vascular
steroid hormone receptor activity|steroid binding|nuclear envelope|membrane|int

(50kDa dystrophin-associated glycoprotein)

d/antisense RNA
cell morphogenesis|actin binding|plasma membrane|establishment or maintenance c
centrosome|cilium|smoothened signaling pathway|protein transport|intraciliary t
tif) ligan ko04060,kc Cytokine-cytokine receptor interaction|Chemokine signaling
transmembrane signaling receptor activity|vacuolar membrane|plasma membrane|int

cytoplasm|plasma membrane|integral component of plasma membrane|glycosaminoglyc
Golgi membrane|phospholipid binding|cytoplasm|cytosol|intracellular protein tra
intracellular
mp component A
menting de ko03450 Non-homologous end-joining
in protein ligase 2
transmembr ko04610 Complement and coagulation cascades
ide exchange factor (GEF) 17
phosphopro ko00564,kc Glycerophospholipid metabolism|Glycerolipid metabolism|Ethe
RNA binding|nucleus|cytosol|plasma membrane|membrane
protein deneddylation|cytoplasm|COP9 signalosome
ar ribonucleoprotein D (AU-rich element RNA binding protein 1, 37kDa)
Golgi memb ko04072,kc Phospholipase D signaling pathway|Endocytosis
ATP bindir ko04010,kc MAPK signaling pathway|Estrogen signaling pathway|Protein p
nucleus|cytoplasm|cytosol|polysome|protein folding|microtubule-based process|tu
MAPK cascade|liver development|regulation of acute inflammatory response|serine
teoglycan ko04512 ECM-receptor interaction
integral component of membrane|cell junction|synaptic vesicle membrane|melanoso
autophagosome membrane|lysosomal membrane|autophagy|integral component of membr
plasma membrane|signaling|neuromuscular junction|glutamatergic synapse|choliner
integral component of membrane
activatior ko04015,kc Rap1 signaling pathway|PI3K-Akt signaling pathway|Phospholi
or recepto ko04060 Cytokine-cytokine receptor interaction
epithelial to mesenchymal transition|cytoplasm|filamin binding|actin filament b
integral component of membrane
integral component of plasma membrane|homophilic cell adhesion via plasma membr

peptidyl-prolyl cis-trans isomerase activity|calcium ion binding|endoplasmic re
DNA binding|nucleus|metal ion binding

actin binding|signaling receptor binding|cytoplasm|cytosol|brush border|cell cc
-N-acetylg ko00603 Glycosphingolipid biosynthesis - globo series
nucleus|plasma membrane|cytoskeleton organization|adenylate cyclase-inhibiting
nucleoplasm
ATP bindir ko00562,kc Inositol phosphate metabolism|Regulation of actin cytoskele

trophic fa ko04024,kc cAMP signaling pathway|Neurotrophin signaling pathway|MAPK
RNA polymerase II proximal promoter sequence-specific DNA binding|DNA-binding t
protein serine/threonine kinase activity|ATP binding|cytoplasm|cytoskeleton|int
RNA cap bi ko03013 RNA transport

GABA-A rec ko04726,kc Serotonergic synapse|GABAergic synapse|Retrograde endocanna
regulation of receptor recycling|GTPase activator activity|early endosome|cytos
G protein-coupled receptor activity|gonadotropin-releasing hormone receptor act
RNA-directed DNA polymerase activity|DNA recombination|type II site-specific de
tRNA-intron endonuclease activity|nucleic acid binding|nucleoplasm|nucleolus|tR
phospholipase activity|calcium ion binding|cytosol|phosphatidylinositol biosynt
inorganic ko04972,kc Pancreatic secretion|Proximal tubule bicarbonate reclamatio
Golgi cis cisterna|Golgi membrane|spindle pole|Golgi apparatus|cis-Golgi networ
extracellular region|cellular protein modification process|zinc ion binding|glu
Golgi memb ko04141 Protein processing in endoplasmic reticulum
negative regulation of transcription by RNA polymerase II|RNA polymerase II pro
ubiquitin ko04111 Cell cycle - yeast
cytoplasm|cytoskeleton|adherens junction|bicellular tight junction|protein binc
GTPase activator activity|cytosol|regulation of G protein-coupled receptor sigr
plasma membrane|positive regulation of gene expression|positive regulation of e
nber 3
negative regulation of transcription by RNA polymerase II|chromatin|epithelial
to (CD73) ko00230,kc Purine metabolism|Pyrimidine metabolism|Nicotinate and nico
ko04114 Oocyte meiosis
drome chro ko00512 Mucin type O-Glycan biosynthesis
NA 1
endoplasmi ko04530 Tight junction
microfibril|extracellular matrix structural constituent|extracellular region|ex
microfibril|extracellular matrix structural constituent|extracellular region|ex
structural ko04151,kc PI3K-Akt signaling pathway|ECM-receptor interaction|Focal a
DNA binding|nucleus|metal ion binding
MAPK casca ko04014 Ras signaling pathway
protein serine/threonine phosphatase activity|cytosol|negative regulation of pr
actin binding|Rho GTPase binding|actin cytoskeleton organization|perinuclear re
signaling receptor binding|extracellular space|transmembrane receptor protein t
4-hydroxyb ko00130 Ubiquinone and other terpenoid-quinone biosynthesis
G protein- ko04024,kc cAMP signaling pathway|Cholinergic synapse|Calcium signalin
negative regulation of transcription by RNA polymerase II|RNA polymerase II pro
nucleic acid binding|extracellular space|plasma membrane|ultradian rhythm|exter
leukocyte ko04024,kc cAMP signaling pathway|MAPK signaling pathway|Axon guidance
chromatin ko03015 mRNA surveillance pathway
DNA-binding transcription factor activity, RNA polymerase II-specific|DNA bindi
G2/M transition of mitotic cell cycle|nucleus|nucleolus|cytosol|apoptotic proce
cytosol|transmembrane receptor protein tyrosine kinase signaling pathway|axon g
l (galacto ko00603,kc Glycosphingolipid biosynthesis - globo series|Glycosphingol
cytosol

atricopeptide repeats 2
membrane a ko04360 Axon guidance
ine signal ko04630,kc Jak-STAT signaling pathway|Osteoclast differentiation|Insul
angiogenes ko00970 Aminoacyl-tRNA biosynthesis

nuclear e›ko03018 RNA degradation
nucleoplasko04120,kcUbiquitin mediated proteolysis|Progesterone-mediated oocyte
tif) ligan‹ko04060,kcCytokine-cytokine receptor interaction|Chemokine signaling
ıg protein 20
DNA binding|DNA-binding transcription factor activity|nucleus|metal ion binding
cytosol|focal adhesion|multicellular organism development|ruffle membrane|negat
DNA-binding transcription factor activity, RNA polymerase II-specific|DNA bindi
actin filament organization|heart development|cytoskeletal protein binding|stru
3'-5'-exorko03018 RNA degradation
plasma membrane|protein phosphatase regulator activity
RNA cap binding|RNA binding|mRNA 3'-UTR binding|cytoplasm|translational initiat
skeletal system development|inorganic phosphate transmembrane transporter activ
ɔlastosis ko04014,kcRas signaling pathway|Dorso-ventral axis formation|MAPK sig
protein tyrosine phosphatase activity|nucleus|nucleoplasm|cytoplasm|endosome|ea
NA-damage-ko04068,kcFoxO signaling pathway|p53 signaling pathway|Apoptosis|MAPK
ko04060,kcCytokine-cytokine receptor interaction|Signaling pathways r
dolichyl-pko00510 N-Glycan biosynthesis
spliceosonko04011,kcMAPK signaling pathway - yeast|Cell cycle - yeast|RNA trans
phagocytic cup|morphogenesis of an epithelial sheet|GTPase activator activity|c
ɔn factor 1
metanephrcko04360 Axon guidance
kidney development|metalloendopeptidase activity|plasma membrane|proteolysis|en
mitochondrial promoter sequence-specific DNA binding|RNA binding|DNA-directed 5
regulation of alternative mRNA splicing, via spliceosome|endocardial cushion de
farnesyl-cko00909,kcSesquiterpenoid and triterpenoid biosynthesis|Steroid biosy
morphogenesis of an epithelial sheet|cytoplasm|cell-cell junction|bicellular ti
cell adhesko04514 Cell adhesion molecules (CAMs)
calcium icko04144 Endocytosis
cytoplasm|signal transduction|nervous system development|axon guidance|axon|neg
hematopoietic progenitor cell differentiation
ko04350 TGF-beta signaling pathway
ıtaining, X-linked 2

RNA binding|double-stranded RNA binding|cytoplasm|endoplasmic reticulum|rough e
tissue honko00561,kcGlycerolipid metabolism|Pentose and glucuronate interconver
negative regulation of transcription by RNA polymerase II|DNA-binding transcrip
extracellular exosome
DNA binding|DNA-binding transcription factor activity|nucleus|nucleoplasm|bicel
stress fiber|positive regulation of protein phosphorylation|actin binding|cytop

low-affinity glucose:sodium symporter activity|glucose:sodium symporter activit
extracellular region|integral component of membrane|negative regulation of cell
fatty-acylko00562,kcInositol phosphate metabolism|Propanoate metabolism|beta-Al
ein signal ko04921,kcOxytocin signaling pathway|cGMP-PKG signaling pathway|Olfac
cytoplasm|ko00520,kcAmino sugar and nucleotide sugar metabolism|Fructose and ma
Golgi membrane|GTPase activity|GTP binding|endoplasmic reticulum membrane|Golgi
GTPase activity|GTP binding|endoplasmic reticulum|endoplasmic reticulum organiz
ɔoprotein receptor
condensed ko03013 RNA transport
virus receɛko04145,kcPhagosome|Regulation of actin cytoskeleton|PI3K-Akt signali
lement regıko04640,kcHematopoietic cell lineage|Complement and coagulation casca

nucleus
)lastosis virus E26 oncogene homolog
ko03440 Homologous recombination
hyaluronoglucosaminidase activity|hyaluronic acid binding|extracellular regior
G2/M trans:ko04114,kcOocyte meiosis|Cell cycle|Hippo signaling pathway|PI3K-Akt

lysophosphko00564 Glycerophospholipid metabolism
ribosomal large subunit export from nucleus|regulation of cyclin-dependent prot
negative rko04924,kcRenin secretion|Axon guidance|Glucagon signaling pathway|T
pantothenako00770 Pantothenate and CoA biosynthesis
centrosome|centriole|spindle|cytosol|microtubule|plasma membrane|centriolar sub
ected), mu ko03450 Non-homologous end-joining

skeletal :ko00520,kcAmino sugar and nucleotide sugar metabolism|Glycosphingolip
ubiquitin ligase complex|protein polyubiquitination|autophagy of mitochondrion|
sensory perception of sound|phosphatase binding|stereocilium|stereocilium maint
acid phosphatase activity|non-membrane spanning protein tyrosine phosphatase ac
eye develcko04015 Rap1 signaling pathway
G protein-ko04730 Long-term depression
1, regulatory subunit 13 like
RNA-directed DNA polymerase activity|endonuclease activity|DNA recombination|me
nuclear chromosome, telomeric region|translation repressor activity, mRNA regul
nucleoplasm|cytosol
serine C-pko04071,kcSphingolipid signaling pathway|Sphingolipid metabolism
actin binding|cytosol|cytoskeleton|ubiquitin-dependent protein catabolic proces
establishmko04530 Tight junction
endosome|plasma membrane|heparin binding|integral component of membrane|transpc
calcium ion binding|integral component of plasma membrane|cell adhesion|homophi
1, heavy cko04962,kcVasopressin-regulated water reabsorption|Phagosome
DNA damageko00310 Lysine degradation
plasma membrane|drug transmembrane transport|drug transmembrane transporter act
factor binding protein 7

DNA-binding transcription factor activity, RNA polymerase II-specific|ovarian f
microtubule cytoskeleton organization|tubulin-tyrosine ligase activity|ATP binc
extracellular region|nucleus|cytosol|cytoskeleton|extracellular exosome|apoptot
isease 1 (autosomal dominant)
nucleoplasko04120,kcUbiquitin mediated proteolysis|HIF-1 signaling pathway
DNA bindirko04390,kcHippo signaling pathway|Melanogenesis|mTOR signaling pathwa
antigen prko04612 Antigen processing and presentation
extracellular region
regulatiorko03040 Spliceosome
t 1, s subcko04610 Complement and coagulation cascades
integral component of membrane
negative regulation of systemic arterial blood pressure|inward rectifier potass
response to dietary excess|phospholipid binding|cytoplasm|cytosol|cytoskeleton|
RNA polymerase II proximal promoter sequence-specific DNA binding|RNA polymeras
MAPK cascako04014 Ras signaling pathway
protein phko03015,kcmRNA surveillance pathway|Hippo signaling pathway|Tight jun
etitive matrix 5
g protein :ko04062,kcChemokine signaling pathway|MAPK signaling pathway|Rap1 sig

ATP binding|cytoplasm|Golgi apparatus|cytosol|apoptotic process|inflammatory re
2-oxoglutako00310 Lysine degradation
cytoplasmic exosome (RNase complex)|translation elongation factor activity|GTPa
actin monomer binding|cytoplasm|cytoskeleton|actin filament organization|regula
integral component of membrane
magnesium ko00230,kcPurine metabolism|Biosynthesis of amino acids|Pentose phosp
negative rko00760 Nicotinate and nicotinamide metabolism
chromatin ko04140,kcRegulation of autophagy|Glucagon signaling pathway|Adipocyt
idoglycan-binding, domain containing 2
ko04520,kcAdherens junction|Leukocyte transendothelial migration|Regu
DNA binding|nucleolus|early endosome|cytosol|protein targeting to membrane|endc
one S-transko00982,kcDrug metabolism - cytochrome P450|Glutathione metabolism|Me
RNA binding|nucleus|mRNA processing|RNA splicing|anatomical structure morphoger
ng 1
e, serine/ko04630 Jak-STAT signaling pathway
microfibril|extracellular matrix structural constituent|extracellular region|ce
intrinsic component of membrane|lysosome localization|organelle transport along
actin binding|plasma membrane|cellular defense response|signal transduction|act
negative regulation of transcription, DNA-templated
trix associated, actin dependent regulator of chromatin, subfamily a, member 4
mitochondrion|oxidoreductase activity
acute inflammatory response|DNA binding|chromatin binding|nucleus|cytosol|prote
extracellular space|enzyme activator activity|peptidase inhibitor activity|resp
nuclease activity|nucleus|metal ion binding
peptide amidation|response to hypoxia|long-chain fatty acid metabolic process|p
plasma memko04745,kcPhototransduction - fly|Aldosterone synthesis and secretion
integral component of membrane
guanylate ko04921,kcOxytocin signaling pathway|Renin secretion|Gap junction|Cir
Golgi cis cisterna|Golgi apparatus|cis-Golgi network|cytosol|Golgi organizatior
plasma memko04080 Neuroactive ligand-receptor interaction
RNA bindirko04141,kcProtein processing in endoplasmic reticulum|N-Glycan biosyn
ctor 2D ko04022 cGMP-PKG signaling pathway
podosome|nucleus|cytoplasm|cytosol|plasma membrane|focal adhesion|cytoskeleton
containing 102B
e similarity 219, member A
DNA-binding transcription factor activity, RNA polymerase II-specific|angiogene
ER ubiquitko04141 Protein processing in endoplasmic reticulum
fic 7
lysosome|lysosomal membrane|late endosome|cytosol|protein transport|membrane|er
GTPase activator activity|cytoplasm|intracellular protein transport|Rab GTPase
cartilage ko04974 Protein digestion and absorption
integral cko03018 RNA degradation
RNA polymerase II proximal promoter sequence-specific DNA binding|DNA-binding t
DNA binding|nucleus
2-acylglycko04975,kcFat digestion and absorption|Glycerolipid metabolism
tRNA bindiko00970,kcAminoacyl-tRNA biosynthesis|Selenocompound metabolism
ion moleculko04650,kcNatural killer cell mediated cytotoxicity|Cell adhesion mol
membrane|oxidoreductase activity, acting on the aldehyde or oxo group of donors
nucleic acid binding|cytoplasm|cytosol|regulation of transcription, DNA-templat
cytosol|nuko00983 Drug metabolism - other enzymes
early endcko04514 Cell adhesion molecules (CAMs)

nucleoplasm|intracellular membrane-bounded organelle
structural constituent of cytoskeleton|cytosol|intermediate filament|plasma mem
RNA polymeko04550 Signaling pathways regulating pluripotency of stem cells
virus receptor activity|acrosomal vesicle|signaling receptor binding|integrin b
MAPK cascade|Ras guanyl-nucleotide exchange factor activity|signaling receptor
amino acid transmembrane transport|plasma membrane|integral component of plasma
taining family, member 3
phosphatidylserine binding|phospholipid transporter activity|endoplasmic reticu
RNA binding|motor activity|microtubule motor activity|ATP binding|endoplasmic r
structural molecule activity|cytosol|enzyme binding|keratinization|keratin fila
linked 1
toll-like receptor signaling pathway|MyD88-dependent toll-like receptor signali
voltage-gated potassium channel activity|plasma membrane|potassium ion transpor
autophagosome assembly|mitochondrion|endoplasmic reticulum|apoptotic process|vi
actin binding|cytoplasm|microtubule organizing center|microtubule cytoskeleton
n molecule ko04514 Cell adhesion molecules (CAMs)
type A ko04024,kccAMP signaling pathway|Renin secretion|Calcium signaling pa
amyloid-beta binding|steroid binding|endoplasmic reticulum|plasma membrane|inte
receptor recycling|endosome membrane|early endosome membrane|transferrin transp
stress fibko04910,kcInsulin signaling pathway|Adherens junction|PPAR signaling
transcription corepressor activity|extracellular space|nucleus|nucleoplasm|tran
l
RNA binding|mRNA 3'-UTR binding|polynucleotide adenylyltransferase activity|ATP
RNA polymerase II regulatory region sequence-specific DNA binding|DNA-binding t
euron 1, tko03013 RNA transport
endoplasmiko04141 Protein processing in endoplasmic reticulum
nucleus|cytoplasm|plasma membrane|G protein-coupled receptor signaling pathway|
se, quinonko00130 Ubiquinone and other terpenoid-quinone biosynthesis
estradiol 17-beta-dehydrogenase activity|cytosol|estrogen biosynthetic process|
leukocyte mediated cytotoxicity|platelet degranulation|extracellular region|cyt
e alpha-2,ko00533,kcGlycosaminoglycan biosynthesis - keratan sulfate|Glycosphin
extracellular space|endosome|endoplasmic reticulum|Golgi apparatus|polysacchari
inorganic ko04971,kcGastric acid secretion|Pancreatic secretion|Salivary secret
amyloid-beko04390,kcHippo signaling pathway|Melanogenesis|mTOR signaling pathwa
ko01230,kcBiosynthesis of amino acids|Alanine, aspartate and glutamat
protein kinase activity|protein serine/threonine kinase activity|ATP binding|pr
integral component of membrane
calcium activated cation channel activity|intracellular calcium activated chlor
nucleus|peroxisome proliferator activated receptor signaling pathway|fat cell c
chromatin|blood vessel maturation|chromatin binding|RNA binding|nucleus|cytopla
positive rko04060 Cytokine-cytokine receptor interaction
host gene 14
P-body|cytosol|muscle organ development|protein ubiquitination|neurogenesis|pro
negative regulation of transcription by RNA polymerase II|RNA polymerase II reg
ko04610 Complement and coagulation cascades
protein serine/threonine kinase activity|protein serine/threonine/tyrosine kina
4 (gut) ko04550 Signaling pathways regulating pluripotency of stem cells
virus receko04360 Axon guidance
regulatiorko04068,kcFoxO signaling pathway|Progesterone-mediated oocyte maturat
actin bincko04810 Regulation of actin cytoskeleton
calcium-dependent phospholipid binding|plasma membrane|extracellular exosome|ce

extracellular matrix structural constituent|calcium ion binding|collagen bindir
amyloid-bεko04024,kccAMP signaling pathway|Dopaminergic synapse|Retrograde endo
negative regulation of transcription by RNA polymerase II|four-way junction DNA
neuron migration|low-density lipoprotein particle receptor activity|integral cc
G protein-ko04080,kcNeuroactive ligand-receptor interaction|cAMP signaling path
cytoplasm
phospholipase A2 activity|plasma membrane|phosphatidylcholine biosynthetic proc
ιase compl‹ko01200,kcCarbon metabolism|Oxidative phosphorylation|Citrate cycle (
angiogeneѕko04810,kcRegulation of actin cytoskeleton|Adherens junction|Fc gamma
mitotic cytokinesis|angiogenesis|GTPase activity|GTP binding|nucleus|cytoplasm|
nucleus|nucleoplasm|mitochondrion|zinc ion binding|nuclear receptor transcripti
microtubulko04540,kcGap junction|Phagosome
trophin-as:ko04512 ECM-receptor interaction
ə, sub-fam:ko04142,kcLysosome|ABC transporters
RNA binding|ubiquitin-protein transferase activity|cytoplasm|cytosol|zinc ion b
retinoid metabolic process|extracellular space|endoplasmic reticulum|Golgi lume
extracellιko04142 Lysosome
2
virus receptor activity|inorganic phosphate transmembrane transporter activity|
ιosphoryla:ko00230,kcPurine metabolism|Pyrimidine metabolism|Nicotinate and nico
protease binding|podosome|phosphatidylinositol-4,5-bisphosphate binding|cytosol
B cell homko04151 PI3K-Akt signaling pathway
nucleoplasm
response tko04141 Protein processing in endoplasmic reticulum
l
factor, beta-induced, 68kDa

RNA-directed DNA polymerase activity|DNA recombination|type II site-specific de
G2/M transition of mitotic cell cycle|acrosomal vesicle|centrosome|microtubule
ubiquitin-protein transferase activity|cytoplasm|polar microtubule|cell cycle|p
reading frame 58

endopeptidase activity|serine-type endopeptidase activity|nucleus|mitochondrial
Swr1 complex|DNA binding|transcription coactivator activity|helicase activity|h
serine-type endopeptidase activity|scavenger receptor activity|plasma membrane|
cytochromεko00520 Amino sugar and nucleotide sugar metabolism
57kDa
-protein coding RNA 643
Golgi membrane|nucleolus|cytoplasm|plasma membrane|integral component of membra
DNA-binding transcription factor activity, RNA polymerase II-specific|lymphangi
kemia, cytoplasmic
reading frame 27
chromatin silencing at rDNA|RNA polymerase I CORE element sequence-specific DNA
cysteine-type endopeptidase activity|thiol-dependent ubiquitin-specific proteas
lgalactosaιko00512 Mucin type O-Glycan biosynthesis

DNA-binding transcription factor activity, RNA polymerase II-specific|nucleus|s
maturation of SSU-rRNA from tricistronic rRNA transcript (SSU-rRNA, 5.8S rRNA,
negative regulation of transcription by RNA polymerase II|RNA polymerase II prc
spindle pole|microtubule bundle formation|RNA binding|extracellular space|cytop
plasma memko04514 Cell adhesion molecules (CAMs)

actin binc ko04270 Vascular smooth muscle contraction
stearoyl-(ko04212,kc Longevity regulating pathway - worm|Biosynthesis of unsatur
tif) recep ko04060,kc Cytokine-cytokine receptor interaction|Chemokine signaling
endosome|trans-Golgi network|cytosol|integral component of membrane|retrograde
Golgi membrane|endoplasmic reticulum membrane|endoplasmic reticulum-Golgi inter
ubiquitin-protein transferase activity|calmodulin binding|nucleoplasm|centroson
ATP binding|magnesium ion homeostasis|magnesium ion transmembrane transporter a
RNA polymerase I transcription factor complex|DNA binding|nucleoplasm|transcrip
cytokine a ko04390,kc Hippo signaling pathway|TGF-beta signaling pathway
DNA-binding transcription factor activity, RNA polymerase II-specific|DNA bindi
autophagos ko04141 Protein processing in endoplasmic reticulum
negative regulation of transcription by RNA polymerase II|enzyme inhibitor acti
coupled receptor E5

nuclear-transcribed mRNA catabolic process, nonsense-mediated decay|eye develop
negative regulation of transcription by RNA polymerase II|DNA-binding transcrip
GTPase activity|GTP binding|cytoplasm|lysosomal membrane|vacuolar membrane|plas
actin binc ko04310 Wnt signaling pathway
reading frame 54
renal wate ko04540,kc Gap junction|GABAergic synapse|Rap1 signaling pathway|Longe
extracellular region|apoptotic process|azurophil granule lumen|neutrophil degra
nucleus|nucleoplasm|cytosol|nuclear speck|endodeoxyribonuclease activity, produ
activatior ko04916,kc Melanogenesis|mTOR signaling pathway|Axon guidance|Hippo si
cytoplasm| ko04310 Wnt signaling pathway
cytoplasm|negative regulation of signal transduction
ember 35
angiogenesis|chondrocyte differentiation|Notch binding|integrin binding|insulir
regulatory region RNA binding|RNA binding|aconitate hydratase activity|cytoplas
RNA polymerase II complex binding|mRNA binding|nucleoplasm|spliceosomal complex
plasma mem ko04514 Cell adhesion molecules (CAMs)
integral component of membrane|WW domain binding
MAPK cascade|inactivation of MAPK activity|stem cell factor receptor binding|nu
se 1 ko00240,kc Pyrimidine metabolism|Selenocompound metabolism
rotein
DNA-binding transcription factor activity, RNA polymerase II-specific|DNA bindi
lgalactosar ko00512 Mucin type O-Glycan biosynthesis
negative regulation of transcription by RNA polymerase II|nucleosome|double-str
heart looping|actin binding|nucleus|cytoskeleton organization|heart development
ected), de ko03030,kc DNA replication|Mismatch repair|Homologous recombination|Nu
endoplasmic reticulum membrane|multicellular organism development|integral comp
skeletal m ko04080 Neuroactive ligand-receptor interaction
ubiquitin ligase complex|protein polyubiquitination|adaptive immune response|ub
protein serine/threonine kinase activity|ATP binding|integral component of memb
interaction 1 (breast)
nucleoplas ko03008,kc Ribosome biogenesis in eukaryotes|mRNA surveillance pathway
mesoderm f ko04060,kc Cytokine-cytokine receptor interaction|Axon guidance|Hippo
ide alpha-! ko00510,kc N-Glycan biosynthesis|Other types of O-glycan biosynthesis
DNA-binding transcription factor activity, RNA polymerase II-specific|DNA bindi
fatty acic ko03320,kc PPAR signaling pathway|Peroxisome
phosphatidylinositol-4,5-bisphosphate binding|cytosol|plasma membrane|sensory p
positive regulation of JNK cascade|metal ion binding|protein autoubiquitinatior

catalytic activity|cytoplasm|peroxisome
metalloendopeptidase activity|mitochondrion|mitochondrial matrix|proteolysis|pe
ESCRT I cc ko04144 Endocytosis
calcium ion binding|plasma membrane|cell-cell adherens junction|cell-cell junct

ubiquitin ligase complex|nucleus|ubiquitin conjugating enzyme binding|ubiquitin
epidermal ko04144 Endocytosis
ubiquitin ligase complex|chromatin binding|ubiquitin-protein transferase activi

O-phospho-ko00260,kc Glycine, serine and threonine metabolism|Carbon metabolism|
angiogenes ko04270 Vascular smooth muscle contraction
e similarity 127, member C
calcium ion binding|calcium-dependent phospholipid binding
RNA polymerase I transcription factor complex|fibrillar center|RNA binding|DNA-
proximal promoter sequence-specific DNA binding|DNA-binding transcription facto
brainstem ko00230,kc Purine metabolism|One carbon pool by folate
P-body|cytosol|neurogenesis
Golgi memb ko04612 Antigen processing and presentation
Golgi membrane|calcium-transporting ATPase activity|calcium ion binding|ATP bin
regulation of alternative mRNA splicing, via spliceosome|RNA binding|single-str
extracellular region|nucleus|nucleoplasm|cytoplasm|visual perception|locomotory
endopeptic ko04610 Complement and coagulation cascades
ide exchang ko04910,kc Insulin signaling pathway|Rap1 signaling pathway|Neurotroph
positive regulation of cytokine production|cytoplasm|endoplasmic reticulum|Golg
chromosome, centromeric region|condensed chromosome, centromeric region|nuclear
ase, serine 2
actin binding|calmodulin binding|cytosol|cell-cell adherens junction|focal adhe
ko00982,kc Drug metabolism - cytochrome P450|Glutathione metabolism|Me
reproduction|DNA binding|nucleoplasm|transcription, DNA-templated|cell cycle|tr

lase 1 ko00330,kc Arginine and proline metabolism|Glutathione metabolism
RNA binding|cytoplasm|ribosome binding|positive regulation of translation|posit
transcription corepressor activity|extracellular region|extracellular space|tra
negative regulation of protein phosphorylation|actin binding|monooxygenase acti
ATP binding|cell|cation transport|cellular calcium ion homeostasis|membrane|int
nuclear inner membrane|integral component of membrane|fat cell differentiation|
mixed-lineage leukemia; translocated to, 1
ko04919,kc Thyroid hormone signaling pathway|Notch signaling pathway|D
amino acid cleaving enzyme)
eptide ko04080 Neuroactive ligand-receptor interaction

ber 2
angiogenes ko04514,kc Cell adhesion molecules (CAMs)|Leukocyte transendothelial m
hematopoietic progenitor cell differentiation|RNA binding|nucleus|cytidine to u
lpha 1 ko04974,kc Protein digestion and absorption|Platelet activation|PI3K-A

metalloendopeptidase activity|integral component of plasma membrane|proteolysis
family 7, ko04740 Olfactory transduction
RNA binding|response to bacterium|RNA adenylyltransferase activity
endoplasmic reticulum|calcium ion transport|integral component of endoplasmic r
nucleus|mitochondrial respiratory chain complex IV|response to bacterium|electr

RNA binding|nucleus|nucleolus|cytoplasm|poly(A) binding|nuclear speck|dendrite
condensed chromosome kinetochore|nucleoplasm|cytosol|CENP-A containing nucleoso
1
calcium ion binding|intracellular|mitochondrion|mitochondrial inner membrane|mi
cytosol|identical protein binding|protein-containing complex binding|negative r
Ser-tRNA(Ala) hydrolase activity|nucleic acid binding|alanine-tRNA ligase activ
DNA-binding transcription factor activity, RNA polymerase II-specific|DNA bindi
SNARE binding|phosphatidylserine binding|calcium ion binding|calcium-dependent

mitotic sister chromatid segregation|kinetochore|condensed chromosome kinetocho
RNA polymerase II proximal promoter sequence-specific DNA binding|chromatin bin
nucleoplasm|transcription factor complex|spliceosomal complex|cytosol|transcrip
in utero embryonic development|calcium ion binding|endoplasmic reticulum|endopl
plasma membrane|integral component of membrane
ubiquitin-protein transferase activity|cytosol|ubiquitin-dependent protein cata
ociated 2-like
extracellular region|extracellular space|Golgi lumen|transforming growth factor
cytoplasm|centrosome|spindle organization|protein kinase binding|cell projectio
negative regulation of transcription by RNA polymerase II|RNA methylation|RNA b
regulatior ko04062,kc Chemokine signaling pathway|Dopaminergic synapse
nucleoplasm|endoplasmic reticulum membrane|Golgi apparatus|protein transport|in
P-body|RNA binding|nucleus|cytosol|metal ion binding
sp) box helicase 1
skeletal m ko04068,kc FoxO signaling pathway|Glutamatergic synapse
asma, 1
receptor recycling|early endosome|trans-Golgi network|cytosol|endosome organiza
integral component of membrane
DNA replication|chromatin organization|NuRD complex|negative regulation of cell

DNA-binding transcription factor activity, RNA polymerase II-specific|DNA bindi
GTPase act ko04144 Endocytosis
angiogenesis|negative regulation of endothelial cell proliferation|actin bindir
Golgi membrane|Golgi stack|apoptotic process|integral component of membrane|neg
cytosol|membrane|protein homodimerization activity|extracellular exosome
manchette|calmodulin binding|cytoplasm|spermatid development|sperm axoneme asse
extracellu ko03320 PPAR signaling pathway
structural molecule activity|nucleus|cytoplasm|cytosol|intermediate filament|vi
calcium ion binding|calcium-dependent phospholipid binding|phosphatidylinositol
protein ty ko04144 Endocytosis
integral component of plasma membrane|cell surface receptor signaling pathway|r
Golgi membrane|plasma membrane|integral component of membrane|cytoplasmic vesic
elegans)
Golgi apparatus|spermatogenesis|cell differentiation
mRNA splic ko03040 Spliceosome
histone acetyltransferase complex|transcription coactivator activity|nucleus|nu
nase 11 ko04211,kc Longevity regulating pathway|AMPK signaling pathway|Adipocy

MAML1-RBP-ko04330 Notch signaling pathway
DNA-binding transcription factor activity, RNA polymerase II-specific|DNA bindi
f) ligand ko04060,kc Cytokine-cytokine receptor interaction|Chemokine signaling
Golgi cis ko00534,kc Glycosaminoglycan biosynthesis - heparan sulfate / heparin|

voltage-gated potassium channel activity|potassium channel regulator activity|i
tRNA binding|DNA binding|DNA helicase activity|single-stranded DNA binding|RNA
autophagy
rowth factoko04390 Hippo signaling pathway

p53 bindinko04921,koOxytocin signaling pathway|AMPK signaling pathway
negative regulation of transcription by RNA polymerase II|DNA-binding transcrip
nucleosome|nuclear chromatin|DNA binding|chromatin organization|protein heteroc
regulation of gastrulation|regulation of epithelial to mesenchymal transition|b
calcium ion binding|cytosol|central nervous system development|membrane|calcium
calcium ion binding|extracellular region|transforming growth factor beta activa
r and W2 domains 1
23 ko04914,koProgesterone-mediated oocyte maturation|Oocyte meiosis|Cell
protein phko04910 Insulin signaling pathway
RNA polymerase II proximal promoter sequence-specific DNA binding|chromatin bin
DNA-binding transcription factor activity, RNA polymerase II-specific|nucleic a
neuron migration|extracellular region|extracellular space|plasma membrane|brain
respiratory system process|calcium ion binding|endoplasmic reticulum membrane|m
intracelluko04911 Insulin secretion
ss-complemeko03420 Nucleotide excision repair
microfilament motor activity|signaling receptor binding|calmodulin binding|ATP
se
RNA polymerase II regulatory region sequence-specific DNA binding|DNA-binding t
cytosol|identical protein binding|regulation of intracellular signal transducti
nucleoplasko04360,koAxon guidance|Hedgehog signaling pathway
MAPK cascako04010 MAPK signaling pathway
protein 6 ko04913,koOvarian steroidogenesis|Hippo signaling pathway|TGF-beta si
extracellular region|plasma membrane|integral component of membrane
Golgi membrane|trans-Golgi network|plasma membrane|ion transport|regulation of
sialate O-acetylesterase activity|regulation of immune system process|extracell

patched ligand maturation|determination of left/right symmetry|embryonic patter
ionotropicko04080 Neuroactive ligand-receptor interaction
hyaluronic acid binding|extracellular region|cell adhesion|central nervous syst
cytoplasm|centrosome|microtubule|chemical synaptic transmission|multicellular o
negative regulation of transcription by RNA polymerase II|p53 binding|DNA-bindi
Golgi membko04974 Protein digestion and absorption
renal systko04142,koLysosome|Antigen processing and presentation
-protein coding RNA 702
DNA-binding transcription factor activity, RNA polymerase II-specific|DNA bindi
EKC/KEOPS complex|tRNA threonylcarbamoyladenosine modification|metalloendopepti
nase 4) regulator
integral component of membrane
structural constituent of ribosome|nucleoplasm|mitochondrion|mitochondrial inne
cytosol|plko04150 mTOR signaling pathway
magnesium ion binding|cytosol|nucleotide metabolic process|hydrolase activity|p

DNA damage checkpoint|recombinational repair|nucleus|nucleoplasm|chromosome|cel
endoplasmiko04512 ECM-receptor interaction
endoplasmic reticulum membrane|multicellular organism development|integral comp
-protein coding RNA 1094

collagen binding|collagen trimer|extracellular space|integral component of plas
platelet degranulation|DNA binding|serine-type endopeptidase inhibitor activity
stress fiber|actin binding
Golgi membrane|ubiquitin-protein transferase activity|Golgi stack|trans-Golgi n
cytosol|membrane|protein homodimerization activity|extracellular exosome

alpha-ketc ko00640,kc Propanoate metabolism|Valine, leucine and isoleucine degrad
angiogenes ko04060,kc Cytokine-cytokine receptor interaction|Rap1 signaling pathw
G2/M transition of mitotic cell cycle|structural molecule activity|cytoplasm|in
e protein l ko04530 Tight junction
protein serine/threonine kinase activity|Notch binding|ATP binding|nucleus|cytc

clathrin-coated pit|receptor-mediated endocytosis|integral component of membran
DNA-binding transcription factor activity, RNA polymerase II-specific|endochonc
cytoplasm|ko04013 MAPK signaling pathway - fly
nucleus
RNA polymerase II proximal promoter sequence-specific DNA binding|DNA-binding t
n 3
-protein coding RNA 460
protein serine/threonine kinase activity|ATP binding|protein phosphorylation
3',5'-cycl ko00230 Purine metabolism
RNA polymerase II regulatory region sequence-specific DNA binding|DNA-binding t
MAPK cascade|actin binding|Ras guanyl-nucleotide exchange factor activity|cytop
microtubule bundle formation|neuron migration|cytoplasm|microtubule|nervous sys
regulation of transcription involved in G1/S transition of mitotic cell cycle|r
defense re ko04060 Cytokine-cytokine receptor interaction
autophagy ko04620,kc Toll-like receptor signaling pathway|Lysosome|Osteoclast di
nucleus|nucleoplasm|integral component of membrane
phospholipid transporter activity|endoplasmic reticulum membrane|plasma membran
negative regulation of protein phosphorylation|cytoplasm|energy reserve metabol
rotein kinase 1 interacting protein 1-like
multicellular organism development

DNA-binding transcription factor activity, RNA polymerase II-specific|DNA bindi
angiogenes ko04060 Cytokine-cytokine receptor interaction
ATP bindir ko00280 Valine, leucine and isoleucine degradation
DNA binding|nucleoplasm|cytosol|nuclear body|metal ion binding
endoplasmi ko01040,kc Biosynthesis of unsaturated fatty acids|Fatty acid elongati
angiogenesis|extracellular matrix structural constituent|extracellular region|c
ed 1, pseudogene
syntaxin binding
RNA polymerase II proximal promoter sequence-specific DNA binding|inflammatory
ko00350 Tyrosine metabolism

eading frame 110
RNA bindir ko01212,kc Fatty acid metabolism|Fatty acid elongation|Valine, leucine
kidney development|immune response-activating cell surface receptor signaling p
maturatior ko03008 Ribosome biogenesis in eukaryotes
nase 13
transmembr ko04060,kc Cytokine-cytokine receptor interaction|Apoptosis|Natural ki
nuclear-transcribed mRNA catabolic process, nonsense-mediated decay|RNA binding

tRNA dihydrouridine synthase activity|flavin adenine dinucleotide binding
DNA-binding transcription factor activity, RNA polymerase II-specific|DNA bindi
MAPK casca ko04650,kc Natural killer cell mediated cytotoxicity|Rap1 signaling pa
structural ko03010 Ribosome
semaphorir ko04360 Axon guidance
DNA binding|nucleus|metal ion binding
tenance coi ko04113,kc Meiosis - yeast|Cell cycle - yeast|DNA replication|Cell cyc
tif) ligand 12
cytosol|sensory perception of sound|ubiquitin protein ligase binding
in kinase beta (DMPK-like)
RNA polymerase II proximal promoter sequence-specific DNA binding|chromatin bir
integral component of membrane
neural tuk ko04310 Wnt signaling pathway
RNA binding|actin binding|cytoplasm|cytoskeleton|focal adhesion|cell-substrate
amino acid transmembrane transport|plasma membrane|amino acid transmembrane tra
ko04978,kc Mineral absorption|Longevity regulating pathway - worm
regulation of tolerance induction|nucleus|cytosol|plasma membrane|zinc ion binc
cytosol|zinc ion binding|positive regulation of autophagy|protein ubiquitinatic
p300 ko04066,kc HIF-1 signaling pathway|Adherens junction|Glucagon signalin
GTPase act ko04014 Ras signaling pathway
positive r ko04060,kc Cytokine-cytokine receptor interaction|Jak-STAT signaling p
plasma men ko04514 Cell adhesion molecules (CAMs)
scavenger receptor activity|extracellular matrix structural constituent|immune
3-hydroxya ko00900,kc Terpenoid backbone biosynthesis|Carbon metabolism|Fatty aci
L-asparta ko00710,kc Carbon fixation in photosynthetic organisms|Phenylalanine,
platelet degranulation|phospholipid binding|nucleoplasm|endosome|microtubule or
extracellular exosome
plasma membrane|integral component of membrane|axon|perinuclear region of cytop
liver deve ko00230 Purine metabolism
calcium ion binding|integral component of plasma membrane|cell adhesion|homophi
cytokine activity|extracellular space|chemotaxis|integral component of membrane
phospholipid binding|exocytosis|protein homodimerization activity|protein heter
nucleosomal binding domain 4
myeloid ce ko04330 Notch signaling pathway
RNA-directed DNA polymerase activity|DNA recombination|type II site-specific de
DNA-binding transcription factor activity, RNA polymerase II-specific|DNA bindi
otein ko04210,kc Apoptosis|TNF signaling pathway
intracellular calcium activated chloride channel activity|chloride channel acti
regulatior ko04024,kc cAMP signaling pathway|Adrenergic signaling in cardiomyocyt
ruffle|GTF ko04144,kc Endocytosis|Phagosome|Vasopressin-regulated water reabsorpt
cardiac ve ko04971 Gastric acid secretion
RNA binding|nucleus|mRNA processing|heart development|zinc ion binding|RNA spli
Golgi membrane|cytoplasm|mitochondrion|endoplasmic reticulum|Golgi apparatus|va
negative r ko04550 Signaling pathways regulating pluripotency of stem cells
cytoplasm
serine-type endopeptidase inhibitor activity|extracellular space|cytoplasm|nega
RNA polymerase II core promoter sequence-specific DNA binding|DNA-binding trans
SH3/SH2 adaptor activity|GTPase activator activity|cytosol|membrane|intracellul
nucleoplasm|cytoplasm
intracellular|plasma membrane|cell-cell junction|bicellular tight junction|exoc
ein

integral component of membrane
fumarylaceko00350,kcTyrosine metabolism|Styrene degradation
nucleus|centrosome|cytosol|zonula adherens|cell junction|cell-cell adhesion mec
1
ıase 6 ko04110,kcCell cycle|p53 signaling pathway|PI3K-Akt signaling pathway
cytosol|membrane|protein homodimerization activity|extracellular exosome
positive regulation of I-kappaB kinase/NF-kappaB signaling
or (ligand`ko04060,kcCytokine-cytokine receptor interaction|Intestinal immune ne
amino acicko04150 mTOR signaling pathway
tin filament), gelsolin-like
DNA-binding transcription factor activity, RNA polymerase II-specific|DNA bindi
spindle pole|centrosome|centriole|cytosol|cell junction|apical junction assembl
-protein coding RNA 900
ameboidal-type cell migration|endoplasmic reticulum|Golgi apparatus|sphingolipi
nuclear chromatin|RNA polymerase II regulatory region sequence-specific DNA bir
integral component of membrane

stress fiber|nucleus|cytoplasm|cytoskeleton|protein phosphatase binding|lamelli

GTPase actko04062,kcChemokine signaling pathway|Cholinergic synapse|Serotonergi
1
calcium ion binding|calcium-dependent phospholipid binding|microtubule organizi
integral component of membrane|negative regulation of dendritic cell differenti

nucleus|Golgi apparatus|cytosol|Rab guanyl-nucleotide exchange factor activity|
extracellular space|integral component of membrane
ko04141 Protein processing in endoplasmic reticulum
skeletal sko04974 Protein digestion and absorption
manganese ion transmembrane transporter activity|zinc ion transmembrane transpc
angiogeneko04974,kcProtein digestion and absorption|PI3K-Akt signaling pathway
actin binding|cytoplasm|microtubule organizing center|cytoskeleton|smooth muscl
G2/M transition of mitotic cell cycle|nucleus|nucleolus|cytosol|apoptotic proce

Golgi membrane|lysosomal membrane|Golgi stack|trans-Golgi network|zinc ion binc
RNA polymerase II regulatory region sequence-specific DNA binding|RNA polymeras
neural tubko04310 Wnt signaling pathway
negative regulation of transcription by RNA polymerase II|RNA polymerase II reg
establishment of mitotic spindle orientation|nucleus|nucleolus|cytosol|bicellul
RNA polymerase II proximal promoter sequence-specific DNA binding|chromatin bir
extracellular space|plasma membrane|membrane|integral component of membrane|reg
serine-tyrko04974,kcProtein digestion and absorption|Neuroactive ligand-recepto
extracellular region|plasma membrane|homophilic cell adhesion via plasma membra
glycerol kko03320,kcPPAR signaling pathway|Glycerolipid metabolism|Plant-pathog
nuclear chromatin|RNA polymerase II repressing transcription factor binding|DNA

nuclear chromatin|RNA polymerase II proximal promoter sequence-specific DNA bir
protein serine/threonine kinase activity|ATP binding|nuclear outer membrane|enc
protein polyubiquitination|ubiquitin-protein transferase activity|nucleus|cytos
guanyl-nucleotide exchange factor activity|Rho guanyl-nucleotide exchange factc
negative regulation of transcription by RNA polymerase II|endodermal cell fate
magnesium ko00052,kcGalactose metabolism|Streptomycin biosynthesis|Purine metab

N4-(beta-Nko04142,kcLysosome|Other glycan degradation
integral component of membrane
RNA binding|tRNA (guanine) methyltransferase activity|tRNA methylation
catalytic ko00670 One carbon pool by folate
e, sub-fam ko02010 ABC transporters
actin bincko04810,kcRegulation of actin cytoskeleton|Adherens junction|Fc gamma
transcription, DNA-templated|DRM complex
acrosomal vesicle|MAP-kinase scaffold activity|cytoplasm|microtubule organizing
nucleus|cyko04144 Endocytosis
[pyruvate dehydrogenase (lipoamide)] phosphatase activity|cytoplasm|mitochondri
double-strand break repair via homologous recombination|DNA binding|DNA helicas
endopeptickko04614 Renin-angiotensin system
ko04630 Jak-STAT signaling pathway

tor-bound ko04150 mTOR signaling pathway
ssociated 1, BEN and BTB (POZ) domain containing
cyclin-dependent protein kinase holoenzyme complex|nucleus|cyclin-dependent prc
cytosol|cell-cell adherens junction|cell cycle|protein ubiquitination|cell divi
G1/S transition of mitotic cell cycle|DNA binding|nucleus|cytoplasm|cytosol|pla
endoplasmic reticulum membrane|ergosterol biosynthetic process|integral compone
l
glutathiorko00982,kcDrug metabolism - cytochrome P450|Glutathione metabolism|Me
cytoplasm|immune response|response to virus|response to bacterium
negative regulation of transcription by RNA polymerase II|DNA-binding transcrip
nucleus|nucleoplasm|nucleolus|cytoplasm|rRNA processing|cysteine-type peptidase
DNA-binding transcription factor activity, RNA polymerase II-specific|DNA bindi
-protein coding RNA 1085
cytoplasm|plasma membrane|protein C-terminus binding|regulation of signaling re
bisphosphcko00260,kcGlycine, serine and threonine metabolism|Methane metabolism
guanyl-nucleotide exchange factor activity|Rho guanyl-nucleotide exchange facto
calcium ion binding|extracellular region
regulation of heart rate|actin binding|structural molecule activity|calmodulin
Golgi membrane|MAPK cascade|magnesium ion binding|protein kinase activity|prote
positive rko04150 mTOR signaling pathway
MAPK cascako04014,kcRas signaling pathway|MAPK signaling pathway - fly
double-strand break repair via homologous recombination|chromatin|nuclear chron
enase 1 (Nko01200,kcCarbon metabolism|Peroxisome|Citrate cycle (TCA cycle)|Carb
magnesium ko00230,kcPurine metabolism|Biosynthesis of amino acids|Pentose phosp
GDP-dissociation inhibitor activity|GTPase activator activity|nucleus|nucleopla
d autophagy modulator 1
transmembrane signaling receptor activity|plasma membrane|integral component of

autophagosome assembly|autophagy of mitochondrion|cytosol|protein lipidation|au
rhombotin-like 1)
magnesium ko04914,kcProgesterone-mediated oocyte maturation|Neurotrophin signal
lysosome|eko04144 Endocytosis
GTPase actko04666,kcFc gamma R-mediated phagocytosis|Endocytosis
-neuraminyko00604 Glycosphingolipid biosynthesis - ganglio series

tRNA bindiko00970 Aminoacyl-tRNA biosynthesis
mitochondrion|protein targeting to mitochondrion|anterograde axonal transport|r

protein kinase activity|non-membrane spanning protein tyrosine kinase activity|
endonucleolytic cleavage in ITS1 to separate SSU-rRNA from 5.8S rRNA and LSU-rF
microtubule cytoskeleton organization|magnesium ion binding|neuron migration|ph
DNA binding
\ 1
otor, beta ko04060,kcCytokine-cytokine receptor interaction|Jak-STAT signaling p
phospholipase A2 activity|nuclear envelope lumen|cytoplasm|O-acyltransferase ac
lens morphogenesis in camera-type eye|guanyl-nucleotide exchange factor activit
in utero embryonic development|somitogenesis|neural tube closure|embryonic plac
calcium ion binding|extracellular region
DNA-binding transcription factor activity, RNA polymerase II-specific|DNA bindi
ranscription factor 1
endoplasmic reticulum-Golgi intermediate compartment|plasma membrane|cell adhes
cytosol|membrane|protein homodimerization activity|extracellular exosome
cytoskeleton|muscle organ development|neuromuscular junction development|peptic
lgalactosarko00512 Mucin type O-Glycan biosynthesis
actin binding|nucleus|nucleoplasm|spindle|cytosol|plasma membrane|cell cortex|e
endoplasmiko01040,kcBiosynthesis of unsaturated fatty acids|Fatty acid elongati
cation chako04726,kcSerotonergic synapse|Pancreatic secretion|Axon guidance|Glu
ATP binding|cytoplasm|microtubule organizing center|cytosol|I-kappaB kinase/NF-
ceramide pko04071,kcSphingolipid signaling pathway|Sphingolipid metabolism
hrough (NMIko04080,kcNeuroactive ligand-receptor interaction|Calcium signaling p
nucleus|cytosol|cell cycle|cell division|regulation of cell cycle
extracellular region|cytoplasm
G protein-coupled receptor activity|calcium ion binding|cytoplasm|plasma membra
protein tyrosine phosphatase activity|nucleus|cytoplasm|negative regulation of
actor 3 ko04110 Cell cycle
cytosol|Rab GTPase binding|cytoplasmic vesicle|extracellular exosome
translatico04914,kcProgesterone-mediated oocyte maturation|Oocyte meiosis|Dors
phosphatidylinositol binding
catalytic ko04071,kcSphingolipid signaling pathway|Lysosome|Sphingolipid metabo
osteoblastko04310 Wnt signaling pathway
aldo-keto reductase (NADP) activity|voltage-gated potassium channel activity|cy
skeletal system development|protein kinase activity|non-membrane spanning prote
or, alpha-induced protein 2
proteasomeko03050 Proteasome
DNA binding|nucleus|metal ion binding
single-stranded RNA binding|endoribonuclease activity|mitochondrial matrix|zinc
DNA binding|nucleus|metal ion binding
containing 74A
amino acico04727,kcGABAergic synapse|Protein digestion and absorption|Glutamat
calcium ion binding|collagen binding|glycosaminoglycan binding|extracellular sp
platelet degranulation|actin binding|protein phosphatase inhibitor activity|pla
endopeptidase activity|metalloendopeptidase activity|integral component of plas
adaptive immune response|antigen processing and presentation of exogenous pepti
ase 1
mitochondrion|lipid droplet|regulation of lipid metabolic process|extrinsic apo
nucleus|cytosol|DNA repair|chromatin organization|nucleosome binding
RNA bindirko03040 Spliceosome
serine-type endopeptidase inhibitor activity|extracellular region|extracellular

ossification|extracellular space|extracellular matrix|negative regulation of os
amyloid-beta binding|extracellular matrix structural constituent|extracellular
osteoblast ko00061,kc Fatty acid biosynthesis|Insulin signaling pathway|Fatty aci
ceptor alpha
Golgi membrane|sulfotransferase activity|integral component of membrane|chondrc
mannosyl-c ko00510,kc N-Glycan biosynthesis|Protein processing in endoplasmic ret
calcium ion binding|cytoplasm|endoplasmic reticulum membrane|lipid droplet|micr
negative regulation of transcription by RNA polymerase II|RNA polymerase II prc
exocyst|SN ko04530 Tight junction
VIII, proc ko04610 Complement and coagulation cascades
MAPK casca ko04062,kc Chemokine signaling pathway|Neurotrophin signaling pathway|
ko04011,kc MAPK signaling pathway - yeast|Tryptophan metabolism|Longev
alpha-1,3- ko00510,kc N-Glycan biosynthesis|Various types of N-glycan biosynthesi
mitochondrion|mitochondrial intermembrane space|endosome|Golgi apparatus|trans-
cell differentiation
dase 16 (membrane-inserted)
sulfur ami ko00260,kc Glycine, serine and threonine metabolism|Biosynthesis of am
6-phosphof ko00680,kc Methane metabolism|RNA degradation|Galactose metabolism|Car
retinoid metabolic process|retinoic acid biosynthetic process|retinoid binding|
amyloid-beta binding|neuron migration|nucleus|cytoplasm|regulation of transcrip
ATP bindir ko00970 Aminoacyl-tRNA biosynthesis
Golgi apparatus|plasma membrane|integral component of membrane|regulation of pr
ko04662,kc B cell receptor signaling pathway|T cell receptor signaling
transcription coactivator activity|nucleus|nucleoplasm|nucleolus|beta-catenin b
translation elongation factor activity|nucleus|cytoplasm|endoplasmic reticulum|
cell morphogenesis|ruffle|lymphangiogenesis|amino acid transmembrane transport|
chromosome, centromeric region|condensed nuclear chromosome, centromeric region
2
ko04977 Vitamin digestion and absorption
negative regulation of transcription by RNA polymerase II|RNA polymerase II prc
protein ph ko03015,kc mRNA surveillance pathway|Hippo signaling pathway|Hippo sig
amidase ac ko00970 Aminoacyl-tRNA biosynthesis
actin binding|ATP binding|Arp2/3 protein complex|Arp2/3 complex-mediated actin
protein in ko03013 RNA transport
protein-L-isoaspartate (D-aspartate) O-methyltransferase activity|cytoplasm
e similarity 219, member B
skeletal system development|response to hypoxia|negative regulation of cell-mat
Rho guanyl ko04360 Axon guidance
ptor, alpha ko04630 Jak-STAT signaling pathway
chromosome, centromeric region|nucleolus|zinc ion binding|midbody|metal ion bir
Golgi memb ko00532 Glycosaminoglycan biosynthesis - chondroitin sulfate / derm
1 ko00604,kc Glycosphingolipid biosynthesis - ganglio series|Other glyca
ne kinase, ko04722 Neurotrophin signaling pathway
integral component of membrane
se 3 (endo ko04066,kc HIF-1 signaling pathway|Estrogen signaling pathway|Platelet
negative regulation of transcription by RNA polymerase II|nuclear chromatin|RNA
serine-type endopeptidase inhibitor activity|signaling receptor binding|glycosa
calcium ion binding|extracellular region|endoplasmic reticulum|endoplasmic reti
cation channel activity|plasma membrane|cation transport|mechanosensitive ion c
integral component of membrane

-protein coding RNA 475
ɔsphatase 1ko04214,kcApoptosis - fly|MAPK signaling pathway|MAPK signaling pathw
osteoblast differentiation|signaling receptor binding|frizzled binding|extracel
receptor recycling|endosome membrane|early endosome membrane|transferrin transp
cytoplasm|plasma membrane|G protein-coupled receptor signaling pathway|integral
DNA synthesis involved in DNA repair|DNA strand renaturation|DNA binding|ATP bi
synaptonemal complex|meiotic gene conversion|synapsis|reciprocal meiotic recomb
endoplasmic reticulum membrane|plasma membrane|integral component of membrane
MAPK cascako04152,kcAMPK signaling pathway|mTOR signaling pathway
ɔeta and oıko04060,kcCytokine-cytokine receptor interaction|Toll-like receptor s
T cell medko04650   Natural killer cell mediated cytotoxicity
RNA polymerase I core binding|RNA polymerase I CORE element sequence-specific D
in containing 1
stress fiber|actin binding|calmodulin binding|extracellular region|cytoskeleton
immunologiko04662,kcB cell receptor signaling pathway|T cell receptor signaling

protein kinase inhibitor activity|cytoplasm|intracellular signal transduction|r
calcium ion binding|plasma membrane|cell-cell adherens junction|cell-cell junct
cell|cell volume homeostasis|potassium ion import|potassium:chloride symporter
negative regulation of transcription by RNA polymerase II|RNA polymerase II dis
RNA polymeko04010,kcMAPK signaling pathway|Oxytocin signaling pathway|Axon guid
lens morphko04514,kcCell adhesion molecules (CAMs)|Adherens junction
Rab GDP-dissociation inhibitor activity|GTPase activator activity|Golgi apparat

ɔtein-1 (ubiquitin carboxy-terminal hydrolase)

extracellular region|integral component of membrane|cytoplasmic vesicle membran
Golgi apparatus|cytoskeleton|plasma membrane|integral component of plasma membr
DNA-binding transcription factor activity, RNA polymerase II-specific|DNA bindi
ferric-chelate reductase activity|lysosomal membrane|integral component of memb
protein ubiquitination|protein maturation by iron-sulfur cluster transfer|negat
zinc ion binding|Cul2-RING ubiquitin ligase complex|regulation of axon guidance
negative rko04550,kcSignaling pathways regulating pluripotency of stem cells|Hi
actin binding|structural molecule activity|intracellular|cytoplasm|cytoskeleton
cytosol|cell cycle|midbody|cell division
GTPase activator activity|cytosol|intracellular protein transport|Rab GTPase bi
plasma memko04974,kcProtein digestion and absorption|Bile secretion|Mineral abs
DNA binding|nucleus|Golgi apparatus|regulation of transcription, DNA-templated|
amyloid-beko04970   Salivary secretion
e similarity 43, member A
eading frame 48
cytoplasm|cytosol|endosomal vesicle fusion|intracellular membrane-bounded organ
structuralko03010   Ribosome
region

integral component of membrane|extracellular exosome

MAPK cascako04066,kcHIF-1 signaling pathway|Axon guidance|Glucagon signaling pa
actin binding|cytosol|focal adhesion|actin cytoskeleton|cadherin binding
zinc ion binding|cytoplasmic stress granule|stress granule disassembly|proteasc
positive regulation of protein phosphorylation|extracellular space|cell|endopla

positive ıko04144   Endocytosis
ko04145,kcPhagosome|Rap1 signaling pathway|p53 signaling pathway|TGF-
cytoplasm|Cul3-RING ubiquitin ligase complex|proteasome-mediated ubiquitin-depe
autophagy of mitochondrion|response to hypoxia|mitochondrial outer membrane|res
ruffle|actin binding|guanyl-nucleotide exchange factor activity|Rho guanyl-nucl
ı-regulated in cancer 1-like
through (NMD candidate)
Golgi memŀko04060,kcCytokine-cytokine receptor interaction|MAPK signaling pathw
cytoplasmic pattern recognition receptor signaling pathway|cytoplasm|I-kappaB k
hydrolase activity, hydrolyzing O-glycosyl compounds|mitochondrion|mitochondria
calcium-dependent cysteine-type endopeptidase activity|cytoplasm|focal adhesion
RNA-directed DNA polymerase activity|DNA recombination|type II site-specific de
osine kina:ko04066,kcHIF-1 signaling pathway|Adherens junction|Calcium signaling
protein polyubiquitination|DNA double-strand break processing|nucleus|nucleopla
nucleic acid binding|nucleus|cytoplasm|plasma membrane|positive regulation of g
3, catalyko04924,kcRenin secretion|Axon guidance|Glucagon signaling pathway|T
integral component of membrane|identical protein binding
centriole
cilium movement|mitochondrion|axoneme|motile cilium
magnesium ion binding|voltage-gated calcium channel activity|calcium ion bindir
MAPK cascako04923,kcRegulation of lipolysis in adipocytes|Adipocytokine signali
DNA-binding transcription factor activity, RNA polymerase II-specific|DNA bindi
magnesium ion binding|UTP binding|RNA binding|polynucleotide adenylyltransferas
kidney development|chondrocyte development|glomerular filtration|arylsulfatase
activatiorko04620,kcToll-like receptor signaling pathway|Neurotrophin signaling
catalytic activity|plasma membrane
-protein coding RNA 839
Rho GDP-dissociation inhibitor activity|GTPase activator activity|cytosol|cytos
ıor protein 2
ın blood gıko04640,kcHematopoietic cell lineage|ECM-receptor interaction
positive regulation of base-excision repair
cytosol|cilium|nervous system development|lipid binding|ciliary transition zone
endoplasmic reticulum|cytosol|plasma membrane|cell surface|water channel activi
DNA-binding transcription factor activity, RNA polymerase II-specific|DNA bindi

actin binding|tropomyosin binding|cytosol|striated muscle thin filament|actin f
intracellular|zinc ion binding
RNA binding
Golgi memŀko00514   Other types of O-glycan biosynthesis
collagen binding|extracellular region|heparin binding|peptidase activator activ
cytoplasm|outer dynein arm assembly
e B   ko00860,kcPorphyrin and chlorophyll metabolism|Riboflavin metabolism
ko04215,kcApoptosis - multiple species|Apoptosis|Jak-STAT signaling p
oeta 1,3-gako00601   Glycosphingolipid biosynthesis - lacto and neolacto series
3-hydroxyŀko00650,kcButanoate metabolism|Synthesis and degradation of ketone bo
e 3, extracellular
oocyte matko04144   Endocytosis
nucleoplasm|cytosol
photoreceptor outer segment|photoreceptor inner segment|NAD+ kinase activity|nu
nucleus
ko04810,kcRegulation of actin cytoskeleton|PI3K-Akt signaling pathway

protein pcko04120,kcUbiquitin mediated proteolysis|Protein processing in endopl
Rho guanyl-nucleotide exchange factor activity|cytosol|cytoskeleton|synapse ass

DNA-bindirko00310 Lysine degradation
tisense loıko04215,kcApoptosis - multiple species|Apoptosis|Jak-STAT signaling p
rotein kinako04620,kcToll-like receptor signaling pathway|Apoptosis - fly|MAPK s
se 2
phosphatidylinositol-4,5-bisphosphate binding|phosphatidylinositol-5-phosphate
endochondral ossification|transcription corepressor activity|nucleus|regulation
protein phosphatase inhibitor activity|nucleolus|negative regulation of phospha
e, sub-famko04976,kcBile secretion|cAMP signaling pathway|ABC transporters
actor 5 ko04010,kcMAPK signaling pathway|Rap1 signaling pathway|Regulation of
integral component of membrane
RNA-directko04915,kcEstrogen signaling pathway|Cholinergic synapse|Melanogenesi
DNA-binding transcription factor activity, RNA polymerase II-specific|protease
nucleus|cytoplasm|apoptotic process|cell cycle arrest|regulation of DNA stabili
or receptoko04060 Cytokine-cytokine receptor interaction
early endosome|plasma membrane|magnesium ion transmembrane transporter activity
endoplasmic reticulum
e, sub-famko02010 ABC transporters
nucleotide binding|single-stranded DNA binding|single-stranded RNA binding|nucl
G protein-ko04924,kcRenin secretion|Gap junction|Leukocyte transendothelial mig
endosome|response to bacterium
endothelial cell activation involved in immune response|Notch binding|intracell
epeat family member 8
Golgi membrane|Golgi apparatus|peptidyl-tyrosine sulfation|inflammatory respons
transporteko04013 MAPK signaling pathway - fly
ossificatiko04060,kcCytokine-cytokine receptor interaction|NF-kappa B signaling
RNA binding|nucleoplasm|cytoplasm|transcription by RNA polymerase II|transcript
isease 1 (autosomal dominant) pseudogene 6
adaptive immune response|plasma membrane
action potko04924,kcRenin secretion|Gap junction|Glucagon signaling pathway|Rap
nd activatko04630 Jak-STAT signaling pathway
ko04360 Axon guidance
cytoplasm|ko04974 Protein digestion and absorption
blood vessel remodeling|extracellular matrix structural constituent|glycosaminc
extracellular space|insulin receptor signaling pathway|regulation of autophagy|
DNA binding|nucleus|metal ion binding
nily 1E, eko00140 Steroid hormone biosynthesis
ruffle|motor activity|actin binding|calmodulin binding|ATP binding|nucleus|nucl
ctivator 1ko04744 Phototransduction
olymerase ko04214,kcApoptosis - fly|Longevity regulating pathway - worm|Apoptos
metalloendopeptidase activity|extracellular region|integral component of membra

cytoplasm

cell activation|protease binding|connective tissue replacement involved in infl
Golgi membrane|GTPase activator activity|Golgi apparatus|cytosol|plasma membran
RNA bindirko03013 RNA transport
Golgi membrane|cytosol|Rho GTPase binding|protein phosphatase binding|regulatic

nembrane|cilium|small GTPase binding|ciliary basal body|protein dimerization act
le hormone processing|integral component of Golgi membrane
K signaling pathway - fly
cell adhesion|membrane|cytokine-mediated signaling pathway

asm|cytoplasm|intermediate filament|plasma membrane|cell-cell junction|adherens

cycle
llipodium|positive regulation of cell migration|negative regulation of transfor
cadian entrainment|Salivary secretion|Purine metabolism|Long-term depression|Pl
tein catabolic process|cellular response to DNA damage stimulus|postsynaptic de

nding|nucleus|nuclear envelope|nucleoplasm|mRNA processing|mRNA export from nuc
|membrane|Wnt signaling pathway|Wnt-protein binding|negative regulation of Wnt
sm|Pyruvate metabolism

y|Ubiquitin mediated proteolysis|T cell receptor signaling pathway

tochore assembly
ystem|protein kinase binding

by cytochrome P450|Steroid hormone biosynthesis|Linoleic acid metabolism|Arach

component of membrane

ity|early endosome membrane|phosphatidylinositol-3-phosphate binding|negative r
ng|nucleus|methyltransferase activity|regulation of gene expression|metal ion b
|autophagosome|actin filament|intracellular protein transport|vesicle docking i
process|lamellipodium|negative regulation of apoptotic process|negative regula
dhesion

racellular membrane-bounded organelle|cell division

g
ule|endosome organization|lysosome organization|microtubule binding|endosome to
Isp90 protein binding|chaperone-mediated protein folding|regulation of cellular
ation of proteasomal ubiquitin-dependent protein catabolic process|cellular res

protein-coupled receptor signaling pathway|negative regulation of signal trans
lcium signaling pathway|Taste transduction

ınscription factor binding|fibrillar center|p53 binding|DNA binding|transcriptic
sm|Valine, leucine and isoleucine degradation|Carbon metabolism|Carbon fixation
n|Leukocyte transendothelial migration|Rap1 signaling pathway|Focal adhesion
otion factor activity, RNA polymerase II-specific|DNA binding|DNA-binding transc
on paired donors, with incorporation or reduction of molecular oxygen, 2-oxoglu

gral component of plasma membrane|focal adhesion|transmembrane receptor protein

logous recombination|Nucleotide excision repair
oximal promoter sequence-specific DNA binding|DNA-binding transcription factor a
TNF signaling pathway|Retrograde endocannabinoid signaling|Arachidonic acid met
tion|microtubule cytoskeleton organization|pericentriolar material|spindle pole|
ay

ondrion|neuron migration|RNA binding|protein serine/threonine kinase activity|A
ort|positive regulation of neuron projection development|aminophospholipid tran

g receptor activity|collagen binding|ATP binding|cytoplasm|integral component of
egative regulation of blood pressure|proteolysis involved in cellular protein c
membrane|platelet alpha granule lumen
-kappaB signaling|negative regulation of phosphatidylinositol 3-kinase signaling
ulin receptor|plasma membrane|immune response|integral component of membrane|Ig
oximal promoter sequence-specific DNA binding|DNA-binding transcription factor a
asma membrane|phagocytosis|positive regulation of endothelial cell migration|me

actin cytoskeleton|Ras signaling pathway|PI3K-Akt signaling pathway
e|signal transduction|locomotory behavior|negative regulation of protein ubiquit
a membrane|receptor guanylyl cyclase signaling pathway|G protein-coupled recept

orso-ventral axis formation

rbohydrate digestion and absorption|Protein digestion and absorption|Pancreatic
ation|nuclear speck|phosphatase regulator activity|regulation of endocytosis|re
t|negative regulation of neuron projection development|hydrolase activity, acti
culum membrane|plasma membrane|external side of plasma membrane|cell surface|en
hemical synaptic transmission|nervous system development|locomotory behavior|sy

osphatidylinositol transporter activity|phospholipid transport|regulation of li
way - fly
gulating pathway|Longevity regulating pathway - worm|Regulation of mitophagy -
oter DNA-binding transcription activator activity, RNA polymerase II-specific|u

ding|protein transporter activity|signaling receptor activity|identical protein
membrane|structural constituent of myelin sheath|myelination

oximal promoter sequence-specific DNA binding|DNA-binding transcription factor a

r exploration behavior
DNA-templated|mRNA processing|signal transduction|RNA splicing|nuclear speck|B c

eukin-18-mediated signaling pathway|interleukin-18 binding|T-helper 1 type immun
thway|Jak-STAT signaling pathway|Osteoclast differentiation|Thyroid hormone sig
metal ion binding|ubiquitin protein ligase activity|rescue of stalled ribosome
ng|nucleus|metal ion binding
ry valve morphogenesis|ventricular septum development|plasma membrane|integral c

ng|nucleus|metal ion binding

guidance|Rap1 signaling pathway|Sphingolipid signaling pathway|Natural killer
F-kappa B signaling pathway

atrix structural constituent|extracellular region|extracellular space|endoplasmi
ng|DNA-binding transcription factor activity|nucleus|regulation of transcriptio

anchored component of membrane|synapse
e biosynthesis

signaling pathway

on|peptidyl-lysine hydroxylation|procollagen-proline 3-dioxygenase activity|L-as

embrane|bicellular tight junction|cytoskeletal protein binding|positive regulati

athway - fly

logous recombination|Nucleotide excision repair
nent of endoplasmic reticulum membrane|UDP-xylosyltransferase activity
GTP binding|extracellular region|translational elongation|platelet alpha granule

transcription factor activity, RNA polymerase II-specific|proximal promoter DNA-
ng|nucleus|neuron differentiation|positive regulation of cell growth|cellular r
pH|sodium:proton antiporter activity|potassium:proton antiporter activity|integ

axonemal outer doublet|regulation of microtubule motor activity
Carbon metabolism|Biosynthesis of amino acids|Glycolysis / Gluconeogenesis
thway|Phosphatidylinositol signaling system|Glycerolipid metabolism

: protein tyrosine kinase activity|transmembrane signaling receptor activity|ATI

integral component of membrane|digestive tract development

ear speck|protein N-terminus binding|C2H2 zinc finger domain binding|U2-type cat

gulatory region sequence-specific DNA binding|proximal promoter DNA-binding trar
ng glycosyl groups
LSU-rRNA)|RNA binding|nucleus|nucleoplasm|nucleolus|centrosome|cytosol|rRNA prc
asma membrane|cation transport|mechanosensitive ion channel activity|integral c

;|extracellular region|nucleus|nucleoplasm|nucleolus|cytoplasm|mitochondrion|prc
Longevity regulating pathway|AMPK signaling pathway

ndoplasmic reticulum|endoplasmic reticulum membrane|plasma membrane|regulation c

nponent of plasma membrane|adenylate cyclase-inhibiting G protein-coupled recept

ne|protein-containing complex|response to interferon-gamma|response to interferc
signaling pathway -multiple species|Hedgehog signaling pathway - fly|Hippo sign
:ytosis|actin filament organization|establishment or maintenance of cell polarit
signaling pathway
eoxyribonuclease activity|transposition, RNA-mediated|reverse transcription invc
rane|regulation of ryanodine-sensitive calcium-release channel activity
erization activity
on|mitogen-activated protein kinase binding

Cell cycle|Hedgehog signaling pathway - fly|Hedgehog signaling pathway|Ubiquiti

fly|TGF-beta signaling pathway|Endocytosis|Hedgehog signaling pathway
nent|spermatogenesis|zinc ion binding|PcG protein complex|PRC1 complex|identical
B signaling pathway
fungus

o-ventral axis formation
binding|response to yeast|extracellular region|extracellular space|inflammator

gulatory region sequence-specific DNA binding|DNA-binding transcription factor a
eplication-independent nucleosome assembly|protein import into nucleus|NLS-bear
n binding|negative regulation of axon extension|axon initial segment|main axon|
ament|substantia nigra development|cell differentiation|nuclear membrane|cytopl
naptic vesicle priming|cell junction|cytoplasmic vesicle membrane|presynaptic me
some|Cell adhesion molecules (CAMs)|Antigen processing and presentation

Pase binding|activation of GTPase activity

TCA cycle)

ytoplasm|protein phosphorylation|activation of JUN kinase activity|MAP kinase ki

se ion|protein homodimerization activity|protein kinase C signaling|response to

athway

nergic signaling in cardiomyocytes|Aldosterone synthesis and secretion|Insulin
ty|ATP binding|intracellular|integral component of plasma membrane|protein phos
rotein transport|integral component of membrane|cell migration|growth factor bir
n binding|oligosaccharide catabolic process

merization
way|Calcium signaling pathway|Oxytocin signaling pathway
dhesion

reticulum|Protein export
response|integral component of membrane|MHC class I protein complex

asma membrane|endosome membrane|integral component of membrane|intracellular mem
ponent of plasma membrane|heterophilic cell-cell adhesion via plasma membrane ce

acid binding|transcription corepressor activity|nucleus|regulation of gene expre
some|Cell adhesion molecules (CAMs)|Antigen processing and presentation

binding|response to hypoxia|single-stranded DNA binding|single-stranded RNA binc

es|Oxytocin signaling pathway|Cardiac muscle contraction
vity|transmembrane receptor protein tyrosine phosphatase activity|plasma membra
|ECM-receptor interaction|Focal adhesion

oximal promoter sequence-specific DNA binding|proximal promoter DNA-binding trar
ng|perikaryon|histone H3 acetylation|metal ion binding|response to electrical st
ouridine synthase activity|positive regulation of mitochondrial translation
interaction
g pathway|Neuroactive ligand-receptor interaction
ation|cytosol|multicellular organism development|nuclear speck|sequence-specifi
ng|extracellular region|nucleus|endoplasmic reticulum membrane|cell adhesion|hc

ration|Axon guidance|GABAergic synapse|Rap1 signaling pathway|Long-term depress

ıl component of membrane
: tethering to Golgi

ɔud development|organ induction|nucleoplasm|Golgi apparatus|cytosol|plasma membı

ɔtide exchange factor activity|TRAPP complex|early endosome to Golgi transport|(

:tivity|DNA recombination|zinc ion binding|DNA integration
:ivity|metallopeptidase activity|dipeptidyl-peptidase activity|cellular amino ac
iation
ıigration|Tight junction
ercalated disc|integral component of membrane|regulation of cardiac conduction

.ty|nucleus|nucleoplasm|cytoplasm|cytosol|regulation of transcription by RNA pol
 in vesicular transport
ıicrotubule-based movement|dynein complex|intraciliary transport involved in cil
sm|regulation of transcription, DNA-templated|ubiquitin-dependent protein catabc
oactive ligand-receptor interaction|Taste transduction
ase II|vasculogenesis|chromatin binding|transcription corepressor activity|prot
TP binding|cytoplasm|cytosol|plasma membrane|focal adhesion|cell cortex|endocyt

se|Adherens junction|Fc epsilon RI signaling pathway|Axon guidance|Osteoclast d

ose metabolism

ıg|nucleoplasm|cytoplasm|cytosol|cytoskeleton|protein phosphorylation|regulatior
 apparatus|cytosol|immune response|actin cytoskeleton|protein localization to nu

: monoubiquitination|ubiquitin protein ligase binding|HULC complex|histone H2B ι
g pathway|Longevity regulating pathway - multiple species
ɔmponent of plasma membrane|integral component of membrane|hyaluronan catabolic

ır space|growth factor activity|positive regulation of pathway-restricted SMAD ŗ

: activity|Rho guanyl-nucleotide exchange factor activity|GTPase activator acti
ransduction|cell differentiation|regulation of small GTPase mediated signal trar
ɔr activity|cytosol|G protein-coupled receptor signaling pathway|Rho GTPase binc

ng pathway|Neuroactive ligand-receptor interaction|PI3K-Akt signaling pathway

ıs recombination|chromosome, telomeric region|sex chromosome|ATP binding|nucleus
rganism development

.ow-density lipoprotein particle receptor activity|extracellular space|nuclear e
eceptor activity|G protein-coupled receptor activity|intracellular|plasma membra
ros development|nucleus|cytoplasm|mesoderm development|axon|sequence-specific DN

nucleotide sugar metabolism
ranscription factor activity, RNA polymerase II-specific|proximal promoter DNA-

ı lumen|endoplasmic reticulum membrane|endoplasmic reticulum-Golgi intermediate

ansport|spermatogenesis|endosome to lysosome transport|peptidyl-lysine hydroxyl
tion development|Rab guanyl-nucleotide exchange factor activity|retrograde tran

ostsynaptic density|actin cytoskeleton|Z disc|positive regulation of actin filam

ate digestion and absorption|Neomycin, kanamycin and gentamicin biosynthesis|Ga

ım membrane|transmembrane receptor protein serine/threonine kinase signaling pat
ı via MHC class I|endopeptidase activity|aminopeptidase activity|cytoplasm|endop
ent of membrane|mitochondrial respiratory chain complex I assembly
Proximal tubule bicarbonate reclamation|Carbon metabolism|Alanine, aspartate an
entromeric region|condensed nuclear chromosome kinetochore|condensed nuclear chr

el activity|plasma membrane|integral component of plasma membrane|signal transdu

ıondrial inner membrane|mitochondrial transport|regulation of cell death|ATP tra
oithelial cell differentiation|extracellular exosome
n|Fc epsilon RI signaling pathway|Sphingolipid signaling pathway|Fc gamma R-med

idase activity involved in apoptotic process|DNA damage response, signal transc
cycle|Ubiquitin mediated proteolysis|Cell cycle - yeast|Meiosis - yeast
otion factor activity, RNA polymerase II-specific|DNA binding|DNA-binding transc
terone synthesis and secretion|Calcium signaling pathway|Insulin secretion|GnRH
of transcription, DNA-templated|spermatogenesis
n|cytoplasm|cytosol|cytoskeleton|cell-cell adherens junction|double-strand brea
ransport|Rab GTPase binding|activation of GTPase activity

origin of replication recognition complex|pericentric heterochromatin|nucleolus

essor activity|nucleus|nucleoplasm|cytoplasm|zinc ion binding|regulation of carc

ive regulation of enamel mineralization

brane|response to endoplasmic reticulum stress|negative regulation of neuron dea
eoxyribonuclease activity|transposition, RNA-mediated|reverse transcription invo
y|spindle pole body organization

ve regulation of heat generation|beta-3 adrenergic receptor binding|skin develo
xenobiotic metabolic process|positive regulation of triglyceride catabolic proc
to mitochondrion|mitochondrial processing peptidase complex|metal ion binding

ic acid metabolism|Taurine and hypotaurine metabolism
ay|Hippo signaling pathway - fly|Oocyte meiosis|Cell cycle|Hippo signaling path

crotubule|plasma membrane|retrograde vesicle-mediated transport, Golgi to ER|mi
zinc ion binding|xenobiotic catabolic process|protein homotetramerization|extra

ng|protein-containing complex scaffold activity|negative regulation of hippo sig

plasma membrane|multicellular organism growth|odontogenesis of dentin-containing
ell projection organization|desmosome|negative regulation of cell growth|keratin

etabolism|Lysine degradation|Pentose and glucuronate interconversions|beta-Alan
oplasm|plasma membrane|proteolysis|spermatogenesis|serine-type peptidase activi

nsport|protein transport|protein ubiquitination|cytoplasmic vesicle|ubiquitin p

ranscription factor activity, RNA polymerase II-specific|proximal promoter DNA-
g system

l component of membrane|TRAIL-activated apoptotic signaling pathway|regulation o
vity regulating pathway|Ovarian steroidogenesis|Platelet activation|Progesteron

ly|transcription coactivator activity|structural molecule activity|structural o
3K-Akt signaling pathway|Jak-STAT signaling pathway|FoxO signaling pathway|Oxyt
tochondrial membrane|mitochondrial proton-transporting ATP synthase complex ass
a-receptor activity|extracellular region
lic cell adhesion via plasma membrane adhesion molecules|brain development
ion of receptor recycling|positive regulation of protein phosphorylation|protea

ırin vesicle coat|membrane organization

›plasm|proteolysis
ıg|microtubule cytoskeleton|septin complex|septin filament array|protein-contair
.ng|DNA-binding transcription factor activity|nucleus|regulation of transcriptic
metabolism|Peroxisome

.on|interstitial matrix|extracellular space|heparin binding|positive regulation
› regulation of plasminogen activation

:oplasm|endoplasmic reticulum membrane|octopamine biosynthetic process|integral
;ulatory region sequence-specific DNA binding|DNA-binding transcription factor ε
ng pathway|Longevity regulating pathway|Ovarian steroidogenesis|Platelet activa
ınscription factor binding|DNA binding|transcription corepressor activity|RNA bi
Glycosaminoglycan biosynthesis - chondroitin sulfate / dermatan sulfate

ıse activity|GTP binding|nucleus|cytoplasm|lysosomal membrane|Golgi apparatus|pl
system|protein kinase binding
ıtegral component of membrane|cerebellum development|cerebellar Purkinje cell lε
›oplasm|endoplasmic reticulum|cytosol|regulation of transcription, DNA-templatec

· pathway

:ional protein modification
sm

ıg|microtubule cytoskeleton|septin complex|septin filament array|protein-contair

·eticulum membrane|regulation of store-operated calcium entry
translation|DNA damage response, signal transduction by p53 class mediator resι
.on|apoptotic process|negative regulation of cell proliferation|negative regulat

.sc|cell junction|positive regulation of actin filament bundle assembly

'K signaling pathway|Insulin signaling pathway|FoxO signaling pathway|Thyroid ho

:ulum|endoplasmic reticulum membrane|Golgi apparatus|endoplasmic reticulum orgar

:ytoskeleton organization|nuclear migration|regulation of cell shape|membrane|ir
ıg

: density|membrane|nuclear matrix|cell junction|dendrite|cortical cytoskeleton|r
it junction|cytoskeletal protein binding|negative regulation of keratinocyte pro
t|Hippo signaling pathway|Hippo signaling pathway - fly

.crotubule binding|positive regulation of cell-cell adhesion|establishment of ce
: promoter binding|RNA binding|double-stranded RNA binding|single-stranded RNA b

.lpha-Linolenic acid metabolism|MAPK signaling pathway|Ovarian steroidogenesis|S
eceptor interaction|PI3K-Akt signaling pathway|Jak-STAT signaling pathway|Prola
ganization|protein transporter activity|protein transport|lung development|Hsp7(
c synapse|Retrograde endocannabinoid signaling|GABAergic synapse|Phototransduct
checkpoint|response to ionizing radiation|SOSS complex

.ng|postsynaptic density|nuclear matrix|protein-containing complex|negative regu
otion factor activity, RNA polymerase II-specific|proximal promoter DNA-binding

to Golgi transport|phosphatidylinositol binding|identical protein binding|intra

:eptor binding|post-translational protein modification|negative regulation of ir

ıatan sulfate

:ane
:oid hormone signaling pathway

.ng|nucleoplasm|metal ion binding

ism|Carbon metabolism|Biosynthesis of amino acids|Pentose phosphate pathway|Fru

ement and coagulation cascades

: cell cycle|cyclin-dependent protein kinase holoenzyme complex|nucleus|cytoplas
ent protein catabolic process|ubiquitin conjugating enzyme binding|positive regu

aling pathway|Adipocytokine signaling pathway|PPAR signaling pathway|Fatty acid
.ex assembly|positive regulation of apoptotic process|unfolded protein binding|p
|ECM-receptor interaction|Focal adhesion

cell growth|positive regulation of proteasomal ubiquitin-dependent protein cata

r region|cytosol|membrane|metal ion binding|extracellular exosome
n domain specific binding|negative regulation of viral transcription|innate immu
ne
regulation of protein phosphorylation|nucleic acid binding|nucleus|cytoplasm|re

omponent of membrane|peptidyl-L-cysteine S-palmitoylation|protein-cysteine S-pal

y|Hippo signaling pathway|Wnt signaling pathway|Signaling pathways regulating p
tion|Geraniol degradation|Caprolactam degradation|Fatty acid elongation|Valine,
or activity|GTPase activator activity|cytosol|actin cytoskeleton organization|SH

ion|membrane|Rab GTPase binding|melanocyte differentiation|GTP-dependent protein
al component of plasma membrane|cell surface receptor signaling pathway|G protei

rotein complex assembly|integral component of membrane|negative regulation of tr

tion|GnRH signaling pathway

c anion transmembrane transporter activity|thyroid hormone transmembrane transp

ng center|cell cortex|neuron projection development|negative regulation of tubul

athway

n|Salivary secretion

r region of cytoplasm
sion|Rap1 signaling pathway

ated disc|membrane|protein domain specific binding|bleb|identical protein bindi
vity|membrane|neutrophil degranulation|tertiary granule lumen

ge factor activity|cytoplasm|early endosome|endoplasmic reticulum|Golgi apparatu
ng|hormone activity|extracellular matrix structural constituent|calcium ion bind

racellular component
led methylated DNA binding

g-term depression|Platelet activation|Dopaminergic synapse|Salivary secretion|A
)NA-templated|zinc ion binding

ng|nucleoplasm|regulation of apoptotic process|metal ion binding
it of membrane|Wnt signaling pathway|negative regulation of ossification|limb de
t|Hippo signaling pathway|Hippo signaling pathway - fly

promoter DNA-binding transcription activator activity, RNA polymerase II-specif
ng transcription repressor activity, RNA polymerase II-specific|fibrillar cente
nucleus|cytoplasm|cytosol|DNA recombination|identical protein binding|sequence-
on activity|E-box binding
membrane|palmitoyltransferase activity|protein palmitoylation|protein-cysteine
sma membrane|membrane protein ectodomain proteolysis|positive regulation of gene
osol|striated muscle thin filament|muscle contraction|cytoskeleton organization|

tory region sequence-specific DNA binding|RNA polymerase II proximal promoter se
ellular region|collagen trimer|extracellular space|endoplasmic reticulum lumen|c
ng pathway|Regulation of actin cytoskeleton|Ras signaling pathway|PI3K-Akt sign
rimidine nucleotide transport|mitochondrion organization|pyrimidine nucleotide t
n ADP-ribosylation|negative regulation of gene expression|regulation of chromat

rane|filopodium|dendrite|growth cone|neuron projection development|neuronal cell

nding|intraciliary transport particle B|motile cilium|intraciliary transport inv

plasm|membrane|integral component of membrane|nuclear membrane

differentiation|integral component of membrane
g|nuclear envelope|integral component of nuclear inner membrane|nuclear envelope
focal adhesion|apical plasma membrane|nuclear body|lamellipodium|cell junction|c
|ECM-receptor interaction|Focal adhesion
ition|keratin filament|intermediate filament cytoskeleton|cornification
tubule|plasma membrane|cell cycle|integrin-mediated signaling pathway|microtubul
ers

led RNA binding|cytoplasm|endosome|early endosome|plasma membrane|protein transp
T cell receptor signaling pathway|MAPK signaling pathway
iary transition zone|cilium assembly
olic process|ubiquitin protein ligase activity
ronucleus|male pronucleus|DNA binding|iron ion binding|nucleus|chromosome|cytopl

ıne|cell-cell adherens junction|cell cortex|cell cycle|homophilic cell adhesion

|Longevity regulating pathway - worm|TNF signaling pathway|Osteoclast different
.lular space|nucleus|nucleolus|cytosol|plasma membrane|locomotory behavior|cell
phagosome|cellular calcium ion homeostasis|cellular iron ion homeostasis|cellula

transendothelial migration|Focal adhesion|Regulation of actin cytoskeleton|Tig

protein deubiquitination
.ng|cytosol|male meiosis I|multicellular organism development|germ cell developm

ı-Golgi vesicle-mediated transport|Golgi organization|protein transporter activi

ı|Glucagon signaling pathway|Carbon metabolism|Biosynthesis of amino acids|Glyco
ling, bridging|establishment of epithelial cell polarity
.ng|nucleus|metal ion binding
orane|calcium ion homeostasis
junction|sarcolemma|intermediate filament-based process|perinuclear region of

sponse to hypoxia|startle response|aggressive behavior|neuropeptide hormone acti
ylation|Synaptic vesicle cycle|mTOR signaling pathway
region|nucleus|nucleolus|plasma membrane|cilium|axoneme|regulation of transcri

.ing pathway|retina layer formation|Wnt signaling pathway|regulation of angiogen
otion factor activity, RNA polymerase II-specific|neural tube closure|lens morpl
orter activity|symporter activity|monocarboxylic acid transport|membrane
ı
.s|cytoskeleton organization|endoderm development|learning|long-term memory|ante
:ubule binding|stress fiber assembly|cilium assembly|Golgi ribbon formation

otor activity|plasma membrane|G protein-coupled receptor signaling pathway|adeny

.ral process|keratinization|keratin filament|extracellular exosome|cornificatio
ay|Insulin secretion

athway|Hematopoietic cell lineage|FoxO signaling pathway|PI3K-Akt signaling pat

:ytosis|negative regulation of cell proliferation|positive regulation of autopha
eoxyribonuclease activity|transposition, RNA-mediated|reverse transcription invo
esis
ion|Oocyte meiosis|Cell cycle
limerization activity

:ed signal transduction|regulation of cell shape|nuclear speck|regulation of act
it of membrane

lynein complex|microtubule|cell cycle|regulation of mitotic cell cycle|identical
oxidation-reduction process|tRNA-4-demethylwyosine synthase activity

hway|p53 signaling pathway|MAPK signaling pathway|Apoptosis - multiple species|
ı membrane adhesion molecules|Notch signaling pathway|axon guidance|central nerv

|Selenocompound metabolism

of membrane|Wnt signaling pathway|PDZ domain binding|protein stabilization|prote

:ion of cell migration|regulation of Rho protein signal transduction

cortex|actin filament organization|establishment or maintenance of cell polarit
nicrotubule|cilium|retrograde vesicle-mediated transport, Golgi to ER|microtubul
egral component of plasma membrane|carbohydrate metabolic process|integral compo
regulation of fat cell differentiation|metal ion binding
ı|GABAergic synapse|Two-component system|Biosynthesis of amino acids|Alanine, as
:or complex|outflow tract morphogenesis|cardiac septum development|plasma membra
: membrane|dopamine receptor binding
nosphate binding|cytoplasm|plasma membrane|anatomical structure morphogenesis|pl
endopeptidase activity|extracellular space|endoplasmic reticulum|proteolysis|gl
action
.imation

.-4,5-bisphosphate binding|phosphatidylinositol-3,4,5-trisphosphate binding|cyto
osylmethionine-dependent methyltransferase activity|site-specific DNA-methyltran

.ty

Proximal tubule bicarbonate reclamation|Carbon metabolism|Alanine, aspartate an
ı-protein transferase activity|chromosome|nucleolus|microtubule organizing cente

:in capping protein complex|regulation of cell size|positive regulation of macro
.ng|nucleus|metal ion binding

: matrix structural constituent|calcium ion binding|basement membrane|extracellu

athway

l binding|nucleus|nuclear envelope|endoplasmic reticulum membrane|cytosol|plasma

pathway|Renin secretion

eus|cytoplasm|cell cycle|microtubule cytoskeleton|carbon-sulfur lyase activity|

post-translational protein modification|positive regulation of protein cataboli

l GTPase binding|ciliary basal body|identical protein binding|protein dimerizat

ction|Hippo signaling pathway - fly|Dopaminergic synapse|Oocyte meiosis|Long-te

pathway

tabolism of xenobiotics by cytochrome P450

cell differentiation|dendrite|postsynaptic membrane|perinuclear region of cyto

ndosome|endoplasmic reticulum|trans-Golgi network|cytosol|intracellular protein

|Apoptosis|Protein processing in endoplasmic reticulum|p53 signaling pathway|Lo

etabolism|Lysine degradation|beta-Alanine metabolism|Histidine metabolism|Pyruv

|ECM-receptor interaction|Focal adhesion
eoxyribonuclease activity|transposition, RNA-mediated|reverse transcription invo
horing|ciliary basal body|cilium assembly
m membrane|cytosol|positive regulation of cell proliferation|membrane|negative
space|cytoplasm|endoplasmic reticulum|carbohydrate metabolic process|apoptotic
tion factor activity, RNA polymerase II-specific|DNA binding|transcription core
thway - yeast|Regulation of mitophagy - yeast|Phospholipase D signaling pathway
cell migration|regulation of cell proliferation|histone deacetylase binding|al

s|Renin secretion|Neuroactive ligand-receptor interaction|Sphingolipid signalin

|endosome membrane|membrane|protein palmitoylation|cell junction|dendritic spi

ximal promoter sequence-specific DNA binding|DNA-binding transcription factor a

:omponent of membrane|interleukin-17 receptor activity|interleukin-17-mediated s

-specific|XY body|DNA binding|chromatin binding|transcription coactivator activi

ıation|transferase activity|metal ion binding

ılar matrix disassembly|positive regulation of smoothened signaling pathway|prot
.nding|nucleoplasm|cytosol|RNA processing|response to oxidative stress|poly(A) b
granule lumen|specific granule lumen|neutrophil degranulation
oximal promoter sequence-specific DNA binding|RNA polymerase II distal enhancer
oximal promoter sequence-specific DNA binding|DNA-binding transcription factor a
oximal promoter sequence-specific DNA binding|RNA polymerase II distal enhancer
:otein binding|calyx of Held|regulation of synaptic transmission, glutamatergic|
f actin cytoskeleton
protein heterooligomerization
;ulatory region sequence-specific DNA binding|DNA-binding transcription factor a

.eoside triphosphate catabolic process|extracellular exosome

:osol|cell-cell junction|centrosome cycle|regulation of G2/M transition of mitot

.ng|DNA-binding transcription factor activity|nucleus|regulation of transcriptic
ıratus|cytosol|intracellular protein transport|ER to Golgi vesicle-mediated trar
.ng|histone acetylation
.x|cytosol|iron-sulfur cluster assembly|small molecule metabolic process|2 iron,

ing pathway|TNF signaling pathway
egulating pluripotency of stem cells|TGF-beta signaling pathway

:ilium movement involved in cell motility|9+2 motile cilium

ıs|nucleoplasm|cytosol|transcription-coupled nucleotide-excision repair|protein

ıctivity, RNA polymerase II-specific|DNA binding|nucleus|zinc ion binding

ıbrane|viral process|cytoplasmic vesicle|response to interferon-gamma|protein hc

|ECM-receptor interaction|Focal adhesion

process

protein phosphorylation|cell cycle|signal transduction by protein phosphorylati
nent of membrane|response to endoplasmic reticulum stress|cell redox homeostasi
osome|cytosol|plasma membrane|retrograde vesicle-mediated transport, Golgi to E

Pase binding|metal ion binding|exocytic vesicle
ndensation|positive regulation of protein ubiquitination|metal ion binding|nega

-kappaB signaling|negative regulation of phosphatidylinositol 3-kinase signaling
y|Phagosome|PI3K-Akt signaling pathway|NF-kappa B signaling pathway

carboxylic acid metabolism|Carbon metabolism
rotein-coupled receptor signaling pathway|integral component of membrane

tion of fat cell differentiation|15-oxoprostaglandin 13-oxidase activity
-type exopeptidase activity|regulation of synaptic vesicle exocytosis

ndosome membrane|protein transport|endosomal transport|cytoplasmic vesicle memb

g system
binoid signaling|Neuroactive ligand-receptor interaction

tethering to Golgi
tus|plasma membrane|transcription factor binding|integral component of membrane
pathway
atus|ion channel binding|regulation of defense response to virus by virus|prote

st|Endocytosis
II-specific|DNA-binding transcription repressor activity, RNA polymerase II-spe
nscription factor binding|DNA binding|transcription corepressor activity|nucleu

atan sulfate
|cell adhesion|integrin-mediated signaling pathway|negative regulation of gene
ranscription factor activity, RNA polymerase II-specific|DNA-binding transcript

tabolism of xenobiotics by cytochrome P450
binding|nucleus|unconventional myosin complex|Z disc|filamentous actin|cardiac
ransferring glycosyl groups

egral component of membrane

ule|positive regulation of inflammatory response|T cell receptor signaling pathw

n of proteasomal ubiquitin-dependent protein catabolic process|positive regulati
sport vesicle|cytoplasmic vesicle|neuron projection|neuronal cell body
reticulum membrane|endoplasmic reticulum-Golgi intermediate compartment|Golgi ap

tein-coupled receptor signaling pathway|regulation of blood pressure|regulation

maturation
brane|integral component of membrane|oxidoreductase activity

by RNA polymerase II|cell cycle|membrane|nuclear speck|erythrocyte differentiat
ing|nucleus|metal ion binding
protein amino acid acetylation|mitotic sister chromatid cohesion|N-acetyltrans
ation of G2/M transition of mitotic cell cycle|protein transport|membrane|prote
asm|cytoplasm|cytosol|apoptotic process|post-translational protein modification|
moter DNA-binding transcription activator activity, RNA polymerase II-specific|I
-binding transcription factor activity, RNA polymerase II-specific|distal enhan
eoxyribonuclease activity|transposition, RNA-mediated|reverse transcription invo

region|positive regulation of cell-substrate adhesion|extracellular matrix const

|PI3K-Akt signaling pathway|ECM-receptor interaction|Focal adhesion
acellular|cytoplasm|microtubule|focal adhesion|dystrophin-associated glycoprotei
ing|nucleus|metal ion binding
thesis|Various types of N-glycan biosynthesis

nuclear chromosome, telomeric region|DNA-binding transcription factor activity,
na membrane|sodium ion transport|endosome membrane|lithium:proton antiporter act

anterior/posterior pattern specification|dentate gyrus development|cerebral cort
intermediate compartment|trans-Golgi network|DNA metabolic process|Golgi organi

Hippo signaling pathway|Hippo signaling pathway - fly
ar space|endoplasmic reticulum lumen|proteolysis|heparin binding|metallopeptida
of plasma membrane|copper ion transport|cellular copper ion homeostasis|recycli

process|acyl-CoA metabolic process

:ranscription factor activity, RNA polymerase II-specific|proximal promoter DNA-
NA-binding transcription factor activity, RNA polymerase II-specific|DNA-binding

:ranscription factor activity, RNA polymerase II-specific|DNA-binding transcript
reticulum organization|integral component of membrane|protein homodimerization

ng|DNA-binding transcription factor activity|nucleus|transcription by RNA polym

gnaling pathway|positive regulation of fat cell differentiation|metal ion bindir
ucleoplasm|endoplasmic reticulum|endoplasmic reticulum membrane|plasma membrane|
tabolism of xenobiotics by cytochrome P450
ng|nucleus|nucleoplasm|cytoplasm|protein phosphorylation|cellular response to I

ution|neuron cellular homeostasis

lium|protein transport|BBSome|ciliary basal body|establishment of anatomical st

g|RNA splicing|miRNA binding|mRNA transport|cellular response to sodium arsenite

filament|plasma membrane|integral component of plasma membrane|amino acid tran

:ytoplasm|thiosulfate-thiol sulfurtransferase activity|sulfide oxidation, using
:ranscription factor activity, RNA polymerase II-specific|proximal promoter DNA-
g extracellular matrix
s|trehalose catabolic process|protein-glucosylgalactosylhydroxylysine glucosidas
rbonate reclamation|GABAergic synapse|Alanine, aspartate and glutamate metaboli
g|microtubule cytoskeleton
etrograde endocannabinoid signaling
oclast differentiation
esis|DNA binding|DNA-binding transcription factor activity|transcription corepre
of protein phosphorylation|actin binding|cytoplasm|polysome|negative regulation

limerization activity

in-dependent protein catabolic process|regulation of ARF protein signal transdu
eton|synapse assembly|F-actin capping protein complex|postsynaptic density|prote

sport|Insulin secretion

JDP-alpha-D-glucose:glucosyl-glycogenin alpha-D-glucosyltransferase activity
g system
bolism
le-mediated transport|protein localization to Golgi membrane
|integral component of membrane|palmitoyltransferase activity|protein-cysteine
of plasma membrane|extracellular exosome|phospholipase A2 activity (consuming

on|extracellular space|proteolysis|multicellular organism development|heparin b
protein|membrane|ATPase activity|ubiquitin-dependent ERAD pathway|endoplasmic r
ion|Sphingolipid signaling pathway

y|Wnt signaling pathway|Signaling pathways regulating pluripotency of stem cell
al component of membrane|iron import into the mitochondrion|iron ion homeostasi

atan sulfate

lothelial cell proliferation|signaling receptor binding|calcium ion binding|extr

toplasm|septin ring|positive regulation of cell proliferation|microtubule cytos
ding|nucleus|regulation of gene expression|chromatin DNA binding|sequence-speci
s

y|Hippo signaling pathway|Wnt signaling pathway|Signaling pathways regulating p

tide exchange factor activity|TRAPP complex|early endosome to Golgi transport|
cleoplasm|cytoplasm|mRNA processing|RNA splicing|metal ion binding

gnal transduction|Ras protein signal transduction
ne|cytosol|plasma membrane|nervous system development|positive regulation of ma
n|extracellular space|endoplasmic reticulum lumen|Golgi apparatus|negative regu

eotide sugar metabolism|Pentose phosphate pathway|Starch and sucrose metabolism
inding|positive regulation of transcription by RNA polymerase II|protein hetero
eptidase activity|cytoplasm|proteolysis|peptidase activity|cysteine-type peptic
membrane|positive regulation of ATPase activity|protein localization to nucleus

on|Rab GTPase binding|actin filament polymerization|filamin binding|neuron pro
ity|integral component of membrane|apical plasma membrane
ng|nucleus|regulation of transcription, DNA-templated|zinc ion binding|identica
l signaling pathway|Long-term depression|Platelet activation|Sphingolipid signa
|mitochondrion|cytosol|DNA repair|mesodermal cell fate specification|transcript

l structure morphogenesis|negative regulation of translation|regulation of cyto
thesis|Various types of N-glycan biosynthesis

crosis factor-mediated signaling pathway|kinase binding|negative regulation of l
mbrane|Golgi-associated vesicle membrane|Golgi cisterna membrane|regulation of
ganism development|germ cell development|spermatogenesis|zinc ion binding|cytop

idylcholine biosynthetic process|choline transmembrane transporter activity|cho
donucleolytic cleavage in ITS1 to separate SSU-rRNA from 5.8S rRNA and LSU-rRNA

e|phosphatidylinositol transporter activity|integral component of membrane|cort
solic DNA-sensing pathway
n|Apoptosis
osphatidylinositol biosynthetic process|adult locomotory behavior|inositol mono

thway|Phosphatidylinositol signaling system|Glycerolipid metabolism

helicase activity|catalytic step 2 spliceosome
membrane|adherens junction|axon guidance|negative regulation of cell prolifera
cleotide excision repair|Purine metabolism|Pyrimidine metabolism|Base excision
ding|RNA polymerase II distal enhancer sequence-specific DNA binding|DNA-bindir
binding|detection of temperature stimulus involved in sensory perception of pa
osphate diphosphatase activity|bis(5'-adenosyl)-hexaphosphatase activity|bis(5'
ide binding
ratus|plasma membrane|flavin-linked sulfhydryl oxidase activity|integral compor
ation or depolymerization|cytoplasmic microtubule organization|neuron projectic

migration|Rap1 signaling pathway|Hippo signaling pathway|Tight junction|Thyroid
transmembrane transporter activity|integral component of membrane|cytoplasmic
imerization activity

ical protein binding|neutrophil degranulation
ranscription factor activity, RNA polymerase II-specific|proximal promoter DNA-
mbrane|positive regulation of apoptotic process|ciliary membrane|cilium assembl
euroblast proliferation|mitochondrion|centrosome|microtubule|postsynaptic densi
ate digestion and absorption|Neomycin, kanamycin and gentamicin biosynthesis|Ga
|ECM-receptor interaction|Focal adhesion
ling|transport vesicle|extracellular matrix organization|lung development|extrac
onent of membrane|catechol O-methyltransferase activity|developmental process|r
ximal promoter sequence-specific DNA binding|DNA-binding transcription factor a

:ess|cholesterol binding|integral component of membrane|ubiquitin-dependent ERAI
ıkyrin binding|M band|regulation of intracellular estrogen receptor signaling pa
orter activity|symporter activity|monocarboxylic acid transport|urate metabolic

y|Wnt signaling pathway|Signaling pathways regulating pluripotency of stem cell
gulatory region sequence-specific DNA binding|RNA polymerase II proximal promote
hways regulating pluripotency of stem cells

|Vascular smooth muscle contraction
ıcellular protein transport|autophagy|regulation of autophagy|vesicle-mediated t
y|AMPK signaling pathway

se binding|clathrin binding|negative regulation of mitotic nuclear division|extr
asm|cytosol|signal transduction|regulation of G protein-coupled receptor signal
response|extracellular space|inflammatory response|extracellular matrix|toll-l

gnal transduction|nervous system development|associative learning|trans-Golgi ne

like receptor signaling pathway|Longevity regulating pathway - multiple species
ıse activity|protein tyrosine kinase activity|ATP binding|nucleus|regulation of
of membrane|ubiquitin-dependent ERAD pathway|ubiquitin protein ligase binding
ılation of cell proliferation|membrane|melanosome|positive regulation of neuroge
ion transport|cellular calcium ion homeostasis|calcium, potassium:sodium antipo
onhomologous end joining|protein C-terminus binding|nuclear body|intra-S DNA dam

es|Oxytocin signaling pathway|Cardiac muscle contraction

g system
thway|Phosphatidylinositol signaling system|Glycerolipid metabolism
-binding transcription factor activity, RNA polymerase II-specific|distal enhar

l transmembrane transporter activity|FAD transmembrane transporter activity|foli

tracellular matrix structural constituent|calcium ion binding|extracellular reg

ay|p53 signaling pathway|PI3K-Akt signaling pathway|Focal adhesion|Wnt signalin

regulation of protein phosphorylation|DNA binding|cytosol|membrane|Wnt signalir

gulation of amyloid-beta formation

n sprouting angiogenesis|extracellular space|regulation of endothelial cell mig
gradation of ketone bodies|Butanoate metabolism

t|integral component of membrane|Rab GTPase binding|regulation of exocytosis|sy
cycle
ine phosphorylation|intracellular signal transduction|metal ion binding

lation of lipolysis in adipocytes

e|mitochondrion organization|microtubule-based movement|microtubule binding|ATF
e
TCA cycle)
plasmic reticulum membrane|3'-phosphoadenosine 5'-phosphosulfate transmembrane t

abolic process

\- worm|Oocyte meiosis|Cell cycle|Hippo signaling pathway|PI3K-Akt signaling pat
on molecules|integral component of membrane
nesis|Rap1 signaling pathway|Hematopoietic cell lineage|Phospholipase D signali
endosome membrane

ation

on channel binding|protein localization to plasma membrane
idase inhibitor activity|serine-type endopeptidase inhibitor activity|extracell

tive regulation of insulin secretion
pathway
nation|eye photoreceptor cell development|NADP-retinol dehydrogenase activity
way|Inflammatory mediator regulation of TRP channels|Renin secretion

atan sulfate

n binding

n serine/threonine phosphatase activity|calcium ion binding|nucleus|nucleoplasm|
tion|negative regulation of cell proliferation|protein heterodimerization activ
eoxyribonuclease activity|transposition, RNA-mediated|reverse transcription invo
eolus|cytoplasm|cytoplasmic stress granule|transposition, RNA-mediated|cytoplas

:ess|protein ubiquitination|regulation of mitotic metaphase/anaphase transition|

.thway|Phosphatidylinositol signaling system|Glycerolipid metabolism
ıal transduction|positive regulation of transcription by RNA polymerase II|innei
.thesis|Various types of N-glycan biosynthesis
ılopment|extracellular exosome

mitochondrion|tRNA methylation
.ng|DNA-binding transcription factor activity|nucleus|regulation of transcriptic
-kappaB signaling|negative regulation of phosphatidylinositol 3-kinase signaling
:ranscription factor activity, RNA polymerase II-specific|DNA-binding transcript
'K signaling pathway|PPAR signaling pathway
:tabolic process|lipid transport|membrane|integral component of membrane|inner e
ɔtic density|cell junction|early endosome membrane|glutamate receptor binding|pı
ıl component of membrane|protein kinase binding
Synaptic vesicle cycle
:ransporter activity|negative regulation of transmembrane transport|protein phos
ubiquitination|protein heterodimerization activity|extracellular exosome

:ific binding|metal ion binding|RNA polymerase binding

tiple species|Hippo signaling pathway|Hippo signaling pathway - fly
ɔximal promoter sequence-specific DNA binding|DNA-binding transcription factor a
ıin specific binding|identical protein binding|regulation of mRNA splicing, via
regulation of apoptotic process|cell-cell junction organization|metal ion bindi
.nding|mRNA binding|cytosol|negative regulation of translation|cell junction|tra

. and sucrose metabolism

gulatory region sequence-specific DNA binding|DNA-binding transcription factor a
:ane

scription factor activity, RNA polymerase II-specific|DNA-binding transcription

ɔndrial fission|GTPase activity|GTP binding|nucleus|nuclear pore|cytoplasm|cytos

:ellular|integral component of plasma membrane|cell-cell junction|cell surface ı
.ation of muscle contraction|negative regulation of cardiac muscle cell apoptoti

n signaling pathway
rase II|RNA polymerase II proximal promoter sequence-specific DNA binding|DNA-bi
ation of apoptotic process|negative regulation of apoptotic process|positive reg
B signaling pathway|Ras signaling pathway
vesicle membrane|extracellular matrix|otolith morphogenesis

nding|nucleus|regulation of gene expression|chromatin DNA binding|sequence-speci
vision|protein kinase binding|regulation of protein localization|cell division|r
ytosol|DNA replication|tRNA metabolic process|metal ion binding|D-tyrosyl-tRNA(T

of histone H3-K36 methylation|RNA polymerase II regulatory region sequence-speci

ent of cytoskeleton|structural constituent of eye lens|nucleus|cytoplasm|peroxis
oximal promoter sequence-specific DNA binding|DNA-binding transcription factor a

ivity|Rho guanyl-nucleotide exchange factor activity|calmodulin binding|ATP binc

sis|cell surface|integral component of membrane|viral process|carbohydrate bindi
gy of mitochondrion|autophagosome|integral component of membrane|cytoplasmic ves
ional protein modification
ndocytosis|Synaptic vesicle cycle

component of membrane|flagellated sperm motility|ion transmembrane transport|r
Insulin signaling pathway|MAPK signaling pathway|Rap1 signaling pathway|Regulat
sma membrane|focal adhesion|cell cortex|cytoskeleton organization|cell cycle|cel
protein phosphatase 1 binding|postsynaptic density|actin cytoskeleton|calcium-m

microtubule plus-end

1 signaling pathway|Long-term depression|Ovarian steroidogenesis|Platelet activ

P binding|cytoplasm|cytosol|protein phosphorylation|actin cytoskeleton|membrane

ase activity|non-membrane spanning protein tyrosine kinase activity|ATP binding|
gnaling pathway
es|Oxytocin signaling pathway|Cardiac muscle contraction
t|integral component of membrane|glycolipid translocation

ell cycle|cell adhesion|peptidase activity|membrane|metal ion binding|cell divis

otion factor activity, RNA polymerase II-specific|DNA binding|DNA-binding transc

.e

ıssembly|male meiotic nuclear division|female meiotic nuclear division|fertiliza
rity|ATP binding|nucleus|cytoplasm|alanyl-tRNA aminoacylation|regulation of tran
ıtophagy|viral life cycle|late endosome membrane|endosome transport via multives

ein C-terminus binding|microtubule-severing ATPase activity|regulation of doubl
growth|intracellular receptor signaling pathway|wound healing

olymerase II transcription|mRNA 3'-end processing by stem-loop binding and clea
rine phosphorylation|intracellular signal transduction|metal ion binding
membrane|proteolysis|signal transduction|cell-cell signaling|zinc ion binding|p

rtoskeleton organization|regulation of cell shape|cell migration|Rho GTPase binc
evelopment
.c anion transmembrane transporter activity|prostaglandin transport|sodium-indep

response|cell adhesion|signal transduction|cell-cell signaling|positive regulati
|ECM-receptor interaction|Focal adhesion
.tuent of myelin sheath|clathrin-coated vesicle|cytoplasmic vesicle|myelination|
g metabolism - other enzymes|beta-Alanine metabolism
ller cell mediated cytotoxicity

otion factor activity, RNA polymerase II-specific|proximal promoter DNA-binding
ınsport|COPI vesicle coat|toxin transport

Rap1 signaling pathway

. component of plasma membrane|autophagy|negative regulation of calcium ion tran
actin cytoskeleton|Ras signaling pathway|PI3K-Akt signaling pathway

gulation of MAPK cascade|perinuclear region of cytoplasm|positive regulation of

ıl side of plasma membrane|integral component of membrane|clathrin-coated vesicl
.cing, via spliceosome|nuclear chromosome, telomeric region|nuclear chromatin|p5

ll adhesion molecules (CAMs)|Intestinal immune network for IgA production|Ras s
ılation of macrophage chemotaxis|myoblast migration involved in skeletal muscle
gulatory region sequence-specific DNA binding|DNA-binding transcription represso

of plasma membrane|cell adhesion|homophilic cell adhesion via plasma membrane ac

dhesion|Complement and coagulation cascades
y|Apoptosis|Protein processing in endoplasmic reticulum|Neurotrophin signaling

|ECM-receptor interaction|Focal adhesion
|ECM-receptor interaction|Focal adhesion
activity|transcription coactivator activity|transcription corepressor activity|
of membrane|extracellular exosome
eoxyribonuclease activity|transposition, RNA-mediated|reverse transcription invo
culum membrane|plasma membrane|integral component of plasma membrane|cell-cell
y|Focal adhesion|NOD-like receptor signaling pathway|Ubiquitin mediated proteol

ne|cell differentiation|negative regulation of Notch signaling pathway

asmic reticulum|MAPK signaling pathway - fly
ligase binding|histone deacetylase binding|regulation of protein complex assemb

bohydrate binding
system development|DNA binding|nucleus|sequence-specific DNA binding
eticulum membrane|Golgi apparatus|integral component of membrane|post-translatio

lular region|basement membrane|extracellular space|peptidyl-lysine oxidation|pr

otion factor activity, RNA polymerase II-specific|neuron migration|maternal plac
formation|protein serine/threonine kinase activity|ATP binding|cytoplasm|centro

cell junction|filamin binding|regulation of integrin activation|cell junction as

assembly|regulation of transcription, DNA-templated|nucleosome positioning|chr

ription elongation from RNA polymerase II promoter|ubiquitin-dependent protein c
quitin-dependent protein catabolic process|zinc ion binding|endomembrane system

eoxyribonuclease activity|transposition, RNA-mediated|reverse transcription invo

from RNA polymerase II promoter|multicellular organism development|nuclear bo

ort|Rab GTPase binding|retrograde transport, endosome to Golgi|regulation of GTI
ore|nucleus|nucleoplasm|cytoplasm|cytosol|mitotic cell cycle checkpoint|nuclear
Glyoxylate and dicarboxylate metabolism|Pyruvate metabolism|Citrate cycle (TCA
ase inhibitor activity|extracellular region|hyaluronan metabolic process|platele

immune response|cyclin-dependent protein serine/threonine kinase inhibitor acti

carboxylic acid metabolism|Carbon metabolism
ng pathway
activity|cysteine-type endopeptidase inhibitor activity|calcium ion binding|col

s of O-glycan biosynthesis|Galactose metabolism|N-Glycan biosynthesis|Various t

lic cell adhesion via plasma membrane adhesion molecules|growth cone|cell-cell
ism

ay|Ras signaling pathway|PI3K-Akt signaling pathway|Focal adhesion
n kinase inhibitor activity|nucleus|cytoplasm|regulation of G2/M transition of

embrane|protein phosphorylation|inflammatory response|protein C-terminus binding

ase activity|mitochondrial intermembrane space|cytosol|ubiquitin-dependent prote

ng|nucleus|metal ion binding
e|cytoplasm|superoxide metabolic process|heart development|phosphatidylinositol-
cle contraction
ellular region|Golgi lumen|cell aging|heparin binding|keratan sulfate biosynthet
on|Intestinal immune network for IgA production|Cytokine-cytokine receptor inte
ignaling pathway|Hematopoietic cell lineage|Phospholipase D signaling pathway|R
process|metal ion binding
gi membrane

opment|integral component of membrane|tissue regeneration|hyaloid vascular plexu
tion|histone H4-K8 acetylation|histone H4-K12 acetylation|metal ion binding
um|ER membrane protein complex

plasma membrane|lipid metabolic process|glycerophospholipid biosynthetic process

cycle

em development|extracellular matrix

some|Cell adhesion molecules (CAMs)|Antigen processing and presentation
y

metal ion binding|ubiquitin protein ligase activity|rescue of stalled ribosome
ism
olgi apparatus|cytosol|plasma membrane|glycosphingolipid metabolic process|lipi
n birth or egg hatching

vity|membrane|integral component of membrane|negative regulation of activin rec
ng|nucleus|metal ion binding

on|Focal adhesion|Regulation of actin cytoskeleton

athway
orylation|G protein-coupled receptor signaling pathway|adenylate cyclase-activa
e|GTPase activity|structural constituent of cytoskeleton|GTP binding|cytoplasm|
a membrane|intracellular protein transport|ER to Golgi vesicle-mediated transpo
activity|phosphatidylethanolamine acyl-chain remodeling|carboxylic ester hydrol
nce|Rap1 signaling pathway
ng|nucleus|metal ion binding
oter DNA-binding transcription repressor activity, RNA polymerase II-specific|I
by RNA polymerase II|CHRAC|histone acetylation|ACF complex|metal ion binding
ding
ng|nucleus|metal ion binding
anchored component of membrane
veola|glycerol metabolic process|muscle contraction|myoblast fusion|blood circu
action

-activated receptor activity|Golgi apparatus|plasma membrane|heparin binding|neg
ng|DNA-binding transcription factor activity|nucleus|regulation of transcriptio
rocess|lipid binding|sterol transporter activity|cholesterol binding|membrane|r

actin cytoskeleton|Ras signaling pathway|PI3K-Akt signaling pathway

aling pathway

surface receptor signaling pathway|membrane|regulation of calcium ion transmembr

ntegral component of plasma membrane|cobalt ion transport|zinc ion transport|ce
mbrane|UDP-glucuronate biosynthetic process|carbohydrate transport|pyrimidine r
ell-matrix adhesion|regulation of cell shape|cell junction|actin cytoskeleton r
e monoubiquitination|histone deubiquitination|nuclear receptor transcription co
nscription coactivator activity|nucleoplasm|histone H3-K36 methylation|protein-

›sphatidylinositol phosphorylation|phosphatidylinositol-mediated signaling

.vity|protein transport|intracellular membrane-bounded organelle
|ECM-receptor interaction|Focal adhesion
›plasm|cytosol|regulation of transcription, DNA-templated|calcium-mediated signa

/ity|signaling receptor binding|FK506 binding|cytoplasm|cytosol|'de novo' protei

ıse mediated signal transduction|regulation of Ral protein signal transduction

.ng|RNA binding|nucleus|RNA polymerase II, core complex|transcription by RNA pol

:ranscription factor activity, RNA polymerase II-specific|actin monomer binding|
:hannel activity|plasma membrane|integral component of plasma membrane|potassium
oactive ligand-receptor interaction|Taste transduction

ıdrial inner membrane|mitochondrial matrix|cytosol|rRNA binding|ribosomal small

interaction

ˢpace|cell adhesion|cell-matrix adhesion|positive regulation of gene expression|

ıigration|Tight junction
ıbrane|regulated exocytosis|synaptic vesicle membrane organization|extracellular

.on|cell surface|positive regulation of T cell chemotaxis|integral component of

:lass I|antigen processing and presentation of exogenous peptide antigen via MHC
ıbrane|extrinsic component of cytoplasmic side of plasma membrane|organelle memb
gulatory region sequence-specific DNA binding|DNA-binding transcription factor a
›rotein ubiquitination|actin cytoskeleton organization|cell junction|POZ domain
›tion factor activity, RNA polymerase II-specific|nucleus|nucleoplasm|DNA methyl
›nstituent|extracellular region|extracellular space|plasma membrane|chemotaxis|c

:racellular protein transport|vesicle-mediated transport

g system
ˢndosome|early endosome|endoplasmic reticulum membrane|Golgi apparatus|plasma me

naling pathway|VEGF signaling pathway|Ras signaling pathway|PI3K-Akt signaling
s|granzyme-mediated apoptotic signaling pathway|protein processing|negative regu
athway|Hippo signaling pathway - fly
ity
ng|metal ion binding|oxidation-reduction process

me|cytosol|plasma membrane|negative regulation of translation|negative regulati
|SCF-dependent proteasomal ubiquitin-dependent protein catabolic process
membrane potential|presynaptic membrane|ion channel binding|synapse|regulation
growth factor receptor binding|extracellular space|endoplasmic reticulum membran
ndrial fission|GTPase activity|GTP binding|nucleus|cytoplasm|endoplasmic reticu
orso-ventral axis formation
|Renin-angiotensin system
-binding transcription factor activity, RNA polymerase II-specific|formation of

asmic reticulum
eoxyribonuclease activity|transposition, RNA-mediated|reverse transcription invo
and sucrose metabolism
|extracellular exosome

s|nucleoplasm|cytoplasm|RNA export from nucleus|mRNA export from nucleus|membra

y|cilium assembly|establishment of epithelial cell polarity|ciliary transition

al component of plasma membrane|heterophilic cell-cell adhesion via plasma memb

protein transport|ER to Golgi vesicle-mediated transport|retrograde vesicle-me

r activity|cytosol|cytoskeleton|plasma membrane|transforming growth factor beta
cleus|cytosol|cytoplasmic stress granule|stress granule assembly|perikaryon|neg
tuent of myelin sheath|clathrin-coated vesicle|cytoplasmic vesicle|myelination|
matrix|extracellular exosome|response to transforming growth factor beta
phospholipid binding|plasma membrane|regulation of dopamine secretion|integral

oplasmic reticulum membrane|extracellular exosome
e activity|lysosome|cytosol|ubiquitin-dependent protein catabolic process|zinc

nent of membrane|divalent inorganic cation transmembrane transporter activity

ng|nucleus|metal ion binding
f RNA splicing
tion factor activity, RNA polymerase II-specific|DNA-binding transcription repr

;ulatory region sequence-specific DNA binding|DNA-binding transcription factor a

ıigration|Tight junction

ıerase II-specific|ubiquitin-protein transferase activity|nucleus|cytosol|metal

.ng|nucleus|metal ion binding
›th muscle contraction|actomyosin structure organization|negative regulation of

.ng|nucleus|metal ion binding

cytoskeleton|Gap junction|Rap1 signaling pathway|Phospholipase D signaling path
›po signaling pathway|Hedgehog signaling pathway|T cell receptor signaling pathw
l in inflammatory response|plasma membrane|integral component of plasma membrane
.on|fibrillar collagen trimer|extracellular space|Golgi lumen|visual perception|

ıding|cytoplasm|membrane|positive regulation of NF-kappaB transcription factor a

tabolism of xenobiotics by cytochrome P450

c synapse|Retrograde endocannabinoid signaling|GABAergic synapse|Dopaminergic s
membrane|integral component of plasma membrane|cell-cell junction|Wnt signaling
:tin filament-based movement|actin cortical patch|actin filament binding|positiv
rity|ATP binding|nucleus|cytoplasm|alanyl-tRNA aminoacylation|regulation of tran
›ximal promoter sequence-specific DNA binding|DNA-binding transcription factor a

:hetic process|cholesterol metabolic process|electron transfer activity|sterol m
signaling pathway|Calcium signaling pathway|Regulation of actin cytoskeleton|N

toskeleton
:ion of immune response|T cell receptor signaling pathway
ıorylation|transcription factor binding|mitogen-activated protein kinase kinase
ıerase II intronic transcription regulatory region sequence-specific DNA binding

:y|protein serine/threonine kinase activity|cyclin-dependent protein serine/thre

›rotein signal transduction
ıctivity|potassium channel regulator activity|membrane|ion channel binding|prote

:ansport
led RNA binding|lysosome|lysosomal membrane|plasma membrane|RNA catabolic proces

;ociated vesicle|cis-Golgi network|cytosol|plasma membrane|retrograde vesicle-me

ng|nucleus|metal ion binding
athway

fly|TGF-beta signaling pathway|Endocytosis|Hedgehog signaling pathway

binding|guanyl-nucleotide exchange factor activity|GTPase activator activity|cy

llular region|extracellular space|cytoplasm|immune response|cell adhesion|cyste

transport|retrograde vesicle-mediated transport, Golgi to ER|membrane|COPII-coa
e lumen|extracellular exosome
ytoplasm
esion|Calcium signaling pathway|Regulation of actin cytoskeleton|Platelet activ

embrane|interleukin-17 receptor activity|defense response to fungus|granulocyte
otubule plus-end|microtubule plus-end binding
oclast differentiation|Intestinal immune network for IgA production|T cell rece
rolyl cis-trans isomerase activity|protein folding|catalytic step 2 spliceosome
nding|DNA-binding transcription factor activity, RNA polymerase II-specific|pro

ation of lipid storage|integral component of membrane|hydrolase activity|extrac

uclear envelope|rough endoplasmic reticulum|signal transduction|female pregnanc
asma membrane|integral component of plasma membrane|cell adhesion|negative regu

ng|nucleus|metal ion binding
ular region|cytoplasm|plasma membrane|cell-cell junction|cell adhesion|homophil
mbrane transporter activity|chloride transmembrane transporter activity|sulfate

positive regulation of cell proliferation|positive regulation of nuclear cell c

thetic process|glycosaminoglycan catabolic process|cell surface|membrane|integr
athway
ity|plasma membrane|integral component of membrane|leak channel activity|ion tr
|Carbon metabolism|Glyoxylate and dicarboxylate metabolism|Cyanoamino acid meta
ral component of plasma membrane
rotein ubiquitination|transferase activity|cytoplasmic vesicle membrane|early e
ay|Ras signaling pathway|PI3K-Akt signaling pathway|Focal adhesion
ion|signal transduction|coreceptor activity|BMP signaling pathway|identical pro

lgi apparatus|plasma membrane|Golgi organization|integral component of membrane
t of plasma membrane|nucleobase-containing compound metabolic process|lactation

binding|nucleus|plasma membrane|immune response|signal transduction|cell surfa

nt organization|cell communication by chemical coupling|cell communication by e

s

ate metabolism|Carbon metabolism|Biosynthesis of amino acids|Fructose and manno

F signaling pathway|Osteoclast differentiation|Cytokine-cytokine receptor inter
regulation of protein ubiquitination|regulation of growth|positive regulation c

ed glycosylation via arginine
Synaptic vesicle cycle

ellular protein transport|heart development|regulation of smoothened signaling
ns junction|signal transduction|beta-catenin binding|cytoskeletal protein bindi
egative regulation of chondrocyte development|actin filament bundle organizatic
zation activity|perinuclear region of cytoplasm|defense response to virus|cellu
oliferation
ng|nucleoplasm|double-strand break repair via nonhomologous end joining|zinc ic
ding|proximal promoter DNA-binding transcription repressor activity, RNA polyme

n antiporter activity|integral component of membrane|late endosome membrane|reg

itochondrial ribosome|mitochondrial small ribosomal subunit|mitochondrial trans

apparatus|cytosol|ubiquitin-dependent protein catabolic process|membrane|protei
ranscription factor activity, RNA polymerase II-specific|proximal promoter DNA-
cid binding|transcription corepressor activity|nucleus|regulation of gene expre

tivity|potassium channel activity|plasma membrane|potassium ion transport|volta
transport|neuron projection development|cytoplasmic vesicle|regulation of tumor
se activity|nucleus|cytosol|protein ubiquitination|SCF ubiquitin ligase complex
pecies

idase activity|serine-type endopeptidase activity|cytokine activity|calcium ior
atogenesis|integral component of membrane|positive regulation of bone mineraliz

›sphatidylinositol phosphorylation|phosphatidylinositol-mediated signaling
. transduction

cleotide sugar metabolism|Starch and sucrose metabolism|Galactose metabolism
ıt of membrane|protein import into mitochondrial matrix|mitochondrial respirator

ization|nervous system development|microtubule binding|neurogenesis|negative re

ller cell mediated cytotoxicity
toskeleton|PI3K-Akt signaling pathway|ECM-receptor interaction|Focal adhesion
cell-cell junction|cell-cell adherens junction|cell-cell junction assembly|cell
actor activity, RNA polymerase II-specific|DNA replication origin binding|histc
embrane|focal adhesion|cell adhesion|negative regulation of cell adhesion|signal

›tion by RNA polymerase II|transcription elongation from RNA polymerase II promc

ranscription by RNA polymerase II|3'-phosphoadenosine 5'-phosphosulfate metabol

ng|DNA-binding transcription factor activity|nucleus|metal ion binding

tivity, transferring acyl groups
eleton
zinc ion binding|protein processing
isomerase activity|intracellular|mitochondrion|mitochondrial matrix|carotenoid
sport|GDP binding|secretory granule membrane|phagocytic vesicle membrane|Rab prc
ıent of membrane|basolateral plasma membrane|apical plasma membrane|cell junctic

gral component of Golgi membrane|N-acetylneuraminate 7-O(or 9-O)-acetyltransfera
toskeleton|regulation of cytoskeleton organization

ndoplasmic reticulum
ytoplasm|rRNA processing|CURI complex|UTP-C complex

ınsforming growth factor beta receptor signaling pathway|extracellular matrix|mi
›plasm|cytoskeleton organization|peptidyl-serine phosphorylation|intracellular s

on|Fatty acid metabolism

on|Fatty acid metabolism

 phosphorylation|innate immune response

le hormone processing|integral component of Golgi membrane
ein homodimerization activity|positive regulation of MAP kinase activity|positiv

ty|peptidyl-lysine trimethylation|protein-containing complex

y|plasma membrane|multicellular organism development|spermatogenesis|cell diffe

 membrane|negative regulation of inflammatory response|response to transforming
opment|membrane|integral component of membrane|rough endoplasmic reticulum memb

ling|cell surface|extracellular matrix binding|collagen-containing extracellular

ive regulation of cell-substrate adhesion|extracellular matrix organization|ext

g system
sma membrane|microtubule-based movement|microtubule binding|ATPase activity

ing center|cytosol|plasma membrane|cell proliferation|cerebral cortex developme
embrane|integrator complex|U2 snRNA 3'-end processing|snRNA transcription by RNA

ignaling in cardiomyocytes|AMPK signaling pathway|Sphingolipid signaling pathwa

 proximal promoter sequence-specific DNA binding|DNA-binding transcription fact
ar space|nucleoplasm|endosome|Golgi lumen|cytosol|plasma membrane|glycosaminogly

otein modification process|proteolysis|ubiquitin-dependent protein catabolic pr

ene expression|integral component of membrane|embryonic limb morphogenesis|BMP s
ration|outflow tract morphogenesis|endothelium development|cardiac conduction s

ion involved in sprouting angiogenesis|positive regulation of receptor internal
ane

ım lumen|Golgi apparatus|cellular calcium ion homeostasis|response to oxidative

ıe binding

ır region|positive regulation of endothelial cell migration|integral component c

.ng|DNA-binding transcription factor activity|nucleus|regulation of transcriptic
:acellular space|transforming growth factor beta receptor signaling pathway|sali

transmembrane transporter activity|mitochondrion|mitochondrial inner membrane|a
ıulus|response to UV|nuclear speck|translesion synthesis|positive regulation of
·beta signaling pathway|PI3K-Akt signaling pathway|ECM-receptor interaction|Foca

:otein phosphorylation|apoptotic process|nuclear speck|intracellular signal trar

:ranscription factor activity, RNA polymerase II-specific|proximal promoter DNA-
stsynaptic density membrane|synaptic membrane adhesion|regulation of presynapse

.cleotide sugar metabolism|Ascorbate and aldarate metabolism|Starch and sucrose
.thesis|Various types of N-glycan biosynthesis
ATP binding|nucleus|nucleoplasm|nucleolus|cytoplasm|Golgi apparatus|cytosol|prot

cycle|Ubiquitin mediated proteolysis|Cell cycle - yeast|Meiosis - yeast
ɔse|regulation of presynapse assembly

:ranscription factor activity, RNA polymerase II-specific|proximal promoter DNA-

al killer cell mediated cytotoxicity|MAPK signaling pathway|Chemokine signaling
.ng|nucleus|nucleolus|negative regulation of transcription, DNA-templated|metal

ıoter DNA-binding transcription activator activity, RNA polymerase II-specific|s
cardiac muscle cell development

.tathione metabolism|Cysteine and methionine metabolism
.ng|nucleus|positive regulation of transcription, DNA-templated|metal ion bindir

.dhesion
ɔximal promoter sequence-specific DNA binding|DNA-binding transcription factor a
.dhesion

|Neuroactive ligand-receptor interaction

ubiquitin-dependent protein catabolic process|protein deubiquitination|ubiquiti

oolymerase II-specific|RNA binding|nucleus|zinc ion binding|chromatin silencing
se II distal enhancer sequence-specific DNA binding|DNA-binding transcription fa
naling pathway
or activity|intracellular|cytosol|G protein-coupled receptor signaling pathway|F
endosome membrane|receptor tyrosine kinase binding|identical protein binding|pr

TCA cycle)
ne
esicle-mediated transport
ucleus|nucleoplasm|cytoplasm|cytosol|mRNA processing|circadian rhythm|cellular r

omponent of membrane
cell-cell adherens junction|bicellular tight junction|cell cortex|cell cycle|cel

ty

or activity|extracellular space|intracellular|nucleus|nucleoplasm|cytoplasm|Golg

drion
orane|response to endoplasmic reticulum stress|negative regulation of neuron dea
s|Ran GTPase binding|protein transporter activity|protein-containing complex

ymerase II-specific|DNA-binding transcription factor activity|nucleus|nucleoplas

f plasma membrane|smoothened signaling pathway|neuroblast proliferation|zinc ion
etal ion binding|cellular response to cadmium ion|cellular response to copper ic
nuscle organ development|sequence-specific DNA binding

orotein metabolic process|intracellular membrane-bounded organelle
thway|mTOR signaling pathway

ulation of phosphorylation

by cytochrome P450|Tyrosine metabolism|beta-Alanine metabolism|Histidine metab
c acid metabolism
ne response to tumor cell|metalloendopeptidase activity|integrin binding|plasma
ng|nucleus|cellular response to DNA damage stimulus|metal ion binding
g system
ation|post-translational protein modification|Cul4-RING E3 ubiquitin ligase comp

n signal transduction

embrane|negative regulation of cell proliferation|Wnt signaling pathway|midbody|

se regulator activity|protein kinase binding|flagellated sperm motility|positive
endothelial migration|Long-term potentiation|Rap1 signaling pathway|Adrenergic
ı serine/threonine kinase activity|ATP binding|cytoplasm|Golgi apparatus|cytosol
ı membrane
opressin-regulated water reabsorption|Melanogenesis|Dopaminergic synapse|TNF si
phosphorylation|apoptotic process|cell cycle|regulation of mitotic cell cycle|r
ucose metabolic process|apoptotic process|cellular response to DNA damage stimu
ı membrane|sodium:bicarbonate symporter activity|bicarbonate transport|integral

ıigration

signaling pathway|Leukocyte transendothelial migration|Platelet activation|Axo
membrane|nucleoplasm|cytosol|protein import into nucleus|protein export from r
ıdothelial cell migration|protein kinase binding|mitogen-activated protein kinas

on|ATP biosynthetic process|mitochondrion organization|protein-containing compl
transport|Rab GTPase binding|regulation of GTPase activity|activation of GTPase
ıesion|axon guidance|opsonization|extracellular matrix|positive regulation of ir

g system

vesicle-mediated transport|filopodium|endocytic recycling|alpha-tubulin binding
tivating transcription factor binding|hair follicle development|transcription cc
n-lysine N-methyltransferase activity|peptidyl-lysine methylation
embrane|Golgi apparatus|trans-Golgi network|cytosol|plasma membrane|bile acid bi
l matrix|mitochondrial translation|ribosome disassembly|mitochondrial translati
tyrosine and tryptophan biosynthesis|Isoquinoline alkaloid biosynthesis|Tyrosin

gulatory region sequence-specific DNA binding|DNA-binding transcription factor a

ıctivity
inase activity|protein serine/threonine/tyrosine kinase activity|ATP binding|cy
ıitochondrion|membrane|integral component of membrane|heat shock protein binding
pecies

ısm|DNA strand elongation involved in DNA replication|replication fork protectic

plasmic reticulum membrane|neuron differentiation
rane|sarcoplasmic reticulum membrane
ent|tRNA wobble cytosine modification|nuclear euchromatin|mitochondrion|DNA repa
membrane|protein localization|protein kinase binding|azurophil granule lumen|r
dium|filopodium|axon extension

RNA polymerase II|lysine-acetylated histone binding
ıucleus|nucleoplasm|nucleolus|cytoplasm|translational initiation|positive regula

ase binding|activation of GTPase activity

.asma membrane|inositol metabolic process|peripheral nervous system development|

r|axonemal central apparatus
junction|cell differentiation
loplasmic reticulum|cytosol|brush border|negative regulation of phosphatase acti

ısm|cytoplasm|sequence-specific DNA binding|positive regulation of transcriptior

ation|Fatty acid degradation
some|Cell adhesion molecules (CAMs)|Antigen processing and presentation
ınsport

osome

integral component of membrane|regulation of growth|positive regulation of grov
splicing|nuclear speck|RNA N6-methyladenosine methyltransferase complex|metal i
component of postsynaptic density membrane|regulation of postsynaptic density a

receptor complex|outflow tract septum morphogenesis|vascular endothelial growth
ng|nucleus|cellular response to DNA damage stimulus|metal ion binding
ration|outflow tract morphogenesis|endothelium development|cardiac conduction s
re regulation of synapse assembly
n silencing at rDNA|nucleoplasm|nuclear heterochromatin|nucleolus|DNA methylati
aining complex|substrate adhesion-dependent cell spreading
se II proximal promoter sequence-specific DNA binding|DNA-binding transcription
atan sulfate

somal complex|U2 snRNP|regulation of transcription by RNA polymerase II|viral ge

pecies|Apoptosis|TNF signaling pathway|RIG-I-like receptor signaling pathway

r lipid metabolism|Phospholipase D signaling pathway|Sphingolipid metabolism|Fc
ıronal cell body|axon terminus|postsynaptic membrane|dendritic spine maintenance
lic cell adhesion via plasma membrane adhesion molecules
pathway|TNF signaling pathway

on of cell proliferation|embryonic body morphogenesis|camera-type eye developmer
ease activity|nucleus|cytoplasm|lysosome|cytosol|plasma membrane|ubiquitin-depe

vity|integral component of membrane|basolateral plasma membrane|protein-contair

on|Fatty acid metabolism
ve regulation of synapse assembly

Methane metabolism|Biosynthesis of amino acids
space|nucleus|early endosome|endoplasmic reticulum lumen|rough endoplasmic retic

metallopeptidase activity|cell surface|negative regulation of chondrocyte diffe

n|axoneme|apoptotic process|activation of cysteine-type endopeptidase activity i
.y|nucleus|Swi5-Sfr1 complex|cellular response to ionizing radiation

:ion
onstituent|extracellular space|blood coagulation|extracellular matrix|cellular r
asma membrane|cell-cell junction|cell adhesion|homophilic cell adhesion via plas

unction|Tight junction|Axon guidance|Rap1 signaling pathway|Long-term depressio
ansport of viral protein in host cell|negative regulation of protein binding|hos
:ranscription factor activity, RNA polymerase II-specific|DNA binding|chromatin
ne|plasma membrane|integral component of plasma membrane|cell volume homeostasis
ling|multivesicular body|Golgi apparatus|cytosol|plasma membrane|integral compor

:ion|protein autophosphorylation
.ament|plasma membrane|cell-cell junction|cell-cell adherens junction|cell-cell

paratus|integral component of membrane|perinuclear region of cytoplasm

ase activity|protein tyrosine kinase activity|non-membrane spanning protein tyrc

ar region|positive regulation of endothelial cell migration|integral component c

oskeleton organization|regulation of cell shape|motile cilium|photoreceptor conr
athway|Hippo signaling pathway - fly

.bolism
ale binding|cell migration|motile cilium|cilium-dependent cell motility

nervous system development|heparin binding|animal organ morphogenesis|integral c
oximal promoter sequence-specific DNA binding|DNA-binding transcription factor a

or recycling|positive regulation of neurotransmitter secretion|synaptic transmis
eoxyribonuclease activity|transposition, RNA-mediated|reverse transcription invc

:ule activity|structural constituent of cytoskeleton|cytoplasm|centrosome|centri
starvation|response to wounding|vesicle membrane|membrane|positive regulation c

:ein homodimerization activity|metal ion binding

ament bundle|actin filament binding|actin filament bundle assembly|actin filamer

ing pathway|Dopaminergic synapse|TNF signaling pathway|Osteoclast differentiati
atan sulfate
it of plasma membrane|signal transduction|negative regulation of epidermal growt

al calcium ion transmembrane transport|uniporter activity|calcium-mediated sigr

of plasma membrane|zinc ion transport|cellular zinc ion homeostasis|organelle m

acid binding|nucleus|nucleoplasm|positive regulation of nuclear cell cycle DNA r
ex|plus-end kinesin complex|microtubule|cilium|retrograde vesicle-mediated trar
'hospholipase D signaling pathway|Endocytosis|Synaptic vesicle cycle

:ellular space|endoplasmic reticulum lumen|Golgi apparatus|protein phosphorylati
RNA polymerase II|metal ion binding

binding|protein heterodimerization activity|extracellular exosome
n transport|protein ubiquitination|GDP binding|Rab protein signal transduction|
s factor-mediated signaling pathway|positive regulation of fat cell differentia
stitial matrix|multicellular organism development|cell differentiation|extracel

:ytosol|plasma membrane|caveola|transcription initiation from RNA polymerase I p
Gluconeogenesis
on|Rab guanyl-nucleotide exchange factor activity|Rab GTPase binding|cytoplasmic
ing pathway
; involved in ureteric bud morphogenesis|nucleoplasm|dorsal/ventral pattern form

idopeptidase activity involved in apoptotic process
icleoplasm|lysosomal membrane|endoplasmic reticulum membrane|cytosol|ubiquitin-c
ation|nuclear speck|phosphatase regulator activity|regulation of endocytosis|re

ir junction development|zinc ion binding|protein ubiquitination|cell junction|ne
slation factor activity, RNA binding|membrane|protein kinase regulator activity|

anscription corepressor binding|DNA binding|transcription corepressor activity|
ng|nucleus|metal ion binding

ıidance|intracellular signal transduction|metal ion binding|ephrin receptor binc

ı|ER to Golgi vesicle-mediated transport|neural crest formation|neural crest cel

ıding|nucleus|regulation of gene expression|chromatin DNA binding|sequence-speci
ı|Renin secretion

ɔnse|dendritic cell chemotaxis|G protein-coupled receptor activity|plasma membra
ıne

ɔrgan morphogenesis|protein deubiquitination|positive regulation of peroxisome p
ɘ regulation of signal transduction
 via plasma membrane adhesion molecules|axon guidance|monocarboxylic acid transm

racellular region|extracellular space|proteolysis|peptidase activity|metallopept
teroidogenesis
ylation|Synaptic vesicle cycle|mTOR signaling pathway
rganelle
synapse assembly|glutamatergic synapse|presynapse assembly|integral component of
ı|spindle microtubule|chromosome segregation|regulation of microtubule polymeriz
sm|Peroxisome

bolism|Lysine degradation

nembrane|integral component of membrane|rough endoplasmic reticulum membrane|nuc
ılation of protein localization|positive regulation of membrane tubulation

telomeric region|nucleus|nucleoplasm|telomere maintenance via telomerase|telomer

rlation|peptidyl-threonine phosphorylation|protein phosphatase binding|positive

ulin secretion|Vascular smooth muscle contraction|cGMP-PKG signaling pathway

ling|centrosome separation|regulation of cilium assembly|mitotic cytokinetic prc

dipocytokine signaling pathway|PPAR signaling pathway|Quorum sensing|Fatty acid

rane signaling receptor activity|ATP binding|cytoplasm|plasma membrane|integral
of glycogen biosynthetic process|positive regulation of glycogen (starch) syntha

negative regulation of cell proliferation|collagen fibril organization|collagen-

ntegral component of membrane|growth factor binding|regulation of epidermal grov
ng|nucleus|regulation of transcription, DNA-templated|zinc ion binding|negative

ion|Bile secretion
plasma membrane|bicellular tight junction|integral component of membrane|spinal

region|extracellular space|intracellular|protein targeting|transforming growth f

eticulum membrane|Golgi apparatus|microtubule organizing center|plasma membrane|

ated transport|protein transport|Golgi transport complex|trans-Golgi network mem
NA polymerase II-specific|DNA binding|nucleoplasm|cytosol|nuclear membrane|histo

ng|cell adhesion|multicellular organism development|positive regulation of cell-
moter DNA-binding transcription activator activity, RNA polymerase II-specific|s

transduction|Ral GTPase binding|positive regulation of GTPase activity

embrane|regulation of protein stability|cellular response to drug|negative regul
region DNA binding|motile cilium assembly|positive regulation of transcription,
ce|anatomical structure development
port into nucleus|nuclear localization sequence binding|Ran GTPase binding|nucle
n|cytoskeleton|plasma membrane|protein dephosphorylation|cytoskeletal protein bi
ulin secretion|Vascular smooth muscle contraction|cGMP-PKG signaling pathway
thiol-dependent ubiquitinyl hydrolase activity|proteasome assembly|positive reg

-4,5-bisphosphate binding|cytoplasm|plasma membrane|microvillus|brush border|ep
dhesion
ay|Adherens junction|Osteoclast differentiation|Hippo signaling pathway|TGF-bet

of membrane|carbohydrate binding
zation
ent of plasma membrane|cell surface receptor signaling pathway|G protein-couplec

RNA binding|nucleus|nucleoplasm|RNA lariat debranching enzyme activity|metal ic

quitination|mitotic cell cycle|ubiquitin-protein transferase activity|centrosome
ıbrane|focal adhesion|negative regulation of cell proliferation|response to musc

oximal promoter sequence-specific DNA binding|proximal promoter DNA-binding tran
s
way
ing pathway
of protein phosphorylation|protease binding|regulation of oxidative phosphoryla
fic binding|intracellular signal transduction|actin filament reorganization
ndoplasmic reticulum|Golgi apparatus|plasma membrane|integral component of plasm
ty|interleukin-1 receptor activity|extracellular region|plasma membrane|immune

asma membrane|integral component of plasma membrane|cellular amino acid metabol
region|extracellular space|intracellular|nucleus|secondary lysosome|multivesicu

l component of Golgi membrane|integral component of endoplasmic reticulum membr

egral component of membrane|metal ion binding|ubiquitin protein ligase activity
apparatus|UDP-N-acetylglucosamine metabolic process|carbohydrate transport|int

tion|Salivary secretion|Calcium signaling pathway|Insulin secretion|Regulation
ell proliferation|neutrophil degranulation|ficolin-1-rich granule lumen

ndosome|early endosome|trans-Golgi network|plasma membrane|retrograde vesicle-me
|mitotic spindle organization|centriole replication|cerebral cortex development
membrane, translocation|integral component of membrane|collagen biosynthetic pr
rane
pathway|Cytosolic DNA-sensing pathway|T cell receptor signaling pathway|TNF si
or signaling pathway|brain development|astrocyte development|microglia developm

athway|Signaling pathways regulating pluripotency of stem cells

ation of glycolytic process|transcription by RNA polymerase II|mRNA processing|
aling pathway|MAPK signaling pathway|Gap junction|Rap1 signaling pathway|Calciu
utophagosome|cytosol|ubiquitin-dependent protein catabolic process|positive regu
resorption|regulation of neuronal synaptic plasticity
gral component of plasma membrane|focal adhesion|transmembrane receptor protein

ar junction development|zinc ion binding|protein ubiquitination|cell junction|ne
nvolved in DNA repair|regulation of transcription, DNA-templated|nuclear speck|
sion|membrane

: reticulum lumen|endoplasmic reticulum membrane|B cell differentiation|ubiquiti
:ion elongation factor complex|snRNA transcription by RNA polymerase II
'alcium signaling pathway|MAPK signaling pathway
ıction|Ran GTPase binding|protein transporter activity|regulation of gene silenc
e metabolism|Arginine biosynthesis

ılum lumen|heparin binding|BMP signaling pathway|response to starvation|post-tra
integral component of membrane|late endosome membrane|lysosome localization|den
ı membrane|drug transmembrane transport|organic cation transmembrane transporter
:ein transport|membrane|endocytic recycling|trans-Golgi network membrane|retrogr
thesis|Various types of N-glycan biosynthesis
ʼity
structural constituent|extracellular region|extracellular space|endoplasmic reti

y|Wnt signaling pathway|Signaling pathways regulating pluripotency of stem cell

ı|Golgi cisterna membrane
sma membrane|positive regulation of cytosolic calcium ion concentration|lamellip

:ion|cell adhesion|homophilic cell adhesion via plasma membrane adhesion molecul
ʼtoplasmic microtubule|intracellular protein transport|membrane|response to bref
ıg|extracellular region|basement membrane|cell-matrix adhesion|positive regulati
:ranscription factor activity, RNA polymerase II-specific|DNA-binding transcript
ge ribosomal subunit|ribosomal large subunit biogenesis|ribosomal small subunit

ʼity|ligase activity|metal ion binding|ubiquitin-dependent endocytosis

transendothelial migration|Axon guidance|Rap1 signaling pathway|Neurotrophin s

.asm|cytoplasm|intermediate filament|plasma membrane|cell-cell junction|adherens

golipid biosynthesis - lacto and neolacto series
ıding|nucleus|regulation of gene expression|chromatin DNA binding|sequence-speci

.ng|cerebral cortex radially oriented cell migration|forebrain ventricular zone
.opment|transcription corepressor activity|nucleus|transcription factor complex|
. adherens junction|exocytosis|Golgi to plasma membrane transport|integral compo
:ess|proteasome regulatory particle, lid subcomplex|ubiquitin binding|proteasome
:|Golgi apparatus|cytosol|protein transport|kinesin binding|transport vesicle|re

ınsporter activity|ATP binding|nucleus|nucleoplasm|Golgi apparatus|spindle|kines
sitive regulation of canonical Wnt signaling pathway
.ty|peptidyl-lysine trimethylation|protein-containing complex
se activity|cytosol|ubiquitin-dependent protein catabolic process|Wnt signaling
athway
. cytotoxicity
:oxyribonuclease activity|transposition, RNA-mediated|reverse transcription invo
.thway|Antigen processing and presentation|Hematopoietic cell lineage

:omponent of membrane|apical plasma membrane|eye photoreceptor cell development|
phospholipid binding|plasma membrane|integral component of plasma membrane|regu

[illegible] regulatory region sequence-specific DNA binding|DNA-binding transcription fact

[illegible] smooth muscle contraction|Olfactory transduction|Salivary secretion|Long-term
:egral component of membrane|heme binding

of cell polarity|signal transduction|activation of adenylate cyclase activity|ac
:ransport particle B|motile cilium|axoneme assembly|intraciliary anterograde tra
pathway
:egral component of plasma membrane|ion transport|cellular iron ion homeostasis|

:an biosynthetic process|cell adhesion|negative regulation of fibroblast migrati
insport|protein domain specific binding|trans-Golgi network membrane|regulation

r lipid metabolism|Phospholipase D signaling pathway|Sphingolipid metabolism|Fc

rocessing in endoplasmic reticulum|Spliceosome|Antigen processing and presentat
ibulin complex assembly|tubulin binding|prefoldin complex|intracellular membrane
:-type endopeptidase activity|intracellular|response to xenobiotic stimulus|inte

ome|regulated exocytosis|synaptic vesicle membrane organization
:ane|cytoplasmic vesicle|phosphatidylinositol-3-phosphate binding|autophagosome
:gic synapse|postsynaptic specialization

pase D signaling pathway|Neuroactive ligand-receptor interaction

oundle|skeletal system morphogenesis|negative regulation of chondrocyte developm

:ane adhesion molecules|heterophilic cell-cell adhesion via plasma membrane cell

:ticulum|protein folding

ortex|protein targeting|G protein-coupled receptor signaling pathway|chemical sy

G protein-coupled receptor signaling pathway|negative regulation of adenylate c

ton|Phosphatidylinositol signaling system

signaling pathway
ranscription factor activity, RNA polymerase II-specific|DNA-binding transcript
racellular signal transduction

binoid signaling|Neuroactive ligand-receptor interaction
sol|intracellular protein transport|Rab GTPase binding|activation of GTPase acti
ivity|plasma membrane|signal transduction|integral component of membrane|negati
eoxyribonuclease activity|transposition, RNA-mediated|reverse transcription invo
NA splicing, via endonucleolytic cleavage and ligation|mRNA processing
hetic process|ER to Golgi vesicle-mediated transport|lipid binding|phosphatidyl
n|Bile secretion
k|microtubule|protein glycosylation|ER to Golgi vesicle-mediated transport|micr
taminyl-peptide cyclotransferase activity|peptidyl-pyroglutamic acid biosynthet

ximal promoter sequence-specific DNA binding|DNA-binding transcription factor a

ling, bridging|establishment of epithelial cell polarity
aling pathway|negative regulation of signal transduction|membrane
ndopeptidase activity|integral component of membrane|enzyme binding|positive re

to mesenchymal transition|DNA binding|chromatin binding|DNA-binding transcripti
tinamide metabolism

tracellular matrix organization|embryonic eye morphogenesis|post-embryonic eye
tracellular matrix organization|embryonic eye morphogenesis|post-embryonic eye
dhesion

otein kinase activity|protein tyrosine/serine/threonine phosphatase activity|pc
gion of cytoplasm|regulation of mitochondrial fission
yrosine kinase signaling pathway|extracellular exosome

g pathway|Regulation of actin cytoskeleton|Neuroactive ligand-receptor interact
ximal promoter sequence-specific DNA binding|DNA-binding transcription factor a
nal side of plasma membrane|oxidoreductase activity
|Rap1 signaling pathway|Phospholipase D signaling pathway|Regulation of actin c

ng|nucleus|stem cell population maintenance|neural tube development|embryonic f
ss|cytoplasmic vesicle|FasL biosynthetic process
uidance|positive regulation of MAPK cascade|regulation of neurotrophin TRK rece
ipid biosynthesis - lacto and neolacto series

in signaling pathway|Ubiquitin mediated proteolysis|Prolactin signaling pathway

maturation|Cell cycle - yeast|Cell cycle
pathway

;
:ive regulation of fibroblast growth factor receptor signaling pathway|negative
.ng|nucleus|metal ion binding
ıctural constituent of muscle|actin cytoskeleton|Z disc|metal ion binding

:ion|eukaryotic initiation factor 4E binding|cell proliferation|translation acti
rity|plasma membrane|integral component of plasma membrane|locomotory behavior|p
naling pathway - fly
ırly endosome|cytosol|negative regulation of epithelial cell migration|protein t
. signaling pathway|Cell cycle
egulating pluripotency of stem cells|TGF-beta signaling pathway

port
:ytoplasm|cytosol|phagocytosis, engulfment|actin filament organization|signal tr

ızyme activator activity|zinc ion binding|extracellular matrix|anchored componen
j'-3' RNA polymerase activity|mitochondrion|mitochondrial matrix|mitochondrial t
evelopment|RNA binding|mRNA 3'-UTR binding|nucleoplasm|cytosol|mRNA processing|c
nthesis
.ght junction|exocytosis|neurotransmitter secretion|cytoskeletal protein binding

;ative regulation of autophagosome assembly

endoplasmic reticulum|cytosol|microtubule associated complex|plasma membrane|pro
rsions|Galactose metabolism|Fructose and mannose metabolism
otion factor activity, RNA polymerase II-specific|RNA polymerase II repressing t

.lular tight junction|DNA replication-independent nucleosome assembly|regulation
olasm|negative regulation of cell migration|actomyosin structure organization|my

:y|plasma membrane|carbohydrate metabolic process|sodium ion transport|hexose tr
. migration|wound healing, spreading of cells|negative regulation of integrin bi
anine metabolism|Valine, leucine and isoleucine degradation|Carbon metabolism
tory transduction
nnose metabolism
. apparatus|trans-Golgi network|cytosol|plasma membrane|intracellular protein tr
:ation|Golgi organization|membrane|integral component of membrane|identical prot

ng pathway|ECM-receptor interaction|Focal adhesion
.des

ı|nucleus|cytoplasm|endoplasmic reticulum|plasma membrane|clathrin-coated pit|se
signaling pathway

:ein serine/threonine kinase activity|establishment of mitotic spindle orientati
cell receptor signaling pathway|Natural killer cell mediated cytotoxicity|Dopam

ɔdistal appendage

ıid biosynthesis - ganglio series|Other glycan degradation|Glycosphingolipid bio
ubiquitin-protein transferase activity|nucleus|cytoplasm|mitochondrion|cytosol|
:enance
:tivity|cytoplasm|cytoplasmic side of plasma membrane|extracellular exosome

ıtal ion binding
.atory element binding|RNA polymerase II regulatory region sequence-specific DN/

ss|protein ubiquitination|Cul3-RING ubiquitin ligase complex|ion homeostasis|ren

ɔrt vesicle membrane|cytoplasmic vesicle membrane|secretory granule membrane|ear
.lic cell adhesion via plasma membrane adhesion molecules|chemical synaptic trar

:ivity|drug:proton antiporter activity|organic cation transport|integral compone

follicle development|DNA binding|transcription coactivator activity|nucleoplasm|
ling|cell|C-terminal protein-tyrosinylation|regulation of axon extension|positiv
:ic signaling pathway|regulation of NIK/NF-kappaB signaling

y|Wnt signaling pathway|Signaling pathways regulating pluripotency of stem cell

sium channel activity|potassium channel activity|plasma membrane|integral compor
plasma membrane|clathrin-coated pit|microtubule binding|cytoskeletal protein bi
se II distal enhancer sequence-specific DNA binding|proximal promoter DNA-bindir

ction|Hippo signaling pathway - fly|Dopaminergic synapse|Oocyte meiosis|Long-te

naling pathway|Platelet activation|Ras signaling pathway

esponse|negative regulation of NF-kappaB transcription factor activity|Pyrin dom

ase activity|GTP binding|cytosol|translational elongation|GTP metabolic process|
ation of cell migration|sequestering of actin monomers

hate pathway|Carbon metabolism

okine signaling pathway|Longevity regulating pathway|AMPK signaling pathway|Ins

lation of actin cytoskeleton|Focal adhesion
ocytosis|zinc ion binding|Rab guanyl-nucleotide exchange factor activity|Rab GTF
tabolism of xenobiotics by cytochrome P450
nesis|negative regulation of RNA splicing|erythrocyte maturation|regulation of c

ell adhesion|UV protection|regulation of collagen metabolic process|elastic fibe
g microtubule|cytoplasmic side of lysosomal membrane|BORC complex
tin cytoskeleton|membrane|extracellular exosome

ein acetylation|cell proliferation|male gonad development|response to toxic subs
ponse to lipopolysaccharide|response to tumor necrosis factor|white fat cell dif

peptidylglycine monooxygenase activity|peptidylamidoglycolate lyase activity|cop
|Retrograde endocannabinoid signaling

cadian entrainment|Salivary secretion|Purine metabolism|Long-term depression|Pl
n|Golgi cisterna membrane

thesis|Various types of N-glycan biosynthesis

organization|skeletal muscle tissue development|actin cytoskeleton|midbody|clea

esis|morphogenesis of an epithelium|DNA binding|DNA-binding transcription factor

ndosomal transport|AP-type membrane coat adaptor complex|late endosome membrane
binding|late endosome to Golgi transport|melanosome|positive regulation of GTPa

transcription factor activity, RNA polymerase II-specific|proximal promoter DNA-

ecules (CAMs)|Leukocyte transendothelial migration|TNF signaling pathway|NF-kap
s, NAD or NADP as acceptor
ted|protein methylation|methyltransferase activity|histone-arginine N-methyltran

ıbrane|Notch signaling pathway|structural constituent of muscle|dystrophin-assoc

›inding|extracellular region|extracellular space|nucleoplasm|cytoplasm|plasma me
binding|intracellular|plasma membrane|cell surface receptor signaling pathway|r
ı membrane|sodium ion transport|amino acid transport|amino acid transmembrane tı

ılum|endoplasmic reticulum membrane|cytosol|lipid binding|negative regulation oi
:eticulum|Golgi apparatus|kinesin complex|microtubule|retrograde vesicle-mediate
ıment|extracellular exosome|cornification

ng pathway|death receptor binding|nucleus|cytoplasm|cytosol|plasma membrane|apc
:t|voltage-gated potassium channel complex|integral component of membrane|perinu
.ral process|protein localization to phagophore assembly site|ion channel bindir

.thway|Neuroactive ligand-receptor interaction|Vascular smooth muscle contractio
:gral component of plasma membrane|membrane|heme binding|smooth endoplasmic reti
›ort|perinuclear region of cytoplasm|recycling endosome
pathway
ıscription factor complex|focal adhesion|signal transduction|animal organ morphc

› binding|nucleus|nucleolus|cytosol|mRNA polyadenylation|snRNA processing|nuclea
:ranscription factor activity, RNA polymerase II-specific|nucleus|regulation of

negative regulation of signal transduction|axon|dendritic shaft|perikaryon|denc

steroid catabolic process|identical protein binding|testosterone 17-beta-dehydı
:osol|plasma membrane|vesicle docking involved in exocytosis|protein transport|a
golipid biosynthesis - globo series|Mucin type O-Glycan biosynthesis|Glycosphin
.de binding
ion|Bile secretion
y|Wnt signaling pathway|Signaling pathways regulating pluripotency of stem cell
e metabolism|Arginine biosynthesis
:otein phosphorylation|positive regulation of protein autophosphorylation|positi

:ide channel activity|voltage-gated chloride channel activity|chloride channel a
lifferentiation
ısm|regulation of DNA replication|membrane|viral process|nuclear membrane|defens

›tein-containing complex|regulation of protein binding|post-translational protei
gulatory region sequence-specific DNA binding|DNA-binding transcription factor a

ıse activity|protein tyrosine kinase activity|ATP binding|nucleus|cytoplasm|cytc

ion|p53 signaling pathway|Cell cycle

:llular response to calcium ion

ıg|extracellular region|basement membrane|plasma membrane|cell adhesion|cell-mat
cannabinoid signaling|Long-term depression|Neuroactive ligand-receptor interact
binding|double-strand break repair via homologous recombination|nuclear chroma
omponent of plasma membrane|clathrin-coated pit|endocytosis|signal transduction|
way|Taste transduction|Serotonergic synapse

ess|phospholipase A1 activity|integral component of membrane|lipase activity|sh
TCA cycle)
R-mediated phagocytosis
early endosome|late endosome|cytosol|plasma membrane|cell cortex|intracellular
on coactivator activity|nuclear replication fork|positive regulation of transcr

inding|regulation of type I interferon production|interferon-beta production|re
n|plasma membrane|glycosaminoglycan biosynthetic process|glycosaminoglycan cata

sodium:phosphate symporter activity|plasma membrane|integral component of plasm
tinamide metabolism
|superoxide metabolic process|phosphatidylinositol-5-phosphate binding|superoxi

oxyribonuclease activity|transposition, RNA-mediated|reverse transcription invo
organizing center|cytosol|multicellular organism development|spermatogenesis|po
rotein ubiquitination|Cul3-RING ubiquitin ligase complex|regulation of cytokine

inner membrane|proteolysis|regulation of mitochondrion organization|integral c
istone acetyltransferase activity|ATP binding|nucleus|nucleoplasm|Golgi apparat
exocytosis|serine-type peptidase activity|axon|dendrite|cytoplasmic vesicle|zym

ne|midbody|wound healing|positive regulation of cell cycle|epithelial cell prol
ogenesis|DNA-binding transcription factor activity|nucleus|regulation of transc

binding|RNA binding|chromatin silencing complex|nucleolus|cytosol|DNA methylat
e activity|cytoplasm|ubiquitin-dependent protein catabolic process|transforming

equence-specific DNA binding|metal ion binding
LSU-rRNA)|RNA binding|ATP binding|nucleoplasm|nucleolus|rRNA processing|ATP-dep
ximal promoter sequence-specific DNA binding|DNA-binding transcription factor a
lasm|cytoskeleton|microtubule binding|regulation of autophagy|apical plasma mem

ated fatty acids|Fatty acid metabolism|PPAR signaling pathway|AMPK signaling pa
pathway
transport, endosome to Golgi|phospholipid translocation
mediate compartment|cytosol|plasma membrane|retrograde vesicle-mediated transpo
ne|cytosol|plasma membrane|ubiquitin-dependent protein catabolic process|zinc io
ctivity|integral component of membrane|basolateral plasma membrane|intracellula
ption by RNA polymerase I|transcription initiation from RNA polymerase I promote

ng|nucleus|metal ion binding

vity|nucleus|cytoplasm|mitochondrial intermembrane space|cytosol|protein import

pment|intracellular|brain development|heart development
ption factor activity, RNA polymerase II-specific|nucleoplasm|anterior/posterior
sma membrane|cell cycle|chromosome segregation|membrane|midbody|late endosome me

vity regulating pathway|Ovarian steroidogenesis|Platelet activation|Progesteron
nulation
cing 5'-phosphomonoesters|IRE1-mediated unfolded protein response|metal ion bin
gnaling pathway|Wnt signaling pathway|Signaling pathways regulating pluripotenc

n-like growth factor binding|extracellular region|cytoplasm|gap junction|cell ac
sm|mitochondrion|cytosol|tricarboxylic acid cycle|citrate metabolic process|regu
c|cytoplasm|mRNA cleavage factor complex|termination of RNA polymerase II transc

ucleus|cytosol|plasma membrane|caveola|negative regulation of protein kinase act

ng|nucleus|spinal cord motor neuron cell fate specification|visceral motor neur

randed DNA binding|RNA binding|nucleus|nuclear euchromatin|nucleosome assembly|r
:|positive regulation of transcription via serum response element binding|NAD(P)
cleotide excision repair|Purine metabolism|Pyrimidine metabolism|Base excision
ponent of membrane

biquitin-protein transferase activity|cytoplasm|endoplasmic reticulum membrane|c
brane

|RNA transport
signaling pathway|TGF-beta signaling pathway|Signaling pathways regulating plur

ng|nucleus|metal ion binding

perception of sound|cell death|negative regulation of cell proliferation|membran
n|ubiquitin protein ligase activity

ɘptide metabolic process|protein processing involved in protein targeting to mit

:ion assembly|cell adhesion|homophilic cell adhesion via plasma membrane adhesic

ı-like protein binding|protein neddylation|positive regulation of ubiquitin-prot

.ty|nucleus|nucleoplasm|cytosol|double-strand break repair|double-strand break ı

Methane metabolism|Vitamin B6 metabolism|Biosynthesis of amino acids

-directed 5'-3' RNA polymerase activity|nucleus|nucleoplasm|chromosome|nucleolus
)r activity|nucleus|regulation of transcription, DNA-templated|regulation of tra

ıding|Golgi apparatus|trans-Golgi network|calcium ion transport|manganese ion tı
:anded RNA binding|nucleus|nucleoplasm|meiotic cell cycle
 behavior|protein secretion|cell junction|AMPA glutamate receptor complex|posit

.in signaling pathway|Focal adhesion
ǵi apparatus|centrosome|microtubule binding|membrane|cytoskeleton-dependent intı
: chromosome, telomeric region|chromatin|nucleus|nuclear envelope|nuclear inner

ɘsion|microtubule binding|postsynaptic density|actin cytoskeleton|epithelial cel
tabolism of xenobiotics by cytochrome P450
:anscriptional repressor complex|regulation of cell cycle|DNA biosynthetic proce

:ive regulation of translational initiation|N6-methyladenosine-containing RNA bi
ınscription factor complex|regulation of transcription by RNA polymerase II|mult
.vity|cytoplasm|cytosol|intermediate filament|cytoskeleton organization|signal t
:egral component of membrane|ATPase activity|metal ion binding
 protein homooligomerization|protein heterooligomerization

)orso-ventral axis formation

ıigration|Tight junction
ıridine editing
.kt signaling pathway|ECM-receptor interaction|Focal adhesion

;|enzyme activator activity|zinc ion binding|glial cell differentiation|extracel

:eticulum membrane|regulation of store-operated calcium entry
:on transport chain|proton transmembrane transport

cytoplasm|regulation of mRNA stability|metal ion binding|negative regulation of
ome assembly

tochondrial intermembrane space|mitochondrial calcium ion transmembrane transpo
regulation of protein localization to cilium|negative regulation of protein loca
vity|ATP binding|nucleus|cytoplasm|alanyl-tRNA aminoacylation|regulation of tran
ng|nucleus|protein domain specific binding|metal ion binding|neuron fate commit
phospholipid binding|plasma membrane|regulation of dopamine secretion|integral

ore|cytoplasm|spindle organization|chromosome segregation|microtubule binding|mi
nding|nucleus|regulation of gene expression|chromatin DNA binding|sequence-speci
ption by RNA polymerase III|RNA splicing|response to virus|actin cytoskeleton|vi
asmic reticulum lumen|camera-type eye development|post-translational protein mo

abolic process|SCF ubiquitin ligase complex|intracellular signal transduction|pr

beta receptor complex assembly|keratan sulfate biosynthetic process|extracellu
on
binding|O-methyltransferase activity|RNA methyltransferase activity|S-adenosylme

ntegral component of membrane|vesicle-mediated transport|COPII-coated ER to Golg

ation|clathrin-coated vesicle|retrograde transport, endosome to Golgi|protein ho

growth|ESC/E(Z) complex|cellular heat acclimation

ng|nucleus|lymphocyte differentiation|positive regulation of natural killer cel

ng|metallocarboxypeptidase activity|extracellular space|cytoplasm|endoplasmic re
gative regulation of apoptotic process|regulation of calcium-mediated signaling

embly|Hsp70 protein binding|motile cilium|sperm flagellum|cilium organization|ex

ral process|keratinization|keratin filament|extracellular exosome|cornification
-4,5-bisphosphate binding|phosphatidylinositol-3,4,5-trisphosphate binding|intr

negative regulation of viral-induced cytoplasmic pattern recognition receptor si
cle

ucleoplasm|cytoplasm|cytosol|plasma membrane|apoptotic process|nuclear speck|neg
tokine signaling pathway|FoxO signaling pathway|PI3K-Akt signaling pathway|mTOR

ng|nucleus|metal ion binding
pathway
Glycosaminoglycan biosynthesis - chondroitin sulfate / dermatan sulfate

ntegral component of membrane|apical plasma membrane|ion channel binding|regula
binding|ATP binding|nucleus|cytoplasm|DNA replication|DNA repair|DNA recombinat

tion factor activity, RNA polymerase II-specific|DNA-binding transcription fact
limerization activity
basal cortex|regulation of microtubule cytoskeleton organization|positive regula
-mediated signaling|positive regulation of insulin secretion involved in cellul
tion|transforming growth factor beta binding|collagen-containing extracellular

cycle|Ubiquitin mediated proteolysis|Cell cycle - yeast|Meiosis - yeast

ding|nucleus|regulation of gene expression|chromatin DNA binding|sequence-speci
cid binding|metal ion binding
development|spinal cord association neuron differentiation|anchored component
itochondrion organization|skeletal muscle satellite cell differentiation|skelet

binding|nuclear pore|nucleoplasm|nucleolus|cytoplasm|cytosol|plasma membrane|mi

ranscription activator activity, RNA polymerase II-specific|DNA binding|nucleus
on|cellular response to L-arginine|negative regulation of TORC1 signaling

gnaling pathway

pH|sodium:proton antiporter activity|potassium:proton antiporter activity|integ
ular space|lysosome|carbohydrate metabolic process|extracellular exosome

n specification|dorsal/ventral pattern formation|peptide transport|integral com

em development|extracellular matrix structural constituent conferring compressi
rganism development|sperm axoneme assembly|microtubule binding|protein polyglut
ng transcription factor activity|lysosome|cell-cell adherens junction|transcrip

ng|nucleoplasm|nucleolus|metal ion binding
dase activity|mitochondrion|metal ion binding|N(6)-L-threonylcarbamoyladenine s

r membrane|mitochondrial small ribosomal subunit|translation|cell junction|mito

yrimidine nucleoside salvage|pseudouridine 5'-phosphatase activity

l cycle|cellular response to UV|positive regulation of G0 to G1 transition|cell

onent of membrane

sma membrane|positive regulation of cytosolic calcium ion concentration|negative
r|nucleus|endoplasmic reticulum lumen|plasma membrane|G protein-coupled receptor

network|zinc ion binding|integral component of membrane

ation
ay|Ras signaling pathway|PI3K-Akt signaling pathway|Focal adhesion
ntermediate filament|central nervous system development|brain development|interm

osol|clathrin-coated pit|protein phosphorylation|endocytosis|extrinsic component

ne
dral bone growth|DNA binding|nucleoplasm|tongue development|metal ion binding|ro

transcription factor activity, RNA polymerase II-specific|proximal promoter DNA-

transcription factor activity, RNA polymerase II-specific|proximal promoter DNA-
plasm|microtubule organizing center|cytosol|ER to Golgi vesicle-mediated transpo
stem development|microtubule cytoskeleton|axon initial segment
regulation of transcription involved in G2/M transition of mitotic cell cycle|ne

fferentiation|Apoptosis

ne|phosphatidylinositol transporter activity|integral component of membrane|cort
ic process|positive regulation of gene expression|regulation of mitochondrial f

ng|nucleus|positive regulation by host of viral release from host cell|positive

on|Fatty acid metabolism
collagen type VIII trimer|extracellular space|endoplasmic reticulum lumen|cell a

response to antigenic stimulus|chromatin binding|nucleus|nucleoplasm|chromatin

and isoleucine degradation|Fatty acid degradation
pathway|signaling receptor binding|nucleus|nucleoplasm|cytoplasm|cytosol|chromat

ller cell mediated cytotoxicity
g|protein kinase activity|protein serine/threonine kinase activity|ATP binding|r

.ng|DNA-binding transcription factor activity|nucleus|nucleoplasm|cytoplasm|sper
.thway|Fc epsilon RI signaling pathway|Fc gamma R-mediated phagocytosis|Ras sign

le

ıding|nucleus|regulation of gene expression|chromatin DNA binding|sequence-speci

junction assembly|cell surface|fibroblast migration
ansporter activity|L-alanine transmembrane transporter activity|L-proline transm

ling|positive regulation of cell proliferation|transferase activity|ubiquitin cc
on|PML body|transferase activity|stem cell population maintenance|negative regul
.g pathway|Cell cycle|Melanogenesis|Wnt signaling pathway|cAMP signaling pathway

athway|Signaling pathways regulating pluripotency of stem cells

response|polysaccharide binding|collagen-containing extracellular matrix
d degradation|Tryptophan metabolism|Lysine degradation|Synthesis and degradatio
tyrosine and tryptophan biosynthesis|Isoquinoline alkaloid biosynthesis|Tyrosin
rganizing center|cytosol|plasma membrane|intracellular protein transport|Rab GTI

plasm|extracellular exosome

.lic cell adhesion via plasma membrane adhesion molecules
e
rodimerization activity

eoxyribonuclease activity|transposition, RNA-mediated|reverse transcription invc
.ng|nuclear body|metal ion binding

.vity|plasma membrane|chloride transport|ion transmembrane transport|chloride ch
es|Calcium signaling pathway|Thyroid hormone signaling pathway|cGMP-PKG signali
ion|Ras signaling pathway

.cing|positive regulation of RNA splicing
acuolar transport|negative regulation of gene expression|integral component of m

ative regulation of endopeptidase activity|positive regulation of transforming g
scription factor activity, RNA polymerase II-specific|DNA-binding transcription
.ar signal transduction|metal ion binding|regulation of small GTPase mediated si

cytosis|neurotransmitter secretion|postsynaptic density|protein transport|basola

liated by cadherin|zonula adherens maintenance|extracellular exosome|delta-cater

twork for IgA production|NF-kappa B signaling pathway

.ng|nucleus|metal ion binding
.y|cilium assembly|establishment of epithelial cell polarity|ciliary transition

.d metabolic process|sphinganine-1-phosphate aldolase activity|carboxy-lyase act
ıding|proximal promoter DNA-binding transcription activator activity, RNA polyme

.podium|cell junction|actin cytoskeleton reorganization|early endosome membrane|

c synapse|Retrograde endocannabinoid signaling|GABAergic synapse|Dopaminergic s

.ng center|cytosol|plasma membrane|cell cortex|vesicle fusion|positive regulatic
.ation

regulation of Rab protein signal transduction

ɔrter activity|cytoplasm|plasma membrane|integral component of plasma membrane|c
|ECM-receptor interaction|Focal adhesion
.e contraction|muscle organ development|structural constituent of muscle|actin c
ɔss|cytoplasmic vesicle|FasL biosynthetic process

ling|integral component of membrane|protein ubiquitination|transferase activity
se II proximal promoter sequence-specific DNA binding|DNA-binding transcription

gulatory region sequence-specific DNA binding|DNA-binding transcription factor a
.ar tight junction|positive regulation of cell proliferation|apical plasma membr
ıding|nucleus|regulation of gene expression|chromatin DNA binding|sequence-speci
gulation of lipid metabolic process|azurophil granule membrane|neutrophil degran
r interaction|Pancreatic secretion
ıne adhesion molecules|neuron cell-cell adhesion|nervous system development|axor
en interaction
binding|ATP-dependent DNA helicase activity|histone deacetylase activity|ATP b

ıding|nucleus|nucleoplasm|cytosol|DNA replication|DNA replication-dependent nucl
loplasmic reticulum membrane|cytosol|plasma membrane|protein phosphorylation|cel
sol|cellular protein modification process|ubiquitin-dependent protein catabolic
ɔr activity|cytoplasm|cytosol|plasma membrane|cell-cell junction|G protein-coupl
commitment|blastocyst growth|single-stranded DNA binding|RNA binding|nucleoplas
olism|Amino sugar and nucleotide sugar metabolism|Pentose phosphate pathway|Sta

, R-mediated phagocytosis

; center|cytosol|activation of JUN kinase activity|JUN kinase binding|vesicle-me

.on|mitochondrial matrix|regulation of acetyl-CoA biosynthetic process from pyru
;e activity|chromatin binding|ATP binding|nucleus|chromosome|cellular response t

)tein serine/threonine kinase regulator activity|mediator complex|positive regul
.sion
ısma membrane|negative regulation of cell growth|identical protein binding|posit
:nt of membrane|transport vesicle|protein binding, bridging

tabolism of xenobiotics by cytochrome P450

)tion factor activity, RNA polymerase II-specific|startle response|DNA binding|I
: activity|protein desumoylation|MLL1 complex
.ng|nucleus|negative regulation of transcription, DNA-templated|metal ion bindir

:ceptor activity|type 1 cannabinoid receptor binding|myelin sheath|negative regu
ı|Glucagon signaling pathway|Carbon metabolism|Biosynthesis of amino acids|Glyco
)r activity|centrosome|cytosol|kinesin binding|myelination in peripheral nervous

binding|intracellular|cytoplasm|cytoskeleton|muscle contraction|dystrophin-asso
:in serine/threonine kinase activity|ATP binding|cytoplasm|Golgi apparatus|Golgi

ıatin|proximal promoter DNA-binding transcription activator activity, RNA polyme
on fixation pathways in prokaryotes|2-Oxocarboxylic acid metabolism|Biosynthesi
hate pathway|Carbon metabolism
ısm|cytosol|Rab-protein geranylgeranyltransferase complex|intracellular protein

: plasma membrane|cell adhesion|cell surface receptor signaling pathway|blood cc

ıtophagy|extrinsic component of membrane|phosphatidylinositol-3-phosphate bindir

ing pathway|MAPK signaling pathway|Oocyte meiosis|Long-term potentiation|mTOR s

:etrograde axonal transport|positive regulation of mitochondrion organization|ax

cytoplasm|focal adhesion|protein phosphorylation|actin cytoskeleton|cell migrat
RNA from tricistronic rRNA transcript (SSU-rRNA, 5.8S rRNA, LSU-rRNA)|endonucle
osphatidylserine binding|protein serine/threonine kinase activity|ATP binding|p

athway
tivity|phospholipase A1 activity|integral component of membrane|lipid catabolic
y|intracellular|signal transduction|small GTPase mediated signal transduction|p
enta development|maternal placenta development|heart looping|endoplasmic reticu

ng|nucleus|nervous system development|metal ion binding

sion|signal transduction|coreceptor activity|BMP signaling pathway|identical pro

lase activity|Z disc

xocytosis|cytoskeleton organization|cell cycle|oxidoreductase activity, acting
on|Fatty acid metabolism
tamatergic synapse
-kappaB signaling|negative regulation of phosphatidylinositol 3-kinase signaling

athway

ne|regulation of transcription, DNA-templated|homophilic cell adhesion via plas
protein kinase activity|negative regulation of signal transduction|phosphatase

o-ventral axis formation

lism

tosol|plasma membrane|brain development|heart development|skeletal muscle tissu
in tyrosine kinase activity|ATP binding|extracellular region|Golgi apparatus|pr

ion binding|RNA phosphodiester bond hydrolysis, endonucleolytic

ergic synapse
ace|metalloendopeptidase inhibitor activity|negative regulation of endopeptidas
sma membrane|platelet alpha granule membrane
sma membrane|proteolysis|membrane|Wnt signaling pathway|Wnt-protein binding|nega
de antigen via MHC class I, TAP-dependent|ATP binding|protein transport|peptide

ptotic signaling pathway|positive regulation of cold-induced thermogenesis|posi

space|negative regulation of endopeptidase activity|membrane|secretory granule

steoclast differentiation|bone marrow development
region|collagen trimer|extracellular space|endoplasmic reticulum lumen|plasma m
d metabolism|AMPK signaling pathway

itin sulfate metabolic process|dermatan sulfate biosynthetic process|chondroiti
iculum
otubule organizing center|cytosol|lipid metabolic process|phosphatidylinositol
ximal promoter sequence-specific DNA binding|DNA-binding transcription factor a

Natural killer cell mediated cytotoxicity|Estrogen signaling pathway|Phospholip
ity regulating pathway - worm|Glyoxylate and dicarboxylate metabolism|Peroxisom
s
-Golgi network|cytosol|plasma membrane|retrograde vesicle-mediated transport, Gc

ino acids|Cysteine and methionine metabolism|Selenocompound metabolism
bon metabolism|AMPK signaling pathway|Biosynthesis of amino acids|Pentose phosp
nucleoplasm|lipid droplet|cytosol|vitamin A metabolic process|retinal binding|l
tion, DNA-templated|cell cycle arrest|axon guidance|transcription factor bindir

otein stability|regulation of neuron differentiation|negative regulation of smc
pathway|NF-kappa B signaling pathway
inding|positive regulation of epithelial to mesenchymal transition|regulation c
translational elongation|response to virus
signaling receptor binding|cytosol|plasma membrane|integral component of plasma
|chromatin binding|satellite DNA binding|chromosome|nuclear body|centromeric DN

ximal promoter sequence-specific DNA binding|DNA-binding transcription factor a
naling pathway - fly|Dopaminergic synapse|Adrenergic signaling in cardiomyocyte

nucleation|extracellular exosome

rix adhesion|regulation of systemic arterial blood pressure|extracellular matri

ding
atan sulfate
n degradation|Galactose metabolism|Sphingolipid metabolism|Glycosaminoglycan de

activation|Arginine and proline metabolism|Calcium signaling pathway|VEGF sign
polymerase II regulatory region sequence-specific DNA binding|DNA-binding tran
minoglycan binding|extracellular region|extracellular space|cytosol|blood coagu
culum membrane|Golgi apparatus|sarcoplasmic reticulum lumen|melanosome
hannel activity|response to mechanical stimulus|integral component of membrane|

ay - fly
lular region|extracellular space|heparin binding|cell surface|Wnt signaling pat
port|perinuclear region of cytoplasm|recycling endosome
. component of membrane|bile acid receptor activity|G protein-coupled bile acid
nding|nucleus|DNA-dependent DNA replication|regulation of DNA repair|DNA recomb
bination|protein sumoylation|SUMO transferase activity|metal ion binding|chiasma

ignaling pathway|Natural killer cell mediated cytotoxicity|Jak-STAT signaling p

DNA binding|RNA polymerase I general transcription initiation factor activity|in

n|cell-cell junction|focal adhesion|cytoskeleton organization|membrane|actomyosi
pathway|NF-kappa B signaling pathway

negative regulation of protein tyrosine kinase activity
tion assembly|homophilic cell adhesion via plasma membrane adhesion molecules|cy
activity|integral component of membrane|chloride ion homeostasis|potassium ion
stal enhancer sequence-specific DNA binding|DNA-binding transcription factor act
ance|B cell receptor signaling pathway|T cell receptor signaling pathway|cGMP-P

cus|protein transport|Rab protein signal transduction|negative regulation of axo

ne
rane|cell-matrix adhesion|muscle organ development|dystrophin-associated glycopr
ng|nucleus|metal ion binding
brane|oxidoreductase activity|electron transport chain|metal ion binding
tive regulation of double-strand break repair via homologous recombination|posit
e
ppo signaling pathway|TGF-beta signaling pathway
n|central nervous system development|dystrophin-associated glycoprotein complex|

nding|activation of GTPase activity
orption|Proximal tubule bicarbonate reclamation
zinc ion binding|integral component of membrane|vesicle-mediated transport

nelle

thway|Neurotrophin signaling pathway|Melanogenesis|Dopaminergic synapse|Adrener

ome binding|positive regulation of intracellular protein transport|cellular resp
asmic reticulum|Golgi apparatus|protein phosphorylation|response to bacterium|bi

beta signaling pathway|PI3K-Akt signaling pathway|ECM-receptor interaction|Foca
endent protein catabolic process|protein-containing complex binding|protein homo
sponse to organonitrogen compound|macroautophagy|integral component of mitochond
eotide exchange factor activity|cytoplasm|Golgi apparatus|cytosol|cytoskeleton|

ay|Gap junction|Rap1 signaling pathway|Phospholipase D signaling pathway|Ras si
kinase/NF-kappaB signaling|positive regulation of I-kappaB kinase/NF-kappaB sigr
l matrix|cytosol|glucuronoside catabolic process|cellular glucuronidation|mycop
|proteolysis|signal transduction|cell surface|synapse|extracellular exosome
eoxyribonuclease activity|transposition, RNA-mediated|reverse transcription invo
pathway|ErbB signaling pathway|Focal adhesion
asm|cytoplasm|regulation of DNA repair|postreplication repair|double-strand brea
gene expression|regulation of neuron projection development|positive regulation
cell receptor signaling pathway|Natural killer cell mediated cytotoxicity|Dopam

g|cytoplasm|Golgi apparatus|cytosol|plasma membrane|regulation of neuron projec
ng pathway|Longevity regulating pathway|AMPK signaling pathway|Insulin signalin
ng|DNA-binding transcription factor activity|nucleus|zinc ion binding
e activity|ATP binding|mitochondrion|mRNA polyadenylation|manganese ion binding
activity|calcium ion binding|glycosaminoglycan binding|extracellular space|endo
pathway|NF-kappa B signaling pathway

keleton|negative regulation of cell adhesion|Rho protein signal transduction|me

|lipoprotein transport|cilium assembly
ty|integral component of membrane|dendrite|endosomal lumen acidification|protei
ng|nucleus|nucleoplasm|cytoplasm|metal ion binding|protein dimerization activit

ilament|muscle contraction|membrane|myofibril|sarcomere|myofibril assembly|posi

ity|cellular response to leukemia inhibitory factor

athway|Ras signaling pathway|PI3K-Akt signaling pathway|NF-kappa B signaling pa

dies

cleus|nucleolus|cytoplasm|endoplasmic reticulum|Golgi apparatus|cytosol|sphing

|ECM-receptor interaction|Focal adhesion

asmic reticulum|MAPK signaling pathway - fly
sembly|cytoskeletal protein binding|negative regulation of phosphatase activity|

athway|Ras signaling pathway|PI3K-Akt signaling pathway|NF-kappa B signaling pa
ignaling pathway|Adherens junction|NOD-like receptor signaling pathway|TNF sign

binding|phosphatidylinositol-3-phosphate binding|phosphatidylinositol-3,4-bispł
ı of transcription, DNA-templated|nervous system development|transcription facto
ıtase activity

actin cytoskeleton|Ras signaling pathway|PI3K-Akt signaling pathway

s|Serotonergic synapse|Retrograde endocannabinoid signaling|GABAergic synapse|R
binding|DNA binding|nucleus|cell cycle|negative regulation of cell cycle|metal
ty|negative regulation of intrinsic apoptotic signaling pathway in response to

|integral component of membrane

eolus|cytoplasm|cytoplasmic stress granule|transposition, RNA-mediated|cytoplas
ration|Axon guidance|GABAergic synapse|Rap1 signaling pathway|Long-term depress

ular|nucleus|nucleoplasm|nucleolus|cytoplasm|cytosol|plasma membrane|apoptotic

se|protein-tyrosine sulfotransferase activity|membrane|integral component of Gol

pathway|Osteoclast differentiation|Prolactin signaling pathway
ion elongation from RNA polymerase II promoter|NELF complex|negative regulatior

1 signaling pathway|Long-term depression|Platelet activation|Sphingolipid signa

oglycan binding|extracellular region|Golgi lumen|cell surface|peptide cross-linł
negative regulation of gluconeogenesis|extracellular exosome

eoplasm|cytoplasm|lysosomal membrane|Golgi apparatus|cytosol|actin filament|pla

is|Base excision repair
ıne|metal ion binding

ammatory response wound healing|platelet degranulation|cytokine activity|extrac
ıe|Golgi organization|signal transduction|actin cytoskeleton|cell junction|cytop

on of phosphoprotein phosphatase activity|COPII vesicle coating

:ivity|dynein light intermediate chain binding|cilium assembly|regulation of neu

; junction|cell-cell adherens junction|cell-cell junction assembly|heart develop

:ming growth factor beta receptor signaling pathway|negative regulation of SMAD
atelet activation|Vascular smooth muscle contraction|cGMP-PKG signaling pathway
ensity|protein ubiquitination|SCF ubiquitin ligase complex|cerebral cortex radia

:leus|RNA splicing|dosage compensation by inactivation of X chromosome|viral pro
signaling pathway|integral component of organelle membrane|positive regulation

ıidonic acid metabolism

:egulation of epidermal growth factor receptor signaling pathway|metal ion bindi
)inding
involved in exocytosis|autophagy|endosome organization|lysosome organization|enc
ation of cysteine-type endopeptidase activity involved in apoptotic process|regu

) lysosome transport|protein transport|interkinetic nuclear migration|cytoskelet
response to heat|negative regulation of Rho-dependent protein serine/threonine
sponse to UV

sduction|membrane

on coactivator activity|transcription corepressor activity|nucleus|nucleoplasm|t
a pathways in prokaryotes

cription factor activity|nucleus|nucleoplasm|regulation of transcription by RNA
utarate as one donor, and incorporation of one atom each of oxygen into both don

tyrosine kinase signaling pathway|cell proliferation|glial cell migration|cell

activity, RNA polymerase II-specific|proximal promoter DNA-binding transcription
abolism|Ovarian steroidogenesis|VEGF signaling pathway|Oxytocin signaling pathw
|microtubule bundle formation|calmodulin binding|cytoplasm|Golgi apparatus|centr

ATP binding|nucleoplasm|cytoplasm|mitochondrion|actin filament|plasma membrane|p
nsmembrane transporter activity|aminophospholipid transport|membrane|integral co

f plasma membrane|female pregnancy|lactation|negative regulation of signal trans
catabolic process|extracellular exosome|positive regulation of blood vessel diam

g|regulation of protein ubiquitination|negative regulation of NF-kappaB transcri
gG binding|beta-2-microglobulin binding
activity, RNA polymerase II-specific|nucleus|zinc ion binding|adult behavior|tra
embrane|phospholipase A2 inhibitor activity|hippocampus development|axon|dendrit

tination|ubiquitin conjugating enzyme binding|G-protein beta-subunit binding|pos
tor signaling pathway|locomotory behavior|regulation of G protein-coupled recept

secretion|Aldosterone-regulated sodium reabsorption|Proximal tubule bicarbonat
egulation of bone mineralization
ing on carbon-nitrogen (but not peptide) bonds|SH3 domain binding|lamellipodium
ndosome membrane|integral component of membrane|reproductive process|metal ion b
ynaptic vesicle|regulation of gene expression|protein transport|multicellular or

ipid metabolic process|phosphatidylinositol binding|regulation of apoptotic proc

yeast
urogenital system development|branching involved in ureteric bud morphogenesis|I

n binding|intracellular membrane-bounded organelle

activity, RNA polymerase II-specific|proximal promoter DNA-binding transcription

:ell activation|regulation of RNA splicing|protein kinase A binding

ne response|receptor antagonist activity|extracellular exosome|cellular response
;naling pathway|Prolactin signaling pathway

:omponent of plasma membrane|heart development|learning|feeding behavior|retina

cell mediated cytotoxicity|Ras signaling pathway|Focal adhesion|Wnt signaling p

ic reticulum|immune response|response to oxidative stress|heme binding|extracel
on by RNA polymerase II|multicellular organism development|visceral mesoderm-en

scorbic acid binding|collagen metabolic process|collagen biosynthetic process|ca

ion of gene expression|actomyosin structure organization|wound healing|apical pa

e lumen|intracellular membrane-bounded organelle

-binding transcription repressor activity, RNA polymerase II-specific|nucleus|re
response to oxidative stress|metal ion binding|negative regulation of cell death
gral component of membrane|protein homodimerization activity|intracellular memb

? binding|cytoplasm|plasma membrane|integral component of plasma membrane|prote[illegible]

:alytic step 2 spliceosome|catalytic step 2 spliceosome

nscription repressor activity, RNA polymerase II-specific|DNA-binding transcrip[illegible]

ocessing|protein ubiquitination|cell junction|estrogen receptor binding|small-su[illegible]
component of membrane|lamellipodium membrane|endoplasmic reticulum-Golgi interme[illegible]

otein phosphorylation|membrane|AU-rich element binding|negative regulation of tr[illegible]

of cell shape|integral component of membrane|ion transmembrane transport|phospho[illegible]

tor signaling pathway|negative regulation of adenylate cyclase activity|phosphol[illegible]

on-alpha|response to interferon-beta|negative regulation of viral genome replica[illegible]
naling pathway|Hedgehog signaling pathway|FoxO signaling pathway|Circadian rhyth[illegible]
ty|Rho protein signal transduction|regulation of cell shape|protein kinase bind[illegible]

olved in RNA-mediated transposition|metal ion binding|nucleic acid phosphodieste[illegible]

n mediated proteolysis|TGF-beta signaling pathway|Cell cycle - yeast|Circadian

l protein binding

ry response|opsonization|extracellular matrix organization|extracellular matrix

activity, RNA polymerase II-specific|transcription factor activity, RNA polymera[illegible]
ring protein import into nucleus|ribosomal protein import into nucleus|nuclear [illegible]
|anterograde synaptic vesicle transport|protein localization to axon|positive re[illegible]
lasmic ribonucleoprotein granule|myelin sheath|intermediate filament cytoskelet[illegible]
embrane|intracellular membrane-bounded organelle|postsynaptic membrane|positive

inase kinase kinase activity|signal transduction by protein phosphorylation|stre

dexamethasone

secretion|Circadian entrainment|Glutamatergic synapse|cAMP signaling pathway|MA
sphorylation|nervous system development|central nervous system development|respo
nding|regulation of epidermal growth factor receptor signaling pathway|regulatio

mbrane-bounded organelle|positive regulation of telomerase activity
ell adhesion molecules|cell surface receptor signaling pathway|G protein-couple

ession|PML body|protein sumoylation|negative regulation of transforming growth f

ding|ATP-dependent peptidase activity|serine-type endopeptidase activity|ATP bin

ane|integral component of plasma membrane|protein dephosphorylation|axonogenesis

nscription repressor activity, RNA polymerase II-specific|positive regulation of
timulus|MOZ/MORF histone acetyltransferase complex

ic DNA binding|transcription regulatory region DNA binding|negative regulation o
omophilic cell adhesion via plasma membrane adhesion molecules|cell surface|kin

ion|Platelet activation|Sphingolipid signaling pathway|Progesterone-mediated oc

ane|negative regulation of cell proliferation|negative regulation of GTPase act

COPII vesicle coating|protein complex oligomerization|TRAPPII protein complex

cid biosynthetic process|integral component of membrane|dipeptidase activity|neu

lymerase II|cell cycle|transcription factor binding|negative regulation of cell

lium assembly|dynein light chain binding|dynein heavy chain binding|embryonic sk
olic process|PML body|axon|negative regulation of axon extension|nuclear membran

tein kinase C binding|nucleus|nucleoplasm|cytoplasm|cytosol|cell-cell junction a
tosis|cytoskeleton organization|actin filament organization|establishment or mai

lifferentiation|Phospholipase D signaling pathway|Platelet activation|Sphingolip

n of blood pressure|positive regulation of T cell chemotaxis|kinase activity|bas
ucleus|protein homodimerization activity|perinuclear region of cytoplasm|interfe

ubiquitination|protein homodimerization activity|metal ion binding

process|cadherin binding|extracellular exosome|regulation of sprouting angiogen

protein phosphorylation|BMP signaling pathway|regulation of apoptotic process|re

vity|cytosol|plasma membrane|G protein-coupled receptor signaling pathway|small
nsduction|glutamatergic synapse|regulation of postsynapse organization
ding|PDZ domain binding|receptor tyrosine kinase binding|regulation of Rho prote

s|nucleoplasm|cellular response to DNA damage stimulus|sister chromatid cohesion

envelope lumen|endosome|early endosome|endoplasmic reticulum|Golgi apparatus|tra
ane|integral component of plasma membrane|adenylate cyclase-activating G protein
NA binding|negative regulation of neuron differentiation|positive regulation of

-binding transcription repressor activity, RNA polymerase II-specific|DNA bindin

compartment|protein folding|response to unfolded protein|glycoprotein metabolic

lation|cell differentiation|collagen fibril organization|collagen metabolic proc
nsport, endosome to Golgi

ment bundle assembly|dendritic spine|perikaryon|postsynaptic membrane|regulation

lactose metabolism|Carbon metabolism|Fructose and mannose metabolism|Glycolysis

thway|integral component of membrane|sphingolipid biosynthetic process|metal ion
plasmic reticulum lumen|endoplasmic reticulum membrane|proteolysis|regulation of

d glutamate metabolism|Arginine biosynthesis
romosome, centromeric region|nucleosome|nuclear nucleosome|condensed chromosome

uction|chemical synaptic transmission|zinc ion binding|response to zinc ion|liga

ansport|integral component of membrane|cellular response to oxidative stress|cel

liated phagocytosis|Ras signaling pathway

duction by p53 class mediator resulting in cell cycle arrest|extrinsic apoptotic

cription factor activity|transcription corepressor activity|nucleus|nucleoplasm
I signaling pathway|Vascular smooth muscle contraction|cGMP-PKG signaling pathwa

ak repair|DNA damage response, signal transduction by p53 class mediator resulti

s|cytoplasm|microtubule organizing center|DNA replication initiation|chromatin

diac muscle hypertrophy|cellular protein metabolic process|positive regulation

ath|positive regulation of ERAD pathway
olved in RNA-mediated transposition|metal ion binding|nucleic acid phosphodieste

opment|positive regulation of ubiquitin-protein transferase activity|fat pad dev
cess|integral component of membrane|lipase activity|serine hydrolase activity|de

way|PI3K-Akt signaling pathway

icrotubule-based movement|membrane|kinesin I complex|antigen processing and pres
acellular exosome|NADPH binding

gnaling|negative regulation of organ growth

g tooth|skeletal system morphogenesis|face morphogenesis|bone development
n filament|apical cortex|negative regulation of cilium assembly

ine metabolism|Histidine metabolism|Pyruvate metabolism|Arginine and proline me
ity|membrane|anchored component of membrane

protein ligase binding|identical protein binding|negative regulation of Notch si

-binding transcription repressor activity, RNA polymerase II-specific|RNA polyme

of apoptotic process|positive regulation of apoptotic process|TRAIL binding
e-mediated oocyte maturation|Regulation of lipolysis in adipocytes|Melanogenesi

constituent of cell wall|extracellular region|nucleus|cytoplasm|cytosol|cytoske
ocin signaling pathway|ErbB signaling pathway
sembly

ase binding|growth plate cartilage development|calcium channel activity|calcium

ning complex scaffold activity|cilium assembly
on, DNA-templated|metal ion binding

of cell-substrate adhesion|extracellular matrix organization|collagen-containi

component of membrane|oxidoreductase activity, acting on paired donors, with i
activity, RNA polymerase II-specific|proximal promoter DNA-binding transcriptio
tion|Vasopressin-regulated water reabsorption|Progesterone-mediated oocyte matu
inding|nucleus|nucleoplasm|Notch signaling pathway|viral process|transcriptiona

lasma membrane|intracellular protein transport|Golgi to plasma membrane transpo

ayer development|cerebellar granular layer development|cerebellar Purkinje cell
d|signal transduction|epidermis development|retinal binding|retinol binding|emb

ning complex scaffold activity|cilium assembly

ulting in cell cycle arrest|cell proliferation|CCR4-NOT complex|gene silencing
tion of intracellular estrogen receptor signaling pathway|regulation of Rho pro

rmone signaling pathway|Longevity regulating pathway - multiple species

nization|axonogenesis|integral component of membrane|axon|identical protein bin

ntegral component of membrane|nuclear membrane|meiotic nuclear membrane microtu

nuclear membrane|positive regulation of protein dephosphorylation|neuron project
oliferation|membrane|basolateral plasma membrane|apicolateral plasma membrane|in

ell polarity|ureteric bud invasion
binding|translation initiation factor activity|endoribonuclease activity|nucleus

Serotonergic synapse|Fc epsilon RI signaling pathway|Linoleic acid metabolism|Et
actin signaling pathway
) protein binding|cellular response to heat|regulation of cellular response to h
ion|Dopaminergic synapse|Ras signaling pathway|PI3K-Akt signaling pathway|Circa

ulation of apoptotic process|negative regulation of transcription, DNA-templatec
transcription activator activity, RNA polymerase II-specific|proximal promoter

acellular membrane-bounded organelle

nflammatory response|regulation of ubiquitin-protein transferase activity

actose and mannose metabolism|Glycolysis / Gluconeogenesis

sm|protein phosphorylation|regulation of mitotic nuclear division|positive regul
ulation of proteasomal ubiquitin-dependent protein catabolic process|metal ion b

l metabolism
positive regulation of telomerase RNA localization to Cajal body|negative regula

abolic process

ıne response|negative regulation of transcription, DNA-templated|negative regulа

ɛceptor tyrosine kinase binding|metal ion binding

lmitoyltransferase activity

ɔluripotency of stem cells
leucine and isoleucine degradation|Butanoate metabolism|Fatty acid metabolism
REBP signaling pathway|regulation of Rho protein signal transduction|positive re

ı binding|BLOC-2 complex|early endosome membrane|lysosome localization|protein l
in-coupled receptor signaling pathway|adenylate cyclase-activating G protein-cou

ransforming growth factor beta receptor signaling pathway|androgen receptor sigı

ɔorter activity|sodium-independent organic anion transport

lin deacetylation

ing|protein self-association|positive regulation of transcription by RNA polymeı

ıs|cytoskeleton|cytoskeleton organization|regulation of cell shape|lamellipodiuı
ding|extracellular region|basement membrane|extracellular space|intracellular|eı

ldosterone synthesis and secretion|Insulin secretion|Thyroid hormone synthesis|

evelopment|regulation of canonical Wnt signaling pathway|negative regulation of

fic|chromatin binding|nucleus|sequence-specific DNA binding|positive regulation
er|T cell antigen processing and presentation|DNA binding|chromatin binding|nucl
-specific DNA binding

S-palmitoyltransferase activity|cytoplasmic vesicle membrane|high-density lipo
expression|integral component of membrane|SH3 domain binding|extracellular mat
|Golgi organization|regulation of mitotic nuclear division|cytoskeletal adaptor

equence-specific DNA binding|RNA polymerase II distal enhancer sequence-specific
cell adhesion|skeletal muscle tissue development|cell differentiation|extracellu
aling pathway
transmembrane transporter activity|integral component of membrane|regulation of
in assembly|negative regulation of NF-kappaB transcription factor activity|NAD

body|metal ion binding|regulation of postsynaptic neurotransmitter receptor ac

volved in cilium assembly|intraciliary transport|protein stabilization|cilium as

organization|mitotic spindle organization|nuclear migration|microtubule bindin
cell differentiation

le binding|regulation of fibroblast migration|positive regulation of G2/M transi

port into membrane raft|interleukin-17 production|protein-containing complex|dsR

lasm|5-methylcytosine catabolic process|protein O-linked glycosylation|DNA demet

via plasma membrane adhesion molecules|heterophilic cell-cell adhesion via plas

iation|Rap1 signaling pathway|Fc epsilon RI signaling pathway|GnRH signaling pa
surface|positive regulation of neuron projection development|integral component
ar zinc ion homeostasis|zinc ion binding|regulation of mitochondrion organizatio

ht junction|Vascular smooth muscle contraction|cGMP-PKG signaling pathway

ment|spermatogenesis|piRNA metabolic process|posttranscriptional gene silencing

ity|Golgi transport complex|trans-Golgi network membrane|protein-containing comp

lysis / Gluconeogenesis

cytoplasm

ivity|extracellular region|endoplasmic reticulum lumen|plasma membrane|signal tr

iption, DNA-templated|G protein-coupled receptor signaling pathway|brain develop

esis
ogenesis in camera-type eye|transcription corepressor activity|nucleus|nucleopl

rior/posterior pattern specification|actin cytoskeleton|cell migration|regulati

late cyclase-inhibiting G protein-coupled receptor signaling pathway|G protein-

hway

agy|Ras GTPase binding|clathrin-coated vesicle|negative regulation of cell growt
olved in RNA-mediated transposition|metal ion binding|nucleic acid phosphodieste

cin filament polymerization|regulation of actin cytoskeleton organization|cadhe

l protein binding|cell division

Serotonergic synapse|Apoptosis
vous system development|axon|positive regulation of Notch signaling pathway|den

ein localization to plasma membrane|negative regulation of canonical Wnt signal

cy|Rho protein signal transduction|regulation of cell shape|male gonad developm
le-based movement|microtubule binding|ATPase activity|antigen processing and pr
onent of membrane|L-ascorbic acid metabolic process|secretory granule membrane|

spartate and glutamate metabolism|Arginine biosynthesis|Glutamatergic synapse
ane|integral component of plasma membrane|negative regulation of cell adhesion|

hosphatidylinositol-5-phosphate binding|phosphatidylinositol-3-phosphate binding
lycoprotein catabolic process|multicellular organism development|metallopeptida

osol|plasma membrane|endosome organization|blood coagulation|endosomal transpor
nsferase (adenine-specific) activity|toxin metabolic process|peptidyl-glutamine

d glutamate metabolism|Arginine biosynthesis
er|cytosol|DNA replication|regulation of DNA replication|mRNA processing|ubiqui

omolecule biosynthetic process|positive regulation of cardiac muscle hypertroph

ular space|axon guidance|glial cell migration|extracellular matrix|dendrite reg

a membrane|cellular calcium ion homeostasis|autophagy|hemostasis|cell prolifera

|pericentric heterochromatin assembly|positive regulation of cytokinesis|regula

ic process

tion activity|dynein light intermediate chain binding|cilium assembly|protein t

erm depression|Adrenergic signaling in cardiomyocytes|AMPK signaling pathway|TGF

oplasm|glutamatergic synapse|postsynaptic density, intracellular component|posts

transport|mitochondrion organization|membrane|vesicle-mediated transport|antig

ongevity regulating pathway|Sphingolipid signaling pathway

rate metabolism|Arginine and proline metabolism|Valine, leucine and isoleucine d

olved in RNA-mediated transposition|metal ion binding|nucleic acid phosphodiest

regulation of protein ubiquitination|negative regulation of NF-kappaB transcri
process|inflammatory response|activation of NF-kappaB-inducing kinase activity
epressor activity|nucleus|negative regulation of cell proliferation|protein dim
|Regulation of actin cytoskeleton|Fc gamma R-mediated phagocytosis|Phosphatidyl
lpha-tubulin binding|negative regulation of tubulin deacetylation

ng pathway|cGMP-PKG signaling pathway

ne|recycling endosome membrane|protein localization to membrane|glutamatergic s

activity, RNA polymerase II-specific|proximal promoter DNA-binding transcriptio

signaling pathway

ity|nucleus|nucleoplasm|cytoplasm|nucleosome disassembly|chromatin remodeling|re

tein homooligomerization|protein heterooligomerization
binding|cytoplasmic stress granule|positive regulation of pathway-restricted SMA

sequence-specific DNA binding|DNA-binding transcription factor activity, RNA po
activity, RNA polymerase II-specific|proximal promoter DNA-binding transcription
sequence-specific DNA binding|DNA-binding transcription factor activity, RNA po
|extracellular exosome

activity, RNA polymerase II-specific|DNA-binding transcription activator activit

tic cell cycle|establishment of cell polarity|microtubule organizing center orga

on, DNA-templated|negative regulation of transcription, DNA-templated|metal ion
nsport|retrograde vesicle-mediated transport, Golgi to ER|intra-Golgi vesicle-me

2 sulfur cluster binding|4 iron, 4 sulfur cluster binding|protein maturation|p

phosphorylation|activation of NF-kappaB-inducing kinase activity|COP9 signalos

omodimerization activity|intracellular membrane-bounded organelle|perinuclear re

ion|stress-activated protein kinase signaling cascade|activation of protein kina
is
ER|cytoskeletal adaptor activity|protein transport|Rab GTPase binding|cytoplasmi

ative regulation of autophagosome maturation|negative regulation of autophagoson

g|regulation of protein ubiquitination|negative regulation of NF-kappaB transcri

brane|extrinsic component of cytoplasmic side of plasma membrane|cytoplasmic ves

e|cytokine-mediated signaling pathway|cell junction|insulin secretion|secretory

ein localization to plasma membrane

ecific|chromatin binding|nucleus|nucleoplasm|histone demethylase activity|histon
us|nucleoplasm|cytoplasm|central nervous system development|nuclear matrix|prote

expression|cell junction|positive regulation of transforming growth factor beta
tion activator activity, RNA polymerase II-specific|DNA-binding transcription fa

muscle fiber development

vay|positive regulation of T-helper 17 cell differentiation

ion of apoptotic process

oparatus|cytosol|intracellular protein transport|ER to Golgi vesicle-mediated tı

of blood vessel diameter

cion|chromosome condensation|methylated histone binding|bHLH transcription facto

sferase activity|H4 histone acetyltransferase activity|histone acetylation|NatA
ein polyglutamylation|ciliary basal body|cilium assembly|ciliary basal body-plas
|regulation of ubiquitin-protein transferase activity|branching involved in pros
DNA binding|DNA-binding transcription factor activity|nucleus|nucleoplasm|regula
ncer DNA-binding transcription activator activity, RNA polymerase II-specific|DN
olved in RNA-mediated transposition|metal ion binding|nucleic acid phosphodieste

cituent conferring elasticity|extracellular matrix organization|extracellular ma

in complex|membrane|transport vesicle membrane|protein-containing complex|synaps

RNA polymerase II-specific|DNA binding|transcription coactivator activity|tran
civity|sodium:proton antiporter activity|integral component of membrane|basolate

cex regionalization|cell proliferation in forebrain|forebrain cell migration|neu
ization|cell surface|membrane|myosin complex|cell migration|actomyosin structure

ase activity|zinc ion binding|extracellular matrix disassembly|extracellular mat
ing endosome|regulation of copper ion transmembrane transport

-binding transcription activator activity, RNA polymerase II-specific|DNA-bindir
g transcription repressor activity, RNA polymerase II-specific|transcription cor

cion factor activity|nucleus|nucleoplasm|anatomical structure morphogenesis|sequ
activity|protein heterodimerization activity|protein homooligomerization|protei

nerase II|positive regulation of chondrocyte differentiation|cell fate commitmer

ng|negative regulation of ERK1 and ERK2 cascade|positive regulation of glucocort
|cation transport|integral component of membrane|ion transmembrane transport|phc

DNA damage stimulus|cell adhesion|microtubule cytoskeleton|regulation of cell ac

cructure orientation|sensory processing|ciliary membrane|cilium assembly|non-mot

e|ribonucleoprotein complex

nsport|glutamine transport|cell surface|amino acid transmembrane transporter act

sulfide:quinone oxidoreductase
-binding transcription activator activity, RNA polymerase II-specific|angiogenes

se activity
sm|Arginine biosynthesis|Glutamatergic synapse

essor activity|nucleus|nucleoplasm|cytosol|regulation of transcription by RNA pc
of protein complex assembly|negative regulation of protein autophosphorylation

ıction
ein kinase binding|actin cytoskeleton organization|hemopoiesis|spectrin binding

S-palmitoyltransferase activity|cytoplasmic vesicle membrane|Golgi-associated v
1,2-dipalmitoylphosphatidylcholine)|phospholipase A2 activity consuming 1,2-dic

binding|peptidase activator activity|extracellular exosome|cellular response to
reticulum unfolded protein response|heat shock protein binding|cellular response

s
is

racellular region|extracellular space|cell adhesion|cell surface|negative regula

skeleton|integral component of membrane|viral process|septin complex|septin fila
ific DNA binding|MLL3/4 complex|metal ion binding|dioxygenase activity|histone d

pluripotency of stem cells

COPII vesicle coating|protein complex oligomerization|TRAPPII protein complex

acroautophagy|cell differentiation|protein-containing complex|negative regulatio
ulation of macroautophagy|flavin-linked sulfhydryl oxidase activity|integral con

n|Glycolysis / Gluconeogenesis
odimerization activity
dase activity|metal ion binding
s

jection development|actin cytoskeleton reorganization|endocytic recycling|substr

al protein binding
aling pathway|Inositol phosphate metabolism|Melanogenesis|Dopaminergic synapse|S
tion factor binding|striated muscle tissue development|histone dephosphorylation

ɔkine biosynthetic process|regulation of mRNA stability|translation regulator ac

lipopolysaccharide-mediated signaling pathway|negative regulation of protein bi
ion transmembrane transport|positive regulation of I-kappaB kinase/NF-kappaB s
plasmic stress granule|negative regulation of translation|perinuclear region of

oline transport|membrane|integral component of membrane|choline catabolic proce
A from tricistronic rRNA transcript (SSU-rRNA, 5.8S rRNA, LSU-rRNA)|condensed n

ical endoplasmic reticulum|phosphatidylinositol binding|positive regulation of

phosphate 1-phosphatase activity|cardiac muscle hypertrophy in response to stre

ation|negative regulation of angiogenesis|cell differentiation|negative regulat
repair
g transcription factor activity, RNA polymerase II-specific|proximal promoter
ain
-adenosyl)-pentaphosphatase activity|inositol phosphate metabolic process|meta

ent of Golgi membrane|nuclear membrane|cell redox homeostasis
n development

hormone signaling pathway|Signaling pathways regulating pluripotency of stem c
vesicle membrane|brush border membrane|sodium-independent organic anion transp

-binding transcription activator activity, RNA polymerase II-specific|nucleus|r
ly|ciliary basal body-plasma membrane docking
ity|Wnt signaling pathway|cell junction|positive regulation of Wnt signaling pa
lactose metabolism|Carbon metabolism|Fructose and mannose metabolism|Glycolysis

ellular matrix|secretory granule lumen|neutrophil degranulation|face morphogen
neurotransmitter catabolic process|dopamine metabolic process|catecholamine cat
activity, RNA polymerase II-specific|proximal promoter DNA-binding transcriptio

) pathway|ubiquitin protein ligase binding|SREBP signaling pathway|protein-conta
athway|regulation of growth|identical protein binding|post-translational protei
process

s
er sequence-specific DNA binding|DNA-binding transcription factor activity, RNA

transport|cytoplasmic vesicle membrane|myosin V binding|vesicle|Rab protein sig

racellular exosome
ling pathway|negative regulation of signal transduction|nuclear matrix|cell jun
Like receptor 4 signaling pathway|innate immune response|lipopolysaccharide rec

etwork transport vesicle membrane|postsynaptic density|telencephalon developmen

:
RNA splicing|protein autophosphorylation

enesis|positive regulation of neural precursor cell proliferation
orter activity|symporter activity|calcium ion transmembrane transport
nage checkpoint|identical protein binding|FHA domain binding

ncer DNA-binding transcription activator activity, RNA polymerase II-specific|h

ic acid transport|integral component of membrane|folic acid metabolic process|f

gion|extracellular space|endoplasmic reticulum lumen|extracellular matrix organ

ng pathway|Jak-STAT signaling pathway|AMPK signaling pathway|FoxO signaling pat

ng pathway|positive regulation of transcription by RNA polymerase II|metal ion

gration|negative regulation of BMP signaling pathway|endothelial cell activatior

ntaxin binding|SNARE complex|neuromuscular junction|glucose homeostasis|negativ

Pase activity|cytoskeleton-dependent intracellular transport|mitochondrial membr

transporter activity|3'-phosphoadenosine 5'-phosphosulfate biosynthetic process

hway|MAPK signaling pathway - fly

ng pathway|Ras signaling pathway|PI3K-Akt signaling pathway

lular region|plasma membrane|embryo implantation|metalloendopeptidase inhibitor

|protein dephosphorylation|cell cycle arrest|protein phosphatase regulator activ
vity|extracellular exosome
olved in RNA-mediated transposition|metal ion binding|nucleic acid phosphodieste
smic ribonucleoprotein granule|identical protein binding|ribonucleoprotein compl

|proteasome-mediated ubiquitin-dependent protein catabolic process|positive regu

r ear development|chondroitin sulfate proteoglycan biosynthetic process|positive

on, DNA-templated|metal ion binding
g|regulation of protein ubiquitination|negative regulation of NF-kappaB transcri
tion activator activity, RNA polymerase II-specific|epithelial to mesenchymal tr

ear development
rotein homodimerization activity|dendritic spine|dendritic shaft|intracellular r

sphatase 2A binding

activity, RNA polymerase II-specific|nucleus|zinc ion binding|transcription regu
spliceosome|protein complex oligomerization
ing|cell-cell adhesion|negative regulation of neural precursor cell proliferatio
anslation repressor activity|dendrite|regulation of mRNA stability|synapse|posit

activity, RNA polymerase II-specific|DNA-binding transcription factor activity|r

activator activity, RNA polymerase II-specific|branching involved in blood vess

sol|defense response|microtubule binding|response to virus|protein transport|mem

receptor signaling pathway|adenylate cyclase-activating G protein-coupled recept
ic process|protein homodimerization activity|positive regulation of angiogenesis

inding transcription factor activity, RNA polymerase II-specific|proximal promot
gulation of transcription, DNA-templated|positive regulation of DNA-binding tran

ific DNA binding|MLL3/4 complex|metal ion binding|dioxygenase activity|histone c
regulation of mitotic spindle organization
Tyr) deacylase activity

ific DNA binding|blastocyst hatching|transcription corepressor activity|nucleus

some|cytosol|polysome|intermediate filament|plasma membrane|protein C-terminus b
activity, RNA polymerase II-specific|proximal promoter DNA-binding transcription

ding|cytosol|plasma membrane|G protein-coupled receptor signaling pathway|multic

ing|secretory granule membrane|specific granule membrane|signaling receptor acti
sicle|protein localization to phagophore assembly site|late nucleophagy

regulation of intracellular pH|sperm principal piece
ion of actin cytoskeleton|ErbB signaling pathway|Fc gamma R-mediated phagocytos
ll adhesion|signal transduction|integrin-mediated signaling pathway|cell migrati
mediated signaling|protein kinase binding|protein domain specific binding|cell

ation|Vasopressin-regulated water reabsorption|Regulation of lipolysis in adipo

e|peptidyl-serine phosphorylation|intracellular signal transduction|regulation c

|nucleus|cytoplasm|cell proliferation|peptidyl-serine phosphorylation|peptidyl-t

sion

cription factor activity|nucleus|chromatin organization|regulation of transcript

ation|negative regulation of apoptotic process|protein heterodimerization activi
nslational fidelity|metal ion binding
sicular body sorting pathway|multivesicular body assembly|viral budding via host

le-strand break repair via homologous recombination|nuclear matrix|ATPase activi

avage|histone mRNA metabolic process|nuclear body|histone pre-mRNA 3'end process

peptide binding|peptide catabolic process|metalloaminopeptidase activity|extrace

ding|actin cytoskeleton organization|GTPase activating protein binding|intracel

pendent organic anion transport

ion of cell migration|ovulation|neutrophil degranulation|negative regulation of

|cholesterol homeostasis|membrane raft

transcription repressor activity, RNA polymerase II-specific|RNA polymerase II

nsport into cytosol|membrane|integral component of membrane|enzyme binding|ubiqu

cell migration involved in sprouting angiogenesis|positive regulation of vascu

le|perikaryon
53 binding|positive regulation of neuroblast proliferation|chromatin binding|his

signaling pathway|Antigen processing and presentation|Calcium signaling pathway|
regeneration|nuclear membrane|positive regulation of myoblast differentiation|r
or activity, RNA polymerase II-specific|skeletal system development|DNA-binding

dhesion molecules|platelet alpha granule membrane

pathway|Sphingolipid signaling pathway|MAPK signaling pathway - fly

|GTPase inhibitor activity|nucleus|nucleoplasm|mitochondrion|cytosol|JNK cascade

olved in RNA-mediated transposition|metal ion binding|nucleic acid phosphodieste
junction|focal adhesion|axon guidance|fibroblast growth factor receptor signalin
ysis|NF-kappa B signaling pathway

oly|low-density lipoprotein particle receptor binding|3M complex

onal protein modification|cellular protein metabolic process|extracellular exos

rotein deamination|response to lipopolysaccharide|aorta development|oxidation-re

centa development|negative regulation of endothelial cell proliferation|retinoic
osome|microtubule organizing center|microtubule|protein phosphorylation|cell cyc

ssembly|metal ion binding|cell-cell adhesion

romosome condensation|nucleosomal DNA binding|negative regulation of chromatin s

catabolic process|proteasome regulatory particle, lid subcomplex|protein deubiqu
n|integral component of membrane|negative regulation of cell cycle|ubiquitin pro

olved in RNA-mediated transposition|metal ion binding|nucleic acid phosphodieste

dy|NELF complex|negative regulation of transcription elongation from RNA polymer

Pase activity|activation of GTPase activity
body|dendrite|protein N-terminus binding|cell division|establishment of locali
cycle)|Valine, leucine and isoleucine degradation|Propanoate metabolism|Glycoly
et dense granule lumen|extracellular exosome

ivity|nucleus|nucleoplasm|cytoplasm|heart development|snRNA binding|innate immu

llagen binding|extracellular space|cytoplasm|cell adhesion|nervous system devel

ypes of N-glycan biosynthesis|Glycosphingolipid biosynthesis - lacto and neolac

contact zone

mitotic cell cycle|protein kinase A catalytic subunit binding|negative regulat

g|negative regulation of ATPase activity|protein-containing complex|phosphatidy

ein catabolic process|nuclear speck|SCF ubiquitin ligase complex|SCF-dependent

-5-phosphate binding|positive regulation of gene expression|superoxide-generati

ic process|extracellular matrix structural constituent conferring compression
eraction|MAPK signaling pathway|TGF-beta signaling pathway|FoxO signaling pathwa
Ras signaling pathway|PI3K-Akt signaling pathway

is regression

s|extracellular exosome|cellular response to calcium ion

id binding|endosome membrane|negative regulation of autophagy|membrane|ceramide

ceptor signaling pathway|inhibin binding|activin receptor antagonist activity

ating G protein-coupled receptor signaling pathway|digestion|feeding behavior|po
|microtubule|microtubule-based process|centrosome cycle
ort|antigen processing and presentation|Rab protein signal transduction
lase activity|lipid homeostasis

DNA-binding transcription activator activity, RNA polymerase II-specific|nucleus

ulation|integral component of membrane|cytoplasmic vesicle membrane|regulation o

gative regulation of cell proliferation|fibroblast growth factor receptor signal
on, DNA-templated|metal ion binding
nuclear membrane|filopodium tip|sterol binding|intracellular membrane-bounded or

rane transport

ellular zinc ion homeostasis|zinc ion binding|response to zinc ion|regulation of
nucleotide-sugar transmembrane transporter activity|integral component of membra
reorganization|protein homodimerization activity|synapse
oactivator activity|positive regulation of transcription, DNA-templated|DUBm con
-lysine N-methyltransferase activity|peptidyl-lysine trimethylation|histone-lys

aling|P granule|regulation of mRNA stability

in folding|smooth muscle contraction|response to glucose|negative regulation of

lymerase II|metal ion binding

|nucleus|nucleoplasm|cytoplasm|actin cytoskeleton|microtubule cytoskeleton|inte
n ion transport|voltage-gated potassium channel complex|integral component of me

subunit binding|mitochondrial translational elongation|mitochondrial translatio

|negative regulation of gene expression|positive regulation of cell-substrate ac

r exosome

membrane|protein-containing complex|response to cytokine|positive regulation of

C class I, TAP-dependent|antigen processing and presentation of exogenous peptic
brane contact site
activity, RNA polymerase II-specific|DNA-binding transcription repressor activit
binding|dendrite cytoplasm|protein-containing complex scaffold activity|neurona
lation|zinc ion binding|NuRD complex|nuclear speck|protein binding, bridging|sec
cell adhesion|axon guidance|heparin binding|fibroblast growth factor receptor si

embrane|integral component of plasma membrane|chloride transport|endosome membra

pathway|Focal adhesion
ılation of bone mineralization|platelet alpha granule lumen|biomineral tissue de

ion of cell growth|positive regulation of protein binding|positive regulation of

ı of synaptic plasticity|presynaptic active zone|cytoskeleton of presynaptic act
ıe|cytosol|plasma membrane|integral component of plasma membrane|cell-cell junct
ılum membrane|cytosol|apoptotic process|defense response|signal transduction|mic

f primary germ layer|neural tube closure|cardiac chamber development|optic cup f

olved in RNA-mediated transposition|metal ion binding|nucleic acid phosphodieste

ane|nuclear speck|ATPase activity|mRNA 3'-end processing|identical protein bind

fiber|ciliary basal body-plasma membrane docking

orane cell adhesion molecules|signal transduction|regulation of myelination|pos

ediated transport, Golgi to ER|adult locomotory behavior|membrane|cerebellar Pur

a receptor signaling pathway|G protein-coupled receptor signaling pathway|small
gative regulation of canonical Wnt signaling pathway
|cholesterol homeostasis|membrane raft

component of membrane|vesicle-mediated transport|calcium ion regulated exocytos

ion binding|protein deubiquitination|positive regulation of proteasomal ubiquit

ressor activity, RNA polymerase II-specific|chromatin binding|DNA-binding transc

ıctivity, RNA polymerase II-specific|DNA-binding transcription repressor activi

ion binding

vascular smooth muscle cell proliferation

ıway|Ras signaling pathway|PI3K-Akt signaling pathway|Focal adhesion
ay|Signaling pathways regulating pluripotency of stem cells|Melanogenesis|Dopam
|cellular amino acid metabolic process|nitric oxide biosynthetic process|amino
|response to organic cyclic compound|keratan sulfate biosynthetic process|extra

ıctivity

ynapse|Ras signaling pathway|PI3K-Akt signaling pathway|Taste transduction|Circ
pathway, calcium modulating pathway|sensory perception of sound|cell surface|
e regulation of actin nucleation|actin cortical patch localization
ıslational fidelity|metal ion binding
ıctivity, RNA polymerase II-specific|proximal promoter DNA-binding transcriptior

ıetabolic process|hormone biosynthetic process|small molecule metabolic process
leuroactive ligand-receptor interaction|Phospholipase D signaling pathway|PI3K-A

binding|ubiquitin protein ligase binding|positive regulation of proteasomal ub
|DNA-binding transcription repressor activity, RNA polymerase II-specific|DNA-

eonine kinase activity|ATP binding|nucleus|cytoplasm|regulation of transcriptior

ein localization to plasma membrane|regulation of potassium ion transmembrane t

s|response to glucose|integral component of membrane|dsRNA transport|AP-2 adap

ediated transport, Golgi to ER|post-Golgi vesicle-mediated transport|vesicle do

ytoplasm|plasma membrane|G protein-coupled receptor signaling pathway|adenylate

eine-type peptidase activity|endosomal transport|polysaccharide binding|laminin

ated ER to Golgi transport vesicle|integral component of Golgi membrane|integra

ation|Vascular smooth muscle contraction|cGMP-PKG signaling pathway

e chemotaxis|interleukin-17-mediated signaling pathway|positive regulation of cy

eptor signaling pathway|NF-kappa B signaling pathway
e
oximal promoter DNA-binding transcription repressor activity, RNA polymerase II-

cellular exosome

cy|embryo implantation|response to xenobiotic stimulus|response to ozone|phospho
ulation of cell adhesion|apical plasma membrane|cell migration|negative regulati

lic cell adhesion via plasma membrane adhesion molecules|membrane|integral compo
e transmembrane transporter activity|anion:anion antiporter activity|integral co

cycle DNA replication|positive regulation of G2/M transition of mitotic cell cyc

ral component of membrane|identical protein binding|lysosomal lumen|leukocyte mi

ransmembrane transport|regulation of ion transmembrane transport|sodium ion tran
abolism|One carbon pool by folate|Biosynthesis of amino acids

endosome membrane|intracellular membrane-bounded organelle

otein binding|membrane raft|positive regulation of transcription, DNA-templated

e|specific granule membrane|neutrophil degranulation|phospholipid translocation
n|nucleoside transport|uridine transport|membrane|basolateral plasma membrane|ap

ace receptor signaling pathway|cytoplasmic side of plasma membrane|GDP binding|r

electrical coupling|postsynaptic density|actin cytoskeleton|dendrite|growth cone

ose metabolism|Glycolysis / Gluconeogenesis

raction|Toll-like receptor signaling pathway|MAPK signaling pathway|NOD-like rec
of protein kinase B signaling|cellular response to gamma radiation

pathway|microtubule cytoskeleton|membrane|cytoplasmic vesicle|multicellular org
ing|negative regulation of cell proliferation|postsynaptic density|maintenance o
on|negative regulation of bone mineralization involved in bone maturation
ular response to interleukin-1|cellular response to tumor necrosis factor

on binding|histone demethylase activity|histone demethylase activity (H3-K36 spe
erase II-specific|histone deacetylase activity|nucleus|nucleolus|mitochondrion|p

gulation of intracellular pH|recycling endosome|potassium ion transmembrane tran

slational elongation|mitochondrial translational termination

in deubiquitination
-binding transcription repressor activity, RNA polymerase II-specific|DNA bindin
ession|PML body|protein sumoylation|negative regulation of transforming growth f

age-gated potassium channel complex|integral component of membrane|protein homoc
r necrosis factor production|positive regulation of interleukin-6 production|ret
x|SCF-dependent proteasomal ubiquitin-dependent protein catabolic process|entrai

n binding|extracellular region|extracellular space|Golgi apparatus|proteolysis|r
zation|biomineral tissue development|positive regulation of osteoblast prolifera

ry chain complex I assembly|mitochondrial respiratory chain complex IV assembly

egulation of cell migration|positive regulation of microtubule polymerization|ne

l-cell signaling|cytoplasmic side of plasma membrane|postsynaptic density|cell
one acetyltransferase activity|nucleus|nucleoplasm|cytosol|DNA replication|regu
l transduction|membrane|nuclear speck|regulation of cell adhesion mediated by in

oter|spermatogenesis|transcription elongation factor complex|regulation of epith

lic process|extracellular exosome

dioxygenase activity|carotenoid metabolic process|carotene metabolic process|ca
otein signal transduction|retrograde transport, endosome to Golgi|identical prot
on|cytoplasmic vesicle|paranodal junction|Schmidt-Lanterman incisure|cell-cell

ase activity

icrofibril binding|lobar bronchus epithelium development
signal transduction

ve regulation of JNK cascade|protein heterodimerization activity|perinuclear reg

erentiation|flagellated sperm motility|motile cilium|regulation of ion transmemb

g growth factor beta
brane|positive regulation of collagen biosynthetic process|protein folding in en

r matrix

tracellular matrix|collagen V binding

ent|nuclear hormone receptor binding
A polymerase II

ay|PI3K-Akt signaling pathway

tor activity, RNA polymerase II-specific|proximal promoter DNA-binding transcrip
ycan biosynthetic process|glycosaminoglycan catabolic process|axon guidance|Schw

rocess|protein monoubiquitination|regulation of autophagy|regulation of phosphat

signaling pathway|limb morphogenesis|cell migration involved in gastrulation|neg
system development|mitral valve development|pulmonary valve formation|ventricula

lization|positive regulation of transcription from RNA polymerase II promoter in

stress|embryo implantation|negative regulation of gene expression|enzyme bindi[illegible]

[illegible]f membrane|positive regulation of angiogenesis|ER membrane protein complex|pos[illegible]

[illegible]n, DNA-templated|zinc ion binding
[illegible]vary gland morphogenesis|positive regulation of pathway-restricted SMAD protei[illegible]

[illegible]cyl carnitine transport|amino acid transport|basic amino acid transmembrane tr[illegible]
protein ubiquitination|ubiquitin binding|metal ion binding|K63-linked polyubiq[illegible]
[illegible]l adhesion

[illegible]sduction|positive regulation of apoptotic process|positive regulation of fibro[illegible]

-binding transcription activator activity, RNA polymerase II-specific|skeletal [illegible]
assembly

metabolism

[illegible]ein phosphorylation|signal transduction|intrinsic apoptotic signaling pathway [illegible]

-binding transcription activator activity, RNA polymerase II-specific|cell fate

[illegible] pathway|Hippo signaling pathway -multiple species|Axon guidance|Regulation of
ion binding|protein dimerization activity

[illegible]ingle-stranded DNA binding|nucleus|cytoplasm|regulation of transcription, DNA-[illegible]

[illegible]g

[illegible]ctivity, RNA polymerase II-specific|proximal promoter DNA-binding transcriptio[illegible]

in binding

at centromere|chromatin silencing by small RNA|nuclear RNA-directed RNA polymer
actor activity, RNA polymerase II-specific|proximal promoter DNA-binding transcr

Rho protein signal transduction|regulation of Rho protein signal transduction|po
rotein homodimerization activity|intracellular membrane-bounded organelle|negati

response to amino acid starvation|ribonucleoprotein granule|transcription regula

ll adhesion|protein localization|endomembrane system|apical plasma membrane|nucl

gi apparatus|cytosol|immune response|response to bacterium|positive regulation o

ath|positive regulation of ERAD pathway

sm|transcription factor complex|transcription factor binding|transcription regul

n binding|dorsal/ventral pattern formation|negative regulation of signal transdu
on|cellular response to zinc ion

olism|Glycolysis / Gluconeogenesis|Phenylalanine metabolism

a membrane|adherens junction|cell-matrix adhesion|integrin-mediated signaling pa

plex

|vesicle|microtubule severing|nuclear export|spindle midzone assembly|negative r

regulation of cyclin-dependent protein serine/threonine kinase activity
signaling in cardiomyocytes|Phospholipase D signaling pathway
l|protein phosphorylation|JNK cascade|chemical synaptic transmission|multicellu

gnaling pathway|Adrenergic signaling in cardiomyocytes|Aldosterone synthesis ar
negative regulation of apoptotic process|protein autophosphorylation|negative re
ılus|cell proliferation|response to ionizing radiation|membrane|cell migration|p
component of membrane|basolateral plasma membrane|apical plasma membrane|cytop

n guidance|Focal adhesion|Regulation of actin cytoskeleton|TGF-beta signaling p
nucleus|nucleocytoplasmic transport|Ran GTPase binding|protein transporter acti
se kinase kinase binding|positive regulation of lipopolysaccharide-mediated sig

lex disassembly|MICOS complex|mitochondrial nucleoid organization|positive regu
activity
nterleukin-6 production|positive regulation of tumor necrosis factor production

oregulator activity|nucleus|transcription factor complex|nucleolus|plasma membra

iosynthetic process|oxysterol binding|lipid binding|sterol transporter activity
ional termination
ne metabolism|Carbon metabolism|Arginine and proline metabolism|2-Oxocarboxylic

activity, RNA polymerase II-specific|DNA-binding transcription repressor activi

ytoplasm|spindle|spindle microtubule|protein phosphorylation|mitotic spindle or
g

on complex|DNA duplex unwinding

air|DNA dealkylation involved in DNA repair|regulation of translational initiat
neutrophil degranulation

ation of type I interferon production|activating transcription factor binding|A

|myo-inositol transport|integral component of membrane|regulation of respiratory

ivity|basolateral plasma membrane|myosin complex|protein domain specific binding

n, DNA-templated|RNA polymerase II transcription factor complex|sequence-specifi

wth rate|negative regulation of apoptotic process|DBD domain binding|negative re
ion binding|mRNA methylation|regulation of stem cell population maintenance
assembly|synaptic membrane adhesion|regulation of presynapse assembly

factor-activated receptor activity|extracellular region|plasma membrane|cell ac

system development|mitral valve development|pulmonary valve formation|ventricula

ion|histone H4-K20 trimethylation|histone H3-K9 trimethylation|histone H4 acety

factor activity, RNA polymerase II-specific|transcription factor activity, RNA

enome replication|regulation of DNA-templated transcription, elongation

gamma R-mediated phagocytosis|Fat digestion and absorption
e

nt
endent protein catabolic process|protein deubiquitination|negative regulation of

ning complex|magnesium:sodium antiporter activity|cellular response to magnesium

culum|endoplasmic reticulum-Golgi intermediate compartment|Golgi-associated ves

erentiation|metal ion binding|proteolysis involved in cellular protein catabolic

involved in apoptotic process|smoothened signaling pathway|intraciliary transpor

response to fluid shear stress
sma membrane adhesion molecules|cell-cell signaling|nervous system development|

on|Longevity regulating pathway|Sphingolipid signaling pathway|T cell receptor s
st cell|positive regulation of viral genome replication|negative regulation of v
binding|nucleus|cytoplasm|regulation of transcription by RNA polymerase II|tran
s|taurine transport|aspartate transmembrane transport|membrane|ion channel compl
nent of plasma membrane|humoral immune response|cell-cell signaling|multicellula

junction assembly|cell adhesion|signal transduction|positive regulation of gene

osine kinase activity|GTPase inhibitor activity|epidermal growth factor receptor

of membrane|positive regulation of angiogenesis|ER membrane protein complex|posi

necting cilium|ciliary basal body|DNA damage response, detection of DNA damage|r

component of membrane|alpha-2A adrenergic receptor binding|alpha-2B adrenergic r
activity, RNA polymerase II-specific|chromatin binding|nucleus|cell differentiat

ssion, dopaminergic|actin binding|copper ion binding|calcium ion binding|phospho
olved in RNA-mediated transposition|metal ion binding|nucleic acid phosphodieste

iole|spindle pole body|spindle|polar microtubule|cytosol|microtubule nucleation
of cell migration|cell leading edge|protein localization to Golgi apparatus|nega

nt network formation|bone development

on|Circadian entrainment|cAMP signaling pathway|MAPK signaling pathway|Choliner

ch factor-activated receptor activity|protein kinase activator activity|cytoplas

naling|integral component of mitochondrial inner membrane|positive regulation of

membrane|cadmium ion transmembrane transport|zinc ion import across plasma membr

replication|midbody|metal ion binding|protein stabilization|extracellular exosom
nsport, Golgi to ER|microtubule-based movement|mitotic spindle organization|mito

ion|phosphotransferase activity, alcohol group as acceptor|manganese ion binding

|perinuclear region of cytoplasm
ation|positive regulation of lipid biosynthetic process
llular matrix organization

promoter|termination of RNA polymerase I transcription|rRNA transcription|protei

c vesicle

mation|sequence-specific DNA binding

dependent protein catabolic process|integral component of membrane|late endosome
egulation of bone mineralization

euromuscular junction|ubiquitin protein ligase activity
|protein kinase binding|positive regulation of kinase activity|cellular response

|RNA binding|extracellular region|extracellular space|nucleoplasm|cytoplasm|sign

ding|ephrin receptor signaling pathway|regulation of axonogenesis|regulation of

ll development|Wnt signaling pathway|COPII vesicle coat|COPII-coated ER to Golgi

ific DNA binding|MLL3/4 complex|metal ion binding|dioxygenase activity|histone

ane|integral component of plasma membrane|immune response|humoral immune respons

proliferator activated receptor signaling pathway|PR-DUB complex|peroxisome pro

membrane transporter activity|cell junction|axon|plasma membrane lactate transpo

cidase activity|nuclear speck|extracellular matrix disassembly|metal ion binding

f postsynaptic density membrane|positive regulation of amyloid-beta formation
zation or depolymerization|cell division

clear membrane|cholesterol homeostasis

rase inhibitor activity|telomere capping|DEAD/H-box RNA helicase binding|telome

regulation of phosphoprotein phosphatase activity|intracellular membrane-bound

ocess

l metabolism

component of plasma membrane|transmembrane receptor protein tyrosine kinase sig
ase activity|glycogen binding

-containing extracellular matrix

wth factor receptor signaling pathway|regulation of protein secretion|negative r
e regulation of transcription, DNA-templated

cord development|dendrite|neuronal cell body|receptor complex

factor beta receptor signaling pathway|heparin binding|protein secretion|growth

|determination of left/right symmetry|heart development|nuclear speck

mbrane|glycosylation
one H4-K5 acetylation|histone H4-K8 acetylation|histone H4-K16 acetylation|metal

-substrate adhesion|collagen-containing extracellular matrix|extracellular exoso
single-stranded DNA binding|nucleus|positive regulation of transcription by RNA

lation of MAPK cascade|metal ion binding|ubiquitin protein ligase activity
DNA-templated|metal ion binding|dynein complex binding|positive regulation of

ear membrane|nuclear periphery
inding|cytoplasmic side of plasma membrane|sodium channel regulator activity|neg

gulation of translation

pidermal growth factor receptor signaling pathway|regulation of cell shape|respo

a signaling pathway|FoxO signaling pathway|Endocytosis

d receptor signaling pathway|adenylate cyclase-activating G protein-coupled rece

on binding

e|cytosol|ubiquitin-dependent protein catabolic process|cell proliferation|SCF c
cle activity|cellular homeostasis|kinase binding|collagen metabolic process|mult

nscription repressor activity, RNA polymerase II-specific|fibrillar center|nucle

ation|response to ischemia|protein kinase activity|protein serine/threonine kina

na membrane|drug transmembrane transport|Golgi organization|sensory perception o
response|signal transduction|external side of plasma membrane|integral componen

lic process|amino acid transport|amino acid transmembrane transporter activity|t
ular body|microtubule|inflammatory response|signal transduction|female pregnancy

rane|intracellular membrane-bounded organelle|UDP-galactose transmembrane transp

y
tegral component of Golgi membrane|UDP-N-acetylglucosamine transmembrane transpo

of actin cytoskeleton|Neuroactive ligand-receptor interaction|Taste transductio

ediated transport, Golgi to ER|endocytosis|integral component of membrane|early
c|neurogenesis
rocess|protein insertion into ER membrane

gnaling pathway|Osteoclast differentiation|cAMP signaling pathway|NOD-like rece
nent|integral component of membrane|myelination|myelin sheath|oligodendrocyte di

cell proliferation|RNA splicing|histone acetyltransferase binding|positive regul
um signaling pathway|Ras signaling pathway|PI3K-Akt signaling pathway|Regulation
ulation of autophagy|PML body|cytoplasmic vesicle|ubiquitin binding|positive reg

tyrosine kinase signaling pathway|cell proliferation|glial cell migration|cell

euromuscular junction|ubiquitin protein ligase activity
|neuromuscular junction

in-dependent ERAD pathway|Hsp70 protein binding|response to endoplasmic reticulu

cing by miRNA

anslational protein modification|cellular protein metabolic process|extracellula
ndrite morphogenesis|positive regulation of dendrite development
r activity|quaternary ammonium group transmembrane transporter activity|organic
rade transport, endosome to Golgi|perinuclear region of cytoplasm|recycling endo

iculum lumen|phagocytosis, recognition|phagocytosis, engulfment|cell adhesion|si

s

podium|negative regulation of actin filament depolymerization|positive regulatio

les|cellular response to starvation|intercalated disc|integral component of memb
feldin A|extracellular exosome|extrinsic component of Golgi membrane
ion of cell-substrate adhesion|extracellular matrix organization|glomerular base
tion activator activity, RNA polymerase II-specific|morphogenesis of an epitheli
biogenesis

signaling pathway|Longevity regulating pathway|Toll-like receptor signaling path

s junction|cell-cell adherens junction|cell-cell junction assembly|heart develop

ific DNA binding|MLL3/4 complex|metal ion binding|dioxygenase activity|histone d

progenitor cell division|positive regulation of Wnt signaling pathway|regulatio
|multicellular organism development|animal organ morphogenesis|negative regulati
onent of membrane|GTP-Rho binding|regulation of SNARE complex assembly|negative
e assembly|endopeptidase activator activity|proteasome binding
egulation of Arp2/3 complex-mediated actin nucleation|cellular protein localizat

sin complex|microtubule|microtubule-based movement|microtubule binding|protein t

pathway|protein deubiquitination|regulation of protein stability|thiol-dependen

olved in RNA-mediated transposition|metal ion binding|nucleic acid phosphodieste

|retina development in camera-type eye
ılation of dopamine secretion|synaptic vesicle exocytosis|vesicle-mediated trans

:or activity, RNA polymerase II-specific|proximal promoter DNA-binding transcri[illegible]

depression|Platelet activation|Circadian entrainment|cGMP-PKG signaling pathway

:tin polymerization or depolymerization|adenylate cyclase binding|postsynaptic [illegible]
ansport|intraciliary transport involved in cilium assembly|ciliary basal body|i[illegible]

|siderophore transport|transmembrane transporter activity|integral component of

ion|hyaluronan biosynthetic process|cellular response to platelet-derived growt[illegible]
of protein secretion|phosphatidylinositol-4-phosphate binding

: gamma R-mediated phagocytosis|Fat digestion and absorption

.ion|Longevity regulating pathway - multiple species|Endocytosis
ɔ-bounded organelle
ɔgral component of membrane|negative regulation of natural killer cell activati[illegible]

maturation

nent|actin filament bundle organization|negative regulation of bone mineralizat[illegible]

l adhesion molecules|signal transduction|positive regulation of neuron projecti[illegible]

ynaptic transmission|synaptic vesicle|vesicle membrane|glutamate secretion|memb[illegible]

:yclase activity|regulation of cell shape|postsynaptic density|basolateral plas[illegible]

:ion activator activity, RNA polymerase II-specific|morphogenesis of an epitheli

ivity
ive regulation of neuron migration
olved in RNA-mediated transposition|metal ion binding|nucleic acid phosphodieste

linositol transporter activity|endomembrane system|membrane|COPII-coated ER to (

rotubule nucleation|Golgi organization|centrosome cycle|microtubule binding|asyn
:ic process, using glutaminyl-peptide cyclotransferase|specific granule lumen|ne

activity, RNA polymerase II-specific|proximal promoter DNA-binding transcription

egulation of cell migration|zymogen activation|negative regulation of interferon

ion factor activity|transcription coactivator activity|transcription corepressor

morphogenesis|collagen-containing extracellular matrix|positive regulation of c
morphogenesis|collagen-containing extracellular matrix|positive regulation of c

ositive regulation of gene expression|positive regulation of epithelial cell mig

ion|PI3K-Akt signaling pathway
activity, RNA polymerase II-specific|proximal promoter DNA-binding transcription

ytoskeleton|Tight junction|Ras signaling pathway

forelimb morphogenesis|metal ion binding

eptor signaling pathway

regulation of MAP kinase activity|negative regulation of Ras protein signal tra

ivator activity|cytoplasmic stress granule|membrane|positive regulation of macro
phosphate ion transmembrane transporter activity|integral component of membrane

transport|nuclear body|protein kinase binding|endocytic recycling|ciliary basal

ransduction|regulation of GTPase activity|regulation of small GTPase mediated si

nt of membrane|drinking behavior|metalloaminopeptidase activity
transcription|transcription initiation from mitochondrial promoter|mitochondrion
cellular response to DNA damage stimulus|RNA splicing|regulation of myotube diff

g|postsynaptic density|protein transport|basolateral plasma membrane|PDZ domain

otein phosphatase 1 binding|cytoplasmic stress granule|membrane|dendrite|cellula

transcription factor binding|DNA binding|transcription corepressor activity|hist

n of transcription by RNA polymerase II|viral process|nuclear body|PML body
yosin II head/neck binding|metal ion binding|positive regulation of stress fiber

ransmembrane transport|integral component of membrane|extracellular exosome
iosynthetic process|negative regulation of stress fiber assembly

ransport|retrograde vesicle-mediated transport, Golgi to ER|intra-Golgi vesicle-
tein binding|protein homooligomerization|endoplasmic reticulum tubular network m

ensory perception of sound|positive regulation of peptidyl-threonine phosphoryla

ion|microtubule cytoskeleton organization|RNA binding|nucleus|nucleoplasm|centro
inergic synapse|Osteoclast differentiation|Glutamatergic synapse|MAPK signaling

osynthesis - globo series|Glycosaminoglycan degradation|Lysosome
|ubiquitin-dependent protein catabolic process|protein targeting to mitochondrio

A binding|DNA-binding transcription factor activity, RNA polymerase II-specific

nal sodium ion absorption|protein K48-linked ubiquitination|distal tubule morpho

rly endosome membrane|perinuclear region of cytoplasm
nsmission|synapse assembly|integral component of membrane|calcium-dependent cel

ent of membrane|vesicle|transmembrane transport

|nucleolus|spermatogenesis|fertilization|post-embryonic development|multicellula
ve regulation of mitotic cell cycle|regulation of metaphase plate congression

s

nent of plasma membrane|potassium ion transport|voltage-gated potassium channel
inding|tubulin binding|SH3 domain binding|AP-2 adaptor complex|clathrin-coated v
ng transcription activator activity, RNA polymerase II-specific|skeletal system

erm depression|Adrenergic signaling in cardiomyocytes|AMPK signaling pathway|TGF

nain binding|intracellular membrane-bounded organelle|positive regulation of cys

|positive regulation of mRNA catabolic process

sulin signaling pathway|FoxO signaling pathway|PI3K-Akt signaling pathway|Circad

Pase binding|early endosome membrane|recycling endosome

cell differentiation

er assembly|collagen-containing extracellular matrix|cellular response to UV-B|e

stance|positive regulation of protein modification process|protein-DNA complex|s
fferentiation

pper ion binding|calcium ion binding|extracellular region|trans-Golgi network|p

atelet activation|Vascular smooth muscle contraction|cGMP-PKG signaling pathway

avage furrow|positive regulation of cytokinesis|microtubule minus-end|costamere

r activity|nucleus|regulation of transcription, DNA-templated|multicellular orga

ase activity|activation of GTPase activity

-binding transcription activator activity, RNA polymerase II-specific|in utero e

ppa B signaling pathway

nsferase activity|protein-arginine N-methyltransferase activity|peptidyl-argini

:iated glycoprotein complex|viral process|apicolateral plasma membrane|Z disc|ke

embrane|integral component of plasma membrane|cell-cell junction|adherens juncti
nervous system development|axon guidance|external side of plasma membrane|glial
ransporter activity|symporter activity

f sequestering of triglyceride|sterol transporter activity|cholesterol binding|p
ed transport, Golgi to ER|microtubule-based movement|microtubule binding|ATP-dep

optotic process|inflammatory response|signal transduction|cell surface receptor
uclear region of cytoplasm|protein homooligomerization|potassium ion transmembra
ng|protein localization to plasma membrane

on|cGMP-PKG signaling pathway
iculum membrane|specific granule membrane|neuron projection|neuronal cell body|n

ogenesis|Wnt signaling pathway|nuclear body|negative regulation of transcription

ar speck|enzyme binding|metal ion binding|RNA uridylyltransferase activity|pre-m
somitogenesis|protein homodimerization activity|positive regulation of transcri

dritic spine head|regulation of postsynaptic membrane potential|presynapse|posts

rogenase (NADP+) activity
apical plasma membrane|syntaxin-1 binding|syntaxin-3 binding|specific granule|az
ngolipid biosynthesis - ganglio series

s

ive regulation of MAP kinase activity|metal ion binding|regulation of ERK1 and E

activity|cytoplasm|plasma membrane|cation transport|chloride transport|phospholi

se response to bacterium|positive regulation of I-kappaB kinase/NF-kappaB signal

in modification
activity, RNA polymerase II-specific|DNA-binding transcription repressor activit

osol|spermatogenesis|protein C-terminus binding|protein kinase binding|actin cyt

rix adhesion|extracellular matrix organization|collagen-containing extracellul
ion|Circadian entrainment|Glutamatergic synapse
tin|RNA polymerase II proximal promoter sequence-specific DNA binding|RNA poly
|integral component of membrane|neuron projection development|regulation of gro

ort-chain carboxylesterase activity|cellular lipid metabolic process|phosphati

protein transport|apoptotic process|actin filament organization|cell adhesion|
iption by RNA polymerase II

sponse to type I interferon|defense response to virus|protein K63-linked ubiqu
bolic process|smoothened signaling pathway|regulation of signal transduction|n

a membrane|ion transport|sodium:inorganic phosphate symporter activity|sodium-

de-generating NADPH oxidase activator activity|cell junction|extracellular mat

lved in RNA-mediated transposition|metal ion binding|nucleic acid phosphodiest
sitive regulation of cell proliferation|regulation of G2/M transition of mitot
sis|chromosome passenger complex localization to spindle midzone|cell division

omponent of membrane|regulation of proteolysis|membrane protein proteolysis|re
us|chromatin remodeling|regulation of transcription by RNA polymerase II|viral
ogen activation|synaptic cleft|terminal bouton

iferation|positive regulation of histone deacetylase activity
ription by RNA polymerase II|mesoderm development|insulin receptor signaling p

ion|chromatin remodeling|transcription, DNA-templated|regulation of transcript
growth factor beta receptor signaling pathway|spermatogenesis|cysteine-type p

endent 3'-5' RNA helicase activity
ctivity, RNA polymerase II-specific|proximal promoter DNA-binding transcriptio
brane|apicolateral plasma membrane|lateral plasma membrane|midbody|protein hom

ıthway

ɔrt, Golgi to ER|Golgi organization|positive regulation of organelle organizatic
ɔn binding|membrane|integral component of membrane|viral process|specific granu
ar membrane-bounded organelle
er|termination of RNA polymerase I transcription|transcription by RNA polymerase

: into nucleus|cell cycle|response to mechanical stimulus|response to glucose|ke

r pattern specification|microtubule cytoskeleton|neuron differentiation|sequence
embrane|azurophil granule membrane|alpha-tubulin binding|neutrophil degranulatic

ıe-mediated oocyte maturation|Regulation of lipolysis in adipocytes|Melanogenesi

ıding
:y of stem cells

dhesion|growth factor activity|heparin binding|regulation of gene expression|fil
ılation of translation|protoporphyrinogen IX biosynthetic process|iron ion trans
cription|mRNA polyadenylation|RNA splicing|nuclear matrix|RNA polymerase core en

:ivity|fibroblast growth factor receptor signaling pathway|negative regulation c

ron differentiation|retinal ganglion cell axon guidance|sequence-specific DNA b

regulation of transcription, DNA-templated|nucleosome positioning|chromosome cor
ⅠH oxidase activity|oxidoreductase activity|oxidoreductase activity, acting on p
repair

cytosol|ubiquitin-dependent protein catabolic process|integral component of meml

ripotency of stem cells

ıe|positive regulation of MAPK cascade|inner ear receptor cell differentiation|r

cochondrion|metal ion binding

on molecules|cytoskeletal protein binding|response to cold|cell surface|integra

tein transferase activity|cullin family protein binding

repair via nonhomologous end joining|ubiquitin-dependent protein catabolic proce

s|mitochondrion|cytosol|transcription initiation from RNA polymerase I promoter
anscription by RNA polymerase II|protein-containing complex|protein homodimeriza

ransport|cellular calcium ion homeostasis|epidermis development|proton-exporting

tive regulation of smooth muscle cell differentiation|glutamatergic synapse|ext

racellular transport|cytoplasmic microtubule organization|positive regulation of
membrane|nucleoplasm|nuclear euchromatin|nuclear heterochromatin|spindle|chroma

ll differentiation|actomyosin structure organization|negative regulation of ATPa

ess

inding
cicellular organism development|transcription factor binding|cell proliferation
transduction|blood coagulation|NAD(P)H oxidase activity|oxidoreductase activity,

llular matrix|trans-Golgi network membrane|cell-cell adhesion mediated by cadhe

[illegible] mRNA polyadenylation|axon cytoplasm|ribonucleoprotein complex

[illegible]rt|defense response|integral component of mitochondrial membrane|calcium chann[illegible]
[illegible]lization to ciliary membrane
[illegible]slational fidelity|metal ion binding
[illegible]ment
component of membrane|vesicle-mediated transport|calcium ion regulated exocyto[illegible]

[illegible]dbody|positive regulation of intracellular transport|centriolar satellite|micr[illegible]
[illegible]fic DNA binding|MLL3/4 complex|metal ion binding|dioxygenase activity|histone [illegible]
[illegible]ral process|negative regulation of protein ubiquitination|negative regulation [illegible]
[illegible]dification|cellular protein metabolic process

[illegible]oteasome-mediated ubiquitin-dependent protein catabolic process|post-translati[illegible]

[illegible]lar matrix structural constituent conferring compression resistance|collagen f[illegible]

[illegible]thionine-dependent methyltransferase activity|snRNA metabolic process|snRNA bi[illegible]

[illegible]i transport vesicle|regulation of ER to Golgi vesicle-mediated transport|endop[illegible]

[illegible]modimerization activity|recycling endosome

[illegible]l differentiation|lymph node development|Peyer's patch development

[illegible]ticulum|proteolysis|cell cycle arrest|response to wounding|negative regulation

[illegible]tracellular exosome

[illegible]
[illegible]acellular|endosome organization|endosomal transport|phosphatidylinositol-3,4-b[illegible]

[illegible]gnaling pathway|positive regulation of I-kappaB kinase/NF-kappaB signaling|ext[illegible]

[illegible]ative regulation of cell growth|ciliary basal body|histone H3 acetylation|hist[illegible]
[illegible] signaling pathway

ation of ventricular cardiac muscle cell membrane repolarization|potassium ion e
tion|translation|DNA-dependent ATPase activity|transcription factor binding|RNA-

tor activity|nucleoplasm|transcription regulatory region DNA binding|positive re

ation of basement membrane assembly involved in embryonic body morphogenesis
lar response to glucose stimulus|positive regulation of exocytosis|negative regu
matrix|extracellular exosome|positive regulation of mesenchymal stem cell proli

ific DNA binding|MLL3/4 complex|metal ion binding|dioxygenase activity|histone d

of plasma membrane|GABA-ergic synapse|regulation of synaptic membrane adhesion
tal muscle satellite cell maintenance involved in skeletal muscle regeneration|p

icrovillus|brush border|protein targeting|protein targeting to membrane|protein

s|nucleoplasm|cytoplasm|neuron differentiation|positive regulation of transcript

gral component of membrane|protein homodimerization activity|intracellular membr

mponent of membrane|basolateral plasma membrane|regulation of protein secretion

ion resistance|extracellular matrix organization|extracellular matrix|synapse|co
tamylation|axon|dendrite|adult behavior|motile cilium|vesicle localization|tubul
ption by RNA polymerase II|zinc ion binding|identical protein binding|innate imm

synthase activity

ochondrial translation|mitochondrial translational elongation|mitochondrial tran

lular response to ionizing radiation

e regulation of platelet activation|positive regulation of gene expression|regul
r signaling pathway|heparin binding|membrane|integral component of membrane|plat

mediate filament binding|positive regulation of intermediate filament depolymeri

t of plasma membrane|regulation of endocytosis|clathrin-coated vesicle|cell lead

oof of mouth development|mesenchyme development

-binding transcription repressor activity, RNA polymerase II-specific|blood vess

-binding transcription repressor activity, RNA polymerase II-specific|somitogene
ort|Golgi organization|lysosomal transport|axon guidance|spectrin|membrane|kines

egative regulation of transcription by RNA polymerase II|RNA polymerase II dista

tical endoplasmic reticulum|phosphatidylinositol binding|positive regulation of
fusion|cellular response to leptin stimulus|positive regulation of transcription

e regulation by host of viral process|metal ion binding

adhesion|positive regulation of cell-substrate adhesion|extracellular matrix str

remodeling|beta-catenin binding|response to activity|hippocampus development|ch

tin organization|ubiquitin-dependent protein catabolic process|apoptotic process

nucleus|nucleoplasm|cytoplasm|cytosol|DNA repair|mRNA export from nucleus|pepti

rmatogenesis|positive regulation of cell proliferation|epidermis development|cel
aling pathway|T cell receptor signaling pathway|NF-kappa B signaling pathway

ific DNA binding|MLL3/4 complex|metal ion binding|dioxygenase activity|histone d

membrane transporter activity|L-tryptophan transmembrane transporter activity|sy

onjugating enzyme binding|negative regulation of T cell proliferation|ubiquitin
lation of viral transcription|identical protein binding|protein homodimerization
-|Notch signaling pathway|Jak-STAT signaling pathway|TGF-beta signaling pathway|

on of ketone bodies|Pyruvate metabolism|Glyoxylate and dicarboxylate metabolism|
e metabolism|Fat digestion and absorption|Carbon metabolism|Arginine and prolin
Pase binding|extrinsic component of membrane|transport vesicle membrane|platelet

olved in RNA-mediated transposition|metal ion binding|nucleic acid phosphodieste

hannel complex|extracellular exosome|volume-sensitive chloride channel activity
ng pathway

membrane|metal ion transport|positive regulation of protein ubiquitination|negat

growth factor beta1 production|positive regulation of collagen biosynthetic proc
activator activity, RNA polymerase II-specific|branching involved in blood vess
ignal transduction

ateral plasma membrane|neuron projection|synapse|postsynaptic membrane|synaptic

ain binding|epithelial cell-cell adhesion

 fiber|ciliary basal body-plasma membrane docking

tivity|carboxylic acid metabolic process|sphingolipid catabolic process|pyridoxa
erase II-specific|angiogenesis|in utero embryonic development|heart looping|suck

|early endosome lumen|filamentous actin|recycling endosome lumen|protein homodim

synapse|Ras signaling pathway|PI3K-Akt signaling pathway|Circadian entrainment|G

on of glucose transmembrane transport|cytoplasmic vesicle membrane|positive regu

cellular zinc ion homeostasis|ferrous iron transmembrane transporter activity|in

cytoskeleton|actin cytoskeleton organization|filamentous actin

 factor activity, RNA polymerase II-specific|DNA-binding transcription activator

activity, RNA polymerase II-specific|DNA binding|chromatin binding|nucleus|regul
rane|apical junction complex|apical cortex|regulation of establishment of planar
ific DNA binding|MLL3/4 complex|metal ion binding|dioxygenase activity|histone d
ulation

nogenesis|axon guidance|axonal fasciculation|brain development|axon|neuron proje

binding|nucleus|nucleoplasm|cytoplasm|centrosome|regulation of transcription by

leosome assembly|DNA replication-independent nucleosome assembly|chromatin remod
llular calcium ion homeostasis|nuclear envelope organization|regulation of heart
 process|nucleocytoplasmic transport|cellular response to starvation|Z disc|posi
led receptor signaling pathway|lamellipodium|endocytic vesicle|regulation of Rho
sm|nucleolus|transcription by RNA polymerase II|transcription elongation from RN
arch and sucrose metabolism|Glycolysis / Gluconeogenesis

ediated transport|kinesin binding|receptor signaling complex scaffold activity|p

uvate|oxidoreductase activity
to DNA damage stimulus|female gamete generation|enzyme binding|MutLbeta complex

lation of cyclin-dependent protein serine/threonine kinase activity|positive reg

tive regulation of transcription, DNA-templated|positive regulation of transcrip

DNA-binding transcription factor activity|nucleus|regulation of transcription by

ng

ulation of signaling receptor activity
olysis / Gluconeogenesis
s system|actin cytoskeleton organization|regulation of Rho protein signal transd

ociated glycoprotein complex|syntrophin complex|cell junction|PDZ domain binding
i-associated vesicle|cytosol|protein phosphorylation|apoptotic process|cellular

erase II-specific|cellular glucose homeostasis|chromatin binding|double-stranded
s of amino acids|Glutathione metabolism

transport|small GTPase mediated signal transduction|Rab GTPase binding|protein

oagulation|blood coagulation, intrinsic pathway|platelet activation|identical pr

ng|phagophore assembly site membrane|protein localization to phagophore assembly

signaling pathway

xonal transport of mitochondrion|integral component of mitochondrial outer membr

:ion|substrate adhesion-dependent cell spreading|identical protein binding|prote
olytic cleavage to generate mature 5'-end of SSU-rRNA from (SSU-rRNA, 5.8S rRNA,
phosphatidylinositol-4,5-bisphosphate binding|cytoplasm|cytoskeleton|plasma meml

: process|N-acyltransferase activity|phosphatidylcholine metabolic process
positive regulation of peptidyl-serine phosphorylation|response to drug
ılum|endoplasmic reticulum membrane|endoplasmic reticulum-Golgi intermediate cor

otein binding|membrane raft|positive regulation of transcription, DNA-templated

on paired donors, with incorporation or reduction of molecular oxygen, NAD(P)H

g|regulation of protein ubiquitination|negative regulation of NF-kappaB transcri

sma membrane adhesion molecules|G protein-coupled receptor signaling pathway|int
activity|ubiquitin protein ligase binding|peptidyl-tyrosine dephosphorylation|c

ıe development|learning or memory|voltage-gated potassium channel complex|potass
rotein transport|peptidyl-tyrosine phosphorylation|cell differentiation|bone mir

se activity|peptide cross-linking via chondroitin 4-sulfate glycosaminoglycan|ex

ative regulation of Wnt signaling pathway|integral component of organelle membra
e antigen-transporting ATPase activity|integral component of membrane|MHC prote

itive regulation of extrinsic apoptotic signaling pathway

e lumen|neutrophil degranulation|collagen-containing extracellular matrix|extrac

nembrane|integral component of plasma membrane|heparin binding|integral componen

in-glucuronate 5-epimerase activity

biosynthetic process|brain development|phototransduction|phosphatidylcholine tı
activity, RNA polymerase II-specific|core promoter sequence-specific DNA binding

ase D signaling pathway|ErbB signaling pathway|Insulin signaling pathway|Ras si
ne|Carbon metabolism|Longevity regulating pathway|FoxO signaling pathway|Longevi

olgi to ER|Golgi organization|cell proliferation|glycoprotein biosynthetic proce

hate pathway|Fructose and mannose metabolism|Glycolysis / Gluconeogenesis
lipid homeostasis|all-trans-retinol binding
ng|membrane|lamellipodium|extracellular matrix organization|negative regulation

oothened signaling pathway|ciliary membrane

of cell morphogenesis|negative regulation of transforming growth factor beta rec

a membrane|water transport|Rho protein signal transduction|negative regulation c
NA binding|nuclear pericentric heterochromatin|sequence-specific DNA binding

activity, RNA polymerase II-specific|proximal promoter DNA-binding transcriptio
s|AMPK signaling pathway|Tight junction|Sphingolipid signaling pathway|PI3K-Akt

ix structural constituent|extracellular space|trans-Golgi network|cell adhesion

gradation|Lysosome

naling pathway|Sphingolipid signaling pathway|PI3K-Akt signaling pathway|Arginin
nscription factor activity, RNA polymerase II-specific|chondrocyte differentiat
ulation|heparin binding|negative regulation of cell proliferation|negative regu

|regulation of membrane potential|detection of mechanical stimulus involved in s

:hway|positive regulation of Wnt signaling pathway|bone mineralization|embryoni(

receptor activity|cell surface bile acid receptor signaling pathway|regulation
)ination|enzyme activator activity|membrane|ATPase activity|regulation of DNA-de
a assembly

)athway|Osteoclast differentiation|PI3K-Akt signaling pathway

n utero embryonic development|nucleus|nucleoplasm|nucleolus|transcription initia

in structure organization|regulation of actin filament-based process|specific gr

ytoskeletal protein binding|cell surface|calcium-dependent cell-cell adhesion vi
homeostasis|chloride transmembrane transport
:ivity, RNA polymerase II-specific|DNA-binding transcription repressor activity,
'KG signaling pathway|Wnt signaling pathway

)nogenesis|negative regulation of protein targeting to membrane

:otein complex|sarcoglycan complex|dendrite membrane|sarcolemma

:ive regulation of ubiquitin-dependent protein catabolic process

|syntrophin complex|PDZ domain binding|sarcolemma|neuroligin family protein binc

·gic signaling in cardiomyocytes|Aldosterone synthesis and secretion|Insulin sec

)onse to arsenite ion
iomineral tissue development|protein serine/threonine kinase activator activity

ıl adhesion
ooligomerization|cullin family protein binding
drial outer membrane
|cytoskeleton organization|G protein-coupled receptor signaling pathway|regulati

gnaling pathway|PI3K-Akt signaling pathway|Regulation of actin cytoskeleton|Foc
naling|innate immune response|protein homooligomerization
phenolic acid acyl-glucuronide esterase activity

olved in RNA-mediated transposition|metal ion binding|nucleic acid phosphodieste

ak repair via nonhomologous end joining|cell proliferation|positive regulation o
of neuron projection development|growth cone|neuronal cell body|dendritic spine
ninergic synapse|Osteoclast differentiation|Glutamatergic synapse|MAPK signaling

ction development|postsynaptic density|protein kinase binding|cell junction|axon
ng pathway|FoxO signaling pathway|cGMP-PKG signaling pathway|Longevity regulatin

g|identical protein binding|protein homodimerization activity|intracellular memb
oplasmic reticulum|Golgi stack|plasma membrane|N-acetylglucosamine-6-sulfatase a

embrane|regulation of Rho protein signal transduction|negative regulation of apo

in homooligomerization|proximal tubule development
cy

itive regulation of actin filament polymerization|actin nucleation|pointed-end a

athway

olipid biosynthetic process|negative regulation of apoptotic process|sphingolipi

|cell junction|filopodium|dendrite|Rac guanyl-nucleotide exchange factor activi[illegible]

[illegible]thway
[illegible]aling pathway|Osteoclast differentiation|RIG-I-like receptor signaling pathway|

[illegible]osphate binding|intermediate filament cytoskeleton|phosphatidylinositol-4-phos[illegible]
[illegible]r binding|cell proliferation|Schwann cell differentiation|negative regulation [illegible]

[illegible]ap1 signaling pathway|Long-term depression|Dopaminergic synapse|Circadian entra[illegible]
ion binding
DNA damage

[illegible]smic ribonucleoprotein granule|identical protein binding|ribonucleoprotein comp[illegible]
[illegible]ion|Platelet activation|Sphingolipid signaling pathway|Progesterone-mediated oc[illegible]

process|positive regulation of protein kinase A signaling|nuclear speck|cellul[illegible]

[illegible]gi membrane|protein homodimerization activity|3'-phosphoadenosine 5'-phosphosu[illegible]

[illegible] of transcription elongation from RNA polymerase II promoter

[illegible]ling pathway|Melanogenesis|Dopaminergic synapse|Salivary secretion|Adrenergic s[illegible]

[illegible]ing via chondroitin 4-sulfate glycosaminoglycan|extracellular matrix structura[illegible]

[illegible]sma membrane|microvillus|clathrin-coated pit|cell cortex|intracellular protein

[illegible]ellular region|basement membrane|extracellular space|endoplasmic reticulum lum[illegible]
[illegible]lasmic vesicle membrane|regulation of small GTPase mediated signal transductio[illegible]

ıron death|protein transport from ciliary membrane to plasma membrane

oment|positive regulation of sodium ion transport|intercalated disc|integral coı

protein signal transduction

ally oriented cell migration|cerebral cortex tangential migration|corticospinal

ocess|nuclear speck|RNA N6-methyladenosine methyltransferase complex
of protein oligomerization|metal ion binding|positive regulation of protein ox

ing

dosome to lysosome transport|syntaxin binding|AP-3 adaptor complex|clathrin-coa
ılation of CD4-positive, alpha-beta T cell differentiation|positive regulation

ton-dependent intracellular transport|cytoplasmic microtubule organization|centı
kinase activity

transcription factor complex|cytoplasm|cytosol|regulation of transcription by RN

polymerase II|nervous system development|embryonic pattern specification|nuclea
nors|histone demethylase activity (H3-K4 specific)|histone H3-K4 demethylation|r

surface|apical plasma membrane|protein kinase binding|chondroitin sulfate bios

n repressor activity, RNA polymerase II-specific|DNA-binding transcription facto
vay|NF-kappa B signaling pathway
rosome|cytosol|cytoskeleton|microtubule|chromosome segregation|centrosome cycle

protein phosphorylation|lipid binding|positive regulation of neuron projection
omponent of membrane|apical plasma membrane|transport vesicle membrane|azurophi

sduction|regulation of extracellular matrix disassembly|smooth muscle cell migra
neter

iption factor activity|response to lipopolysaccharide|negative regulation of in

anscription regulatory region DNA binding|positive regulation of transcription,
te|phagocytic vesicle membrane|animal organ regeneration|specific granule|defens

sitive regulation of protein sumoylation|phosphatidylinositol 3-kinase binding|
tor signaling pathway|regulation of adenylate cyclase activity|extracellular exo

e reclamation|Salivary secretion|Adrenergic signaling in cardiomyocytes|Endocri

|negative regulation of cell migration|growth cone|filamin binding|filamentous a
binding|toxin transport
rganism growth|dendritic spine|synapse|Schaffer collateral - CA1 synapse|regula

cess|positive regulation of phosphatidylinositol 3-kinase activity|inositol lip

DNA binding|nucleus|nucleoplasm|transcription factor complex|cytoplasm|steroid

n activator activity, RNA polymerase II-specific|transcription cofactor binding

e to hydrogen peroxide|cellular response to cytokine stimulus|cellular response

l binding|retinol binding|lung development|adrenal gland development|female gen

pathway|cAMP signaling pathway|MAPK signaling pathway|Fc epsilon RI signaling pa

lular matrix organization|hydrogen peroxide catabolic process|metal ion binding
doderm interaction involved in midgut development|heart development|DNA binding,

atalytic complex

art of cell|positive regulation of cell adhesion|positive regulation of keratin

egulation of transcription by RNA polymerase II|dopamine receptor signaling path
n|cellular response to retinoic acid
rane-bounded organelle|regulation of intracellular pH|recycling endosome|recycl

in phosphorylation|signal transduction|transmembrane receptor protein tyrosine l

tion activator activity, RNA polymerase II-specific|extracellular space|nucleus

ıbunit processome|post-translational protein modification|Cul4-RING E3 ubiquitin
ediate compartment membrane|positive regulation of integrin activation|positive

ranslation|negative regulation of viral genome replication|negative regulation o

olipid translocation

lipase C-activating G protein-coupled receptor signaling pathway|regulation of l

ation|negative regulation of viral entry into host cell|defense response to viru
m|Wnt signaling pathway
ing|cell projection assembly|actin cytoskeleton organization|cell projection|reg

er bond hydrolysis

rhythm|Wnt signaling pathway

|specific granule lumen|identical protein binding|neutrophil degranulation|nega

ase II core promoter sequence-specific DNA binding|proximal promoter DNA-binding
localization sequence binding|Ran GTPase binding|membrane|nuclear membrane|prote
egulation of anterograde dense core granule transport|regulation of cellular pro
on|neurofilament cytoskeleton organization|Schaffer collateral - CA1 synapse|pos
regulation of exocytosis|metal ion binding|parallel fiber to Purkinje cell syna

ess-activated protein kinase signaling cascade|activation of protein kinase act

APK signaling pathway|Cholinergic synapse|Retrograde endocannabinoid signaling|V
onse to virus|postsynaptic density|endosomal transport|peptidyl-serine phosphor
on of protein secretion|negative regulation of protein secretion|regulation of

d receptor signaling pathway|adenylate cyclase-activating G protein-coupled rec

factor beta receptor signaling pathway|metal ion binding|negative regulation of

nding|nucleus|nucleoplasm|mitochondrion|mitochondrial matrix|cytosol|protein qu

s|central nervous system development|learning or memory|cytokine-mediated signa

f cytokine production|activation of innate immune response|double-stranded DNA

of transcription, DNA-templated
esin binding|cell junction|X11-like protein binding|postsynaptic membrane|posit

ocyte maturation|Regulation of lipolysis in adipocytes|Melanogenesis|Dopaminergi

tivity|negative regulation of fibroblast growth factor receptor signaling pathwa

urotransmitter catabolic process|metal ion binding|N-formylglutamate deformylase

proliferation|Wnt signaling pathway|positive regulation of histone acetylation

keletal system morphogenesis|cilium assembly|ciliary tip|ciliary base
ne|intracellular membrane-bounded organelle|protein K27-linked ubiquitination|po

assembly|SUMO transferase activity|protein kinase binding|NAD-dependent histone
intenance of cell polarity|Rho protein signal transduction|regulation of cell sh

oid signaling pathway|T cell receptor signaling pathway|Focal adhesion

solateral plasma membrane|apical plasma membrane|peptidyl-serine phosphorylation
eron-gamma-mediated signaling pathway|type I interferon signaling pathway|cellu

nesis

egulation of MAPK cascade|cell development|SMAD protein signal transduction

GTPase mediated signal transduction|brain development|membrane|actin cytoskele

ein signal transduction|positive regulation of apoptotic process|cadherin bindi

ı|PML body|nuclear speck|cell junction|Smc5-Smc6 complex|positive regulation of

ans-Golgi network|integral component of plasma membrane|protein targeting|prote[illegible]
ı-coupled receptor signaling pathway|activation of adenylate cyclase activity|s[illegible]
neuron differentiation|specification of loop of Henle identity

ıg|steroid hormone receptor activity|nucleoplasm|transcription initiation from [illegible]

: process|cell surface|response to endoplasmic reticulum stress|specific granul[illegible]

:ess|post-translational protein modification|protein-containing complex binding

ı of long-term neuronal synaptic plasticity|regulation of stress fiber assembly

; / Gluconeogenesis|Amino sugar and nucleotide sugar metabolism|Starch and sucr[illegible]

ı binding|extracellular exosome
f blood pressure|metallopeptidase activity|zinc ion binding|integral component [illegible]

inner kinetochore|chromatin binding|nucleus|nucleoplasm|cytosol|nucleosome ass[illegible]

ınd-gated ion channel activity|ion transmembrane transport|regulation of membra[illegible]

llular response to calcium ion

: signaling pathway via death domain receptors|protein binding, bridging|regula[illegible]

|cytosol|plasma membrane|mRNA catabolic process|somatic stem cell population ma[illegible]
ıy

ing in cell cycle arrest|telomere maintenance via telomerase|positive regulatio[illegible]

organization|methyl-CpG binding|telomeric heterochromatin|methylated histone bi

of calcineurin-NFAT signaling cascade

er bond hydrolysis

velopment|negative regulation of adenylate cyclase-activating adrenergic recept
eacetylase activity|organelle membrane

sentation of exogenous peptide antigen via MHC class II|kinesin binding|protein-

etabolism|Limonene and pinene degradation|Valine, leucine and isoleucine degrada

ignaling pathway|extracellular exosome|extracellular vesicle biogenesis|extrace

erase II transcription factor binding|RNA polymerase II repressing transcriptio

s|Salivary secretion|Adrenergic signaling in cardiomyocytes|Aldosterone synthes

leton|intermediate filament|plasma membrane|cell-cell junction|cell-cell adhere

ion binding|calcium-dependent phospholipid binding|phosphatidylinositol-4,5-bi

ng extracellular matrix

ncorporation or reduction of molecular oxygen, reduced ascorbate as one donor, a
n activator activity, RNA polymerase II-specific|nucleus|cytoplasm|nervous syste
uration|Regulation of lipolysis in adipocytes|Melanogenesis|Salivary secretion|A
l repressor complex|negative regulation of transcription, DNA-templated|positive

rt|enzyme activator activity|zinc ion binding|viral process|vesicle-mediated tra

differentiation|cell junction|negative regulation of apoptotic process|regulati
ryonic forelimb morphogenesis|regulation of retinoic acid receptor signaling pa

by RNA
tein signal transduction|negative regulation of programmed cell death|negative r

ding|protein homooligomerization|endoplasmic reticulum tubular network|endoplasm

bule tethering complex|actin filament binding|nucleus localization|establishmen

tion|perikaryon|regulation of neuron apoptotic process|synapse|postsynaptic meml
ntermediate filament binding|hemidesmosome|negative regulation of cell migratio

s|nucleoplasm|cytoplasm|cytosol|polysome|mRNA cap binding complex|translation|W

her lipid metabolism|Long-term depression|Phospholipase D signaling pathway|Ara

neat
idian entrainment|Glutamatergic synapse

d|presynaptic active zone|cellular response to retinoic acid|cellular response
DNA-binding transcription repressor activity, RNA polymerase II-specific|urete

lation of cell proliferation|cyclin-dependent protein serine/threonine kinase r
binding|ubiquitin protein ligase activity

ation of rRNA processing

ation of viral entry into host cell|positive regulation of viral entry into hos

egulation of stress fiber assembly

localization to membrane
upled receptor signaling pathway|integral component of membrane|cytoplasmic ves

naling pathway|early endosome membrane|intracellular membrane-bounded organelle

rase II|regulation of stress fiber assembly|positive regulation of stress fiber

n|actin cytoskeleton organization|small GTPase binding|ruffle membrane|regulati
ndoplasmic reticulum lumen|glucose metabolic process|heart development|heparin

Circadian entrainment|Phosphatidylinositol signaling system|Glutamatergic synap

canonical Wnt signaling pathway

of protein kinase B signaling|negative regulation of apoptotic signaling pathwa
leus|nucleoplasm|histone demethylase activity|histone demethylase activity (H3-I

oprotein particle assembly
trix organization|metal ion binding|collagen-containing extracellular matrix|pos
activity|structural constituent of muscle|intercalated disc|sarcomere|Z disc|sl

c DNA binding|DNA-binding transcription factor activity, RNA polymerase II-spec:
ular matrix organization|collagen catabolic process|protein binding, bridging|ex

mitochondrial membrane potential|pyrimidine nucleotide import into mitochondri
biosynthesis via nicotinamide riboside salvage pathway|intracellular membrane-l

ctivity|glutamatergic synapse

ssembly|ciliary tip

ng|endosome membrane|nucleokinesis involved in cell motility in cerebral cortex

ition of mitotic cell cycle|protein kinase binding|protein domain specific bind:

RNA transport|toll-like receptor 3 signaling pathway|positive regulation of grow

thylation of male pronucleus|positive regulation of transcription by RNA polyme

sma membrane cell adhesion molecules|visual perception|response to bacterium|cl[illegible]

[illegible]thway|Inflammatory mediator regulation of TRP channels
[illegible]t of membrane|protein kinase binding|protein kinase activator activity|cytoplas[illegible]
[illegible]n|vesicle membrane|integral component of membrane|regulation of macroautophagy

by RNA|DNA methylation involved in gamete generation|P granule|pi-body

[illegible]lex binding

[illegible]ansduction|G protein-coupled receptor signaling pathway|neuropeptide signaling

[illegible]ment|regulation of G protein-coupled receptor signaling pathway|anterior/poste[illegible]

lasm|transcription factor complex|cytoplasm|centrosome|transcription, DNA-templ[illegible]

ion of cell morphogenesis|cell junction|early endosome membrane|neuronal cell b[illegible]

-coupled peptide receptor activity|integral component of membrane|positive regu[illegible]

th|negative regulation of TOR signaling|negative regulation of GTPase activity|
er bond hydrolysis

rin binding|regulation of small GTPase mediated signal transduction|regulation (

drite self-avoidance|cell-cell adhesion mediator activity|parallel fiber to Purl

ing pathway

ent|ATPase activity|Rab GTPase binding|protein kinase binding|actin cytoskeleton
esentation of exogenous peptide antigen via MHC class II|intraciliary transport
dehydroascorbic acid transmembrane transporter activity|specific granule membran

positive regulation of transcription of Notch receptor target|synapse assembly|

g|intrinsic apoptotic signaling pathway in response to DNA damage by p53 class m
se activity|zinc ion binding|response to bacterium|cell surface|negative regula

t|late endosome membrane|phosphatidylinositol-3,4-bisphosphate binding|actin fi
methylation|arsonoacetate metabolic process|positive regulation of cell growth

tin-dependent protein catabolic process|cellular response to DNA damage stimulus

y|regulation of striated muscle tissue development|cerebellar granule cell diff

eneration|response to axon injury|collagen-containing extracellular matrix

tion|regulation of cell shape|response to salt stress|cellular water homeostasis

tion of chromosome organization|centromere complex assembly|spindle midzone|cel

ransport from ciliary membrane to plasma membrane

-beta signaling pathway|Sphingolipid signaling pathway|PI3K-Akt signaling pathw

synapse to nucleus signaling pathway

en processing and presentation|phagocytic vesicle membrane|GTP-dependent protei

legradation|Ascorbate and aldarate metabolism|Glycolysis / Gluconeogenesis

er bond hydrolysis

ption factor activity|regulation of proteasomal ubiquitin-dependent protein cat
|chitin binding|response to mechanical stimulus|positive regulation of peptidyl-
erization activity
inositol signaling system

ynapse|anchored component of postsynaptic density membrane|anchored component o

n repressor activity, RNA polymerase II-specific|nucleus|metal ion binding

egulation of transcription by RNA polymerase II|nervous system development|insu

AD protein phosphorylation|snRNA stem-loop binding|protein homodimerization act

olymerase II-specific|proximal promoter DNA-binding transcription repressor act
n activator activity, RNA polymerase II-specific|fibrillar center|chondrocyte d
olymerase II-specific|proximal promoter DNA-binding transcription activator act

ty, RNA polymerase II-specific|chromatin binding|DNA-binding transcription fact

anization|centriolar satellite|tube formation|ciliary basal body-plasma membrane

binding
ediated transport|COPI vesicle coat

protein maturation by iron-sulfur cluster transfer

ome|negative regulation of cell proliferation|COP9 signalosome assembly|post-tr

egion of cytoplasm|xenophagy|positive regulation of autophagosome maturation

ase activity|specific granule membrane|identical protein binding|protein homodi

ic vesicle|protein localization to Golgi apparatus|dynactin binding|mRNA transp

ne assembly

iption factor activity|response to lipopolysaccharide|negative regulation of in

sicle|cleavage furrow formation|positive regulation of GTPase activity|extracel

granule|spectrin binding|transport vesicle membrane|response to insulin|peptid

ne demethylase activity (H3-K9 specific)|histone H3-K9 demethylation|methylated
ein domain specific binding|cell junction|perinuclear region of cytoplasm|neuro

a receptor signaling pathway|ruffle membrane|positive regulation of cell adhesi
actor activity|nucleus|mitochondrial outer membrane|regulation of transcription

ransport|retrograde vesicle-mediated transport, Golgi to ER|intra-Golgi vesicle-

or binding|negative regulation of erythrocyte differentiation|cell division|pos

complex|establishment of mitotic sister chromatid cohesion|peptidyl-lysine ace
sma membrane docking
state gland morphogenesis|epithelial cell maturation involved in prostate gland
ation of transcription, DNA-templated|multicellular organism development|positi
NA-binding transcription repressor activity, RNA polymerase II-specific|neural
er bond hydrolysis

atrix|extracellular matrix binding|collagen-containing extracellular matrix|ext

se

nscription corepressor activity|nucleus|nucleoplasm|nucleolus|cytoplasm|DNA dam
eral plasma membrane|apical plasma membrane|cell junction|flagellated sperm mot

uron differentiation|response to drug|sequence-specific DNA binding|renal syste
e organization|actomyosin|regulation of macrophage activation|negative regulati

trix|defense response to bacterium|tooth eruption|extracellular matrix binding|

ng transcription factor activity|nucleus|transcription factor complex|regulatio
repressor activity|nucleus|nucleoplasm|cytosol|regulation of transcription by RN

uence-specific DNA binding|cell fate commitment|regulation of gamma-delta T cel
in heterooligomerization

nt|negative regulation of transcription, DNA-templated|positive regulation of tr

ticoid receptor signaling pathway
ospholipid translocation

dhesion|regulation of myosin-light-chain-phosphatase activity|intracellular sig

tile cilium|non-motile cilium assembly

tivity|L-alanine transmembrane transporter activity|L-cystine transmembrane tra

sis|DNA-binding transcription factor activity|transcription corepressor activit

olymerase II|viral genome replication|sequence-specific DNA binding|negative re
|cellular response to amino acid starvation|cellular response to glucose starva

|cytoplasmic vesicle|positive regulation of protein binding|protein homodimeriz

vesicle membrane
oleoylphosphatidylethanolamine)

leukemia inhibitory factor
e to heat|cellular response to unfolded protein|endoplasmic reticulum chaperone

ation of Notch signaling pathway|anatomical structure development|collagen-conta

ament array|protein-containing complex scaffold activity|positive regulation of
demethylase activity (H3-K27 specific)

on of GTPase activity|negative regulation of guanyl-nucleotide exchange factor a
mponent of Golgi membrane|platelet alpha granule lumen|specific granule lumen|in

rate adhesion-dependent cell spreading|actinin binding|neuron projection|metal

Salivary secretion|Adrenergic signaling in cardiomyocytes|Aldosterone synthesis
n|positive regulation of DNA repair|anatomical structure development|extrinsic a

ctivity|mRNA 5'-UTR binding|mRNA transport

nding|protein-containing complex|identical protein binding|positive regulation c
ignaling|intracellular pH reduction
cytoplasm|oogenesis|regulation of cell cycle|positive regulation of nuclear-tra

ss|transmembrane transport|extracellular exosome
uclear chromosome|fibrillar center|hematopoietic progenitor cell differentiation

insulin secretion involved in cellular response to glucose stimulus|insulin bin

ess|axon|dendrite|phosphatidylinositol catabolic process|early endosome membrane

ion of vascular endothelial growth factor receptor signaling pathway|gonadotrop

sequence-specific DNA binding|proximal promoter DNA-binding transcription repres

l ion binding|m7G(5')pppN diphosphatase activity|inositol diphosphate tetrakisph

cells|Focal adhesion|Wnt signaling pathway
ort

egulation of transcription by RNA polymerase II|transcription by RNA polymerase

thway|negative regulation of protein binding|intermediate filament cytoskeleton
/ Gluconeogenesis|Amino sugar and nucleotide sugar metabolism|Starch and sucro

esis|extracellular exosome|ficolin-1-rich granule lumen
abolic process|auditory receptor cell development|L-dopa O-methyltransferase ac
n activator activity, RNA polymerase II-specific|chromatin binding|DNA-binding

aining complex|negative regulation of cholesterol biosynthetic process|negative
ı modification|negative regulation of smoothened signaling pathway|protein homo(

polymerase II-specific|proximal promoter DNA-binding transcription repressor a(

ıal transduction|neuron projection|synapse organization

ction|dendrite|GTPase regulator activity|synapse
eptor complex

t|axon|mitochondrial membrane|neuronal cell body|dendritic spine head|postsynap

eart looping|secondary heart field specification|outflow tract morphogenesis|ca

plate import into mitochondrion

ization|extracellular matrix|post-translational protein modification|cellular p

ıway|Thyroid hormone signaling pathway|Prolactin signaling pathway|Oxytocin sig

binding|protein stabilization|positive regulation of canonical Wnt signaling pa

n|inner ear development|regulation of pathway-restricted SMAD protein phosphoryl

ve regulation of insulin secretion|neuronal dense core vesicle|regulation of syn

rane|organelle transport along microtubule

activity|membrane|extracellular matrix organization|negative regulation of cel

vity

er bond hydrolysis
lex

ılation of protein catabolic process|regulation of dendrite morphogenesis|ubiqu[illegible]

e regulation of DNA-binding transcription factor activity|cartilage homeostasis

iption factor activity|response to lipopolysaccharide|negative regulation of in[illegible]
ransition|DNA binding|DNA-binding transcription factor activity|transcription fa[illegible]

membrane-bounded organelle|cell body|postsynaptic membrane|positive regulation [illegible]

ılatory region DNA binding|positive regulation of transcription, DNA-templated

on|negative regulation of hepatocyte proliferation|positive regulation of integ[illegible]
tive regulation of translation

nucleus|nucleoplasm|regulation of transcription by RNA polymerase II|multicellu[illegible]

sel morphogenesis|neural crest cell migration|DNA-binding transcription factor [illegible]

mbrane|mitochondrial membrane|response to interferon-alpha|innate immune respons[illegible]

tor signaling pathway|synapse assembly|brain development|carbohydrate binding|a[illegible]
s|unfolded protein binding|chaperone binding|chaperone-mediated protein folding

ter DNA-binding transcription activator activity, RNA polymerase II-specific|neu
nscription factor activity

demethylase activity (H3-K27 specific)

|heart development|transcription factor binding|negative regulation of bone min

binding|negative regulation of neuron projection development|astrocyte developm
n repressor activity, RNA polymerase II-specific|DNA binding|double-stranded RN

cellular organism development|structural constituent of muscle|nuclear body|myo

ivity|macrophage activation|neutrophil degranulation|tertiary granule membrane|

sis|Focal adhesion
ion|lamellipodium|positive regulation of cell migration|regulation of growth|ac
junction|filopodium|dendrite|regulation of actin filament polymerization|cortic

ocytes|Melanogenesis|Dopaminergic synapse|Salivary secretion|Adrenergic signali

of cellular component organization

threonine phosphorylation|regulation of RNA splicing|protein autophosphorylatio

tion, DNA-templated|negative regulation of protein sumoylation|identical protei

ity|male genitalia morphogenesis|cell division

t ESCRT complex|calcium-dependent protein binding|extracellular exosome

ity|cytoplasmic microtubule organization|cell division

sing complex|U7 snRNA binding|positive regulation of G1/S transition of mitotic

ellular exosome

lular membrane-bounded organelle

inflammatory response|tertiary granule lumen|ficolin-1-rich granule lumen

transcription factor binding|vasculogenesis|in utero embryonic development|out

uitin protein ligase binding|mitochondrial membrane|negative regulation of prot

lar endothelial growth factor signaling pathway|positive regulation of blood ve

stone deacetylase activity|nucleus|nucleoplasm|transcription factor complex|reg

NF-kappa B signaling pathway
negative regulation of skeletal muscle satellite cell proliferation|negative re
transcription factor activity|transcription corepressor activity|carboxypeptid

e|negative regulation of tumor necrosis factor-mediated signaling pathway|posit

er bond hydrolysis
ng pathway|protein binding, bridging|extracellular matrix|organelle membrane|ne

ome

eduction process|collagen-containing extracellular matrix

c acid binding|positive regulation of systemic arterial blood pressure|DNA-bind
cle|nervous system development|microtubule binding|positive regulation of cell p

silencing|cadherin binding|negative regulation of DNA recombination

uitination|ubiquitin binding|proteasome assembly|endopeptidase activator activi
otein ligase activity

er bond hydrolysis

rase II promoter

zation in cell
rsis / Gluconeogenesis

ne response|negative regulation of cyclin-dependent protein serine/threonine ki

opment|metalloendopeptidase inhibitor activity|negative regulation of cell-subs

:to series

ion of protein import into nucleus|negative regulation of cAMP-dependent protei

linositol binding|regulation of membrane potential|negative regulation of ion t

proteasomal ubiquitin-dependent protein catabolic process|post-translational pr

ng NADPH oxidase activator activity|extracellular matrix disassembly|cell junct

resistance|extracellular matrix|keratan sulfate catabolic process|lysosomal lum
ay|Endocytosis

transport|negative regulation of interleukin-1 beta secretion|intermembrane li[illegible]

ɔsitive regulation of gene expression|protein-containing complex binding|positi[illegible]

s|nucleoplasm|transferase activity|metal ion binding

of vascular endothelial growth factor receptor signaling pathway|cytoplasmic ve[illegible]

ling pathway|integral component of membrane|fibroblast growth factor binding|tr[illegible]

rganelle|perinuclear endoplasmic reticulum

f proton transport|membrane|apical plasma membrane|secretory granule|secretory [illegible]
ane|chondroitin sulfate biosynthetic process|embryonic skeletal system developm[illegible]

nplex
ine N-methyltransferase activity|peptidyl-lysine monomethylation|peptidyl-lysin[illegible]

heart rate|regulation of release of sequestered calcium ion into cytosol by sa

rcellular bridge|actin filament binding
embrane|apical plasma membrane|potassium ion leak channel activity|cell junctio

onal termination

dhesion|cell migration|negative regulation of angiogenesis|extracellular matrix

f cell adhesion|regulation of peptidyl-tyrosine phosphorylation|regulation of m

de antigen via MHC class I, TAP-independent|regulation of T cell anergy|signali

ty, RNA polymerase II-specific|DNA binding|DNA-binding transcription factor act
al cell body|postsynaptic membrane|actin filament binding
quence-specific DNA binding|negative regulation of transcription, DNA-templated
ignaling pathway|extracellular matrix

ane|antiporter activity|solute:proton antiporter activity|chloride ion binding|

evelopment|mast cell secretory granule organization|T cell secretory granule org

f peptidyl-serine phosphorylation|receptor complex|positive regulation of transc

tive zone|calcium ion-regulated exocytosis of neurotransmitter|positive regulati
tion|focal adhesion|axon guidance|synapse assembly|heart development|fibroblast
crotubule binding|response to virus|membrane|nuclear membrane|mitochondrial memb

formation involved in camera-type eye development|DNA binding|transcription coac

er bond hydrolysis

ing

itive regulation of myelination|central nervous system myelin formation|protein

rkinje cell layer maturation|COPI vesicle coat|transport vesicle|COPI-coated ves

GTPase mediated signal transduction|regulation of cell shape|apical plasma memb

sis|clathrin binding|exocytic vesicle|cellular response to calcium ion

tin-dependent protein catabolic process|thiol-dependent ubiquitinyl hydrolase ac

cription factor activity|transcription coactivator activity|transcription corepr

ty, RNA polymerase II-specific|DNA-binding transcription activator activity, RN[illegible]

[illegible]inergic synapse|Hedgehog signaling pathway - fly|PI3K-Akt signaling pathway|Fo[illegible]
acid transport|amino acid transmembrane transporter activity|basic amino acid [illegible]
[illegible]ellular matrix structural constituent conferring compression resistance|extrac[illegible]

[illegible]adian entrainment|Glutamatergic synapse
[illegible]egative regulation of cell-substrate adhesion|Wnt signaling pathway|Wnt-protei[illegible]

[illegible] repressor activity, RNA polymerase II-specific|nucleus|nucleoplasm|cytosol|re[illegible]

|2 iron, 2 sulfur cluster binding|cellular response to cAMP|cellular response t[illegible]
[illegible]kt signaling pathway|Complement and coagulation cascades

iquitin-dependent protein catabolic process|regulation of MAP kinase activity|n[illegible]
[illegible]inding transcription factor activity|nucleus|cytoplasm|ectoderm development|ax[illegible]

[illegible], DNA-templated|protein phosphorylation|apoptotic process|cell proliferation|r[illegible]

[illegible]ansport|positive regulation of protein localization to plasma membrane

tor complex binding|AP-1 adaptor complex binding|glucose homeostasis|type B pan[illegible]

[illegible]king involved in exocytosis|response to toxic substance|protein transport|synt[illegible]

cyclase-inhibiting G protein-coupled receptor signaling pathway|visual learning

binding|collagen-containing extracellular matrix|extracellular exosome

l component of endoplasmic reticulum membrane|endoplasmic reticulum-Golgi interm

ytokine production involved in inflammatory response|positive regulation of inte

-specific|in utero embryonic development|DNA binding|DNA-binding transcription

olipase A2 inhibitor activity|secretory granule|response to lipopolysaccharide|r
ion of cell-cell adhesion|lamellipodium|filopodium|positive regulation of cell r

onent of membrane|cell junction|desmosome|keratinization|gamma-catenin binding|p
omponent of membrane|basolateral plasma membrane|oxalate transmembrane transpor

cle|aggresome|protein kinase binding|protein kinase activator activity|Dbf4-depe

igration|collagen-containing extracellular matrix

nsmembrane transport|regulation of resting membrane potential|calcium ion transm

|anchored component of plasma membrane

|tertiary granule membrane
pical plasma membrane|sleep|excitatory postsynaptic potential|cellular response

nidbody|spindle midzone|chromosome organization|metaphase plate congression|mit

e|cortical cytoskeleton|maintenance of protein location in cell|actomyosin|post

ceptor signaling pathway|NF-kappa B signaling pathway|Inflammatory mediator reg

ganism growth|ciliary basal body|cilium assembly|head development|vesicle tethe
of cell polarity|cell junction|receptor signaling complex scaffold activity|neg

ecific)|histone H3-K36 demethylation
positive regulation of cell proliferation|negative regulation of cell prolifera

nsport|sodium ion import across plasma membrane

ng|DNA-binding transcription factor activity|nucleus|regulation of transcriptio
factor beta receptor signaling pathway|metal ion binding|negative regulation of

oligomerization|potassium ion transmembrane transport|regulation of delayed rec
trograde transport, endosome to Golgi|regulation of inflammatory response|WASH
inment of circadian clock by photoperiod|post-translational protein modificatio

multicellular organism development|growth factor activity|peptidase activity|me
ation|positive regulation of osteoblast differentiation|spermatid differentiati

egative regulation of interleukin-2 production|positive regulation of invadopod[illegible]

junction|desmosome|regulation of cell adhesion|midbody|keratinization|positive [illegible]
lation of transcription, DNA-templated|zinc ion binding|H4 histone acetyltransf[illegible]
ntegrin|cell projection|metal ion binding|perinuclear region of cytoplasm|negat[illegible]

nelial to mesenchymal transition|positive regulation of DNA-templated transcrip[illegible]

arotene catabolic process|xanthophyll metabolic process|oxidoreductase activity,
tein binding|neutrophil degranulation|phagocytic vesicle
junction organization|establishment of endothelial barrier|tricellular tight ju[illegible]

gion of cytoplasm|positive regulation of apoptotic signaling pathway

brane transport|sperm-egg recognition|CatSper complex|regulation of cilium beat

ndoplasmic reticulum|positive regulation of osteoblast differentiation|regulati[illegible]

ption activator activity, RNA polymerase II-specific|distal enhancer DNA-bindin[illegible]
wann cell differentiation|fibroblast growth factor binding|heparan sulfate prot[illegible]

tidylinositol 3-kinase signaling|membrane|viral process|protein ubiquitination|[illegible]

gative regulation of smoothened signaling pathway|embryonic skeletal system mor[illegible]
ar septum development|atrial septum development|septum primum development|atria[illegible]

nvolved in myocardial precursor cell differentiation|mesenchymal to epithelial [illegible]

ng|heme binding|endoplasmic reticulum unfolded protein response|response to vita

itive regulation of p38MAPK cascade

n phosphorylation|hemopoiesis|cell differentiation|BMP signaling pathway|positi

ansporter activity|acyl carnitine transmembrane transporter activity|ornithine
uitin modification-dependent protein binding|error-free translesion synthesis

plast apoptotic process|regulation of reactive oxygen species metabolic process

system development|nucleus|transcription factor complex|cytoplasm|multicellular

in response to oxidative stress|cellular response to starvation|membrane|signal

determination|chromatin binding|nucleus|positive regulation of chromatin bindi

actin cytoskeleton|ErbB signaling pathway|Fc gamma R-mediated phagocytosis|Ras

templated|positive regulation of transcription by RNA polymerase II

n repressor activity, RNA polymerase II-specific|DNA-binding transcription fact

rase complex
ription activator activity, RNA polymerase II-specific|DNA-binding transcriptio

ositive regulation of apoptotic process|regulation of small GTPase mediated sig
ive regulation of insulin receptor signaling pathway|leukocyte migration

atory region DNA binding|pronucleus|positive regulation of translation|negative

lear body|establishment of cell polarity|cell junction|protein-containing compl

of gene expression|negative regulation of endopeptidase activity|membrane|negat

latory region DNA binding

ction|cell surface|regulation of fibroblast growth factor receptor signaling p

thway|metallopeptidase activity|male gonad development|cell surface|integral c

regulation of canonical Wnt signaling pathway|negative regulation of protein lo

lar organism development|postsynaptic density|regulation of cell-cell adhesion|s

ıd secretion|Insulin secretion|PI3K-Akt signaling pathway|Thyroid hormone synthe
egulation of insulin secretion involved in cellular response to glucose stimulus
ositive regulation of cell growth|mitochondrion localization
lasmic vesicle|stereocilium|synapse|regulation of intracellular pH|auditory rece

athway|Sphingolipid signaling pathway|Vascular smooth muscle contraction|cGMP-P
vity|structural constituent of nuclear pore|nuclear membrane|identical protein b
naling pathway|protein-containing complex scaffold activity|protein-containing c

lation of release of cytochrome c from mitochondria|positive regulation of mito

|induction of bacterial agglutination|innate immune response|metal ion binding|

ane|multicellular organism development|epithelial structure maintenance|enzyme b

|sterol transport|membrane|protein domain specific binding|cell junction|intrace

acid metabolism|Biosynthesis of amino acids|Alanine, aspartate and glutamate me

ty, RNA polymerase II-specific|DNA binding|DNA-binding transcription factor act

ganization|spermatogenesis|attachment of spindle microtubules to kinetochore|his

ion|regulation of translational elongation|ferrous iron binding|oxidoreductase a

TP-dependent 3'-5' RNA helicase activity|ribosomal large subunit binding|positi

y gaseous exchange

g|axon|axolemma|actin-dependent ATPase activity|forebrain development|cytoplasm

ic double-stranded DNA binding|ribonucleoprotein complex

egulation of intrinsic apoptotic signaling pathway by p53 class mediator

dhesion|axon guidance|heparin binding|positive regulation of endothelial cell m

ar septum development|atrial septum development|septum primum development|atria

lation|protein homooligomerization|histone H3-K4 trimethylation|histone H3-K27

polymerase II core promoter sequence-specific DNA binding|proximal promoter DN

f protein ubiquitination|regulation of protein stability|adenosine receptor bin

n ion|divalent inorganic cation transmembrane transporter activity

icle|cis-Golgi network|trans-Golgi network|membrane|Golgi cisterna membrane|pos

: process|cellular response to interleukin-1|cellular response to tumor necrosis

:t particle B|photoreceptor connecting cilium|intraciliary transport involved in

:ell junction|intracellular membrane-bounded organelle

signaling pathway|Signaling pathways regulating pluripotency of stem cells|Natur
viral genome replication|negative regulation of defense response to virus by hos
nscription initiation from RNA polymerase II promoter|termination of RNA polymer
lex|transmembrane transport|cellular response to osmotic stress|anion transmembr
ar organism development|metalloendopeptidase inhibitor activity|cell proliferat

e expression|intermediate filament binding|desmosome|structural constituent of

: binding|ATP binding|nucleus|cytoplasm|endosome|plasma membrane|clathrin-coated

itive regulation of p38MAPK cascade

motile cilium assembly|cilium assembly

receptor binding|alpha-2C adrenergic receptor binding|identical protein binding
tion|negative regulation of DNA-binding transcription factor activity|metal ion

olipid binding|extracellular space|nucleus|mitochondrion|cytosol|plasma membrane
er bond hydrolysis

|single fertilization|gamma-tubulin small complex|membrane|cytoplasmic microtub
ative regulation of Rho protein signal transduction|establishment of Golgi loca

gic synapse|Prolactin signaling pathway|Estrogen signaling pathway|Apoptosis|B

smic vesicle membrane|vesicle|intracellular membrane-bounded organelle|receptor

f insulin secretion|calcium channel complex|calcium import into the mitochondri

rane

ne|protein heterotrimerization|SET domain binding
otic centrosome separation|determination of left/right symmetry|microtubule bin

g|positive regulation of bone mineralization|biomineral tissue development|oste

in-containing complex|rRNA primary transcript binding|identical protein binding

e membrane|intracellular membrane-bounded organelle|metal ion binding|protein a

e to amino acid starvation|positive regulation of transcription from RNA polyme

nal transduction|growth factor activity|heparin binding|cell proliferation|tran

small GTPase mediated signal transduction

i transport vesicle|Cul3-RING ubiquitin ligase complex|identical protein bindin[illegible]

demethylase activity (H3-K27 specific)

se|G protein-coupled receptor signaling pathway|oxysterol binding|T cell chemot[illegible]

liferator activated receptor binding|positive regulation of fat cell differenti[illegible]

ort|synapse|dendrite self-avoidance|cell-cell adhesion mediator activity

g|collagen-containing extracellular matrix

re assembly|regulation of telomere maintenance via telomerase|negative regulati[illegible]

ed organelle|negative regulation of ERK1 and ERK2 cascade

gnaling pathway|sensory perception of sound|negative regulation of signal transc

regulation of protein secretion

factor binding|extracellular matrix|collagen-containing extracellular matrix|ex

l ion binding|MLL1 complex|regulation of signal transduction by p53 class media

ome|extracellular vesicle
polymerase II

non-motile cilium assembly

gative regulation of epidermal growth factor receptor signaling pathway|negative

onse to bacterium|positive regulation of epithelial cell migration|lamellipodium

eptor signaling pathway|peripheral nervous system development|integral componen

complex assembly|negative regulation of G2/M transition of mitotic cell cycle|pr
ticellular organism growth|intracellular signal transduction|metal ion binding|r

eus|microtubule organizing center|cytosol|plasma membrane|membrane|positive regu

ase activity|ATP binding|nucleus|cytoplasm|mitochondrion|mitochondrial outer mem

of sound|bile acid and bile salt transport|apical plasma membrane|stereocilium|i
nt of membrane|negative regulation of interferon-gamma production|positive regul

taurine transport|integral component of membrane
y|learning or memory|hormone-mediated signaling pathway|secretory granule|dendr

port

ort

on

endosome membrane|phospholipid translocation|perinuclear region of cytoplasm|re

eptor signaling pathway|NF-kappa B signaling pathway|Chemokine signaling pathway
ifferentiation|axon development

lation of transcription by RNA polymerase II|regulation of G1/S transition of mi
n of actin cytoskeleton|Focal adhesion|Endocytosis
gulation of transcription, DNA-templated|negative regulation of cellular protein

surface|apical plasma membrane|protein kinase binding|chondroitin sulfate biosy

ım stress|IRE1-mediated unfolded protein response|chaperone binding|misfolded pı

ar exosome

cation transport|quaternary ammonium group transport|membrane|toxin transmembra
osome|EARP complex

ingle fertilization|phosphatidylethanolamine binding|external side of plasma mer

on of actin filament bundle assembly|regulation of protein localization|negative

orane|desmosome|cytoplasmic vesicle|keratinization|extracellular exosome|cornif

ement membrane development|laminin binding|laminin-1 binding|proteoglycan bindi
ium|bundle of His development|atrioventricular valve morphogenesis|endocardial

ıway|Platelet activation|Sphingolipid signaling pathway|T cell receptor signalir

oment|positive regulation of sodium ion transport|intercalated disc|integral cor

demethylase activity (H3-K27 specific)

on of actin cytoskeleton organization|gamma-tubulin binding|negative regulation
ion of gene expression|Wnt signaling pathway|cellular response to extracellular
regulation of exocytosis|exocyst localization|cadherin binding involved in cel

tion|AP-1 adaptor complex binding|vesicle targeting, trans-Golgi to endosome|ne

transport|vesicle-mediated transport|ATPase activity|protein kinase binding|mid

ıt ubiquitinyl hydrolase activity|protein K48-linked deubiquitination|positive

er bond hydrolysis

sport|calcium ion regulated exocytosis|syntaxin binding|transport vesicle|clath

otion activator activity, RNA polymerase II-specific|proximal promoter DNA-bind

,

density|cortical actin cytoskeleton|identical protein binding|regulation of ade
ntraciliary transport|cilium assembly|protein localization to cilium|ciliary ti

organelle membrane

n factor stimulus|identical protein binding|estrous cycle|extracellular polysac

on|positive regulation of protein catabolic process|regulation of protein secre

ion involved in bone maturation

on development|membrane|axon|protein homodimerization activity|neuron projectio

rane|myosin binding|endocytic vesicle|PDZ domain binding|positive regulation of

na membrane|apicolateral plasma membrane|axon|intrinsic component of the cytopl

ial sheet|DNA-binding transcription factor activity|nucleus|nucleoplasm|cytosol

er bond hydrolysis

Golgi transport vesicle|receptor tyrosine kinase binding|cell projection|cell bo

mmetric cell division|negative regulation of autophagy|protein transport|protei
eutrophil degranulation|extracellular exosome|tertiary granule lumen|ficolin-1-

n activator activity, RNA polymerase II-specific|angiogenesis|hematopoietic prog

n-gamma production|negative regulation of interleukin-10 production|negative reg

r activity|RNA binding|protein kinase activity|ubiquitin-protein transferase ac

cold-induced thermogenesis
cold-induced thermogenesis

gration|positive regulation of cell-substrate adhesion|histone dephosphorylatio

n activator activity, RNA polymerase II-specific|eye development|DNA binding|DN

ansduction|negative regulation of ERK1 and ERK2 cascade

pautophagy|negative regulation of translation|translation initiation factor bin
|outer membrane|regulation of bone mineralization|inorganic diphosphate transme

body|ubiquitin-dependent protein catabolic process via the multivesicular body

ignal transduction|negative regulation of small GTPase mediated signal transduc

organization|protein-containing complex|mitochondrial DNA-directed RNA polyme
ferentiation|positive regulation of myotube differentiation|cell differentiatio

binding|neuron projection|synapse|postsynaptic membrane|L27 domain binding|MPP

ar response to oxidative stress|cytoplasmic ribonucleoprotein granule|neuronal

tone deacetylase activity|nucleus|nucleoplasm|cytoplasm|histone deacetylation|p

assembly|regulation of focal adhesion assembly

-mediated transport|membrane|viral process|peptidyl-cysteine methylation|antige
membrane|endoplasmic reticulum tubular network membrane organization

ation|hyaluronan biosynthetic process|hyaluronan catabolic process|positive regu

osome|centriole|cytosol|DNA repair|cellular response to DNA damage stimulus|mit
g pathway|Oocyte meiosis|Calcium signaling pathway|VEGF signaling pathway|cGMP-P

on|regulation of neuron projection development|protein ubiquitination|SCF ubiqu

|DNA-binding transcription repressor activity, RNA polymerase II-specific|doubl

ogenesis

l-cell adhesion via plasma membrane cell adhesion molecules

ar organism growth|metal ion binding|oocyte development|homeostasis of number o

complex|potassium ion leak channel activity|regulation of ion transmembrane tr
vesicle|positive regulation of receptor-mediated endocytosis|clathrin coat asse
development|morphogenesis of an epithelium|respiratory system process|DNA bind

-beta signaling pathway|Sphingolipid signaling pathway|PI3K-Akt signaling pathw

steine-type endopeptidase activity involved in apoptotic process|innate immune r

lian rhythm|Oxytocin signaling pathway|Longevity regulating pathway - multiple s

elastic fiber|supramolecular fiber organization

skeletal muscle cell differentiation|intrinsic apoptotic signaling pathway in re

lasma membrane|regulation of transcription by RNA polymerase II|central nervous

r

|actin filament binding|invadopodium

anism development|lung development|embryonic limb morphogenesis|limb morphogenes

embryonic development|DNA-binding transcription factor activity|nucleus|nucleopl

ne methylation, to asymmetrical-dimethyl arginine|negative regulation of protein

eratinization|sarcolemma|costamere|response to estrogen|protein-containing comp

ion|cell-cell adherens junction|bicellular tight junction|mitochondrion organiz
cell-derived neurotrophic factor receptor activity|extrinsic component of memb

phospholipid transport|membrane|integral component of membrane|negative regulat
pendent microtubule motor activity, plus-end-directed|vesicle-mediated transpor

signaling pathway|endosome membrane|positive regulation of gene expression|pos
ane transport|regulation of delayed rectifier potassium channel activity

neutrophil degranulation|cell body|synapse

, DNA-templated|repressing transcription factor binding|negative regulation of

mRNA cleavage required for polyadenylation
iption by RNA polymerase II|metal ion binding|embryonic skeletal system develop

synapse|glutamatergic synapse|anchored component of presynaptic membrane|anchor

zurophil granule|zymogen granule membrane|regulation of mast cell degranulation

ERK2 cascade|regulation of cell cycle G2/M phase transition|protein kinase comp

ipase C-activating G protein-coupled receptor signaling pathway|multicellular o

ling|innate immune response|positive regulation of cell cycle|negative regulati

ty, RNA polymerase II-specific|fibrillar center|behavioral fear response|neural

toskeleton organization|cytoplasmic vesicle|negative regulation of protein auto

ar matrix|extracellular exosome|basement membrane organization

merase II distal enhancer sequence-specific DNA binding|DNA-binding transcripti
wth

dylcholine metabolic process|acylglycerol lipase activity|medium-chain fatty ac

Rho protein signal transduction|endosome to lysosome transport|regulation of ce

itination
euron differentiation|lysosomal lumen|anchored component of plasma membrane|col

dependent phosphate transmembrane transporter activity|membrane|phosphate ion t

rix organization|phosphatidylinositol-3-phosphate binding|phosphatidylinositol

er bond hydrolysis
ic cell cycle|regulation of centrosome duplication|microtubule cytoskeleton|cel

gulation of protein targeting to mitochondrion|regulation of reactive oxygen sp
process|gene silencing|nuclear body|ATPase activity|protein-containing complex

athway|anatomical structure morphogenesis|response to hormone|cell differentiat

ion, DNA-templated|histone deacetylation|nuclear speck|nuclear receptor binding
eptidase activity|protein deubiquitination|BMP signaling pathway|co-SMAD bindin

n repressor activity, RNA polymerase II-specific|fibrillar center|endothelial c
odimerization activity|establishment or maintenance of epithelial cell apical/b

on|integral component of membrane|transport vesicle|endoplasmic reticulum-Golgi
le membrane|neutrophil degranulation|tertiary granule membrane|ficolin-1-rich g

e II|microtubule cytoskeleton|positive regulation of gene expression, epigenetic

eratinocyte differentiation|ubiquitin protein ligase binding|response to estrad

e-specific DNA binding|skeletal system morphogenesis
on|beta-tubulin binding|spindle midzone|cell division|extracellular exosome|fic

s|Salivary secretion|Adrenergic signaling in cardiomyocytes|Aldosterone synthes

oblast migration|negative regulation of myotube differentiation|smooth muscle
sport|cellular iron ion homeostasis|brain development|aging|post-embryonic deve
nzyme binding

of peptidyl-threonine phosphorylation|negative regulation of phosphatase activi

inding|negative regulation of neuron differentiation|metal ion binding|neuron de

ndensation|chromatin DNA binding|nucleosomal DNA binding|negative regulation of
paired donors, with incorporation or reduction of molecular oxygen, NAD(P)H as

brane|ubiquitin conjugating enzyme binding|positive regulation of proteasomal u

necrotic cell death|pyroptosis|cellular response to tumor necrosis factor|cellu

l component of membrane|calcium-dependent cell-cell adhesion via plasma membrane

ess|cellular response to DNA damage stimulus|response to ionizing radiation|pro

|termination of RNA polymerase I transcription|transmembrane receptor protein ty
ation activity|positive regulation of transcription by RNA polymerase II|protein

g ATPase activity, phosphorylative mechanism|manganese-transporting ATPase activ

rinsic component of synaptic membrane|regulation of vascular smooth muscle cell

f T cell proliferation|defense response to protozoan|positive regulation of T ce
atin remodeling|cellular response to DNA damage stimulus|enzyme binding|protein

ase activity|neuronal cell body|dendritic spine|cadherin binding involved in cel

|azurophil granule lumen|regulation of growth|neutrophil degranulation|cofactor
acting on paired donors, with incorporation or reduction of molecular oxygen,

rin|cadherin binding|detection of temperature stimulus involved in sensory perce

el complex|calcium import into the mitochondrion|identical protein binding|prote

sis|clathrin binding|exocytic vesicle|cellular response to calcium ion

otubule plus-end|cell division|regulation of attachment of spindle microtubules
demethylase activity (H3-K27 specific)
of intrinsic apoptotic signaling pathway

onal protein modification

ibril organization|extracellular matrix|keratan sulfate catabolic process|lysos

nding|negative regulation of chromatin binding|snRNA modification|positive regu

lasmic reticulum exit site

of endothelial cell migration|negative regulation of angiogenesis|negative regu

isphosphate binding|actin filament binding|negative regulation of serine-type e

racellular exosome|negative regulation of NIK/NF-kappaB signaling

one H4-K5 acetylation|histone H4-K8 acetylation|histone H4-K12 acetylation|meta

export|ventricular cardiac muscle cell action potential|membrane repolarization
-dependent ATPase activity|zinc ion binding|membrane|axon|growth cone|ATP-depend

egulation of transcription by RNA polymerase II|metal ion binding

ulation of insulin secretion
iferation|positive regulation of mesenchymal stem cell differentiation

demethylase activity (H3-K27 specific)

|regulation of presynapse assembly
positive regulation of skeletal muscle cell proliferation|response to muscle ac

C-terminus binding|basal plasma membrane|membrane|lateral plasma membrane|uncor

tion by RNA polymerase II|cell motility|response to fibroblast growth factor

rane-bounded organelle|regulation of intracellular pH|recycling endosome|recycl

|diaphragm development|protein homotrimerization|peptide transmembrane transpor

ollagen-containing extracellular matrix
lin-glutamic acid ligase activity
mune response|cadherin binding involved in cell-cell adhesion|negative regulatio

nslational termination

lation of glucose metabolic process|positive regulation of MAPK cascade|protein
telelet alpha granule membrane|identical protein binding|post-translational prote[illegible]

ization|embryonic camera-type eye development|negative regulation of protein bi[illegible]

ding edge|regulation of protein localization|AP-2 adaptor complex binding|termi[illegible]

sel development|DNA binding|DNA-binding transcription factor activity|nucleus|r[illegible]

esis|transcription corepressor activity|nucleus|Notch signaling pathway|transcr[illegible]
sin binding|actin cytoskeleton organization|spectrin binding|filamentous actin|[illegible]

al enhancer sequence-specific DNA binding|DNA-binding transcription factor acti[illegible]

insulin secretion involved in cellular response to glucose stimulus|insulin bi[illegible]
n by RNA polymerase II|negative regulation of glucose import|negative regulatio[illegible]

ructural constituent conferring tensile strength|extracellular matrix organizat[illegible]

hromatin DNA binding|histone demethylase activity|sequence-specific DNA binding

s|synaptonemal complex assembly|spermatogenesis|brain development|proteasomal p[illegible]

dyl-serine phosphorylation|regulation of telomere maintenance|telomeric DNA bin[illegible]

ll differentiation|intracellular membrane-bounded organelle|metal ion binding

demethylase activity (H3-K27 specific)

ymporter activity|L-alanine transport|proline transport|tryptophan transport|in

binding|negative regulation of DNA damage response, signal transduction by p53
n activity|positive regulation of I-kappaB kinase/NF-kappaB signaling|innate im
FoxO signaling pathway|Thyroid hormone signaling pathway|Long-term potentiation

Carbon fixation pathways in prokaryotes|Valine, leucine and isoleucine degradat
ne metabolism|2-Oxocarboxylic acid metabolism|Biosynthesis of amino acids|Alanin
t alpha granule membrane|neurexin family protein binding|positive regulation of

er bond hydrolysis

tive regulation of transporter activity|multivesicular body membrane|positive re

cess|positive regulation of glomerular mesangial cell proliferation|positive re
sel morphogenesis|neural crest cell migration|DNA-binding transcription factor a

vesicle transport|inner ear development|protein-containing complex assembly|ex

al phosphate binding|intracellular membrane-bounded organelle|cadherin binding
kling behavior|heart morphogenesis|cardiac right ventricle formation|cardiac ne[illegible]

merization activity|dendritic spine|postsynaptic membrane|metal ion binding|per[illegible]

glutamatergic synapse

ulation of vesicle fusion|ruffle membrane|cellular response to insulin stimulus

ntegral component of membrane|lamellipodium|zinc ion transmembrane transport|zi[illegible]

r activity, RNA polymerase II-specific|DNA-binding transcription factor activit[illegible]

lation of transcription by RNA polymerase II|proximal/distal pattern formation|[illegible]
r polarity|symmetric cell division|negative regulation of protein localization [illegible]
demethylase activity (H3-K27 specific)

ection development|anchored component of membrane|negative regulation of neuron

RNA polymerase II|zinc ion binding|membrane|NuRD complex|protein-containing co[illegible]

deling|cell cycle|DNA-dependent ATPase activity|NuRD complex|NURF complex|chrom[illegible]
t contraction|muscle cell apoptotic process|regulation of myotube differentiati[illegible]
itive regulation of proteasomal ubiquitin-dependent protein catabolic process|p[illegible]
protein signal transduction|endothelial cell chemotaxis|positive regulation o[illegible]
NA polymerase II promoter|Wnt signaling pathway|protein ubiquitination|Cdc73/Pa[illegible]

positive regulation of cell migration|retrograde transport, endosome to Golgi|po

binding|MutSalpha complex binding|MutSbeta complex binding|DNA duplex unwinding

gulation of transcription by RNA polymerase II|positive regulation of phosphory

ption by RNA polymerase II|positive regulation of JAK-STAT cascade|positive regu

y RNA polymerase II|central nervous system development|maternal behavior|negati

duction|centrosome duplication|positive regulation of stress fiber assembly|mit

g|neuromuscular junction|protein-containing complex|sarcolemma|ion channel bind
response to starvation|vesicle membrane|membrane|apical plasma membrane|signal

d DNA binding|single-stranded DNA binding|RNA binding|nucleus|nucleolus|cytopla

geranylgeranylation|post-translational protein modification

rotein binding

y site|phosphatidylinositol-3,5-bisphosphate binding

rane|cellular response to steroid hormone stimulus|cellular response to hypoxia

ein autophosphorylation|focal adhesion assembly|regulation of focal adhesion as
 LSU-rRNA)|endonucleolytic cleavage in 5'-ETS of tricistronic rRNA transcript
orane|protein phosphorylation|cytoskeleton organization|regulation of neuron pr

npartment|Golgi apparatus|intracellular protein transport|ER to Golgi vesicle-m

|anchored component of plasma membrane

 as one donor, and incorporation of one atom of oxygen|actin filament depolymer

iption factor activity|response to lipopolysaccharide|negative regulation of in

tegral component of membrane|Wnt signaling pathway|neural plate anterior/poster
collagen-activated tyrosine kinase receptor signaling pathway|collagen-activate

sium channel regulator activity|membrane|protein domain specific binding|axon|e
neralization|positive regulation of bone mineralization|positive regulation of

xtracellular matrix|extracellular matrix binding|negative regulation of cell mo

ane|positive regulation of protein oligomerization|metal ion binding|head devel
in binding|ATPase activity, coupled to transmembrane movement of substances|TAP

cellular exosome

nt of membrane|extracellular matrix organization|extracellular matrix|axonogenes

ransporter activity|phosphatidylinositol transporter activity|protein transport
g|proximal promoter DNA-binding transcription activator activity, RNA polymerase

gnaling pathway|Focal adhesion|Prolactin signaling pathway
ty regulating pathway - multiple species

ess|protein secretion|gene expression|regulation of mitochondrion organization|

of cell growth|growth cone|intracellular signal transduction|positive regulatio

ceptor signaling pathway|somatic stem cell population maintenance|skeletal muscl

of cell proliferation|regulation of cell shape|positive regulation of epithelia

n repressor activity, RNA polymerase II-specific|RNA polymerase II transcription
signaling pathway|Cell cycle - yeast

|heparin binding|regulation of Notch signaling pathway|response to mechanical s

e biosynthesis|Oxytocin signaling pathway|cGMP-PKG signaling pathway
ion|DNA-binding transcription factor activity|ATP binding|nucleus|nucleoplasm|nu
lation of plasminogen activation|negative regulation of sodium ion transport|ne

sensory perception|detection of mechanical stimulus|cellular response to mechan

c forelimb morphogenesis|embryonic hindlimb morphogenesis|negative regulation o[illegible]

of bicellular tight junction assembly
ependent DNA replication initiation|replisome|DNA replication, Okazaki fragment

ation from RNA polymerase I promoter|nucleolus organization|cytoplasm organizat[illegible]

ranule lumen|interleukin-12-mediated signaling pathway|neutrophil degranulation

ia plasma membrane cell adhesion molecules|catenin complex|adherens junction or[illegible]

RNA polymerase II-specific|DNA binding|DNA-binding transcription factor activ[illegible]

ding

cretion|Circadian entrainment|Wnt signaling pathway|cAMP signaling pathway|Choli

|tooth eruption|calcium ion homeostasis|extracellular exosome|enamel mineraliza[illegible]

ion of cell shape|lamellipodium|actin cytoskeleton organization|filopodium|smal[illegible]

[illegible]al adhesion

[illegible]r bond hydrolysis

[illegible]f neuron projection development|protein ubiquitination|UBC13-MMS2 complex|posi[illegible]
[illegible]|dendritic shaft|metal ion binding|presynapse|postsynapse|dendritic microtubul[illegible]
[illegible] pathway|Oocyte meiosis|Calcium signaling pathway|VEGF signaling pathway|cGMP-[illegible]

[illegible]|dendrite|dense core granule|cytoplasmic vesicle|intracellular membrane-bounde[illegible]
[illegible]g pathway - multiple species

[illegible]rane-bounded organelle|histone mRNA catabolic process
[illegible]ctivity|response to wounding|cell surface|positive regulation of vascular endo[illegible]

[illegible]ptotic process|Rac GTPase binding|negative regulation of axonogenesis|positive

[illegible]ctin filament capping

[illegible]d binding|perinuclear region of cytoplasm

ty|extrinsic component of cytoplasmic side of plasma membrane|regulation of Rho

AMPK signaling pathway|T cell receptor signaling pathway|NF-kappa B signaling p

phate binding|phosphatidic acid binding|phosphatidylinositol-3,5-bisphosphate b
of transcription by RNA polymerase III|myelination|identical protein binding|re

inment|Oxytocin signaling pathway|Glutamatergic synapse

lex
ocyte maturation|Regulation of lipolysis in adipocytes|Melanogenesis|Dopaminergi

ar response to heat|negative regulation of apoptotic process|regulation of DNA-

lfate metabolic process

signaling in cardiomyocytes|Aldosterone synthesis and secretion|Insulin secretio

l constituent conferring compression resistance|transport vesicle|extracellular

transport|endocytosis|sensory perception of sound|membrane|unconventional myos

en|aging|growth factor activity|metalloendopeptidase inhibitor activity|zinc io
n|establishment of Golgi localization|maintenance of Golgi location|organelle t

nponent of membrane|sodium channel regulator activity|intermediate filament bin

tract morphogenesis|anterior commissure morphogenesis|cell junction|presynapti

idation

ted vesicle|protein binding, bridging|HOPS complex|late endosome membrane|CORVE
of JNK cascade|metal ion binding|perinuclear region of cytoplasm|protein autoub

riolar satellite|dynactin binding|microtubule anchoring at centrosome|identical

NA polymerase II|positive regulation of neuron projection development|regulatio

ar body|cell differentiation|lung development|protein heterodimerization activi
negative regulation of osteoblast differentiation|negative regulation of transc

ynthetic process|chondroitin sulfate catabolic process|dermatan sulfate biosynt

or activity|steroid hormone receptor activity|transcription coactivator activit

|centriole replication|brain development|microtubule binding|regulation of G2/M

development|membrane|Wnt signaling pathway|lateral plasma membrane|peptidyl-ser
l granule membrane|specific granule membrane|protein localization to endosome|n

ation|cell differentiation|protein tyrosine kinase collagen receptor activity|c

terferon-alpha production|negative regulation of interferon-beta production|neg

DNA-templated
se response to bacterium|neuronal cell body|neutrophil degranulation|positive r

regulation of cAMP-mediated signaling|positive regulation of protein kinase B s
osome

ne and other factor-regulated calcium reabsorption|Insulin secretion|Thyroid h

actin|chondroitin sulfate binding|cell body|synapse|neuron development|response

tion of synaptic vesicle exocytosis

id-mediated signaling|positive regulation of protein kinase B signaling|positiv

biosynthetic process|sex differentiation|transcription factor binding|positive

|DNA-binding transcription repressor activity, RNA polymerase II-specific|DNA-b

to tumor necrosis factor

italia development|protein-containing complex|retinol transmembrane transporter

athway|Regulation of actin cytoskeleton|VEGF signaling pathway|Fc gamma R-mediat

|oxidation-reduction process|collagen-containing extracellular matrix|extracell
, bending|anatomical structure morphogenesis|regulation of Wnt signaling pathwa

ocyte migration

hway|metal ion binding

ing endosome membrane|potassium ion transmembrane transport|sodium ion import a

‹inase signaling pathway|cell proliferation|negative regulation of signal trans‹

|nucleoplasm|positive regulation of transcription by RNA polymerase II|developm‹

ı ligase complex
 regulation of cell-cell adhesion mediated by integrin|regulation of membrane p‹

of transcription, DNA-templated|positive regulation of transcription, DNA-templa‹

olood pressure|G protein-coupled peptide receptor activity|natriuretic peptide ‹

ıs|type I interferon signaling pathway

gulation of small GTPase mediated signal transduction|retina vasculature morpho‹

tive regulation by host of viral exo-alpha-sialidase activity|negative regulati‹

g transcription activator activity, RNA polymerase II-specific|proximal promote‹
ein-containing complex|nuclear periphery
otein localization|axon cytoplasm|proximal neuron projection
stsynapse|structural constituent of postsynaptic actin cytoskeleton|structural ‹
apse|glutamatergic synapse|dense core granule exocytosis

ivity|intracellular signal transduction

ascular smooth muscle contraction|Calcium signaling pathway|GnRH signaling pat
lation|central nervous system projection neuron axonogenesis|forebrain develop
roteasomal protein catabolic process

ptor signaling pathway|postsynaptic density|integral component of membrane|lat

transcription initiation from RNA polymerase II promoter|SUMO ligase activity|

lity control for misfolded or incompletely synthesized proteins|mitochondrion

ling pathway|intrinsic component of plasma membrane|peptidyl-tyrosine dephospho

inding|RNA binding|nucleus|nucleoplasm|nucleolus|cytosol|autophagy|inflammator

ive regulation of synaptic transmission|positive regulation of synapse assembly

ic synapse|Adrenergic signaling in cardiomyocytes|Circadian entrainment|Glutamat

ay|negative regulation of epidermal growth factor receptor signaling pathway|ne

e activity

|histone binding|transcription regulatory region DNA binding|negative regulatio

ositive regulation of transcription, DNA-templated|metal ion binding|androgen r

deacetylase activity (H3-K14 specific)|negative regulation of interleukin-2 pr
hape|Rac protein signal transduction|cytokine-mediated signaling pathway|protei

n|peptidyl-threonine phosphorylation|extrinsic component of membrane|protein ki
lar response to interferon-gamma|cellular response to interleukin-1|cellular re

ton organization|response to lipopolysaccharide|regulation of Rho protein signa

ng|regulation of small GTPase mediated signal transduction|cell chemotaxis|acti

maintenance of mitotic sister chromatid cohesion|interchromatin granule|site o

in targeting to lysosome|lipid transport|post-Golgi vesicle-mediated transport|
permatogenesis|G protein-coupled peptide receptor activity|hormone-mediated sig

RNA polymerase II promoter|zinc ion binding|PML body|signaling receptor activit

e lumen|neutrophil degranulation|cell redox homeostasis|extracellular exosome

|recycling endosome

|spine apparatus|Schaffer collateral - CA1 synapse|modification of dendritic sp

ose metabolism|Streptomycin biosynthesis

of membrane|antigen processing and presentation of endogenous peptide antigen v

embly|viral process|nucleosomal DNA binding|nuclear pericentric heterochromatin

ne potential|neuron projection|synapse|nervous system process

tion of apoptotic process|positive regulation of apoptotic process|death domain

intenance|identical protein binding|protein homodimerization activity|negative

n of peptidyl-threonine phosphorylation|enzyme binding|CCR4-NOT complex|positiv

nding|intracellular membrane-bounded organelle|establishment of protein localiz[illegible]

or signaling pathway|negative regulation of locomotion involved in locomotory b[illegible]

-containing complex|cadherin binding

ation|Chloroalkane and chloroalkene degradation|Ascorbate and aldarate metaboli[illegible]

llular vesicle|protein binding, bridging involved in substrate recognition for [illegible]

n factor binding|nucleus|transcription factor complex|gastrulation|neural crest

sis and secretion|Insulin secretion|Thyroid hormone synthesis|Circadian entrain[illegible]

ns junction|zonula adherens|fascia adherens|focal adhesion|cytoskeletal anchori[illegible]

sphosphate binding|basement membrane|vesicle budding from membrane|cytoskeletal

and incorporation of one atom of oxygen|secretory granule membrane|dopamine cat
em development|nuclear speck|negative regulation of DNA-binding transcription f
Adrenergic signaling in cardiomyocytes|Aldosterone synthesis and secretion|Insul
e regulation of neurogenesis|extracellular exosome

ansport|protein ubiquitination|GDP binding|identical protein binding|innate imm

ion of neuron apoptotic process|negative regulation of neuron apoptotic process
thway|positive regulation of collateral sprouting|extracellular exosome

regulation of vitamin D receptor signaling pathway|kidney epithelium developmen

mic reticulum tubular network membrane|endoplasmic reticulum tubular network me

t of protein localization to membrane|cytoskeletal anchoring at nuclear membran

orane|regulation of neuronal synaptic plasticity|calcium-dependent protein bind
n|wound healing|cell projection|keratin filament|intermediate filament cytoskel

nt signaling pathway, calcium modulating pathway|protein C-terminus binding|pos

achidonic acid metabolism|GnRH signaling pathway|VEGF signaling pathway|Platelet

to hypoxia|glutamatergic synapse|GABA-ergic synapse
ric bud development|branching involved in ureteric bud morphogenesis|morphogene

egulator activity|protein kinase binding|positive regulation of cell cycle

t cell|negative regulation of neurogenesis|defense response to virus|ubiquitin [illegible]

icle|protein dimerization activity

|WW domain binding|negative regulation of pathway-restricted SMAD protein phosp[illegible]

assembly|establishment of centrosome localization

on of Rho protein signal transduction|regulation of GTPase activity|filopodium [illegible]
binding|protein kinase A signaling|extracellular matrix constituent conferring [illegible]

ɔse|Cholinergic synapse|Oocyte meiosis|Retrograde endocannabinoid signaling|Vas[illegible]

ay
K4 specific)|histone demethylase activity (H3-trimethyl-K4 specific)|histone H3-

sitive regulation of cell-cell adhesion mediated by cadherin
keletal muscle thin filament assembly|skeletal muscle myosin thick filament ass[illegible]

ific|proximal promoter DNA-binding transcription activator activity, RNA polyme[illegible]
xtracellular matrix|cell-cell adhesion

on
bounded organelle|negative regulation of viral genome replication|negative regu[illegible]

radial glia guided migration|positive regulation of cell migration|nuclear mig[illegible]

ing|regulation of cell migration|midbody|small GTPase binding|early endosome me[illegible]

wth rate|response to exogenous dsRNA|membrane raft|T cell receptor signaling pa[illegible]

rase II|methylcytosine dioxygenase activity|DNA demethylation|histone H3-K4 tri[illegible]

eavage furrow|signaling receptor activity|protein homodimerization activity|cel

nic vesicle membrane|glucose homeostasis|positive regulation of I-kappaB kinase,
|regulation of autophagosome size|ATPase activity|cation-transporting ATPase ac

pathway|chemical synaptic transmission|aging|response to radiation|glial cell

rior pattern specification|enzyme binding|negative regulation of smoothened sig

ated|transforming growth factor beta receptor signaling pathway|zinc ion bindin

ody|dendritic spine|membrane raft|regulation of neuronal synaptic plasticity|mR

lation of cerebellar granule cell precursor proliferation|prosaposin receptor a

identical protein binding|regulation of catalytic activity|protein localization

of cell motility

kinje cell synapse|anchored component of presynaptic membrane

n organization|trans-Golgi network membrane|retrograde transport, endosome to G
involved in cilium assembly|organelle transport along microtubule|ciliary tip
ne|cell projection|perikaryon|neutrophil degranulation|D-glucose transmembrane

regulation of cell shape|semaphorin receptor activity|protein domain specific b

mediator|positive regulation of apoptotic process|phosphatidylinositol-3,4-bisp
tion of endothelial cell migration|protein transport|vesicle-mediated transport

lament binding|collagen-containing extracellular matrix|negative regulation of
|methylarsonite methyltransferase activity|methylation|DNA methylation on adeni

s|zinc ion binding|nuclear speck|protein kinase binding|protein-containing comp

erentiation|neuron differentiation|positive regulation of cell growth|axon|skel

s|negative regulation of gene expression|response to organic cyclic compound|mer

l division

way|Cell cycle - yeast|MAPK signaling pathway - fly|Meiosis - yeast

n binding|Rab protein signal transduction|melanosome membrane|endosome to melan

abolic process|regulation of protein localization|protein-containing complex|re
-threonine phosphorylation|carbohydrate binding|lung development|extracellular r

f postsynaptic recycling endosome membrane|regulation of postsynapse organizati

lin receptor signaling pathway|animal organ morphogenesis|SWI/SNF complex|prosta

ivity|positive regulation of SMAD protein signal transduction

ivity, RNA polymerase II-specific|DNA binding|DNA-binding transcription factor a
ifferentiation|DNA binding|double-stranded DNA binding|transcription corepresso
ivity, RNA polymerase II-specific|angiogenesis|negative regulation of cell-matr

or activity|single-stranded RNA binding|nucleus|brain development|proximal/dista

e docking

anslational protein modification|perinuclear region of cytoplasm|extracellular

nerization activity|neutrophil degranulation|protein autophosphorylation|extrac

ort|centrosome localization|dynein light intermediate chain binding|regulation

terferon-alpha production|negative regulation of interferon-beta production|neg

lular exosome

yl-tyrosine dephosphorylation|insulin secretion involved in cellular response t

histone binding|histone methyltransferase complex|metal ion binding|histone de
n apoptotic process

on mediated by integrin|negative regulation of protein import into nucleus|kera
by RNA polymerase II|positive regulation of transcription by RNA polymerase II

-mediated transport|protein secretion|membrane|COPI vesicle coat|transport vesi

itive regulation of protein tyrosine kinase activity|molecular function regulat

tyltransferase activity|mitotic sister chromatid cohesion, centromeric

development|regulation of extrinsic apoptotic signaling pathway via death doma
ve regulation of transcription, DNA-templated|positive regulation of transcript
crest cell migration|somitogenesis|neural tube closure|nucleus|nucleolus|cytoso

racellular vesicle

age response, signal transduction by p53 class mediator resulting in transcript
ility|synaptic vesicle membrane|mitochondrial membrane|ion transmembrane transp

n development
on of apoptotic process|ADP binding|Golgi vesicle budding|positive regulation o

collagen-containing extracellular matrix|negative regulation of cold-induced th

n of transcription, DNA-templated|segment specification|sensory organ developmen
NA polymerase II|cell differentiation|sequence-specific DNA binding

l differentiation|negative regulation of transcription, DNA-templated|positive r

ranscription by RNA polymerase II|asymmetric neuroblast division|positive regula

nal transduction|regulation of cell proliferation|metal ion binding|regulation o

nsporter activity|L-glutamine transmembrane transporter activity|L-proline trans

y|nucleus|nucleoplasm|mitochondrion|regulation of transcription by RNA polymeras

gulation of transcription, DNA-templated
tion|ribosome binding|positive regulation of neuron differentiation|negative reg

ation activity|plasma membrane raft|protein heterodimerization activity|leukocy

complex|protein refolding|ATPase activity, coupled|intracellular membrane-boun[illegible]

aining extracellular matrix

viral process|cilium assembly

activity
ntracellular membrane-bounded organelle|neutrophil degranulation|post-translati[illegible]

ion binding|actin filament binding|recycling endosome|bicellular tight junction

and secretion|Insulin secretion|Thyroid hormone synthesis|Circadian entrainment
apoptotic signaling pathway in absence of ligand|mitochondrial outer membrane p[illegible]

of I-kappaB kinase/NF-kappaB signaling|negative regulation of cysteine-type end

anscribed mRNA catabolic process, deadenylation-dependent decay|negative regula

n|RNA binding|nucleus|nucleolus|endoplasmic reticulum|mitotic metaphase plate c

nding

e|negative regulation of peptidyl-serine phosphorylation|phosphatidylinositol p

in secretion|positive regulation of embryonic development|negative regulation o

ssor activity, RNA polymerase II-specific|RNA polymerase II repressing transcri

nosphate diphosphatase activity|inositol bisdiphosphate tetrakisphosphate dipho

II|negative regulation of cell proliferation|negative regulation of erythrocyt

|postsynaptic membrane|ciliary base|non-motile cilium assembly
ose metabolism|Streptomycin biosynthesis

tivity|orcinol O-methyltransferase activity
transcription factor activity|nucleus|nucleoplasm|DNA replication|regulation of

regulation of fatty acid biosynthetic process|transmembrane transport
oligomerization|cullin family protein binding

ctivity, RNA polymerase II-specific|DNA-binding transcription repressor activit

tic membrane|phosphatidic acid binding|glutamatergic synapse|postsynaptic modul

rdiac right ventricle morphogenesis|ventricular trabecula myocardium morphogene

rotein metabolic process

naling pathway

thway

lation|positive regulation of ERK1 and ERK2 cascade|positive regulation of sprou

naptic vesicle exocytosis

l migration|anchored component of membrane|embryonic forelimb morphogenesis|regu

itin protein ligase activity|mitotic spindle|negative regulation of sodium ion

terferon-alpha production|negative regulation of interferon-beta production|neg
actor activity, RNA polymerase II distal enhancer sequence-specific binding|nuc

of synapse assembly|excitatory synapse|synaptic vesicle clustering|presynapse

rin-mediated signaling pathway

lar organism development|metal ion binding|cellular response to peptide

activity|nucleus|nervous system development|axon guidance|cerebellum developmen

se|regulation of nucleocytoplasmic transport|mRNA transport|defense response to

xon|cell-cell adhesion via plasma-membrane adhesion molecules

uron migration|osteoblast development|chromatin binding|nucleus|nucleoplasm|tra

eralization|heat shock protein binding|histone H2A monoubiquitination|odontogen

ent|nuclear matrix|protein domain specific binding|cell leading edge|positive r
A binding|nucleus|cytoplasm|cytosol|cytoskeleton|plasma membrane|regulation of

fibril|Z disc|ankyrin binding|M band|titin binding|regulation of Rho protein si

cell-cell adhesion|ficolin-1-rich granule membrane

tin filament bundle assembly|cell division|positive regulation of protein tyros
al actin cytoskeleton|neuron projection development|neuromuscular junction|prot

ng in cardiomyocytes|Aldosterone synthesis and secretion|Insulin secretion|Thyr

n

n binding|negative regulation of neuron differentiation|protein heterodimerizat

cell cycle

flow tract septum morphogenesis|right ventricular cardiac muscle tissue morphog

ein binding|endoplasmic reticulum calcium ion homeostasis|negative regulation o

ssel endothelial cell proliferation involved in sprouting angiogenesis

ulation of transcription by RNA polymerase II|protein demethylation|blood coagu

gulation of satellite cell differentiation
ase activity|metallocarboxypeptidase activity|extracellular matrix structural c

ive regulation of cholesterol efflux|viral process|transcriptional repressor co[illegible]

[illegible]uron projection|synapse|chemorepellent activity|positive regulation of synapse [illegible]

ing transcription factor activity|steroid hormone receptor activity|transcripti[illegible]
[illegible]proliferation|microtubule cytoskeleton|establishment of cell polarity|dendrite|[illegible]

ty|proteasome binding

nase activity|negative regulation of transcription, DNA-templated|7SK snRNA bin

trate adhesion|negative regulation of endopeptidase activity|negative regulatio

n kinase activity

ransport|modulation of chemical synaptic transmission

otein modification

ion|phosphatidylinositol-3-phosphate binding|phosphatidylinositol binding|posit

en|collagen-containing extracellular matrix|extracellular exosome|extracellular

pid transfer|intermembrane lipid transfer activity|negative regulation of NLRP3

ve regulation of angiogenesis|negative regulation of blood pressure

sicle|nuclear membrane|T-tubule organization|cellular response to heat|intracel

ansport vesicle|cell-cell contact zone|protein homooligomerization|ventricular

granule membrane
ent

e dimethylation|histone methyltransferase activity (H3-K4 specific)|positive re

rcoplasmic reticulum|regulation of cardiac muscle contraction by regulation of [illegible]

[illegible]|dendrite|brush border membrane|potassium channel complex|response to nicotine

constituent conferring elasticity|negative regulation of transforming growth f[illegible]

itochondrial outer membrane permeabilization involved in apoptotic signaling pa[illegible]

[illegible]g receptor binding|endoplasmic reticulum|Golgi apparatus|plasma membrane|integ[illegible]

ivity|nucleus|nucleoplasm|chromosome|cytosol|regulation of transcription, DNA-t[illegible]

[illegible]early endosome membrane|late endosome membrane|ion transmembrane transport

ganization|maintenance of protease location in mast cell secretory granule|main

cription by RNA polymerase II|metal ion binding|positive regulation of dendrite

ion of synaptic transmission|presynaptic active zone cytoplasmic component|regu
growth factor receptor signaling pathway|cell junction|protein binding, bridgi
orane|response to type I interferon|identical protein binding|negative regulati

ctivator activity|nucleus|nucleoplasm|nucleosome disassembly|chromatin remodeli

homodimerization activity|neuron projection|protein heterodimerization activit

sicle|pigmentation|Golgi localization

orane|actin cytoskeleton organization|cell junction|regulation of Rho protein s

ctivity|ubiquitin binding|protein K48-linked deubiquitination

ressor activity|nucleus|nucleoplasm|transcription factor complex|cytosol|regula

A polymerase II-specific|DNA-binding transcription factor activity|nucleoplasm|

cal adhesion|Wnt signaling pathway|Circadian rhythm - fly|Insulin signaling path
transmembrane transporter activity|arginine transmembrane transporter activity|
ellular matrix organization|collagen fibril organization|extracellular matrix|p

n binding|cytokine binding|PDZ domain binding|neuron differentiation|dendrite|c

gulation of transcription by RNA polymerase II|aggresome|metal ion binding

o forskolin

egative regulation of interleukin-10 biosynthetic process|negative regulation o
onogenesis|visual perception|dorsal/ventral axis specification|Wnt signaling pa

egulation of mRNA processing

creatic cell proliferation|RNA transport|nucleic acid transmembrane transporter

axin binding|Golgi cisterna membrane|protein-containing complex binding|protein

g|cell migration involved in gastrulation|cell-cell adhesion involved in gastru

mediate compartment membrane|intracellular membrane-bounded organelle

erleukin-6 secretion

factor activity|nucleus|nucleoplasm|regulation of transcription, DNA-templated|

negative regulation of interferon-gamma production|negative regulation of inter
migration|microvillus membrane|regulation of microvillus assembly|positive regu

protein stabilization|cornification
ter activity|regulation of membrane potential

endent protein kinase complex|intracellular membrane-bounded organelle|protein

membrane transport|potassium ion transmembrane transport

to glucose stimulus|cellular response to hypoxia|presynapse|postsynapse|neurot

otic spindle|negative regulation of high voltage-gated calcium channel activity

synaptic membrane|cadherin binding|regulation of neuronal synaptic plasticity|r

ulation of TRP channels

ring to Golgi
ative regulation of cell migration|polarized epithelial cell differentiation|mi

tion|posttranscriptional regulation of gene expression|nuclear body|cellular pr

n, DNA-templated|negative regulation of cell fate commitment|positive regulatio
transcription initiation from RNA polymerase II promoter|SUMO ligase activity|

tifier potassium channel activity
complex|vesicle tethering to Golgi|embryonic brain development
n|rhythmic process

tallopeptidase activity|zinc ion binding|extracellular matrix disassembly|cell
on|positive regulation of bone development

ium disassembly

regulation of cytokinesis|positive regulation of GTPase activity|cadherin bindi[illegible]
[illegible]erase activity|stress-activated protein kinase signaling cascade|histone bindin[illegible]
ive regulation of B cell receptor signaling pathway|protein-containing complex [illegible]

tion, elongation|enhancer binding|snRNA transcription by RNA polymerase II|posi[illegible]

[illegible] acting on single donors with incorporation of molecular oxygen, incorporation

[illegible]ction|bicellular tight junction assembly

frequency involved in ciliary motility

on of bone remodeling

g transcription activator activity, RNA polymerase II-specific|kidney developmen
eoglycan catabolic process|extracellular matrix|intrinsic component of plasma me

SCF ubiquitin ligase complex|protein phosphatase binding|SCF-dependent proteasom

phogenesis|positive regulation of axon extension involved in axon guidance|epibo
l ventricular junction remodeling|integral component of plasma membrane|gap junc

transition involved in metanephros morphogenesis|cytokine activity|extracellular

amin D|negative regulation of multicellular organism growth|protein homodimeriza

ve regulation of BMP signaling pathway|negative regulation of BMP signaling path

transport|integral component of membrane|L-histidine transmembrane transport|L-a

organism development|spermatogenesis|single fertilization|male gonad developmen

transduction by protein phosphorylation|negative regulation of cell migration|

ng|positive regulation of transcription by RNA polymerase II|metal ion binding|

signaling pathway|T cell receptor signaling pathway|Focal adhesion

or activity|nucleus|plasma membrane|negative regulation of epidermal growth fac

n factor activity|transcription factor activity, RNA polymerase II distal enhan

nal transduction

regulation of mRNA splicing, via spliceosome|perinuclear region of cytoplasm|p

ex|phosphatidylinositol binding|apical junction complex|establishment or mainte

ive regulation by symbiont of host apoptotic process|protection from natural ki

athway|negative regulation of apoptotic process|negative regulation of smoothen

omponent of membrane|SH3 domain binding|extracellular matrix disassembly|extrac

calization to nucleus

signal transduction by protein phosphorylation|cell junction|regulation of cell

esis|cAMP signaling pathway|Cholinergic synapse|AMPK signaling pathway|cGMP-PKG
s

eptor cell development

PKG signaling pathway
binding|nuclear pore central transport channel|mRNA transport|protein localizat
complex|positive regulation of JUN kinase activity|positive regulation of angio

chondrial outer membrane permeabilization involved in apoptotic signaling pathwa

defense response to fungus|defense response to virus|positive regulation of mac

binding|LIM domain binding|regulation of cell migration|cell leading edge|somat

ellular cholesterol transport|sterol binding|intracellular membrane-bounded orga

etabolism|Arginine biosynthesis|Tropane, piperidine and pyridine alkaloid biosyn

ivity|transcription corepressor activity|nucleus|nucleoplasm|cytosol|regulation

stone modification|midbody|spindle pole centrosome|chromosome passenger complex

activity, acting on paired donors, with incorporation or reduction of molecular

ve regulation of MAPK cascade|positive regulation of transcription by RNA polym

ic vesicle|neuron projection|neuronal cell body|myelin sheath|plasma membrane ra

igration|membrane|integral component of membrane|viral process|semaphorin recep

l ventricular junction remodeling|integral component of plasma membrane|gap junc

trimethylation|positive regulation of ATP metabolic process

A-binding transcription activator activity, RNA polymerase II-specific|proximal

ding|protein localization to cell surface|thiol-dependent ubiquitinyl hydrolase

t-translational protein modification|cellular protein metabolic process|extrace

s factor|cellular response to BMP stimulus

n cilium assembly|ciliary basal body|regulation of apoptotic process|dendrite t

ral killer cell mediated cytotoxicity|Estrogen signaling pathway|Melanogenesis|
st|negative regulation of helicase activity|defense response to virus|type I in
rase II transcription|positive regulation of transcription by RNA polymerase II
rane transport
ion|response to virus|cell surface|membrane|apical plasma membrane|negative reg

epidermis|keratinization|intracellular membrane-bounded organelle|neutrophil de

d pit|adherens junction|endocytosis|cell surface receptor signaling pathway|sma

|transition metal ion binding|perinuclear region of cytoplasm|cellular response
binding

e|cell cortex|fatty acid metabolic process|neutral lipid metabolic process|phos

ule organization|gamma-tubulin binding|meiotic cell cycle|microtubule nucleatio
lization|extracellular exosome|14-3-3 protein binding|positive regulation of in

cell receptor signaling pathway|Oxytocin signaling pathway

complex|cadherin binding|extracellular exosome

on|glucose homeostasis|identical protein binding|protein complex oligomerizatio

ding|anterograde axonal transport|ATP-dependent microtubule motor activity, plu

oclast maturation|regulation of fibroblast growth factor receptor signaling pat

|intracellular membrane-bounded organelle|membrane raft|positive regulation of

utoubiquitination|ubiquitin protein ligase activity

rase II promoter in response to stress|ribosome binding|cadherin binding|cellul

scriptional repressor complex|IRE1-mediated unfolded protein response|collagen-

g|intracellular membrane-bounded organelle|COPII vesicle coating|negative regula

axis|osteoclast differentiation|leukocyte chemotaxis|positive regulation of B c

ation|positive regulation of transcription by RNA polymerase II|metal ion bindi

on of telomere maintenance via telomerase|positive regulation of telomere maint

duction|cell surface|astrocyte development|Wnt-protein binding|cell differentia

xtracellular exosome|supramolecular fiber organization

tor

e regulation of mitotic cell cycle|negative regulation of membrane protein ecto

n|actin filament polymerization|actin filament depolymerization|filopodium|posi

t of membrane|negative regulation of angiogenesis|calcineurin-NFAT signaling ca

rotein ubiquitination|SCF ubiquitin ligase complex|SCF-dependent proteasomal ub
nulticellular organismal homeostasis

ulation of I-kappaB kinase/NF-kappaB signaling|cadherin binding|extracellular e

nbrane|mitochondrial inner membrane|mitochondrial intermembrane space|cytosol|c

regulation of microvillus assembly|ion transmembrane transport|phospholipid tra
lation of interleukin-5 production|interleukin-33-mediated signaling pathway|po

ite|dense core granule|cellular response to drug|cellular response to potassium

ecycling endosome

|Apoptosis|RIG-I-like receptor signaling pathway|B cell receptor signaling pat

itotic cell cycle

localization

ynthetic process|chondroitin sulfate catabolic process|dermatan sulfate biosynt

rotein binding|extracellular exosome|negative regulation of IRE1-mediated unfol

ane transporter activity|regulation of appetite|histamine uptake

nbrane|membrane|extrinsic component of plasma membrane|apoptotic cell clearance

e regulation of cell-cell adhesion mediated by integrin|isotype switching|cadhe

ication|cardiac muscle cell-cardiac muscle cell adhesion|bundle of His cell-Purl

ng|collagen-containing extracellular matrix|extracellular exosome|basement memb
cushion development|cardiac left ventricle formation|ventricular septum develop

ng pathway|Signaling pathways regulating pluripotency of stem cells|Progesterone

nponent of membrane|sodium channel regulator activity|intermediate filament bin

of neuron differentiation|canonical Wnt signaling pathway|regulation of microt
stimulus|negative regulation of protein binding|regulation of growth|identical
l-cell adhesion

gative regulation of substrate adhesion-dependent cell spreading|negative regul

body|cleavage furrow|regulation of cytokinesis|intercellular bridge|midbody abs

regulation of canonical Wnt signaling pathway

rin binding|intracellular membrane-bounded organelle|exocytic vesicle|cellular [illegible]

ing transcription repressor activity, RNA polymerase II-specific|nucleus|nucleo[illegible]

nylate cyclase activity
p|ciliary base|intraciliary transport particle B binding|manchette assembly

charide biosynthetic process|hyaluronan synthase activity|extracellular matrix [illegible]

tion

n|protein heterodimerization activity|camera-type eye morphogenesis|neuron deve[illegible]

transforming growth factor beta receptor signaling pathway|cytoplasmic vesicle

asmic side of the plasma membrane|cytoplasmic vesicle|filopodium membrane|D3 do[illegible]

|multicellular organism development|blood circulation|anterior/posterior patter[illegible]

[illegible]ody

[illegible] kinase binding|syntaxin binding|COPII-coated ER to Golgi transport vesicle|ne[illegible]
rich granule lumen

[illegible]genitor cell differentiation|DNA-binding transcription factor activity|nucleus|[illegible]

[illegible]gulation of interleukin-17 production|negative regulation of tumor necrosis fac[illegible]

[illegible]tivity|nucleus|nucleoplasm|nuclear euchromatin|nuclear heterochromatin|DNA repa[illegible]

[illegible]|positive regulation of cell migration|protein-containing complex|negative reg[illegible]

[illegible]-binding transcription factor activity|transcription coregulator activity|tran[illegible]

ding|TOR signaling|TORC1 signaling|positive regulation of viral genome replicat
nbrane transporter activity|transmembrane transport

sorting pathway|early endosome to late endosome transport|cilium assembly|posi

tion

rase complex|mitochondrial nucleoid|sequence-specific DNA binding
n|mRNA 3'-UTR AU-rich region binding|regulation of mRNA stability|positive regu

7-DLG1-LIN7 complex|presynapse|glutamatergic synapse|protein localization to ba

cell body|cell body|positive regulation of viral genome replication|positive re

rotein-containing complex|histone deacetylase binding

n processing and presentation|protein domain specific binding|secretory granule

ılation of cell migration|clathrin-coated vesicle membrane|clathrin heavy chain

otic spindle organization|tubulin binding|kinase regulator activity|nuclear cyc
PKG signaling pathway|Long-term potentiation|B cell receptor signaling pathway|W

itin ligase complex|protein kinase binding|ubiquitin protein ligase binding|reg

e-stranded DNA binding|double-stranded telomeric DNA binding|single-stranded DN

f cells|parental behavior

ansport|regulation of resting membrane potential|cardiac conduction|potassium i
nbly|membrane organization|clathrin-dependent endocytosis|energy homeostasis|pl
ing|DNA-binding transcription factor activity|transcription factor activity, RN

vay|Cell cycle - yeast|Meiosis - yeast

response|positive regulation of interleukin-1 beta secretion

species|mTOR signaling pathway

esponse to DNA damage by p53 class mediator|positive regulation of apoptotic pr

system development|heart development|lactation|zinc ion binding|response to pH

sis|skeletal system morphogenesis

lasm|blood coagulation|adult behavior|regulation of cellular pH|regulation of c

n ubiquitination|histone arginine methylation|protein-arginine omega-N asymmetr

lex binding|sarcomere organization|cell differentiation involved in embryonic p

ation|heterophilic cell-cell adhesion via plasma membrane cell adhesion molecul
rane|anchored component of membrane|receptor complex|extracellular exosome

ion of cell migration|nuclear membrane|activation of protein kinase B activity|
t|ATPase activity|axon|dendrite|cytoskeleton-dependent intracellular transport|

itive regulation of type I interferon production|positive regulation of interle

canonical Wnt signaling pathway|beta-catenin-TCF complex assembly

nent|positive regulation of myotome development

ed component of postsynaptic density membrane

|neutrophil degranulation|cytolytic granule|phagocytic vesicle|extracellular ex

lex

rganism development|iodide transmembrane transporter activity|iodide transport|

on of smooth muscle cell differentiation|positive regulation of G1/S transition

tube closure|DNA-binding transcription factor activity|extracellular region|nu

phosphorylation|regulation of protein localization|metal ion binding|positive r

on factor activity, RNA polymerase II-specific|proximal promoter sequence-speci

id biosynthetic process|medium-chain fatty acid catabolic process|phosphatidyls

ll shape|endosome membrane|positive regulation of endothelial cell migration|me

lagen-containing extracellular matrix

ransmembrane transport|signaling receptor activity|extracellular exosome

binding|cell projection|phosphatidylinositol-3,4-bisphosphate binding|phosphati

l differentiation|centriolar satellite|intraciliary transport involved in ciliu

ecies metabolic process
|histone binding|ATP-dependent chromatin remodeling|histone exchange|perinuclea

ion|chromatin DNA binding|sequence-specific DNA binding|positive regulation of

|rDNA heterochromatin|histone binding|metal ion binding|lysine-acetylated histo
g

ell proliferation|nucleus|transcription, DNA-templated|regulation of transcript
asal polarity|positive regulation of protein targeting to membrane|microtubule

intermediate compartment membrane|azurophil granule membrane|neutrophil degran
ranule membrane

c

iol|response to progesterone|regulation of cell proliferation|response to hydro

olin-1-rich granule membrane

sis and secretion|Insulin secretion|Thyroid hormone synthesis|Circadian entrainm

cell migration|negative regulation of cell growth|axon|dendrite|extracellular
lopment|response to iron(II) ion|response to iron(III) ion|response to zinc ion

ty|negative regulation of angiogenesis|protein kinase binding|phosphatase bindi

evelopment|peripheral nervous system neuron development

chromatin silencing|transcriptionally active chromatin|negative regulation of
one donor, and incorporation of one atom of oxygen|sulfur oxidation|actin filam

biquitin-dependent protein catabolic process|cytolytic granule|metal ion bindin

lar response to virus|cardiolipin binding|positive regulation of intrinsic apop

e cell adhesion molecules|catenin complex|adherens junction organization|synapt

tein ubiquitination|nucleosome binding|protein-containing complex|negative regu

yrosine kinase signaling pathway|rRNA transcription|positive regulation of gene
n-containing complex assembly

vity|membrane|integral component of membrane|calcium-dependent cell-cell adhesi

dedifferentiation

ell activation|dynein light intermediate chain binding
domain specific binding|nuclear pericentric heterochromatin|identical protein l

ll-cell adhesion

binding|extracellular exosome
NAD(P)H as one donor, and incorporation of one atom of oxygen|SH3 domain bindi

eption of pain|extracellular exosome|neuronal stem cell population maintenance|

ein heterodimerization activity|protein homooligomerization|mitochondrial calci

to kinetochore|protein localization to centrosome|mitotic spindle|regulation o

omal lumen|collagen-containing extracellular matrix

lation of G1/S transition of mitotic cell cycle

ulation of blood vessel endothelial cell migration|apical part of cell|labyrintl

ndopeptidase activity|negative regulation of phospholipase A2 activity

l ion binding|negative regulation of canonical Wnt signaling pathway|negative r

during action potential|regulation of heart rate by cardiac conduction|membran
dent 5’-3’ RNA helicase activity|ribosome binding|ATP-dependent 5’-3’ DNA helic

tivity involved in regulation of muscle adaptation|oxidoreductase activity|mult

nventional myosin complex|nuclear body|Ral GTPase binding|vesicle transport alo

ing endosome membrane|potassium ion transmembrane transport|sodium ion import a

ter activity

on of protein localization to nucleus

homooligomerization|positive regulation of protein kinase B signaling|protein [illegible]
in modification|cellular protein metabolic process|transition metal ion binding

nding|negative regulation of catalytic activity|intermediate filament cytoskele[illegible]

nal bouton|positive regulation of Notch signaling pathway|protein autophosphory[illegible]

egulation of transcription by RNA polymerase II|anatomical structure morphogene[illegible]

iption factor binding|cell differentiation|sequence-specific DNA binding|protei[illegible]
myosin tail binding|photoreceptor connecting cilium|dynactin binding|protein se[illegible]

vity, RNA polymerase II-specific|proximal promoter DNA-binding transcription re[illegible]

nding
n of insulin receptor signaling pathway|regulation of mitochondrial membrane po[illegible]

ion|extracellular matrix|endodermal cell differentiation|camera-type eye morpho[illegible]

|MLL3/4 complex|cell fate commitment|endothelial cell differentiation|positive [illegible]

rotein catabolic process|membrane|internal peptidyl-lysine acetylation|natural [illegible]

ding|protein autophosphorylation|phosphatidylinositol phosphorylation|metal ion

tegral component of membrane|L-proline import across plasma membrane|L-tryptopha

class mediator|protein stabilization|protein autoubiquitination|MDM2/MDM4 fami
mune response|negative regulation of viral entry into host cell|positive regula
n

tion|Two-component system|Fatty acid metabolism|Butanoate metabolism|Propanoate
ne, aspartate and glutamate metabolism|Arginine biosynthesis|Tropane, piperidine
exocytosis|negative regulation of insulin secretion|metal ion binding|positive

egulation of I-kappaB kinase/NF-kappaB signaling|intracellular membrane-bounded

gulation of platelet-derived growth factor production
activity|nucleus|nervous system development|axon guidance|cerebellum developmen

tracellular exosome|L27 domain binding|MPP7-DLG1-LIN7 complex|presynapse|protei

ural crest cell migration involved in outflow tract morphogenesis|regulation of

inuclear region of cytoplasm|alpha-actinin binding|recycling endosome membrane|

|insulin receptor signaling pathway via phosphatidylinositol 3-kinase|intracell

nc ion import across plasma membrane

y|nucleus|nucleoplasm|transcription, DNA-templated|cell-matrix adhesion|Notch s

subpallium development|hippocampus development|forebrain neuron differentiation
to cell cortex

differentiation|regulation of synaptic plasticity|dendrite self-avoidance|cell-

mplex|histone deacetylase binding|ATP-dependent chromatin remodeling

atin assembly|protein-containing complex|CAF-1 complex|ESC/E(Z) complex|histone
on|regulation of skeletal muscle contraction by calcium ion signaling|myosin ph
ost-translational protein modification|regulation of skeletal muscle fiber deve
f apoptotic process|positive regulation of I-kappaB kinase/NF-kappaB signaling|
f1 complex|stem cell population maintenance|positive regulation of transcriptio

ɔsitive regulation of neuron differentiation|perinuclear region of cytoplasm|st

g|recombinational interstrand cross-link repair|protein-containing complex bind

lation of RNA polymerase II C-terminal domain

ulation of cell cycle arrest

ve regulation of transcription, DNA-templated|positive regulation of transcript

otic spindle assembly|activation of GTPase activity

ing|synapse|nitric-oxide synthase binding|ATPase binding|regulation of ventricu
transduction by protein phosphorylation|microvillus assembly|negative regulati

sm|regulation of DNA replication|chromatin organization|regulation of transcrip

|cellular response to gonadotropin-releasing hormone|axon cytoplasm

sembly
(SSU-rRNA, 5.8S rRNA, LSU-rRNA)|DNA binding|transcription coactivator activity|[illegible]
ojection development|microtubule cytoskeleton|Wnt signaling pathway|peptidyl-se[illegible]

ediated transport|Golgi organization|positive regulation of gene expression|int[illegible]

ization|midbody|cell projection|metal ion binding|cell division|oxidation-reduc[illegible]

terferon-alpha production|negative regulation of interferon-beta production|neg[illegible]

ior regionalization|regulation of cell-cell adhesion|dendrite morphogenesis|Wnt
d signaling pathway|identical protein binding|regulation of protein binding|reg[illegible]

xtrinsic component of cytoplasmic side of plasma membrane|dendrite cytoplasm|po[illegible]
chondrocyte differentiation|limb morphogenesis|multicellular organism growth|ne[illegible]

tility

opment|positive regulation of protein oxidation
complex|TAP2 binding

sis involved in innervation

|phospholipid transport|membrane|midbody|receptor tyrosine kinase binding|phospl
e II-specific|skeletal system development|DNA-binding transcription factor acti

cell migration|enzyme binding|lamellipodium assembly|Golgi cisterna|positive re

on of apoptotic process|negative regulation of apoptotic process|synapse

le cell differentiation|positive regulation of transcription by RNA polymerase

l to mesenchymal transition|folic acid transport|membrane|basolateral plasma mem

n factor binding|RNA polymerase II activating transcription factor binding|DNA-l

timulus|response to muscle activity|positive regulation of smooth muscle cell m

ucleolus|cytoplasm|cytosol|regulation of transcription, DNA-templated|transcrip
gative regulation of endopeptidase activity|negative regulation of protein proc

ical stimulus

f odontogenesis of dentin-containing tooth|lung growth|epithelial tube branchin

processing|identical protein binding|single-stranded DNA-dependent ATPase acti

ion|cell proliferation|positive regulation of neuron projection development|rib

|cadherin binding|cellular response to mechanical stimulus|tertiary granule lum

ganization|protein homodimerization activity|cell-cell adhesion mediated by cad

ity|steroid hormone receptor activity|nuclear receptor activity|thyroid hormone

nergic synapse|Phototransduction - fly|Oocyte meiosis|Calcium signaling pathwa

tion

l GTPase binding|regulation of Rho protein signal transduction|positive regulat

tive regulation of proteasomal ubiquitin-dependent protein catabolic process|er
e|positive regulation of dendritic spine maintenance
PKG signaling pathway|Long-term potentiation|B cell receptor signaling pathway|W

d organelle|calyx of Held|postsynaptic membrane|positive regulation of exocytos

thelial growth factor production|esophagus smooth muscle contraction|positive r

regulation of axonogenesis|regulation of small GTPase mediated signal transduc

protein signal transduction|dendritic spine|dendrite morphogenesis

pathway|MAPK signaling pathway - fly|Wnt signaling pathway

inding|negative regulation of calcium ion transmembrane transport via high volt
gulation of epidermis development|positive regulation of tau-protein kinase act

c synapse|Adrenergic signaling in cardiomyocytes|Circadian entrainment|Glutama

templated transcription in response to stress|negative regulation of Notch sign

on|Thyroid hormone synthesis|Circadian entrainment|Glutamatergic synapse|Cholin

matrix organization|chondroitin sulfate biosynthetic process|chondroitin sulfa

in complex|RNA polymerase II, holoenzyme|actin filament-based movement|endocyti

binding|positive regulation of cell proliferation|response to hormone|respons
ransport along microtubule

ding|cell junction|desmosome|keratinization|protein-containing complex scaffold

c membrane|proteasome-mediated ubiquitin-dependent protein catabolic process|sy

T complex|regulation of SNARE complex assembly|viral entry into host cell|metal
iquitination|ubiquitin protein ligase activity|regulation of CD8-positive, alph

protein binding|early endosome to late endosome transport|dynein light chain b

n of lipid metabolic process|titin binding|I band|cellular response to drug|ske

ty
ription, DNA-templated|histone demethylase activity (H3-K36 specific)|histone H

hetic process|lamellipodium membrane|intracellular signal transduction|lysosoma

y|nuclear receptor activity|nucleus|nucleoplasm|cytosol|transcription initiatio

transition of mitotic cell cycle|tubulin binding|protein kinase binding|neurog

ine phosphorylation|peptidyl-threonine phosphorylation|establishment of cell po
eutrophil degranulation|phospholipid translocation|positive regulation of prote

ollagen-activated tyrosine kinase receptor signaling pathway|peptidyl-tyrosine

ative regulation of interleukin-1 beta production|negative regulation of interl

egulation of angiogenesis|calcium-dependent protein binding|positive regulation

ignaling

ormone signaling pathway|Thyroid hormone synthesis|cGMP-PKG signaling pathway|Ca

to axon injury|actin filament bundle assembly|protein homooligomerization|posi

e regulation of ERK1 and ERK2 cascade

regulation of cell proliferation|animal organ morphogenesis|anterior/posterior

inding transcription factor activity|nucleus|nucleoplasm|nervous system develop

activity|retinol transport|signaling receptor activity|vocal learning|camera-t

ted phagocytosis|Chemokine signaling pathway|B cell receptor signaling pathway

ular exosome
y|cell differentiation|proteoglycan biosynthetic process|sequence-specific DNA

cross plasma membrane

duction|positive regulation of cardiac muscle cell apoptotic process|positive r

ent of secondary female sexual characteristics|metal ion binding

otential|detection of mechanical stimulus|cellular response to mechanical stimu

ated|defense response to virus|ribonucleoprotein complex

receptor activity|peptide hormone binding|pancreatic juice secretion|chloride i

genesis in camera-type eye|extracellular exosome|positive regulation of cell mi

on by host of viral glycoprotein metabolic process|innate immune response|posit

r DNA-binding transcription repressor activity, RNA polymerase II-specific|RNA

constituent of postsynaptic intermediate filament cytoskeleton|cellular respons

way|Taste transduction|cGMP-PKG signaling pathway|Serotonergic synapse|Long-te
ment|intracellular signal transduction|axon extension|dendrite morphogenesis|ne

rotoxin receptor activity|cell junction|carbohydrate binding|axon|growth cone|c

negative regulation of histone H3-K9 acetylation

organization|aging|response to hormone|response to aluminum ion|membrane|mitoch

rylation|synapse|oligodendrocyte differentiation|regulation of oligodendrocyte

y response|transcription factor binding|cell proliferation|regulation of autoph

|regulation of synapse maturation|postsynaptic endosome|neurotransmitter recept

:ergic synapse|cAMP signaling pathway|Cholinergic synapse|Retrograde endocannab:

gative regulation of MAP kinase activity|negative regulation of Ras protein sig

n of transcription, DNA-templated|positive regulation of transcription, DNA-tem

eceptor binding|regulation of androgen receptor signaling pathway|ubiquitin pro

oduction|activating transcription factor binding|negative regulation of osteobl
n kinase binding|cell projection assembly|actin cytoskeleton organization|Cdc42

nase binding|signal transduction by protein phosphorylation|signal transduction
sponse to tumor necrosis factor

l transduction|inner ear morphogenesis|positive regulation of apoptotic process

vation of GTPase activity|positive regulation of protein localization to plasma

f double-strand break|cell division|positive regulation of chromosome segregati

receptor-mediated endocytosis|cholesterol metabolic process|endosome membrane|r
naling pathway|protein-hormone receptor activity|bone mineralization|male genit

y|protein homodimerization activity|histone deacetylase binding|positive regula

ine|glutamatergic synapse|postsynaptic modulation of chemical synaptic transmis

ia MHC class I|peptide binding|peptide catabolic process|metalloaminopeptidase

|CENP-A containing nucleosome assembly|protein heterodimerization activity|kine

binding|cellular response to mechanical stimulus|positive regulation of apopto

regulation of neuron differentiation|negative regulation of transcription, DNA-

e regulation of protein autophosphorylation|positive regulation of peptidyl-ser

ation to chromatin

ehavior|negative regulation of cold-induced thermogenesis

sm|Glycolysis / Gluconeogenesis

ubiquitination|arrestin family protein binding

cell fate specification|nuclear body|dorsal/ventral neural tube patterning|sig

ment|Glutamatergic synapse|Bile secretion|cAMP signaling pathway|Cholinergic syn

ng at plasma membrane|cytoplasmic side of plasma membrane|intercalated disc|act

protein binding|regulation of plasminogen activation|Rab GTPase binding|phosph

abolic process|norepinephrine biosynthetic process
actor activity|transcription regulatory region DNA binding|negative regulation
lin secretion|Thyroid hormone synthesis|Circadian entrainment|Glutamatergic syna

une response

|membrane raft|postsynaptic membrane|negative regulation of extrinsic apoptotic

t|glomerular visceral epithelial cell migration|negative regulation of G1/S tra

mbrane organization

e

ing|regulation of dendrite morphogenesis|cellular response to amino acid stimul
eton organization|intermediate filament organization|intermediate filament bund

t-embryonic development|RNA secondary structure unwinding|miRNA metabolic proce

activation|Ras signaling pathway|Vascular smooth muscle contraction|Inflammat

sis of a branching structure|kidney development|vasculature development|nucleus

protein ligase activity|negative regulation of viral release from host cell

horylation|R-SMAD binding

assembly|metal ion binding
elasticity|extracellular matrix organization|extracellular matrix|cell adhesion

cular smooth muscle contraction|Calcium signaling pathway|GnRH signaling pathway

-K4 demethylation|methylated histone binding|histone methyltransferase complex|

embly|M band|protein localization to Golgi apparatus|post-translational protein

rase II-specific|proximal promoter DNA-binding transcription repressor activity

lation of fibroblast proliferation|protein poly-ADP-ribosylation|protein auto-A

ration along microfilament|nuclear membrane|meiotic nuclear membrane microtubul

mbrane|negative regulation of GTPase activity|chromosome, centromeric core doma

thway|B cell receptor signaling pathway|extracellular exosome|protein localizat

methylation

l adhesion molecule binding|cell division|collagen-containing extracellular mat

/NF-kappaB signaling|intracellular membrane-bounded organelle|receptor complex|
tivity|cellular cation homeostasis|transport vesicle|manganese ion binding|vesi

proliferation|sensory perception of pain|dendrite|symmetric synapse|response to

naling pathway involved in ventral spinal cord patterning|central nervous syste

g|cell proliferation|negative regulation of cell proliferation|anterior/posteri

NA transport|protein homooligomerization|long-term synaptic potentiation|dendri

ctivity|peptide binding|receptor complex|positive regulation of MAPK cascade|ne

to lysosome|extracellular exosome|cellular response to amino acid stimulus

olgi|cell projection|extracellular exosome

transporter activity|extracellular exosome|tertiary granule membrane|ficolin-1-

inding|lamellipodium|regulation of cell migration|lamellipodium membrane|positi

hosphate binding|negative regulation of protein kinase B signaling|phosphatidyl
|extracellular matrix organization|extracellular matrix|aorta morphogenesis|int

serine-type endopeptidase activity|negative regulation of phospholipase A2 acti
ne|protein-containing complex|eRF1 methyltransferase complex|protein-glutamine [illegible]

lex|multicellular organism growth|embryonic organ development|somite developmen

etal muscle tissue regeneration|sequence-specific DNA binding|perinuclear regio

mbrane|epithelial cell differentiation|social behavior|identical protein bindin

osome transport|AP-1 adaptor complex binding|AP-3 adaptor complex binding|BLOC-

gulation of intracellular estrogen receptor signaling pathway|response to endop
matrix|response to tumor necrosis factor|specific granule lumen|neutrophil degr

on|negative regulation of protein localization to microtubule|positive regulati

ate gland development|negative regulation of proteasomal ubiquitin-dependent pr

activity|transcription corepressor activity|nucleus|nucleoplasm|regulation of t
r activity|nucleus|DNA replication|negative regulation of cell proliferation|re
ix adhesion|negative regulation of leukocyte migration|transcription factor act

al pattern formation|subpallium development|hippocampus development|olfactory b

exosome

ellular exosome|lymphocyte aggregation|regulation of lymphocyte migration

of microtubule cytoskeleton organization|dynein complex binding|minus-end-direc

ative regulation of interleukin-1 beta production|negative regulation of interl

o glucose stimulus|neuronal cell body|perikaryon|response to estrogen|axon term

methylase activity (H3-K36 specific)|histone H3-K36 demethylation

tinocyte proliferation|actin filament binding|keratinocyte migration|negative r
|protein dimerization activity|regulation of carbohydrate metabolic process by [illegible]

cle|organelle transport along microtubule

or|positive regulation of signaling receptor activity

in receptors|regulation of DNA damage checkpoint
ion by RNA polymerase II|metal ion binding|protein dimerization activity|C2H2 z
l|nervous system development|phosphatase regulator activity|corpus callosum mor

ion of p21 class mediator|transcription factor binding|negative regulation of e
ort|identical protein binding|regulation of insulin secretion involved in cellu

f protein secretion|actin filament binding|Golgi ribbon formation|asymmetric Go

ermogenesis

nt|respiratory gaseous exchange|transcription factor binding|rhombomere 5 devel

regulation of transcription by RNA polymerase II

ation of cartilage development|cellular response to transforming growth factor

of signal transduction by p53 class mediator|regulation of cellular senescence

smembrane transporter activity|L-serine transmembrane transporter activity|L-th

se II|signal transduction|cell differentiation|purine-rich negative regulatory

gulation of cell death|regulation of eIF2 alpha phosphorylation by amino acid s

te migration|leukocyte tethering or rolling|actin filament binding|barbed-end a

ded organelle|protein binding involved in protein folding|unfolded protein bind

onal protein modification|cellular protein metabolic process|intercellular brid

assembly|positive regulation of protein targeting to mitochondrion

t|Phosphatidylinositol signaling system|Glutamatergic synapse|Cholinergic synaps
ermeabilization|negative regulation of extrinsic apoptotic signaling pathway in

opeptidase activity involved in apoptotic process|CARD domain binding|negative [illegible]

tion of apoptotic signaling pathway

ongression|5S rRNA binding|preribosome, large subunit precursor|ribosomal large

hosphate 5-phosphatase activity|phosphatidylinositol phosphate 4-phosphatase ac

f apoptotic process|hypomethylation of CpG island|cadherin binding|negative reg

ption factor binding|DNA-binding transcription activator activity, RNA polymera

sphatase activity|inositol diphosphate pentakisphosphate diphosphatase activity

e differentiation|metal ion binding

transcription, DNA-templated|transcription factor binding|viral genome replica

y, RNA polymerase II-specific|nucleus|cytoplasm|mRNA export from nucleus|transc

ation of chemical synaptic transmission|positive regulation of long-term synapt

sis|DNA-binding transcription factor activity|nucleus|nucleoplasm|transcription

uting angiogenesis

ulation of canonical Wnt signaling pathway|negative regulation of metalloendope

transmembrane transporter activity

ative regulation of interleukin-1 beta production|negative regulation of interl
leus|transcription factor complex|transcription factor binding|extracellular ma

t|midbrain-hindbrain boundary morphogenesis|rhombomere 2 development|thalamus d

virus|regulation of cell cycle|type I interferon signaling pathway|membrane fu

nscription factor complex|chromatin remodeling|embryonic pattern specification|

esis|histone deacetylase binding|transcription regulatory region DNA binding|ne

egulation of collagen biosynthetic process|identical protein binding|neuron pro
transcription by RNA polymerase II|positive regulation of type I interferon pro

gnal transduction|protein localization to M-band|sarcolemma|positive regulation

ine kinase activity|actin filament reorganization|positive regulation of substr
ein homodimerization activity|neuronal cell body|ion channel binding|dendritic

oid hormone synthesis|Circadian entrainment|Glutamatergic synapse|Bile secretio

ion activity

enesis|DNA binding|transcription coactivator activity|transcription corepressor

f RNA splicing|cellular response to unfolded protein|negative regulation of apo

lation|transcription factor binding|regulation of double-strand break repair vi

onstituent|calmodulin binding|collagen binding|extracellular region|extracellul

mplex|regulation of lipid metabolic process|B cell differentiation|cyclin bindi

assembly|cell adhesion involved in heart morphogenesis|extracellular exosome|ba

on corepressor activity|nuclear receptor activity|nucleus|nucleoplasm|cytosol|r
midbody|intracellular signal transduction|ciliary basal body|neuron projection|

ding|positive regulation of signal transduction by p53 class mediator

n of neuron projection development|postsynaptic density|sarcoplasm|central nerv

ive regulation of multicellular organism growth|SH2 domain binding|cell project

vesicle

inflammasome complex assembly|ceramide 1-phosphate binding|ceramide 1-phosphat

lular membrane-bounded organelle|muscle fiber development|extracellular exosome

septum morphogenesis|diaphragm development|cell-cell adhesion via plasma-membra

gulation of transcription, DNA-templated|positive regulation of transcription b

the release of sequestered calcium ion|release of sequestered calcium ion into

|sodium ion transmembrane transport|identical protein binding|perikaryon|intrac

actor beta receptor signaling pathway|negative regulation of vascular endotheli

thway|positive regulation of T cell migration

ral component of plasma membrane|immune response|cell surface|ER to Golgi trans

emplated|cellular response to DNA damage stimulus|methyl-CpG binding|methyl-CpN

tenance of granzyme B location in T cell secretory granule|mast cell granule|ne

morphogenesis|positive regulation of dendritic spine morphogenesis|positive re

lation of synaptic vesicle exocytosis
ng|extracellular matrix|neuron projection development|protein homodimerization [illegible]
on of viral genome replication|innate immune response|perinuclear region of cyt[illegible]

ng|maintenance of chromatin silencing|regulation of transcription by RNA polyme[illegible]

y|neuron development|positive regulation of oligodendrocyte differentiation|cel

ignal transduction|positive regulation of apoptotic process|apical part of cell

tion of transcription by RNA polymerase II|immune response|pattern specificatio[illegible]

cytosol|intermediate filament cytoskeleton|positive regulation of transcription

ıway|Thyroid hormone signaling pathway|Prolactin signaling pathway|Chemokine si
L-lysine transmembrane transporter activity|membrane|cell junction|macrophage a
ositive regulation of transforming growth factor beta1 production|keratan sulfa

lathrin-coated vesicle membrane|clathrin-coated endocytic vesicle membrane|regu

f fat cell differentiation|ubiquitin-protein transferase regulator activity
thway|forebrain development|chromatin DNA binding|embryonic eye morphogenesis|r

activity|RNA transmembrane transporter activity|regulation of insulin secretio

N-terminus binding|COPII vesicle coating|regulation of protein transport|regul

lation|basement membrane organization

muscle organ development|post-embryonic development|gene silencing|oligodendroc[illegible]

leukin-13 production|negative regulation of interleukin-4 production|negative r[illegible]
lation of cell-cell adhesion mediated by integrin|slit diaphragm|intracellular [illegible]

serine/threonine kinase activator activity|positive regulation of DNA replicati[illegible]

ransmitter reuptake

egulation of dendrite development|neural precursor cell proliferation|glutamate

dbrain development|negative regulation of hippo signaling|positive regulation o

otein-containing complex localization|protein homodimerization activity|histone

n of cell fate commitment|regulation of transcription from RNA polymerase II pr
negative regulation of histone H3-K9 acetylation

differentiation|vesicle|high-density lipoprotein particle assembly|identical pr

ng|perinuclear region of cytoplasm|spindle midzone|cornification|mitotic spindl
g|histone H3 acetylation|histone H4-K5 acetylation|histone H4-K8 acetylation|hi
assembly

tive regulation of transcription by RNA polymerase II|stem cell differentiation

of two atoms of oxygen|retinoic acid metabolic process|retinal metabolic proce

nt|chondrocyte differentiation|mesenchymal to epithelial transition involved in
embrane|myelin assembly|negative regulation of fibroblast growth factor recepto

mal ubiquitin-dependent protein catabolic process|phosphatidylinositol 3-kinase

oly involved in gastrulation with mouth forming second|embryonic heart tube lef
ction|connexin complex|potassium ion transport|cell communication by chemical c

r space|intracellular|apoptotic process|signal transduction|cell-cell signaling

ation activity|response to peptide hormone|post-translational protein modificat

hway|forebrain development|camera-type eye development|negative regulation of o

arginine transmembrane transport|L-lysine transmembrane transport|mitochondrial

nt|anterior/posterior pattern specification|proximal/distal pattern formation|e

stress-activated protein kinase signaling cascade|activation of protein kinase

protein dimerization activity|brown fat cell differentiation|adipose tissue dev

tor-activated receptor activity|sequence-specific DNA binding|negative regulati

cer sequence-specific binding|nucleus|nucleoplasm|cytosol|regulation of transcr

re-mRNA intronic binding|response to peptide|positive regulation of mRNA bindin

nance of epithelial cell apical/basal polarity|cell division

ller cell mediated cytotoxicity|cysteine-type endopeptidase inhibitor activity

ed signaling pathway|skeletal system morphogenesis|ciliary membrane|epithelial

ellular matrix organization|negative regulation of cell growth|negative regulat

migration|axon|dendrite|stress-activated protein kinase signaling cascade|acti

signaling pathway|Estrogen signaling pathway

ion to nuclear pore
genesis|regulation of protein K63-linked ubiquitination|positive regulation of

ay|positive regulation of cristae formation|negative regulation of ATP citrate

rophage cytokine production|extracellular exosome|cellular response to lipopoly

ic stem cell population maintenance|regulation of kinase activity|positive regu

anelle|positive regulation of growth of symbiont in host|perinuclear region of

ıthesis|Cysteine and methionine metabolism|Phenylalanine metabolism

of transcription by RNA polymerase II|positive regulation of transcription by

|regulation of cytokinesis|positive regulation of cytokinesis|histone serine ki

oxygen, 2-oxoglutarate as one donor, and incorporation of one atom each of oxy

erase I|positive regulation of NF-kappaB transcription factor activity|NLRP3 in

aft|calcium-dependent protein binding|actin filament binding|cellular localizat

tor activity|growth factor binding|cytokine binding|facial nerve structural org[illegible]

ction|connexin complex|potassium ion transport|cell communication by chemical c[illegible]

promoter DNA-binding transcription repressor activity, RNA polymerase II-speci

activity|identical protein binding|metal ion binding

llular exosome|response to cisplatin|extrinsic component of Golgi membrane|lume[illegible]

erminus|motile cilium assembly|negative regulation of epithelial cell prolifera[illegible]

Ras signaling pathway|PI3K-Akt signaling pathway|Focal adhesion|MAPK signaling [illegible]
terferon signaling pathway|cellular response to type I interferon|cellular resp[illegible]
|metal ion binding

ulation of cell growth|negative regulation of cell migration|anchored component

granulation|intermediate filament bundle assembly|cadherin binding|cornificatio[illegible]

ll GTPase mediated signal transduction|membrane|phosphorylation|clathrin-coated

to norepinephrine stimulus|negative regulation of adenylate cyclase-activating

pholipid metabolic process|activation of cysteine-type endopeptidase activity i[illegible]

n by interphase microtubule organizing center|mitotic spindle assembly
tracellular protein transport|cellular response to chemokine|negative regulatio

n|mitochondrial calcium ion homeostasis|positive regulation of mitochondrial ca

s-end-directed|microtubule cytoskeleton|membrane|ATPase activity|kinesin II com

hway|post-translational protein modification|cellular protein metabolic process

cell motility

ar response to leucine starvation

containing extracellular matrix

ation of canonical Wnt signaling pathway

ell proliferation|dendritic cell homeostasis|T follicular helper cell different

ng

enance via telomerase|DNA duplex unwinding|telomeric DNA binding|single-strande

tion|axon|Wnt-activated receptor activity|negative regulation of apoptotic proc

domain proteolysis|ATPase binding|liver regeneration|regulation of membrane dep

tive regulation of cell migration|positive regulation of actin filament depolym

scade|positive regulation of synapse assembly

iquitin-dependent protein catabolic process|post-translational protein modifica

xosome

ytoskeleton|protein phosphorylation|ubiquitin-dependent protein catabolic proce

nslocation|negative regulation of transcription, DNA-templated
sitive regulation of macrophage activation|positive regulation of inflammatory

ion|peptide binding|hormone metabolic process|varicosity|perikaryon|axon termi

way|Adipocytokine signaling pathway

hetic process|lamellipodium membrane|intracellular signal transduction|lysosoma

ded protein response

|post-translational protein modification|cellular protein metabolic process|pos

rin binding|actin filament bundle assembly|positive regulation of mast cell che

kinje myocyte adhesion involved in cell communication|cell adhesive protein bin

rane organization|cell periphery
ment|DNA binding|DNA-binding transcription factor activity|nucleus|nucleoplasm|

-mediated oocyte maturation|Regulation of lipolysis in adipocytes|Natural kill

ding|cell junction|desmosome|keratinization|protein-containing complex scaffold

ubule cytoskeleton organization
protein binding|negative regulation of transcription, DNA-templated|negative r

ation of cell motility

cission

response to calcium ion|presynapse

plasm|cytoplasm|muscle organ development|protein deubiquitination|cell differen

assembly

lopment|cell adhesion molecule binding|self proteolysis|regulation of homophili

|regulation of protein stability|negative regulation of proteasomal ubiquitin-d

pamine receptor binding|dendritic spine membrane|intracellular membrane-bounded

n specification|dorsal/ventral pattern formation|nuclear speck|rhombomere 3 dev

gative regulation of protein binding|Golgi cisterna membrane|endoplasmic reticu

anterior/posterior pattern specification|rhombomere development|glossopharyngea

tor production|identical protein binding|positive regulation of regulatory T ce

ir|chromatin organization|transcription initiation from RNA polymerase II promo

ulation of peptidyl-serine phosphorylation|calmodulin-dependent protein phospha

scription corepressor activity|nucleus|transcription by RNA polymerase II|trans

ion|cadherin binding|mRNA 5'-UTR binding|mRNA stabilization|cellular response t

tive regulation of Wnt protein secretion|extracellular exosome|cellular respons

lation of myoblast differentiation|mRNA stabilization|mRNA destabilization|3'-U

solateral plasma membrane

gulation by virus of viral protein levels in host cell|extracellular exosome|gl

membrane|cytoplasmic vesicle|myosin V binding|Rab protein signal transduction|

binding|clathrin-coated endocytic vesicle|ER retention sequence binding|positi

lin-dependent protein kinase holoenzyme complex|microtubule anchoring|neuroendo
Wnt signaling pathway|Oxytocin signaling pathway

ulation of protein stability|protein-containing complex|regulation of locomotio

A binding|transcription factor activity, RNA polymerase II distal enhancer sequ

on transmembrane transport
asma membrane tubulation|positive regulation of feeding behavior
A polymerase II distal enhancer sequence-specific binding|nucleus|pattern speci

ocess|negative regulation of cell cycle|negative regulation of fibroblast proli

|toxin metabolic process|cell surface|membrane|integral component of membrane|p

ell proliferation|sequence-specific DNA binding|regulation of epidermal cell di

ic methyltransferase activity|ribosome binding|metal ion binding

lacenta development|extracellular exosome|cornification|cell periphery|terminal

es|heart development|beta-catenin binding|germ cell migration|epithelial struct

cortical endoplasmic reticulum|sterol binding|phosphatidylserine acyl-chain rem
neuron projection|axon cytoplasm|anterograde neuronal dense core vesicle transp

ukin-17 production|positive regulation of interleukin-23 production|positive re

osome|tertiary granule|cellular response to interferon-gamma

apical plasma membrane|ion transmembrane transport|cellular response to heat|ch

of mitotic cell cycle|positive regulation of MDA-5 signaling pathway|positive

cleus|transcription factor complex|cytoplasm|regulation of transcription by RNA

egulation of stress fiber assembly|negative regulation of protein serine/threon

fic DNA binding|proximal promoter DNA-binding transcription repressor activity,

erine 1-acylhydrolase activity|1-acyl-2-lysophosphatidylserine acylhydrolase ac

mbrane|cell migration|GDP binding|protein kinase binding|cell differentiation|r

dylinositol-4-phosphate binding|osteoclast fusion

m assembly|ciliary transition zone|protein homodimerization activity|intracellu

r region of cytoplasm

transcription, DNA-templated|positive regulation of transcription by RNA polyme

ne binding

ion, DNA-templated|cell cycle|regulation of mitotic cell cycle|zinc ion binding
bundle|positive regulation of microtubule motor activity

ulation

gen peroxide|positive regulation of apoptotic process|platelet-derived growth f

nent|Glutamatergic synapse|Bile secretion|cAMP signaling pathway|Cholinergic syr

matrix|cell adhesion mediated by integrin|endothelial cell chemotaxis|neuronal
|skeletal muscle atrophy|response to activity|negative regulation of translatio

ng|protein serine/threonine kinase inhibitor activity|regulation of MAPK cascad

DNA recombination|histone H3-K4 trimethylation|histone H3-K27 trimethylation
ent depolymerization|NADPH:sulfur oxidoreductase activity|metal ion binding|oxi

g|ubiquitin protein ligase activity

totic signaling pathway

ic transmission, glutamatergic|protein homodimerization activity|synaptic cleft

lation of transcription elongation from RNA polymerase II promoter|histone H2A [illegible]

expression, epigenetic

on via plasma membrane cell adhesion molecules|cellular manganese ion homeostas

binding|negative regulation of transcription, DNA-templated|rhythmic process|ne

ng|Rab GTPase binding|sulfur oxidation|protein kinase binding|actin filament de

cell-cell adhesion via plasma-membrane adhesion molecules

um ion homeostasis|positive regulation of mitochondrial calcium ion concentrati

f metaphase plate congression|mitotic spindle pole|positive regulation of spind

hine layer blood vessel development|negative regulation of lymphangiogenesis|re

egulation of G1/S transition of mitotic cell cycle

e repolarization during ventricular cardiac muscle cell action potential|negati
ase activity|protein homooligomerization|ribonucleoprotein complex

icellular organismal response to stress|cellular response to oxidative stress|l

ng actin filament|positive regulation of cell migration|cytoplasmic vesicle mem

cross plasma membrane

heterotrimerization|negative regulation of platelet aggregation|positive regula
|extracellular exosome

ton|stem cell proliferation|positive regulation of neural precursor cell prolif

lation|protein stabilization|membrane organization|regulation of clathrin-depen

sis|cell differentiation|positive regulation of multicellular organism growth|n

n dimerization activity|regulation of neurogenesis|sequence-specific double-str
lf-association|dynein intermediate chain binding|protein homooligomerization|ac

pressor activity, RNA polymerase II-specific|distal enhancer DNA-binding transc

tential|positive regulation of fat cell proliferation|mitochondrion morphogenes

genesis|epithelial cell proliferation|collagen-containing extracellular matrix

regulation of transcription by RNA polymerase II|metal ion binding|mesodermal c

killer cell activation|cell differentiation|lung development|ubiquitin-dependen

binding|regulation of response to DNA damage stimulus

an transmembrane transport

ly protein binding|negative regulation of proteasomal protein catabolic process
tion of DNA-binding transcription factor activity|positive regulation of NF-kap

metabolism|Benzoate degradation
e and pyridine alkaloid biosynthesis|Cysteine and methionine metabolism|Phenyla
regulation of protein secretion|exocytic vesicle|multivesicular body sorting p

organelle|perinuclear region of cytoplasm|WW domain binding|negative regulatio

t|midbrain-hindbrain boundary morphogenesis|rhombomere 2 development|thalamus d

n localization to basolateral plasma membrane

secondary heart field cardioblast proliferation|apoptotic process involved in [illegible]

excitatory chemical synaptic transmission

ular protein transmembrane transport|protein localization to plasma membrane|po

ignaling pathway|anterior/posterior pattern specification|positive regulation o

|regulation of transcription from RNA polymerase II promoter involved in forebr

-cell adhesion mediator activity

deacetylase binding
osphatase regulator activity|peptidyl-serine phosphorylation|integral component
lopment
perinuclear region of cytoplasm|regulation of small GTPase mediated signal tran
n elongation from RNA polymerase II promoter|positive regulation of transcripti

riated muscle cell differentiation|positive regulation of muscle cell different

ing|mismatch repair involved in maintenance of fidelity involved in DNA-depende

ion by RNA polymerase II|protein heterodimerization activity

lar cardiac muscle cell membrane repolarization|ventricular cardiac muscle cell
on of cell migration|stress-activated protein kinase signaling cascade|activati

tion by RNA polymerase II|transcription factor binding|release from viral laten

RNA binding|nucleus|transcription factor complex|nucleolus|regulation of transc
rine phosphorylation|establishment of cell polarity|dendrite|intracellular sign

egral component of membrane|COPII-coated ER to Golgi transport vesicle|COPI-coa

tion process|FAD binding

ative regulation of interleukin-1 beta production|negative regulation of interl

signaling pathway, planar cell polarity pathway
ulation of osteoclast differentiation|negative regulation of osteoclast differe

tassium channel complex|neuronal cell body|perikaryon|juxtaparanode region of a
gative regulation of Golgi to plasma membrane protein transport|lung alveolus d

hatidylcholine binding|cleavage furrow|Golgi cisterna membrane|phosphatidylinos
vity|nucleus|regulation of transcription by RNA polymerase II|transforming grow

gulation of TOR signaling|Golgi cisterna membrane|Golgi to plasma membrane prot

II|canonical Wnt signaling pathway|beta-catenin-TCF complex assembly|beta-caten

mbrane|apical plasma membrane|chemokine binding|lamellipodium|cell junction|pla

binding transcription repressor activity, RNA polymerase II-specific|T-helper c

igration|extracellular matrix organization|extracellular matrix|neuromuscular j

tion by RNA polymerase II|protein phosphorylation|core-binding factor complex|h
essing|negative regulation of phosphatidylinositol 3-kinase signaling|cerebella

g involved in lung morphogenesis|trachea cartilage morphogenesis|dopaminergic n

vity|innate immune response|metal ion binding|perinuclear region of cytoplasm

osome biogenesis|positive regulation of transcription, DNA-templated|homeostasi

en

herin|cadherin binding|cell-cell adhesion|glutamatergic synapse|GABA-ergic syna

receptor activity|nucleus|nucleoplasm|transcription initiation from RNA polyme

|GnRH signaling pathway|Gastric acid secretion|Inflammatory mediator regulatio

ion of apoptotic process|regulation of GTPase activity|filopodium assembly|meta

ror-free postreplication DNA repair|negative regulation of neuron apoptotic pro

Wnt signaling pathway|Oxytocin signaling pathway

is|phosphatidylinositol-mediated signaling|perinuclear region of cytoplasm|posi

egulation of Wnt signaling pathway|heparan sulfate proteoglycan metabolic proce

tion|extracellular exosome|semaphorin-plexin signaling pathway|regulation of ac

age-gated calcium channel
ivity

:ergic synapse|cAMP signaling pathway|Cholinergic synapse|Retrograde endocannab[illegible]

aling pathway|positive regulation of protein kinase C activity|positive regulat

ergic synapse|Phototransduction - fly|Retrograde endocannabinoid signaling|Vascu

te catabolic process|dermatan sulfate biosynthetic process|extracellular matrix

c vesicle|DNA damage response, signal transduction by p53 class mediator|clathr

e to organic substance|negative regulation of endopeptidase activity|cytokine-m

activity|adherens junction maintenance|ion channel binding|intermediate filame

napse|postsynaptic membrane|synapse assembly involved in innervation|glutamater

ion binding|presynapse|glutamatergic synapse|regulation of synaptic vesicle ex
a-beta T cell proliferation|negative regulation of extrinsic apoptotic signalin

inding|dynein intermediate chain binding|negative regulation of neurogenesis|Go

letal muscle cell differentiation|response to muscle stretch|histone deacetylas

3-K36 demethylation

l lumen|tissue remodeling|positive regulation of peptidyl-tyrosine phosphorylat

n from RNA polymerase II promoter|signal transduction|zinc ion binding|negative

enesis|cell junction|microtubule organizing center organization|positive regula

larity|protein kinase activator activity|dendrite|activation of protein kinase
in exit from endoplasmic reticulum

autophosphorylation|negative regulation of apoptotic process|receptor complex|w

eukin-12 production|negative regulation of interleukin-6 production|negative re

of DNA metabolic process|positive regulation of DNA-binding transcription fact

ardiac muscle contraction

tive regulation of filopodium assembly|actin crosslink formation|exocytic vesic

pattern specification|proximal/distal pattern formation|positive regulation of

ment|blood coagulation|sequence-specific DNA binding|RING-like zinc finger doma

ype eye development|ear development|nose morphogenesis|lung alveolus developmen

binding|Peyer's patch morphogenesis

egulation of neuron projection development|phosphatidylinositol 3-kinase signal

lus

on binding|protein-containing complex|regulation of osteoblast proliferation|po

gration involved in sprouting angiogenesis

ive regulation of nitric oxide biosynthetic process|negative regulation of vira

polymerase II activating transcription factor binding|blood vessel development|

e to leukemia inhibitory factor

rm potentiation|Oxytocin signaling pathway|Cardiac muscle contraction
gative regulation of protein localization to nucleus

alcium-mediated signaling using intracellular calcium source|presynaptic membra

ondrial DNA metabolic process|cellular response to oxidative stress|mitochondri

progenitor proliferation|perineuronal net

agy|membrane|nuclear speck|hemopoiesis|myeloid cell differentiation|monocyte di

or transport to postsynaptic membrane|glutamatergic synapse|GABA-ergic synapse|

inoid signaling|Tight junction|cGMP-PKG signaling pathway|Gastric acid secretio

nal transduction|negative regulation of neurotrophin TRK receptor signaling pat

plated|lysine-acetylated histone binding|regulation of signal transduction by p

tein ligase activity|protein K48-linked ubiquitination|protein K6-linked ubiqui

ast differentiation|metal ion binding|repressing transcription factor binding|1
protein signal transduction|cell projection|metal ion binding|regulation of sm

by trans-phosphorylation|stress-activated protein kinase signaling cascade|act

|regulation of vascular permeability|negative regulation of neutrophil degranul

membrane

on|cellular senescence

egulation of smooth muscle cell migration|membrane|low-density lipoprotein part
alia development|circadian regulation of gene expression|negative regulation of

tion of retinoic acid receptor signaling pathway

sion|positive regulation of postsynaptic cytosolic calcium concentration|spine

activity

tochore assembly|protein localization to chromosome, centromeric region

tic signaling pathway

templated|metal ion binding|protein heterodimerization activity

ine phosphorylation|protein-containing complex binding|cadherin binding|cellula

nal transduction involved in regulation of gene expression|negative regulation

napse|Oocyte meiosis|Retrograde endocannabinoid signaling|Calcium signaling path

in cytoskeleton|apicolateral plasma membrane|lateral plasma membrane|catenin co

olipase A2 inhibitor activity|collagen fibril organization|biomineral tissue de

of smoothened signaling pathway|negative regulation of transcription, DNA-templ
apse|Bile secretion|cAMP signaling pathway|Cholinergic synapse|Oocyte meiosis|R

signaling pathway via death domain receptors

nsition of mitotic cell cycle

us|cellular response to electrical stimulus|cellular response to gonadotropin s
le assembly|negative regulation of epithelial cell proliferation|negative regul

ss|positive regulation of gene expression|negative regulation of gene expressio

ory mediator regulation of TRP channels|Glutamatergic synapse

|sex determination|branchiomeric skeletal muscle development|epithelial cell di

mediated by integrin|activation of protein kinase A activity|sequestering of B

y|Taste transduction|cGMP-PKG signaling pathway|Gastric acid secretion|Estrogen

metal ion binding|androgen receptor binding|dioxygenase activity|regulation of

modification|sarcomere organization|perinuclear region of cytoplasm|cardiac mu

, RNA polymerase II-specific|distal enhancer DNA-binding transcription activato

DP-ribosylation|K63-linked polyubiquitin modification-dependent protein binding

e tethering complex|identical protein binding|protein membrane anchor|meiotic c

in|establishment of protein localization|focal adhesion assembly|Rac GTPase bin

ion to membrane raft

rix|extracellular exosome

positive regulation of neuron differentiation|positive regulation of inflammato
cle|multivesicular body membrane|regulation of intracellular protein transport|

estradiol|response to lipopolysaccharide|synaptic vesicle lumen|cellular respo

m neuron differentiation|embryonic camera-type eye development|phosphatidylinos

or axis specification|negative regulation of Schwann cell proliferation|myotube

tic spine morphogenesis|regulation of dendritic spine morphogenesis|postsynapti

gative regulation of neuron differentiation|negative regulation of smoothened s

rich granule membrane|glucose transmembrane transport

ve regulation of protein binding|aorta development|regulation of GTPase activit

inositol-3,5-bisphosphate binding
racellular membrane-bounded organelle|positive regulation of melanocyte differe

vity
N-methyltransferase activity

t|ubiquitin protein ligase activity

n of cytoplasm|positive regulation of NF-kappaB transcription factor activity|s

g|calcium-dependent protein binding|response to calcium ion|membrane fusion|ext

2 complex binding|melanosome|Mitochondria-associated ER Membrane|phagocytic ves

lasmic reticulum stress|neuron projection|negative regulation of apoptotic proc
anulation|positive regulation of angiogenesis|perinuclear region of cytoplasm|c

on of protein localization to endosome

otein catabolic process|protein-containing complex|ATP-dependent chromatin remo

ranscription, DNA-templated|embryonic body morphogenesis|negative regulation of
sponse to bacterium|glial cell differentiation|principal sensory nucleus of tri
ivity, RNA polymerase II distal enhancer sequence-specific binding|nucleus|nucl

ulb development|regulation of transcription from RNA polymerase II promoter inv

ted organelle transport along microtubule|microtubule anchoring at microtubule

eukin-12 production|negative regulation of interleukin-6 production|negative re

inus|ubiquitin-like protein ligase binding|synapse|positive regulation of trans

egulation of timing of anagen|basement membrane organization|negative regulatio
regulation of transcription from RNA polymerase II promoter

inc finger domain binding
phogenesis|hippocampus development|cell proliferation in forebrain|corticospina

ndothelial cell migration|viral process|nuclear body|PML body|kinase binding|pr
lar response to glucose stimulus|clathrin-dependent endocytosis|sperm principal

lgi ribbon formation|positive regulation of opsonization

opment|rhombomere 6 development|abducens nerve formation|T cell differentiation

beta stimulus|positive regulation of mesenchymal stem cell differentiation

reonine transmembrane transporter activity|symporter activity|L-alanine transpo

element binding|wound healing|negative regulation of transcription, DNA-templat

tarvation|cellular response to hydrogen peroxide|positive regulation of transla

ctin filament capping|actin filament bundle assembly|transmembrane transport|pr

ing|chaperone cofactor-dependent protein refolding|misfolded protein binding|ex

ge|cell redox homeostasis|extracellular exosome|tertiary granule lumen

se|Phototransduction - fly|Retrograde endocannabinoid signaling|Vascular smooth
absence of ligand

regulation of interleukin-1 beta secretion|positive regulation of NF-kappaB tra

subunit biogenesis|regulation of signal transduction by p53 class mediator|pro

tivity|negative regulation of tyrosine phosphorylation of STAT protein|protein

ulation of Notch signaling pathway|negative regulation of transcription, DNA-te

se II-specific|DNA binding|nucleus|transcription factor complex|regulation of t

|inositol-1-diphosphate-2,3,4,5,6-pentakisphosphate diphosphatase activity|inos

tion|cell junction|positive regulation of transcription by RNA polymerase II|sy

ription factor binding|eukaryotic initiation factor 4E binding|cell proliferati

ic potentiation

factor complex|transforming growth factor beta receptor signaling pathway|prot

ptidase activity

eukin-12 production|negative regulation of interleukin-6 production|negative re
trix organization|establishment of planar polarity of embryonic epithelium|sequ

evelopment|forebrain neuron development|cerebellar granule cell precursor proli

sion

nuclear matrix|commitment of neuronal cell to specific neuron type in forebrain

gative regulation of transcription, DNA-templated|negative regulation of histon

jection|regulation of mRNA stability|intermediate filament organization|phagocy
duction|protein homodimerization activity|cadherin binding|negative regulation

 of apoptotic process|sarcomere organization|metal ion binding|regulation of sm

ate adhesion-dependent cell spreading|protein tyrosine kinase binding
spine neck|positive regulation of protein kinase activity|actin filament bindin

n|cAMP signaling pathway|Vascular smooth muscle contraction|Calcium signaling pa

activity|nucleus|nucleoplasm|cytoplasm|gonadal mesoderm development|heart deve

ptotic process|negative regulation of immunoglobulin secretion|endoribonuclease

a homologous recombination|positive regulation of neuron projection development

ar space|nucleus|cytoplasm|peptide metabolic process|muscle organ development|z

ng|negative regulation of toll-like receptor signaling pathway|positive regulat

sement membrane organization|regulation of neuron migration

egulation of transcription by RNA polymerase II|lipid metabolic process|signal
gamma-tubulin binding|cilium organization|positive regulation of cilium assembl

ous system neuron differentiation|neurogenesis|neuromuscular junction|node of R

ion|positive regulation of fat cell differentiation|skeletal system morphogenes

e transporter activity|ceramide 1-phosphate transport

ne adhesion molecules

y RNA polymerase II|histone methyltransferase activity (H3-K36 specific)|positi

cytosol by sarcoplasmic reticulum|membrane|neuronal action potential propagatio

ellular membrane-bounded organelle|synapse|recycling endosome|regulation of res

al growth factor receptor signaling pathway|extracellular matrix|negative regul

port vesicle membrane|membrane|viral process|secretory granule membrane|phagocy

pG binding|nuclear body|protein kinase binding|protein homodimerization activit

gative regulation of cytokine secretion

gulation of canonical Wnt signaling pathway

activity|axon terminus|axonal growth cone|chemorepellent activity|embryonic mor
oplasm|defense response to virus|type I interferon signaling pathway|membrane f

rase II|embryo implantation|SWI/SNF complex|nuclear receptor binding|stem cell

l adhesion molecule binding|cardiac muscle cell proliferation|cardiac cell fate

|metal ion binding|regulation of small GTPase mediated signal transduction|extr

n process|central nervous system development|transcription factor binding|zinc

by RNA polymerase II|metal ion binding|defense response to virus

gnaling pathway|B cell receptor signaling pathway|ErbB signaling pathway|mTOR s
ctivation|regulation of macrophage activation|regulation of inflammatory respon
te catabolic process|lysosomal lumen|positive regulation of transcription by RN

lation of vascular endothelial growth factor receptor signaling pathway|ubiquit

etina development in camera-type eye

n involved in cellular response to glucose stimulus

ation of ER to Golgi vesicle-mediated transport|toxin transport|negative regula

yte cell fate specification|positive regulation of chondrocyte differentiation|

egulation of interleukin-5 production|response to silicon dioxide|response to c
membrane-bounded organelle|membrane raft|extracellular exosome|glomerular visce

on

rgic synapse|postsynaptic cytosol

f hippo signaling|intracellular signal transduction|ciliary basal body|negative

deacetylase binding|protein-containing complex binding|negative regulation of

omoter involved in spinal cord motor neuron fate specification|regulation of tr

otein binding|positive regulation of cartilage development

e|cell-cell adhesion
stone H4-K12 acetylation|transcription regulatory region DNA binding|negative r

|positive regulation of neurogenesis|negative regulation of intrinsic apoptotic

ss|metal ion binding|regulation of mitochondrial membrane potential|oxidation-r

metanephros morphogenesis|DNA-binding transcription factor activity|nucleus|tr
r signaling pathway|lysosomal lumen|laminin binding|membrane raft|synapse|ancho

regulatory subunit binding|modulation by host of viral RNA genome replication

t/right pattern formation|left/right pattern formation|coronary vasculature dev
oupling|positive regulation of cell communication by chemical coupling|intercal

|positive regulation of cell proliferation|proximal/distal pattern formation|ce

ion|cellular protein metabolic process|decidualization|regulation of hormone bi

steoblast differentiation|transforming growth factor beta binding|negative regu

L-ornithine transmembrane transport

mbryonic limb morphogenesis|histone deacetylase binding|positive regulation of

activity|response to hydrogen peroxide|cadherin binding|protein autophosphoryla

elopment|positive regulation of cold-induced thermogenesis

on of transcription, DNA-templated|metal ion binding

iption, DNA-templated|multicellular organism development|anterior/posterior pat

g|mRNA CDS binding|ribonucleoprotein complex

involved in apoptotic process|negative regulation of apoptotic process|collagen

tube branching involved in lung morphogenesis|hedgehog family protein binding

ion of cell migration|collagen catabolic process|motile cilium|tissue regenerat

n cytoskeleton reorganization|activation of protein kinase activity|postsynapti

p38MAPK cascade|positive regulation of NIK/NF-kappaB signaling|regulation of pr

synthase activity

saccharide

lation of cellular component biogenesis|negative regulation of transcription, D

cytoplasm|phosphatidylinositol-4-phosphate binding|perinuclear endoplasmic reti

RNA polymerase II|metal ion binding

nase activity|oocyte development|spindle midzone|mitotic spindle midzone assemb

gen into both donors|neuron projection development|oxidative DNA demethylase ac

flammasome complex|positive regulation of NLRP3 inflammasome complex assembly

ion|early endosome to recycling endosome transport|extracellular exosome|apical

anization|vestibulocochlear nerve structural organization|nerve development|gon

oupling|positive regulation of cell communication by chemical coupling|intercal

fic|blood vessel development|outflow tract morphogenesis|atrioventricular valve

nal side of Golgi membrane|regulation of protein targeting

tion|left/right pattern formation|ciliary tip|non-motile cilium assembly

pathway|Cholinergic synapse|Jak-STAT signaling pathway|Phospholipase D signaling
onse to exogenous dsRNA

of membrane|regulation of actin cytoskeleton organization|response to interfer

n|cell-cell adhesion|ficolin-1-rich granule membrane|negative regulation of mRN

vesicle|cell differentiation|cytoplasmic vesicle membrane|ubiquitin protein li

G protein-coupled receptor signaling pathway

nvolved in apoptotic process|mitochondrial membrane organization|ferrous iron b

n of Rho guanyl-nucleotide exchange factor activity

lcium ion concentration|uniplex complex

plex|Rho GTPase binding|antigen processing and presentation of exogenous peptid

|positive regulation of osteoblast differentiation|ATP metabolic process|regula

iation|positive regulation of ERK1 and ERK2 cascade|regulation of astrocyte che

d telomeric DNA binding|positive regulation of helicase activity|positive regul

ess|positive regulation of I-kappaB kinase/NF-kappaB signaling|receptor complex

olarization during action potential|regulation of sodium ion transmembrane tran

erization|epithelial cell differentiation|positive regulation of actin filament

tion|cell division

ss|response to oxidative stress|mitochondrion organization|regulation of hydrog

response|positive regulation of chemokine secretion

nus|regulated exocytosis|behavioral response to ethanol|corticotropin-releasing

l lumen|tissue remodeling|positive regulation of peptidyl-tyrosine phosphorylat

itive regulation of phagocytosis|collagen-containing extracellular matrix|extra

motaxis|negative regulation of peptidyl-serine dephosphorylation

ding involved in bundle of His cell-Purkinje myocyte communication|regulation o

cytoplasm|transcription initiation from RNA polymerase II promoter|cell-cell si

er cell mediated cytotoxicity|Carbohydrate digestion and absorption|Aldosterone-

activity|adherens junction maintenance|ion channel binding|intermediate filame

egulation of response to cytokine stimulus|repressing transcription factor bind

tiation

c cell adhesion

ependent protein catabolic process|positive regulation of cytokinesis|protein h

 organelle|positive regulation of filopodium assembly|synapse maturation|negati

elopment|rhombomere 4 development|facial nerve structural organization|sequence

lum-Golgi intermediate compartment membrane|identical protein binding|cadherin

l nerve morphogenesis|thyroid gland development|positive regulation of transcri

ll differentiation|endopeptidase activator activity|positive regulation of coll

ter|Ras protein signal transduction|embryo implantation|zinc ion binding|viral

tase activity|cellular response to drug|peptidyl-threonine dephosphorylation|po

cription factor binding|visual learning|response to mechanical stimulus|pancrea

o rapamycin|response to amino acid starvation

e to cytokine stimulus|positive regulation of homophilic cell adhesion|positive

TR-mediated mRNA destabilization|pre-mRNA intronic binding|positive regulation

utamatergic synapse|modification of postsynaptic structure|positive regulation

trans-Golgi network membrane|protein localization to Golgi apparatus|early endo

ve regulation of release of sequestered calcium ion into cytosol|positive regul

crine cell differentiation|mitotic spindle assembly|mitotic spindle pole

n|ubiquitin binding|post-translational protein modification|negative regulation

ence-specific binding|RNA binding|nucleus|DNA replication factor A complex|DNA

fication process|respiratory gaseous exchange|anterior/posterior pattern specif

feration|protein-containing complex assembly|regulation of female gonad develop

rotein amidation|protein kinase binding|ovulation cycle process|secretory granu

fferentiation|protein heterodimerization activity

web

ure maintenance|intercalated disc|basolateral plasma membrane|apicolateral plas

odeling|intracellular membrane-bounded organelle|fat cell differentiation|posit
ort|retrograde neuronal dense core vesicle transport

gulation of interleukin-6 production|positive regulation of interleukin-8 produ

loride channel complex|positive regulation of insulin secretion involved in cel

regulation of RIG-I signaling pathway

polymerase II|transcription by RNA polymerase II|germ cell development|visual

ine kinase activity

RNA polymerase II-specific|RNA polymerase II repressing transcription factor b

tivity|phospholipase A2 activity (consuming 1,2-dipalmitoylphosphatidylcholine)

egulation of cell migration|negative regulation of cell migration|establishment

lar membrane-bounded organelle|protein-containing complex binding|intercellular

rase II|promoter-specific chromatin binding

|PML body|identical protein binding|protein homodimerization activity|intracell

actor receptor signaling pathway|response to calcium ion|negative regulation of

ıapse|Oocyte meiosis|Retrograde endocannabinoid signaling|Calcium signaling patl

cell body|intracellular membrane-bounded organelle|type B pancreatic cell proli
n|osteoclast differentiation|iron-responsive element binding|translation repres

e|negative regulation of MAPK cascade|positive regulation of DNA damage respons

dation-reduction process|FAD binding

|axon terminus|cell-cell adhesion mediated by cadherin|cadherin binding|regulat

monoubiquitination|site of double-strand break|interstrand cross-link repair|hi

is|manganese ion binding|actin cytoskeleton reorganization|Golgi calcium ion ho

gative regulation of G0 to G1 transition|site of DNA damage|histone methyltrans

polymerization|midbody|negative regulation of cysteine-type endopeptidase activ

on|calcium ion import|uniplex complex

le assembly

gulation of cellular senescence

ve regulation of delayed rectifier potassium channel activity

ung alveolus development|skeletal muscle fiber development|calcium ion homeosta

brane|positive regulation of actin filament polymerization|actin-dependent ATPa

tion of aldosterone secretion

eration

dent endocytosis

egative regulation of DNA-binding transcription factor activity|negative regula

anded DNA binding
tin filament capping|photoreceptor disc membrane

ription activator activity, RNA polymerase II-specific|distal enhancer DNA-bind

is|cellular response to cytokine stimulus|cellular response to interleukin-6|ce

ell differentiation|dioxygenase activity|cardiac muscle cell differentiation|re

t ERAD pathway|Hsp70 protein binding|polyubiquitin modification-dependent prote

|negative regulation of intrinsic apoptotic signaling pathway in response to DN
paB transcription factor activity|interferon-gamma-mediated signaling pathway|p

lanine metabolism
athway

n of protein transport

evelopment|forebrain neuron development|cerebellar granule cell precursor proli

heart morphogenesis|AT DNA binding|transcription coactivator activity|nucleus|n

sitive regulation of protein targeting to membrane|positive regulation of gluco

f gene expression|aggresome|nuclear body|glossopharyngeal nerve morphogenesis|t

ain neuron fate commitment|cerebral cortex GABAergic interneuron differentiatio

of mitochondrial outer membrane|nuclear membrane|sarcoplasmic reticulum membra

sduction|presynapse|regulation of protein catabolic process at presynapse, modu
on by RNA polymerase II|positive regulation of histone H3-K4 methylation|histon

iation|protein homooligomerization|extracellular exosome

nt DNA replication|protein localization to chromatin|MCM8-MCM9 complex

action potential|negative regulation of peptidyl-cysteine S-nitrosylation|regu
on of protein kinase activity|response to hydrogen peroxide|identical protein b

cy|replication fork processing|gene conversion|interstrand cross-link repair|po

ription by RNA polymerase II|transcription by RNA polymerase II|spinal cord mot
al transduction|tau-protein kinase activity|regulation of dendrite development|

ted vesicle|COPI-coated vesicle membrane|somite rostral/caudal axis specificati

eukin-12 production|negative regulation of interleukin-6 production|negative re

ntiation|negative regulation of bone resorption|phosphoprotein binding|regulati

xon|ion channel binding|myoblast differentiation|oxidation-reduction process|di
evelopment|embryonic digestive tract development|roof of mouth development

itol binding|cytoplasmic ribonucleoprotein granule|cell body|phosphatidic acid
th factor beta receptor signaling pathway|cell-cell signaling|circadian rhythm|

ein transport|negative regulation of apoptotic process|intracellular membrane-b

in-TCF complex

telet activation|filopodium|lung development|positive regulation of cell migrat

ell lineage commitment|outflow tract morphogenesis|atrioventricular valve morph

unction|response to estradiol|cellular response to fibroblast growth factor sti

emopoiesis|neuron differentiation|intracellular membrane-bounded organelle|nega
r granular layer morphogenesis|negative regulation of blood coagulation|negativ

euron differentiation|positive regulation of canonical Wnt signaling pathway

s of number of cells|negative regulation of intrinsic apoptotic signaling pathw

pse|integral component of presynaptic active zone membrane|integral component o

rase II promoter|signal transduction|zinc ion binding|entrainment of circadian

[illegible] of TRP channels|Olfactory transduction|Long-term potentiation|ErbB signaling [illegible]

l ion binding|regulation of small GTPase mediated signal transduction

cess|positive regulation of DNA repair|positive regulation of synapse assembly|

tive regulation of synaptic transmission|postsynapse|glutamatergic synapse|pres

ss|glomerular basement membrane development|glial cell-derived neurotrophic fac

tin cytoskeleton reorganization

inoid signaling|Tight junction|cGMP-PKG signaling pathway|Gastric acid secretion

ion of NIK/NF-kappaB signaling|regulation of cell cycle G1/S phase transition|r

ılar smooth muscle contraction|Calcium signaling pathway|GnRH signaling pathway

|sarcolemma|lysosomal lumen|extracellular matrix binding|collagen-containing ex

in-coated vesicle membrane|cytoplasmic vesicle|filamentous actin|nuclear membra

ediated signaling pathway|extracellular matrix disassembly|peptidase inhibitor

nt bundle assembly|alpha-catenin binding|cadherin binding|maintenance of animal

gic synapse|presynaptic cytosol|postsynaptic cytosol

ocytosis
g pathway

lgi localization|dynein light intermediate chain binding|FHF complex|protein lo

e binding|positive regulation of apoptotic process|positive regulation of DNA d

ion|collagen-containing extracellular matrix|extracellular exosome

regulation of neuron projection development|sequence-specific DNA binding|reti

tion of microtubule polymerization|microtubule plus-end|gamma-tubulin binding|t

activity|intracellular signal transduction|establishment or maintenance of epit

ound healing, spreading of cells|metal ion binding|smooth muscle cell-matrix ad

gulation of tumor necrosis factor production|intracellular signal transduction|

or activity|response to glucocorticoid|extracellular exosome|response to growth

le|cellular response to cytokine stimulus

G2/M transition of mitotic cell cycle|regulation of ossification|adrenal gland

in binding

t|positive regulation of behavior|digestive tract morphogenesis|embryonic diges

ing|membrane|cell differentiation|negative regulation of apoptotic process|rece

sitive regulation of urine volume|peptide binding|hormone binding|protein homod

l entry into host cell|virion binding|positive regulation of phagocytosis|negat

vasculogenesis|muscular septum morphogenesis|outflow tract morphogenesis|pulmon

ne|neuron projection|synapse|cell adhesion molecule binding|positive regulation

al nucleoid|ADP binding|sequence-specific DNA binding|chaperone-mediated protei

fferentiation|positive regulation of type I interferon production|positive regu

vesicle-mediated transport in synapse|integral component of postsynaptic densit

n|Chemokine signaling pathway|Estrogen signaling pathway|Serotonergic synapse|O

hway|bud elongation involved in lung branching|epithelial to mesenchymal transi

53 class mediator|negative regulation of G1/S transition of mitotic cell cycle

tination

4-3-3 protein binding|positive regulation of cell migration involved in sprouti
all GTPase mediated signal transduction|positive regulation of protein targetin

ivation of protein kinase activity|positive regulation of ion transmembrane tra

ation|negative regulation of inflammatory response|positive regulation of phago

icle binding|ADP-ribosylation factor binding|Golgi cisterna|negative regulation
 toll-like receptor signaling pathway|intestinal stem cell homeostasis|innate i

apparatus assembly

r response to ionizing radiation|ankyrin repeat binding

of Wnt signaling pathway|forebrain development|middle ear morphogenesis|muscle

hway|GnRH signaling pathway|Taste transduction|cGMP-PKG signaling pathway|Gastr

mplex|cell migration|protein kinase binding|protein phosphatase binding|Z disc|

velopment|positive regulation of vesicle fusion|positive regulation of chondroc

ated|positive regulation of transcription, DNA-templated|positive regulation of
etrograde endocannabinoid signaling|Phospholipase D signaling pathway|GnRH signa

timulus|apical dendrite|positive regulation of neuron migration
ation of keratinocyte migration|negative regulation of wound healing|cell perip

n|membrane|RISC complex|cell junction|production of siRNA involved in RNA inter

fferentiation|glomerulus development|histone deacetylase binding|bHLH transcrip

MP in extracellular matrix|sequestering of TGFbeta in extracellular matrix|gluc

signaling pathway|Inflammatory mediator regulation of TRP channels|Apoptosis|Pa

androgen receptor signaling pathway

scle fiber development|positive regulation of dendrite morphogenesis|actin fila

r activity, RNA polymerase II-specific|DNA-binding transcription factor activit

|negative regulation of protein K63-linked ubiquitination

ell cycle|centrosome localization|cytoskeletal anchoring at nuclear membrane|nu

ding|cell division|negative regulation of focal adhesion assembly|positive regu

ry response|positive regulation of macrophage cytokine production|positive regu
ion transmembrane transport|cellular response to oxidative stress|neuron projec

nse to oxidative stress|response to nicotine|locomotory exploration behavior|ne

itol binding|embryonic digit morphogenesis|protein-containing complex binding|n

differentiation|nuclear body|PML body|transcriptional repressor complex|protei

c density membrane|postsynaptic endosome|glutamatergic synapse|regulation of po

ignaling pathway|negative regulation of astrocyte differentiation|ciliary membr

y|endothelial cell migration|regulation of angiogenesis|positive regulation of

ntiation|endothelial cell-matrix adhesion|negative regulation of sprouting angi

triated muscle cell differentiation|positive regulation of protein metabolic pr

racellular exosome

icle|protein localization to membrane|phagosome maturation|melanosome assembly

ess|positive regulation of glial cell proliferation|UFM1 transferase activity|p
artilage development|positive regulation of protein kinase B signaling|extracel

deling|positive regulation of transcription, DNA-templated|positive regulation

gene expression|sequence-specific DNA binding|transcription regulatory region
geminal nerve development|anterior commissure morphogenesis|lung development|fo
eoplasm|transcription factor binding|anterior/posterior pattern specification|n

olved in forebrain neuron fate commitment|cerebral cortex GABAergic interneuron

organizing center

gulation of tumor necrosis factor production|intracellular signal transduction|

cription by RNA polymerase II|GTPase binding|response to cAMP|positive regulati

n of canonical Wnt signaling pathway|establishment of epithelial cell polarity|

l tract morphogenesis|positive regulation of Wnt signaling pathway|positive reg

otein domain specific binding|response to retinoic acid|negative regulation of
 piece|positive regulation of osteoclast development

in thymus|brain segmentation|inner ear morphogenesis|protein homodimerization

rt|L-cystine transport|proline transport|L-serine transport|threonine transport

ed|positive regulation of transcription by RNA polymerase II

tional initiation in response to starvation|cellular response to acidic pH|cell

otein-containing complex assembly

tracellular exosome

muscle contraction|Phospholipase D signaling pathway|GnRH signaling pathway|Th

nscription factor activity|cellular response to lipopolysaccharide|cellular res

tein localization to nucleolus

homodimerization activity|neuronal cell body|phosphatidylinositol-4-phosphate p

mplated|positive regulation of transcription, DNA-templated|positive regulation

ranscription by RNA polymerase II|PcG protein complex|sequence-specific DNA bin

itol-3-diphosphate-1,2,4,5,6-pentakisphosphate diphosphatase activity|inositol-

napse maturation|ureter development

on|DNA binding, bending|response to wounding|anterior/posterior pattern specifi

ein domain specific binding|activin responsive factor complex|negative regulati

gulation of tumor necrosis factor production|intracellular signal transduction|
ence-specific DNA binding|negative regulation of transcription, DNA-templated|p

feration|inner ear morphogenesis|autonomic nervous system development

|sequence-specific DNA binding|embryonic skeletal system morphogenesis|cartilag

e H3-K4 methylation|roof of mouth development|specification of axis polarity|ne

tic vesicle|positive regulation of translation|Bergmann glial cell differentiat
of transcription, DNA-templated|positive regulation of NF-kappaB transcription

all GTPase mediated signal transduction

g|GTPase binding|regulation of filopodium assembly|negative regulation of stres

athway|GnRH signaling pathway|Pancreatic secretion|Gastric acid secretion|Serot[illegible]

lopment|blood coagulation|transcription factor binding|zinc ion binding|lung de

inhibitor activity|negative regulation of endoribonuclease activity|intrinsic

|oxidoreductase activity|enzyme binding|cerebral cortex development|nuclear rec

inc ion binding|protein processing|collagen-containing extracellular matrix|ext

ion of peroxisome proliferator activated receptor signaling pathway|regulation

transduction|skeletal muscle tissue development|zinc ion binding|fertilization|
y|regulation of centrosome cycle|tau-protein kinase activity|cell division|posi

anvier|extracellular matrix binding

is|positive regulation of stress fiber assembly|bone development|regulation of

ve regulation of muscle cell differentiation

n|calcium channel inhibitor activity|protein maturation by protein folding|Z di

ting membrane potential|cardiac conduction|potassium ion transmembrane transpor

ation of collagen biosynthetic process|integrin alpha4-beta1 complex|identical

tic vesicle membrane|early endosome membrane|regulation of interleukin-12 produ

y|sequence-specific DNA binding|negative regulation of transcription, DNA-templ

phogenesis|response to axon injury|positive regulation of synapse assembly|head
usion

population maintenance|intracellular estrogen receptor signaling pathway|androg

specification|self proteolysis|positive regulation of gastrulation

acellular exosome

ion binding|cell proliferation|negative regulation of cell proliferation|regula

ignaling pathway
se|L-arginine transmembrane transporter activity|L-arginine import across plasm
A polymerase II|cartilage development|collagen-containing extracellular matrix|

in protein ligase binding|locomotion involved in locomotory behavior|substrate

tion of autophagosome assembly

cell fate commitment|negative regulation of transcription, DNA-templated|positi

ytokine|negative regulation of T cell proliferation|response to drug|regulation
ral epithelial cell development|epithelial tube formation

regulation of T cell proliferation|regulation of apoptotic process|apical prot

transcription, DNA-templated|protein homooligomerization|negative regulation of

anscription from RNA polymerase II promoter involved in ventral spinal cord int

egulation of transcription, DNA-templated|positive regulation of transcription

signaling pathway in response to DNA damage by p53 class mediator|positive reg

eduction process|beta,beta-carotene-9',10'-cleaving oxygenase activity|regulati

anscription factor complex|protein import into nucleus|mesodermal cell fate spe
red component of plasma membrane|leukocyte migration|collagen-containing extrac

elopment|determination of heart left/right asymmetry|extracellular exosome|dete
ated disc|gap junction assembly|embryonic limb morphogenesis|embryonic heart tu

ll surface|regulation of epithelial to mesenchymal transition|morphogen activit

osynthetic process|perinuclear region of cytoplasm|cellular response to hypoxia

lation of CD4-positive, alpha-beta T cell activation|negative regulation of CD4

transcription by RNA polymerase II|uterus development

tion|metal ion binding|regulation of axon regeneration|neuron projection morpho

tern specification|nuclear body|myeloid cell differentiation|positive regulatio

-containing extracellular matrix|extracellular exosome|cellular response to est

ion|innate immune response|metal ion binding|cardiac epithelial to mesenchymal

c membrane|positive regulation of JNK cascade|protein autophosphorylation|neuro

otein autoubiquitination

NA-templated|positive regulation of transcription by RNA polymerase II

culum

ly|cell division|meiotic cell cycle

tivity|chemoattractant activity|RNA repair|negative regulation of neuron apopto

dendrite

adotrophin-releasing hormone neuronal migration to the hypothalamus|axon|ventra

ated disc|gap junction assembly|embryonic limb morphogenesis|embryonic heart tu

morphogenesis|pulmonary valve morphogenesis|epithelial to mesenchymal transiti

g pathway|Regulation of actin cytoskeleton|VEGF signaling pathway|Insulin signa

on-gamma|response to interferon-alpha|response to interferon-beta|azurophil gra

A catabolic process|messenger ribonucleoprotein complex

gase binding|peptidyl-tyrosine autophosphorylation|regulation of cell prolifera

inding|zinc ion binding|adult locomotory behavior|response to iron(II) ion|regu

e antigen via MHC class II|midbody|positive regulation of cytokinesis|intracili

tion of phosphorus metabolic process|extracellular exosome|enamel mineralizatio

motaxis

ation of telomerase activity|negative regulation of telomerase activity|positiv

|axon terminus|inner ear development|positive regulation of NF-kappaB transcrip

sporter activity

bundle assembly|actin filament bundle|filopodium tip|regulation of microvillus

en peroxide metabolic process|negative regulation of gene expression|regulation

hormone binding|regulation of corticotropin secretion|negative regulation of c

ion|collagen-containing extracellular matrix|extracellular exosome

cellular exosome|extracellular vesicle

f heart rate by cardiac conduction|regulation of ventricular cardiac muscle cel

gnaling|pattern specification process|heart development|transcription factor bi

-regulated sodium reabsorption|TNF signaling pathway|Osteoclast differentiation

nt bundle assembly|alpha-catenin binding|cadherin binding|maintenance of animal

ing|response to interleukin-1|negative regulation of canonical Wnt signaling pa

omodimerization activity|dendritic spine|dendritic shaft|endothelial cell migra

ve regulation of dopamine receptor signaling pathway|cellular response to elect

-specific DNA binding|positive regulation of transcription by RNA polymerase II

binding|COPII vesicle coating|spindle assembly|protein homotetramerization|posi

ption by RNA polymerase II|embryonic skeletal system morphogenesis|regulation o

agen catabolic process|negative regulation of CD4-positive, alpha-beta T cell p

process|protein sumoylation|SUMO transferase activity|ubiquitin protein ligase

sitive regulation of cysteine-type endopeptidase activity involved in apoptotic

s development|sequence-specific DNA binding|negative regulation of myeloid cell

regulation of adherens junction organization|positive regulation of early endo

of skeletal muscle fiber differentiation|positive regulation of 3'-UTR-mediated

of long-term synaptic potentiation

some to Golgi transport|retrograde transport, endosome to Golgi|neutrophil degr

ation of protein targeting to membrane|positive regulation of protein kinase C

of lymphocyte differentiation|negative regulation of cyclin-dependent protein

unwinding involved in DNA replication|DNA replication initiation|nervous system

ication|positive regulation of receptor biosynthetic process|cell migration|neg

ment

le membrane|L-ascorbic acid binding|response to estradiol|regulation of actin c

ma membrane|cell junction|PDZ domain binding|filopodium|growth cone|neutrophil

ive regulation of glucose import|positive regulation of insulin receptor signal

ction|toll-like receptor 9 signaling pathway|defense response to bacterium|iden

lular response to glucose stimulus|protein dimerization activity|detection of t

learning|anatomical structure morphogenesis|regulation of mammary gland epithel

inding|enhancer sequence-specific DNA binding|DNA binding|DNA-binding transcrip

|phospholipase A2 activity consuming 1,2-dioleoylphosphatidylethanolamine)

or maintenance of actin cytoskeleton polarity|late endosome membrane|cell divi

bridge|cilium assembly|protein localization to centrosome|positive regulation

ular membrane-bounded organelle|sequence-specific DNA binding

cell division|cellular response to tumor cell

hway|GnRH signaling pathway|Pancreatic secretion|cGMP-PKG signaling pathway|Gas

feration|positive regulation of Notch signaling pathway|negative regulation of
sor activity|axon|response to retinoic acid|erythrocyte homeostasis|response to

e, signal transduction by p53 class mediator|vasculogenesis involved in coronar

ion of synapse organization|synaptic membrane|cell-cell adhesion|glutamatergic

stone H2A-K13 ubiquitination|histone H2A-K15 ubiquitination|histone binding|ubi

meostasis|Golgi calcium ion transport|positive regulation of I-kappaB kinase/NF

ferase binding

ity involved in apoptotic process|intercellular bridge|metal ion binding|oxidat

sis|regulation of ryanodine-sensitive calcium-release channel activity|cellular

se activity|filamentous actin|ruffle membrane|positive regulation of cell migra

tion of transcription, DNA-templated|neuromuscular process controlling balance

ing transcription repressor activity, RNA polymerase II-specific|DNA-binding tr

llular response to tumor necrosis factor|cellular response to fatty acid|negati

sponse to fungicide|cellular response to hydrogen peroxide|histone H3-K27 demet

in binding|ubiquitin protein ligase binding|negative regulation of proteasomal

A damage by p53 class mediator|positive regulation of protein polyubiquitinatio
ositive regulation of protein localization to nucleus|negative regulation of vi

feration|inner ear morphogenesis|autonomic nervous system development

ucleoplasm|transcription factor complex|regulation of transcription, DNA-templa

se import in response to insulin stimulus

hyroid gland development|positive regulation of neuron differentiation|positive

n|cerebral cortex GABAergic interneuron fate commitment|cell differentiation|ne

ne|intracellular signal transduction|metal ion binding|regulation of cardiac co

lating synaptic transmission
e H3-K4 trimethylation|RNA polymerase II C-terminal domain phosphoserine bindin

lation of sodium ion transmembrane transport
inding|protein homodimerization activity|regulation of apoptotic process|protei

sitive regulation by host of viral transcription|positive regulation by host of

or neuron differentiation|small-subunit processome assembly
establishment of mitochondrion localization|phosphatidic acid binding

on|Golgi cisterna membrane|endoplasmic reticulum-Golgi intermediate compartment

gulation of tumor necrosis factor production|intracellular signal transduction|

on of release of sequestered calcium ion into cytosol|platelet aggregation|nega

aphragm development|NADPH binding|regulation of delayed rectifier potassium cha

binding
cell proliferation|negative regulation of cell proliferation|cellular response

ounded organelle|protein retention in Golgi apparatus|Golgi vesicle budding|pos

ion|lamellipodium membrane|cytoplasmic vesicle|filopodium membrane|microvillus

ogenesis|mitral valve formation|tricuspid valve formation|nucleus|nucleoplasm|t

mulus|metal ion binding|cell adhesion molecule binding|collagen-containing extr

tive regulation of CD4-positive, alpha-beta T cell differentiation|positive reg
e regulation of cell growth|regulation of cell migration|platelet alpha granule

ay by p53 class mediator|regulation of DNA-templated transcription, initiation

f postsynaptic specialization membrane

clock by photoperiod|sequence-specific DNA binding|neuron development|detection

pathway|Oxytocin signaling pathway

ubiquitin conjugating enzyme activity|extracellular exosome|protein K63-linked

ynaptic cytosol|postsynaptic cytosol|voltage-gated calcium channel activity inv

tor receptor signaling pathway|negative regulation of fibroblast growth factor

n|Chemokine signaling pathway|Estrogen signaling pathway|Serotonergic synapse|O

egulation of hemopoiesis

|Pancreatic secretion|cGMP-PKG signaling pathway|Gastric acid secretion|Estroge

tracellular matrix|extracellular exosome

ne|ruffle membrane|response to drug|identical protein binding|ADP binding|apica

activity|extracellular matrix|platelet alpha granule lumen|response to cytokine

organ identity|ventricular cardiac muscle tissue morphogenesis|cornification|p

calization to centrosome|neuronal stem cell population maintenance

amage response, signal transduction by p53 class mediator|sarcomere organizatio

noic acid-responsive element binding|positive regulation of transcription by RN

ranscription regulatory region DNA binding|regulation of neuron differentiation

helial cell apical/basal polarity|cadherin binding|protein autophosphorylation|

hesion|positive regulation of ERK1 and ERK2 cascade

phosphatidylinositol 3-kinase regulatory subunit binding|T cell activation|nega

factor

development|embryonic limb morphogenesis|embryonic hemopoiesis|regulation of ce

tive tract development|developmental growth|smooth muscle tissue development|ar

ptor complex|positive regulation of ERK1 and ERK2 cascade|cellular response to

imerization activity|phosphatidylinositol-mediated signaling|negative regulatio

ive regulation of exo-alpha-sialidase activity|negative regulation of glycoprot

ary valve morphogenesis|tricuspid valve morphogenesis|tricuspid valve formation

. of synapse assembly|positive regulation of synapse maturation

n complex assembly|protein homooligomerization|proteolysis involved in cellular

.lation of interleukin-1 beta production|regulation of gene expression, epigenet

y membrane|integral component of spine apparatus membrane

xytocin signaling pathway

tion involved in cardiac fibroblast development|negative regulation of ERK1 and

ng angiogenesis|negative regulation of NIK/NF-kappaB signaling
g to mitochondrion

nsporter activity|intracellular signal transduction|maintenance of lens transpa

cytosis|modulation of chemical synaptic transmission|neuromuscular process cont

of protein binding|negative regulation of protein oligomerization|protein loca
mmune response|negative regulation of transcription, DNA-templated|positive reg

organ morphogenesis|embryonic skeletal system morphogenesis

ic acid secretion|Chemokine signaling pathway|Vascular smooth muscle contractio

desmosome|keratinization|protein-DNA complex|adherens junction organization|nuc

yte differentiation|negative regulation of low-density lipoprotein particle rec

' transcription by RNA polymerase II|metal ion binding|regulation of transcripti
aling pathway|Taste transduction|cGMP-PKG signaling pathway|Gastric acid secret

hery|perinucleolar compartment|regulation of epithelium regeneration|keratin fi

ference|dendrite|gene silencing by RNA|pre-miRNA processing|micro-ribonucleopro

tion factor binding|positive regulation of transcription by RNA polymerase II|p

ose homeostasis|identical protein binding|camera-type eye development|post-tran

ancreatic secretion|Long-term potentiation|Serotonergic synapse|Oxytocin signal

ment binding|muscle alpha-actinin binding|cardiac myofibril assembly|cardiac mu

y|transcription coregulator activity|nucleus|cytoplasm|endoplasmic reticulum me

clear matrix anchoring at nuclear membrane

lation of attachment of spindle microtubules to kinetochore|chromosome passenge

lation of protein tyrosine kinase activity|extracellular exosome|positive regul
tion|neuronal cell body|lysosomal lumen|protein autophosphorylation|positive re

uronal cell body|perikaryon|response to morphine|axon terminus|post-translation

egative regulation of smoothened signaling pathway|embryonic neurocranium morph

n kinase binding|protein domain specific binding|olfactory bulb development|mye

stsynaptic neurotransmitter receptor internalization|vesicle-mediated intercell

ane|negative regulation of hydrogen peroxide-induced cell death

axonogenesis|dichotomous subdivision of terminal units involved in salivary gla

ogenesis

ocess|cellular response to mechanical stimulus|regulation of barbed-end actin f

rotein ufmylation|response to L-glutamate|protein K69-linked ufmylation
lular exosome|positive regulation of ERK1 and ERK2 cascade|response to interleu

of transcription by RNA polymerase II|protein N-terminus binding|npBAF complex|

DNA binding|negative regulation of transcription, DNA-templated|positive regula
rebrain development|hindbrain development|negative regulation of DNA binding|ce
uclear membrane|sequence-specific DNA binding|negative regulation of keratinocy

fate commitment|odontogenesis of dentin-containing tooth|positive regulation o

phosphatidylinositol 3-kinase regulatory subunit binding|T cell activation|nega

on of type B pancreatic cell proliferation|dense core granule maturation

negative regulation of stem cell proliferation|positive regulation of transform

ulation of JUN kinase activity|sequence-specific DNA binding|positive regulatio

viral transcription|response to cytokine|response to type I interferon|response

activity|sequence-specific DNA binding|negative regulation of erythrocyte diffe

|membrane|integral component of membrane|dendrite|hydroxyproline transport|L-hy

ular response to UV-C|cellular response to benomyl|negative regulation of trans

yroid hormone signaling pathway|Pancreatic secretion|cGMP-PKG signaling pathway

ponse to hypoxia|cellular response to UV-C|caspase binding|protease inhibitor c

hosphatase activity|clathrin-coated endocytic vesicle|phosphatidylinositol deph

of skeletal muscle fiber development|extracellular exosome|negative regulation

ding|positive regulation of transcription by RNA polymerase II|metal ion bindin

5-diphosphate-1,2,3,4,6-pentakisphosphate diphosphatase activity|inositol-1,5-b

cation|negative regulation of transcription by transcription factor localizatio

on of intracellular estrogen receptor signaling pathway|embryonic heart tube an

phosphatidylinositol 3-kinase regulatory subunit binding|T cell activation|nega
ositive regulation of transcription, DNA-templated|positive regulation of trans

e development|roof of mouth development|cellular response to organic substance

gative regulation of tooth mineralization

ion|SMAD protein signal transduction|lens fiber cell development|cellular respo
factor activity

s fiber assembly|regulation of synapse structural plasticity|regulation of syna

onergic synapse|Inflammatory mediator regulation of TRP channels|Endocrine and

velopment|negative regulation of fat cell differentiation|negative regulation o

apoptotic signaling pathway in response to endoplasmic reticulum stress|respons

eptor transcription coactivator activity|negative regulation of protein binding

racellular exosome|regulation of collagen fibril organization

of fat cell differentiation|negative regulation of fat cell differentiation|pos

anterior/posterior pattern specification|radial pattern formation|negative regu
tive regulation of protein localization to centrosome

brood size|adipose tissue development|phosphatidylinositol-4-phosphate binding|

sc|insulin secretion|cyclic nucleotide binding|negative regulation of phosphopr

t|inward rectifier potassium channel complex

protein binding|elastic fiber assembly|negative regulation of cell activation|n

ction|regulation of interleukin-6 production|protection from natural killer cel

ated|metal ion binding

. development|synaptic membrane|cell-cell adhesion via plasma-membrane adhesion

en receptor signaling pathway|forebrain development|nucleosome binding|nucleoso

.tion of mesenchymal cell proliferation|regulation of transforming growth factor

a membrane|L-ornithine transmembrane transport
extracellular exosome|response to growth factor

adhesion-dependent cell spreading|extracellular matrix-cell signaling|non-canon

ve regulation of transcription by RNA polymerase II|protein heterodimerization

. of mRNA stability|regulation of inflammatory response|response to glucocortico

ein localization|zonula adherens assembly|establishment or maintenance of epith

' G1/S transition of mitotic cell cycle by negative regulation of transcription

erneuron specification|central nervous system myelination|endocrine pancreas de

by RNA polymerase II|response to sorbitol|response to hydroxyurea|response to a

ulation of neural precursor cell proliferation

on of reactive oxygen species metabolic process

cification|transcription factor binding|cell proliferation|anatomical structure
ellular matrix|extracellular exosome|positive regulation of skeletal muscle cel

rmination of digestive tract left/right asymmetry|craniofacial suture morphogen
be development|foramen ovale closure|cell projection|negative regulation of blo

y|collagen fibril organization|transmembrane receptor protein tyrosine kinase a

|regulation of store-operated calcium entry

-positive, alpha-beta T cell proliferation

genesis|extracellular exosome|execution phase of apoptosis

n of transcription by RNA polymerase II|embryonic skeletal system morphogenesis

rogen stimulus

transition|extracellular exosome|negative regulation of receptor binding|cellul

n projection morphogenesis|dendrite morphogenesis|extracellular exosome|positiv

tic process|developmental growth|dioxygenase activity|regulation of mitochondri

l trunk neural crest cell migration|signaling receptor activity|identical prote

be development|foramen ovale closure|cell projection|negative regulation of blo

on involved in endocardial cushion formation|endocardial cushion morphogenesis|

ling pathway|FoxO signaling pathway|Thyroid hormone signaling pathway|Prolactin

nule membrane|B cell activation|identical protein binding|protein homodimerizat

tion|identical protein binding|metal ion binding|perinuclear region of cytoplas

lation of phospholipase activity|negative regulation of platelet-derived growth

ary transport involved in cilium assembly|extracellular exosome|plus-end-direct

n|odontoblast differentiation|dentinogenesis

e regulation of DNA strand elongation|telomeric D-loop disassembly|telomeric D-

tion factor activity|positive regulation of ERK1 and ERK2 cascade|coreceptor ac

length|lysophosphatidic acid binding|cellular response to hepatocyte growth fa

. of mitochondrion organization|calcium-dependent protein kinase activity|positi

orticotropin secretion|cellular response to calcium ion|cellular response to co

l action potential

nding|negative regulation of cell proliferation|negative regulation of epitheli

|Adrenergic signaling in cardiomyocytes|Ras signaling pathway|PI3K-Akt signalin

organ identity|ventricular cardiac muscle tissue morphogenesis|cornification|p

thway|positive regulation of anoikis

tion|cadherin binding|regulation of synaptic plasticity|extracellular exosome|S

rical stimulus|protein localization to plasma membrane

|embryonic skeletal system morphogenesis|neural nucleus development

tive regulation of protein glycosylation|importin-alpha family protein binding|

f neurogenesis|cartilage development|definitive hemopoiesis|face development

roliferation|negative regulation of CD8-positive, alpha-beta T cell proliferati

binding|Krueppel-associated box domain binding|positive regulation of protein i

process|negative regulation of protein kinase activity by regulation of protei

differentiation|positive regulation of mitotic cell cycle|positive regulation

some to late endosome transport

mRNA stabilization|mRNA CDS binding|sequence-specific mRNA binding|ribonucleop

anulation|extracellular exosome|endosome to plasma membrane transport vesicle|m

activity

serine/threonine kinase activity|protein heterodimerization activity|glial cyto

ι development|transcription factor binding|positive regulation of cell prolifera

ative regulation of angiogenesis|lung development|thyroid gland development|reg

ytoskeleton organization|odontogenesis|response to drug|neuron projection|perik

chemotaxis|actin cytoskeleton reorganization|neuromuscular junction|protein-con

ing pathway|positive regulation of protein kinase B signaling|phosphatidylinosi

tical protein binding|negative regulation of apoptotic process|positive regulat

emperature stimulus involved in sensory perception of pain|extracellular exosom

ial cell proliferation|negative regulation of transcription, DNA-templated|posi

ption factor activity|transcription coactivator activity|transcription corepress

sion site|cleavage furrow|regulation of actin cytoskeleton organization|positiv

of intracellular protein transport|ciliary basal body-plasma membrane docking

tric acid secretion|Chemokine signaling pathway|Vascular smooth muscle contract

insulin secretion|smooth muscle cell proliferation|negative regulation of infla
copper ion|metal ion binding|4 iron, 4 sulfur cluster binding|cellular respons

y vascular morphogenesis|negative regulation of ERK1 and ERK2 cascade|negative

synapse

quitin binding|isotype switching|positive regulation of DNA repair|metal ion bi

-kappaB signaling|metal ion binding|calcium ion transmembrane transport|ATP hyd

ion-reduction process|FAD binding|regulation of regulated secretory pathway|hip

' response to caffeine|positive regulation of response to oxidative stress

.tion by vascular endothelial growth factor signaling pathway|Fc-gamma receptor

anscription factor activity|nucleus|cytoplasm|DNA repair|regulation of transcri

ve regulation of protein localization to plasma membrane|regulation of reactive

hylation|histone demethylase activity (H3-K27 specific)|positive regulation of

ubiquitin-dependent protein catabolic process|regulation of cell proliferation|

n|negative regulation of protein autoubiquitination
ral release from host cell

g

ted|heart development|adult heart development|transcription factor binding|mese

regulation of transcription by RNA polymerase II|embryonic skeletal system mor

gative regulation of BMP signaling pathway|odontogenesis of dentin-containing t

nduction

g

n autophosphorylation|perinuclear region of cytoplasm|neuron projection morphog

' viral genome replication|regulation of insulin receptor signaling pathway|posi

membrane|negative regulation of GTPase activity|multicellular organism growth|

phosphatidylinositol 3-kinase regulatory subunit binding|T cell activation|nega

tive regulation of platelet aggregation

nnel activity|negative regulation of delayed rectifier potassium channel activi

to starvation|bone mineralization|somatic stem cell population maintenance|regu

itive regulation of protein secretion|leukocyte tethering or rolling|cell adhes

membrane|ruffle membrane|cell projection|negative regulation of apoptotic proce

ranscription factor complex|heart development|blood coagulation|transcription f

acellular matrix|cellular response to vitamin K|cellular response to tumor necr

ulation of CD8-positive, alpha-beta T cell differentiation|negative regulation
|extrinsic component of external side of plasma membrane|neuromuscular junction

. of temperature stimulus involved in sensory perception of pain

ubiquitination

olved in regulation of presynaptic cytosolic calcium levels|regulation of synap

receptor signaling pathway|embryonic skeletal system development|cartilage deve

xytocin signaling pathway

n signaling pathway|Inflammatory mediator regulation of TRP channels|Long-term

l part of cell|positive regulation of transcription by RNA polymerase II|perinu

|negative regulation of apoptotic process|negative regulation of catalytic acti

›rotein localization to plasma membrane|cardiac muscle cell action potential inv

n|myoblast differentiation|positive regulation of protein secretion|cardiac mus

A polymerase II

|negative regulation of neuron differentiation|positive regulation of transcrip

tau protein binding|tau-protein kinase activity|regulation of axonogenesis|regu

tive regulation of I-kappaB kinase/NF-kappaB signaling|negative regulation of i

ll proliferation|negative regulation of DNA-binding transcription factor activi

·tery morphogenesis|cognition|neuromuscular process|head development|head morphc

retinoic acid

on of smooth muscle cell proliferation|positive regulation of nitric-oxide synth

ein metabolic process|tertiary granule lumen

|endocardial cushion to mesenchymal transition involved in heart valve formatic

· protein catabolic process|G-quadruplex DNA binding|DNA polymerase binding|oxic

:ic|cellular response to glucose starvation|intrinsic apoptotic signaling pathwa

l ERK2 cascade

ırency|chemokine (C-X-C motif) ligand 12 signaling pathway|intracellular membrar

:rolling balance|regulation of small GTPase mediated signal transduction|negativ

ılization to Golgi apparatus|low-density lipoprotein particle|negative regulatic
;ulation of transcription, DNA-templated|bone remodeling|digestive tract develop

n|Inflammatory mediator regulation of TRP channels|Pancreatic secretion|Purine

:lear hormone receptor binding|specific granule lumen|regulation of cell prolife

:eptor catabolic process|osteoclast development|melanosome|fibrinolysis|identica

on from RNA polymerase II promoter involved in kidney development|cell differen
ion|Chemokine signaling pathway|Vascular smooth muscle contraction|Inflammatory

lament binding

otein complex|siRNA loading onto RISC involved in RNA interference|posttranscrip

rotein dimerization activity|lung alveolus development|spleen development|embry

ıslational protein modification|cellular protein metabolic process|protein-conta

ing pathway

ıscle tissue morphogenesis|3M complex

ımbrane|cytosol|transcription by RNA polymerase II|heme biosynthetic process|inf

ːr complex localization to kinetochore|activation of GTPase activity|negative re

ation of canonical Wnt signaling pathway
gulation of protein secretion|regulation of endopeptidase activity|zinc ion hom

al protein modification|cellular protein metabolic process|response to ethanol|

ogenesis|bone development|bronchus morphogenesis|smoothened signaling pathway i

lination in peripheral nervous system|positive regulation of Wnt signaling path

ular transport|regulation of long-term synaptic potentiation|regulation of long

ınd branching|coronary vasculature development|semaphorin-plexin signaling pathw

'ilament capping

ıkin-1|response to interleukin-6|cellular response to interleukin-1|cellular res

nBAF complex

ation of transcription, DNA-templated|metal ion binding|stem cell differentiatio
erebellar mossy fiber|positive regulation of transcription, DNA-templated|positi
te differentiation|negative regulation of monocyte differentiation|negative reg

of cell differentiation|negative regulation of Notch signaling pathway|negative

ıtive regulation of I-kappaB kinase/NF-kappaB signaling|negative regulation of i

ıing growth factor-beta secretion

n of melanocyte differentiation|positive regulation of transcription by RNA pol

to interferon-gamma|nuclear periphery|identical protein binding|protein homodi

erentiation|negative regulation of osteoclast differentiation|protein heterodime

droxyproline transmembrane transporter activity|synaptic transmission, glutamat

scription from RNA polymerase II promoter in response to stress|neuron projectic

|Gastric acid secretion|Chemokine signaling pathway|Serotonergic synapse|Inflam

:omplex|inhibition of cysteine-type endopeptidase activity

ıosphorylation|phosphatidylinositol-mediated signaling|negative regulation of ax

ı of DNA biosynthetic process|positive regulation of transcription regulatory re

ıg

ɔisdiphosphate-2,3,4,6-tetrakisphosphate 1-diphosphatase activity|inositol-1,5-b

n|negative regulation of transcription by competitive promoter binding|Wnt sigr

terior/posterior pattern specification|enhancer binding|aorta morphogenesis|bHL

ıtive regulation of I-kappaB kinase/NF-kappaB signaling|negative regulation of i
scription by RNA polymerase II|embryonic digestive tract development|embryonic c

ɔnse to lipopolysaccharide|cellular response to muramyl dipeptide|cellular respo

ıpse assembly|excitatory postsynaptic potential|positive regulation of dendritic

other factor-regulated calcium reabsorption|Estrogen signaling pathway|Phosphol

›f transcription, DNA-templated|positive regulation of transcription by RNA poly

;e to L-glutamate|negative regulation of endoplasmic reticulum stress-induced ir

;|demethylase activity|histone demethylase activity|histone demethylase activity

:itive regulation of transcription by RNA polymerase II|negative regulation of J

ılation of endothelial cell migration|forebrain development|response to estradic

podosome assembly|protein localization to membrane|phosphatidylinositol-3,5-bis

rotein phosphatase activity|sarcoplasmic reticulum membrane|response to vitamin

negative regulation of pathway-restricted SMAD protein phosphorylation|collagen-

l mediated cytotoxicity|peptide antigen binding|MHC class I protein complex|neu

molecules|glutamatergic synapse|integral component of postsynaptic membrane|syn

me mobilization|glucocorticoid receptor signaling pathway|ATP-dependent chromat

beta receptor signaling pathway|cytokine-mediated signaling pathway|regulatio

ical Wnt signaling pathway|signaling receptor activity|progesterone secretion|p

activity|astrocyte differentiation|erythrocyte development|cartilage developmen

id|extracellular exosome|response to fibroblast growth factor|polychlorinated b

elial cell apical/basal polarity|postsynaptic membrane|positive regulation of s

from RNA polymerase II promoter|positive regulation of intrinsic apoptotic sig

velopment|regulation of myelination|negative regulation of glial cell different

ctinomycin D|response to dithiothreitol|response to anisomycin|positive regulat

morphogenesis|anterior/posterior axis specification|cell migration|regulation
l differentiation

esis|fasciculation of sensory neuron axon
od pressure|positive regulation of vasoconstriction|artery morphogenesis|protei

ctivator activity|negative regulation of cell growth|embryonic limb morphogenes

s|positive regulation of branching involved in ureteric bud morphogenesis

ar response to phorbol 13-acetate 12-myristate|response to hypobaric hypoxia

e regulation of p38MAPK cascade|regulation of AMPA receptor activity

al translation|methylcytosine dioxygenase activity|oxidative demethylation|DNA

:in binding|metal ion binding|vascular endothelial growth factor receptor signal

ood pressure|positive regulation of vasoconstriction|artery morphogenesis|protei

cardiac ventricle morphogenesis|DNA-binding transcription factor activity|trans

signaling pathway|Chemokine signaling pathway|Endocytosis|Serotonergic synapse

ion activity|positive regulation of I-kappaB kinase/NF-kappaB signaling|neutrop

m|WW domain binding|positive regulation of peptidyl-tyrosine phosphorylation|Gr

factor receptor signaling pathway|regulation of glutamate secretion|regulation

ed vesicle transport along microtubule|mitotic spindle assembly|ciliary tip|int

-loop binding|shelterin complex|establishment of protein localization to telomer

:tivity involved in Wnt signaling pathway, planar cell polarity pathway

ıctor stimulus|positive regulation of multicellular organism growth|identical pr

ve regulation of peptidase activity|membrane|macroautophagy|positive regulatior

caine|cellular response to cAMP|cellular response to tumor necrosis factor|cell

al to mesenchymal transition|lung development|embryonic limb morphogenesis|nega

g pathway|Focal adhesion|Phosphatidylinositol signaling system|cAMP signaling p

rotein localization to plasma membrane|cardiac muscle cell action potential inv

chaffer collateral - CA1 synapse|glutamatergic synapse|regulation of synaptic v

mitotic spindle|Golgi ribbon formation|Golgi disassembly|spindle assembly invol

on

mport into nucleus|DNA methylation involved in embryo development|positive regu

n phosphorylation|negative regulation of transcription, DNA-templated|positive

of transcription by RNA polymerase II|perinuclear region of cytoplasm|response

protein complex|positive regulation of stem cell differentiation|negative regula

minus-end-directed organelle transport along microtubule

›plasmic inclusion|classical Lewy body|Lewy neurite|negative regulation of oxida

ıtion|cell differentiation|purine-rich negative regulatory element binding|dendr

;ulation of mammary gland epithelial cell proliferation|multicellular organism g

:aryon|response to copper ion|perinuclear region of cytoplasm|regulation of prot

ntaining complex|homotypic cell-cell adhesion|identical protein binding|neuron p

tol-4-phosphate binding|protein localization to nuclear pore

ion of I-kappaB kinase/NF-kappaB signaling|protein self-association|regulation

ne|chloride transmembrane transport

tive regulation of transcription, DNA-templated|metal ion binding|embryonic ske

sor activity|RNA binding|nucleus|nucleoplasm|transcription factor complex|cytopl

re regulation of apoptotic process|intracellular membrane-bounded organelle|posi

ion|Inflammatory mediator regulation of TRP channels|Long-term potentiation|Pur

mmatory response|cell chemotaxis|negative regulation of SMAD protein signal tr
e to iron(III) ion|cellular response to manganese ion|cellular response to reti

regulation of cell migration involved in sprouting angiogenesis|regulation of

nding|K63-linked polyubiquitin modification-dependent protein binding|protein K

lrolysis coupled cation transmembrane transport

pocampal mossy fiber expansion

signaling pathway involved in phagocytosis|membrane raft|phagocytic vesicle|pos

ption, DNA-templated|heme binding|regulation of transcription from RNA polymera

oxygen species metabolic process|positive regulation of reactive oxygen specie

cold-induced thermogenesis

intrinsic apoptotic signaling pathway in response to DNA damage by p53 class me

enchymal cell proliferation|positive regulation of cardiac muscle hypertrophy|po

phogenesis|cartilage development

ooth|negative regulation of neuron apoptotic process|positive regulation of cel

;enesis|extracellular exosome|cell periphery|regulation of hydrogen peroxide-inc

tive regulation of insulin receptor signaling pathway|regulation of DNA strand

post-anal tail morphogenesis|zymogen granule membrane|intracellular membrane-bo

ative regulation of I-kappaB kinase/NF-kappaB signaling|negative regulation of i

ty|proximal dendrite

ılation of circadian rhythm|positive regulation of osteoclast differentiation|ne

ɜion molecule production|phosphatidylinositol-4-phosphate binding|Golgi ribbon f

ɜss|wound healing, spreading of cells|membrane raft|lymph node development|chape

'actor binding|regulation of definitive erythrocyte differentiation|transcriptic

rosis factor|cellular response to transforming growth factor beta stimulus|negat

of cell cycle|positive regulation of transcription, DNA-templated|peripheral ne
ı|secretion by cell|secretory granule organization|negative regulation of protei

ıtic vesicle exocytosis

·lopment|bone development|innervation|positive regulation of canonical Wnt signa

potentiation|Endocrine and other factor-regulated calcium reabsorption|Serotone

ıclear region of cytoplasm|actin filament binding|regulation of secretion|minus-

.vity|response to peptide hormone|post-translational protein modification|cellul

volved in contraction|ventricular cardiac muscle cell action potential|cell-cell

scle tissue morphogenesis|RNA polymerase II sequence-specific DNA-binding transc

otion, DNA-templated|negative regulation of centriole replication|perinuclear re

ulation of cytoskeleton organization|mitochondrion localization|axon development

innate immune response|negative regulation of fibroblast proliferation|perinucle

ty|sequence-specific DNA binding|negative regulation of neuron differentiation|

ɔgenesis|face morphogenesis|lung vasculature development|diaphragm development|e

ıase activity|extracellular exosome|negative regulation of cold-induced thermoge

ɔn|cardiac ventricle morphogenesis|cardiac left ventricle morphogenesis|cardiac

lation-dependent protein catabolic process

ay in response to DNA damage by p53 class mediator|identical protein binding|neg

ne-bounded organelle|positive regulation of potassium ion transport|protein auto

ve regulation of blood vessel remodeling|Schaffer collateral - CA1 synapse|gluta

›n of MAP kinase activity|cellular protein metabolic process|protein retention i
›ment|negative regulation of cytokine secretion|canonical Wnt signaling pathway

metabolism|Estrogen signaling pathway|Phospholipase D signaling pathway|Oxytoci

eration|positive regulation of protein import into nucleus|protein homodimerizat

al protein binding|negative regulation of development of symbiont involved in in

ntiation involved in kidney development|non-motile cilium|positive regulation of
mediator regulation of TRP channels|Pancreatic secretion|Purine metabolism|End

›tional gene silencing by RNA|production of miRNAs involved in gene silencing by

yonic digestive tract morphogenesis|reproductive structure development|gland dev

ıining complex binding|negative regulation of osteoclast differentiation|embryor

:lammatory response|cholesterol metabolic process|lipid binding|anatomical struc

:gulation of substrate adhesion-dependent cell spreading|regulation of ruffle as

neostasis|phosphatidic acid binding|cellular response to manganese ion|cellular

response to calcium ion|general adaptation syndrome, behavioral process|cell bo

nvolved in dorsal/ventral neural tube patterning|protein localization to cilium

way|embryonic limb morphogenesis|BMP signaling pathway|negative regulation of t

-term synaptic depression|extracellular vesicle|positive regulation of AMPA rec

/ay|semaphorin-plexin signaling pathway involved in axon guidance

sponse to tumor necrosis factor|interleukin-8 secretion

›n
.ve regulation of transcription by RNA polymerase II|Clara cell differentiation|
;ulation of transcription, DNA-templated|positive regulation of transcription by

regulation of photoreceptor cell differentiation|embryonic cranial skeleton mor

nnate immune response|negative regulation of fibroblast proliferation|perinucle

ymerase II|metal ion binding|positive regulation of melanin biosynthetic proces

merization activity|negative regulation of DNA binding|negative regulation of I

erization activity|thymus development

ergic|melanosome|neuronal cell body|cognition|extracellular exosome

on extension|cellular response to leucine starvation

matory mediator regulation of TRP channels|Long-term potentiation|Endocrine and

xon regeneration|regulation of protein kinase B signaling|inositol monophosphate

egion DNA binding

oisdiphosphate-2,3,4,6-tetrakisphosphate 5-diphosphatase activity|inositol-3,5-b

naling pathway|negative regulation of angiogenesis|poly(A)+ mRNA export from nuc

.H transcription factor binding|sequence-specific DNA binding|transcription regu

innate immune response|negative regulation of fibroblast proliferation|perinucle
camera-type eye morphogenesis|genitalia development|roof of mouth development

›nse to interferon-gamma|scaffold protein binding|keratin filament binding

: spine development|regulation of dendritic spine morphogenesis|cellular respons

ipase D signaling pathway|Oxytocin signaling pathway

ymerase II|embryonic organ development|positive regulation of cardiac muscle cel

ntrinsic apoptotic signaling pathway|negative regulation of hypoxia-induced intr

y (H3-K4 specific)|histone demethylase activity (H3-K9 specific)|protein-contair

[illegible]NK cascade|negative regulation of inflammatory response|negative regulation of

[illegible]l|protein homodimerization activity|sequence-specific DNA binding|negative regu[illegible]

sphosphate binding|positive regulation of adipose tissue development|cranial ske

E|ion transmembrane transport|calcium channel complex|calcium-mediated signalir

-containing extracellular matrix|extracellular exosome|protein homotrimerizatior

ıtrophil degranulation|regulation of immune response|recycling endosome membrane

naptic membrane adhesion|neuron projection extension

:in remodeling|positive regulation of transcription, DNA-templated|chromatin-med

n of T cell differentiation in thymus|negative regulation of endothelial cell di

›rotein homodimerization activity|Wnt-activated receptor activity|positive regul

ıt|cardiac muscle cell differentiation|positive regulation of cartilage developm

›iphenyl binding

smoothened signaling pathway|positive regulation of synapse assembly|epithelial

naling pathway in response to DNA damage

ciation|positive regulation of glial cell differentiation|negative regulation of

:ion of histone H4 acetylation|positive regulation of protein localization to nu

of ossification|regulation of chondrocyte differentiation|middle ear morphogene

n complex oligomerization|gap junction hemi-channel activity|regulation of carc

sis|negative regulation of bone mineralization|negative regulation of BMP signal

demethylation|1-ethyladenine demethylase activity|class I DNA-(apurinic or apyr

ing pathway|axon extension involved in axon guidance|negative chemotaxis|sympat

n complex oligomerization|gap junction hemi-channel activity|regulation of carc

scription corepressor activity|nucleus|nucleoplasm|cytoplasm|Notch signaling pat

|Apoptosis|Long-term potentiation|B cell receptor signaling pathway|ErbB signal

ɔhil degranulation|negative regulation of viral genome replication|innate immune

·b2-EGFR complex|regulation of clathrin-dependent endocytosis

ı of dopamine secretion|actin cytoskeleton|membrane|inclusion body|oxidoreductas

:raciliary transport particle B binding|axon cytoplasm

·e|G-rich strand telomeric DNA binding|8-hydroxy-2'-deoxyguanosine DNA binding|[illegible]

·otein binding|protein homodimerization activity|cysteine-type endopeptidase inh

[illegible] of macroautophagy|negative regulation of macroautophagy|kinase activity|pepti[illegible]

[illegible]ular response to estrogen stimulus|cellular response to estradiol stimulus|reg[illegible]

ıtive regulation of cell migration|protein-containing complex|protein-DNA comple

athway|Cholinergic synapse|Jak-STAT signaling pathway|Phospholipase D signaling

rolved in contraction|ventricular cardiac muscle cell action potential|cell-cell

vesicle exocytosis

.ved in meiosis|mitotic spindle assembly|positive regulation of ubiquitin protei

ılation of DNA binding|sequence-specific DNA binding|innate immune response|posi

regulation of growth|metal ion binding|perinuclear region of cytoplasm|positive

to growth factor|positive regulation of cardiac muscle myoblast proliferation

ıtion of cytoplasmic translation

ıtive stress-induced neuron death|negative regulation of hydrogen peroxide-induc

ite cytoplasm|neuronal cell body|sequence-specific DNA binding|SMAD binding|pos

;rowth|positive regulation of apoptotic process|positive regulation of myeloid c

:ein secretion|protein homooligomerization|response to glucocorticoid|maternal p

projection|cell body|membrane raft|cell-cell junction organization|gamma-delta T

of inflammatory response|defense response to Gram-positive bacterium|positive r

eletal system development

.asm|regulation of transcription by RNA polymerase II|RNA localization|cellular

.tive regulation of angiogenesis|negative regulation of cell cycle|actin filamen

ine metabolism|Estrogen signaling pathway|Phospholipase D signaling pathway|Oxy

ınsduction|negative regulation of cell death|hematopoietic stem cell homeostasis
noic acid|cellular response to epidermal growth factor stimulus|cellular respor

protein deacetylation

[illegible]63-linked ubiquitination|histone H2A K63-linked ubiquitination

sitive regulation of gene expression, epigenetic|actin filament binding|mRNA tra

ase II promoter in response to hypoxia

es metabolic process|negative regulation of ATP biosynthetic process|positive re

ediator|identical protein binding|ribosome binding|negative regulation of apopto

›sitive regulation of gene expression|negative regulation of gene expression|neg

.l differentiation|negative regulation of Notch signaling pathway|positive regul

luced cell death

elongation|cellular response to X-ray|modulation by host of RNA binding by viru

ounded organelle|embryonic morphogenesis|labyrinthine layer blood vessel develop

nnate immune response|negative regulation of fibroblast proliferation|perinucle

:gative regulation of transcription, DNA-templated|positive regulation of transc

:ormation|asymmetric Golgi ribbon formation

:rone binding|positive regulation of cellular component movement|lymphatic endot

:nal repressor complex|erythrocyte differentiation|megakaryocyte differentiation

:ive regulation of substrate adhesion-dependent cell spreading|positive regulati

:rvous system neuron development|negative regulation of epithelial cell prolifer
:n catabolic process|mating plug formation|negative regulation of proteolysis|ne

ıling pathway|liver regeneration|regulation of hepatocyte proliferation

rgic synapse|Oxytocin signaling pathway

-end directed microfilament motor activity|extracellular exosome

.ar protein metabolic process|negative regulation of membrane protein ectodomair

. signaling involved in cardiac conduction|cell communication by electrical cou[illegible]

cription factor binding|R-SMAD binding|cellular response to lipopolysaccharide|c

egion of cytoplasm|extracellular exosome|regulation of mitotic cell cycle spindl

:|regulation of microtubule cytoskeleton organization|establishment or maintenar

ear region of cytoplasm|negative regulation of epithelial cell proliferation|neg

positive regulation of transcription by RNA polymerase II|protein heterodimeriz

embryonic camera-type eye formation|eyelid development in camera-type eye|uterus

enesis

right ventricle morphogenesis|ventricular trabecula myocardium morphogenesis|DN

;ative regulation of DNA binding|negative regulation of viral genome replicatior

phosphorylation|regulation of inflammatory response|ion homeostasis|cellular hy

amatergic synapse

in Golgi apparatus|positive regulation of protein catabolic process|negative reg
involved in metanephric kidney development|cell differentiation involved in met

n signaling pathway|Longevity regulating pathway - multiple species

tion activity|neutrophil degranulation|negative regulation of blood vessel endot

nteraction with host|S100 protein binding|bone sialoprotein binding|positive reg

f protein localization to nucleus
locrine and other factor-regulated calcium reabsorption|Estrogen signaling pathw

, miRNA|siRNA binding|miRNA binding|miRNA mediated inhibition of translation|mRN

,elopment|androgen receptor binding|Sertoli cell differentiation|roof of mouth c

ıic eye morphogenesis|post-embryonic eye morphogenesis|collagen-containing extra

:ture morphogenesis|cholesterol binding|integral component of membrane|protein c

ssembly|mitotic spindle midzone

response to zinc ion|phosphatidylinositol-3,5-bisphosphate binding|extracellula

ody fiber|cellular response to vitamin D|cellular response to cAMP|cellular resp

n|ganglion development|receptor localization to non-motile cilium|ciliary base|

transforming growth factor beta receptor signaling pathway|negative regulation

eptor activity

type I pneumocyte differentiation|type II pneumocyte differentiation|salivary g
y RNA polymerase II|embryonic skeletal system morphogenesis|stem cell differenti

rphogenesis|negative regulation of oligodendrocyte differentiation|branching mor

ear region of cytoplasm|negative regulation of epithelial cell proliferation|neg

ss|developmental pigmentation|collateral sprouting|positive regulation of axonog

DNA-binding transcription factor activity|maintenance of protein location|regula

l other factor-regulated calcium reabsorption|Estrogen signaling pathway|Calcium

3-phosphatase activity|inositol monophosphate 4-phosphatase activity|recycling

isdiphosphate-2,3,4,6-tetrakisphosphate 5-diphosphatase activity|diphosphoinosi

:leus|TBP-class protein binding|cell differentiation|positive regulation of Wnt

ılatory region DNA binding|positive regulation of transcription, DNA-templated|ı

ear region of cytoplasm|negative regulation of epithelial cell proliferation|neg

se to toxic substance|postsynaptic actin cytoskeleton|postsynaptic actin cytoske

.l proliferation|ventricular septum morphogenesis|negative regulation of cell de

:insic apoptotic signaling pathway|negative regulation of transcription from RN/

ning complex|histone H3-K9 demethylation|positive regulation of histone ubiquiti

B cell receptor signaling pathway|response to mitochondrial depolarisation|nega

ılation of cyclin-dependent protein serine/threonine kinase activity|negative re

eletal system development

ng using intracellular calcium source|protein refolding|T cell proliferation|res

n|negative regulation of ERK1 and ERK2 cascade|integrin binding involved in cell

e|interferon-gamma-mediated signaling pathway|type I interferon signaling pathwa

liated maintenance of transcription|cardiac muscle cell differentiation|placenta

ifferentiation|positive regulation of neuron differentiation|negative regulation

lation of JUN kinase activity|protein-containing complex binding|positive regula

ment|cellular response to transforming growth factor beta stimulus|negative regu

tube branching involved in lung morphogenesis|positive regulation of dendritic

[illegible] transcription, DNA-templated|neuromuscular process controlling balance

ıcleus

esis|protein-containing complex binding|negative regulation of cell differentiat

diac muscle contraction|regulation of ventricular cardiac muscle cell membrane r

ling pathway|negative regulation of chondrocyte differentiation|negative regulat

rimidinic site) endonuclease activity|tRNA demethylation|tRNA demethylase activi

:hetic ganglion development|trigeminal ganglion development|sensory neuron axon

liac muscle contraction|regulation of ventricular cardiac muscle cell membrane r

:hway|positive regulation of transcription of Notch receptor target|heart develo

ing pathway|Fc epsilon RI signaling pathway|Oxytocin signaling pathway|Longevit

e response|membrane raft|defense response to virus|type I interferon signaling p

se activity|kinesin binding|regulation of transmembrane transporter activity|cel

:egulation of DNA helicase activity|positive regulation of DNA helicase activity

ıibitor activity involved in apoptotic process|actin nucleation|actin filament s

lase activator activity|protein ubiquitination|peptidyl-serine phosphorylation|r

ılation of cellular response to stress|cellular response to gonadotropin-releasi

ex|embryonic forelimb morphogenesis|forelimb morphogenesis|sequence-specific DN

pathway|AMPK signaling pathway|VEGF signaling pathway|Fc gamma R-mediated phag

signaling involved in cardiac conduction|cell communication by electrical cou

in ligase activity

itive regulation of DNA repair|negative regulation of single stranded viral RNA

e regulation of chemotaxis|negative regulation of protein transport|positive reg

ced neuron death|positive regulation of autophagy of mitochondrion|Lewy body cor

stsynapse|dendritic transport of messenger ribonucleoprotein complex|glutamaterg

cell differentiation|negative regulation of erythrocyte differentiation|positive

›rocess involved in female pregnancy|limb development|extracellular exosome

[illegible] cell activation|cardiac muscle fiber development|regulation of immune respons[illegible]

[illegible]egulation of NF-kappaB transcription factor activity|type I interferon signali[illegible]

response to DNA damage stimulus|spermatogenesis|zinc ion binding|anterior/poste

nt bundle assembly|endothelial tube morphogenesis|cellular response to hydrogen

tocin signaling pathway|Longevity regulating pathway - multiple species

s|collagen-containing extracellular matrix|endothelial cell-cell adhesion|negati
nse to hypoxia|cellular response to mercaptoethanol|cellular response to oxygen-

ansport|stereocilium membrane|extracellular exosome|cellular response to interfe

egulation of ATP biosynthetic process

otic process|intracellular membrane-bounded organelle|negative regulation of pro

gative regulation of cardiac muscle cell apoptotic process|neural crest cell dev

lation of transcription by RNA polymerase II|negative regulation of photorecepto

ıs|modulation by host of viral RNA-binding transcription factor activity

oment|protein localization to plasma membrane

ear region of cytoplasm|negative regulation of epithelial cell proliferation|neg

:ription by RNA polymerase II|metal ion binding|cellular response to peptide

:helial cell fate commitment|leading edge of lamellipodium|actin-mediated cell c

ı|platelet formation|granulocyte differentiation|negative regulation of protein

ion of chemokine (C-C motif) ligand 2 secretion|neuron projection extension|bone

:ation|response to transforming growth factor beta
:gative regulation of smoothened signaling pathway|regulation of timing of cell

ı proteolysis|cartilage development|extracellular exosome|negative regulation oí

oling involved in cardiac conduction|bundle of His cell-Purkinje myocyte adhesi

:ellular response to mechanical stimulus|cellular response to interleukin-1|cel

le assembly checkpoint|mitotic spindle pole|ciliary basal body-plasma membrane

ıce of cell polarity regulating cell shape|microtubule bundle|regulation of mic

gative regulation of inflammatory response|negative regulation of cytokine prod

zation activity|spleen development|thymus development|embryonic organ developme

s morphogenesis|alveolar primary septum development|pulmonary artery morphogenes

NA-binding transcription factor activity|transcription corepressor activity|nucl

n|innate immune response|negative regulation of innate immune response|negative

ypotonic response|negative regulation of pancreatic juice secretion|negative reg

gulation of neurogenesis|protein maturation|recycling endosome|extracellular exc
tanephros development|metanephric glomerulus development|metanephric nephron tub

thelial cell migration|alpha-catenin binding|cadherin binding|positive regulatio

gulation by host of viral process|virion binding|positive regulation of fibrobla

vay|Oxytocin signaling pathway|Longevity regulating pathway - multiple species

NA cleavage involved in gene silencing by miRNA|miRNA loading onto RISC involved

development|lung morphogenesis|lung vasculature development|bronchiole developme

acellular matrix|cellular response to transforming growth factor beta stimulus|c

domain specific binding|integral component of endoplasmic reticulum membrane|ery

ar exosome biogenesis|negative regulation of neuron death|polyamine transmembran

ponse to transforming growth factor beta stimulus|response to epinephrine|cellul

9+0 non-motile cilium|intraciliary transport particle A binding|negative regulat

of BMP signaling pathway|ubiquitin protein ligase binding|negative regulation of

gland cavitation|cell differentiation involved in salivary gland development|lu
iation

rphogenesis of a nerve|cartilage development|positive regulation of amacrine ce

gative regulation of inflammatory response|negative regulation of cytokine produ

genesis|mammillary axonal complex development|R-SMAD binding|melanocyte migratio

ation of angiogenesis|negative regulation of transcription, DNA-templated|positi

ı signaling pathway|Wnt signaling pathway|Oxytocin signaling pathway

g endosome|clathrin-dependent endocytosis|regulation of cell motility|regulation

itol polyphosphate metabolic process|diadenosine pentaphosphate catabolic proces

signaling pathway|B cell differentiation|negative regulation of vascular endotl

ɔositive regulation of transcription by RNA polymerase II|SMAD binding|axial mes

gative regulation of inflammatory response|negative regulation of cytokine produ

eleton organization|glutamatergic synapse|negative regulation of long-term synap

eath|positive regulation of male gonad development|negative regulation of female

A polymerase II promoter in response to endoplasmic reticulum stress|negative re

ination|cellular response to UV|histone demethylase activity (H3-dimethyl-K4 spe

ative regulation of protein K63-linked ubiquitination

egulation of transcription, DNA-templated|positive regulation of transcription,

sponse to hydrogen peroxide|ion channel binding|positive regulation of axon rege

l-matrix adhesion|positive regulation of extracellular matrix assembly|negative

ay|extracellular exosome|integral component of lumenal side of endoplasmic retic

blood vessel development|npBAF complex|nBAF complex|toxin transport

of transcription, DNA-templated|positive regulation of transcription by RNA p

ation of transcription, DNA-templated|protein heterodimerization activity|posit

ılation of cardiac muscle cell differentiation|positive regulation of mesenchyma

spine development|protein-containing complex assembly|protein localization to [illegible]

tion|negative regulation of transcription, DNA-templated|embryonic digestive tra

repolarization|regulation of atrial cardiac muscle cell membrane depolarization

tion of osteoblast proliferation|BMP binding|protein homodimerization activity|r

ity

guidance|sympathetic neuron projection extension|sympathetic neuron projection

repolarization|regulation of atrial cardiac muscle cell membrane depolarization

opment|transcription factor binding|mesenchymal cell development|cell different

:y regulating pathway - multiple species|mTOR signaling pathway

oathway|extracellular exosome|negative regulation of intracellular transport of

ll junction|growth cone|Hsp70 protein binding|synaptic vesicle membrane|platele

y|G-rich single-stranded DNA binding

severing|actin filament binding|barbed-end actin filament capping|regulation of

respiratory electron transport chain|axon|growth cone|integral component of mit

ing hormone|negative regulation of corticotropin-releasing hormone receptor act

\ binding|positive regulation of transcription, DNA-templated|positive regulatio

gocytosis|FoxO signaling pathway|Thyroid hormone signaling pathway|Regulation of

oling involved in cardiac conduction|bundle of His cell-Purkinje myocyte adhesio

replication via double stranded DNA intermediate|negative regulation of transcr

gulation of stress fiber assembly|positive regulation of focal adhesion assembly

:e|Lewy body corona|negative regulation of G1/S transition of mitotic cell cycle

gic synapse

e regulation of transcription by RNA polymerase II|lung alveolus development|emb

e|cell adhesion molecule binding|leukocyte migration|defense response to virus|r

ng pathway|interleukin-1-mediated signaling pathway|response to interleukin-1|3’

erior pattern specification|response to UV-C|negative regulation of gene express

peroxide|cellular response to ionizing radiation

ive regulation of monocyte chemotaxis|negative regulation of NIK/NF-kappaB sign[illegible]
-glucose deprivation|response to kainic acid|response to hypobaric hypoxia

eron-gamma|positive regulation of protein targeting to membrane|positive regulat

oteolysis|regulation of embryonic development|protein stabilization|misfolded pr

velopment|protein-containing complex|activating transcription factor binding|reg

or cell differentiation|embryonic skeletal system development|negative regulatio

gative regulation of inflammatory response|negative regulation of cytokine produ

contraction|invadopodium|positive regulation of extracellular matrix disassembly

binding|regulation of chemokine production|embryonic hemopoiesis|megakaryocyte

e regeneration

differentiation|positive regulation of astrocyte differentiation|detection of [illegible]

f trophoblast cell migration|negative regulation of metallopeptidase activity|re

on involved in cell communication|cell adhesive protein binding involved in bun

lular response to tumor necrosis factor|cellular response to organic cyclic com

docking

rotubule binding

uction involved in inflammatory response|negative regulation of NLRP3 inflammas

nt|embryonic skeletal system development|RNA polymerase II transcription factor

sis|paramesonephric duct development|vitamin A import|ductus arteriosus closure

leus|nucleoplasm|cytoplasm|Notch signaling pathway|heart development|transcripti

regulation of transcription, DNA-templated|positive regulation of transcriptio[illegible]

gulation of potassium ion transmembrane transporter activity|negative regulatio

osome|positive regulation of protein exit from endoplasmic reticulum|negative r[illegible]
oule morphogenesis|positive regulation of branching involved in ureteric bud mo[illegible]

on of angiogenesis|positive regulation of transcription by RNA polymerase II|cel

ast proliferation|protein heterotetramerization|positive regulation of calcium

d in gene silencing by miRNA|positive regulation of angiogenesis|positive regula

ent|diaphragm development|respiratory system development|negative regulation of

cellular response to insulin-like growth factor stimulus|negative regulation of

ythrocyte differentiation|protein-containing complex|cellular response to oxida

ne transport|cupric ion binding|regulation of autophagy of mitochondrion|positi

lar response to virus|neuronal dense core vesicle lumen|positive regulation of l

tion of smoothened signaling pathway involved in dorsal/ventral neural tube pat

f activin receptor signaling pathway|protein-containing complex|somatic stem ce

ng ciliated cell differentiation|commissural neuron axon guidance|negative regu

ll differentiation

ıction involved in inflammatory response|negative regulation of NLRP3 inflammas

on|positive regulation of lens fiber cell differentiation|regulation of melanos

ive regulation of transcription, DNA-templated|negative regulation of protein e

n of endocytic recycling

ss|diadenosine hexaphosphate catabolic process|adenosine 5'-(hexahydrogen penta

helial growth factor receptor signaling pathway|protein-DNA complex|protein loca

soderm development|androgen receptor binding|negative regulation of androgen rec

ıction involved in inflammatory response|negative regulation of NLRP3 inflammas[illegible]

ptic potentiation|positive regulation of long-term synaptic depression|negative

e gonad development

egulation of apoptotic signaling pathway

ecific)|histone H3-K4 demethylation|positive regulation of chromatin binding|te

DNA-templated|blood vessel morphogenesis|limb development|placenta blood vesse

eneration|negative regulation of release of sequestered calcium ion into cytoso

regulation of collagen fibril organization|negative regulation of macrophage m

culum membrane|regulation of dendritic cell differentiation

olymerase II|embryonic camera-type eye morphogenesis|embryonic skeletal system r

ive regulation of DNA-binding transcription factor activity|canonical Wnt signa

al stem cell differentiation

adherens junction|metanephric collecting duct development|cell-cell adhesion

act morphogenesis|embryonic cranial skeleton morphogenesis|anatomical structure

|regulation of ventricular cardiac muscle cell membrane depolarization|ventricul

negative regulation of apoptotic process|vascular endothelial growth factor rec

guidance|glutamatergic synapse|integral component of postsynaptic membrane|regu

|regulation of ventricular cardiac muscle cell membrane depolarization|ventricul

iation|glomerulus development|skeletal muscle cell differentiation|microsatelli

viral material

t alpha granule membrane|negative regulation of microtubule polymerization|recep

actin nucleation|actin filament capping|cytoplasmic actin-based contraction in[illegible]

ochondrial outer membrane|regulation of protein ubiquitination|positive regulat[illegible]

ivity|regulation of NMDA receptor activity

on of transcription by RNA polymerase II|positive regulation of cardioblast dif

actin cytoskeleton|cGMP-PKG signaling pathway|Prolactin signaling pathway|Chem

on involved in cell communication|cell adhesive protein binding involved in bun

ription, DNA-templated|positive regulation of transcription, DNA-templated|prot

y|peptidyl-serine dephosphorylation|intrinsic apoptotic signaling pathway|regul

e

ɔryonic skeletal system morphogenesis|embryonic skeletal system development|bro[illegible]

negative regulation of cardiac muscle cell proliferation|transepithelial transp[illegible]

[illegible]-UTR-mediated mRNA stabilization|TIR domain binding|cellular response to mecha[illegible]

sion|nuclear matrix|protein deubiquitination|cell differentiation|Ino80 complex

aling|negative regulation of chondrocyte proliferation|negative regulation of se

tion of cellular response to insulin stimulus|positive regulation of vascular e[illegible]

rotein binding|endoplasmic reticulum stress-induced pre-emptive quality control

gulation of tissue remodeling|odontogenesis of dentin-containing tooth|embryoni[illegible]

[illegible]on of oligodendrocyte differentiation|cellular response to transforming growth [illegible]

ıction involved in inflammatory response|negative regulation of NLRP3 inflammas[illegible]

y|tetraspanin-enriched microdomain|regulation of substrate adhesion-dependent c

development|positive regulation of interferon-gamma biosynthetic process|negat

nechanical stimulus involved in sensory perception|regulation of synaptic trans

egulation of integrin-mediated signaling pathway

dle of His cell-Purkinje myocyte communication|regulation of heart rate by card

pound|cellular response to hypoxia|cellular response to transforming growth fac

ome complex assembly|negative regulation of NIK/NF-kappaB signaling

complex

ion factor binding|anterior/posterior axis specification|positive regulation of

n by RNA polymerase II|defense response to virus|cellular response to ionizing

n of potassium ion transmembrane transport|negative regulation of creatine tran

egulation of neuron death|negative regulation of amyloid-beta formation|positiv
rphogenesis|positive regulation of canonical Wnt signaling pathway|negative reg

ll adhesion molecule binding|detection of mechanical stimulus|positive regulati

ion transport|catabolism by host of symbiont protein|negative regulation by hos

ation of transcription by RNA polymerase II|negative regulation of translationa

androgen receptor signaling pathway|E-box binding|metanephric mesenchymal cell

osteoclast development

tive stress|positive regulation of transcription from RNA polymerase II promote

ve regulation of exosomal secretion|regulation of chaperone-mediated autophagy|a

behavioral fear response

terning|protein localization to photoreceptor outer segment

ll population maintenance|camera-type eye development|positive regulation of DN

lation of pri-miRNA transcription by RNA polymerase II|negative regulation of m

ome complex assembly|negative regulation of NIK/NF-kappaB signaling

ome organization

xport from nucleus|retinoic acid receptor signaling pathway|positive regulation

phosphate) catabolic process

alization to nucleus|protein homodimerization activity|response to peptide horm

ceptor signaling pathway|co-SMAD binding|R-SMAD binding|cellular response to cy

ome complex assembly|negative regulation of NIK/NF-kappaB signaling

regulation of spontaneous neurotransmitter secretion|growth cone lamellipodium

lomeric DNA binding|neuron maturation|negative regulation of DNA binding|MRF bi

l development|trophoblast giant cell differentiation|regulation of transcriptio

l|positive regulation of sequestering of calcium ion|regulation of cytosolic ca

igration|EMILIN complex

morphogenesis|semicircular canal morphogenesis|regulation of smooth muscle cell

ling pathway|Wnt signaling pathway, planar cell polarity pathway|membrane organ

development|nephron development|nephron morphogenesis|mesenchymal stem cell ma

lar septum morphogenesis|atrial septum morphogenesis|connexin binding|ventricula

eptor 2 binding|negative regulation of osteoblast differentiation|positive regu

ulation of postsynapse organization|neural crest cell migration involved in aut[illegible]

lar septum morphogenesis|atrial septum morphogenesis|connexin binding|ventricul[illegible]

te binding|protein homodimerization activity|sequence-specific DNA binding|posi[illegible]

ptor internalization|protein destabilization|response to magnesium ion|negative

volved in cell motility|regulation of wound healing|protein-containing complex a

ion of protein ubiquitination|ubiquitin protein ligase binding|activation of pr[illegible]

ferentiation|cardiac muscle cell differentiation|pericardium development|negati

nokine signaling pathway|Estrogen signaling pathway|Inflammatory mediator regul

dle of His cell-Purkinje myocyte communication|regulation of heart rate by card

ein autophosphorylation|protein complex oligomerization|convergent extension in

ation of cellular protein localization|negative regulation of cell-cell adhesio

nchiole development|trachea morphogenesis|epithelial tube branching involved in

ort|connexin binding|AV node cell to bundle of His cell communication|AV node c

nical stimulus|cellular response to oxidised low-density lipoprotein particle s

|PcG protein complex|negative regulation of interferon-beta production|cellular

ensory perception of pain|bone regeneration

ndothelial growth factor signaling pathway|regulation of bicellular tight junct

intrinsic apoptotic signaling pathway in response to endoplasmic reticulum str

c digit morphogenesis|protein homodimerization activity|negative regulation of a

factor beta stimulus|cellular response to BMP stimulus|positive regulation of an

ome complex assembly|negative regulation of NIK/NF-kappaB signaling

ell spreading|positive regulation of platelet aggregation|regulation of myofibr

ive regulation of interleukin-4 biosynthetic process|negative regulation of fat

nission, glutamatergic|long-term synaptic potentiation|innervation|seminal vesi

iac conduction|cell-cell adhesion|regulation of ventricular cardiac muscle cell

tor beta stimulus|negative regulation of DNA biosynthetic process

heart rate|negative regulation of transcription by transcription factor locali

radiation|intrinsic apoptotic signaling pathway by p53 class mediator|activatio

smembrane transporter activity|cellular response to chemokine|negative regulati

e regulation of choline O-acetyltransferase activity|negative regulation of tau
ulation of cold-induced thermogenesis|epithelial cell proliferation involved in

on of DNA-binding transcription factor activity|protein heterooligomerization|e

t of symbiont molecular function|endocardial cell differentiation|extracellular

l initiation|metal ion binding|positive regulation of nuclear-transcribed mRNA

differentiation|metanephric glomerular capillary formation

r in response to stress|cholesterol homeostasis|protein-containing complex bind

autophagosome organization|integral component of lysosomal membrane|regulation

A binding|nose morphogenesis|negative regulation of cell differentiation|negati

esenchymal cell proliferation involved in lung development|negative regulation

of DNA-binding transcription factor activity|negative regulation of cellular c

one|sequence-specific DNA binding|transcription regulatory region DNA binding|n

tokine stimulus|nodal signaling pathway involved in determination of lateral me

nding|negative regulation of DNA-binding transcription factor activity|negative

n involved in lymphatic endothelial cell fate commitment

lcium ion concentration|response to redox state|regulation of ryanodine-sensiti

differentiation|cartilage development|E-box binding|cellular response to amino

ization|retina vasculature morphogenesis in camera-type eye|cerebellum vasculat

intenance involved in nephron morphogenesis|condensed mesenchymal cell prolifer

ar cardiac muscle cell action potential|SA node cell action potential|gap junct

lation of angiogenesis|negative regulation of transcription, DNA-templated|posi

onomic nervous system development|semaphorin-plexin signaling pathway involved

ar cardiac muscle cell action potential|SA node cell action potential|gap junct

tive regulation of neuron differentiation|negative regulation of transcription,

regulation of transporter activity|response to lipopolysaccharide|negative reg

assembly|extracellular exosome|cellular response to epidermal growth factor sti

otein kinase B activity|positive regulation of synaptic transmission, dopaminer

ve regulation of cardiac muscle cell proliferation|positive regulation of cardi

ation of TRP channels|Apoptosis|B cell receptor signaling pathway|ErbB signalin

iac conduction|cell-cell adhesion|regulation of ventricular cardiac muscle cell

volved in axis elongation|embryonic placenta morphogenesis|chromo shadow domain

n mediated by cadherin

lung morphogenesis|lung goblet cell differentiation|lobar bronchus epithelium

ell-bundle of His cell adhesion involved in cell communication|cell adhesive pr

timulus|positive regulation of cytokine production involved in inflammatory res

response to UV|response to prostaglandin F|histone deacetylase binding|sequenc

ion assembly

ess|extracellular exosome|proteasome binding|ER-associated misfolded protein ca

apoptotic process|negative regulation of DNA binding|negative regulation of DNA

macrine cell differentiation|negative regulation of cellular response to transf

oblast contraction|invadopodium organization|regulation of lamellipodium morpho

cell differentiation|regulation of megakaryocyte differentiation|metal ion bin

cle epithelium development|collagen-containing extracellular matrix|negative re

action potential

zation|negative regulation of gene expression|negative regulation of cardiac mu

n of cysteine-type endopeptidase activity|negative regulation of cysteine-type

on of sodium ion transmembrane transporter activity

-protein kinase activity|positive regulation of ER to Golgi vesicle-mediated tr
renal tubule morphogenesis

xtracellular exosome|cornification|endothelial cell-cell adhesion|gamma-catenin

exosome|protein localization to plasma membrane|voltage-gated calcium channel

poly(A) tail shortening|regulation of gene silencing by miRNA|extracellular exo

ing|cell redox homeostasis|positive regulation of transcription, DNA-templated|

of glucosylceramidase activity|regulation of lysosomal protein catabolic proces

ve regulation of osteoblast differentiation|positive regulation of transcriptio

of epithelial cell proliferation involved in lung morphogenesis

omponent movement|interferon-gamma-mediated signaling pathway|type I interferon

egative regulation of cyclin-dependent protein serine/threonine kinase activity

soderm left/right asymmetry|negative regulation of androgen receptor activity

regulation of DNA damage response, signal transduction by p53 class mediator|t

ve calcium-release channel activity|negative regulation of ryanodine-sensitive

acid stimulus|cochlea morphogenesis

ure morphogenesis|retinal blood vessel morphogenesis|cellular response to retin

ation|mesenchymal cell differentiation involved in kidney development|regulatio

ion channel activity involved in SA node cell-atrial cardiac muscle cell electr

tive regulation of transcription by RNA polymerase II|negative regulation of bo

in neuron projection guidance|facioacoustic ganglion development|dorsal root ga

ion channel activity involved in SA node cell-atrial cardiac muscle cell electr

DNA-templated|positive regulation of transcription by RNA polymerase II|protei

ulation of monooxygenase activity|positive regulation of peptidyl-serine phosph

mulus|terminal web assembly|positive regulation of protein localization to plas

gic|positive regulation of peptidyl-serine phosphorylation|positive regulation

ac muscle cell proliferation|atrial septum morphogenesis|cell migration involve

g pathway|Fc epsilon RI signaling pathway|Oxytocin signaling pathway|Longevity [illegible]

action potential

binding|positive regulation of methylation-dependent chromatin silencing|RNA p

development|lung-associated mesenchyme development|trachea cartilage morphogene

otein binding involved in AV node cell-bundle of His cell communication|regulat

ponse

e-specific DNA binding|transcription regulatory region DNA binding|positive reg

tabolic process|tail-anchored membrane protein insertion into ER membrane|BAT3

-binding transcription factor activity|sequence-specific DNA binding|tongue dev

orming growth factor beta stimulus

genesis

ding|cardiac muscle tissue morphogenesis|definitive erythrocyte differentiation

gulation of platelet aggregation|extracellular vesicle

scle cell apoptotic process|mesenchymal cell development|cardiac muscle hypertr

endopeptidase activity

ansport|positive regulation of early endosome to recycling endosome transport|n

-TCF7L2 complex|cellular response to indole-3-methanol|protein localization to

activity involved in regulation of cytosolic calcium levels|positive regulation

some|endoribonuclease activity, cleaving siRNA-paired mRNA|RISC-loading complex

protein heterodimerization activity|lipid homeostasis|cellular response to chol

s|negative regulation of lysosomal protein catabolic process|peptidyl-aspartic

n by RNA polymerase II|SMAD binding|histone deacetylase inhibitor activity|nega

signaling pathway|chromo shadow domain binding|regulation of extrinsic apoptot

|negative regulation of transcription, DNA-templated|positive regulation of tra

ranscription regulatory region DNA binding|positive regulation of cell size|neg

calcium-release channel activity|chaperone-mediated protein folding|negative re

oic acid|glutamatergic synapse|positive regulation of neuron projection arboriz

n of branching involved in ureteric bud morphogenesis|mesenchymal stem cell pro

ical coupling|SA node cell to atrial cardiac muscle cell communication by elect

ne remodeling|receptor ligand activity|determination of dorsal identity|positiv

nglion morphogenesis|cellular response to leukemia inhibitory factor

ical coupling|SA node cell to atrial cardiac muscle cell communication by elect

n heterodimerization activity|AF-1 domain binding|regulation of neurogenesis|ca

orylation|response to interferon-gamma|cellular response to oxidative stress|ne

ma membrane|regulation of lamellipodium morphogenesis|positive regulation of la

of dopamine secretion|cellular response to oxidative stress|positive regulation

d in coronary vasculogenesis|positive regulation of secondary heart field cardi

regulating pathway - multiple species|mTOR signaling pathway

olymerase II transcription factor complex|negative regulation of DNA demethylat

sis|cartilage morphogenesis|intestinal epithelial cell maturation|mesenchymal-e

ion of AV node cell action potential

ulation of transcription by RNA polymerase II|SMAD binding|camera-type eye morp

complex|positive regulation of ERAD pathway|maintenance of unfolded protein inv

elopment|negative regulation of osteoblast differentiation|positive regulation

|primitive erythrocyte differentiation|negative regulation of mast cell differe

rophy in response to stress|transcriptional repressor complex|cell differentiati

egative regulation of aspartic-type endopeptidase activity involved in amyloid

plasma membrane|bundle of His cell-Purkinje myocyte adhesion involved in cell c

. of viral life cycle|positive regulation of low-density lipoprotein particle cl

:|endoribonuclease activity, cleaving miRNA-paired mRNA|mRNA cleavage involved i

esterol|negative regulation of transcription from RNA polymerase II promoter in

acid autophosphorylation

.tive regulation of fibroblast proliferation|camera-type eye morphogenesis|neurc

ic signaling pathway via death domain receptors|regulation of Fas signaling pat

nscription by RNA polymerase II|repressing transcription factor binding|DNA con

;ative regulation of transcription, DNA-templated|positive regulation of transcr

gulation of insulin secretion involved in cellular response to glucose stimulus

ation|cellular response to leukemia inhibitory factor

liferation|positive regulation of chondrocyte proliferation

rical coupling|atrial cardiac muscle cell to AV node cell communication by elec

e regulation of NF-kappaB transcription factor activity|regulation of focal adh

rical coupling|atrial cardiac muscle cell to AV node cell communication by elec

rdiac epithelial to mesenchymal transition|ventricular septum morphogenesis|neg

gative regulation of histone acetylation|regulation of locomotion|histone bindi

.mellipodium morphogenesis

. of protein dephosphorylation|intracellular signal transduction|peptidyl-serine

oblast proliferation

ion|negative regulation of viral release from host cell|promoter-specific chrom

epithelial cell signaling|mammary gland epithelial cell differentiation|mammary

hogenesis|chromosome organization|cellular response to interleukin-1|negative r

olved in ERAD pathway|protein localization to cytosolic proteasome complex invc

of transcription, DNA-templated|positive regulation of transcription by RNA pol

ntiation|ventricular septum morphogenesis|atrial septum morphogenesis|transcrip

on|ascending aorta morphogenesis|dorsal aorta morphogenesis|microsatellite binc

precursor protein catabolic process|negative regulation of metalloendopeptidase

communication|cell adhesive protein binding involved in bundle of His cell-Purki

learance|positive regulation of low-density lipoprotein particle receptor bindin

n gene silencing by siRNA|mRNA cap binding|positive regulation of nuclear-trans

ι response to stress|regulation of odontoblast differentiation

on development|skeletal muscle fiber development|cell motility|roof of mouth dev

:hway

ıformation change

·iption by RNA polymerase II|guanine metabolic process|flavin adenine dinucleoti

s|cell communication by electrical coupling involved in cardiac conduction|regul

ctrical coupling|AV node cell to bundle of His cell communication by electrical

hesion assembly|positive regulation of telomerase activity|limb development|nega

:trical coupling|AV node cell to bundle of His cell communication by electrical

;ative regulation of androgen receptor signaling pathway|cellular response to BM

ng|dopamine biosynthetic process|response to drug|mitochondrial ATP synthesis c

: autophosphorylation|TORC2 signaling|positive regulation of I-kappaB kinase/NF-

ıatin binding|regulation of genetic imprinting

gland alveolus development|cell-cell signaling involved in mammary gland develo

regulation of pri-miRNA transcription by RNA polymerase II

›lved in ERAD pathway|ubiquitin-specific protease binding

.ymerase II|protein heterodimerization activity|sympathetic nervous system devel

›tional activation by promoter-enhancer looping

ling|umbilical cord morphogenesis|protein homodimerization activity|histone deac

e activity involved in amyloid precursor protein catabolic process|positive regu

[illegible]nje myocyte communication|regulation of heart rate by cardiac conduction|positi[illegible]

[illegible]g|positive regulation of low-density lipoprotein receptor activity|positive re[illegible]

scribed mRNA catabolic process, deadenylation-dependent decay|positive regulatic

velopment|retina development in camera-type eye|face morphogenesis|bone morphoge

.de binding|androgen receptor binding|positive regulation of DNA-binding transcı

.ation of cardiac conduction

coupling|bundle of His cell to Purkinje myocyte communication by electrical cou

ative regulation of pathway-restricted SMAD protein phosphorylation|ureteric bud

coupling|bundle of His cell to Purkinje myocyte communication by electrical cou

MP stimulus|proximal tubule development|sequence-specific double-stranded DNA bi

:oupled electron transport|identical protein binding|alpha-tubulin binding|neuro

-kappaB signaling|regulation of protein complex assembly|protein kinase B bindir

[illegible]pment

.opment|thymus development|peripheral nervous system neuron development|roof of

cetylase binding|sequence-specific DNA binding|cell fate commitment|regulation o

ılation of protein localization to early endosome|negative regulation of neurofi

ive regulation of canonical Wnt signaling pathway|cell-cell adhesion|regulation

gulation of receptor-mediated endocytosis involved in cholesterol transport

on of trophoblast cell migration|positive regulation of miRNA mediated inhibitio

enesis|SMAD protein signal transduction|protein homotrimerization|repressing tra

ription factor activity|muscle cell development|response to fungicide|telomeric

ıpling|Purkinje myocyte to ventricular cardiac muscle cell communication by elec

l formation|negative regulation of monocyte chemotaxis|negative regulation of ca

ıpling|Purkinje myocyte to ventricular cardiac muscle cell communication by elec

ınding|negative regulation of androgen receptor activity

›nal cell body|cysteine-type endopeptidase inhibitor activity involved in apopt‹

ng|negative regulation of neuron apoptotic process|cell body|positive regulation

mouth development|mesenchyme development|cartilage morphogenesis|coronary arte

of inner ear auditory receptor cell differentiation|negative regulation of Notch

ibrillary tangle assembly|positive regulation of endocytic recycling

of ventricular cardiac muscle cell action potential|ficolin-1-rich granule lum

on of translation|ribonucleoprotein complex

anscription factor binding

repeat-containing RNA binding|cellular response to cAMP|cellular response to ga

ctrical coupling|cell communication by electrical coupling involved in cardiac c

anonical Wnt signaling pathway|positive regulation of branching involved in ure

ctrical coupling|cell communication by electrical coupling involved in cardiac c

otic process|regulation of macrophage activation|negative regulation of neuron a

n of translation|negative regulation of JNK cascade|perinuclear region of cytop

ry morphogenesis|visceral serous pericardium development|cardiac neural crest ce

n signaling pathway|negative regulation of transcription, DNA-templated|positive

en

amma radiation|positive regulation of cold-induced thermogenesis|negative regul

conduction|gap junction channel activity involved in cardiac conduction electri

teric bud morphogenesis|negative regulation of branching involved in ureteric b

conduction|gap junction channel activity involved in cardiac conduction electric

apoptotic process|positive regulation of endocytosis|negative regulation of exo

lasm|protein stabilization|positive regulation of DNA-binding transcription fac

ell development involved in outflow tract morphogenesis|cell proliferation invo

e regulation of transcription, DNA-templated|positive regulation of transcripti

ation of intrinsic apoptotic signaling pathway in response to DNA damage by p53

cal coupling|gap junction channel activity involved in atrial cardiac muscle ce

ud morphogenesis|negative regulation of osteoclast proliferation|positive regul

cal coupling|gap junction channel activity involved in atrial cardiac muscle ce

cytosis|tau protein binding|regulation of long-term neuronal synaptic plasticit

tor activity|positive regulation of ubiquitin-protein transferase activity|regu

lved in outflow tract morphogenesis|determination of heart left/right asymmetry

on by RNA polymerase II|protein heterodimerization activity|regulation of neuro

class mediator|regulation of cellular protein localization|DNA repair complex

ll-AV node cell electrical coupling|gap junction channel activity involved in A

ation of peptidyl-tyrosine autophosphorylation|negative regulation of bone trab

ll-AV node cell electrical coupling|gap junction channel activity involved in A

y|perinuclear region of cytoplasm|synaptic vesicle endocytosis|synaptic vesicle

lation of mitochondrial membrane potential|positive regulation of protein kinas

|positive regulation of ERK1 and ERK2 cascade|E-box binding|positive regulation

genesis|smooth muscle cell differentiation|ventricular cardiac muscle cell deve

V node cell-bundle of His cell electrical coupling|gap junction channel activit

ecula formation|negative regulation of bone mineralization involved in bone mat

V node cell-bundle of His cell electrical coupling|gap junction channel activit

transport|arachidonic acid binding|positive regulation of inflammatory respons

e B signaling|C3HC4-type RING finger domain binding|regulation of proteasomal p

of p38MAPK cascade|primary palate development|positive regulation of transcrip

lopment|positive regulation of cardiac muscle cell proliferation|cardiac epithe

y involved in bundle of His cell-Purkinje myocyte electrical coupling|gap junct

uration|positive regulation of NIK/NF-kappaB signaling|positive regulation of s

y involved in bundle of His cell-Purkinje myocyte electrical coupling|gap junct

e|synapse organization|regulation of acyl-CoA biosynthetic process|phosphoprote

protein catabolic process|cellular response to hypoxia|establishment of protein

tion regulatory region DNA binding|positive regulation of transcription from RN

:lial to mesenchymal transition|heart trabecula formation|cardiac septum morphog

:ion channel activity involved in Purkinje myocyte-ventricular cardiac muscle ce

:ignaling receptor activity|positive regulation of cardiac muscle cell different

ion channel activity involved in Purkinje myocyte-ventricular cardiac muscle ce

ein binding|positive regulation of release of sequestered calcium ion into cytos

localization to mitochondrion|maintenance of protein location in mitochondrion|

IA polymerase II promoter involved in norepinephrine biosynthetic process|positi

;enesis|ventricular septum morphogenesis|atrial septum morphogenesis|negative re

ell electrical coupling|regulation of heart rate by cardiac conduction|disordere

ciation

ell electrical coupling|regulation of heart rate by cardiac conduction|disordere

sol|negative regulation of dopamine uptake involved in synaptic transmission|neg

positive regulation of mitochondrial fission|positive regulation of release of

.ve regulation of semaphorin-plexin signaling pathway involved in outflow tract

egulation of transcription initiation from RNA polymerase II promoter|labyrinthi

ed domain specific binding|positive regulation of blood vessel diameter|regulati

ed domain specific binding|positive regulation of blood vessel diameter|regulati

gative regulation of serotonin uptake|negative regulation of norepinephrine upta

cytochrome c from mitochondria|negative regulation of mitochondrial fission|cel

morphogenesis

ine layer blood vessel development|arterial endothelial cell differentiation|ca

ion of AV node cell action potential|regulation of bundle of His cell action po

ion of AV node cell action potential|regulation of bundle of His cell action po

ake|excitatory postsynaptic potential|long-term synaptic potentiation|positive r

llular response to toxic substance|Lewy body|astrocyte projection|positive regu

rdiac vascular smooth muscle cell development|coronary vasculature morphogenesi:

tential|regulation of Purkinje myocyte action potential|regulation of atrial ca

tential|regulation of Purkinje myocyte action potential|regulation of atrial ca

regulation of inositol phosphate biosynthetic process|negative regulation of th

lation of mitophagy in response to mitochondrial depolarization|mitochondrion t[illegible]

s|pulmonary artery morphogenesis|Notch signaling involved in heart development|

rdiac muscle cell action potential|regulation of renin secretion into blood str

rdiac muscle cell action potential|regulation of renin secretion into blood str

rombin-activated receptor signaling pathway|response to interleukin-1|dynein co

ɔ lysosome transport|regulation of cellular response to oxidative stress|positi

protein-DNA complex assembly|cochlea development|vascular smooth muscle cell de

eam|regulation of membrane depolarization during cardiac muscle cell action pot

eam|regulation of membrane depolarization during cardiac muscle cell action pot

mplex binding|cellular response to copper ion|positive regulation of protein se

ve regulation of histone deacetylase activity|regulation of synaptic vesicle tr

velopment|sequence-specific double-stranded DNA binding|negative regulation of

ential|vasomotion

ential|vasomotion

rine/threonine kinase activity|postsynapse|supramolecular fiber|positive regula

ansport|negative regulation of autophagosome assembly|positive regulation of mi

transcription regulatory region DNA binding|negative regulation of cardiac vasc

tion of neuron death|cuprous ion binding|positive regulation of glutathione per

tochondrial electron transport, NADH to ubiquinone|regulation of autophagy of m

ular smooth muscle cell differentiation|negative regulation of transcription fr

roxidase activity|positive regulation of hydrogen peroxide catabolic process|neg

ıitochondrion|negative regulation of autophagy of mitochondrion|negative regulat

·om RNA polymerase II promoter involved in smooth muscle cell differentiation|re

;ative regulation of chaperone-mediated autophagy|regulation of presynapse assem

:ion of oxidative stress-induced cell death|negative regulation of oxidative str

egulation of vasculogenesis

ıbly

ress-induced neuron death|regulation of protein targeting to mitochondrion|negat

:ive regulation of hypoxia-induced intrinsic apoptotic signaling pathway|negati[illegible]

ve regulation of hydrogen peroxide-induced neuron intrinsic apoptotic signaling

pathway|negative regulation of intrinsic apoptotic signaling pathway in respons

se to hydrogen peroxide|positive regulation of cristae formation|positive regul

ation of protein targeting to mitochondrion|positive regulation of free ubiquit

in chain polymerization|positive regulation of NMDA glutamate receptor activity

|cellular response to hydrogen sulfide|regulation of reactive oxygen species me

:tabolic process|negative regulation of reactive oxygen species metabolic proces

ss|positive regulation of ATP biosynthetic process|negative regulation of intrir

ısic apoptotic signaling pathway
